# Mapping under-5 and neonatal mortality in Africa, 2000–15: a baseline analysis for the Sustainable Development Goals

**DOI:** 10.1016/S0140-6736(17)31758-0

**Published:** 2017-11-11

**Authors:** Nick Golding, Roy Burstein, Joshua Longbottom, Annie J Browne, Nancy Fullman, Aaron Osgood-Zimmerman, Lucas Earl, Samir Bhatt, Ewan Cameron, Daniel C Casey, Laura Dwyer-Lindgren, Tamer H Farag, Abraham D Flaxman, Maya S Fraser, Peter W Gething, Harry S Gibson, Nicholas Graetz, L Kendall Krause, Xie Rachel Kulikoff, Stephen S Lim, Bonnie Mappin, Chloe Morozoff, Robert C Reiner, Amber Sligar, David L Smith, Haidong Wang, Daniel J Weiss, Christopher J L Murray, Catherine L Moyes, Simon I Hay

**Affiliations:** a School of BioSciences, University of Melbourne, Parkville, VIC, Australia; b Big Data Institute, Li Ka Shing Centre for Health Information and Discovery, University of Oxford, Oxford, UK; c Institute for Health Metrics and Evaluation, University of Washington, Seattle, WA, USA; d Department of Infectious Disease Epidemiology, Imperial College London, London, UK; e Bill & Melinda Gates Foundation, Seattle, WA, USA

## Abstract

**Background:**

During the Millennium Development Goal (MDG) era, many countries in Africa achieved marked reductions in under-5 and neonatal mortality. Yet the pace of progress toward these goals substantially varied at the national level, demonstrating an essential need for tracking even more local trends in child mortality. With the adoption of the Sustainable Development Goals (SDGs) in 2015, which established ambitious targets for improving child survival by 2030, optimal intervention planning and targeting will require understanding of trends and rates of progress at a higher spatial resolution. In this study, we aimed to generate high-resolution estimates of under-5 and neonatal all-cause mortality across 46 countries in Africa.

**Methods:**

We assembled 235 geographically resolved household survey and census data sources on child deaths to produce estimates of under-5 and neonatal mortality at a resolution of 5 × 5 km grid cells across 46 African countries for 2000, 2005, 2010, and 2015. We used a Bayesian geostatistical analytical framework to generate these estimates, and implemented predictive validity tests. In addition to reporting 5 × 5 km estimates, we also aggregated results obtained from these estimates into three different levels—national, and subnational administrative levels 1 and 2—to provide the full range of geospatial resolution that local, national, and global decision makers might require.

**Findings:**

Amid improving child survival in Africa, there was substantial heterogeneity in absolute levels of under-5 and neonatal mortality in 2015, as well as the annualised rates of decline achieved from 2000 to 2015. Subnational areas in countries such as Botswana, Rwanda, and Ethiopia recorded some of the largest decreases in child mortality rates since 2000, positioning them well to achieve SDG targets by 2030 or earlier. Yet these places were the exception for Africa, since many areas, particularly in central and western Africa, must reduce under-5 mortality rates by at least 8·8% per year, between 2015 and 2030, to achieve the SDG 3.2 target for under-5 mortality by 2030.

**Interpretation:**

In the absence of unprecedented political commitment, financial support, and medical advances, the viability of SDG 3.2 achievement in Africa is precarious at best. By producing under-5 and neonatal mortality rates at multiple levels of geospatial resolution over time, this study provides key information for decision makers to target interventions at populations in the greatest need. In an era when precision public health increasingly has the potential to transform the design, implementation, and impact of health programmes, our 5 × 5 km estimates of child mortality in Africa provide a baseline against which local, national, and global stakeholders can map the pathways for ending preventable child deaths by 2030.

**Funding:**

Bill & Melinda Gates Foundation.

## Introduction

Improvement of child survival is a long-standing international priority,^1^, ^2^, ^3^ and as shown in the last few decades, substantial progress has been accomplished in reducing child mortality and absolute inequalities in rates of child death across countries worldwide.^4^, ^5^ Yet by the conclusion of the Millennium Development Goals (MDGs), which aimed to reduce under-5 mortality by two-thirds from 1990 to 2015, only 57 of 195 countries and territories worldwide met or exceeded the pace of progress required to achieve MDG 4 (ie, a 4·4% annualised rate of decline) during that period.^5^ Additionally, despite narrowing disparities over time, geographic inequalities persisted among countries with the lowest and highest child mortality rates. In sub-Saharan Africa, for example, this divergence in 2015 spanned from 15·6 deaths per 1000 livebirths in Botswana to 135·0 deaths per 1000 livebirths in the Central African Republic.^5^ National mortality rates, although useful for macro-level comparisons,^4^, ^6^, ^7^ obscure variations in child survival at lower administrative units (eg, districts), the levels at which most health programme planning and implementation occur. Without advancing the aims of precision public health, which includes robust subnational monitoring of child mortality levels and trends, health authorities face sizeable challenges to optimally funding and targeting interventions for the populations who most need them.^8^, ^9^, ^10^, ^11^ Ending all preventable child deaths by 2030 is the bold aim set forth by the Sustainable Development Goals (SDGs),^3^ and an ambition that requires a much better understanding of where exactly the largest gaps remain in improving child survival.

**Research in context**

**Evidence before this study**

Since the adoption of the Millennium Development Goals, monitoring levels and trends of child mortality has been a key component of benchmarking progress in child health, as has the prioritisation of policy and financial attention to achieve these targets. International assessments, such as the Global Burden of Diseases, Injuries, and Risk Factors Study (GBD), systematically quantify under-5 mortality rates at national levels and over time, and increasingly more studies have analysed subnational under-5 mortality trends in sub-Saharan Africa, Latin America, and Asia. Although this work is of high relevance to decision making, marked inequalities in child survival could remain beyond provincial and even district levels, and differing data sources and methodological approaches might limit comparability across subnational studies. In the absence of fully operational and representative vital registration systems for tracking highly local trends in child mortality, a promising approach is to harness the plethora of available geolocated data.

**Added value of this study**

To our knowledge, this study offers the first quantification of levels and trends of under-5 and neonatal mortality at a resolution of 5 × 5 km in 46 countries in Africa between 2000 and 2015. Our methodological approach includes several advantages over recent efforts to produce local estimates of child mortality. We made full use of geo-referenced data from 235 household sample surveys and censuses and related covariates over space and time. We used child death data from both complete and summary birth histories and synthesised data that could be precisely located to survey-cluster GPS locations with data that could only be designated to administrative levels. We used country estimates from the latest iteration of the GBD to calibrate the aggregation of our 5 × 5 km estimates to national levels.

**Implications of all the available evidence**

Our study offers a highly granular baseline analysis for assessing progress toward child mortality targets established under Sustainable Development Goal (SDG) 3.2: reducing under-5 mortality to 25 deaths per 1000 livebirths or lower, and neonatal mortality to 12 deaths per 1000 livebirths or lower in all countries by 2030. Although both under-5 mortality and neonatal mortality rates have generally improved throughout Africa since 2000, striking heterogeneity emerged both in terms of 2015 mortality rates and annualised rates of decline achieved between 2000 and 2015. Areas in Botswana, Rwanda, and Ethiopia recorded some of the largest decreases in under-5 mortality since 2000, which could position these places well to reach SDG 3.2 by 2030 or earlier. Yet attaining SDG 3.2 remains a very ambitious goal for much of Africa, with several areas—particularly in central and western Africa—needing to reduce under-5 mortality rates by at least 8·8% per year between 2015 and 2013, to achieve the SDG 3.2 target by 2030. This kind of unprecedented progress in improving child survival will require substantial inter-sectoral investment and innovation, ranging from the expansion of high-quality health care to heightened socioeconomic prospects across communities. By generating estimates of child mortality across a range of subnational levels—from 5 × 5 km pixels to the first and second administrative levels—our study offers decision makers greater precision with which they can design and target high-impact child health interventions in Africa.

Advances in both data availability and statistical methods have facilitated subnational assessments of under-5 mortality in several sub-Saharan African countries, including provinces in South Africa,^4^ regions in Tanzania,^12^ states in Nigeria,^13^ counties in Kenya,^4^ and districts in Ghana, Mozambique, Uganda, and Zambia.^14^, ^15^, ^16^ Such work has unveiled the initial magnitude of subnational disparities in all-cause child deaths, but it is likely that much more heterogeneity remains within formal administrative units. In an ideal setting, national vital registration or health information systems would routinely capture local data on deaths and births, and estimates of child survival could be generated at a similar resolution on the basis of vital registration data. However, few countries in sub-Saharan Africa have complete or high-quality vital registration systems,^17^, ^18^, ^19^ and thus often rely on household surveys and periodic censuses to assess their demographic and health profiles.^20^ These data sources often include geolocated cluster-level or administrative-area-level identification. They can therefore be analysed with spatially explicit methods such as model-based geostatistics, which quantify spatial differences in variables from geolocated data. Use of model-based geostatistics allows for the synthesis of disparate geographical data into gridded maps and thus yields comparable high-resolution estimates over larger study areas.^21^, ^22^ Previous studies have used model-based geostatistics methods to produce gridded estimates of infant mortality and child mortality in Mali,^23^ and advancements in computational statistics now allow for high-resolution estimation of health outcomes and related indicators at the continental scale.^24^, ^25^, ^26^, ^27^

Recent analyses^28^, ^29^ have sought to quantify under-5 mortality with greater geospatial resolution in sub-Saharan Africa, including one study that produced 10 × 10 kilometre (km) estimates of all-cause under-5 mortality in 28 countries.^29^ However, these studies feature several data and methodological shortcomings. First, previous studies exclusively drew from the Demographic and Health Survey (DHS) series for their data sources,^28^, ^29^ constraining their analyses to a limited subset of surveys with both complete birth histories and GPS-identified survey clusters. Second, popular spatial interpolation methods such as kernel-density estimation can be overly sensitive to data variations that result from small numbers found at the survey cluster rather than true subnational differences in child survival. Without a more stable estimation approach that accounts for spatial and temporal correlations in the data, misleading or implausible results can arise (eg, localities experiencing increases in under-5 mortality that exceeded an average of 10% per year from the 1980s to 2000s).^29^ Third, recent analyses do not have the temporal specificity or timeliness that is of greatest demand from policy makers; for instance, a study that presented decade-wise estimates for the 1980s, 1990s, and 2000s^29^ could not detail the potential effects of the MDGs on under-5 mortality trends. Last, previous studies have not calibrated the aggregation of geospatial estimates to externally validated national estimates,^4^, ^5^, ^30^ a key step to ensuring the internal consistency of subnational results. Since high-resolution estimates of child mortality could serve as a crucial input for local health programme funding and deployment, it is of equally high importance to address these outstanding data and analytic challenges.

In this study, we aimed to advance geospatial analysis of child mortality in Africa by using a larger set of data sources and types than previously published, as well as applying advanced modelling techniques^22^, ^24^, ^27^, ^31^ in order to generate high-resolution estimates of under-5 and neonatal all-cause mortality in 46 countries in Africa in 2000, 2005, 2010, and 2015.

## MethodsOverview

Our analysis provides estimates of under-5 mortality (the probability of death before age 5 years per 1000 livebirths) and neonatal mortality (probability of death within first month of life per 1000 livebirths) for each 5 × 5 km cell in 46 countries in Africa. These countries accounted for 54% of global under-5 deaths in 2015,^5^ and they fully overlap with the African countries included in the EQUitable Impact Sensitive Tool (EQUIST), a UNICEF-supported initiative that aims to maximise the effect of health policies for children who reside in low-income countries. Analytical steps are described below and in the appendix. Our study follows the Guidelines for Accurate and Transparent Health Estimates Reporting (GATHER). The GATHER checklist appears in the appendix.

### Data

We extracted data on child mortality and geographical locations from censuses and several household survey series, including the DHS, UNICEF Multiple Indicator Cluster Surveys, and other country-specific surveys. We included data sources if they had summary or complete birth history modules and subnational geographical information. When available at the cluster level (ie, a group of neighbouring households or a village associated with a set of geolocated data), data were extracted together with cluster GPS or located on the basis of precise location names (n=59 279). In the absence of geographic coordinates, we extracted names of the smallest available administrative area units (n= 6111). The appendix lists all data sources (pp 7–22).

We estimated child mortality rates in 5-year periods, such that mortality rates for each period of time represented probabilities of death for a synthetic cohort. Thus, mortality rates at each point in time reflected average mortality rates over the corresponding years. For the estimates, we refer to 1998–2002 as 2000, 2003–07 as 2005, 2008–12 as 2010, and 2013–17 as 2015. These estimates were disaggregated by monthly probabilities of death for the age groups of first month of life, 1–11 months, 12–35 months, and 36–59 months. As such, exposures and deaths could fall into one of 16 age-group–time period bins. A month counted as an exposure if the child was alive at the beginning of the month. Probability of death before reaching age 5 years for a given period can then be calculated directly as 1 minus the product of age-bin-specific survival probabilities (appendix p 23).^32^ Only livebirths were captured and we did not estimate stillbirths.

**Figure 1:**
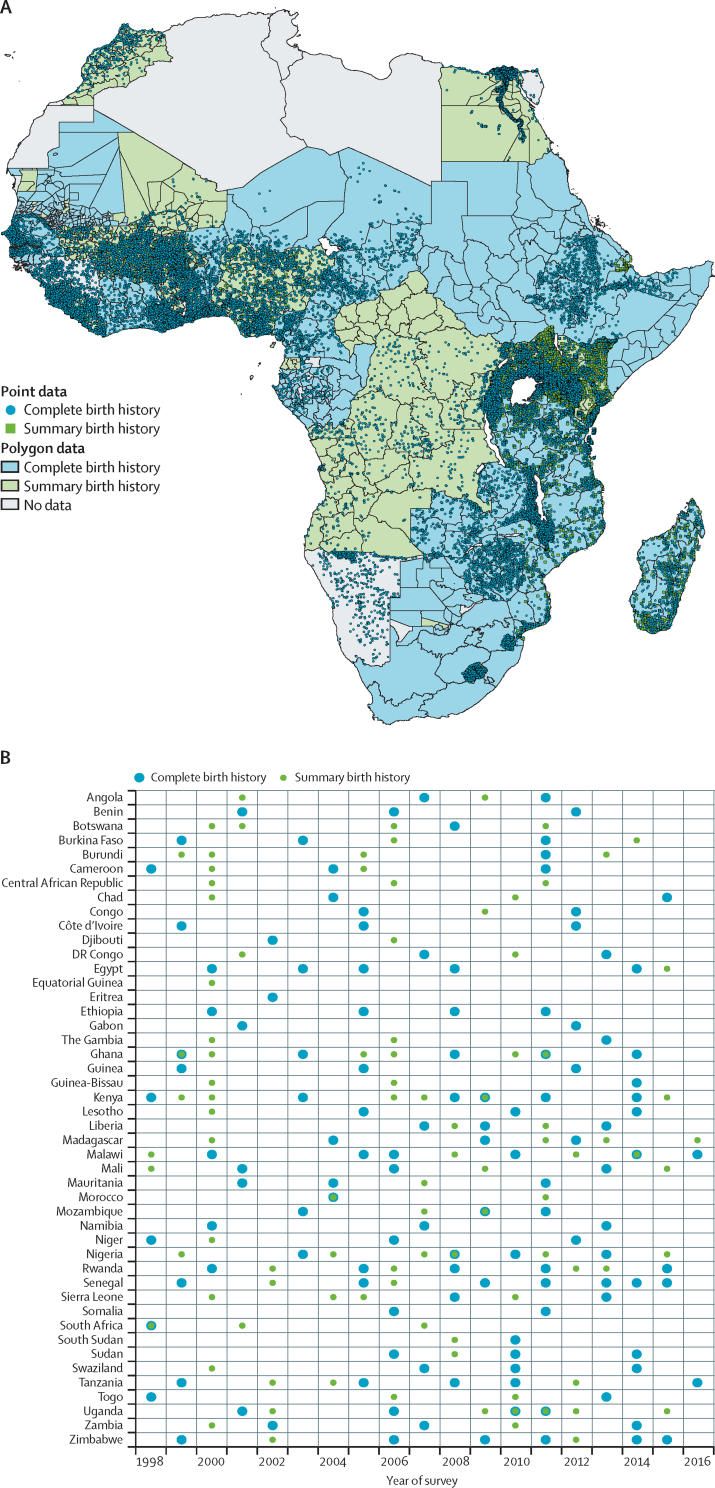
Data availability by type and country, 2000–15 All data are mapped (A), and shown by country and year of survey (B). Surveys can contribute mortality data up to 17 years before the time of the survey. (A) Complete birth history data are displayed in preference to summary birth histories when both have been used in that location. Cluster locations are mapped as points, and polygon data as shapes where available. (B) Data on summary birth histories are green and complete birth histories data are blue.

We extracted two types of child mortality data, namely complete birth histories and summary birth histories. Data availability by type and country is shown in figure 1, and is further detailed in the appendix (pp 7–22). Records of complete birth histories contain dates of birth and death, as applicable, of all children of sampled women. We summed the total exposure months and death events within each age group and time period at the most precise available geographic level (ie, either cluster points or administrative polygons). Summary birth history records provide considerably less information than complete birth histories. They only include mothers' reports of the total number of children ever born and those who died, and do not include children's age or date of death. However, summary birth history data are relatively easy to collect and are widely available in Multiple Indicator Cluster Surveys and censuses. After we extended methodological advancements in model-based approaches to adjust data on summary birth histories,^33^ we developed a model using data in which both complete and summary birth histories were available, to partition summary birth history data into age-specific and period-specific mortality probabilities, and applied this relationship to datasets in which summary birth history data were available. Additional detail on our summary birth histories-adjustment model is in the appendix (pp 24–31). Finally, bias ratios calculated for GBD 2016,^5^ which are specific to data type, source, and year, were applied to both complete birth histories and summary birth histories data (appendix p 31).

When cluster-level geographic coordinates were not available for a given country, we matched reported administrative units with first-level, second-level, or third-level administrative divisions from the Global Administrative Unit Layers database or the Database of Global Administrative Areas. We refer to the geographical data that only contained administrative boundaries as polygons. If data could not be mapped to administrative units (polygons) from these sources, we matched them using other sources or modified available polygons on the basis of the available information about polygon boundaries. In several cases, for example, we disaggregated an administrative unit into city boundaries and the surrounding rural area to match the disaggregation of the available mortality data. We resampled polygon data into geographically dispersed points and weighted them on the basis of population. In terms of sample size, polygons contributed 41% of the total data (appendix pp 32–37).

To inform our model, we compiled several layers of sociodemographic and health-related covariates at the 5×5 km pixel level (value estimates for each 5×5 km grid cell in the study area), all of which have shown some relationship with child survival or overall child health outcomes. The covariates were average years of educational attainment of women aged 15–49 years,^34^, ^35^ prevalence of wasting and stunting in children younger than 5 years,^36^ *Plasmodium falciparum* parasite rate,^24^ a proxy index of fertility (based on the ratio of women aged 15–49 years and children under 5 years) per pixel,^37^, ^38^ and total population.^39^ Additionally, we included several covariate layers that were reflective of potential environmental and infrastructural factors related to overall development and thus child survival (ie, an enhanced vegetation index,^40^ daytime land-surface temperature,^40^ proportion of land under irrigation,^41^ urban-rural distinction,^42^ brightness of night-time light from the Defense Meteorological Satellite Program,^43^, ^44^ and accessibility to cities with populations greater than 50 000).^45^ When several years' worth of data were available, we either took the synoptic mean from available years in each estimation period or used the mid-period-year estimate. Two covariate layers, irrigation and accessibility, were only available for the year 2000, and therefore did not vary over time in our analysis. The appendix contains more information on our spatial covariates, including plots of all covariates (pp 38–39).

We then used an ensemble method, stacked generalisation, to select covariates, capture possible non-linear effects, and to account for the complex interactions between them.^31^ For each age group and year, we fit four sub-models, namely generalised additive models, boosted regression trees, lasso regression, and ridge regression, and predicted for each cluster in the data using five-fold cross-validation. The four sub-model predictions were included as covariates when fitting the full geostatistical model described below. This approach removes covariates that are not predictive and identifies optimal combinations of covariates that are predictive, and thus is expected to improve overall predictive performance over a singular model.^31^ Because of their heightened predictive validity, ensemble modelling approaches are increasingly used in population health measurement,^46^ as well as other fields.^47^, ^48^ More details on this approach are in the appendix (pp 39–41).

### Analysis

We fitted four separate Bayesian model-based geostatistics models (ie, one for each age group) to estimate the monthly probability of mortality in any given pixel. We modelled covariate effects using the ensemble approach discussed above, and the model explicitly accounted for spatiotemporal autocorrelation by modelling the covariance of data residuals in space and time. This allowed us to leverage the correlation structure of the data to more accurately predict mortality within locations where data on child death were absent. Pixel-level uncertainty intervals (UIs) were generated from 1000 draws (ie, candidate maps)^22^ that were created from the posterior distributions of modelled parameters. The appendix includes additional detail of our model and estimation process (pp 41–46).

We then aggregated pixel-level estimates from the 1000 candidate maps up to two subnational administrative units and national levels.^49^ Such aggregation allowed us to further calibrate estimates of under-5 and neonatal mortality to national GBD estimates for the 2000, 2005, 2010, and 2015 periods.^5^ We achieved this by calculating the ratio of the population-weighted posterior mean national-level estimate from our analysis to mean national estimates for the same time period from GBD,^5^ and then multiplying each cell in the posterior sample by this ratio. The median for these ratios was 1·01 (IQR 0·95–1·07), indicating generally close agreement with GBD estimates. The appendix includes scatterplots comparing our national-level estimates from this analysis with GBD estimates (p 50).

For reported results, we masked all final model outputs for which land cover was classified as barren or sparsely vegetated, on the basis of MODIS satellite data in 2013,^50^ the most recent year of available data, as well as areas in which total population density was less than ten individuals per 1 × 1 km pixel in 2015.^39^

We validated our models using spatially stratified five-fold out-of-sample cross validation and report bias (mean error), total variance (root-mean-square error), and 95% cluster-level data coverage within prediction intervals. We stratified space either by the first or second subnational administrative unit. By aggregating predictions and observations to administrative units, we could increase sample sizes to a level high enough for better determining model fit; this was not feasible at the cluster level because of data noisiness due to very small sample sizes. Generally, across our four models and at both levels of aggregation, we found out-of-sample mean error to be very close to zero, indicating no systematic bias. Correlations between aggregated out-of-sample model fit and aggregated holdout data ranged from 0·81 to 0·94 for administrative level 1, and from 0·62 to 0·87 for administrative level 2. Root-mean-square error relative to mean estimated probability ranged from 17·7% to 33·3% for administrative level 1 and 32·7% to 50·0% for administrative level 2. These metrics indicate relatively good model fits; however, these metrics are sensitive to sample sizes at aggregation and average probabilities at different age groups, and thus should be interpreted within that context. The 95% prediction interval at the cluster level covered 94% of the out-of-sample data points, indicating that our models' fit could reproduce out-of-sample data within the specified level of uncertainty. Detail on validation procedures and full results is in the appendix (pp 46–49).

### Data sharing

Data are available at http://ghdx.healthdata.org/.

### Role of the funding source

The funders of the study had no role in study design, data collection, data analysis, data interpretation, or writing of the report. The corresponding author had full access to all the data in the study and had final responsibility for the decision to submit for publication.

## Results

**Figure 2:**
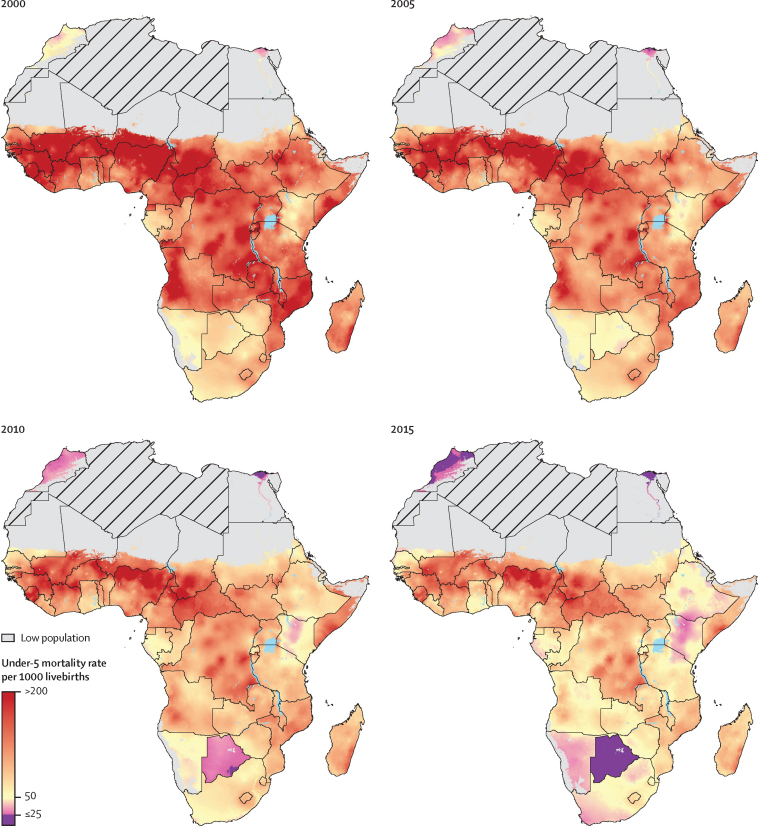
Under-5 mortality rates at the 5 × 5 km resolution in 2000, 2005, 2010, and 2015 Data are at 5 × 5 km resolution. All pixels with an under-5 mortality rate equal to or fewer than 25 deaths per 1000 livebirths (the Sustainable Development Goal 3.2 target for under-5 mortality) are coloured purple. Pixels with fewer than ten people and classified as barren or sparsely vegetated are coloured in grey. Grey areas with diagonal lines are not included in this analysis. km=kilometre.

Although under-5 mortality rates decreased throughout Africa between 2000 and 2015 (figure 2), stark disparities endured across the continent and within national borders. On the basis of pixel-level estimates, we found that in 2000, most of sub-Saharan Africa recorded under-5 mortality rates exceeding 138 deaths per 1000 livebirths, while large swathes of Nigeria, Niger, Sierra Leone, and Mali, along with other countries in western and central Africa, surpassed 200 deaths per 1000 livebirths. By 2015, half of sub-Saharan Africa had under-5 mortality rates below 72 deaths per 1000 livebirths, and increasingly more places in Africa neared or had fewer than 25 deaths per 1000 livebirths, the SDG3.2 target for 2030. Nonetheless, 118 locations at the second administrative level in Chad, Mali, Burkina Faso, the Central African Republic, and Nigeria still faced average under-5 mortality rates higher than 170 per 1000 livebirths in 2015. Further, sizeable within-country inequalities remained. For instance, Nigeria had a national under-5 mortality rate of 115·4 deaths (95% uncertainty interval [UI] 99·6–132·2) per 1000 livebirths in 2015,^5^ yet at the local government area level (administrative level 2), under-5 mortality rates ranged from 54·8 (48·2–62·9) deaths per 1000 livebirths in the Osun local governmental area of Osogbo to 214·6 (190·2–240·6) deaths per 1000 livebirths in the Bauchi local governmental area of Ningi.

Somewhat similar geographic patterns emerged for neonatal mortality as were found for under-5 mortality (appendix), with Botswana and Egypt having large areas meeting the SDG target (ie, 12 deaths per 1000 livebirths). In 2015, Côte d'Ivoire had one of the widest gaps between districts, ranging from 29·6 (95% UI 23·9–35·7) neonatal deaths per 1000 livebirths in Cavally in Montagnes district to 52·9 (43·2–64·1) neonatal deaths per 1000 livebirths in Bagoue in Savanes district. Côte d'Ivoire, Mali, and Nigeria all had second administrative units with mean neonatal mortality rates greater than 50 deaths per 1000 livebirths. More information on neonatal mortality can be found in the appendix.

**Figure 3:**
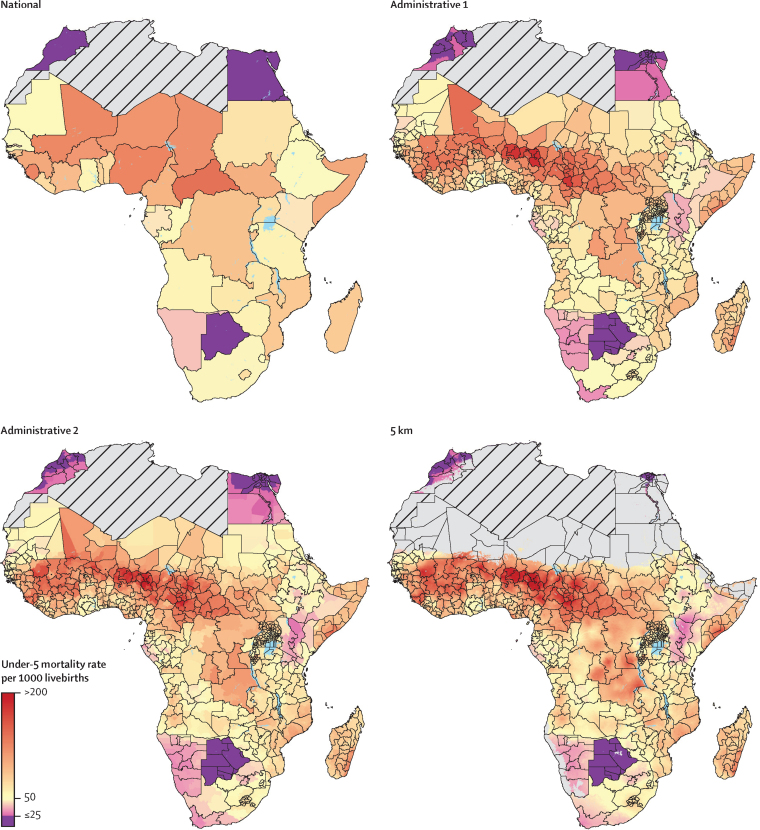
Under-5 mortality rates at the national, first and second administrative, and 5 × 5 km levels in 2015 All locations with a mortality rate equal to or fewer than 25 deaths per 1000 livebirths (the Sustainable Development Goal 3.2 target for under-5 mortality) are coloured purple. Pixels with fewer than ten people and classified as barren or sparsely vegetated are coloured in grey. Grey areas with diagonal lines are not included in this analysis. km=kilometre.

Figure 3 illustrates the effects of disaggregating under-5 mortality estimates across levels of geospatial granularity (national, first and second administrative levels, and the 5 × 5 km grid), and how inequalities in child survival can be masked by geographic aggregation. In 2015, Egypt and Morocco achieved the SDG3.2 target for under-5 mortality, at 20·6 deaths per 1000 livebirths in Egypt and 22·9 deaths per 1000 livebirths in Morocco.^5^ Still, approximately 25% of each country's population lived in areas with mortality rates higher than the SDG threshold. Botswana was the only other African nation that met the SDG 3.2 target by 2015, with an under-5 mortality rate of 15·6 deaths per 1000 livebirths.^5^ The appendix includes full geographical disaggregation of neonatal mortality rates by administrative level.

**Figure 4:**
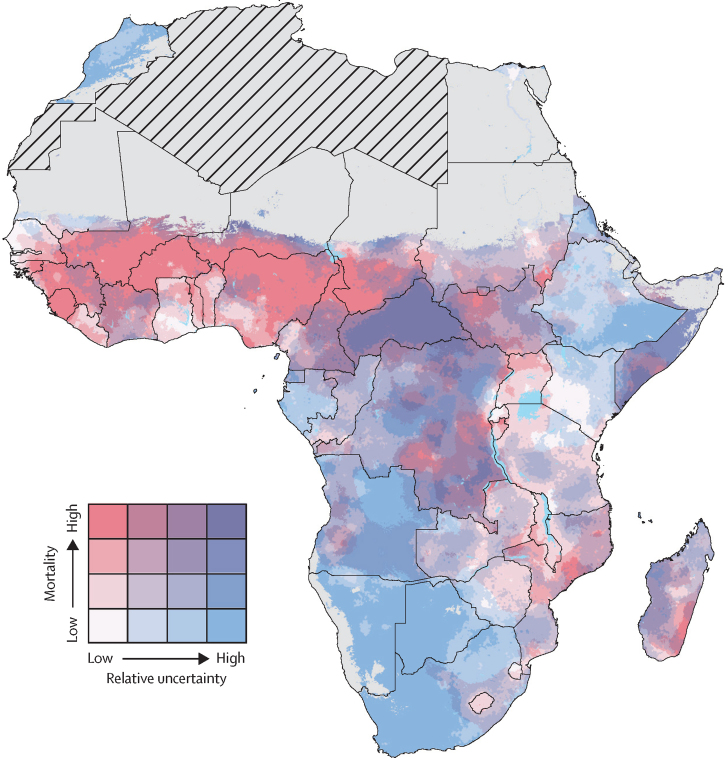
Overlapping population-weighted quartiles of under-5 mortality and relative uncertainty in 2015 Under-5 mortality rate quartile cutoff points were 56, 80, and 102 deaths per 1000 livebirths. Relative uncertainty was computed as the ratio of the 95% uncertainty intervals and under-5 mortality rate for each pixel. Cutoff points for uncertainty were 29%, 35%, and 41%. The lowest quartile of mortality is white, and the highest is dark pink. The lowest quartile for uncertainty is white and the highest is blue. These colours overlap such that areas coloured purple have both high under-5 mortality rates and high relative uncertainty. Pixels with fewer than ten people and classified as barren or sparsely vegetated are coloured in grey. Grey areas with diagonal lines are not included in this analysis.

Figure 4 compares dimensions of under-5 mortality, from low to high, against relative uncertainty, as measured by the ratio of the 95% UI range to the mean, in 2015. Senegal, Gambia, Ghana, Kenya, Rwanda, Tanzania, and Zimbabwe had large areas with relatively low under-5 mortality and low uncertainty, whereas most of the Central African Republic and Somalia had both relatively high under-5 mortality and uncertainty. There were geographical areas of high mortality and low uncertainty in northern and eastern Nigeria, Mali, Burkina Faso, northern Cameroon, and southwestern Chad. By contrast, most of Botswana, Namibia, eastern Ethiopia, eastern Angola, and South Africa had relatively low under-5 mortality rates, but these estimates were accompanied by relatively high uncertainty. More plots of uncertainty in predictions are available in the appendix (pp 44–45).

**Figure 5:**
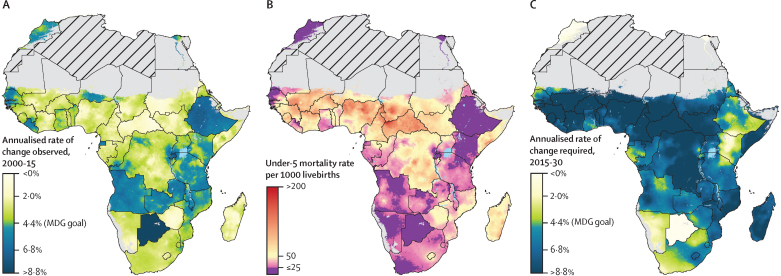
Annualised rates of decline in under-5 mortality during the MDG era, with projections to 2030, and needed rates of decline to reach the SDG target 4·4% is the annualised rate of decline that was equivalent to the pace of progress required to meet Millennium Development Goal 4. (A) Annualised rates of decline for under-5 mortality from 2000 to 2015. Pixels coloured blue exceeded the annualised rate of decline between 2000 and 2015, whereas pixels coloured green to yellow had a slower rate of annualised decline during this time. (B) Predicted under-5 mortality rates in 2030, based on annualised rates of decline achieved between 2000 and 2015. Pixel-level under-5 mortality rates were predicted for 2030 on the basis of annualised rates of decline achieved from 2000 to 2015. Based on this prediction, pixels for which under-5 mortality rates equalled or were less than 25 deaths per 1000 livebirths in 2030 are coloured purple. (C) Rates of decline required to reach the SDG 3.2 target for under-5 mortality by 2030 (25 deaths per 1000 livebirths). Pixels coloured blue will need to achieve a 4·4% or greater decline per year from 2015 to 2030 to achieve the SDG 3.2 target for under-5 mortality. Pixels coloured green to yellow can meet the SDG 3.2 target by 2030 at a pace slower than a 4·4% reduction per year from 2015 to 2030. Pixels with fewer than ten people and classified as barren or sparsely vegetated are coloured in grey. Grey areas with diagonal lines are not included in this analysis. SDG=Sustainable Development Goal.

In many countries, under-5 mortality decreased by more than 4·4% per year from 2000 to 2015 (figure 5),^5^ a rate that exceeded the pace of progress established under MDG 4 (ie, a two-thirds reduction by 2015). Further, average annualised rates of decline in Botswana, Ethiopia, Liberia, Rwanda, and Angola exceeded 6·0%. Many other countries, including Burundi, Malawi, Togo, Uganda, and Tanzania, had second-level administrative areas that had a mixture of annualised rates of decline between 2·0% and 4·4%, as well as those that exceeded a 4·4% decrease each year; results for administrative levels 1 and 2 are in the appendix. By contrast, areas throughout 17 countries, including the Central African Republic, South Sudan, Lesotho, and Madagascar, had no second-level administrative areas that achieved an average annualised rate of decline exceeding 4·4% from 2000 to 2015. Nigeria had wide disparities in terms of progress, with annualised rates of change spanning from 0·7% annual decline (95% UI 0·2% to −1·6%) to a 5·0% annual decline (−4·1 to −5·9%). On the basis of pixel-level annualised rates of decline achieved from 2000 to 2015, and projections of these rates through 2030 (figure 5), several countries could have localities achieving SDG 3.2 if past rates of decline in under-5 mortality are sustained over the next 15 years. These locations were primarily in northern, southern, and eastern Africa, but also included areas in Senegal, Liberia, and Ghana. 20 of the 46 countries had second-level administrative areas that, on the basis of average current trajectories, could reduce under-5 mortality rates to 25 deaths per 1000 livebirths. However, only 26·1% of second administrative-level areas had an annualised rate of decline from 2000 to 2015 that was faster than the MDG 4 target of 4·4% per year. At least 60% of these locations need to match or surpass the rates of progress achieved from 2000 to 2015 to meet the SDG3.2 target for under-5 mortality by 2030 (figure 5). For instance, within the Central African Republic, Mali, Sierra Leone, Niger, Chad, and Burkina Faso, the majority of populations live in areas where annual declines of 8·8% or more are needed to achieve SDG 3.2.

Additional results can be found in the appendix and explored in a dynamic data visualisation.

## Discussion

To the best of our knowledge, our study offers the first quantification of 5 × 5 km estimates of under-5 and neonatal mortality in 46 African countries, highlighting a mixture of impressive gains and enduring disparities in child survival across the continent. By 2015, nearly all locations had a reduction in under-5 mortality rates from 2000, with many areas of Ethiopia, Botswana, and Rwanda recording particularly large reductions. Yet in Chad, Mali, the Central African Republic, and Sierra Leone, more than half of populations live in places where under-5 mortality rates still exceeded 120 deaths per 1000 livebirths in 2015. Despite achieving notable rates of decline between 2000 and 2015, most of Africa must substantially accelerate reductions in under-5 mortality to meet the SDG 3.2 target of 25 deaths per 1000 livebirths by 2030. These results underscore the crucial importance of tracking geospatially granular patterns in child survival, particularly if all countries aim to end all preventable child deaths by 2030.

Charting 5 × 5 km trends in child mortality from 2000 to 2015 provides the foundation from which local achievements and challenges during the MDG era can be better understood. Although the duration of MDG assessment spanned from 1990 to 2015, its greatest catalytic effects on political, social, and financial commitments to improving child health in Africa mainly occurred from 2000—when the MDGs were established—to 2015. Nationally, 19 countries in Africa met or exceeded the MDG4 rate of reduction (4·4% each year) between 2000 and 2015.^5^ Although several factors probably influenced this progress during the 2000s, the confluence of escalated development assistance focused on child health,^51^ the rapid scale-up of multiple interventions that target childhood illnesses (eg, vaccination, malaria control, and HIV prevention),^25^, ^52^, ^53^, ^54^, ^55^, ^56^ and heightened overall socioeconomic development undoubtedly contributed to improving child survival in many African countries. By contrast, it is an unlikely coincidence that many places with slower gains (ie, much of the Central African Republic, Chad, and Somalia) also received less international funding for newborn and child health^26^ and had some of Africa's lowest levels of overall coverage for key maternal and child health interventions.^55^ In Nigeria, for example, where annualised declines in under-5 mortality ranged from 0·7% each year to 5·0% per year since 2000, large differences in state-level trends for various maternal and child health interventions also occurred during that time.^13^ National case studies have explored drivers of MDG 4 progress in sub-Saharan Africa,^54^, ^57^, ^58^, ^59^, ^60^ but few delve into more local factors and their association with changes in under-5 and neonatal survival at a high geographic resolution. In-depth analyses that link our 5 × 5 km estimates of child mortality to more granular measures of intervention coverage and other indicators, akin to a recent study on the effects of malaria control in Africa,^25^ could strengthen inputs into local health policy and resource allocation planning.

In the transition from MDG 4 to SDG 3.2, child survival targets changed from achieving relative rates of progress to attaining absolute levels by 2030. This shift is heralded by many,^61^, ^62^, ^63^ because setting specific thresholds could encourage a greater focus on the places which bear the highest toll of child deaths. Effectively directing such attention might be a challenge in Africa, however, especially since more than 75% of the continent's children live in areas where annual declines in under-5 mortality must exceed the pace of reductions they achieved from 2000 to 2015 to meet the SDG 3.2 target by 2030. From 2000 to 2015, only 26·1% of second administrative level areas recorded an annualised rate exceeding the MDG target of 4·4%, whereas at least 60% of these locations will need to at least match their current pace to make the SDG 3.2 target for under-5 mortality a reality. Of particular concern are the areas encompassing nearly 27% of Africa's population that must at least double the MDG 4 rate to achieve the SDG 3.2 target by 2030, a pace that is unprecedented in the last few decades. Envisaging such a feat is difficult without the occurrence of substantial medical breakthroughs, such as a fully effective malaria vaccine,^64^ or considerably extending access to high-quality health care, eliminating risk factors that account for a large proportion of child deaths,^36^ and bolstering socioeconomic factors that directly affect child survival,^7^ or all of the above. That is not to say achieving SDG3.2 is impossible in Africa, since there have been several instances in which the introduction and scale-up of cost-effective interventions swiftly reduced child mortality in many countries,^45^ such as the expansion of measles immunisation, screening and treatment of maternal syphilis during antenatal care, and the provision of oral rehydration therapy for severe diarrhoea. At a time when precision public health could offer transformative power for local intervention design and implementation,^9^, ^10^ these results and initiatives, such as EQUIST, which account for subnational variations in health, intervention effectiveness, and costs, are vital going forward in the SDG era. However, staying the current course and failing to address more systemic barriers to improving child survival will not be sufficient to meet the SDG 3.2 target for most of Africa.

This study offers the analytical framework from which we aim to extend geospatial modelling of child mortality to an increased number of locations, with a heightened focus on estimating specific causes with greater temporal resolution. By expanding our high-resolution estimation of child mortality to additional high-burden countries outside of Africa, we aim to generate estimates in locations that represent 95% of under-5 deaths globally.^5^ We also intend to disaggregate estimates by year rather than by 5-year intervals. Ultimately, our goal is to generate cause-specific mortality estimates at the 5 × 5 km resolution globally, but this undertaking necessitates improved encoding or identification of specific causes of death by precise locations. A recent analysis^24^ produced 5 × 5 km estimates of malaria mortality in Africa by age group,^24^ including children younger than 5 years, which served as an initial foray into this kind of high-resolution, cause-specific mortality mapping. Generating pixel-level estimates of mortality for children younger than 5 years from other causes that disproportionately affect children, such as diarrhoeal diseases and lower respiratory infections, are also future analytical priorities.

The production of more geospatially granular estimates of key child health outcomes hinges upon heightened accessibility to and collection of geo-referenced data. Increased data availability would both facilitate cause-specific mortality estimation and reduce uncertainty in all-cause estimates, which particularly affect locations with substantive geographical or temporal data gaps. In an ideal setting, agencies involved in local data collection and management would work with the array of data users to identify best practices for sharing geo-referenced data and thus creating global goods, while maintaining privacy and respecting data use agreements. Increasing the availability of geolocated data, from surveys to censuses to facility records, would greatly strengthen the precision of local monitoring of child health needs. By highlighting geographical areas where data gaps are most strikingly pronounced, we hope to encourage more collaboration between data users and providers.

Although household survey and census data offer good information on child deaths, they are inferior substitutes to high-quality, fully representative vital registration systems for providing timely, continuous, and complete subnational information on births and deaths. Vital registration systems should remain the gold standard for routine monitoring of national and local child mortality, and in recent years both political and financial investments in improving vital registration have increased.^65^, ^66^, ^67^ Moreover, SDG indicator 17.19.2 explicitly outlines targets for birth and death registration completeness,^3^ and efforts such as the Bloomberg Data for Health Initiative seek to swiftly strengthen existing vital registration systems and to help build vital statistics infrastructure in places where they are inadequate or non-existent. Nonetheless, massive disparities persist in birth and death registration levels, and Africa has some of the largest gaps in the establishment, coverage, and quality of vital registration.^68^ Using household survey and census data to inform child mortality estimation is likely to be a necessity in many parts of the world for the immediate future, but as vital registration systems continue to improve, future analyses should involve developing methods to integrate vital registration data with survey and census data within the model-based geostatistics framework.

Our findings should be interpreted within the context of some methodological limitations. First, we assumed no migration, which implied that all recorded births, deaths, and exposures were assumed to occur in the survey location. The ability to properly measure and incorporate indices of migration has been an enduring challenge for large-scale demographic and epidemiological studies,^4^, ^5^, ^69^, ^70^ and though innovative efforts such as the WorldPop project^71^ are improving the quantification of population movement, they do not yet provide the temporal and geographical resolution necessary for our analysis. Continued data collation and methodological advances are required to appropriately account for migration in mortality estimation. Second, we modelled death probabilities in age groups separately, because of software and computation limitations. This allowed us to avoid potential age-composition bias in small clusters, but ultimately meant ignoring a high level of correlation in these data. Future work is needed to develop methods that enable computationally efficient estimation of the under-5 survival curve simultaneously rather than approximating survival in a piece-wise manner. Third, the included set of spatial covariates does not represent the full universe of potential correlates for drivers of under-5 mortality (eg, exposure to unsafe water and sanitation) because of an absence of high-resolution spatial data or layers for these particular indicators. As these measures become available, future studies should incorporate them into modelling approaches. Fourth, to include a massive amount of polygon data in our spatially continuous model, we re-sampled polygon data to points. It is possible that this procedure introduced over-smoothing, although these effects are probably minimal given their agreement with other subnational mortality models (appendix pp 50–52). Future research will need to develop computational methods for scaling up geostatistical integration of point and polygon data to continental-scale mapping studies.^72^ Last, the potential effects of urban slums on child mortality were not explicitly quantified. Although our study offers the highest geospatial resolution of child mortality to date, the 5 × 5 km pixel-level remains too coarse to fully account for intra-city slums and disparities.

Amid impressive overall gains in decreasing under-5 mortality rates in Africa, sizeable populations within the continent have yet to experience such improvements in child survival. The SDG 3.2 targets for under-5 and neonatal mortality demand extraordinary progress in child health for Africa—and without substantial, sustained commitments to financing better access to improved health care and targeting interventions to high-burden areas, we are likely to fall short of these aims. Monitoring high-resolution trends in child mortality is a vital tool for swiftly recognising local child health needs and prioritising resources accordingly. With continued and expanded mapping efforts, collectively we can garner greater recognition of and attention to communities in which child survival remains tenuous.
