# Supplementary material for: Mapping under-5 and neonatal mortality in Africa, 2000–15: a baseline analysis for the Sustainable Development Goals
Source: Lancet. 2017 Nov 11;390(10108):2171–82. doi: 10.1016/S0140-6736(17)31758-0 (PMC5687451; doi:10.1016/S0140-6736(17)31758-0)
Supplement: Supplementary appendix [file mmc1.pdf]

THE LANCET

Supplementary appendix

This appendix formed part of the original submission and has been peer reviewed. We post it as supplied by the authors.

Supplement to: Golding N, Burstein R, Longbottom J, et al. Mapping under-5 and neonatal mortality in Africa, 2000–15: a baseline analysis for the Sustainable Development Goals. *Lancet* 2017; published online Sept 25. http://dx.doi.org/10.1016/S0140-6736(17)31758-0.

Methods appendix to Mapping under-5 and neonatal mortality in Africa, 2000–2015: a baseline analysis for the Sustainable Development Goals

This appendix provides further methodological detail, including several supplemental figures and tables, for "Mapping under-5 and neonatal mortality in Africa, 2000–2015: a baseline analysis for the Sustainable Development Goals".

Table of Contents

1. **GATHER Checklist** **5**

2. **Geographic Inclusion** **6**

3. **Mortality data sources** **7**

4. **Data outliers** **22**

5. **Tabulating CBH data** **22**

6. **Adjustment of summary birth history data** **24**
   - **6.1 Data preparation** **26**
   - **6.2 SBH adjustment models** **26**
   - **6.2.1 Model fits of SBH adjustment models** **27**
   - **6.3 Out of sample validation of SBH Adjustment Model** **30**
   - **6.4 Inverse-variance weighting of SBH observations** **31**

7. **Applying GBD bias correction** **31**

8. **Spatial integration over polygon records** **32**

9. **Raw data plots** **32**

10. **Covariates and covariate transformation** **38**
    - **10.1 Covariates** **38**
    - **10.2 Covariate selection and transformation with stacking** **39**

11. **Geostatistical model** **41**
    - **11.1 Model Geographies** **41**
    - **11.2 Model description** **41**
    - **11.3 Priors** **42**
    - **11.4 Model fitting** **42**
    - **11.5 Model Results** **42**
    - **11.6 Model validation** **46**
    - **11.6.1 Spatial aggregation** **47**
    - **11.6.2 Metrics of predictive validity** **47**

12. **Calibration to national estimates** **50**

13. **Verification and comparison against other subnational child mortality models** **51**
    - **13.1 Gavi Full Country Evaluation small area estimates** **51**
    - **13.2 GBD 2015 subnational estimates in Kenya and South Africa** **52**

14. **Source code** **53**

References **53**

Figures and Tables

Supplementary Figure 1. Countries included in mapping under-5 and neonatal mortality rates. 7
Supplementary Figure 2: Lexis diagram 23
Supplementary Figure 3: Comparison of SBH and CBH mortality. 25
Supplementary Figure 4. Out of sample fits for SBH adjustment models. 30
Supplementary Figure 5: Illustration of k-means clustering for selecting spatial integration points. 32
Supplementary Figure 6: Model input data for age bin 1 33
Supplementary Figure 7: Model input data for age bin 2 34
Supplementary Figure 8: Model input data for age bin 3 35
Supplementary Figure 9: Model input data for age bin 4 36
Supplementary Figure 10: Combined input data for age bins1-4 37
Supplementary Figure 11: Images of spatial covariates used (2015 values shown here). 39
Supplementary Figure 12: Plots illustrating stacking. 40
Supplementary Figure 13: Mean, lower, and upper credible interval predictions for under-5 mortality (5q0) in 2000, 2005, 2010, and 2015. 44
Supplementary Figure 14: Mean, lower, and upper credible interval predictions for neonatal mortality in 2000, 2005, 2010, and 2015. 45
Supplementary Figure 15: Illustration of sample size impact on the ability to retrieve true probability from empirical binomial estimates. 47
Supplementary Figure 16: Data estimates for aggregated administrative 1 level holdouts versus mean out of sample predictions for the same locations. 49
Supplementary Figure 17: Comparison of nationally aggregated population-weighted geospatial estimates, and GBD estimates for the same years. 50
Supplementary Figure 18: Comparison of district- and county-level aggregated population-weighted geospatial estimates, and Gavi-FCE estimates of under-5 mortality for the same years. 51
Supplementary Figure 19: Comparison of subnational aggregated population-weighted geospatial estimates, and GBD estimates of under-5 mortality for the same years. 52

Supplementary Table 1. Sources of mortality data used in the model. 7
Supplementary Table 2: SBH Adjustment model results for the *p* model. 27
Supplementary Table 3: SBH Adjustment model results for the *N* model. 29
Supplementary Table 4. Out-of-sample predictive validity metrics for SBH adjustment models 30
Supplementary Table 5. Description of covariate layers used and their sources. 38
Supplementary Table 6. Pairwise correlations between stacking model fits. 41
Supplementary Table 7: Model fits for each age-specific model. 43
Supplementary Table 8. Posterior expectation of the deviance for each model, both full and null, and their relative differences. 46
Supplementary Table 9. Out-of-sample predictive validity for first administrative-level holdout predictions 48
Supplementary Table 10. Out-of-sample predictive validity for second administrative-level holdout predictions 48

1. GATHER Checklist

Item #	Checklist item	Reference
**Objectives and funding**		
**1**	Define the indicator(s), populations (including age, sex, and geographic entities), and time period(s) for which estimates were made.	Main text (Methods)
**2**	List the funding sources for the work.	Main text (Acknowledgements)
**Data Inputs**		
For all data inputs from multiple sources that are synthesized as part of the study:		
**3**	Describe how the data were identified and how the data were accessed.	Main text (Methods)
**4**	Specify the inclusion and exclusion criteria. Identify all ad-hoc exclusions.	Main text (Methods); Methods appendix (Sections 2 and 4)
**5**	Provide information on all included data sources and their main characteristics. For each data source used, report reference information or contact name/institution, population represented, data collection method, year(s) of data collection, sex and age range, diagnostic criteria or measurement method, and sample size, as relevant.	Methods appendix (Supplementary Table 1) and available through ghdx.healthdata.org/
**6**	Identify and describe any categories of input data that have potentially important biases (e.g., based on characteristics listed in item 5).	Main text (Methods); Methods appendix (Section 6)
For data inputs that contribute to the analysis but were not synthesized as part of the study:		
**7**	Describe and give sources for any other data inputs.	Methods appendix (Section 10.1)
For all data inputs:		
**8**	Provide all data inputs in a file format from which data can be efficiently extracted (e.g., a spreadsheet rather than a PDF), including all relevant meta-data listed in item 5. For any data inputs that cannot be shared because of ethical or legal reasons, such as third-party ownership, provide a contact name or the name of the institution that retains the right to the data.	Available through ghdx.healthdata.org/ or on request
**Data analysis**		

**9**	Provide a conceptual overview of the data analysis method. A diagram may be helpful.	Main text (Methods);
**10**	Provide a detailed description of all steps of the analysis, including mathematical formulae. This description should cover, as relevant, data cleaning, data pre-processing, data adjustments and weighting of data sources, and mathematical or statistical model(s).	Main text (Methods); methods appendix
**11**	Describe how candidate models were evaluated and how the final model(s) were selected.	Main text (Analysis); methods appendix section 11.6)
**12**	Provide the results of an evaluation of model performance, if done, as well as the results of any relevant sensitivity analysis.	Main text (Analysis); methods appendix (Supplementary tables 9 and 10, Supplementary Figure 13)
**13**	Describe methods for calculating uncertainty of the estimates. State which sources of uncertainty were, and were not, accounted for in the uncertainty analysis.	Main text (Analysis); methods appendix (Section 11)
**14**	State how analytic or statistical source code used to generate estimates can be accessed.	Code is provided in an online repository
**Results and Discussion**		
**15**	Provide published estimates in a file format from which data can be efficiently extracted.	Raster files for spatial data and csvs of administrative level 1 and 2 estimates to be made available at ghdx.healthdata.org. PDF tables of administrative level 1 and 2 estimates in appendix.
**16**	Report a quantitative measure of the uncertainty of the estimates (e.g. uncertainty intervals).	Main text (Results; Figure 4); Appendix (Supplementary figures 13 and 14); Appendix tables
**17**	Interpret results in light of existing evidence. If updating a previous set of estimates, describe the reasons for changes in estimates.	Main text (Research in Context)
**18**	Discuss limitations of the estimates. Include a discussion of any modelling assumptions or data limitations that affect interpretation of the estimates.	Main text (Limitations)

2. Geographic Inclusion

This analysis aimed to provide high-resolution under-5 and neonatal mortality rate maps across 46 countries in Africa considered among priority countries for monitoring child mortality by the Countdown to 2015 for

Maternal, Newborn, and Child Survival.[1] To this list we added Namibia so that the resulting maps also had continuous coverage of sub-Saharan Africa, a region over which mortality rates and other development metrics are often aggregated.

It was not possible to extend the analysis to include Algeria, Tunisia, Libya, or Western Sahara due to a lack of subnational mortality data from these countries. Cape Verde, Comoros, Mauritius, and São Tomé and Príncipe are each geographically isolated from included countries and cover very small geographic areas, so including these countries in the analysis would provide little advance over existing national estimates. The map of 46 included countries is shown in Supplementary Figure 1.

Supplementary Figure 1. Countries included in mapping under-5 and neonatal mortality rates.

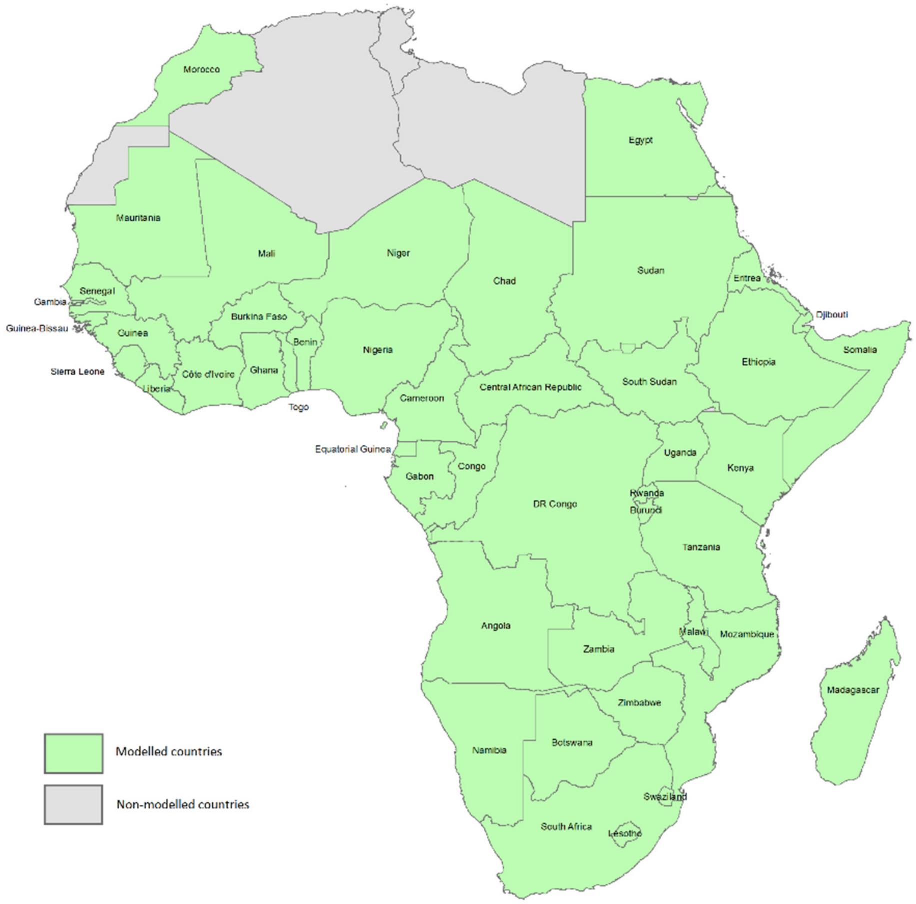

3. Mortality data sources

Supplementary Table 1. Sources of mortality data used in the model. The datasets and reports from which complete birth history (CBH) and summary birth history (SBH) were extracted for each country are detailed under Citation, and the institutions that provided these data are given under Source. Further details are given marked for those sources labelled † as follows: ICF International. 2004-2012. Demographic Health Surveys (various). Calverton, Maryland: ICF International, 2012. CBH surveys marked with * included both

CBH and SBH data and were used in the SBH adjustment model described in section 6. Admin = Administrative level

Country	Year	Data Type	Source	Geographic level	Citation
**Angola**	2011	CBH*	DHS Program†	Point	Cosep Consultoria, Consaúde & ICF International. Angola Malaria Indicator Survey 2011 [Dataset] AOBR61DT. (ICF International [Distributor], 2011, Calverton, Maryland, USA, 2011).
**Angola**	2009	SBH	Discussion Forum of the Fifth National Conference of Civil Society (Angola)	Admin 1	Ministerio do Planeamento. Integrated Survey on the Welfare of Population 218pp (Luanda, Angola, 2011).
**Angola**	2007	CBH	DHS Program†	Point	Consultoria de Serviços e Pesquisas–COSEP Lda, Consultoria de Gestão e Administração em Saúde–Consaúde Lda (Angola) & Macro International Inc. Angola Malaria Indicator Survey 2006-07 [Dataset] AOIR51DT. 112pp (ICF International [Distributor], Calverton, Maryland, USA, 2007).
**Angola**	2001	SBH	UNICEF	Admin 1	National Institute of Statistics & United Nations Children's Fund. MICS Multiple Indicator Cluster Survey - Assessing the Situation of Angolan Children and Women at the Beginning of the Millennium [Dataset]. 142pp (Luanda, Angola, 2003).
**Benin**	2012	CBH*	DHS Program†	Point	Institut National de la Statistique et de l'Analyse Économique & ICF International. Enquête Démographique et de Santé du Bénin 2011-2012 [Dataset] BJBR61DT. (ICF International [Distributor], Calverton, Maryland, USA, 2013)
**Benin**	2006	CBH*	DHS Program†	Admin 1	Institut National de la Statistique et de l'Analyse Économique & Macro International Inc. Enquête Démographique et de Santé (EDSB-III) - Bénin 2006 [Dataset] BJBR51DT. (ICF International [Distributor], Calverton, Maryland, USA, 2007).
**Benin**	2001	CBH*	DHS Program†	Point	Institut National de la Statistique et de l'Analyse Économique & ORC Macro. Enquête Démographique et de Santé au Bénin 2001 [Dataset] BJBR41DT. (ICF International [Distributor], Calverton, Maryland, USA, 2002).
**Botswana**	2011	SBH	IPUMS INTERNATIONAL	Admin 1	Central Statistics Office (Botswana) & Minnesota Population Center. Botswana Population and Housing Census 2011 from the Integrated Public Use Microdata Series, International: [Machine-readable database]. (University of Minnesota, Minneapolis, USA, 2017).
**Botswana**	2008	CBH	Central Statistics Office (Botswana)	Admin 2	Central Statistics Office (Botswana). Botswana Family Health Survey 2007-2008 [Dataset]. (Gaborone, Botswana, 2009).
**Botswana**	2006	SBH	Central Statistics Office (Botswana)	Admin 2	Central Statistics Office (Botswana). Botswana Demographic Survey 2006 [Dataset]. (Gaborone, Botswana, 2006).
**Botswana**	2001	SBH	IPUMS INTERNATIONAL	Admin 1	Central Statistics Office (Botswana) & Minnesota Population Center. Botswana Population and Housing Census 2001 from the Integrated Public Use Microdata Series, International: [Machine-readable database]. (University of Minnesota, Minneapolis, USA, 2017).
**Botswana**	2000	SBH	UNICEF	Admin 1	Central Statistics Office (Botswana) & United Nations Children's Fund (UNICEF). Botswana Multiple Indicator Cluster Survey 2000 [Dataset]. (New York, USA, 2015).
**Burkina Faso**	2014	SBH	DHS Program†	Point	Institut National de la Statistique et de la Démographie, Programme National de Lutte contre le Paludisme Ouagadougou Burkina Faso & ICF International. Enquête sur les Indicateurs du Paludisme au Burkina Faso (EIPBF) 2014. 170pp (Rockville, Maryland, USA, 2015).
**Burkina Faso**	2011	CBH*	DHS Program†	Point	Institut National de la Statistique et de la Démographie & ICF International. Enquête Démographique et de Santé et à Indicateurs Multiples du Burkina Faso 2010 [Dataset] BFBR62DT. (ICF International [Distributor], Calverton, Maryland, USA, 2012).
**Burkina Faso**	2006	SBH	IPUMS INTERNATIONAL	Admin 3	Minnesota Population Center & National Institute of Statistics and Demography (Burkina Faso). Burkina Faso Population and Housing Census 2006 from the Integrated Public Use Microdata Series, International: [Machine-readable database]. (University of Minnesota, Minneapolis, USA, 2013).

Country	Year	Data Type	Source	Geographic level	Citation
**Burkina Faso**	2006	SBH	UNICEF	Point	United Nations Children's Fund & Institut National de la Statistique et la Demographie. Burkina Faso Enquete par Grappes a Indicateurs Muliples 2006 [Dataset]. (United Nations Children's Fund, 2008).
**Burkina Faso**	2003	CBH*	DHS Program†	Point	Institut National de la Statistique et de la Démographie & ORC Macro. Enquête Démographique et de Santé du Burkina Faso 2003 [Dataset] BFBR43DT. (ICF International [Distributor], Calverton, Maryland, USA, 2004).
**Burkina Faso**	1999	CBH*	DHS Program†	Point	Institut National de la Statistique et de la Démographie & Macro International Inc. Enquête Démographique et de Santé, Burkina Faso 1998-1999 [Dataset] BFBR31DT. (ICF International [Distributor], Calverton, Maryland, USA, 2000).
**Burundi**	2013	SBH	DHS Program†	Point	Institut de Statistiques et d'Études Économiques du Burundi, Ministère de la Santé Publique et de la Lutte contre le Sida (Burundi) & ICF International. Enquête sur les Indicateurs du Paludisme Burundi 2012 [Dataset] BUHR6HSV. 138pp (ICF International [Distributor], Bujumbura, Burundi, 2013).
**Burundi**	2011	CBH*	DHS Program†	Point	Institut de Statistiques et d'Études Économiques du Burundi, Ministère de la Santé Publique et de la Lutte contre le Sida (Burundi) & ICF International. Enquête Démographique et de Santé Burundi 2010 [Dataset] BUBR61DT. (ICF International [Distributor], Bujumbura, Burundi, 2012).
**Burundi**	2005	SBH	UNICEF	Point	Institut de Statistiques et d'Études Économiques du Burundi. Enquête Nationale d'Évaluation des Conditions de vie de l'Enfant et de la Femme au Burundi-2005 [Dataset]. 192pp (Bujumbura, Burundi, 2008).
**Burundi**	2000	SBH	UNICEF	Admin 1	Buzingo, D., Habimana, F. & Nduwabika, N. Enquête Nationale d'Evaluation des Conditions de vie de l'Enfant et de la Femme au Burundi (ENECEF-BURUNDI 2000) [Dataset]. 44pp (Burundi, 2001).
**Burundi**	1999	SBH	World Bank	Admin 1	Vincent, R. Enquete Prioritaire 1998 Etude Nationale sur les Conditions de vie des Populations. 119pp (Bujumbura, Burundi, 2001).
**Cameroon**	2011	CBH*	DHS Program†	Point	Institut National de la Statistique & ICF. International. Enquête Démographique et de Santé et à Indicateurs Multiples du Cameroun 2011 [Dataset] CMBR60DT. (ICF International [Distributor], Calverton, Maryland, USA, 2012).
**Cameroon**	2005	SBH	IPUMS INTERNATIONAL	Admin 3	Minnesota Population Center, National Institute of Statistics (Cameroon) & Central Bureau of the Census and Population Studies (Cameroon). Cameroon Population and Housing Census 2005 from the Integrated Public Use Microdata Series, International: [Machine-readable database]. (University of Minnesota, Minneapolis, USA, 2013).
**Cameroon**	2004	CBH*	DHS Program†	Point	Institut National de la Statistique & ORC Macro. Enquête Démographique et de Santé du Cameroun 2004 [Dataset] CMBR44DT. (ICF International [Distributor], Calverton, Maryland, USA, 2004).
**Cameroon**	2000	SBH	UNICEF	Admin 1	Fonds des Nations Unies pour l'Enfrance & Ministère de l'Economie et des Finances Gouvernement du Cameroun. Enquête à Indicateurs Multiples (MICS) au Cameroun 2000 [Dataset]. 45 (Cameroun, 2002).
**Cameroon**	1998	CBH*	DHS Program†	Admin 1	Fotso, M. et al. Enquête Démographique et de Santé, Cameroun 1998 [Dataset] CMBR31FL. (Bureau Central des Recensements et des Études de Population & Macro International Inc [Distributor], Calverton, Maryland, USA, 1999).
**Central African Republic**	2011	SBH	UNICEF	Admin 1	Institut Centrafricain des Statistiques et des Études Économiques et Sociales. Enquête par grappes à indicateurs multiples – MICS couplée avec la sérologie VIH, RCA, 2010 Rapport final [Dataset]. 229pp (Bangui, République Centrafricaine, 2012).
**Central African Republic**	2006	SBH	UNICEF	Admin 1	Institut Centrafricain des Statistiques et des Etudes Economiques et Sociales. Résultats de l'enquête nationale à indicateurs multiples couplée avec la sérologie VIH et anémie en RCA 2006 [Dataset]. 378pp (Bangui, République Centrafricaine, 2009).
**Central African Republic**	2000	SBH	UNICEF	Admin 1	United Nations Children's Fund & Ministere du Plan et de la Cooperation Internationale. Enquete a Indicateurs Multiples - MICS 2000 Rapport Final [Dataset]. 304pp (Bangui République Centrafricaine, 2001).
**Chad**	2015	CBH*	DHS Program†	Point	National Institute of Statistical Economic and Demographic Studies (Chad) & ICF International. Chad Demographic and Health Survey 2014-2015 [Dataset] TDBR71DT. (ICF International [Distributor], Fairfax, USA, 2016).

Country	Year	Data Type	Source	Geographic level	Citation
**Chad**	2010	SBH	UNICEF	Admin 2	Institut national de la statistique des études économiques et démographiques. Enquête par grappes à indicateurs multiples Tchad 2010 [Dataset]. 364pp (Chad, 2011).
**Chad**	2004	CBH*	DHS Program†	Admin 1	Ouagadjio, B. et al. Enquête Démographique et de Santé Tchad 2004 [Dataset] TDBR41DT. (ICF International [Distributor], Calverton, Maryland, USA, 2004).
**Chad**	2000	SBH	UNICEF	Admin 1	Direction de la Statistique des Etudes Economiques et Démographiques. Enquete par grappes a indicateurs multiples - Rapport complet [Dataset]. 117pp (Chad, 2001).
**Congo**	2012	CBH*	DHS Program†	Admin 1	Centre Nationale de la Statistique et des Études Économiques (Congo) & ICF International. Enquête Démographique et de Santé du Congo (EDSC-II) 2011-2012 [Dataset] CGBR60DT. (ICF International [Distributor], Calverton, Maryland, USA 2013).
**Congo**	2009	SBH	DHS Program†	Admin 1	Centre National de la Statistique et des Études Économiques & ICF Macro. Enquête de Séroprévalence et sur les Indicateurs du Sida – Congo 2009 [Dataset] CDBR61DT. (ICF Macro [Distributor], Calverton, Maryland, USA, 2009).
**Congo**	2005	CBH*	DHS Program†	Admin 1	Centre National de la Statistique et des Études Économiques & ORC Macro. Enquête Démographique et de Santé du Congo 2005 [Dataset] CGBR51DT. (ICF International [Distributor], Calverton, Maryland, USA 2006).
**Cote d'Ivoire**	2012	CBH*	DHS Program†	Point	Institut National de la Statistique & ICF International. Enquête Démographique et de Santé et à Indicateurs Multiples de Côte d'Ivoire 2011-2012 [Dataset] CIBR61DT. (ICF International [Distributor], Calverton, Maryland, USA, 2012).
**Cote d'Ivoire**	2005	CBH	DHS Program†	> Admin 1	Institut National de la Statistique, Ministère de la Lutte contre le Sida (Côte d'Ivoire) & Macro, O. Enquête sur les Indicateurs du Sida, Côte d'Ivoire 2005 [Dataset] CIBR50DT. 283pp (ICF International [Distributor], Calverton, Maryland, USA, 2006).
**Cote d'Ivoire**	1999	CBH*	DHS Program†	Point	Institut National de la Statistique (Côte d'Ivoire) & ORC Macro. Enquête Démographique et de Santé, Côte d'Ivoire 1998-1999 [Dataset] CIBR3ADT. (ICF International [Distributor], Calverton, Maryland, USA, 2001).
**Djibouti**	2012	CBH	Pan Arab Project for Family Health (PAPFAM)	Admin 1	Department of Statistics and Demographic Studies (Djibouti), League of Arab States, Ministry of Health (Djibouti), Pan Arab Project for Family Health (PAPFAM). Djibouti Family Health Survey 2012 [Dataset]. (2002).
**Djibouti**	2006	SBH	UNICEF	Point & Admin 2	Ministry of Economy Finance and Planning in charge of Privatization (Djibouti), Ministry of Health (Djibouti) & United Nations Children's Fund. Djibouti Multiple Indicator Cluster Survey 2006 [Dataset]. 210pp (New York, USA, 2006).
**Djibouti**	2002	CBH	Pan Arab Project for Family Health (PAPFAM)	National	Department of Statistics and Demographic Studies (Djibouti), League of Arab States, Ministry of Health (Djibouti) & Pan Arab Project for Family Health. Djibouti Family Health Survey 2002 [Dataset]. (2002).
**DR Congo**	2013	CBH*	DHS Program†	Point	Ministère du Plan et Suivi de la Mise en œuvre de la Révolution de la Modernité, Ministère de la Santé Publique & ICF International. Enquête Démographique et de Santé en République Démocratique du Congo 2013-2014 [Dataset] CDBR61DT. (ICF International [Distributor], Rockville, Maryland, USA, 2014).
**DR Congo**	2010	SBH	UNICEF	Admin 1	Institut National de la Statistique & Fonds des Nations Unies pour l'Enfrance. Enquete par Grappes a Indicateurs Multiples en Republique du Congo (MICS-RDC 2010) [Dataset]. 384pp (Republique Democratique du Congo, 2011).
**DR Congo**	2007	CBH*	DHS Program†	Point	Ministère du Plan & Macro International. Enquête Démographique et de Santé, République Démocratique du Congo 2007 [Dataset] CDBR50DT. (ICF International [Distributor], Calverton, Maryland, USA, 2008).
**DR Congo**	2001	SBH	UNICEF	Admin 1	Ministère du Plan et de la Reconstruction, Fonds des Nations Unies pour l'Enfance & Agence des Etats-Unis pour le Développement International. Enquête Nationale Sur la Situation des Enfants et Des Femmes MICS2/2001 [Dataset]. 258pp (Kinshasa, Democratic Republic of the Congo, 2002).
**Egypt**	2015	SBH	DHS Program†	Admin 1	El-Zanaty and Associates, ICF International, Ministry of Health and Population (Egypt) & National Population Council (Egypt). Egypt Special Demographic and Health Survey 2015 [Dataset] EGIQ73DT. (ICF International [Distributor], Fairfax, USA, 2015).

Country	Year	Data Type	Source	Geographic level	Citation
**Egypt**	2014	CBH*	DHS Program†	Point	Ministry of Health and Population (Egypt), El-Zanaty and Associates (Egypt) & ICF International. Egypt Demographic and Health Survey 2014 [Dataset] EGBR61DT. (ICF International [Distributor], Cairo, Egypt and Rockville, Maryland, USA, 2015).
**Egypt**	2008	CBH*	DHS Program†	Point	El-Zanaty, F. & Way, A. Egypt Demographic and Health Survey 2008 [Dataset] EGBR5ADT. (ICF International [Distributor], Cairo, Egypt, 2009).
**Egypt**	2005	CBH*	DHS Program†	Point	El-Zanaty, F. & Way, A. Egypt Demographic and Health Survey 2005 [Dataset] EGBR51DT. (ICF International [Distributor], Cairo, Egypt, 2006).
**Egypt**	2003	CBH*	DHS Program†	Point	El-Zanaty, F. & Way, A. A. 2003 Egypt Interim Demographic and Health Survey [Dataset] EGBR4ADT. (ICF International [Distributor], Cairo, Egypt, 2004).
**Egypt**	2000	CBH*	DHS Program†	Point	El-Zanaty, F. & Way, A. Egypt Demographic and Health Survey 2000 [Dataset] EGBR42DT. (ICF International [Distributor], Calverton, Maryland, USA, 2001).
**Equatorial Guinea**	2000	SBH	UNICEF	Admin 1	Ministry of Planning Economic Development and Public Investment (Equatorial Guinea) & United Nations Children's Fund. Encuesta de Indicadores Multiples (MICS 2000) [Dataset]. 79pp (Malabo, Equatorial Guinea, 2001).
**Eritrea**	2002	CBH*	DHS Program†	Admin 1	National Statistics and Evaluation Office (Eritrea) & ORC Macro. Eritrea Demographic and Health Survey 2002 [Dataset] 331pp (Calverton, Maryland, USA, 2003).
**Ethiopia**	2011	CBH*	DHS Program†	Point	Central Statistical Agency (Ethiopia) & ICF International. Ethiopia Demographic and Health Survey 2011 [Dataset] ETBR61DT. (ICF International [Distributor], Addis Ababa, Ethiopia and Calverton, Maryland, USA, 2012).
**Ethiopia**	2008	CBH	Ministry of Health (Ethiopia)	Admin 2	Ethiopian Health and Nutrition Research Center (EHNRI), Macro International, Inc, Ministry of Health (Ethiopia). Ethiopia Global Fund Household Health Coverage Survey 2008 [Dataset]. (2008).
**Ethiopia**	2007	SBH	IPUMS INTERNATIONAL	Point & Admin 3	Central Statistical Agency (Ethiopia) & Minnesota Population Center. Ethiopia Population and Housing Census 2007 from the Integrated Public Use Microdata Series, International: [Machine-readable database]. (University of Minnesota, Minneapolis, USA, 2015).
**Ethiopia**	2005	CBH	DHS Program†	Point	Central Statistical Agency (Ethiopia) & ORC Macro. Ethiopia Demographic and Health Survey 2005 [Dataset] ETBR51DT. (ICF International [Distributor], Addis Ababa, Ethiopia and Calverton, Maryland, USA, 2006).
**Ethiopia**	2000	CBH	DHS Program†	Point	Central Statistical Authority (Ethiopia) & ORC Macro. Ethiopia Demographic and Health Survey 2000 [Dataset] ETBR41DT. (ICF International [Distributor], Addis Ababa, Ethiopia and Calverton, Maryland, USA, 2001).
**Gabon**	2012	CBH*	DHS Program†	Point	Direction Générale de la Statistique & ICF International. Enquête Démographique et de Santé du Gabon 2012 [Dataset] GABR60DT. (ICF International [Distributor], Calverton, Maryland, USA and Libreville, Gabon, 2013).
**Gabon**	2001	CBH*	DHS Program†	Admin 2	Direction Générale de la Statistique, Des Études Économiques (Gabon) & ORC Macro. Enquête Démographique et de Santé Gabon 2000 [Dataset] GABR41DT. 385pp (ICF International [Distributor], Calverton, Maryland, USA, 2001).
**Gambia**	2013	CBH*	DHS Program†	Admin 2	The Gambia Bureau of Statistics & ICF International. The Gambia Demographic and Health Survey 2013 [Dataset] GMBR60DT. (ICF International [Distributor], Banjul, The Gambia and Rockville, Maryland, USA, 2014).
**Gambia**	2006	SBH	UNICEF	Admin 2	Gambia Bureau of Statistics & United Nations Children's Fund. The Gambia Multiple Indicator Cluster Survey 2005-2006 [Dataset]. 263pp (Bakau, The Gambia, 2007).
**Gambia**	2000	SBH	UNICEF	Admin 1	Central Statistics Department (Gambia) & United Nations Children's Fund. The Gambia Multiple Indicator Cluster Survey Report, 2000 [Dataset]. 116pp (New York, USA, 2002).
**Ghana**	2014	CBH*	DHS Program†	Point	Ghana Statistical Service, Ghana Health Service & ICF International. Ghana Demographic and Health Survey 2014 [Dataset] GHBR71DT. 530pp (ICF International [Distributor], Rockville, Maryland, USA, 2015).
**Ghana**	2011	CBH*	UNICEF	Point	Ghana Statistical Service. Ghana Multiple Indicator Cluster Survey with an Enhanced Malaria Module and Biomarker, 2011 [Dataset]. (Accra, Ghana, 2012).

Country	Year	Data Type	Source	Geographic level	Citation
**Ghana**	2011	SBH	UNICEF	Point	Institute of Statistical Social and Economic Research & United Nations Children's Fund. Ghana-Accra, Multiple Indicator Cluster Survey in 5 High Densly Populated Localities, 2010-2011 [Dataset]. 273pp (Institute of Statistical Social and Economic Research, Accra, Ghana, 2012).
**Ghana**	2010	SBH	IPUMS INTERNATIONAL	Admin 2	Ghana Statistical Service & Minnesota Population Center. Ghana Census 2010 from the Integrated Public Use Microdata Series, International: [Machine-readable database]. (University of Minnesota, Minneapolis, USA).
**Ghana**	2008	CBH	DHS Program†	Admin 4	Ghana Statistical Service, Ghana Health Service & Macro International. Ghana Maternal Health Survey 2007 [Dataset] GHIQ51DT. 243pp (ICF International [Distributor], Calverton, Maryland, USA, 2009).
**Ghana**	2008	CBH*	DHS Program†	Point	Ghana Statistical Service, Ghana Health Service & ICF Macro. Ghana Demographic and Health Survey 2008 [Dataset] GHBR5ADT. (ICF International [Distributor], Accra, Ghana, 2009).
**Ghana**	2006	SBH	UNICEF	Admin 1	Ministry of Health (Ghana), Ghana Statistical Service, and United Nations Children's Fund. Ghana Multiple Indicator Cluster Survey 2006 [Dataset]. United Nations Children's Fund, New York, USA, pp 273
**Ghana**	2005	SBH	Ghana Statistical Service	Admin 1	Ghana Statistical Service. Ghana Living Standards Measurement Survey 2005-2006 [Dataset]. (Accra, Ghana, 2006).
**Ghana**	2003	CBH*	DHS Program†	Point	Ghana Statistical Service, Noguchi Memorial Institute for Medical Research & ORC Macro International. Ghana Demographic and Health Survey 2003 [Dataset] GHBR4BDT. (ICF International [Distributor], Calverton, Maryland, USA, 2004).
**Ghana**	2000	SBH	IPUMS INTERNATIONAL	Admin 2	Ghana Statistical Service & Minnesota Population Center. Ghana Population and Housing Census 2000 from the Integrated Public Use Microdata Series, International: [Machine-readable database]. (University of Minnesota, Minneapolis, USA).
**Ghana**	1999	CBH*	DHS Program†	Point	Ghana Statistical Service & Macro International Inc. Ghana Demographic and Health Survey 1998 [Dataset] GHBR41DT. (ICF International [Distributor], Calverton, Maryland, USA, 1999).
**Ghana**	1999	SBH	Ghana Statistical Service	Admin 2	Ghana Statistical Service. Ghana Living Standards Survey 1998-1999 [Dataset]. (1999).
**Guinea**	2012	CBH*	DHS Program†	Point	Institut National de la Statistique (Guinée) & ICF International. Enquête Démographique et de Santé et à Indicateurs Multiples (EDS-MICS) [Dataset] GNBR61DT. (ICF International [Distributor], Calverton, Maryland, USA, 2013).
**Guinea**	2005	CBH*	DHS Program†	Point	Direction Nationale de la Statistique (Guinée) & ORC Macro. Enquête Démographique et de Santé, Guinée 2005 [Dataset] GNBR52DT. (ICF International [Distributor], Calverton, Maryland, USA, 2006).
**Guinea**	1999	CBH*	DHS Program†	Point	Direction Nationale de la Statistique (Guinée) & Macro International Inc. Enquête Démographique et de Santé, Guinée 1999 [Dataset] GNBR41DT. (ICF International [Distributor], Calverton, Maryland, USA, 2000).
**Guinea Bissau**	2014	CBH*	UNICEF	Admin 1	National Statistics Institute (Guinea-Bissau) & United Nations Children's Fund (UNICEF). Guinea-Bissau Multiple Cluster Indicator Survey 2014 [Dataset]. (New York, USA, 2016).
**Guinea Bissau**	2006	SBH	UNICEF	Admin 1	Ministère de l`Economie - Secrétariat d`Etat du Plan et à l`Intégration Régionale. Enquête par Grappes à Indicateurs Multiples, Guinée-Bissau, 2006 [Dataset]. 259pp (Bissau, Guinée-Bissau, 2006).
**Guinea Bissau**	2000	SBH	UNICEF	Admin 1	Secretary State of Planning, National Institute of Statistics and Census & United Nations Children's Fund. Guinea-Bissau Multiple Indicator Cluster Survey 2000 [Dataset]. 31pp (New York, USA, 2000).
**Kenya**	2015	SBH	DHS Program†	Point	National Malaria Control Programme, Ministry of Health, Kenya National Bureau of Statistics & ICF International. Kenya Malaria Indicator Survey 2015 [Dataset] KEIR7HDT. 165pp (ICF International [Distributor], Nairobi, Kenya, and Rockville, Maryland, USA, 2016).
**Kenya**	2014	CBH*	DHS Program†	Point	Kenya National Bureau of Statistics et al. Kenya Demographic and Health Survey 2014 [Dataset] KEBR70DT. (ICF International [Distributer], Rockville, Maryland, USA, 2015).

Country	Year	Data Type	Source	Geographic level	Citation
**Kenya**	2014	CBH*	UNICEF	Point	Kenya National Bureau of Statistics, Population Studies and Research Institute, University of Nairobi (Kenya), United Nations Children's Fund (UNICEF). Kenya - Bungoma County Multiple Indicator Survey 2013-2014 [Dataset]. (New York, USA, 2015).
**Kenya**	2014	CBH*	UNICEF	Point	Kenya National Bureau of Statistics, Population Studies and Research Institute, University of Nairobi (Kenya), United Nations Children's Fund (UNICEF). Kenya - Kakamega County Multiple Indicator Survey 2013-2014 [Dataset]. (New York, USA, 2015).
**Kenya**	2014	CBH*	UNICEF	Point	Kenya National Bureau of Statistics, Population Studies and Research Institute, University of Nairobi (Kenya), United Nations Children's Fund (UNICEF). Kenya - Turkana County Multiple Indicator Survey 2013-2014 [Dataset]. (New York, USA, 2015).
**Kenya**	2011	CBH*	UNICEF	Point	Kenya National Bureau of Statistics. Nyanza Province Multiple Indicator Cluster Survey 2011 [Dataset]. (Kenya National Bureau of Statistics, Nairobi, Kenya, 2013).
**Kenya**	2009	CBH*	DHS Program†	Point	Kenya National Bureau of Statistics & ICF Macro. Kenya Demographic and Health Survey 2008-09 [Dataset] KEBR52DT. (ICF International [Distributor], Calverton, Maryland, USA, 2010).
**Kenya**	2009	SBH	IPUMS INTERNATIONAL	Admin 1	Minnesota Population Center & Kenya National Bureau of Statistics. Kenya Population Census 2009 from the Integrated Public Use Microdata Series, International: [Machine-readable database]. (University of Minnesota, Minneapolis, USA, 2013).
**Kenya**	2009	SBH	Kenya National Bureau of Statistics	Admin 2	Kenya National Bureau of Statistics, USAID, United Nations Population Fund (UNFPA), United States Census Bureau. Kenya Population and Housing Census 2009. (Kenya National Bureau of Statistics, Nairobi, Kenya 2010).
**Kenya**	2008	CBH	UNICEF	Point	United Nations Children's Fund & Kenya National Bureau of Statistics. Kenya - Eastern Province Multiple Indicator Cluster Survey 2008 [Dataset]. (Kenya National Bureau of Statistics, Nairobi, Kenya, 2009).
**Kenya**	2007	SBH	Kenya National Bureau of Statistics	Point	National AIDS/STI Control Programme. Kenya AIDS Indicator Survey: Final Report. 384 (Nairobi, Kenya, 2009).
**Kenya**	2007	SBH	Kenya National Bureau of Statistics	Point	Centers for Disease Control and Prevention (CDC), KEMRI Wellcome Trust Research Programme (KWTRP), Kenya National Bureau of Statistics, Ministry of Public Health and Sanitation (Kenya), National Coordinating Agency for Population and Development (Kenya), Population Services International (PSI). Kenya Malaria Indicator Survey 2007 [Dataset].
**Kenya**	2007	SBH	UNICEF	Point	United Nations Children's Fund & Kenya National Bureau of Statistics. Kenya - North Eastern Province Multiple Indicator Cluster Survey 2007 [Dataset]. (Kenya National Bureau of Statistics, Nairobi, Kenya).
**Kenya**	2006	SBH	Central Bureau of Statistics (Kenya)	Point	Central Bureau of Statistics (Kenya), UK Department for International Development (DFID), United States Agency for International Development (USAID), European Union (EU), Danish International Development Agency (DANIDA), World Bank (WB), et al. Kenya Integrated Household Budget Survey 2005-2006 [Dataset]. (Nairobi, Kenya).
**Kenya**	2003	CBH*	DHS Program†	Point	Central Bureau of Statistics (Kenya), Ministry of Health (Kenya) & ORC Macro. Kenya Demographic and Health Survey 2003 [Dataset] KEBR42DT. (ICF International [Distributor], Calverton, Maryland, USA, 2004).
**Kenya**	2000	SBH	UNICEF	Point	Central Bureau of Statistics (Kenya) & United Nations Children's Fund. Kenya Multiple Indicator Cluster Survey 2000 [Dataset]. 31pp (New York, USA, 2000).
**Kenya**	1999	SBH	IPUMS INTERNATIONAL	Admin 2	Central Bureau of Statistics (Kenya) & Minnesota Population Center. Kenya Population and Housing Census 1999 from the Integrated Public Use Microdata Series, International: [Machine-readable database]. (University of Minnesota, Minneapolis, USA, 2001).
**Kenya**	1999	SBH	Central Bureau of Statistics (Kenya)	Point	Central Bureau of Statistics (Kenya), UK Department for International Development (DFID), United Nations Development Programme (UNDP), United Nations Population Fund (UNFPA), United States Agency for International Development (USAID). Kenya Population and Housing Census 1999 [Dataset].

Country	Year	Data Type	Source	Geographic level	Citation
**Kenya**	1998	CBH*	DHS Program†	Point	National Council for Population and Development, Central Bureau of Statistics, Office of the Vice President and Ministry of Planning and National Development (Kenya) & Macro International Inc. Kenya Demographic and Health Survey 1998 [Dataset] KEBR3ADT. (ICF International [Distributor], Calverton, Maryland, USA, 1999).
**Lesotho**	2014	CBH*	DHS Program†	Point	Ministry of Health and Social Welfare (Lesotho) & ICF International. Lesotho Demographic and Health Survey 2014 [dataset] LSBR71DT. (ICF International [Distributor], Fairfax, USA, 2016).
**Lesotho**	2010	CBH*	DHS Program†	Point	Ministry of Health and Social Welfare (Lesotho) & ICF Macro. Lesotho Demographic and Health Survey 2009 [Dataset] LSBR60DT. (ICF International [Distributor], Maseru, Lesotho, 2010).
**Lesotho**	2005	CBH*	DHS Program†	Point	Ministry of Health and Social Welfare (Lesotho), Bureau of Statistics (Lesotho) & ORC Macro. Lesotho Demographic and Health Survey 2004 [Dataset] LSBR41DT. (ICF International [Distributor], Calverton, Maryland, USA, 2005).
**Lesotho**	2000	SBH	UNICEF	Admin 1	Bureau of Statistics (Lesotho) & United Nations Children's Fund. 2000 End Decade Multiple Indicator Cluster Survey (EMICS) [Dataset]. 100pp (New York, USA, 2002).
**Liberia**	2013	CBH*	DHS Program†	Point	Liberia Institute of Statistics and Geo-Information Services, Ministry of Health and Social Welfare (Liberia), National AIDS Control Program (Liberia) & ICF International. Liberia Demographic and Health Survey 2013 [Dataset] LBBR6ADT. (ICF International [Distributor], Monrovia, Liberia, 2014).
**Liberia**	2011	SBH	DHS Program†	Point	National Malaria Control Program (Liberia), Ministry of Health and Social Welfare, Liberia Institute of Statistics and Geo-Information Services & ICF International. Liberia Malaria Indicator Survey 2011 [Dataset] LBBR61DT. 124pp (ICF International [Distributor], Monrovia, Liberia, 2012).
**Liberia**	2009	CBH*	DHS Program†	Point	National Malaria Control Program (Liberia), Ministry of Health and Social Welfare, Liberia Institute of Statistics and Geo-Information Services & ICF Macro. Liberia Malaria Indicator Survey 2009 [Dataset] LBBR5ADT. (ICF International [Distributor], Monrovia, Liberia, 2009).
**Liberia**	2008	SBH	IPUMS INTERNATIONAL	Admin 2	Liberia Institute for Statistics and Geo-information Services & Minnesota Population Center. Liberia Census 2008 from the Integrated Public Use Microdata Series, International: [Machine-readable database]. (University of Minnesota, Minneapolis, USA).
**Liberia**	2007	CBH*	DHS Program†	Point	Liberia Institute of Statistics and Geo-Information Services (Liberia), Ministry of Health and Social Welfare (Liberia), National AIDS Control Program (Liberia) & Macro International Inc. Liberia Demographic and Health Survey 2007 [Dataset] LBBR51DT. (ICF International [Distributor], Monrovia, Liberia, 2008).
**Madagascar**	2016	SBH	DHS Program†	Admin 1	ICF International, Ministry of Public Health (Madagascar), National Institute of Statistics (Madagascar), National Program for the Fight Against Malaria (PNLP) (Madagascar), Pasteur Institute of Madagascar (IPM). Madagascar Malaria Indicator Survey 2016 [Dataset]. (ICF International [Distributor], Fairfax, USA, 2017).
**Madagascar**	2013	SBH	DHS Program†	Point	Institut National de la Statistique, Programme National de lutte contre le Paludisme, Institut Pasteur de Madagascar & ICF International. Enquête sur les Indicateurs du Paludisme (EIPM) 2013 [Dataset] MDBR6HDT. 179pp (ICF International [Distributor], Calverton, Maryland, USA, 2013).
**Madagascar**	2012	CBH*	UNICEF	Point	Institut National de la Statistique & Fonds des Nations Unies pour l’enfance. Madagascar Sud Enquête par Grappes à Indicateurs Multiples (MICS) 2012 [Dataset]. (Fonds des Nations Unies pour l’enfance (UNICEF), New York, USA, 2013).
**Madagascar**	2011	SBH	DHS Program†	Point	Institut National de la Statistique, Programme National de lutte contre le Paludisme, Institut Pasteur de Madagascar & ICF International. Enquête sur les Indicateurs du Paludisme à Madagascar (EIPMD) 2011 [Dataset] MDBR61DT. (ICF International [Distributor], Calverton, Maryland, USA, 2012).
**Madagascar**	2009	CBH*	DHS Program†	Point	Institut National de la Statistique & ICF Macro. Enquête Démographique et de Santé de Madagascar 2008-2009 [Dataset] MDBR51DT. (ICF International [Distributor], Antananarivo, Madagascar, 2010).
**Madagascar**	2004	CBH*	DHS Program†	Admin 2	Mariko, S. & Rabeza, V. Enquête de Base sur la Santé de la Reproduction et la Survie des Enfants dans les zones d’intervention USAID, à Madagascar - EBSRSE 2003-2004 [Dataset] MDBR41DT. (ICF International [Distributor], Calverton, Maryland, USA 2005).

Country	Year	Data Type	Source	Geographic level	Citation
**Madagascar**	2000	SBH	UNICEF	Admin 1	Institut National de la Statistique & Fonds des Nations Unies pour l'Enfrance. Enquete a Indicateurs Multiples MICS 2000 Madagascar [Dataset]. 237pp (New York, USA, 2001).
**Malawi**	2016	CBH	DHS Program†	Point	ICF International, Ministry of Health (Malawi), National Statistical Office of Malawi. Malawi Demographic and Health Survey 2015-2016 [Dataset]. (ICF International [Distributor], Fairfax, USA, 2017).
**Malawi**	2014	CBH*	UNICEF	Admin 2	National Statistical Office. Malawi MDG Endline Survey 2014. 684pp (Zomba, Malawi, 2015).
**Malawi**	2014	SBH	DHS Program†	Point	National Malaria Control Programme (Malawi) & ICF International. Malawi Malaria Indicator Survey (MIS) 2014 [Dataset] MWIR71DT. 124pp (ICF International [Distributor], Lilongwe, Malawi, and Rockville, Maryland, USA, 2014).
**Malawi**	2012	SBH	DHS Program†	Point	National Malaria Control Programme (Malawi) & ICF International. Malawi Malaria Indicator Survey (MIS) 2012 [Dataset] MWBR6HDT. 115pp (ICF International [Distributor], Lilongwe, Malawi, and Calverton, Maryland, USA, 2012).
**Malawi**	2010	CBH*	DHS Program†	Point	National Statistical Office & ICF Macro. Malawi Demographic and Health Survey 2010 [Dataset] MWBR61DT. (ICF International [Distributor], Zomba, Malawi, and Calverton, Maryland, USA, 2011).
**Malawi**	2008	SBH	IPUMS INTERNATIONAL	Admin 2	National Statistical Office (Malawi) & Minnesota Population Center. Malawi Population and Housing Census 2008 from the Integrated Public Use Microdata Series, International: [Machine-readable database]. (University of Minnesota, Minneapolis, USA, 2011).
**Malawi**	2006	CBH*	UNICEF	Admin 2	National Statistical Office & United Nations Children's Fund. Malawi Multiple Indicator Cluster Survey 2006 [Dataset]. (National Statistical Office & United Nations Children's Fund, Lilongwe, Malawi, 2008).
**Malawi**	2005	CBH*	DHS Program†	Point	National Statistical Office (Malawi) & ORC Macro. Malawi Demographic and Health Survey 2004 [Dataset] MWBR4DDT. (ICF International [Distributor], Calverton, Maryland, USA, 2005).
**Malawi**	2000	CBH*	DHS Program†	Point	National Statistical Office (Malawi) & ORC Macro. Malawi Demographic and Health Survey 2000 [Dataset] MWBR41DT. (ICF International [Distributor], Zomba, Malawi and Calverton, Maryland, USA, 2001).
**Malawi**	1998	SBH	IPUMS INTERNATIONAL	Admin 2	National Statistical Office (Malawi) & Minnesota Population Center. Malawi Population and Housing Census 1998 from the Integrated Public Use Microdata Series, International: [Machine-readable database]. (University of Minnesota, Minneapolis, USA, 2011).
**Mali**	2015	SBH	DHS Program†	Point	Programme National de Lutte contre le Paludisme, Institut National de la Statistique, INFO-STAT, Institut National de la Recherche en Santé Publique & ICF International. Enquête sur les Indicateurs du Paludisme au Mali (EIPM) 2015 [Dataset] MLIR70SV. 180pp (ICF International [Distributor], Rockville, Maryland, USA, 2016).
**Mali**	2013	CBH*	DHS Program†	Point	Cellule de Planification et de Statistique, Institut National de la Statistique , INFO-STAT & ICF International. Enquête Démographique et de Santé au Mali 2012-2013 [Dataset] MLBR6HDT. (ICF International [Distributor], Rockville, Maryland, USA, 2014).
**Mali**	2009	SBH	IPUMS INTERNATIONAL	Admin 3	Central Census Bureau (Mali) & Minnesota Population Center. Mali Census 2009 from the Integrated Public Use Microdata Series, International: [Machine-readable database]. (University of Minnesota, Minneapolis, USA, 2009).
**Mali**	2006	CBH*	DHS Program†	Point	Cellule de Planification et de Statistique du Ministère de la Santé, Direction Nationale de la Statistique et de l'Informatique du Ministère de l'Économie de l'Industrie et du Commerce & Macro International Inc. Enquête Démographique et de Santé du Mali 2006 [Dataset] MLBR53DT. (ICF International [Distributor], Calverton, Maryland, USA, 2007).
**Mali**	2001	CBH*	DHS Program†	Point	Cellule de Planification et de Statistique du Ministère de la Santé, Direction Nationale de la Statistique et de l'Informatique & ORC Macro. Enquête Démographique et de Santé au Mali 2001 [Dataset] MLBR41DT. (ICF International [Distributor], Calverton, Maryland, USA, 2002).

Country	Year	Data Type	Source	Geographic level	Citation
**Mali**	1998	SBH	IPUMS INTERNATIONAL	Admin 3	Central Census Bureau (Mali) & Minnesota Population Center. Mali General Population and Housing Census 1998 from the Integrated Public Use Microdata Series, International: [Machine-readable database]. (University of Minnesota, Minneapolis, USA).
**Mauritania**	2011	CBH*	UNICEF	Admin 3	Office National de la Statistique (Mauritania) & Fonds des Nations unies pour l'Enfance Mauitanie Enquête par grappes à indicateurs multiples 2011 [Dataset]. (New York, USA, 2014).
**Mauritania**	2007	SBH	UNICEF	Admin 3	Office National de la Statistique (Mauritania) & Fonds des Nations Unies pour l'Enfrance. Mauritanie Enquête par Grappes à Indicateurs Multiples 2007 [Dataset]. 221pp (New York, USA, 2008).
**Mauritania**	2004	CBH*	DHS Program†	Admin 1	Isselmou & Ould, A. Enquête sur la Mortalité Infantile et le Paludisme (EMIP) 2003-2004 [Dataset] MRIQ4AFL. (ICF International [Distributor], Calverton, Maryland, USA, 2004).
**Mauritania**	2001	CBH*	DHS Program†	Admin 1	Office National de la Statistique (Mauritanie) & Macro, O. Enquête Démographique et de Santé Mauritanie 2000-2001 [Dataset]. (ICF Internations; [Distributor], Calverton, Maryland, USA, 2001).
**Morocco**	2011	SBH	PAPFAM	Admin 2	Ministry of Health (Morocco), Pan Arab Project for Family Health (PAPFAM), United Nations Children's Fund (UNICEF), United Nations Population Fund (UNFPA), World Health Organization (WHO). Morocco National Survey on Population and Family Health 2010-2011 [Dataset].
**Morocco**	2004	CBH*	DHS Program†	Point	Ministère de la Santé (Maroc), ORC Macro & Ligue des États Arabes. Enquête sur la Population et la Santé Familiale (EPSF) 2003-2004 [Dataset] MABR43DT. (ICF International [Distributor], Calverton, Maryland, USA, 2005).
**Morocco**	2004	SBH	IPUMS INTERNATIONAL	Admin 2	Minnesota Population Center & High Commission for Planning (Morocco). Morocco Population and Housing Census 2004 from the Integrated Public Use Microdata Series, International: [Machine-readable database]. (University of Minnesota, Minneapolis, USA, 2012).
**Mozambique**	2011	CBH*	DHS Program†	Point	Ministerio da Saude, Instituto Nacional de Estatística & ICF International. Moçambique Inquérito Demográfico e de Saúde 2011 [Dataset] MZBR62DT. (ICF International [Distributor], Calverton, Maryland, USA, 2013).
**Mozambique**	2009	CBH	UNICEF	Admin 1	De Araujo, S. N. et al. Final Report of the Multiple Indicator Cluster Survey, 2008 [Dataset]. (Maputo, Mozambique, 2009).
**Mozambique**	2009	SBH	DHS Program†	Point	Instituto Nacional de Saúde, Instituto Nacional de Estatística & ICF Macro. Inquérito Nacional de Prevalência, Riscos Comportamentais e Informação sobre o HIV e SIDA em Moçambique 2009 [Dataset] MZIR51DT. (ICF International [Distributor], Calverton, Maryland, USA, 2010).
**Mozambique**	2007	SBH	IPUMS INTERNATIONAL	Admin 3	Minnesota Population Center & National Institute of Statistics and Demography (Mozambique). Integrated Public Use Microdata Series, International: [Machine-readable database]. (University of Minnesota, Minneapolis, USA, 2007).
**Mozambique**	2003	CBH*	DHS Program†	Admin 1	Instituto Nacional de Estatística (Moçambique) & ORC Macro. Mocambique Inquérito Demográfico e de Saúde 2003 [Dataset] MZBR41DT. (ICF International [Distributor], Calverton, Maryland, USA, 2005).
**Namibia**	2013	CBH*	DHS Program†	Point	The Namibia Ministry of Health and Social Services & ICF International. The Namibia Demographic and Health Survey 2013 [Dataset] NMBR61DT. (ICF International [Distributor], Windhoek, Namibia, and Rockville, Maryland, USA, 2014).
**Namibia**	2007	CBH*	DHS Program†	Point	Ministry of Health and Social Services (Namibia) & Macro International Inc. Namibia Demographic and Health Survey 2006-07 [Dataset] NMBR51DT. (ICF International [Distributor], Windhoek, Namibia and Calverton, Maryland, USA, 2008).
**Namibia**	2000	CBH*	DHS Program†	Point	Ministry of Health and Social Services (Namibia). Namibia Demographic and Health Survey 2000 [Dataset] NMBR41DT. (ICF International [Distributor], Windhoek, Namibia, 2003).
**Niger**	2012	CBH*	DHS Program†	Admin 1	Institut National de la Statistique & ICF International. Enquête Démographique et de Santé et à Indicateurs Multiples du Niger 2012 [Dataset] NIBR61DT. (ICF International [Distributor], Calverton, Maryland, USA 2013).

Country	Year	Data Type	Source	Geographic level	Citation
**Niger**	2006	CBH*	DHS Program†	Admin 1	Institut National de la Statistique & Macro International Inc. Enquête Démographique et de Santé et à Indicateurs Multiples du Niger 2006 [Dataset] NIBR51DT. (ICF International [Distributor], Calverton, Maryland, USA 2007).
**Niger**	2000	SBH	UNICEF	Admin 1	Republique du Niger & Fonds des Nations Unies pour l'Enfrance. Enquete a indicateurs multiples 2000 (MICS2) [Dataset]. 175pp (Niamey, Niger, 2000).
**Niger**	1998	CBH*	DHS Program†	Point	Sabine, A., Seroussi, M., Kourguéni, A. I., Koché, H. & Barrère, B. Enquête Démographique et de Santé, Niger 1998 [Dataset] NIBR31DT. (ICF International [Distributor], Calverton, Maryland, USA, 1998).
**Nigeria**	2015	SBH	DHS Program†	Point	ICF International, National Bureau of Statistics (Nigeria), National Malaria Control Programme (Nigeria), National Population Commission of Nigeria. Nigeria Malaria Indicator Survey 2015 [Dataset]. (ICF International [Distributor], Fairfax, USA, 2016)
**Nigeria**	2013	CBH*	DHS Program†	Point	National Population Commission (Nigeria) & ICF International. Nigeria Demographic and Health Survey 2013 [Dataset] NGBR6ADT. (ICF International [Distributor], Abuja, Nigeria, and Rockville, Maryland, USA, 2014).
**Nigeria**	2011	SBH	UNICEF	Admin 1	National Bureau of Statistics. Nigeria Multiple Indicator Cluster Survey 2011 Main Report [Dataset]. 420pp (Abuja, Nigeria, 2011).
**Nigeria**	2010	CBH*	DHS Program†	Point	National Population Commission (Nigeria), National Malaria Control Programme (Nigeria) & ICF International. Nigeria Malaria Indicator Survey 2010 [Dataset] NGBR61DT. (ICF International [Distributor], Abuja, Nigeria, 2012).
**Nigeria**	2008	CBH*	DHS Program†	Point	National Population Commission (Nigeria) & ICF Macro. Nigeria Demographic and Health Survey 2008 [Dataset] NGBR53DT. (ICF International [Distributor], Abuja, Nigeria, 2009).
**Nigeria**	2008	SBH	National Bureau of Statistics (Nigeria)	Admin 1	Central Bank of Nigeria, National Bureau of Statistics (Nigeria), Nigerian Communications Commission (NCC). Nigeria General Household Survey 2008 [Dataset].
**Nigeria**	2007	SBH	UNICEF	Admin 1	National Bureau of Statistics. Nigeria Multiple Indicator Cluster Survey 2007 Final Report [Dataset]. 294pp (Abuja, Nigeria, 2007).
**Nigeria**	2004	SBH	National Bureau of Statistics (Nigeria)	Admin 1	National Bureau of Statistics - Federal Government of Nigeria. Nigeria Living Standards Survey 2003-2004. (Nigeria, 2004).
**Nigeria**	2003	CBH*	DHS Program†	Point	National Population Commission (Nigeria) & ORC Macro. Nigeria Demographic and Health Survey 2003 [Dataset] NGBR4BDT. (ICF International [Distributor], Calverton, Maryland, USA, 2004).
**Nigeria**	1999	SBH	UNICEF	Admin 1	United Nations Children's Fund & National Bureau of Statistics (Nigeria). Multiple Indicator Cluster Survey (1999) Nigeria [Dataset]. (2002).
**Rwanda**	2015	CBH*	DHS Program†	Point	National Institute of Statistics of Rwanda, ICF International & Ministry of Health (Rwanda). Rwanda Demographic and Health Survey 2014-2015 [Dataset] RWBR70DT. (ICF International [Distributor], Fairfax, USA, 2016).
**Rwanda**	2013	SBH	DHS Program†	Admin 1	Malaria and Other Parasitic Diseases Division (MAL & OPD Division-RBC) [Rwanda] & ICF International. Rwanda Malaria Indicator Survey 2013 [Dataset] RWHR6QDT. 103pp (ICF International [Distributor], 2014, Rockville, Maryland, USA, 2014).
**Rwanda**	2012	SBH	National Institute of Statistics of Rwanda	Admin 2	National Institute of Statistics of Rwanda. Rwanda Population and Housing Census 2012 [Dataset]. (Kigali, Rwanda, 2015).
**Rwanda**	2011	CBH*	DHS Program†	Point	National Institute of Statistics of Rwanda & ICF International. Rwanda Demographic and Health Survey 2010 [Dataset] RWBR61DT. (ICF International [Distributor], Calverton, Maryland, USA, 2012).
**Rwanda**	2008	CBH*	DHS Program†	Point	Ministère de la Santé, Institut National de la Statistique du Rwanda & ICF Macro. Enquête Intermédiaire sur les indicateurs Démographiques et de Santé, Rwanda 2007-2008 [Dataset] RWBR5ADT. (ICF International [Distributor], Calverton, Maryland, USA, 2009).

Country	Year	Data Type	Source	Geographic level	Citation
**Rwanda**	2006	SBH	National Institute of Statistics of Rwanda	Admin 2	National Institue of Statistics Rwanda. Integrated Living Conditions Survey 2005/6. 57pp (Kigali, Rwanda, 2006).
**Rwanda**	2005	CBH*	DHS Program†	Point	Institut National de la Statistique du Rwanda & ORC Macro. Rwanda Demographic and Health Survey 2005 [Dataset] RWBR53DT. (ICF International [Distributor], Calverton, Maryland, USA, 2006).
**Rwanda**	2002	SBH	IPUMS INTERNATIONAL	Admin 1	National Census Commission (Rwanda) & Minnesota Population Center. Rwanda Population and Housing Census 2002 from the Integrated Public Use Microdata Series, International: [Machine-readable database]. (University of Minnesota, Minneapolis, USA).
**Rwanda**	2000	CBH*	DHS Program†	Admin 1	Office National de la Population (Rwanda) & ORC Macro. Enquête Démographique et de Santé, Rwanda 2000 [Dataset] RWBR41DT. (ICF International [Distributor], Kigali, Rwanda et Calverton, Maryland, USA, 2001).
**Senegal**	2015	CBH*	DHS Program†	Admin 1	Cheikh Anta Diop University, ICF International, National Agency of Statistics and Demography (Senegal). Senegal Continuous Demographic and Health Survey 2015 [Dataset]. (ICF International [Distributor], Fairfax, USA, 2016).
**Senegal**	2014	CBH*	DHS Program†	Admin 1	Agence Nationale de la Statistique et de la Démographie (Sénégal) & ICF International. Sénégal : Enquête Démographique et de Santé Continue (EDS-Continue 2012-14), Rapport Régional [Dataset] SNBR70DT. (ICF International [Distributor], Rockville, Maryland, USA, 2015).
**Senegal**	2013	CBH*	DHS Program†	Point	Agence Nationale de la Statistique et de la Démographie (Sénégal) & ICF International. Enquête Démographique et de Santé Continue (EDS-Continue 2012-2013) [Dataset] SNBR6CDT. (ICF International [Distributor], Calverton, Maryland, USA, 2013).
**Senegal**	2011	CBH*	DHS Program†	Point	Agence Nationale de la Statistique et de la Démographie (Sénégal) & ICF International. Enquête Démographique et de Santé à Indicateurs Multiples au Sénégal (EDS-MICS) 2010-2011 [Dataset] SNBR61DT. (ICF International [Distributor], Calverton, Maryland, USA, 2012).
**Senegal**	2009	CBH*	DHS Program†	Point	Ndiaye, S. & Ayad, M. Enquête Nationale sur le Paludisme au Sénégal 2008-2009 [Dataset] SNBR5HDT. (ICF International [Distributor], Calverton, Maryland, USA, 2009).
**Senegal**	2006	SBH	DHS Program†	Admin 1	Ndiaye, Salif & Mohamed Ayad. Enquête Nationale sur le Paludisme au Sénégal 2006 [Dataset] SNIR50DT. (Macro International Inc [Distributor], Calverton, Maryland, USA, 2007).
**Senegal**	2005	CBH*	DHS Program†	Point	Ndiaye, S. & Ayad, M. Enquête Démographique et de Santé au Sénégal 2005 [Dataset] SNBR4HDT. (ICF International [Distributor], Calverton, Maryland, USA, 2006).
**Senegal**	2002	SBH	IPUMS INTERNATIONAL	Admin 2	Directorate of Forecasting and Statistics (Senegal) & Minnesota Population Center. Senegal General Population and Housing Census 2002 from the Integrated Public Use Microdata Series, International: [Machine-readable database]. (University of Minnesota, Minneapolis, USA).
**Senegal**	1999	CBH*	DHS Program†	Admin 1	Groupe SERDHA, Macro International, Inc, Ministry of Health and Prevention (Senegal). Senegal Demographic and Health Survey 1999 [Dataset]. (Macro International, Inc. [Distributor], Calverton, USA).
**Sierra Leone**	2013	CBH*	DHS Program†	Point	Statistics Sierra Leone & ICF International. Sierra Leone Demographic and Health Survey 2013 [Dataset] SLBR61DT. (ICF International [Distributor], Freetown, Sierra Leone and Rockville, Maryland, USA, 2014).
**Sierra Leone**	2010	SBH	UNICEF	Admin 2	Statistics Sierra Leone & United Nations Children's Fund. Sierra Leone Multiple Indicator Cluster Survey 2010, Final Report [Dataset]. 253pp (Freetown, Sierra Leone, 2011).
**Sierra Leone**	2008	CBH*	DHS Program†	Point	Statistics Sierra Leone & ICF Macro. Sierra Leone Demographic and Health Survey 2008 [Dataset] SLBR51DT. (ICF International [Distributor], Calverton, Maryland, USA, 2009).
**Sierra Leone**	2005	SBH	UNICEF	Admin 2	Statistics Sierra Leone & United Nations Children's Fund. Sierra Leone Multiple Indicator Cluster Survey 2005, Final Report [Dataset]. 261pp (Freetown, Sierra Leone, 2007).

Country	Year	Data Type	Source	Geographic level	Citation
**Sierra Leone**	2004	SBH	IPUMS INTERNATIONAL	Point & Admin 3	Minnesota Population Center. Sierra Leone Census 2004 from the Integrated Public Use Microdata Series, International: [Machine-readable database]. (University of Minnesota, Minneapolis, USA).
**Sierra Leone**	2000	SBH	UNICEF	Admin 1	Central Statistics Office (Sierra Leone) & United Nations Children's Fund. A Household Survey Report (MICS-2) [Dataset]. 144pp (New York, USA, 2000).
**Somalia**	2011	CBH*	UNICEF	Point	United Nations Children's Fund Somalia & Somaliland Ministry of Planning and National Development. Somaliland Multiple Indicator Cluster Survey 2011 [Dataset]. (Nairobi, Kenya, 2014).
**Somalia**	2011	CBH*	UNICEF	Admin 1	United Nations Children's Fund Somalia & Ministry of Planning and International Cooperation. Northeast Zone Multiple Indicator Cluster Survey 2011, [Dataset]. (Nairobi, Kenya, 2014).
**Somalia**	2006	CBH*	UNICEF	Admin 1	Pan Arab Project for Family Health & United Nations Children's Fund. Somalia Multiple Indicator Cluster Survey 2006 [Dataset]. (New York, USA, 2006).
**South Africa**	2011	CBH	DataFirst	Admin 1	Southern Africa Labour and Development Research Unit. National Income Dynamics Study 2010-2011, Wave 2 [Dataset]. (Cape Town, South Africa, 2012).
**South Africa**	2007	SBH	IPUMS INTERNATIONAL	Admin 3	Statistics South Africa & Minnesota Population Center. South Africa Community Survey 2007 from the Integrated Public Use Microdata Series, International: [Machine-readable database]. (University of Minnesota, Minneapolis, USA).
**South Africa**	2001	SBH	IPUMS INTERNATIONAL	Admin 3	Statistics South Africa & Minnesota Population Center. South Africa Census 2001 from the Integrated Public Use Microdata Series, International: [Machine-readable database]. (University of Minnesota, Minneapolis, USA).
**South Africa**	1998	CBH*	DHS Program†	Admin 1	Department of Health (South Africa), Macro International Inc & South African Medical Research Council. South Africa Demographic and Health Survey 1998 [Dataset] ZABR31DT. 420pp (Macro International, Inc [Distributor], Calverton, USA, 1998).
**South Africa**	1998	SBH	International Food Policy Research Institute (IFPRI)	Admin 1	International Food Policy Research Institute (IFPRI), University of Natal UoW, Data Research Africa (DRA), Policy and Praxis, Southern Africa Labour Development Research Unit (SALDRU) S, chool of Economics, et al. South Africa KwaZulu-Natal Income Dynamics Study 1998 [Dataset]. (Durban, South Africa).
**South Sudan**	2010	CBH	UNICEF	Admin 1	Ministry of Health & National Bureau of Statistics. South Sudan Household Survey 2010, [Dataset]. (Juba, South Sudan, 2013).
**South Sudan**	2008	SBH	IPUMS INTERNATIONAL	Admin 2	National Population Census Council (Sudan), Central Bureau of Statistics (Sudan), Southern Sudan Centre for Census, Statistics and Evaluation & Minnesota Population Center. Sudan Population and Housing Census 2008 from the Integrated Public Use Microdata Series, International: [Machine-readable database]. (University of Minnesota, Minneapolis, USA, 2011).
**Sudan**	2014	CBH*	UNICEF	Admin 1	Central Bureau of Statistics & United Nations Children’s Fund Sudan. Multiple Indicator Cluster Survey 2014 of Sudan [Dataset]. (United Nations Children’s Fund and Central Bureau of Statistics [Distributor], Khartoum, Sudan, 2016).
**Sudan**	2010	CBH	UNICEF	Admin 1	Federal Ministry of Health & Central Bureau of Statistics. Sudan Household and Health Survey - Round 2, 2010, [Dataset]. (Khartoum, Republic of Sudan, 2012).
**Sudan**	2008	SBH	IPUMS INTERNATIONAL	Admin 3	National Population Census Council (Sudan), Central Bureau of Statistics (Sudan), Southern Sudan Centre for Census, Statistics and Evaluation & Minnesota Population Center. Sudan Population and Housing Census 2008 from the Integrated Public Use Microdata Series, International: [Machine-readable database]. (University of Minnesota, Minneapolis, USA, 2011).
**Sudan**	2006	CBH	Pan Arab Project for Family Health (PAPFAM)	Admin 1	Ministry of Health (Southern Sudan), Federal Ministry of Health (Sudan), Southern Sudan Centre for Census, Statistics and Evaluation (SSCCSE), Central Bureau of Statistics (Sudan). Sudan Family Health Survey 2006 [Dataset].
**Swaziland**	2014	CBH	UNICEF	Admin 1	Central Statistical Office (Swaziland), United Nations Children's Fund (UNICEF), United Nations Educational, Scientific and Cultural Organization (UNESCO), United Nations Population Fund (UNFPA). Swaziland Multiple Indicator Cluster Survey 2014 [Dataset]. (New York, USA, 2016).

Country	Year	Data Type	Source	Geographic level	Citation
**Swaziland**	2010	CBH*	UNICEF	Admin 1	Central Statistical Office & United Nations Children's Fund. Swaziland Multiple Indicator Cluster Survey 2010 [Dataset]. (Mbabane, Swaziland, 2011).
**Swaziland**	2007	CBH*	DHS Program†	Point	Central Statistical Office (Swaziland) & Macro International Inc. Swaziland Demographic and Health Survey 2006-07 [Dataset] SZBR51DT. (ICF International [Distributor], Mbabane, Swaziland, 2008).
**Swaziland**	2000	SBH	UNICEF	Admin 1	Central Statistical Office (Swaziland) & United Nations Children's Fund. Swaziland Multiple Indicator Cluster Survey 2000 [Dataset]. 101pp (New York, USA, 2000).
**Tanzania**	2016	CBH*	DHS Program†	Point	ICF International, Ministry of Health (Zanzibar), Ministry of Health CD, Gender, Elderly and Children (MoHCDEC) (Tanzania), National Bureau of Statistics (Tanzania), Office of Chief Government Statistician (OCGS-Zanzibar). Tanzania Demographic and Health Survey 2015-2016 [Dataset]. (ICF International [Distributor], Fairfax, USA, 2016).
**Tanzania**	2012	SBH	IPUMS INTERNATIONAL	Admin 2	National Bureau of Statistics (Tanzania) & Minnesota Population Center. Tanzania Population and Housing Census 2012 from the Integrated Public Use Microdata Series, International: [Machine-readable database]. (University of Minnesota, Minneapolis, USA, 2017).
**Tanzania**	2012	SBH	DHS Program†	Point	Tanzania Commission for AIDS (TACAIDS), Zanzibar AIDS Commission (ZAC), National Bureau of Statistics (NBS), Office of the Chief Government Statistician (OCGS) & ICF International. Tanzania HIV/AIDS and Malaria Indicator Survey 2011-12 [Dataset] TZBR6ASV. 16pp (ICF International [Distributor], Dar es Salaam, Tanzania, 2013).
**Tanzania**	2010	CBH*	DHS Program†	Point	National Bureau of Statistics (Tanzania) & ICF Macro. Tanzania Demographic and Health Survey 2010 [Dataset] TZBR63DT. (ICF International [Distributor], Dar es Salaam, Tanzania, 2011).
**Tanzania**	2008	CBH	DHS Program†	Point	Tanzania Commission for AIDS, Zanzibar AIDS Commission, National Bureau of Statistics, Office of the Chief Government Statistician & Macro International Inc. Tanzania HIV/AIDS and Malaria Indicator Survey 2007-08 [Dataset] TZBR51DT. (ICF International [Distributor], Dar es Salaam, Tanzania, 2008).
**Tanzania**	2005	CBH*	DHS Program†	Admin 1	National Bureau of Statistics (Tanzania) & ORC Macro. Tanzania Demographic and Health Survey 2004-05 [Dataset] TZBR4IDT. (ICF International [Distributor], Dar es Salaam, Tanzania, 2005).
**Tanzania**	2004	SBH	DHS Program†	Point	Tanzania Commission for AIDS (TACAIDS), National Bureau of Statistics (NBS) & ORC Macro. Tanzania HIV/AIDS Indicator Survey 2003-04 [Dataset] TZIR4ADT. (ORC Macro [Distributor], Calverton, Maryland, USA, 2005).
**Tanzania**	2002	SBH	IPUMS INTERNATIONAL	Admin 2	National Bureau of Statistics (Tanzania) & Minnesota Population Center. Tanzania Population and Housing Census 2002 from the Integrated Public Use Microdata Series, International: [Machine-readable database]. (University of Minnesota, Minneapolis, USA).
**Tanzania**	1999	CBH*	DHS Program†	Point	National Bureau of Statistics (Tanzania) & Macro International Inc. Tanzania Reproductive and Child Health Survey 1999 [Dataset] TZBR41DT. (ICF International [Distributor], Calverton, Maryland, USA, 2000).
**Togo**	2013	CBH*	DHS Program†	Point	Ministère de la Planification du Développement et de l'Aménagement du Territoire, Ministère de la Santé & ICF International. Enquête Démographique et de Santé au Togo 2013-2014 [Dataset] TGBR61DT. (ICF International [Distributor], Rockville, Maryland, USA, 2015).
**Togo**	2010	SBH	UNICEF	Admin 1	Direction Générale de la Statistique et de la Comptabilité Nationale. Enquête par grappes à indicateurs multiples MICS Togo, 2010, Rapport final [Dataset]. 244pp (New York, USA, 2012).
**Togo**	2006	SBH	UNICEF	Admin 1	Direction Générale de la Statistique et de la Comptabilité Nationale. Résultats de l'enquête nationale à indicateurs multiples Togo 2006 [Dataset]. 304pp (New York, USA, 2007).
**Togo**	1998	CBH*	DHS Program†	Point	Anipah, K. et al. Enquete Demographique et de Sante, Togo 1998 [Dataset] TGBR31DT. (ICF International [Distributor], Calverton, Maryland, USA, 1999).
**Uganda**	2015	SBH	DHS Program†	Point	Uganda Bureau of Statistics & ICF International. Uganda Malaria Indicator Survey 2014-15 [Dataset] UGIR72DT. 139pp (ICF International [Distributor], Kampala, Uganda, and Rockville, Maryland, USA, 2015).

Country	Year	Data Type	Source	Geographic level	Citation
**Uganda**	2012	SBH	World Bank	Point	Uganda Bureau of Statistics. Uganda Living Standards Measurement Survey - Integrated Survey on Agriculture 2011-2012 [Dataset]. (Kampala, Uganda, 2013).
**Uganda**	2011	CBH*	DHS Program†	Point	Uganda Bureau of Statistics & ICF International Inc. Uganda Demographic and Health Survey 2011 [Dataset] UGBR60DT. (ICF International [Distributor], Kampala, Uganda and Calverton, Maryland, USA, 2012).
**Uganda**	2011	SBH	DHS Program†	Point	Centers for Disease Control and Prevention (CDC), ICF International, Ministry of Health (Uganda), Uganda Bureau of Statistics, Uganda Viral Research Institute. Uganda AIDS Indicator Survey 2011 [Dataset]. (ICF International [Distributor], Calverton, USA, 2012).
**Uganda**	2011	SBH	World Bank	Point	Uganda Bureau of Statistics. The Uganda National Panel Survey 2010/11 [Dataset]. (Kampala, Uganda, 2013)
**Uganda**	2010	CBH*	DHS Program†	Point	Uganda Bureau of Statistics & ICF Macro. Uganda Malaria Indicator Survey 2009 [Dataset] UGBR5HDT. (ICF International [Distributor], Calverton, Maryland, USA, 2010).
**Uganda**	2009	SBH	World Bank	Point	Uganda Bureau of Statistics. Uganda Living Standards Measurement Survey - Integrated Survey on Agriculture 2009-2010. (World Bank, Washington DC, USA).
**Uganda**	2006	CBH*	DHS Program†	Point	Uganda Bureau of Statistics & Macro International Inc. Uganda Demographic and Health Survey 2006 [Dataset] UGBR52DT. (ICF International [Distributor], Calverton, Maryland, USA, 2007).
**Uganda**	2002	SBH	IPUMS INTERNATIONAL	Admin 2	Uganda Bureau of Statistics & Minnesota Population Center. Uganda Population and Housing Census 2002 from the Integrated Public Use Microdata Series, International: [Machine-readable database]. (University of Minnesota, Minneapolis, USA).
**Uganda**	2001	CBH*	DHS Program†	Point	Uganda Bureau of Statistics & ORC Macro. Uganda Demographic and Health Survey 2000-2001 [Dataset] UGBR41DT. (ICF International [Distributor], Calverton, Maryland, USA, 2001).
**Zambia**	2014	CBH*	DHS Program†	Point	Central Statistical Office (Zambia), Ministry of Health (Zambia) & ICF International. Zambia Demographic and Health Survey 2013-14 [Dataset] ZMBR61DT. (ICF International [Distributor], Rockville, Maryland, USA, 2014).
**Zambia**	2010	SBH	IPUMS INTERNATIONAL	Admin 3	Central Statistical Office (Zambia) & Minnesota Population Center. Zambia Census 2010 from the Integrated Public Use Microdata Series, International: [Machine-readable database]. (University of Minnesota, Minneapolis, USA).
**Zambia**	2007	CBH*	DHS Program†	Point	Central Statistical Office, Ministry of Health, Tropical Diseases Research Centre, University of Zambia & Macro International Inc. Zambia Demographic and Health Survey 2007 [Dataset] ZMBR51DT. (ICF International [Distributor], Calverton, Maryland, USA, 2009).
**Zambia**	2002	CBH*	DHS Program†	Admin 1	Central Statistical Office (Zambia), Central Board of Health (Zambia) & ORC Macro. Zambia Demographic and Health Survey 2001-2002 [Dataset] ZMBR42DT. (ICF International [Distributor], Calverton, Maryland, USA, 2003).
**Zambia**	2000	SBH	IPUMS INTERNATIONAL	Admin 3	Central Statistical Office (Zambia) & Minnesota Population Center. Zambia Census 2000 from the Integrated Public Use Microdata Series, International: [Machine-readable database]. (University of Minnesota, Minneapolis, USA).
**Zimbabwe**	2015	CBH*	DHS Program†	Point	Zimbabwe National Statistics Agency & ICF International. Zimbabwe Demographic and Health Survey 2015 [Dataset] ZWBR70DT. 575pp (ICF International [Distributor], Rockville, Maryland, USA, 2016).
**Zimbabwe**	2014	CBH*	UNICEF	Admin 1	Zimbabwe National Statistics Agency. Zimbabwe Multiple Indicator Cluster Survey 2014 [Dataset]. (Harare, Zimbabwe, 2015).
**Zimbabwe**	2012	SBH	Zimbabwe National Statistics Agency	Point & Admin 2	Zimbabwe National Statistics Agency. Zimbabwe Population Census 2012. 152pp (Harare, Zimbabwe, 2013).
**Zimbabwe**	2011	CBH*	DHS Program†	Point	Zimbabwe National Statistics Agency & ICF International. Zimbabwe Demographic and Health Survey 2010-11 [Dataset] ZWBR62DT. (ICF International [Distributor], Calverton, Maryland, USA, 2012).

Country	Year	Data Type	Source	Geographic level	Citation
**Zimbabwe**	2009	CBH*	UNICEF	Admin 1	Zimbabwe National Statistics Agency & United Nations Children's Fund. Zimbabwe Multiple Indicator Monitoring Survey (MIMS) 2009 [Dataset]. (Harare, Zimbabwe, 2010).
**Zimbabwe**	2006	CBH*	DHS Program†	Point	Central Statistical Office (Zimbabwe) & Macro International Inc. Zimbabwe Demographic and Health Survey 2005-06 [Dataset] ZWBR51DT. (ICF International [Distributor], Calverton, Maryland, USA, 2007).
**Zimbabwe**	2002	SBH	Zimbabwe National Statistics Agency	Admin 1	Central Statistical Office (Zimbabwe). Zimbabwe Population and Housing Census 2002. 173pp (Zimbabwe, 2004).
**Zimbabwe**	1999	CBH*	DHS Program†	Point	Central Statistical Office (Zimbabwe) & Macro International Inc. Zimbabwe Demographic and Health Survey 1999 [Dataset] ZWBR42DT. (ICF International [Distributor], Calverton, Maryland, USA, 2000).

4. Data outliers

Several surveys were identified for inclusion but were ultimately excluded from the final analysis for concerns about data quality. The 2007 Ethiopia census[2] was dropped due to 80% missingness in SBH data and unrealistic geographic distribution of mortality risk. A CBH dataset was dropped if the percent of children who died missing either birth or death date exceeded 10%, or exceeded 5% and had reported data quality issues. Such surveys were South Africa NIDS 2011, subnational MICS surveys from Kenyan counties Bungoma, Turkana, Kakamega (2013-2014), and Somalia NE Zone (2011), and the Djibouti PAPFAM survey 2012. Finally, we dropped data points from Diffa region (2012 Niger DHS) and Jonglei and Unity states (South Sudan 2010 MICS) as they were highly discordant with data points from the same surveys in nearby regions with similar sociodemographic profiles.

5. Tabulating CBH data

For each survey cluster we tabulated monthly exposures and deaths for each of four age bins over four separate five-year periods, as illustrated in Supplementary Figure 2. This tabulation process is similar to that used to compute under-5 mortality estimates for GBD 2016.[3] The exception to this is that we use four component bins: month 0, months 1-11, months 12-35, and months 36–59, whereas GBD uses six bins: 0 months, 1–11 months, 12–23 months, 24–36 months, 36–47 months, and 48–59 months. For areas larger than survey clusters, survey weights were used in the tabulation step to make weighted mortality rates for each of the component bins, which were then applied to the observed tabulated exposure months to estimate an equivalent number of deaths. Estimates for the 2015 period may be slightly overestimated due to the relatively smaller amount of data in the later years of the period.

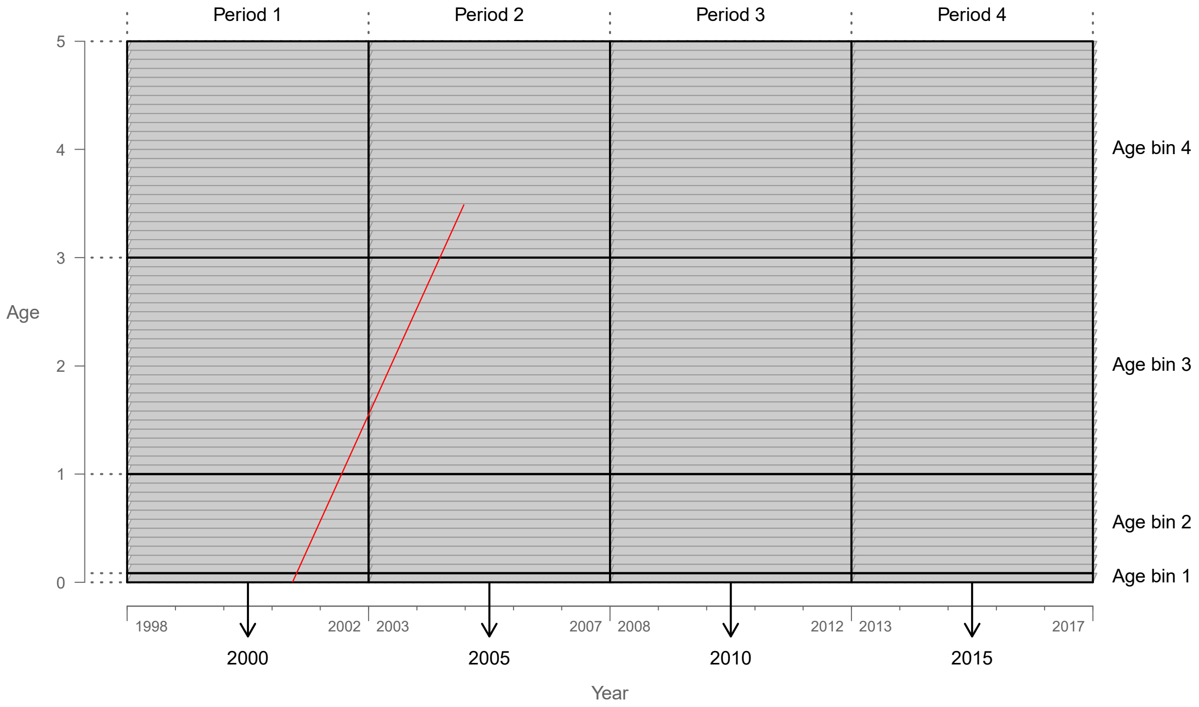

Supplementary Figure 2: Lexis diagram illustrating periods and age bins within which CBH data were tabulated. For each month (grey parallelograms) within each age bin/period pair, (rectangles bounded by solid black lines) we tabulated the number of exposures (number of children alive and entering the month of life within the given period) and number of deaths (exposures who died during that month) for each survey cluster, or geographic area comprising multiple clusters, where cluster-specific data were unavailable. For example, if we observed a living child who was 3.5 years old in a January 2004 survey, their life history would be represented with the red line in the figure, extending back to midway through 2001 when they were born. This child would have contributed exposure months in the cells (month/period combinations) crossed by their life history: (age bin 4/period 2005: 6 months; 3/2005: 18 months; 3/2000: 6 months, 2/2000: 12 months; and 1/2000, 1 month). If the same child had died at age 3.5 in January 2004, they would have contributed the same number of exposure months, but also a death event in age bin 4/period 2005.

6. Adjustment of summary birth history data

In a summary birth history (SBH) survey module, a woman is asked a minimum of two questions: the number of children ever born (CEB) to them and the number of those who have died (CD). While providing much less information than the full account of life histories available in CBH data, SBH is widely available. Of the 235 surveys for which we had microdata, 104 contained only summary birth histories, most notably in many Unicef MICS and census datasets.

To be usable for child mortality estimation, SBH needs to be adjusted such that they are representative of age- and time-specific mortality. This involves an adjustment of the rate as well as localizing the mortality rates in time.[4] Recent model-based improvements have been made on classical indirect methods[4–6] by relating the CBH- and SBH-derived rates from surveys where both are available, along with information on parity and maternal age.[7]

SBH and CBH survey modules capture different information on child births and deaths. For CBH data we can tabulate information for each child to directly estimate deaths and exposure months for any desired age bin and time period, while in SBH, the only mortality data available is the CD/CEB ratio for each mother interviewed. Ultimately, for this analysis, we wish to approximate CBH-style binomial samples from available information in SBH. The strong linear relationship between the logit of the CD/CEB ratio and the logit under-5 probability of death (5q0) has been noted in the past and used as motivation for a model-based approach to adjusting SBH to match CBH data.[7] In this analysis, we had the added challenge of dealing with data that need to be child-age disaggregated, which has not been well explored in the literature. Using surveys with both SBH and CBH modules, we found evidence that logit of CD/CEB had a strong relationship with the monthly probabilities of death tabulated in our analytical age groups from CBH data. The plots in Supplementary Figure 3 show the bivariate relationships across ages and lag-times between in surveys that collect both CBH and SBH data. These plots give strong motivation that a modelling approach should work for smaller age bins as well.

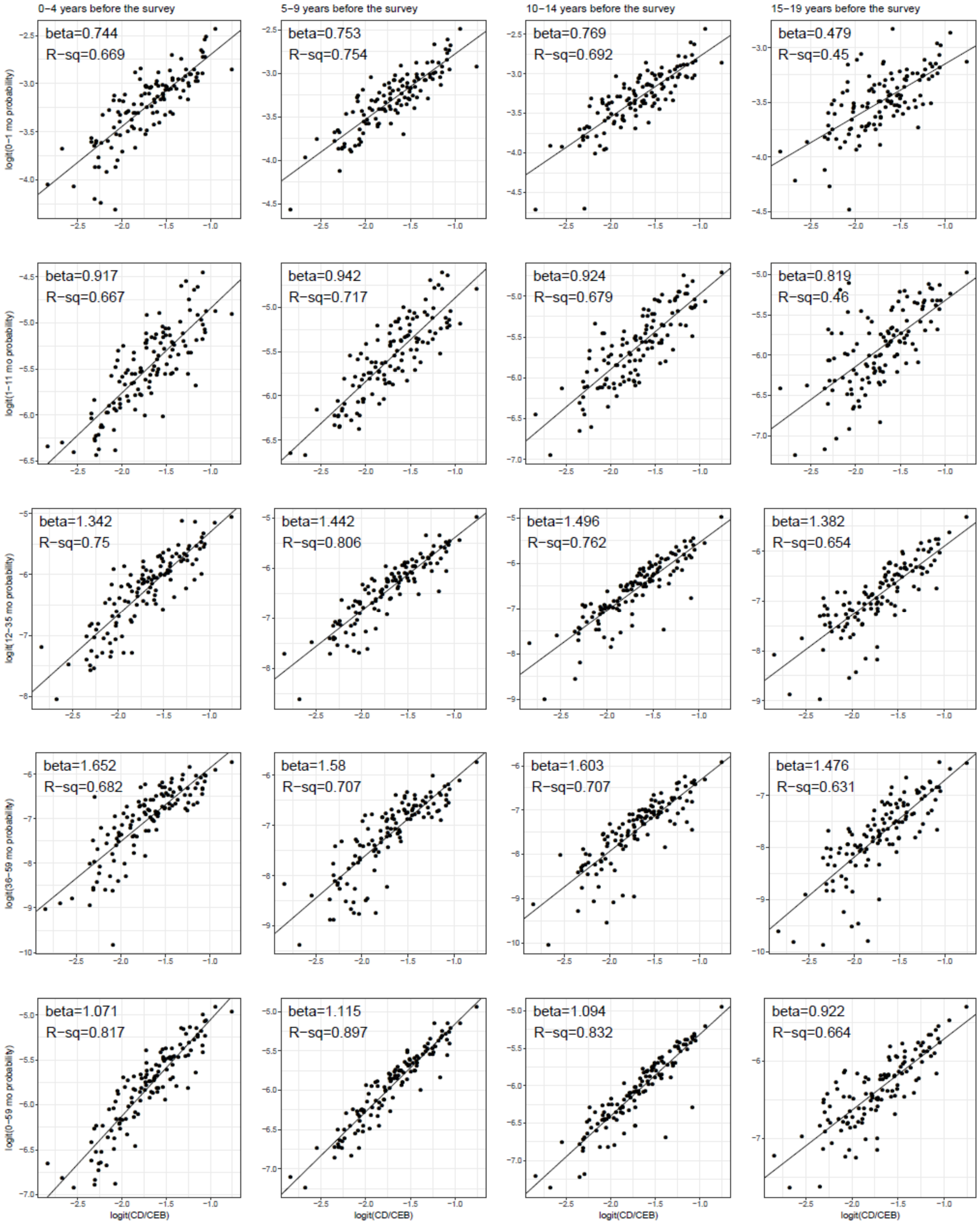

Supplementary Figure 3: Comparison of SBH and CBH mortality. Using surveys that collect both SBH and CBH mortality data, we find a strong relationship between the logit of the monthly probability of death for all of our analytical age bins, as well as with the full under-5 bin, with the logit of CD/CEB for each survey. The logit of CD/CEB is highly predictive of CBH tabulated age-specific monthly mortality probability going back up to 19 years before the survey. Lines drawn show a linear regression line, with a beta coefficient and R-squared provided. SBH = Summary birth history; CBH = Complete birth history; CD = Children died; CEB = Children ever born.

6.1 Data preparation

First, we collated data on over four million children from 119 datasets (mostly DHS) collected since 1998 (the beginning of our study period) that contain both SBH and CBH data, these are marked with an asterisk on Supplementary table 1. We tabulated the CBH deaths and exposure months at the age-period level as described in the section above. Thus, each survey produced up to 16 rows of data, one for each child age bin (month 0, months 1–11, months 12–35, and months 36–59) and time period bin (centred at 2000, 2005, 2010, 2015), each with the number of CBH-tabulated deaths and exposures and SBH-tabulated CD and CEB for the cluster.

We will refer to the CBH-SBH combined dataset as the training set, and the SBH-only data which we aim to adjust as the prediction dataset. The prediction dataset will be aggregated to the level of the smallest possible geography – either survey cluster or some identifiable administrative unit. We will refer from now on to this smallest geography simply as cluster.

In order to use a logit transformation of the CD/CEB ratio in the modelling step (described in the following subsection), we had to ensure the CD data did not include zeroes since the logit of 0 is negative infinity. To do so, we fitted the following Bayesian hierarchical shrinkage model to adjust death counts in surveys that contained clusters with no deaths recorded:

(1)

$$CD_i \sim Binomial(q_i, CEB_i)$$
$$logit(q_i) = \alpha + \gamma_i$$
$$\gamma_i \sim N(0, \sigma^2)$$

where *i* is cluster and γ is a normal independent random effect by cluster. Models were fit separately for each survey. A minimally informative prior, $\log\left(\frac{1}{\sigma^2}\right) \sim loggamma(1, 0.00005)$, was placed on σ. The effect of this adjustment was extremely small, correlation between original CD and adjusted was greater than 0.9999. We then used $\widehat{q_i}$ in place of the CD/CEB ratio in the full SBH adjustment model, described in the next section.

6.2 SBH adjustment models

We developed a two-stage regression model for cross-walking SBH data into age and period bin approximate binomial samples to match the CBH input data. In the first model we estimate $\hat{p}$, the bin specific monthly probability of death. In the second model we estimate $\widehat{N}$, the number of exposure months in that bin. From these two quantities we then simulate bin-specific deaths $\widehat{N^+}$. $\widehat{N}$ and $\widehat{N^+}$ are used as training data at SBH clusters in the geostatistical model described in detail later in this document. 10,000 $\widehat{N^+}$ draws are simulated from draws taken from the predictive posterior distributions of $\widehat{N}$ and $\hat{p}$ and the resulting variance is used to down-weight these observations when fitting the geostatistical model. We describe these models in detail below.

The $\hat{p}$ model was fit to the training set at the survey, age bin, period, and level, and then used to predict to SBH-only data at the smallest identifiable geography.

We fit the following Bayesian hierarchical logistic regression model to the training data:

(2)

$$N^{+}_{s,a,p} \sim Binomial(p_{s,a,p},\ N_{s,a,p})$$
$$log(p_{s,a,p}/(1-p_{s,a,p})) = \beta_0 + logit(\hat{q}_s)\,(\beta_1 + Age\beta_2 + Lag_{s,p}\beta_3 + Age * Lag_{s,p}\beta_4) + \boldsymbol{X}_{s,a,p}\boldsymbol{\beta_{fe}} + \gamma_s + \gamma_{country}$$
$$\gamma_s \sim N(0, \sigma_s^2)$$
$$\gamma_{country} \sim N(0, \sigma_{country}^2)$$

where $N_{s,a,p}$ are the number of monthly exposures recorded in a survey *s*, age bin *a,* and time period *p*, and $N^{+}_{s,a,p}$ are the number of deaths resulting from all monthly exposures. $N_{s,a,p}$ and $N^{+}_{i,a,p}$ both come from direct tabulations of the CBH data. α_0 is a global intercept term. $\hat{q}_i$ is the zero-adjusted CD/CEB ratio described above in equation (1). The logit of CD/CEB is modified by interactions with child age bin, lag, and the interaction of child age bin and lag. Lag represents the number of periods between the survey to time of mortality risk. We further included fixed effects for all individual terms from the interactions as well as for mean maternal age, year, parity ratios between 15-19 and 20-24 year olds, parity ratios between 20-24 and 25-29 year olds, the proportions of mothers in the survey in various maternal age bins (15-19, 20-24, and 25-39), and the number of years of coverage the survey had in the period bin. We represent the values of these fixed effect covariates with the bolded matrix element $\boldsymbol{X}_{s,a,p}$ and their associated coefficients in the vector $\boldsymbol{\beta}_{fe}$. Survey weights were used to make estimates of the independent variables at areas larger than clusters in the prediction data set.

We fit another model to estimate $\widehat{N}$, the expected number of exposure months of life in each child age, period bin. For this second model, the target of inference was a number and not a ratio, so we chose to fit the model on the training set aggregated to the survey-admin1 level, and thus more similar to numbers seen in the prediction dataset. This model was very similar to the model for $\hat{p}$ described in equation (2) but with several key differences: 1) we fit to $N_{i,a,p}$ using a Poisson likelihood; 2) we used log(CEB) instead of logit(CD/CEB) as the main effect driving the adjustment; 3) we added a fixed effects term for log(CD) and a random effect on survey-admin1; and 4) we replaced the interaction with lag with an interaction with number of years in the period covered by the survey.

In addition, two prediction set censuses (Zimbabwe 2002, and Zimbabwe 2012) were only available in tabulated form. As such, for these three surveys we trained and predicted slightly modified $\hat{p}$ and $\widehat{N}$ models, using proportion of women instead of proportion of mothers fixed effects.

All models were fitted using R-INLA. The following minimally informative priors (http://www.r-inla.org/models/latent-models) were used: $\beta \sim N(0, 1000)$, $\log\left(\frac{1}{\sigma^2}\right) \sim loggamma(1{,}0.00005)$.

6.2.1 Model fits of SBH adjustment models

Supplementary Table 2: SBH Adjustment model results for the *p* model. SBH=Summary birth history. Reference levels for factor variables lag and child age bin are lag 0 and child age bin 1.

Variable	Mean coef. estimate	2.5% UI	97.5% UI
Intercept	-4.3226	-5.629	-3.0253

Variable	Mean coef. estimate	2.5% UI	97.5% UI
CD/CEB	0.1168	0.026	0.2071
lag 1	0.2435	-0.0841	0.5709
lag 2	0.5205	0.1469	0.8936
lag 3	0.1055	-0.4127	0.6232
child age bin 2	-1.6537	-1.7677	-1.5399
child age bin 3	-1.5178	-1.6369	-1.3987
child age bin 4	-2.2989	-2.4504	-2.1474
mean maternal age	-0.0075	-0.0322	0.0173
period	0.123	0.0991	0.1465
proportion mothers 15-19	3.5498	1.1685	5.909
proportion mothers 20-24	1.5733	0.1497	2.9987
proportion mothers 25-39	0.9012	-0.3119	2.1217
P(15-19)/P(20-24)	-1.3741	-2.079	-0.6674
P(20-24)/P(25-25)	0.6768	0.0632	1.2865
years in period	-0.041	-0.051	-0.0309
CD/CEB * lag 1	0.1887	0.1317	0.2456
CD/CEB * lag 2	0.3763	0.3123	0.4403
CD/CEB * lag 3	0.3049	0.2217	0.388
CD/CEB * child age bin 2	0.414	0.3499	0.4781
CD/CEB * child age bin 3	0.9688	0.9002	1.0374
CD/CEB * child age bin 4	0.9449	0.8569	1.0331
lag 1 * child age bin 2	-0.1006	-0.2422	0.0409
lag 2 * child age bin 2	-0.0961	-0.2549	0.0625
lag 3 * child age bin 2	0.3137	0.1049	0.5224
lag 1 * child age bin 3	0.0649	-0.0832	0.2128
lag 2 * child age bin 3	-0.081	-0.2488	0.0866
lag 3 * child age bin 3	-0.2431	-0.4618	-0.0245
lag 1 * child age bin 4	-0.2064	-0.4000	-0.0129
lag 2 * child age bin 4	-0.4383	-0.6594	-0.2174
lag 3 * child age bin 4	-0.4778	-0.7686	-0.1871
lag 1 * mean maternal age	0.0086	-0.0017	0.0189
lag 2 * mean maternal age	0.0123	0.0000	0.0238
lag 3 * mean maternal age	0.0203	0.0041	0.0365
CD/CEB * lag 1 * child age bin 2	-0.076	-0.155	0.003
CD/CEB * lag 2 * child age bin 2	-0.1101	-0.1971	-0.0232
CD/CEB * lag 3 * child age bin 2	0.0212	-0.0885	0.1308
CD/CEB * lag 1 * child age bin 3	0.003	-0.0817	0.0876
CD/CEB * lag 2 * child age bin 3	-0.1392	-0.233	-0.0455
CD/CEB * lag 3 * child age bin 3	-0.3275	-0.4439	-0.2111
CD/CEB * lag 1 * child age bin 4	-0.0792	-0.1908	0.0323
CD/CEB * lag 2 * child age bin 4	-0.2944	-0.4183	-0.1706
CD/CEB * lag 3 * child age bin 4	-0.4218	-0.5768	-0.2667
Variance of country random effect	0.0101	0.0052	0.0212
Variance of survey random effect	0.0056	0.0037	0.0090

Supplementary Table 3: SBH Adjustment model results for the *N* model. SBH=Summary birth history. Reference levels for factor variables lag and child age bin are lag 0 and child age bin 1.

Variable	Mean coef. estimate	2.5% UI	97.5% UI
Intercept	4.6750	3.5904	5.7556
log(CEB)	0.9499	0.9069	0.9928
lag 1	-0.8378	-0.9235	-0.7523
lag 2	-1.7154	-1.8859	-1.5452
lag 3	-2.7182	-2.9740	-2.4630
child age bin 2	2.5561	2.5188	2.5933
child age bin 3	3.3709	3.3344	3.4074
child age bin 4	3.3548	3.3182	3.3913
mean maternal age	-0.1140	-0.1255	-0.1026
period	-0.1662	-0.2511	-0.0813
proportion mothers 15-19	-2.2269	-2.8797	-1.5749
proportion mothers 20-24	-1.5814	-2.0317	-1.1316
proportion mothers 25-39	-0.8190	-1.1570	-0.4815
P(15-19)/P(20-24)	-1.2373	-3.6473	1.1637
P(20-24)/P(25-25)	-3.4624	-5.8158	-1.1069
years in period	0.5652	0.5573	0.5730
log(CD)	-0.0578	-0.0955	-0.0201
log(CEB) * years in period	-0.0039	-0.0049	-0.0029
log(CEB) * child age bin 2	-0.0154	-0.0203	-0.0105
log(CEB) * child age bin 3	-0.0302	-0.0350	-0.0254
log(CEB) * child age bin 4	-0.0279	-0.0327	-0.0231
child age bin 2 * years in period	-0.0476	-0.0558	-0.0394
child age bin 3 * years in period	-0.0744	-0.0824	-0.0664
child age bin 4 * years in period	-0.0923	-0.1003	-0.0842
log(CEB) * child age bin 2 * years in period	0.0032	0.0022	0.0043
log(CEB) * child age bin 3 * years in period	0.0063	0.0053	0.0074
log(CEB) * child age bin 4 * years in period	0.0054	0.0044	0.0065
lag 1 * mean maternal age	0.0096	0.0092	0.0099
lag 2 * mean maternal age	0.0363	0.0359	0.0367
lag 3 * mean maternal age	0.0621	0.0615	0.0627
Variance for country random effect	0.0355	0.0110	0.1446
Variance for admin1 random effect	0.1501	0.1408	0.1600
Variance for survey	0.1942	0.1407	0.2760

6.3 Out of sample validation of SBH Adjustment Model

We used five-fold cross validation to test how well these models predicted *p* and *N* out-of-sample. Using the two training datasets, we fit each of the models five times, each time holding out the response variable from random subset of one-fifth of the surveys. The plot below shows the out-of-sample fits by child age bin, and the table below shows summary predictive validity metrics for the two models for each child age-year bin.

Supplementary Figure 4. Out of sample fits for SBH adjustment models. SBH = summary birth history. X axis: Out of sample data at from the SBH adjustment model. Y axis: observed truth from CBH tabulations. 4A shows out of sample predictions from the *p* model for monthly probability of death. 4B shows the out of sample predictions from the *N* model for monthly exposures. Plotted in modelled logit and log scales, respectively. Colours indicate child-age groups. Red line indicates unity. SBH=Summary birth history.

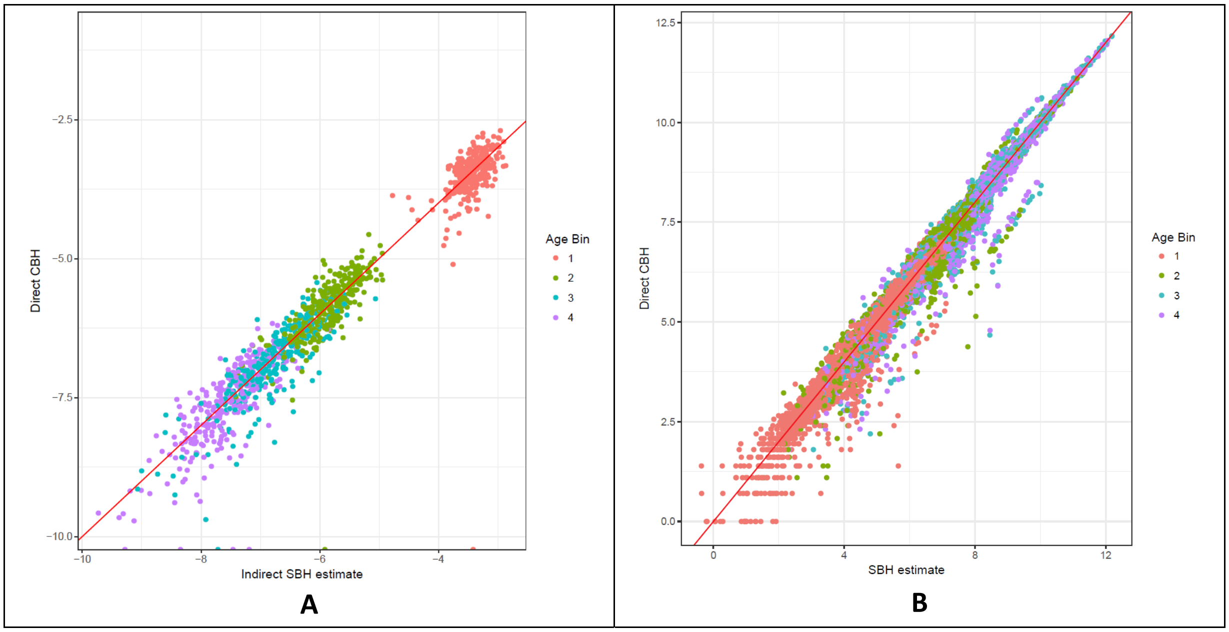

Supplementary Table 4. Out-of-sample predictive validity metrics for SBH adjustment models in probability and exposure month space, respectively. Calculated using five holdout folds.

Age Bin	Year	Mean *p* (observed)	mean $\hat{p}$	RMSE	ME	Mean *N* (observed)	Mean $\hat{N}$	RMSE	ME
1	**2015**	0.02534	0.02446	0.00996	-0.00089	181.5	188.6	66.5	7.1
2	**2015**	0.00166	0.00204	0.00075	0.00038	2068.0	2104.4	685.5	36.4
3	**2015**	0.00067	0.00077	0.00028	0.00010	4299.3	4247.1	1428.4	-52.2
4	**2015**	0.00041	0.00037	0.00020	-0.00004	4138.4	4128.8	1347.0	-9.6
1	**2010**	0.02866	0.02670	0.00716	-0.00196	407.5	433.8	94.4	26.3
2	**2010**	0.00236	0.00239	0.00075	0.00004	4279.0	4469.9	756.3	190.9
3	**2010**	0.00095	0.00096	0.00036	0.00002	8843.8	8934.3	1152.2	90.5
4	**2010**	0.00044	0.00044	0.00020	0.00000	8354.2	8123.0	1156.8	-231.2
1	**2005**	0.03120	0.03126	0.00723	0.00006	421.9	418.1	95.4	-3.8

Age Bin	Year	Mean p (observed)	mean $\hat{p}$	RMSE	ME	Mean N (observed)	Mean $\hat{N}$	RMSE	ME
2	**2005**	0.00324	0.00302	0.00090	-0.00022	4346.7	4307.7	815.6	-39.0
3	**2005**	0.00132	0.00133	0.00040	0.00001	8572.6	8559.1	1313.4	-13.5
4	**2005**	0.00059	0.00060	0.00020	0.00001	7691.9	7659.0	1108.8	-33.0
1	**2000**	0.03482	0.03633	0.00795	0.00152	386.6	373.9	73.5	-12.7
2	**2000**	0.00399	0.00379	0.00110	-0.00020	3915.9	3835.7	609.8	-80.2
3	**2000**	0.00174	0.00178	0.00049	0.00005	7637.5	7608.4	1157.5	-29.1
4	**2000**	0.00080	0.00079	0.00030	-0.00001	6592.9	6750.3	1424.1	157.3

6.4 Inverse-variance weighting of SBH observations

To account for the fact that the adjusted SBH approximated binomial data points were modelled, we chose to weight SBH observations in the prediction proportionally to the uncertainty in each prediction from the two-stage model. These weights are then used when fitting the full geostatistical model.

For each observation of SBH-only data, we took 10,000 draws from the predictive posterior distributions of $\hat{p}$ and $\hat{N}$. For each draw m we then simulated $\widehat{N^+}_m$ from a binomial distribution with parameters $\widehat{p_m}$ and $\widehat{N_m}$, giving us 10,000 corresponding draws of $\widehat{N^+}$. We then took the weights as the inverse of the ratio of the simulated variance of $\widehat{N^+}$and the expected binomial variance $E[\hat{N}]E[\hat{p}](1 - E[\hat{p}])$. As defined, the weights represent the proportion of excess variance in these observations induced by the two-stages of *N* and *p* modelling.

In 7.2% of our predicted SBH observations, weights exceeded 1 because small N and small monthly death probabilities resulted in 10,000 draws of nearly all zeros. This caused very low variance of observed deaths across the draws (lower than expected) and inflated weights. We view this as a computational issue in managing to capture low binomial variance via simulation in situations where we have high confidence that that the outcome should be 0. Taking more draws from the predictive posterior distributions of $\hat{p}$ and $\hat{N}$ is computationally expensive and for these observations we set the weight to 1. Weights ranged from 0.00013 to 1.0, with 25% falling below 0.36, a median weight of 0.75, and a 75th quantile of 0.91.

7. Applying GBD bias correction

Prior to geostatistical modelling, we adjusted mortality data by applying the bias correction ratios used by Wang and colleagues for GBD 2016.[3] Briefly, raw under-5 mortality data may differ due to various source-specific biases, sampling-related or non-sampling, and it is thus desirable to account for them when possible in order to generate a consistent time series of under-5 mortality. To adjust for this, a non-linear mixed effects model with source-type specific fixed effects and source-specific random effects nested within a location was fit by Wang and colleagues.[3] In addition, one high-quality reference source is chosen as the reference based on expert opinion and general data quality from specific survey series for each country. In the case of Africa, this is typically complete birth histories from DHS. For all non-reference sources, data are adjusted based on the difference between the combination of source-type fixed effects and source specific random effects between the source of interest and the reference source. The ratio of the unadjusted mortality to the adjusted from the model are thus country-source-year specific. We converted these to

monthly ratios and multiplied the number of deaths in each bin-period-cluster by them for all matching records from non-reference data sources.

8. Spatial integration over polygon records

Supplementary Figure 5, shown below, illustrates the k-means clustering method used to generate spatial integration points for areal mortality estimates. For each geographic area, we extracted a raster layer of population counts with cell area of approximately 5x5 km from an Africa-wide population raster for 2010 from the WorldPop project (panel A).[8] We then sampled 10,000 point locations with replacement from each area, with sampling probability proportional to cell population, to simulate a random sample of the population of the area (panel B). Finally, we applied k-means clustering to the latitudes and longitudes of these locations, with J clusters, such that there was one cluster per 100 raster cells within each polygon area. For each of the J cluster centres, we computed the integration weight as the proportion of the representative points falling within this cluster (being geographically closer to that cluster centre than any other cluster centre) (panel C). These weighted points (pseudo-clusters, representing areal estimates) are then combined with true geographically referenced clusters to create the dataset to train the geostatistical model.

Supplementary Figure 5: Illustration of k-means clustering for selecting spatial integration points and weights for polygon mortality data, applied to the district of Makonde, Zimbabwe. A) Spatial raster of cell-level population counts (blue cells indicating larger populations), B) locations of 10,000 population-representative point locations (with added spatial jitter for illustrative purposes), C) 11 spatial integration points selected by k-means clustering, with the size of each point proportional to its integration weight.

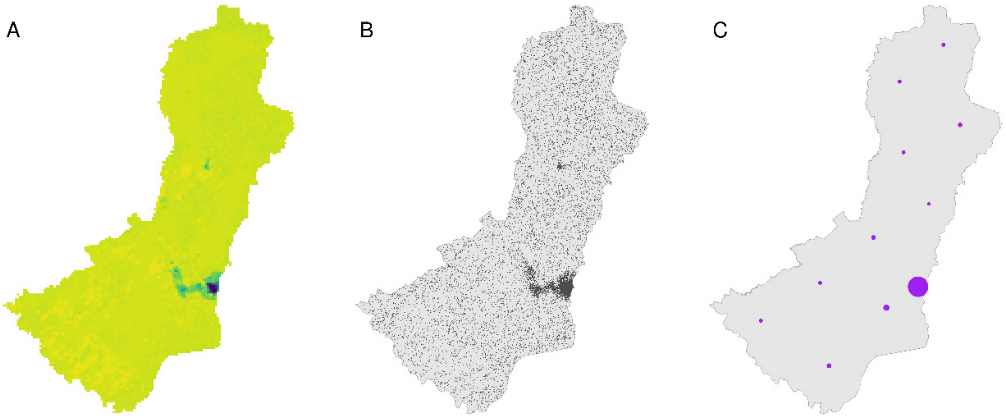

9. Raw data plots

The plots in Supplementary Figures 6 through 10 show the model input data for the four age bins over time. Monthly probabilities of death are plotted over a 48 x 48 pixel lattice over Africa. Pixels represent average mortality probabilities from points falling within each pixel. Plots are unweighted by sample size and are meant only to give a general idea of data coverage and values.

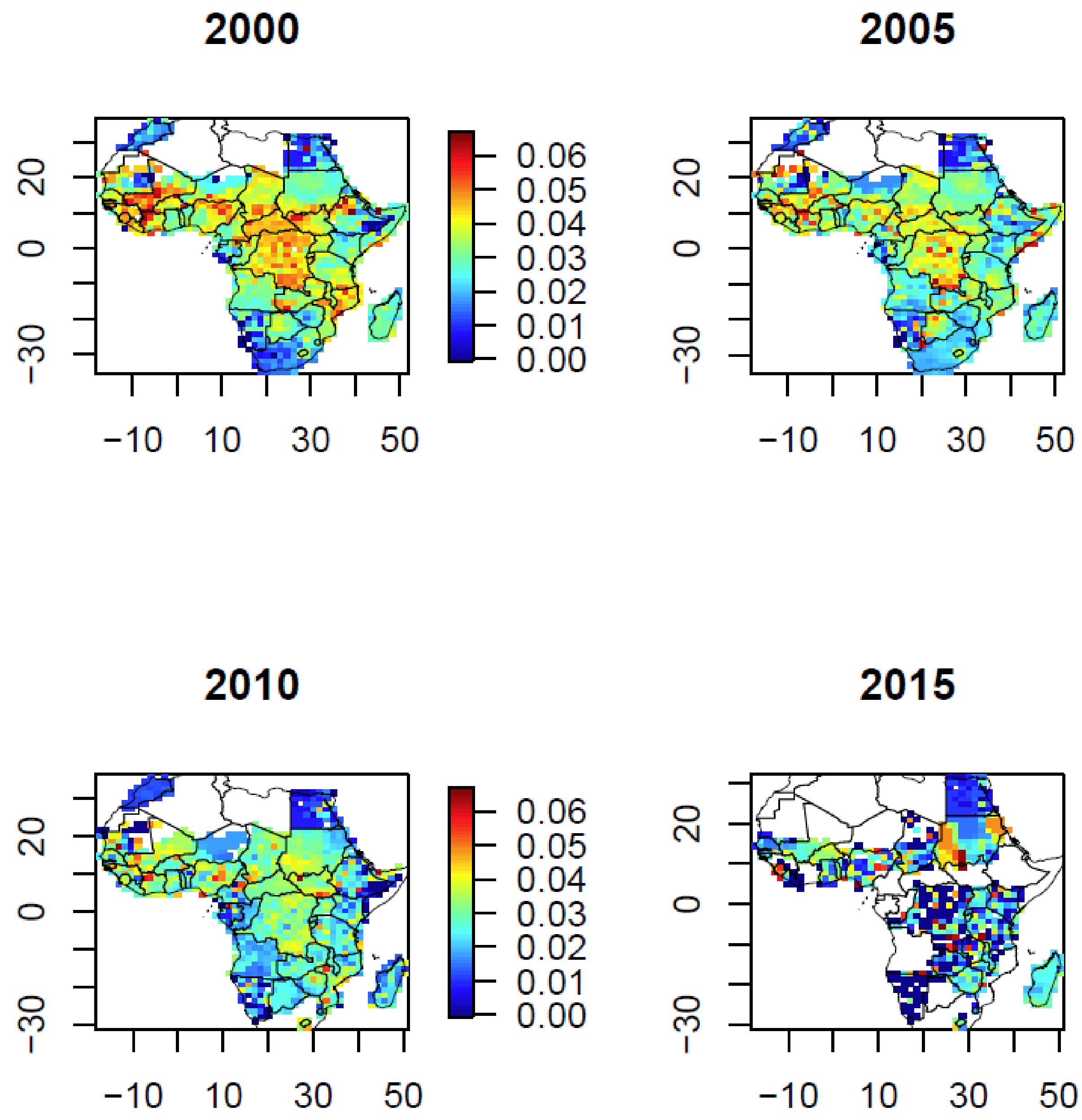

Supplementary Figure 6: Model input data for age bin 1 (neonatal), values indicate the mean monthly probability of death among clusters in each grid cell.

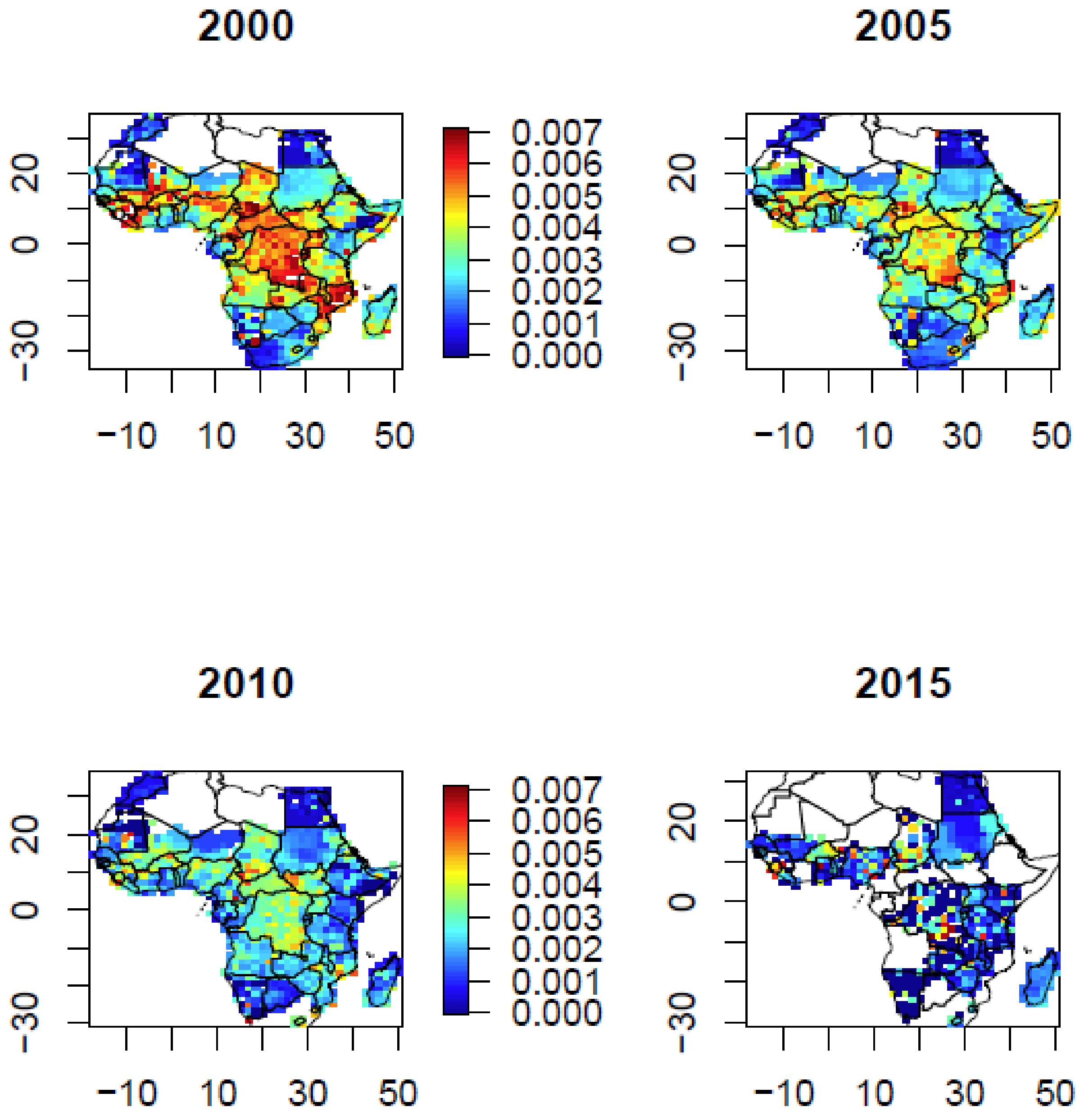

Supplementary Figure 7: Model input data for age bin 2 (1 – 11 months), values indicate the mean monthly probability of death among clusters in each grid cell.

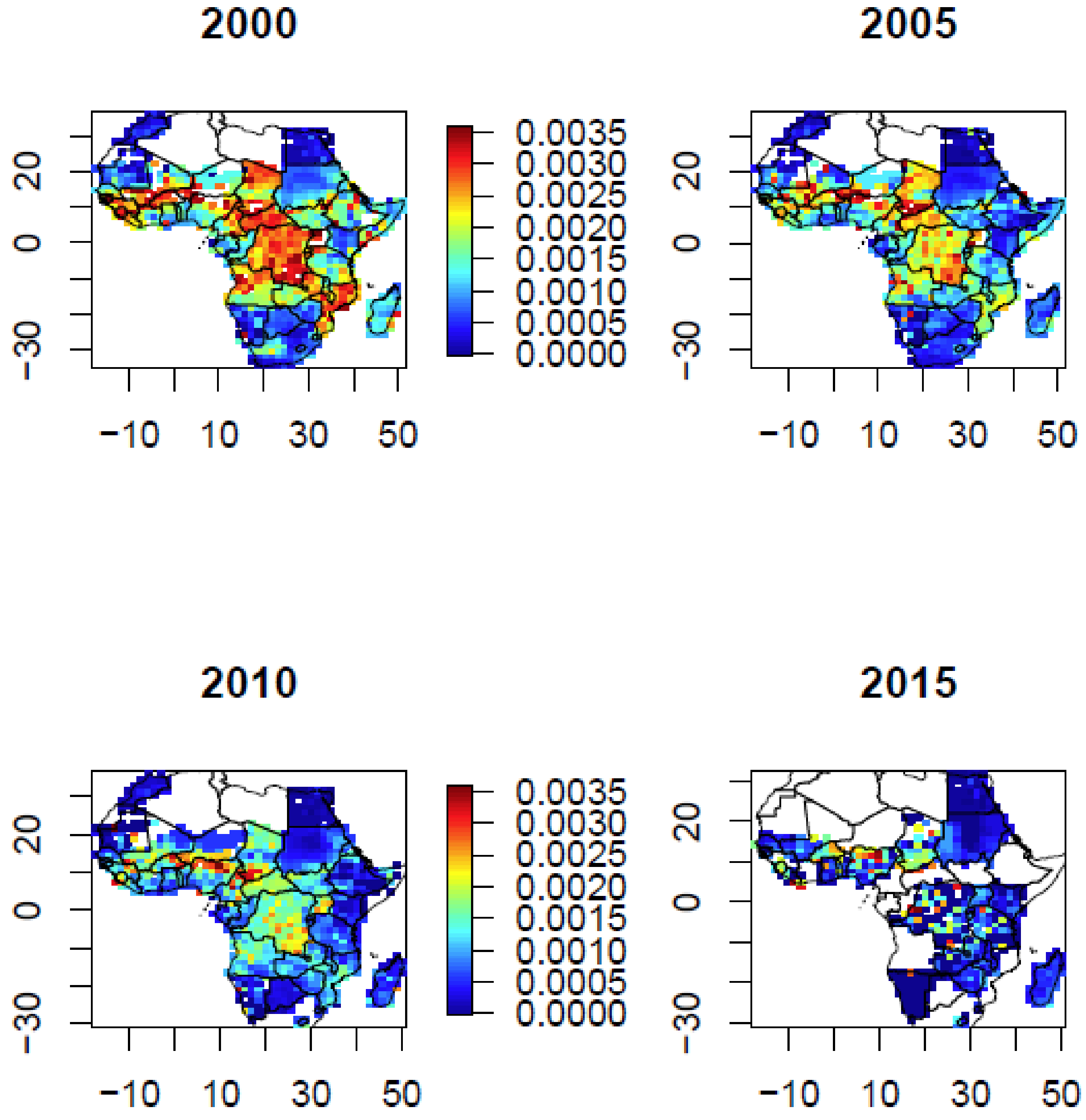

Supplementary Figure 8: Model input data for age bin 3 (12 – 35 months), values indicate the mean monthly probability of death among clusters in each grid cell.

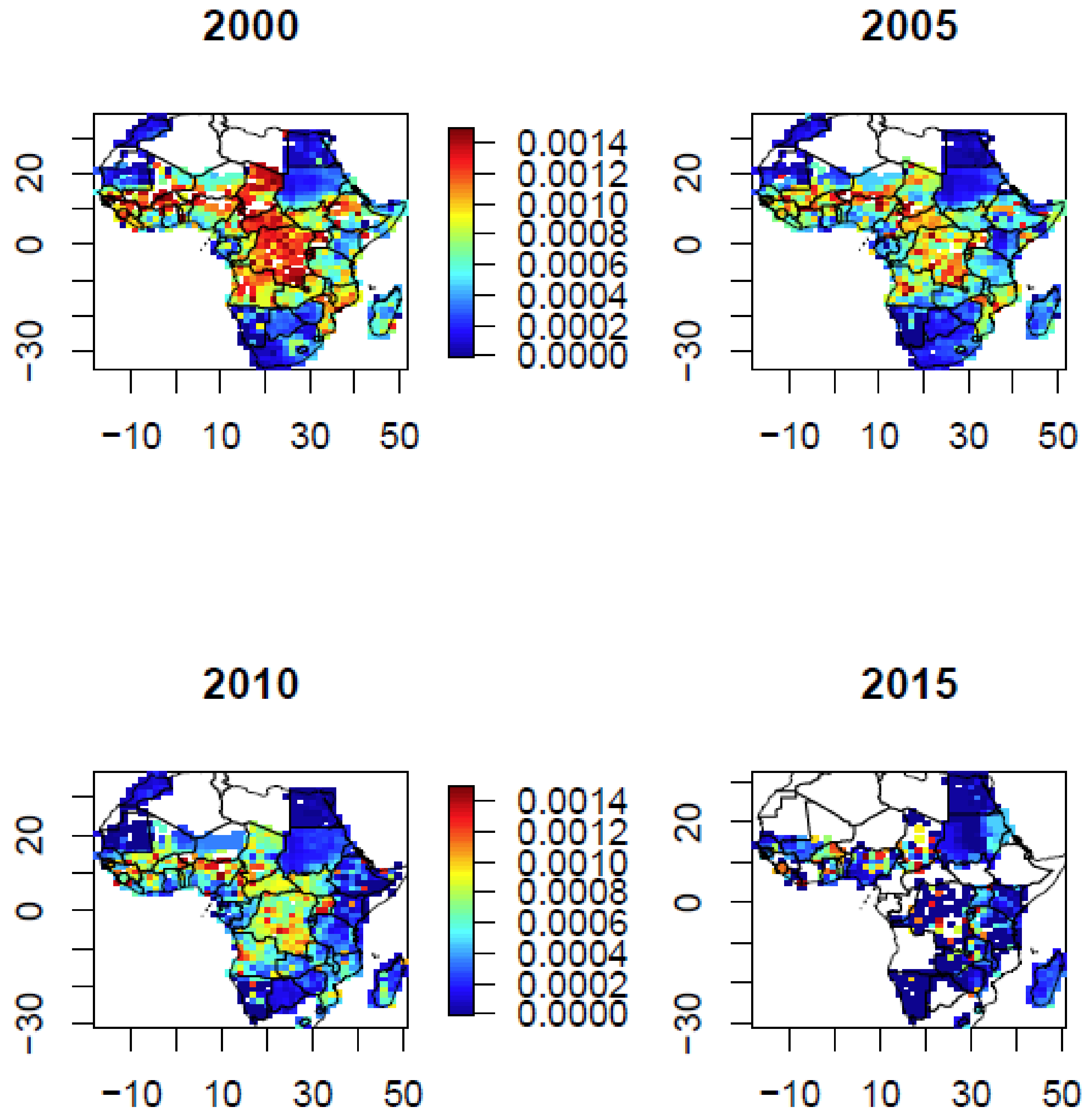

Supplementary Figure 9: **Model input data for age bin 4 (36 – 59 months), values indicate the mean monthly probability of death among clusters in each grid cell.**

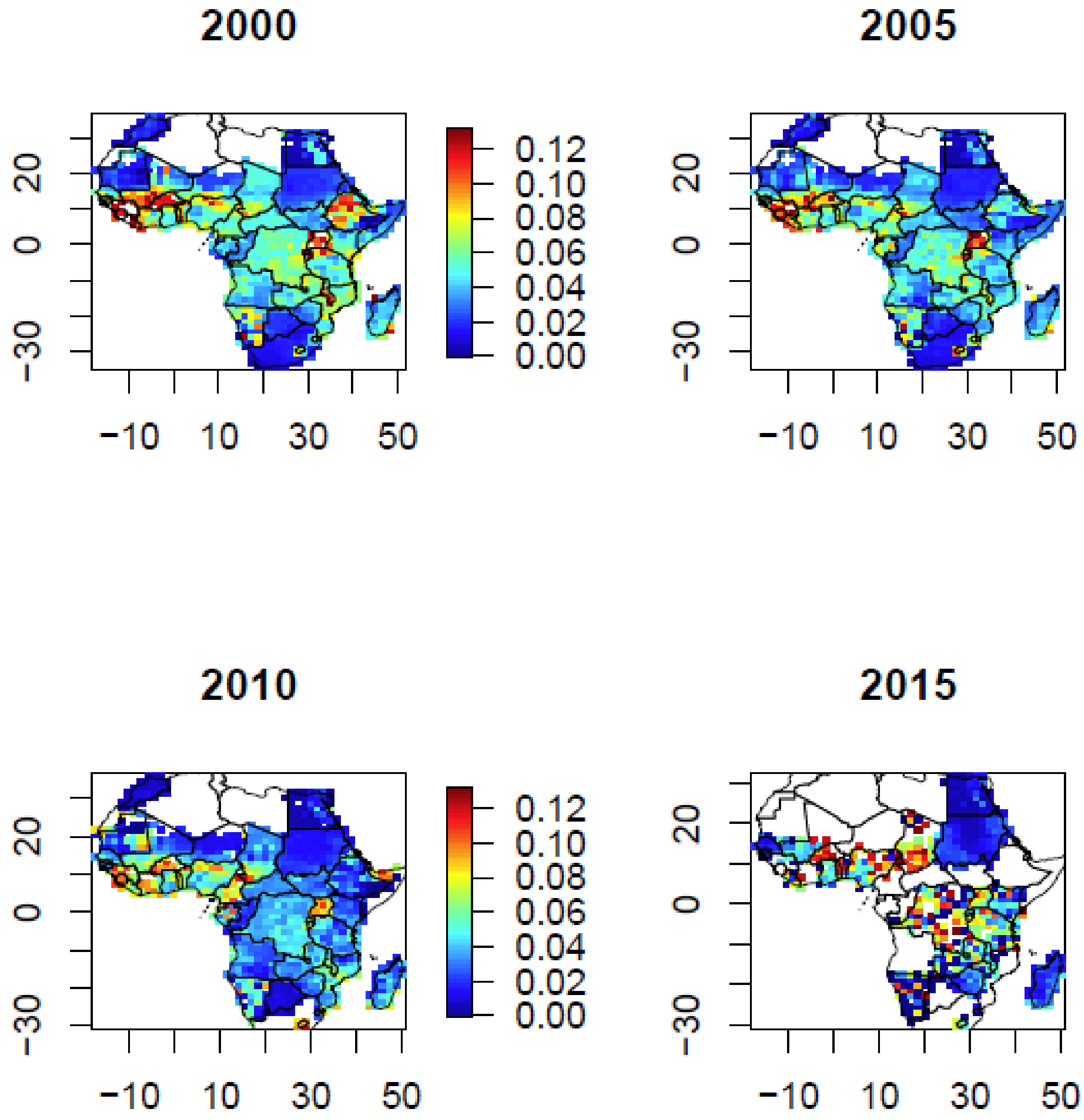

Supplementary Figure 10: Combined input data for age bins1-4, approximating under-5 mortality.

10.Covariates and covariate transformation

10.1 Covariates

Supplementary Table 5. Description of covariate layers used and their sources. For years, if multiple years were provided (included in parentheses), we took a synoptic mean.

Plot	Covariate	Years	Source
A	Travel time (in seconds) to nearest population centre (of at least 50,000 population)	2000	Uchida & Nelson, 2008: Agglomeration Index: Towards a New Measure of Urban Concentration http://forobs.jrc.ec.europa.eu/products/gam/index.php
B	Night-time light intensity	2000 (1997-2002); 2005 (2003-2007); 2010 (2008-2012); 2015 (2013)	Image and data processing by NOAA's National Geophysical Data Center. DMSP data collected by US Air Force Weather Agency https://www.ngdc.noaa.gov/eog/dmsp/downloadV4composites.html
C	Population	2000, 2005, 2010, 2015	Christopher T. Lloyd, Alessandro Sorichetta & Andrew J. Tatem. High resolution global gridded data for use in population studies. Scientific Data 4, Article number: 170001 (2017) doi:10.1038/sdata.2017.1 http://www.worldpop.org.uk/data/get_data/
D	Fertility proxy index (ratio of under-5 children to women of reproductive age [15-49 years])	2000, 2005, 2010, 2015	Christopher T. Lloyd, Alessandro Sorichetta & Andrew J. Tatem. High resolution global gridded data for use in population studies. Scientific Data 4, Article number: 170001 (2017) doi:10.1038/sdata.2017.1 http://www.worldpop.org.uk/data/get_data/
E	Irrigation (area irrigated per pixel)	2000	Siebert at al 2005: Development and validation of the global map of irrigation areas https://www.uni-frankfurt.de/45218039/Global_Irrigation_Map
F	Urban-rural dichotomous (as measured by Boolean classification)	2000 (1999-2002); 2005 (2003-2008); 2010 (2008-2012); 2015 (2013-2014)	Pesaresi et al 2016: Operating procedure for the production of the Global Human Settlement Layer from Landsat data of the epochs 1975, 1990, 2000, and 2014 http://ghsl.jrc.ec.europa.eu/data.php
G	Enhanced vegetation index (0-1 scale)	2000 (2000-2002); 2005 (2003-2007); 2010 (2008-2012); 2015 (2013-2015)	Huete et al 1999: MODIS vegetation index Algorithm theoretical basis document https://lpdaac.usgs.gov/dataset_discovery/modis/modis_products_table/mod13a1
H	Average daytime temperature in C°	2000 (2000-2002); 2005 (2003-2007); 2010 (2008-2012); 2015 (2013-2015)	Wan 1999: MODIS Land-Surface Temperature Algorithm Theoretical Basis Document https://lpdaac.usgs.gov/dataset_discovery/modis/modis_products_table/mod11a2
I	*Plasmodium falciparum* parasite rate (*Pf*PR)	(1998-2002)->2000 (1998-2000); 2005 (2003-2007); 2010 (2008-2012); 2015 (2013-2016)	Bhatt et al 2015: The effect of malaria control on Plasmodium falciparum in Africa between 2000 and 2015 http://www.map.ox.ac.uk/
J	Average years of education for women aged 15-49 years	(2000-2002)->2000 (2000-2002); 2005 (2003-2007); 2010 (2008-2012); 2015 (2013-2015)	Currently unpublished, correspondence to Simon Hay.
K	Prevalence of moderate and severe stunting for children under 5 (as measured by -2SD height for age Z-scores)	(2000-2002)->2000 (2000-2002); 2005 (2003-2007); 2010 (2008-2012); 2015 (2013-2015)	Currently unpublished, correspondence to Simon Hay.
L	Prevalence of moderate and severe wasting for children under 5 (as measured by -2SD weight for height Z-scores)	(2000-2002)->2000 (2000-2002); 2005 (2003-2007); 2010 (2008-2012); 2015 (2013-2015)	Currently unpublished, correspondence to Simon Hay.

Supplementary Figure 11: Images of spatial covariates used (2015 values shown here). EVI = enhanced vegetation index; LST = land surface temperature; PfPR = *Plasmodium falciparum* parasite rate.

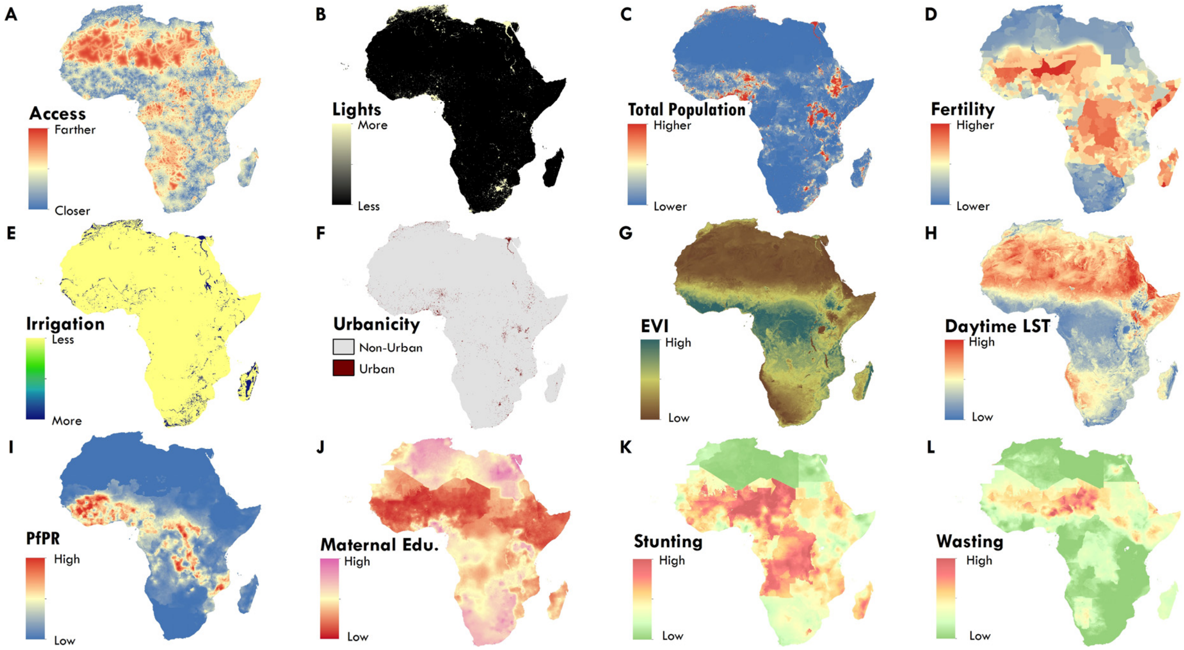

10.2 Covariate selection and transformation with stacking

Stacked generalization/regression, or stacking, is a method of combining (ie, ensembling) multiple predictive models in order to enhance predictive validity relative to a single approach or model[9,10] and has been shown to be effective for geostatistical exercises.[11] In short, a suite of child models are fit using different modelling approaches and then combined, or stacked, by using a secondary learner, or stacker. Our implementation of stacking largely follows the approach described in Bhatt and colleagues.[11]

Our stacking hierarchy features two levels: a collection of 'child' models and a stacker model. Although there are possible other configurations, this direct, two-stage approach was taken because of its simplicity, computational tractability, and previous success.[11] The child models used for this study include a generalized additive model, a gradient boost machine (also known as boosted regression trees), and penalized regressions (ridge, lasso, and elastic net). All analyses were performed in R,[12] using the mgcv, dismo, and glmnet packages.

Each child model was fit 5+1 times, where five is the number of folds for out-of-sample cross validation, plus one full data model. Data are divided into five folds and for each fold a given model is fit while leaving out one fold of data and training on the remaining data. The left-out fold subsequently serves as out-of-sample test dataset for prediction. Once the out-of-sample cross-validation process is complete for every fold the child model is then trained on the entire dataset. By the end of this process, each observation has an out-of-sample cross-validated prediction and full-fit prediction for each child model. Both the cross-validated predictions and the full-fit predictions are preserved for the subsequent stacking.

The final stacker model (the geostatistical model described in the next section) is fit using the cross-validated predictions of the child models as covariates. By fitting on the cross-validated predictions we incorporate the generalization capabilities of each child model and otherwise reduce overfitting. If correlations in predictions from a pair of child models exceeded 0.99 we dropped one of the models. In all cases, elastic net and lasso regressions met this threshold of correlation and thus we dropped elastic net. Model correlations are reported in supplementary table 6. Final predictions from the geostatistical model were produced using the full-fit predictions from the constituent child models. Supplementary figure 12 illustrates child model full-fit predictions for each child model for age bin 1 in 2000. Child models were fit separately for each age bin and time period.

Supplementary Figure 12. Plots illustrating child model estimates of mortality for one age bin and year (age bin 1 in 2000). Each of these estimated surfaces is then used as a covariate in the geostatistical model. A. Generalized Additive Model (GAM); B. Boosted Regression Trees (BRT); C. Ridge regression; and D. Lasso regression.

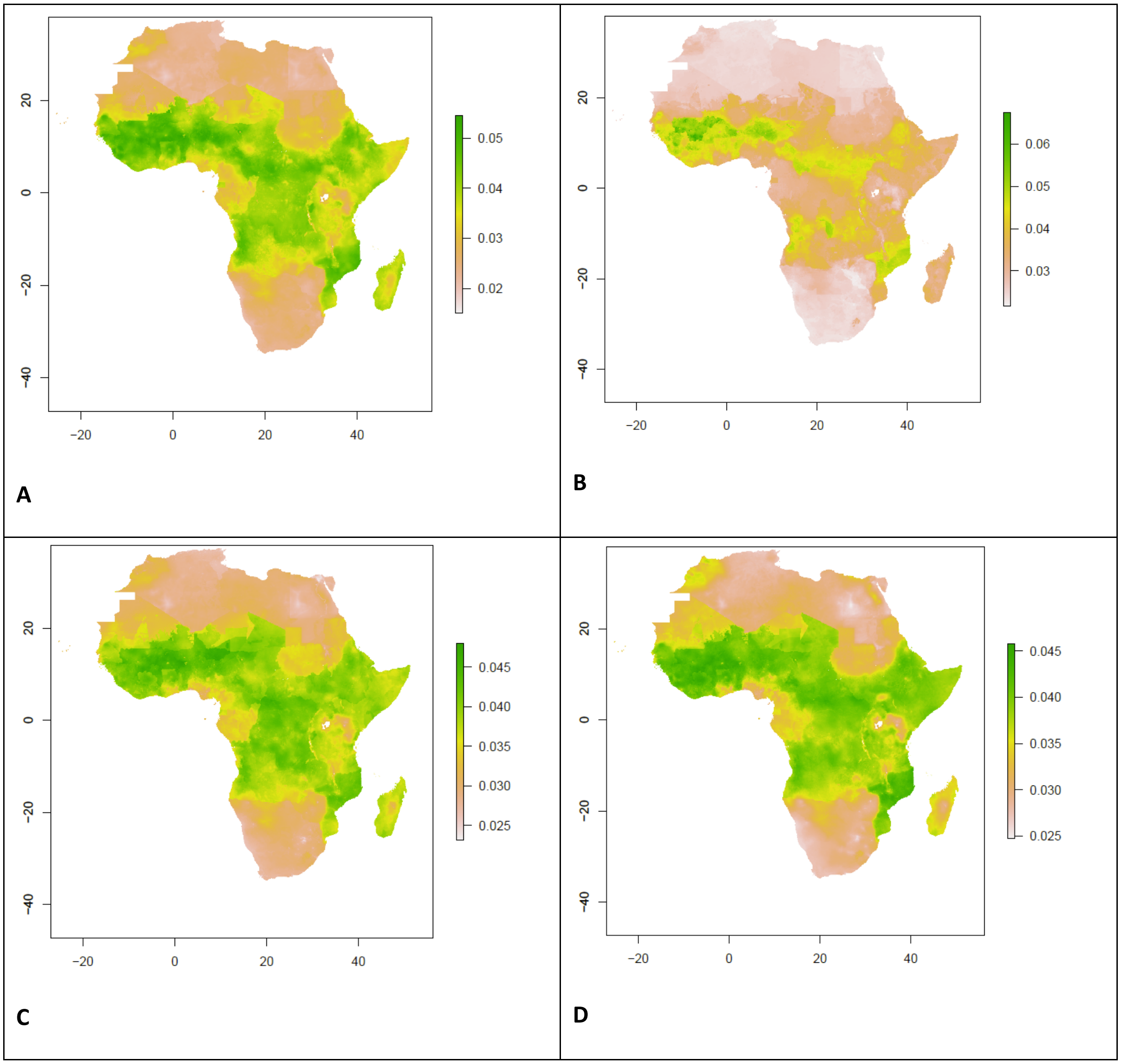

Supplementary Table 6. Pairwise correlations between stacking model fits. Elastic Net was dropped due to high correlation with lasso. * = correlation exceeding 0.99.

Model 1	Model 2	Age Bin 1	Age Bin 2	Age Bin 3	Age Bin 4
**GAM**	**BRT**	0.928	0.947	0.947	0.939
**GAM**	**Ridge**	0.850	0.956	0.961	0.954
**GAM**	**Elastic Net**	0.831	0.941	0.945	0.935
**GAM**	**Lasso**	0.824	0.940	0.940	0.933
**BRT**	**Ridge**	0.820	0.934	0.931	0.922
**BRT**	**Elastic Net**	0.828	0.938	0.938	0.937
**BRT**	**Lasso**	0.822	0.938	0.938	0.938
**Ridge**	**Elastic Net**	0.953	0.978	0.986	0.979
**Ridge**	**Lasso**	0.953	0.974	0.982	0.977
**Elastic Net**	**Lasso**	0.991*	0.997*	0.997*	0.995*

11. Geostatistical model

11.1 Model Geographies

In total, we ran four geostatistical models, one for each age bin.

11.2 Model description

The underlying statistical model, fitted separately for each of the four age bins was a spatially and temporally explicit hierarchical generalised linear regression model for binomial data, using the logit link function:

$$N_i^+ \sim Binomial(p_i, N_i)$$

$$logit(p_i) = \alpha + \boldsymbol{X}_i\boldsymbol{\beta} + \epsilon_{GP\,i}$$

$$\epsilon_{GP} \sim GP\left(0, \boldsymbol{K}_{space} \otimes \boldsymbol{K}_{time}\right)$$

$$\boldsymbol{K}_{space} = \left(\tau 2^{\nu-1}\Gamma(\nu)\right)^{-1}(\kappa\boldsymbol{D})^{\nu}K_{\nu}(\kappa\boldsymbol{D})$$

$$\boldsymbol{K}_{time_{k,l}} = \rho^{|t_k - t_l|}$$

where N_i are the number of monthly exposures recorded in survey cluster/period *i*, N_i^+ are the number of deaths resulting from all monthly exposures, p_i is the estimated cluster-level 5-year period mortality probability of death, modelled as a logit-linear function of the global intercept α, cluster-level covariate values $\boldsymbol{X}_i$ and vector of regression coefficients $\boldsymbol{\beta}$. One fixed effect was included for each of the predictions from the cross-validated 'child' stacking models. Spatiotemporally correlated residuals ϵ_{GP} are drawn from a three-dimensional, zero-mean Gaussian process (GP) with covariance matrix constructed as the Kronecker product of a spatial covariance matrix $\boldsymbol{K}_{space}$ and temporal covariance matrix $\boldsymbol{K}_{time}$. $\boldsymbol{K}_{space}$ was defined by a stationary Matérn covariance function over the Euclidean distance matrix $\boldsymbol{D}$ between all survey cluster locations, with spatial decay parameter κ, spatial smoothness/complexity parameter ν, precision

parameter τ, modified Bessel function of the second kind K_ν, and Gamma function $\Gamma(.)$. Since this Matérn covariance function is the stationary solution of a stochastic partial differential equation (SPDE), it enables the use of efficient statistical machinery for modelling with SPDEs, as described below. We set the complexity parameter ν to be fixed at 2. $\boldsymbol{K}_{time}$ was defined by the covariance function corresponding to the discrete-time autoregressive stochastic process of the first order (AR1). The AR1 process is typically defined over a nominal random variable: $x_t = \rho\, x_{t-1} + N(0, \sigma^2)$ where t indexes time (in our case the period) and ρ (which is constrained such that $|\rho| < 1$) and σ^2 are parameters. When convolving the space and time correlation structures with this definition of the AR1 process, the spatial variance $1/\tau$ and the temporal variance σ^2 would be non-identifiable. We therefore omit σ^2 from our definition above, and represent overall space-time variance via the parameter $1/\tau$.

11.3 Priors

The following minimally informative priors were specified over parameters in all four age bin models: $\alpha \sim N(0, 1000)$, $\beta \sim N(0, 1000)$, $\log((1+\rho)/(1-\rho)) \sim N(0, 0.15)$. INLA sets an uncorrelated multivariate normal prior on log-transformations of κ and τ, and by default it determines priors based on the characteristics of the finite elements mesh (described in the section below). We use the default minimally informative priors that INLA suggests which, in our setting, yielded $\theta_1 = \log(\tau) \sim N(0.378, 10)$ and $\theta_2 = \log(\kappa) \sim N(-1.64, 10)$.

11.4 Model fitting

Models were fitted by integrated nested Laplace approximations (INLA) and a stochastic partial differential equation (SPDE) representation of the Gaussian-Markov random field (GMRF) approximation to the GP model,[14] using the INLA R package.[15] The INLA-SPDE approach makes use of the close correspondence between a GMRF defined on a sufficiently dense lattice and a GP, the efficient numerical routines enabled by representing GMRFs as SPDEs, and efficient inference over the parameters of these models using the INLA method. These approximations enable us to carry out full Bayesian inference over the model for a very large dataset, where other inference methods (such as MCMC) would be computationally prohibitive. While the result is an approximation to the model posterior, this approach has been shown to have extremely high accuracy when compared with MCMC in both theoretical and real-world mapping problems.[16] We defined the GMRF on a lattice constructed by constrained, refined Delaunay triangulation within a convex hull no closer than five decimal degrees from the coastline of Africa (including Madagascar). Over land this lattice was constrained to have edge length no greater than 0.35 decimal degrees and over sea no greater than five decimal degrees. This was the densest possible lattice we were able to use over the study area at this time before running into computational issues. These fitted models were then used to generate 1,000 posterior samples each of mapped monthly mortality probability estimates for each of the four age groups by random sampling from the numerical approximation to the joint posterior density of the model parameters. These estimates were combined to estimate the pixel-level (marginal) predictive posterior mean neonatal and under-5 mortality probabilities and prediction uncertainty intervals (0.025% and 0.975%).

11.5 Model Results

Geostatistical model results are presented in the Supplementary Table 7. Spatial Matérn covariance parameters κ and τ have been transformed (as have their lower and upper 95% uncertainty intervals) to be

more interpretable. Range represents the distance in decimal degrees at which point approximately 90% of correlation has decayed, and is taken to be $\sqrt{8}/\kappa$. Nominal variance can be interpreted as the variance at each data point, in logit space, it is calculated as: $4\pi\kappa^2\tau^2$. The auto-regressive correlation coefficient for time, ρ, has not been transformed. Fixed effects include an intercept and the cross-validated predictions from the four included 'child' stacking models.

Supplementary Table 7: Model fits for each age-specific model. BRT = boosted regression trees; GAM = generalized additive model; GP = Gaussian process

	0 – 1 month			1 – 11 months			12 – 35 months			36 – 59 months		
	median	2.5%	97.5%	median	2.5%	97.5%	median	2.5%	97.5%	median	2.5%	97.5%
intercept	-0.291	-0.669	0.074	-0.235	-0.556	0.076	-0.931	-1.254	-0.622	-1.200	-1.669	-0.752
child model: lasso	0.521	0.229	0.812	0.399	0.210	0.589	0.291	0.132	0.449	0.089	-0.104	0.281
child model: ridge	-0.077	-0.356	0.203	0.038	-0.149	0.224	0.119	-0.060	0.297	0.085	-0.120	0.290
child model: BRT	0.360	0.238	0.481	0.396	0.312	0.478	0.345	0.280	0.409	0.424	0.349	0.500
child model: GAM	0.132	-0.020	0.283	0.138	0.015	0.259	0.128	0.041	0.214	0.255	0.151	0.358
GP: Range (Decimal Degrees)	4.914	4.097	5.869	3.058	2.687	3.477	3.356	2.976	3.885	6.307	5.084	7.767
GP: Nominal Variance	0.058	0.046	0.073	0.099	0.085	0.118	0.160	0.137	0.188	0.146	0.109	0.194
GP: AR1 correlation coefficient ρ	0.940	0.911	0.961	0.941	0.923	0.957	0.956	0.942	0.967	0.971	0.952	0.983

The figures below show model prediction surfaces for neonatal and under-5 mortality for the four study periods, as well as surfaces representing the lower (2.5%) and upper (97.5%) uncertainty bounds for each pixel.

Supplementary Figure 13. Mean, lower, and upper uncertainty interval predictions for under-5 mortality (5q0) in 2000, 2005, 2010, and 2015. Uncertainty intervals were taken as the 2.5% and 97.5% quantiles of 1,000 predictive draws for each pixel.

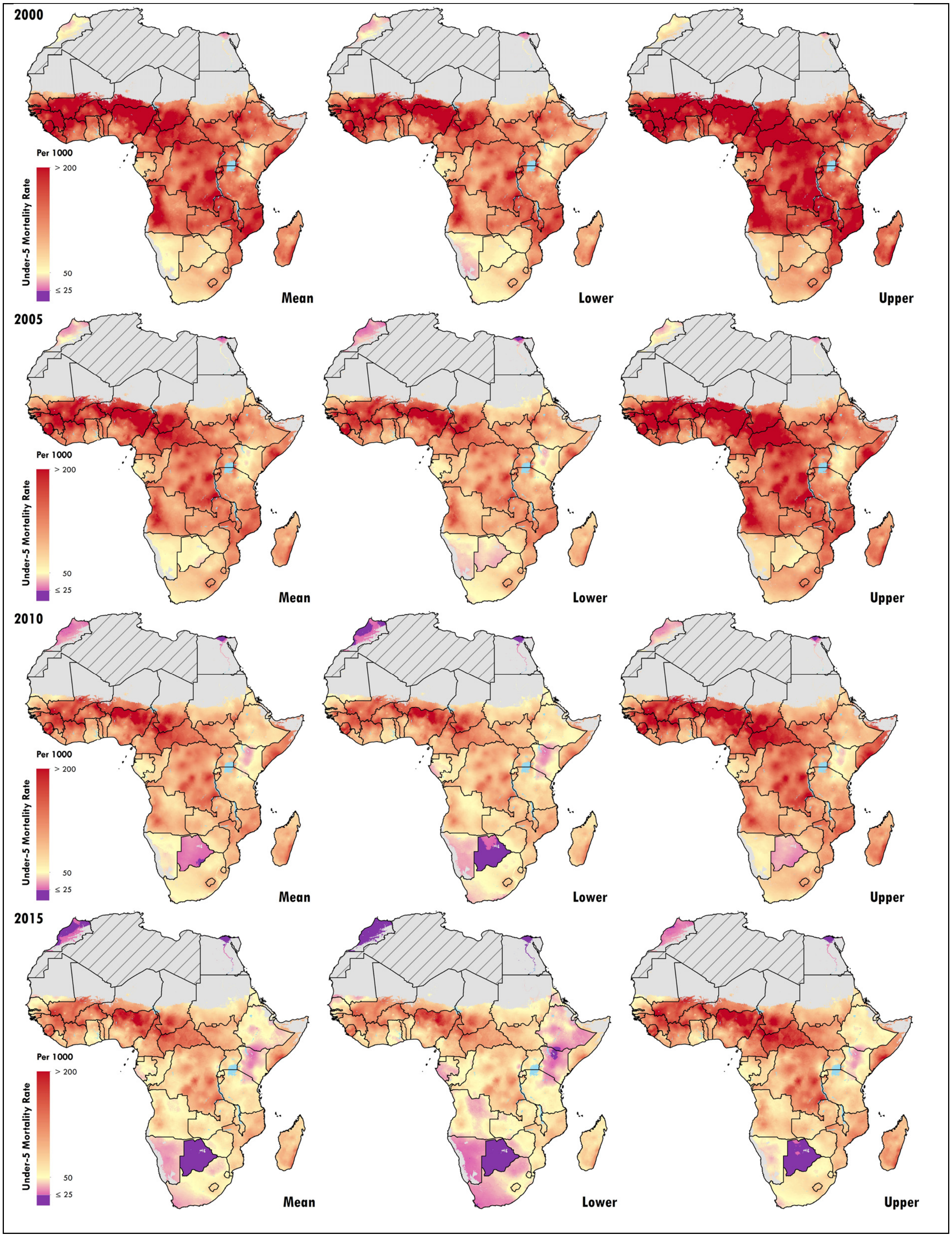

Supplementary Figure 14. Mean, lower, and upper uncertainty interval predictions for neonatal mortality in 2000, 2005, 2010, and 2015. Uncertainty intervals were taken as the 2.5% and 97.5% quantiles of 1,000 predictive draws for each pixel.

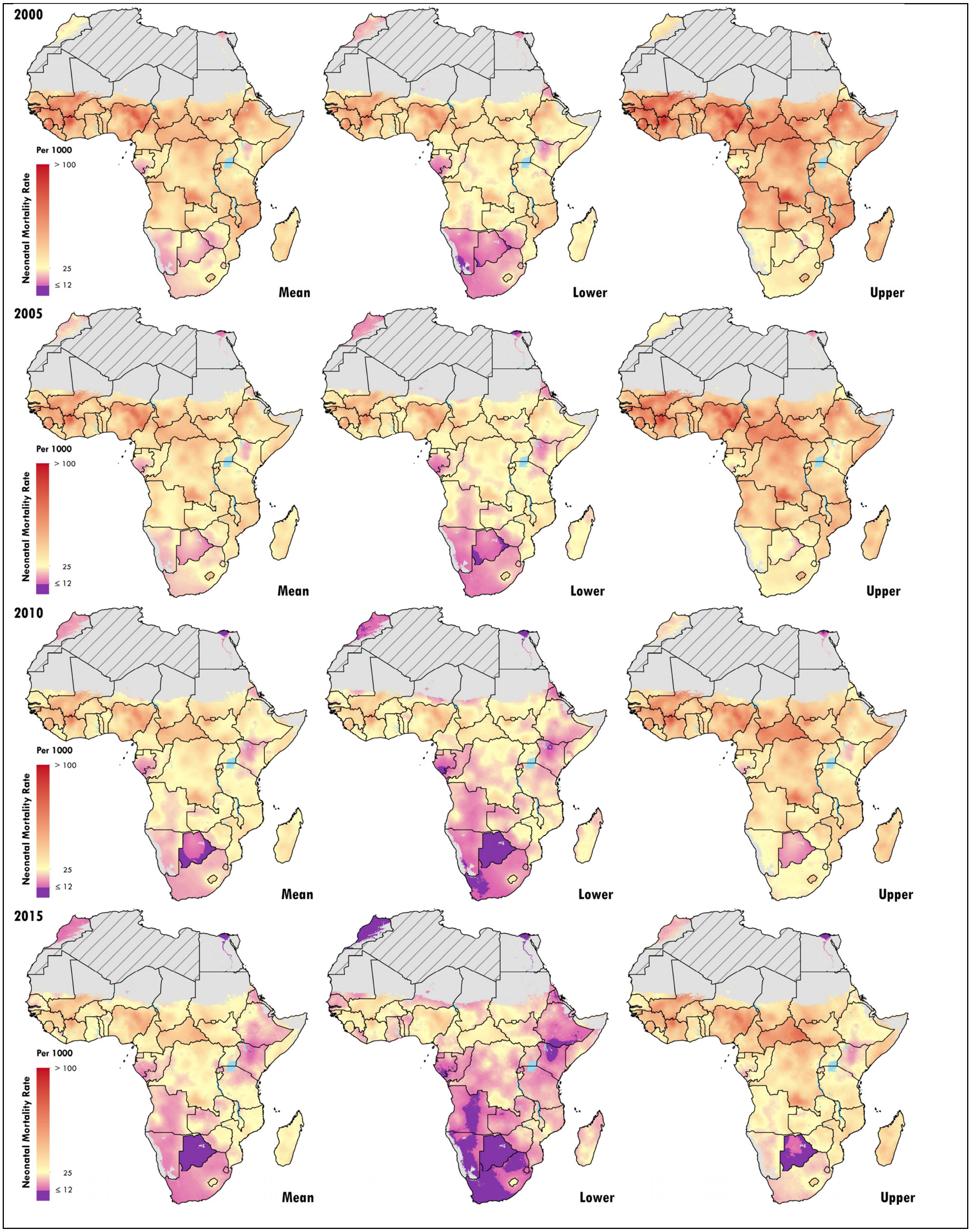

Supplementary Table 8 shows the posterior expectation of the deviance $D(\theta) = -2Log(p(y|\theta))$ for each model. In addition to the full models (described above), we ran a 'null' model, which only included an intercept and the spatiotemporal random effect. The relative differences of these deviances show how much additional information, at minimum, was gained by including covariates. Across all models the percent difference in deviance between the full and null model ranged from 0.99% to 1.32%.

Supplementary Table 8. Posterior expectation of the deviance for each model, both full and null, and their relative differences.

Age bin	Deviance		Percent difference
	Full model	Null model	
**0 – 1 month**	296222.6	300147.3	1.32%
**1 – 11 months**	339985.2	344045.8	1.19%
**12 – 35 month**	322265.1	325793.8	1.09%
**36 – 59 months**	242887.9	245299.5	0.99%

11.6 Model validation

In order to understand how well our model predicts mortality in locations where there are no data, we wish to estimate metrics of out-of-sample predictive validity. These are typically mean error (ME, to indicate level of bias), root-mean-squared error (RMSE, to indicate total variation in errors), correlation, and 95% coverage of the predictive intervals.

Model validation with these particular data presents a unique challenge. For relatively rare binomial data with small sample sizes, there is too much variance in the raw data to accurately assess model fit. In Supplementary Figure 15, we illustrate this using simulated data. Here we show that the empirical estimate taken as the ratio of events to observations in simulated binomial data with small *p* and relatively small *N* cannot reliably retrieve the probability (true p) they were simulated from. This means that even if we modelled mortality perfectly, we may not know it because many of our sample sizes are so small relative to the mortality proportion we are trying to estimate. The plot on the left shows this experiment done with N=100 (plus or minus some noise) and the plot on the right shows the same done with N=500 (plus or minus some noise). Adding sample size decreases variance in the empirical estimates, allowing for validation to be done. Many of our clusters are small, with median CBH clusters at 15, 150, 298, and 268 months for age bins 1 through 4, respectively. Furthermore, the monthly mortality probabilities which we estimate, particularly for age bins 3 and 4 are typically well below 0.001.

Supplementary Figure 15. Illustration of sample size impact on the ability to retrieve true probability from empirical binomial estimates.

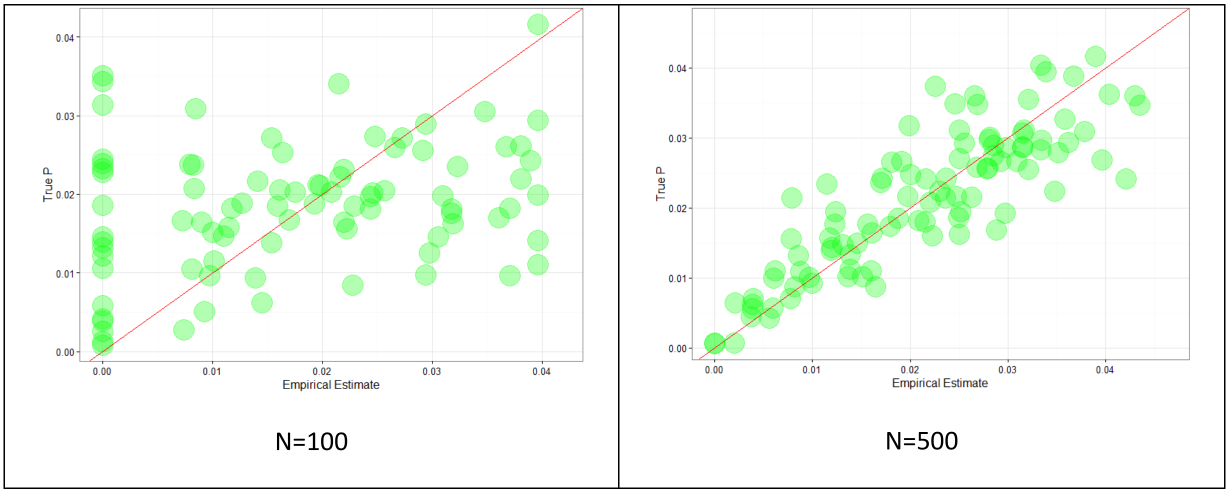

11.6.1 Spatial aggregation

We chose to aggregate spatially proximal data points within administrative 1 and 2 areas to stabilize estimates of model predictive validity. This offers several benefits: 1) points close to each other should be more similar, and thus if we hold out points randomly in space we will likely inflate metrics of predictive validity because we would have predicted them well based on neighboring points; and 2) by aggregating points and thus creating areas of data sparseness in the model fitting, we more closely reflect true data missingness patterns over un-sampled administrative areas. At the same time it is important to note that choice of aggregation level can be somewhat arbitrary and model fit will generally improve with larger sample sizes at larger areas of aggregation, and different administrative levels will have varying sample sizes.

To predict out of sample, stacking and geostatistical models are run five times for each age bin, each time holding out data assigned to each respective fold. All data points were assigned identified membership within a given administrative unit area. Each administrative area was then randomly assigned a fold. This was done for both level 1 and 2 administrative areas. Once each model had run, we calculated ME, RMSE, correlation, and 95% coverage for the data we held out. We aggregated predictions and data estimates at each administrative unit by taking weighted aggregates based on sample size adjusted for integration and SBH-adjustment weights. RMSE, ME, and correlation were calculated by comparing mean aggregated estimates and predictions across administrative units, weighted on the aggregated sample size at each unit. Coverage was calculated by simulating predictions of child deaths from posterior draws of probability of death and observed sample sizes at each cluster location, thus representing our ability to reproduce hold out data within the specified level of certainty.

11.6.2 Metrics of predictive validity

The tables below show how each model performed based on the metrics of bias, total variance, and coverage. Tables are shown for first administrative unit-level predictions (Supplementary Table 9), and second administrative unit-level predictions (Supplementary Table 10). The number of data points and exposure months in a given holdout area could vary greatly, and validation metrics will be sensitive to this.

Supplementary Table 9. Out-of-sample predictive validity for first administrative-level holdout predictions

Age Bin	Avg estimated monthly probability	Mean Exposure months	Mean Error	RMSE	Correlation	95% Coverage
0-1 month	0.0328	783	-0.0003	0.0058	0.8111	95.0%
1-11 months	0.0031	8134	0.0000	0.0006	0.9043	95.3%
12-35 month	0.0013	16640	0.0000	0.0003	0.9432	92.7%
36-59 months	0.0006	17180	0.0000	0.0002	0.8989	93.8%

Supplementary Table 10. Out-of-sample predictive validity for second administrative-level holdout predictions

Age Bin	Avg estimated monthly probability	Mean Exposure months	Mean Error	RMSE	Correlation	95% Coverage
0-1 month	0.0327	104	-0.0002	0.0107	0.6236	95.1%
1-11 months	0.0031	1110	0.0000	0.0011	0.8017	95.5%
12-35 month	0.0013	2342	0.0000	0.0005	0.8670	93.5%
36-59 months	0.0006	2446	0.0000	0.0003	0.7766	94.4%

Supplementary Figure 16. Data estimates for aggregated administrative 1 level holdouts versus mean out of sample predictions for the same locations. Estimates are taken at each point where there was data in a holdout area and then aggregated weighted by data sample size. Red lines indicate equivalence.

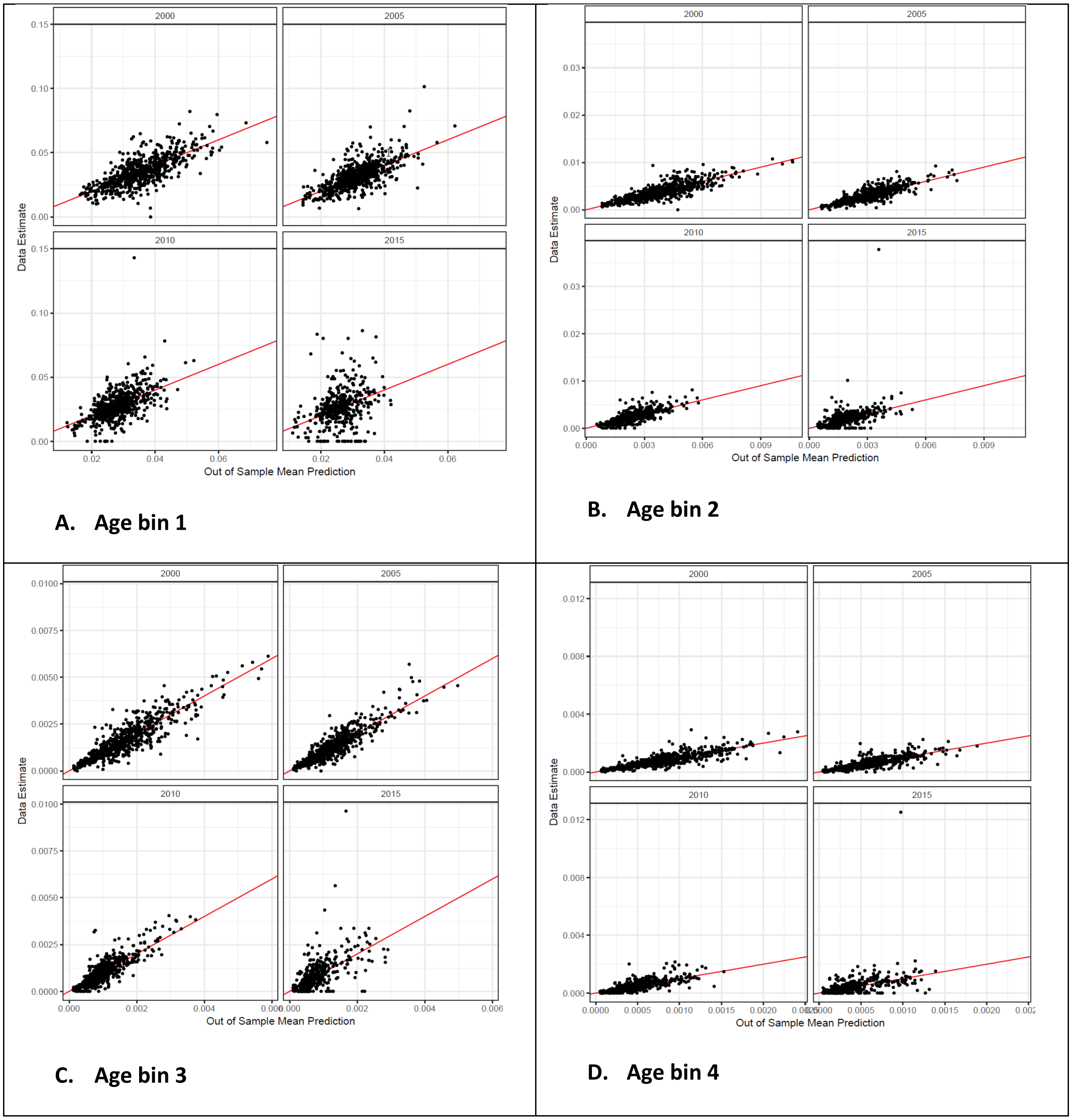

Supplementary Figure 17. Data estimates for aggregated administrative 1 level holdouts versus mean out of sample predictions for the same locations. Estimates are taken at each point where there was data in a holdout area and then aggregated weighted by data sample size. Red lines indicate equivalence.

Predictive agreement in the most recent time period was more difficult to assess because all survey data from this time period (2012-2017) included only data with partial period coverage, and thus smaller sample sizes than the previous time periods. As more data become available for future iterations of this work, we expect these predictions to improve.

12.Calibration to national estimates

As described in the main methods, we calibrated our mapped mortality rate estimates to match at the national level (or first administrative level where available) with mortality estimates from GBD 2016.[3] GBD estimates aggregate a wider pool of mortality data that are only available at a national level, including vital registration data. Supplementary figure 17 shows differences in population-weighted national-level estimates of mortality rates from the uncalibrated maps produced by our geostatistical modelling framework and GBD 2016 estimates for all countries and all four time periods. For each period, the average GBD estimate across the 5-years was used, with exception of the more recent period, for which GBD only makes estimates up to 2016 and thus 2017 was excluded. Maps were scaled to GBD by multiplying each pixel-draw by the ratio of national level GBD estimate to national level geostatistical estimate. National level geospatial estimates generally agreed well with GBD estimates as the median under-5 ratio was 1.01 and neonatal was 1.00 with 95% range of 0.81 and 1.22 and 0.76 and 1.19 respectively. There were some extreme outliers with Botswana in 2015 on the low end (0.33 for child and 0.32 for neonatal), and Madagascar 2015 (1.48 for child) and Central African Republic 2010 (1.40 for neonatal) at the high end.

Supplementary Figure 18. Comparison of nationally aggregated population-weighted geospatial estimates, and GBD estimates for the same years. A) Under-5 mortality estimates. B) Neonatal mortality estimates. Red lines indicate unity. The median ratio between GBD and this study's estimates for under-5 mortality was 1.01 [inter-quartile range (IQR): 0.95, 1.14] and for neonatal mortality 1.00 [IQR 0.93, 1.05]. Red lines indicate equivalence.

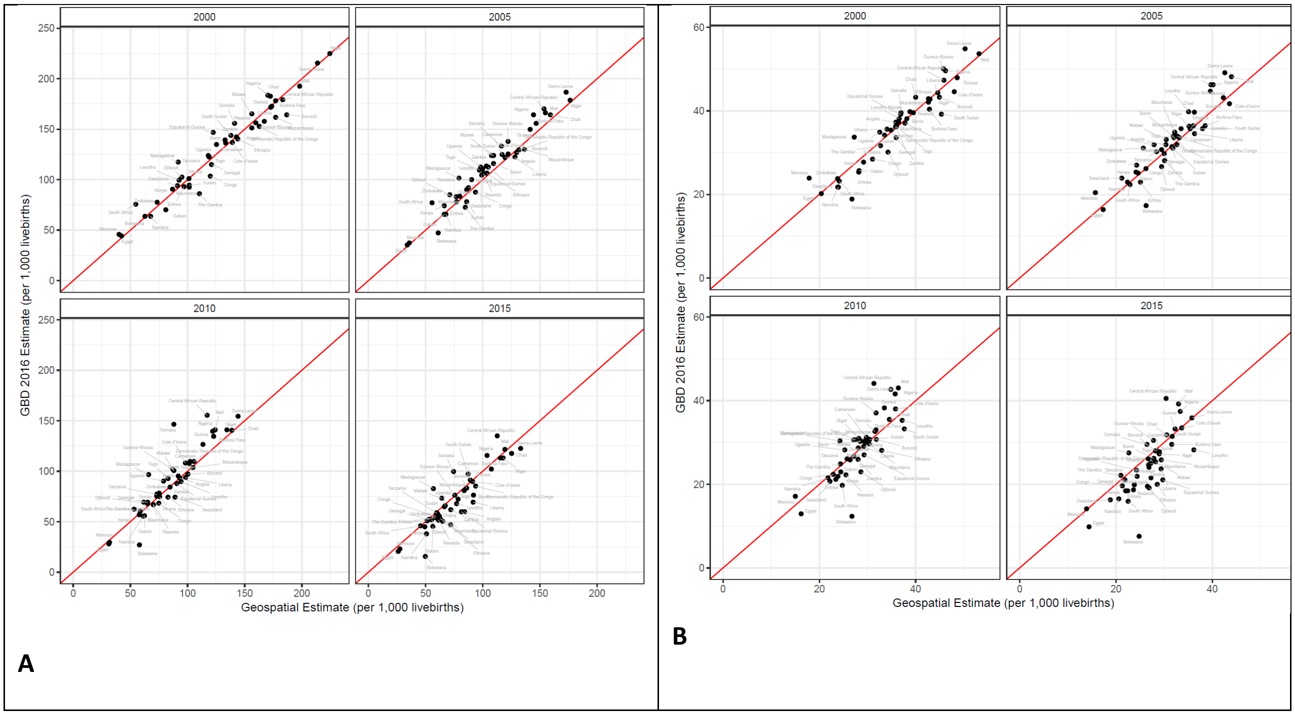

13.Verification and comparison against other subnational child mortality models

13.1 Gavi Full Country Evaluation small area estimates

As part of the Gavi Full Country Evaluation (Gavi FCE),[18] evaluation teams produced under-5 mortality estimates for Mozambique, Zambia, Uganda, Chad, and Cameroon. Supplementary Figure 18 (A, B, C, D, and E) shows agreement between aggregated population-weighted estimates from this study with the district level small area estimates of that one.

Supplementary Figure 19 Comparison of district- and county-level aggregated population-weighted geospatial estimates, and Gavi-FCE estimates of under-5 mortality for the same years. A) Mozambique. B) Zambia, C) Uganda, D) Chad, and E) Cameroon. Red lines indicate equivalence.

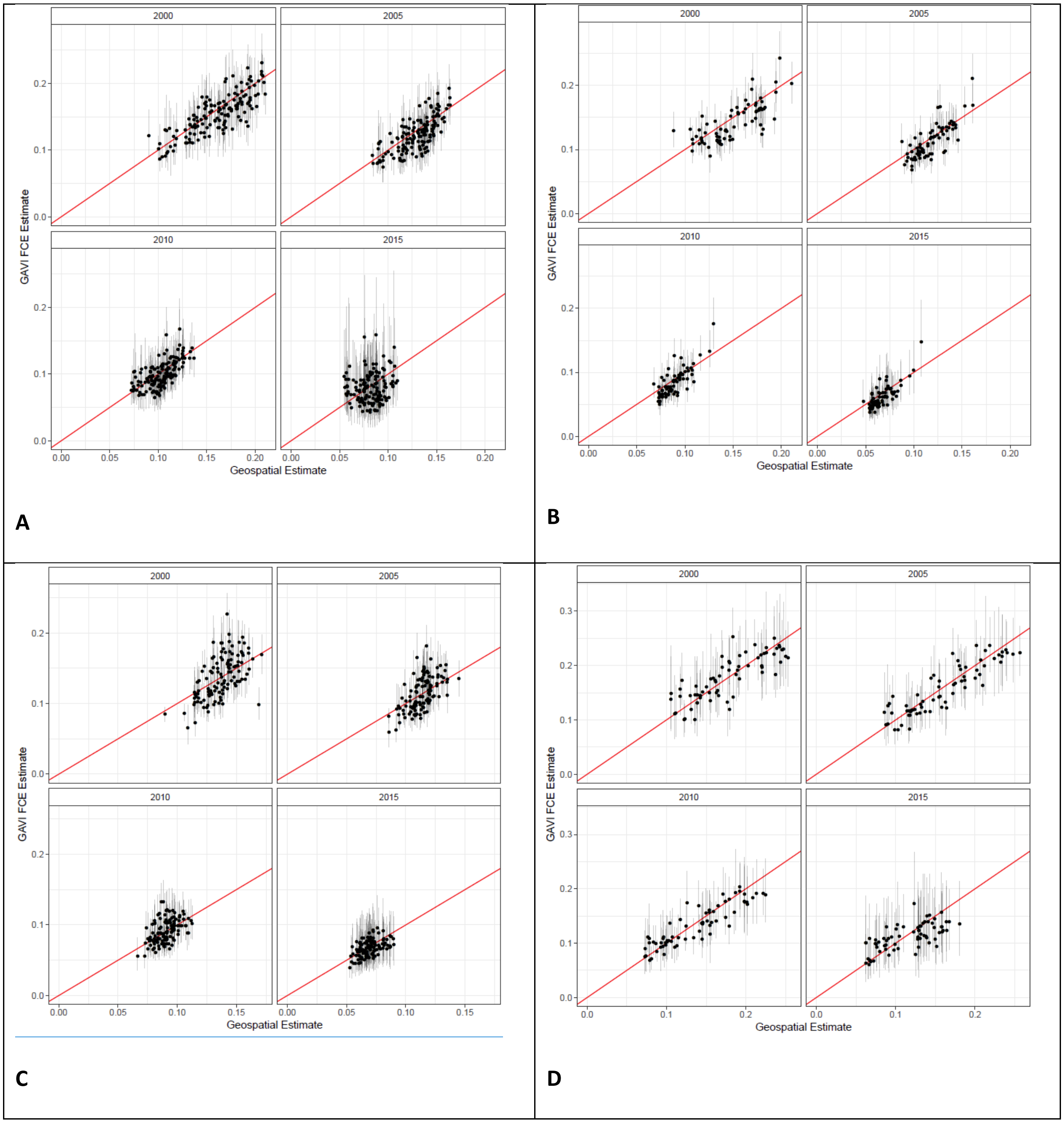

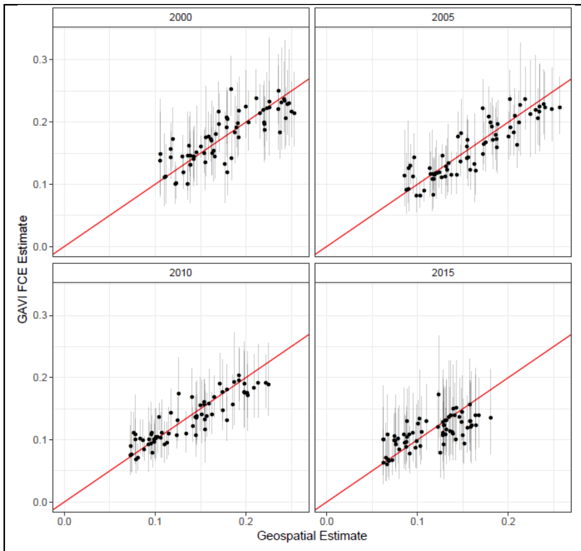

E

13.2 GBD 2015 subnational estimates in Kenya and South Africa

GBD 2016 included subnational estimates for Kenyan counties and South African regions. Comparisons are shown in Supplementary Figure 19 (A and B) below.

Supplementary Figure 20. Comparison of subnational aggregated population-weighted geospatial estimates, and GBD estimates of under-5 mortality for the same years. A) Kenyan Counties. B) South African Regions. Red lines indicate equivalence.

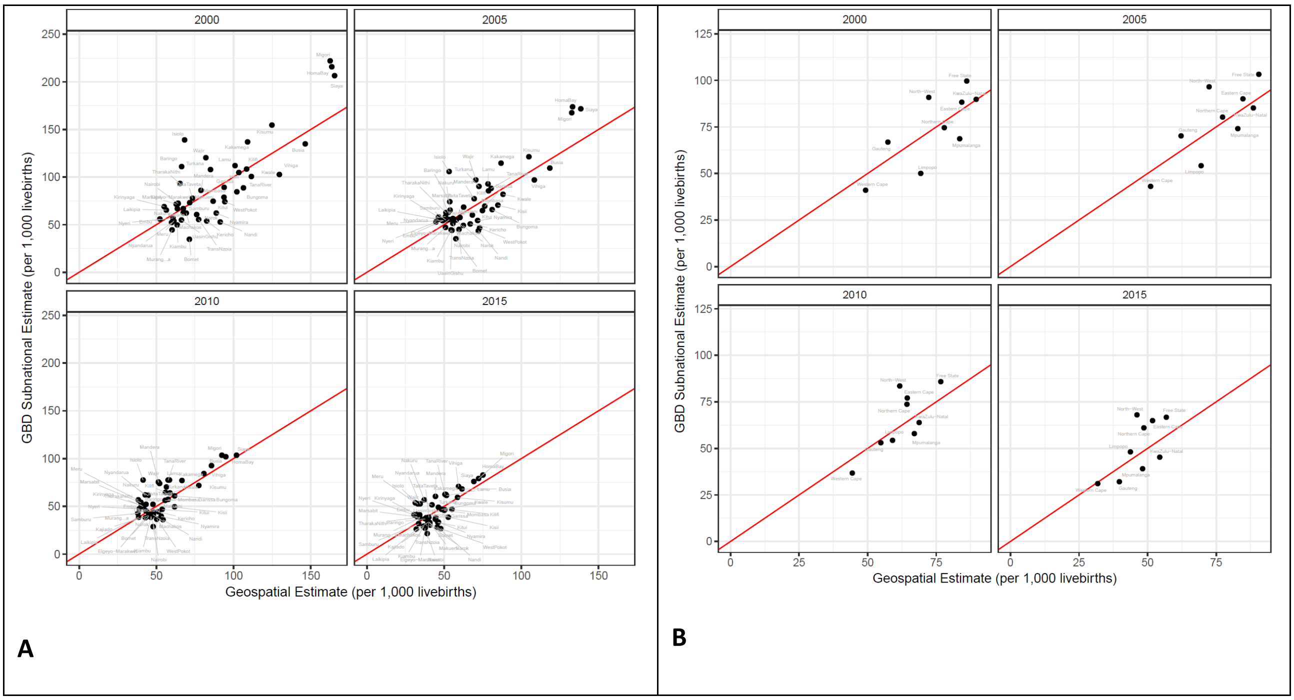

A

B

14. Source code

Source code is available through http://ghdx.healthdata.org/

References

1 Maternal, Newborn & Child Health Data - Countdown to 2030. http://countdown2030.org/ (accessed Feb 5, 2017).

2 Central Statistical Agency (Ethiopia), Government of Ethiopia, United Nations Population Fund (UNFPA), United Nations Development Programme (UNDP). Ethiopia Population and Housing Census 2007. Addis Ababa, Ethiopia: Central Statistical Agency (Ethiopia).

3 GBD 2016 Mortality Collaborators. Global, regional, and national under-5 mortality, adult mortality, age-specific mortality, and life expectancy, 1970-2016: a systematic analysis for the Global Burden of Disease Study 2016. *The Lancet* Under review.

4 Manual X. http://www.un.org/esa/population/publications/Manual_X/Manual_X.htm (accessed July 20, 2016).

5 Trussell TJ. A re-estimation of the multiplying factors for the Brass technique for determining childhood survivorship rates. *Popul Stud* 1975; **29**: 97–107.

6 Methods for estimating fertility and mortality from limited and defective data; 1975. http://unesdoc.unesco.org/Ulis/cgi-bin/ulis.pl?catno=19743&gp=0&lin=1 (accessed Feb 5, 2017).

7 Rajaratnam JK, Tran LN, Lopez AD, Murray CJL. Measuring Under-Five Mortality: Validation of New Low-Cost Methods. *PLOS Med* 2010; **7**: e1000253.

8 Lloyd CT, Sorichetta A, Tatem AJ. High resolution global gridded data for use in population studies. *Sci Data* 2017; **4**: 170001.

9 Wolpert DH. Stacked generalization. *Neural Netw* 1992; **5**: 241–59.

10 Breiman L. Stacked regressions. *Mach Learn* 1996; **24**: 49–64.

11 Bhatt S, Cameron E, Flaxman SR, Weiss DJ, Smith DL, Gething PW. Improved prediction accuracy for disease risk mapping using Gaussian Process stacked generalisation. *ArXiv161203278 Stat* 2016; published online Dec 10. http://arxiv.org/abs/1612.03278 (accessed Feb 5, 2017).

12 R: The R Project for Statistical Computing. https://www.r-project.org/ (accessed Feb 5, 2017).

13 Rue H, Martino S, Chopin N. Approximate Bayesian inference for latent Gaussian models by using integrated nested Laplace approximations. *J R Stat Soc Ser B Stat Methodol* 2009; **71**: 319–92.

14 Lindgren F, Rue H, Lindström J. An explicit link between Gaussian fields and Gaussian Markov random fields: the stochastic partial differential equation approach. *J R Stat Soc Ser B Stat Methodol* 2011; **73**: 423–98.

15 The R-INLA project. http://www.r-inla.org/ (accessed July 27, 2016).

16 Schrödle B, Held L, Riebler A, Danuser J. Using integrated nested Laplace approximations for the evaluation of veterinary surveillance data from Switzerland: a case-study. *J R Stat Soc Ser C Appl Stat* 2011; **60**: 261–79.

17 Blangiardo M, Cameletti M. Spatial and Spatio-temporal Bayesian Models with R - INLA. John Wiley & Sons, 2015.

18 Gavi Full Country Evaluations Team. Gavi Full Country Evaluations: 2015 Dissemination Report. Seattle, WA: IHME, 2015.

Results appendix to Mapping under-5 and neonatal mortality in Africa, 2000–2015: a baseline analysis for the Sustainable Development Goals

This appendix provides additional and more detailed results for “Mapping under-5 and neonatal mortality in Africa, 2000–2015: a baseline analysis for the Sustainable Development Goals”.

Figures and Tables

Supplementary Figure 1. Neonatal mortality rates at the 5x5 km resolution in 2000, 2005, 2010, and 2015.

Supplementary results Figure 2. Neonatal mortality rates at the national, administrative 1, administrative 2, and 5x5 km levels in 2015.

Supplementary results Figure 3. Overlapping population-weighted quartiles of neonatal mortality and relative uncertainty in 2015.

Supplementary results Figure 4. Annualised rates of decline for neonatal mortality from 2000 to 2015 (A); predicted neonatal mortality rates in 2030 based on annualised rates of decline achieved between 2000 and 2015 (B); and rates of decline required to reach the SDG3·2 target for neonatal mortality by 2030 (C).

Supplementary results Table 1. Under-5 mortality estimates for subnational administrative level 1 divisions in Africa, 2000, 2005, 2010, and 2015.

Supplementary results Table 2. Under-5 mortality estimates for subnational administrative levels 1 and 2 divisions in Africa, 2000, 2005, 2010, and 2015.

Supplementary results Table 3. Neonatal mortality estimates for subnational administrative level 1 divisions in Africa, 2000, 2005, 2010, and 2015.

Supplementary results Table 4. Neonatal mortality estimates for subnational administrative levels 1 and 2 in Africa, 2000, 2005, 2010, and 2015.

Supplementary Figure 1. **Neonatal mortality rates at the 5x5 km resolution in 2000, 2005, 2010, and 2015.** All pixels with a neonatal mortality rate equal to or fewer than 12 deaths per 1,000 livebirths, the SDG3·2 target for neonatal mortality, are coloured purple. Pixels with fewer than ten people and classified as "barren or sparsely vegetated" are coloured in gray. Gray areas with diagonal lines are not included in this analysis. SDG = Sustainable Development Goal. km=kilometre.

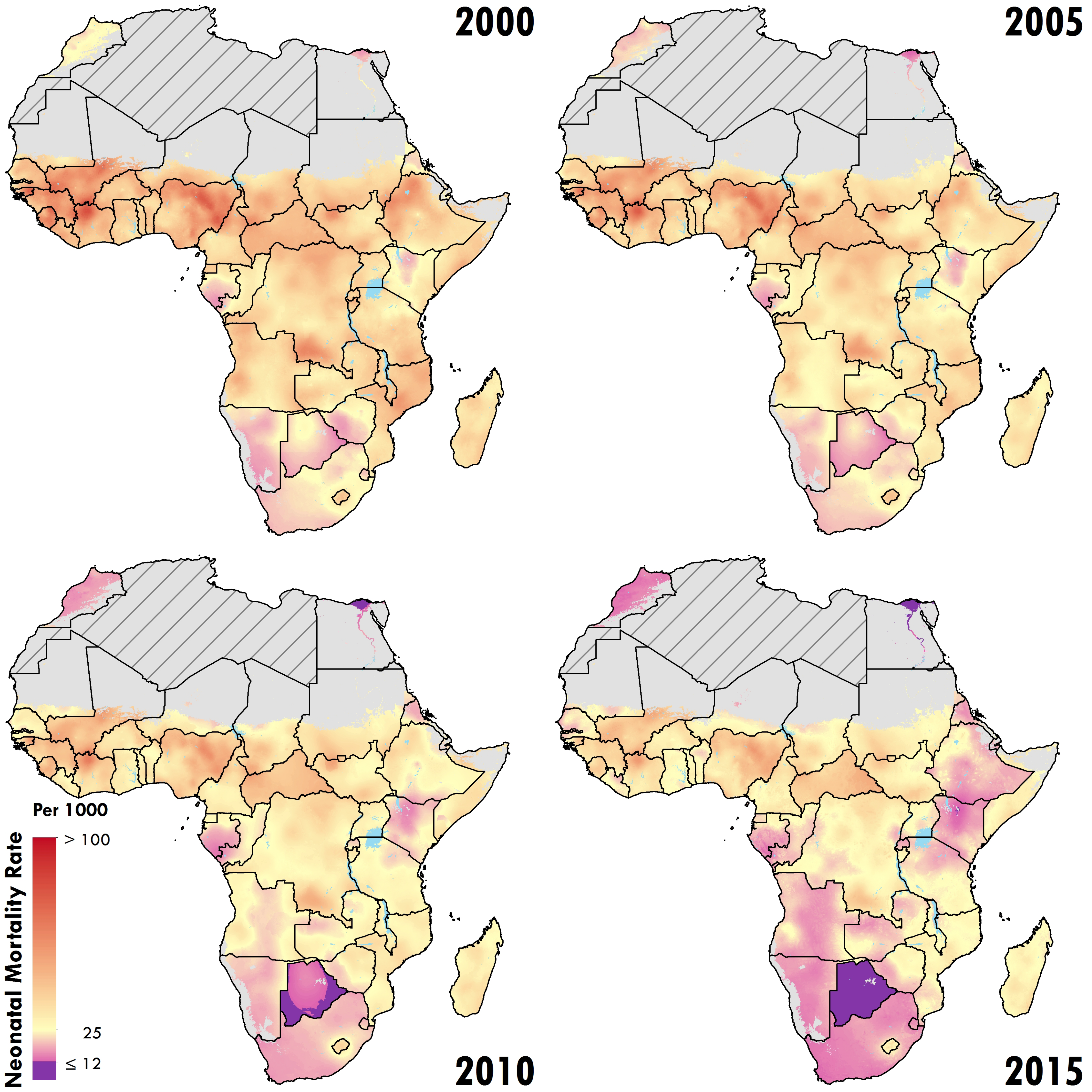

Supplementary results Figure 2. **Neonatal mortality rates at the national, administrative 1, administrative 2, and 5x5 km levels in 2015.** All locations with rate equal to or fewer than 12 deaths per 1,000 livebirths, the SDG3·2 target for neonatal mortality, are coloured purple. Pixels with fewer than ten people and classified as “barren or sparsely vegetated” are coloured in gray. Gray areas with diagonal lines are not included in this analysis. SDG = Sustainable Development Goal. km=kilometre.

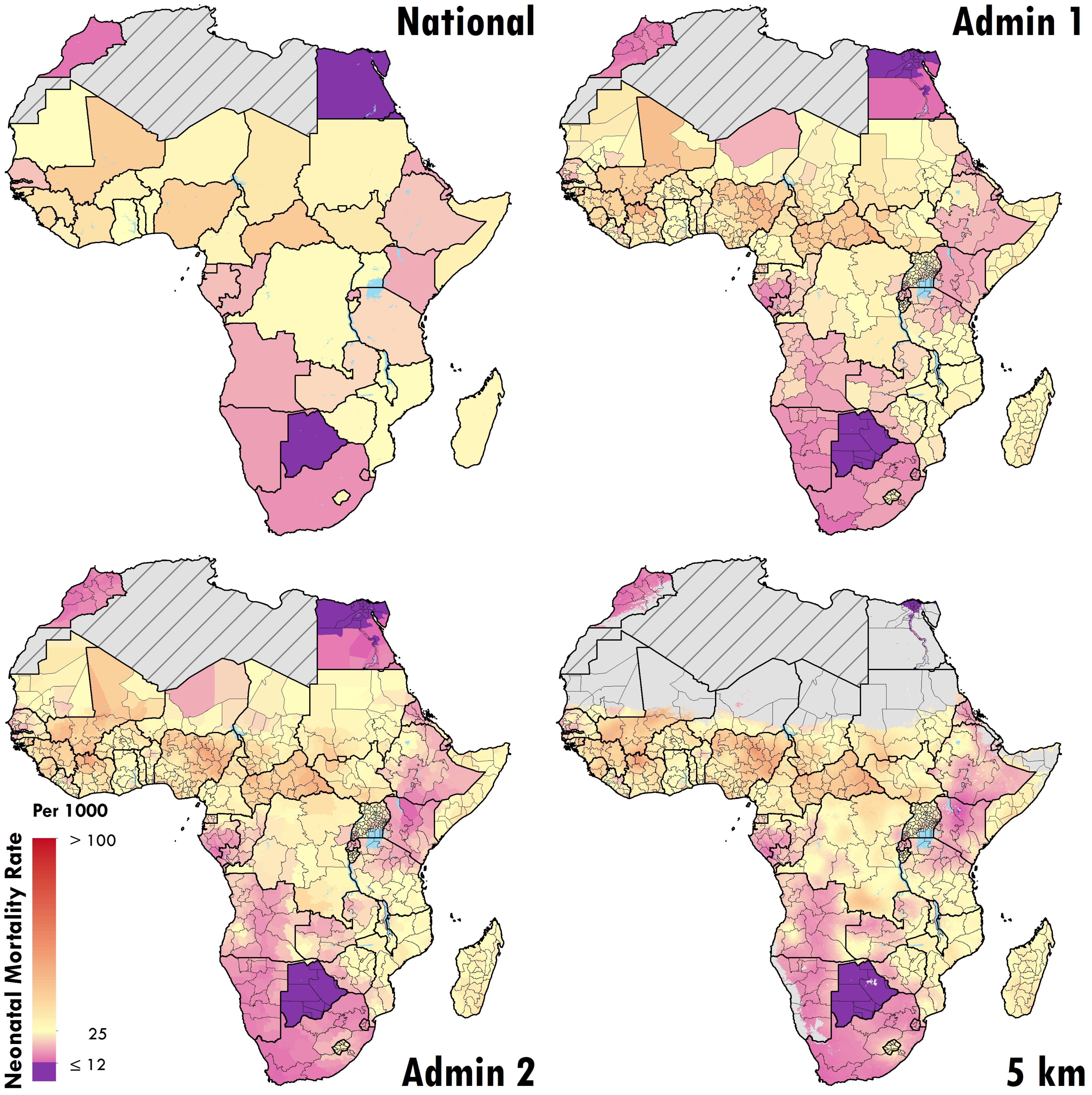

***Supplementary results Figure 3.* Overlapping population-weighted quartiles of neonatal mortality and relative uncertainty in 2015.** Neonatal mortality rate quartile cut-points were 20, 26, and 31 deaths per 1,000 livebirths. Relative uncertainty was computed as the ratio of the 95% UIs and under-5 mortality rate for each pixel. Cut-points for uncertainty were 44%, 52%, and 62%. The lowest quartile of mortality is white, and the highest is dark pink. The lowest quartile for uncertainty is white and the highest is blue. These colours overlap such that areas coloured purple have both high neonatal rates and high relative uncertainty. Pixels with fewer than ten people and classified as "barren or sparsely vegetated" are coloured in gray. Gray areas with diagonal lines are not included in this analysis. UIs = uncertainty intervals.

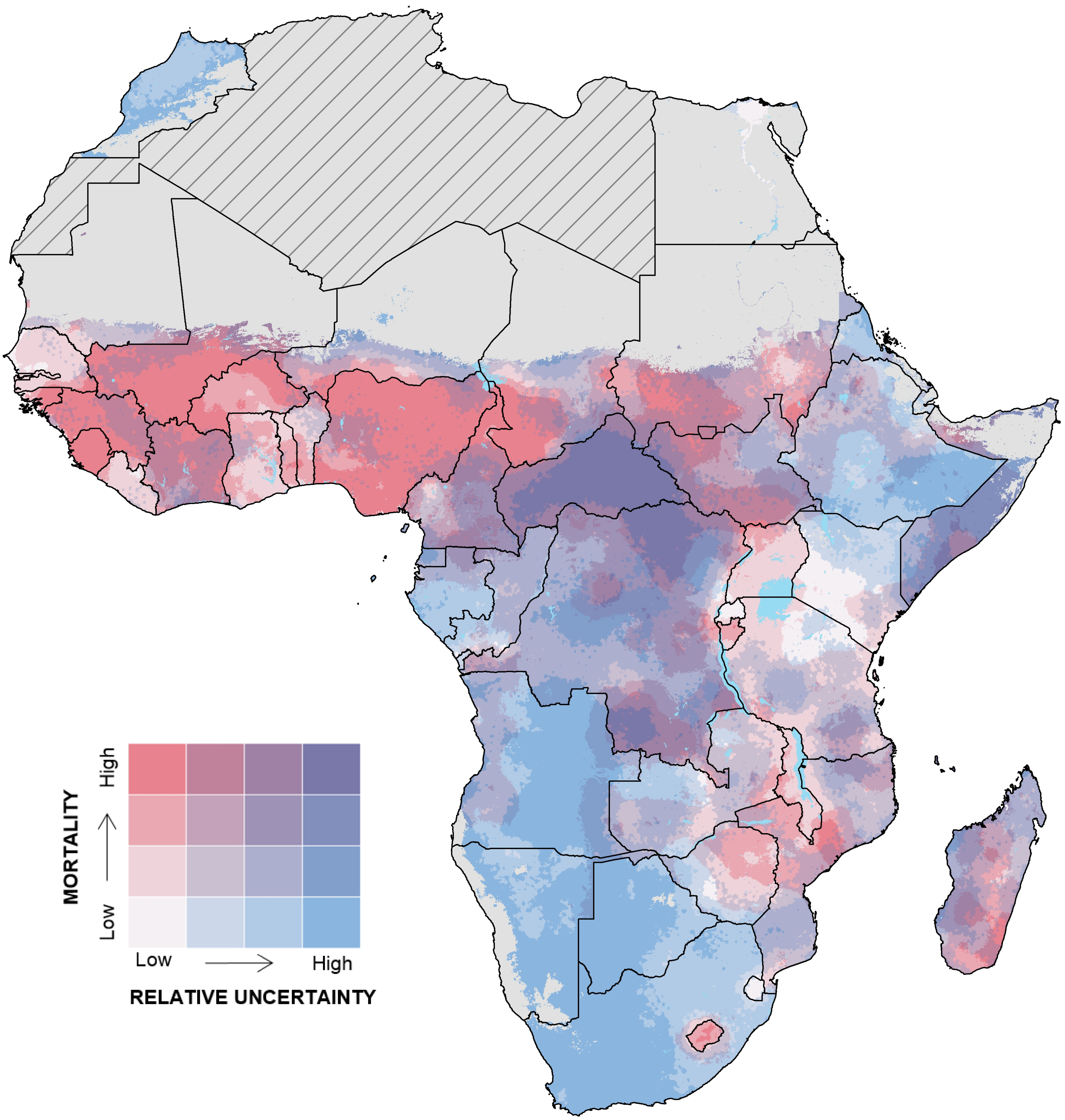

Supplementary results Figure 4. **Annualised rates of decline for neonatal mortality from 2000 to 2015 (A); predicted neonatal mortality rates in 2030 based on annualised rates of decline achieved between 2000 and 2015 (B); and rates of decline required to reach the SDG3·2 target for neonatal mortality by 2030 (C).** 4·4% is the annualised rate of decline that was equivalent to the pace of progress required to meet MDG4. In (A), pixels coloured blue exceeded this pace from 2000 to 2015, while pixels coloured green to yellow had a slower rate of annualised decline during this time. In (B), pixel-level neonatal mortality rates were predicted for 2030 on the basis of annualised rates of decline achieved from 2000 to 2015. Based on this prediction, pixels for which neonatal mortality rates equaled or were less than 12 deaths per 1,000 livebirths in 2030 are coloured purple. In (C), pixels coloured blue will need to achieve a 4·4% or greater decline per year from 2015 to 2030 to achieve the SDG3·2 target for neonatal mortality (12 deaths per 1,000 livebirths). Pixels coloured green to yellow can meet the SDG3·2 target by 2030 at a pace slower than a 4·4% reduction per year from 2015 to 2030. Pixels with fewer than ten people and classified as “barren or sparsely vegetated” are coloured in gray. Gray areas with diagonal lines are not included in this analysis. MDG = Millennium Development Goal. SDG = Sustainable Development Goal.

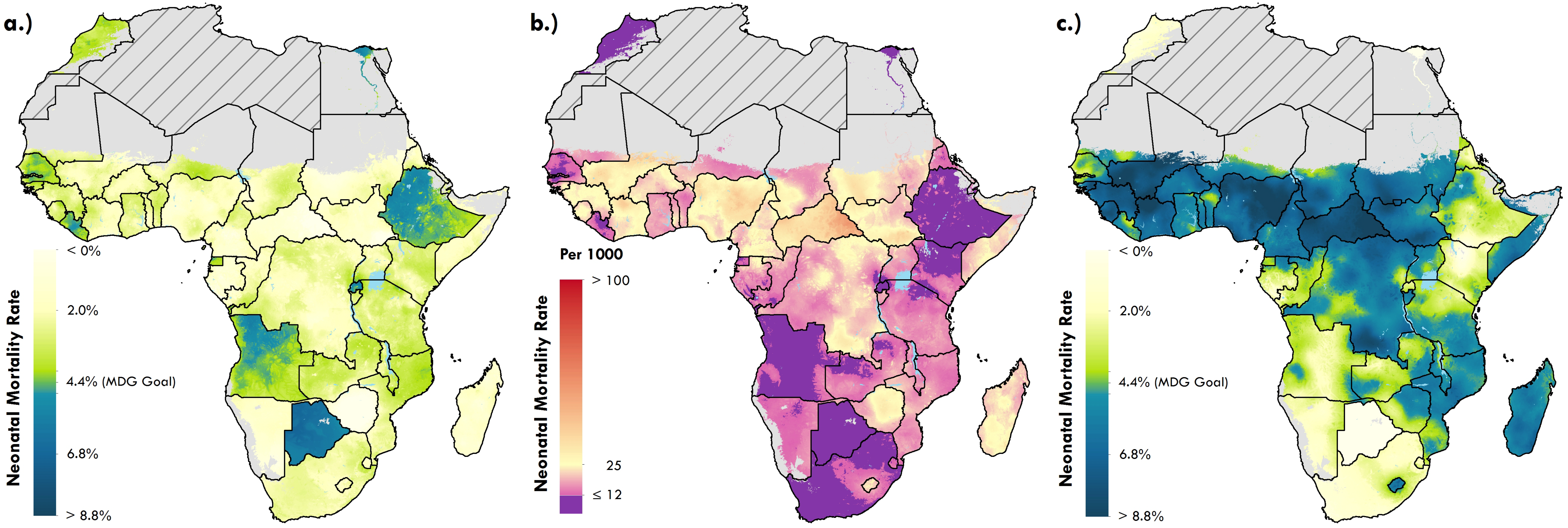

Supplementary results Table 1. **Under-5 mortality estimates for subnational administrative level 1 divisions in Africa, 2000, 2005, 2010, and 2015.** UI = uncertainty interval						
**Admin 0**	**Admin 1**	**GAUL Code**	**Year**	**Under-5 mortality (per 1,000 livebirths)**		
				**Estimate**	**Lower bound, 95% UI**	**Upper bound, 95% UI**
Angola	Bengo	398	2000	161.9	143.5	183.1
Angola	Bengo	398	2005	127.3	112.8	143.9
Angola	Bengo	398	2010	96.6	84.7	110.1
Angola	Bengo	398	2015	64.2	55.3	74.1
Angola	Benguela	399	2000	161.0	141.0	181.0
Angola	Benguela	399	2005	136.4	120.5	153.6
Angola	Benguela	399	2010	104.4	91.4	118.5
Angola	Benguela	399	2015	69.1	59.2	79.1
Angola	Bie	400	2000	144.3	125.2	165.5
Angola	Bie	400	2005	119.3	103.5	137.7
Angola	Bie	400	2010	87.4	75.0	100.2
Angola	Bie	400	2015	59.4	50.3	69.3
Angola	Cabinda	401	2000	154.2	136.5	174.1
Angola	Cabinda	401	2005	112.4	99.9	128.0
Angola	Cabinda	401	2010	89.3	78.7	101.5
Angola	Cabinda	401	2015	58.3	50.3	67.1
Angola	Cuando Cubango	402	2000	144.4	127.4	162.7
Angola	Cuando Cubango	402	2005	124.0	109.8	140.0
Angola	Cuando Cubango	402	2010	94.4	83.5	107.6
Angola	Cuando Cubango	402	2015	60.1	52.4	69.0
Angola	Cuanza Sul	403	2000	206.1	181.7	231.0
Angola	Cuanza Sul	403	2005	169.1	149.0	189.6
Angola	Cuanza Sul	403	2010	116.1	100.8	132.3
Angola	Cuanza Sul	403	2015	82.8	71.3	95.0
Angola	Cunene	404	2000	123.5	109.4	139.4
Angola	Cunene	404	2005	118.9	105.9	134.3
Angola	Cunene	404	2010	91.5	81.1	104.3
Angola	Cunene	404	2015	55.5	48.6	64.0
Angola	Huambo	405	2000	198.6	176.9	224.7
Angola	Huambo	405	2005	158.0	142.3	177.3
Angola	Huambo	405	2010	100.0	87.8	113.0
Angola	Huambo	405	2015	73.4	63.6	84.4
Angola	Huila	406	2000	188.9	172.0	206.9
Angola	Huila	406	2005	158.6	145.9	172.5
Angola	Huila	406	2010	120.4	108.9	132.6
Angola	Huila	406	2015	79.6	71.0	89.0
Angola	Kuanza Norte	407	2000	186.3	163.2	213.0
Angola	Kuanza Norte	407	2005	144.0	124.7	164.3
Angola	Kuanza Norte	407	2010	102.6	88.3	117.9
Angola	Kuanza Norte	407	2015	73.7	62.4	86.0
Angola	Luanda	408	2000	101.8	91.2	113.1
Angola	Luanda	408	2005	85.7	77.7	94.8
Angola	Luanda	408	2010	74.1	65.8	83.9
Angola	Luanda	408	2015	48.6	42.0	56.4
Angola	Lunda Norte	409	2000	121.6	106.3	139.7
Angola	Lunda Norte	409	2005	103.8	91.2	118.0
Angola	Lunda Norte	409	2010	73.8	64.7	84.1
Angola	Lunda Norte	409	2015	52.6	45.5	60.4
Angola	Lunda Sul	410	2000	107.2	92.6	124.9
Angola	Lunda Sul	410	2005	92.2	79.4	106.8
Angola	Lunda Sul	410	2010	70.0	59.9	81.6
Angola	Lunda Sul	410	2015	51.1	43.2	60.1
Angola	Malanje	411	2000	124.3	109.3	139.1
Angola	Malanje	411	2005	103.8	91.2	116.9
Angola	Malanje	411	2010	76.7	67.1	87.0
Angola	Malanje	411	2015	53.3	45.8	61.4
Angola	Moxico	412	2000	121.7	103.2	141.3
Angola	Moxico	412	2005	97.7	83.8	113.3
Angola	Moxico	412	2010	76.4	64.8	89.0
Angola	Moxico	412	2015	55.3	46.4	65.3
Angola	Namibe	413	2000	133.0	115.7	152.2
Angola	Namibe	413	2005	111.5	98.4	127.1
Angola	Namibe	413	2010	91.5	80.1	104.7
Angola	Namibe	413	2015	58.6	50.5	68.1
Angola	Uige	414	2000	168.3	150.4	188.2
Angola	Uige	414	2005	123.5	110.7	137.7
Angola	Uige	414	2010	87.8	77.2	98.7

Admin 0	Admin 1	GAUL Code	Year	Under-5 mortality (per 1,000 livebirths)		
				Estimate	Lower bound, 95% UI	Upper bound, 95% UI
Angola	Uige	414	2015	61.1	52.8	69.9
Angola	Zaire	415	2000	135.1	118.6	153.7
Angola	Zaire	415	2005	108.1	94.4	123.0
Angola	Zaire	415	2010	86.7	74.9	98.8
Angola	Zaire	415	2015	53.2	45.3	61.2
Benin	Alibori	609	2000	168.9	159.2	179.5
Benin	Alibori	609	2005	142.2	133.7	151.2
Benin	Alibori	609	2010	118.7	110.2	128.4
Benin	Alibori	609	2015	91.1	82.1	101.8
Benin	Atakora	610	2000	153.2	144.7	162.4
Benin	Atakora	610	2005	125.3	118.2	132.7
Benin	Atakora	610	2010	106.5	98.8	114.3
Benin	Atakora	610	2015	84.7	76.7	93.5
Benin	Atlantique	611	2000	121.4	115.2	127.1
Benin	Atlantique	611	2005	101.7	96.4	106.9
Benin	Atlantique	611	2010	84.4	79.3	90.1
Benin	Atlantique	611	2015	70.1	62.9	78.1
Benin	Borgou	612	2000	126.1	117.8	133.7
Benin	Borgou	612	2005	100.2	93.9	106.8
Benin	Borgou	612	2010	84.1	77.5	91.1
Benin	Borgou	612	2015	68.0	60.7	75.8
Benin	Collines	613	2000	146.7	137.9	156.0
Benin	Collines	613	2005	123.2	115.3	131.5
Benin	Collines	613	2010	100.1	92.3	108.0
Benin	Collines	613	2015	82.4	74.0	91.8
Benin	Couffo	614	2000	144.1	137.1	151.7
Benin	Couffo	614	2005	116.5	110.4	123.1
Benin	Couffo	614	2010	94.0	87.9	100.4
Benin	Couffo	614	2015	85.6	76.7	94.8
Benin	Donga	615	2000	149.5	139.4	159.4
Benin	Donga	615	2005	127.2	119.0	136.0
Benin	Donga	615	2010	107.1	99.0	116.1
Benin	Donga	615	2015	85.0	75.9	94.6
Benin	Littoral	616	2000	84.4	78.9	90.5
Benin	Littoral	616	2005	76.6	71.5	81.9
Benin	Littoral	616	2010	67.9	62.9	73.8
Benin	Littoral	616	2015	50.9	45.1	57.5
Benin	Mono	617	2000	116.7	110.1	123.5
Benin	Mono	617	2005	95.7	89.9	101.5
Benin	Mono	617	2010	77.8	72.4	83.8
Benin	Mono	617	2015	68.8	61.4	76.6
Benin	Oueme	618	2000	130.6	123.5	137.4
Benin	Oueme	618	2005	112.0	106.1	118.1
Benin	Oueme	618	2010	89.9	84.1	96.4
Benin	Oueme	618	2015	70.5	63.3	78.5
Benin	Plateau	619	2000	147.4	139.8	155.6
Benin	Plateau	619	2005	121.5	114.5	128.5
Benin	Plateau	619	2010	95.1	88.3	101.9
Benin	Plateau	619	2015	78.8	71.1	87.4
Benin	Zou	620	2000	151.7	144.0	159.3
Benin	Zou	620	2005	125.6	119.0	132.6
Benin	Zou	620	2010	101.6	94.6	108.6
Benin	Zou	620	2015	87.9	78.2	98.5
Botswana	Central	654	2000	64.3	58.4	70.3
Botswana	Central	654	2005	48.6	44.2	53.5
Botswana	Central	654	2010	27.5	24.9	30.4
Botswana	Central	654	2015	16.5	14.5	18.7
Botswana	Chobe	655	2000	78.5	68.3	90.8
Botswana	Chobe	655	2005	58.5	50.9	67.7
Botswana	Chobe	655	2010	32.6	27.9	37.9
Botswana	Chobe	655	2015	19.0	15.9	22.5
Botswana	Ghanzi	656	2000	76.8	65.3	89.9
Botswana	Ghanzi	656	2005	54.1	45.6	62.7
Botswana	Ghanzi	656	2010	28.9	24.3	34.0
Botswana	Ghanzi	656	2015	18.1	15.1	21.4
Botswana	Kgalagadi	657	2000	71.2	60.7	82.3
Botswana	Kgalagadi	657	2005	52.2	44.6	60.6
Botswana	Kgalagadi	657	2010	28.5	24.3	33.6
Botswana	Kgalagadi	657	2015	18.0	15.2	21.2

Admin 0	Admin 1	GAUL Code	Year	Under-5 mortality (per 1,000 livebirths)		
				Estimate	Lower bound, 95% UI	Upper bound, 95% UI
Botswana	Kgatleng	658	2000	55.0	48.4	62.7
Botswana	Kgatleng	658	2005	41.3	36.0	47.8
Botswana	Kgatleng	658	2010	24.3	20.8	28.2
Botswana	Kgatleng	658	2015	14.8	12.2	17.5
Botswana	Kweneng	659	2000	62.1	54.1	70.6
Botswana	Kweneng	659	2005	45.5	39.8	52.0
Botswana	Kweneng	659	2010	25.8	22.2	29.9
Botswana	Kweneng	659	2015	16.0	13.5	18.8
Botswana	Ngamiland	660	2000	81.9	70.9	93.2
Botswana	Ngamiland	660	2005	58.8	50.4	67.7
Botswana	Ngamiland	660	2010	32.4	27.7	37.7
Botswana	Ngamiland	660	2015	19.5	16.5	22.9
Botswana	North East	661	2000	56.7	49.9	64.2
Botswana	North East	661	2005	44.7	39.3	50.7
Botswana	North East	661	2010	25.0	21.7	28.7
Botswana	North East	661	2015	15.3	12.9	17.9
Botswana	South-East	662	2000	48.8	43.1	54.7
Botswana	South-East	662	2005	39.3	34.6	44.7
Botswana	South-East	662	2010	23.2	20.0	26.5
Botswana	South-East	662	2015	14.5	12.0	17.1
Botswana	Southern	663	2000	60.9	54.1	68.3
Botswana	Southern	663	2005	44.6	39.6	50.5
Botswana	Southern	663	2010	25.4	22.0	29.1
Botswana	Southern	663	2015	15.9	13.6	18.5
Burkina Faso	Boucle Du Mouhoun	40276	2000	172.4	165.1	180.3
Burkina Faso	Boucle Du Mouhoun	40276	2005	147.5	141.5	154.0
Burkina Faso	Boucle Du Mouhoun	40276	2010	123.3	116.1	131.2
Burkina Faso	Boucle Du Mouhoun	40276	2015	110.8	102.3	120.5
Burkina Faso	Cascades	40277	2000	205.6	195.0	215.8
Burkina Faso	Cascades	40277	2005	188.2	178.3	198.1
Burkina Faso	Cascades	40277	2010	157.3	147.0	168.1
Burkina Faso	Cascades	40277	2015	136.4	123.8	149.6
Burkina Faso	Centre	40278	2000	117.0	110.4	124.2
Burkina Faso	Centre	40278	2005	100.6	94.8	106.9
Burkina Faso	Centre	40278	2010	90.8	84.0	98.1
Burkina Faso	Centre	40278	2015	70.1	63.0	78.2
Burkina Faso	Centre-est	40279	2000	160.4	153.2	167.7
Burkina Faso	Centre-est	40279	2005	138.6	132.3	145.6
Burkina Faso	Centre-est	40279	2010	118.1	111.3	125.3
Burkina Faso	Centre-est	40279	2015	95.7	87.8	104.6
Burkina Faso	Centre-nord	40280	2000	180.2	173.0	188.3
Burkina Faso	Centre-nord	40280	2005	158.3	151.2	165.4
Burkina Faso	Centre-nord	40280	2010	137.5	129.2	145.9
Burkina Faso	Centre-nord	40280	2015	124.0	113.1	135.7
Burkina Faso	Centre-ouest	40281	2000	171.0	164.2	178.2
Burkina Faso	Centre-ouest	40281	2005	148.7	142.1	155.2
Burkina Faso	Centre-ouest	40281	2010	125.7	117.8	134.2
Burkina Faso	Centre-ouest	40281	2015	108.7	99.4	118.9
Burkina Faso	Centre-sud	40282	2000	165.3	158.1	174.1
Burkina Faso	Centre-sud	40282	2005	138.1	131.3	144.9
Burkina Faso	Centre-sud	40282	2010	120.7	113.0	129.0
Burkina Faso	Centre-sud	40282	2015	94.5	86.5	104.7
Burkina Faso	Est	40283	2000	217.2	207.2	227.1
Burkina Faso	Est	40283	2005	189.5	181.2	198.2
Burkina Faso	Est	40283	2010	162.7	154.1	172.6
Burkina Faso	Est	40283	2015	138.1	127.1	150.1
Burkina Faso	Hauts-bassins	40284	2000	173.2	164.9	181.4
Burkina Faso	Hauts-bassins	40284	2005	159.1	151.4	167.1
Burkina Faso	Hauts-bassins	40284	2010	129.6	121.3	138.4
Burkina Faso	Hauts-bassins	40284	2015	115.6	105.8	126.5
Burkina Faso	Nord	40285	2000	185.7	177.3	193.5
Burkina Faso	Nord	40285	2005	159.0	151.5	166.6
Burkina Faso	Nord	40285	2010	135.6	126.8	144.1
Burkina Faso	Nord	40285	2015	121.1	110.6	132.3
Burkina Faso	Plateau Central	40286	2000	165.9	159.2	172.5
Burkina Faso	Plateau Central	40286	2005	144.3	138.2	150.3
Burkina Faso	Plateau Central	40286	2010	127.7	120.7	134.8
Burkina Faso	Plateau Central	40286	2015	109.3	100.6	119.0
Burkina Faso	Sahel	40287	2000	226.5	216.3	236.3

Admin 0	Admin 1	GAUL Code	Year	Under-5 mortality (per 1,000 livebirths)		
				Estimate	Lower bound, 95% UI	Upper bound, 95% UI
Burkina Faso	Sahel	40287	2005	200.0	190.9	209.2
Burkina Faso	Sahel	40287	2010	181.8	170.4	193.2
Burkina Faso	Sahel	40287	2015	154.8	141.3	169.1
Burkina Faso	Sud-ouest	40288	2000	218.1	208.1	228.5
Burkina Faso	Sud-ouest	40288	2005	192.8	184.1	202.6
Burkina Faso	Sud-ouest	40288	2010	164.9	154.3	175.7
Burkina Faso	Sud-ouest	40288	2015	138.6	126.3	151.4
Burundi	Bubanza	40542	2000	173.9	165.3	183.1
Burundi	Bubanza	40542	2005	129.5	122.6	137.1
Burundi	Bubanza	40542	2010	99.6	93.8	105.6
Burundi	Bubanza	40542	2015	86.1	78.6	94.3
Burundi	Bujumbura Mairie	40543	2000	116.7	110.2	124.0
Burundi	Bujumbura Mairie	40543	2005	103.0	96.8	109.7
Burundi	Bujumbura Mairie	40543	2010	81.9	76.1	87.5
Burundi	Bujumbura Mairie	40543	2015	70.8	64.1	78.1
Burundi	Bujumbura Rural	40544	2000	138.4	131.5	146.0
Burundi	Bujumbura Rural	40544	2005	112.1	106.2	118.7
Burundi	Bujumbura Rural	40544	2010	87.2	81.9	92.8
Burundi	Bujumbura Rural	40544	2015	71.4	65.0	78.5
Burundi	Bururi	40545	2000	124.9	116.6	133.3
Burundi	Bururi	40545	2005	104.2	97.6	111.1
Burundi	Bururi	40545	2010	82.2	76.2	88.3
Burundi	Bururi	40545	2015	66.6	60.2	73.3
Burundi	Cankuzo	40546	2000	145.2	134.8	155.8
Burundi	Cankuzo	40546	2005	120.2	112.2	128.5
Burundi	Cankuzo	40546	2010	96.3	88.7	103.7
Burundi	Cankuzo	40546	2015	84.1	76.2	92.1
Burundi	Cibitoke	40547	2000	190.6	179.8	202.0
Burundi	Cibitoke	40547	2005	134.8	126.9	143.0
Burundi	Cibitoke	40547	2010	103.0	96.3	110.2
Burundi	Cibitoke	40547	2015	93.2	84.2	102.2
Burundi	Gitega	40548	2000	138.2	130.6	146.3
Burundi	Gitega	40548	2005	111.2	105.3	117.4
Burundi	Gitega	40548	2010	89.1	83.8	94.5
Burundi	Gitega	40548	2015	75.3	68.8	82.2
Burundi	Karuzi	40549	2000	170.5	161.1	180.4
Burundi	Karuzi	40549	2005	135.8	128.9	143.4
Burundi	Karuzi	40549	2010	106.3	99.9	113.3
Burundi	Karuzi	40549	2015	94.0	85.7	102.7
Burundi	Kayanza	40550	2000	170.0	162.8	177.8
Burundi	Kayanza	40550	2005	128.4	123.1	134.3
Burundi	Kayanza	40550	2010	95.8	90.4	101.1
Burundi	Kayanza	40550	2015	77.3	71.4	84.0
Burundi	Kirundo	40551	2000	230.4	219.7	240.3
Burundi	Kirundo	40551	2005	168.3	160.4	176.1
Burundi	Kirundo	40551	2010	122.6	115.8	129.4
Burundi	Kirundo	40551	2015	115.8	106.2	126.4
Burundi	Makamba	40552	2000	115.7	107.2	124.7
Burundi	Makamba	40552	2005	100.7	93.5	108.6
Burundi	Makamba	40552	2010	78.6	72.6	85.3
Burundi	Makamba	40552	2015	67.7	60.5	75.5
Burundi	Muramvya	40553	2000	142.8	135.2	150.8
Burundi	Muramvya	40553	2005	111.6	105.5	118.0
Burundi	Muramvya	40553	2010	85.9	80.6	91.4
Burundi	Muramvya	40553	2015	69.6	63.5	76.1
Burundi	Muyinga	40554	2000	207.9	198.0	217.9
Burundi	Muyinga	40554	2005	158.5	150.4	166.7
Burundi	Muyinga	40554	2010	119.8	113.2	127.2
Burundi	Muyinga	40554	2015	105.4	96.7	114.9
Burundi	Mwaro	40555	2000	130.4	123.3	138.4
Burundi	Mwaro	40555	2005	105.1	99.4	111.4
Burundi	Mwaro	40555	2010	83.4	78.5	88.7
Burundi	Mwaro	40555	2015	67.2	61.5	73.7
Burundi	Ngozi	40556	2000	197.8	187.3	208.2
Burundi	Ngozi	40556	2005	150.5	143.1	158.7
Burundi	Ngozi	40556	2010	113.4	106.7	120.3
Burundi	Ngozi	40556	2015	97.2	89.1	105.7
Burundi	Rutana	40557	2000	124.2	116.3	132.9
Burundi	Rutana	40557	2005	107.8	100.9	115.3

Admin 0	Admin 1	GAUL Code	Year	Under-5 mortality (per 1,000 livebirths)		
				Estimate	Lower bound, 95% UI	Upper bound, 95% UI
Burundi	Rutana	40557	2010	85.2	79.4	91.7
Burundi	Rutana	40557	2015	75.6	68.2	82.8
Burundi	Ruyigi	40558	2000	138.9	130.1	147.4
Burundi	Ruyigi	40558	2005	116.7	110.1	123.6
Burundi	Ruyigi	40558	2010	93.1	87.0	99.5
Burundi	Ruyigi	40558	2015	82.9	75.2	91.2
Cameroon	Adamaoua	815	2000	158.2	147.1	169.6
Cameroon	Adamaoua	815	2005	134.2	124.7	144.6
Cameroon	Adamaoua	815	2010	117.3	107.3	128.2
Cameroon	Adamaoua	815	2015	106.7	94.7	119.0
Cameroon	Centre	816	2000	115.6	108.5	123.3
Cameroon	Centre	816	2005	101.8	95.5	108.6
Cameroon	Centre	816	2010	90.9	82.8	99.7
Cameroon	Centre	816	2015	74.4	65.6	84.3
Cameroon	Est	817	2000	150.8	139.8	163.0
Cameroon	Est	817	2005	132.2	122.5	143.5
Cameroon	Est	817	2010	116.0	105.6	127.3
Cameroon	Est	817	2015	100.7	89.4	113.9
Cameroon	Extrême - Nord	818	2000	179.1	171.1	187.7
Cameroon	Extrême - Nord	818	2005	164.9	157.5	173.0
Cameroon	Extrême - Nord	818	2010	153.2	144.4	163.0
Cameroon	Extrême - Nord	818	2015	142.6	130.3	155.5
Cameroon	Littoral	819	2000	97.6	90.3	105.4
Cameroon	Littoral	819	2005	89.1	82.5	96.2
Cameroon	Littoral	819	2010	75.6	68.4	83.6
Cameroon	Littoral	819	2015	58.0	51.0	66.0
Cameroon	Nord	820	2000	211.1	200.7	221.6
Cameroon	Nord	820	2005	190.0	180.2	200.3
Cameroon	Nord	820	2010	156.7	147.5	167.0
Cameroon	Nord	820	2015	143.6	131.0	157.0
Cameroon	Nord - Ouest	821	2000	100.5	94.0	107.1
Cameroon	Nord - Ouest	821	2005	100.7	94.1	107.7
Cameroon	Nord - Ouest	821	2010	84.2	76.8	92.1
Cameroon	Nord - Ouest	821	2015	64.8	57.5	72.5
Cameroon	Ouest	822	2000	106.0	99.1	113.4
Cameroon	Ouest	822	2005	100.3	93.6	107.1
Cameroon	Ouest	822	2010	87.9	79.7	96.6
Cameroon	Ouest	822	2015	66.9	58.9	75.8
Cameroon	Sud	823	2000	137.8	126.9	149.0
Cameroon	Sud	823	2005	122.5	112.6	132.9
Cameroon	Sud	823	2010	107.9	97.9	118.9
Cameroon	Sud	823	2015	88.4	77.9	98.8
Cameroon	Sud - Ouest	824	2000	111.8	104.7	119.1
Cameroon	Sud - Ouest	824	2005	107.0	100.4	114.3
Cameroon	Sud - Ouest	824	2010	88.9	81.5	96.4
Cameroon	Sud - Ouest	824	2015	67.2	59.7	75.1
Central African Republic	Bamingui-Bangoran	853	2000	184.6	158.1	213.3
Central African Republic	Bamingui-Bangoran	853	2005	169.0	143.1	196.1
Central African Republic	Bamingui-Bangoran	853	2010	146.6	124.0	170.9
Central African Republic	Bamingui-Bangoran	853	2015	135.9	115.0	159.6
Central African Republic	Bangui	67154	2000	138.6	118.6	161.1
Central African Republic	Bangui	67154	2005	129.8	111.5	150.6
Central African Republic	Bangui	67154	2010	130.5	111.1	152.6
Central African Republic	Bangui	67154	2015	109.5	91.7	132.3
Central African Republic	Basse-Kotto	854	2000	179.6	155.1	205.6
Central African Republic	Basse-Kotto	854	2005	175.2	150.0	203.1
Central African Republic	Basse-Kotto	854	2010	147.7	125.4	173.7
Central African Republic	Basse-Kotto	854	2015	133.3	111.5	159.0
Central African Republic	Haut-Mbomou	855	2000	152.5	130.3	177.9
Central African Republic	Haut-Mbomou	855	2005	140.3	119.9	163.8
Central African Republic	Haut-Mbomou	855	2010	136.7	114.5	161.0
Central African Republic	Haut-Mbomou	855	2015	125.2	104.7	148.3
Central African Republic	Haute-Kotto	856	2000	163.3	133.9	195.5
Central African Republic	Haute-Kotto	856	2005	163.1	132.9	195.8
Central African Republic	Haute-Kotto	856	2010	136.2	110.6	165.2
Central African Republic	Haute-Kotto	856	2015	131.4	106.0	158.7
Central African Republic	Kémo	857	2000	175.2	148.6	208.1
Central African Republic	Kémo	857	2005	177.7	151.1	208.6
Central African Republic	Kémo	857	2010	158.6	131.8	186.6

Admin 0	Admin 1	GAUL Code	Year	Under-5 mortality (per 1,000 livebirths)		
				Estimate	Lower bound, 95% UI	Upper bound, 95% UI
Central African Republic	Kémo	857	2015	136.4	113.1	163.6
Central African Republic	Lobaye	858	2000	176.2	153.6	200.3
Central African Republic	Lobaye	858	2005	153.0	133.2	174.8
Central African Republic	Lobaye	858	2010	143.0	123.3	166.2
Central African Republic	Lobaye	858	2015	121.7	102.2	141.6
Central African Republic	Mambéré-Kadéï	859	2000	186.7	162.5	215.6
Central African Republic	Mambéré-Kadéï	859	2005	163.5	141.2	189.4
Central African Republic	Mambéré-Kadéï	859	2010	151.4	129.9	177.6
Central African Republic	Mambéré-Kadéï	859	2015	132.5	111.5	155.9
Central African Republic	Mbomou	860	2000	167.7	142.9	195.7
Central African Republic	Mbomou	860	2005	165.9	141.3	191.6
Central African Republic	Mbomou	860	2010	138.4	117.2	161.5
Central African Republic	Mbomou	860	2015	131.9	111.1	156.3
Central African Republic	Nana-Gribizi	861	2000	172.1	144.1	202.8
Central African Republic	Nana-Gribizi	861	2005	173.6	143.8	205.6
Central African Republic	Nana-Gribizi	861	2010	150.9	124.4	180.5
Central African Republic	Nana-Gribizi	861	2015	127.3	104.5	153.2
Central African Republic	Nana-Mambéré	862	2000	182.8	158.1	209.5
Central African Republic	Nana-Mambéré	862	2005	162.8	140.3	186.4
Central African Republic	Nana-Mambéré	862	2010	156.8	133.1	181.5
Central African Republic	Nana-Mambéré	862	2015	136.7	115.4	161.1
Central African Republic	Ombella M'Poko	863	2000	161.1	140.5	182.3
Central African Republic	Ombella M'Poko	863	2005	149.8	131.1	169.6
Central African Republic	Ombella M'Poko	863	2010	143.6	124.6	165.3
Central African Republic	Ombella M'Poko	863	2015	123.4	105.6	145.7
Central African Republic	Ouaka	864	2000	189.9	164.3	222.3
Central African Republic	Ouaka	864	2005	185.1	157.9	214.4
Central African Republic	Ouaka	864	2010	157.7	132.1	183.7
Central African Republic	Ouaka	864	2015	141.9	117.6	169.4
Central African Republic	Ouham	865	2000	187.5	162.8	214.3
Central African Republic	Ouham	865	2005	196.4	168.8	225.6
Central African Republic	Ouham	865	2010	170.5	146.5	199.2
Central African Republic	Ouham	865	2015	152.9	129.9	180.0
Central African Republic	Ouham Pendé	866	2000	202.7	179.8	227.6
Central African Republic	Ouham Pendé	866	2005	206.1	182.5	230.2
Central African Republic	Ouham Pendé	866	2010	187.7	163.6	212.9
Central African Republic	Ouham Pendé	866	2015	178.0	153.5	205.6
Central African Republic	Sangha-Mbaéré	867	2000	180.0	156.3	205.5
Central African Republic	Sangha-Mbaéré	867	2005	156.0	135.7	179.0
Central African Republic	Sangha-Mbaéré	867	2010	147.3	125.4	171.8
Central African Republic	Sangha-Mbaéré	867	2015	125.3	104.6	148.1
Central African Republic	Vakaga	868	2000	149.6	129.4	172.8
Central African Republic	Vakaga	868	2005	139.2	119.4	160.7
Central African Republic	Vakaga	868	2010	127.4	108.8	148.3
Central African Republic	Vakaga	868	2015	114.9	98.5	134.9
Chad	Assongha	12926	2000	146.0	133.4	160.3
Chad	Assongha	12926	2005	128.6	117.7	140.3
Chad	Assongha	12926	2010	98.1	89.5	107.3
Chad	Assongha	12926	2015	79.4	70.6	88.8
Chad	Baguirmi	12910	2000	191.5	177.8	205.7
Chad	Baguirmi	12910	2005	187.2	174.1	200.1
Chad	Baguirmi	12910	2010	164.7	152.3	176.6
Chad	Baguirmi	12910	2015	148.1	134.7	162.3
Chad	Barh Koh	12923	2000	212.1	195.2	230.4
Chad	Barh Koh	12923	2005	180.2	165.9	194.1
Chad	Barh Koh	12923	2010	147.6	135.9	160.0
Chad	Barh Koh	12923	2015	126.3	113.5	139.8
Chad	Barl El Gazal	12914	2000	124.4	114.0	136.8
Chad	Barl El Gazal	12914	2005	103.0	94.4	113.6
Chad	Barl El Gazal	12914	2010	84.7	77.4	92.8
Chad	Barl El Gazal	12914	2015	72.7	65.3	81.1
Chad	Batha Est	12904	2000	134.5	119.6	150.9
Chad	Batha Est	12904	2005	115.9	103.6	129.3
Chad	Batha Est	12904	2010	96.1	85.3	108.1
Chad	Batha Est	12904	2015	86.7	75.7	98.8
Chad	Batha Ouest	12905	2000	134.3	121.7	148.0
Chad	Batha Ouest	12905	2005	122.3	111.5	134.8
Chad	Batha Ouest	12905	2010	101.3	91.4	111.6
Chad	Batha Ouest	12905	2015	91.9	81.5	102.9

Admin 0	Admin 1	GAUL Code	Year	Under-5 mortality (per 1,000 livebirths)		
				Estimate	Lower bound, 95% UI	Upper bound, 95% UI
Chad	Biltine	870	2000	113.7	103.3	124.1
Chad	Biltine	870	2005	92.7	84.4	100.8
Chad	Biltine	870	2010	75.0	68.4	82.1
Chad	Biltine	870	2015	65.7	58.7	73.3
Chad	Borkou	12907	2000	140.2	124.0	156.8
Chad	Borkou	12907	2005	118.8	106.6	131.2
Chad	Borkou	12907	2010	102.6	91.4	114.2
Chad	Borkou	12907	2015	90.1	79.2	102.0
Chad	Daraba	12911	2000	164.2	150.0	180.9
Chad	Daraba	12911	2005	155.6	142.3	171.1
Chad	Daraba	12911	2010	130.6	118.7	143.4
Chad	Daraba	12911	2015	118.1	104.3	132.3
Chad	Ennedi	12908	2000	125.7	111.8	141.9
Chad	Ennedi	12908	2005	103.5	91.8	116.9
Chad	Ennedi	12908	2010	89.5	78.9	100.9
Chad	Ennedi	12908	2015	73.5	64.0	84.5
Chad	Guera	873	2000	194.3	180.1	209.8
Chad	Guera	873	2005	180.5	168.2	193.4
Chad	Guera	873	2010	149.1	138.3	160.1
Chad	Guera	873	2015	129.9	117.5	142.8
Chad	Hadjer Lamis	12912	2000	167.5	157.8	177.5
Chad	Hadjer Lamis	12912	2005	143.6	135.4	152.4
Chad	Hadjer Lamis	12912	2010	121.6	113.5	129.4
Chad	Hadjer Lamis	12912	2015	116.1	106.6	127.2
Chad	Kabia	12920	2000	165.3	153.7	177.3
Chad	Kabia	12920	2005	162.6	152.1	172.8
Chad	Kabia	12920	2010	156.5	145.8	167.8
Chad	Kabia	12920	2015	129.8	117.8	142.5
Chad	Kanem	12915	2000	143.3	132.5	153.6
Chad	Kanem	12915	2005	118.0	109.6	126.7
Chad	Kanem	12915	2010	93.4	86.5	100.8
Chad	Kanem	12915	2015	80.9	73.3	89.3
Chad	Lac	875	2000	141.3	127.9	154.8
Chad	Lac	875	2005	114.8	104.3	125.9
Chad	Lac	875	2010	92.9	84.1	102.1
Chad	Lac	875	2015	82.3	73.6	91.9
Chad	Lac Iro	12924	2000	218.6	195.9	243.9
Chad	Lac Iro	12924	2005	184.1	165.1	204.9
Chad	Lac Iro	12924	2010	152.1	135.4	170.9
Chad	Lac Iro	12924	2015	135.3	118.4	153.9
Chad	Logone Occidental	876	2000	240.6	225.0	256.1
Chad	Logone Occidental	876	2005	235.5	222.3	250.0
Chad	Logone Occidental	876	2010	204.4	192.4	217.9
Chad	Logone Occidental	876	2015	156.9	143.7	171.4
Chad	Logone Oriental	12918	2000	248.4	232.8	263.8
Chad	Logone Oriental	12918	2005	235.7	222.8	248.9
Chad	Logone Oriental	12918	2010	198.5	186.2	211.3
Chad	Logone Oriental	12918	2015	155.9	142.5	170.7
Chad	Mandoul	12925	2000	217.8	203.3	233.1
Chad	Mandoul	12925	2005	195.2	182.9	207.9
Chad	Mandoul	12925	2010	163.6	152.2	174.5
Chad	Mandoul	12925	2015	139.0	125.7	151.8
Chad	Mayo-Boneye	12921	2000	160.3	148.1	173.4
Chad	Mayo-Boneye	12921	2005	151.8	140.3	164.5
Chad	Mayo-Boneye	12921	2010	142.3	131.2	154.5
Chad	Mayo-Boneye	12921	2015	125.7	113.1	138.9
Chad	Mayo-Dala	12922	2000	181.3	169.5	193.8
Chad	Mayo-Dala	12922	2005	170.5	160.2	181.5
Chad	Mayo-Dala	12922	2010	154.3	144.4	164.8
Chad	Mayo-Dala	12922	2015	128.3	116.8	140.1
Chad	Mont De Lam	12919	2000	242.7	219.6	266.3
Chad	Mont De Lam	12919	2005	226.2	205.7	247.0
Chad	Mont De Lam	12919	2010	190.1	172.6	210.6
Chad	Mont De Lam	12919	2015	161.1	143.6	180.8
Chad	Ouaddai	12927	2000	141.0	129.4	153.6
Chad	Ouaddai	12927	2005	120.5	111.0	130.2
Chad	Ouaddai	12927	2010	95.6	87.8	104.4
Chad	Ouaddai	12927	2015	82.3	73.8	91.2
Chad	Salamat	881	2000	190.5	175.8	206.7

Admin 0	Admin 1	GAUL Code	Year	Under-5 mortality (per 1,000 livebirths)		
				Estimate	Lower bound, 95% UI	Upper bound, 95% UI
Chad	Salamat	881	2005	172.0	159.5	185.0
Chad	Salamat	881	2010	141.4	130.6	152.8
Chad	Salamat	881	2015	127.5	115.1	140.8
Chad	Sila	12928	2000	166.3	152.1	181.1
Chad	Sila	12928	2005	146.5	133.7	159.9
Chad	Sila	12928	2010	121.6	111.1	132.9
Chad	Sila	12928	2015	101.3	89.9	113.5
Chad	Tandjile Est	12930	2000	227.5	208.9	247.6
Chad	Tandjile Est	12930	2005	211.7	194.3	230.4
Chad	Tandjile Est	12930	2010	182.4	166.4	197.7
Chad	Tandjile Est	12930	2015	144.3	129.9	160.1
Chad	Tandjile Ouest	12931	2000	221.4	205.5	238.2
Chad	Tandjile Ouest	12931	2005	214.5	200.2	229.8
Chad	Tandjile Ouest	12931	2010	197.5	183.2	212.3
Chad	Tandjile Ouest	12931	2015	150.8	136.2	165.7
Chad	Tibesti	12909	2000	129.9	106.2	157.2
Chad	Tibesti	12909	2005	107.8	86.7	131.5
Chad	Tibesti	12909	2010	92.9	76.1	113.7
Chad	Tibesti	12909	2015	72.9	59.2	88.9
Congo	Bouenza	970	2000	124.2	114.3	134.7
Congo	Bouenza	970	2005	103.1	95.1	111.5
Congo	Bouenza	970	2010	74.9	68.2	82.1
Congo	Bouenza	970	2015	62.2	55.2	69.9
Congo	Brazzaville	190432	2000	96.6	90.1	103.6
Congo	Brazzaville	190432	2005	82.6	76.6	88.9
Congo	Brazzaville	190432	2010	65.9	59.6	72.2
Congo	Brazzaville	190432	2015	50.9	44.7	58.2
Congo	Cuvette	971	2000	103.6	92.8	114.3
Congo	Cuvette	971	2005	93.1	84.6	101.8
Congo	Cuvette	971	2010	73.5	66.2	81.3
Congo	Cuvette	971	2015	60.7	53.4	68.6
Congo	Cuvette-Ouest	972	2000	107.3	96.8	118.2
Congo	Cuvette-Ouest	972	2005	96.5	87.3	106.1
Congo	Cuvette-Ouest	972	2010	77.6	70.2	86.3
Congo	Cuvette-Ouest	972	2015	65.0	57.3	73.6
Congo	Kouilou	190433	2000	99.1	90.7	107.8
Congo	Kouilou	190433	2005	84.1	76.7	92.0
Congo	Kouilou	190433	2010	64.3	58.5	71.2
Congo	Kouilou	190433	2015	57.2	50.2	65.0
Congo	Lekoumou	974	2000	113.3	103.1	125.3
Congo	Lekoumou	974	2005	94.9	86.1	104.8
Congo	Lekoumou	974	2010	72.5	65.0	81.1
Congo	Lekoumou	974	2015	60.2	52.5	68.7
Congo	Likouala	975	2000	101.9	92.5	111.7
Congo	Likouala	975	2005	92.0	84.1	101.2
Congo	Likouala	975	2010	73.6	66.2	81.1
Congo	Likouala	975	2015	61.8	54.6	69.9
Congo	Niari	976	2000	97.9	90.2	106.4
Congo	Niari	976	2005	87.0	80.3	94.4
Congo	Niari	976	2010	65.0	59.2	71.1
Congo	Niari	976	2015	55.8	49.8	62.5
Congo	Plateaux	977	2000	107.4	96.8	117.9
Congo	Plateaux	977	2005	90.9	82.7	99.2
Congo	Plateaux	977	2010	71.9	64.7	79.2
Congo	Plateaux	977	2015	59.4	52.0	67.5
Congo	Point-Noire	190434	2000	93.6	85.4	102.0
Congo	Point-Noire	190434	2005	77.0	70.2	84.3
Congo	Point-Noire	190434	2010	61.8	55.5	68.9
Congo	Point-Noire	190434	2015	55.2	48.1	62.9
Congo	Pool	190431	2000	118.6	109.7	128.2
Congo	Pool	190431	2005	95.3	88.3	102.9
Congo	Pool	190431	2010	72.6	66.2	79.8
Congo	Pool	190431	2015	58.0	51.4	65.0
Congo	Sangha	979	2000	126.9	115.6	138.2
Congo	Sangha	979	2005	115.3	104.7	126.5
Congo	Sangha	979	2010	95.3	85.9	106.2
Congo	Sangha	979	2015	82.5	72.6	92.8
Côte d'Ivoire	Bas Sassandra	190430	2000	132.8	120.9	145.1
Côte d'Ivoire	Bas Sassandra	190430	2005	119.3	108.7	131.3

Admin 0	Admin 1	GAUL Code	Year	Under-5 mortality (per 1,000 livebirths)		
				Estimate	Lower bound, 95% UI	Upper bound, 95% UI
Côte d'Ivoire	Bas Sassandra	190430	2010	99.6	89.9	110.2
Côte d'Ivoire	Bas Sassandra	190430	2015	89.6	79.4	101.7
Côte d'Ivoire	Comoe	16837	2000	128.6	119.1	138.4
Côte d'Ivoire	Comoe	16837	2005	115.1	106.5	124.3
Côte d'Ivoire	Comoe	16837	2010	99.1	90.9	108.2
Côte d'Ivoire	Comoe	16837	2015	80.0	71.6	88.9
Côte d'Ivoire	Denguele	1041	2000	190.0	174.3	207.8
Côte d'Ivoire	Denguele	1041	2005	177.4	161.7	194.1
Côte d'Ivoire	Denguele	1041	2010	156.1	141.5	172.6
Côte d'Ivoire	Denguele	1041	2015	130.6	114.9	148.0
Côte d'Ivoire	District autonome de Abidjan	16838	2000	99.2	88.9	110.5
Côte d'Ivoire	District autonome de Abidjan	16838	2005	102.8	92.7	114.2
Côte d'Ivoire	District autonome de Abidjan	16838	2010	90.8	80.5	101.7
Côte d'Ivoire	District autonome de Abidjan	16838	2015	71.4	61.4	82.3
Côte d'Ivoire	District autonome de Yamoussoukro	16839	2000	116.3	103.3	129.8
Côte d'Ivoire	District autonome de Yamoussoukro	16839	2005	100.6	89.3	112.9
Côte d'Ivoire	District autonome de Yamoussoukro	16839	2010	89.8	78.7	102.2
Côte d'Ivoire	District autonome de Yamoussoukro	16839	2015	77.9	66.9	89.5
Côte d'Ivoire	Gôh-Djiboua	16840	2000	121.5	109.2	134.9
Côte d'Ivoire	Gôh-Djiboua	16840	2005	110.2	99.6	122.5
Côte d'Ivoire	Gôh-Djiboua	16840	2010	94.3	83.9	105.5
Côte d'Ivoire	Gôh-Djiboua	16840	2015	84.3	73.4	96.7
Côte d'Ivoire	Lacs	16841	2000	134.4	123.4	146.6
Côte d'Ivoire	Lacs	16841	2005	123.2	112.0	134.6
Côte d'Ivoire	Lacs	16841	2010	108.4	97.6	119.7
Côte d'Ivoire	Lacs	16841	2015	91.2	80.6	103.4
Côte d'Ivoire	Lagunes	16842	2000	125.7	114.0	137.8
Côte d'Ivoire	Lagunes	16842	2005	119.2	108.3	131.0
Côte d'Ivoire	Lagunes	16842	2010	100.4	90.6	112.1
Côte d'Ivoire	Lagunes	16842	2015	82.9	73.2	94.2
Côte d'Ivoire	Montagnes	16843	2000	169.5	156.7	183.4
Côte d'Ivoire	Montagnes	16843	2005	136.0	125.3	147.5
Côte d'Ivoire	Montagnes	16843	2010	114.4	103.8	125.9
Côte d'Ivoire	Montagnes	16843	2015	100.3	88.3	113.3
Côte d'Ivoire	Sassandra-Marahoue	16844	2000	144.7	131.9	158.3
Côte d'Ivoire	Sassandra-Marahoue	16844	2005	123.2	112.8	133.9
Côte d'Ivoire	Sassandra-Marahoue	16844	2010	107.1	96.4	118.8
Côte d'Ivoire	Sassandra-Marahoue	16844	2015	96.5	85.2	109.9
Côte d'Ivoire	Savanes	1048	2000	192.1	179.2	205.1
Côte d'Ivoire	Savanes	1048	2005	183.8	171.0	196.2
Côte d'Ivoire	Savanes	1048	2010	164.6	150.8	178.2
Côte d'Ivoire	Savanes	1048	2015	136.0	122.2	150.7
Côte d'Ivoire	Vallee Du Bandama	1051	2000	123.2	112.5	133.9
Côte d'Ivoire	Vallee Du Bandama	1051	2005	113.0	102.9	123.1
Côte d'Ivoire	Vallee Du Bandama	1051	2010	102.0	91.7	112.1
Côte d'Ivoire	Vallee Du Bandama	1051	2015	85.9	75.7	96.9
Côte d'Ivoire	Woroba	16845	2000	181.4	169.3	194.5
Côte d'Ivoire	Woroba	16845	2005	158.5	147.3	170.0
Côte d'Ivoire	Woroba	16845	2010	135.1	123.7	147.5
Côte d'Ivoire	Woroba	16845	2015	109.9	98.4	122.4
Côte d'Ivoire	Zanzan	1053	2000	157.0	145.9	168.7
Côte d'Ivoire	Zanzan	1053	2005	144.9	134.5	156.8
Côte d'Ivoire	Zanzan	1053	2010	127.2	116.6	138.3
Côte d'Ivoire	Zanzan	1053	2015	102.2	91.3	114.1
Democratic Republic of the Congo	Bandundu	1066	2000	122.4	113.4	131.8
Democratic Republic of the Congo	Bandundu	1066	2005	109.0	101.5	117.1
Democratic Republic of the Congo	Bandundu	1066	2010	87.7	80.9	94.5
Democratic Republic of the Congo	Bandundu	1066	2015	65.5	59.7	71.6
Democratic Republic of the Congo	Bas-Congo	1067	2000	161.7	149.5	174.0
Democratic Republic of the Congo	Bas-Congo	1067	2005	127.4	118.6	136.7
Democratic Republic of the Congo	Bas-Congo	1067	2010	109.3	100.2	118.4
Democratic Republic of the Congo	Bas-Congo	1067	2015	79.6	71.4	88.3
Democratic Republic of the Congo	Equateur	1068	2000	159.3	149.5	169.4
Democratic Republic of the Congo	Equateur	1068	2005	141.0	132.8	149.7
Democratic Republic of the Congo	Equateur	1068	2010	113.7	106.4	121.1
Democratic Republic of the Congo	Equateur	1068	2015	88.1	80.9	95.0
Democratic Republic of the Congo	Kasai Occidental	1069	2000	177.6	166.8	189.3
Democratic Republic of the Congo	Kasai Occidental	1069	2005	155.5	145.5	165.3
Democratic Republic of the Congo	Kasai Occidental	1069	2010	133.7	124.1	143.7

Admin 0	Admin 1	GAUL Code	Year	Under-5 mortality (per 1,000 livebirths)		
				Estimate	Lower bound, 95% UI	Upper bound, 95% UI
Democratic Republic of the Congo	Kasai Occidental	1069	2015	111.9	101.5	123.0
Democratic Republic of the Congo	Kasai Oriental	1070	2000	139.9	129.9	150.2
Democratic Republic of the Congo	Kasai Oriental	1070	2005	127.9	119.8	136.2
Democratic Republic of the Congo	Kasai Oriental	1070	2010	114.6	106.2	123.8
Democratic Republic of the Congo	Kasai Oriental	1070	2015	94.3	85.9	104.6
Democratic Republic of the Congo	Katanga	1071	2000	165.8	156.1	175.7
Democratic Republic of the Congo	Katanga	1071	2005	142.8	134.9	151.6
Democratic Republic of the Congo	Katanga	1071	2010	122.3	114.6	130.2
Democratic Republic of the Congo	Katanga	1071	2015	104.2	96.4	112.1
Democratic Republic of the Congo	Kinshasa	1072	2000	106.9	99.9	114.6
Democratic Republic of the Congo	Kinshasa	1072	2005	90.9	84.6	97.4
Democratic Republic of the Congo	Kinshasa	1072	2010	79.9	72.4	87.4
Democratic Republic of the Congo	Kinshasa	1072	2015	55.0	48.1	62.4
Democratic Republic of the Congo	Maniema	1073	2000	176.4	164.2	188.2
Democratic Republic of the Congo	Maniema	1073	2005	146.6	137.3	157.1
Democratic Republic of the Congo	Maniema	1073	2010	125.3	115.3	135.7
Democratic Republic of the Congo	Maniema	1073	2015	114.2	103.3	126.0
Democratic Republic of the Congo	Nord-Kivu	1074	2000	131.2	122.7	139.9
Democratic Republic of the Congo	Nord-Kivu	1074	2005	98.6	92.1	105.0
Democratic Republic of the Congo	Nord-Kivu	1074	2010	75.5	69.4	81.2
Democratic Republic of the Congo	Nord-Kivu	1074	2015	57.3	52.0	62.8
Democratic Republic of the Congo	Orientale	1075	2000	160.9	149.4	173.1
Democratic Republic of the Congo	Orientale	1075	2005	137.8	128.5	148.4
Democratic Republic of the Congo	Orientale	1075	2010	116.8	107.8	126.6
Democratic Republic of the Congo	Orientale	1075	2015	95.1	86.6	105.1
Democratic Republic of the Congo	Sud-Kivu	1076	2000	195.5	183.7	207.1
Democratic Republic of the Congo	Sud-Kivu	1076	2005	150.6	142.0	160.1
Democratic Republic of the Congo	Sud-Kivu	1076	2010	118.6	110.9	127.3
Democratic Republic of the Congo	Sud-Kivu	1076	2015	93.1	85.1	101.0
Djibouti	Ali Sabieh	1093	2000	89.0	80.5	98.4
Djibouti	Ali Sabieh	1093	2005	76.7	68.1	86.1
Djibouti	Ali Sabieh	1093	2010	62.0	53.5	71.1
Djibouti	Ali Sabieh	1093	2015	48.0	40.0	56.9
Djibouti	Dikhil	1094	2000	82.2	74.2	90.5
Djibouti	Dikhil	1094	2005	72.9	65.0	81.2
Djibouti	Dikhil	1094	2010	63.6	55.3	72.5
Djibouti	Dikhil	1094	2015	49.0	41.3	56.9
Djibouti	Djibouti	1095	2000	107.7	97.7	118.4
Djibouti	Djibouti	1095	2005	88.4	78.7	99.7
Djibouti	Djibouti	1095	2010	69.0	59.1	79.9
Djibouti	Djibouti	1095	2015	54.2	45.2	64.2
Djibouti	Obock	1096	2000	108.7	94.1	124.8
Djibouti	Obock	1096	2005	93.9	80.1	109.5
Djibouti	Obock	1096	2010	80.0	66.4	94.8
Djibouti	Obock	1096	2015	61.7	50.8	75.1
Djibouti	Tadjourah	1097	2000	103.0	92.4	115.2
Djibouti	Tadjourah	1097	2005	88.7	78.1	100.8
Djibouti	Tadjourah	1097	2010	76.6	65.8	88.4
Djibouti	Tadjourah	1097	2015	59.7	50.4	70.2
Egypt	Alexandria	61510	2000	34.0	29.2	39.1
Egypt	Alexandria	61510	2005	29.8	25.5	34.1
Egypt	Alexandria	61510	2010	25.0	21.3	28.7
Egypt	Alexandria	61510	2015	18.3	15.3	21.5
Egypt	Assiut	61511	2000	66.1	59.9	72.5
Egypt	Assiut	61511	2005	48.7	44.1	53.9
Egypt	Assiut	61511	2010	37.6	33.1	42.2
Egypt	Assiut	61511	2015	27.5	23.4	31.6
Egypt	Aswan	61512	2000	53.0	47.3	59.1
Egypt	Aswan	61512	2005	49.3	43.5	55.2
Egypt	Aswan	61512	2010	38.2	33.2	43.7
Egypt	Aswan	61512	2015	27.3	23.4	31.8
Egypt	Behera	61513	2000	35.4	31.7	39.4
Egypt	Behera	61513	2005	27.8	25.0	31.0
Egypt	Behera	61513	2010	21.8	19.3	24.5
Egypt	Behera	61513	2015	16.8	14.6	19.3
Egypt	Beni Suef	61514	2000	59.6	54.5	65.2
Egypt	Beni Suef	61514	2005	45.1	41.4	49.3
Egypt	Beni Suef	61514	2010	31.8	28.4	35.7
Egypt	Beni Suef	61514	2015	25.7	22.4	29.4

Admin 0	Admin 1	GAUL Code	Year	Under-5 mortality (per 1,000 livebirths)		
				Estimate	Lower bound, 95% UI	Upper bound, 95% UI
Egypt	Cairo	61515	2000	37.4	34.3	40.7
Egypt	Cairo	61515	2005	32.5	29.7	35.5
Egypt	Cairo	61515	2010	27.3	24.6	30.2
Egypt	Cairo	61515	2015	20.8	18.3	23.8
Egypt	Dakahlia	61516	2000	32.3	29.5	35.1
Egypt	Dakahlia	61516	2005	26.2	23.8	28.6
Egypt	Dakahlia	61516	2010	22.4	20.1	24.8
Egypt	Dakahlia	61516	2015	17.0	14.8	19.4
Egypt	Damietta	61517	2000	30.7	27.3	34.4
Egypt	Damietta	61517	2005	25.8	22.9	28.8
Egypt	Damietta	61517	2010	23.4	20.6	26.7
Egypt	Damietta	61517	2015	16.9	14.5	19.6
Egypt	Fayoum	61518	2000	52.4	47.6	57.4
Egypt	Fayoum	61518	2005	39.2	35.2	43.1
Egypt	Fayoum	61518	2010	28.2	25.2	31.9
Egypt	Fayoum	61518	2015	23.4	20.3	27.0
Egypt	Gharbia	61519	2000	32.1	29.2	35.0
Egypt	Gharbia	61519	2005	25.8	23.4	28.3
Egypt	Gharbia	61519	2010	20.8	18.7	23.2
Egypt	Gharbia	61519	2015	16.3	14.2	18.6
Egypt	Giza	61520	2000	40.3	37.2	43.9
Egypt	Giza	61520	2005	33.3	30.5	36.2
Egypt	Giza	61520	2010	27.1	24.4	29.9
Egypt	Giza	61520	2015	21.1	18.6	24.0
Egypt	Hala'ib triangle	40776	2000	70.3	56.0	87.5
Egypt	Hala'ib triangle	40776	2005	59.9	47.6	74.0
Egypt	Hala'ib triangle	40776	2010	48.6	38.4	60.4
Egypt	Hala'ib triangle	40776	2015	36.5	29.0	45.8
Egypt	Ismailia	61521	2000	36.0	32.3	40.2
Egypt	Ismailia	61521	2005	31.5	27.9	35.2
Egypt	Ismailia	61521	2010	26.3	23.2	29.4
Egypt	Ismailia	61521	2015	19.4	16.7	22.4
Egypt	Kafr El-Shikh	61522	2000	32.6	29.0	36.3
Egypt	Kafr El-Shikh	61522	2005	25.9	23.1	28.7
Egypt	Kafr El-Shikh	61522	2010	21.1	18.7	23.8
Egypt	Kafr El-Shikh	61522	2015	16.1	13.9	18.8
Egypt	Kalyoubia	61523	2000	37.3	34.5	40.4
Egypt	Kalyoubia	61523	2005	30.6	28.1	33.2
Egypt	Kalyoubia	61523	2010	25.1	22.8	27.7
Egypt	Kalyoubia	61523	2015	19.3	17.0	21.8
Egypt	Luxor	61524	2000	54.6	48.8	60.9
Egypt	Luxor	61524	2005	45.8	41.0	50.9
Egypt	Luxor	61524	2010	34.1	30.0	38.5
Egypt	Luxor	61524	2015	24.3	20.7	28.1
Egypt	Matrouh	61525	2000	45.5	38.0	53.0
Egypt	Matrouh	61525	2005	38.4	32.1	44.8
Egypt	Matrouh	61525	2010	31.0	25.8	36.5
Egypt	Matrouh	61525	2015	21.6	17.9	25.8
Egypt	Menia	61526	2000	66.6	61.2	72.2
Egypt	Menia	61526	2005	49.8	45.4	54.9
Egypt	Menia	61526	2010	36.5	32.3	41.1
Egypt	Menia	61526	2015	26.9	23.5	31.0
Egypt	Menoufia	61527	2000	35.4	32.3	38.7
Egypt	Menoufia	61527	2005	28.0	25.5	30.7
Egypt	Menoufia	61527	2010	22.3	20.0	25.1
Egypt	Menoufia	61527	2015	17.3	15.1	19.7
Egypt	New Valley	61528	2000	51.7	45.8	58.0
Egypt	New Valley	61528	2005	48.5	43.1	54.4
Egypt	New Valley	61528	2010	41.7	36.7	47.3
Egypt	New Valley	61528	2015	29.0	25.4	33.2
Egypt	North Sinai	61529	2000	40.8	33.4	49.5
Egypt	North Sinai	61529	2005	37.1	30.1	45.0
Egypt	North Sinai	61529	2010	32.2	26.1	39.7
Egypt	North Sinai	61529	2015	22.8	18.0	28.2
Egypt	Port Said	61530	2000	30.5	26.8	34.4
Egypt	Port Said	61530	2005	27.6	24.0	31.4
Egypt	Port Said	61530	2010	25.7	22.1	29.5
Egypt	Port Said	61530	2015	18.7	15.9	22.0
Egypt	Qena	61531	2000	60.7	55.6	66.1

Admin 0	Admin 1	GAUL Code	Year	Under-5 mortality (per 1,000 livebirths)		
				Estimate	Lower bound, 95% UI	Upper bound, 95% UI
Egypt	Qena	61531	2005	48.2	43.9	52.6
Egypt	Qena	61531	2010	35.6	31.6	39.5
Egypt	Qena	61531	2015	25.7	22.3	29.3
Egypt	Red Sea	61532	2000	48.0	40.5	57.2
Egypt	Red Sea	61532	2005	44.7	37.7	53.1
Egypt	Red Sea	61532	2010	40.0	33.4	47.7
Egypt	Red Sea	61532	2015	28.2	23.4	33.6
Egypt	Shrkia	61533	2000	35.9	32.9	39.1
Egypt	Shrkia	61533	2005	29.0	26.6	31.7
Egypt	Shrkia	61533	2010	24.7	22.1	27.2
Egypt	Shrkia	61533	2015	19.1	16.8	21.6
Egypt	South Sinai	61534	2000	43.5	34.9	53.5
Egypt	South Sinai	61534	2005	41.7	33.1	51.4
Egypt	South Sinai	61534	2010	36.8	29.3	45.3
Egypt	South Sinai	61534	2015	26.6	21.3	33.1
Egypt	Suez	61535	2000	35.6	31.1	40.4
Egypt	Suez	61535	2005	32.2	28.1	36.6
Egypt	Suez	61535	2010	26.6	23.1	30.5
Egypt	Suez	61535	2015	19.8	16.7	23.2
Egypt	Suhag	61536	2000	65.2	59.6	71.6
Egypt	Suhag	61536	2005	48.4	43.8	53.3
Egypt	Suhag	61536	2010	36.9	32.9	41.4
Egypt	Suhag	61536	2015	28.2	24.2	32.5
Equatorial Guinea	Bioko Norte	1199	2000	101.1	84.4	120.3
Equatorial Guinea	Bioko Norte	1199	2005	96.7	80.5	115.9
Equatorial Guinea	Bioko Norte	1199	2010	85.4	70.4	104.2
Equatorial Guinea	Bioko Norte	1199	2015	56.3	46.5	67.9
Equatorial Guinea	Bioko Sur	1200	2000	106.0	87.6	127.3
Equatorial Guinea	Bioko Sur	1200	2005	100.0	81.6	120.7
Equatorial Guinea	Bioko Sur	1200	2010	89.5	72.5	108.7
Equatorial Guinea	Bioko Sur	1200	2015	58.9	47.6	72.0
Equatorial Guinea	Centro Sur	1201	2000	153.2	130.1	179.6
Equatorial Guinea	Centro Sur	1201	2005	110.3	94.4	129.8
Equatorial Guinea	Centro Sur	1201	2010	87.0	73.9	103.4
Equatorial Guinea	Centro Sur	1201	2015	63.0	52.3	76.3
Equatorial Guinea	Kientem	1202	2000	179.1	156.4	206.7
Equatorial Guinea	Kientem	1202	2005	124.6	108.8	143.9
Equatorial Guinea	Kientem	1202	2010	98.5	84.5	114.8
Equatorial Guinea	Kientem	1202	2015	71.6	60.4	84.8
Equatorial Guinea	Litoral	1203	2000	150.3	127.1	178.0
Equatorial Guinea	Litoral	1203	2005	108.9	91.5	130.2
Equatorial Guinea	Litoral	1203	2010	82.4	69.1	98.4
Equatorial Guinea	Litoral	1203	2015	60.0	49.9	72.8
Equatorial Guinea	Welenzas	1204	2000	162.2	140.5	187.8
Equatorial Guinea	Welenzas	1204	2005	115.3	99.8	133.8
Equatorial Guinea	Welenzas	1204	2010	91.4	78.2	107.1
Equatorial Guinea	Welenzas	1204	2015	66.2	55.1	79.0
Eritrea	Anseba	1205	2000	79.4	71.1	88.7
Eritrea	Anseba	1205	2005	68.6	60.2	77.5
Eritrea	Anseba	1205	2010	58.3	50.6	67.6
Eritrea	Anseba	1205	2015	48.9	42.1	57.0
Eritrea	Archipelagos	1206	2000	113.0	88.9	144.7
Eritrea	Archipelagos	1206	2005	91.8	71.8	117.7
Eritrea	Archipelagos	1206	2010	84.0	65.5	109.8
Eritrea	Archipelagos	1206	2015	68.6	53.3	89.2
Eritrea	Debub	1207	2000	98.6	89.7	108.3
Eritrea	Debub	1207	2005	77.2	68.2	87.3
Eritrea	Debub	1207	2010	66.5	58.1	75.8
Eritrea	Debub	1207	2015	55.2	47.3	64.2
Eritrea	Debubawi Keih Bahri	1208	2000	131.3	116.5	146.8
Eritrea	Debubawi Keih Bahri	1208	2005	103.9	90.7	118.7
Eritrea	Debubawi Keih Bahri	1208	2010	93.8	80.7	107.8
Eritrea	Debubawi Keih Bahri	1208	2015	75.1	63.2	87.3
Eritrea	Gash Barka	1209	2000	98.7	89.6	108.7
Eritrea	Gash Barka	1209	2005	79.1	70.5	88.3
Eritrea	Gash Barka	1209	2010	67.4	59.3	76.4
Eritrea	Gash Barka	1209	2015	55.9	48.3	64.2
Eritrea	Maekel	1210	2000	77.7	69.2	86.6
Eritrea	Maekel	1210	2005	65.4	56.7	74.9

Admin 0	Admin 1	GAUL Code	Year	Under-5 mortality (per 1,000 livebirths)		
				Estimate	Lower bound, 95% UI	Upper bound, 95% UI
Eritrea	Maekel	1210	2010	59.5	50.8	70.1
Eritrea	Maekel	1210	2015	48.7	40.4	58.3
Eritrea	Semenawi Keih Bahri	1211	2000	100.1	90.3	110.8
Eritrea	Semenawi Keih Bahri	1211	2005	82.4	72.9	92.5
Eritrea	Semenawi Keih Bahri	1211	2010	71.2	62.3	81.1
Eritrea	Semenawi Keih Bahri	1211	2015	58.0	50.3	66.5
Ethiopia	Addis Ababa	1227	2000	69.6	62.0	77.8
Ethiopia	Addis Ababa	1227	2005	61.9	54.7	69.9
Ethiopia	Addis Ababa	1227	2010	49.2	42.5	56.6
Ethiopia	Addis Ababa	1227	2015	33.3	28.2	39.2
Ethiopia	Afar	1228	2000	139.8	130.6	149.6
Ethiopia	Afar	1228	2005	110.6	102.8	118.9
Ethiopia	Afar	1228	2010	80.3	73.2	88.1
Ethiopia	Afar	1228	2015	53.4	47.1	60.0
Ethiopia	Amhara	1229	2000	148.4	140.0	156.9
Ethiopia	Amhara	1229	2005	116.5	109.5	123.8
Ethiopia	Amhara	1229	2010	79.6	73.5	86.3
Ethiopia	Amhara	1229	2015	52.3	47.2	57.6
Ethiopia	Beneshangul Gumu	1230	2000	174.9	164.9	185.6
Ethiopia	Beneshangul Gumu	1230	2005	142.4	133.9	151.2
Ethiopia	Beneshangul Gumu	1230	2010	94.3	87.1	102.2
Ethiopia	Beneshangul Gumu	1230	2015	60.6	54.0	67.6
Ethiopia	Dire Dawa	1231	2000	114.0	103.8	125.0
Ethiopia	Dire Dawa	1231	2005	95.3	86.5	105.1
Ethiopia	Dire Dawa	1231	2010	68.8	61.4	77.2
Ethiopia	Dire Dawa	1231	2015	46.0	39.5	53.4
Ethiopia	Gambela	1232	2000	129.7	118.9	141.0
Ethiopia	Gambela	1232	2005	106.9	97.3	117.4
Ethiopia	Gambela	1232	2010	76.7	69.4	85.1
Ethiopia	Gambela	1232	2015	49.7	43.7	56.4
Ethiopia	Hareri	1233	2000	114.6	105.1	125.0
Ethiopia	Hareri	1233	2005	99.2	91.3	107.7
Ethiopia	Hareri	1233	2010	69.4	61.9	77.1
Ethiopia	Hareri	1233	2015	47.7	41.2	54.7
Ethiopia	Oromia	47678	2000	136.7	130.8	143.1
Ethiopia	Oromia	47678	2005	103.7	98.9	108.5
Ethiopia	Oromia	47678	2010	73.2	69.1	77.8
Ethiopia	Oromia	47678	2015	48.2	44.5	52.4
Ethiopia	SNNPR	1236	2000	142.6	134.5	151.0
Ethiopia	SNNPR	1236	2005	107.3	100.6	114.0
Ethiopia	SNNPR	1236	2010	73.6	67.4	80.3
Ethiopia	SNNPR	1236	2015	48.9	44.0	54.6
Ethiopia	Somali	47679	2000	106.9	99.1	115.8
Ethiopia	Somali	47679	2005	94.2	87.5	101.8
Ethiopia	Somali	47679	2010	67.1	61.6	73.2
Ethiopia	Somali	47679	2015	43.3	39.2	47.9
Ethiopia	Tigray	1237	2000	121.3	113.6	129.5
Ethiopia	Tigray	1237	2005	91.9	85.1	98.3
Ethiopia	Tigray	1237	2010	67.6	61.7	73.8
Ethiopia	Tigray	1237	2015	44.8	40.0	50.2
Gabon	Estuaire	1275	2000	63.2	55.9	71.1
Gabon	Estuaire	1275	2005	60.8	54.0	68.5
Gabon	Estuaire	1275	2010	54.3	47.7	61.8
Gabon	Estuaire	1275	2015	44.1	37.4	52.2
Gabon	Haut-Ogooue	1276	2000	71.1	62.1	80.4
Gabon	Haut-Ogooue	1276	2005	64.8	56.7	73.5
Gabon	Haut-Ogooue	1276	2010	51.4	44.6	58.6
Gabon	Haut-Ogooue	1276	2015	42.9	36.6	50.1
Gabon	Moyen-Ogooue	1277	2000	67.5	59.0	77.2
Gabon	Moyen-Ogooue	1277	2005	60.1	52.8	68.3
Gabon	Moyen-Ogooue	1277	2010	50.4	43.8	57.9
Gabon	Moyen-Ogooue	1277	2015	43.6	37.2	50.9
Gabon	Ngounie	1278	2000	73.1	65.1	82.0
Gabon	Ngounie	1278	2005	64.1	57.4	71.4
Gabon	Ngounie	1278	2010	53.7	47.7	60.8
Gabon	Ngounie	1278	2015	46.3	40.3	53.3
Gabon	Nyanga	1279	2000	66.3	58.5	75.5
Gabon	Nyanga	1279	2005	61.5	54.4	70.0
Gabon	Nyanga	1279	2010	48.3	42.3	55.1

Admin 0	Admin 1	GAUL Code	Year	Under-5 mortality (per 1,000 livebirths)		
				Estimate	Lower bound, 95% UI	Upper bound, 95% UI
Gabon	Nyanga	1279	2015	41.4	35.5	47.9
Gabon	Ogooue-Ivindo	1280	2000	97.7	88.1	107.0
Gabon	Ogooue-Ivindo	1280	2005	90.2	81.3	98.9
Gabon	Ogooue-Ivindo	1280	2010	73.4	66.0	81.0
Gabon	Ogooue-Ivindo	1280	2015	64.1	55.9	73.0
Gabon	Ogooue-Maritime	1282	2000	52.5	44.1	62.7
Gabon	Ogooue-Maritime	1282	2005	51.2	42.9	60.9
Gabon	Ogooue-Maritime	1282	2010	44.1	36.4	52.6
Gabon	Ogooue-Maritime	1282	2015	37.1	30.0	44.7
Gabon	Ogooue-lolo	1281	2000	72.0	63.6	81.6
Gabon	Ogooue-lolo	1281	2005	64.1	56.2	71.9
Gabon	Ogooue-lolo	1281	2010	54.0	47.0	62.0
Gabon	Ogooue-lolo	1281	2015	46.1	39.0	53.9
Gabon	Woleu-Ntem	1283	2000	95.9	85.8	106.8
Gabon	Woleu-Ntem	1283	2005	88.3	78.4	98.2
Gabon	Woleu-Ntem	1283	2010	74.7	65.5	84.6
Gabon	Woleu-Ntem	1283	2015	63.9	55.1	73.3
Gambia	Central River	1285	2000	109.9	104.2	115.7
Gambia	Central River	1285	2005	84.4	79.6	89.1
Gambia	Central River	1285	2010	66.9	62.6	71.3
Gambia	Central River	1285	2015	57.6	52.2	63.3
Gambia	Kanifing Municipal Council	112869	2000	54.7	50.3	59.3
Gambia	Kanifing Municipal Council	112869	2005	54.7	50.6	59.2
Gambia	Kanifing Municipal Council	112869	2010	50.3	46.2	54.9
Gambia	Kanifing Municipal Council	112869	2015	42.4	37.8	47.4
Gambia	Lower River	1287	2000	105.6	100.2	111.5
Gambia	Lower River	1287	2005	83.6	79.4	88.2
Gambia	Lower River	1287	2010	62.0	58.2	65.9
Gambia	Lower River	1287	2015	52.6	48.1	57.6
Gambia	North Bank	1288	2000	96.1	91.4	100.8
Gambia	North Bank	1288	2005	78.3	74.7	82.2
Gambia	North Bank	1288	2010	61.5	58.2	65.5
Gambia	North Bank	1288	2015	51.8	47.4	56.7
Gambia	Upper River	1289	2000	138.0	129.7	147.0
Gambia	Upper River	1289	2005	106.0	99.7	112.3
Gambia	Upper River	1289	2010	84.8	78.9	91.3
Gambia	Upper River	1289	2015	74.7	66.9	82.4
Gambia	West Coast	1290	2000	67.5	62.8	72.6
Gambia	West Coast	1290	2005	62.0	58.1	66.2
Gambia	West Coast	1290	2010	54.1	50.0	58.4
Gambia	West Coast	1290	2015	45.8	41.0	50.8
Ghana	Ashanti	1324	2000	87.3	82.4	92.1
Ghana	Ashanti	1324	2005	80.4	76.0	85.3
Ghana	Ashanti	1324	2010	69.0	64.7	74.0
Ghana	Ashanti	1324	2015	49.2	44.1	54.4
Ghana	Brong Ahafo	1325	2000	101.8	96.6	107.2
Ghana	Brong Ahafo	1325	2005	91.0	86.4	95.7
Ghana	Brong Ahafo	1325	2010	72.4	68.0	77.3
Ghana	Brong Ahafo	1325	2015	55.3	50.1	60.8
Ghana	Central	1326	2000	107.6	101.8	114.0
Ghana	Central	1326	2005	91.3	86.5	96.6
Ghana	Central	1326	2010	76.9	71.9	81.9
Ghana	Central	1326	2015	60.0	53.9	66.2
Ghana	Eastern	1327	2000	90.3	85.7	95.2
Ghana	Eastern	1327	2005	78.7	74.5	83.3
Ghana	Eastern	1327	2010	66.8	62.9	70.9
Ghana	Eastern	1327	2015	49.6	44.6	54.8
Ghana	Greater Accra	1328	2000	70.8	66.1	75.9
Ghana	Greater Accra	1328	2005	65.7	61.3	70.2
Ghana	Greater Accra	1328	2010	58.6	54.2	63.3
Ghana	Greater Accra	1328	2015	44.2	39.1	49.6
Ghana	Northern	1329	2000	140.7	135.0	146.9
Ghana	Northern	1329	2005	124.4	118.6	129.8
Ghana	Northern	1329	2010	95.3	90.1	100.6
Ghana	Northern	1329	2015	82.0	75.3	89.3
Ghana	Upper East	1330	2000	122.2	116.2	128.5
Ghana	Upper East	1330	2005	104.4	99.3	110.1
Ghana	Upper East	1330	2010	81.7	76.4	86.9
Ghana	Upper East	1330	2015	60.0	54.5	66.1

Admin 0	Admin 1	GAUL Code	Year	Under-5 mortality (per 1,000 livebirths)		
				Estimate	Lower bound, 95% UI	Upper bound, 95% UI
Ghana	Upper West	1331	2000	145.8	138.7	153.0
Ghana	Upper West	1331	2005	126.5	120.2	133.2
Ghana	Upper West	1331	2010	96.8	90.5	103.4
Ghana	Upper West	1331	2015	76.0	69.0	83.8
Ghana	Volta	1332	2000	92.6	87.9	97.3
Ghana	Volta	1332	2005	81.2	77.1	85.2
Ghana	Volta	1332	2010	66.8	63.0	71.0
Ghana	Volta	1332	2015	53.6	48.6	59.0
Ghana	Western	1333	2000	101.6	95.8	107.7
Ghana	Western	1333	2005	90.4	85.3	95.6
Ghana	Western	1333	2010	71.8	66.8	76.5
Ghana	Western	1333	2015	57.3	51.6	63.2
Guinea	Boke	40700	2000	165.1	154.9	176.3
Guinea	Boke	40700	2005	141.8	132.8	151.8
Guinea	Boke	40700	2010	113.1	104.7	121.8
Guinea	Boke	40700	2015	90.4	81.5	99.1
Guinea	Conakry	40701	2000	95.4	86.4	104.3
Guinea	Conakry	40701	2005	89.4	80.8	98.4
Guinea	Conakry	40701	2010	73.9	66.2	81.8
Guinea	Conakry	40701	2015	51.8	45.3	58.9
Guinea	Faranah	40702	2000	201.8	191.9	213.4
Guinea	Faranah	40702	2005	175.1	165.6	185.5
Guinea	Faranah	40702	2010	153.1	143.2	163.7
Guinea	Faranah	40702	2015	128.1	115.5	141.1
Guinea	Kankan	40703	2000	207.2	196.6	217.8
Guinea	Kankan	40703	2005	192.3	181.6	203.1
Guinea	Kankan	40703	2010	167.2	156.4	177.6
Guinea	Kankan	40703	2015	140.7	127.8	154.1
Guinea	Kindia	40704	2000	161.1	151.6	170.6
Guinea	Kindia	40704	2005	139.8	131.4	148.8
Guinea	Kindia	40704	2010	117.8	109.3	126.5
Guinea	Kindia	40704	2015	100.6	90.5	111.1
Guinea	Labe	40705	2000	177.6	166.6	189.2
Guinea	Labe	40705	2005	150.8	140.8	160.8
Guinea	Labe	40705	2010	124.2	115.0	134.2
Guinea	Labe	40705	2015	103.7	93.0	114.9
Guinea	Mamou	40706	2000	158.6	148.3	169.3
Guinea	Mamou	40706	2005	141.9	132.3	152.0
Guinea	Mamou	40706	2010	120.0	110.8	129.7
Guinea	Mamou	40706	2015	99.9	89.3	110.3
Guinea	Nzerekore	40707	2000	181.5	173.0	190.3
Guinea	Nzerekore	40707	2005	145.2	138.5	153.0
Guinea	Nzerekore	40707	2010	119.8	111.7	128.0
Guinea	Nzerekore	40707	2015	99.2	90.4	108.5
Guinea-Bissau	Bafata	1385	2000	208.7	195.8	222.1
Guinea-Bissau	Bafata	1385	2005	177.6	167.4	188.8
Guinea-Bissau	Bafata	1385	2010	132.1	123.3	141.6
Guinea-Bissau	Bafata	1385	2015	107.2	97.4	117.2
Guinea-Bissau	Biombo	1386	2000	126.3	116.1	137.2
Guinea-Bissau	Biombo	1386	2005	96.5	89.0	104.5
Guinea-Bissau	Biombo	1386	2010	76.8	70.2	83.4
Guinea-Bissau	Biombo	1386	2015	57.9	51.7	64.4
Guinea-Bissau	Bolama/bijagos	1387	2000	115.3	101.3	128.7
Guinea-Bissau	Bolama/bijagos	1387	2005	89.6	79.5	100.5
Guinea-Bissau	Bolama/bijagos	1387	2010	75.2	66.6	85.2
Guinea-Bissau	Bolama/bijagos	1387	2015	59.1	51.4	67.3
Guinea-Bissau	Cacheu	1388	2000	147.5	137.7	157.9
Guinea-Bissau	Cacheu	1388	2005	112.1	104.8	119.8
Guinea-Bissau	Cacheu	1388	2010	85.6	79.2	92.2
Guinea-Bissau	Cacheu	1388	2015	64.9	58.6	71.6
Guinea-Bissau	Gabu	1389	2000	216.3	202.1	231.1
Guinea-Bissau	Gabu	1389	2005	196.0	183.5	209.4
Guinea-Bissau	Gabu	1389	2010	154.3	144.3	166.2
Guinea-Bissau	Gabu	1389	2015	110.1	100.1	121.0
Guinea-Bissau	Oio	1390	2000	167.4	158.1	177.5
Guinea-Bissau	Oio	1390	2005	133.9	126.8	142.3
Guinea-Bissau	Oio	1390	2010	97.5	91.1	103.7
Guinea-Bissau	Oio	1390	2015	74.3	67.6	81.4
Guinea-Bissau	Quinara	1391	2000	145.6	135.5	155.6

Admin 0	Admin 1	GAUL Code	Year	Under-5 mortality (per 1,000 livebirths)		
				Estimate	Lower bound, 95% UI	Upper bound, 95% UI
Guinea-Bissau	Quinara	1391	2005	119.5	111.7	128.0
Guinea-Bissau	Quinara	1391	2010	92.4	85.5	99.3
Guinea-Bissau	Quinara	1391	2015	75.4	68.0	83.4
Guinea-Bissau	Sector Autonomo De Bissau	1392	2000	96.7	89.2	104.7
Guinea-Bissau	Sector Autonomo De Bissau	1392	2005	79.6	73.6	85.8
Guinea-Bissau	Sector Autonomo De Bissau	1392	2010	66.2	60.9	71.5
Guinea-Bissau	Sector Autonomo De Bissau	1392	2015	51.0	45.7	56.7
Guinea-Bissau	Tombali	1393	2000	141.6	129.3	153.8
Guinea-Bissau	Tombali	1393	2005	118.4	108.7	128.5
Guinea-Bissau	Tombali	1393	2010	96.1	87.6	105.2
Guinea-Bissau	Tombali	1393	2015	80.2	71.4	89.0
Kenya	Central	51325	2000	59.6	57.3	62.0
Kenya	Central	51325	2005	50.8	48.4	53.4
Kenya	Central	51325	2010	43.4	40.3	46.7
Kenya	Central	51325	2015	34.4	31.2	37.9
Kenya	Coast	51326	2000	98.1	94.1	102.3
Kenya	Coast	51326	2005	78.0	74.0	82.1
Kenya	Coast	51326	2010	56.4	52.4	60.6
Kenya	Coast	51326	2015	50.4	45.6	55.8
Kenya	Eastern	51327	2000	69.6	67.4	72.0
Kenya	Eastern	51327	2005	56.5	54.3	59.0
Kenya	Eastern	51327	2010	45.3	42.6	47.8
Kenya	Eastern	51327	2015	37.7	34.6	40.9
Kenya	Ilemi triangle	61033	2000	95.6	82.6	110.7
Kenya	Ilemi triangle	61033	2005	84.2	72.1	97.8
Kenya	Ilemi triangle	61033	2010	67.0	56.7	78.5
Kenya	Ilemi triangle	61033	2015	55.3	45.9	65.2
Kenya	Nairobi	51328	2000	64.0	60.4	67.7
Kenya	Nairobi	51328	2005	55.6	52.0	59.6
Kenya	Nairobi	51328	2010	47.9	43.8	52.3
Kenya	Nairobi	51328	2015	39.0	34.6	44.0
Kenya	North Eastern	51329	2000	87.3	83.2	91.8
Kenya	North Eastern	51329	2005	73.6	69.7	77.7
Kenya	North Eastern	51329	2010	54.9	51.2	59.0
Kenya	North Eastern	51329	2015	44.2	40.3	48.5
Kenya	Nyanza	51330	2000	137.8	134.6	141.3
Kenya	Nyanza	51330	2005	113.6	109.9	117.5
Kenya	Nyanza	51330	2010	82.1	78.8	85.9
Kenya	Nyanza	51330	2015	63.4	58.6	68.3
Kenya	Rift Valley	51331	2000	73.0	71.0	75.0
Kenya	Rift Valley	51331	2005	59.4	57.4	61.2
Kenya	Rift Valley	51331	2010	46.6	44.6	48.6
Kenya	Rift Valley	51331	2015	39.9	37.3	42.6
Kenya	Western	51332	2000	117.2	113.7	120.6
Kenya	Western	51332	2005	94.6	91.0	98.1
Kenya	Western	51332	2010	71.1	67.6	74.7
Kenya	Western	51332	2015	53.6	49.2	58.1
Lesotho	Berea	1803	2000	94.5	87.7	102.1
Lesotho	Berea	1803	2005	104.9	97.2	114.0
Lesotho	Berea	1803	2010	86.3	79.2	94.5
Lesotho	Berea	1803	2015	68.8	61.6	76.6
Lesotho	Butha Buthe	1804	2000	94.4	85.7	103.3
Lesotho	Butha Buthe	1804	2005	100.4	91.6	109.9
Lesotho	Butha Buthe	1804	2010	82.3	74.5	91.6
Lesotho	Butha Buthe	1804	2015	66.7	58.4	75.8
Lesotho	Leribe	1805	2000	95.1	88.1	102.8
Lesotho	Leribe	1805	2005	103.7	95.8	112.0
Lesotho	Leribe	1805	2010	85.0	78.1	93.2
Lesotho	Leribe	1805	2015	68.9	61.9	77.5
Lesotho	Mafeteng	1806	2000	95.7	87.5	103.9
Lesotho	Mafeteng	1806	2005	107.9	99.2	117.7
Lesotho	Mafeteng	1806	2010	88.1	80.1	96.6
Lesotho	Mafeteng	1806	2015	70.2	62.3	78.6
Lesotho	Maseru	1807	2000	95.8	88.8	102.5
Lesotho	Maseru	1807	2005	107.7	100.3	116.1
Lesotho	Maseru	1807	2010	87.7	80.6	95.2
Lesotho	Maseru	1807	2015	69.8	62.7	77.5
Lesotho	Mohale's Hoek	1808	2000	108.1	100.2	116.8
Lesotho	Mohale's Hoek	1808	2005	118.7	110.6	128.1

Admin 0	Admin 1	GAUL Code	Year	Under-5 mortality (per 1,000 livebirths)		
				Estimate	Lower bound, 95% UI	Upper bound, 95% UI
Lesotho	Mohale's Hoek	1808	2010	94.9	87.4	103.7
Lesotho	Mohale's Hoek	1808	2015	74.2	66.3	82.4
Lesotho	Mokhotlong	1809	2000	105.4	97.1	115.1
Lesotho	Mokhotlong	1809	2005	107.4	99.2	117.1
Lesotho	Mokhotlong	1809	2010	87.1	79.1	96.0
Lesotho	Mokhotlong	1809	2015	68.0	59.7	77.5
Lesotho	Qacha's Nek	1810	2000	101.0	91.5	109.9
Lesotho	Qacha's Nek	1810	2005	109.0	99.7	118.9
Lesotho	Qacha's Nek	1810	2010	88.4	80.1	98.4
Lesotho	Qacha's Nek	1810	2015	67.6	59.2	76.4
Lesotho	Quthing	1811	2000	108.5	98.8	119.2
Lesotho	Quthing	1811	2005	119.0	108.4	130.8
Lesotho	Quthing	1811	2010	94.9	85.4	105.8
Lesotho	Quthing	1811	2015	73.6	64.7	83.4
Lesotho	Thaba Tseka	1812	2000	108.7	100.9	117.0
Lesotho	Thaba Tseka	1812	2005	114.6	106.9	123.1
Lesotho	Thaba Tseka	1812	2010	92.7	85.1	101.1
Lesotho	Thaba Tseka	1812	2015	71.8	64.5	79.9
Liberia	Bomi	1814	2000	210.5	198.3	221.9
Liberia	Bomi	1814	2005	146.6	138.2	155.2
Liberia	Bomi	1814	2010	111.2	104.0	118.9
Liberia	Bomi	1814	2015	87.5	78.3	97.1
Liberia	Bong	1815	2000	175.1	165.1	185.6
Liberia	Bong	1815	2005	121.1	114.1	128.4
Liberia	Bong	1815	2010	93.3	86.7	100.1
Liberia	Bong	1815	2015	71.1	63.9	79.1
Liberia	Gbarpolu	1816	2000	198.6	186.3	211.0
Liberia	Gbarpolu	1816	2005	138.5	130.4	147.3
Liberia	Gbarpolu	1816	2010	106.4	99.3	114.0
Liberia	Gbarpolu	1816	2015	81.1	72.5	90.3
Liberia	Grand Bassa	1817	2000	191.1	179.7	202.5
Liberia	Grand Bassa	1817	2005	135.2	127.4	143.9
Liberia	Grand Bassa	1817	2010	102.4	94.8	109.9
Liberia	Grand Bassa	1817	2015	82.9	74.1	92.2
Liberia	Grand Cape Mount	1818	2000	245.6	233.1	259.0
Liberia	Grand Cape Mount	1818	2005	178.1	169.0	188.0
Liberia	Grand Cape Mount	1818	2010	138.4	129.3	147.8
Liberia	Grand Cape Mount	1818	2015	111.4	100.7	124.1
Liberia	Grand Gedeh	1819	2000	158.0	147.4	169.2
Liberia	Grand Gedeh	1819	2005	121.7	113.7	130.3
Liberia	Grand Gedeh	1819	2010	89.6	82.4	97.6
Liberia	Grand Gedeh	1819	2015	69.1	61.5	78.3
Liberia	Grand Kru	1820	2000	151.1	139.7	162.6
Liberia	Grand Kru	1820	2005	120.6	112.6	128.7
Liberia	Grand Kru	1820	2010	94.1	86.6	101.7
Liberia	Grand Kru	1820	2015	78.9	69.8	89.6
Liberia	Lofa	1821	2000	184.2	173.2	195.6
Liberia	Lofa	1821	2005	130.7	123.4	139.2
Liberia	Lofa	1821	2010	98.1	91.4	105.5
Liberia	Lofa	1821	2015	76.0	68.3	84.0
Liberia	Margibi	1822	2000	181.8	172.1	190.9
Liberia	Margibi	1822	2005	123.0	116.6	130.0
Liberia	Margibi	1822	2010	93.6	87.2	99.9
Liberia	Margibi	1822	2015	73.6	66.4	81.9
Liberia	Maryland	1823	2000	135.7	126.2	146.2
Liberia	Maryland	1823	2005	110.8	103.6	119.3
Liberia	Maryland	1823	2010	83.1	76.2	90.5
Liberia	Maryland	1823	2015	67.6	59.4	76.8
Liberia	Montserrado	1824	2000	159.3	150.4	168.5
Liberia	Montserrado	1824	2005	115.0	108.2	121.4
Liberia	Montserrado	1824	2010	87.5	81.4	94.0
Liberia	Montserrado	1824	2015	64.8	57.4	72.5
Liberia	Nimba	1825	2000	152.6	143.2	163.1
Liberia	Nimba	1825	2005	108.8	102.0	116.4
Liberia	Nimba	1825	2010	79.3	72.7	85.3
Liberia	Nimba	1825	2015	61.0	54.1	68.2
Liberia	River Gee	1827	2000	161.5	150.3	172.7
Liberia	River Gee	1827	2005	127.1	119.8	135.7
Liberia	River Gee	1827	2010	96.1	88.4	104.5

Admin 0	Admin 1	GAUL Code	Year	Under-5 mortality (per 1,000 livebirths)		
				Estimate	Lower bound, 95% UI	Upper bound, 95% UI
Liberia	River Gee	1827	2015	81.6	72.2	92.0
Liberia	Rivercess	1826	2000	179.1	166.7	191.8
Liberia	Rivercess	1826	2005	131.6	123.0	141.3
Liberia	Rivercess	1826	2010	99.8	92.4	108.3
Liberia	Rivercess	1826	2015	81.5	72.5	91.6
Liberia	Sinoe	1828	2000	158.7	146.9	170.1
Liberia	Sinoe	1828	2005	122.9	114.6	131.8
Liberia	Sinoe	1828	2010	99.1	90.8	107.8
Liberia	Sinoe	1828	2015	80.7	71.5	90.5
Madagascar	Alaotra Mangoro	41750	2000	111.7	102.8	120.9
Madagascar	Alaotra Mangoro	41750	2005	99.9	92.2	108.2
Madagascar	Alaotra Mangoro	41750	2010	91.3	83.7	99.7
Madagascar	Alaotra Mangoro	41750	2015	76.6	68.3	85.4
Madagascar	Amoron I Mania	41751	2000	113.7	105.1	122.5
Madagascar	Amoron I Mania	41751	2005	104.9	97.2	113.1
Madagascar	Amoron I Mania	41751	2010	99.5	91.1	108.7
Madagascar	Amoron I Mania	41751	2015	86.3	76.0	96.5
Madagascar	Analamanga	41752	2000	77.5	71.4	84.2
Madagascar	Analamanga	41752	2005	74.7	69.1	80.8
Madagascar	Analamanga	41752	2010	75.7	69.0	83.1
Madagascar	Analamanga	41752	2015	68.4	60.3	77.3
Madagascar	Analanjirofo	41753	2000	117.9	106.6	129.4
Madagascar	Analanjirofo	41753	2005	96.6	87.8	107.2
Madagascar	Analanjirofo	41753	2010	85.4	75.9	95.4
Madagascar	Analanjirofo	41753	2015	71.3	62.1	80.8
Madagascar	Androy	41754	2000	115.3	107.0	123.5
Madagascar	Androy	41754	2005	97.1	90.7	104.1
Madagascar	Androy	41754	2010	91.2	84.2	98.5
Madagascar	Androy	41754	2015	78.6	70.2	88.2
Madagascar	Anosy	41755	2000	140.2	130.1	151.3
Madagascar	Anosy	41755	2005	125.3	117.0	134.3
Madagascar	Anosy	41755	2010	114.8	105.9	124.5
Madagascar	Anosy	41755	2015	103.4	92.2	116.5
Madagascar	Atsimo Andrefana	41756	2000	115.3	106.7	124.3
Madagascar	Atsimo Andrefana	41756	2005	96.4	89.5	103.5
Madagascar	Atsimo Andrefana	41756	2010	86.7	79.9	94.4
Madagascar	Atsimo Andrefana	41756	2015	80.4	72.3	89.5
Madagascar	Atsimo Atsinanana	41757	2000	171.5	158.8	185.1
Madagascar	Atsimo Atsinanana	41757	2005	151.3	141.1	161.8
Madagascar	Atsimo Atsinanana	41757	2010	139.2	128.6	151.0
Madagascar	Atsimo Atsinanana	41757	2015	123.9	110.9	138.3
Madagascar	Atsinanana	41758	2000	122.8	113.4	132.5
Madagascar	Atsinanana	41758	2005	110.0	100.9	119.1
Madagascar	Atsinanana	41758	2010	102.7	93.3	112.7
Madagascar	Atsinanana	41758	2015	85.6	76.1	94.7
Madagascar	Betsiboka	41759	2000	121.9	109.7	135.2
Madagascar	Betsiboka	41759	2005	105.1	95.3	116.9
Madagascar	Betsiboka	41759	2010	97.9	86.7	109.5
Madagascar	Betsiboka	41759	2015	87.2	75.7	100.0
Madagascar	Boeny	41760	2000	119.8	107.9	134.0
Madagascar	Boeny	41760	2005	101.2	91.8	112.3
Madagascar	Boeny	41760	2010	92.0	81.4	103.4
Madagascar	Boeny	41760	2015	81.9	71.3	93.8
Madagascar	Bongolava	41761	2000	102.4	91.1	113.5
Madagascar	Bongolava	41761	2005	92.9	83.9	103.1
Madagascar	Bongolava	41761	2010	86.0	77.3	96.2
Madagascar	Bongolava	41761	2015	79.6	69.9	90.3
Madagascar	Diana	41762	2000	98.2	87.5	110.5
Madagascar	Diana	41762	2005	82.9	73.3	93.5
Madagascar	Diana	41762	2010	75.2	65.8	85.9
Madagascar	Diana	41762	2015	70.7	61.1	82.0
Madagascar	Haute Matsiatra	41763	2000	128.7	119.3	139.8
Madagascar	Haute Matsiatra	41763	2005	115.3	107.2	124.1
Madagascar	Haute Matsiatra	41763	2010	108.7	99.7	118.9
Madagascar	Haute Matsiatra	41763	2015	93.2	82.8	104.5
Madagascar	Ihorombe	41764	2000	134.7	122.7	148.2
Madagascar	Ihorombe	41764	2005	118.0	107.5	129.9
Madagascar	Ihorombe	41764	2010	109.4	99.5	120.8
Madagascar	Ihorombe	41764	2015	93.3	82.6	105.5

Admin 0	Admin 1	GAUL Code	Year	Under-5 mortality (per 1,000 livebirths)		
				Estimate	Lower bound, 95% UI	Upper bound, 95% UI
Madagascar	Itasy	41765	2000	93.2	85.8	101.1
Madagascar	Itasy	41765	2005	85.1	78.8	91.8
Madagascar	Itasy	41765	2010	85.7	78.2	94.0
Madagascar	Itasy	41765	2015	75.8	67.2	85.4
Madagascar	Melaky	41766	2000	114.5	100.6	129.0
Madagascar	Melaky	41766	2005	96.7	85.1	108.5
Madagascar	Melaky	41766	2010	86.0	74.9	97.4
Madagascar	Melaky	41766	2015	86.6	74.0	99.4
Madagascar	Menabe	41767	2000	122.7	110.2	136.3
Madagascar	Menabe	41767	2005	103.3	92.6	114.6
Madagascar	Menabe	41767	2010	91.1	81.1	101.5
Madagascar	Menabe	41767	2015	85.3	75.1	96.3
Madagascar	Sava	41768	2000	97.2	87.0	107.8
Madagascar	Sava	41768	2005	83.6	75.0	93.0
Madagascar	Sava	41768	2010	75.4	67.2	85.3
Madagascar	Sava	41768	2015	70.6	61.4	81.1
Madagascar	Sofia	41769	2000	106.5	96.8	116.4
Madagascar	Sofia	41769	2005	88.3	80.9	96.2
Madagascar	Sofia	41769	2010	78.9	70.5	87.0
Madagascar	Sofia	41769	2015	74.2	64.2	83.7
Madagascar	Vakinankaratra	41770	2000	98.8	91.1	107.1
Madagascar	Vakinankaratra	41770	2005	90.2	82.8	97.8
Madagascar	Vakinankaratra	41770	2010	89.9	82.0	99.1
Madagascar	Vakinankaratra	41770	2015	78.4	69.4	89.2
Madagascar	Vatovavy Fitovinany	41771	2000	173.0	159.6	186.0
Madagascar	Vatovavy Fitovinany	41771	2005	150.9	140.4	163.0
Madagascar	Vatovavy Fitovinany	41771	2010	138.9	127.3	152.0
Madagascar	Vatovavy Fitovinany	41771	2015	118.8	106.4	132.9
Malawi	Area under National Administration	65268	2000	142.6	137.9	147.2
Malawi	Area under National Administration	65268	2005	110.3	106.4	114.1
Malawi	Area under National Administration	65268	2010	87.5	84.2	91.1
Malawi	Area under National Administration	65268	2015	65.3	61.3	69.4
Malawi	Central Region	1888	2000	169.1	165.4	172.9
Malawi	Central Region	1888	2005	129.3	125.9	132.9
Malawi	Central Region	1888	2010	103.0	99.5	106.6
Malawi	Central Region	1888	2015	78.4	73.6	83.5
Malawi	Northern Region	1889	2000	133.7	129.3	138.6
Malawi	Northern Region	1889	2005	104.4	100.6	108.2
Malawi	Northern Region	1889	2010	84.4	80.4	88.3
Malawi	Northern Region	1889	2015	68.0	63.2	73.1
Malawi	Southern Region	1890	2000	170.9	167.6	174.2
Malawi	Southern Region	1890	2005	128.3	125.3	131.1
Malawi	Southern Region	1890	2010	100.8	97.8	103.8
Malawi	Southern Region	1890	2015	75.3	71.0	80.1
Mali	Bamako	1926	2000	111.6	105.7	118.2
Mali	Bamako	1926	2005	97.5	92.1	103.3
Mali	Bamako	1926	2010	83.0	77.4	89.1
Mali	Bamako	1926	2015	71.9	65.4	79.1
Mali	Gao	1927	2000	147.7	137.3	159.1
Mali	Gao	1927	2005	136.8	126.2	147.6
Mali	Gao	1927	2010	132.8	120.8	145.6
Mali	Gao	1927	2015	116.3	103.9	130.8
Mali	Kayes	1928	2000	205.5	198.2	213.5
Mali	Kayes	1928	2005	166.2	159.1	172.9
Mali	Kayes	1928	2010	144.4	137.3	152.0
Mali	Kayes	1928	2015	129.1	119.7	140.2
Mali	Kidal	1929	2000	100.6	86.5	114.2
Mali	Kidal	1929	2005	87.6	74.5	101.3
Mali	Kidal	1929	2010	80.2	67.6	92.9
Mali	Kidal	1929	2015	80.4	67.2	94.9
Mali	Koulikoro	1930	2000	179.7	174.0	185.7
Mali	Koulikoro	1930	2005	156.3	150.8	161.8
Mali	Koulikoro	1930	2010	131.2	125.6	136.7
Mali	Koulikoro	1930	2015	118.0	110.0	126.6
Mali	Mopti	1931	2000	208.0	200.9	214.8
Mali	Mopti	1931	2005	174.5	167.8	181.8
Mali	Mopti	1931	2010	149.9	142.8	156.6
Mali	Mopti	1931	2015	129.9	120.0	140.7
Mali	Segou	1932	2000	216.0	209.0	224.0

Admin 0	Admin 1	GAUL Code	Year	Under-5 mortality (per 1,000 livebirths)		
				Estimate	Lower bound, 95% UI	Upper bound, 95% UI
Mali	Segou	1932	2005	189.4	181.9	196.8
Mali	Segou	1932	2010	154.5	147.1	162.0
Mali	Segou	1932	2015	135.2	124.6	145.7
Mali	Sikasso	1933	2000	211.8	205.5	218.3
Mali	Sikasso	1933	2005	190.8	184.1	197.3
Mali	Sikasso	1933	2010	160.0	152.9	166.9
Mali	Sikasso	1933	2015	137.0	127.0	147.3
Mali	Tombouctou	1934	2000	207.7	196.4	220.2
Mali	Tombouctou	1934	2005	176.6	165.4	187.9
Mali	Tombouctou	1934	2010	159.9	147.1	172.3
Mali	Tombouctou	1934	2015	141.0	126.5	156.9
Mauritania	Adrar	2004	2000	89.0	79.3	100.2
Mauritania	Adrar	2004	2005	89.6	79.7	100.2
Mauritania	Adrar	2004	2010	73.0	63.8	82.7
Mauritania	Adrar	2004	2015	58.8	50.5	67.9
Mauritania	Assaba	2005	2000	87.5	81.5	93.3
Mauritania	Assaba	2005	2005	77.3	71.9	82.6
Mauritania	Assaba	2005	2010	64.5	59.0	70.4
Mauritania	Assaba	2005	2015	53.6	47.5	60.3
Mauritania	Brakna	2006	2000	102.7	96.1	109.8
Mauritania	Brakna	2006	2005	84.7	79.3	90.6
Mauritania	Brakna	2006	2010	62.7	57.5	68.4
Mauritania	Brakna	2006	2015	48.1	42.4	53.6
Mauritania	Dakhlet-Nouadhibou	2007	2000	78.3	68.2	88.4
Mauritania	Dakhlet-Nouadhibou	2007	2005	76.8	67.1	87.2
Mauritania	Dakhlet-Nouadhibou	2007	2010	61.6	53.4	70.5
Mauritania	Dakhlet-Nouadhibou	2007	2015	49.3	42.0	57.1
Mauritania	Gorgol	2008	2000	105.1	98.9	111.9
Mauritania	Gorgol	2008	2005	86.9	81.6	92.4
Mauritania	Gorgol	2008	2010	69.2	63.6	75.2
Mauritania	Gorgol	2008	2015	57.9	51.8	65.1
Mauritania	Guidimakha	2009	2000	108.1	101.9	115.8
Mauritania	Guidimakha	2009	2005	92.2	86.7	98.4
Mauritania	Guidimakha	2009	2010	72.3	66.8	78.3
Mauritania	Guidimakha	2009	2015	60.0	53.6	67.3
Mauritania	Hodh Ech Chargi	2010	2000	105.5	98.2	113.4
Mauritania	Hodh Ech Chargi	2010	2005	98.2	91.0	106.1
Mauritania	Hodh Ech Chargi	2010	2010	84.8	77.3	92.6
Mauritania	Hodh Ech Chargi	2010	2015	74.3	66.4	83.4
Mauritania	Hodh El Gharbi	2011	2000	81.4	75.7	88.3
Mauritania	Hodh El Gharbi	2011	2005	74.1	68.8	80.0
Mauritania	Hodh El Gharbi	2011	2010	63.1	57.3	68.9
Mauritania	Hodh El Gharbi	2011	2015	52.6	46.6	58.9
Mauritania	Inchiri	2012	2000	84.9	72.3	98.2
Mauritania	Inchiri	2012	2005	84.6	72.2	97.7
Mauritania	Inchiri	2012	2010	71.1	60.2	83.6
Mauritania	Inchiri	2012	2015	58.1	48.4	68.2
Mauritania	Nouakchott	2013	2000	81.1	74.3	88.8
Mauritania	Nouakchott	2013	2005	76.1	68.9	83.5
Mauritania	Nouakchott	2013	2010	63.2	55.9	70.9
Mauritania	Nouakchott	2013	2015	49.1	42.2	56.8
Mauritania	Tagant	2014	2000	73.0	65.4	81.7
Mauritania	Tagant	2014	2005	71.3	63.9	79.1
Mauritania	Tagant	2014	2010	60.8	53.3	68.7
Mauritania	Tagant	2014	2015	49.0	42.6	56.7
Mauritania	Tiris-Zemmour	2015	2000	81.5	69.7	95.7
Mauritania	Tiris-Zemmour	2015	2005	83.5	71.1	97.2
Mauritania	Tiris-Zemmour	2015	2010	69.4	57.5	81.9
Mauritania	Tiris-Zemmour	2015	2015	58.9	48.1	71.0
Mauritania	Trarza	2016	2000	84.8	79.7	90.4
Mauritania	Trarza	2016	2005	75.1	70.3	80.3
Mauritania	Trarza	2016	2010	59.4	54.7	64.4
Mauritania	Trarza	2016	2015	44.4	39.7	49.6
Morocco	Chaouia - Ouardigha	147326	2000	40.7	36.3	46.1
Morocco	Chaouia - Ouardigha	147326	2005	32.6	28.9	36.6
Morocco	Chaouia - Ouardigha	147326	2010	27.4	23.9	31.3
Morocco	Chaouia - Ouardigha	147326	2015	22.2	18.9	26.4
Morocco	Doukkala - Abda	147327	2000	44.3	38.3	51.5
Morocco	Doukkala - Abda	147327	2005	36.0	30.7	41.7

Admin 0	Admin 1	GAUL Code	Year	Under-5 mortality (per 1,000 livebirths)		
				Estimate	Lower bound, 95% UI	Upper bound, 95% UI
Morocco	Doukkala - Abda	147327	2010	28.3	23.7	33.1
Morocco	Doukkala - Abda	147327	2015	22.8	18.9	27.2
Morocco	Fès - Boulemane	147328	2000	47.8	41.9	54.3
Morocco	Fès - Boulemane	147328	2005	39.2	34.4	44.8
Morocco	Fès - Boulemane	147328	2010	31.0	26.3	36.1
Morocco	Fès - Boulemane	147328	2015	24.7	20.5	29.5
Morocco	Gharb - Chrarda - Béni Hssen	147329	2000	45.8	40.4	51.8
Morocco	Gharb - Chrarda - Béni Hssen	147329	2005	36.3	31.5	41.3
Morocco	Gharb - Chrarda - Béni Hssen	147329	2010	28.3	24.2	32.8
Morocco	Gharb - Chrarda - Béni Hssen	147329	2015	22.8	18.9	26.9
Morocco	Grand Casablanca	147330	2000	32.6	27.8	37.7
Morocco	Grand Casablanca	147330	2005	27.8	23.9	32.2
Morocco	Grand Casablanca	147330	2010	24.5	20.2	28.9
Morocco	Grand Casablanca	147330	2015	19.9	16.4	24.2
Morocco	Guelmim - Es-Semara	147331	2000	53.4	46.3	61.4
Morocco	Guelmim - Es-Semara	147331	2005	43.3	37.2	49.9
Morocco	Guelmim - Es-Semara	147331	2010	34.1	28.9	39.6
Morocco	Guelmim - Es-Semara	147331	2015	26.8	22.6	31.9
Morocco	Laâyoune - Boujdour - Sakia El Hamra	147332	2000	54.1	42.3	68.8
Morocco	Laâyoune - Boujdour - Sakia El Hamra	147332	2005	46.2	36.5	58.9
Morocco	Laâyoune - Boujdour - Sakia El Hamra	147332	2010	41.1	32.2	51.5
Morocco	Laâyoune - Boujdour - Sakia El Hamra	147332	2015	31.0	24.1	39.6
Morocco	Marrakech - Tensift - Al Haouz	147333	2000	49.0	43.4	55.2
Morocco	Marrakech - Tensift - Al Haouz	147333	2005	40.7	35.8	46.3
Morocco	Marrakech - Tensift - Al Haouz	147333	2010	30.3	25.9	35.6
Morocco	Marrakech - Tensift - Al Haouz	147333	2015	24.1	20.3	28.4
Morocco	Meknès - Tafilalet	2107	2000	51.6	45.7	58.1
Morocco	Meknès - Tafilalet	2107	2005	41.2	36.4	46.5
Morocco	Meknès - Tafilalet	2107	2010	32.3	27.9	37.0
Morocco	Meknès - Tafilalet	2107	2015	25.9	22.0	30.2
Morocco	Oriental	2109	2000	46.2	40.1	52.6
Morocco	Oriental	2109	2005	35.9	30.9	41.0
Morocco	Oriental	2109	2010	29.9	25.3	35.0
Morocco	Oriental	2109	2015	23.4	19.4	27.7
Morocco	Rabat - Salé - Zemmour - Zaer	147334	2000	34.7	30.4	39.5
Morocco	Rabat - Salé - Zemmour - Zaer	147334	2005	29.3	25.6	33.5
Morocco	Rabat - Salé - Zemmour - Zaer	147334	2010	25.4	21.5	29.5
Morocco	Rabat - Salé - Zemmour - Zaer	147334	2015	20.5	16.9	24.3
Morocco	Souss - Massa - Draâ	147335	2000	53.4	47.5	59.8
Morocco	Souss - Massa - Draâ	147335	2005	44.0	39.2	49.2
Morocco	Souss - Massa - Draâ	147335	2010	32.1	28.2	36.9
Morocco	Souss - Massa - Draâ	147335	2015	25.4	21.8	29.9
Morocco	Tadla - Azilal	147336	2000	49.7	43.1	57.1
Morocco	Tadla - Azilal	147336	2005	39.5	33.8	45.3
Morocco	Tadla - Azilal	147336	2010	29.4	24.5	34.5
Morocco	Tadla - Azilal	147336	2015	24.4	20.1	29.2
Morocco	Tanger - Tétouan	147337	2000	52.0	45.4	59.4
Morocco	Tanger - Tétouan	147337	2005	40.9	35.2	47.0
Morocco	Tanger - Tétouan	147337	2010	33.3	27.9	38.8
Morocco	Tanger - Tétouan	147337	2015	26.1	21.5	31.1
Morocco	Taza - Al Hoceima - Taounate	147338	2000	53.4	46.6	60.3
Morocco	Taza - Al Hoceima - Taounate	147338	2005	42.5	37.6	48.2
Morocco	Taza - Al Hoceima - Taounate	147338	2010	32.0	27.8	37.0
Morocco	Taza - Al Hoceima - Taounate	147338	2015	25.3	21.4	30.0
Mozambique	Cabo Delgado	2112	2000	186.2	177.2	195.2
Mozambique	Cabo Delgado	2112	2005	141.5	134.5	148.6
Mozambique	Cabo Delgado	2112	2010	107.8	99.8	116.0
Mozambique	Cabo Delgado	2112	2015	89.9	80.7	99.5
Mozambique	Gaza	2113	2000	137.3	129.3	144.2
Mozambique	Gaza	2113	2005	116.1	109.7	122.7
Mozambique	Gaza	2113	2010	89.8	82.3	97.3
Mozambique	Gaza	2113	2015	76.1	68.1	84.8
Mozambique	Inhambane	2114	2000	127.8	120.0	136.5
Mozambique	Inhambane	2114	2005	111.3	104.8	118.5
Mozambique	Inhambane	2114	2010	91.7	84.1	99.3
Mozambique	Inhambane	2114	2015	72.8	65.0	81.1
Mozambique	Manica	2115	2000	151.6	144.6	158.7
Mozambique	Manica	2115	2005	136.2	130.0	142.7
Mozambique	Manica	2115	2010	106.9	100.5	113.9

Admin 0	Admin 1	GAUL Code	Year	Under-5 mortality (per 1,000 livebirths)		
				Estimate	Lower bound, 95% UI	Upper bound, 95% UI
Mozambique	Manica	2115	2015	82.6	75.4	90.5
Mozambique	Maputo	41373	2000	106.3	100.9	112.0
Mozambique	Maputo	41373	2005	89.0	84.2	94.1
Mozambique	Maputo	41373	2010	73.9	68.1	80.1
Mozambique	Maputo	41373	2015	58.1	51.5	65.6
Mozambique	Nampula	2118	2000	167.8	160.2	175.3
Mozambique	Nampula	2118	2005	133.9	127.8	140.3
Mozambique	Nampula	2118	2010	105.7	98.2	113.5
Mozambique	Nampula	2118	2015	86.6	77.8	96.0
Mozambique	Niassa	2119	2000	148.3	141.1	155.7
Mozambique	Niassa	2119	2005	112.1	106.6	118.1
Mozambique	Niassa	2119	2010	90.5	84.9	96.3
Mozambique	Niassa	2119	2015	68.4	63.2	74.6
Mozambique	Sofala	2120	2000	160.4	153.7	167.7
Mozambique	Sofala	2120	2005	128.7	122.6	135.0
Mozambique	Sofala	2120	2010	101.6	94.9	108.4
Mozambique	Sofala	2120	2015	80.6	73.0	88.5
Mozambique	Tete	2121	2000	164.2	158.4	170.5
Mozambique	Tete	2121	2005	129.4	124.6	134.7
Mozambique	Tete	2121	2010	106.3	100.7	111.9
Mozambique	Tete	2121	2015	80.0	74.3	86.3
Mozambique	Zambezia	2122	2000	190.6	182.6	197.9
Mozambique	Zambezia	2122	2005	151.4	145.5	158.7
Mozambique	Zambezia	2122	2010	122.0	114.8	129.3
Mozambique	Zambezia	2122	2015	97.7	90.0	105.7
Namibia	Caprivi	2137	2000	81.4	72.6	91.1
Namibia	Caprivi	2137	2005	77.7	68.9	87.0
Namibia	Caprivi	2137	2010	68.8	60.6	77.7
Namibia	Caprivi	2137	2015	50.4	43.2	58.7
Namibia	Erongo	2138	2000	46.2	38.9	54.4
Namibia	Erongo	2138	2005	50.0	42.4	59.3
Namibia	Erongo	2138	2010	44.8	37.7	53.2
Namibia	Erongo	2138	2015	32.5	26.7	39.3
Namibia	Hardap	2139	2000	49.8	43.1	58.0
Namibia	Hardap	2139	2005	51.2	43.8	59.5
Namibia	Hardap	2139	2010	44.2	37.8	51.8
Namibia	Hardap	2139	2015	34.2	28.5	40.4
Namibia	Karas	2140	2000	46.2	39.8	53.3
Namibia	Karas	2140	2005	49.8	42.8	57.5
Namibia	Karas	2140	2010	44.7	38.7	52.1
Namibia	Karas	2140	2015	34.6	29.4	40.7
Namibia	Kavango	2141	2000	76.4	68.0	85.7
Namibia	Kavango	2141	2005	74.4	65.9	83.3
Namibia	Kavango	2141	2010	59.3	52.5	67.0
Namibia	Kavango	2141	2015	45.8	39.6	53.2
Namibia	Khomas	2142	2000	42.8	36.6	49.6
Namibia	Khomas	2142	2005	45.0	38.8	52.8
Namibia	Khomas	2142	2010	39.9	33.9	47.1
Namibia	Khomas	2142	2015	30.1	24.9	36.1
Namibia	Kunene	2143	2000	62.3	54.8	70.9
Namibia	Kunene	2143	2005	60.1	53.4	68.1
Namibia	Kunene	2143	2010	51.2	45.1	58.2
Namibia	Kunene	2143	2015	38.0	32.8	43.8
Namibia	Ohangwena	2144	2000	78.4	70.9	86.8
Namibia	Ohangwena	2144	2005	81.2	73.1	89.6
Namibia	Ohangwena	2144	2010	64.9	57.6	72.4
Namibia	Ohangwena	2144	2015	48.6	41.9	55.6
Namibia	Omaheke	2145	2000	64.3	56.5	73.5
Namibia	Omaheke	2145	2005	57.5	50.5	65.5
Namibia	Omaheke	2145	2010	49.5	42.7	56.8
Namibia	Omaheke	2145	2015	39.2	33.5	45.7
Namibia	Omusati	2146	2000	64.0	56.5	71.9
Namibia	Omusati	2146	2005	66.7	58.9	74.9
Namibia	Omusati	2146	2010	57.1	49.6	65.3
Namibia	Omusati	2146	2015	40.7	35.1	47.3
Namibia	Oshana	2147	2000	62.8	56.3	69.8
Namibia	Oshana	2147	2005	66.8	60.0	74.1
Namibia	Oshana	2147	2010	55.8	49.0	63.1
Namibia	Oshana	2147	2015	41.1	35.2	47.4

Admin 0	Admin 1	GAUL Code	Year	Under-5 mortality (per 1,000 livebirths)		
				Estimate	Lower bound, 95% UI	Upper bound, 95% UI
Namibia	Oshikoto	2148	2000	69.9	62.4	77.5
Namibia	Oshikoto	2148	2005	71.1	63.3	79.0
Namibia	Oshikoto	2148	2010	59.0	52.3	66.3
Namibia	Oshikoto	2148	2015	43.3	37.2	49.8
Namibia	Otjozondjupa	2149	2000	56.0	49.5	62.9
Namibia	Otjozondjupa	2149	2005	56.0	49.4	63.2
Namibia	Otjozondjupa	2149	2010	47.7	42.2	54.2
Namibia	Otjozondjupa	2149	2015	35.0	30.5	40.5
Niger	Agadez	2202	2000	95.9	86.1	106.5
Niger	Agadez	2202	2005	82.8	74.1	91.8
Niger	Agadez	2202	2010	74.0	65.6	82.6
Niger	Agadez	2202	2015	66.6	58.3	75.8
Niger	Diffa	2203	2000	149.6	137.2	164.1
Niger	Diffa	2203	2005	116.5	105.5	129.2
Niger	Diffa	2203	2010	108.9	97.1	121.3
Niger	Diffa	2203	2015	87.6	77.2	100.3
Niger	Dosso	2204	2000	225.8	215.2	237.5
Niger	Dosso	2204	2005	193.6	184.5	203.3
Niger	Dosso	2204	2010	159.6	149.2	170.3
Niger	Dosso	2204	2015	136.3	123.1	149.8
Niger	Maradi	2205	2000	260.2	249.5	271.4
Niger	Maradi	2205	2005	194.8	186.1	203.9
Niger	Maradi	2205	2010	140.4	132.4	148.9
Niger	Maradi	2205	2015	117.4	106.3	129.4
Niger	Niamey	2206	2000	125.0	115.3	134.5
Niger	Niamey	2206	2005	108.7	100.8	116.7
Niger	Niamey	2206	2010	95.7	86.7	104.6
Niger	Niamey	2206	2015	78.5	69.1	88.2
Niger	Tahoua	2207	2000	226.4	214.9	237.8
Niger	Tahoua	2207	2005	176.5	167.2	186.3
Niger	Tahoua	2207	2010	125.7	117.1	135.3
Niger	Tahoua	2207	2015	113.6	102.2	125.4
Niger	Tillaberi	2208	2000	212.0	202.3	222.4
Niger	Tillaberi	2208	2005	183.3	174.3	192.4
Niger	Tillaberi	2208	2010	164.5	155.2	175.0
Niger	Tillaberi	2208	2015	124.2	112.9	135.8
Niger	Zinder	2209	2000	245.3	233.4	257.2
Niger	Zinder	2209	2005	190.7	180.5	200.3
Niger	Zinder	2209	2010	148.2	137.8	158.4
Niger	Zinder	2209	2015	115.5	103.9	127.0
Nigeria	Abia	65698	2000	141.7	133.1	150.7
Nigeria	Abia	65698	2005	137.3	128.9	145.5
Nigeria	Abia	65698	2010	112.5	104.7	120.6
Nigeria	Abia	65698	2015	82.6	74.3	92.7
Nigeria	Abuja	2221	2000	120.5	110.8	129.9
Nigeria	Abuja	2221	2005	106.4	98.0	114.8
Nigeria	Abuja	2221	2010	92.7	84.6	102.1
Nigeria	Abuja	2221	2015	75.6	66.7	85.2
Nigeria	Adamawa	2211	2000	223.2	211.9	234.6
Nigeria	Adamawa	2211	2005	197.2	187.4	207.6
Nigeria	Adamawa	2211	2010	159.5	149.6	169.4
Nigeria	Adamawa	2211	2015	137.1	125.0	150.6
Nigeria	Akwa Ibom	2212	2000	134.8	124.8	145.4
Nigeria	Akwa Ibom	2212	2005	133.0	123.9	142.9
Nigeria	Akwa Ibom	2212	2010	108.5	99.9	118.5
Nigeria	Akwa Ibom	2212	2015	85.8	76.2	95.8
Nigeria	Anambra	2213	2000	124.6	115.8	134.2
Nigeria	Anambra	2213	2005	117.4	109.5	125.7
Nigeria	Anambra	2213	2010	102.5	94.6	110.4
Nigeria	Anambra	2213	2015	75.0	66.8	83.5
Nigeria	Bauchi	65699	2000	271.8	259.5	282.8
Nigeria	Bauchi	65699	2005	231.7	222.2	241.8
Nigeria	Bauchi	65699	2010	199.6	189.3	209.8
Nigeria	Bauchi	65699	2015	181.3	166.6	197.3
Nigeria	Bayelsa	65700	2000	169.6	157.7	182.2
Nigeria	Bayelsa	65700	2005	151.6	140.8	162.5
Nigeria	Bayelsa	65700	2010	132.7	122.6	143.0
Nigeria	Bayelsa	65700	2015	101.9	91.3	113.3
Nigeria	Benue	2215	2000	155.0	145.8	165.3

Admin 0	Admin 1	GAUL Code	Year	Under-5 mortality (per 1,000 livebirths)		
				Estimate	Lower bound, 95% UI	Upper bound, 95% UI
Nigeria	Benue	2215	2005	135.9	127.8	144.4
Nigeria	Benue	2215	2010	119.2	110.5	127.8
Nigeria	Benue	2215	2015	100.2	90.3	111.3
Nigeria	Borno	2216	2000	208.7	197.2	219.9
Nigeria	Borno	2216	2005	175.4	166.3	185.1
Nigeria	Borno	2216	2010	144.0	134.8	153.8
Nigeria	Borno	2216	2015	125.3	113.9	138.2
Nigeria	Cross River	2217	2000	137.9	130.0	146.2
Nigeria	Cross River	2217	2005	133.7	125.6	141.8
Nigeria	Cross River	2217	2010	114.9	107.0	123.3
Nigeria	Cross River	2217	2015	88.3	79.5	97.6
Nigeria	Delta	2218	2000	123.7	114.9	133.0
Nigeria	Delta	2218	2005	110.9	103.6	118.0
Nigeria	Delta	2218	2010	103.2	95.2	110.5
Nigeria	Delta	2218	2015	79.0	70.8	87.5
Nigeria	Ebonyi	65701	2000	180.0	169.6	190.6
Nigeria	Ebonyi	65701	2005	162.5	153.4	172.6
Nigeria	Ebonyi	65701	2010	136.3	126.5	146.9
Nigeria	Ebonyi	65701	2015	103.1	92.1	114.5
Nigeria	Edo	2219	2000	109.7	102.1	118.1
Nigeria	Edo	2219	2005	102.1	95.3	109.3
Nigeria	Edo	2219	2010	92.5	85.6	100.1
Nigeria	Edo	2219	2015	70.2	63.0	78.7
Nigeria	Ekiti	65702	2000	96.7	89.1	105.4
Nigeria	Ekiti	65702	2005	93.2	85.7	101.0
Nigeria	Ekiti	65702	2010	79.4	72.1	87.1
Nigeria	Ekiti	65702	2015	61.1	54.4	69.0
Nigeria	Enugu	65703	2000	133.7	125.2	142.8
Nigeria	Enugu	65703	2005	130.3	121.5	138.8
Nigeria	Enugu	65703	2010	118.1	109.7	127.3
Nigeria	Enugu	65703	2015	85.1	75.7	95.0
Nigeria	Gombe	65704	2000	250.9	238.8	263.7
Nigeria	Gombe	65704	2005	212.6	202.4	224.0
Nigeria	Gombe	65704	2010	176.0	165.7	186.4
Nigeria	Gombe	65704	2015	155.2	140.7	170.4
Nigeria	Imo	2222	2000	140.4	131.6	150.8
Nigeria	Imo	2222	2005	133.8	125.3	143.4
Nigeria	Imo	2222	2010	111.9	103.5	121.0
Nigeria	Imo	2222	2015	80.5	72.1	89.8
Nigeria	Jigawa	2223	2000	287.5	276.7	298.6
Nigeria	Jigawa	2223	2005	251.4	241.5	261.3
Nigeria	Jigawa	2223	2010	210.0	200.1	220.6
Nigeria	Jigawa	2223	2015	183.5	167.9	200.5
Nigeria	Kaduna	2224	2000	182.6	173.8	192.3
Nigeria	Kaduna	2224	2005	155.3	146.9	163.8
Nigeria	Kaduna	2224	2010	131.6	123.6	140.7
Nigeria	Kaduna	2224	2015	124.3	113.6	136.5
Nigeria	Kano	2225	2000	262.7	251.8	273.7
Nigeria	Kano	2225	2005	218.5	209.2	227.9
Nigeria	Kano	2225	2010	169.8	160.9	178.8
Nigeria	Kano	2225	2015	158.7	144.4	173.5
Nigeria	Katsina	2226	2000	279.2	267.3	290.5
Nigeria	Katsina	2226	2005	247.0	237.7	256.3
Nigeria	Katsina	2226	2010	208.8	198.7	219.0
Nigeria	Katsina	2226	2015	169.3	155.0	184.9
Nigeria	Kebbi	2227	2000	213.5	202.2	224.9
Nigeria	Kebbi	2227	2005	199.3	189.7	209.6
Nigeria	Kebbi	2227	2010	183.2	173.1	193.2
Nigeria	Kebbi	2227	2015	160.4	146.1	174.7
Nigeria	Kogi	2228	2000	129.0	120.7	138.1
Nigeria	Kogi	2228	2005	114.4	107.0	122.0
Nigeria	Kogi	2228	2010	100.3	92.9	107.7
Nigeria	Kogi	2228	2015	79.1	71.2	87.5
Nigeria	Kwara	2229	2000	102.7	94.9	111.1
Nigeria	Kwara	2229	2005	92.5	85.2	99.5
Nigeria	Kwara	2229	2010	84.3	77.2	92.1
Nigeria	Kwara	2229	2015	74.1	66.5	83.2
Nigeria	Lagos	2230	2000	87.7	81.0	94.8
Nigeria	Lagos	2230	2005	88.3	81.9	94.9

Admin 0	Admin 1	GAUL Code	Year	Under-5 mortality (per 1,000 livebirths)		
				Estimate	Lower bound, 95% UI	Upper bound, 95% UI
Nigeria	Lagos	2230	2010	83.4	76.5	90.2
Nigeria	Lagos	2230	2015	67.5	59.4	76.3
Nigeria	Nassarawa	65705	2000	163.4	152.9	174.6
Nigeria	Nassarawa	65705	2005	141.2	132.6	150.5
Nigeria	Nassarawa	65705	2010	124.5	115.2	134.0
Nigeria	Nassarawa	65705	2015	109.2	98.4	121.1
Nigeria	Niger	2231	2000	156.2	147.7	165.8
Nigeria	Niger	2231	2005	136.6	129.3	144.1
Nigeria	Niger	2231	2010	122.4	114.2	130.7
Nigeria	Niger	2231	2015	113.4	103.2	124.3
Nigeria	Ogun	2232	2000	107.7	101.1	114.8
Nigeria	Ogun	2232	2005	103.8	97.5	110.5
Nigeria	Ogun	2232	2010	90.4	83.8	96.6
Nigeria	Ogun	2232	2015	76.0	68.1	84.0
Nigeria	Ondo	65706	2000	112.2	104.7	120.1
Nigeria	Ondo	65706	2005	106.2	99.0	113.8
Nigeria	Ondo	65706	2010	92.7	85.4	100.1
Nigeria	Ondo	65706	2015	73.0	65.4	81.5
Nigeria	Osun	2234	2000	88.4	81.5	95.4
Nigeria	Osun	2234	2005	84.3	78.3	90.9
Nigeria	Osun	2234	2010	73.9	67.4	80.8
Nigeria	Osun	2234	2015	58.6	52.2	65.6
Nigeria	Oyo	2235	2000	94.6	87.5	101.9
Nigeria	Oyo	2235	2005	87.9	81.3	94.4
Nigeria	Oyo	2235	2010	77.8	71.6	84.0
Nigeria	Oyo	2235	2015	65.8	58.7	73.2
Nigeria	Plateau	65707	2000	167.3	157.1	178.0
Nigeria	Plateau	65707	2005	154.0	145.0	164.3
Nigeria	Plateau	65707	2010	137.2	127.4	148.5
Nigeria	Plateau	65707	2015	119.4	107.7	132.0
Nigeria	Rivers	65708	2000	141.7	131.5	152.6
Nigeria	Rivers	65708	2005	131.2	122.0	140.8
Nigeria	Rivers	65708	2010	107.3	97.8	116.9
Nigeria	Rivers	65708	2015	80.1	71.7	89.5
Nigeria	Sokoto	65709	2000	264.1	250.9	276.0
Nigeria	Sokoto	65709	2005	253.1	241.6	264.8
Nigeria	Sokoto	65709	2010	199.9	188.0	211.9
Nigeria	Sokoto	65709	2015	171.6	156.3	188.4
Nigeria	Taraba	2239	2000	206.5	196.0	218.1
Nigeria	Taraba	2239	2005	180.3	170.9	189.6
Nigeria	Taraba	2239	2010	147.0	137.7	156.9
Nigeria	Taraba	2239	2015	135.2	123.2	147.5
Nigeria	Yobe	2240	2000	217.0	205.5	228.9
Nigeria	Yobe	2240	2005	185.1	175.1	195.2
Nigeria	Yobe	2240	2010	162.8	152.6	174.0
Nigeria	Yobe	2240	2015	140.3	127.3	154.2
Nigeria	Zamfara	65710	2000	273.6	261.5	287.0
Nigeria	Zamfara	65710	2005	250.8	240.3	262.2
Nigeria	Zamfara	65710	2010	218.5	206.6	231.6
Nigeria	Zamfara	65710	2015	179.0	162.7	194.8
Rwanda	East/Iburasirazuba	21969	2000	192.3	186.1	198.8
Rwanda	East/Iburasirazuba	21969	2005	128.4	123.8	133.7
Rwanda	East/Iburasirazuba	21969	2010	77.6	73.9	81.7
Rwanda	East/Iburasirazuba	21969	2015	60.7	55.8	65.6
Rwanda	Kigali City/Umujyi wa Kigali	21970	2000	113.2	108.4	117.9
Rwanda	Kigali City/Umujyi wa Kigali	21970	2005	87.0	83.1	91.1
Rwanda	Kigali City/Umujyi wa Kigali	21970	2010	59.4	56.1	63.0
Rwanda	Kigali City/Umujyi wa Kigali	21970	2015	47.2	43.4	51.6
Rwanda	North/Amajyaruguru	21971	2000	159.4	153.5	165.0
Rwanda	North/Amajyaruguru	21971	2005	104.3	100.0	108.5
Rwanda	North/Amajyaruguru	21971	2010	63.7	60.1	67.2
Rwanda	North/Amajyaruguru	21971	2015	47.9	43.9	51.9
Rwanda	South/Amajyepfo	21972	2000	147.9	143.1	152.9
Rwanda	South/Amajyepfo	21972	2005	104.6	100.8	108.1
Rwanda	South/Amajyepfo	21972	2010	70.2	66.9	73.6
Rwanda	South/Amajyepfo	21972	2015	55.1	51.4	59.4
Rwanda	West/Iburengerazuba	21973	2000	143.4	138.2	148.8
Rwanda	West/Iburengerazuba	21973	2005	98.7	94.8	102.7
Rwanda	West/Iburengerazuba	21973	2010	64.1	60.6	67.6

Admin 0	Admin 1	GAUL Code	Year	Under-5 mortality (per 1,000 livebirths)		
				Estimate	Lower bound, 95% UI	Upper bound, 95% UI
Rwanda	West/Iburengerazuba	21973	2015	49.1	45.1	53.1
Senegal	Dakar	2636	2000	57.7	53.0	62.5
Senegal	Dakar	2636	2005	53.0	48.4	57.8
Senegal	Dakar	2636	2010	46.2	41.7	51.0
Senegal	Dakar	2636	2015	37.4	32.9	42.6
Senegal	Diourbel	47585	2000	129.6	123.7	135.7
Senegal	Diourbel	47585	2005	99.2	94.2	104.2
Senegal	Diourbel	47585	2010	72.5	67.7	77.1
Senegal	Diourbel	47585	2015	54.9	49.9	60.2
Senegal	Fatick	47586	2000	118.2	113.4	123.3
Senegal	Fatick	47586	2005	90.6	86.4	94.9
Senegal	Fatick	47586	2010	67.2	63.5	71.1
Senegal	Fatick	47586	2015	50.5	46.3	54.8
Senegal	Kaffrine	1378	2000	147.2	140.6	154.1
Senegal	Kaffrine	1378	2005	111.4	106.1	116.8
Senegal	Kaffrine	1378	2010	80.8	76.1	85.6
Senegal	Kaffrine	1378	2015	60.1	54.7	66.7
Senegal	Kaolack	1373	2000	132.1	126.7	137.8
Senegal	Kaolack	1373	2005	97.4	93.1	101.9
Senegal	Kaolack	1373	2010	67.8	64.1	71.5
Senegal	Kaolack	1373	2015	51.2	46.9	55.8
Senegal	Kedougou	1374	2000	192.8	180.7	205.3
Senegal	Kedougou	1374	2005	158.0	148.3	168.6
Senegal	Kedougou	1374	2010	126.2	117.5	136.1
Senegal	Kedougou	1374	2015	103.9	93.2	115.2
Senegal	Kolda	1375	2000	181.3	173.6	188.6
Senegal	Kolda	1375	2005	145.9	139.5	152.3
Senegal	Kolda	1375	2010	101.8	96.3	107.9
Senegal	Kolda	1375	2015	86.0	78.9	93.6
Senegal	Louga	47587	2000	108.1	102.7	114.1
Senegal	Louga	47587	2005	86.5	82.0	91.1
Senegal	Louga	47587	2010	66.2	61.7	70.7
Senegal	Louga	47587	2015	48.8	44.3	53.6
Senegal	Matam	47588	2000	113.0	106.7	119.2
Senegal	Matam	47588	2005	92.9	87.9	98.1
Senegal	Matam	47588	2010	75.6	70.4	81.1
Senegal	Matam	47588	2015	58.2	52.5	64.2
Senegal	Saint louis	47589	2000	100.4	94.8	105.9
Senegal	Saint louis	47589	2005	79.2	75.2	83.6
Senegal	Saint louis	47589	2010	62.6	58.3	67.6
Senegal	Saint louis	47589	2015	46.0	41.6	50.9
Senegal	Sedhiou	1376	2000	170.1	161.3	178.6
Senegal	Sedhiou	1376	2005	127.5	121.1	134.0
Senegal	Sedhiou	1376	2010	83.8	78.9	88.9
Senegal	Sedhiou	1376	2015	66.0	60.3	72.4
Senegal	Tambacounda	1377	2000	154.4	147.0	162.3
Senegal	Tambacounda	1377	2005	123.0	117.1	129.0
Senegal	Tambacounda	1377	2010	98.7	92.9	104.6
Senegal	Tambacounda	1377	2015	79.5	73.2	86.4
Senegal	Thies	2644	2000	83.1	78.5	87.4
Senegal	Thies	2644	2005	68.1	64.4	72.1
Senegal	Thies	2644	2010	54.2	50.5	58.1
Senegal	Thies	2644	2015	42.0	37.9	46.2
Senegal	Ziguinchor	2645	2000	107.5	100.7	114.6
Senegal	Ziguinchor	2645	2005	87.9	82.8	93.9
Senegal	Ziguinchor	2645	2010	64.9	60.2	70.2
Senegal	Ziguinchor	2645	2015	50.0	45.1	55.5
Sierra Leone	Eastern	2654	2000	239.3	230.4	248.5
Sierra Leone	Eastern	2654	2005	199.8	192.4	207.3
Sierra Leone	Eastern	2654	2010	164.4	155.9	173.0
Sierra Leone	Eastern	2654	2015	137.3	125.8	150.2
Sierra Leone	Northern	2655	2000	203.2	195.7	210.7
Sierra Leone	Northern	2655	2005	180.5	173.9	187.1
Sierra Leone	Northern	2655	2010	151.5	144.8	158.7
Sierra Leone	Northern	2655	2015	121.9	112.8	132.1
Sierra Leone	Southern	2656	2000	240.2	231.5	249.6
Sierra Leone	Southern	2656	2005	203.1	195.2	210.6
Sierra Leone	Southern	2656	2010	163.7	155.8	171.8
Sierra Leone	Southern	2656	2015	135.2	124.0	147.4

Admin 0	Admin 1	GAUL Code	Year	Under-5 mortality (per 1,000 livebirths)		
				Estimate	Lower bound, 95% UI	Upper bound, 95% UI
Sierra Leone	Western Area	2657	2000	147.4	137.3	159.0
Sierra Leone	Western Area	2657	2005	144.3	134.0	155.3
Sierra Leone	Western Area	2657	2010	120.5	110.6	129.8
Sierra Leone	Western Area	2657	2015	85.5	75.6	95.7
Somalia	Awdal	2688	2000	103.8	93.9	115.3
Somalia	Awdal	2688	2005	90.8	81.7	100.3
Somalia	Awdal	2688	2010	90.6	79.6	101.7
Somalia	Awdal	2688	2015	69.8	59.6	81.1
Somalia	Bakool	2689	2000	130.7	111.5	152.3
Somalia	Bakool	2689	2005	123.9	106.0	144.4
Somalia	Bakool	2689	2010	113.3	94.8	134.1
Somalia	Bakool	2689	2015	93.0	77.5	110.6
Somalia	Banadir	2692	2000	203.9	185.5	222.8
Somalia	Banadir	2692	2005	167.4	151.3	185.0
Somalia	Banadir	2692	2010	161.0	139.6	183.2
Somalia	Banadir	2692	2015	123.9	105.0	143.7
Somalia	Bari	2690	2000	120.9	105.6	139.0
Somalia	Bari	2690	2005	108.4	95.1	124.4
Somalia	Bari	2690	2010	118.1	102.4	136.8
Somalia	Bari	2690	2015	94.5	79.2	112.0
Somalia	Bay	2691	2000	183.8	162.9	205.5
Somalia	Bay	2691	2005	161.6	141.9	181.2
Somalia	Bay	2691	2010	130.5	112.8	150.1
Somalia	Bay	2691	2015	103.8	88.4	122.2
Somalia	Galgaduud	2693	2000	147.8	125.7	173.7
Somalia	Galgaduud	2693	2005	125.5	107.8	145.9
Somalia	Galgaduud	2693	2010	124.7	105.1	148.7
Somalia	Galgaduud	2693	2015	88.2	73.1	104.8
Somalia	Gedo	2694	2000	115.7	103.1	128.6
Somalia	Gedo	2694	2005	106.2	94.1	119.2
Somalia	Gedo	2694	2010	99.8	87.4	113.8
Somalia	Gedo	2694	2015	83.5	72.0	96.4
Somalia	Hiraan	2695	2000	123.8	104.3	144.2
Somalia	Hiraan	2695	2005	112.7	96.1	131.6
Somalia	Hiraan	2695	2010	114.7	96.8	136.9
Somalia	Hiraan	2695	2015	88.1	72.6	106.5
Somalia	Juba Dhexe	2698	2000	152.8	131.8	180.1
Somalia	Juba Dhexe	2698	2005	132.6	114.4	154.3
Somalia	Juba Dhexe	2698	2010	131.4	111.0	157.0
Somalia	Juba Dhexe	2698	2015	101.5	83.9	120.9
Somalia	Juba Hoose	2696	2000	162.9	142.3	189.2
Somalia	Juba Hoose	2696	2005	133.9	116.8	153.9
Somalia	Juba Hoose	2696	2010	128.9	110.9	150.5
Somalia	Juba Hoose	2696	2015	101.5	84.5	120.0
Somalia	Mudug	2700	2000	134.0	117.3	151.3
Somalia	Mudug	2700	2005	119.7	104.9	137.0
Somalia	Mudug	2700	2010	124.4	107.8	143.0
Somalia	Mudug	2700	2015	92.5	78.0	108.0
Somalia	Nugaal	2701	2000	144.4	124.3	165.3
Somalia	Nugaal	2701	2005	122.5	105.3	141.6
Somalia	Nugaal	2701	2010	122.3	104.0	141.9
Somalia	Nugaal	2701	2015	87.9	73.1	104.9
Somalia	Sanaag	2702	2000	122.2	107.7	136.5
Somalia	Sanaag	2702	2005	109.4	97.0	122.6
Somalia	Sanaag	2702	2010	119.1	103.6	134.1
Somalia	Sanaag	2702	2015	84.1	71.4	97.6
Somalia	Shabelle Dhexe	2699	2000	158.3	139.9	179.4
Somalia	Shabelle Dhexe	2699	2005	136.5	120.3	153.6
Somalia	Shabelle Dhexe	2699	2010	130.4	113.2	150.9
Somalia	Shabelle Dhexe	2699	2015	107.4	90.6	125.3
Somalia	Shabelle Hoose	2697	2000	204.6	185.2	224.1
Somalia	Shabelle Hoose	2697	2005	172.3	156.3	190.5
Somalia	Shabelle Hoose	2697	2010	156.8	137.8	177.4
Somalia	Shabelle Hoose	2697	2015	137.1	118.9	158.7
Somalia	Sool	2703	2000	136.8	121.7	153.9
Somalia	Sool	2703	2005	124.3	109.8	139.0
Somalia	Sool	2703	2010	126.1	110.2	143.6
Somalia	Sool	2703	2015	89.5	76.9	104.2
Somalia	Togdheer	2704	2000	146.4	133.3	161.2

Admin 0	Admin 1	GAUL Code	Year	Under-5 mortality (per 1,000 livebirths)		
				Estimate	Lower bound, 95% UI	Upper bound, 95% UI
Somalia	Togdheer	2704	2005	138.4	126.7	152.4
Somalia	Togdheer	2704	2010	145.1	129.4	160.8
Somalia	Togdheer	2704	2015	98.3	84.5	113.1
Somalia	Woqooyi Galbeed	2705	2000	112.6	103.5	121.7
Somalia	Woqooyi Galbeed	2705	2005	101.1	94.1	109.1
Somalia	Woqooyi Galbeed	2705	2010	105.2	96.1	115.1
Somalia	Woqooyi Galbeed	2705	2015	76.5	66.5	87.0
South Africa	Eastern Cape	77310	2000	89.2	83.2	95.7
South Africa	Eastern Cape	77310	2005	89.7	82.6	96.2
South Africa	Eastern Cape	77310	2010	68.9	62.1	75.8
South Africa	Eastern Cape	77310	2015	55.0	48.9	61.8
South Africa	Free State	2707	2000	86.4	80.2	93.4
South Africa	Free State	2707	2005	90.9	84.2	98.4
South Africa	Free State	2707	2010	76.8	69.8	84.6
South Africa	Free State	2707	2015	57.0	50.9	64.0
South Africa	Gauteng	2708	2000	57.4	51.6	63.3
South Africa	Gauteng	2708	2005	62.2	55.5	69.2
South Africa	Gauteng	2708	2010	54.8	47.5	62.9
South Africa	Gauteng	2708	2015	39.8	34.1	46.7
South Africa	KwaZulu-Natal	77311	2000	91.4	85.4	97.1
South Africa	KwaZulu-Natal	77311	2005	90.1	84.1	97.0
South Africa	KwaZulu-Natal	77311	2010	70.5	64.6	77.3
South Africa	KwaZulu-Natal	77311	2015	55.4	49.8	62.2
South Africa	Limpopo	77312	2000	69.2	63.0	75.3
South Africa	Limpopo	77312	2005	69.3	63.0	75.9
South Africa	Limpopo	77312	2010	59.0	52.8	66.1
South Africa	Limpopo	77312	2015	43.7	38.0	49.8
South Africa	Mpumalanga	77313	2000	84.2	78.1	89.7
South Africa	Mpumalanga	77313	2005	83.4	77.5	89.2
South Africa	Mpumalanga	77313	2010	67.5	61.8	73.8
South Africa	Mpumalanga	77313	2015	48.5	43.5	54.4
South Africa	North West	77314	2000	73.3	67.9	79.3
South Africa	North West	77314	2005	73.1	66.6	79.6
South Africa	North West	77314	2010	62.2	55.7	69.0
South Africa	North West	77314	2015	46.6	40.5	52.8
South Africa	Northern Cape	77315	2000	78.3	71.0	86.9
South Africa	Northern Cape	77315	2005	77.6	69.5	86.2
South Africa	Northern Cape	77315	2010	64.6	57.1	72.4
South Africa	Northern Cape	77315	2015	48.9	42.8	55.8
South Africa	Western Cape	2714	2000	50.7	44.9	56.6
South Africa	Western Cape	2714	2005	52.6	46.0	59.7
South Africa	Western Cape	2714	2010	45.7	39.4	53.5
South Africa	Western Cape	2714	2015	32.8	27.6	39.0
South Sudan	Central Equatoria	2748	2000	151.7	142.3	161.0
South Sudan	Central Equatoria	2748	2005	130.2	121.4	139.0
South Sudan	Central Equatoria	2748	2010	106.7	98.1	115.4
South Sudan	Central Equatoria	2748	2015	97.2	86.8	108.6
South Sudan	Eastern Equatoria	2750	2000	128.6	119.7	137.9
South Sudan	Eastern Equatoria	2750	2005	112.0	104.3	119.8
South Sudan	Eastern Equatoria	2750	2010	100.8	92.5	109.5
South Sudan	Eastern Equatoria	2750	2015	88.0	79.5	97.8
South Sudan	El Buheyrat	2746	2000	126.4	117.9	136.5
South Sudan	El Buheyrat	2746	2005	104.8	97.0	113.2
South Sudan	El Buheyrat	2746	2010	96.2	87.7	106.0
South Sudan	El Buheyrat	2746	2015	91.4	81.1	103.4
South Sudan	Jonglei	2751	2000	126.3	117.1	135.7
South Sudan	Jonglei	2751	2005	107.3	99.3	115.4
South Sudan	Jonglei	2751	2010	93.8	86.0	102.8
South Sudan	Jonglei	2751	2015	84.8	75.6	94.7
South Sudan	Northern Bahr El Ghazal	2754	2000	184.7	173.5	196.6
South Sudan	Northern Bahr El Ghazal	2754	2005	158.0	147.3	168.5
South Sudan	Northern Bahr El Ghazal	2754	2010	143.6	131.8	155.8
South Sudan	Northern Bahr El Ghazal	2754	2015	121.4	107.7	136.0
South Sudan	Unity	2747	2000	131.1	121.6	140.6
South Sudan	Unity	2747	2005	112.8	104.4	121.8
South Sudan	Unity	2747	2010	106.1	96.5	116.5
South Sudan	Unity	2747	2015	100.2	88.9	113.0
South Sudan	Upper Nile	37021	2000	137.1	128.7	146.4
South Sudan	Upper Nile	37021	2005	115.8	108.2	124.2

Admin 0	Admin 1	GAUL Code	Year	Under-5 mortality (per 1,000 livebirths)		
				Estimate	Lower bound, 95% UI	Upper bound, 95% UI
South Sudan	Upper Nile	37021	2010	97.0	89.4	104.9
South Sudan	Upper Nile	37021	2015	85.5	76.7	95.4
South Sudan	Warab	2765	2000	162.0	152.7	171.7
South Sudan	Warab	2765	2005	137.5	129.0	146.7
South Sudan	Warab	2765	2010	122.1	112.5	133.3
South Sudan	Warab	2765	2015	113.6	101.3	126.5
South Sudan	Western Bahr El Ghazal	2766	2000	151.6	141.8	162.3
South Sudan	Western Bahr El Ghazal	2766	2005	130.2	120.4	139.8
South Sudan	Western Bahr El Ghazal	2766	2010	117.0	106.6	128.0
South Sudan	Western Bahr El Ghazal	2766	2015	103.7	93.1	115.2
South Sudan	Western Equatoria	2768	2000	151.2	140.3	162.6
South Sudan	Western Equatoria	2768	2005	135.7	125.5	145.6
South Sudan	Western Equatoria	2768	2010	112.1	102.1	123.1
South Sudan	Western Equatoria	2768	2015	105.2	94.1	118.0
Sudan	Abyei	124	2000	128.4	114.3	143.3
Sudan	Abyei	124	2005	106.2	94.5	118.0
Sudan	Abyei	124	2010	110.0	96.4	124.4
Sudan	Abyei	124	2015	101.5	87.4	116.5
Sudan	Al Jazeera	2745	2000	84.5	78.9	90.8
Sudan	Al Jazeera	2745	2005	64.3	59.8	69.2
Sudan	Al Jazeera	2745	2010	60.8	56.1	65.7
Sudan	Al Jazeera	2745	2015	55.3	49.7	62.0
Sudan	Blue Nile	2749	2000	187.7	178.2	198.2
Sudan	Blue Nile	2749	2005	149.9	142.3	157.9
Sudan	Blue Nile	2749	2010	126.2	118.1	135.0
Sudan	Blue Nile	2749	2015	100.1	90.2	110.5
Sudan	Gadaref	68805	2000	122.2	114.6	129.6
Sudan	Gadaref	68805	2005	94.4	88.7	100.6
Sudan	Gadaref	68805	2010	88.3	82.5	95.1
Sudan	Gadaref	68805	2015	79.2	71.7	87.5
Sudan	Kassala	68783	2000	84.3	78.4	90.2
Sudan	Kassala	68783	2005	70.3	65.5	75.5
Sudan	Kassala	68783	2010	69.2	63.6	75.3
Sudan	Kassala	68783	2015	64.0	56.7	71.9
Sudan	Khartoum	2753	2000	78.7	72.7	84.7
Sudan	Khartoum	2753	2005	62.9	58.3	67.8
Sudan	Khartoum	2753	2010	61.7	56.0	67.3
Sudan	Khartoum	2753	2015	50.2	44.1	56.9
Sudan	Nile	2761	2000	72.7	66.1	79.1
Sudan	Nile	2761	2005	60.9	55.8	65.9
Sudan	Nile	2761	2010	58.9	53.7	64.5
Sudan	Nile	2761	2015	49.7	44.0	55.9
Sudan	Northern	2755	2000	66.9	61.1	73.1
Sudan	Northern	2755	2005	60.5	55.4	65.8
Sudan	Northern	2755	2010	59.5	53.9	65.3
Sudan	Northern	2755	2015	47.4	42.2	53.1
Sudan	Northern Darfur	2757	2000	78.1	73.2	83.5
Sudan	Northern Darfur	2757	2005	71.5	67.3	76.1
Sudan	Northern Darfur	2757	2010	70.5	65.7	76.0
Sudan	Northern Darfur	2757	2015	68.1	61.7	74.7
Sudan	Northern Kordofan	68784	2000	89.1	83.7	95.4
Sudan	Northern Kordofan	68784	2005	75.6	71.1	80.6
Sudan	Northern Kordofan	68784	2010	71.6	67.1	77.0
Sudan	Northern Kordofan	68784	2015	64.4	58.5	70.9
Sudan	Red Sea	40772	2000	80.0	73.8	86.7
Sudan	Red Sea	40772	2005	69.8	64.3	75.6
Sudan	Red Sea	40772	2010	66.7	60.9	73.0
Sudan	Red Sea	40772	2015	59.0	52.2	66.2
Sudan	Sennar	2762	2000	101.2	94.8	108.3
Sudan	Sennar	2762	2005	80.9	76.0	86.1
Sudan	Sennar	2762	2010	73.6	68.5	79.5
Sudan	Sennar	2762	2015	62.7	56.1	69.7
Sudan	Southern Darfur	2764	2000	96.7	91.4	102.2
Sudan	Southern Darfur	2764	2005	85.0	80.3	89.7
Sudan	Southern Darfur	2764	2010	81.4	76.2	86.6
Sudan	Southern Darfur	2764	2015	78.8	72.4	86.0
Sudan	Southern Kordofan	4150	2000	125.6	118.1	133.8
Sudan	Southern Kordofan	4150	2005	105.5	99.3	111.5
Sudan	Southern Kordofan	4150	2010	97.0	90.5	103.8

Admin 0	Admin 1	GAUL Code	Year	Under-5 mortality (per 1,000 livebirths)		
				Estimate	Lower bound, 95% UI	Upper bound, 95% UI
Sudan	Southern Kordofan	4150	2015	85.8	77.7	94.0
Sudan	Western Darfur	2769	2000	102.1	95.7	108.6
Sudan	Western Darfur	2769	2005	91.7	86.7	96.8
Sudan	Western Darfur	2769	2010	82.2	76.8	87.4
Sudan	Western Darfur	2769	2015	79.2	72.0	86.2
Sudan	White Nile	2770	2000	94.6	88.5	100.7
Sudan	White Nile	2770	2005	73.3	68.6	77.8
Sudan	White Nile	2770	2010	68.8	63.7	73.9
Sudan	White Nile	2770	2015	59.5	53.2	66.1
Swaziland	Hhohho	2782	2000	81.5	75.5	88.0
Swaziland	Hhohho	2782	2005	88.0	81.6	95.2
Swaziland	Hhohho	2782	2010	64.2	58.8	70.1
Swaziland	Hhohho	2782	2015	48.9	43.1	55.5
Swaziland	Lubombo	2783	2000	100.5	93.3	107.7
Swaziland	Lubombo	2783	2005	106.4	98.8	113.9
Swaziland	Lubombo	2783	2010	80.1	73.4	87.2
Swaziland	Lubombo	2783	2015	55.9	49.5	63.4
Swaziland	Manzini	2784	2000	91.8	85.3	98.6
Swaziland	Manzini	2784	2005	101.2	94.1	108.4
Swaziland	Manzini	2784	2010	75.8	69.6	82.3
Swaziland	Manzini	2784	2015	52.6	46.5	59.3
Swaziland	Shiselweni	2785	2000	103.2	94.9	111.6
Swaziland	Shiselweni	2785	2005	107.4	99.0	115.9
Swaziland	Shiselweni	2785	2010	84.0	76.7	91.9
Swaziland	Shiselweni	2785	2015	58.6	51.9	66.3
Togo	Centrale	2970	2000	137.3	127.8	148.0
Togo	Centrale	2970	2005	122.6	114.4	132.1
Togo	Centrale	2970	2010	104.5	97.0	112.7
Togo	Centrale	2970	2015	84.3	75.7	94.4
Togo	Kara	2971	2000	143.2	134.2	153.7
Togo	Kara	2971	2005	132.2	123.8	141.1
Togo	Kara	2971	2010	108.3	100.3	116.8
Togo	Kara	2971	2015	88.5	80.1	97.7
Togo	Maritime	2972	2000	98.4	92.2	105.1
Togo	Maritime	2972	2005	85.7	80.0	91.0
Togo	Maritime	2972	2010	72.6	67.4	77.9
Togo	Maritime	2972	2015	56.0	50.0	62.0
Togo	Plateaux	2973	2000	123.5	115.3	132.1
Togo	Plateaux	2973	2005	106.5	100.0	113.5
Togo	Plateaux	2973	2010	91.5	84.8	98.0
Togo	Plateaux	2973	2015	80.7	73.1	89.5
Togo	Savanes	2974	2000	154.9	145.9	164.2
Togo	Savanes	2974	2005	142.7	134.6	150.5
Togo	Savanes	2974	2010	118.1	110.1	125.8
Togo	Savanes	2974	2015	91.0	82.7	99.8
Uganda	Abim	743	2000	153.6	140.3	168.7
Uganda	Abim	743	2005	129.6	118.4	142.0
Uganda	Abim	743	2010	104.5	94.4	115.0
Uganda	Abim	743	2015	82.0	72.1	92.5
Uganda	Adjumani	3104	2000	144.1	133.2	156.4
Uganda	Adjumani	3104	2005	122.8	113.4	132.6
Uganda	Adjumani	3104	2010	102.4	93.4	111.9
Uganda	Adjumani	3104	2015	81.0	72.2	91.4
Uganda	Agago	790	2000	162.2	148.6	177.3
Uganda	Agago	790	2005	135.6	124.6	147.8
Uganda	Agago	790	2010	108.3	98.3	118.5
Uganda	Agago	790	2015	85.6	75.3	95.6
Uganda	Alebtong	852	2000	141.0	131.8	150.5
Uganda	Alebtong	852	2005	120.0	112.6	128.4
Uganda	Alebtong	852	2010	96.7	90.0	103.7
Uganda	Alebtong	852	2015	77.9	69.7	85.9
Uganda	Amolatar	47067	2000	122.2	113.1	132.3
Uganda	Amolatar	47067	2005	100.4	93.4	108.2
Uganda	Amolatar	47067	2010	85.0	78.8	92.5
Uganda	Amolatar	47067	2015	68.5	61.0	76.6
Uganda	Amudat	744	2000	129.5	119.9	139.3
Uganda	Amudat	744	2005	104.5	96.7	113.5
Uganda	Amudat	744	2010	77.6	70.6	85.4
Uganda	Amudat	744	2015	66.4	58.8	74.1

Admin 0	Admin 1	GAUL Code	Year	Under-5 mortality (per 1,000 livebirths)		
				Estimate	Lower bound, 95% UI	Upper bound, 95% UI
Uganda	Amuria	47068	2000	132.3	122.9	143.2
Uganda	Amuria	47068	2005	112.0	103.8	120.4
Uganda	Amuria	47068	2010	90.9	84.4	98.4
Uganda	Amuria	47068	2015	72.1	64.6	80.2
Uganda	Amuru	745	2000	157.2	145.5	169.2
Uganda	Amuru	745	2005	133.2	123.2	143.7
Uganda	Amuru	745	2010	108.5	99.1	117.9
Uganda	Amuru	745	2015	88.9	78.8	100.8
Uganda	Apac	746	2000	128.7	120.6	137.7
Uganda	Apac	746	2005	106.9	99.9	114.1
Uganda	Apac	746	2010	89.5	82.7	96.1
Uganda	Apac	746	2015	73.2	65.4	81.6
Uganda	Arua	1057	2000	165.3	156.5	174.6
Uganda	Arua	1057	2005	137.7	129.5	146.7
Uganda	Arua	1057	2010	107.4	99.1	115.8
Uganda	Arua	1057	2015	83.8	75.2	93.3
Uganda	Budaka	747	2000	123.2	116.4	130.3
Uganda	Budaka	747	2005	101.5	96.1	107.3
Uganda	Budaka	747	2010	83.1	77.8	88.8
Uganda	Budaka	747	2015	65.3	59.2	72.0
Uganda	Bududa	1080	2000	116.2	109.1	123.9
Uganda	Bududa	1080	2005	93.1	87.2	99.4
Uganda	Bududa	1080	2010	76.9	71.2	82.8
Uganda	Bududa	1080	2015	58.8	53.3	65.1
Uganda	Bugiri	748	2000	155.4	146.9	164.3
Uganda	Bugiri	748	2005	127.8	120.1	135.1
Uganda	Bugiri	748	2010	100.1	93.4	107.1
Uganda	Bugiri	748	2015	74.6	67.5	82.2
Uganda	Buhweju	778	2000	151.1	140.9	160.4
Uganda	Buhweju	778	2005	120.5	111.9	129.5
Uganda	Buhweju	778	2010	84.4	77.6	92.2
Uganda	Buhweju	778	2015	63.8	57.2	71.4
Uganda	Buikwe	749	2000	111.1	104.1	118.9
Uganda	Buikwe	749	2005	100.3	94.1	106.9
Uganda	Buikwe	749	2010	85.9	80.1	92.4
Uganda	Buikwe	749	2015	56.7	50.7	62.9
Uganda	Bukedea	788	2000	114.7	108.4	121.4
Uganda	Bukedea	788	2005	94.9	89.4	100.9
Uganda	Bukedea	788	2010	78.2	72.9	83.9
Uganda	Bukedea	788	2015	63.2	57.2	70.0
Uganda	Bukomansimbi	750	2000	138.4	129.4	147.4
Uganda	Bukomansimbi	750	2005	116.5	108.7	124.5
Uganda	Bukomansimbi	750	2010	100.1	92.9	108.5
Uganda	Bukomansimbi	750	2015	70.6	63.5	79.2
Uganda	Bukwo	751	2000	94.6	86.9	102.3
Uganda	Bukwo	751	2005	73.7	67.3	80.2
Uganda	Bukwo	751	2010	58.7	53.4	64.5
Uganda	Bukwo	751	2015	48.8	43.5	54.6
Uganda	Bulambuli	752	2000	109.6	102.8	116.8
Uganda	Bulambuli	752	2005	89.1	83.0	95.4
Uganda	Bulambuli	752	2010	73.8	68.0	79.5
Uganda	Bulambuli	752	2015	58.5	52.7	65.2
Uganda	Buliisa	753	2000	157.6	143.3	172.8
Uganda	Buliisa	753	2005	133.1	121.2	145.2
Uganda	Buliisa	753	2010	105.1	95.7	116.2
Uganda	Buliisa	753	2015	82.0	71.9	92.6
Uganda	Bundibugyo	754	2000	153.3	141.0	167.3
Uganda	Bundibugyo	754	2005	126.4	115.7	137.4
Uganda	Bundibugyo	754	2010	91.6	82.9	101.1
Uganda	Bundibugyo	754	2015	70.8	62.3	79.6
Uganda	Bushenyi	755	2000	144.4	134.9	154.1
Uganda	Bushenyi	755	2005	115.8	107.8	124.3
Uganda	Bushenyi	755	2010	80.8	74.3	88.3
Uganda	Bushenyi	755	2015	61.9	55.3	69.2
Uganda	Busia	3110	2000	160.0	152.8	168.1
Uganda	Busia	3110	2005	131.3	124.5	138.6
Uganda	Busia	3110	2010	103.2	96.7	110.1
Uganda	Busia	3110	2015	73.7	66.7	81.0
Uganda	Butaleja	47071	2000	133.8	125.9	141.8

Admin 0	Admin 1	GAUL Code	Year	Under-5 mortality (per 1,000 livebirths)		
				Estimate	Lower bound, 95% UI	Upper bound, 95% UI
Uganda	Butaleja	47071	2005	109.7	103.5	116.1
Uganda	Butaleja	47071	2010	88.9	83.4	95.2
Uganda	Butaleja	47071	2015	68.7	62.3	75.7
Uganda	Butambala	757	2000	105.4	98.3	114.0
Uganda	Butambala	757	2005	95.0	88.2	102.2
Uganda	Butambala	757	2010	82.1	75.7	89.1
Uganda	Butambala	757	2015	59.2	53.4	66.4
Uganda	Buvuma	1082	2000	137.7	126.8	148.9
Uganda	Buvuma	1082	2005	117.0	107.6	126.5
Uganda	Buvuma	1082	2010	98.4	90.3	106.7
Uganda	Buvuma	1082	2015	62.6	55.9	69.8
Uganda	Buyende	756	2000	130.2	121.3	139.4
Uganda	Buyende	756	2005	110.8	103.5	118.4
Uganda	Buyende	756	2010	90.1	83.8	96.6
Uganda	Buyende	756	2015	70.6	63.8	78.5
Uganda	Dokolo	789	2000	126.8	117.9	136.2
Uganda	Dokolo	789	2005	105.0	98.6	112.5
Uganda	Dokolo	789	2010	87.1	80.9	94.2
Uganda	Dokolo	789	2015	70.6	63.3	78.1
Uganda	Gomba	1086	2000	115.4	107.6	123.7
Uganda	Gomba	1086	2005	101.8	95.4	109.1
Uganda	Gomba	1086	2010	87.8	81.4	94.8
Uganda	Gomba	1086	2015	64.5	57.8	71.9
Uganda	Gulu	1089	2000	154.9	143.6	166.8
Uganda	Gulu	1089	2005	129.1	119.9	139.1
Uganda	Gulu	1089	2010	104.3	95.7	113.5
Uganda	Gulu	1089	2015	85.5	75.3	96.4
Uganda	Hoima	3112	2000	138.4	127.5	149.7
Uganda	Hoima	3112	2005	118.9	109.7	129.0
Uganda	Hoima	3112	2010	95.8	88.0	104.0
Uganda	Hoima	3112	2015	75.5	67.1	84.2
Uganda	Ibanda	47072	2000	164.8	154.8	175.4
Uganda	Ibanda	47072	2005	135.6	127.0	145.4
Uganda	Ibanda	47072	2010	98.0	90.8	106.5
Uganda	Ibanda	47072	2015	73.7	66.6	82.1
Uganda	Iganga	758	2000	139.0	131.6	146.9
Uganda	Iganga	758	2005	117.1	110.3	123.5
Uganda	Iganga	758	2010	93.7	87.7	100.3
Uganda	Iganga	758	2015	69.0	62.3	76.1
Uganda	Isingiro	47073	2000	166.3	156.1	178.1
Uganda	Isingiro	47073	2005	133.8	124.7	143.5
Uganda	Isingiro	47073	2010	94.7	87.6	102.9
Uganda	Isingiro	47073	2015	66.6	59.5	74.3
Uganda	Jinja	3114	2000	111.0	104.9	117.4
Uganda	Jinja	3114	2005	101.2	95.6	106.8
Uganda	Jinja	3114	2010	86.1	80.9	91.9
Uganda	Jinja	3114	2015	57.7	52.2	63.4
Uganda	Kaabong	47074	2000	145.6	134.0	157.9
Uganda	Kaabong	47074	2005	120.0	109.6	130.9
Uganda	Kaabong	47074	2010	95.8	87.0	104.9
Uganda	Kaabong	47074	2015	77.1	67.9	86.9
Uganda	Kabale	3115	2000	150.3	142.6	157.7
Uganda	Kabale	3115	2005	107.5	101.4	113.7
Uganda	Kabale	3115	2010	72.6	67.6	78.0
Uganda	Kabale	3115	2015	53.7	48.6	59.5
Uganda	Kabarole	42180	2000	148.1	138.0	158.7
Uganda	Kabarole	42180	2005	122.6	113.4	131.5
Uganda	Kabarole	42180	2010	89.7	82.2	98.1
Uganda	Kabarole	42180	2015	70.2	62.4	78.5
Uganda	Kaberamaido	42181	2000	121.7	113.1	130.2
Uganda	Kaberamaido	42181	2005	101.4	95.0	108.9
Uganda	Kaberamaido	42181	2010	83.6	77.9	90.0
Uganda	Kaberamaido	42181	2015	67.9	61.3	75.3
Uganda	Kalangala	3117	2000	129.7	117.0	143.2
Uganda	Kalangala	3117	2005	106.8	96.3	118.0
Uganda	Kalangala	3117	2010	86.3	77.8	96.1
Uganda	Kalangala	3117	2015	61.1	54.2	69.7
Uganda	Kaliro	47075	2000	133.5	124.4	142.5
Uganda	Kaliro	47075	2005	112.9	106.2	120.2

Admin 0	Admin 1	GAUL Code	Year	Under-5 mortality (per 1,000 livebirths)		
				Estimate	Lower bound, 95% UI	Upper bound, 95% UI
Uganda	Kaliro	47075	2010	91.4	85.2	98.2
Uganda	Kaliro	47075	2015	71.3	64.4	79.2
Uganda	Kalungu	759	2000	126.9	118.1	135.9
Uganda	Kalungu	759	2005	107.4	99.5	116.0
Uganda	Kalungu	759	2010	92.1	85.0	99.8
Uganda	Kalungu	759	2015	65.9	59.1	73.9
Uganda	Kampala	42182	2000	80.5	76.2	85.3
Uganda	Kampala	42182	2005	77.6	73.5	81.7
Uganda	Kampala	42182	2010	69.8	65.6	74.5
Uganda	Kampala	42182	2015	50.8	45.6	56.3
Uganda	Kamuli	760	2000	126.6	119.5	134.0
Uganda	Kamuli	760	2005	109.5	102.9	115.9
Uganda	Kamuli	760	2010	89.6	83.6	96.4
Uganda	Kamuli	760	2015	65.8	59.5	72.7
Uganda	Kamwenge	42183	2000	166.7	156.1	178.6
Uganda	Kamwenge	42183	2005	138.2	129.1	148.0
Uganda	Kamwenge	42183	2010	101.1	93.5	109.6
Uganda	Kamwenge	42183	2015	78.4	70.1	87.2
Uganda	Kanungu	42184	2000	135.8	125.1	147.7
Uganda	Kanungu	42184	2005	104.6	96.6	113.5
Uganda	Kanungu	42184	2010	71.3	64.7	78.2
Uganda	Kanungu	42184	2015	53.4	47.3	60.2
Uganda	Kapchorwa	761	2000	108.2	100.7	116.2
Uganda	Kapchorwa	761	2005	86.6	80.0	93.4
Uganda	Kapchorwa	761	2010	70.6	65.0	76.5
Uganda	Kapchorwa	761	2015	55.8	49.7	62.2
Uganda	Kasese	3121	2000	125.7	116.8	134.8
Uganda	Kasese	3121	2005	103.8	96.2	111.7
Uganda	Kasese	3121	2010	77.1	70.9	84.0
Uganda	Kasese	3121	2015	58.7	52.3	65.5
Uganda	Katakwi	47078	2000	130.3	120.5	140.6
Uganda	Katakwi	47078	2005	109.6	101.3	118.1
Uganda	Katakwi	47078	2010	88.4	81.2	95.7
Uganda	Katakwi	47078	2015	68.6	61.1	76.7
Uganda	Kayunga	42185	2000	116.2	110.1	122.5
Uganda	Kayunga	42185	2005	101.2	95.4	107.1
Uganda	Kayunga	42185	2010	85.2	79.6	91.4
Uganda	Kayunga	42185	2015	58.9	53.2	65.1
Uganda	Kibaale	3123	2000	147.6	137.3	158.0
Uganda	Kibaale	3123	2005	123.1	114.9	131.9
Uganda	Kibaale	3123	2010	97.0	89.8	104.7
Uganda	Kibaale	3123	2015	77.6	70.0	86.4
Uganda	Kiboga	1234	2000	121.0	111.1	132.0
Uganda	Kiboga	1234	2005	100.9	92.7	109.2
Uganda	Kiboga	1234	2010	82.5	75.3	90.4
Uganda	Kiboga	1234	2015	65.6	58.5	74.0
Uganda	Kibuku	1235	2000	128.0	120.4	135.6
Uganda	Kibuku	1235	2005	106.6	100.7	112.7
Uganda	Kibuku	1235	2010	86.1	80.3	92.2
Uganda	Kibuku	1235	2015	69.1	62.4	76.2
Uganda	Kiruhura	47079	2000	167.7	156.9	179.4
Uganda	Kiruhura	47079	2005	140.3	130.6	150.7
Uganda	Kiruhura	47079	2010	105.4	97.7	114.1
Uganda	Kiruhura	47079	2015	77.3	70.0	85.7
Uganda	Kiryandongo	1241	2000	138.0	126.2	149.2
Uganda	Kiryandongo	1241	2005	114.1	104.7	124.0
Uganda	Kiryandongo	1241	2010	94.3	86.5	103.0
Uganda	Kiryandongo	1241	2015	75.4	67.0	85.2
Uganda	Kisoro	3125	2000	165.4	155.1	175.6
Uganda	Kisoro	3125	2005	116.1	108.6	123.9
Uganda	Kisoro	3125	2010	76.3	70.5	82.1
Uganda	Kisoro	3125	2015	55.1	49.5	61.1
Uganda	Kitgum	763	2000	168.5	152.9	182.5
Uganda	Kitgum	763	2005	138.1	125.7	151.6
Uganda	Kitgum	763	2010	110.6	100.0	122.1
Uganda	Kitgum	763	2015	88.3	78.0	100.4
Uganda	Koboko	47080	2000	157.6	145.6	170.0
Uganda	Koboko	47080	2005	133.0	122.7	143.7
Uganda	Koboko	47080	2010	106.4	97.2	116.9

Admin 0	Admin 1	GAUL Code	Year	Under-5 mortality (per 1,000 livebirths)		
				Estimate	Lower bound, 95% UI	Upper bound, 95% UI
Uganda	Koboko	47080	2015	83.8	73.7	95.3
Uganda	Kole	764	2000	142.4	133.8	151.7
Uganda	Kole	764	2005	119.8	112.8	127.3
Uganda	Kole	764	2010	98.3	91.6	105.2
Uganda	Kole	764	2015	79.8	71.6	88.1
Uganda	Kotido	765	2000	136.8	125.4	148.9
Uganda	Kotido	765	2005	115.3	104.6	125.8
Uganda	Kotido	765	2010	96.6	87.4	106.5
Uganda	Kotido	765	2015	74.8	65.5	84.9
Uganda	Kumi	1261	2000	116.3	109.2	123.8
Uganda	Kumi	1261	2005	97.8	91.9	104.1
Uganda	Kumi	1261	2010	80.0	74.6	85.6
Uganda	Kumi	1261	2015	65.2	58.8	72.0
Uganda	Kween	766	2000	104.5	96.4	112.5
Uganda	Kween	766	2005	82.4	75.8	89.2
Uganda	Kween	766	2010	65.8	60.1	71.5
Uganda	Kween	766	2015	53.1	47.5	59.3
Uganda	Kyankwanzi	1344	2000	129.6	118.2	141.7
Uganda	Kyankwanzi	1344	2005	108.9	99.7	118.4
Uganda	Kyankwanzi	1344	2010	88.4	80.9	96.6
Uganda	Kyankwanzi	1344	2015	71.2	63.1	80.3
Uganda	Kyegegwa	1361	2000	159.7	146.7	173.8
Uganda	Kyegegwa	1361	2005	132.4	122.5	143.2
Uganda	Kyegegwa	1361	2010	104.6	95.7	114.3
Uganda	Kyegegwa	1361	2015	82.2	73.2	91.8
Uganda	Kyenjojo	1362	2000	160.2	149.2	172.2
Uganda	Kyenjojo	1362	2005	133.4	124.6	143.2
Uganda	Kyenjojo	1362	2010	99.7	91.8	108.8
Uganda	Kyenjojo	1362	2015	79.2	70.6	88.5
Uganda	Lamwo	767	2000	164.5	148.9	180.5
Uganda	Lamwo	767	2005	135.4	122.4	149.5
Uganda	Lamwo	767	2010	112.9	101.4	125.1
Uganda	Lamwo	767	2015	90.3	79.6	102.4
Uganda	Lira	1370	2000	139.5	130.2	149.2
Uganda	Lira	1370	2005	117.6	110.0	125.1
Uganda	Lira	1370	2010	97.2	90.6	104.0
Uganda	Lira	1370	2015	78.2	69.6	86.5
Uganda	Luuka	768	2000	133.5	126.6	140.8
Uganda	Luuka	768	2005	114.0	108.1	120.3
Uganda	Luuka	768	2010	92.1	86.5	98.5
Uganda	Luuka	768	2015	67.8	61.4	74.7
Uganda	Luwero	47083	2000	106.5	99.4	114.4
Uganda	Luwero	47083	2005	92.6	86.8	99.3
Uganda	Luwero	47083	2010	77.9	71.8	84.3
Uganda	Luwero	47083	2015	55.4	49.8	62.4
Uganda	Lwengo	1366	2000	157.5	147.9	167.7
Uganda	Lwengo	1366	2005	128.0	120.2	136.6
Uganda	Lwengo	1366	2010	105.7	97.9	114.1
Uganda	Lwengo	1366	2015	73.8	66.3	82.4
Uganda	Lyantonde	1372	2000	170.2	158.4	182.2
Uganda	Lyantonde	1372	2005	138.4	129.7	149.1
Uganda	Lyantonde	1372	2010	111.2	103.0	120.4
Uganda	Lyantonde	1372	2015	78.7	71.1	88.0
Uganda	Manafwa	769	2000	123.7	117.2	131.3
Uganda	Manafwa	769	2005	99.4	93.9	105.3
Uganda	Manafwa	769	2010	81.6	76.2	87.6
Uganda	Manafwa	769	2015	61.9	56.1	68.2
Uganda	Maracha	780	2000	163.3	152.6	174.3
Uganda	Maracha	780	2005	136.6	128.0	146.2
Uganda	Maracha	780	2010	106.9	98.4	116.2
Uganda	Maracha	780	2015	83.8	74.4	93.6
Uganda	Masaka	770	2000	131.1	122.4	141.8
Uganda	Masaka	770	2005	109.6	100.7	118.8
Uganda	Masaka	770	2010	93.5	85.9	102.2
Uganda	Masaka	770	2015	66.0	59.2	73.9
Uganda	Masindi	771	2000	136.6	123.7	150.4
Uganda	Masindi	771	2005	113.8	103.1	124.5
Uganda	Masindi	771	2010	91.6	83.1	100.3
Uganda	Masindi	771	2015	72.3	63.6	81.4

Admin 0	Admin 1	GAUL Code	Year	Under-5 mortality (per 1,000 livebirths)		
				Estimate	Lower bound, 95% UI	Upper bound, 95% UI
Uganda	Mayuge	42189	2000	144.6	135.9	153.0
Uganda	Mayuge	42189	2005	122.3	115.0	129.7
Uganda	Mayuge	42189	2010	98.3	91.7	105.0
Uganda	Mayuge	42189	2015	68.4	61.9	75.6
Uganda	Mbale	47085	2000	117.2	111.0	124.0
Uganda	Mbale	47085	2005	95.9	90.4	101.5
Uganda	Mbale	47085	2010	79.0	73.6	84.7
Uganda	Mbale	47085	2015	61.4	55.7	67.7
Uganda	Mbarara	47086	2000	155.1	146.4	165.2
Uganda	Mbarara	47086	2005	123.0	115.3	131.5
Uganda	Mbarara	47086	2010	86.5	80.1	93.5
Uganda	Mbarara	47086	2015	64.6	58.4	72.0
Uganda	Mitooma	1360	2000	142.7	133.2	152.5
Uganda	Mitooma	1360	2005	113.4	105.7	121.4
Uganda	Mitooma	1360	2010	78.7	72.6	85.5
Uganda	Mitooma	1360	2015	60.0	53.8	66.9
Uganda	Mityana	47087	2000	114.9	107.1	123.7
Uganda	Mityana	47087	2005	97.3	90.5	104.2
Uganda	Mityana	47087	2010	80.9	74.4	87.2
Uganda	Mityana	47087	2015	61.3	54.9	68.7
Uganda	Moroto	772	2000	149.2	137.1	162.7
Uganda	Moroto	772	2005	122.5	112.1	133.5
Uganda	Moroto	772	2010	97.5	88.3	107.5
Uganda	Moroto	772	2015	79.8	69.1	91.1
Uganda	Moyo	3136	2000	143.6	132.8	155.0
Uganda	Moyo	3136	2005	121.6	112.8	131.1
Uganda	Moyo	3136	2010	101.9	93.4	111.1
Uganda	Moyo	3136	2015	79.5	70.4	89.4
Uganda	Mpigi	1363	2000	106.1	99.4	114.1
Uganda	Mpigi	1363	2005	94.9	88.5	101.7
Uganda	Mpigi	1363	2010	80.8	75.1	87.5
Uganda	Mpigi	1363	2015	57.9	52.3	64.7
Uganda	Mubende	47088	2000	133.6	124.1	143.4
Uganda	Mubende	47088	2005	112.2	104.8	120.4
Uganda	Mubende	47088	2010	92.5	85.5	100.5
Uganda	Mubende	47088	2015	72.0	65.0	80.1
Uganda	Mukono	1367	2000	107.7	102.6	113.0
Uganda	Mukono	1367	2005	96.0	91.2	100.9
Uganda	Mukono	1367	2010	80.7	75.9	85.8
Uganda	Mukono	1367	2015	55.7	50.2	61.4
Uganda	Nakapiripirit	773	2000	145.9	133.5	158.9
Uganda	Nakapiripirit	773	2005	119.3	109.3	130.6
Uganda	Nakapiripirit	773	2010	92.7	83.6	102.5
Uganda	Nakapiripirit	773	2015	74.7	65.6	85.0
Uganda	Nakaseke	47089	2000	111.0	103.7	119.1
Uganda	Nakaseke	47089	2005	94.2	87.3	101.3
Uganda	Nakaseke	47089	2010	78.9	72.7	86.1
Uganda	Nakaseke	47089	2015	58.8	52.9	65.8
Uganda	Nakasongola	42195	2000	111.9	102.7	122.7
Uganda	Nakasongola	42195	2005	94.2	87.0	102.2
Uganda	Nakasongola	42195	2010	82.0	75.1	89.3
Uganda	Nakasongola	42195	2015	60.9	54.0	68.5
Uganda	Namayingo	1368	2000	177.0	166.7	187.8
Uganda	Namayingo	1368	2005	143.4	134.6	153.1
Uganda	Namayingo	1368	2010	113.3	105.2	121.7
Uganda	Namayingo	1368	2015	77.4	69.0	85.6
Uganda	Namutumba	774	2000	137.0	128.9	145.5
Uganda	Namutumba	774	2005	115.2	108.7	122.2
Uganda	Namutumba	774	2010	92.6	86.8	98.8
Uganda	Namutumba	774	2015	71.6	65.1	79.4
Uganda	Napak	775	2000	157.3	145.2	169.2
Uganda	Napak	775	2005	129.5	119.0	140.1
Uganda	Napak	775	2010	105.0	95.7	114.7
Uganda	Napak	775	2015	84.3	74.1	95.3
Uganda	Nebbi	776	2000	167.1	155.7	179.4
Uganda	Nebbi	776	2005	142.4	132.8	153.1
Uganda	Nebbi	776	2010	107.9	99.2	117.3
Uganda	Nebbi	776	2015	85.0	76.6	95.3
Uganda	Ngora	777	2000	116.6	109.6	124.0

Admin 0	Admin 1	GAUL Code	Year	Under-5 mortality (per 1,000 livebirths)		
				Estimate	Lower bound, 95% UI	Upper bound, 95% UI
Uganda	Ngora	777	2005	97.7	91.9	104.3
Uganda	Ngora	777	2010	79.5	74.1	85.1
Uganda	Ngora	777	2015	64.4	58.3	71.0
Uganda	Ntoroko	779	2000	159.9	146.9	174.3
Uganda	Ntoroko	779	2005	133.4	122.0	144.2
Uganda	Ntoroko	779	2010	100.2	90.9	109.7
Uganda	Ntoroko	779	2015	76.9	68.0	86.2
Uganda	Ntungamo	3141	2000	153.2	143.8	162.1
Uganda	Ntungamo	3141	2005	118.0	110.7	125.6
Uganda	Ntungamo	3141	2010	79.3	73.6	85.5
Uganda	Ntungamo	3141	2015	59.0	53.2	65.4
Uganda	Nwoya	1364	2000	161.6	148.7	175.5
Uganda	Nwoya	1364	2005	137.0	126.1	148.9
Uganda	Nwoya	1364	2010	109.9	100.1	119.6
Uganda	Nwoya	1364	2015	89.7	79.7	101.4
Uganda	Otuke	781	2000	151.0	139.8	163.0
Uganda	Otuke	781	2005	129.5	120.2	140.3
Uganda	Otuke	781	2010	104.2	95.9	112.6
Uganda	Otuke	781	2015	82.4	73.4	91.9
Uganda	Oyam	782	2000	147.2	137.9	156.8
Uganda	Oyam	782	2005	123.9	116.3	132.2
Uganda	Oyam	782	2010	101.2	94.1	109.1
Uganda	Oyam	782	2015	83.0	74.3	92.8
Uganda	Pader	783	2000	165.5	152.7	179.4
Uganda	Pader	783	2005	138.8	128.1	150.2
Uganda	Pader	783	2010	111.2	102.2	121.2
Uganda	Pader	783	2015	89.8	80.0	100.9
Uganda	Pallisa	1369	2000	120.4	112.7	127.8
Uganda	Pallisa	1369	2005	100.6	94.5	106.9
Uganda	Pallisa	1369	2010	82.2	76.5	88.2
Uganda	Pallisa	1369	2015	66.1	59.8	73.3
Uganda	Rakai	1371	2000	162.7	151.9	173.3
Uganda	Rakai	1371	2005	131.6	122.8	140.9
Uganda	Rakai	1371	2010	105.9	98.2	114.5
Uganda	Rakai	1371	2015	73.3	66.0	82.2
Uganda	Rubirizi	784	2000	147.2	136.5	157.5
Uganda	Rubirizi	784	2005	119.9	110.9	128.8
Uganda	Rubirizi	784	2010	86.3	79.2	94.6
Uganda	Rubirizi	784	2015	65.3	58.6	72.5
Uganda	Rukungiri	42197	2000	138.3	129.2	147.6
Uganda	Rukungiri	42197	2005	107.5	100.5	115.2
Uganda	Rukungiri	42197	2010	73.8	68.2	79.8
Uganda	Rukungiri	42197	2015	56.2	50.2	62.8
Uganda	Serere	1365	2000	118.3	110.5	126.0
Uganda	Serere	1365	2005	99.0	92.4	105.8
Uganda	Serere	1365	2010	80.6	74.8	86.9
Uganda	Serere	1365	2015	65.0	58.5	71.4
Uganda	Sheema	762	2000	151.1	142.6	160.0
Uganda	Sheema	762	2005	119.3	112.1	126.9
Uganda	Sheema	762	2010	82.7	76.7	89.3
Uganda	Sheema	762	2015	63.3	56.8	70.3
Uganda	Sironko	785	2000	111.6	105.1	117.9
Uganda	Sironko	785	2005	90.6	84.7	96.4
Uganda	Sironko	785	2010	75.1	69.4	80.6
Uganda	Sironko	785	2015	59.0	53.6	65.6
Uganda	Soroti	786	2000	117.1	108.5	125.1
Uganda	Soroti	786	2005	97.9	91.3	105.0
Uganda	Soroti	786	2010	80.6	74.7	86.8
Uganda	Soroti	786	2015	65.1	58.6	72.0
Uganda	Ssembabule	3145	2000	157.3	146.8	167.8
Uganda	Ssembabule	3145	2005	130.5	122.3	140.3
Uganda	Ssembabule	3145	2010	110.1	102.0	119.3
Uganda	Ssembabule	3145	2015	77.6	69.9	87.1
Uganda	Tororo	47090	2000	142.1	134.8	149.7
Uganda	Tororo	47090	2005	115.4	109.0	121.7
Uganda	Tororo	47090	2010	91.5	85.9	97.5
Uganda	Tororo	47090	2015	69.8	63.3	76.6
Uganda	Wakiso	42200	2000	95.9	91.3	100.9
Uganda	Wakiso	42200	2005	86.5	82.3	90.9

Admin 0	Admin 1	GAUL Code	Year	Under-5 mortality (per 1,000 livebirths)		
				Estimate	Lower bound, 95% UI	Upper bound, 95% UI
Uganda	Wakiso	42200	2010	75.9	71.7	80.8
Uganda	Wakiso	42200	2015	53.5	48.6	58.9
Uganda	Yumbe	42201	2000	153.2	143.2	163.5
Uganda	Yumbe	42201	2005	129.6	121.2	138.9
Uganda	Yumbe	42201	2010	105.2	96.9	114.1
Uganda	Yumbe	42201	2015	83.9	74.6	94.5
Uganda	Zombo	787	2000	166.2	154.1	178.9
Uganda	Zombo	787	2005	139.8	129.7	151.0
Uganda	Zombo	787	2010	106.1	97.2	115.3
Uganda	Zombo	787	2015	83.2	74.2	93.7
United Republic of Tanzania	Arusha	48357	2000	67.3	62.3	72.1
United Republic of Tanzania	Arusha	48357	2005	55.1	51.1	59.3
United Republic of Tanzania	Arusha	48357	2010	46.4	42.4	50.4
United Republic of Tanzania	Arusha	48357	2015	38.7	34.4	43.2
United Republic of Tanzania	Dar-es-salaam	48358	2000	99.0	91.9	105.9
United Republic of Tanzania	Dar-es-salaam	48358	2005	89.8	83.1	96.5
United Republic of Tanzania	Dar-es-salaam	48358	2010	75.3	69.8	81.5
United Republic of Tanzania	Dar-es-salaam	48358	2015	59.5	53.1	66.4
United Republic of Tanzania	Dodoma	48359	2000	132.4	123.7	141.2
United Republic of Tanzania	Dodoma	48359	2005	104.5	97.4	111.1
United Republic of Tanzania	Dodoma	48359	2010	82.0	75.4	88.3
United Republic of Tanzania	Dodoma	48359	2015	64.7	58.2	71.4
United Republic of Tanzania	Geita	115002	2000	126.1	118.3	134.4
United Republic of Tanzania	Geita	115002	2005	103.8	97.1	111.0
United Republic of Tanzania	Geita	115002	2010	82.0	75.8	88.1
United Republic of Tanzania	Geita	115002	2015	65.0	58.8	71.7
United Republic of Tanzania	Iringa	115003	2000	121.0	112.8	130.1
United Republic of Tanzania	Iringa	115003	2005	95.9	88.6	103.3
United Republic of Tanzania	Iringa	115003	2010	79.3	72.5	86.0
United Republic of Tanzania	Iringa	115003	2015	60.9	54.0	68.1
United Republic of Tanzania	Kagera	115004	2000	154.7	147.7	162.3
United Republic of Tanzania	Kagera	115004	2005	118.8	113.0	124.7
United Republic of Tanzania	Kagera	115004	2010	87.1	82.3	92.2
United Republic of Tanzania	Kagera	115004	2015	67.2	61.7	73.2
United Republic of Tanzania	Kaskazini Pemba	48371	2000	112.2	104.4	120.3
United Republic of Tanzania	Kaskazini Pemba	48371	2005	79.5	73.0	85.7
United Republic of Tanzania	Kaskazini Pemba	48371	2010	56.6	51.5	62.2
United Republic of Tanzania	Kaskazini Pemba	48371	2015	48.5	42.6	54.6
United Republic of Tanzania	Kaskazini Unguja	48372	2000	103.6	96.6	111.4
United Republic of Tanzania	Kaskazini Unguja	48372	2005	71.4	65.8	77.1
United Republic of Tanzania	Kaskazini Unguja	48372	2010	59.5	54.4	64.6
United Republic of Tanzania	Kaskazini Unguja	48372	2015	48.7	43.1	54.3
United Republic of Tanzania	Katavi	115005	2000	140.7	130.3	151.6
United Republic of Tanzania	Katavi	115005	2005	114.9	105.8	124.1
United Republic of Tanzania	Katavi	115005	2010	86.8	79.5	94.7
United Republic of Tanzania	Katavi	115005	2015	72.0	64.8	80.1
United Republic of Tanzania	Kigoma	48362	2000	127.0	119.8	134.8
United Republic of Tanzania	Kigoma	48362	2005	102.5	96.6	109.1
United Republic of Tanzania	Kigoma	48362	2010	80.0	74.8	85.7
United Republic of Tanzania	Kigoma	48362	2015	66.4	60.5	72.7
United Republic of Tanzania	Kilimanjaro	48363	2000	59.5	55.3	63.8
United Republic of Tanzania	Kilimanjaro	48363	2005	54.4	50.4	58.7
United Republic of Tanzania	Kilimanjaro	48363	2010	47.7	43.7	52.2
United Republic of Tanzania	Kilimanjaro	48363	2015	38.8	34.4	43.6
United Republic of Tanzania	Kusini Pemba	48378	2000	105.1	97.3	112.9
United Republic of Tanzania	Kusini Pemba	48378	2005	71.4	66.0	77.3
United Republic of Tanzania	Kusini Pemba	48378	2010	54.1	49.2	59.3
United Republic of Tanzania	Kusini Pemba	48378	2015	46.5	40.9	52.8
United Republic of Tanzania	Kusini Unguja	48379	2000	93.8	87.2	100.3
United Republic of Tanzania	Kusini Unguja	48379	2005	69.9	64.8	75.0
United Republic of Tanzania	Kusini Unguja	48379	2010	61.8	56.8	67.5
United Republic of Tanzania	Kusini Unguja	48379	2015	49.5	43.9	55.3
United Republic of Tanzania	Lindi	48364	2000	156.0	146.6	166.5
United Republic of Tanzania	Lindi	48364	2005	126.7	117.6	135.5
United Republic of Tanzania	Lindi	48364	2010	88.3	80.8	95.6
United Republic of Tanzania	Lindi	48364	2015	65.3	58.1	72.5
United Republic of Tanzania	Manyara	48365	2000	83.1	77.9	88.5
United Republic of Tanzania	Manyara	48365	2005	66.5	62.1	71.1
United Republic of Tanzania	Manyara	48365	2010	54.0	49.9	58.4

Admin 0	Admin 1	GAUL Code	Year	Under-5 mortality (per 1,000 livebirths)		
				Estimate	Lower bound, 95% UI	Upper bound, 95% UI
United Republic of Tanzania	Manyara	48365	2015	45.0	40.4	50.3
United Republic of Tanzania	Mara	48366	2000	159.8	151.3	168.0
United Republic of Tanzania	Mara	48366	2005	132.1	124.9	139.4
United Republic of Tanzania	Mara	48366	2010	97.2	91.1	103.5
United Republic of Tanzania	Mara	48366	2015	72.9	66.3	79.8
United Republic of Tanzania	Mbeya	48367	2000	130.1	123.2	137.7
United Republic of Tanzania	Mbeya	48367	2005	103.5	97.7	109.6
United Republic of Tanzania	Mbeya	48367	2010	80.0	74.9	85.7
United Republic of Tanzania	Mbeya	48367	2015	64.6	58.3	70.9
United Republic of Tanzania	Mjini Magharibi	48382	2000	86.7	80.8	92.9
United Republic of Tanzania	Mjini Magharibi	48382	2005	65.6	60.7	70.6
United Republic of Tanzania	Mjini Magharibi	48382	2010	59.3	54.5	64.1
United Republic of Tanzania	Mjini Magharibi	48382	2015	47.5	42.1	53.3
United Republic of Tanzania	Morogoro	48368	2000	135.4	127.6	144.6
United Republic of Tanzania	Morogoro	48368	2005	107.3	100.1	114.6
United Republic of Tanzania	Morogoro	48368	2010	83.4	77.5	89.4
United Republic of Tanzania	Morogoro	48368	2015	65.3	59.0	72.1
United Republic of Tanzania	Mtwara	48369	2000	150.5	141.3	159.9
United Republic of Tanzania	Mtwara	48369	2005	123.9	115.7	132.3
United Republic of Tanzania	Mtwara	48369	2010	84.3	77.6	91.3
United Republic of Tanzania	Mtwara	48369	2015	63.2	56.5	70.7
United Republic of Tanzania	Mwanza	115006	2000	120.7	113.3	127.5
United Republic of Tanzania	Mwanza	115006	2005	99.2	92.8	105.7
United Republic of Tanzania	Mwanza	115006	2010	79.2	73.7	84.7
United Republic of Tanzania	Mwanza	115006	2015	60.2	53.6	66.3
United Republic of Tanzania	Njombe	115007	2000	114.9	107.0	123.1
United Republic of Tanzania	Njombe	115007	2005	94.3	87.1	101.1
United Republic of Tanzania	Njombe	115007	2010	75.4	69.6	81.7
United Republic of Tanzania	Njombe	115007	2015	56.9	51.0	63.3
United Republic of Tanzania	Pwani	48373	2000	138.1	130.2	146.9
United Republic of Tanzania	Pwani	48373	2005	107.2	100.5	113.8
United Republic of Tanzania	Pwani	48373	2010	83.8	78.2	89.8
United Republic of Tanzania	Pwani	48373	2015	64.9	58.6	71.9
United Republic of Tanzania	Rukwa	115008	2000	157.5	146.9	167.8
United Republic of Tanzania	Rukwa	115008	2005	124.5	116.6	133.4
United Republic of Tanzania	Rukwa	115008	2010	93.2	86.1	100.9
United Republic of Tanzania	Rukwa	115008	2015	74.5	67.1	83.1
United Republic of Tanzania	Ruvuma	48375	2000	132.8	125.0	141.3
United Republic of Tanzania	Ruvuma	48375	2005	111.4	104.0	118.9
United Republic of Tanzania	Ruvuma	48375	2010	82.4	75.9	89.1
United Republic of Tanzania	Ruvuma	48375	2015	58.6	52.8	65.3
United Republic of Tanzania	Shinyanga	115009	2000	119.9	112.1	126.8
United Republic of Tanzania	Shinyanga	115009	2005	100.6	94.2	106.9
United Republic of Tanzania	Shinyanga	115009	2010	78.7	73.4	83.9
United Republic of Tanzania	Shinyanga	115009	2015	59.3	53.0	65.6
United Republic of Tanzania	Simiyu	115010	2000	137.2	128.8	146.7
United Republic of Tanzania	Simiyu	115010	2005	113.5	106.2	121.1
United Republic of Tanzania	Simiyu	115010	2010	80.6	74.5	86.6
United Republic of Tanzania	Simiyu	115010	2015	61.3	54.6	67.9
United Republic of Tanzania	Singida	48377	2000	105.3	98.3	113.0
United Republic of Tanzania	Singida	48377	2005	83.5	78.1	89.8
United Republic of Tanzania	Singida	48377	2010	67.2	62.2	72.7
United Republic of Tanzania	Singida	48377	2015	52.5	47.4	58.6
United Republic of Tanzania	Tabora	48380	2000	122.1	114.4	130.4
United Republic of Tanzania	Tabora	48380	2005	98.3	91.8	105.1
United Republic of Tanzania	Tabora	48380	2010	75.9	70.1	81.9
United Republic of Tanzania	Tabora	48380	2015	57.3	51.5	63.2
United Republic of Tanzania	Tanga	48381	2000	120.8	113.7	128.6
United Republic of Tanzania	Tanga	48381	2005	95.9	89.8	102.2
United Republic of Tanzania	Tanga	48381	2010	71.1	65.9	76.3
United Republic of Tanzania	Tanga	48381	2015	58.1	52.4	64.6
Zambia	Central	3426	2000	145.3	137.9	153.5
Zambia	Central	3426	2005	116.0	109.2	122.4
Zambia	Central	3426	2010	83.6	78.2	89.3
Zambia	Central	3426	2015	63.5	58.1	69.7
Zambia	Copperbelt	3427	2000	114.8	108.0	122.5
Zambia	Copperbelt	3427	2005	96.7	90.6	103.2
Zambia	Copperbelt	3427	2010	76.1	70.1	82.3
Zambia	Copperbelt	3427	2015	55.8	49.9	62.3

Admin 0	Admin 1	GAUL Code	Year	Under-5 mortality (per 1,000 livebirths)		
				Estimate	Lower bound, 95% UI	Upper bound, 95% UI
Zambia	Eastern	3428	2000	173.9	166.2	181.8
Zambia	Eastern	3428	2005	134.9	128.6	141.9
Zambia	Eastern	3428	2010	101.0	95.0	106.7
Zambia	Eastern	3428	2015	76.4	70.7	82.3
Zambia	Luapula	3429	2000	186.9	177.4	196.9
Zambia	Luapula	3429	2005	139.6	131.9	147.7
Zambia	Luapula	3429	2010	106.9	99.8	114.3
Zambia	Luapula	3429	2015	80.3	73.3	87.5
Zambia	Lusaka	3430	2000	108.2	100.9	115.8
Zambia	Lusaka	3430	2005	96.9	89.7	104.4
Zambia	Lusaka	3430	2010	73.9	67.4	80.1
Zambia	Lusaka	3430	2015	56.3	49.9	63.3
Zambia	North-Western	3431	2000	134.1	125.9	142.2
Zambia	North-Western	3431	2005	103.5	97.0	110.3
Zambia	North-Western	3431	2010	76.0	70.0	81.9
Zambia	North-Western	3431	2015	57.4	51.8	63.4
Zambia	Northern	3432	2000	170.7	163.2	177.8
Zambia	Northern	3432	2005	128.2	122.1	134.7
Zambia	Northern	3432	2010	91.3	86.6	96.4
Zambia	Northern	3432	2015	70.5	64.8	76.3
Zambia	Southern	3433	2000	120.8	113.5	128.4
Zambia	Southern	3433	2005	99.5	93.4	106.1
Zambia	Southern	3433	2010	75.8	70.5	81.7
Zambia	Southern	3433	2015	55.8	50.3	61.3
Zambia	Western	3434	2000	153.4	144.3	163.5
Zambia	Western	3434	2005	119.9	112.0	128.0
Zambia	Western	3434	2010	90.8	84.3	98.1
Zambia	Western	3434	2015	71.6	64.4	79.4
Zimbabwe	Bulawayo	3435	2000	54.2	48.7	59.9
Zimbabwe	Bulawayo	3435	2005	61.2	55.5	67.5
Zimbabwe	Bulawayo	3435	2010	58.9	53.0	65.2
Zimbabwe	Bulawayo	3435	2015	44.3	39.2	50.5
Zimbabwe	Harare	3436	2000	63.1	58.2	67.9
Zimbabwe	Harare	3436	2005	74.3	69.0	79.6
Zimbabwe	Harare	3436	2010	76.4	70.6	82.1
Zimbabwe	Harare	3436	2015	55.3	49.4	61.7
Zimbabwe	Manicaland	3437	2000	95.9	90.5	101.5
Zimbabwe	Manicaland	3437	2005	99.7	94.0	105.7
Zimbabwe	Manicaland	3437	2010	91.1	84.7	97.2
Zimbabwe	Manicaland	3437	2015	67.5	61.2	74.6
Zimbabwe	Mashonaland Central	3438	2000	86.9	80.9	93.2
Zimbabwe	Mashonaland Central	3438	2005	89.8	84.2	95.7
Zimbabwe	Mashonaland Central	3438	2010	86.8	81.1	92.7
Zimbabwe	Mashonaland Central	3438	2015	65.6	59.7	71.9
Zimbabwe	Mashonaland East	69550	2000	79.7	74.9	85.0
Zimbabwe	Mashonaland East	69550	2005	85.3	80.2	90.2
Zimbabwe	Mashonaland East	69550	2010	82.9	77.7	88.4
Zimbabwe	Mashonaland East	69550	2015	61.5	56.0	67.4
Zimbabwe	Mashonaland West	3440	2000	85.6	80.1	91.4
Zimbabwe	Mashonaland West	3440	2005	90.1	85.1	95.3
Zimbabwe	Mashonaland West	3440	2010	86.1	80.5	91.3
Zimbabwe	Mashonaland West	3440	2015	65.1	59.2	71.3
Zimbabwe	Masvingo	3441	2000	77.9	72.5	83.8
Zimbabwe	Masvingo	3441	2005	80.6	74.8	86.1
Zimbabwe	Masvingo	3441	2010	78.2	72.5	84.2
Zimbabwe	Masvingo	3441	2015	59.5	53.7	66.0
Zimbabwe	Matabeleland North	69549	2000	69.8	64.7	74.8
Zimbabwe	Matabeleland North	69549	2005	70.6	65.6	75.8
Zimbabwe	Matabeleland North	69549	2010	67.0	62.2	72.1
Zimbabwe	Matabeleland North	69549	2015	50.8	46.2	56.0
Zimbabwe	Matabeleland South	3443	2000	63.0	58.7	67.4
Zimbabwe	Matabeleland South	3443	2005	66.1	61.7	71.0
Zimbabwe	Matabeleland South	3443	2010	61.9	57.2	67.0
Zimbabwe	Matabeleland South	3443	2015	50.2	45.3	55.6
Zimbabwe	Midlands	3444	2000	69.8	65.4	74.9
Zimbabwe	Midlands	3444	2005	77.0	72.4	82.0
Zimbabwe	Midlands	3444	2010	77.2	72.1	82.7
Zimbabwe	Midlands	3444	2015	58.7	53.2	64.3

Supplementary results Table 2. **Under-5 mortality estimates for subnational administrative levels 1 and 2 divisions in Africa, 2000, 2005, 2010, and 2015.** UI = uncertainty interval							
**Admin 0**	**Admin 1**	**Admin 2**	**GAUL Code**	**Year**	**Under-5 mortality (per 1,000 livebirths)**		
					**Estimate**	**Lower bound, 95% UI**	**Upper bound, 95% UI**
Angola	Bengo	Ambriz	4206	2000	135.7	113.0	162.6
Angola	Bengo	Ambriz	4206	2005	108.8	90.8	129.9
Angola	Bengo	Ambriz	4206	2010	88.8	74.0	106.7
Angola	Bengo	Ambriz	4206	2015	55.8	46.0	67.3
Angola	Bengo	Bula Atumba	4207	2000	193.0	162.1	227.6
Angola	Bengo	Bula Atumba	4207	2005	147.2	124.0	174.3
Angola	Bengo	Bula Atumba	4207	2010	105.4	88.5	125.6
Angola	Bengo	Bula Atumba	4207	2015	75.5	62.8	90.7
Angola	Bengo	Dande	4208	2000	146.2	127.4	167.1
Angola	Bengo	Dande	4208	2005	116.3	101.6	133.3
Angola	Bengo	Dande	4208	2010	91.9	78.9	106.4
Angola	Bengo	Dande	4208	2015	58.9	50.1	69.5
Angola	Bengo	Dembos	4209	2000	199.4	166.4	235.8
Angola	Bengo	Dembos	4209	2005	149.1	125.4	177.8
Angola	Bengo	Dembos	4209	2010	108.8	90.4	130.5
Angola	Bengo	Dembos	4209	2015	75.3	62.0	91.3
Angola	Bengo	Icolo E Bengo	4210	2000	144.1	123.5	165.4
Angola	Bengo	Icolo E Bengo	4210	2005	113.4	98.3	130.0
Angola	Bengo	Icolo E Bengo	4210	2010	88.0	75.8	103.1
Angola	Bengo	Icolo E Bengo	4210	2015	58.3	49.5	69.0
Angola	Bengo	Nambuangongo	4211	2000	156.1	133.2	181.9
Angola	Bengo	Nambuangongo	4211	2005	124.5	105.5	145.5
Angola	Bengo	Nambuangongo	4211	2010	95.2	80.3	112.5
Angola	Bengo	Nambuangongo	4211	2015	62.4	51.9	74.2
Angola	Bengo	Pango Aluquem	4212	2000	200.7	169.6	237.5
Angola	Bengo	Pango Aluquem	4212	2005	153.0	128.5	183.3
Angola	Bengo	Pango Aluquem	4212	2010	110.6	92.8	132.2
Angola	Bengo	Pango Aluquem	4212	2015	76.0	63.6	91.2
Angola	Bengo	Quissama	4213	2000	161.6	139.5	187.6
Angola	Bengo	Quissama	4213	2005	133.3	115.4	153.2
Angola	Bengo	Quissama	4213	2010	99.1	85.0	115.5
Angola	Bengo	Quissama	4213	2015	65.4	55.3	77.3
Angola	Benguela	Baia Farta	4214	2000	169.6	139.0	202.0
Angola	Benguela	Baia Farta	4214	2005	140.6	117.4	168.6
Angola	Benguela	Baia Farta	4214	2010	107.6	89.6	127.6
Angola	Benguela	Baia Farta	4214	2015	70.5	57.6	84.4
Angola	Benguela	Balombo	4215	2000	190.0	163.2	220.9
Angola	Benguela	Balombo	4215	2005	158.6	136.3	183.1
Angola	Benguela	Balombo	4215	2010	105.2	89.3	122.8
Angola	Benguela	Balombo	4215	2015	71.9	59.8	85.0
Angola	Benguela	Benguela	4216	2000	141.7	119.3	166.6
Angola	Benguela	Benguela	4216	2005	122.3	102.6	142.1
Angola	Benguela	Benguela	4216	2010	96.0	80.8	112.7
Angola	Benguela	Benguela	4216	2015	62.6	51.8	74.9
Angola	Benguela	Bocoio	4217	2000	190.4	164.3	221.4
Angola	Benguela	Bocoio	4217	2005	157.1	136.3	182.0
Angola	Benguela	Bocoio	4217	2010	112.1	95.2	130.5
Angola	Benguela	Bocoio	4217	2015	74.6	62.2	87.5
Angola	Benguela	Caimbambo	4218	2000	203.1	172.6	235.8
Angola	Benguela	Caimbambo	4218	2005	169.9	145.0	196.9
Angola	Benguela	Caimbambo	4218	2010	126.3	107.5	147.8
Angola	Benguela	Caimbambo	4218	2015	83.4	69.4	97.9
Angola	Benguela	Chongoroi	4219	2000	199.2	171.7	228.1
Angola	Benguela	Chongoroi	4219	2005	164.7	143.1	189.7
Angola	Benguela	Chongoroi	4219	2010	124.4	106.6	144.9
Angola	Benguela	Chongoroi	4219	2015	83.2	70.2	97.3
Angola	Benguela	Cubal	4220	2000	210.4	179.7	243.2
Angola	Benguela	Cubal	4220	2005	177.1	152.0	205.3
Angola	Benguela	Cubal	4220	2010	129.0	110.5	150.0
Angola	Benguela	Cubal	4220	2015	86.9	72.8	102.1
Angola	Benguela	Ganda	4221	2000	207.7	178.1	240.6
Angola	Benguela	Ganda	4221	2005	172.7	149.3	200.7
Angola	Benguela	Ganda	4221	2010	122.3	104.8	143.4
Angola	Benguela	Ganda	4221	2015	82.2	69.6	95.6
Angola	Benguela	Lobito	4222	2000	135.0	114.8	157.0
Angola	Benguela	Lobito	4222	2005	115.5	98.8	133.2
Angola	Benguela	Lobito	4222	2010	93.2	78.7	109.7
Angola	Benguela	Lobito	4222	2015	61.3	51.2	72.5
Angola	Bie	Andulo	4223	2000	145.4	121.4	169.6
Angola	Bie	Andulo	4223	2005	119.9	101.6	140.2
Angola	Bie	Andulo	4223	2010	83.6	69.9	98.4
Angola	Bie	Andulo	4223	2015	58.4	48.2	69.9
Angola	Bie	Camacupa	4224	2000	130.2	106.6	158.3
Angola	Bie	Camacupa	4224	2005	110.5	90.7	134.3
Angola	Bie	Camacupa	4224	2010	85.6	69.8	102.4
Angola	Bie	Camacupa	4224	2015	55.5	44.9	66.8
Angola	Bie	Catabola	4225	2000	139.6	113.9	168.1
Angola	Bie	Catabola	4225	2005	118.9	98.5	144.4
Angola	Bie	Catabola	4225	2010	88.0	72.2	106.4
Angola	Bie	Catabola	4225	2015	60.2	49.0	73.2
Angola	Bie	Chinguar	4226	2000	166.4	141.2	194.8
Angola	Bie	Chinguar	4226	2005	132.7	113.5	154.3

Admin 0	Admin 1	Admin 2	GAUL Code	Year	Under-5 mortality (per 1,000 livebirths)		
					Estimate	Lower bound, 95% UI	Upper bound, 95% UI
Angola	Bie	Chinguar	4226	2010	94.2	80.5	109.9
Angola	Bie	Chinguar	4226	2015	65.8	55.1	78.2
Angola	Bie	Chitembo	4227	2000	162.0	135.2	194.6
Angola	Bie	Chitembo	4227	2005	130.8	109.3	155.6
Angola	Bie	Chitembo	4227	2010	97.0	80.8	116.7
Angola	Bie	Chitembo	4227	2015	66.3	54.6	80.0
Angola	Bie	Cuemba	4228	2000	114.5	91.8	140.2
Angola	Bie	Cuemba	4228	2005	100.0	80.5	121.8
Angola	Bie	Cuemba	4228	2010	76.8	61.7	94.6
Angola	Bie	Cuemba	4228	2015	50.8	40.8	61.8
Angola	Bie	Cunhinga	4229	2000	146.0	122.4	172.5
Angola	Bie	Cunhinga	4229	2005	120.4	101.3	143.0
Angola	Bie	Cunhinga	4229	2010	85.5	71.6	101.0
Angola	Bie	Cunhinga	4229	2015	59.9	49.5	71.1
Angola	Bie	Kuito	4230	2000	142.6	117.1	170.0
Angola	Bie	Kuito	4230	2005	117.6	98.2	140.9
Angola	Bie	Kuito	4230	2010	86.4	70.9	104.1
Angola	Bie	Kuito	4230	2015	58.1	47.4	71.2
Angola	Bie	N'harea	4231	2000	127.9	104.1	154.5
Angola	Bie	N'harea	4231	2005	107.3	88.1	130.2
Angola	Bie	N'harea	4231	2010	77.7	62.9	93.8
Angola	Bie	N'harea	4231	2015	52.7	42.2	63.7
Angola	Cabinda	Belize	4232	2000	170.7	153.4	190.8
Angola	Cabinda	Belize	4232	2005	131.9	118.7	148.0
Angola	Cabinda	Belize	4232	2010	95.8	85.1	107.6
Angola	Cabinda	Belize	4232	2015	61.8	53.7	70.6
Angola	Cabinda	Buco-zau	4233	2000	165.3	147.4	185.1
Angola	Cabinda	Buco-zau	4233	2005	124.6	110.8	140.0
Angola	Cabinda	Buco-zau	4233	2010	93.0	81.9	104.9
Angola	Cabinda	Buco-zau	4233	2015	61.6	53.2	70.8
Angola	Cabinda	Cabinda	4234	2000	151.8	129.9	175.8
Angola	Cabinda	Cabinda	4234	2005	108.6	93.6	127.3
Angola	Cabinda	Cabinda	4234	2010	88.5	76.5	103.0
Angola	Cabinda	Cabinda	4234	2015	57.5	48.6	67.6
Angola	Cabinda	Cacongo (landana)	4235	2000	144.7	128.2	162.8
Angola	Cabinda	Cacongo (landana)	4235	2005	107.7	95.3	122.4
Angola	Cabinda	Cacongo (landana)	4235	2010	85.6	75.2	98.2
Angola	Cabinda	Cacongo (landana)	4235	2015	56.6	48.6	65.5
Angola	Cuando Cubango	Calai	4236	2000	110.4	93.9	130.7
Angola	Cuando Cubango	Calai	4236	2005	100.7	85.2	118.2
Angola	Cuando Cubango	Calai	4236	2010	81.1	68.1	96.6
Angola	Cuando Cubango	Calai	4236	2015	51.8	42.9	62.4
Angola	Cuando Cubango	Cuangar	4237	2000	123.7	105.3	146.5
Angola	Cuando Cubango	Cuangar	4237	2005	114.9	98.0	135.3
Angola	Cuando Cubango	Cuangar	4237	2010	88.2	74.2	105.6
Angola	Cuando Cubango	Cuangar	4237	2015	55.2	46.1	66.9
Angola	Cuando Cubango	Cuchi	4238	2000	177.4	145.1	215.5
Angola	Cuando Cubango	Cuchi	4238	2005	147.9	121.8	179.4
Angola	Cuando Cubango	Cuchi	4238	2010	109.1	89.3	133.5
Angola	Cuando Cubango	Cuchi	4238	2015	69.9	56.8	87.1
Angola	Cuando Cubango	Cuito Cuanavale	4239	2000	142.1	115.1	172.6
Angola	Cuando Cubango	Cuito Cuanavale	4239	2005	117.1	94.8	143.4
Angola	Cuando Cubango	Cuito Cuanavale	4239	2010	90.6	73.5	110.7
Angola	Cuando Cubango	Cuito Cuanavale	4239	2015	59.5	47.8	73.9
Angola	Cuando Cubango	Dirico	4240	2000	117.1	99.1	138.4
Angola	Cuando Cubango	Dirico	4240	2005	102.1	86.3	120.4
Angola	Cuando Cubango	Dirico	4240	2010	81.4	68.7	96.2
Angola	Cuando Cubango	Dirico	4240	2015	52.6	43.4	63.1
Angola	Cuando Cubango	Mavinga	4241	2000	134.2	110.6	160.6
Angola	Cuando Cubango	Mavinga	4241	2005	112.4	93.3	134.7
Angola	Cuando Cubango	Mavinga	4241	2010	93.0	76.6	111.2
Angola	Cuando Cubango	Mavinga	4241	2015	60.7	49.8	73.2
Angola	Cuando Cubango	Menongue	4242	2000	158.8	133.0	189.2
Angola	Cuando Cubango	Menongue	4242	2005	135.4	113.9	160.7
Angola	Cuando Cubango	Menongue	4242	2010	99.3	82.9	119.0
Angola	Cuando Cubango	Menongue	4242	2015	62.1	51.2	75.0
Angola	Cuando Cubango	Nankova	4243	2000	135.4	109.7	170.4
Angola	Cuando Cubango	Nankova	4243	2005	119.2	96.1	148.1
Angola	Cuando Cubango	Nankova	4243	2010	93.2	74.8	116.6
Angola	Cuando Cubango	Nankova	4243	2015	58.1	46.0	72.7
Angola	Cuando Cubango	Rivungo	4244	2000	144.8	124.8	167.9
Angola	Cuando Cubango	Rivungo	4244	2005	120.6	103.7	139.3
Angola	Cuando Cubango	Rivungo	4244	2010	98.2	83.7	114.0
Angola	Cuando Cubango	Rivungo	4244	2015	64.6	54.6	76.3
Angola	Cuanza Sul	Amboim	4245	2000	233.6	197.6	273.5
Angola	Cuanza Sul	Amboim	4245	2005	189.2	161.0	222.6
Angola	Cuanza Sul	Amboim	4245	2010	128.6	106.7	152.6
Angola	Cuanza Sul	Amboim	4245	2015	89.8	73.2	107.2
Angola	Cuanza Sul	Cassongue	4246	2000	198.8	169.6	232.1
Angola	Cuanza Sul	Cassongue	4246	2005	169.8	145.4	197.6
Angola	Cuanza Sul	Cassongue	4246	2010	109.0	92.7	128.5
Angola	Cuanza Sul	Cassongue	4246	2015	81.2	67.4	96.8
Angola	Cuanza Sul	Conda	4247	2000	225.2	193.4	261.6
Angola	Cuanza Sul	Conda	4247	2005	184.7	159.6	215.4
Angola	Cuanza Sul	Conda	4247	2010	126.5	107.0	149.1

Admin 0	Admin 1	Admin 2	GAUL Code	Year	Under-5 mortality (per 1,000 livebirths) Estimate	Lower bound, 95% UI	Upper bound, 95% UI
Angola	Cuanza Sul	Conda	4247	2015	90.3	75.9	107.1
Angola	Cuanza Sul	Ebo	4248	2000	215.1	181.0	252.2
Angola	Cuanza Sul	Ebo	4248	2005	179.9	153.4	210.0
Angola	Cuanza Sul	Ebo	4248	2010	122.8	102.6	144.2
Angola	Cuanza Sul	Ebo	4248	2015	92.5	76.7	110.1
Angola	Cuanza Sul	Libolo	4249	2000	208.0	174.8	246.2
Angola	Cuanza Sul	Libolo	4249	2005	170.6	142.4	202.4
Angola	Cuanza Sul	Libolo	4249	2010	119.3	99.4	144.4
Angola	Cuanza Sul	Libolo	4249	2015	88.1	72.0	107.0
Angola	Cuanza Sul	Mussende	4250	2000	163.4	138.7	191.6
Angola	Cuanza Sul	Mussende	4250	2005	137.3	115.6	161.2
Angola	Cuanza Sul	Mussende	4250	2010	96.2	80.9	113.6
Angola	Cuanza Sul	Mussende	4250	2015	71.3	58.9	85.4
Angola	Cuanza Sul	Porto Amboim	4251	2000	190.6	160.3	227.5
Angola	Cuanza Sul	Porto Amboim	4251	2005	156.1	132.5	183.8
Angola	Cuanza Sul	Porto Amboim	4251	2010	110.3	91.6	132.9
Angola	Cuanza Sul	Porto Amboim	4251	2015	69.8	57.2	84.9
Angola	Cuanza Sul	Quibala	4252	2000	194.4	164.4	227.7
Angola	Cuanza Sul	Quibala	4252	2005	163.7	137.0	193.1
Angola	Cuanza Sul	Quibala	4252	2010	111.5	93.2	133.0
Angola	Cuanza Sul	Quibala	4252	2015	81.5	67.8	97.7
Angola	Cuanza Sul	Quilenda	4253	2000	229.9	195.0	270.6
Angola	Cuanza Sul	Quilenda	4253	2005	188.9	159.3	221.1
Angola	Cuanza Sul	Quilenda	4253	2010	129.5	107.5	154.3
Angola	Cuanza Sul	Quilenda	4253	2015	93.1	76.3	110.8
Angola	Cuanza Sul	Seles	4254	2000	213.9	183.8	250.7
Angola	Cuanza Sul	Seles	4254	2005	174.7	149.6	205.0
Angola	Cuanza Sul	Seles	4254	2010	119.5	101.0	140.9
Angola	Cuanza Sul	Seles	4254	2015	87.7	73.9	104.4
Angola	Cuanza Sul	Sumbe	4255	2000	202.3	170.7	239.2
Angola	Cuanza Sul	Sumbe	4255	2005	156.0	131.9	183.0
Angola	Cuanza Sul	Sumbe	4255	2010	112.4	95.1	134.0
Angola	Cuanza Sul	Sumbe	4255	2015	75.0	63.0	89.6
Angola	Cuanza Sul	Waku Kungu	4256	2000	192.6	161.7	227.0
Angola	Cuanza Sul	Waku Kungu	4256	2005	163.2	137.3	193.6
Angola	Cuanza Sul	Waku Kungu	4256	2010	107.4	89.7	127.1
Angola	Cuanza Sul	Waku Kungu	4256	2015	78.9	65.0	94.6
Angola	Cunene	Cahama	4257	2000	132.6	110.7	156.2
Angola	Cunene	Cahama	4257	2005	120.5	102.1	143.0
Angola	Cunene	Cahama	4257	2010	94.8	80.1	113.5
Angola	Cunene	Cahama	4257	2015	58.2	48.4	69.2
Angola	Cunene	Cuanhama	4258	2000	127.5	108.5	149.2
Angola	Cunene	Cuanhama	4258	2005	124.7	105.8	146.8
Angola	Cunene	Cuanhama	4258	2010	94.1	79.1	112.0
Angola	Cunene	Cuanhama	4258	2015	57.1	47.9	68.3
Angola	Cunene	Curoca	4259	2000	114.4	95.4	136.2
Angola	Cunene	Curoca	4259	2005	104.0	87.9	122.8
Angola	Cunene	Curoca	4259	2010	83.0	69.5	97.9
Angola	Cunene	Curoca	4259	2015	50.9	42.6	60.6
Angola	Cunene	Cuvelai	4260	2000	147.7	123.3	178.5
Angola	Cunene	Cuvelai	4260	2005	137.6	114.6	167.3
Angola	Cunene	Cuvelai	4260	2010	107.4	89.1	131.8
Angola	Cunene	Cuvelai	4260	2015	65.0	53.3	79.4
Angola	Cunene	Namacunde	4261	2000	116.6	99.9	135.9
Angola	Cunene	Namacunde	4261	2005	114.8	98.0	134.1
Angola	Cunene	Namacunde	4261	2010	86.1	73.0	101.7
Angola	Cunene	Namacunde	4261	2015	53.6	44.7	63.1
Angola	Cunene	Ombadja	4262	2000	115.2	97.7	135.1
Angola	Cunene	Ombadja	4262	2005	112.8	97.0	133.8
Angola	Cunene	Ombadja	4262	2010	89.1	76.1	105.7
Angola	Cunene	Ombadja	4262	2015	52.1	43.6	61.7
Angola	Huambo	Bailundo	4263	2000	184.2	157.9	213.8
Angola	Huambo	Bailundo	4263	2005	154.9	133.4	180.1
Angola	Huambo	Bailundo	4263	2010	97.2	82.8	113.7
Angola	Huambo	Bailundo	4263	2015	71.0	59.8	84.3
Angola	Huambo	Caala	4264	2000	209.4	183.6	240.3
Angola	Huambo	Caala	4264	2005	162.1	142.4	184.6
Angola	Huambo	Caala	4264	2010	104.6	90.8	120.8
Angola	Huambo	Caala	4264	2015	76.7	65.9	89.4
Angola	Huambo	Ekunha	4265	2000	198.5	173.0	227.9
Angola	Huambo	Ekunha	4265	2005	162.2	142.2	185.3
Angola	Huambo	Ekunha	4265	2010	99.0	85.4	115.4
Angola	Huambo	Ekunha	4265	2015	73.2	62.2	86.4
Angola	Huambo	Huambo	4266	2000	192.1	168.9	219.1
Angola	Huambo	Huambo	4266	2005	148.2	130.6	169.3
Angola	Huambo	Huambo	4266	2010	94.9	82.2	109.3
Angola	Huambo	Huambo	4266	2015	68.8	58.7	79.9
Angola	Huambo	Katchiungo	4267	2000	185.0	160.5	213.6
Angola	Huambo	Katchiungo	4267	2005	144.9	125.4	166.6
Angola	Huambo	Katchiungo	4267	2010	96.8	83.0	111.3
Angola	Huambo	Katchiungo	4267	2015	71.0	60.2	83.1
Angola	Huambo	Londuimbali	4268	2000	200.0	171.7	232.1
Angola	Huambo	Londuimbali	4268	2005	171.2	146.5	199.6
Angola	Huambo	Londuimbali	4268	2010	102.2	86.6	119.2
Angola	Huambo	Londuimbali	4268	2015	76.9	64.1	91.3

Admin 0	Admin 1	Admin 2	GAUL Code	Year	Under-5 mortality (per 1,000 livebirths)		
					Estimate	Lower bound, 95% UI	Upper bound, 95% UI
Angola	Huambo	Longonjo	4269	2000	218.7	188.3	250.8
Angola	Huambo	Longonjo	4269	2005	185.2	161.5	213.1
Angola	Huambo	Longonjo	4269	2010	113.7	98.0	132.3
Angola	Huambo	Longonjo	4269	2015	85.4	72.1	100.0
Angola	Huambo	Mungo	4270	2000	167.5	139.8	198.4
Angola	Huambo	Mungo	4270	2005	140.6	117.2	165.2
Angola	Huambo	Mungo	4270	2010	90.3	75.6	107.4
Angola	Huambo	Mungo	4270	2015	66.1	54.5	79.4
Angola	Huambo	Tchikala-tcholo.	4271	2000	195.3	170.2	223.9
Angola	Huambo	Tchikala-tcholo.	4271	2005	153.9	135.2	175.8
Angola	Huambo	Tchikala-tcholo.	4271	2010	102.4	88.5	118.3
Angola	Huambo	Tchikala-tcholo.	4271	2015	75.0	63.8	87.5
Angola	Huambo	Tchindjenje	4272	2000	216.5	184.4	251.7
Angola	Huambo	Tchindjenje	4272	2005	189.7	163.7	219.1
Angola	Huambo	Tchindjenje	4272	2010	116.8	99.8	136.5
Angola	Huambo	Tchindjenje	4272	2015	85.3	72.0	100.0
Angola	Huambo	Ukuma	4273	2000	214.3	184.8	248.7
Angola	Huambo	Ukuma	4273	2005	185.1	159.9	214.3
Angola	Huambo	Ukuma	4273	2010	109.9	94.7	127.9
Angola	Huambo	Ukuma	4273	2015	82.5	69.7	97.0
Angola	Huila	Caconda	4274	2000	222.5	192.7	256.4
Angola	Huila	Caconda	4274	2005	186.6	162.1	214.2
Angola	Huila	Caconda	4274	2010	132.8	114.7	154.2
Angola	Huila	Caconda	4274	2015	90.5	76.9	106.5
Angola	Huila	Cacula	4275	2000	177.6	156.3	202.3
Angola	Huila	Cacula	4275	2005	144.3	128.1	163.5
Angola	Huila	Cacula	4275	2010	114.2	99.7	130.1
Angola	Huila	Cacula	4275	2015	76.2	65.2	88.3
Angola	Huila	Caluquembe	4276	2000	215.4	190.3	244.0
Angola	Huila	Caluquembe	4276	2005	180.2	158.9	201.8
Angola	Huila	Caluquembe	4276	2010	132.3	116.3	150.7
Angola	Huila	Caluquembe	4276	2015	90.0	77.4	104.1
Angola	Huila	Chibia	4277	2000	177.6	155.5	201.5
Angola	Huila	Chibia	4277	2005	147.4	129.4	165.9
Angola	Huila	Chibia	4277	2010	109.8	94.7	125.8
Angola	Huila	Chibia	4277	2015	71.9	61.3	84.8
Angola	Huila	Chicomba	4278	2000	224.2	195.3	254.8
Angola	Huila	Chicomba	4278	2005	191.7	167.4	218.7
Angola	Huila	Chicomba	4278	2010	148.9	128.9	173.7
Angola	Huila	Chicomba	4278	2015	102.2	85.9	120.2
Angola	Huila	Chipindo	4279	2000	217.3	182.4	257.2
Angola	Huila	Chipindo	4279	2005	180.1	151.6	215.7
Angola	Huila	Chipindo	4279	2010	129.6	108.1	155.4
Angola	Huila	Chipindo	4279	2015	88.7	73.5	107.0
Angola	Huila	Gambos	4280	2000	169.4	144.9	193.7
Angola	Huila	Gambos	4280	2005	143.8	124.1	165.6
Angola	Huila	Gambos	4280	2010	109.6	93.9	128.1
Angola	Huila	Gambos	4280	2015	69.8	59.3	81.6
Angola	Huila	Humpata	4281	2000	133.4	113.8	153.2
Angola	Huila	Humpata	4281	2005	114.3	99.4	130.6
Angola	Huila	Humpata	4281	2010	91.1	78.2	106.2
Angola	Huila	Humpata	4281	2015	57.3	48.3	67.9
Angola	Huila	Jamba	4282	2000	192.5	159.1	232.5
Angola	Huila	Jamba	4282	2005	164.0	134.2	200.3
Angola	Huila	Jamba	4282	2010	132.0	108.0	159.6
Angola	Huila	Jamba	4282	2015	83.5	68.7	101.6
Angola	Huila	Kuvango	4283	2000	191.2	156.8	231.0
Angola	Huila	Kuvango	4283	2005	157.5	129.5	189.4
Angola	Huila	Kuvango	4283	2010	117.7	96.7	143.0
Angola	Huila	Kuvango	4283	2015	77.3	63.1	94.6
Angola	Huila	Lubango	4284	2000	139.1	119.3	159.2
Angola	Huila	Lubango	4284	2005	118.2	102.2	135.2
Angola	Huila	Lubango	4284	2010	91.9	78.6	106.2
Angola	Huila	Lubango	4284	2015	58.3	49.4	68.7
Angola	Huila	Matala	4285	2000	189.2	160.6	223.1
Angola	Huila	Matala	4285	2005	163.1	139.6	190.4
Angola	Huila	Matala	4285	2010	134.8	115.1	157.4
Angola	Huila	Matala	4285	2015	84.0	70.2	100.0
Angola	Huila	Quilengues	4286	2000	198.0	171.1	226.4
Angola	Huila	Quilengues	4286	2005	158.2	136.8	180.3
Angola	Huila	Quilengues	4286	2010	119.6	102.6	137.6
Angola	Huila	Quilengues	4286	2015	84.0	71.1	98.1
Angola	Huila	Quipungo	4287	2000	199.9	174.1	228.3
Angola	Huila	Quipungo	4287	2005	169.6	149.6	192.7
Angola	Huila	Quipungo	4287	2010	131.7	114.3	150.9
Angola	Huila	Quipungo	4287	2015	84.0	71.4	97.6
Angola	Kuanza Norte	Ambaca	4288	2000	164.8	142.5	190.0
Angola	Kuanza Norte	Ambaca	4288	2005	122.7	106.1	140.8
Angola	Kuanza Norte	Ambaca	4288	2010	87.9	74.6	102.9
Angola	Kuanza Norte	Ambaca	4288	2015	65.4	54.8	77.5
Angola	Kuanza Norte	Banga	4289	2000	180.3	153.1	214.1
Angola	Kuanza Norte	Banga	4289	2005	141.1	119.6	166.6
Angola	Kuanza Norte	Banga	4289	2010	100.7	84.4	120.1
Angola	Kuanza Norte	Banga	4289	2015	74.7	62.3	90.6
Angola	Kuanza Norte	Bolongongo	4290	2000	185.3	158.2	219.1

Admin 0	Admin 1	Admin 2	GAUL Code	Year	Under-5 mortality (per 1,000 livebirths)		
					Estimate	Lower bound, 95% UI	Upper bound, 95% UI
Angola	Kuanza Norte	Bolongongo	4290	2005	142.0	120.9	166.3
Angola	Kuanza Norte	Bolongongo	4290	2010	100.0	84.1	118.2
Angola	Kuanza Norte	Bolongongo	4290	2015	72.9	61.2	87.2
Angola	Kuanza Norte	Cambambe	4291	2000	198.4	168.3	231.8
Angola	Kuanza Norte	Cambambe	4291	2005	155.7	131.3	183.0
Angola	Kuanza Norte	Cambambe	4291	2010	111.6	94.8	132.2
Angola	Kuanza Norte	Cambambe	4291	2015	79.0	65.3	94.3
Angola	Kuanza Norte	Cazengo (ndalatando)	4292	2000	195.6	165.3	229.5
Angola	Kuanza Norte	Cazengo (ndalatando)	4292	2005	150.2	125.8	176.1
Angola	Kuanza Norte	Cazengo (ndalatando)	4292	2010	105.9	87.7	125.9
Angola	Kuanza Norte	Cazengo (ndalatando)	4292	2015	74.1	61.1	88.5
Angola	Kuanza Norte	Golungo Alto	4293	2000	205.6	172.9	245.1
Angola	Kuanza Norte	Golungo Alto	4293	2005	159.6	132.9	192.8
Angola	Kuanza Norte	Golungo Alto	4293	2010	113.6	94.0	137.2
Angola	Kuanza Norte	Golungo Alto	4293	2015	79.1	65.3	94.9
Angola	Kuanza Norte	Gonguembo	4294	2000	193.5	163.7	229.4
Angola	Kuanza Norte	Gonguembo	4294	2005	150.8	126.5	178.1
Angola	Kuanza Norte	Gonguembo	4294	2010	108.2	90.0	129.4
Angola	Kuanza Norte	Gonguembo	4294	2015	78.1	64.8	94.2
Angola	Kuanza Norte	Lucala	4295	2000	184.0	156.1	216.1
Angola	Kuanza Norte	Lucala	4295	2005	149.1	124.7	176.4
Angola	Kuanza Norte	Lucala	4295	2010	107.6	88.4	127.6
Angola	Kuanza Norte	Lucala	4295	2015	79.1	65.5	94.9
Angola	Kuanza Norte	Quiculungo	4296	2000	175.7	149.8	206.4
Angola	Kuanza Norte	Quiculungo	4296	2005	133.3	113.1	156.3
Angola	Kuanza Norte	Quiculungo	4296	2010	94.7	79.0	112.9
Angola	Kuanza Norte	Quiculungo	4296	2015	69.6	57.5	84.1
Angola	Kuanza Norte	Samba Caju	4297	2000	167.5	144.1	196.7
Angola	Kuanza Norte	Samba Caju	4297	2005	132.7	113.3	156.0
Angola	Kuanza Norte	Samba Caju	4297	2010	95.4	80.5	113.9
Angola	Kuanza Norte	Samba Caju	4297	2015	72.0	60.1	86.9
Angola	Luanda	Cacuaco	4298	2000	107.8	96.9	120.4
Angola	Luanda	Cacuaco	4298	2005	89.5	80.6	99.5
Angola	Luanda	Cacuaco	4298	2010	75.5	66.7	85.2
Angola	Luanda	Cacuaco	4298	2015	49.1	42.4	57.6
Angola	Luanda	Luanda	4299	2000	97.5	87.2	108.2
Angola	Luanda	Luanda	4299	2005	82.6	74.6	91.9
Angola	Luanda	Luanda	4299	2010	72.8	64.7	82.4
Angola	Luanda	Luanda	4299	2015	47.7	40.9	55.4
Angola	Luanda	Viana	4300	2000	106.1	94.9	117.8
Angola	Luanda	Viana	4300	2005	88.8	80.6	98.0
Angola	Luanda	Viana	4300	2010	75.4	67.3	85.0
Angola	Luanda	Viana	4300	2015	49.7	43.0	58.0
Angola	Lunda Norte	Cambulo	4301	2000	145.0	123.7	170.7
Angola	Lunda Norte	Cambulo	4301	2005	128.0	109.6	152.1
Angola	Lunda Norte	Cambulo	4301	2010	93.7	78.5	111.5
Angola	Lunda Norte	Cambulo	4301	2015	68.3	57.5	81.4
Angola	Lunda Norte	Capenda Camulemba	4302	2000	115.5	93.9	144.2
Angola	Lunda Norte	Capenda Camulemba	4302	2005	96.1	77.7	119.7
Angola	Lunda Norte	Capenda Camulemba	4302	2010	68.0	54.8	84.1
Angola	Lunda Norte	Capenda Camulemba	4302	2015	48.3	38.7	59.8
Angola	Lunda Norte	Caungula	4303	2000	109.2	87.2	135.8
Angola	Lunda Norte	Caungula	4303	2005	91.7	73.4	114.2
Angola	Lunda Norte	Caungula	4303	2010	64.9	51.8	79.4
Angola	Lunda Norte	Caungula	4303	2015	46.0	36.3	56.9
Angola	Lunda Norte	Chitato	4304	2000	141.2	120.1	165.9
Angola	Lunda Norte	Chitato	4304	2005	122.2	103.1	143.9
Angola	Lunda Norte	Chitato	4304	2010	87.6	74.3	103.6
Angola	Lunda Norte	Chitato	4304	2015	61.5	51.4	72.7
Angola	Lunda Norte	Cuango	4305	2000	108.1	87.3	131.3
Angola	Lunda Norte	Cuango	4305	2005	89.0	72.5	109.5
Angola	Lunda Norte	Cuango	4305	2010	63.4	50.9	77.3
Angola	Lunda Norte	Cuango	4305	2015	46.8	37.8	57.1
Angola	Lunda Norte	Cuilo	4306	2000	117.3	94.9	142.9
Angola	Lunda Norte	Cuilo	4306	2005	102.2	83.0	124.3
Angola	Lunda Norte	Cuilo	4306	2010	72.7	59.6	88.2
Angola	Lunda Norte	Cuilo	4306	2015	49.8	40.5	60.9
Angola	Lunda Norte	Lubalo	4307	2000	112.2	91.9	136.5
Angola	Lunda Norte	Lubalo	4307	2005	93.4	76.9	113.1
Angola	Lunda Norte	Lubalo	4307	2010	66.6	55.0	81.0
Angola	Lunda Norte	Lubalo	4307	2015	47.4	38.4	58.2
Angola	Lunda Norte	Lucapa	4308	2000	125.2	101.6	152.2
Angola	Lunda Norte	Lucapa	4308	2005	108.6	88.3	131.4
Angola	Lunda Norte	Lucapa	4308	2010	77.9	63.3	94.4
Angola	Lunda Norte	Lucapa	4308	2015	54.8	43.6	67.0
Angola	Lunda Norte	Xa-muteba	4309	2000	112.4	93.2	136.5
Angola	Lunda Norte	Xa-muteba	4309	2005	95.1	78.4	116.5
Angola	Lunda Norte	Xa-muteba	4309	2010	65.8	54.5	80.0
Angola	Lunda Norte	Xa-muteba	4309	2015	47.2	38.9	57.4
Angola	Lunda Sul	Cacolo	4310	2000	104.3	86.2	127.6
Angola	Lunda Sul	Cacolo	4310	2005	88.8	73.4	108.4
Angola	Lunda Sul	Cacolo	4310	2010	66.0	54.4	81.0
Angola	Lunda Sul	Cacolo	4310	2015	46.3	37.1	56.8
Angola	Lunda Sul	Dala	4311	2000	102.7	84.6	122.8
Angola	Lunda Sul	Dala	4311	2005	85.9	70.8	102.4

Admin 0	Admin 1	Admin 2	GAUL Code	Year	Under-5 mortality (per 1,000 livebirths)		
					Estimate	Lower bound, 95% UI	Upper bound, 95% UI
Angola	Lunda Sul	Dala	4311	2010	67.2	55.0	80.8
Angola	Lunda Sul	Dala	4311	2015	49.4	40.3	60.1
Angola	Lunda Sul	Muconda	4312	2000	115.6	98.2	136.2
Angola	Lunda Sul	Muconda	4312	2005	101.9	86.0	118.7
Angola	Lunda Sul	Muconda	4312	2010	78.3	65.8	93.4
Angola	Lunda Sul	Muconda	4312	2015	58.3	48.5	69.5
Angola	Lunda Sul	Saurimo	4313	2000	104.7	84.8	128.5
Angola	Lunda Sul	Saurimo	4313	2005	89.8	72.9	109.9
Angola	Lunda Sul	Saurimo	4313	2010	67.0	54.5	82.0
Angola	Lunda Sul	Saurimo	4313	2015	48.6	39.0	60.1
Angola	Malanje	Cacuso	4314	2000	163.0	138.5	187.4
Angola	Malanje	Cacuso	4314	2005	133.8	113.5	155.0
Angola	Malanje	Cacuso	4314	2010	98.8	83.5	114.7
Angola	Malanje	Cacuso	4314	2015	69.2	58.5	82.2
Angola	Malanje	Calandula	4315	2000	137.3	119.8	158.1
Angola	Malanje	Calandula	4315	2005	109.8	95.3	127.4
Angola	Malanje	Calandula	4315	2010	83.3	72.3	98.5
Angola	Malanje	Calandula	4315	2015	59.2	50.1	70.5
Angola	Malanje	Cambundi-catembo	4316	2000	113.7	92.9	139.1
Angola	Malanje	Cambundi-catembo	4316	2005	97.3	79.3	119.7
Angola	Malanje	Cambundi-catembo	4316	2010	69.3	55.9	85.0
Angola	Malanje	Cambundi-catembo	4316	2015	47.5	37.8	59.3
Angola	Malanje	Cangandala	4317	2000	127.3	107.3	149.8
Angola	Malanje	Cangandala	4317	2005	108.9	92.4	128.0
Angola	Malanje	Cangandala	4317	2010	79.5	66.7	93.5
Angola	Malanje	Cangandala	4317	2015	54.0	44.2	64.8
Angola	Malanje	Caombo	4318	2000	109.8	92.7	130.2
Angola	Malanje	Caombo	4318	2005	92.7	77.9	110.6
Angola	Malanje	Caombo	4318	2010	68.8	57.5	81.3
Angola	Malanje	Caombo	4318	2015	50.2	41.6	60.1
Angola	Malanje	Kiwaba N'zogi	4319	2000	118.1	100.6	137.6
Angola	Malanje	Kiwaba N'zogi	4319	2005	100.8	86.0	117.9
Angola	Malanje	Kiwaba N'zogi	4319	2010	74.8	63.0	88.0
Angola	Malanje	Kiwaba N'zogi	4319	2015	52.8	43.9	63.6
Angola	Malanje	Kunda Dia-baze	4320	2000	105.5	86.0	128.8
Angola	Malanje	Kunda Dia-baze	4320	2005	89.5	73.7	109.6
Angola	Malanje	Kunda Dia-baze	4320	2010	64.3	52.4	78.5
Angola	Malanje	Kunda Dia-baze	4320	2015	47.3	38.4	57.6
Angola	Malanje	Luquembo	4321	2000	113.0	91.5	137.3
Angola	Malanje	Luquembo	4321	2005	97.4	78.8	119.7
Angola	Malanje	Luquembo	4321	2010	69.4	56.1	84.6
Angola	Malanje	Luquembo	4321	2015	47.8	38.2	58.8
Angola	Malanje	Malanje	4322	2000	121.3	102.3	141.9
Angola	Malanje	Malanje	4322	2005	102.2	87.1	119.1
Angola	Malanje	Malanje	4322	2010	76.0	63.8	88.9
Angola	Malanje	Malanje	4322	2015	51.3	42.1	60.7
Angola	Malanje	Marimba	4323	2000	105.5	86.2	128.4
Angola	Malanje	Marimba	4323	2005	88.8	72.8	108.3
Angola	Malanje	Marimba	4323	2010	65.0	52.8	78.9
Angola	Malanje	Marimba	4323	2015	47.7	38.8	57.9
Angola	Malanje	Massango	4324	2000	119.6	98.7	144.2
Angola	Malanje	Massango	4324	2005	95.5	79.2	115.8
Angola	Malanje	Massango	4324	2010	71.9	59.3	86.9
Angola	Malanje	Massango	4324	2015	52.4	42.4	63.8
Angola	Malanje	Mucari	4325	2000	114.2	95.7	135.2
Angola	Malanje	Mucari	4325	2005	98.9	83.4	117.1
Angola	Malanje	Mucari	4325	2010	69.6	58.0	82.1
Angola	Malanje	Mucari	4325	2015	47.4	38.8	57.3
Angola	Malanje	Quela	4326	2000	108.7	90.5	129.4
Angola	Malanje	Quela	4326	2005	94.2	78.9	113.8
Angola	Malanje	Quela	4326	2010	66.0	55.3	78.6
Angola	Malanje	Quela	4326	2015	46.5	38.6	56.2
Angola	Malanje	Quirima	4327	2000	111.5	89.0	137.5
Angola	Malanje	Quirima	4327	2005	96.9	77.0	119.9
Angola	Malanje	Quirima	4327	2010	70.8	56.9	87.0
Angola	Malanje	Quirima	4327	2015	48.1	37.9	59.7
Angola	Moxico	Alto Zambeze	4328	2000	118.6	104.0	134.7
Angola	Moxico	Alto Zambeze	4328	2005	96.4	84.4	110.0
Angola	Moxico	Alto Zambeze	4328	2010	76.9	67.2	89.1
Angola	Moxico	Alto Zambeze	4328	2015	59.0	50.5	68.8
Angola	Moxico	Camanongue	4329	2000	113.3	91.3	137.5
Angola	Moxico	Camanongue	4329	2005	87.4	70.7	106.0
Angola	Moxico	Camanongue	4329	2010	69.2	55.9	84.3
Angola	Moxico	Camanongue	4329	2015	52.4	41.8	65.4
Angola	Moxico	Kameia Lumege	4330	2000	115.6	92.6	143.3
Angola	Moxico	Kameia Lumege	4330	2005	94.0	75.1	116.5
Angola	Moxico	Kameia Lumege	4330	2010	73.6	58.7	92.5
Angola	Moxico	Kameia Lumege	4330	2015	54.3	42.1	68.3
Angola	Moxico	Leua	4331	2000	118.2	93.9	146.2
Angola	Moxico	Leua	4331	2005	90.8	73.0	113.6
Angola	Moxico	Leua	4331	2010	71.7	57.3	89.7
Angola	Moxico	Leua	4331	2015	53.8	42.2	68.7
Angola	Moxico	Luacano	4332	2000	114.7	94.6	138.8
Angola	Moxico	Luacano	4332	2005	97.5	80.2	117.7
Angola	Moxico	Luacano	4332	2010	74.5	61.1	90.7

Admin 0	Admin 1	Admin 2	GAUL Code	Year	Under-5 mortality (per 1,000 livebirths)		
					Estimate	Lower bound, 95% UI	Upper bound, 95% UI
Angola	Moxico	Luacano	4332	2015	56.0	45.3	68.9
Angola	Moxico	Luau	4333	2000	124.5	103.2	147.9
Angola	Moxico	Luau	4333	2005	106.4	88.5	126.7
Angola	Moxico	Luau	4333	2010	79.6	65.4	96.1
Angola	Moxico	Luau	4333	2015	61.4	50.1	75.1
Angola	Moxico	Luchazes	4334	2000	138.3	112.2	167.7
Angola	Moxico	Luchazes	4334	2005	109.8	88.7	133.3
Angola	Moxico	Luchazes	4334	2010	87.3	70.4	107.5
Angola	Moxico	Luchazes	4334	2015	61.3	49.7	75.1
Angola	Moxico	Luena (moxico)	4335	2000	123.2	101.2	149.2
Angola	Moxico	Luena (moxico)	4335	2005	97.6	79.6	118.5
Angola	Moxico	Luena (moxico)	4335	2010	76.5	63.1	93.0
Angola	Moxico	Luena (moxico)	4335	2015	53.5	43.3	65.0
Angola	Moxico	Lumbala N'guimbo	4336	2000	139.7	119.0	164.3
Angola	Moxico	Lumbala N'guimbo	4336	2005	109.9	93.3	128.6
Angola	Moxico	Lumbala N'guimbo	4336	2010	94.6	79.6	111.6
Angola	Moxico	Lumbala N'guimbo	4336	2015	69.7	58.1	82.7
Angola	Namibe	Bibala	4337	2000	148.2	128.2	170.2
Angola	Namibe	Bibala	4337	2005	120.0	104.8	137.3
Angola	Namibe	Bibala	4337	2010	96.0	82.6	111.5
Angola	Namibe	Bibala	4337	2015	62.3	52.9	73.4
Angola	Namibe	Camacuio	4338	2000	156.9	131.5	186.1
Angola	Namibe	Camacuio	4338	2005	125.9	106.0	149.3
Angola	Namibe	Camacuio	4338	2010	102.2	85.7	121.7
Angola	Namibe	Camacuio	4338	2015	66.3	54.7	80.4
Angola	Namibe	Namibe	4339	2000	114.5	95.0	138.8
Angola	Namibe	Namibe	4339	2005	101.2	84.5	120.5
Angola	Namibe	Namibe	4339	2010	85.8	72.3	102.3
Angola	Namibe	Namibe	4339	2015	54.4	45.1	64.8
Angola	Namibe	Tombua	4340	2000	114.3	92.8	140.3
Angola	Namibe	Tombua	4340	2005	99.4	81.6	121.2
Angola	Namibe	Tombua	4340	2010	81.8	67.1	100.9
Angola	Namibe	Tombua	4340	2015	51.5	42.4	63.1
Angola	Namibe	Virei	4341	2000	131.3	111.6	154.0
Angola	Namibe	Virei	4341	2005	111.1	93.7	129.6
Angola	Namibe	Virei	4341	2010	89.9	75.6	105.8
Angola	Namibe	Virei	4341	2015	57.5	47.6	69.6
Angola	Uige	Alto Cauale	4342	2000	148.2	123.9	175.0
Angola	Uige	Alto Cauale	4342	2005	110.4	92.1	129.6
Angola	Uige	Alto Cauale	4342	2010	81.7	67.6	96.9
Angola	Uige	Alto Cauale	4342	2015	58.4	47.7	70.7
Angola	Uige	Ambuila	4343	2000	171.1	146.3	201.2
Angola	Uige	Ambuila	4343	2005	129.0	108.6	152.7
Angola	Uige	Ambuila	4343	2010	95.7	80.9	113.4
Angola	Uige	Ambuila	4343	2015	64.3	54.0	76.4
Angola	Uige	Bembe	4344	2000	171.4	143.4	206.1
Angola	Uige	Bembe	4344	2005	127.4	105.8	152.5
Angola	Uige	Bembe	4344	2010	96.5	80.2	115.7
Angola	Uige	Bembe	4344	2015	64.6	53.1	77.4
Angola	Uige	Buengas	4345	2000	185.4	158.2	214.4
Angola	Uige	Buengas	4345	2005	132.6	113.0	153.4
Angola	Uige	Buengas	4345	2010	97.1	80.7	113.6
Angola	Uige	Buengas	4345	2015	65.9	54.6	77.3
Angola	Uige	Bungo	4346	2000	168.9	145.4	198.1
Angola	Uige	Bungo	4346	2005	121.4	104.4	140.5
Angola	Uige	Bungo	4346	2010	83.9	70.9	98.0
Angola	Uige	Bungo	4346	2015	61.3	51.5	72.6
Angola	Uige	Damba	4347	2000	178.7	153.7	206.2
Angola	Uige	Damba	4347	2005	128.6	110.6	149.3
Angola	Uige	Damba	4347	2010	91.9	78.7	107.9
Angola	Uige	Damba	4347	2015	62.1	51.8	73.5
Angola	Uige	Maquela Do Zombo	4348	2000	177.2	156.1	198.9
Angola	Uige	Maquela Do Zombo	4348	2005	132.5	117.4	148.4
Angola	Uige	Maquela Do Zombo	4348	2010	95.4	83.0	107.8
Angola	Uige	Maquela Do Zombo	4348	2015	60.8	52.0	70.3
Angola	Uige	Mucaba	4349	2000	176.8	149.8	210.7
Angola	Uige	Mucaba	4349	2005	126.6	106.6	149.7
Angola	Uige	Mucaba	4349	2010	87.0	72.4	103.7
Angola	Uige	Mucaba	4349	2015	61.7	50.8	73.3
Angola	Uige	Negage	4350	2000	164.0	143.0	188.3
Angola	Uige	Negage	4350	2005	120.7	104.8	138.0
Angola	Uige	Negage	4350	2010	83.7	70.5	97.0
Angola	Uige	Negage	4350	2015	59.9	49.9	70.8
Angola	Uige	Puri	4351	2000	159.5	133.8	187.4
Angola	Uige	Puri	4351	2005	117.3	97.6	136.3
Angola	Uige	Puri	4351	2010	81.3	67.2	95.7
Angola	Uige	Puri	4351	2015	59.7	49.0	71.4
Angola	Uige	Quimbele	4352	2000	166.0	144.1	188.9
Angola	Uige	Quimbele	4352	2005	125.4	109.4	143.0
Angola	Uige	Quimbele	4352	2010	91.3	77.9	104.8
Angola	Uige	Quimbele	4352	2015	62.1	52.1	73.1
Angola	Uige	Quitexe	4353	2000	179.0	155.6	208.8
Angola	Uige	Quitexe	4353	2005	136.0	116.8	156.1
Angola	Uige	Quitexe	4353	2010	98.9	84.8	115.2
Angola	Uige	Quitexe	4353	2015	66.3	56.1	79.1

Admin 0	Admin 1	Admin 2	GAUL Code	Year	Under-5 mortality (per 1,000 livebirths)		
					Estimate	Lower bound, 95% UI	Upper bound, 95% UI
Angola	Uige	Santa Cruz	4354	2000	145.2	124.2	169.6
Angola	Uige	Santa Cruz	4354	2005	110.8	94.2	128.7
Angola	Uige	Santa Cruz	4354	2010	81.9	68.8	96.3
Angola	Uige	Santa Cruz	4354	2015	58.8	48.4	69.4
Angola	Uige	Sanza Pombo	4355	2000	161.9	137.5	191.5
Angola	Uige	Sanza Pombo	4355	2005	117.5	99.4	138.1
Angola	Uige	Sanza Pombo	4355	2010	85.6	72.3	102.0
Angola	Uige	Sanza Pombo	4355	2015	63.1	52.1	75.7
Angola	Uige	Songo	4356	2000	175.0	148.3	206.5
Angola	Uige	Songo	4356	2005	128.6	107.6	153.1
Angola	Uige	Songo	4356	2010	92.4	77.7	109.0
Angola	Uige	Songo	4356	2015	64.0	53.1	76.3
Angola	Uige	Songo/mucaba	4357	2000	175.4	149.1	207.1
Angola	Uige	Songo/mucaba	4357	2005	128.8	108.9	152.3
Angola	Uige	Songo/mucaba	4357	2010	91.5	76.5	107.9
Angola	Uige	Songo/mucaba	4357	2015	64.2	52.8	76.3
Angola	Uige	Uige	4358	2000	169.4	143.9	198.6
Angola	Uige	Uige	4358	2005	119.6	101.4	139.1
Angola	Uige	Uige	4358	2010	82.7	69.3	97.5
Angola	Uige	Uige	4358	2015	56.9	47.6	68.5
Angola	Zaire	Cuimba	4359	2000	157.4	134.5	180.9
Angola	Zaire	Cuimba	4359	2005	121.0	103.2	139.6
Angola	Zaire	Cuimba	4359	2010	89.6	76.2	104.7
Angola	Zaire	Cuimba	4359	2015	55.4	46.5	65.8
Angola	Zaire	M'banza Congo	4360	2000	146.7	124.1	172.4
Angola	Zaire	M'banza Congo	4360	2005	115.7	97.0	135.9
Angola	Zaire	M'banza Congo	4360	2010	88.3	74.4	103.4
Angola	Zaire	M'banza Congo	4360	2015	54.9	45.2	64.7
Angola	Zaire	N'zeto	4361	2000	142.6	117.4	172.8
Angola	Zaire	N'zeto	4361	2005	113.0	92.9	138.0
Angola	Zaire	N'zeto	4361	2010	91.5	75.5	111.6
Angola	Zaire	N'zeto	4361	2015	57.6	46.9	71.3
Angola	Zaire	Noqui	4362	2000	127.1	109.8	148.3
Angola	Zaire	Noqui	4362	2005	105.5	91.0	122.0
Angola	Zaire	Noqui	4362	2010	85.4	72.3	98.8
Angola	Zaire	Noqui	4362	2015	50.5	42.2	59.8
Angola	Zaire	Soyo	4363	2000	123.4	101.7	148.6
Angola	Zaire	Soyo	4363	2005	98.2	80.5	118.4
Angola	Zaire	Soyo	4363	2010	82.7	67.9	98.9
Angola	Zaire	Soyo	4363	2015	51.1	42.1	61.5
Angola	Zaire	Tomboco	4364	2000	140.7	116.9	168.5
Angola	Zaire	Tomboco	4364	2005	111.2	92.5	133.7
Angola	Zaire	Tomboco	4364	2010	90.3	74.7	109.0
Angola	Zaire	Tomboco	4364	2015	56.6	46.6	68.0
Benin	Alibori	Banikoara	5861	2000	172.8	158.7	186.9
Benin	Alibori	Banikoara	5861	2005	141.8	129.8	154.4
Benin	Alibori	Banikoara	5861	2010	115.2	103.5	127.7
Benin	Alibori	Banikoara	5861	2015	90.2	79.4	102.2
Benin	Alibori	Gogounou	5862	2000	137.0	125.9	148.4
Benin	Alibori	Gogounou	5862	2005	110.7	101.7	120.7
Benin	Alibori	Gogounou	5862	2010	90.7	81.7	99.8
Benin	Alibori	Gogounou	5862	2015	73.1	63.9	82.3
Benin	Alibori	Kandi	5863	2000	147.9	136.2	162.7
Benin	Alibori	Kandi	5863	2005	126.9	115.9	139.4
Benin	Alibori	Kandi	5863	2010	105.3	95.2	117.3
Benin	Alibori	Kandi	5863	2015	81.8	71.5	93.2
Benin	Alibori	Karimama	5864	2000	219.3	200.7	237.4
Benin	Alibori	Karimama	5864	2005	183.9	167.9	199.0
Benin	Alibori	Karimama	5864	2010	155.1	140.2	172.0
Benin	Alibori	Karimama	5864	2015	109.1	96.2	123.2
Benin	Alibori	Malanville	5865	2000	196.5	181.9	212.5
Benin	Alibori	Malanville	5865	2005	170.4	157.3	184.6
Benin	Alibori	Malanville	5865	2010	147.4	135.3	161.7
Benin	Alibori	Malanville	5865	2015	110.9	98.7	125.6
Benin	Alibori	Segbana	5866	2000	150.2	135.8	165.8
Benin	Alibori	Segbana	5866	2005	129.4	117.0	142.9
Benin	Alibori	Segbana	5866	2010	109.7	98.9	123.0
Benin	Alibori	Segbana	5866	2015	84.0	73.3	96.0
Benin	Atakora	Boukoumbe	5867	2000	153.9	142.6	165.9
Benin	Atakora	Boukoumbe	5867	2005	127.5	118.3	137.6
Benin	Atakora	Boukoumbe	5867	2010	108.7	98.7	117.9
Benin	Atakora	Boukoumbe	5867	2015	89.9	80.0	101.5
Benin	Atakora	Kerou	5868	2000	158.7	144.3	174.0
Benin	Atakora	Kerou	5868	2005	126.0	114.9	137.6
Benin	Atakora	Kerou	5868	2010	105.8	94.4	117.4
Benin	Atakora	Kerou	5868	2015	85.0	74.5	96.8
Benin	Atakora	Kobli	5869	2000	162.1	149.4	175.5
Benin	Atakora	Kobli	5869	2005	132.8	122.6	143.7
Benin	Atakora	Kobli	5869	2010	115.2	104.5	126.5
Benin	Atakora	Kobli	5869	2015	91.9	81.7	103.5
Benin	Atakora	Kouande	5870	2000	147.7	135.6	160.0
Benin	Atakora	Kouande	5870	2005	120.3	110.6	130.5
Benin	Atakora	Kouande	5870	2010	101.1	91.2	111.7
Benin	Atakora	Kouande	5870	2015	82.3	72.8	93.0
Benin	Atakora	Materi	5871	2000	169.0	156.3	183.1

Admin 0	Admin 1	Admin 2	GAUL Code	Year	Under-5 mortality (per 1,000 livebirths)		
					Estimate	Lower bound, 95% UI	Upper bound, 95% UI
Benin	Atakora	Materi	5871	2005	138.5	127.9	149.6
Benin	Atakora	Materi	5871	2010	120.2	109.0	131.8
Benin	Atakora	Materi	5871	2015	90.8	80.4	101.8
Benin	Atakora	Natitingou	5872	2000	133.9	123.5	145.3
Benin	Atakora	Natitingou	5872	2005	112.8	103.8	121.8
Benin	Atakora	Natitingou	5872	2010	94.9	86.0	104.0
Benin	Atakora	Natitingou	5872	2015	75.1	66.6	84.6
Benin	Atakora	Pehonko	5873	2000	140.4	127.6	153.2
Benin	Atakora	Pehonko	5873	2005	112.7	102.0	123.1
Benin	Atakora	Pehonko	5873	2010	94.5	84.5	104.7
Benin	Atakora	Pehonko	5873	2015	77.1	67.4	87.9
Benin	Atakora	Tanguieta	5874	2000	164.5	152.6	177.7
Benin	Atakora	Tanguieta	5874	2005	134.7	124.7	145.7
Benin	Atakora	Tanguieta	5874	2010	114.9	104.5	125.8
Benin	Atakora	Tanguieta	5874	2015	88.8	78.7	99.8
Benin	Atakora	Toukountouna	5875	2000	150.9	138.6	163.3
Benin	Atakora	Toukountouna	5875	2005	123.4	113.7	134.0
Benin	Atakora	Toukountouna	5875	2010	104.0	93.9	115.3
Benin	Atakora	Toukountouna	5875	2015	85.0	75.3	96.3
Benin	Atlantique	Abomey-calavi	5876	2000	111.0	104.4	117.5
Benin	Atlantique	Abomey-calavi	5876	2005	94.8	89.0	100.5
Benin	Atlantique	Abomey-calavi	5876	2010	79.8	74.4	86.0
Benin	Atlantique	Abomey-calavi	5876	2015	62.5	55.9	69.7
Benin	Atlantique	Allada	5877	2000	133.8	126.1	141.2
Benin	Atlantique	Allada	5877	2005	109.8	103.4	116.5
Benin	Atlantique	Allada	5877	2010	90.4	84.1	96.9
Benin	Atlantique	Allada	5877	2015	81.7	72.8	90.8
Benin	Atlantique	Kpomasse	5878	2000	116.4	109.1	123.2
Benin	Atlantique	Kpomasse	5878	2005	95.4	89.3	101.8
Benin	Atlantique	Kpomasse	5878	2010	78.8	73.2	85.1
Benin	Atlantique	Kpomasse	5878	2015	68.2	60.8	76.1
Benin	Atlantique	Ouidah	5879	2000	111.6	104.8	118.6
Benin	Atlantique	Ouidah	5879	2005	91.9	85.8	98.0
Benin	Atlantique	Ouidah	5879	2010	76.3	70.9	82.2
Benin	Atlantique	Ouidah	5879	2015	61.3	54.8	68.3
Benin	Atlantique	So-ava	5880	2000	131.1	123.7	138.3
Benin	Atlantique	So-ava	5880	2005	111.4	105.5	117.4
Benin	Atlantique	So-ava	5880	2010	90.5	84.8	97.1
Benin	Atlantique	So-ava	5880	2015	72.1	64.6	80.3
Benin	Atlantique	Toffo	5881	2000	141.5	134.0	149.2
Benin	Atlantique	Toffo	5881	2005	116.2	109.5	122.9
Benin	Atlantique	Toffo	5881	2010	96.2	89.5	102.6
Benin	Atlantique	Toffo	5881	2015	85.3	76.3	94.5
Benin	Atlantique	Tori-bossito	5882	2000	133.6	126.2	140.7
Benin	Atlantique	Tori-bossito	5882	2005	110.0	103.3	116.2
Benin	Atlantique	Tori-bossito	5882	2010	88.2	82.4	94.3
Benin	Atlantique	Tori-bossito	5882	2015	79.6	71.1	88.7
Benin	Atlantique	Ze	5883	2000	141.1	133.5	148.5
Benin	Atlantique	Ze	5883	2005	117.7	110.9	124.3
Benin	Atlantique	Ze	5883	2010	96.3	89.9	102.7
Benin	Atlantique	Ze	5883	2015	83.9	75.0	93.6
Benin	Borgou	Bembereke	5884	2000	119.7	109.1	131.1
Benin	Borgou	Bembereke	5884	2005	95.4	86.4	104.6
Benin	Borgou	Bembereke	5884	2010	81.3	72.7	90.8
Benin	Borgou	Bembereke	5884	2015	65.6	57.0	74.7
Benin	Borgou	Kalale	5885	2000	124.2	112.5	137.1
Benin	Borgou	Kalale	5885	2005	100.4	91.0	110.7
Benin	Borgou	Kalale	5885	2010	88.2	78.8	99.3
Benin	Borgou	Kalale	5885	2015	71.9	62.1	82.1
Benin	Borgou	Ndali	5886	2000	127.3	115.9	138.2
Benin	Borgou	Ndali	5886	2005	100.9	92.1	110.0
Benin	Borgou	Ndali	5886	2010	85.3	76.1	94.5
Benin	Borgou	Ndali	5886	2015	69.7	61.1	79.6
Benin	Borgou	Nikki	5887	2000	120.3	108.9	131.8
Benin	Borgou	Nikki	5887	2005	94.1	85.7	103.7
Benin	Borgou	Nikki	5887	2010	82.1	73.0	92.0
Benin	Borgou	Nikki	5887	2015	67.6	58.8	77.4
Benin	Borgou	Parakou	5888	2000	120.9	109.7	132.8
Benin	Borgou	Parakou	5888	2005	94.7	85.6	105.2
Benin	Borgou	Parakou	5888	2010	75.7	67.5	85.1
Benin	Borgou	Parakou	5888	2015	59.5	51.6	68.9
Benin	Borgou	Perere	5889	2000	125.6	114.0	138.0
Benin	Borgou	Perere	5889	2005	98.8	89.4	108.6
Benin	Borgou	Perere	5889	2010	84.4	75.5	94.1
Benin	Borgou	Perere	5889	2015	69.6	60.7	79.6
Benin	Borgou	Sinende	5890	2000	130.2	119.3	142.9
Benin	Borgou	Sinende	5890	2005	103.4	93.9	113.6
Benin	Borgou	Sinende	5890	2010	87.4	78.2	98.0
Benin	Borgou	Sinende	5890	2015	69.7	60.7	79.6
Benin	Borgou	Tchaourou	5891	2000	143.1	131.7	156.0
Benin	Borgou	Tchaourou	5891	2005	116.0	105.9	126.6
Benin	Borgou	Tchaourou	5891	2010	94.1	84.6	104.3
Benin	Borgou	Tchaourou	5891	2015	76.1	67.1	86.6
Benin	Collines	Bante	5892	2000	150.1	137.4	165.3
Benin	Collines	Bante	5892	2005	129.4	118.3	141.6

Admin 0	Admin 1	Admin 2	GAUL Code	Year	Under-5 mortality (per 1,000 livebirths)		
					Estimate	Lower bound, 95% UI	Upper bound, 95% UI
Benin	Collines	Bante	5892	2010	107.0	96.6	118.0
Benin	Collines	Bante	5892	2015	89.2	78.9	101.9
Benin	Collines	Dassa	5893	2000	149.1	138.3	160.8
Benin	Collines	Dassa	5893	2005	124.5	115.1	134.8
Benin	Collines	Dassa	5893	2010	100.5	91.6	109.6
Benin	Collines	Dassa	5893	2015	84.0	74.6	94.9
Benin	Collines	Glazoue	5894	2000	148.7	137.7	160.3
Benin	Collines	Glazoue	5894	2005	125.5	115.9	136.2
Benin	Collines	Glazoue	5894	2010	102.4	93.2	111.9
Benin	Collines	Glazoue	5894	2015	83.9	74.3	95.6
Benin	Collines	Ouesse	5895	2000	143.7	130.5	158.8
Benin	Collines	Ouesse	5895	2005	120.8	109.5	133.9
Benin	Collines	Ouesse	5895	2010	97.5	87.1	108.9
Benin	Collines	Ouesse	5895	2015	78.9	69.3	91.1
Benin	Collines	Savalou	5896	2000	150.8	139.1	161.7
Benin	Collines	Savalou	5896	2005	127.2	117.5	136.9
Benin	Collines	Savalou	5896	2010	104.3	95.1	113.4
Benin	Collines	Savalou	5896	2015	88.0	78.3	99.6
Benin	Collines	Save	5897	2000	135.1	122.2	148.5
Benin	Collines	Save	5897	2005	109.0	97.7	120.8
Benin	Collines	Save	5897	2010	86.8	76.8	97.0
Benin	Collines	Save	5897	2015	66.8	57.7	76.8
Benin	Couffo	Aplahoue	5898	2000	148.8	140.4	158.2
Benin	Couffo	Aplahoue	5898	2005	119.9	112.6	127.2
Benin	Couffo	Aplahoue	5898	2010	96.7	89.2	103.9
Benin	Couffo	Aplahoue	5898	2015	88.4	79.0	98.4
Benin	Couffo	Djakotome	5899	2000	132.4	125.0	140.7
Benin	Couffo	Djakotome	5899	2005	108.5	101.7	114.9
Benin	Couffo	Djakotome	5899	2010	86.4	80.1	92.5
Benin	Couffo	Djakotome	5899	2015	79.3	70.8	87.9
Benin	Couffo	Dogbo-tota	5900	2000	133.4	125.9	141.6
Benin	Couffo	Dogbo-tota	5900	2005	109.0	102.3	115.4
Benin	Couffo	Dogbo-tota	5900	2010	85.5	79.5	91.9
Benin	Couffo	Dogbo-tota	5900	2015	79.2	70.9	88.3
Benin	Couffo	Klouekanme	5901	2000	152.7	144.9	161.1
Benin	Couffo	Klouekanme	5901	2005	123.1	116.4	130.0
Benin	Couffo	Klouekanme	5901	2010	99.8	93.1	107.0
Benin	Couffo	Klouekanme	5901	2015	90.0	80.7	100.1
Benin	Couffo	Lalo	5902	2000	151.5	143.8	159.9
Benin	Couffo	Lalo	5902	2005	121.7	115.0	128.6
Benin	Couffo	Lalo	5902	2010	100.3	93.4	107.3
Benin	Couffo	Lalo	5902	2015	89.7	80.2	99.5
Benin	Couffo	Toviklin	5903	2000	143.5	135.9	151.9
Benin	Couffo	Toviklin	5903	2005	115.5	108.8	122.3
Benin	Couffo	Toviklin	5903	2010	94.3	87.6	101.4
Benin	Couffo	Toviklin	5903	2015	85.5	76.3	94.9
Benin	Donga	Bassila	5904	2000	156.7	144.9	169.9
Benin	Donga	Bassila	5904	2005	134.4	123.7	146.1
Benin	Donga	Bassila	5904	2010	111.6	102.4	122.9
Benin	Donga	Bassila	5904	2015	89.8	79.9	101.4
Benin	Donga	Djougou	5905	2000	145.3	133.6	157.3
Benin	Donga	Djougou	5905	2005	122.6	112.8	132.3
Benin	Donga	Djougou	5905	2010	103.6	94.6	113.9
Benin	Donga	Djougou	5905	2015	82.2	72.6	92.9
Benin	Donga	Kopargo	5906	2000	149.5	138.7	161.0
Benin	Donga	Kopargo	5906	2005	126.9	118.1	136.8
Benin	Donga	Kopargo	5906	2010	107.3	98.6	117.2
Benin	Donga	Kopargo	5906	2015	86.2	76.3	96.8
Benin	Donga	Ouake	5907	2000	154.8	144.1	166.1
Benin	Donga	Ouake	5907	2005	134.4	124.5	144.9
Benin	Donga	Ouake	5907	2010	113.5	104.4	124.0
Benin	Donga	Ouake	5907	2015	87.1	76.9	97.4
Benin	Littoral	Cotonou	5908	2000	84.4	78.9	90.5
Benin	Littoral	Cotonou	5908	2005	76.6	71.5	81.9
Benin	Littoral	Cotonou	5908	2010	67.9	62.9	73.8
Benin	Littoral	Cotonou	5908	2015	50.9	45.1	57.5
Benin	Mono	Athieme	5909	2000	114.6	107.4	122.0
Benin	Mono	Athieme	5909	2005	93.7	87.5	100.2
Benin	Mono	Athieme	5909	2010	74.6	69.2	80.6
Benin	Mono	Athieme	5909	2015	67.8	60.1	75.5
Benin	Mono	Bopa	5910	2000	128.8	121.9	136.0
Benin	Mono	Bopa	5910	2005	106.2	100.4	112.3
Benin	Mono	Bopa	5910	2010	87.3	81.6	93.8
Benin	Mono	Bopa	5910	2015	78.0	69.6	86.5
Benin	Mono	Come	5911	2000	106.1	97.7	114.4
Benin	Mono	Come	5911	2005	86.5	79.6	93.8
Benin	Mono	Come	5911	2010	72.1	66.1	78.8
Benin	Mono	Come	5911	2015	59.2	52.1	66.7
Benin	Mono	Grand-popo	5912	2000	111.2	102.8	119.6
Benin	Mono	Grand-popo	5912	2005	89.9	82.7	97.4
Benin	Mono	Grand-popo	5912	2010	74.8	68.5	81.8
Benin	Mono	Grand-popo	5912	2015	64.4	56.6	72.3
Benin	Mono	Houeyogbe	5913	2000	116.2	109.0	123.8
Benin	Mono	Houeyogbe	5913	2005	96.2	89.8	102.7
Benin	Mono	Houeyogbe	5913	2010	77.0	71.3	83.4

Admin 0	Admin 1	Admin 2	GAUL Code	Year	Under-5 mortality (per 1,000 livebirths)		
					Estimate	Lower bound, 95% UI	Upper bound, 95% UI
Benin	Mono	Houeyogbe	5913	2015	69.1	61.6	77.2
Benin	Mono	Lokossa	5914	2000	116.7	110.2	123.5
Benin	Mono	Lokossa	5914	2005	95.8	89.7	101.8
Benin	Mono	Lokossa	5914	2010	76.3	71.2	82.0
Benin	Mono	Lokossa	5914	2015	69.5	61.9	77.4
Benin	Oueme	Adjara	5915	2000	124.5	116.8	132.3
Benin	Oueme	Adjara	5915	2005	109.0	102.6	115.7
Benin	Oueme	Adjara	5915	2010	87.5	81.0	94.3
Benin	Oueme	Adjara	5915	2015	66.6	59.6	74.5
Benin	Oueme	Adjohoun	5916	2000	146.4	139.0	153.8
Benin	Oueme	Adjohoun	5916	2005	122.9	116.6	129.0
Benin	Oueme	Adjohoun	5916	2010	98.6	92.2	105.4
Benin	Oueme	Adjohoun	5916	2015	82.8	74.4	92.3
Benin	Oueme	Aguegue	5917	2000	140.9	132.1	149.1
Benin	Oueme	Aguegue	5917	2005	118.7	112.0	125.8
Benin	Oueme	Aguegue	5917	2010	94.0	87.6	101.5
Benin	Oueme	Aguegue	5917	2015	74.2	66.3	82.8
Benin	Oueme	Akpro-misserete	5918	2000	128.0	120.6	135.3
Benin	Oueme	Akpro-misserete	5918	2005	109.5	103.2	115.9
Benin	Oueme	Akpro-misserete	5918	2010	88.6	82.5	95.4
Benin	Oueme	Akpro-misserete	5918	2015	68.9	61.7	76.9
Benin	Oueme	Avrankou	5919	2000	140.2	131.9	148.5
Benin	Oueme	Avrankou	5919	2005	117.6	110.5	124.7
Benin	Oueme	Avrankou	5919	2010	93.0	86.6	99.9
Benin	Oueme	Avrankou	5919	2015	74.8	67.0	83.4
Benin	Oueme	Bonou	5920	2000	146.5	137.8	155.8
Benin	Oueme	Bonou	5920	2005	121.2	113.1	129.0
Benin	Oueme	Bonou	5920	2010	98.5	91.1	106.3
Benin	Oueme	Bonou	5920	2015	81.5	72.6	91.4
Benin	Oueme	Dangbo	5921	2000	143.4	136.1	150.6
Benin	Oueme	Dangbo	5921	2005	121.4	115.2	127.4
Benin	Oueme	Dangbo	5921	2010	95.4	89.4	102.4
Benin	Oueme	Dangbo	5921	2015	80.0	71.9	89.1
Benin	Oueme	Porto-novo	5922	2000	118.8	111.2	126.3
Benin	Oueme	Porto-novo	5922	2005	104.2	97.7	110.8
Benin	Oueme	Porto-novo	5922	2010	84.0	78.0	90.8
Benin	Oueme	Porto-novo	5922	2015	62.7	56.0	70.1
Benin	Oueme	Seme-kpodji	5923	2000	128.2	119.4	137.0
Benin	Oueme	Seme-kpodji	5923	2005	111.9	104.9	119.2
Benin	Oueme	Seme-kpodji	5923	2010	90.6	83.9	98.3
Benin	Oueme	Seme-kpodji	5923	2015	68.8	61.3	77.1
Benin	Plateau	Adja-ouere	5924	2000	150.4	141.2	160.3
Benin	Plateau	Adja-ouere	5924	2005	124.2	116.0	132.3
Benin	Plateau	Adja-ouere	5924	2010	98.1	90.4	106.1
Benin	Plateau	Adja-ouere	5924	2015	80.8	72.3	90.2
Benin	Plateau	Ifangni	5925	2000	139.2	130.8	147.6
Benin	Plateau	Ifangni	5925	2005	116.9	109.6	124.8
Benin	Plateau	Ifangni	5925	2010	93.3	86.8	100.3
Benin	Plateau	Ifangni	5925	2015	75.4	67.6	84.2
Benin	Plateau	Ketou	5926	2000	149.7	138.5	161.1
Benin	Plateau	Ketou	5926	2005	120.1	110.5	129.7
Benin	Plateau	Ketou	5926	2010	93.3	85.0	101.6
Benin	Plateau	Ketou	5926	2015	77.0	68.4	86.3
Benin	Plateau	Pobe	5927	2000	149.1	138.9	159.4
Benin	Plateau	Pobe	5927	2005	121.6	113.0	130.2
Benin	Plateau	Pobe	5927	2010	94.2	86.1	102.0
Benin	Plateau	Pobe	5927	2015	79.0	69.9	88.4
Benin	Plateau	Sakete	5928	2000	148.1	139.5	156.6
Benin	Plateau	Sakete	5928	2005	125.1	117.5	132.9
Benin	Plateau	Sakete	5928	2010	97.9	91.2	104.7
Benin	Plateau	Sakete	5928	2015	82.5	74.3	92.4
Benin	Zou	Abomey	5929	2000	144.1	135.0	153.6
Benin	Zou	Abomey	5929	2005	121.7	113.2	129.8
Benin	Zou	Abomey	5929	2010	96.7	89.1	104.4
Benin	Zou	Abomey	5929	2015	87.5	76.8	99.1
Benin	Zou	Agbangnizoun	5930	2000	153.1	144.6	162.1
Benin	Zou	Agbangnizoun	5930	2005	126.1	118.5	133.6
Benin	Zou	Agbangnizoun	5930	2010	105.0	97.6	112.9
Benin	Zou	Agbangnizoun	5930	2015	92.6	82.5	104.2
Benin	Zou	Bohicon	5931	2000	142.0	133.5	151.2
Benin	Zou	Bohicon	5931	2005	119.1	111.1	126.9
Benin	Zou	Bohicon	5931	2010	95.7	88.2	103.4
Benin	Zou	Bohicon	5931	2015	84.4	74.0	95.5
Benin	Zou	Cove	5932	2000	150.2	141.0	160.4
Benin	Zou	Cove	5932	2005	124.0	116.1	132.3
Benin	Zou	Cove	5932	2010	97.9	90.5	106.1
Benin	Zou	Cove	5932	2015	82.2	72.9	92.5
Benin	Zou	Djidja	5933	2000	165.4	154.9	175.3
Benin	Zou	Djidja	5933	2005	135.9	127.5	145.0
Benin	Zou	Djidja	5933	2010	111.8	103.3	120.6
Benin	Zou	Djidja	5933	2015	97.0	86.5	109.6
Benin	Zou	Ouinhi	5934	2000	146.5	136.8	156.8
Benin	Zou	Ouinhi	5934	2005	120.6	112.1	128.9
Benin	Zou	Ouinhi	5934	2010	96.8	89.0	105.1
Benin	Zou	Ouinhi	5934	2015	79.4	70.3	88.9

Admin 0	Admin 1	Admin 2	GAUL Code	Year	Under-5 mortality (per 1,000 livebirths)		
					Estimate	Lower bound, 95% UI	Upper bound, 95% UI
Benin	Zou	Za-kpota	5935	2000	158.1	149.3	167.5
Benin	Zou	Za-kpota	5935	2005	130.1	122.5	138.0
Benin	Zou	Za-kpota	5935	2010	104.6	97.1	113.0
Benin	Zou	Za-kpota	5935	2015	88.9	78.8	100.6
Benin	Zou	Zangnanado	5936	2000	149.5	140.8	160.1
Benin	Zou	Zangnanado	5936	2005	122.8	114.5	130.7
Benin	Zou	Zangnanado	5936	2010	98.7	90.9	106.9
Benin	Zou	Zangnanado	5936	2015	80.1	71.3	89.8
Benin	Zou	Zogbodome	5937	2000	154.6	147.1	162.9
Benin	Zou	Zogbodome	5937	2005	126.7	119.6	133.5
Benin	Zou	Zogbodome	5937	2010	103.7	96.6	110.4
Benin	Zou	Zogbodome	5937	2015	89.2	79.7	99.6
Botswana	Central	Central	6322	2000	64.3	58.4	70.3
Botswana	Central	Central	6322	2005	48.6	44.2	53.5
Botswana	Central	Central	6322	2010	27.5	24.9	30.4
Botswana	Central	Central	6322	2015	16.5	14.5	18.7
Botswana	Chobe	Chobe	6323	2000	78.5	68.3	90.8
Botswana	Chobe	Chobe	6323	2005	58.5	50.9	67.7
Botswana	Chobe	Chobe	6323	2010	32.6	27.9	37.9
Botswana	Chobe	Chobe	6323	2015	19.0	15.9	22.5
Botswana	Ghanzi	Ghanzi	6324	2000	76.8	65.3	89.9
Botswana	Ghanzi	Ghanzi	6324	2005	54.1	45.6	62.7
Botswana	Ghanzi	Ghanzi	6324	2010	28.9	24.3	34.0
Botswana	Ghanzi	Ghanzi	6324	2015	18.1	15.1	21.4
Botswana	Kgalagadi	Kgalagadi	6325	2000	71.2	60.7	82.3
Botswana	Kgalagadi	Kgalagadi	6325	2005	52.2	44.6	60.6
Botswana	Kgalagadi	Kgalagadi	6325	2010	28.5	24.3	33.6
Botswana	Kgalagadi	Kgalagadi	6325	2015	18.0	15.2	21.2
Botswana	Kgatleng	Kgatleng	6326	2000	55.0	48.4	62.7
Botswana	Kgatleng	Kgatleng	6326	2005	41.3	36.0	47.8
Botswana	Kgatleng	Kgatleng	6326	2010	24.3	20.8	28.2
Botswana	Kgatleng	Kgatleng	6326	2015	14.8	12.2	17.5
Botswana	Kweneng	Kweneng	6327	2000	62.1	54.1	70.6
Botswana	Kweneng	Kweneng	6327	2005	45.5	39.8	52.0
Botswana	Kweneng	Kweneng	6327	2010	25.8	22.2	29.9
Botswana	Kweneng	Kweneng	6327	2015	16.0	13.5	18.8
Botswana	Ngamiland	Ngamiland	6328	2000	81.9	70.9	93.2
Botswana	Ngamiland	Ngamiland	6328	2005	58.8	50.4	67.7
Botswana	Ngamiland	Ngamiland	6328	2010	32.4	27.7	37.7
Botswana	Ngamiland	Ngamiland	6328	2015	19.5	16.5	22.9
Botswana	North East	North East	6329	2000	56.7	49.9	64.2
Botswana	North East	North East	6329	2005	44.7	39.3	50.7
Botswana	North East	North East	6329	2010	25.0	21.7	28.7
Botswana	North East	North East	6329	2015	15.3	12.9	17.9
Botswana	South-East	South-East	6330	2000	48.8	43.1	54.7
Botswana	South-East	South-East	6330	2005	39.3	34.6	44.7
Botswana	South-East	South-East	6330	2010	23.2	20.0	26.5
Botswana	South-East	South-East	6330	2015	14.5	12.0	17.1
Botswana	Southern	Southern	6331	2000	60.9	54.1	68.3
Botswana	Southern	Southern	6331	2005	44.6	39.6	50.5
Botswana	Southern	Southern	6331	2010	25.4	22.0	29.1
Botswana	Southern	Southern	6331	2015	15.9	13.6	18.5
Burkina Faso	Boucle Du Mouhoun	Bale	154444	2000	174.4	162.7	186.6
Burkina Faso	Boucle Du Mouhoun	Bale	154444	2005	147.4	138.1	157.9
Burkina Faso	Boucle Du Mouhoun	Bale	154444	2010	125.8	115.2	137.1
Burkina Faso	Boucle Du Mouhoun	Bale	154444	2015	112.7	101.5	125.1
Burkina Faso	Boucle Du Mouhoun	Banwa	154442	2000	185.5	172.9	198.7
Burkina Faso	Boucle Du Mouhoun	Banwa	154442	2005	160.3	150.1	172.1
Burkina Faso	Boucle Du Mouhoun	Banwa	154442	2010	136.0	125.0	148.4
Burkina Faso	Boucle Du Mouhoun	Banwa	154442	2015	120.8	108.6	135.5
Burkina Faso	Boucle Du Mouhoun	Kossi	154443	2000	182.8	170.9	194.8
Burkina Faso	Boucle Du Mouhoun	Kossi	154443	2005	157.9	147.6	168.1
Burkina Faso	Boucle Du Mouhoun	Kossi	154443	2010	130.5	120.2	141.0
Burkina Faso	Boucle Du Mouhoun	Kossi	154443	2015	114.4	102.8	127.8
Burkina Faso	Boucle Du Mouhoun	Mouhoun	154445	2000	162.8	152.2	174.1
Burkina Faso	Boucle Du Mouhoun	Mouhoun	154445	2005	139.1	129.9	148.0
Burkina Faso	Boucle Du Mouhoun	Mouhoun	154445	2010	117.4	107.4	128.0
Burkina Faso	Boucle Du Mouhoun	Mouhoun	154445	2015	106.9	95.7	119.6
Burkina Faso	Boucle Du Mouhoun	Nayala	154446	2000	156.8	145.3	168.0
Burkina Faso	Boucle Du Mouhoun	Nayala	154446	2005	132.9	123.5	143.3
Burkina Faso	Boucle Du Mouhoun	Nayala	154446	2010	108.8	99.1	118.8
Burkina Faso	Boucle Du Mouhoun	Nayala	154446	2015	101.1	90.2	113.4
Burkina Faso	Boucle Du Mouhoun	Sourou	154447	2000	166.1	154.7	177.7
Burkina Faso	Boucle Du Mouhoun	Sourou	154447	2005	141.1	130.9	151.3
Burkina Faso	Boucle Du Mouhoun	Sourou	154447	2010	114.7	105.3	124.3
Burkina Faso	Boucle Du Mouhoun	Sourou	154447	2015	104.4	94.2	116.0
Burkina Faso	Cascades	Comoe	154448	2000	204.8	193.3	216.3
Burkina Faso	Cascades	Comoe	154448	2005	187.9	177.1	198.6
Burkina Faso	Cascades	Comoe	154448	2010	157.5	146.5	169.6
Burkina Faso	Cascades	Comoe	154448	2015	136.9	123.3	151.4
Burkina Faso	Cascades	Leraba	154449	2000	208.1	194.7	221.9
Burkina Faso	Cascades	Leraba	154449	2005	189.2	175.2	202.6
Burkina Faso	Cascades	Leraba	154449	2010	156.3	143.0	169.7
Burkina Faso	Cascades	Leraba	154449	2015	134.8	120.5	150.3
Burkina Faso	Centre	Kadiogo	154450	2000	117.0	110.4	124.2

Admin 0	Admin 1	Admin 2	GAUL Code	Year	Under-5 mortality (per 1,000 livebirths)		
					Estimate	Lower bound, 95% UI	Upper bound, 95% UI
Burkina Faso	Centre	Kadiogo	154450	2005	100.6	94.8	106.9
Burkina Faso	Centre	Kadiogo	154450	2010	90.8	84.0	98.1
Burkina Faso	Centre	Kadiogo	154450	2015	70.1	63.0	78.2
Burkina Faso	Centre-est	Boulgou	154451	2000	156.2	147.6	164.1
Burkina Faso	Centre-est	Boulgou	154451	2005	132.6	125.0	140.1
Burkina Faso	Centre-est	Boulgou	154451	2010	112.6	104.9	120.8
Burkina Faso	Centre-est	Boulgou	154451	2015	88.9	80.4	98.3
Burkina Faso	Centre-est	Koulpelogo	154452	2000	165.3	153.5	177.5
Burkina Faso	Centre-est	Koulpelogo	154452	2005	146.0	135.9	156.6
Burkina Faso	Centre-est	Koulpelogo	154452	2010	125.5	115.5	136.3
Burkina Faso	Centre-est	Koulpelogo	154452	2015	101.5	90.9	112.8
Burkina Faso	Centre-est	Kouritenga	154453	2000	163.5	153.4	174.5
Burkina Faso	Centre-est	Kouritenga	154453	2005	143.1	134.9	152.3
Burkina Faso	Centre-est	Kouritenga	154453	2010	121.8	112.8	131.6
Burkina Faso	Centre-est	Kouritenga	154453	2015	102.5	92.7	113.3
Burkina Faso	Centre-nord	Bam	154454	2000	174.8	164.6	185.7
Burkina Faso	Centre-nord	Bam	154454	2005	152.3	143.1	161.9
Burkina Faso	Centre-nord	Bam	154454	2010	132.0	122.0	142.7
Burkina Faso	Centre-nord	Bam	154454	2015	119.2	107.0	132.2
Burkina Faso	Centre-nord	Namentenga	154455	2000	193.8	183.4	204.6
Burkina Faso	Centre-nord	Namentenga	154455	2005	171.5	162.0	181.4
Burkina Faso	Centre-nord	Namentenga	154455	2010	148.1	137.7	159.4
Burkina Faso	Centre-nord	Namentenga	154455	2015	132.7	119.9	146.6
Burkina Faso	Centre-nord	Sanmatenga	154456	2000	175.0	166.1	183.9
Burkina Faso	Centre-nord	Sanmatenga	154456	2005	153.7	145.5	161.9
Burkina Faso	Centre-nord	Sanmatenga	154456	2010	134.1	124.8	143.2
Burkina Faso	Centre-nord	Sanmatenga	154456	2015	121.4	109.7	133.6
Burkina Faso	Centre-ouest	Boulkiemde	154457	2000	166.4	158.1	175.1
Burkina Faso	Centre-ouest	Boulkiemde	154457	2005	146.5	138.6	154.6
Burkina Faso	Centre-ouest	Boulkiemde	154457	2010	124.5	115.5	134.3
Burkina Faso	Centre-ouest	Boulkiemde	154457	2015	107.6	97.4	119.0
Burkina Faso	Centre-ouest	Sanguie	154458	2000	163.2	154.0	172.9
Burkina Faso	Centre-ouest	Sanguie	154458	2005	141.6	133.5	150.3
Burkina Faso	Centre-ouest	Sanguie	154458	2010	118.7	109.4	128.0
Burkina Faso	Centre-ouest	Sanguie	154458	2015	105.9	95.7	117.6
Burkina Faso	Centre-ouest	Sissili	154459	2000	180.8	169.8	192.8
Burkina Faso	Centre-ouest	Sissili	154459	2005	154.5	144.5	164.8
Burkina Faso	Centre-ouest	Sissili	154459	2010	129.9	119.9	140.9
Burkina Faso	Centre-ouest	Sissili	154459	2015	110.5	98.7	122.9
Burkina Faso	Centre-ouest	Ziro	154460	2000	184.7	172.5	196.9
Burkina Faso	Centre-ouest	Ziro	154460	2005	159.3	148.8	171.0
Burkina Faso	Centre-ouest	Ziro	154460	2010	135.2	123.3	147.3
Burkina Faso	Centre-ouest	Ziro	154460	2015	113.9	101.3	127.3
Burkina Faso	Centre-sud	Bazega	154461	2000	170.3	160.7	180.1
Burkina Faso	Centre-sud	Bazega	154461	2005	143.6	135.2	151.6
Burkina Faso	Centre-sud	Bazega	154461	2010	127.9	118.4	137.6
Burkina Faso	Centre-sud	Bazega	154461	2015	102.6	92.6	113.5
Burkina Faso	Centre-sud	Nahouri	154462	2000	159.7	149.2	170.3
Burkina Faso	Centre-sud	Nahouri	154462	2005	131.5	123.1	140.3
Burkina Faso	Centre-sud	Nahouri	154462	2010	112.2	103.1	122.3
Burkina Faso	Centre-sud	Nahouri	154462	2015	86.1	77.1	96.7
Burkina Faso	Centre-sud	Zoundweogo	154463	2000	164.1	154.7	174.7
Burkina Faso	Centre-sud	Zoundweogo	154463	2005	137.1	128.4	145.5
Burkina Faso	Centre-sud	Zoundweogo	154463	2010	119.2	110.2	128.9
Burkina Faso	Centre-sud	Zoundweogo	154463	2015	92.2	83.0	103.0
Burkina Faso	Est	Gnagna	154464	2000	216.7	203.9	229.8
Burkina Faso	Est	Gnagna	154464	2005	187.4	175.8	199.4
Burkina Faso	Est	Gnagna	154464	2010	166.6	154.8	179.2
Burkina Faso	Est	Gnagna	154464	2015	145.2	130.7	160.6
Burkina Faso	Est	Gourma	154465	2000	201.6	188.2	215.5
Burkina Faso	Est	Gourma	154465	2005	176.9	165.7	188.7
Burkina Faso	Est	Gourma	154465	2010	149.1	138.1	162.0
Burkina Faso	Est	Gourma	154465	2015	124.9	112.3	138.7
Burkina Faso	Est	Komonjdjari	154466	2000	237.1	217.5	258.1
Burkina Faso	Est	Komonjdjari	154466	2005	207.8	190.1	225.1
Burkina Faso	Est	Komonjdjari	154466	2010	178.9	162.4	197.0
Burkina Faso	Est	Komonjdjari	154466	2015	151.3	134.0	170.9
Burkina Faso	Est	Kompienga	154467	2000	195.8	180.7	212.0
Burkina Faso	Est	Kompienga	154467	2005	170.4	157.4	184.5
Burkina Faso	Est	Kompienga	154467	2010	150.9	136.9	166.5
Burkina Faso	Est	Kompienga	154467	2015	120.7	106.3	135.4
Burkina Faso	Est	Tapoa	154468	2000	232.1	215.7	248.6
Burkina Faso	Est	Tapoa	154468	2005	203.4	189.7	218.3
Burkina Faso	Est	Tapoa	154468	2010	169.3	155.4	185.6
Burkina Faso	Est	Tapoa	154468	2015	142.7	127.9	159.5
Burkina Faso	Hauts-bassins	Houet	154469	2000	165.3	155.8	174.8
Burkina Faso	Hauts-bassins	Houet	154469	2005	151.8	142.6	161.1
Burkina Faso	Hauts-bassins	Houet	154469	2010	123.1	114.1	132.9
Burkina Faso	Hauts-bassins	Houet	154469	2015	110.4	99.5	122.2
Burkina Faso	Hauts-bassins	Kenedougou	154470	2000	193.0	182.0	205.4
Burkina Faso	Hauts-bassins	Kenedougou	154470	2005	179.9	169.0	192.8
Burkina Faso	Hauts-bassins	Kenedougou	154470	2010	146.4	135.2	158.4
Burkina Faso	Hauts-bassins	Kenedougou	154470	2015	130.8	118.2	145.2
Burkina Faso	Hauts-bassins	Tuy	154471	2000	182.4	169.9	194.7
Burkina Faso	Hauts-bassins	Tuy	154471	2005	164.5	153.7	175.8

Admin 0	Admin 1	Admin 2	GAUL Code	Year	Under-5 mortality (per 1,000 livebirths)		
					Estimate	Lower bound, 95% UI	Upper bound, 95% UI
Burkina Faso	Hauts-bassins	Tuy	154471	2010	136.6	125.8	148.7
Burkina Faso	Hauts-bassins	Tuy	154471	2015	119.6	107.5	133.3
Burkina Faso	Nord	Loroum	154472	2000	212.2	198.8	225.8
Burkina Faso	Nord	Loroum	154472	2005	181.8	169.9	194.6
Burkina Faso	Nord	Loroum	154472	2010	159.5	146.2	173.5
Burkina Faso	Nord	Loroum	154472	2015	138.2	123.0	154.5
Burkina Faso	Nord	Passore	154473	2000	168.4	159.6	177.7
Burkina Faso	Nord	Passore	154473	2005	145.3	136.7	153.5
Burkina Faso	Nord	Passore	154473	2010	125.2	115.4	134.6
Burkina Faso	Nord	Passore	154473	2015	112.8	102.3	124.4
Burkina Faso	Nord	Yatenga	154474	2000	190.7	180.7	200.7
Burkina Faso	Nord	Yatenga	154474	2005	162.6	153.7	171.3
Burkina Faso	Nord	Yatenga	154474	2010	137.7	127.8	147.7
Burkina Faso	Nord	Yatenga	154474	2015	123.2	111.4	135.9
Burkina Faso	Nord	Zondoma	154475	2000	180.5	168.5	192.6
Burkina Faso	Nord	Zondoma	154475	2005	154.2	144.3	164.5
Burkina Faso	Nord	Zondoma	154475	2010	128.5	118.3	139.2
Burkina Faso	Nord	Zondoma	154475	2015	115.9	104.1	128.4
Burkina Faso	Plateau Central	Ganzourgou	154476	2000	167.7	158.5	177.2
Burkina Faso	Plateau Central	Ganzourgou	154476	2005	143.8	136.2	152.1
Burkina Faso	Plateau Central	Ganzourgou	154476	2010	126.6	118.2	135.5
Burkina Faso	Plateau Central	Ganzourgou	154476	2015	106.1	97.1	116.7
Burkina Faso	Plateau Central	Kourweogo	154477	2000	165.7	155.7	175.4
Burkina Faso	Plateau Central	Kourweogo	154477	2005	147.1	138.4	156.4
Burkina Faso	Plateau Central	Kourweogo	154477	2010	130.6	120.8	140.8
Burkina Faso	Plateau Central	Kourweogo	154477	2015	114.6	103.4	127.0
Burkina Faso	Plateau Central	Oubritenga	154478	2000	163.5	154.8	172.3
Burkina Faso	Plateau Central	Oubritenga	154478	2005	143.3	135.2	151.3
Burkina Faso	Plateau Central	Oubritenga	154478	2010	127.4	118.4	136.6
Burkina Faso	Plateau Central	Oubritenga	154478	2015	110.4	100.2	121.4
Burkina Faso	Sahel	Oudalan	154479	2000	217.5	201.8	234.5
Burkina Faso	Sahel	Oudalan	154479	2005	191.0	176.4	206.9
Burkina Faso	Sahel	Oudalan	154479	2010	182.0	165.0	200.7
Burkina Faso	Sahel	Oudalan	154479	2015	153.7	136.1	173.9
Burkina Faso	Sahel	Seno	154480	2000	253.2	236.6	271.7
Burkina Faso	Sahel	Seno	154480	2005	225.3	210.7	240.5
Burkina Faso	Sahel	Seno	154480	2010	205.8	187.8	224.6
Burkina Faso	Sahel	Seno	154480	2015	174.0	154.3	194.2
Burkina Faso	Sahel	Soum	154481	2000	207.5	195.0	221.3
Burkina Faso	Sahel	Soum	154481	2005	182.5	171.2	194.0
Burkina Faso	Sahel	Soum	154481	2010	161.8	150.5	174.4
Burkina Faso	Sahel	Soum	154481	2015	140.2	125.9	155.9
Burkina Faso	Sahel	Yagha	154482	2000	234.3	216.4	253.5
Burkina Faso	Sahel	Yagha	154482	2005	206.9	191.6	223.2
Burkina Faso	Sahel	Yagha	154482	2010	185.0	169.2	202.0
Burkina Faso	Sahel	Yagha	154482	2015	155.7	139.1	175.5
Burkina Faso	Sud-ouest	Bougouriba	154483	2000	228.4	213.1	245.5
Burkina Faso	Sud-ouest	Bougouriba	154483	2005	199.4	185.9	213.2
Burkina Faso	Sud-ouest	Bougouriba	154483	2010	171.9	157.2	186.9
Burkina Faso	Sud-ouest	Bougouriba	154483	2015	145.7	130.7	161.9
Burkina Faso	Sud-ouest	Ioba	154484	2000	199.9	188.4	212.6
Burkina Faso	Sud-ouest	Ioba	154484	2005	169.8	159.6	180.2
Burkina Faso	Sud-ouest	Ioba	154484	2010	147.7	135.5	159.2
Burkina Faso	Sud-ouest	Ioba	154484	2015	124.4	112.2	138.2
Burkina Faso	Sud-ouest	Noumbiel	154485	2000	204.6	189.9	219.2
Burkina Faso	Sud-ouest	Noumbiel	154485	2005	189.3	176.0	202.9
Burkina Faso	Sud-ouest	Noumbiel	154485	2010	157.5	143.1	171.3
Burkina Faso	Sud-ouest	Noumbiel	154485	2015	130.6	115.5	146.8
Burkina Faso	Sud-ouest	Poni	154486	2000	231.2	217.7	245.4
Burkina Faso	Sud-ouest	Poni	154486	2005	208.4	196.5	221.1
Burkina Faso	Sud-ouest	Poni	154486	2010	176.9	163.3	190.6
Burkina Faso	Sud-ouest	Poni	154486	2015	148.7	133.9	165.0
Burundi	Bubanza	Bubanza	40559	2000	182.8	172.4	193.8
Burundi	Bubanza	Bubanza	40559	2005	134.0	126.1	142.7
Burundi	Bubanza	Bubanza	40559	2010	103.3	96.8	110.2
Burundi	Bubanza	Bubanza	40559	2015	92.4	84.1	101.3
Burundi	Bubanza	Gihanga	48356	2000	174.2	164.7	184.0
Burundi	Bubanza	Gihanga	48356	2005	130.6	123.3	138.4
Burundi	Bubanza	Gihanga	48356	2010	102.5	96.2	109.2
Burundi	Bubanza	Gihanga	48356	2015	90.0	81.8	98.6
Burundi	Bubanza	Mpanda	40561	2000	171.0	162.3	180.5
Burundi	Bubanza	Mpanda	40561	2005	126.7	119.9	134.4
Burundi	Bubanza	Mpanda	40561	2010	97.6	91.6	103.8
Burundi	Bubanza	Mpanda	40561	2015	84.2	76.7	92.2
Burundi	Bubanza	Musigati	40562	2000	175.6	166.6	185.3
Burundi	Bubanza	Musigati	40562	2005	130.2	123.4	137.7
Burundi	Bubanza	Musigati	40562	2010	99.0	93.5	105.1
Burundi	Bubanza	Musigati	40562	2015	85.2	77.9	92.9
Burundi	Bubanza	Rugazi	40563	2000	160.0	152.0	168.6
Burundi	Bubanza	Rugazi	40563	2005	122.8	116.4	129.7
Burundi	Bubanza	Rugazi	40563	2010	93.3	87.5	98.7
Burundi	Bubanza	Rugazi	40563	2015	75.7	69.2	82.6
Burundi	Bujumbura Mairie	Buterere	40565	2000	138.9	131.3	147.2
Burundi	Bujumbura Mairie	Buterere	40565	2005	114.2	107.6	121.2
Burundi	Bujumbura Mairie	Buterere	40565	2010	89.6	83.5	95.7

Admin 0	Admin 1	Admin 2	GAUL Code	Year	Under-5 mortality (per 1,000 livebirths)		
					Estimate	Lower bound, 95% UI	Upper bound, 95% UI
Burundi	Bujumbura Mairie	Buterere	40565	2015	78.2	71.1	85.8
Burundi	Bujumbura Mairie	Buyenzi	40566	2000	110.5	104.3	117.5
Burundi	Bujumbura Mairie	Buyenzi	40566	2005	100.7	94.5	107.2
Burundi	Bujumbura Mairie	Buyenzi	40566	2010	81.1	75.2	86.7
Burundi	Bujumbura Mairie	Buyenzi	40566	2015	70.5	63.9	77.9
Burundi	Bujumbura Mairie	Bwiza	40567	2000	110.5	104.3	117.5
Burundi	Bujumbura Mairie	Bwiza	40567	2005	100.7	94.5	107.2
Burundi	Bujumbura Mairie	Bwiza	40567	2010	81.1	75.2	86.7
Burundi	Bujumbura Mairie	Bwiza	40567	2015	70.5	63.9	77.9
Burundi	Bujumbura Mairie	Cibitoke	40568	2000	110.5	104.3	117.5
Burundi	Bujumbura Mairie	Cibitoke	40568	2005	100.7	94.5	107.2
Burundi	Bujumbura Mairie	Cibitoke	40568	2010	81.1	75.2	86.7
Burundi	Bujumbura Mairie	Cibitoke	40568	2015	70.5	63.9	77.9
Burundi	Bujumbura Mairie	Gihosha	40569	2000	117.3	111.1	124.3
Burundi	Bujumbura Mairie	Gihosha	40569	2005	102.9	96.8	109.1
Burundi	Bujumbura Mairie	Gihosha	40569	2010	80.9	75.4	86.3
Burundi	Bujumbura Mairie	Gihosha	40569	2015	70.2	63.7	77.6
Burundi	Bujumbura Mairie	Kamenge	40570	2000	117.3	111.1	124.3
Burundi	Bujumbura Mairie	Kamenge	40570	2005	102.9	96.8	109.1
Burundi	Bujumbura Mairie	Kamenge	40570	2010	80.9	75.4	86.3
Burundi	Bujumbura Mairie	Kamenge	40570	2015	70.2	63.7	77.6
Burundi	Bujumbura Mairie	Kanyosha	40571	2000	121.0	113.8	129.8
Burundi	Bujumbura Mairie	Kanyosha	40571	2005	105.0	98.1	112.5
Burundi	Bujumbura Mairie	Kanyosha	40571	2010	82.9	76.9	89.2
Burundi	Bujumbura Mairie	Kanyosha	40571	2015	70.4	63.5	77.7
Burundi	Bujumbura Mairie	Kinama	40572	2000	134.0	127.0	141.3
Burundi	Bujumbura Mairie	Kinama	40572	2005	113.5	107.3	120.2
Burundi	Bujumbura Mairie	Kinama	40572	2010	88.0	82.5	93.8
Burundi	Bujumbura Mairie	Kinama	40572	2015	74.9	68.3	82.3
Burundi	Bujumbura Mairie	Kinindo	40573	2000	106.9	100.6	114.2
Burundi	Bujumbura Mairie	Kinindo	40573	2005	98.8	92.4	105.6
Burundi	Bujumbura Mairie	Kinindo	40573	2010	79.9	74.1	85.6
Burundi	Bujumbura Mairie	Kinindo	40573	2015	69.0	62.2	76.2
Burundi	Bujumbura Mairie	Musaga	40574	2000	106.9	100.6	114.2
Burundi	Bujumbura Mairie	Musaga	40574	2005	98.8	92.4	105.6
Burundi	Bujumbura Mairie	Musaga	40574	2010	79.9	74.1	85.6
Burundi	Bujumbura Mairie	Musaga	40574	2015	69.0	62.2	76.2
Burundi	Bujumbura Mairie	Ngagara	40575	2000	110.5	104.3	117.5
Burundi	Bujumbura Mairie	Ngagara	40575	2005	100.7	94.5	107.2
Burundi	Bujumbura Mairie	Ngagara	40575	2010	81.1	75.2	86.7
Burundi	Bujumbura Mairie	Ngagara	40575	2015	70.5	63.9	77.9
Burundi	Bujumbura Mairie	Nyakabiga	40576	2000	119.7	113.0	127.4
Burundi	Bujumbura Mairie	Nyakabiga	40576	2005	102.7	96.5	109.4
Burundi	Bujumbura Mairie	Nyakabiga	40576	2010	81.4	75.9	87.3
Burundi	Bujumbura Mairie	Nyakabiga	40576	2015	71.0	64.2	78.4
Burundi	Bujumbura Mairie	Rohero	40577	2000	111.7	105.2	119.1
Burundi	Bujumbura Mairie	Rohero	40577	2005	100.2	94.0	106.9
Burundi	Bujumbura Mairie	Rohero	40577	2010	80.5	74.7	86.2
Burundi	Bujumbura Mairie	Rohero	40577	2015	69.7	63.1	77.2
Burundi	Bujumbura Rural	Bugarama	40580	2000	135.1	126.0	144.2
Burundi	Bujumbura Rural	Bugarama	40580	2005	111.3	104.1	119.2
Burundi	Bujumbura Rural	Bugarama	40580	2010	88.1	81.8	95.1
Burundi	Bujumbura Rural	Bugarama	40580	2015	71.8	64.7	79.2
Burundi	Bujumbura Rural	Isale	40581	2000	141.1	134.1	148.7
Burundi	Bujumbura Rural	Isale	40581	2005	112.5	106.5	118.8
Burundi	Bujumbura Rural	Isale	40581	2010	86.1	80.4	91.5
Burundi	Bujumbura Rural	Isale	40581	2015	69.8	63.6	76.6
Burundi	Bujumbura Rural	Kabezi	40582	2000	136.2	127.9	146.0
Burundi	Bujumbura Rural	Kabezi	40582	2005	110.6	103.2	118.7
Burundi	Bujumbura Rural	Kabezi	40582	2010	86.7	80.7	93.6
Burundi	Bujumbura Rural	Kabezi	40582	2015	70.9	64.0	78.7
Burundi	Bujumbura Rural	Kanyosha1	40753	2000	131.1	124.2	139.2
Burundi	Bujumbura Rural	Kanyosha1	40753	2005	110.1	103.6	116.9
Burundi	Bujumbura Rural	Kanyosha1	40753	2010	85.1	79.6	91.0
Burundi	Bujumbura Rural	Kanyosha1	40753	2015	70.4	63.8	77.8
Burundi	Bujumbura Rural	Mubimbi	40584	2000	149.3	142.2	157.2
Burundi	Bujumbura Rural	Mubimbi	40584	2005	115.1	109.3	121.7
Burundi	Bujumbura Rural	Mubimbi	40584	2010	89.0	83.4	94.2
Burundi	Bujumbura Rural	Mubimbi	40584	2015	72.0	65.8	78.9
Burundi	Bujumbura Rural	Mugongomanga	40754	2000	129.7	123.1	137.0
Burundi	Bujumbura Rural	Mugongomanga	40754	2005	106.3	100.4	112.2
Burundi	Bujumbura Rural	Mugongomanga	40754	2010	83.1	78.1	88.4
Burundi	Bujumbura Rural	Mugongomanga	40754	2015	66.2	60.5	72.7
Burundi	Bujumbura Rural	Muhuta	40586	2000	134.8	126.1	144.2
Burundi	Bujumbura Rural	Muhuta	40586	2005	110.2	103.1	117.7
Burundi	Bujumbura Rural	Muhuta	40586	2010	86.7	80.7	93.9
Burundi	Bujumbura Rural	Muhuta	40586	2015	70.3	63.4	77.9
Burundi	Bujumbura Rural	Mukike	40755	2000	129.6	122.4	137.6
Burundi	Bujumbura Rural	Mukike	40755	2005	107.8	101.6	114.5
Burundi	Bujumbura Rural	Mukike	40755	2010	84.0	79.0	89.8
Burundi	Bujumbura Rural	Mukike	40755	2015	67.5	61.6	74.2
Burundi	Bujumbura Rural	Mutambu	40756	2000	134.0	126.2	142.8
Burundi	Bujumbura Rural	Mutambu	40756	2005	108.1	101.6	115.4
Burundi	Bujumbura Rural	Mutambu	40756	2010	84.6	79.2	90.9
Burundi	Bujumbura Rural	Mutambu	40756	2015	68.4	62.2	75.7

Admin 0	Admin 1	Admin 2	GAUL Code	Year	Under-5 mortality (per 1,000 livebirths)		
					Estimate	Lower bound, 95% UI	Upper bound, 95% UI
Burundi	Bujumbura Rural	Mutimbuzi	40589	2000	150.3	142.4	158.9
Burundi	Bujumbura Rural	Mutimbuzi	40589	2005	121.6	114.8	129.1
Burundi	Bujumbura Rural	Mutimbuzi	40589	2010	94.3	88.0	100.7
Burundi	Bujumbura Rural	Mutimbuzi	40589	2015	81.0	73.7	89.0
Burundi	Bujumbura Rural	Nyabiraba	40757	2000	134.8	127.9	142.6
Burundi	Bujumbura Rural	Nyabiraba	40757	2005	108.5	102.4	115.1
Burundi	Bujumbura Rural	Nyabiraba	40757	2010	84.5	79.2	90.2
Burundi	Bujumbura Rural	Nyabiraba	40757	2015	67.6	61.5	74.6
Burundi	Bururi	Burambi	40591	2000	132.0	122.5	141.2
Burundi	Bururi	Burambi	40591	2005	108.2	101.1	115.6
Burundi	Bururi	Burambi	40591	2010	85.9	79.4	92.8
Burundi	Bururi	Burambi	40591	2015	70.3	63.6	77.7
Burundi	Bururi	Bururi	40592	2000	119.5	111.1	128.2
Burundi	Bururi	Bururi	40592	2005	100.9	94.5	108.1
Burundi	Bururi	Bururi	40592	2010	79.4	73.5	85.6
Burundi	Bururi	Bururi	40592	2015	64.0	57.5	70.9
Burundi	Bururi	Buyengero	40593	2000	128.8	118.9	138.3
Burundi	Bururi	Buyengero	40593	2005	105.5	98.2	113.0
Burundi	Bururi	Buyengero	40593	2010	83.6	77.0	90.2
Burundi	Bururi	Buyengero	40593	2015	67.3	60.6	74.7
Burundi	Bururi	Matana	40594	2000	122.7	113.3	132.8
Burundi	Bururi	Matana	40594	2005	102.4	95.5	110.1
Burundi	Bururi	Matana	40594	2010	81.0	75.1	87.4
Burundi	Bururi	Matana	40594	2015	64.7	58.5	71.6
Burundi	Bururi	Mugamba	40595	2000	125.3	117.1	134.4
Burundi	Bururi	Mugamba	40595	2005	104.9	98.2	112.2
Burundi	Bururi	Mugamba	40595	2010	81.8	76.4	87.9
Burundi	Bururi	Mugamba	40595	2015	65.1	59.3	71.6
Burundi	Bururi	Rumonge	40596	2000	126.8	117.1	137.3
Burundi	Bururi	Rumonge	40596	2005	105.4	97.5	114.1
Burundi	Bururi	Rumonge	40596	2010	83.5	75.8	90.9
Burundi	Bururi	Rumonge	40596	2015	69.0	61.6	77.0
Burundi	Bururi	Rutovu	40597	2000	119.7	111.5	128.4
Burundi	Bururi	Rutovu	40597	2005	103.1	96.8	109.7
Burundi	Bururi	Rutovu	40597	2010	81.0	75.2	87.0
Burundi	Bururi	Rutovu	40597	2015	67.2	60.5	74.0
Burundi	Bururi	Songa	40598	2000	122.0	112.4	132.1
Burundi	Bururi	Songa	40598	2005	101.5	94.6	108.9
Burundi	Bururi	Songa	40598	2010	80.2	74.2	86.8
Burundi	Bururi	Songa	40598	2015	63.5	57.2	70.4
Burundi	Bururi	Vyanda	40599	2000	122.8	113.7	132.4
Burundi	Bururi	Vyanda	40599	2005	103.0	95.8	110.9
Burundi	Bururi	Vyanda	40599	2010	80.6	73.9	87.3
Burundi	Bururi	Vyanda	40599	2015	63.7	56.9	70.9
Burundi	Cankuzo	Cankuzo	40600	2000	147.0	136.7	157.2
Burundi	Cankuzo	Cankuzo	40600	2005	121.9	113.9	130.2
Burundi	Cankuzo	Cankuzo	40600	2010	96.6	89.7	104.0
Burundi	Cankuzo	Cankuzo	40600	2015	83.8	76.1	91.9
Burundi	Cankuzo	Cendajuru	40601	2000	136.7	126.0	147.6
Burundi	Cankuzo	Cendajuru	40601	2005	113.8	105.4	123.2
Burundi	Cankuzo	Cendajuru	40601	2010	91.9	84.7	99.5
Burundi	Cankuzo	Cendajuru	40601	2015	81.6	73.3	90.4
Burundi	Cankuzo	Gisagara	40602	2000	135.6	124.8	146.6
Burundi	Cankuzo	Gisagara	40602	2005	112.8	104.3	121.9
Burundi	Cankuzo	Gisagara	40602	2010	91.1	83.2	98.6
Burundi	Cankuzo	Gisagara	40602	2015	79.5	71.1	87.7
Burundi	Cankuzo	Kigamba	40603	2000	160.7	149.4	172.2
Burundi	Cankuzo	Kigamba	40603	2005	131.3	122.1	140.3
Burundi	Cankuzo	Kigamba	40603	2010	104.5	96.6	113.1
Burundi	Cankuzo	Kigamba	40603	2015	90.5	82.2	99.7
Burundi	Cankuzo	Mishiha	40604	2000	145.4	134.3	157.7
Burundi	Cankuzo	Mishiha	40604	2005	120.3	111.2	130.2
Burundi	Cankuzo	Mishiha	40604	2010	96.8	88.0	105.2
Burundi	Cankuzo	Mishiha	40604	2015	85.0	76.2	94.0
Burundi	Cibitoke	Buganda	40605	2000	200.9	187.4	215.0
Burundi	Cibitoke	Buganda	40605	2005	144.3	134.8	153.8
Burundi	Cibitoke	Buganda	40605	2010	111.5	103.4	120.2
Burundi	Cibitoke	Buganda	40605	2015	104.3	94.1	115.4
Burundi	Cibitoke	Bukinanyana	40606	2000	187.7	177.8	197.9
Burundi	Cibitoke	Bukinanyana	40606	2005	133.4	126.1	140.8
Burundi	Cibitoke	Bukinanyana	40606	2010	102.3	96.2	108.9
Burundi	Cibitoke	Bukinanyana	40606	2015	90.6	82.8	99.4
Burundi	Cibitoke	Mabayi	40607	2000	179.7	170.4	189.7
Burundi	Cibitoke	Mabayi	40607	2005	126.9	119.8	134.1
Burundi	Cibitoke	Mabayi	40607	2010	94.8	88.5	100.9
Burundi	Cibitoke	Mabayi	40607	2015	83.4	76.0	91.3
Burundi	Cibitoke	Mugina	40608	2000	176.8	166.4	187.8
Burundi	Cibitoke	Mugina	40608	2005	125.3	117.3	133.1
Burundi	Cibitoke	Mugina	40608	2010	94.1	87.2	101.0
Burundi	Cibitoke	Mugina	40608	2015	84.9	76.7	93.3
Burundi	Cibitoke	Murwi	40609	2000	198.5	186.4	211.1
Burundi	Cibitoke	Murwi	40609	2005	139.1	130.8	147.9
Burundi	Cibitoke	Murwi	40609	2010	108.1	100.9	115.8
Burundi	Cibitoke	Murwi	40609	2015	97.5	88.1	107.3
Burundi	Cibitoke	Rugombo	40610	2000	204.0	189.7	219.0

Admin 0	Admin 1	Admin 2	GAUL Code	Year	Under-5 mortality (per 1,000 livebirths)		
					Estimate	Lower bound, 95% UI	Upper bound, 95% UI
Burundi	Cibitoke	Rugombo	40610	2005	143.2	132.9	154.0
Burundi	Cibitoke	Rugombo	40610	2010	109.4	101.0	118.5
Burundi	Cibitoke	Rugombo	40610	2015	100.8	90.0	111.5
Burundi	Gitega	Bugendana	40611	2000	151.5	142.8	160.8
Burundi	Gitega	Bugendana	40611	2005	118.4	112.3	124.9
Burundi	Gitega	Bugendana	40611	2010	94.0	87.9	99.7
Burundi	Gitega	Bugendana	40611	2015	80.2	73.5	87.3
Burundi	Gitega	Bukirasazi	40612	2000	126.9	118.6	136.2
Burundi	Gitega	Bukirasazi	40612	2005	106.8	99.9	113.9
Burundi	Gitega	Bukirasazi	40612	2010	85.7	79.4	91.9
Burundi	Gitega	Bukirasazi	40612	2015	72.1	64.8	79.4
Burundi	Gitega	Buraza	40613	2000	124.9	116.4	134.1
Burundi	Gitega	Buraza	40613	2005	105.2	98.3	112.3
Burundi	Gitega	Buraza	40613	2010	83.8	77.6	90.0
Burundi	Gitega	Buraza	40613	2015	70.1	62.9	77.4
Burundi	Gitega	Giheta	40614	2000	144.4	135.1	153.8
Burundi	Gitega	Giheta	40614	2005	113.8	107.2	120.8
Burundi	Gitega	Giheta	40614	2010	91.0	84.9	97.0
Burundi	Gitega	Giheta	40614	2015	76.7	70.0	84.1
Burundi	Gitega	Gishubi	40615	2000	129.4	121.7	138.0
Burundi	Gitega	Gishubi	40615	2005	105.2	98.7	111.4
Burundi	Gitega	Gishubi	40615	2010	85.1	79.5	90.7
Burundi	Gitega	Gishubi	40615	2015	71.3	64.8	78.3
Burundi	Gitega	Gitega	40616	2000	135.4	126.2	144.6
Burundi	Gitega	Gitega	40616	2005	109.1	102.1	116.4
Burundi	Gitega	Gitega	40616	2010	88.2	82.0	94.6
Burundi	Gitega	Gitega	40616	2015	74.8	67.7	82.6
Burundi	Gitega	Itaba	40617	2000	132.5	124.1	141.3
Burundi	Gitega	Itaba	40617	2005	110.2	103.3	117.3
Burundi	Gitega	Itaba	40617	2010	89.1	82.7	95.1
Burundi	Gitega	Itaba	40617	2015	77.4	69.8	84.9
Burundi	Gitega	Makebuko	40618	2000	133.5	125.6	142.6
Burundi	Gitega	Makebuko	40618	2005	108.4	101.4	115.4
Burundi	Gitega	Makebuko	40618	2010	88.1	82.2	94.1
Burundi	Gitega	Makebuko	40618	2015	74.0	66.9	81.2
Burundi	Gitega	Mutaho	40619	2000	163.9	155.7	173.0
Burundi	Gitega	Mutaho	40619	2005	127.6	121.2	134.2
Burundi	Gitega	Mutaho	40619	2010	98.6	92.8	104.5
Burundi	Gitega	Mutaho	40619	2015	83.4	76.4	90.7
Burundi	Gitega	Nyanrusange	40620	2000	130.0	121.7	138.5
Burundi	Gitega	Nyanrusange	40620	2005	105.0	98.7	111.7
Burundi	Gitega	Nyanrusange	40620	2010	85.0	79.6	90.8
Burundi	Gitega	Nyanrusange	40620	2015	71.0	64.6	77.8
Burundi	Gitega	Ryansoro	40621	2000	125.4	117.1	134.0
Burundi	Gitega	Ryansoro	40621	2005	104.1	97.6	111.0
Burundi	Gitega	Ryansoro	40621	2010	83.5	77.9	89.4
Burundi	Gitega	Ryansoro	40621	2015	69.6	63.0	76.6
Burundi	Karuzi	Bugenyuzi	40622	2000	176.3	166.8	186.7
Burundi	Karuzi	Bugenyuzi	40622	2005	139.8	132.6	147.7
Burundi	Karuzi	Bugenyuzi	40622	2010	108.7	102.0	115.6
Burundi	Karuzi	Bugenyuzi	40622	2015	96.9	88.5	105.8
Burundi	Karuzi	Buhiga	40623	2000	172.9	162.0	185.1
Burundi	Karuzi	Buhiga	40623	2005	139.1	130.9	148.0
Burundi	Karuzi	Buhiga	40623	2010	109.4	101.5	117.5
Burundi	Karuzi	Buhiga	40623	2015	96.6	87.7	106.2
Burundi	Karuzi	Gihogazi	40624	2000	169.9	160.9	179.6
Burundi	Karuzi	Gihogazi	40624	2005	135.2	128.8	142.3
Burundi	Karuzi	Gihogazi	40624	2010	104.2	97.9	110.7
Burundi	Karuzi	Gihogazi	40624	2015	90.2	82.8	98.2
Burundi	Karuzi	Gitaramuka	40625	2000	189.2	178.6	200.2
Burundi	Karuzi	Gitaramuka	40625	2005	149.2	141.3	158.0
Burundi	Karuzi	Gitaramuka	40625	2010	116.3	108.6	124.3
Burundi	Karuzi	Gitaramuka	40625	2015	101.9	92.7	111.8
Burundi	Karuzi	Mutumba	40626	2000	156.1	145.0	168.2
Burundi	Karuzi	Mutumba	40626	2005	125.3	116.5	134.3
Burundi	Karuzi	Mutumba	40626	2010	99.5	91.8	107.3
Burundi	Karuzi	Mutumba	40626	2015	89.1	80.4	99.0
Burundi	Karuzi	Nyabikere	40627	2000	154.2	144.3	164.7
Burundi	Karuzi	Nyabikere	40627	2005	123.7	116.2	131.8
Burundi	Karuzi	Nyabikere	40627	2010	98.4	91.5	105.5
Burundi	Karuzi	Nyabikere	40627	2015	88.5	80.3	97.4
Burundi	Karuzi	Shombo	40628	2000	153.8	144.7	163.6
Burundi	Karuzi	Shombo	40628	2005	123.2	116.3	130.5
Burundi	Karuzi	Shombo	40628	2010	97.6	91.3	104.0
Burundi	Karuzi	Shombo	40628	2015	87.0	79.4	95.2
Burundi	Kayanza	Butaganzwa	40629	2000	161.2	153.0	170.1
Burundi	Kayanza	Butaganzwa	40629	2005	124.1	118.0	130.9
Burundi	Kayanza	Butaganzwa	40629	2010	92.5	87.0	98.2
Burundi	Kayanza	Butaganzwa	40629	2015	75.2	68.9	82.1
Burundi	Kayanza	Gahombo	40630	2000	176.1	168.0	184.3
Burundi	Kayanza	Gahombo	40630	2005	133.3	127.5	139.6
Burundi	Kayanza	Gahombo	40630	2010	99.5	93.6	105.4
Burundi	Kayanza	Gahombo	40630	2015	77.1	71.1	83.9
Burundi	Kayanza	Gatara	40631	2000	169.6	162.0	177.9
Burundi	Kayanza	Gatara	40631	2005	125.8	120.3	131.7

Admin 0	Admin 1	Admin 2	GAUL Code	Year	Under-5 mortality (per 1,000 livebirths)		
					Estimate	Lower bound, 95% UI	Upper bound, 95% UI
Burundi	Kayanza	Gatara	40631	2010	94.2	88.7	99.7
Burundi	Kayanza	Gatara	40631	2015	73.1	67.4	79.6
Burundi	Kayanza	Kabarore	40632	2000	175.3	166.8	184.4
Burundi	Kayanza	Kabarore	40632	2005	128.1	121.4	135.3
Burundi	Kayanza	Kabarore	40632	2010	95.7	89.6	101.8
Burundi	Kayanza	Kabarore	40632	2015	80.8	74.2	88.4
Burundi	Kayanza	Kayanza	40633	2000	173.8	165.7	181.8
Burundi	Kayanza	Kayanza	40633	2005	127.4	121.7	133.8
Burundi	Kayanza	Kayanza	40633	2010	95.5	89.7	101.5
Burundi	Kayanza	Kayanza	40633	2015	75.7	69.6	82.5
Burundi	Kayanza	Matongo	40634	2000	163.0	155.0	171.5
Burundi	Kayanza	Matongo	40634	2005	123.6	117.9	129.8
Burundi	Kayanza	Matongo	40634	2010	92.0	86.8	97.6
Burundi	Kayanza	Matongo	40634	2015	72.7	66.9	79.3
Burundi	Kayanza	Muhanga	40635	2000	182.1	173.3	191.7
Burundi	Kayanza	Muhanga	40635	2005	143.0	136.2	150.5
Burundi	Kayanza	Muhanga	40635	2010	105.7	99.3	112.1
Burundi	Kayanza	Muhanga	40635	2015	88.9	81.7	97.0
Burundi	Kayanza	Muruta	40636	2000	168.0	160.2	176.5
Burundi	Kayanza	Muruta	40636	2005	124.1	118.1	130.7
Burundi	Kayanza	Muruta	40636	2010	93.9	88.3	99.4
Burundi	Kayanza	Muruta	40636	2015	76.3	70.2	83.4
Burundi	Kayanza	Rango	40637	2000	165.4	157.1	174.3
Burundi	Kayanza	Rango	40637	2005	131.1	124.7	138.4
Burundi	Kayanza	Rango	40637	2010	96.9	91.0	102.6
Burundi	Kayanza	Rango	40637	2015	79.0	72.5	86.1
Burundi	Kirundo	Bugabira	40638	2000	221.0	207.8	233.9
Burundi	Kirundo	Bugabira	40638	2005	154.3	145.4	163.5
Burundi	Kirundo	Bugabira	40638	2010	111.5	104.2	119.3
Burundi	Kirundo	Bugabira	40638	2015	108.2	98.5	119.2
Burundi	Kirundo	Busoni	40639	2000	234.7	223.2	245.9
Burundi	Kirundo	Busoni	40639	2005	169.1	159.8	177.7
Burundi	Kirundo	Busoni	40639	2010	122.4	115.2	129.8
Burundi	Kirundo	Busoni	40639	2015	115.6	105.2	126.8
Burundi	Kirundo	Bwambarangwe	40640	2000	234.7	221.0	249.7
Burundi	Kirundo	Bwambarangwe	40640	2005	177.0	165.6	188.0
Burundi	Kirundo	Bwambarangwe	40640	2010	129.4	120.9	138.4
Burundi	Kirundo	Bwambarangwe	40640	2015	118.7	107.7	131.0
Burundi	Kirundo	Gitobe	40641	2000	234.8	221.6	248.6
Burundi	Kirundo	Gitobe	40641	2005	178.8	167.9	189.0
Burundi	Kirundo	Gitobe	40641	2010	131.5	123.2	140.2
Burundi	Kirundo	Gitobe	40641	2015	124.1	112.8	136.8
Burundi	Kirundo	Kirundo	40642	2000	232.2	220.7	243.0
Burundi	Kirundo	Kirundo	40642	2005	167.4	159.1	175.4
Burundi	Kirundo	Kirundo	40642	2010	121.9	114.7	129.4
Burundi	Kirundo	Kirundo	40642	2015	116.7	106.7	127.8
Burundi	Kirundo	Ntega	40643	2000	224.5	213.4	235.2
Burundi	Kirundo	Ntega	40643	2005	163.3	155.1	172.2
Burundi	Kirundo	Ntega	40643	2010	118.9	111.7	126.4
Burundi	Kirundo	Ntega	40643	2015	112.8	103.1	122.9
Burundi	Kirundo	Vumbi	40644	2000	231.1	218.2	241.9
Burundi	Kirundo	Vumbi	40644	2005	174.0	164.6	182.7
Burundi	Kirundo	Vumbi	40644	2010	128.2	120.5	136.5
Burundi	Kirundo	Vumbi	40644	2015	118.7	107.9	129.5
Burundi	Makamba	Kayogoro	40646	2000	111.8	103.4	120.5
Burundi	Makamba	Kayogoro	40646	2005	98.8	91.8	107.1
Burundi	Makamba	Kayogoro	40646	2010	78.2	72.0	84.7
Burundi	Makamba	Kayogoro	40646	2015	71.0	63.2	79.1
Burundi	Makamba	Kibago	40647	2000	111.8	103.4	121.0
Burundi	Makamba	Kibago	40647	2005	100.1	92.8	108.9
Burundi	Makamba	Kibago	40647	2010	77.9	71.6	85.0
Burundi	Makamba	Kibago	40647	2015	69.9	62.1	78.4
Burundi	Makamba	Mabanda	40648	2000	116.3	107.3	125.9
Burundi	Makamba	Mabanda	40648	2005	101.5	93.9	110.3
Burundi	Makamba	Mabanda	40648	2010	78.6	72.3	86.0
Burundi	Makamba	Mabanda	40648	2015	66.0	58.7	74.1
Burundi	Makamba	Makamba	40649	2000	114.8	105.7	124.5
Burundi	Makamba	Makamba	40649	2005	99.0	91.5	107.2
Burundi	Makamba	Makamba	40649	2010	76.9	70.6	83.9
Burundi	Makamba	Makamba	40649	2015	64.3	57.3	72.3
Burundi	Makamba	Nyanza-Lac	40650	2000	122.8	113.2	133.3
Burundi	Makamba	Nyanza-Lac	40650	2005	103.2	95.5	112.3
Burundi	Makamba	Nyanza-Lac	40650	2010	81.8	74.5	89.3
Burundi	Makamba	Nyanza-Lac	40650	2015	70.1	62.0	78.5
Burundi	Makamba	Vugizo	40651	2000	123.5	113.9	133.7
Burundi	Makamba	Vugizo	40651	2005	106.3	98.5	114.6
Burundi	Makamba	Vugizo	40651	2010	81.3	74.5	88.4
Burundi	Makamba	Vugizo	40651	2015	66.1	58.8	73.9
Burundi	Muramvya	Bukeye	40652	2000	148.6	140.2	157.3
Burundi	Muramvya	Bukeye	40652	2005	114.9	109.0	121.5
Burundi	Muramvya	Bukeye	40652	2010	87.1	81.6	92.6
Burundi	Muramvya	Bukeye	40652	2015	70.3	64.4	76.9
Burundi	Muramvya	Kiganda	40653	2000	136.5	128.5	144.6
Burundi	Muramvya	Kiganda	40653	2005	107.3	101.0	113.8
Burundi	Muramvya	Kiganda	40653	2010	83.9	78.8	89.5

Admin 0	Admin 1	Admin 2	GAUL Code	Year	Under-5 mortality (per 1,000 livebirths)		
					Estimate	Lower bound, 95% UI	Upper bound, 95% UI
Burundi	Muramvya	Kiganda	40653	2015	67.6	61.4	74.1
Burundi	Muramvya	Mbuye	40654	2000	147.1	139.1	156.1
Burundi	Muramvya	Mbuye	40654	2005	115.4	109.3	122.1
Burundi	Muramvya	Mbuye	40654	2010	88.8	83.4	94.4
Burundi	Muramvya	Mbuye	40654	2015	72.4	66.2	79.1
Burundi	Muramvya	Muramvya	40655	2000	140.8	133.4	148.4
Burundi	Muramvya	Muramvya	40655	2005	110.2	104.2	116.6
Burundi	Muramvya	Muramvya	40655	2010	84.9	79.5	90.2
Burundi	Muramvya	Muramvya	40655	2015	68.7	62.7	75.1
Burundi	Muramvya	Rutegama	40656	2000	140.9	132.9	149.7
Burundi	Muramvya	Rutegama	40656	2005	110.1	103.6	116.8
Burundi	Muramvya	Rutegama	40656	2010	85.7	80.4	91.5
Burundi	Muramvya	Rutegama	40656	2015	69.7	63.6	76.3
Burundi	Muyinga	Buhinyuza	40657	2000	175.7	164.5	187.6
Burundi	Muyinga	Buhinyuza	40657	2005	139.1	130.5	147.9
Burundi	Muyinga	Buhinyuza	40657	2010	110.0	102.4	118.0
Burundi	Muyinga	Buhinyuza	40657	2015	95.3	86.9	104.9
Burundi	Muyinga	Butihinda	40658	2000	222.0	208.8	235.5
Burundi	Muyinga	Butihinda	40658	2005	170.2	159.4	180.2
Burundi	Muyinga	Butihinda	40658	2010	127.9	119.5	136.2
Burundi	Muyinga	Butihinda	40658	2015	114.3	104.0	125.8
Burundi	Muyinga	Gashoho	40659	2000	226.9	214.1	239.0
Burundi	Muyinga	Gashoho	40659	2005	175.2	165.1	185.4
Burundi	Muyinga	Gashoho	40659	2010	130.2	122.0	139.1
Burundi	Muyinga	Gashoho	40659	2015	117.7	106.7	129.3
Burundi	Muyinga	Gasorwe	40660	2000	204.6	193.3	216.0
Burundi	Muyinga	Gasorwe	40660	2005	157.5	148.6	166.0
Burundi	Muyinga	Gasorwe	40660	2010	121.3	113.6	129.1
Burundi	Muyinga	Gasorwe	40660	2015	105.7	96.1	116.0
Burundi	Muyinga	Giteranyi	40661	2000	235.4	221.9	250.1
Burundi	Muyinga	Giteranyi	40661	2005	170.0	159.3	181.2
Burundi	Muyinga	Giteranyi	40661	2010	121.8	113.2	131.1
Burundi	Muyinga	Giteranyi	40661	2015	107.9	97.7	119.1
Burundi	Muyinga	Muyinga	40662	2000	198.8	187.4	210.6
Burundi	Muyinga	Muyinga	40662	2005	153.1	144.1	161.5
Burundi	Muyinga	Muyinga	40662	2010	117.7	110.2	125.4
Burundi	Muyinga	Muyinga	40662	2015	101.5	92.3	111.2
Burundi	Muyinga	Mwakiro	40663	2000	163.8	152.7	176.0
Burundi	Muyinga	Mwakiro	40663	2005	132.0	123.4	141.3
Burundi	Muyinga	Mwakiro	40663	2010	104.6	96.8	112.3
Burundi	Muyinga	Mwakiro	40663	2015	93.0	84.2	102.4
Burundi	Mwaro	Bisoro	40664	2000	125.8	117.8	134.2
Burundi	Mwaro	Bisoro	40664	2005	102.3	96.3	109.1
Burundi	Mwaro	Bisoro	40664	2010	81.6	76.5	87.6
Burundi	Mwaro	Bisoro	40664	2015	66.7	60.5	73.4
Burundi	Mwaro	Gisozi	40665	2000	124.9	117.5	132.8
Burundi	Mwaro	Gisozi	40665	2005	103.6	97.6	110.1
Burundi	Mwaro	Gisozi	40665	2010	81.0	76.0	86.6
Burundi	Mwaro	Gisozi	40665	2015	64.5	58.7	70.8
Burundi	Mwaro	Kayokwe	40666	2000	129.2	121.7	137.5
Burundi	Mwaro	Kayokwe	40666	2005	104.0	98.2	110.5
Burundi	Mwaro	Kayokwe	40666	2010	82.8	77.7	88.2
Burundi	Mwaro	Kayokwe	40666	2015	66.7	60.8	73.1
Burundi	Mwaro	Ndava	40667	2000	134.8	127.0	143.0
Burundi	Mwaro	Ndava	40667	2005	106.8	100.7	113.0
Burundi	Mwaro	Ndava	40667	2010	85.2	80.1	91.0
Burundi	Mwaro	Ndava	40667	2015	68.6	62.7	75.2
Burundi	Mwaro	Nyabihanga	40668	2000	133.4	125.1	142.0
Burundi	Mwaro	Nyabihanga	40668	2005	106.8	100.7	113.7
Burundi	Mwaro	Nyabihanga	40668	2010	85.1	79.7	91.1
Burundi	Mwaro	Nyabihanga	40668	2015	68.5	62.3	75.1
Burundi	Mwaro	Rusaka	40669	2000	129.0	122.0	136.6
Burundi	Mwaro	Rusaka	40669	2005	104.4	98.6	110.3
Burundi	Mwaro	Rusaka	40669	2010	82.2	77.4	87.5
Burundi	Mwaro	Rusaka	40669	2015	65.9	60.2	72.2
Burundi	Ngozi	Busiga	40670	2000	178.0	169.6	186.5
Burundi	Ngozi	Busiga	40670	2005	131.3	124.9	138.0
Burundi	Ngozi	Busiga	40670	2010	97.6	91.8	103.9
Burundi	Ngozi	Busiga	40670	2015	77.3	71.2	84.1
Burundi	Ngozi	Gashikanwa	40671	2000	202.5	190.0	215.1
Burundi	Ngozi	Gashikanwa	40671	2005	153.8	145.1	163.9
Burundi	Ngozi	Gashikanwa	40671	2010	115.9	108.1	123.8
Burundi	Ngozi	Gashikanwa	40671	2015	99.6	90.5	109.0
Burundi	Ngozi	Kiremba	40672	2000	214.0	200.5	226.9
Burundi	Ngozi	Kiremba	40672	2005	163.8	154.4	174.4
Burundi	Ngozi	Kiremba	40672	2010	124.0	115.4	132.6
Burundi	Ngozi	Kiremba	40672	2015	108.3	98.2	118.6
Burundi	Ngozi	Marangara	40673	2000	220.2	207.3	231.9
Burundi	Ngozi	Marangara	40673	2005	167.4	158.5	177.5
Burundi	Ngozi	Marangara	40673	2010	123.0	115.4	131.1
Burundi	Ngozi	Marangara	40673	2015	108.7	99.3	118.8
Burundi	Ngozi	Mwumba	40674	2000	189.4	180.1	199.1
Burundi	Ngozi	Mwumba	40674	2005	141.3	134.5	149.1
Burundi	Ngozi	Mwumba	40674	2010	105.5	99.4	112.1
Burundi	Ngozi	Mwumba	40674	2015	88.9	81.5	97.0

Admin 0	Admin 1	Admin 2	GAUL Code	Year	Under-5 mortality (per 1,000 livebirths)		
					Estimate	Lower bound, 95% UI	Upper bound, 95% UI
Burundi	Ngozi	Ngozi	40675	2000	183.1	173.9	192.4
Burundi	Ngozi	Ngozi	40675	2005	140.1	133.3	147.5
Burundi	Ngozi	Ngozi	40675	2010	105.3	99.1	111.8
Burundi	Ngozi	Ngozi	40675	2015	87.7	80.3	95.4
Burundi	Ngozi	Nyamurenza	40676	2000	206.4	194.7	218.5
Burundi	Ngozi	Nyamurenza	40676	2005	154.6	146.1	164.1
Burundi	Ngozi	Nyamurenza	40676	2010	115.6	108.3	123.6
Burundi	Ngozi	Nyamurenza	40676	2015	98.8	90.1	108.4
Burundi	Ngozi	Ruhororo	40677	2000	190.4	180.0	201.3
Burundi	Ngozi	Ruhororo	40677	2005	148.5	140.7	157.2
Burundi	Ngozi	Ruhororo	40677	2010	112.9	106.2	120.1
Burundi	Ngozi	Ruhororo	40677	2015	99.3	90.5	108.1
Burundi	Ngozi	Tangara	40678	2000	198.5	187.7	210.1
Burundi	Ngozi	Tangara	40678	2005	152.7	144.4	161.2
Burundi	Ngozi	Tangara	40678	2010	118.4	110.8	126.0
Burundi	Ngozi	Tangara	40678	2015	104.0	94.7	113.7
Burundi	Rutana	Bukemba	40679	2000	113.2	105.0	122.1
Burundi	Rutana	Bukemba	40679	2005	99.4	92.5	107.3
Burundi	Rutana	Bukemba	40679	2010	79.9	73.7	86.4
Burundi	Rutana	Bukemba	40679	2015	70.9	63.3	78.8
Burundi	Rutana	Giharo	40680	2000	123.9	115.2	133.4
Burundi	Rutana	Giharo	40680	2005	108.1	100.5	116.1
Burundi	Rutana	Giharo	40680	2010	87.8	81.3	94.8
Burundi	Rutana	Giharo	40680	2015	80.2	72.1	88.8
Burundi	Rutana	Gitanga	40681	2000	121.4	112.9	130.6
Burundi	Rutana	Gitanga	40681	2005	106.0	98.9	113.7
Burundi	Rutana	Gitanga	40681	2010	80.6	74.5	87.0
Burundi	Rutana	Gitanga	40681	2015	68.4	61.5	75.9
Burundi	Rutana	Mpinga-Kayove	40682	2000	127.7	118.7	137.6
Burundi	Rutana	Mpinga-Kayove	40682	2005	110.4	102.4	118.8
Burundi	Rutana	Mpinga-Kayove	40682	2010	86.8	80.4	93.7
Burundi	Rutana	Mpinga-Kayove	40682	2015	77.6	69.6	85.8
Burundi	Rutana	Musongati	40683	2000	130.8	121.8	140.8
Burundi	Rutana	Musongati	40683	2005	112.3	104.4	120.6
Burundi	Rutana	Musongati	40683	2010	88.1	81.7	94.9
Burundi	Rutana	Musongati	40683	2015	78.1	70.0	86.2
Burundi	Rutana	Rutana	40684	2000	124.6	115.5	134.0
Burundi	Rutana	Rutana	40684	2005	107.1	99.4	115.1
Burundi	Rutana	Rutana	40684	2010	83.5	77.1	90.2
Burundi	Rutana	Rutana	40684	2015	71.7	64.2	79.6
Burundi	Ruyigi	Butaganzwa1	40685	2000	140.5	131.7	149.9
Burundi	Ruyigi	Butaganzwa1	40685	2005	117.8	110.0	125.4
Burundi	Ruyigi	Butaganzwa1	40685	2010	93.0	86.6	99.7
Burundi	Ruyigi	Butaganzwa1	40685	2015	83.4	75.2	91.8
Burundi	Ruyigi	Butezi	40686	2000	147.5	138.5	156.8
Burundi	Ruyigi	Butezi	40686	2005	120.7	113.7	127.8
Burundi	Ruyigi	Butezi	40686	2010	95.8	89.4	102.2
Burundi	Ruyigi	Butezi	40686	2015	84.1	76.1	92.5
Burundi	Ruyigi	Bweru	40687	2000	145.6	135.7	155.4
Burundi	Ruyigi	Bweru	40687	2005	121.6	114.0	129.5
Burundi	Ruyigi	Bweru	40687	2010	95.9	89.1	103.0
Burundi	Ruyigi	Bweru	40687	2015	83.2	75.5	91.5
Burundi	Ruyigi	Gisuru	40688	2000	132.6	123.8	142.5
Burundi	Ruyigi	Gisuru	40688	2005	110.8	103.7	119.2
Burundi	Ruyigi	Gisuru	40688	2010	90.1	83.4	97.1
Burundi	Ruyigi	Gisuru	40688	2015	80.2	72.5	88.8
Burundi	Ruyigi	Kinyinya	40689	2000	131.5	121.3	142.9
Burundi	Ruyigi	Kinyinya	40689	2005	113.0	104.5	122.3
Burundi	Ruyigi	Kinyinya	40689	2010	92.4	84.7	100.3
Burundi	Ruyigi	Kinyinya	40689	2015	83.6	74.7	92.6
Burundi	Ruyigi	Nyabitsinda	40690	2000	137.7	127.7	148.3
Burundi	Ruyigi	Nyabitsinda	40690	2005	117.6	109.2	126.5
Burundi	Ruyigi	Nyabitsinda	40690	2010	94.0	86.7	101.5
Burundi	Ruyigi	Nyabitsinda	40690	2015	86.3	77.7	95.3
Burundi	Ruyigi	Ruyigi	40691	2000	143.7	134.8	152.8
Burundi	Ruyigi	Ruyigi	40691	2005	121.2	114.1	128.8
Burundi	Ruyigi	Ruyigi	40691	2010	94.3	87.7	100.9
Burundi	Ruyigi	Ruyigi	40691	2015	82.0	74.6	90.3
Cameroon	Adamaoua	Djerem	12462	2000	159.1	141.5	178.7
Cameroon	Adamaoua	Djerem	12462	2005	130.8	116.0	148.2
Cameroon	Adamaoua	Djerem	12462	2010	117.9	102.5	135.5
Cameroon	Adamaoua	Djerem	12462	2015	107.5	91.7	125.2
Cameroon	Adamaoua	Faro - Et - Déo	12463	2000	174.2	155.2	195.5
Cameroon	Adamaoua	Faro - Et - Déo	12463	2005	147.3	130.5	165.3
Cameroon	Adamaoua	Faro - Et - Déo	12463	2010	120.3	105.0	137.6
Cameroon	Adamaoua	Faro - Et - Déo	12463	2015	111.9	95.3	131.0
Cameroon	Adamaoua	Mayo-Banyo	12464	2000	174.1	158.0	192.0
Cameroon	Adamaoua	Mayo-Banyo	12464	2005	150.0	135.7	165.1
Cameroon	Adamaoua	Mayo-Banyo	12464	2010	131.1	116.4	148.1
Cameroon	Adamaoua	Mayo-Banyo	12464	2015	117.0	101.0	134.0
Cameroon	Adamaoua	Mbéré	12465	2000	169.3	150.5	189.3
Cameroon	Adamaoua	Mbéré	12465	2005	146.8	130.3	164.6
Cameroon	Adamaoua	Mbéré	12465	2010	130.7	114.6	149.0
Cameroon	Adamaoua	Mbéré	12465	2015	119.6	101.7	139.2
Cameroon	Adamaoua	Vina	12466	2000	137.8	122.9	153.5

Admin 0	Admin 1	Admin 2	GAUL Code	Year	Under-5 mortality (per 1,000 livebirths)		
					Estimate	Lower bound, 95% UI	Upper bound, 95% UI
Cameroon	Adamaoua	Vina	12466	2005	115.3	102.1	129.4
Cameroon	Adamaoua	Vina	12466	2010	99.5	87.2	114.0
Cameroon	Adamaoua	Vina	12466	2015	90.5	77.6	105.5
Cameroon	Centre	Haute - Sanaga	12467	2000	134.3	119.2	150.5
Cameroon	Centre	Haute - Sanaga	12467	2005	119.6	106.0	135.1
Cameroon	Centre	Haute - Sanaga	12467	2010	108.5	94.7	124.8
Cameroon	Centre	Haute - Sanaga	12467	2015	92.6	78.8	108.8
Cameroon	Centre	Lékié	12468	2000	130.8	119.7	141.5
Cameroon	Centre	Lékié	12468	2005	111.4	102.4	121.2
Cameroon	Centre	Lékié	12468	2010	99.2	88.7	110.4
Cameroon	Centre	Lékié	12468	2015	82.8	71.7	95.1
Cameroon	Centre	Mbam - Et - Inoubou	12469	2000	122.8	110.3	136.0
Cameroon	Centre	Mbam - Et - Inoubou	12469	2005	109.4	98.9	120.0
Cameroon	Centre	Mbam - Et - Inoubou	12469	2010	100.4	88.4	112.7
Cameroon	Centre	Mbam - Et - Inoubou	12469	2015	83.4	71.3	96.5
Cameroon	Centre	Mbam - Et - Kim	12470	2000	143.4	127.8	159.2
Cameroon	Centre	Mbam - Et - Kim	12470	2005	124.2	110.9	138.3
Cameroon	Centre	Mbam - Et - Kim	12470	2010	114.9	101.4	129.6
Cameroon	Centre	Mbam - Et - Kim	12470	2015	99.5	85.9	114.7
Cameroon	Centre	Mfoundi	12473	2000	103.2	95.0	112.7
Cameroon	Centre	Mfoundi	12473	2005	91.1	83.5	99.4
Cameroon	Centre	Mfoundi	12473	2010	81.3	72.5	90.8
Cameroon	Centre	Mfoundi	12473	2015	64.6	55.2	74.8
Cameroon	Centre	Méfou - Et - Afamba	12471	2000	117.7	108.4	127.1
Cameroon	Centre	Méfou - Et - Afamba	12471	2005	103.4	95.5	111.7
Cameroon	Centre	Méfou - Et - Afamba	12471	2010	91.3	81.8	101.4
Cameroon	Centre	Méfou - Et - Afamba	12471	2015	74.9	64.9	86.4
Cameroon	Centre	Méfou - Et - Akono	12472	2000	119.8	110.2	129.6
Cameroon	Centre	Méfou - Et - Akono	12472	2005	105.4	96.3	114.3
Cameroon	Centre	Méfou - Et - Akono	12472	2010	92.9	82.9	103.7
Cameroon	Centre	Méfou - Et - Akono	12472	2015	76.3	66.1	88.6
Cameroon	Centre	Nyong - Et - Kéllé	12474	2000	130.9	117.1	145.8
Cameroon	Centre	Nyong - Et - Kéllé	12474	2005	115.2	102.3	127.7
Cameroon	Centre	Nyong - Et - Kéllé	12474	2010	101.6	89.4	115.2
Cameroon	Centre	Nyong - Et - Kéllé	12474	2015	83.9	72.1	97.0
Cameroon	Centre	Nyong - Et - Mfoumou	12475	2000	126.3	113.7	141.1
Cameroon	Centre	Nyong - Et - Mfoumou	12475	2005	114.1	101.7	127.9
Cameroon	Centre	Nyong - Et - Mfoumou	12475	2010	101.1	87.8	115.3
Cameroon	Centre	Nyong - Et - Mfoumou	12475	2015	85.9	73.4	100.6
Cameroon	Centre	Nyong - Et - So'o	12476	2000	127.3	115.4	139.6
Cameroon	Centre	Nyong - Et - So'o	12476	2005	112.7	101.9	123.3
Cameroon	Centre	Nyong - Et - So'o	12476	2010	98.8	87.3	111.2
Cameroon	Centre	Nyong - Et - So'o	12476	2015	84.0	72.1	96.8
Cameroon	Est	Boumba - Et - Ngoko	12477	2000	160.1	141.1	179.5
Cameroon	Est	Boumba - Et - Ngoko	12477	2005	140.3	124.1	156.9
Cameroon	Est	Boumba - Et - Ngoko	12477	2010	125.2	108.5	143.0
Cameroon	Est	Boumba - Et - Ngoko	12477	2015	108.6	92.7	125.9
Cameroon	Est	Haut - Nyong	12478	2000	146.8	132.0	162.2
Cameroon	Est	Haut - Nyong	12478	2005	131.8	118.9	146.3
Cameroon	Est	Haut - Nyong	12478	2010	115.4	101.9	129.0
Cameroon	Est	Haut - Nyong	12478	2015	99.9	86.1	114.1
Cameroon	Est	Kadeï	12479	2000	163.3	146.6	183.9
Cameroon	Est	Kadeï	12479	2005	140.8	126.8	158.5
Cameroon	Est	Kadeï	12479	2010	118.8	104.5	136.5
Cameroon	Est	Kadeï	12479	2015	103.9	88.9	122.4
Cameroon	Est	Lom - Et - Djerem	12480	2000	139.6	125.5	155.2
Cameroon	Est	Lom - Et - Djerem	12480	2005	121.5	109.3	134.8
Cameroon	Est	Lom - Et - Djerem	12480	2010	109.6	96.6	123.1
Cameroon	Est	Lom - Et - Djerem	12480	2015	95.1	80.8	109.5
Cameroon	Extrême - Nord	Diamaré	12481	2000	170.9	158.3	183.4
Cameroon	Extrême - Nord	Diamaré	12481	2005	157.7	146.1	169.8
Cameroon	Extrême - Nord	Diamaré	12481	2010	147.8	133.5	162.3
Cameroon	Extrême - Nord	Diamaré	12481	2015	139.7	122.6	156.9
Cameroon	Extrême - Nord	Logone - Et - Chari	12482	2000	187.3	176.1	199.5
Cameroon	Extrême - Nord	Logone - Et - Chari	12482	2005	169.2	158.9	178.9
Cameroon	Extrême - Nord	Logone - Et - Chari	12482	2010	144.8	134.4	155.1
Cameroon	Extrême - Nord	Logone - Et - Chari	12482	2015	143.5	130.4	158.2
Cameroon	Extrême - Nord	Mayo-Danay	12483	2000	147.5	136.7	158.8
Cameroon	Extrême - Nord	Mayo-Danay	12483	2005	145.3	135.1	156.1
Cameroon	Extrême - Nord	Mayo-Danay	12483	2010	146.6	134.8	158.7
Cameroon	Extrême - Nord	Mayo-Danay	12483	2015	138.0	123.4	154.1
Cameroon	Extrême - Nord	Mayo-Kani	12484	2000	172.5	160.1	184.5
Cameroon	Extrême - Nord	Mayo-Kani	12484	2005	161.5	150.0	174.3
Cameroon	Extrême - Nord	Mayo-Kani	12484	2010	158.7	145.6	174.1
Cameroon	Extrême - Nord	Mayo-Kani	12484	2015	146.2	130.3	164.2
Cameroon	Extrême - Nord	Mayo-Sava	12485	2000	206.2	189.6	224.6
Cameroon	Extrême - Nord	Mayo-Sava	12485	2005	180.4	165.6	196.9
Cameroon	Extrême - Nord	Mayo-Sava	12485	2010	165.9	149.6	182.6
Cameroon	Extrême - Nord	Mayo-Sava	12485	2015	155.3	136.0	176.5
Cameroon	Extrême - Nord	Mayo-Tsanaga	12486	2000	201.6	187.8	215.4
Cameroon	Extrême - Nord	Mayo-Tsanaga	12486	2005	181.6	170.1	194.5
Cameroon	Extrême - Nord	Mayo-Tsanaga	12486	2010	161.5	148.5	176.1
Cameroon	Extrême - Nord	Mayo-Tsanaga	12486	2015	141.2	126.5	158.3
Cameroon	Littoral	Moungo	12487	2000	101.4	93.6	109.6
Cameroon	Littoral	Moungo	12487	2005	95.2	87.4	103.4

Admin 0	Admin 1	Admin 2	GAUL Code	Year	Under-5 mortality (per 1,000 livebirths)		
					Estimate	Lower bound, 95% UI	Upper bound, 95% UI
Cameroon	Littoral	Moungo	12487	2010	78.9	71.0	87.3
Cameroon	Littoral	Moungo	12487	2015	60.9	53.2	69.5
Cameroon	Littoral	Nkam	12488	2000	111.9	101.6	123.5
Cameroon	Littoral	Nkam	12488	2005	97.8	89.6	108.4
Cameroon	Littoral	Nkam	12488	2010	82.1	73.2	91.9
Cameroon	Littoral	Nkam	12488	2015	64.6	56.1	74.6
Cameroon	Littoral	Sanaga - Maritime	12489	2000	115.8	103.0	129.1
Cameroon	Littoral	Sanaga - Maritime	12489	2005	102.1	90.4	114.4
Cameroon	Littoral	Sanaga - Maritime	12489	2010	88.8	78.0	101.3
Cameroon	Littoral	Sanaga - Maritime	12489	2015	68.7	58.8	80.4
Cameroon	Littoral	Wouril	12490	2000	92.7	84.6	101.8
Cameroon	Littoral	Wouril	12490	2005	84.5	77.1	92.8
Cameroon	Littoral	Wouril	12490	2010	72.1	64.7	81.0
Cameroon	Littoral	Wouril	12490	2015	55.0	47.8	63.5
Cameroon	Nord	Bénoue	12498	2000	201.8	186.5	218.0
Cameroon	Nord	Bénoue	12498	2005	176.1	161.8	190.7
Cameroon	Nord	Bénoue	12498	2010	137.9	125.6	151.9
Cameroon	Nord	Bénoue	12498	2015	128.9	114.2	143.8
Cameroon	Nord	Faro	12499	2000	202.5	184.5	221.6
Cameroon	Nord	Faro	12499	2005	175.1	159.3	192.0
Cameroon	Nord	Faro	12499	2010	141.5	127.4	158.5
Cameroon	Nord	Faro	12499	2015	132.1	116.5	151.1
Cameroon	Nord	Mayo - Rey	12501	2000	219.0	201.9	235.8
Cameroon	Nord	Mayo - Rey	12501	2005	206.8	191.5	223.8
Cameroon	Nord	Mayo - Rey	12501	2010	178.7	163.6	196.2
Cameroon	Nord	Mayo - Rey	12501	2015	162.2	144.5	182.7
Cameroon	Nord	Mayo-Louti	12500	2000	223.5	208.6	240.0
Cameroon	Nord	Mayo-Louti	12500	2005	205.5	191.6	220.5
Cameroon	Nord	Mayo-Louti	12500	2010	177.3	162.6	193.2
Cameroon	Nord	Mayo-Louti	12500	2015	158.6	141.2	178.9
Cameroon	Nord - Ouest	Boyo	12491	2000	98.6	89.8	107.9
Cameroon	Nord - Ouest	Boyo	12491	2005	99.5	90.8	108.9
Cameroon	Nord - Ouest	Boyo	12491	2010	81.0	72.5	90.0
Cameroon	Nord - Ouest	Boyo	12491	2015	61.8	54.3	70.2
Cameroon	Nord - Ouest	Bui	12492	2000	98.6	90.6	107.2
Cameroon	Nord - Ouest	Bui	12492	2005	99.5	90.8	108.8
Cameroon	Nord - Ouest	Bui	12492	2010	83.7	74.8	92.9
Cameroon	Nord - Ouest	Bui	12492	2015	62.9	54.9	72.2
Cameroon	Nord - Ouest	Donga - Mantung	12493	2000	123.3	112.5	135.0
Cameroon	Nord - Ouest	Donga - Mantung	12493	2005	117.5	107.2	128.6
Cameroon	Nord - Ouest	Donga - Mantung	12493	2010	99.0	88.7	110.1
Cameroon	Nord - Ouest	Donga - Mantung	12493	2015	84.0	73.7	95.3
Cameroon	Nord - Ouest	Menchum	12494	2000	111.7	101.1	123.4
Cameroon	Nord - Ouest	Menchum	12494	2005	108.9	98.7	119.9
Cameroon	Nord - Ouest	Menchum	12494	2010	86.8	77.3	97.5
Cameroon	Nord - Ouest	Menchum	12494	2015	69.0	60.5	79.4
Cameroon	Nord - Ouest	Mezam	12495	2000	84.8	77.9	92.2
Cameroon	Nord - Ouest	Mezam	12495	2005	88.9	81.2	96.8
Cameroon	Nord - Ouest	Mezam	12495	2010	75.7	68.0	84.0
Cameroon	Nord - Ouest	Mezam	12495	2015	55.4	47.9	63.4
Cameroon	Nord - Ouest	Momo	12496	2000	95.2	86.5	105.5
Cameroon	Nord - Ouest	Momo	12496	2005	98.1	89.4	108.4
Cameroon	Nord - Ouest	Momo	12496	2010	81.5	72.8	91.3
Cameroon	Nord - Ouest	Momo	12496	2015	59.4	51.1	68.1
Cameroon	Nord - Ouest	Ngo-Ketunjia	12497	2000	95.5	87.7	104.1
Cameroon	Nord - Ouest	Ngo-Ketunjia	12497	2005	95.9	88.4	104.8
Cameroon	Nord - Ouest	Ngo-Ketunjia	12497	2010	80.3	71.5	89.9
Cameroon	Nord - Ouest	Ngo-Ketunjia	12497	2015	59.4	51.8	68.3
Cameroon	Ouest	Bamboutos	12502	2000	100.8	93.3	108.1
Cameroon	Ouest	Bamboutos	12502	2005	98.2	90.9	105.7
Cameroon	Ouest	Bamboutos	12502	2010	83.2	75.1	92.1
Cameroon	Ouest	Bamboutos	12502	2015	61.1	53.3	70.0
Cameroon	Ouest	Haut-Nkam	12503	2000	100.2	91.7	109.9
Cameroon	Ouest	Haut-Nkam	12503	2005	93.9	85.9	102.6
Cameroon	Ouest	Haut-Nkam	12503	2010	79.6	71.6	88.9
Cameroon	Ouest	Haut-Nkam	12503	2015	61.4	53.8	70.8
Cameroon	Ouest	Hauts-Plateaux	12504	2000	98.8	90.8	107.5
Cameroon	Ouest	Hauts-Plateaux	12504	2005	94.6	86.9	103.1
Cameroon	Ouest	Hauts-Plateaux	12504	2010	83.6	74.9	93.3
Cameroon	Ouest	Hauts-Plateaux	12504	2015	62.1	54.0	71.1
Cameroon	Ouest	Koung-Khi	12505	2000	100.8	92.5	110.1
Cameroon	Ouest	Koung-Khi	12505	2005	95.8	88.0	104.3
Cameroon	Ouest	Koung-Khi	12505	2010	86.7	77.6	96.9
Cameroon	Ouest	Koung-Khi	12505	2015	64.2	55.6	74.0
Cameroon	Ouest	Mifi	12507	2000	93.5	85.1	103.1
Cameroon	Ouest	Mifi	12507	2005	91.5	83.1	100.9
Cameroon	Ouest	Mifi	12507	2010	81.9	72.8	91.9
Cameroon	Ouest	Mifi	12507	2015	61.5	52.9	71.4
Cameroon	Ouest	Ménoua	12506	2000	100.3	92.3	108.6
Cameroon	Ouest	Ménoua	12506	2005	98.5	90.4	107.0
Cameroon	Ouest	Ménoua	12506	2010	82.4	73.9	91.4
Cameroon	Ouest	Ménoua	12506	2015	61.3	53.3	70.3
Cameroon	Ouest	Nde	12508	2000	106.2	95.2	118.6
Cameroon	Ouest	Nde	12508	2005	97.8	87.8	108.7
Cameroon	Ouest	Nde	12508	2010	89.8	79.0	101.5

Admin 0	Admin 1	Admin 2	GAUL Code	Year	Under-5 mortality (per 1,000 livebirths)		
					Estimate	Lower bound, 95% UI	Upper bound, 95% UI
Cameroon	Ouest	Nde	12508	2015	69.8	60.0	81.7
Cameroon	Ouest	Noun	12509	2000	131.3	120.6	141.9
Cameroon	Ouest	Noun	12509	2005	116.7	107.8	126.7
Cameroon	Ouest	Noun	12509	2010	105.9	94.8	117.2
Cameroon	Ouest	Noun	12509	2015	84.3	73.6	96.2
Cameroon	Sud	Dja - Et - Lobo	12516	2000	136.5	121.6	151.6
Cameroon	Sud	Dja - Et - Lobo	12516	2005	123.0	109.3	136.9
Cameroon	Sud	Dja - Et - Lobo	12516	2010	110.5	97.1	125.5
Cameroon	Sud	Dja - Et - Lobo	12516	2015	94.0	80.7	108.3
Cameroon	Sud	Mvila	12517	2000	134.5	120.5	150.3
Cameroon	Sud	Mvila	12517	2005	118.4	105.9	132.6
Cameroon	Sud	Mvila	12517	2010	104.0	91.3	118.6
Cameroon	Sud	Mvila	12517	2015	84.6	72.4	97.6
Cameroon	Sud	Océan	12518	2000	139.3	122.3	157.6
Cameroon	Sud	Océan	12518	2005	124.5	109.3	141.5
Cameroon	Sud	Océan	12518	2010	106.4	93.0	122.0
Cameroon	Sud	Océan	12518	2015	83.9	72.3	99.0
Cameroon	Sud	Vallée - Du - Ntem	12519	2000	148.2	131.3	166.7
Cameroon	Sud	Vallée - Du - Ntem	12519	2005	128.6	113.3	144.8
Cameroon	Sud	Vallée - Du - Ntem	12519	2010	114.8	100.0	131.3
Cameroon	Sud	Vallée - Du - Ntem	12519	2015	91.3	77.7	107.5
Cameroon	Sud - Ouest	Fako	12510	2000	107.2	97.2	117.4
Cameroon	Sud - Ouest	Fako	12510	2005	100.1	90.6	110.9
Cameroon	Sud - Ouest	Fako	12510	2010	84.3	74.7	95.3
Cameroon	Sud - Ouest	Fako	12510	2015	63.8	54.9	73.3
Cameroon	Sud - Ouest	Kupé Manenguba	12511	2000	111.8	102.2	121.7
Cameroon	Sud - Ouest	Kupé Manenguba	12511	2005	109.4	100.3	119.3
Cameroon	Sud - Ouest	Kupé Manenguba	12511	2010	90.2	80.8	100.4
Cameroon	Sud - Ouest	Kupé Manenguba	12511	2015	66.9	57.9	75.9
Cameroon	Sud - Ouest	Lebialem	12512	2000	94.7	86.7	103.7
Cameroon	Sud - Ouest	Lebialem	12512	2005	96.7	88.5	105.8
Cameroon	Sud - Ouest	Lebialem	12512	2010	80.1	71.5	89.4
Cameroon	Sud - Ouest	Lebialem	12512	2015	58.6	50.3	67.2
Cameroon	Sud - Ouest	Manyu	12513	2000	116.4	106.2	127.3
Cameroon	Sud - Ouest	Manyu	12513	2005	111.3	101.5	121.4
Cameroon	Sud - Ouest	Manyu	12513	2010	93.3	84.2	103.6
Cameroon	Sud - Ouest	Manyu	12513	2015	70.1	61.2	79.6
Cameroon	Sud - Ouest	Meme	12514	2000	111.4	101.8	121.6
Cameroon	Sud - Ouest	Meme	12514	2005	106.1	97.0	116.8
Cameroon	Sud - Ouest	Meme	12514	2010	87.3	77.9	97.2
Cameroon	Sud - Ouest	Meme	12514	2015	66.8	58.0	76.5
Cameroon	Sud - Ouest	Ndian	12515	2000	135.1	121.9	148.7
Cameroon	Sud - Ouest	Ndian	12515	2005	128.0	116.0	140.3
Cameroon	Sud - Ouest	Ndian	12515	2010	104.8	93.5	116.8
Cameroon	Sud - Ouest	Ndian	12515	2015	81.7	71.4	93.4
Central African Republic	Bamingui-Bangoran	Bamingui	12835	2000	183.6	152.3	217.7
Central African Republic	Bamingui-Bangoran	Bamingui	12835	2005	174.9	144.7	207.6
Central African Republic	Bamingui-Bangoran	Bamingui	12835	2010	151.5	125.5	180.7
Central African Republic	Bamingui-Bangoran	Bamingui	12835	2015	140.3	116.0	167.8
Central African Republic	Bamingui-Bangoran	Ndélé	12836	2000	184.9	157.4	214.9
Central African Republic	Bamingui-Bangoran	Ndélé	12836	2005	167.7	141.0	195.5
Central African Republic	Bamingui-Bangoran	Ndélé	12836	2010	145.5	121.4	170.0
Central African Republic	Bamingui-Bangoran	Ndélé	12836	2015	134.9	112.9	159.4
Central African Republic	Bangui	Bangui	67155	2000	138.6	118.6	161.1
Central African Republic	Bangui	Bangui	67155	2005	129.8	111.5	150.6
Central African Republic	Bangui	Bangui	67155	2010	130.5	111.1	152.6
Central African Republic	Bangui	Bangui	67155	2015	109.5	91.7	132.3
Central African Republic	Basse-Kotto	Alindao	12837	2000	183.4	153.3	215.4
Central African Republic	Basse-Kotto	Alindao	12837	2005	179.6	148.0	214.3
Central African Republic	Basse-Kotto	Alindao	12837	2010	151.6	125.8	182.2
Central African Republic	Basse-Kotto	Alindao	12837	2015	138.5	112.2	169.6
Central African Republic	Basse-Kotto	Kembé	12838	2000	175.1	147.8	206.4
Central African Republic	Basse-Kotto	Kembé	12838	2005	169.4	143.4	200.5
Central African Republic	Basse-Kotto	Kembé	12838	2010	141.3	118.1	169.2
Central African Republic	Basse-Kotto	Kembé	12838	2015	126.5	103.5	153.0
Central African Republic	Basse-Kotto	Mingala	12839	2000	181.0	151.2	216.3
Central African Republic	Basse-Kotto	Mingala	12839	2005	178.5	147.9	215.3
Central African Republic	Basse-Kotto	Mingala	12839	2010	148.9	121.4	182.4
Central African Republic	Basse-Kotto	Mingala	12839	2015	137.7	110.4	172.5
Central African Republic	Basse-Kotto	Mobaye	12840	2000	178.3	148.7	210.4
Central African Republic	Basse-Kotto	Mobaye	12840	2005	173.0	143.5	204.4
Central African Republic	Basse-Kotto	Mobaye	12840	2010	146.6	121.9	176.4
Central African Republic	Basse-Kotto	Mobaye	12840	2015	131.2	107.6	158.9
Central African Republic	Basse-Kotto	Zangba	12841	2000	181.4	151.5	214.6
Central African Republic	Basse-Kotto	Zangba	12841	2005	178.3	150.2	210.6
Central African Republic	Basse-Kotto	Zangba	12841	2010	152.9	127.1	181.5
Central African Republic	Basse-Kotto	Zangba	12841	2015	135.7	111.2	163.4
Central African Republic	Haut-Mbomou	Bambouti	12842	2000	156.7	133.8	184.5
Central African Republic	Haut-Mbomou	Bambouti	12842	2005	143.1	121.1	166.9
Central African Republic	Haut-Mbomou	Bambouti	12842	2010	145.6	121.3	172.5
Central African Republic	Haut-Mbomou	Bambouti	12842	2015	131.7	110.0	158.6
Central African Republic	Haut-Mbomou	Djéma	12843	2000	149.3	125.1	176.6
Central African Republic	Haut-Mbomou	Djéma	12843	2005	136.2	114.9	160.8
Central African Republic	Haut-Mbomou	Djéma	12843	2010	137.3	115.0	162.8
Central African Republic	Haut-Mbomou	Djéma	12843	2015	128.6	106.7	155.3

Admin 0	Admin 1	Admin 2	GAUL Code	Year	Under-5 mortality (per 1,000 livebirths)		
					Estimate	Lower bound, 95% UI	Upper bound, 95% UI
Central African Republic	Haut-Mbomou	Obo	12844	2000	154.7	128.8	183.5
Central African Republic	Haut-Mbomou	Obo	12844	2005	141.2	118.2	167.9
Central African Republic	Haut-Mbomou	Obo	12844	2010	137.8	113.2	164.5
Central African Republic	Haut-Mbomou	Obo	12844	2015	124.9	102.7	150.5
Central African Republic	Haut-Mbomou	Zémio	12845	2000	145.3	117.1	177.8
Central African Republic	Haut-Mbomou	Zémio	12845	2005	137.7	111.8	168.6
Central African Republic	Haut-Mbomou	Zémio	12845	2010	128.4	103.4	158.3
Central African Republic	Haut-Mbomou	Zémio	12845	2015	121.2	98.8	146.5
Central African Republic	Haute-Kotto	Bria	12846	2000	165.7	133.4	203.1
Central African Republic	Haute-Kotto	Bria	12846	2005	166.2	133.9	202.8
Central African Republic	Haute-Kotto	Bria	12846	2010	137.2	109.2	168.0
Central African Republic	Haute-Kotto	Bria	12846	2015	131.2	103.3	162.7
Central African Republic	Haute-Kotto	Ouadda	12847	2000	156.4	126.6	187.9
Central African Republic	Haute-Kotto	Ouadda	12847	2005	153.6	123.4	185.5
Central African Republic	Haute-Kotto	Ouadda	12847	2010	133.1	105.6	161.5
Central African Republic	Haute-Kotto	Ouadda	12847	2015	132.7	104.6	162.9
Central African Republic	Haute-Kotto	Yalinga	12848	2000	145.9	120.4	174.2
Central African Republic	Haute-Kotto	Yalinga	12848	2005	144.3	118.0	173.6
Central African Republic	Haute-Kotto	Yalinga	12848	2010	130.3	107.9	156.4
Central African Republic	Haute-Kotto	Yalinga	12848	2015	129.8	106.0	156.2
Central African Republic	Kémo	Dékoa	12849	2000	174.7	144.5	212.1
Central African Republic	Kémo	Dékoa	12849	2005	178.3	147.2	216.9
Central African Republic	Kémo	Dékoa	12849	2010	157.2	127.8	192.5
Central African Republic	Kémo	Dékoa	12849	2015	134.6	109.2	163.9
Central African Republic	Kémo	Mala	12850	2000	173.5	141.7	212.1
Central African Republic	Kémo	Mala	12850	2005	176.4	143.4	215.9
Central African Republic	Kémo	Mala	12850	2010	155.4	125.0	192.0
Central African Republic	Kémo	Mala	12850	2015	134.6	107.7	166.4
Central African Republic	Kémo	Ndjoukou	12851	2000	177.0	147.8	208.5
Central African Republic	Kémo	Ndjoukou	12851	2005	178.9	149.3	210.3
Central African Republic	Kémo	Ndjoukou	12851	2010	161.4	133.9	191.9
Central African Republic	Kémo	Ndjoukou	12851	2015	139.6	114.5	167.1
Central African Republic	Kémo	Sibut	12852	2000	174.6	142.9	210.1
Central African Republic	Kémo	Sibut	12852	2005	176.4	145.5	213.7
Central African Republic	Kémo	Sibut	12852	2010	158.6	129.5	193.8
Central African Republic	Kémo	Sibut	12852	2015	136.2	110.2	166.8
Central African Republic	Lobaye	Boda	12853	2000	177.3	149.9	211.7
Central African Republic	Lobaye	Boda	12853	2005	153.4	129.1	183.6
Central African Republic	Lobaye	Boda	12853	2010	140.8	117.2	168.6
Central African Republic	Lobaye	Boda	12853	2015	121.4	99.2	146.1
Central African Republic	Lobaye	Boganangone	12854	2000	183.6	151.6	220.7
Central African Republic	Lobaye	Boganangone	12854	2005	164.2	135.8	198.3
Central African Republic	Lobaye	Boganangone	12854	2010	151.2	122.8	183.6
Central African Republic	Lobaye	Boganangone	12854	2015	129.2	103.4	157.5
Central African Republic	Lobaye	Boganda	12855	2000	182.2	153.0	217.9
Central African Republic	Lobaye	Boganda	12855	2005	159.9	134.4	191.4
Central African Republic	Lobaye	Boganda	12855	2010	147.0	121.6	175.7
Central African Republic	Lobaye	Boganda	12855	2015	128.1	103.2	154.9
Central African Republic	Lobaye	Mbaïki	12856	2000	175.0	150.5	200.6
Central African Republic	Lobaye	Mbaïki	12856	2005	151.0	130.7	173.8
Central African Republic	Lobaye	Mbaïki	12856	2010	141.9	121.2	165.4
Central African Republic	Lobaye	Mbaïki	12856	2015	120.8	101.0	142.2
Central African Republic	Lobaye	Mongoumba	12857	2000	172.0	147.1	197.7
Central African Republic	Lobaye	Mongoumba	12857	2005	150.7	130.2	173.6
Central African Republic	Lobaye	Mongoumba	12857	2010	144.0	123.1	168.5
Central African Republic	Lobaye	Mongoumba	12857	2015	117.7	98.6	139.3
Central African Republic	Mambéré-Kadéï	Amada-Gaza	12858	2000	184.9	158.1	214.4
Central African Republic	Mambéré-Kadéï	Amada-Gaza	12858	2005	160.0	136.4	185.2
Central African Republic	Mambéré-Kadéï	Amada-Gaza	12858	2010	153.9	130.0	181.8
Central African Republic	Mambéré-Kadéï	Amada-Gaza	12858	2015	132.6	110.6	156.7
Central African Republic	Mambéré-Kadéï	Berbérati	12859	2000	186.6	158.8	218.7
Central African Republic	Mambéré-Kadéï	Berbérati	12859	2005	162.4	137.7	193.6
Central African Republic	Mambéré-Kadéï	Berbérati	12859	2010	149.9	125.6	180.2
Central African Republic	Mambéré-Kadéï	Berbérati	12859	2015	131.0	108.3	159.8
Central African Republic	Mambéré-Kadéï	Carnot	12860	2000	185.8	157.1	220.8
Central African Republic	Mambéré-Kadéï	Carnot	12860	2005	162.7	136.0	194.2
Central African Republic	Mambéré-Kadéï	Carnot	12860	2010	151.1	126.2	183.4
Central African Republic	Mambéré-Kadéï	Carnot	12860	2015	133.7	108.1	163.7
Central African Republic	Mambéré-Kadéï	Dédé-Mokouba	12861	2000	191.5	166.0	221.7
Central African Republic	Mambéré-Kadéï	Dédé-Mokouba	12861	2005	163.1	140.9	190.0
Central African Republic	Mambéré-Kadéï	Dédé-Mokouba	12861	2010	153.7	128.8	181.9
Central African Republic	Mambéré-Kadéï	Dédé-Mokouba	12861	2015	131.7	109.5	157.2
Central African Republic	Mambéré-Kadéï	Gadzi	12862	2000	185.7	154.2	222.8
Central African Republic	Mambéré-Kadéï	Gadzi	12862	2005	168.9	140.2	201.9
Central African Republic	Mambéré-Kadéï	Gadzi	12862	2010	153.4	124.9	186.2
Central African Republic	Mambéré-Kadéï	Gadzi	12862	2015	134.5	107.9	165.0
Central African Republic	Mambéré-Kadéï	Gamboula	12863	2000	188.4	162.3	216.4
Central African Republic	Mambéré-Kadéï	Gamboula	12863	2005	161.1	138.8	186.3
Central African Republic	Mambéré-Kadéï	Gamboula	12863	2010	151.7	128.2	178.5
Central African Republic	Mambéré-Kadéï	Gamboula	12863	2015	132.9	110.7	157.7
Central African Republic	Mambéré-Kadéï	Sosso-Nakombo	12864	2000	189.8	160.7	221.7
Central African Republic	Mambéré-Kadéï	Sosso-Nakombo	12864	2005	164.0	139.4	193.6
Central African Republic	Mambéré-Kadéï	Sosso-Nakombo	12864	2010	153.0	128.0	183.4
Central African Republic	Mambéré-Kadéï	Sosso-Nakombo	12864	2015	131.1	108.5	157.7
Central African Republic	Mbomou	Bakouma	12865	2000	174.4	143.6	211.2

Admin 0	Admin 1	Admin 2	GAUL Code	Year	Under-5 mortality (per 1,000 livebirths)		
					Estimate	Lower bound, 95% UI	Upper bound, 95% UI
Central African Republic	Mbomou	Bakouma	12865	2005	173.7	141.7	209.7
Central African Republic	Mbomou	Bakouma	12865	2010	144.3	117.6	175.8
Central African Republic	Mbomou	Bakouma	12865	2015	141.9	114.7	173.6
Central African Republic	Mbomou	Bangassou	12866	2000	165.5	137.0	197.7
Central African Republic	Mbomou	Bangassou	12866	2005	163.9	135.6	194.9
Central African Republic	Mbomou	Bangassou	12866	2010	136.8	111.5	164.5
Central African Republic	Mbomou	Bangassou	12866	2015	131.6	107.6	159.5
Central African Republic	Mbomou	Gambo	12867	2000	171.3	143.3	203.4
Central African Republic	Mbomou	Gambo	12867	2005	168.9	143.8	198.6
Central African Republic	Mbomou	Gambo	12867	2010	139.6	117.3	167.6
Central African Republic	Mbomou	Gambo	12867	2015	129.0	107.3	155.5
Central African Republic	Mbomou	Ouango	12868	2000	167.2	138.2	198.9
Central African Republic	Mbomou	Ouango	12868	2005	164.8	138.2	194.6
Central African Republic	Mbomou	Ouango	12868	2010	135.7	112.5	163.0
Central African Republic	Mbomou	Ouango	12868	2015	124.5	102.1	150.9
Central African Republic	Mbomou	Rafai	12869	2000	162.5	133.0	196.0
Central African Republic	Mbomou	Rafai	12869	2005	160.8	130.0	195.5
Central African Republic	Mbomou	Rafai	12869	2010	142.0	115.3	175.3
Central African Republic	Mbomou	Rafai	12869	2015	142.3	115.1	175.0
Central African Republic	Nana-Gribizi	Kaga-Bandoro	12870	2000	172.2	143.4	204.1
Central African Republic	Nana-Gribizi	Kaga-Bandoro	12870	2005	174.1	144.4	206.5
Central African Republic	Nana-Gribizi	Kaga-Bandoro	12870	2010	151.2	125.1	181.2
Central African Republic	Nana-Gribizi	Kaga-Bandoro	12870	2015	127.2	105.0	154.5
Central African Republic	Nana-Gribizi	Mbrès	12871	2000	171.1	138.5	207.7
Central African Republic	Nana-Gribizi	Mbrès	12871	2005	170.4	136.8	210.3
Central African Republic	Nana-Gribizi	Mbrès	12871	2010	148.6	118.3	184.1
Central African Republic	Nana-Gribizi	Mbrès	12871	2015	127.7	101.7	159.2
Central African Republic	Nana-Mambéré	Abba	12872	2000	186.4	157.5	217.4
Central African Republic	Nana-Mambéré	Abba	12872	2005	161.7	135.5	190.7
Central African Republic	Nana-Mambéré	Abba	12872	2010	157.0	130.2	187.6
Central African Republic	Nana-Mambéré	Abba	12872	2015	135.6	110.3	163.0
Central African Republic	Nana-Mambéré	Baboua	12873	2000	179.4	156.1	206.2
Central African Republic	Nana-Mambéré	Baboua	12873	2005	156.6	136.2	179.6
Central African Republic	Nana-Mambéré	Baboua	12873	2010	155.6	132.7	180.4
Central African Republic	Nana-Mambéré	Baboua	12873	2015	134.6	114.0	158.9
Central African Republic	Nana-Mambéré	Baoro	12874	2000	183.3	152.2	219.3
Central African Republic	Nana-Mambéré	Baoro	12874	2005	167.9	138.8	200.9
Central African Republic	Nana-Mambéré	Baoro	12874	2010	156.3	128.4	190.0
Central African Republic	Nana-Mambéré	Baoro	12874	2015	137.3	111.8	167.6
Central African Republic	Nana-Mambéré	Bouar	12875	2000	183.5	155.9	214.0
Central African Republic	Nana-Mambéré	Bouar	12875	2005	164.3	140.3	192.1
Central African Republic	Nana-Mambéré	Bouar	12875	2010	157.5	131.8	186.6
Central African Republic	Nana-Mambéré	Bouar	12875	2015	137.5	114.4	164.6
Central African Republic	Ombella M'Poko	Bimbo	12876	2000	158.2	136.4	180.7
Central African Republic	Ombella M'Poko	Bimbo	12876	2005	145.2	125.9	167.2
Central African Republic	Ombella M'Poko	Bimbo	12876	2010	141.2	121.1	164.2
Central African Republic	Ombella M'Poko	Bimbo	12876	2015	121.3	102.1	144.5
Central African Republic	Ombella M'Poko	Boali	12877	2000	170.2	142.0	199.1
Central African Republic	Ombella M'Poko	Boali	12877	2005	159.0	133.6	188.3
Central African Republic	Ombella M'Poko	Boali	12877	2010	150.7	125.0	180.7
Central African Republic	Ombella M'Poko	Boali	12877	2015	127.6	104.1	155.3
Central African Republic	Ombella M'Poko	Bogangolo	12878	2000	173.8	144.6	209.9
Central African Republic	Ombella M'Poko	Bogangolo	12878	2005	175.3	144.3	216.3
Central African Republic	Ombella M'Poko	Bogangolo	12878	2010	158.1	129.1	194.7
Central African Republic	Ombella M'Poko	Bogangolo	12878	2015	135.2	108.7	166.3
Central African Republic	Ombella M'Poko	Bossembélé	12879	2000	167.5	138.4	201.0
Central African Republic	Ombella M'Poko	Bossembélé	12879	2005	163.0	134.8	197.0
Central African Republic	Ombella M'Poko	Bossembélé	12879	2010	147.9	120.2	179.1
Central African Republic	Ombella M'Poko	Bossembélé	12879	2015	127.2	103.2	156.9
Central African Republic	Ombella M'Poko	Damara	12880	2000	181.2	154.1	211.7
Central African Republic	Ombella M'Poko	Damara	12880	2005	172.9	146.1	206.9
Central African Republic	Ombella M'Poko	Damara	12880	2010	161.2	133.5	194.3
Central African Republic	Ombella M'Poko	Damara	12880	2015	136.9	113.2	164.9
Central African Republic	Ombella M'Poko	Yaloké	12881	2000	167.3	138.7	203.0
Central African Republic	Ombella M'Poko	Yaloké	12881	2005	163.8	135.3	199.1
Central African Republic	Ombella M'Poko	Yaloké	12881	2010	148.0	121.2	180.2
Central African Republic	Ombella M'Poko	Yaloké	12881	2015	129.9	104.5	160.7
Central African Republic	Ouaka	Bakala	12882	2000	191.3	158.1	233.0
Central African Republic	Ouaka	Bakala	12882	2005	186.4	152.5	226.2
Central African Republic	Ouaka	Bakala	12882	2010	158.4	127.6	191.8
Central African Republic	Ouaka	Bakala	12882	2015	146.3	116.2	179.7
Central African Republic	Ouaka	Bambari	12883	2000	186.9	156.6	221.3
Central African Republic	Ouaka	Bambari	12883	2005	183.0	151.8	215.4
Central African Republic	Ouaka	Bambari	12883	2010	154.4	127.4	185.8
Central African Republic	Ouaka	Bambari	12883	2015	139.5	113.7	169.8
Central African Republic	Ouaka	Grimari	12884	2000	197.3	164.2	236.0
Central African Republic	Ouaka	Grimari	12884	2005	191.3	158.7	230.8
Central African Republic	Ouaka	Grimari	12884	2010	167.9	137.9	202.7
Central African Republic	Ouaka	Grimari	12884	2015	145.1	117.5	176.5
Central African Republic	Ouaka	Ippy	12885	2000	185.2	149.2	227.6
Central African Republic	Ouaka	Ippy	12885	2005	180.8	146.3	219.1
Central African Republic	Ouaka	Ippy	12885	2010	148.6	119.5	181.0
Central African Republic	Ouaka	Ippy	12885	2015	141.9	113.6	175.1
Central African Republic	Ouaka	Kouango	12886	2000	193.2	162.4	228.3
Central African Republic	Ouaka	Kouango	12886	2005	187.6	157.7	220.4

Admin 0	Admin 1	Admin 2	GAUL Code	Year	Under-5 mortality (per 1,000 livebirths)		
					Estimate	Lower bound, 95% UI	Upper bound, 95% UI
Central African Republic	Ouaka	Kouango	12886	2010	163.0	134.7	194.5
Central African Republic	Ouaka	Kouango	12886	2015	143.1	117.2	172.6
Central African Republic	Ouham	Batangafo	12892	2000	191.4	160.9	224.1
Central African Republic	Ouham	Batangafo	12892	2005	202.6	170.9	238.1
Central African Republic	Ouham	Batangafo	12892	2010	172.6	144.7	205.5
Central African Republic	Ouham	Batangafo	12892	2015	154.5	126.9	186.0
Central African Republic	Ouham	Bossangoa	12894	2000	181.1	150.8	215.1
Central African Republic	Ouham	Bossangoa	12894	2005	188.5	155.6	225.2
Central African Republic	Ouham	Bossangoa	12894	2010	165.0	135.7	200.8
Central African Republic	Ouham	Bossangoa	12894	2015	149.5	123.7	183.8
Central African Republic	Ouham	Bouca	12895	2000	189.7	157.6	229.0
Central African Republic	Ouham	Bouca	12895	2005	193.7	159.8	236.2
Central African Republic	Ouham	Bouca	12895	2010	170.2	140.4	206.8
Central African Republic	Ouham	Bouca	12895	2015	149.6	122.4	185.6
Central African Republic	Ouham	Kabo	12896	2000	190.4	160.7	223.5
Central African Republic	Ouham	Kabo	12896	2005	195.2	165.4	228.5
Central African Republic	Ouham	Kabo	12896	2010	168.7	141.9	199.5
Central African Republic	Ouham	Kabo	12896	2015	144.4	121.0	172.7
Central African Republic	Ouham	Markounda	12897	2000	204.1	177.2	233.9
Central African Republic	Ouham	Markounda	12897	2005	224.4	194.3	257.9
Central African Republic	Ouham	Markounda	12897	2010	193.7	167.3	225.1
Central African Republic	Ouham	Markounda	12897	2015	177.9	151.7	208.3
Central African Republic	Ouham	Nana-Bakassa	12898	2000	185.3	153.2	222.5
Central African Republic	Ouham	Nana-Bakassa	12898	2005	198.8	161.9	236.6
Central African Republic	Ouham	Nana-Bakassa	12898	2010	171.2	140.9	207.3
Central African Republic	Ouham	Nana-Bakassa	12898	2015	157.0	127.2	192.2
Central African Republic	Ouham	Nangha Boguila	12893	2000	193.8	164.2	228.6
Central African Republic	Ouham	Nangha Boguila	12893	2005	208.4	173.8	246.1
Central African Republic	Ouham	Nangha Boguila	12893	2010	181.6	151.1	217.5
Central African Republic	Ouham	Nangha Boguila	12893	2015	168.3	139.6	202.8
Central African Republic	Ouham Pendé	Bocaranga	12887	2000	197.2	170.3	232.3
Central African Republic	Ouham Pendé	Bocaranga	12887	2005	194.3	165.7	227.8
Central African Republic	Ouham Pendé	Bocaranga	12887	2010	180.4	150.7	214.3
Central African Republic	Ouham Pendé	Bocaranga	12887	2015	169.6	141.2	202.0
Central African Republic	Ouham Pendé	Bossemtélé	114827	2000	187.2	155.3	224.0
Central African Republic	Ouham Pendé	Bossemtélé	114827	2005	183.0	150.8	219.0
Central African Republic	Ouham Pendé	Bossemtélé	114827	2010	164.6	134.4	199.1
Central African Republic	Ouham Pendé	Bossemtélé	114827	2015	155.1	125.5	192.0
Central African Republic	Ouham Pendé	Bozoum	114828	2000	190.6	161.2	223.0
Central African Republic	Ouham Pendé	Bozoum	114828	2005	187.6	157.9	221.8
Central African Republic	Ouham Pendé	Bozoum	114828	2010	169.7	140.9	202.0
Central African Republic	Ouham Pendé	Bozoum	114828	2015	158.3	129.8	192.0
Central African Republic	Ouham Pendé	Koui	12889	2000	191.5	165.7	223.6
Central African Republic	Ouham Pendé	Koui	12889	2005	184.0	158.5	214.5
Central African Republic	Ouham Pendé	Koui	12889	2010	173.7	147.4	205.1
Central African Republic	Ouham Pendé	Koui	12889	2015	161.6	135.4	193.3
Central African Republic	Ouham Pendé	Ngaoundaye	12890	2000	209.5	185.2	237.5
Central African Republic	Ouham Pendé	Ngaoundaye	12890	2005	216.1	190.3	246.1
Central African Republic	Ouham Pendé	Ngaoundaye	12890	2010	200.7	174.8	231.7
Central African Republic	Ouham Pendé	Ngaoundaye	12890	2015	192.3	163.5	223.2
Central African Republic	Ouham Pendé	Paoua	12891	2000	209.6	182.3	240.9
Central African Republic	Ouham Pendé	Paoua	12891	2005	219.2	189.5	251.6
Central African Republic	Ouham Pendé	Paoua	12891	2010	195.6	168.0	227.7
Central African Republic	Ouham Pendé	Paoua	12891	2015	186.4	157.4	220.5
Central African Republic	Sangha-Mbaéré	Bambio	12899	2000	177.9	150.5	212.4
Central African Republic	Sangha-Mbaéré	Bambio	12899	2005	155.2	131.7	181.1
Central African Republic	Sangha-Mbaéré	Bambio	12899	2010	143.0	120.3	170.5
Central African Republic	Sangha-Mbaéré	Bambio	12899	2015	123.0	100.6	146.9
Central African Republic	Sangha-Mbaéré	Bayanga	12900	2000	169.7	143.6	198.2
Central African Republic	Sangha-Mbaéré	Bayanga	12900	2005	149.5	127.1	175.7
Central African Republic	Sangha-Mbaéré	Bayanga	12900	2010	142.4	119.1	169.2
Central African Republic	Sangha-Mbaéré	Bayanga	12900	2015	117.8	98.7	141.9
Central African Republic	Sangha-Mbaéré	Nola	12901	2000	182.3	156.8	210.3
Central African Republic	Sangha-Mbaéré	Nola	12901	2005	157.3	135.8	182.2
Central African Republic	Sangha-Mbaéré	Nola	12901	2010	149.1	125.7	175.9
Central African Republic	Sangha-Mbaéré	Nola	12901	2015	127.2	106.2	152.0
Central African Republic	Vakaga	Birao	12902	2000	149.5	129.1	173.4
Central African Republic	Vakaga	Birao	12902	2005	138.6	118.6	160.7
Central African Republic	Vakaga	Birao	12902	2010	127.3	108.7	148.3
Central African Republic	Vakaga	Birao	12902	2015	113.8	97.2	134.3
Central African Republic	Vakaga	Ouanda-Djallé	12903	2000	150.6	121.4	183.8
Central African Republic	Vakaga	Ouanda-Djallé	12903	2005	144.5	115.8	177.9
Central African Republic	Vakaga	Ouanda-Djallé	12903	2010	129.0	102.9	158.7
Central African Republic	Vakaga	Ouanda-Djallé	12903	2015	125.0	100.3	155.0
Chad	Assongha	Barde	65374	2000	147.5	132.2	164.5
Chad	Assongha	Barde	65374	2005	131.5	118.9	145.5
Chad	Assongha	Barde	65374	2010	102.7	92.2	113.2
Chad	Assongha	Barde	65374	2015	83.2	73.2	94.5
Chad	Assongha	Guergne	65492	2000	151.9	135.9	168.6
Chad	Assongha	Guergne	65492	2005	135.5	121.9	150.0
Chad	Assongha	Guergne	65492	2010	99.8	89.9	110.6
Chad	Assongha	Guergne	65492	2015	80.0	70.0	91.0
Chad	Assongha	Kado	65505	2000	156.4	140.9	174.0
Chad	Assongha	Kado	65505	2005	139.7	126.9	154.1
Chad	Assongha	Kado	65505	2010	105.3	95.7	116.3

Admin 0	Admin 1	Admin 2	GAUL Code	Year	Under-5 mortality (per 1,000 livebirths)		
					Estimate	Lower bound, 95% UI	Upper bound, 95% UI
Chad	Assongha	Kado	65505	2015	82.9	72.9	94.0
Chad	Assongha	Mabrone	65561	2000	133.8	119.6	149.7
Chad	Assongha	Mabrone	65561	2005	114.4	102.1	127.3
Chad	Assongha	Mabrone	65561	2010	89.5	80.3	100.2
Chad	Assongha	Mabrone	65561	2015	73.9	64.7	84.3
Chad	Assongha	Molou	65607	2000	143.1	127.6	159.8
Chad	Assongha	Molou	65607	2005	125.5	112.0	139.3
Chad	Assongha	Molou	65607	2010	95.8	85.8	106.3
Chad	Assongha	Molou	65607	2015	78.5	68.4	89.3
Chad	Assongha	Troane	65690	2000	141.4	126.1	159.4
Chad	Assongha	Troane	65690	2005	122.3	108.9	137.1
Chad	Assongha	Troane	65690	2010	95.1	84.6	107.1
Chad	Assongha	Troane	65690	2015	77.6	67.6	89.4
Chad	Baguirmi	Ba-illi	65362	2000	199.0	179.7	218.9
Chad	Baguirmi	Ba-illi	65362	2005	193.8	174.6	212.7
Chad	Baguirmi	Ba-illi	65362	2010	172.2	154.6	189.3
Chad	Baguirmi	Ba-illi	65362	2015	154.8	137.4	173.3
Chad	Baguirmi	Batha-lairi	65377	2000	203.9	179.8	229.5
Chad	Baguirmi	Batha-lairi	65377	2005	200.7	177.3	225.6
Chad	Baguirmi	Batha-lairi	65377	2010	173.2	153.1	195.8
Chad	Baguirmi	Batha-lairi	65377	2015	150.8	130.8	174.3
Chad	Baguirmi	Bogomoro	65412	2000	178.6	158.4	200.8
Chad	Baguirmi	Bogomoro	65412	2005	182.0	162.2	203.8
Chad	Baguirmi	Bogomoro	65412	2010	165.1	146.9	187.2
Chad	Baguirmi	Bogomoro	65412	2015	148.9	129.8	171.0
Chad	Baguirmi	Bougoumene	65419	2000	185.2	167.9	203.9
Chad	Baguirmi	Bougoumene	65419	2005	167.7	151.9	184.5
Chad	Baguirmi	Bougoumene	65419	2010	154.1	139.8	169.3
Chad	Baguirmi	Bougoumene	65419	2015	136.9	122.6	153.7
Chad	Baguirmi	Bousso	65423	2000	207.3	185.0	231.4
Chad	Baguirmi	Bousso	65423	2005	207.4	186.6	229.5
Chad	Baguirmi	Bousso	65423	2010	180.1	161.1	199.5
Chad	Baguirmi	Bousso	65423	2015	161.8	141.9	183.0
Chad	Baguirmi	Deredia	65440	2000	180.0	160.0	201.7
Chad	Baguirmi	Deredia	65440	2005	179.8	161.4	201.0
Chad	Baguirmi	Deredia	65440	2010	158.1	140.2	178.4
Chad	Baguirmi	Deredia	65440	2015	139.6	122.3	161.1
Chad	Baguirmi	Dourbali/abouguerne	65461	2000	167.3	149.8	187.6
Chad	Baguirmi	Dourbali/abouguerne	65461	2005	158.4	141.3	175.9
Chad	Baguirmi	Dourbali/abouguerne	65461	2010	138.4	122.6	154.9
Chad	Baguirmi	Dourbali/abouguerne	65461	2015	125.4	110.6	142.5
Chad	Baguirmi	Mai-ache	65565	2000	166.7	148.4	186.4
Chad	Baguirmi	Mai-ache	65565	2005	154.1	137.7	171.8
Chad	Baguirmi	Mai-ache	65565	2010	134.6	119.7	149.8
Chad	Baguirmi	Mai-ache	65565	2015	119.4	105.2	135.1
Chad	Baguirmi	Mandjafa	65568	2000	161.4	144.2	178.1
Chad	Baguirmi	Mandjafa	65568	2005	155.9	140.5	173.0
Chad	Baguirmi	Mandjafa	65568	2010	143.3	128.5	160.6
Chad	Baguirmi	Mandjafa	65568	2015	128.4	114.2	144.8
Chad	Baguirmi	Massenya	65582	2000	188.0	170.4	207.9
Chad	Baguirmi	Massenya	65582	2005	186.2	169.6	204.8
Chad	Baguirmi	Massenya	65582	2010	162.5	147.2	180.0
Chad	Baguirmi	Massenya	65582	2015	142.8	127.3	161.8
Chad	Baguirmi	Miltou-gourgara	65599	2000	221.2	196.2	247.7
Chad	Baguirmi	Miltou-gourgara	65599	2005	219.9	196.6	245.0
Chad	Baguirmi	Miltou-gourgara	65599	2010	192.0	171.8	215.4
Chad	Baguirmi	Miltou-gourgara	65599	2015	178.8	156.0	203.6
Chad	Barh Koh	Balimba	65368	2000	212.4	189.4	235.5
Chad	Barh Koh	Balimba	65368	2005	175.9	158.0	193.7
Chad	Barh Koh	Balimba	65368	2010	139.6	124.7	156.2
Chad	Barh Koh	Balimba	65368	2015	115.9	101.0	131.5
Chad	Barh Koh	Banda	65370	2000	218.3	197.3	241.1
Chad	Barh Koh	Banda	65370	2005	185.0	166.8	203.6
Chad	Barh Koh	Banda	65370	2010	148.4	133.3	164.6
Chad	Barh Koh	Banda	65370	2015	127.2	111.9	143.8
Chad	Barh Koh	Djoli	65448	2000	218.1	196.4	242.0
Chad	Barh Koh	Djoli	65448	2005	187.2	168.6	206.4
Chad	Barh Koh	Djoli	65448	2010	157.0	141.1	175.2
Chad	Barh Koh	Djoli	65448	2015	137.4	120.9	156.1
Chad	Barh Koh	Kokaga	65528	2000	215.2	192.1	239.9
Chad	Barh Koh	Kokaga	65528	2005	177.6	158.9	196.4
Chad	Barh Koh	Kokaga	65528	2010	144.5	129.3	162.2
Chad	Barh Koh	Kokaga	65528	2015	117.1	102.8	133.2
Chad	Barh Koh	Korbol	65536	2000	236.7	210.3	265.4
Chad	Barh Koh	Korbol	65536	2005	216.2	192.3	241.3
Chad	Barh Koh	Korbol	65536	2010	191.8	170.6	215.5
Chad	Barh Koh	Korbol	65536	2015	173.6	151.1	198.2
Chad	Barh Koh	Koumogo	65545	2000	211.4	190.0	231.9
Chad	Barh Koh	Koumogo	65545	2005	178.4	161.3	195.6
Chad	Barh Koh	Koumogo	65545	2010	142.3	127.6	157.4
Chad	Barh Koh	Koumogo	65545	2015	119.8	104.8	135.2
Chad	Barh Koh	Maro	65577	2000	206.5	182.3	232.7
Chad	Barh Koh	Maro	65577	2005	173.1	153.6	196.5
Chad	Barh Koh	Maro	65577	2010	139.9	123.4	158.6
Chad	Barh Koh	Maro	65577	2015	120.3	104.0	139.7

Admin 0	Admin 1	Admin 2	GAUL Code	Year	Under-5 mortality (per 1,000 livebirths)		
					Estimate	Lower bound, 95% UI	Upper bound, 95% UI
Chad	Barh Koh	Moussafoyo	65622	2000	216.2	192.1	243.1
Chad	Barh Koh	Moussafoyo	65622	2005	180.0	160.6	201.8
Chad	Barh Koh	Moussafoyo	65622	2010	144.7	128.0	164.1
Chad	Barh Koh	Moussafoyo	65622	2015	124.3	107.5	143.2
Chad	Barh Koh	Niellim	65637	2000	217.1	192.6	246.4
Chad	Barh Koh	Niellim	65637	2005	193.3	173.0	217.1
Chad	Barh Koh	Niellim	65637	2010	168.4	149.4	189.4
Chad	Barh Koh	Niellim	65637	2015	153.7	133.5	175.8
Chad	Barh Koh	Sarh	65663	2000	198.1	175.6	222.8
Chad	Barh Koh	Sarh	65663	2005	163.7	145.8	181.2
Chad	Barh Koh	Sarh	65663	2010	128.7	114.3	145.1
Chad	Barh Koh	Sarh	65663	2015	105.2	91.5	119.7
Chad	Barl El Gazal	Michemire	65597	2000	135.2	122.0	150.4
Chad	Barl El Gazal	Michemire	65597	2005	111.5	100.8	123.3
Chad	Barl El Gazal	Michemire	65597	2010	87.1	78.5	96.2
Chad	Barl El Gazal	Michemire	65597	2015	72.6	63.9	82.0
Chad	Barl El Gazal	Moussoro	65623	2000	126.8	114.0	142.0
Chad	Barl El Gazal	Moussoro	65623	2005	105.6	95.4	117.5
Chad	Barl El Gazal	Moussoro	65623	2010	87.9	79.3	97.5
Chad	Barl El Gazal	Moussoro	65623	2015	76.1	67.2	85.8
Chad	Barl El Gazal	Salal	65657	2000	111.4	96.9	130.8
Chad	Barl El Gazal	Salal	65657	2005	91.1	79.5	105.5
Chad	Barl El Gazal	Salal	65657	2010	75.9	66.6	87.9
Chad	Barl El Gazal	Salal	65657	2015	65.3	56.2	77.2
Chad	Batha Est	Dhok/dopdop	65442	2000	137.2	118.7	156.6
Chad	Batha Est	Dhok/dopdop	65442	2005	116.9	101.3	132.9
Chad	Batha Est	Dhok/dopdop	65442	2010	95.4	82.3	108.5
Chad	Batha Est	Dhok/dopdop	65442	2015	84.7	72.3	98.9
Chad	Batha Est	Kouka-adjob	65542	2000	140.3	121.1	160.9
Chad	Batha Est	Kouka-adjob	65542	2005	121.4	105.6	138.2
Chad	Batha Est	Kouka-adjob	65542	2010	101.6	88.2	116.4
Chad	Batha Est	Kouka-adjob	65542	2015	89.1	76.3	103.0
Chad	Batha Est	Massalat-oum-hadjer	65581	2000	138.5	120.1	157.7
Chad	Batha Est	Massalat-oum-hadjer	65581	2005	116.2	100.9	132.4
Chad	Batha Est	Massalat-oum-hadjer	65581	2010	96.1	83.0	110.6
Chad	Batha Est	Massalat-oum-hadjer	65581	2015	88.2	74.7	103.4
Chad	Batha Est	Mesmedje	65595	2000	144.4	125.9	164.3
Chad	Batha Est	Mesmedje	65595	2005	127.7	110.9	145.5
Chad	Batha Est	Mesmedje	65595	2010	105.4	92.6	120.3
Chad	Batha Est	Mesmedje	65595	2015	89.6	77.8	103.4
Chad	Batha Est	Ratanine-har-djombo	65654	2000	127.8	110.1	147.7
Chad	Batha Est	Ratanine-har-djombo	65654	2005	112.0	97.0	129.2
Chad	Batha Est	Ratanine-har-djombo	65654	2010	93.0	80.1	107.5
Chad	Batha Est	Ratanine-har-djombo	65654	2015	85.0	72.2	99.5
Chad	Batha Est	Sedamis-mr-mn-assine	65664	2000	136.3	118.0	157.1
Chad	Batha Est	Sedamis-mr-mn-assine	65664	2005	119.1	103.3	137.6
Chad	Batha Est	Sedamis-mr-mn-assine	65664	2010	100.6	86.3	114.8
Chad	Batha Est	Sedamis-mr-mn-assine	65664	2015	86.9	73.5	101.2
Chad	Batha Est	Zioud-amsak	65697	2000	136.6	117.9	157.3
Chad	Batha Est	Zioud-amsak	65697	2005	116.2	100.5	132.3
Chad	Batha Est	Zioud-amsak	65697	2010	96.2	82.8	110.4
Chad	Batha Est	Zioud-amsak	65697	2015	87.4	74.3	102.3
Chad	Batha Ouest	Djaatne	65445	2000	133.8	113.0	158.1
Chad	Batha Ouest	Djaatne	65445	2005	117.7	98.7	139.7
Chad	Batha Ouest	Djaatne	65445	2010	96.7	81.4	114.3
Chad	Batha Ouest	Djaatne	65445	2015	90.3	74.3	108.4
Chad	Batha Ouest	Fitri	65469	2000	140.6	125.5	157.4
Chad	Batha Ouest	Fitri	65469	2005	132.9	118.5	148.6
Chad	Batha Ouest	Fitri	65469	2010	110.5	98.4	124.6
Chad	Batha Ouest	Fitri	65469	2015	98.5	85.8	112.9
Chad	Batha Ouest	Khozam	65516	2000	121.3	102.1	144.0
Chad	Batha Ouest	Khozam	65516	2005	108.2	91.5	128.7
Chad	Batha Ouest	Khozam	65516	2010	89.7	76.1	105.2
Chad	Batha Ouest	Khozam	65516	2015	83.5	68.8	99.8
Chad	Batha Ouest	Kouka-ati	65543	2000	147.5	130.4	167.6
Chad	Batha Ouest	Kouka-ati	65543	2005	132.4	115.3	149.8
Chad	Batha Ouest	Kouka-ati	65543	2010	109.4	94.9	123.4
Chad	Batha Ouest	Kouka-ati	65543	2015	95.4	82.5	110.3
Chad	Batha Ouest	Medego	65592	2000	158.3	140.7	179.2
Chad	Batha Ouest	Medego	65592	2005	143.9	128.0	162.4
Chad	Batha Ouest	Medego	65592	2010	120.1	105.5	137.2
Chad	Batha Ouest	Medego	65592	2015	103.7	88.8	120.1
Chad	Batha Ouest	Ouled-himed	65647	2000	116.1	100.2	135.7
Chad	Batha Ouest	Ouled-himed	65647	2005	101.6	87.3	118.2
Chad	Batha Ouest	Ouled-himed	65647	2010	85.4	73.3	100.1
Chad	Batha Ouest	Ouled-himed	65647	2015	77.3	65.1	90.8
Chad	Batha Ouest	Ouled-rachid	65648	2000	125.7	107.7	147.0
Chad	Batha Ouest	Ouled-rachid	65648	2005	111.9	96.1	132.1
Chad	Batha Ouest	Ouled-rachid	65648	2010	92.1	79.6	108.7
Chad	Batha Ouest	Ouled-rachid	65648	2015	86.1	73.3	103.1
Chad	Batha Ouest	Salamat	65658	2000	137.9	118.2	159.6
Chad	Batha Ouest	Salamat	65658	2005	123.9	106.7	143.3
Chad	Batha Ouest	Salamat	65658	2010	100.8	85.8	117.1
Chad	Batha Ouest	Salamat	65658	2015	95.7	80.3	113.6
Chad	Biltine	Abou-charib-i	65352	2000	119.6	105.8	135.1

Admin 0	Admin 1	Admin 2	GAUL Code	Year	Under-5 mortality (per 1,000 livebirths)		
					Estimate	Lower bound, 95% UI	Upper bound, 95% UI
Chad	Biltine	Abou-charib-i	65352	2005	99.9	88.1	112.1
Chad	Biltine	Abou-charib-i	65352	2010	78.1	68.6	88.4
Chad	Biltine	Abou-charib-i	65352	2015	68.8	59.2	78.3
Chad	Biltine	Abou-charib-ii	65353	2000	130.4	116.0	146.5
Chad	Biltine	Abou-charib-ii	65353	2005	110.5	98.4	123.9
Chad	Biltine	Abou-charib-ii	65353	2010	87.1	77.0	98.5
Chad	Biltine	Abou-charib-ii	65353	2015	75.0	65.0	85.9
Chad	Biltine	Arada	65360	2000	121.4	104.9	139.3
Chad	Biltine	Arada	65360	2005	101.5	87.4	116.1
Chad	Biltine	Arada	65360	2010	81.6	70.4	94.1
Chad	Biltine	Arada	65360	2015	73.3	62.2	86.3
Chad	Biltine	Bali	65367	2000	124.5	111.0	139.2
Chad	Biltine	Bali	65367	2005	100.4	90.0	112.4
Chad	Biltine	Bali	65367	2010	83.0	74.0	94.1
Chad	Biltine	Bali	65367	2015	69.7	59.9	80.0
Chad	Biltine	Birak	65407	2000	121.2	108.6	135.6
Chad	Biltine	Birak	65407	2005	97.3	87.5	108.1
Chad	Biltine	Birak	65407	2010	80.8	72.3	91.2
Chad	Biltine	Birak	65407	2015	68.8	59.7	78.5
Chad	Biltine	Djimeze	65447	2000	129.4	115.4	145.5
Chad	Biltine	Djimeze	65447	2005	107.3	95.5	120.6
Chad	Biltine	Djimeze	65447	2010	85.7	76.7	97.0
Chad	Biltine	Djimeze	65447	2015	70.6	60.8	80.5
Chad	Biltine	Dourene	65462	2000	105.2	90.1	121.9
Chad	Biltine	Dourene	65462	2005	84.5	72.8	97.7
Chad	Biltine	Dourene	65462	2010	68.4	58.7	79.4
Chad	Biltine	Dourene	65462	2015	63.2	53.5	74.2
Chad	Biltine	Fare	65467	2000	115.3	102.5	130.5
Chad	Biltine	Fare	65467	2005	91.8	81.6	103.3
Chad	Biltine	Fare	65467	2010	75.0	66.9	84.5
Chad	Biltine	Fare	65467	2015	65.3	56.3	75.2
Chad	Biltine	Gnere	65476	2000	128.2	113.5	144.8
Chad	Biltine	Gnere	65476	2005	106.3	94.0	120.7
Chad	Biltine	Gnere	65476	2010	84.4	74.5	95.8
Chad	Biltine	Gnere	65476	2015	69.3	60.0	79.4
Chad	Biltine	Gourouf	65488	2000	111.6	95.3	129.7
Chad	Biltine	Gourouf	65488	2005	90.2	77.0	105.2
Chad	Biltine	Gourouf	65488	2010	72.9	62.2	85.3
Chad	Biltine	Gourouf	65488	2015	65.5	54.7	77.8
Chad	Biltine	Guereda	65491	2000	120.2	106.7	136.2
Chad	Biltine	Guereda	65491	2005	95.3	84.9	108.3
Chad	Biltine	Guereda	65491	2010	78.9	70.1	89.8
Chad	Biltine	Guereda	65491	2015	65.2	55.9	75.4
Chad	Biltine	Kapka-bakaore	65507	2000	99.8	85.9	114.9
Chad	Biltine	Kapka-bakaore	65507	2005	80.3	69.9	92.6
Chad	Biltine	Kapka-bakaore	65507	2010	66.2	57.7	76.3
Chad	Biltine	Kapka-bakaore	65507	2015	61.3	52.2	71.2
Chad	Biltine	Kassine	65510	2000	125.7	111.3	141.1
Chad	Biltine	Kassine	65510	2005	105.1	93.1	118.3
Chad	Biltine	Kassine	65510	2010	82.6	73.7	93.3
Chad	Biltine	Kassine	65510	2015	69.9	60.5	79.9
Chad	Biltine	Kobe-nord-est	65521	2000	95.1	81.7	109.4
Chad	Biltine	Kobe-nord-est	65521	2005	77.2	67.1	88.7
Chad	Biltine	Kobe-nord-est	65521	2010	65.4	56.8	75.9
Chad	Biltine	Kobe-nord-est	65521	2015	57.3	49.0	67.8
Chad	Biltine	Kobe-nord-ouest	65522	2000	99.0	85.4	113.4
Chad	Biltine	Kobe-nord-ouest	65522	2005	79.5	68.9	90.2
Chad	Biltine	Kobe-nord-ouest	65522	2010	66.3	57.8	76.2
Chad	Biltine	Kobe-nord-ouest	65522	2015	59.5	50.8	69.4
Chad	Biltine	Kobe-sud	65523	2000	100.8	87.2	114.6
Chad	Biltine	Kobe-sud	65523	2005	79.7	69.7	89.8
Chad	Biltine	Kobe-sud	65523	2010	66.2	57.9	75.4
Chad	Biltine	Kobe-sud	65523	2015	57.0	48.6	66.9
Chad	Biltine	Kodoye-i	65524	2000	125.1	109.6	141.6
Chad	Biltine	Kodoye-i	65524	2005	104.3	91.8	118.1
Chad	Biltine	Kodoye-i	65524	2010	81.8	71.2	93.2
Chad	Biltine	Kodoye-i	65524	2015	71.1	61.0	81.6
Chad	Biltine	Kodoye-ii	65525	2000	129.5	113.8	146.3
Chad	Biltine	Kodoye-ii	65525	2005	110.3	98.0	123.8
Chad	Biltine	Kodoye-ii	65525	2010	86.9	76.1	98.4
Chad	Biltine	Kodoye-ii	65525	2015	75.2	64.3	86.6
Chad	Biltine	Kolonga	65532	2000	118.7	102.9	135.4
Chad	Biltine	Kolonga	65532	2005	96.3	84.3	109.8
Chad	Biltine	Kolonga	65532	2010	76.5	66.6	87.1
Chad	Biltine	Kolonga	65532	2015	65.8	55.8	76.2
Chad	Biltine	Koursigue	65547	2000	112.2	96.7	128.8
Chad	Biltine	Koursigue	65547	2005	88.6	77.4	102.0
Chad	Biltine	Koursigue	65547	2010	70.9	61.8	81.3
Chad	Biltine	Koursigue	65547	2015	62.0	52.2	72.7
Chad	Biltine	Lima	65558	2000	117.0	103.2	132.3
Chad	Biltine	Lima	65558	2005	91.7	80.9	104.0
Chad	Biltine	Lima	65558	2010	74.0	65.0	83.7
Chad	Biltine	Lima	65558	2015	63.3	54.1	73.3
Chad	Biltine	Mabraone	65560	2000	109.8	94.8	126.5
Chad	Biltine	Mabraone	65560	2005	88.5	76.6	101.8

Admin 0	Admin 1	Admin 2	GAUL Code	Year	Under-5 mortality (per 1,000 livebirths)		
					Estimate	Lower bound, 95% UI	Upper bound, 95% UI
Chad	Biltine	Mabraone	65560	2010	71.0	61.6	82.2
Chad	Biltine	Mabraone	65560	2015	63.9	54.4	74.2
Chad	Biltine	Mimi	65600	2000	121.4	105.3	138.1
Chad	Biltine	Mimi	65600	2005	101.0	88.5	115.0
Chad	Biltine	Mimi	65600	2010	79.6	68.8	90.3
Chad	Biltine	Mimi	65600	2015	71.6	60.7	83.1
Chad	Biltine	Moore	65610	2000	108.9	94.6	123.3
Chad	Biltine	Moore	65610	2005	85.3	74.6	97.2
Chad	Biltine	Moore	65610	2010	70.2	62.0	79.7
Chad	Biltine	Moore	65610	2015	59.7	51.4	69.4
Chad	Biltine	Ouled-djema	65646	2000	122.5	106.4	141.1
Chad	Biltine	Ouled-djema	65646	2005	101.7	88.5	115.5
Chad	Biltine	Ouled-djema	65646	2010	79.5	69.1	91.3
Chad	Biltine	Ouled-djema	65646	2015	71.3	60.8	82.3
Chad	Biltine	Ouli-koure	65649	2000	129.2	114.2	145.4
Chad	Biltine	Ouli-koure	65649	2005	105.8	93.6	119.9
Chad	Biltine	Ouli-koure	65649	2010	84.3	74.8	95.4
Chad	Biltine	Ouli-koure	65649	2015	69.4	60.3	79.7
Chad	Biltine	Troa	65689	2000	105.5	91.8	121.7
Chad	Biltine	Troa	65689	2005	83.8	73.0	96.3
Chad	Biltine	Troa	65689	2010	67.8	59.2	77.9
Chad	Biltine	Troa	65689	2015	59.8	51.1	69.6
Chad	Borkou	Borkou	65417	2000	140.2	124.0	156.8
Chad	Borkou	Borkou	65417	2005	118.8	106.6	131.2
Chad	Borkou	Borkou	65417	2010	102.6	91.4	114.2
Chad	Borkou	Borkou	65417	2015	90.1	79.2	102.0
Chad	Daraba	Amladoba	65358	2000	187.2	167.2	209.4
Chad	Daraba	Amladoba	65358	2005	181.5	163.1	203.6
Chad	Daraba	Amladoba	65358	2010	150.6	134.1	168.7
Chad	Daraba	Amladoba	65358	2015	133.7	116.7	153.8
Chad	Daraba	Bokoro/tania	65414	2000	160.2	142.5	181.9
Chad	Daraba	Bokoro/tania	65414	2005	151.3	133.4	171.6
Chad	Daraba	Bokoro/tania	65414	2010	126.9	111.3	143.3
Chad	Daraba	Bokoro/tania	65414	2015	118.1	100.4	137.3
Chad	Daraba	Dababa	65425	2000	164.8	147.6	185.0
Chad	Daraba	Dababa	65425	2005	159.6	142.9	178.3
Chad	Daraba	Dababa	65425	2010	133.4	118.2	149.9
Chad	Daraba	Dababa	65425	2015	120.6	104.0	138.3
Chad	Daraba	Mayakne	65584	2000	148.5	130.4	168.8
Chad	Daraba	Mayakne	65584	2005	139.7	122.9	158.8
Chad	Daraba	Mayakne	65584	2010	117.1	102.0	133.9
Chad	Daraba	Mayakne	65584	2015	108.7	92.8	125.9
Chad	Daraba	Moito	65605	2000	139.2	124.8	155.3
Chad	Daraba	Moito	65605	2005	127.9	114.5	142.0
Chad	Daraba	Moito	65605	2010	106.9	95.6	119.2
Chad	Daraba	Moito	65605	2015	96.4	84.6	109.4
Chad	Daraba	Yessie/ngama	65694	2000	204.8	180.5	231.5
Chad	Daraba	Yessie/ngama	65694	2005	197.9	175.8	222.7
Chad	Daraba	Yessie/ngama	65694	2010	168.2	148.6	189.7
Chad	Daraba	Yessie/ngama	65694	2015	151.0	130.6	173.4
Chad	Ennedi	Ennedi	65464	2000	125.7	111.8	141.9
Chad	Ennedi	Ennedi	65464	2005	103.5	91.8	116.9
Chad	Ennedi	Ennedi	65464	2010	89.5	78.9	100.9
Chad	Ennedi	Ennedi	65464	2015	73.5	64.0	84.5
Chad	Guera	Bidio	65404	2000	200.5	178.2	227.0
Chad	Guera	Bidio	65404	2005	186.5	165.3	208.5
Chad	Guera	Bidio	65404	2010	152.6	135.2	169.5
Chad	Guera	Bidio	65404	2015	132.3	115.6	152.1
Chad	Guera	Bitkine	65410	2000	203.1	181.3	226.8
Chad	Guera	Bitkine	65410	2005	189.6	170.4	211.0
Chad	Guera	Bitkine	65410	2010	160.5	143.5	179.5
Chad	Guera	Bitkine	65410	2015	141.8	123.5	162.1
Chad	Guera	Dadjo-i	65428	2000	180.1	162.5	198.3
Chad	Guera	Dadjo-i	65428	2005	164.4	148.6	180.0
Chad	Guera	Dadjo-i	65428	2010	134.3	120.7	147.1
Chad	Guera	Dadjo-i	65428	2015	116.6	103.1	132.0
Chad	Guera	Dadjo-ii	65429	2000	178.8	161.0	199.0
Chad	Guera	Dadjo-ii	65429	2005	163.0	146.8	181.6
Chad	Guera	Dadjo-ii	65429	2010	132.2	118.7	147.8
Chad	Guera	Dadjo-ii	65429	2015	110.7	98.2	125.8
Chad	Guera	Daguela	65431	2000	217.3	190.5	247.2
Chad	Guera	Daguela	65431	2005	195.8	172.1	222.5
Chad	Guera	Daguela	65431	2010	164.0	145.8	185.1
Chad	Guera	Daguela	65431	2015	144.8	126.4	166.8
Chad	Guera	Dangaleat	65432	2000	191.7	172.4	212.9
Chad	Guera	Dangaleat	65432	2005	181.9	164.4	200.5
Chad	Guera	Dangaleat	65432	2010	147.5	132.1	164.6
Chad	Guera	Dangaleat	65432	2015	129.4	112.5	147.1
Chad	Guera	Dekakire	65436	2000	216.3	189.2	246.5
Chad	Guera	Dekakire	65436	2005	215.9	189.9	245.9
Chad	Guera	Dekakire	65436	2010	192.2	168.8	220.0
Chad	Guera	Dekakire	65436	2015	171.6	147.2	199.6
Chad	Guera	Djonkor-aboutelfane	65449	2000	179.2	160.7	201.0
Chad	Guera	Djonkor-aboutelfane	65449	2005	164.6	147.2	182.9
Chad	Guera	Djonkor-aboutelfane	65449	2010	133.6	119.6	148.3

Admin 0	Admin 1	Admin 2	GAUL Code	Year	Under-5 mortality (per 1,000 livebirths)		
					Estimate	Lower bound, 95% UI	Upper bound, 95% UI
Chad	Guera	Djonkor-aboutelfane	65449	2015	112.3	98.4	128.9
Chad	Guera	Djonkor-guera	65450	2000	210.0	186.5	233.2
Chad	Guera	Djonkor-guera	65450	2005	198.7	179.4	220.3
Chad	Guera	Djonkor-guera	65450	2010	165.8	148.5	183.6
Chad	Guera	Djonkor-guera	65450	2015	145.7	128.1	165.8
Chad	Guera	Gogmi	65477	2000	224.4	193.4	258.3
Chad	Guera	Gogmi	65477	2005	217.5	190.3	250.8
Chad	Guera	Gogmi	65477	2010	181.8	157.9	208.2
Chad	Guera	Gogmi	65477	2015	161.0	136.8	188.8
Chad	Guera	Kenga	65513	2000	201.2	182.4	221.0
Chad	Guera	Kenga	65513	2005	190.5	173.4	209.0
Chad	Guera	Kenga	65513	2010	157.3	142.1	173.3
Chad	Guera	Kenga	65513	2015	138.0	121.4	155.6
Chad	Guera	Koffa	65526	2000	214.0	186.0	243.5
Chad	Guera	Koffa	65526	2005	202.3	176.3	232.7
Chad	Guera	Koffa	65526	2010	166.5	144.9	189.7
Chad	Guera	Koffa	65526	2015	145.0	124.1	168.2
Chad	Guera	Melfi	65593	2000	226.5	196.1	258.8
Chad	Guera	Melfi	65593	2005	219.7	190.4	250.9
Chad	Guera	Melfi	65593	2010	186.9	162.5	212.5
Chad	Guera	Melfi	65593	2015	168.8	142.6	195.2
Chad	Guera	Mokofi	65606	2000	218.8	193.0	249.0
Chad	Guera	Mokofi	65606	2005	215.5	190.5	243.5
Chad	Guera	Mokofi	65606	2010	183.4	160.7	207.3
Chad	Guera	Mokofi	65606	2015	162.6	138.7	188.9
Chad	Guera	Moubi-goz	65612	2000	153.5	134.4	174.5
Chad	Guera	Moubi-goz	65612	2005	131.9	115.8	150.3
Chad	Guera	Moubi-goz	65612	2010	106.9	94.2	121.9
Chad	Guera	Moubi-goz	65612	2015	90.7	79.0	104.8
Chad	Guera	Moubi-hadaba	65613	2000	153.5	135.5	173.7
Chad	Guera	Moubi-hadaba	65613	2005	135.4	120.4	152.6
Chad	Guera	Moubi-hadaba	65613	2010	111.1	98.2	126.3
Chad	Guera	Moubi-hadaba	65613	2015	90.8	79.2	104.9
Chad	Guera	Moubi-zarga	65614	2000	167.7	146.8	191.9
Chad	Guera	Moubi-zarga	65614	2005	150.7	132.3	173.0
Chad	Guera	Moubi-zarga	65614	2010	120.0	106.0	137.6
Chad	Guera	Moubi-zarga	65614	2015	102.7	89.2	119.2
Chad	Guera	Mousmare	65621	2000	221.2	190.5	253.5
Chad	Guera	Mousmare	65621	2005	219.7	191.4	251.1
Chad	Guera	Mousmare	65621	2010	192.2	167.4	220.8
Chad	Guera	Mousmare	65621	2015	176.2	150.6	208.1
Chad	Guera	Sara-arabe	65662	2000	200.7	179.2	223.4
Chad	Guera	Sara-arabe	65662	2005	186.0	167.9	206.0
Chad	Guera	Sara-arabe	65662	2010	154.4	138.5	171.3
Chad	Guera	Sara-arabe	65662	2015	132.6	116.8	151.2
Chad	Guera	Sorki	65668	2000	218.3	190.3	251.9
Chad	Guera	Sorki	65668	2005	206.0	179.2	237.7
Chad	Guera	Sorki	65668	2010	171.1	149.0	195.6
Chad	Guera	Sorki	65668	2015	147.4	125.4	171.6
Chad	Guera	Yalnass	65692	2000	181.6	160.4	204.8
Chad	Guera	Yalnass	65692	2005	173.1	154.2	193.2
Chad	Guera	Yalnass	65692	2010	140.2	124.7	156.2
Chad	Guera	Yalnass	65692	2015	122.1	106.8	140.5
Chad	Hadjer Lamis	Afrouk	65354	2000	195.5	181.4	210.7
Chad	Hadjer Lamis	Afrouk	65354	2005	168.3	156.1	181.3
Chad	Hadjer Lamis	Afrouk	65354	2010	145.5	133.0	157.5
Chad	Hadjer Lamis	Afrouk	65354	2015	137.8	124.3	152.7
Chad	Hadjer Lamis	El-fass	65463	2000	197.1	182.6	213.5
Chad	Hadjer Lamis	El-fass	65463	2005	170.4	157.6	184.4
Chad	Hadjer Lamis	El-fass	65463	2010	152.0	139.6	164.6
Chad	Hadjer Lamis	El-fass	65463	2015	141.8	129.0	157.2
Chad	Hadjer Lamis	Kadada	65502	2000	185.4	172.7	198.4
Chad	Hadjer Lamis	Kadada	65502	2005	151.5	141.1	162.2
Chad	Hadjer Lamis	Kadada	65502	2010	127.8	117.5	137.7
Chad	Hadjer Lamis	Kadada	65502	2015	125.6	114.1	138.9
Chad	Hadjer Lamis	Katoa	65511	2000	150.4	135.9	166.8
Chad	Hadjer Lamis	Katoa	65511	2005	136.6	122.9	150.6
Chad	Hadjer Lamis	Katoa	65511	2010	127.4	113.3	142.7
Chad	Hadjer Lamis	Katoa	65511	2015	119.5	104.0	136.9
Chad	Hadjer Lamis	Madiago	65562	2000	181.4	166.3	198.4
Chad	Hadjer Lamis	Madiago	65562	2005	162.1	148.4	176.9
Chad	Hadjer Lamis	Madiago	65562	2010	148.6	135.1	162.9
Chad	Hadjer Lamis	Madiago	65562	2015	139.2	124.4	156.1
Chad	Hadjer Lamis	Mani	65572	2000	172.9	158.7	187.4
Chad	Hadjer Lamis	Mani	65572	2005	146.4	134.9	158.3
Chad	Hadjer Lamis	Mani	65572	2010	121.4	110.9	132.2
Chad	Hadjer Lamis	Mani	65572	2015	115.9	103.8	128.5
Chad	Hadjer Lamis	Massaguet	65578	2000	173.2	155.5	192.1
Chad	Hadjer Lamis	Massaguet	65578	2005	152.6	136.6	169.0
Chad	Hadjer Lamis	Massaguet	65578	2010	133.7	119.8	148.6
Chad	Hadjer Lamis	Massaguet	65578	2015	126.4	111.8	143.3
Chad	Hadjer Lamis	Massakory	65579	2000	157.5	144.4	172.0
Chad	Hadjer Lamis	Massakory	65579	2005	134.9	122.9	147.6
Chad	Hadjer Lamis	Massakory	65579	2010	110.8	100.5	121.3
Chad	Hadjer Lamis	Massakory	65579	2015	104.3	93.2	115.4

Admin 0	Admin 1	Admin 2	GAUL Code	Year	Under-5 mortality (per 1,000 livebirths)		
					Estimate	Lower bound, 95% UI	Upper bound, 95% UI
Chad	Hadjer Lamis	Ndjamena	65629	2000	162.4	151.4	173.9
Chad	Hadjer Lamis	Ndjamena	65629	2005	140.0	130.2	149.8
Chad	Hadjer Lamis	Ndjamena	65629	2010	117.4	107.8	126.4
Chad	Hadjer Lamis	Ndjamena	65629	2015	112.2	101.6	124.0
Chad	Kabia	Berem	65397	2000	184.9	169.4	202.5
Chad	Kabia	Berem	65397	2005	186.7	172.4	202.4
Chad	Kabia	Berem	65397	2010	174.7	161.2	189.7
Chad	Kabia	Berem	65397	2015	142.5	128.3	158.0
Chad	Kabia	Djarao	65446	2000	170.1	155.0	185.3
Chad	Kabia	Djarao	65446	2005	168.4	155.2	182.3
Chad	Kabia	Djarao	65446	2010	163.3	149.7	178.0
Chad	Kabia	Djarao	65446	2015	135.5	121.2	151.3
Chad	Kabia	Domo	65456	2000	181.5	166.1	197.9
Chad	Kabia	Domo	65456	2005	183.2	168.2	198.8
Chad	Kabia	Domo	65456	2010	172.0	157.7	187.1
Chad	Kabia	Domo	65456	2015	139.1	124.3	155.6
Chad	Kabia	Fianga	65468	2000	146.9	135.0	158.7
Chad	Kabia	Fianga	65468	2005	141.2	129.4	152.9
Chad	Kabia	Fianga	65468	2010	139.7	126.7	152.6
Chad	Kabia	Fianga	65468	2015	117.0	103.9	130.3
Chad	Kabia	Gami	65475	2000	169.4	152.2	187.9
Chad	Kabia	Gami	65475	2005	166.2	150.9	183.2
Chad	Kabia	Gami	65475	2010	160.9	145.4	178.9
Chad	Kabia	Gami	65475	2015	136.5	121.2	155.1
Chad	Kabia	Gounou	65486	2000	165.8	152.3	180.4
Chad	Kabia	Gounou	65486	2005	164.1	151.4	177.2
Chad	Kabia	Gounou	65486	2010	158.1	145.3	171.6
Chad	Kabia	Gounou	65486	2015	131.1	118.3	145.6
Chad	Kabia	Gounou-gaya	65487	2000	172.3	157.4	188.8
Chad	Kabia	Gounou-gaya	65487	2005	173.4	158.9	188.1
Chad	Kabia	Gounou-gaya	65487	2010	164.8	150.6	179.0
Chad	Kabia	Gounou-gaya	65487	2015	136.1	122.4	152.1
Chad	Kabia	Kera	65514	2000	154.4	142.1	167.6
Chad	Kabia	Kera	65514	2005	150.4	138.1	162.6
Chad	Kabia	Kera	65514	2010	146.9	134.2	160.6
Chad	Kabia	Kera	65514	2015	123.5	110.3	136.9
Chad	Kabia	Leo-mbassa	65556	2000	181.4	164.5	197.4
Chad	Kabia	Leo-mbassa	65556	2005	178.6	165.1	193.7
Chad	Kabia	Leo-mbassa	65556	2010	169.9	155.9	184.3
Chad	Kabia	Leo-mbassa	65556	2015	138.6	124.3	154.2
Chad	Kabia	Malboum/hollom	65567	2000	156.3	144.0	169.3
Chad	Kabia	Malboum/hollom	65567	2005	153.2	141.9	165.6
Chad	Kabia	Malboum/hollom	65567	2010	149.8	136.9	163.0
Chad	Kabia	Malboum/hollom	65567	2015	125.6	112.2	139.8
Chad	Kabia	Tagal	65671	2000	172.5	157.9	188.2
Chad	Kabia	Tagal	65671	2005	171.0	157.4	185.1
Chad	Kabia	Tagal	65671	2010	161.9	148.9	176.1
Chad	Kabia	Tagal	65671	2015	133.5	120.1	148.2
Chad	Kabia	Tikem	65683	2000	156.2	141.8	171.4
Chad	Kabia	Tikem	65683	2005	149.1	134.6	163.9
Chad	Kabia	Tikem	65683	2010	145.8	131.0	160.8
Chad	Kabia	Tikem	65683	2015	120.6	106.5	136.4
Chad	Kabia	Youe	65696	2000	149.8	136.8	162.8
Chad	Kabia	Youe	65696	2005	141.7	129.0	154.3
Chad	Kabia	Youe	65696	2010	140.5	126.4	154.9
Chad	Kabia	Youe	65696	2015	117.7	104.2	132.0
Chad	Kanem	Amdobak	65357	2000	157.8	142.9	175.3
Chad	Kanem	Amdobak	65357	2005	129.4	117.4	142.6
Chad	Kanem	Amdobak	65357	2010	102.2	92.3	112.8
Chad	Kanem	Amdobak	65357	2015	94.4	83.2	105.7
Chad	Kanem	Dokora	65455	2000	154.8	140.3	172.2
Chad	Kanem	Dokora	65455	2005	127.7	115.1	141.7
Chad	Kanem	Dokora	65455	2010	99.7	89.9	110.8
Chad	Kanem	Dokora	65455	2015	86.3	76.0	98.0
Chad	Kanem	Illili	65497	2000	149.8	133.2	166.3
Chad	Kanem	Illili	65497	2005	127.0	114.1	141.5
Chad	Kanem	Illili	65497	2010	96.1	86.2	106.4
Chad	Kanem	Illili	65497	2015	88.5	78.0	101.4
Chad	Kanem	Moal	65602	2000	161.7	146.9	178.3
Chad	Kanem	Moal	65602	2005	133.6	121.1	147.0
Chad	Kanem	Moal	65602	2010	102.8	92.9	113.7
Chad	Kanem	Moal	65602	2015	88.3	78.0	101.0
Chad	Kanem	Mondo	65608	2000	163.3	147.9	179.7
Chad	Kanem	Mondo	65608	2005	136.2	122.9	150.4
Chad	Kanem	Mondo	65608	2010	103.8	93.9	114.2
Chad	Kanem	Mondo	65608	2015	96.5	85.1	109.9
Chad	Kanem	Motoa	65611	2000	166.0	149.5	183.1
Chad	Kanem	Motoa	65611	2005	138.8	126.0	154.0
Chad	Kanem	Motoa	65611	2010	106.2	95.9	117.9
Chad	Kanem	Motoa	65611	2015	103.5	90.7	118.2
Chad	Kanem	Mourzigui	65620	2000	157.1	142.4	172.6
Chad	Kanem	Mourzigui	65620	2005	130.9	119.0	144.4
Chad	Kanem	Mourzigui	65620	2010	101.9	92.6	112.9
Chad	Kanem	Mourzigui	65620	2015	92.1	81.5	105.2
Chad	Kanem	Ngouri	65636	2000	155.1	142.3	169.3

Admin 0	Admin 1	Admin 2	GAUL Code	Year	Under-5 mortality (per 1,000 livebirths)		
					Estimate	Lower bound, 95% UI	Upper bound, 95% UI
Chad	Kanem	Ngouri	65636	2005	128.2	117.8	139.4
Chad	Kanem	Ngouri	65636	2010	101.9	93.1	111.1
Chad	Kanem	Ngouri	65636	2015	87.6	78.3	97.9
Chad	Kanem	Nokou	65639	2000	118.6	102.2	139.3
Chad	Kanem	Nokou	65639	2005	93.0	80.4	108.2
Chad	Kanem	Nokou	65639	2010	78.4	67.0	91.6
Chad	Kanem	Nokou	65639	2015	66.3	56.5	78.1
Chad	Kanem	Ntiona	65640	2000	112.8	94.1	133.6
Chad	Kanem	Ntiona	65640	2005	90.3	75.4	108.7
Chad	Kanem	Ntiona	65640	2010	76.5	63.9	90.6
Chad	Kanem	Ntiona	65640	2015	62.5	51.5	75.5
Chad	Kanem	Rig-rig	65655	2000	126.3	109.8	143.6
Chad	Kanem	Rig-rig	65655	2005	99.9	87.3	114.1
Chad	Kanem	Rig-rig	65655	2010	81.0	70.2	92.6
Chad	Kanem	Rig-rig	65655	2015	67.9	58.7	78.3
Chad	Kanem	Sultanat	65670	2000	135.0	121.7	148.6
Chad	Kanem	Sultanat	65670	2005	112.3	102.0	123.7
Chad	Kanem	Sultanat	65670	2010	86.8	78.6	96.0
Chad	Kanem	Sultanat	65670	2015	75.1	66.3	84.5
Chad	Lac	Bol	65415	2000	141.3	127.9	154.8
Chad	Lac	Bol	65415	2005	114.8	104.3	125.9
Chad	Lac	Bol	65415	2010	92.9	84.1	102.1
Chad	Lac	Bol	65415	2015	82.3	73.6	91.9
Chad	Lac Iro	Alako	65355	2000	221.7	193.8	251.3
Chad	Lac Iro	Alako	65355	2005	187.2	163.7	212.8
Chad	Lac Iro	Alako	65355	2010	155.4	136.1	176.9
Chad	Lac Iro	Alako	65355	2015	140.0	119.3	160.6
Chad	Lac Iro	Bale	65366	2000	229.4	200.9	260.1
Chad	Lac Iro	Bale	65366	2005	189.9	165.8	216.4
Chad	Lac Iro	Bale	65366	2010	154.2	135.0	176.1
Chad	Lac Iro	Bale	65366	2015	137.6	117.6	158.6
Chad	Lac Iro	Baltoubaye	65369	2000	239.4	211.3	270.7
Chad	Lac Iro	Baltoubaye	65369	2005	202.3	179.3	226.5
Chad	Lac Iro	Baltoubaye	65369	2010	171.7	152.1	193.0
Chad	Lac Iro	Baltoubaye	65369	2015	155.6	135.5	177.2
Chad	Lac Iro	Bohobe	65413	2000	237.2	209.9	264.2
Chad	Lac Iro	Bohobe	65413	2005	199.0	177.1	220.6
Chad	Lac Iro	Bohobe	65413	2010	164.5	146.8	182.8
Chad	Lac Iro	Bohobe	65413	2015	148.6	129.8	169.6
Chad	Lac Iro	Boum-kebir	65420	2000	214.9	185.2	246.0
Chad	Lac Iro	Boum-kebir	65420	2005	187.5	163.5	216.9
Chad	Lac Iro	Boum-kebir	65420	2010	154.3	132.7	178.8
Chad	Lac Iro	Boum-kebir	65420	2015	139.6	118.0	162.9
Chad	Lac Iro	Dindje	65444	2000	227.0	200.1	256.1
Chad	Lac Iro	Dindje	65444	2005	190.5	168.8	216.3
Chad	Lac Iro	Dindje	65444	2010	154.5	136.1	175.3
Chad	Lac Iro	Dindje	65444	2015	136.6	117.8	157.1
Chad	Lac Iro	Koskobo	65539	2000	219.8	191.9	252.3
Chad	Lac Iro	Koskobo	65539	2005	181.2	157.6	209.3
Chad	Lac Iro	Koskobo	65539	2010	147.5	128.0	171.7
Chad	Lac Iro	Koskobo	65539	2015	130.0	110.0	152.6
Chad	Lac Iro	Kotongoro	65540	2000	227.6	203.7	253.4
Chad	Lac Iro	Kotongoro	65540	2005	192.6	172.7	213.1
Chad	Lac Iro	Kotongoro	65540	2010	157.6	141.7	175.6
Chad	Lac Iro	Kotongoro	65540	2015	137.3	120.6	155.9
Chad	Lac Iro	Kyabe	65550	2000	209.4	184.4	237.9
Chad	Lac Iro	Kyabe	65550	2005	170.3	148.5	193.7
Chad	Lac Iro	Kyabe	65550	2010	137.2	120.2	156.4
Chad	Lac Iro	Kyabe	65550	2015	115.9	98.8	133.8
Chad	Lac Iro	Marabe	65574	2000	227.9	202.0	254.9
Chad	Lac Iro	Marabe	65574	2005	191.6	171.3	214.8
Chad	Lac Iro	Marabe	65574	2010	156.9	139.4	176.6
Chad	Lac Iro	Marabe	65574	2015	138.0	120.8	158.0
Chad	Lac Iro	Moufa	65615	2000	219.3	188.9	251.1
Chad	Lac Iro	Moufa	65615	2005	187.7	162.7	217.4
Chad	Lac Iro	Moufa	65615	2010	158.0	136.6	182.6
Chad	Lac Iro	Moufa	65615	2015	142.9	120.2	165.9
Chad	Lac Iro	Ndjokou	65630	2000	212.5	184.6	244.5
Chad	Lac Iro	Ndjokou	65630	2005	178.1	154.3	209.7
Chad	Lac Iro	Ndjokou	65630	2010	148.6	127.1	173.7
Chad	Lac Iro	Ndjokou	65630	2015	133.0	112.8	157.4
Chad	Lac Iro	Simegotobe	65666	2000	238.0	210.1	268.5
Chad	Lac Iro	Simegotobe	65666	2005	198.9	175.4	222.8
Chad	Lac Iro	Simegotobe	65666	2010	163.3	144.6	184.4
Chad	Lac Iro	Simegotobe	65666	2015	149.8	130.4	172.3
Chad	Lac Iro	Singako	65667	2000	213.0	182.8	246.4
Chad	Lac Iro	Singako	65667	2005	182.3	157.3	211.4
Chad	Lac Iro	Singako	65667	2010	153.5	131.6	179.0
Chad	Lac Iro	Singako	65667	2015	137.5	115.6	163.6
Chad	Logone Occidental	Bah	65364	2000	233.0	213.1	252.3
Chad	Logone Occidental	Bah	65364	2005	237.4	219.0	256.1
Chad	Logone Occidental	Bah	65364	2010	206.3	189.4	224.4
Chad	Logone Occidental	Bah	65364	2015	163.3	146.8	180.4
Chad	Logone Occidental	Bao	65373	2000	243.8	224.1	264.7
Chad	Logone Occidental	Bao	65373	2005	240.2	221.8	261.0

Admin 0	Admin 1	Admin 2	GAUL Code	Year	Under-5 mortality (per 1,000 livebirths)		
					Estimate	Lower bound, 95% UI	Upper bound, 95% UI
Chad	Logone Occidental	Bao	65373	2010	212.6	195.3	231.0
Chad	Logone Occidental	Bao	65373	2015	161.6	144.2	180.2
Chad	Logone Occidental	Bebalem	65378	2000	247.9	226.9	271.0
Chad	Logone Occidental	Bebalem	65378	2005	237.8	219.0	259.2
Chad	Logone Occidental	Bebalem	65378	2010	202.6	186.1	221.2
Chad	Logone Occidental	Bebalem	65378	2015	150.9	135.3	169.4
Chad	Logone Occidental	Beinamar	65387	2000	227.1	206.2	248.9
Chad	Logone Occidental	Beinamar	65387	2005	218.1	198.6	239.4
Chad	Logone Occidental	Beinamar	65387	2010	198.4	180.9	219.8
Chad	Logone Occidental	Beinamar	65387	2015	159.8	141.9	178.8
Chad	Logone Occidental	Beissa	65388	2000	233.7	212.2	255.3
Chad	Logone Occidental	Beissa	65388	2005	231.6	211.9	253.0
Chad	Logone Occidental	Beissa	65388	2010	209.7	191.0	231.3
Chad	Logone Occidental	Beissa	65388	2015	168.9	151.0	189.6
Chad	Logone Occidental	Beladjia	65393	2000	254.2	231.7	276.2
Chad	Logone Occidental	Beladjia	65393	2005	252.4	232.7	274.5
Chad	Logone Occidental	Beladjia	65393	2010	214.0	195.9	233.8
Chad	Logone Occidental	Beladjia	65393	2015	160.7	143.8	179.9
Chad	Logone Occidental	Benoye	65395	2000	245.3	225.4	266.6
Chad	Logone Occidental	Benoye	65395	2005	233.2	215.2	253.1
Chad	Logone Occidental	Benoye	65395	2010	197.8	181.6	215.0
Chad	Logone Occidental	Benoye	65395	2015	148.5	133.4	166.4
Chad	Logone Occidental	Besseye	65402	2000	254.9	230.8	278.9
Chad	Logone Occidental	Besseye	65402	2005	245.3	225.1	268.9
Chad	Logone Occidental	Besseye	65402	2010	203.3	185.4	222.9
Chad	Logone Occidental	Besseye	65402	2015	155.0	138.5	174.6
Chad	Logone Occidental	Biramanda	65408	2000	244.4	225.6	263.0
Chad	Logone Occidental	Biramanda	65408	2005	238.9	221.2	257.2
Chad	Logone Occidental	Biramanda	65408	2010	208.7	193.1	225.5
Chad	Logone Occidental	Biramanda	65408	2015	155.8	139.9	173.0
Chad	Logone Occidental	Bourou	65421	2000	244.8	224.9	267.6
Chad	Logone Occidental	Bourou	65421	2005	234.3	216.1	253.7
Chad	Logone Occidental	Bourou	65421	2010	199.8	183.5	218.2
Chad	Logone Occidental	Bourou	65421	2015	144.9	130.1	162.0
Chad	Logone Occidental	Dadjile	65426	2000	242.1	222.6	262.8
Chad	Logone Occidental	Dadjile	65426	2005	238.3	219.9	259.2
Chad	Logone Occidental	Dadjile	65426	2010	212.5	194.9	231.4
Chad	Logone Occidental	Dadjile	65426	2015	162.6	145.2	180.9
Chad	Logone Occidental	Deli	65438	2000	237.3	218.8	257.6
Chad	Logone Occidental	Deli	65438	2005	240.2	222.7	259.7
Chad	Logone Occidental	Deli	65438	2010	211.3	195.5	228.2
Chad	Logone Occidental	Deli	65438	2015	165.2	149.0	182.2
Chad	Logone Occidental	Dodinda	65454	2000	235.1	215.0	254.6
Chad	Logone Occidental	Dodinda	65454	2005	237.1	218.8	256.8
Chad	Logone Occidental	Dodinda	65454	2010	204.2	187.8	223.2
Chad	Logone Occidental	Dodinda	65454	2015	162.5	146.2	180.1
Chad	Logone Occidental	Gore-loc	65480	2000	249.3	228.9	271.7
Chad	Logone Occidental	Gore-loc	65480	2005	235.4	217.4	256.0
Chad	Logone Occidental	Gore-loc	65480	2010	200.2	183.2	217.8
Chad	Logone Occidental	Gore-loc	65480	2015	148.4	133.0	166.3
Chad	Logone Occidental	Kaga	65506	2000	241.5	222.2	261.2
Chad	Logone Occidental	Kaga	65506	2005	244.7	226.7	264.6
Chad	Logone Occidental	Kaga	65506	2010	212.5	195.3	230.7
Chad	Logone Occidental	Kaga	65506	2015	161.3	145.0	179.5
Chad	Logone Occidental	Koutoutou	65548	2000	245.4	225.7	265.8
Chad	Logone Occidental	Koutoutou	65548	2005	234.6	215.9	254.0
Chad	Logone Occidental	Koutoutou	65548	2010	201.6	186.2	219.2
Chad	Logone Occidental	Koutoutou	65548	2015	149.9	134.6	168.3
Chad	Logone Occidental	Krim-krim	65549	2000	239.6	218.1	264.4
Chad	Logone Occidental	Krim-krim	65549	2005	240.1	220.8	263.5
Chad	Logone Occidental	Krim-krim	65549	2010	215.5	197.0	234.9
Chad	Logone Occidental	Krim-krim	65549	2015	165.7	147.8	185.5
Chad	Logone Occidental	Lao-kassi	65554	2000	228.2	209.3	248.9
Chad	Logone Occidental	Lao-kassi	65554	2005	228.7	211.3	249.7
Chad	Logone Occidental	Lao-kassi	65554	2010	211.4	194.9	230.7
Chad	Logone Occidental	Lao-kassi	65554	2015	168.7	151.9	188.0
Chad	Logone Occidental	Manso	65573	2000	247.7	226.6	270.7
Chad	Logone Occidental	Manso	65573	2005	237.4	217.6	258.3
Chad	Logone Occidental	Manso	65573	2010	205.2	187.2	224.4
Chad	Logone Occidental	Manso	65573	2015	148.4	132.2	166.9
Chad	Logone Occidental	Mbalkabra	65587	2000	242.0	223.5	260.8
Chad	Logone Occidental	Mbalkabra	65587	2005	242.3	225.4	261.2
Chad	Logone Occidental	Mbalkabra	65587	2010	212.3	196.7	229.2
Chad	Logone Occidental	Mbalkabra	65587	2015	158.8	143.2	175.8
Chad	Logone Occidental	Mballa	65588	2000	243.8	224.4	262.0
Chad	Logone Occidental	Mballa	65588	2005	243.7	225.7	262.2
Chad	Logone Occidental	Mballa	65588	2010	208.2	191.8	226.2
Chad	Logone Occidental	Mballa	65588	2015	156.3	141.0	173.5
Chad	Logone Occidental	Mbaouroye	65589	2000	262.7	240.5	286.2
Chad	Logone Occidental	Mbaouroye	65589	2005	240.6	222.7	261.5
Chad	Logone Occidental	Mbaouroye	65589	2010	202.3	186.4	219.6
Chad	Logone Occidental	Mbaouroye	65589	2015	151.2	135.5	168.5
Chad	Logone Occidental	Moundou/koutou	65616	2000	233.9	212.4	254.3
Chad	Logone Occidental	Moundou/koutou	65616	2005	232.8	212.8	254.4
Chad	Logone Occidental	Moundou/koutou	65616	2010	184.3	167.4	202.6

Admin 0	Admin 1	Admin 2	GAUL Code	Year	Under-5 mortality (per 1,000 livebirths) Estimate	Lower bound, 95% UI	Upper bound, 95% UI
Chad	Logone Occidental	Moundou/koutou	65616	2015	143.0	127.0	159.6
Chad	Logone Occidental	Ngondong	65635	2000	231.7	210.9	251.0
Chad	Logone Occidental	Ngondong	65635	2005	229.2	211.6	248.7
Chad	Logone Occidental	Ngondong	65635	2010	199.4	183.0	217.8
Chad	Logone Occidental	Ngondong	65635	2015	161.8	145.4	179.3
Chad	Logone Occidental	Saar	65656	2000	247.2	225.5	270.5
Chad	Logone Occidental	Saar	65656	2005	236.0	216.4	256.1
Chad	Logone Occidental	Saar	65656	2010	201.0	184.1	219.8
Chad	Logone Occidental	Saar	65656	2015	144.2	128.4	161.4
Chad	Logone Occidental	Tagoro	65673	2000	244.4	223.7	266.2
Chad	Logone Occidental	Tagoro	65673	2005	242.9	223.6	265.1
Chad	Logone Occidental	Tagoro	65673	2010	216.2	198.1	235.3
Chad	Logone Occidental	Tagoro	65673	2015	161.7	143.7	181.1
Chad	Logone Occidental	Tapol	65676	2000	237.3	215.0	259.2
Chad	Logone Occidental	Tapol	65676	2005	224.3	205.3	245.6
Chad	Logone Occidental	Tapol	65676	2010	198.5	180.9	218.8
Chad	Logone Occidental	Tapol	65676	2015	162.5	144.9	181.3
Chad	Logone Occidental	Tchaouen/saoua	65678	2000	242.8	221.9	266.3
Chad	Logone Occidental	Tchaouen/saoua	65678	2005	234.1	213.7	253.9
Chad	Logone Occidental	Tchaouen/saoua	65678	2010	198.4	181.6	217.3
Chad	Logone Occidental	Tchaouen/saoua	65678	2015	145.7	129.7	163.6
Chad	Logone Oriental	Bebedjia	65379	2000	256.8	235.3	278.7
Chad	Logone Oriental	Bebedjia	65379	2005	252.1	233.3	273.4
Chad	Logone Oriental	Bebedjia	65379	2010	206.9	190.9	224.1
Chad	Logone Oriental	Bebedjia	65379	2015	156.6	140.5	174.8
Chad	Logone Oriental	Beboni	65381	2000	252.1	231.4	273.9
Chad	Logone Oriental	Beboni	65381	2005	244.3	227.3	265.1
Chad	Logone Oriental	Beboni	65381	2010	209.5	193.5	226.3
Chad	Logone Oriental	Beboni	65381	2015	157.5	140.8	175.1
Chad	Logone Oriental	Beboto	65383	2000	264.2	240.8	289.6
Chad	Logone Oriental	Beboto	65383	2005	247.2	226.3	270.2
Chad	Logone Oriental	Beboto	65383	2010	208.9	190.3	229.9
Chad	Logone Oriental	Beboto	65383	2015	170.2	151.1	192.6
Chad	Logone Oriental	Bekan	65391	2000	259.5	231.8	288.7
Chad	Logone Oriental	Bekan	65391	2005	239.8	215.8	266.3
Chad	Logone Oriental	Bekan	65391	2010	200.9	179.8	222.9
Chad	Logone Oriental	Bekan	65391	2015	169.7	148.7	192.6
Chad	Logone Oriental	Bero	65398	2000	262.6	240.0	286.3
Chad	Logone Oriental	Bero	65398	2005	252.6	232.2	275.1
Chad	Logone Oriental	Bero	65398	2010	218.2	200.2	238.1
Chad	Logone Oriental	Bero	65398	2015	166.0	147.6	185.8
Chad	Logone Oriental	Beti	65403	2000	263.8	242.6	287.6
Chad	Logone Oriental	Beti	65403	2005	248.3	228.7	268.2
Chad	Logone Oriental	Beti	65403	2010	220.1	201.8	239.0
Chad	Logone Oriental	Beti	65403	2015	170.9	152.1	191.8
Chad	Logone Oriental	Bodo	65411	2000	259.0	236.4	286.3
Chad	Logone Oriental	Bodo	65411	2005	238.8	218.2	261.1
Chad	Logone Oriental	Bodo	65411	2010	207.7	187.1	229.1
Chad	Logone Oriental	Bodo	65411	2015	166.0	146.2	186.9
Chad	Logone Oriental	Boro	65418	2000	222.1	201.1	242.3
Chad	Logone Oriental	Boro	65418	2005	213.5	195.3	233.2
Chad	Logone Oriental	Boro	65418	2010	174.2	158.5	192.1
Chad	Logone Oriental	Boro	65418	2015	135.9	120.9	152.3
Chad	Logone Oriental	Boye-bessao	65424	2000	239.8	219.6	260.5
Chad	Logone Oriental	Boye-bessao	65424	2005	241.6	222.8	263.6
Chad	Logone Oriental	Boye-bessao	65424	2010	204.0	188.1	222.9
Chad	Logone Oriental	Boye-bessao	65424	2015	164.8	149.2	182.8
Chad	Logone Oriental	Doba	65452	2000	256.2	235.0	278.0
Chad	Logone Oriental	Doba	65452	2005	234.8	215.9	254.6
Chad	Logone Oriental	Doba	65452	2010	197.7	180.7	214.0
Chad	Logone Oriental	Doba	65452	2015	151.7	135.0	170.5
Chad	Logone Oriental	Gore	65479	2000	256.1	227.7	286.8
Chad	Logone Oriental	Gore	65479	2005	232.5	206.6	260.0
Chad	Logone Oriental	Gore	65479	2010	190.1	169.2	212.9
Chad	Logone Oriental	Gore	65479	2015	161.2	140.5	184.0
Chad	Logone Oriental	Kaba-donia	65498	2000	255.3	231.9	279.1
Chad	Logone Oriental	Kaba-donia	65498	2005	255.1	232.2	277.7
Chad	Logone Oriental	Kaba-donia	65498	2010	218.1	199.4	238.5
Chad	Logone Oriental	Kaba-donia	65498	2015	171.4	152.6	191.3
Chad	Logone Oriental	Kaba-roangar	65499	2000	249.9	217.3	283.7
Chad	Logone Oriental	Kaba-roangar	65499	2005	224.7	196.6	254.0
Chad	Logone Oriental	Kaba-roangar	65499	2010	186.3	162.0	213.0
Chad	Logone Oriental	Kaba-roangar	65499	2015	161.3	140.1	188.4
Chad	Logone Oriental	Kara/maibo-goulaye	65508	2000	242.2	221.4	263.3
Chad	Logone Oriental	Kara/maibo-goulaye	65508	2005	222.8	204.0	240.6
Chad	Logone Oriental	Kara/maibo-goulaye	65508	2010	194.3	178.6	210.8
Chad	Logone Oriental	Kara/maibo-goulaye	65508	2015	149.5	133.7	166.9
Chad	Logone Oriental	Kome/ndobele	65534	2000	269.6	245.6	296.1
Chad	Logone Oriental	Kome/ndobele	65534	2005	261.9	239.1	285.3
Chad	Logone Oriental	Kome/ndobele	65534	2010	221.4	202.1	243.4
Chad	Logone Oriental	Kome/ndobele	65534	2015	174.7	155.8	197.4
Chad	Logone Oriental	Maibo-mbaye	65566	2000	250.5	226.9	273.9
Chad	Logone Oriental	Maibo-mbaye	65566	2005	230.2	209.6	250.3
Chad	Logone Oriental	Maibo-mbaye	65566	2010	198.4	181.2	215.3
Chad	Logone Oriental	Maibo-mbaye	65566	2015	149.4	132.3	167.6

Admin 0	Admin 1	Admin 2	GAUL Code	Year	Under-5 mortality (per 1,000 livebirths) Estimate	Lower bound, 95% UI	Upper bound, 95% UI
Chad	Logone Oriental	Mango	65570	2000	253.9	231.8	276.9
Chad	Logone Oriental	Mango	65570	2005	233.7	214.1	253.9
Chad	Logone Oriental	Mango	65570	2010	196.9	180.1	214.1
Chad	Logone Oriental	Mango	65570	2015	148.8	132.6	167.4
Chad	Logone Oriental	Mbaikoro	65585	2000	231.8	210.7	252.2
Chad	Logone Oriental	Mbaikoro	65585	2005	229.7	210.2	250.1
Chad	Logone Oriental	Mbaikoro	65585	2010	179.4	163.6	198.1
Chad	Logone Oriental	Mbaikoro	65585	2015	138.0	122.7	154.5
Chad	Logone Oriental	Mbikou	65590	2000	256.1	235.2	277.3
Chad	Logone Oriental	Mbikou	65590	2005	256.5	238.1	277.3
Chad	Logone Oriental	Mbikou	65590	2010	219.6	201.5	239.0
Chad	Logone Oriental	Mbikou	65590	2015	165.5	148.5	184.1
Chad	Logone Oriental	Miandoum	65596	2000	262.1	238.2	286.9
Chad	Logone Oriental	Miandoum	65596	2005	258.5	237.2	282.0
Chad	Logone Oriental	Miandoum	65596	2010	223.9	204.2	245.3
Chad	Logone Oriental	Miandoum	65596	2015	171.5	152.5	192.0
Chad	Logone Oriental	Miladi	65598	2000	255.1	232.5	277.1
Chad	Logone Oriental	Miladi	65598	2005	258.8	238.0	279.4
Chad	Logone Oriental	Miladi	65598	2010	220.8	201.7	240.4
Chad	Logone Oriental	Miladi	65598	2015	169.8	152.3	189.7
Chad	Logone Oriental	Nankesse	65625	2000	255.5	231.7	279.8
Chad	Logone Oriental	Nankesse	65625	2005	247.8	227.5	270.0
Chad	Logone Oriental	Nankesse	65625	2010	212.8	194.7	231.4
Chad	Logone Oriental	Nankesse	65625	2015	158.6	140.8	178.3
Chad	Logone Oriental	Nassian	65626	2000	246.9	224.0	270.6
Chad	Logone Oriental	Nassian	65626	2005	232.4	211.1	254.6
Chad	Logone Oriental	Nassian	65626	2010	199.2	180.9	218.0
Chad	Logone Oriental	Nassian	65626	2015	148.5	130.6	167.0
Chad	Logone Oriental	Sama	65661	2000	243.0	223.2	264.4
Chad	Logone Oriental	Sama	65661	2005	229.6	212.0	247.6
Chad	Logone Oriental	Sama	65661	2010	199.3	183.0	216.8
Chad	Logone Oriental	Sama	65661	2015	152.2	135.8	169.5
Chad	Logone Oriental	Timberi	65684	2000	264.9	240.5	290.9
Chad	Logone Oriental	Timberi	65684	2005	254.9	232.7	278.4
Chad	Logone Oriental	Timberi	65684	2010	213.8	195.4	235.7
Chad	Logone Oriental	Timberi	65684	2015	174.1	155.8	195.6
Chad	Logone Oriental	Yamodo	65693	2000	248.3	217.5	280.9
Chad	Logone Oriental	Yamodo	65693	2005	222.0	195.3	252.0
Chad	Logone Oriental	Yamodo	65693	2010	184.5	162.0	212.3
Chad	Logone Oriental	Yamodo	65693	2015	157.5	135.0	183.5
Chad	Mandoul	Bangoul	65372	2000	229.7	210.1	251.4
Chad	Mandoul	Bangoul	65372	2005	213.9	196.4	232.1
Chad	Mandoul	Bangoul	65372	2010	184.6	167.2	201.2
Chad	Mandoul	Bangoul	65372	2015	151.6	134.1	169.9
Chad	Mandoul	Bebo-pen	65380	2000	236.6	216.9	259.2
Chad	Mandoul	Bebo-pen	65380	2005	222.0	204.4	239.8
Chad	Mandoul	Bebo-pen	65380	2010	195.5	177.8	213.2
Chad	Mandoul	Bebo-pen	65380	2015	159.4	141.6	178.3
Chad	Mandoul	Beboro	65382	2000	216.7	196.9	238.5
Chad	Mandoul	Beboro	65382	2005	193.5	176.9	211.2
Chad	Mandoul	Beboro	65382	2010	162.8	148.4	179.2
Chad	Mandoul	Beboro	65382	2015	138.8	122.9	155.5
Chad	Mandoul	Bedaya	65384	2000	206.2	184.7	229.4
Chad	Mandoul	Bedaya	65384	2005	176.8	158.5	193.8
Chad	Mandoul	Bedaya	65384	2010	142.5	127.4	158.2
Chad	Mandoul	Bedaya	65384	2015	125.5	110.0	142.0
Chad	Mandoul	Bedjondo	65385	2000	242.8	220.5	266.0
Chad	Mandoul	Bedjondo	65385	2005	227.8	208.0	249.0
Chad	Mandoul	Bedjondo	65385	2010	195.9	177.2	213.8
Chad	Mandoul	Bedjondo	65385	2015	158.0	139.6	178.2
Chad	Mandoul	Begara	65386	2000	228.8	206.5	252.7
Chad	Mandoul	Begara	65386	2005	198.3	180.9	218.7
Chad	Mandoul	Begara	65386	2010	170.9	154.1	189.0
Chad	Mandoul	Begara	65386	2015	142.8	125.7	160.6
Chad	Mandoul	Bekamba-koumra	65389	2000	223.0	203.9	244.9
Chad	Mandoul	Bekamba-koumra	65389	2005	205.9	188.9	223.6
Chad	Mandoul	Bekamba-koumra	65389	2010	174.0	158.0	190.2
Chad	Mandoul	Bekamba-koumra	65389	2015	142.5	125.9	159.5
Chad	Mandoul	Bekamba-moissala	65390	2000	213.3	191.9	235.9
Chad	Mandoul	Bekamba-moissala	65390	2005	181.9	164.5	199.9
Chad	Mandoul	Bekamba-moissala	65390	2010	148.9	133.9	166.2
Chad	Mandoul	Bekamba-moissala	65390	2015	128.5	112.2	145.0
Chad	Mandoul	Bekourou	65392	2000	234.9	210.3	261.8
Chad	Mandoul	Bekourou	65392	2005	205.3	184.8	229.1
Chad	Mandoul	Bekourou	65392	2010	175.1	156.5	194.7
Chad	Mandoul	Bekourou	65392	2015	149.4	129.8	169.5
Chad	Mandoul	Bengoro	65394	2000	237.7	216.8	260.0
Chad	Mandoul	Bengoro	65394	2005	212.6	195.5	230.7
Chad	Mandoul	Bengoro	65394	2010	184.1	167.9	201.3
Chad	Mandoul	Bengoro	65394	2015	153.4	136.5	171.2
Chad	Mandoul	Bessada	65399	2000	204.6	184.1	226.6
Chad	Mandoul	Bessada	65399	2005	180.7	163.1	198.8
Chad	Mandoul	Bessada	65399	2010	148.9	134.2	164.3
Chad	Mandoul	Bessada	65399	2015	129.6	114.0	146.2
Chad	Mandoul	Bessara	65401	2000	227.6	205.8	251.3

Admin 0	Admin 1	Admin 2	GAUL Code	Year	Under-5 mortality (per 1,000 livebirths)		
					Estimate	Lower bound, 95% UI	Upper bound, 95% UI
Chad	Mandoul	Bessara	65401	2005	195.5	178.3	216.2
Chad	Mandoul	Bessara	65401	2010	167.9	151.8	185.8
Chad	Mandoul	Bessara	65401	2015	144.2	127.5	162.5
Chad	Mandoul	Dembo	65439	2000	218.9	193.3	245.4
Chad	Mandoul	Dembo	65439	2005	186.9	165.6	210.8
Chad	Mandoul	Dembo	65439	2010	158.3	138.5	178.7
Chad	Mandoul	Dembo	65439	2015	137.1	117.6	159.1
Chad	Mandoul	Dilingala	65443	2000	236.6	215.1	260.6
Chad	Mandoul	Dilingala	65443	2005	207.3	189.0	227.5
Chad	Mandoul	Dilingala	65443	2010	179.5	162.3	197.0
Chad	Mandoul	Dilingala	65443	2015	151.2	132.7	169.1
Chad	Mandoul	Dobo	65453	2000	201.5	181.4	223.2
Chad	Mandoul	Dobo	65453	2005	180.7	162.9	199.5
Chad	Mandoul	Dobo	65453	2010	151.6	135.7	168.0
Chad	Mandoul	Dobo	65453	2015	135.4	118.6	152.8
Chad	Mandoul	Doubadene	65458	2000	230.8	206.1	258.4
Chad	Mandoul	Doubadene	65458	2005	198.0	177.8	221.5
Chad	Mandoul	Doubadene	65458	2010	168.3	149.9	188.2
Chad	Mandoul	Doubadene	65458	2015	145.0	125.9	165.5
Chad	Mandoul	Gabian	65471	2000	215.8	190.4	240.7
Chad	Mandoul	Gabian	65471	2005	182.1	161.3	203.9
Chad	Mandoul	Gabian	65471	2010	150.0	132.8	169.3
Chad	Mandoul	Gabian	65471	2015	131.4	114.5	151.5
Chad	Mandoul	Gon	65478	2000	224.0	198.5	251.7
Chad	Mandoul	Gon	65478	2005	194.1	173.3	217.8
Chad	Mandoul	Gon	65478	2010	165.5	147.2	186.4
Chad	Mandoul	Gon	65478	2015	142.3	122.4	163.3
Chad	Mandoul	Goundi	65485	2000	201.2	179.9	224.9
Chad	Mandoul	Goundi	65485	2005	185.2	166.1	207.6
Chad	Mandoul	Goundi	65485	2010	154.9	138.5	173.2
Chad	Mandoul	Goundi	65485	2015	140.7	121.9	159.2
Chad	Mandoul	Koldaga	65529	2000	241.1	216.7	270.1
Chad	Mandoul	Koldaga	65529	2005	218.0	197.2	243.3
Chad	Mandoul	Koldaga	65529	2010	183.0	163.7	204.2
Chad	Mandoul	Koldaga	65529	2015	158.0	137.9	180.3
Chad	Mandoul	Koumra	65546	2000	200.9	183.2	220.7
Chad	Mandoul	Koumra	65546	2005	178.3	162.8	194.5
Chad	Mandoul	Koumra	65546	2010	142.7	129.9	156.2
Chad	Mandoul	Koumra	65546	2015	122.7	108.6	136.8
Chad	Mandoul	Mahim-toki	65564	2000	205.7	186.3	227.2
Chad	Mandoul	Mahim-toki	65564	2005	188.7	170.5	208.5
Chad	Mandoul	Mahim-toki	65564	2010	156.2	140.7	172.7
Chad	Mandoul	Mahim-toki	65564	2015	135.4	119.9	151.3
Chad	Mandoul	Matekaga	65583	2000	201.0	181.3	221.5
Chad	Mandoul	Matekaga	65583	2005	182.9	165.8	200.8
Chad	Mandoul	Matekaga	65583	2010	147.5	133.9	162.2
Chad	Mandoul	Matekaga	65583	2015	129.6	115.0	145.5
Chad	Mandoul	Moissala	65604	2000	217.0	195.4	238.9
Chad	Mandoul	Moissala	65604	2005	188.9	171.2	208.8
Chad	Mandoul	Moissala	65604	2010	158.1	142.9	175.6
Chad	Mandoul	Moissala	65604	2015	136.4	120.2	154.1
Chad	Mandoul	Mouroum-goulaye	65617	2000	212.1	190.4	233.7
Chad	Mandoul	Mouroum-goulaye	65617	2005	194.2	174.7	214.2
Chad	Mandoul	Mouroum-goulaye	65617	2010	160.7	144.9	177.8
Chad	Mandoul	Mouroum-goulaye	65617	2015	135.9	120.7	153.4
Chad	Mandoul	Nadili	65624	2000	220.0	196.0	244.1
Chad	Mandoul	Nadili	65624	2005	186.6	167.2	207.2
Chad	Mandoul	Nadili	65624	2010	158.4	141.4	176.2
Chad	Mandoul	Nadili	65624	2015	136.7	119.7	156.6
Chad	Mandoul	Nderguigui	65628	2000	235.9	214.3	258.1
Chad	Mandoul	Nderguigui	65628	2005	221.3	201.8	241.7
Chad	Mandoul	Nderguigui	65628	2010	191.0	173.8	208.6
Chad	Mandoul	Nderguigui	65628	2015	151.0	134.5	169.5
Chad	Mandoul	Ngalo	65631	2000	209.0	189.4	230.7
Chad	Mandoul	Ngalo	65631	2005	181.6	166.0	198.9
Chad	Mandoul	Ngalo	65631	2010	149.8	135.5	165.7
Chad	Mandoul	Ngalo	65631	2015	128.7	113.1	145.0
Chad	Mandoul	Ngangara	65634	2000	197.9	178.4	218.7
Chad	Mandoul	Ngangara	65634	2005	182.6	165.0	201.7
Chad	Mandoul	Ngangara	65634	2010	148.6	134.8	164.6
Chad	Mandoul	Ngangara	65634	2015	130.5	115.2	146.7
Chad	Mandoul	Peni-tchad	65652	2000	225.2	205.5	246.5
Chad	Mandoul	Peni-tchad	65652	2005	208.0	191.0	225.4
Chad	Mandoul	Peni-tchad	65652	2010	174.6	159.7	190.0
Chad	Mandoul	Peni-tchad	65652	2015	139.6	124.2	156.4
Chad	Mandoul	Takoua	65674	2000	237.1	215.9	260.1
Chad	Mandoul	Takoua	65674	2005	208.6	190.8	228.0
Chad	Mandoul	Takoua	65674	2010	181.7	164.7	199.0
Chad	Mandoul	Takoua	65674	2015	151.6	134.0	169.1
Chad	Mandoul	Yomi	65695	2000	234.2	214.4	255.9
Chad	Mandoul	Yomi	65695	2005	219.5	201.9	237.5
Chad	Mandoul	Yomi	65695	2010	193.2	175.5	210.2
Chad	Mandoul	Yomi	65695	2015	156.0	138.4	174.3
Chad	Mayo-Boneye	Bongor	65416	2000	147.6	134.0	163.2
Chad	Mayo-Boneye	Bongor	65416	2005	140.6	127.8	154.0

Admin 0	Admin 1	Admin 2	GAUL Code	Year	Under-5 mortality (per 1,000 livebirths)		
					Estimate	Lower bound, 95% UI	Upper bound, 95% UI
Chad	Mayo-Boneye	Bongor	65416	2010	136.1	123.7	150.5
Chad	Mayo-Boneye	Bongor	65416	2015	119.5	105.5	134.9
Chad	Mayo-Boneye	Djoumane	65451	2000	177.6	159.4	196.7
Chad	Mayo-Boneye	Djoumane	65451	2005	168.6	153.5	186.9
Chad	Mayo-Boneye	Djoumane	65451	2010	156.3	141.2	172.9
Chad	Mayo-Boneye	Djoumane	65451	2015	129.0	114.6	145.7
Chad	Mayo-Boneye	Ere	65466	2000	188.4	170.8	206.8
Chad	Mayo-Boneye	Ere	65466	2005	180.5	165.1	197.6
Chad	Mayo-Boneye	Ere	65466	2010	166.1	151.1	181.8
Chad	Mayo-Boneye	Ere	65466	2015	133.8	119.9	150.0
Chad	Mayo-Boneye	Ham	65494	2000	167.9	149.9	186.7
Chad	Mayo-Boneye	Ham	65494	2005	158.0	143.2	174.8
Chad	Mayo-Boneye	Ham	65494	2010	150.8	136.1	168.3
Chad	Mayo-Boneye	Ham	65494	2015	126.0	111.1	143.7
Chad	Mayo-Boneye	Kim	65519	2000	191.1	171.5	211.3
Chad	Mayo-Boneye	Kim	65519	2005	182.7	165.2	202.3
Chad	Mayo-Boneye	Kim	65519	2010	163.2	148.1	180.7
Chad	Mayo-Boneye	Kim	65519	2015	135.3	120.8	152.4
Chad	Mayo-Boneye	Kolobo	65530	2000	175.0	156.6	194.5
Chad	Mayo-Boneye	Kolobo	65530	2005	166.9	151.9	185.3
Chad	Mayo-Boneye	Kolobo	65530	2010	157.2	141.9	174.3
Chad	Mayo-Boneye	Kolobo	65530	2015	130.8	115.6	148.6
Chad	Mayo-Boneye	Koumi	65544	2000	142.4	129.3	157.1
Chad	Mayo-Boneye	Koumi	65544	2005	129.0	117.6	140.8
Chad	Mayo-Boneye	Koumi	65544	2010	124.5	112.6	137.5
Chad	Mayo-Boneye	Koumi	65544	2015	115.8	102.6	131.2
Chad	Mayo-Boneye	Magao	65563	2000	148.2	133.5	164.3
Chad	Mayo-Boneye	Magao	65563	2005	138.9	124.5	153.2
Chad	Mayo-Boneye	Magao	65563	2010	132.9	119.4	147.5
Chad	Mayo-Boneye	Magao	65563	2015	120.9	106.5	138.7
Chad	Mayo-Boneye	Mitau	65601	2000	156.3	139.8	173.5
Chad	Mayo-Boneye	Mitau	65601	2005	152.3	137.1	168.4
Chad	Mayo-Boneye	Mitau	65601	2010	141.7	127.2	158.0
Chad	Mayo-Boneye	Mitau	65601	2015	127.2	112.6	144.0
Chad	Mayo-Boneye	Mogroum	65603	2000	154.0	138.7	170.2
Chad	Mayo-Boneye	Mogroum	65603	2005	141.9	128.6	157.6
Chad	Mayo-Boneye	Mogroum	65603	2010	132.8	119.2	148.2
Chad	Mayo-Boneye	Mogroum	65603	2015	123.0	107.8	140.5
Chad	Mayo-Boneye	Ngam	65632	2000	177.5	157.2	198.5
Chad	Mayo-Boneye	Ngam	65632	2005	169.9	150.4	191.9
Chad	Mayo-Boneye	Ngam	65632	2010	153.0	134.7	172.6
Chad	Mayo-Boneye	Ngam	65632	2015	135.5	118.1	155.2
Chad	Mayo-Boneye	Teleme	65681	2000	143.7	130.9	157.7
Chad	Mayo-Boneye	Teleme	65681	2005	132.6	120.8	146.2
Chad	Mayo-Boneye	Teleme	65681	2010	130.9	118.8	144.9
Chad	Mayo-Boneye	Teleme	65681	2015	115.0	101.6	130.7
Chad	Mayo-Boneye	Tougoude	65687	2000	165.5	148.8	182.6
Chad	Mayo-Boneye	Tougoude	65687	2005	158.4	143.6	174.3
Chad	Mayo-Boneye	Tougoude	65687	2010	150.1	136.6	166.3
Chad	Mayo-Boneye	Tougoude	65687	2015	130.5	115.5	147.8
Chad	Mayo-Boneye	Toura	65688	2000	155.4	139.4	172.4
Chad	Mayo-Boneye	Toura	65688	2005	149.7	134.8	166.3
Chad	Mayo-Boneye	Toura	65688	2010	142.0	127.3	158.6
Chad	Mayo-Boneye	Toura	65688	2015	127.1	111.9	145.1
Chad	Mayo-Dala	Binder	65405	2000	172.6	158.9	187.2
Chad	Mayo-Dala	Binder	65405	2005	159.6	146.4	173.6
Chad	Mayo-Dala	Binder	65405	2010	150.2	137.3	164.3
Chad	Mayo-Dala	Binder	65405	2015	123.1	109.6	138.1
Chad	Mayo-Dala	Bipare	65406	2000	190.1	175.9	205.8
Chad	Mayo-Dala	Bipare	65406	2005	168.9	155.8	183.9
Chad	Mayo-Dala	Bipare	65406	2010	143.6	130.9	157.3
Chad	Mayo-Dala	Bipare	65406	2015	123.8	108.8	139.3
Chad	Mayo-Dala	Dari	65435	2000	206.3	187.4	225.2
Chad	Mayo-Dala	Dari	65435	2005	189.7	173.5	206.5
Chad	Mayo-Dala	Dari	65435	2010	165.1	150.0	181.8
Chad	Mayo-Dala	Dari	65435	2015	140.0	124.9	156.3
Chad	Mayo-Dala	Doue	65460	2000	183.5	167.2	200.8
Chad	Mayo-Dala	Doue	65460	2005	172.4	157.3	188.1
Chad	Mayo-Dala	Doue	65460	2010	156.5	142.2	172.1
Chad	Mayo-Dala	Doue	65460	2015	128.8	115.1	144.1
Chad	Mayo-Dala	Erde-pala	65465	2000	168.5	153.5	183.4
Chad	Mayo-Dala	Erde-pala	65465	2005	161.6	148.5	175.9
Chad	Mayo-Dala	Erde-pala	65465	2010	150.1	137.2	165.0
Chad	Mayo-Dala	Erde-pala	65465	2015	125.2	112.2	139.4
Chad	Mayo-Dala	Gagal	65474	2000	192.2	174.8	209.6
Chad	Mayo-Dala	Gagal	65474	2005	187.0	172.3	203.4
Chad	Mayo-Dala	Gagal	65474	2010	171.8	157.8	188.3
Chad	Mayo-Dala	Gagal	65474	2015	141.7	127.0	156.1
Chad	Mayo-Dala	Gouey-goudoum	65481	2000	164.2	150.0	179.5
Chad	Mayo-Dala	Gouey-goudoum	65481	2005	160.2	146.5	174.0
Chad	Mayo-Dala	Gouey-goudoum	65481	2010	154.1	140.7	168.6
Chad	Mayo-Dala	Gouey-goudoum	65481	2015	126.2	112.8	140.7
Chad	Mayo-Dala	Gouin	65482	2000	163.7	149.0	179.4
Chad	Mayo-Dala	Gouin	65482	2005	156.2	142.3	170.2
Chad	Mayo-Dala	Gouin	65482	2010	150.6	136.7	166.0

Admin 0	Admin 1	Admin 2	GAUL Code	Year	Under-5 mortality (per 1,000 livebirths)		
					Estimate	Lower bound, 95% UI	Upper bound, 95% UI
Chad	Mayo-Dala	Gouin	65482	2015	123.7	110.5	138.9
Chad	Mayo-Dala	Goumadji	65484	2000	195.2	177.4	211.8
Chad	Mayo-Dala	Goumadji	65484	2005	183.6	167.9	198.9
Chad	Mayo-Dala	Goumadji	65484	2010	163.9	149.9	179.4
Chad	Mayo-Dala	Goumadji	65484	2015	137.8	122.5	153.2
Chad	Mayo-Dala	Guegou	65490	2000	186.9	172.6	202.1
Chad	Mayo-Dala	Guegou	65490	2005	170.3	156.9	185.2
Chad	Mayo-Dala	Guegou	65490	2010	144.3	131.6	157.7
Chad	Mayo-Dala	Guegou	65490	2015	122.6	107.7	137.2
Chad	Mayo-Dala	Keuni	65515	2000	210.5	190.8	231.1
Chad	Mayo-Dala	Keuni	65515	2005	203.7	185.7	225.1
Chad	Mayo-Dala	Keuni	65515	2010	181.0	164.1	200.3
Chad	Mayo-Dala	Keuni	65515	2015	147.7	130.7	165.9
Chad	Mayo-Dala	Korbo/kordo	65535	2000	198.5	179.7	219.2
Chad	Mayo-Dala	Korbo/kordo	65535	2005	200.3	183.0	220.0
Chad	Mayo-Dala	Korbo/kordo	65535	2010	184.1	166.6	203.4
Chad	Mayo-Dala	Korbo/kordo	65535	2015	149.2	131.9	166.5
Chad	Mayo-Dala	Lagon	65551	2000	177.5	162.0	193.0
Chad	Mayo-Dala	Lagon	65551	2005	164.6	151.4	178.5
Chad	Mayo-Dala	Lagon	65551	2010	147.8	135.5	160.8
Chad	Mayo-Dala	Lagon	65551	2015	122.6	109.1	136.8
Chad	Mayo-Dala	Lame	65553	2000	192.9	175.8	210.7
Chad	Mayo-Dala	Lame	65553	2005	178.5	162.3	194.3
Chad	Mayo-Dala	Lame	65553	2010	157.3	143.2	171.9
Chad	Mayo-Dala	Lame	65553	2015	128.8	115.1	143.9
Chad	Mayo-Dala	Lere-tchad	65557	2000	173.6	159.9	188.4
Chad	Mayo-Dala	Lere-tchad	65557	2005	158.9	146.7	172.7
Chad	Mayo-Dala	Lere-tchad	65557	2010	136.5	124.8	149.3
Chad	Mayo-Dala	Lere-tchad	65557	2015	117.9	104.7	132.5
Chad	Mayo-Dala	Salamata	65660	2000	208.2	186.9	229.8
Chad	Mayo-Dala	Salamata	65660	2005	195.0	176.8	216.1
Chad	Mayo-Dala	Salamata	65660	2010	173.0	155.8	191.9
Chad	Mayo-Dala	Salamata	65660	2015	143.5	126.3	161.3
Chad	Mayo-Dala	Tagobo-foulbe	65672	2000	194.2	176.4	212.2
Chad	Mayo-Dala	Tagobo-foulbe	65672	2005	178.9	162.8	195.8
Chad	Mayo-Dala	Tagobo-foulbe	65672	2010	156.6	141.8	171.5
Chad	Mayo-Dala	Tagobo-foulbe	65672	2015	128.0	113.9	143.6
Chad	Mayo-Dala	Torrok	65686	2000	160.6	145.9	176.5
Chad	Mayo-Dala	Torrok	65686	2005	155.5	141.3	169.8
Chad	Mayo-Dala	Torrok	65686	2010	150.5	136.8	165.4
Chad	Mayo-Dala	Torrok	65686	2015	123.3	109.8	138.6
Chad	Mont De Lam	Andoum	65359	2000	240.1	215.2	266.1
Chad	Mont De Lam	Andoum	65359	2005	228.7	206.8	252.5
Chad	Mont De Lam	Andoum	65359	2010	193.6	174.8	215.5
Chad	Mont De Lam	Andoum	65359	2015	160.3	141.6	180.8
Chad	Mont De Lam	Bessao	65400	2000	243.7	216.8	273.6
Chad	Mont De Lam	Bessao	65400	2005	225.6	199.8	252.1
Chad	Mont De Lam	Bessao	65400	2010	189.7	168.9	213.3
Chad	Mont De Lam	Bessao	65400	2015	161.4	140.2	183.3
Chad	Mont De Lam	Gadjibian	65473	2000	252.9	227.5	279.8
Chad	Mont De Lam	Gadjibian	65473	2005	242.1	218.3	268.1
Chad	Mont De Lam	Gadjibian	65473	2010	201.9	182.4	223.7
Chad	Mont De Lam	Gadjibian	65473	2015	167.9	149.2	188.3
Chad	Mont De Lam	Loumbogo	65559	2000	247.7	222.8	271.6
Chad	Mont De Lam	Loumbogo	65559	2005	227.0	207.6	249.7
Chad	Mont De Lam	Loumbogo	65559	2010	196.7	178.2	216.9
Chad	Mont De Lam	Loumbogo	65559	2015	163.6	145.2	184.6
Chad	Mont De Lam	Mbaissaye	65586	2000	231.3	204.8	258.7
Chad	Mont De Lam	Mbaissaye	65586	2005	212.0	188.6	238.4
Chad	Mont De Lam	Mbaissaye	65586	2010	177.1	156.3	200.5
Chad	Mont De Lam	Mbaissaye	65586	2015	153.2	131.6	175.7
Chad	Mont De Lam	Mbouroum	65591	2000	245.2	220.9	272.5
Chad	Mont De Lam	Mbouroum	65591	2005	226.9	204.8	251.7
Chad	Mont De Lam	Mbouroum	65591	2010	192.5	173.5	214.5
Chad	Mont De Lam	Mbouroum	65591	2015	161.0	141.7	182.1
Chad	Mont De Lam	Mont-de-lam	65609	2000	238.5	209.7	270.5
Chad	Mont De Lam	Mont-de-lam	65609	2005	219.7	192.5	249.9
Chad	Mont De Lam	Mont-de-lam	65609	2010	183.8	159.1	210.4
Chad	Mont De Lam	Mont-de-lam	65609	2015	161.2	136.5	186.7
Chad	Mont De Lam	Oudoumian	65645	2000	252.1	224.6	282.6
Chad	Mont De Lam	Oudoumian	65645	2005	229.3	204.2	255.3
Chad	Mont De Lam	Oudoumian	65645	2010	193.0	170.9	216.5
Chad	Mont De Lam	Oudoumian	65645	2015	165.4	143.2	189.3
Chad	Mont De Lam	Pandzangue	65650	2000	237.1	213.4	261.9
Chad	Mont De Lam	Pandzangue	65650	2005	226.9	207.2	248.9
Chad	Mont De Lam	Pandzangue	65650	2010	190.8	172.8	211.2
Chad	Mont De Lam	Pandzangue	65650	2015	160.0	142.4	179.8
Chad	Mont De Lam	Pao	65651	2000	242.1	217.7	268.5
Chad	Mont De Lam	Pao	65651	2005	223.7	201.6	247.1
Chad	Mont De Lam	Pao	65651	2010	186.9	166.8	210.1
Chad	Mont De Lam	Pao	65651	2015	159.4	139.0	181.1
Chad	Ouaddai	Abker	65351	2000	139.8	123.6	158.2
Chad	Ouaddai	Abker	65351	2005	120.2	107.2	135.4
Chad	Ouaddai	Abker	65351	2010	96.9	85.7	109.5
Chad	Ouaddai	Abker	65351	2015	83.9	73.1	97.3

Admin 0	Admin 1	Admin 2	GAUL Code	Year	Under-5 mortality (per 1,000 livebirths)		
					Estimate	Lower bound, 95% UI	Upper bound, 95% UI
Chad	Ouaddai	Bakhat	65365	2000	152.6	132.8	174.5
Chad	Ouaddai	Bakhat	65365	2005	129.3	113.9	146.8
Chad	Ouaddai	Bakhat	65365	2010	100.3	87.4	114.5
Chad	Ouaddai	Bakhat	65365	2015	90.5	78.1	105.1
Chad	Ouaddai	Bandala	65371	2000	145.6	127.4	167.5
Chad	Ouaddai	Bandala	65371	2005	123.1	107.4	141.3
Chad	Ouaddai	Bandala	65371	2010	97.5	84.6	111.8
Chad	Ouaddai	Bandala	65371	2015	84.8	72.7	98.4
Chad	Ouaddai	Birguit	65409	2000	165.3	144.1	189.3
Chad	Ouaddai	Birguit	65409	2005	143.9	125.2	165.1
Chad	Ouaddai	Birguit	65409	2010	111.0	96.6	127.3
Chad	Ouaddai	Birguit	65409	2015	96.6	83.4	113.4
Chad	Ouaddai	Bourtail	65422	2000	132.5	116.2	149.9
Chad	Ouaddai	Bourtail	65422	2005	113.7	100.1	128.8
Chad	Ouaddai	Bourtail	65422	2010	90.1	79.0	102.4
Chad	Ouaddai	Bourtail	65422	2015	77.8	66.8	89.9
Chad	Ouaddai	Dadjo	65427	2000	143.1	125.9	163.7
Chad	Ouaddai	Dadjo	65427	2005	120.6	105.9	137.4
Chad	Ouaddai	Dadjo	65427	2010	94.1	82.5	107.5
Chad	Ouaddai	Dadjo	65427	2015	84.1	72.2	98.4
Chad	Ouaddai	Guerri	65493	2000	133.4	117.2	151.1
Chad	Ouaddai	Guerri	65493	2005	115.0	101.2	129.9
Chad	Ouaddai	Guerri	65493	2010	91.9	80.2	103.9
Chad	Ouaddai	Guerri	65493	2015	81.3	70.2	93.3
Chad	Ouaddai	Kachimel-abeche	65501	2000	130.9	115.9	147.4
Chad	Ouaddai	Kachimel-abeche	65501	2005	111.3	98.8	125.1
Chad	Ouaddai	Kachimel-abeche	65501	2010	87.0	77.2	97.6
Chad	Ouaddai	Kachimel-abeche	65501	2015	75.0	64.6	86.0
Chad	Ouaddai	Kadjeske-am-dam	65503	2000	154.6	137.4	174.3
Chad	Ouaddai	Kadjeske-am-dam	65503	2005	133.5	118.8	149.5
Chad	Ouaddai	Kadjeske-am-dam	65503	2010	107.7	95.7	121.2
Chad	Ouaddai	Kadjeske-am-dam	65503	2015	93.0	81.0	106.3
Chad	Ouaddai	Kognere	65527	2000	141.4	124.6	160.7
Chad	Ouaddai	Kognere	65527	2005	120.0	106.1	135.6
Chad	Ouaddai	Kognere	65527	2010	95.1	83.7	108.0
Chad	Ouaddai	Kognere	65527	2015	82.1	70.5	95.1
Chad	Ouaddai	Korio	65537	2000	148.4	132.7	166.2
Chad	Ouaddai	Korio	65537	2005	127.8	114.6	142.4
Chad	Ouaddai	Korio	65537	2010	103.9	93.2	115.7
Chad	Ouaddai	Korio	65537	2015	88.8	78.0	101.5
Chad	Ouaddai	Mandjobo	65569	2000	141.0	125.6	157.8
Chad	Ouaddai	Mandjobo	65569	2005	120.0	107.3	133.5
Chad	Ouaddai	Mandjobo	65569	2010	94.0	84.4	104.9
Chad	Ouaddai	Mandjobo	65569	2015	77.6	67.7	88.4
Chad	Ouaddai	Marfa	65576	2000	142.3	126.6	160.3
Chad	Ouaddai	Marfa	65576	2005	120.7	106.8	135.4
Chad	Ouaddai	Marfa	65576	2010	96.4	85.3	109.1
Chad	Ouaddai	Marfa	65576	2015	81.7	70.6	93.5
Chad	Ouaddai	Massalat-am-dam	65580	2000	144.9	126.9	165.6
Chad	Ouaddai	Massalat-am-dam	65580	2005	121.0	106.4	138.2
Chad	Ouaddai	Massalat-am-dam	65580	2010	95.3	83.4	108.8
Chad	Ouaddai	Massalat-am-dam	65580	2015	84.9	73.1	97.8
Chad	Ouaddai	Ouadi-chok-abougouda	65641	2000	140.0	123.6	157.7
Chad	Ouaddai	Ouadi-chok-abougouda	65641	2005	117.7	103.7	131.7
Chad	Ouaddai	Ouadi-chok-abougouda	65641	2010	93.8	82.4	106.0
Chad	Ouaddai	Ouadi-chok-abougouda	65641	2015	81.2	70.4	93.3
Chad	Ouaddai	Ouadi-hamra	65643	2000	146.2	130.4	163.7
Chad	Ouaddai	Ouadi-hamra	65643	2005	127.4	113.7	141.9
Chad	Ouaddai	Ouadi-hamra	65643	2010	99.6	89.4	110.8
Chad	Ouaddai	Ouadi-hamra	65643	2015	83.6	72.8	95.3
Chad	Salamat	Am-timan	65356	2000	184.5	163.5	209.0
Chad	Salamat	Am-timan	65356	2005	165.4	147.3	186.6
Chad	Salamat	Am-timan	65356	2010	136.2	120.6	153.7
Chad	Salamat	Am-timan	65356	2015	128.4	111.2	149.1
Chad	Salamat	Autochtone	65361	2000	193.3	173.9	216.8
Chad	Salamat	Autochtone	65361	2005	176.4	157.4	196.4
Chad	Salamat	Autochtone	65361	2010	143.0	127.2	159.9
Chad	Salamat	Autochtone	65361	2015	122.8	107.3	141.0
Chad	Salamat	Barh-azoum-am-timan	65375	2000	195.4	169.3	222.9
Chad	Salamat	Barh-azoum-am-timan	65375	2005	177.2	155.3	200.6
Chad	Salamat	Barh-azoum-am-timan	65375	2010	151.0	132.4	172.6
Chad	Salamat	Barh-azoum-am-timan	65375	2015	135.7	116.5	157.0
Chad	Salamat	Dagal	65430	2000	180.6	161.0	202.1
Chad	Salamat	Dagal	65430	2005	161.7	145.5	179.9
Chad	Salamat	Dagal	65430	2010	129.5	116.0	145.2
Chad	Salamat	Dagal	65430	2015	115.6	101.4	130.4
Chad	Salamat	Dar-salim	65433	2000	199.8	173.1	230.6
Chad	Salamat	Dar-salim	65433	2005	181.9	156.5	210.7
Chad	Salamat	Dar-salim	65433	2010	151.3	131.4	175.5
Chad	Salamat	Dar-salim	65433	2015	142.0	120.9	168.6
Chad	Salamat	Doudeye	65459	2000	181.0	156.2	209.1
Chad	Salamat	Doudeye	65459	2005	164.6	140.5	191.1
Chad	Salamat	Doudeye	65459	2010	138.1	117.7	161.8
Chad	Salamat	Doudeye	65459	2015	120.0	100.7	140.8
Chad	Salamat	Hemat-am-timan	65495	2000	199.6	171.9	230.7

Admin 0	Admin 1	Admin 2	GAUL Code	Year	Under-5 mortality (per 1,000 livebirths)		
					Estimate	Lower bound, 95% UI	Upper bound, 95% UI
Chad	Salamat	Hemat-am-timan	65495	2005	179.3	155.0	206.2
Chad	Salamat	Hemat-am-timan	65495	2010	148.6	128.0	170.4
Chad	Salamat	Hemat-am-timan	65495	2015	137.1	117.2	160.5
Chad	Salamat	Hemat-haraze	65496	2000	199.4	169.0	233.1
Chad	Salamat	Hemat-haraze	65496	2005	182.7	154.4	212.8
Chad	Salamat	Hemat-haraze	65496	2010	150.7	127.8	177.8
Chad	Salamat	Hemat-haraze	65496	2015	139.3	116.8	164.8
Chad	Salamat	Kibet-angreb	65517	2000	189.3	169.3	212.6
Chad	Salamat	Kibet-angreb	65517	2005	172.8	155.8	192.8
Chad	Salamat	Kibet-angreb	65517	2010	141.6	127.1	158.7
Chad	Salamat	Kibet-angreb	65517	2015	128.6	113.1	146.5
Chad	Salamat	Kibet-soum-soum	65518	2000	188.1	166.6	212.2
Chad	Salamat	Kibet-soum-soum	65518	2005	170.6	153.2	190.2
Chad	Salamat	Kibet-soum-soum	65518	2010	138.8	123.8	156.4
Chad	Salamat	Kibet-soum-soum	65518	2015	124.5	108.3	143.5
Chad	Salamat	Kouga-tchad	65541	2000	195.2	169.3	222.9
Chad	Salamat	Kouga-tchad	65541	2005	178.5	155.1	206.8
Chad	Salamat	Kouga-tchad	65541	2010	147.2	126.5	170.5
Chad	Salamat	Kouga-tchad	65541	2015	138.1	118.0	163.3
Chad	Salamat	Mangueigne	65571	2000	208.8	179.8	242.8
Chad	Salamat	Mangueigne	65571	2005	190.0	162.4	219.7
Chad	Salamat	Mangueigne	65571	2010	154.7	133.4	180.4
Chad	Salamat	Mangueigne	65571	2015	145.5	123.2	172.6
Chad	Salamat	Rachid	65653	2000	196.0	174.4	221.3
Chad	Salamat	Rachid	65653	2005	176.7	156.7	198.0
Chad	Salamat	Rachid	65653	2010	145.1	128.5	162.6
Chad	Salamat	Rachid	65653	2015	123.8	107.7	141.9
Chad	Salamat	Salamat-am-timan	65659	2000	192.0	170.1	215.5
Chad	Salamat	Salamat-am-timan	65659	2005	171.1	152.2	190.6
Chad	Salamat	Salamat-am-timan	65659	2010	140.9	124.7	157.5
Chad	Salamat	Salamat-am-timan	65659	2015	128.2	111.8	145.6
Chad	Salamat	Torom	65685	2000	188.3	167.6	213.4
Chad	Salamat	Torom	65685	2005	170.5	152.0	191.7
Chad	Salamat	Torom	65685	2010	139.8	125.0	155.5
Chad	Salamat	Torom	65685	2015	122.4	105.3	141.5
Chad	Salamat	Yalnas-abou-deia	65691	2000	192.8	168.9	220.7
Chad	Salamat	Yalnas-abou-deia	65691	2005	176.5	153.2	203.1
Chad	Salamat	Yalnas-abou-deia	65691	2010	143.9	125.6	163.1
Chad	Salamat	Yalnas-abou-deia	65691	2015	124.2	106.0	144.6
Chad	Sila	Barh-azoum-goz-beida	65376	2000	170.4	148.1	193.1
Chad	Sila	Barh-azoum-goz-beida	65376	2005	150.3	130.7	171.3
Chad	Sila	Barh-azoum-goz-beida	65376	2010	127.1	110.9	144.5
Chad	Sila	Barh-azoum-goz-beida	65376	2015	105.3	89.7	122.5
Chad	Sila	Fongoro	65470	2000	168.6	146.0	193.7
Chad	Sila	Fongoro	65470	2005	149.3	128.7	172.0
Chad	Sila	Fongoro	65470	2010	127.6	109.1	149.0
Chad	Sila	Fongoro	65470	2015	107.7	90.5	127.0
Chad	Sila	Goz-beida	65489	2000	158.0	140.2	177.5
Chad	Sila	Goz-beida	65489	2005	134.5	118.9	151.1
Chad	Sila	Goz-beida	65489	2010	107.2	94.4	120.7
Chad	Sila	Goz-beida	65489	2015	96.1	82.1	110.7
Chad	Sila	Kadjeske-goz-beida	65504	2000	153.1	137.6	170.2
Chad	Sila	Kadjeske-goz-beida	65504	2005	130.5	117.7	144.7
Chad	Sila	Kadjeske-goz-beida	65504	2010	104.8	94.1	116.4
Chad	Sila	Kadjeske-goz-beida	65504	2015	90.9	79.6	102.1
Chad	Sila	Koloye	65533	2000	168.1	151.4	185.8
Chad	Sila	Koloye	65533	2005	148.6	134.8	163.3
Chad	Sila	Koloye	65533	2010	120.4	109.1	132.3
Chad	Sila	Koloye	65533	2015	97.0	84.9	109.3
Chad	Sila	Mourro	65619	2000	164.7	143.8	188.2
Chad	Sila	Mourro	65619	2005	147.8	129.8	168.9
Chad	Sila	Mourro	65619	2010	120.2	104.7	138.1
Chad	Sila	Mourro	65619	2015	103.9	88.4	121.1
Chad	Sila	Ouadi-habile	65642	2000	168.2	150.6	187.0
Chad	Sila	Ouadi-habile	65642	2005	145.5	131.3	162.1
Chad	Sila	Ouadi-habile	65642	2010	119.4	106.9	133.1
Chad	Sila	Ouadi-habile	65642	2015	99.7	87.0	112.3
Chad	Sila	Ouadi-kadja	65644	2000	171.9	155.1	191.3
Chad	Sila	Ouadi-kadja	65644	2005	153.8	138.4	171.1
Chad	Sila	Ouadi-kadja	65644	2010	127.9	114.7	142.8
Chad	Sila	Ouadi-kadja	65644	2015	101.3	87.8	114.9
Chad	Sila	Signar	65665	2000	164.2	145.3	185.3
Chad	Sila	Signar	65665	2005	148.9	131.3	169.0
Chad	Sila	Signar	65665	2010	125.9	110.9	143.6
Chad	Sila	Signar	65665	2015	98.5	84.7	112.8
Chad	Tandjile Est	Darbe	65434	2000	231.1	208.4	257.3
Chad	Tandjile Est	Darbe	65434	2005	214.2	192.7	238.3
Chad	Tandjile Est	Darbe	65434	2010	184.1	163.3	204.6
Chad	Tandjile Est	Darbe	65434	2015	145.2	127.8	164.9
Chad	Tandjile Est	Deressia	65441	2000	219.5	195.1	244.4
Chad	Tandjile Est	Deressia	65441	2005	207.8	185.8	232.4
Chad	Tandjile Est	Deressia	65441	2010	178.6	160.2	200.4
Chad	Tandjile Est	Deressia	65441	2015	136.9	120.5	155.1
Chad	Tandjile Est	Dormon	65457	2000	235.9	212.2	262.4
Chad	Tandjile Est	Dormon	65457	2005	222.3	198.5	247.4

Admin 0	Admin 1	Admin 2	GAUL Code	Year	Under-5 mortality (per 1,000 livebirths)		
					Estimate	Lower bound, 95% UI	Upper bound, 95% UI
Chad	Tandjile Est	Dormon	65457	2010	187.3	166.8	209.4
Chad	Tandjile Est	Dormon	65457	2015	140.5	123.5	159.8
Chad	Tandjile Est	Gabri-ngolo	65472	2000	240.6	219.2	266.2
Chad	Tandjile Est	Gabri-ngolo	65472	2005	229.7	206.7	252.8
Chad	Tandjile Est	Gabri-ngolo	65472	2010	193.9	175.6	213.9
Chad	Tandjile Est	Gabri-ngolo	65472	2015	141.8	126.2	159.4
Chad	Tandjile Est	Goulaye	65483	2000	227.2	205.3	252.9
Chad	Tandjile Est	Goulaye	65483	2005	204.9	184.8	227.0
Chad	Tandjile Est	Goulaye	65483	2010	178.4	159.7	198.0
Chad	Tandjile Est	Goulaye	65483	2015	144.9	126.1	164.5
Chad	Tandjile Est	Kabalaye	65500	2000	222.1	199.1	246.2
Chad	Tandjile Est	Kabalaye	65500	2005	212.1	190.8	234.5
Chad	Tandjile Est	Kabalaye	65500	2010	184.1	165.7	205.3
Chad	Tandjile Est	Kabalaye	65500	2015	136.0	120.9	154.5
Chad	Tandjile Est	Kariade-boum	65509	2000	237.5	215.6	261.3
Chad	Tandjile Est	Kariade-boum	65509	2005	220.5	200.7	240.7
Chad	Tandjile Est	Kariade-boum	65509	2010	190.0	171.3	208.8
Chad	Tandjile Est	Kariade-boum	65509	2015	148.8	131.2	166.5
Chad	Tandjile Est	Kimre	65520	2000	230.2	205.5	257.7
Chad	Tandjile Est	Kimre	65520	2005	206.8	184.7	232.8
Chad	Tandjile Est	Kimre	65520	2010	179.9	159.4	201.0
Chad	Tandjile Est	Kimre	65520	2015	144.9	126.2	164.7
Chad	Tandjile Est	Koro-tchad	65538	2000	238.1	217.5	260.7
Chad	Tandjile Est	Koro-tchad	65538	2005	223.9	204.7	242.1
Chad	Tandjile Est	Koro-tchad	65538	2010	195.0	176.9	212.3
Chad	Tandjile Est	Koro-tchad	65538	2015	150.6	132.8	168.3
Chad	Tandjile Est	Lai	65552	2000	233.9	212.3	257.3
Chad	Tandjile Est	Lai	65552	2005	219.1	198.2	242.1
Chad	Tandjile Est	Lai	65552	2010	176.0	158.8	194.0
Chad	Tandjile Est	Lai	65552	2015	134.3	119.5	152.3
Chad	Tandjile Est	Mouroum-touloum	65618	2000	240.0	218.9	264.2
Chad	Tandjile Est	Mouroum-touloum	65618	2005	225.4	204.1	246.2
Chad	Tandjile Est	Mouroum-touloum	65618	2010	194.2	175.6	212.7
Chad	Tandjile Est	Mouroum-touloum	65618	2015	148.0	131.0	165.1
Chad	Tandjile Est	Ndam	65627	2000	220.1	196.4	247.2
Chad	Tandjile Est	Ndam	65627	2005	205.1	183.7	228.7
Chad	Tandjile Est	Ndam	65627	2010	182.5	160.7	204.6
Chad	Tandjile Est	Ndam	65627	2015	158.7	137.6	180.1
Chad	Tandjile Est	Ngamongo	65633	2000	242.4	222.2	266.5
Chad	Tandjile Est	Ngamongo	65633	2005	228.4	208.2	248.9
Chad	Tandjile Est	Ngamongo	65633	2010	196.8	179.6	215.5
Chad	Tandjile Est	Ngamongo	65633	2015	147.6	131.8	164.4
Chad	Tandjile Est	Ninga	65638	2000	231.9	208.2	257.3
Chad	Tandjile Est	Ninga	65638	2005	220.7	198.1	245.8
Chad	Tandjile Est	Ninga	65638	2010	186.4	166.6	208.2
Chad	Tandjile Est	Ninga	65638	2015	138.4	121.6	158.4
Chad	Tandjile Est	Soumraye	65669	2000	227.1	201.3	255.4
Chad	Tandjile Est	Soumraye	65669	2005	210.3	186.4	236.3
Chad	Tandjile Est	Soumraye	65669	2010	183.8	163.3	206.8
Chad	Tandjile Est	Soumraye	65669	2015	147.7	129.1	168.1
Chad	Tandjile Est	Tchaguine-ngolo	65677	2000	212.2	186.5	238.8
Chad	Tandjile Est	Tchaguine-ngolo	65677	2005	199.2	173.4	225.7
Chad	Tandjile Est	Tchaguine-ngolo	65677	2010	179.7	157.1	203.2
Chad	Tandjile Est	Tchaguine-ngolo	65677	2015	150.3	130.4	172.9
Chad	Tandjile Est	Tchedoum	65679	2000	232.7	209.9	258.3
Chad	Tandjile Est	Tchedoum	65679	2005	217.1	194.8	240.3
Chad	Tandjile Est	Tchedoum	65679	2010	188.1	167.7	209.2
Chad	Tandjile Est	Tchedoum	65679	2015	147.4	128.6	166.9
Chad	Tandjile Ouest	Bagaye	65363	2000	216.5	198.1	236.1
Chad	Tandjile Ouest	Bagaye	65363	2005	207.7	190.4	226.1
Chad	Tandjile Ouest	Bagaye	65363	2010	192.8	176.0	209.9
Chad	Tandjile Ouest	Bagaye	65363	2015	147.9	132.2	164.7
Chad	Tandjile Ouest	Bere-tchad	65396	2000	235.7	215.9	257.4
Chad	Tandjile Ouest	Bere-tchad	65396	2005	226.9	208.7	246.9
Chad	Tandjile Ouest	Bere-tchad	65396	2010	197.1	179.0	214.9
Chad	Tandjile Ouest	Bere-tchad	65396	2015	143.5	127.8	160.1
Chad	Tandjile Ouest	Delbian	65437	2000	244.7	222.5	269.0
Chad	Tandjile Ouest	Delbian	65437	2005	237.0	215.8	259.9
Chad	Tandjile Ouest	Delbian	65437	2010	208.2	189.0	228.6
Chad	Tandjile Ouest	Delbian	65437	2015	155.6	137.5	175.1
Chad	Tandjile Ouest	Kelo	65512	2000	210.2	191.0	231.9
Chad	Tandjile Ouest	Kelo	65512	2005	199.7	182.8	218.6
Chad	Tandjile Ouest	Kelo	65512	2010	182.1	165.1	199.2
Chad	Tandjile Ouest	Kelo	65512	2015	139.5	123.4	156.1
Chad	Tandjile Ouest	Kolon	65531	2000	224.9	205.5	244.5
Chad	Tandjile Ouest	Kolon	65531	2005	216.3	199.3	234.8
Chad	Tandjile Ouest	Kolon	65531	2010	194.3	177.4	211.4
Chad	Tandjile Ouest	Kolon	65531	2015	145.9	130.6	162.7
Chad	Tandjile Ouest	Lele	65555	2000	227.2	208.6	246.0
Chad	Tandjile Ouest	Lele	65555	2005	224.1	207.5	242.3
Chad	Tandjile Ouest	Lele	65555	2010	210.2	193.3	227.0
Chad	Tandjile Ouest	Lele	65555	2015	161.0	144.8	179.1
Chad	Tandjile Ouest	Marba	65575	2000	206.6	189.2	224.7
Chad	Tandjile Ouest	Marba	65575	2005	197.5	182.8	214.1
Chad	Tandjile Ouest	Marba	65575	2010	189.7	174.9	205.4

Admin 0	Admin 1	Admin 2	GAUL Code	Year	Under-5 mortality (per 1,000 livebirths)		
					Estimate	Lower bound, 95% UI	Upper bound, 95% UI
Chad	Tandjile Ouest	Marba	65575	2015	146.8	131.2	163.3
Chad	Tandjile Ouest	Mesme	65594	2000	212.2	193.6	231.0
Chad	Tandjile Ouest	Mesme	65594	2005	209.2	193.2	226.6
Chad	Tandjile Ouest	Mesme	65594	2010	201.8	185.8	219.0
Chad	Tandjile Ouest	Mesme	65594	2015	159.6	143.2	177.6
Chad	Tandjile Ouest	Tamion-ngolo	65675	2000	243.1	220.5	267.1
Chad	Tandjile Ouest	Tamion-ngolo	65675	2005	234.8	213.8	256.7
Chad	Tandjile Ouest	Tamion-ngolo	65675	2010	199.5	180.8	219.2
Chad	Tandjile Ouest	Tamion-ngolo	65675	2015	144.9	128.6	163.0
Chad	Tandjile Ouest	Tchoa	65680	2000	233.8	215.7	253.1
Chad	Tandjile Ouest	Tchoa	65680	2005	229.0	212.2	247.4
Chad	Tandjile Ouest	Tchoa	65680	2010	211.6	194.1	229.4
Chad	Tandjile Ouest	Tchoa	65680	2015	161.0	144.2	178.4
Chad	Tibesti	Tibesti	65682	2000	129.9	106.2	157.2
Chad	Tibesti	Tibesti	65682	2005	107.8	86.7	131.5
Chad	Tibesti	Tibesti	65682	2010	92.9	76.1	113.7
Chad	Tibesti	Tibesti	65682	2015	72.9	59.2	88.9
Congo	Bouenza	Boko_Songho	14423	2000	137.2	123.0	151.8
Congo	Bouenza	Boko_Songho	14423	2005	113.3	101.3	125.6
Congo	Bouenza	Boko_Songho	14423	2010	82.3	73.0	92.4
Congo	Bouenza	Boko_Songho	14423	2015	67.3	58.2	77.3
Congo	Bouenza	Kingoue	190496	2000	124.2	111.4	139.8
Congo	Bouenza	Kingoue	190496	2005	98.7	88.0	109.6
Congo	Bouenza	Kingoue	190496	2010	74.0	64.6	83.4
Congo	Bouenza	Kingoue	190496	2015	60.0	51.6	69.5
Congo	Bouenza	Loudima	14424	2000	115.6	102.9	129.1
Congo	Bouenza	Loudima	14424	2005	101.7	90.4	113.8
Congo	Bouenza	Loudima	14424	2010	72.2	63.7	81.2
Congo	Bouenza	Loudima	14424	2015	60.8	52.4	69.9
Congo	Bouenza	Mabombo	190500	2000	128.5	116.5	141.4
Congo	Bouenza	Mabombo	190500	2005	105.6	96.4	116.3
Congo	Bouenza	Mabombo	190500	2010	77.5	69.5	86.6
Congo	Bouenza	Mabombo	190500	2015	63.3	55.5	72.7
Congo	Bouenza	Madingou	14425	2000	126.7	113.3	141.5
Congo	Bouenza	Madingou	14425	2005	104.2	93.5	116.0
Congo	Bouenza	Madingou	14425	2010	76.0	67.5	85.4
Congo	Bouenza	Madingou	14425	2015	62.8	54.3	72.7
Congo	Bouenza	Mfouati	14426	2000	127.0	113.5	140.5
Congo	Bouenza	Mfouati	14426	2005	102.7	92.2	114.1
Congo	Bouenza	Mfouati	14426	2010	74.1	65.8	83.1
Congo	Bouenza	Mfouati	14426	2015	61.8	53.5	70.9
Congo	Bouenza	Mouyondzi	190498	2000	127.0	113.6	141.6
Congo	Bouenza	Mouyondzi	190498	2005	101.5	91.2	113.3
Congo	Bouenza	Mouyondzi	190498	2010	74.5	65.3	83.7
Congo	Bouenza	Mouyondzi	190498	2015	62.2	53.8	72.1
Congo	Bouenza	Nkayi	14428	2000	120.6	109.7	132.4
Congo	Bouenza	Nkayi	14428	2005	103.8	94.5	113.4
Congo	Bouenza	Nkayi	14428	2010	75.0	67.2	83.0
Congo	Bouenza	Nkayi	14428	2015	63.0	54.9	71.6
Congo	Bouenza	Tsiaki	190495	2000	126.6	113.5	141.9
Congo	Bouenza	Tsiaki	190495	2005	101.6	90.6	112.6
Congo	Bouenza	Tsiaki	190495	2010	77.1	67.6	87.0
Congo	Bouenza	Tsiaki	190495	2015	62.2	53.8	71.7
Congo	Bouenza	Yamba	190499	2000	125.1	112.4	138.5
Congo	Bouenza	Yamba	190499	2005	100.0	90.0	110.1
Congo	Bouenza	Yamba	190499	2010	73.1	64.5	81.9
Congo	Bouenza	Yamba	190499	2015	60.0	52.1	69.2
Congo	Brazzaville	Brazzaville	190486	2000	96.6	90.1	103.6
Congo	Brazzaville	Brazzaville	190486	2005	82.6	76.6	88.9
Congo	Brazzaville	Brazzaville	190486	2010	65.9	59.6	72.2
Congo	Brazzaville	Brazzaville	190486	2015	50.9	44.7	58.2
Congo	Cuvette	Boundji	190455	2000	109.0	94.9	125.1
Congo	Cuvette	Boundji	190455	2005	96.9	84.3	110.4
Congo	Cuvette	Boundji	190455	2010	75.7	64.9	87.3
Congo	Cuvette	Boundji	190455	2015	63.0	53.0	73.2
Congo	Cuvette	Loukolela	190442	2000	98.4	85.1	114.3
Congo	Cuvette	Loukolela	190442	2005	90.4	78.2	104.0
Congo	Cuvette	Loukolela	190442	2010	73.4	62.8	85.5
Congo	Cuvette	Loukolela	190442	2015	60.8	51.3	70.6
Congo	Cuvette	Makoua	190446	2000	105.9	89.9	123.7
Congo	Cuvette	Makoua	190446	2005	96.3	82.5	111.7
Congo	Cuvette	Makoua	190446	2010	76.2	65.1	89.0
Congo	Cuvette	Makoua	190446	2015	64.3	53.8	76.7
Congo	Cuvette	Mossaka	14435	2000	100.7	87.0	115.3
Congo	Cuvette	Mossaka	14435	2005	89.2	77.0	101.7
Congo	Cuvette	Mossaka	14435	2010	70.1	59.9	80.5
Congo	Cuvette	Mossaka	14435	2015	56.1	47.8	65.8
Congo	Cuvette	Ngoko	190452	2000	109.7	95.2	125.4
Congo	Cuvette	Ngoko	190452	2005	98.5	85.5	111.9
Congo	Cuvette	Ngoko	190452	2010	77.2	66.9	88.6
Congo	Cuvette	Ngoko	190452	2015	64.7	55.2	75.5
Congo	Cuvette	Ntokou	190505	2000	110.7	94.3	129.4
Congo	Cuvette	Ntokou	190505	2005	99.3	85.6	115.8
Congo	Cuvette	Ntokou	190505	2010	79.0	67.1	92.2
Congo	Cuvette	Ntokou	190505	2015	66.3	56.1	77.2

Admin 0	Admin 1	Admin 2	GAUL Code	Year	Under-5 mortality (per 1,000 livebirths)		
					Estimate	Lower bound, 95% UI	Upper bound, 95% UI
Congo	Cuvette	Owando	190445	2000	103.3	89.9	118.4
Congo	Cuvette	Owando	190445	2005	92.9	81.2	106.2
Congo	Cuvette	Owando	190445	2010	73.2	63.2	83.2
Congo	Cuvette	Owando	190445	2015	60.6	51.1	70.6
Congo	Cuvette	Oyo	190457	2000	105.4	93.0	119.8
Congo	Cuvette	Oyo	190457	2005	93.8	83.0	106.6
Congo	Cuvette	Oyo	190457	2010	73.5	64.7	83.6
Congo	Cuvette	Oyo	190457	2015	60.7	51.7	70.3
Congo	Cuvette	Tchikapika	190483	2000	105.5	92.1	120.6
Congo	Cuvette	Tchikapika	190483	2005	92.5	80.8	105.4
Congo	Cuvette	Tchikapika	190483	2010	72.0	62.3	83.0
Congo	Cuvette	Tchikapika	190483	2015	58.9	50.1	69.4
Congo	Cuvette-Ouest	Etoumbi	190450	2000	109.6	95.8	125.3
Congo	Cuvette-Ouest	Etoumbi	190450	2005	99.2	87.0	112.4
Congo	Cuvette-Ouest	Etoumbi	190450	2010	79.3	69.4	91.2
Congo	Cuvette-Ouest	Etoumbi	190450	2015	65.6	56.3	76.0
Congo	Cuvette-Ouest	Ewo	190456	2000	102.2	89.8	115.7
Congo	Cuvette-Ouest	Ewo	190456	2005	92.6	81.5	104.3
Congo	Cuvette-Ouest	Ewo	190456	2010	73.3	64.1	83.8
Congo	Cuvette-Ouest	Ewo	190456	2015	60.9	52.5	70.7
Congo	Cuvette-Ouest	Kelle	190449	2000	109.0	94.4	124.5
Congo	Cuvette-Ouest	Kelle	190449	2005	98.4	85.4	111.9
Congo	Cuvette-Ouest	Kelle	190449	2010	80.5	69.3	92.4
Congo	Cuvette-Ouest	Kelle	190449	2015	67.4	57.6	78.3
Congo	Cuvette-Ouest	Mbama	190451	2000	108.2	94.8	122.7
Congo	Cuvette-Ouest	Mbama	190451	2005	98.0	86.3	110.3
Congo	Cuvette-Ouest	Mbama	190451	2010	77.1	67.9	88.3
Congo	Cuvette-Ouest	Mbama	190451	2015	64.2	55.2	74.1
Congo	Cuvette-Ouest	Mbomo	14431	2000	119.0	103.9	135.1
Congo	Cuvette-Ouest	Mbomo	14431	2005	106.4	93.9	121.1
Congo	Cuvette-Ouest	Mbomo	14431	2010	88.5	77.2	101.8
Congo	Cuvette-Ouest	Mbomo	14431	2015	77.2	66.9	89.8
Congo	Cuvette-Ouest	Okoyo	14436	2000	100.8	87.4	114.9
Congo	Cuvette-Ouest	Okoyo	14436	2005	88.1	77.1	100.0
Congo	Cuvette-Ouest	Okoyo	14436	2010	70.7	61.3	81.1
Congo	Cuvette-Ouest	Okoyo	14436	2015	59.0	49.8	69.3
Congo	Kouilou	Hinda	190474	2000	98.0	89.9	105.8
Congo	Kouilou	Hinda	190474	2005	82.2	75.0	89.8
Congo	Kouilou	Hinda	190474	2010	63.7	57.3	70.9
Congo	Kouilou	Hinda	190474	2015	56.8	49.8	64.3
Congo	Kouilou	Kakameoka	190471	2000	98.6	87.4	111.1
Congo	Kouilou	Kakameoka	190471	2005	84.4	74.6	95.1
Congo	Kouilou	Kakameoka	190471	2010	63.7	56.6	72.0
Congo	Kouilou	Kakameoka	190471	2015	56.6	48.6	65.3
Congo	Kouilou	Madingo_Kayes	190472	2000	89.3	78.4	101.6
Congo	Kouilou	Madingo_Kayes	190472	2005	78.1	68.9	88.8
Congo	Kouilou	Madingo_Kayes	190472	2010	60.2	52.9	68.8
Congo	Kouilou	Madingo_Kayes	190472	2015	53.5	46.0	62.0
Congo	Kouilou	Mvouti	14441	2000	110.4	99.6	123.3
Congo	Kouilou	Mvouti	14441	2005	93.8	84.3	104.7
Congo	Kouilou	Mvouti	14441	2010	69.4	62.1	78.7
Congo	Kouilou	Mvouti	14441	2015	61.8	54.1	71.1
Congo	Kouilou	Nzambi	190473	2000	79.1	67.0	93.2
Congo	Kouilou	Nzambi	190473	2005	70.6	60.3	82.9
Congo	Kouilou	Nzambi	190473	2010	56.0	47.2	65.6
Congo	Kouilou	Nzambi	190473	2015	50.3	41.9	59.6
Congo	Lekoumou	Bambama	14442	2000	99.0	85.5	114.1
Congo	Lekoumou	Bambama	14442	2005	83.6	71.9	96.3
Congo	Lekoumou	Bambama	14442	2010	67.6	58.0	78.3
Congo	Lekoumou	Bambama	14442	2015	57.9	49.0	68.1
Congo	Lekoumou	Komono	14443	2000	106.5	93.0	122.2
Congo	Lekoumou	Komono	14443	2005	91.0	80.1	103.4
Congo	Lekoumou	Komono	14443	2010	71.2	61.9	81.9
Congo	Lekoumou	Komono	14443	2015	59.5	50.6	69.4
Congo	Lekoumou	Mayeye	190494	2000	125.5	112.8	140.3
Congo	Lekoumou	Mayeye	190494	2005	103.0	91.9	114.1
Congo	Lekoumou	Mayeye	190494	2010	77.6	67.9	87.2
Congo	Lekoumou	Mayeye	190494	2015	62.4	53.6	72.0
Congo	Lekoumou	Sibiti	190493	2000	116.6	104.5	130.2
Congo	Lekoumou	Sibiti	190493	2005	98.4	87.5	110.6
Congo	Lekoumou	Sibiti	190493	2010	72.7	64.2	81.8
Congo	Lekoumou	Sibiti	190493	2015	60.5	51.9	69.7
Congo	Lekoumou	Zanaga	14445	2000	113.3	99.5	129.8
Congo	Lekoumou	Zanaga	14445	2005	92.7	80.9	104.8
Congo	Lekoumou	Zanaga	14445	2010	73.6	63.6	84.4
Congo	Lekoumou	Zanaga	14445	2015	60.3	51.0	70.8
Congo	Likouala	Betou	190437	2000	120.8	104.6	139.0
Congo	Likouala	Betou	190437	2005	105.3	91.3	121.6
Congo	Likouala	Betou	190437	2010	83.1	71.2	95.9
Congo	Likouala	Betou	190437	2015	69.1	58.4	80.5
Congo	Likouala	Bouanila	190443	2000	94.2	80.4	110.9
Congo	Likouala	Bouanila	190443	2005	88.4	75.4	104.0
Congo	Likouala	Bouanila	190443	2010	72.5	61.3	85.7
Congo	Likouala	Bouanila	190443	2015	59.0	49.2	70.1
Congo	Likouala	Dongou	190439	2000	102.1	90.1	115.5

Admin 0	Admin 1	Admin 2	GAUL Code	Year	Under-5 mortality (per 1,000 livebirths)		
					Estimate	Lower bound, 95% UI	Upper bound, 95% UI
Congo	Likouala	Dongou	190439	2005	92.5	81.2	104.4
Congo	Likouala	Dongou	190439	2010	74.6	65.0	85.2
Congo	Likouala	Dongou	190439	2015	65.1	55.7	75.9
Congo	Likouala	Enyelle	190438	2000	111.1	97.5	125.8
Congo	Likouala	Enyelle	190438	2005	97.4	85.7	109.6
Congo	Likouala	Enyelle	190438	2010	77.4	67.9	88.0
Congo	Likouala	Enyelle	190438	2015	69.7	59.4	80.0
Congo	Likouala	Epena	190509	2000	95.0	82.9	108.9
Congo	Likouala	Epena	190509	2005	89.4	78.2	102.8
Congo	Likouala	Epena	190509	2010	73.5	63.5	84.6
Congo	Likouala	Epena	190509	2015	61.1	52.1	71.1
Congo	Likouala	Impfondo	190440	2000	90.0	76.3	105.1
Congo	Likouala	Impfondo	190440	2005	83.0	70.8	97.1
Congo	Likouala	Impfondo	190440	2010	65.4	55.4	77.1
Congo	Likouala	Impfondo	190440	2015	49.1	41.2	58.6
Congo	Likouala	Liranga	190441	2000	93.2	79.5	108.1
Congo	Likouala	Liranga	190441	2005	87.1	74.4	101.3
Congo	Likouala	Liranga	190441	2010	70.5	60.0	82.1
Congo	Likouala	Liranga	190441	2015	57.2	48.1	67.4
Congo	Niari	Banba	190470	2000	85.5	73.4	99.6
Congo	Niari	Banba	190470	2005	77.4	67.1	90.1
Congo	Niari	Banba	190470	2010	58.5	50.3	68.3
Congo	Niari	Banba	190470	2015	51.0	43.9	59.7
Congo	Niari	Divenie	190465	2000	80.0	69.0	92.8
Congo	Niari	Divenie	190465	2005	71.1	61.0	82.4
Congo	Niari	Divenie	190465	2010	56.9	48.7	66.5
Congo	Niari	Divenie	190465	2015	50.5	42.6	59.0
Congo	Niari	Kibangou	190469	2000	83.7	72.4	97.1
Congo	Niari	Kibangou	190469	2005	75.2	65.1	87.2
Congo	Niari	Kibangou	190469	2010	57.6	49.3	67.1
Congo	Niari	Kibangou	190469	2015	49.4	42.3	57.3
Congo	Niari	Kimongo	190478	2000	130.3	118.0	144.9
Congo	Niari	Kimongo	190478	2005	111.9	101.4	124.8
Congo	Niari	Kimongo	190478	2010	81.5	73.0	91.2
Congo	Niari	Kimongo	190478	2015	69.2	60.4	78.6
Congo	Niari	Londela_Kayes	190479	2000	142.3	125.9	161.4
Congo	Niari	Londela_Kayes	190479	2005	117.6	103.9	132.7
Congo	Niari	Londela_Kayes	190479	2010	86.1	75.8	97.8
Congo	Niari	Londela_Kayes	190479	2015	71.5	61.2	82.7
Congo	Niari	Louvakou	190477	2000	99.2	88.9	110.4
Congo	Niari	Louvakou	190477	2005	89.4	79.8	99.0
Congo	Niari	Louvakou	190477	2010	65.0	57.7	73.2
Congo	Niari	Louvakou	190477	2015	55.9	48.4	64.8
Congo	Niari	Makabana	190468	2000	94.2	80.7	109.3
Congo	Niari	Makabana	190468	2005	83.5	71.7	97.3
Congo	Niari	Makabana	190468	2010	62.0	53.0	73.3
Congo	Niari	Makabana	190468	2015	53.3	45.2	63.0
Congo	Niari	Mayoko	190461	2000	88.4	75.6	103.2
Congo	Niari	Mayoko	190461	2005	78.7	67.0	91.4
Congo	Niari	Mayoko	190461	2010	63.4	53.4	74.2
Congo	Niari	Mayoko	190461	2015	54.7	45.8	63.9
Congo	Niari	Mbinda	190462	2000	85.1	72.0	99.9
Congo	Niari	Mbinda	190462	2005	75.9	64.2	89.7
Congo	Niari	Mbinda	190462	2010	61.3	51.0	72.6
Congo	Niari	Mbinda	190462	2015	53.3	44.5	63.7
Congo	Niari	Moungoundou_ Nord	190463	2000	83.5	70.8	98.6
Congo	Niari	Moungoundou_ Nord	190463	2005	75.1	63.5	88.1
Congo	Niari	Moungoundou_ Nord	190463	2010	61.1	51.3	72.5
Congo	Niari	Moungoundou_ Nord	190463	2015	53.5	44.3	63.4
Congo	Niari	Moungoundou_Sud	190464	2000	89.7	77.1	103.9
Congo	Niari	Moungoundou_Sud	190464	2005	79.2	68.1	92.2
Congo	Niari	Moungoundou_Sud	190464	2010	63.2	53.8	73.7
Congo	Niari	Moungoundou_Sud	190464	2015	54.3	45.1	63.7
Congo	Niari	Moutamba	190506	2000	89.4	74.5	105.7
Congo	Niari	Moutamba	190506	2005	78.7	65.9	93.9
Congo	Niari	Moutamba	190506	2010	60.7	50.3	72.7
Congo	Niari	Moutamba	190506	2015	50.9	42.2	61.3
Congo	Niari	Nyanga	190466	2000	76.1	65.4	88.6
Congo	Niari	Nyanga	190466	2005	68.0	58.5	79.1
Congo	Niari	Nyanga	190466	2010	54.2	46.3	63.0
Congo	Niari	Nyanga	190466	2015	47.7	40.3	56.5
Congo	Niari	Yaya	190467	2000	100.0	86.1	115.7
Congo	Niari	Yaya	190467	2005	87.4	75.4	100.9
Congo	Niari	Yaya	190467	2010	67.1	57.3	77.4
Congo	Niari	Yaya	190467	2015	57.6	48.6	67.7
Congo	Plateaux	Abala	190480	2000	107.6	94.8	122.1
Congo	Plateaux	Abala	190480	2005	93.4	82.7	105.1
Congo	Plateaux	Abala	190480	2010	73.7	64.7	84.2
Congo	Plateaux	Abala	190480	2015	61.2	52.6	71.1
Congo	Plateaux	Allembe	190458	2000	105.5	92.0	120.1
Congo	Plateaux	Allembe	190458	2005	92.4	80.8	105.1
Congo	Plateaux	Allembe	190458	2010	73.0	63.5	83.8
Congo	Plateaux	Allembe	190458	2015	60.7	51.8	70.8
Congo	Plateaux	Djambala	190460	2000	111.5	96.2	132.1
Congo	Plateaux	Djambala	190460	2005	90.3	77.2	106.0

Admin 0	Admin 1	Admin 2	GAUL Code	Year	Under-5 mortality (per 1,000 livebirths)		
					Estimate	Lower bound, 95% UI	Upper bound, 95% UI
Congo	Plateaux	Djambala	190460	2010	72.0	61.2	85.2
Congo	Plateaux	Djambala	190460	2015	59.2	49.4	70.7
Congo	Plateaux	Gamboma	190488	2000	107.0	93.4	121.2
Congo	Plateaux	Gamboma	190488	2005	91.1	79.8	103.2
Congo	Plateaux	Gamboma	190488	2010	71.3	61.8	81.4
Congo	Plateaux	Gamboma	190488	2015	58.9	50.0	69.0
Congo	Plateaux	Lekana	190459	2000	102.6	89.5	118.1
Congo	Plateaux	Lekana	190459	2005	84.1	73.1	96.8
Congo	Plateaux	Lekana	190459	2010	69.4	59.7	80.8
Congo	Plateaux	Lekana	190459	2015	57.9	49.4	68.2
Congo	Plateaux	Makotimpoko	190484	2000	104.9	91.2	120.0
Congo	Plateaux	Makotimpoko	190484	2005	90.3	78.3	102.8
Congo	Plateaux	Makotimpoko	190484	2010	71.0	60.9	81.7
Congo	Plateaux	Makotimpoko	190484	2015	56.8	47.9	66.9
Congo	Plateaux	Mbon	190453	2000	105.9	91.2	122.9
Congo	Plateaux	Mbon	190453	2005	89.3	77.1	103.2
Congo	Plateaux	Mbon	190453	2010	72.4	62.0	83.8
Congo	Plateaux	Mbon	190453	2015	60.1	50.8	70.4
Congo	Plateaux	Mpouya	190485	2000	107.5	92.0	126.5
Congo	Plateaux	Mpouya	190485	2005	91.5	77.8	106.2
Congo	Plateaux	Mpouya	190485	2010	72.1	61.4	85.1
Congo	Plateaux	Mpouya	190485	2015	58.7	49.2	68.9
Congo	Plateaux	Ngo	190454	2000	111.6	95.9	128.3
Congo	Plateaux	Ngo	190454	2005	93.2	80.2	107.1
Congo	Plateaux	Ngo	190454	2010	74.1	63.8	85.3
Congo	Plateaux	Ngo	190454	2015	61.1	52.0	70.9
Congo	Plateaux	Ollombo	190481	2000	108.6	94.8	123.8
Congo	Plateaux	Ollombo	190481	2005	94.1	83.3	107.2
Congo	Plateaux	Ollombo	190481	2010	73.2	64.0	83.6
Congo	Plateaux	Ollombo	190481	2015	60.6	51.7	70.9
Congo	Plateaux	Ongogni	190482	2000	110.1	96.2	124.1
Congo	Plateaux	Ongogni	190482	2005	94.2	83.4	106.3
Congo	Plateaux	Ongogni	190482	2010	72.9	63.6	82.8
Congo	Plateaux	Ongogni	190482	2015	60.2	51.3	70.4
Congo	Point-Noire	Pointe-Noire	190475	2000	93.2	85.1	101.7
Congo	Point-Noire	Pointe-Noire	190475	2005	76.5	69.7	83.9
Congo	Point-Noire	Pointe-Noire	190475	2010	61.6	55.2	68.6
Congo	Point-Noire	Pointe-Noire	190475	2015	55.0	47.8	62.6
Congo	Point-Noire	Tchiamba_Nzassi	190476	2000	102.7	93.9	112.0
Congo	Point-Noire	Tchiamba_Nzassi	190476	2005	87.0	79.0	95.8
Congo	Point-Noire	Tchiamba_Nzassi	190476	2010	67.4	60.1	75.4
Congo	Point-Noire	Tchiamba_Nzassi	190476	2015	60.0	51.9	69.0
Congo	Pool	Boko	190502	2000	125.3	110.6	141.8
Congo	Pool	Boko	190502	2005	101.3	90.0	114.2
Congo	Pool	Boko	190502	2010	77.4	68.0	87.8
Congo	Pool	Boko	190502	2015	60.0	51.8	69.0
Congo	Pool	Goma_Tsetse	190489	2000	115.6	107.5	124.5
Congo	Pool	Goma_Tsetse	190489	2005	93.4	86.2	100.8
Congo	Pool	Goma_Tsetse	190489	2010	71.2	64.2	78.4
Congo	Pool	Goma_Tsetse	190489	2015	55.2	48.3	62.9
Congo	Pool	Ignie	190487	2000	111.1	99.5	122.3
Congo	Pool	Ignie	190487	2005	92.0	83.1	101.7
Congo	Pool	Ignie	190487	2010	72.4	64.3	81.4
Congo	Pool	Ignie	190487	2015	58.2	50.3	67.0
Congo	Pool	Kimba	190492	2000	118.5	102.7	137.0
Congo	Pool	Kimba	190492	2005	95.6	82.4	109.7
Congo	Pool	Kimba	190492	2010	74.4	63.5	86.9
Congo	Pool	Kimba	190492	2015	60.1	50.6	71.2
Congo	Pool	Kindamba	190497	2000	120.1	105.1	137.0
Congo	Pool	Kindamba	190497	2005	94.7	82.4	108.5
Congo	Pool	Kindamba	190497	2010	71.7	62.0	82.7
Congo	Pool	Kindamba	190497	2015	57.4	49.0	67.8
Congo	Pool	Kinkala	190490	2000	122.8	110.0	136.8
Congo	Pool	Kinkala	190490	2005	96.2	85.9	106.9
Congo	Pool	Kinkala	190490	2010	72.0	63.7	80.9
Congo	Pool	Kinkala	190490	2015	56.8	49.1	65.3
Congo	Pool	Louingui	190501	2000	124.0	110.1	139.9
Congo	Pool	Louingui	190501	2005	98.3	87.4	110.1
Congo	Pool	Louingui	190501	2010	74.1	64.9	83.9
Congo	Pool	Louingui	190501	2015	57.7	49.9	66.5
Congo	Pool	Loumo	190503	2000	127.3	112.9	143.5
Congo	Pool	Loumo	190503	2005	101.8	90.8	113.9
Congo	Pool	Loumo	190503	2010	76.3	67.3	86.2
Congo	Pool	Loumo	190503	2015	59.2	51.0	68.6
Congo	Pool	Mayama	14462	2000	113.1	100.6	126.9
Congo	Pool	Mayama	14462	2005	91.0	80.6	101.8
Congo	Pool	Mayama	14462	2010	70.9	61.8	80.6
Congo	Pool	Mayama	14462	2015	56.8	49.0	65.6
Congo	Pool	Mbandza_Ndounga	190504	2000	123.3	111.3	135.6
Congo	Pool	Mbandza_Ndounga	190504	2005	99.2	89.8	109.0
Congo	Pool	Mbandza_Ndounga	190504	2010	75.2	67.4	83.4
Congo	Pool	Mbandza_Ndounga	190504	2015	59.4	51.7	67.5
Congo	Pool	Mindouli	14463	2000	122.7	110.1	137.0
Congo	Pool	Mindouli	14463	2005	97.1	86.7	108.9
Congo	Pool	Mindouli	14463	2010	71.7	63.1	81.3

Admin 0	Admin 1	Admin 2	GAUL Code	Year	Under-5 mortality (per 1,000 livebirths)		
					Estimate	Lower bound, 95% UI	Upper bound, 95% UI
Congo	Pool	Mindouli	14463	2015	58.6	50.7	67.7
Congo	Pool	Ngabe	14464	2000	110.6	94.8	126.9
Congo	Pool	Ngabe	14464	2005	92.5	79.8	106.1
Congo	Pool	Ngabe	14464	2010	72.3	61.8	84.7
Congo	Pool	Ngabe	14464	2015	59.2	49.6	69.4
Congo	Pool	Vindza	190491	2000	115.2	100.2	132.9
Congo	Pool	Vindza	190491	2005	92.6	79.6	106.2
Congo	Pool	Vindza	190491	2010	72.3	61.9	84.1
Congo	Pool	Vindza	190491	2015	58.8	50.5	69.7
Congo	Sangha	Mokeko	190508	2000	121.0	104.7	138.7
Congo	Sangha	Mokeko	190508	2005	112.1	97.3	128.1
Congo	Sangha	Mokeko	190508	2010	90.1	77.0	104.0
Congo	Sangha	Mokeko	190508	2015	74.0	62.1	86.9
Congo	Sangha	Ngbala	190448	2000	133.8	116.1	154.4
Congo	Sangha	Ngbala	190448	2005	120.9	104.9	138.9
Congo	Sangha	Ngbala	190448	2010	100.1	85.3	116.4
Congo	Sangha	Ngbala	190448	2015	88.2	73.8	103.4
Congo	Sangha	Ouesso	190507	2000	119.8	102.0	139.6
Congo	Sangha	Ouesso	190507	2005	111.8	95.3	130.3
Congo	Sangha	Ouesso	190507	2010	89.6	75.1	105.4
Congo	Sangha	Ouesso	190507	2015	71.9	59.2	86.3
Congo	Sangha	Pikounda	190444	2000	108.7	92.5	127.7
Congo	Sangha	Pikounda	190444	2005	99.5	85.2	116.8
Congo	Sangha	Pikounda	190444	2010	81.3	68.6	94.6
Congo	Sangha	Pikounda	190444	2015	67.9	57.1	80.0
Congo	Sangha	Sembe	190447	2000	132.4	115.7	149.3
Congo	Sangha	Sembe	190447	2005	118.7	104.9	134.4
Congo	Sangha	Sembe	190447	2010	98.4	86.0	113.4
Congo	Sangha	Sembe	190447	2015	86.0	73.9	100.4
Congo	Sangha	Souanke	14468	2000	128.8	113.6	145.1
Congo	Sangha	Souanke	14468	2005	116.2	102.7	130.8
Congo	Sangha	Souanke	14468	2010	98.2	86.5	112.3
Congo	Sangha	Souanke	14468	2015	88.4	75.7	101.7
Côte d'Ivoire	Bas Sassandra	Gbôkle	16851	2000	126.8	109.4	145.5
Côte d'Ivoire	Bas Sassandra	Gbôkle	16851	2005	119.9	103.6	138.0
Côte d'Ivoire	Bas Sassandra	Gbôkle	16851	2010	101.7	87.2	117.8
Côte d'Ivoire	Bas Sassandra	Gbôkle	16851	2015	93.1	78.5	109.8
Côte d'Ivoire	Bas Sassandra	Nawa	14900	2000	136.9	122.1	151.7
Côte d'Ivoire	Bas Sassandra	Nawa	14900	2005	121.0	108.7	135.3
Côte d'Ivoire	Bas Sassandra	Nawa	14900	2010	101.4	90.1	114.9
Côte d'Ivoire	Bas Sassandra	Nawa	14900	2015	91.4	79.7	106.3
Côte d'Ivoire	Bas Sassandra	San-pedro	16865	2000	130.8	117.4	145.1
Côte d'Ivoire	Bas Sassandra	San-pedro	16865	2005	116.9	104.4	131.1
Côte d'Ivoire	Bas Sassandra	San-pedro	16865	2010	96.1	85.9	108.5
Côte d'Ivoire	Bas Sassandra	San-pedro	16865	2015	85.5	75.0	99.0
Côte d'Ivoire	Comoe	Indenie-Djuablin	16859	2000	131.3	121.2	142.4
Côte d'Ivoire	Comoe	Indenie-Djuablin	16859	2005	114.0	105.0	124.9
Côte d'Ivoire	Comoe	Indenie-Djuablin	16859	2010	101.3	92.1	111.1
Côte d'Ivoire	Comoe	Indenie-Djuablin	16859	2015	79.8	70.3	89.7
Côte d'Ivoire	Comoe	Sud-comoe	16866	2000	126.5	113.3	140.6
Côte d'Ivoire	Comoe	Sud-comoe	16866	2005	116.0	103.9	128.2
Côte d'Ivoire	Comoe	Sud-comoe	16866	2010	97.4	86.9	109.7
Côte d'Ivoire	Comoe	Sud-comoe	16866	2015	80.2	69.7	91.7
Côte d'Ivoire	Denguele	Folon	16849	2000	190.3	174.1	209.3
Côte d'Ivoire	Denguele	Folon	16849	2005	181.4	164.3	201.0
Côte d'Ivoire	Denguele	Folon	16849	2010	162.5	146.5	180.3
Côte d'Ivoire	Denguele	Folon	16849	2015	137.3	120.7	157.5
Côte d'Ivoire	Denguele	Kabadougou	16860	2000	189.9	172.0	208.5
Côte d'Ivoire	Denguele	Kabadougou	16860	2005	175.8	159.3	193.4
Côte d'Ivoire	Denguele	Kabadougou	16860	2010	153.4	138.1	170.6
Côte d'Ivoire	Denguele	Kabadougou	16860	2015	127.8	111.8	146.4
Côte d'Ivoire	District autonome de Abidjan	Abidjan	14911	2000	99.2	88.9	110.5
Côte d'Ivoire	District autonome de Abidjan	Abidjan	14911	2005	102.8	92.7	114.2
Côte d'Ivoire	District autonome de Abidjan	Abidjan	14911	2010	90.8	80.5	101.7
Côte d'Ivoire	District autonome de Abidjan	Abidjan	14911	2015	71.4	61.4	82.3
Côte d'Ivoire	District autonome de Yamoussoukro	Yamoussoukro	14910	2000	116.3	103.3	129.8
Côte d'Ivoire	District autonome de Yamoussoukro	Yamoussoukro	14910	2005	100.6	89.3	112.9
Côte d'Ivoire	District autonome de Yamoussoukro	Yamoussoukro	14910	2010	89.8	78.7	102.2
Côte d'Ivoire	District autonome de Yamoussoukro	Yamoussoukro	14910	2015	77.9	66.9	89.5
Côte d'Ivoire	Gôh-Djiboua	Gôh	16852	2000	124.2	110.8	138.3
Côte d'Ivoire	Gôh-Djiboua	Gôh	16852	2005	108.7	98.1	121.5
Côte d'Ivoire	Gôh-Djiboua	Gôh	16852	2010	93.5	82.3	105.6
Côte d'Ivoire	Gôh-Djiboua	Gôh	16852	2015	84.0	73.2	96.5
Côte d'Ivoire	Gôh-Djiboua	Lôh-Djiboua	16861	2000	118.8	104.7	134.1
Côte d'Ivoire	Gôh-Djiboua	Lôh-Djiboua	16861	2005	111.8	99.8	126.0
Côte d'Ivoire	Gôh-Djiboua	Lôh-Djiboua	16861	2010	95.2	83.6	108.6
Côte d'Ivoire	Gôh-Djiboua	Lôh-Djiboua	16861	2015	84.7	72.5	98.6
Côte d'Ivoire	Lacs	Belier	14909	2000	123.5	110.1	138.5
Côte d'Ivoire	Lacs	Belier	14909	2005	110.7	98.7	124.8
Côte d'Ivoire	Lacs	Belier	14909	2010	96.7	84.7	110.1
Côte d'Ivoire	Lacs	Belier	14909	2015	84.8	73.0	99.2
Côte d'Ivoire	Lacs	Belier	16868	2000	127.2	114.7	140.7
Côte d'Ivoire	Lacs	Belier	16868	2005	116.0	103.9	128.9
Côte d'Ivoire	Lacs	Belier	16868	2010	102.4	91.0	115.1
Côte d'Ivoire	Lacs	Belier	16868	2015	89.0	77.7	101.6

Admin 0	Admin 1	Admin 2	GAUL Code	Year	Under-5 mortality (per 1,000 livebirths)		
					Estimate	Lower bound, 95% UI	Upper bound, 95% UI
Côte d'Ivoire	Lacs	Iffou	16858	2000	140.3	126.2	155.8
Côte d'Ivoire	Lacs	Iffou	16858	2005	130.9	117.4	146.2
Côte d'Ivoire	Lacs	Iffou	16858	2010	118.7	105.5	133.8
Côte d'Ivoire	Lacs	Iffou	16858	2015	94.2	81.7	107.8
Côte d'Ivoire	Lacs	N'Zi	16864	2000	135.8	122.2	149.9
Côte d'Ivoire	Lacs	N'Zi	16864	2005	124.1	111.2	137.4
Côte d'Ivoire	Lacs	N'Zi	16864	2010	107.6	95.4	121.6
Côte d'Ivoire	Lacs	N'Zi	16864	2015	91.7	79.7	105.5
Côte d'Ivoire	Lagunes	Agneby-Tiassa	16846	2000	123.5	109.2	137.5
Côte d'Ivoire	Lagunes	Agneby-Tiassa	16846	2005	118.6	105.6	132.4
Côte d'Ivoire	Lagunes	Agneby-Tiassa	16846	2010	99.5	87.7	112.9
Côte d'Ivoire	Lagunes	Agneby-Tiassa	16846	2015	82.3	71.0	95.0
Côte d'Ivoire	Lagunes	Grands ponts	16854	2000	121.1	105.4	137.3
Côte d'Ivoire	Lagunes	Grands ponts	16854	2005	118.6	103.6	135.0
Côte d'Ivoire	Lagunes	Grands ponts	16854	2010	100.9	87.6	115.9
Côte d'Ivoire	Lagunes	Grands ponts	16854	2015	85.1	72.5	99.8
Côte d'Ivoire	Lagunes	Me	16863	2000	130.8	117.2	145.2
Côte d'Ivoire	Lagunes	Me	16863	2005	120.3	108.5	133.1
Côte d'Ivoire	Lagunes	Me	16863	2010	101.2	90.5	112.7
Côte d'Ivoire	Lagunes	Me	16863	2015	82.4	72.3	94.2
Côte d'Ivoire	Montagnes	Cavally	16848	2000	170.4	155.9	185.5
Côte d'Ivoire	Montagnes	Cavally	16848	2005	138.9	127.0	151.9
Côte d'Ivoire	Montagnes	Cavally	16848	2010	116.0	104.2	128.1
Côte d'Ivoire	Montagnes	Cavally	16848	2015	101.6	89.3	115.8
Côte d'Ivoire	Montagnes	Guemon	16855	2000	167.7	152.3	184.9
Côte d'Ivoire	Montagnes	Guemon	16855	2005	136.5	123.7	150.0
Côte d'Ivoire	Montagnes	Guemon	16855	2010	118.3	105.3	132.4
Côte d'Ivoire	Montagnes	Guemon	16855	2015	107.1	92.9	123.0
Côte d'Ivoire	Montagnes	Tonkpi	16867	2000	170.2	155.6	186.6
Côte d'Ivoire	Montagnes	Tonkpi	16867	2005	134.8	122.7	148.0
Côte d'Ivoire	Montagnes	Tonkpi	16867	2010	111.7	100.0	124.4
Côte d'Ivoire	Montagnes	Tonkpi	16867	2015	96.0	83.5	109.3
Côte d'Ivoire	Sassandra-Marahoue	Haut-Sassandra	16857	2000	151.6	137.7	167.2
Côte d'Ivoire	Sassandra-Marahoue	Haut-Sassandra	16857	2005	128.2	116.6	140.1
Côte d'Ivoire	Sassandra-Marahoue	Haut-Sassandra	16857	2010	111.3	99.3	124.7
Côte d'Ivoire	Sassandra-Marahoue	Haut-Sassandra	16857	2015	100.6	88.4	115.2
Côte d'Ivoire	Sassandra-Marahoue	Marahoue	16862	2000	131.7	118.9	145.1
Côte d'Ivoire	Sassandra-Marahoue	Marahoue	16862	2005	113.9	103.0	125.2
Côte d'Ivoire	Sassandra-Marahoue	Marahoue	16862	2010	99.2	87.8	110.9
Côte d'Ivoire	Sassandra-Marahoue	Marahoue	16862	2015	88.7	77.3	101.2
Côte d'Ivoire	Savanes	Bagoue	16847	2000	206.4	188.7	224.8
Côte d'Ivoire	Savanes	Bagoue	16847	2005	193.0	176.4	211.3
Côte d'Ivoire	Savanes	Bagoue	16847	2010	171.9	154.8	189.7
Côte d'Ivoire	Savanes	Bagoue	16847	2015	144.6	126.8	164.3
Côte d'Ivoire	Savanes	Poro	14929	2000	190.8	175.1	207.0
Côte d'Ivoire	Savanes	Poro	14929	2005	184.3	168.7	200.2
Côte d'Ivoire	Savanes	Poro	14929	2010	164.3	148.1	180.8
Côte d'Ivoire	Savanes	Poro	14929	2015	135.1	119.3	153.1
Côte d'Ivoire	Savanes	Tchologo	14928	2000	181.8	168.1	197.3
Côte d'Ivoire	Savanes	Tchologo	14928	2005	174.9	160.9	189.6
Côte d'Ivoire	Savanes	Tchologo	14928	2010	158.7	145.2	174.0
Côte d'Ivoire	Savanes	Tchologo	14928	2015	130.0	116.6	145.5
Côte d'Ivoire	Vallee Du Bandama	Gbêke	16850	2000	117.1	105.8	128.6
Côte d'Ivoire	Vallee Du Bandama	Gbêke	16850	2005	106.4	96.0	117.0
Côte d'Ivoire	Vallee Du Bandama	Gbêke	16850	2010	95.2	84.9	106.3
Côte d'Ivoire	Vallee Du Bandama	Gbêke	16850	2015	80.0	69.4	91.0
Côte d'Ivoire	Vallee Du Bandama	Hambol	16856	2000	142.2	128.8	156.2
Côte d'Ivoire	Vallee Du Bandama	Hambol	16856	2005	133.9	121.2	146.7
Côte d'Ivoire	Vallee Du Bandama	Hambol	16856	2010	123.7	111.0	137.1
Côte d'Ivoire	Vallee Du Bandama	Hambol	16856	2015	104.9	92.6	118.5
Côte d'Ivoire	Woroba	Bafing	14943	2000	191.2	173.4	210.3
Côte d'Ivoire	Woroba	Bafing	14943	2005	159.6	144.3	175.4
Côte d'Ivoire	Woroba	Bafing	14943	2010	134.6	119.9	150.5
Côte d'Ivoire	Woroba	Bafing	14943	2015	111.2	97.5	126.6
Côte d'Ivoire	Woroba	Bere	14941	2000	175.7	159.5	192.0
Côte d'Ivoire	Woroba	Bere	14941	2005	158.8	145.2	173.9
Côte d'Ivoire	Woroba	Bere	14941	2010	138.2	124.4	153.5
Côte d'Ivoire	Woroba	Bere	14941	2015	113.2	99.5	128.9
Côte d'Ivoire	Woroba	Worodougou	14942	2000	180.7	164.2	198.3
Côte d'Ivoire	Woroba	Worodougou	14942	2005	157.3	142.7	172.0
Côte d'Ivoire	Woroba	Worodougou	14942	2010	131.6	117.2	147.4
Côte d'Ivoire	Woroba	Worodougou	14942	2015	104.9	91.3	119.1
Côte d'Ivoire	Zanzan	Bounkani	14945	2000	191.3	174.4	210.0
Côte d'Ivoire	Zanzan	Bounkani	14945	2005	178.8	163.0	195.8
Côte d'Ivoire	Zanzan	Bounkani	14945	2010	165.8	150.1	184.6
Côte d'Ivoire	Zanzan	Bounkani	14945	2015	133.5	117.8	151.3
Côte d'Ivoire	Zanzan	Gontougo	16853	2000	144.6	133.1	157.4
Côte d'Ivoire	Zanzan	Gontougo	16853	2005	132.7	122.1	145.5
Côte d'Ivoire	Zanzan	Gontougo	16853	2010	113.4	103.2	124.5
Côte d'Ivoire	Zanzan	Gontougo	16853	2015	90.9	80.3	102.5
Democratic Republic of the Congo	Bandundu	Bandundu	14959	2000	123.4	103.8	145.2
Democratic Republic of the Congo	Bandundu	Bandundu	14959	2005	108.1	92.1	126.8
Democratic Republic of the Congo	Bandundu	Bandundu	14959	2010	83.8	71.1	97.6
Democratic Republic of the Congo	Bandundu	Bandundu	14959	2015	61.6	51.2	73.7
Democratic Republic of the Congo	Bandundu	Kikwit	74357	2000	114.4	97.0	133.3

Admin 0	Admin 1	Admin 2	GAUL Code	Year	Under-5 mortality (per 1,000 livebirths)		
					Estimate	Lower bound, 95% UI	Upper bound, 95% UI
Democratic Republic of the Congo	Bandundu	Kikwit	74357	2005	103.4	87.9	121.4
Democratic Republic of the Congo	Bandundu	Kikwit	74357	2010	80.6	67.2	94.4
Democratic Republic of the Congo	Bandundu	Kikwit	74357	2015	61.8	51.2	74.7
Democratic Republic of the Congo	Bandundu	Kwango	14960	2000	124.7	113.5	138.2
Democratic Republic of the Congo	Bandundu	Kwango	14960	2005	106.0	96.5	116.3
Democratic Republic of the Congo	Bandundu	Kwango	14960	2010	85.2	76.9	93.8
Democratic Republic of the Congo	Bandundu	Kwango	14960	2015	65.4	58.2	73.7
Democratic Republic of the Congo	Bandundu	Kwilu	14961	2000	122.4	110.2	135.1
Democratic Republic of the Congo	Bandundu	Kwilu	14961	2005	111.1	101.1	122.2
Democratic Republic of the Congo	Bandundu	Kwilu	14961	2010	88.4	79.5	97.9
Democratic Republic of the Congo	Bandundu	Kwilu	14961	2015	64.3	57.1	72.0
Democratic Republic of the Congo	Bandundu	Maï-Ndombe	74361	2000	118.2	104.5	132.7
Democratic Republic of the Congo	Bandundu	Maï-Ndombe	74361	2005	106.9	94.7	118.7
Democratic Republic of the Congo	Bandundu	Maï-Ndombe	74361	2010	89.9	79.0	100.9
Democratic Republic of the Congo	Bandundu	Maï-Ndombe	74361	2015	70.1	61.4	79.9
Democratic Republic of the Congo	Bandundu	Plateaux	74363	2000	130.7	115.9	146.9
Democratic Republic of the Congo	Bandundu	Plateaux	74363	2005	112.9	99.4	127.5
Democratic Republic of the Congo	Bandundu	Plateaux	74363	2010	92.6	81.1	105.6
Democratic Republic of the Congo	Bandundu	Plateaux	74363	2015	68.5	58.5	79.2
Democratic Republic of the Congo	Bas-Congo	Bas-Fleuve	74352	2000	174.6	157.8	192.7
Democratic Republic of the Congo	Bas-Congo	Bas-Fleuve	74352	2005	136.0	123.0	149.4
Democratic Republic of the Congo	Bas-Congo	Bas-Fleuve	74352	2010	115.4	103.0	128.3
Democratic Republic of the Congo	Bas-Congo	Bas-Fleuve	74352	2015	87.3	76.0	99.9
Democratic Republic of the Congo	Bas-Congo	Boma	74353	2000	134.5	116.1	154.0
Democratic Republic of the Congo	Bas-Congo	Boma	74353	2005	105.0	91.2	120.4
Democratic Republic of the Congo	Bas-Congo	Boma	74353	2010	97.5	84.5	112.3
Democratic Republic of the Congo	Bas-Congo	Boma	74353	2015	71.6	60.8	83.4
Democratic Republic of the Congo	Bas-Congo	Cataractes	14965	2000	162.2	146.4	178.8
Democratic Republic of the Congo	Bas-Congo	Cataractes	14965	2005	127.7	115.4	141.2
Democratic Republic of the Congo	Bas-Congo	Cataractes	14965	2010	108.5	97.6	120.4
Democratic Republic of the Congo	Bas-Congo	Cataractes	14965	2015	76.0	66.3	86.5
Democratic Republic of the Congo	Bas-Congo	Lukaya	14966	2000	161.3	144.7	179.7
Democratic Republic of the Congo	Bas-Congo	Lukaya	14966	2005	129.8	115.8	144.6
Democratic Republic of the Congo	Bas-Congo	Lukaya	14966	2010	110.2	97.3	124.3
Democratic Republic of the Congo	Bas-Congo	Lukaya	14966	2015	81.2	70.3	93.2
Democratic Republic of the Congo	Bas-Congo	Matadi	14967	2000	123.2	106.2	143.0
Democratic Republic of the Congo	Bas-Congo	Matadi	14967	2005	103.8	89.0	120.1
Democratic Republic of the Congo	Bas-Congo	Matadi	14967	2010	96.3	81.2	112.2
Democratic Republic of the Congo	Bas-Congo	Matadi	14967	2015	67.7	56.5	80.5
Democratic Republic of the Congo	Equateur	Equateur	14968	2000	124.8	112.7	137.9
Democratic Republic of the Congo	Equateur	Equateur	14968	2005	114.2	103.4	125.5
Democratic Republic of the Congo	Equateur	Equateur	14968	2010	97.0	87.0	107.1
Democratic Republic of the Congo	Equateur	Equateur	14968	2015	72.2	63.8	80.7
Democratic Republic of the Congo	Equateur	Gbadolite	74355	2000	172.9	144.8	201.8
Democratic Republic of the Congo	Equateur	Gbadolite	74355	2005	154.3	129.5	180.9
Democratic Republic of the Congo	Equateur	Gbadolite	74355	2010	111.9	93.9	131.8
Democratic Republic of the Congo	Equateur	Gbadolite	74355	2015	102.0	83.6	122.2
Democratic Republic of the Congo	Equateur	Mbandaka	14969	2000	112.2	94.8	131.3
Democratic Republic of the Congo	Equateur	Mbandaka	14969	2005	104.2	87.8	121.5
Democratic Republic of the Congo	Equateur	Mbandaka	14969	2010	87.7	73.7	103.9
Democratic Republic of the Congo	Equateur	Mbandaka	14969	2015	64.1	53.2	77.9
Democratic Republic of the Congo	Equateur	Mongala	14970	2000	155.3	140.3	170.2
Democratic Republic of the Congo	Equateur	Mongala	14970	2005	136.8	124.0	150.5
Democratic Republic of the Congo	Equateur	Mongala	14970	2010	108.2	97.9	119.3
Democratic Republic of the Congo	Equateur	Mongala	14970	2015	78.6	70.0	88.1
Democratic Republic of the Congo	Equateur	Nord-Ubangi	14971	2000	175.9	158.3	193.7
Democratic Republic of the Congo	Equateur	Nord-Ubangi	14971	2005	156.7	142.4	173.3
Democratic Republic of the Congo	Equateur	Nord-Ubangi	14971	2010	117.9	106.0	131.0
Democratic Republic of the Congo	Equateur	Nord-Ubangi	14971	2015	102.7	90.6	116.6
Democratic Republic of the Congo	Equateur	Sud-Ubangi	14972	2000	181.6	163.9	200.3
Democratic Republic of the Congo	Equateur	Sud-Ubangi	14972	2005	155.1	140.7	171.5
Democratic Republic of the Congo	Equateur	Sud-Ubangi	14972	2010	123.2	110.3	136.8
Democratic Republic of the Congo	Equateur	Sud-Ubangi	14972	2015	95.5	83.5	108.3
Democratic Republic of the Congo	Equateur	Tshuapa	14973	2000	154.7	139.7	170.2
Democratic Republic of the Congo	Equateur	Tshuapa	14973	2005	142.9	129.8	157.1
Democratic Republic of the Congo	Equateur	Tshuapa	14973	2010	123.5	111.1	136.6
Democratic Republic of the Congo	Equateur	Tshuapa	14973	2015	94.3	84.0	106.3
Democratic Republic of the Congo	Equateur	Zongo	74365	2000	159.4	137.4	183.8
Democratic Republic of the Congo	Equateur	Zongo	74365	2005	135.7	117.3	156.8
Democratic Republic of the Congo	Equateur	Zongo	74365	2010	113.6	97.6	133.1
Democratic Republic of the Congo	Equateur	Zongo	74365	2015	98.6	83.2	119.4
Democratic Republic of the Congo	Kasai Occidental	Kananga	14974	2000	182.2	162.3	203.7
Democratic Republic of the Congo	Kasai Occidental	Kananga	14974	2005	158.2	141.1	176.5
Democratic Republic of the Congo	Kasai Occidental	Kananga	14974	2010	138.1	122.3	154.9
Democratic Republic of the Congo	Kasai Occidental	Kananga	14974	2015	119.7	103.2	137.3
Democratic Republic of the Congo	Kasai Occidental	Kasai	74364	2000	191.4	164.4	222.6
Democratic Republic of the Congo	Kasai Occidental	Kasai	74364	2005	168.8	146.4	196.2
Democratic Republic of the Congo	Kasai Occidental	Kasai	74364	2010	140.0	119.9	162.7
Democratic Republic of the Congo	Kasai Occidental	Kasai	74364	2015	117.0	97.9	137.2
Democratic Republic of the Congo	Kasai Occidental	Kasai	74367	2000	164.2	150.5	179.3
Democratic Republic of the Congo	Kasai Occidental	Kasai	74367	2005	143.6	132.5	156.7
Democratic Republic of the Congo	Kasai Occidental	Kasai	74367	2010	124.4	113.4	135.9
Democratic Republic of the Congo	Kasai Occidental	Kasai	74367	2015	99.9	89.0	111.9
Democratic Republic of the Congo	Kasai Occidental	Lulua	14976	2000	185.3	172.2	199.2
Democratic Republic of the Congo	Kasai Occidental	Lulua	14976	2005	162.3	150.9	173.3

Admin 0	Admin 1	Admin 2	GAUL Code	Year	Under-5 mortality (per 1,000 livebirths)		
					Estimate	Lower bound, 95% UI	Upper bound, 95% UI
Democratic Republic of the Congo	Kasai Occidental	Lulua	14976	2010	139.6	128.1	151.8
Democratic Republic of the Congo	Kasai Occidental	Lulua	14976	2015	119.7	107.4	133.4
Democratic Republic of the Congo	Kasai Oriental	Kabinda	74366	2000	145.4	131.9	159.3
Democratic Republic of the Congo	Kasai Oriental	Kabinda	74366	2005	131.6	120.4	143.4
Democratic Republic of the Congo	Kasai Oriental	Kabinda	74366	2010	114.8	104.2	126.0
Democratic Republic of the Congo	Kasai Oriental	Kabinda	74366	2015	102.6	91.2	115.1
Democratic Republic of the Congo	Kasai Oriental	Mbuji-Mayi	14978	2000	125.7	112.5	140.6
Democratic Republic of the Congo	Kasai Oriental	Mbuji-Mayi	14978	2005	115.9	104.7	127.8
Democratic Republic of the Congo	Kasai Oriental	Mbuji-Mayi	14978	2010	105.4	93.7	117.7
Democratic Republic of the Congo	Kasai Oriental	Mbuji-Mayi	14978	2015	80.2	69.6	91.9
Democratic Republic of the Congo	Kasai Oriental	Mwene-Ditu	74362	2000	136.7	119.0	155.7
Democratic Republic of the Congo	Kasai Oriental	Mwene-Ditu	74362	2005	123.3	109.2	139.9
Democratic Republic of the Congo	Kasai Oriental	Mwene-Ditu	74362	2010	105.7	92.4	120.0
Democratic Republic of the Congo	Kasai Oriental	Mwene-Ditu	74362	2015	95.1	81.2	111.3
Democratic Republic of the Congo	Kasai Oriental	Sankuru	14979	2000	141.1	126.2	157.3
Democratic Republic of the Congo	Kasai Oriental	Sankuru	14979	2005	130.5	117.3	144.7
Democratic Republic of the Congo	Kasai Oriental	Sankuru	14979	2010	119.8	106.4	134.5
Democratic Republic of the Congo	Kasai Oriental	Sankuru	14979	2015	94.6	83.9	107.3
Democratic Republic of the Congo	Kasai Oriental	Tshilenge	14980	2000	146.6	131.8	160.7
Democratic Republic of the Congo	Kasai Oriental	Tshilenge	14980	2005	132.8	121.3	144.6
Democratic Republic of the Congo	Kasai Oriental	Tshilenge	14980	2010	118.3	107.0	130.6
Democratic Republic of the Congo	Kasai Oriental	Tshilenge	14980	2015	97.1	84.9	110.8
Democratic Republic of the Congo	Katanga	Haut-Katanga	14982	2000	181.4	168.8	195.1
Democratic Republic of the Congo	Katanga	Haut-Katanga	14982	2005	150.4	140.0	161.5
Democratic Republic of the Congo	Katanga	Haut-Katanga	14982	2010	133.1	123.5	143.6
Democratic Republic of the Congo	Katanga	Haut-Katanga	14982	2015	107.9	98.3	118.0
Democratic Republic of the Congo	Katanga	Haut-Lomami	14981	2000	169.9	154.2	186.9
Democratic Republic of the Congo	Katanga	Haut-Lomami	14981	2005	150.0	136.4	165.5
Democratic Republic of the Congo	Katanga	Haut-Lomami	14981	2010	125.5	113.5	138.7
Democratic Republic of the Congo	Katanga	Haut-Lomami	14981	2015	109.2	97.6	122.7
Democratic Republic of the Congo	Katanga	Kolwezi	14983	2000	163.4	144.5	183.3
Democratic Republic of the Congo	Katanga	Kolwezi	14983	2005	137.9	122.4	155.8
Democratic Republic of the Congo	Katanga	Kolwezi	14983	2010	122.7	107.7	140.2
Democratic Republic of the Congo	Katanga	Kolwezi	14983	2015	99.4	85.8	115.3
Democratic Republic of the Congo	Katanga	Kolwezi	74359	2000	130.9	108.3	156.6
Democratic Republic of the Congo	Katanga	Kolwezi	74359	2005	113.3	94.1	135.8
Democratic Republic of the Congo	Katanga	Kolwezi	74359	2010	102.7	86.1	124.3
Democratic Republic of the Congo	Katanga	Kolwezi	74359	2015	80.5	66.6	98.8
Democratic Republic of the Congo	Katanga	Likasi	74360	2000	153.8	131.5	179.1
Democratic Republic of the Congo	Katanga	Likasi	74360	2005	131.1	111.3	154.0
Democratic Republic of the Congo	Katanga	Likasi	74360	2010	116.6	98.6	136.9
Democratic Republic of the Congo	Katanga	Likasi	74360	2015	84.7	69.6	100.9
Democratic Republic of the Congo	Katanga	Lualaba	14984	2000	148.9	132.0	166.1
Democratic Republic of the Congo	Katanga	Lualaba	14984	2005	127.9	113.7	143.6
Democratic Republic of the Congo	Katanga	Lualaba	14984	2010	105.6	92.9	119.4
Democratic Republic of the Congo	Katanga	Lualaba	14984	2015	93.0	80.3	105.8
Democratic Republic of the Congo	Katanga	Lubumbashi	14985	2000	125.2	110.0	141.6
Democratic Republic of the Congo	Katanga	Lubumbashi	14985	2005	95.7	84.5	108.6
Democratic Republic of the Congo	Katanga	Lubumbashi	14985	2010	91.7	79.3	105.4
Democratic Republic of the Congo	Katanga	Lubumbashi	14985	2015	67.0	57.0	77.7
Democratic Republic of the Congo	Katanga	Tanganyka	14986	2000	170.2	154.3	186.1
Democratic Republic of the Congo	Katanga	Tanganyka	14986	2005	149.6	136.3	163.1
Democratic Republic of the Congo	Katanga	Tanganyka	14986	2010	125.7	113.4	138.4
Democratic Republic of the Congo	Katanga	Tanganyka	14986	2015	113.3	102.0	126.4
Democratic Republic of the Congo	Kinshasa	Kinshasa	14987	2000	106.9	99.9	114.6
Democratic Republic of the Congo	Kinshasa	Kinshasa	14987	2005	90.9	84.6	97.4
Democratic Republic of the Congo	Kinshasa	Kinshasa	14987	2010	79.9	72.4	87.4
Democratic Republic of the Congo	Kinshasa	Kinshasa	14987	2015	55.0	48.1	62.4
Democratic Republic of the Congo	Maniema	Kindu	74358	2000	140.0	118.9	162.3
Democratic Republic of the Congo	Maniema	Kindu	74358	2005	113.9	97.4	132.3
Democratic Republic of the Congo	Maniema	Kindu	74358	2010	98.8	83.2	114.9
Democratic Republic of the Congo	Maniema	Kindu	74358	2015	90.5	75.9	107.9
Democratic Republic of the Congo	Maniema	Maniema	14988	2000	180.2	168.1	192.9
Democratic Republic of the Congo	Maniema	Maniema	14988	2005	150.0	140.5	160.8
Democratic Republic of the Congo	Maniema	Maniema	14988	2010	128.1	117.7	138.8
Democratic Republic of the Congo	Maniema	Maniema	14988	2015	116.7	105.2	128.7
Democratic Republic of the Congo	Nord-Kivu	Beni	115124	2000	116.6	104.0	130.6
Democratic Republic of the Congo	Nord-Kivu	Beni	115124	2005	90.3	80.1	101.2
Democratic Republic of the Congo	Nord-Kivu	Beni	115124	2010	75.5	66.1	85.3
Democratic Republic of the Congo	Nord-Kivu	Beni	115124	2015	61.3	52.8	70.2
Democratic Republic of the Congo	Nord-Kivu	Butembo	115125	2000	100.2	89.2	111.8
Democratic Republic of the Congo	Nord-Kivu	Butembo	115125	2005	75.7	67.7	84.4
Democratic Republic of the Congo	Nord-Kivu	Butembo	115125	2010	63.1	55.3	71.5
Democratic Republic of the Congo	Nord-Kivu	Butembo	115125	2015	47.3	40.9	54.8
Democratic Republic of the Congo	Nord-Kivu	Goma	74356	2000	125.8	117.5	134.6
Democratic Republic of the Congo	Nord-Kivu	Goma	74356	2005	94.0	87.7	100.7
Democratic Republic of the Congo	Nord-Kivu	Goma	74356	2010	70.1	64.2	75.6
Democratic Republic of the Congo	Nord-Kivu	Goma	74356	2015	53.3	48.1	59.0
Democratic Republic of the Congo	Nord-Kivu	Nord-Kivu	115126	2000	136.7	126.8	146.9
Democratic Republic of the Congo	Nord-Kivu	Nord-Kivu	115126	2005	102.4	95.0	109.8
Democratic Republic of the Congo	Nord-Kivu	Nord-Kivu	115126	2010	77.3	70.7	83.5
Democratic Republic of the Congo	Nord-Kivu	Nord-Kivu	115126	2015	58.0	52.5	63.9
Democratic Republic of the Congo	Orientale	Bas-Uele	14990	2000	169.1	150.6	189.3
Democratic Republic of the Congo	Orientale	Bas-Uele	14990	2005	147.9	133.4	164.8
Democratic Republic of the Congo	Orientale	Bas-Uele	14990	2010	121.5	107.9	136.3

Admin 0	Admin 1	Admin 2	GAUL Code	Year	Under-5 mortality (per 1,000 livebirths)		
					Estimate	Lower bound, 95% UI	Upper bound, 95% UI
Democratic Republic of the Congo	Orientale	Bas-Uele	14990	2015	102.5	90.7	116.5
Democratic Republic of the Congo	Orientale	Haut-Uele	14991	2000	152.4	133.2	174.0
Democratic Republic of the Congo	Orientale	Haut-Uele	14991	2005	129.7	113.7	147.0
Democratic Republic of the Congo	Orientale	Haut-Uele	14991	2010	110.5	95.8	126.9
Democratic Republic of the Congo	Orientale	Haut-Uele	14991	2015	90.8	78.5	105.3
Democratic Republic of the Congo	Orientale	Ituri	14992	2000	173.7	161.3	186.9
Democratic Republic of the Congo	Orientale	Ituri	14992	2005	148.4	138.2	160.2
Democratic Republic of the Congo	Orientale	Ituri	14992	2010	124.8	114.4	135.8
Democratic Republic of the Congo	Orientale	Ituri	14992	2015	102.5	92.0	114.6
Democratic Republic of the Congo	Orientale	Kisangani	14993	2000	156.8	134.3	182.4
Democratic Republic of the Congo	Orientale	Kisangani	14993	2005	135.3	115.3	157.6
Democratic Republic of the Congo	Orientale	Kisangani	14993	2010	117.8	99.5	137.5
Democratic Republic of the Congo	Orientale	Kisangani	14993	2015	89.6	75.3	106.0
Democratic Republic of the Congo	Orientale	Tshopo	14994	2000	159.6	144.5	175.3
Democratic Republic of the Congo	Orientale	Tshopo	14994	2005	138.0	125.1	151.5
Democratic Republic of the Congo	Orientale	Tshopo	14994	2010	117.7	106.4	130.4
Democratic Republic of the Congo	Orientale	Tshopo	14994	2015	92.4	81.9	103.5
Democratic Republic of the Congo	Sud-Kivu	Bukavu	74354	2000	139.4	129.1	149.7
Democratic Republic of the Congo	Sud-Kivu	Bukavu	74354	2005	103.8	96.6	111.7
Democratic Republic of the Congo	Sud-Kivu	Bukavu	74354	2010	82.7	76.3	89.7
Democratic Republic of the Congo	Sud-Kivu	Bukavu	74354	2015	66.2	59.2	73.5
Democratic Republic of the Congo	Sud-Kivu	Sud-Kivu	14995	2000	197.9	185.9	209.6
Democratic Republic of the Congo	Sud-Kivu	Sud-Kivu	14995	2005	152.6	143.7	162.3
Democratic Republic of the Congo	Sud-Kivu	Sud-Kivu	14995	2010	120.2	112.2	129.2
Democratic Republic of the Congo	Sud-Kivu	Sud-Kivu	14995	2015	94.2	86.1	102.3
Djibouti	Ali Sabieh	Ali Sabieh	15272	2000	89.0	80.5	98.4
Djibouti	Ali Sabieh	Ali Sabieh	15272	2005	76.7	68.1	86.1
Djibouti	Ali Sabieh	Ali Sabieh	15272	2010	62.0	53.5	71.1
Djibouti	Ali Sabieh	Ali Sabieh	15272	2015	48.0	40.0	56.9
Djibouti	Dikhil	As Eyla	15273	2000	80.9	71.0	91.2
Djibouti	Dikhil	As Eyla	15273	2005	72.4	63.2	82.1
Djibouti	Dikhil	As Eyla	15273	2010	63.2	54.2	72.9
Djibouti	Dikhil	As Eyla	15273	2015	47.1	39.1	55.4
Djibouti	Dikhil	Dikhil	15274	2000	76.8	68.5	85.8
Djibouti	Dikhil	Dikhil	15274	2005	67.4	59.6	75.9
Djibouti	Dikhil	Dikhil	15274	2010	58.6	50.5	67.4
Djibouti	Dikhil	Dikhil	15274	2015	45.2	37.8	53.5
Djibouti	Dikhil	Yoboki	15275	2000	89.8	80.2	100.3
Djibouti	Dikhil	Yoboki	15275	2005	80.3	70.4	89.9
Djibouti	Dikhil	Yoboki	15275	2010	70.1	60.5	80.6
Djibouti	Dikhil	Yoboki	15275	2015	54.8	46.1	64.3
Djibouti	Djibouti	Djibouti	15276	2000	107.7	97.7	118.4
Djibouti	Djibouti	Djibouti	15276	2005	88.4	78.7	99.7
Djibouti	Djibouti	Djibouti	15276	2010	69.0	59.1	79.9
Djibouti	Djibouti	Djibouti	15276	2015	54.2	45.2	64.2
Djibouti	Obock	Alaili Dadda	15277	2000	109.3	93.6	126.6
Djibouti	Obock	Alaili Dadda	15277	2005	94.6	80.0	111.1
Djibouti	Obock	Alaili Dadda	15277	2010	81.4	66.8	98.0
Djibouti	Obock	Alaili Dadda	15277	2015	62.6	50.7	76.8
Djibouti	Obock	Obock	15278	2000	108.2	93.6	124.8
Djibouti	Obock	Obock	15278	2005	93.2	79.6	108.8
Djibouti	Obock	Obock	15278	2010	78.8	65.7	93.3
Djibouti	Obock	Obock	15278	2015	60.9	50.0	74.3
Djibouti	Tadjourah	Balha	15279	2000	98.8	87.8	111.0
Djibouti	Tadjourah	Balha	15279	2005	86.4	75.7	97.7
Djibouti	Tadjourah	Balha	15279	2010	75.1	64.3	86.9
Djibouti	Tadjourah	Balha	15279	2015	58.8	49.4	69.6
Djibouti	Tadjourah	Dorra	15280	2000	106.8	93.9	122.0
Djibouti	Tadjourah	Dorra	15280	2005	91.8	79.7	106.3
Djibouti	Tadjourah	Dorra	15280	2010	80.7	68.2	95.0
Djibouti	Tadjourah	Dorra	15280	2015	62.5	52.1	75.1
Djibouti	Tadjourah	Randa	15281	2000	105.5	94.1	119.4
Djibouti	Tadjourah	Randa	15281	2005	89.8	78.8	102.4
Djibouti	Tadjourah	Randa	15281	2010	76.2	64.5	88.1
Djibouti	Tadjourah	Randa	15281	2015	60.0	50.2	71.4
Djibouti	Tadjourah	Tadjourah	15282	2000	97.9	88.3	109.3
Djibouti	Tadjourah	Tadjourah	15282	2005	84.0	74.0	94.7
Djibouti	Tadjourah	Tadjourah	15282	2010	71.2	60.8	82.2
Djibouti	Tadjourah	Tadjourah	15282	2015	54.5	45.9	65.3
Egypt	Alexandria	A L Labban	61537	2000	33.5	28.4	39.2
Egypt	Alexandria	A L Labban	61537	2005	29.8	25.2	34.5
Egypt	Alexandria	A L Labban	61537	2010	25.1	21.3	29.1
Egypt	Alexandria	A L Labban	61537	2015	18.9	15.6	22.4
Egypt	Alexandria	Al Amreia	61538	2000	34.5	29.4	39.8
Egypt	Alexandria	Al Amreia	61538	2005	30.3	25.7	34.8
Egypt	Alexandria	Al Amreia	61538	2010	25.2	21.4	28.9
Egypt	Alexandria	Al Amreia	61538	2015	18.5	15.5	21.7
Egypt	Alexandria	Al Attarin	61539	2000	33.5	28.4	39.2
Egypt	Alexandria	Al Attarin	61539	2005	29.8	25.2	34.5
Egypt	Alexandria	Al Attarin	61539	2010	25.1	21.3	29.1
Egypt	Alexandria	Al Attarin	61539	2015	18.9	15.6	22.4
Egypt	Alexandria	Al Dikhila	61540	2000	33.0	28.2	38.5
Egypt	Alexandria	Al Dikhila	61540	2005	30.0	25.4	34.8
Egypt	Alexandria	Al Dikhila	61540	2010	25.8	21.7	29.9
Egypt	Alexandria	Al Dikhila	61540	2015	18.8	15.6	22.1

Admin 0	Admin 1	Admin 2	GAUL Code	Year	Under-5 mortality (per 1,000 livebirths)		
					Estimate	Lower bound, 95% UI	Upper bound, 95% UI
Egypt	Alexandria	Al Gumruk	61541	2000	32.7	27.8	38.2
Egypt	Alexandria	Al Gumruk	61541	2005	29.7	25.1	34.5
Egypt	Alexandria	Al Gumruk	61541	2010	25.6	21.6	29.6
Egypt	Alexandria	Al Gumruk	61541	2015	18.6	15.4	22.1
Egypt	Alexandria	Al Manshiyya	61542	2000	33.5	28.4	39.2
Egypt	Alexandria	Al Manshiyya	61542	2005	29.8	25.2	34.5
Egypt	Alexandria	Al Manshiyya	61542	2010	25.1	21.3	29.1
Egypt	Alexandria	Al Manshiyya	61542	2015	18.9	15.6	22.4
Egypt	Alexandria	Al Raml	61543	2000	33.6	28.9	38.8
Egypt	Alexandria	Al Raml	61543	2005	29.4	25.1	33.7
Egypt	Alexandria	Al Raml	61543	2010	24.6	21.0	28.3
Egypt	Alexandria	Al Raml	61543	2015	18.0	15.0	21.3
Egypt	Alexandria	Bab Sharqi	61544	2000	33.9	29.0	39.3
Egypt	Alexandria	Bab Sharqi	61544	2005	29.8	25.4	34.3
Egypt	Alexandria	Bab Sharqi	61544	2010	25.0	21.3	28.8
Egypt	Alexandria	Bab Sharqi	61544	2015	18.2	15.0	21.4
Egypt	Alexandria	Burg Al-Arab City	61546	2000	39.1	32.7	45.8
Egypt	Alexandria	Burg Al-Arab City	61546	2005	33.2	28.0	38.9
Egypt	Alexandria	Burg Al-Arab City	61546	2010	26.5	22.1	31.0
Egypt	Alexandria	Burg Al-Arab City	61546	2015	19.3	15.9	22.8
Egypt	Alexandria	Burg al-Arab	61545	2000	41.0	34.3	48.5
Egypt	Alexandria	Burg al-Arab	61545	2005	34.2	28.5	40.3
Egypt	Alexandria	Burg al-Arab	61545	2010	27.5	22.7	32.5
Egypt	Alexandria	Burg al-Arab	61545	2015	19.9	16.2	23.8
Egypt	Alexandria	Karmuz	61547	2000	33.5	28.4	39.2
Egypt	Alexandria	Karmuz	61547	2005	29.8	25.2	34.5
Egypt	Alexandria	Karmuz	61547	2010	25.1	21.3	29.1
Egypt	Alexandria	Karmuz	61547	2015	18.9	15.6	22.4
Egypt	Alexandria	Kesm than Al Raml	61548	2000	34.8	30.1	39.9
Egypt	Alexandria	Kesm than Al Raml	61548	2005	29.7	25.6	34.0
Egypt	Alexandria	Kesm than Al Raml	61548	2010	24.5	21.0	28.1
Egypt	Alexandria	Kesm than Al Raml	61548	2015	17.9	15.0	21.1
Egypt	Alexandria	Mina Al-Basal	61549	2000	33.8	28.8	39.3
Egypt	Alexandria	Mina Al-Basal	61549	2005	30.0	25.4	34.6
Egypt	Alexandria	Mina Al-Basal	61549	2010	25.5	21.7	29.4
Egypt	Alexandria	Mina Al-Basal	61549	2015	18.4	15.4	21.8
Egypt	Alexandria	Muharam Bik	61550	2000	33.9	29.0	39.3
Egypt	Alexandria	Muharam Bik	61550	2005	29.8	25.4	34.3
Egypt	Alexandria	Muharam Bik	61550	2010	25.0	21.3	28.8
Egypt	Alexandria	Muharam Bik	61550	2015	18.2	15.0	21.4
Egypt	Alexandria	Muntazah	61551	2000	33.6	29.1	38.4
Egypt	Alexandria	Muntazah	61551	2005	29.3	25.1	33.4
Egypt	Alexandria	Muntazah	61551	2010	24.8	21.4	28.4
Egypt	Alexandria	Muntazah	61551	2015	18.1	15.2	21.3
Egypt	Alexandria	Port Alexandria Police Department	61553	2000	32.7	27.8	38.2
Egypt	Alexandria	Port Alexandria Police Department	61553	2005	29.7	25.1	34.5
Egypt	Alexandria	Port Alexandria Police Department	61553	2010	25.6	21.6	29.6
Egypt	Alexandria	Port Alexandria Police Department	61553	2015	18.6	15.4	22.1
Egypt	Alexandria	Sidi Gabir	61554	2000	33.6	28.9	38.8
Egypt	Alexandria	Sidi Gabir	61554	2005	29.4	25.1	33.7
Egypt	Alexandria	Sidi Gabir	61554	2010	24.6	21.0	28.3
Egypt	Alexandria	Sidi Gabir	61554	2015	18.0	15.0	21.3
Egypt	Alexandria	Zemam Out	61555	2000	41.7	34.6	49.3
Egypt	Alexandria	Zemam Out	61555	2005	34.4	28.6	40.7
Egypt	Alexandria	Zemam Out	61555	2010	27.7	22.9	32.9
Egypt	Alexandria	Zemam Out	61555	2015	20.0	16.2	23.9
Egypt	Assiut	Abnub	61556	2000	67.3	60.7	74.8
Egypt	Assiut	Abnub	61556	2005	49.0	43.8	54.9
Egypt	Assiut	Abnub	61556	2010	37.8	33.1	43.0
Egypt	Assiut	Abnub	61556	2015	27.4	23.2	31.9
Egypt	Assiut	Abu Tig	61557	2000	65.2	58.2	72.3
Egypt	Assiut	Abu Tig	61557	2005	48.0	42.8	53.8
Egypt	Assiut	Abu Tig	61557	2010	37.0	32.2	41.8
Egypt	Assiut	Abu Tig	61557	2015	27.8	23.5	32.5
Egypt	Assiut	Al- Badari	61558	2000	65.5	58.3	73.6
Egypt	Assiut	Al- Badari	61558	2005	48.3	42.8	54.3
Egypt	Assiut	Al- Badari	61558	2010	37.1	32.1	42.2
Egypt	Assiut	Al- Badari	61558	2015	27.9	23.6	32.8
Egypt	Assiut	Al-Ghanayem	61559	2000	64.8	57.6	72.5
Egypt	Assiut	Al-Ghanayem	61559	2005	48.7	43.3	54.8
Egypt	Assiut	Al-Ghanayem	61559	2010	38.3	33.2	43.8
Egypt	Assiut	Al-Ghanayem	61559	2015	28.5	24.2	33.5
Egypt	Assiut	Al-Qusia	61560	2000	67.0	59.6	74.9
Egypt	Assiut	Al-Qusia	61560	2005	49.3	43.9	55.1
Egypt	Assiut	Al-Qusia	61560	2010	37.8	33.2	43.0
Egypt	Assiut	Al-Qusia	61560	2015	27.3	23.1	32.1
Egypt	Assiut	Alfath	61561	2000	66.0	58.7	73.9
Egypt	Assiut	Alfath	61561	2005	48.5	43.1	54.5
Egypt	Assiut	Alfath	61561	2010	37.4	32.5	42.8
Egypt	Assiut	Alfath	61561	2015	27.4	23.1	32.0
Egypt	Assiut	Assuit	61562	2000	66.3	59.3	73.7
Egypt	Assiut	Assuit	61562	2005	48.7	43.3	54.4
Egypt	Assiut	Assuit	61562	2010	37.9	33.0	43.1
Egypt	Assiut	Assuit	61562	2015	27.5	23.2	32.0
Egypt	Assiut	Assuit City	61563	2000	66.8	59.1	75.0

Admin 0	Admin 1	Admin 2	GAUL Code	Year	Under-5 mortality (per 1,000 livebirths)		
					Estimate	Lower bound, 95% UI	Upper bound, 95% UI
Egypt	Assiut	Assuit City	61563	2005	50.4	44.6	56.8
Egypt	Assiut	Assuit City	61563	2010	39.5	34.3	44.9
Egypt	Assiut	Assuit City	61563	2015	27.2	23.0	32.0
Egypt	Assiut	Dayrut	61564	2000	66.9	60.2	74.1
Egypt	Assiut	Dayrut	61564	2005	49.3	44.1	54.8
Egypt	Assiut	Dayrut	61564	2010	37.1	32.7	42.1
Egypt	Assiut	Dayrut	61564	2015	26.9	22.9	31.4
Egypt	Assiut	Kesm Awal Assuit	61565	2000	65.0	58.0	72.8
Egypt	Assiut	Kesm Awal Assuit	61565	2005	48.6	43.1	54.6
Egypt	Assiut	Kesm Awal Assuit	61565	2010	38.0	33.0	43.2
Egypt	Assiut	Kesm Awal Assuit	61565	2015	27.5	23.2	32.4
Egypt	Assiut	Kesm Than Assuit	61566	2000	63.7	56.6	71.2
Egypt	Assiut	Kesm Than Assuit	61566	2005	47.2	42.0	53.1
Egypt	Assiut	Kesm Than Assuit	61566	2010	36.9	32.0	42.1
Egypt	Assiut	Kesm Than Assuit	61566	2015	27.1	22.8	31.8
Egypt	Assiut	Manfalut	61567	2000	66.6	59.4	73.9
Egypt	Assiut	Manfalut	61567	2005	48.7	43.5	54.2
Egypt	Assiut	Manfalut	61567	2010	37.6	33.1	42.6
Egypt	Assiut	Manfalut	61567	2015	27.2	23.2	31.8
Egypt	Assiut	Sahil Silim	61568	2000	66.3	59.3	74.2
Egypt	Assiut	Sahil Silim	61568	2005	48.8	43.5	54.3
Egypt	Assiut	Sahil Silim	61568	2010	37.6	32.8	42.4
Egypt	Assiut	Sahil Silim	61568	2015	27.8	23.4	32.5
Egypt	Assiut	Sidfa	61569	2000	64.9	57.4	72.8
Egypt	Assiut	Sidfa	61569	2005	47.7	42.4	54.0
Egypt	Assiut	Sidfa	61569	2010	36.5	31.4	41.7
Egypt	Assiut	Sidfa	61569	2015	27.8	23.5	32.7
Egypt	Assiut	Zemam Out	61570	2000	66.6	60.3	73.1
Egypt	Assiut	Zemam Out	61570	2005	49.6	44.9	54.8
Egypt	Assiut	Zemam Out	61570	2010	38.9	34.2	43.6
Egypt	Assiut	Zemam Out	61570	2015	27.8	23.9	31.8
Egypt	Aswan	Abu Simbel	61571	2000	60.9	47.4	77.9
Egypt	Aswan	Abu Simbel	61571	2005	55.2	42.7	69.6
Egypt	Aswan	Abu Simbel	61571	2010	45.3	34.7	57.4
Egypt	Aswan	Abu Simbel	61571	2015	32.4	24.5	41.9
Egypt	Aswan	Adfu	61572	2000	53.6	46.6	61.1
Egypt	Aswan	Adfu	61572	2005	50.4	43.8	57.6
Egypt	Aswan	Adfu	61572	2010	37.4	32.3	43.5
Egypt	Aswan	Adfu	61572	2015	26.1	22.0	30.8
Egypt	Aswan	Aswan	61573	2000	51.3	44.2	59.2
Egypt	Aswan	Aswan	61573	2005	48.0	41.0	56.0
Egypt	Aswan	Aswan	61573	2010	39.0	32.5	46.0
Egypt	Aswan	Aswan	61573	2015	28.4	23.1	34.6
Egypt	Aswan	Aswan City	61574	2000	54.7	46.8	63.5
Egypt	Aswan	Aswan City	61574	2005	49.3	41.7	58.1
Egypt	Aswan	Aswan City	61574	2010	40.6	33.9	47.9
Egypt	Aswan	Aswan City	61574	2015	28.2	23.0	34.2
Egypt	Aswan	Daraw	61575	2000	53.8	47.4	60.8
Egypt	Aswan	Daraw	61575	2005	50.8	44.6	57.4
Egypt	Aswan	Daraw	61575	2010	39.5	33.8	45.5
Egypt	Aswan	Daraw	61575	2015	27.3	23.2	32.1
Egypt	Aswan	Kum Umbu	61576	2000	52.1	45.6	59.4
Egypt	Aswan	Kum Umbu	61576	2005	48.3	41.9	55.3
Egypt	Aswan	Kum Umbu	61576	2010	37.2	31.8	43.3
Egypt	Aswan	Kum Umbu	61576	2015	26.9	22.5	31.6
Egypt	Aswan	Nasr	61577	2000	52.7	46.5	59.6
Egypt	Aswan	Nasr	61577	2005	49.2	43.1	55.9
Egypt	Aswan	Nasr	61577	2010	38.1	32.5	43.9
Egypt	Aswan	Nasr	61577	2015	27.3	23.1	32.2
Egypt	Aswan	Tushaka	61578	2000	59.3	45.8	75.2
Egypt	Aswan	Tushaka	61578	2005	55.1	42.5	70.8
Egypt	Aswan	Tushaka	61578	2010	45.4	35.3	58.2
Egypt	Aswan	Tushaka	61578	2015	32.6	24.9	41.7
Egypt	Aswan	Zemam Out	61579	2000	54.7	49.0	60.9
Egypt	Aswan	Zemam Out	61579	2005	50.1	44.3	55.9
Egypt	Aswan	Zemam Out	61579	2010	38.8	34.1	44.2
Egypt	Aswan	Zemam Out	61579	2015	27.6	23.8	32.1
Egypt	Behera	Abu Hummus	61581	2000	35.5	31.3	40.1
Egypt	Behera	Abu Hummus	61581	2005	27.8	24.4	31.6
Egypt	Behera	Abu Hummus	61581	2010	21.6	18.7	24.5
Egypt	Behera	Abu Hummus	61581	2015	16.8	14.4	19.6
Egypt	Behera	Abu-l-Matamir	61580	2000	37.3	32.2	43.0
Egypt	Behera	Abu-l-Matamir	61580	2005	29.7	25.6	34.3
Egypt	Behera	Abu-l-Matamir	61580	2010	23.0	19.7	26.4
Egypt	Behera	Abu-l-Matamir	61580	2015	17.7	14.8	20.7
Egypt	Behera	Al-Dilingat	61582	2000	36.6	32.3	41.2
Egypt	Behera	Al-Dilingat	61582	2005	27.7	24.4	31.3
Egypt	Behera	Al-Dilingat	61582	2010	21.2	18.6	24.3
Egypt	Behera	Al-Dilingat	61582	2015	16.6	14.2	19.3
Egypt	Behera	Al-Mahmudiyya	61583	2000	34.3	30.0	38.7
Egypt	Behera	Al-Mahmudiyya	61583	2005	26.7	23.7	30.0
Egypt	Behera	Al-Mahmudiyya	61583	2010	21.1	18.4	24.0
Egypt	Behera	Al-Mahmudiyya	61583	2015	16.3	14.0	18.8
Egypt	Behera	Al-Rahmaniyya	61584	2000	34.2	30.3	38.5
Egypt	Behera	Al-Rahmaniyya	61584	2005	26.5	23.6	29.5

Admin 0	Admin 1	Admin 2	GAUL Code	Year	Under-5 mortality (per 1,000 livebirths)		
					Estimate	Lower bound, 95% UI	Upper bound, 95% UI
Egypt	Behera	Al-Rahmaniyya	61584	2010	20.9	18.4	23.7
Egypt	Behera	Al-Rahmaniyya	61584	2015	16.0	13.8	18.6
Egypt	Behera	Badr	61585	2000	36.7	33.0	40.8
Egypt	Behera	Badr	61585	2005	27.9	24.8	31.3
Egypt	Behera	Badr	61585	2010	21.7	19.2	24.7
Egypt	Behera	Badr	61585	2015	16.8	14.5	19.5
Egypt	Behera	Damanhur	61586	2000	35.0	30.9	39.3
Egypt	Behera	Damanhur	61586	2005	27.0	23.8	30.3
Egypt	Behera	Damanhur	61586	2010	21.0	18.3	23.9
Egypt	Behera	Damanhur	61586	2015	16.3	14.0	18.8
Egypt	Behera	Hush Isa	61587	2000	36.8	32.1	42.0
Egypt	Behera	Hush Isa	61587	2005	28.4	24.8	32.4
Egypt	Behera	Hush Isa	61587	2010	21.8	19.0	25.0
Egypt	Behera	Hush Isa	61587	2015	16.9	14.4	19.8
Egypt	Behera	Idku	61588	2000	34.7	30.0	39.6
Egypt	Behera	Idku	61588	2005	27.9	24.2	31.7
Egypt	Behera	Idku	61588	2010	22.7	19.6	25.9
Egypt	Behera	Idku	61588	2015	17.0	14.3	19.8
Egypt	Behera	Itay Al-Barud	61589	2000	34.5	30.8	38.5
Egypt	Behera	Itay Al-Barud	61589	2005	26.5	23.7	29.6
Egypt	Behera	Itay Al-Barud	61589	2010	20.8	18.3	23.6
Egypt	Behera	Itay Al-Barud	61589	2015	16.1	13.8	18.6
Egypt	Behera	Kafr Al-Dawwar	61590	2000	35.3	30.9	40.1
Egypt	Behera	Kafr Al-Dawwar	61590	2005	28.8	25.2	32.7
Egypt	Behera	Kafr Al-Dawwar	61590	2010	23.0	20.0	26.1
Egypt	Behera	Kafr Al-Dawwar	61590	2015	17.4	14.8	20.2
Egypt	Behera	Kum Hamada	61591	2000	35.4	31.6	39.3
Egypt	Behera	Kum Hamada	61591	2005	27.0	24.3	30.2
Egypt	Behera	Kum Hamada	61591	2010	21.1	18.8	23.9
Egypt	Behera	Kum Hamada	61591	2015	16.4	14.2	19.0
Egypt	Behera	Nubariyya West	61592	2000	42.1	35.7	49.3
Egypt	Behera	Nubariyya West	61592	2005	34.0	28.7	39.9
Egypt	Behera	Nubariyya West	61592	2010	27.4	23.0	32.0
Egypt	Behera	Nubariyya West	61592	2015	19.8	16.4	23.7
Egypt	Behera	Rashid	61593	2000	34.4	29.9	39.1
Egypt	Behera	Rashid	61593	2005	28.0	24.5	31.6
Egypt	Behera	Rashid	61593	2010	22.9	19.9	26.2
Egypt	Behera	Rashid	61593	2015	17.1	14.4	19.9
Egypt	Behera	Shubra Khît	61594	2000	33.4	30.0	37.3
Egypt	Behera	Shubra Khît	61594	2005	26.0	23.3	29.0
Egypt	Behera	Shubra Khît	61594	2010	20.7	18.3	23.6
Egypt	Behera	Shubra Khît	61594	2015	16.0	13.7	18.4
Egypt	Behera	Wadi Al-NatrUn	61595	2000	40.1	33.8	47.3
Egypt	Behera	Wadi Al-NatrUn	61595	2005	32.1	26.8	37.9
Egypt	Behera	Wadi Al-NatrUn	61595	2010	25.7	21.6	30.4
Egypt	Behera	Wadi Al-NatrUn	61595	2015	18.9	15.6	22.8
Egypt	Behera	Zemam Out	61596	2000	41.1	35.2	47.3
Egypt	Behera	Zemam Out	61596	2005	32.6	27.9	37.7
Egypt	Behera	Zemam Out	61596	2010	26.4	22.4	30.9
Egypt	Behera	Zemam Out	61596	2015	19.1	16.1	22.8
Egypt	Beni Suef	Ahnasya	61597	2000	59.4	54.5	64.8
Egypt	Beni Suef	Ahnasya	61597	2005	44.5	40.6	48.9
Egypt	Beni Suef	Ahnasya	61597	2010	30.6	27.4	34.4
Egypt	Beni Suef	Ahnasya	61597	2015	25.3	21.9	28.8
Egypt	Beni Suef	Al Fashn	61598	2000	63.8	57.1	70.3
Egypt	Beni Suef	Al Fashn	61598	2005	48.3	42.9	53.7
Egypt	Beni Suef	Al Fashn	61598	2010	33.7	29.5	38.4
Egypt	Beni Suef	Al Fashn	61598	2015	25.1	21.4	29.3
Egypt	Beni Suef	Al Wasta	61599	2000	54.4	49.2	59.7
Egypt	Beni Suef	Al Wasta	61599	2005	40.6	36.8	44.9
Egypt	Beni Suef	Al Wasta	61599	2010	29.8	26.5	33.6
Egypt	Beni Suef	Al Wasta	61599	2015	25.6	22.1	29.6
Egypt	Beni Suef	Bani Swayf	61600	2000	59.0	53.3	65.6
Egypt	Beni Suef	Bani Swayf	61600	2005	45.2	40.8	50.1
Egypt	Beni Suef	Bani Swayf	61600	2010	32.0	28.1	36.2
Egypt	Beni Suef	Bani Swayf	61600	2015	26.5	22.7	30.5
Egypt	Beni Suef	Bani Swayf City	61601	2000	59.0	52.9	65.7
Egypt	Beni Suef	Bani Swayf City	61601	2005	47.0	42.0	52.3
Egypt	Beni Suef	Bani Swayf City	61601	2010	34.4	30.0	39.0
Egypt	Beni Suef	Bani Swayf City	61601	2015	26.6	22.7	30.7
Egypt	Beni Suef	Biba	61602	2000	61.2	55.4	67.4
Egypt	Beni Suef	Biba	61602	2005	46.6	42.2	51.3
Egypt	Beni Suef	Biba	61602	2010	32.8	29.0	36.8
Egypt	Beni Suef	Biba	61602	2015	25.8	22.3	29.7
Egypt	Beni Suef	Nasir	61603	2000	58.7	53.5	64.7
Egypt	Beni Suef	Nasir	61603	2005	44.0	39.9	48.4
Egypt	Beni Suef	Nasir	61603	2010	30.9	27.4	34.7
Egypt	Beni Suef	Nasir	61603	2015	26.3	22.7	30.3
Egypt	Beni Suef	Sumusta	61604	2000	61.9	56.4	67.7
Egypt	Beni Suef	Sumusta	61604	2005	46.7	42.3	51.4
Egypt	Beni Suef	Sumusta	61604	2010	32.4	28.7	36.4
Egypt	Beni Suef	Sumusta	61604	2015	24.8	21.5	28.6
Egypt	Beni Suef	Zemam Out	61605	2000	59.2	54.7	64.1
Egypt	Beni Suef	Zemam Out	61605	2005	44.7	40.9	48.6
Egypt	Beni Suef	Zemam Out	61605	2010	31.7	28.6	35.4

Admin 0	Admin 1	Admin 2	GAUL Code	Year	Under-5 mortality (per 1,000 livebirths)		
					Estimate	Lower bound, 95% UI	Upper bound, 95% UI
Egypt	Beni Suef	Zemam Out	61605	2015	25.4	22.2	29.0
Egypt	Cairo	15 Mayu	61606	2000	39.4	35.2	43.8
Egypt	Cairo	15 Mayu	61606	2005	33.7	30.1	37.3
Egypt	Cairo	15 Mayu	61606	2010	27.6	24.5	31.2
Egypt	Cairo	15 Mayu	61606	2015	21.1	18.2	24.4
Egypt	Cairo	Abdin	61607	2000	37.3	34.1	40.8
Egypt	Cairo	Abdin	61607	2005	32.4	29.5	35.5
Egypt	Cairo	Abdin	61607	2010	26.9	24.2	29.8
Egypt	Cairo	Abdin	61607	2015	20.3	17.8	23.3
Egypt	Cairo	Ain Shams	61608	2000	37.0	34.0	40.2
Egypt	Cairo	Ain Shams	61608	2005	31.6	29.0	34.6
Egypt	Cairo	Ain Shams	61608	2010	26.7	24.1	29.6
Egypt	Cairo	Ain Shams	61608	2015	20.1	17.6	22.9
Egypt	Cairo	Al Azbakiyya	61609	2000	37.3	34.1	40.8
Egypt	Cairo	Al Azbakiyya	61609	2005	32.4	29.5	35.5
Egypt	Cairo	Al Azbakiyya	61609	2010	26.9	24.2	29.8
Egypt	Cairo	Al Azbakiyya	61609	2015	20.3	17.8	23.3
Egypt	Cairo	Al Darb al-Ahmar	61610	2000	37.7	34.6	41.2
Egypt	Cairo	Al Darb al-Ahmar	61610	2005	33.1	30.1	36.1
Egypt	Cairo	Al Darb al-Ahmar	61610	2010	27.6	24.9	30.5
Egypt	Cairo	Al Darb al-Ahmar	61610	2015	21.4	18.7	24.4
Egypt	Cairo	Al Khalifa	61611	2000	37.5	34.4	41.0
Egypt	Cairo	Al Khalifa	61611	2005	33.0	30.1	36.1
Egypt	Cairo	Al Khalifa	61611	2010	27.6	25.0	30.6
Egypt	Cairo	Al Khalifa	61611	2015	21.3	18.7	24.3
Egypt	Cairo	Al Matariyya	61612	2000	37.6	34.5	40.7
Egypt	Cairo	Al Matariyya	61612	2005	31.6	28.9	34.6
Egypt	Cairo	Al Matariyya	61612	2010	26.7	24.1	29.5
Egypt	Cairo	Al Matariyya	61612	2015	19.9	17.5	22.7
Egypt	Cairo	Al Sahil	61613	2000	37.5	34.4	40.8
Egypt	Cairo	Al Sahil	61613	2005	31.8	28.9	34.8
Egypt	Cairo	Al Sahil	61613	2010	26.5	23.9	29.4
Egypt	Cairo	Al Sahil	61613	2015	19.9	17.4	22.7
Egypt	Cairo	Al Salam	61614	2000	36.6	33.6	39.8
Egypt	Cairo	Al Salam	61614	2005	31.8	29.1	34.7
Egypt	Cairo	Al Salam	61614	2010	26.7	24.1	29.6
Egypt	Cairo	Al Salam	61614	2015	20.2	17.7	23.0
Egypt	Cairo	Al Sharabiyya	61615	2000	37.4	34.2	40.7
Egypt	Cairo	Al Sharabiyya	61615	2005	32.5	29.6	35.6
Egypt	Cairo	Al Sharabiyya	61615	2010	27.0	24.3	29.8
Egypt	Cairo	Al Sharabiyya	61615	2015	20.8	18.2	23.8
Egypt	Cairo	Al Tibbin	61616	2000	40.8	36.5	45.6
Egypt	Cairo	Al Tibbin	61616	2005	34.1	30.3	38.0
Egypt	Cairo	Al Tibbin	61616	2010	27.8	24.4	31.5
Egypt	Cairo	Al Tibbin	61616	2015	21.3	18.4	24.7
Egypt	Cairo	Al Wayli	61617	2000	37.4	34.2	40.7
Egypt	Cairo	Al Wayli	61617	2005	32.5	29.6	35.6
Egypt	Cairo	Al Wayli	61617	2010	27.0	24.3	29.8
Egypt	Cairo	Al Wayli	61617	2015	20.8	18.2	23.8
Egypt	Cairo	Al Zahir	61618	2000	37.4	34.2	40.7
Egypt	Cairo	Al Zahir	61618	2005	32.5	29.6	35.6
Egypt	Cairo	Al Zahir	61618	2010	27.0	24.3	29.8
Egypt	Cairo	Al Zahir	61618	2015	20.8	18.2	23.8
Egypt	Cairo	Al Zaytun	61619	2000	37.1	33.9	40.3
Egypt	Cairo	Al Zaytun	61619	2005	31.9	29.1	35.0
Egypt	Cairo	Al Zaytun	61619	2010	26.9	24.2	29.8
Egypt	Cairo	Al Zaytun	61619	2015	20.7	18.1	23.6
Egypt	Cairo	Bab Al-Shariyya	61620	2000	37.4	34.2	40.7
Egypt	Cairo	Bab Al-Shariyya	61620	2005	32.5	29.6	35.6
Egypt	Cairo	Bab Al-Shariyya	61620	2010	27.0	24.3	29.8
Egypt	Cairo	Bab Al-Shariyya	61620	2015	20.8	18.2	23.8
Egypt	Cairo	Badr	61621	2000	35.5	31.9	39.8
Egypt	Cairo	Badr	61621	2005	32.2	28.6	36.2
Egypt	Cairo	Badr	61621	2010	27.6	24.3	31.5
Egypt	Cairo	Badr	61621	2015	21.2	18.3	24.7
Egypt	Cairo	Basatin	61622	2000	37.8	34.5	41.4
Egypt	Cairo	Basatin	61622	2005	33.1	30.1	36.2
Egypt	Cairo	Basatin	61622	2010	27.7	24.9	30.7
Egypt	Cairo	Basatin	61622	2015	21.3	18.5	24.3
Egypt	Cairo	Bulaq	61623	2000	37.3	34.1	40.8
Egypt	Cairo	Bulaq	61623	2005	32.4	29.5	35.5
Egypt	Cairo	Bulaq	61623	2010	26.9	24.2	29.8
Egypt	Cairo	Bulaq	61623	2015	20.3	17.8	23.3
Egypt	Cairo	Gamaliyya	61624	2000	37.4	34.2	40.7
Egypt	Cairo	Gamaliyya	61624	2005	32.5	29.6	35.6
Egypt	Cairo	Gamaliyya	61624	2010	27.0	24.3	29.8
Egypt	Cairo	Gamaliyya	61624	2015	20.8	18.2	23.8
Egypt	Cairo	Hadaiq Al-Qubba	61625	2000	37.2	34.1	40.4
Egypt	Cairo	Hadaiq Al-Qubba	61625	2005	32.1	29.2	35.2
Egypt	Cairo	Hadaiq Al-Qubba	61625	2010	26.5	23.9	29.1
Egypt	Cairo	Hadaiq Al-Qubba	61625	2015	20.3	17.8	23.1
Egypt	Cairo	Hilwan	61626	2000	39.1	35.2	43.3
Egypt	Cairo	Hilwan	61626	2005	33.4	29.9	36.9
Egypt	Cairo	Hilwan	61626	2010	27.4	24.4	30.9
Egypt	Cairo	Hilwan	61626	2015	21.0	18.2	24.2

Admin 0	Admin 1	Admin 2	GAUL Code	Year	Under-5 mortality (per 1,000 livebirths)		
					Estimate	Lower bound, 95% UI	Upper bound, 95% UI
Egypt	Cairo	Maadi	61627	2000	37.5	34.3	41.1
Egypt	Cairo	Maadi	61627	2005	33.0	30.0	36.2
Egypt	Cairo	Maadi	61627	2010	27.9	25.1	31.1
Egypt	Cairo	Maadi	61627	2015	21.6	18.9	24.7
Egypt	Cairo	Madinat Nasr-2	61628	2000	36.6	33.6	40.0
Egypt	Cairo	Madinat Nasr-2	61628	2005	32.2	29.3	35.3
Egypt	Cairo	Madinat Nasr-2	61628	2010	27.4	24.6	30.4
Egypt	Cairo	Madinat Nasr-2	61628	2015	21.2	18.5	24.2
Egypt	Cairo	Marg	61629	2000	37.0	34.0	40.2
Egypt	Cairo	Marg	61629	2005	31.6	29.0	34.6
Egypt	Cairo	Marg	61629	2010	26.7	24.1	29.6
Egypt	Cairo	Marg	61629	2015	20.1	17.6	22.9
Egypt	Cairo	Minshat Nasir	61630	2000	37.4	34.2	40.7
Egypt	Cairo	Minshat Nasir	61630	2005	32.5	29.6	35.6
Egypt	Cairo	Minshat Nasir	61630	2010	27.0	24.3	29.8
Egypt	Cairo	Minshat Nasir	61630	2015	20.8	18.2	23.8
Egypt	Cairo	Misr Al-Qadima	61632	2000	37.7	34.5	41.2
Egypt	Cairo	Misr Al-Qadima	61632	2005	32.7	29.7	35.8
Egypt	Cairo	Misr Al-Qadima	61632	2010	27.1	24.4	30.0
Egypt	Cairo	Misr Al-Qadima	61632	2015	20.5	17.9	23.5
Egypt	Cairo	Misr al-Gadida	61631	2000	37.1	33.9	40.3
Egypt	Cairo	Misr al-Gadida	61631	2005	31.9	29.1	35.0
Egypt	Cairo	Misr al-Gadida	61631	2010	26.9	24.2	29.8
Egypt	Cairo	Misr al-Gadida	61631	2015	20.7	18.1	23.6
Egypt	Cairo	Muski	61633	2000	37.4	34.2	40.7
Egypt	Cairo	Muski	61633	2005	32.5	29.6	35.6
Egypt	Cairo	Muski	61633	2010	27.0	24.3	29.8
Egypt	Cairo	Muski	61633	2015	20.8	18.2	23.8
Egypt	Cairo	Nasr City	61634	2000	36.7	33.6	40.3
Egypt	Cairo	Nasr City	61634	2005	32.5	29.6	35.7
Egypt	Cairo	Nasr City	61634	2010	27.8	24.9	31.0
Egypt	Cairo	Nasr City	61634	2015	21.3	18.6	24.3
Egypt	Cairo	New Cairo-1	61635	2000	36.7	33.2	40.5
Egypt	Cairo	New Cairo-1	61635	2005	32.7	29.6	36.2
Egypt	Cairo	New Cairo-1	61635	2010	27.7	24.6	31.4
Egypt	Cairo	New Cairo-1	61635	2015	21.5	18.7	24.7
Egypt	Cairo	New Cairo-2	61636	2000	36.2	32.8	39.9
Egypt	Cairo	New Cairo-2	61636	2005	32.3	29.2	35.7
Egypt	Cairo	New Cairo-2	61636	2010	27.7	24.6	31.3
Egypt	Cairo	New Cairo-2	61636	2015	21.3	18.5	24.5
Egypt	Cairo	New Cairo-3	61637	2000	37.3	34.0	41.0
Egypt	Cairo	New Cairo-3	61637	2005	33.1	30.0	36.4
Egypt	Cairo	New Cairo-3	61637	2010	28.1	25.0	31.5
Egypt	Cairo	New Cairo-3	61637	2015	21.9	19.0	25.2
Egypt	Cairo	Nuzha	61638	2000	36.5	33.4	39.7
Egypt	Cairo	Nuzha	61638	2005	32.0	29.1	35.0
Egypt	Cairo	Nuzha	61638	2010	27.2	24.4	30.2
Egypt	Cairo	Nuzha	61638	2015	20.5	17.9	23.3
Egypt	Cairo	Qasr Al-Nile	61639	2000	37.3	34.1	40.8
Egypt	Cairo	Qasr Al-Nile	61639	2005	32.4	29.5	35.5
Egypt	Cairo	Qasr Al-Nile	61639	2010	26.9	24.2	29.8
Egypt	Cairo	Qasr Al-Nile	61639	2015	20.3	17.8	23.3
Egypt	Cairo	Rud Al-Farag	61640	2000	37.3	34.1	40.8
Egypt	Cairo	Rud Al-Farag	61640	2005	32.4	29.5	35.5
Egypt	Cairo	Rud Al-Farag	61640	2010	26.9	24.2	29.8
Egypt	Cairo	Rud Al-Farag	61640	2015	20.3	17.8	23.3
Egypt	Cairo	Sayyida Zainab	61641	2000	37.7	34.5	41.2
Egypt	Cairo	Sayyida Zainab	61641	2005	32.7	29.7	35.8
Egypt	Cairo	Sayyida Zainab	61641	2010	27.1	24.4	30.0
Egypt	Cairo	Sayyida Zainab	61641	2015	20.5	17.9	23.5
Egypt	Cairo	Shroq	61642	2000	36.0	32.7	39.4
Egypt	Cairo	Shroq	61642	2005	31.8	28.9	35.2
Egypt	Cairo	Shroq	61642	2010	27.3	24.5	30.7
Egypt	Cairo	Shroq	61642	2015	20.9	18.2	24.1
Egypt	Cairo	Shubra	61643	2000	37.4	34.2	40.7
Egypt	Cairo	Shubra	61643	2005	32.5	29.6	35.6
Egypt	Cairo	Shubra	61643	2010	27.0	24.3	29.8
Egypt	Cairo	Shubra	61643	2015	20.8	18.2	23.8
Egypt	Cairo	Tura	61644	2000	38.1	34.6	41.9
Egypt	Cairo	Tura	61644	2005	33.4	30.3	36.6
Egypt	Cairo	Tura	61644	2010	27.9	25.1	31.2
Egypt	Cairo	Tura	61644	2015	21.0	18.2	24.1
Egypt	Cairo	Zamalik	61645	2000	37.3	34.1	40.8
Egypt	Cairo	Zamalik	61645	2005	32.4	29.5	35.5
Egypt	Cairo	Zamalik	61645	2010	26.9	24.2	29.8
Egypt	Cairo	Zamalik	61645	2015	20.3	17.8	23.3
Egypt	Cairo	Zawiyya Al-Hamra	61646	2000	37.2	34.1	40.4
Egypt	Cairo	Zawiyya Al-Hamra	61646	2005	32.1	29.2	35.2
Egypt	Cairo	Zawiyya Al-Hamra	61646	2010	26.5	23.9	29.1
Egypt	Cairo	Zawiyya Al-Hamra	61646	2015	20.3	17.8	23.1
Egypt	Cairo	Zemam Out	61647	2000	36.7	33.4	40.2
Egypt	Cairo	Zemam Out	61647	2005	32.0	29.1	35.1
Egypt	Cairo	Zemam Out	61647	2010	27.5	24.7	30.7
Egypt	Cairo	Zemam Out	61647	2015	20.5	18.0	23.5
Egypt	Dakahlia	Aga	61648	2000	32.7	29.9	35.5

Admin 0	Admin 1	Admin 2	GAUL Code	Year	Under-5 mortality (per 1,000 livebirths) Estimate	Lower bound, 95% UI	Upper bound, 95% UI
Egypt	Dakahlia	Aga	61648	2005	26.4	23.9	28.8
Egypt	Dakahlia	Aga	61648	2010	22.3	20.0	25.0
Egypt	Dakahlia	Aga	61648	2015	16.9	14.7	19.4
Egypt	Dakahlia	Bany Abeed	61649	2000	32.3	29.2	35.6
Egypt	Dakahlia	Bany Abeed	61649	2005	26.2	23.5	28.8
Egypt	Dakahlia	Bany Abeed	61649	2010	22.8	20.1	25.5
Egypt	Dakahlia	Bany Abeed	61649	2015	17.3	14.9	19.8
Egypt	Dakahlia	Bilqas	61650	2000	31.3	28.1	34.7
Egypt	Dakahlia	Bilqas	61650	2005	25.4	22.6	28.4
Egypt	Dakahlia	Bilqas	61650	2010	21.9	19.3	24.7
Egypt	Dakahlia	Bilqas	61650	2015	16.3	14.0	19.1
Egypt	Dakahlia	Dikirnis	61651	2000	31.6	28.5	34.8
Egypt	Dakahlia	Dikirnis	61651	2005	25.7	22.9	28.4
Egypt	Dakahlia	Dikirnis	61651	2010	22.4	19.7	25.2
Egypt	Dakahlia	Dikirnis	61651	2015	16.8	14.5	19.3
Egypt	Dakahlia	E Mansora 2	61652	2000	32.1	28.9	35.3
Egypt	Dakahlia	E Mansora 2	61652	2005	26.3	23.6	29.3
Egypt	Dakahlia	E Mansora 2	61652	2010	22.9	20.3	25.9
Egypt	Dakahlia	E Mansora 2	61652	2015	16.8	14.5	19.5
Egypt	Dakahlia	El Mansora	61653	2000	32.2	29.0	35.5
Egypt	Dakahlia	El Mansora	61653	2005	26.1	23.4	28.9
Egypt	Dakahlia	El Mansora	61653	2010	22.4	19.8	25.3
Egypt	Dakahlia	El Mansora	61653	2015	16.9	14.5	19.5
Egypt	Dakahlia	El Mansora 1	61654	2000	32.1	28.9	35.3
Egypt	Dakahlia	El Mansora 1	61654	2005	26.4	23.7	29.3
Egypt	Dakahlia	El Mansora 1	61654	2010	23.0	20.5	26.0
Egypt	Dakahlia	El Mansora 1	61654	2015	16.9	14.5	19.6
Egypt	Dakahlia	Gamaliyya	61655	2000	31.9	28.1	35.7
Egypt	Dakahlia	Gamaliyya	61655	2005	26.6	23.6	29.7
Egypt	Dakahlia	Gamaliyya	61655	2010	24.2	21.1	27.3
Egypt	Dakahlia	Gamaliyya	61655	2015	17.4	14.9	20.1
Egypt	Dakahlia	Gamsa	61656	2000	31.3	27.4	35.5
Egypt	Dakahlia	Gamsa	61656	2005	26.2	23.0	29.6
Egypt	Dakahlia	Gamsa	61656	2010	23.8	20.6	27.5
Egypt	Dakahlia	Gamsa	61656	2015	16.8	14.2	20.0
Egypt	Dakahlia	MIt Salsil	61662	2000	31.8	28.2	35.6
Egypt	Dakahlia	MIt Salsil	61662	2005	26.1	23.1	29.0
Egypt	Dakahlia	MIt Salsil	61662	2010	23.1	20.3	26.1
Egypt	Dakahlia	MIt Salsil	61662	2015	17.3	14.8	20.0
Egypt	Dakahlia	Mahalet Demna	61657	2000	31.9	28.6	35.4
Egypt	Dakahlia	Mahalet Demna	61657	2005	25.8	22.9	28.6
Egypt	Dakahlia	Mahalet Demna	61657	2010	22.2	19.5	25.1
Egypt	Dakahlia	Mahalet Demna	61657	2015	16.8	14.4	19.5
Egypt	Dakahlia	Manzala	61658	2000	32.8	28.8	37.0
Egypt	Dakahlia	Manzala	61658	2005	26.9	23.7	30.2
Egypt	Dakahlia	Manzala	61658	2010	23.6	20.6	26.8
Egypt	Dakahlia	Manzala	61658	2015	17.6	15.0	20.4
Egypt	Dakahlia	Matariyya	61659	2000	32.4	28.7	36.5
Egypt	Dakahlia	Matariyya	61659	2005	27.4	24.1	30.8
Egypt	Dakahlia	Matariyya	61659	2010	24.7	21.5	28.0
Egypt	Dakahlia	Matariyya	61659	2015	17.9	15.3	20.9
Egypt	Dakahlia	Minya Al-Nasr	61660	2000	31.2	28.1	34.6
Egypt	Dakahlia	Minya Al-Nasr	61660	2005	25.5	22.7	28.2
Egypt	Dakahlia	Minya Al-Nasr	61660	2010	22.4	19.7	25.2
Egypt	Dakahlia	Minya Al-Nasr	61660	2015	16.8	14.5	19.4
Egypt	Dakahlia	Mit Ghamr	61661	2000	34.0	31.1	37.0
Egypt	Dakahlia	Mit Ghamr	61661	2005	27.4	25.1	30.0
Egypt	Dakahlia	Mit Ghamr	61661	2010	21.9	19.6	24.3
Egypt	Dakahlia	Mit Ghamr	61661	2015	17.8	15.5	20.1
Egypt	Dakahlia	Nebro	61663	2000	31.4	28.1	34.4
Egypt	Dakahlia	Nebro	61663	2005	25.2	22.6	28.0
Egypt	Dakahlia	Nebro	61663	2010	21.1	18.9	23.7
Egypt	Dakahlia	Nebro	61663	2015	16.1	13.9	18.6
Egypt	Dakahlia	Shirbin	61664	2000	30.6	27.4	34.2
Egypt	Dakahlia	Shirbin	61664	2005	25.1	22.2	27.9
Egypt	Dakahlia	Shirbin	61664	2010	21.8	19.2	24.8
Egypt	Dakahlia	Shirbin	61664	2015	16.5	14.1	19.3
Egypt	Dakahlia	Sinbillawin	61665	2000	33.4	30.4	36.7
Egypt	Dakahlia	Sinbillawin	61665	2005	26.7	24.1	29.5
Egypt	Dakahlia	Sinbillawin	61665	2010	22.7	20.1	25.4
Egypt	Dakahlia	Sinbillawin	61665	2015	17.6	15.3	20.2
Egypt	Dakahlia	Talkha	61666	2000	31.6	28.4	34.8
Egypt	Dakahlia	Talkha	61666	2005	25.8	23.0	28.6
Egypt	Dakahlia	Talkha	61666	2010	22.4	19.9	25.1
Egypt	Dakahlia	Talkha	61666	2015	16.6	14.3	19.2
Egypt	Dakahlia	Tamy Al-Amdid	61667	2000	33.1	29.9	36.5
Egypt	Dakahlia	Tamy Al-Amdid	61667	2005	26.6	23.9	29.5
Egypt	Dakahlia	Tamy Al-Amdid	61667	2010	22.9	20.1	25.8
Egypt	Dakahlia	Tamy Al-Amdid	61667	2015	17.7	15.2	20.5
Egypt	Damietta	Dumyat	61668	2000	30.9	27.3	35.0
Egypt	Damietta	Dumyat	61668	2005	26.5	23.3	29.9
Egypt	Damietta	Dumyat	61668	2010	24.6	21.4	28.2
Egypt	Damietta	Dumyat	61668	2015	17.5	14.8	20.4
Egypt	Damietta	Dumyat 1	61669	2000	30.9	27.3	34.7
Egypt	Damietta	Dumyat 1	61669	2005	26.1	23.1	29.3

Admin 0	Admin 1	Admin 2	GAUL Code	Year	Under-5 mortality (per 1,000 livebirths)		
					Estimate	Lower bound, 95% UI	Upper bound, 95% UI
Egypt	Damietta	Dumyat 1	61669	2010	24.1	21.0	27.4
Egypt	Damietta	Dumyat 1	61669	2015	17.2	14.7	20.0
Egypt	Damietta	Dumyat 2	61670	2000	30.8	27.2	34.8
Egypt	Damietta	Dumyat 2	61670	2005	26.5	23.3	29.8
Egypt	Damietta	Dumyat 2	61670	2010	24.7	21.4	28.3
Egypt	Damietta	Dumyat 2	61670	2015	17.6	14.9	20.6
Egypt	Damietta	Dumyat Al-Gadida	61671	2000	30.9	27.2	34.9
Egypt	Damietta	Dumyat Al-Gadida	61671	2005	26.3	23.3	29.6
Egypt	Damietta	Dumyat Al-Gadida	61671	2010	24.2	21.1	27.7
Egypt	Damietta	Dumyat Al-Gadida	61671	2015	16.9	14.5	19.9
Egypt	Damietta	Fariskur	61672	2000	30.6	27.2	34.1
Egypt	Damietta	Fariskur	61672	2005	25.3	22.4	28.2
Egypt	Damietta	Fariskur	61672	2010	22.4	19.6	25.4
Egypt	Damietta	Fariskur	61672	2015	16.4	14.1	19.1
Egypt	Damietta	Kafr Sad	61673	2000	30.6	27.2	34.4
Egypt	Damietta	Kafr Sad	61673	2005	25.4	22.5	28.4
Egypt	Damietta	Kafr Sad	61673	2010	22.6	19.7	25.8
Egypt	Damietta	Kafr Sad	61673	2015	16.5	14.1	19.4
Egypt	Damietta	Police Department Port of Damietta	61674	2000	30.7	27.0	34.8
Egypt	Damietta	Police Department Port of Damietta	61674	2005	26.4	23.3	29.8
Egypt	Damietta	Police Department Port of Damietta	61674	2010	24.5	21.3	28.2
Egypt	Damietta	Police Department Port of Damietta	61674	2015	17.2	14.6	20.1
Egypt	Damietta	Ras al-Bar	61675	2000	31.0	27.0	35.3
Egypt	Damietta	Ras al-Bar	61675	2005	26.9	23.4	30.7
Egypt	Damietta	Ras al-Bar	61675	2010	25.0	21.7	28.8
Egypt	Damietta	Ras al-Bar	61675	2015	17.7	14.9	20.8
Egypt	Damietta	Zarqa	61676	2000	30.6	27.5	34.0
Egypt	Damietta	Zarqa	61676	2005	25.1	22.3	27.8
Egypt	Damietta	Zarqa	61676	2010	22.0	19.3	24.9
Egypt	Damietta	Zarqa	61676	2015	16.7	14.4	19.4
Egypt	Fayoum	Abshaway	61677	2000	51.3	45.5	57.2
Egypt	Fayoum	Abshaway	61677	2005	38.3	33.8	42.7
Egypt	Fayoum	Abshaway	61677	2010	27.8	24.5	31.9
Egypt	Fayoum	Abshaway	61677	2015	22.4	19.2	26.1
Egypt	Fayoum	Atsa	61678	2000	54.4	49.0	59.9
Egypt	Fayoum	Atsa	61678	2005	40.8	36.7	45.1
Egypt	Fayoum	Atsa	61678	2010	29.3	26.1	33.2
Egypt	Fayoum	Atsa	61678	2015	23.8	20.6	27.5
Egypt	Fayoum	Fayyum	61679	2000	53.0	48.0	58.3
Egypt	Fayoum	Fayyum	61679	2005	39.5	35.5	43.5
Egypt	Fayoum	Fayyum	61679	2010	28.1	25.0	31.7
Egypt	Fayoum	Fayyum	61679	2015	23.6	20.4	27.4
Egypt	Fayoum	Fayyum City	61680	2000	56.6	51.7	62.1
Egypt	Fayoum	Fayyum City	61680	2005	42.8	38.9	47.2
Egypt	Fayoum	Fayyum City	61680	2010	30.0	26.8	33.6
Egypt	Fayoum	Fayyum City	61680	2015	24.5	21.2	28.2
Egypt	Fayoum	Sinuras	61681	2000	51.1	46.1	56.4
Egypt	Fayoum	Sinuras	61681	2005	38.1	34.1	42.0
Egypt	Fayoum	Sinuras	61681	2010	27.4	24.3	31.1
Egypt	Fayoum	Sinuras	61681	2015	22.8	19.8	26.5
Egypt	Fayoum	Tamya	61682	2000	50.8	46.4	55.5
Egypt	Fayoum	Tamya	61682	2005	37.8	34.2	41.4
Egypt	Fayoum	Tamya	61682	2010	27.6	24.7	31.0
Egypt	Fayoum	Tamya	61682	2015	23.7	20.7	27.2
Egypt	Fayoum	Yousef El sadeq	61683	2000	50.9	45.2	57.1
Egypt	Fayoum	Yousef El sadeq	61683	2005	38.3	33.8	43.2
Egypt	Fayoum	Yousef El sadeq	61683	2010	28.4	24.9	32.5
Egypt	Fayoum	Yousef El sadeq	61683	2015	22.4	19.1	26.3
Egypt	Fayoum	Zemam Out	61684	2000	52.7	47.8	57.9
Egypt	Fayoum	Zemam Out	61684	2005	40.1	36.2	44.3
Egypt	Fayoum	Zemam Out	61684	2010	29.9	26.7	33.6
Egypt	Fayoum	Zemam Out	61684	2015	23.3	20.2	26.9
Egypt	Gharbia	Basyun	61685	2000	32.2	29.0	35.8
Egypt	Gharbia	Basyun	61685	2005	25.6	23.1	28.4
Egypt	Gharbia	Basyun	61685	2010	20.7	18.4	23.3
Egypt	Gharbia	Basyun	61685	2015	15.9	13.7	18.3
Egypt	Gharbia	El Mahalla El Kobra	61686	2000	31.5	28.5	34.5
Egypt	Gharbia	El Mahalla El Kobra	61686	2005	25.4	22.9	27.9
Egypt	Gharbia	El Mahalla El Kobra	61686	2010	21.0	18.8	23.5
Egypt	Gharbia	El Mahalla El Kobra	61686	2015	16.1	13.9	18.5
Egypt	Gharbia	El Mahalla El Kobra 1	61687	2000	31.5	28.6	34.3
Egypt	Gharbia	El Mahalla El Kobra 1	61687	2005	25.8	23.4	28.3
Egypt	Gharbia	El Mahalla El Kobra 1	61687	2010	21.8	19.6	24.3
Egypt	Gharbia	El Mahalla El Kobra 1	61687	2015	16.4	14.3	18.9
Egypt	Gharbia	El Mahalla El Kobra 2	61688	2000	31.1	28.2	34.0
Egypt	Gharbia	El Mahalla El Kobra 2	61688	2005	25.4	23.0	28.0
Egypt	Gharbia	El Mahalla El Kobra 2	61688	2010	21.3	19.1	23.8
Egypt	Gharbia	El Mahalla El Kobra 2	61688	2015	16.2	14.1	18.5
Egypt	Gharbia	Kafr Al-Zayyat	61689	2000	33.1	30.0	36.6
Egypt	Gharbia	Kafr Al-Zayyat	61689	2005	26.1	23.5	29.0
Egypt	Gharbia	Kafr Al-Zayyat	61689	2010	20.8	18.6	23.5
Egypt	Gharbia	Kafr Al-Zayyat	61689	2015	16.1	14.0	18.5
Egypt	Gharbia	Qutur	61690	2000	31.5	28.2	34.9
Egypt	Gharbia	Qutur	61690	2005	25.2	22.6	27.9
Egypt	Gharbia	Qutur	61690	2010	20.5	18.2	23.1

Admin 0	Admin 1	Admin 2	GAUL Code	Year	Under-5 mortality (per 1,000 livebirths)		
					Estimate	Lower bound, 95% UI	Upper bound, 95% UI
Egypt	Gharbia	Qutur	61690	2015	15.8	13.7	18.4
Egypt	Gharbia	Samannud	61691	2000	32.1	29.3	34.9
Egypt	Gharbia	Samannud	61691	2005	26.0	23.6	28.5
Egypt	Gharbia	Samannud	61691	2010	22.0	19.7	24.5
Egypt	Gharbia	Samannud	61691	2015	16.5	14.4	18.8
Egypt	Gharbia	Santa	61692	2000	32.4	29.5	35.4
Egypt	Gharbia	Santa	61692	2005	26.2	23.7	28.8
Egypt	Gharbia	Santa	61692	2010	21.4	19.1	24.1
Egypt	Gharbia	Santa	61692	2015	16.5	14.4	18.9
Egypt	Gharbia	Tanta	61693	2000	31.9	28.8	35.1
Egypt	Gharbia	Tanta	61693	2005	25.6	23.1	28.5
Egypt	Gharbia	Tanta	61693	2010	20.9	18.7	23.7
Egypt	Gharbia	Tanta	61693	2015	16.1	14.0	18.6
Egypt	Gharbia	Tanta 1	61694	2000	31.5	28.4	34.8
Egypt	Gharbia	Tanta 1	61694	2005	25.6	23.0	28.5
Egypt	Gharbia	Tanta 1	61694	2010	21.0	18.7	23.9
Egypt	Gharbia	Tanta 1	61694	2015	16.2	14.0	18.7
Egypt	Gharbia	Tanta 2	61695	2000	31.8	28.6	35.1
Egypt	Gharbia	Tanta 2	61695	2005	25.9	23.2	28.8
Egypt	Gharbia	Tanta 2	61695	2010	21.3	19.0	24.2
Egypt	Gharbia	Tanta 2	61695	2015	16.2	14.1	18.7
Egypt	Gharbia	Zifta	61696	2000	33.1	30.3	36.1
Egypt	Gharbia	Zifta	61696	2005	26.7	24.4	29.3
Egypt	Gharbia	Zifta	61696	2010	19.3	17.2	21.5
Egypt	Gharbia	Zifta	61696	2015	17.2	15.1	19.6
Egypt	Giza	6-Oct-01	61697	2000	41.6	37.4	46.2
Egypt	Giza	6-Oct-01	61697	2005	33.5	30.0	37.4
Egypt	Giza	6-Oct-01	61697	2010	27.2	24.0	30.9
Egypt	Giza	6-Oct-01	61697	2015	21.1	18.1	24.3
Egypt	Giza	6-Oct-02	61698	2000	42.9	38.0	48.1
Egypt	Giza	6-Oct-02	61698	2005	34.1	30.1	38.5
Egypt	Giza	6-Oct-02	61698	2010	27.5	24.1	31.4
Egypt	Giza	6-Oct-02	61698	2015	21.6	18.3	25.0
Egypt	Giza	Al-Aguza	61699	2000	38.3	35.0	41.9
Egypt	Giza	Al-Aguza	61699	2005	32.1	29.1	35.2
Egypt	Giza	Al-Aguza	61699	2010	26.8	24.0	29.7
Egypt	Giza	Al-Aguza	61699	2015	20.2	17.6	23.0
Egypt	Giza	Al-Ahram	61700	2000	39.6	36.4	43.2
Egypt	Giza	Al-Ahram	61700	2005	33.0	29.9	36.1
Egypt	Giza	Al-Ahram	61700	2010	27.0	24.3	30.1
Egypt	Giza	Al-Ahram	61700	2015	20.6	18.0	23.5
Egypt	Giza	Atfeh	61701	2000	50.4	45.5	55.6
Egypt	Giza	Atfeh	61701	2005	39.0	35.2	43.3
Egypt	Giza	Atfeh	61701	2010	29.4	25.9	33.4
Egypt	Giza	Atfeh	61701	2015	25.1	21.6	29.2
Egypt	Giza	Ayat	61702	2000	45.3	41.1	50.0
Egypt	Giza	Ayat	61702	2005	36.5	32.8	40.4
Egypt	Giza	Ayat	61702	2010	28.5	25.4	32.4
Egypt	Giza	Ayat	61702	2015	23.7	20.5	27.6
Egypt	Giza	Badrashain	61703	2000	41.1	37.0	45.5
Egypt	Giza	Badrashain	61703	2005	34.2	30.7	37.9
Egypt	Giza	Badrashain	61703	2010	27.7	24.5	31.2
Egypt	Giza	Badrashain	61703	2015	21.8	18.9	25.3
Egypt	Giza	Bahariya Oasis	64448	2000	46.4	35.2	60.0
Egypt	Giza	Bahariya Oasis	64448	2005	42.9	32.4	55.6
Egypt	Giza	Bahariya Oasis	64448	2010	35.5	26.6	46.5
Egypt	Giza	Bahariya Oasis	64448	2015	25.2	18.8	32.9
Egypt	Giza	Bulaq Al-DakrUr	64449	2000	38.5	35.3	42.1
Egypt	Giza	Bulaq Al-DakrUr	64449	2005	32.6	29.7	35.8
Egypt	Giza	Bulaq Al-DakrUr	64449	2010	26.9	24.2	29.7
Egypt	Giza	Bulaq Al-DakrUr	64449	2015	20.4	17.8	23.2
Egypt	Giza	Duqql	64451	2000	37.7	34.5	41.2
Egypt	Giza	Duqql	64451	2005	32.7	29.7	35.8
Egypt	Giza	Duqql	64451	2010	27.1	24.4	30.0
Egypt	Giza	Duqql	64451	2015	20.5	17.9	23.5
Egypt	Giza	Giza	64453	2000	38.9	35.6	42.9
Egypt	Giza	Giza	64453	2005	33.1	30.0	36.3
Egypt	Giza	Giza	64453	2010	27.3	24.4	30.2
Egypt	Giza	Giza	64453	2015	20.8	18.1	23.8
Egypt	Giza	Hwamdeia	65305	2000	39.8	36.1	44.1
Egypt	Giza	Hwamdeia	65305	2005	33.2	30.0	36.6
Egypt	Giza	Hwamdeia	65305	2010	27.2	24.2	30.3
Egypt	Giza	Hwamdeia	65305	2015	22.1	19.2	25.4
Egypt	Giza	Imbaba	65315	2000	38.6	35.2	42.2
Egypt	Giza	Imbaba	65315	2005	30.5	27.7	33.7
Egypt	Giza	Imbaba	65315	2010	24.5	22.0	27.4
Egypt	Giza	Imbaba	65315	2015	18.8	16.5	21.4
Egypt	Giza	Kardasa	65317	2000	39.2	36.0	42.8
Egypt	Giza	Kardasa	65317	2005	32.1	29.2	35.2
Egypt	Giza	Kardasa	65317	2010	26.3	23.8	29.2
Egypt	Giza	Kardasa	65317	2015	20.2	17.7	23.0
Egypt	Giza	Saf	65321	2000	43.9	39.6	48.5
Egypt	Giza	Saf	65321	2005	35.8	32.2	39.6
Egypt	Giza	Saf	65321	2010	28.1	25.0	31.8
Egypt	Giza	Saf	65321	2015	23.1	19.9	26.8

Admin 0	Admin 1	Admin 2	GAUL Code	Year	Under-5 mortality (per 1,000 livebirths)		
					Estimate	Lower bound, 95% UI	Upper bound, 95% UI
Egypt	Giza	Shaykh Zayed	65336	2000	41.2	37.4	45.5
Egypt	Giza	Shaykh Zayed	65336	2005	33.0	29.8	36.7
Egypt	Giza	Shaykh Zayed	65336	2010	26.8	23.8	30.1
Egypt	Giza	Shaykh Zayed	65336	2015	20.3	17.5	23.3
Egypt	Giza	Umraniyya	65337	2000	39.3	36.0	43.1
Egypt	Giza	Umraniyya	65337	2005	33.1	30.1	36.3
Egypt	Giza	Umraniyya	65337	2010	27.1	24.2	30.1
Egypt	Giza	Umraniyya	65337	2015	20.8	18.2	23.7
Egypt	Giza	Waraq	65345	2000	38.1	34.9	41.6
Egypt	Giza	Waraq	65345	2005	31.9	29.0	35.0
Egypt	Giza	Waraq	65345	2010	26.5	23.8	29.5
Egypt	Giza	Waraq	65345	2015	20.0	17.5	22.9
Egypt	Giza	Zemam out	65350	2000	41.7	38.7	45.1
Egypt	Giza	Zemam out	65350	2005	34.1	31.3	36.9
Egypt	Giza	Zemam out	65350	2010	27.6	25.0	30.4
Egypt	Giza	Zemam out	65350	2015	21.0	18.5	23.8
Egypt	Hala'ib triangle	Hala'ib Triangle	40779	2000	70.3	56.0	87.5
Egypt	Hala'ib triangle	Hala'ib Triangle	40779	2005	59.9	47.6	74.0
Egypt	Hala'ib triangle	Hala'ib Triangle	40779	2010	48.6	38.4	60.4
Egypt	Hala'ib triangle	Hala'ib Triangle	40779	2015	36.5	29.0	45.8
Egypt	Ismailia	Fayid	65711	2000	36.1	31.6	41.1
Egypt	Ismailia	Fayid	65711	2005	32.6	28.5	36.9
Egypt	Ismailia	Fayid	65711	2010	26.7	23.1	30.3
Egypt	Ismailia	Fayid	65711	2015	19.5	16.4	22.8
Egypt	Ismailia	Ismailiyya	65747	2000	36.1	31.9	40.7
Egypt	Ismailia	Ismailiyya	65747	2005	31.7	27.7	35.6
Egypt	Ismailia	Ismailiyya	65747	2010	25.8	22.6	29.1
Egypt	Ismailia	Ismailiyya	65747	2015	19.3	16.4	22.4
Egypt	Ismailia	Ismailiyya 1	65748	2000	35.5	31.4	40.3
Egypt	Ismailia	Ismailiyya 1	65748	2005	31.6	27.6	35.9
Egypt	Ismailia	Ismailiyya 1	65748	2010	26.3	23.0	29.9
Egypt	Ismailia	Ismailiyya 1	65748	2015	19.1	16.2	22.2
Egypt	Ismailia	Ismailiyya 2	65749	2000	35.6	31.6	40.2
Egypt	Ismailia	Ismailiyya 2	65749	2005	31.5	27.7	35.6
Egypt	Ismailia	Ismailiyya 2	65749	2010	26.1	22.9	29.5
Egypt	Ismailia	Ismailiyya 2	65749	2015	19.1	16.2	22.0
Egypt	Ismailia	Ismailiyya 3	65751	2000	35.6	31.6	40.2
Egypt	Ismailia	Ismailiyya 3	65751	2005	31.5	27.7	35.6
Egypt	Ismailia	Ismailiyya 3	65751	2010	26.1	22.9	29.5
Egypt	Ismailia	Ismailiyya 3	65751	2015	19.1	16.2	22.0
Egypt	Ismailia	Qantara Gharb, al-	65752	2000	35.6	31.5	40.0
Egypt	Ismailia	Qantara Gharb, al-	65752	2005	31.1	27.5	35.2
Egypt	Ismailia	Qantara Gharb, al-	65752	2010	26.4	23.0	30.0
Egypt	Ismailia	Qantara Gharb, al-	65752	2015	19.3	16.4	22.3
Egypt	Ismailia	Qantara Sharq, al-	65753	2000	35.7	31.4	40.4
Egypt	Ismailia	Qantara Sharq, al-	65753	2005	31.8	27.9	36.2
Egypt	Ismailia	Qantara Sharq, al-	65753	2010	27.4	23.8	31.2
Egypt	Ismailia	Qantara Sharq, al-	65753	2015	20.0	17.0	23.2
Egypt	Ismailia	Tal al-Kabir, al-	65754	2000	36.9	33.1	41.3
Egypt	Ismailia	Tal al-Kabir, al-	65754	2005	31.4	27.9	35.0
Egypt	Ismailia	Tal al-Kabir, al-	65754	2010	26.5	23.3	29.9
Egypt	Ismailia	Tal al-Kabir, al-	65754	2015	20.0	17.4	23.2
Egypt	Kafr El-Shikh	Al Hamul	65755	2000	32.3	28.2	36.9
Egypt	Kafr El-Shikh	Al Hamul	65755	2005	25.8	22.4	29.3
Egypt	Kafr El-Shikh	Al Hamul	65755	2010	21.5	18.7	24.7
Egypt	Kafr El-Shikh	Al Hamul	65755	2015	16.2	13.6	19.2
Egypt	Kafr El-Shikh	Biyalu	65758	2000	31.6	28.2	35.1
Egypt	Kafr El-Shikh	Biyalu	65758	2005	25.2	22.3	28.1
Egypt	Kafr El-Shikh	Biyalu	65758	2010	20.9	18.5	23.5
Egypt	Kafr El-Shikh	Biyalu	65758	2015	16.0	13.7	18.7
Egypt	Kafr El-Shikh	Burullus	65759	2000	33.7	29.3	38.8
Egypt	Kafr El-Shikh	Burullus	65759	2005	27.1	23.5	31.3
Egypt	Kafr El-Shikh	Burullus	65759	2010	22.9	19.6	26.6
Egypt	Kafr El-Shikh	Burullus	65759	2015	16.9	14.0	20.2
Egypt	Kafr El-Shikh	Disuq	65760	2000	33.0	29.3	36.8
Egypt	Kafr El-Shikh	Disuq	65760	2005	25.9	23.1	28.9
Egypt	Kafr El-Shikh	Disuq	65760	2010	20.7	18.3	23.5
Egypt	Kafr El-Shikh	Disuq	65760	2015	15.9	13.7	18.5
Egypt	Kafr El-Shikh	Fuwwa	65761	2000	34.1	30.0	38.4
Egypt	Kafr El-Shikh	Fuwwa	65761	2005	26.6	23.5	29.9
Egypt	Kafr El-Shikh	Fuwwa	65761	2010	21.2	18.5	23.9
Egypt	Kafr El-Shikh	Fuwwa	65761	2015	16.3	14.1	18.9
Egypt	Kafr El-Shikh	Kafr Al-Shaykh	65762	2000	31.6	27.9	35.2
Egypt	Kafr El-Shikh	Kafr Al-Shaykh	65762	2005	25.1	22.2	28.0
Egypt	Kafr El-Shikh	Kafr Al-Shaykh	65762	2010	20.3	18.0	23.0
Egypt	Kafr El-Shikh	Kafr Al-Shaykh	65762	2015	15.7	13.4	18.4
Egypt	Kafr El-Shikh	Mitubas	65763	2000	34.7	30.1	39.2
Egypt	Kafr El-Shikh	Mitubas	65763	2005	27.7	24.2	31.3
Egypt	Kafr El-Shikh	Mitubas	65763	2010	22.7	19.6	26.1
Egypt	Kafr El-Shikh	Mitubas	65763	2015	17.0	14.5	19.9
Egypt	Kafr El-Shikh	Qillin	65766	2000	32.3	28.9	35.9
Egypt	Kafr El-Shikh	Qillin	65766	2005	25.4	22.8	28.3
Egypt	Kafr El-Shikh	Qillin	65766	2010	20.4	18.1	23.2
Egypt	Kafr El-Shikh	Qillin	65766	2015	15.8	13.6	18.3
Egypt	Kafr El-Shikh	Riyad	65793	2000	32.2	28.4	36.4

Admin 0	Admin 1	Admin 2	GAUL Code	Year	Under-5 mortality (per 1,000 livebirths)		
					Estimate	Lower bound, 95% UI	Upper bound, 95% UI
Egypt	Kafr El-Shikh	Riyad	65793	2005	25.6	22.5	28.7
Egypt	Kafr El-Shikh	Riyad	65793	2010	21.1	18.4	23.9
Egypt	Kafr El-Shikh	Riyad	65793	2015	15.9	13.5	18.7
Egypt	Kafr El-Shikh	Sidi Salim	65801	2000	33.0	28.9	37.3
Egypt	Kafr El-Shikh	Sidi Salim	65801	2005	26.2	23.1	29.4
Egypt	Kafr El-Shikh	Sidi Salim	65801	2010	21.4	18.6	24.5
Egypt	Kafr El-Shikh	Sidi Salim	65801	2015	16.2	13.8	18.9
Egypt	Kalyoubia	Abour	65802	2000	37.2	34.1	40.5
Egypt	Kalyoubia	Abour	65802	2005	31.9	29.2	34.8
Egypt	Kalyoubia	Abour	65802	2010	27.0	24.5	30.0
Egypt	Kalyoubia	Abour	65802	2015	20.1	17.6	22.9
Egypt	Kalyoubia	Al Khanka	65804	2000	37.3	34.3	40.3
Egypt	Kalyoubia	Al Khanka	65804	2005	31.2	28.5	33.9
Egypt	Kalyoubia	Al Khanka	65804	2010	25.9	23.5	28.6
Egypt	Kalyoubia	Al Khanka	65804	2015	19.8	17.5	22.5
Egypt	Kalyoubia	Banha	65806	2000	36.0	33.1	39.0
Egypt	Kalyoubia	Banha	65806	2005	28.8	26.3	31.5
Egypt	Kalyoubia	Banha	65806	2010	23.6	21.3	26.3
Egypt	Kalyoubia	Banha	65806	2015	18.0	15.9	20.4
Egypt	Kalyoubia	Kafr Shukr	65807	2000	35.9	32.9	39.0
Egypt	Kalyoubia	Kafr Shukr	65807	2005	29.0	26.4	31.8
Egypt	Kalyoubia	Kafr Shukr	65807	2010	24.4	21.9	26.9
Egypt	Kalyoubia	Kafr Shukr	65807	2015	18.3	16.1	20.7
Egypt	Kalyoubia	Khsos	65811	2000	37.6	34.5	40.7
Egypt	Kalyoubia	Khsos	65811	2005	31.6	28.9	34.6
Egypt	Kalyoubia	Khsos	65811	2010	26.7	24.1	29.5
Egypt	Kalyoubia	Khsos	65811	2015	19.9	17.5	22.7
Egypt	Kalyoubia	Qaha	65815	2000	38.1	35.1	41.4
Egypt	Kalyoubia	Qaha	65815	2005	30.4	27.6	33.2
Egypt	Kalyoubia	Qaha	65815	2010	24.2	21.8	26.9
Egypt	Kalyoubia	Qaha	65815	2015	18.9	16.6	21.5
Egypt	Kalyoubia	Qalyub	65816	2000	37.7	34.7	41.0
Egypt	Kalyoubia	Qalyub	65816	2005	31.2	28.3	34.2
Egypt	Kalyoubia	Qalyub	65816	2010	25.2	22.8	28.0
Egypt	Kalyoubia	Qalyub	65816	2015	19.5	17.1	22.3
Egypt	Kalyoubia	Qanatir Al-Khayriyya	65817	2000	37.8	34.8	41.4
Egypt	Kalyoubia	Qanatir Al-Khayriyya	65817	2005	30.7	27.8	33.7
Egypt	Kalyoubia	Qanatir Al-Khayriyya	65817	2010	24.6	22.1	27.5
Egypt	Kalyoubia	Qanatir Al-Khayriyya	65817	2015	19.2	16.8	21.8
Egypt	Kalyoubia	Shibin al-Qanatir	65819	2000	37.7	34.8	41.0
Egypt	Kalyoubia	Shibin al-Qanatir	65819	2005	30.4	27.8	33.0
Egypt	Kalyoubia	Shibin al-Qanatir	65819	2010	24.5	22.2	27.2
Egypt	Kalyoubia	Shibin al-Qanatir	65819	2015	19.3	17.0	21.7
Egypt	Kalyoubia	Shubra Al-Khayma 1	65821	2000	37.7	34.6	40.9
Egypt	Kalyoubia	Shubra Al-Khayma 1	65821	2005	31.6	28.8	34.7
Egypt	Kalyoubia	Shubra Al-Khayma 1	65821	2010	26.2	23.6	28.9
Egypt	Kalyoubia	Shubra Al-Khayma 1	65821	2015	19.9	17.4	22.7
Egypt	Kalyoubia	Shubra Al-Khayma 2	65824	2000	37.7	34.6	40.9
Egypt	Kalyoubia	Shubra Al-Khayma 2	65824	2005	31.6	28.8	34.7
Egypt	Kalyoubia	Shubra Al-Khayma 2	65824	2010	26.2	23.6	28.9
Egypt	Kalyoubia	Shubra Al-Khayma 2	65824	2015	19.9	17.4	22.7
Egypt	Kalyoubia	Tukh	65825	2000	37.1	34.3	40.3
Egypt	Kalyoubia	Tukh	65825	2005	29.5	26.9	32.2
Egypt	Kalyoubia	Tukh	65825	2010	23.8	21.4	26.5
Egypt	Kalyoubia	Tukh	65825	2015	18.5	16.3	20.9
Egypt	Kalyoubia	Zemam Out	65828	2000	37.1	34.0	40.3
Egypt	Kalyoubia	Zemam Out	65828	2005	31.4	28.8	34.3
Egypt	Kalyoubia	Zemam Out	65828	2010	26.6	24.1	29.4
Egypt	Kalyoubia	Zemam Out	65828	2015	19.9	17.5	22.7
Egypt	Luxor	Luxor	65830	2000	54.1	48.4	60.4
Egypt	Luxor	Luxor	65830	2005	45.5	40.7	50.7
Egypt	Luxor	Luxor	65830	2010	34.1	29.8	38.6
Egypt	Luxor	Luxor	65830	2015	24.2	20.6	28.1
Egypt	Luxor	Tiba police station	65832	2000	55.2	49.5	61.5
Egypt	Luxor	Tiba police station	65832	2005	45.6	41.0	50.8
Egypt	Luxor	Tiba police station	65832	2010	33.6	29.5	38.1
Egypt	Luxor	Tiba police station	65832	2015	24.0	20.6	27.6
Egypt	Luxor	Zemam Out	65833	2000	56.6	50.7	63.0
Egypt	Luxor	Zemam Out	65833	2005	47.5	42.5	53.0
Egypt	Luxor	Zemam Out	65833	2010	35.0	30.7	39.6
Egypt	Luxor	Zemam Out	65833	2015	24.6	21.0	28.6
Egypt	Matrouh	Al-Hammam	65834	2000	46.5	38.0	55.8
Egypt	Matrouh	Al-Hammam	65834	2005	37.3	30.6	44.8
Egypt	Matrouh	Al-Hammam	65834	2010	29.2	23.8	35.6
Egypt	Matrouh	Al-Hammam	65834	2015	21.0	17.0	25.5
Egypt	Matrouh	Alamn	65835	2000	45.3	36.7	55.0
Egypt	Matrouh	Alamn	65835	2005	37.2	30.1	45.7
Egypt	Matrouh	Alamn	65835	2010	29.5	23.7	36.4
Egypt	Matrouh	Alamn	65835	2015	21.0	16.7	25.9
Egypt	Matrouh	Daba	65837	2000	45.2	35.5	55.8
Egypt	Matrouh	Daba	65837	2005	36.9	29.0	46.0
Egypt	Matrouh	Daba	65837	2010	29.0	23.0	36.0
Egypt	Matrouh	Daba	65837	2015	20.5	16.0	25.6
Egypt	Matrouh	Marsa Matruh	65838	2000	43.7	34.3	53.3
Egypt	Matrouh	Marsa Matruh	65838	2005	37.2	29.6	45.9

Admin 0	Admin 1	Admin 2	GAUL Code	Year	Under-5 mortality (per 1,000 livebirths)		
					Estimate	Lower bound, 95% UI	Upper bound, 95% UI
Egypt	Matrouh	Marsa Matruh	65838	2010	30.3	24.0	37.1
Egypt	Matrouh	Marsa Matruh	65838	2015	20.8	16.5	25.9
Egypt	Matrouh	Salloum	65840	2000	53.0	40.7	66.7
Egypt	Matrouh	Salloum	65840	2005	48.5	37.6	61.2
Egypt	Matrouh	Salloum	65840	2010	40.0	30.9	50.1
Egypt	Matrouh	Salloum	65840	2015	28.1	21.8	35.7
Egypt	Matrouh	Sidi Barani	65841	2000	50.3	38.7	62.8
Egypt	Matrouh	Sidi Barani	65841	2005	43.6	33.5	54.4
Egypt	Matrouh	Sidi Barani	65841	2010	35.7	27.5	44.7
Egypt	Matrouh	Sidi Barani	65841	2015	24.5	18.8	30.4
Egypt	Matrouh	Siwa	65842	2000	49.9	37.6	64.8
Egypt	Matrouh	Siwa	65842	2005	45.5	33.8	59.3
Egypt	Matrouh	Siwa	65842	2010	37.6	27.9	48.9
Egypt	Matrouh	Siwa	65842	2015	27.1	20.1	35.1
Egypt	Menia	Kesm Al-minya	65843	2000	65.6	59.1	72.4
Egypt	Menia	Kesm Al-minya	65843	2005	49.8	44.6	55.9
Egypt	Menia	Kesm Al-minya	65843	2010	36.7	32.0	41.9
Egypt	Menia	Kesm Al-minya	65843	2015	27.2	23.4	31.5
Egypt	Menia	Kesm Mallawi	65845	2000	67.0	60.0	74.6
Egypt	Menia	Kesm Mallawi	65845	2005	49.5	44.0	55.1
Egypt	Menia	Kesm Mallawi	65845	2010	36.8	32.2	42.0
Egypt	Menia	Kesm Mallawi	65845	2015	27.2	23.2	31.7
Egypt	Menia	Markz Abu Qurqas	65846	2000	67.0	60.0	75.1
Egypt	Menia	Markz Abu Qurqas	65846	2005	50.1	44.5	56.4
Egypt	Menia	Markz Abu Qurqas	65846	2010	37.1	32.1	42.7
Egypt	Menia	Markz Abu Qurqas	65846	2015	27.3	23.4	32.0
Egypt	Menia	Markz Al Idwa	65848	2000	65.6	59.0	71.8
Egypt	Menia	Markz Al Idwa	65848	2005	48.8	43.8	54.3
Egypt	Menia	Markz Al Idwa	65848	2010	34.4	30.2	39.3
Egypt	Menia	Markz Al Idwa	65848	2015	25.5	21.9	29.5
Egypt	Menia	Markz Al Minya	65849	2000	66.7	60.0	73.4
Egypt	Menia	Markz Al Minya	65849	2005	50.3	45.1	56.2
Egypt	Menia	Markz Al Minya	65849	2010	37.0	32.4	42.2
Egypt	Menia	Markz Al Minya	65849	2015	27.3	23.5	31.6
Egypt	Menia	Markz Bani Mazar	65850	2000	65.9	59.5	72.6
Egypt	Menia	Markz Bani Mazar	65850	2005	49.4	44.3	55.1
Egypt	Menia	Markz Bani Mazar	65850	2010	35.7	31.3	40.5
Egypt	Menia	Markz Bani Mazar	65850	2015	26.5	22.8	30.7
Egypt	Menia	Markz Dir Mawas	65852	2000	67.3	60.7	74.2
Egypt	Menia	Markz Dir Mawas	65852	2005	49.8	44.5	55.2
Egypt	Menia	Markz Dir Mawas	65852	2010	37.2	32.8	42.3
Egypt	Menia	Markz Dir Mawas	65852	2015	27.1	23.1	31.7
Egypt	Menia	Markz Maghagha	65854	2000	65.2	58.6	71.1
Egypt	Menia	Markz Maghagha	65854	2005	49.1	43.8	54.6
Egypt	Menia	Markz Maghagha	65854	2010	35.1	30.9	39.9
Egypt	Menia	Markz Maghagha	65854	2015	26.1	22.4	30.4
Egypt	Menia	Markz Mallawi	65855	2000	67.4	60.1	75.4
Egypt	Menia	Markz Mallawi	65855	2005	49.9	44.4	55.9
Egypt	Menia	Markz Mallawi	65855	2010	37.1	32.5	42.7
Egypt	Menia	Markz Mallawi	65855	2015	27.3	23.3	31.9
Egypt	Menia	Markz Matay	65857	2000	66.7	59.9	74.2
Egypt	Menia	Markz Matay	65857	2005	49.6	44.4	55.8
Egypt	Menia	Markz Matay	65857	2010	36.0	31.6	41.2
Egypt	Menia	Markz Matay	65857	2015	26.8	23.0	31.2
Egypt	Menia	Markz Samalut	65862	2000	66.9	59.7	75.0
Egypt	Menia	Markz Samalut	65862	2005	50.1	44.7	56.9
Egypt	Menia	Markz Samalut	65862	2010	36.5	31.7	42.2
Egypt	Menia	Markz Samalut	65862	2015	27.2	23.3	31.8
Egypt	Menia	New Minya	65865	2000	65.4	58.6	72.3
Egypt	Menia	New Minya	65865	2005	50.4	45.2	56.6
Egypt	Menia	New Minya	65865	2010	37.7	33.0	43.4
Egypt	Menia	New Minya	65865	2015	27.1	23.4	31.5
Egypt	Menia	Zemam Out	65866	2000	67.0	61.4	72.9
Egypt	Menia	Zemam Out	65866	2005	50.6	46.0	55.8
Egypt	Menia	Zemam Out	65866	2010	37.5	33.3	42.3
Egypt	Menia	Zemam Out	65866	2015	27.2	23.7	31.4
Egypt	Menoufia	Al-Bagur	65872	2000	35.7	32.5	39.2
Egypt	Menoufia	Al-Bagur	65872	2005	28.3	25.7	31.2
Egypt	Menoufia	Al-Bagur	65872	2010	22.7	20.3	25.7
Egypt	Menoufia	Al-Bagur	65872	2015	17.6	15.3	20.0
Egypt	Menoufia	Al-Shuhada	65873	2000	35.1	31.8	38.8
Egypt	Menoufia	Al-Shuhada	65873	2005	27.2	24.5	30.0
Egypt	Menoufia	Al-Shuhada	65873	2010	21.4	19.1	24.3
Egypt	Menoufia	Al-Shuhada	65873	2015	16.6	14.4	19.2
Egypt	Menoufia	Ashmun	65874	2000	37.8	34.4	41.6
Egypt	Menoufia	Ashmun	65874	2005	29.5	26.6	32.6
Egypt	Menoufia	Ashmun	65874	2010	23.4	20.8	26.4
Egypt	Menoufia	Ashmun	65874	2015	18.2	15.8	20.7
Egypt	Menoufia	Birkat Al-Sab	65875	2000	33.1	30.1	36.3
Egypt	Menoufia	Birkat Al-Sab	65875	2005	26.6	24.1	29.4
Egypt	Menoufia	Birkat Al-Sab	65875	2010	21.1	18.7	23.8
Egypt	Menoufia	Birkat Al-Sab	65875	2015	16.7	14.6	19.2
Egypt	Menoufia	Minuf	65876	2000	35.9	32.3	39.7
Egypt	Menoufia	Minuf	65876	2005	28.0	25.2	31.1
Egypt	Menoufia	Minuf	65876	2010	22.1	19.5	25.2

Admin 0	Admin 1	Admin 2	GAUL Code	Year	Under-5 mortality (per 1,000 livebirths)		
					Estimate	Lower bound, 95% UI	Upper bound, 95% UI
Egypt	Menoufia	Minuf	65876	2015	17.2	14.9	19.8
Egypt	Menoufia	Minuf City	65877	2000	35.1	31.5	38.8
Egypt	Menoufia	Minuf City	65877	2005	27.7	24.8	30.7
Egypt	Menoufia	Minuf City	65877	2010	21.8	19.3	25.0
Egypt	Menoufia	Minuf City	65877	2015	17.1	14.8	19.8
Egypt	Menoufia	Quwisna	65879	2000	34.9	32.1	37.9
Egypt	Menoufia	Quwisna	65879	2005	28.0	25.5	30.7
Egypt	Menoufia	Quwisna	65879	2010	22.8	20.6	25.5
Egypt	Menoufia	Quwisna	65879	2015	17.4	15.3	19.8
Egypt	Menoufia	Sadat City	65888	2000	39.5	34.5	44.9
Egypt	Menoufia	Sadat City	65888	2005	31.1	27.1	35.6
Egypt	Menoufia	Sadat City	65888	2010	25.1	21.8	29.1
Egypt	Menoufia	Sadat City	65888	2015	18.5	15.8	22.0
Egypt	Menoufia	Shibin al-Kum	65889	2000	34.1	31.0	37.3
Egypt	Menoufia	Shibin al-Kum	65889	2005	27.2	24.5	29.9
Egypt	Menoufia	Shibin al-Kum	65889	2010	21.9	19.5	24.8
Egypt	Menoufia	Shibin al-Kum	65889	2015	16.8	14.7	19.3
Egypt	Menoufia	Sirs Al-Layyana City	65891	2000	35.5	32.0	39.3
Egypt	Menoufia	Sirs Al-Layyana City	65891	2005	28.0	25.2	31.1
Egypt	Menoufia	Sirs Al-Layyana City	65891	2010	22.4	19.8	25.4
Egypt	Menoufia	Sirs Al-Layyana City	65891	2015	17.4	15.1	19.9
Egypt	Menoufia	Tala	65894	2000	33.7	30.5	37.1
Egypt	Menoufia	Tala	65894	2005	26.5	23.8	29.3
Egypt	Menoufia	Tala	65894	2010	21.1	18.8	23.9
Egypt	Menoufia	Tala	65894	2015	16.4	14.2	18.9
Egypt	New Valley	A-Dakhla Oasis	65896	2000	52.8	44.7	62.0
Egypt	New Valley	A-Dakhla Oasis	65896	2005	52.1	44.3	60.9
Egypt	New Valley	A-Dakhla Oasis	65896	2010	45.8	38.9	53.9
Egypt	New Valley	A-Dakhla Oasis	65896	2015	31.9	26.7	37.9
Egypt	New Valley	Al Farafra Oasis	65898	2000	48.5	38.6	59.3
Egypt	New Valley	Al Farafra Oasis	65898	2005	46.4	37.2	56.3
Egypt	New Valley	Al Farafra Oasis	65898	2010	40.4	31.8	49.5
Egypt	New Valley	Al Farafra Oasis	65898	2015	28.4	22.6	35.1
Egypt	New Valley	Al-Kharga Oasis	65897	2000	50.5	43.6	58.4
Egypt	New Valley	Al-Kharga Oasis	65897	2005	45.2	38.6	52.8
Egypt	New Valley	Al-Kharga Oasis	65897	2010	37.9	32.2	44.6
Egypt	New Valley	Al-Kharga Oasis	65897	2015	26.3	21.9	31.3
Egypt	New Valley	Paris Paris	65903	2000	58.9	46.1	73.5
Egypt	New Valley	Paris Paris	65903	2005	55.9	43.6	69.2
Egypt	New Valley	Paris Paris	65903	2010	46.8	36.6	58.3
Egypt	New Valley	Paris Paris	65903	2015	33.5	26.3	41.5
Egypt	North Sinai	Al-Hasna	65905	2000	41.2	33.5	50.6
Egypt	North Sinai	Al-Hasna	65905	2005	37.5	30.2	46.5
Egypt	North Sinai	Al-Hasna	65905	2010	32.3	25.9	40.0
Egypt	North Sinai	Al-Hasna	65905	2015	23.1	18.5	28.6
Egypt	North Sinai	Bir Al-Abd	65907	2000	37.8	31.4	44.8
Egypt	North Sinai	Bir Al-Abd	65907	2005	34.1	28.2	40.7
Egypt	North Sinai	Bir Al-Abd	65907	2010	29.8	24.5	36.2
Egypt	North Sinai	Bir Al-Abd	65907	2015	21.4	17.5	26.2
Egypt	North Sinai	El Arish 1	65913	2000	40.1	31.4	50.1
Egypt	North Sinai	El Arish 1	65913	2005	37.1	28.9	46.9
Egypt	North Sinai	El Arish 1	65913	2010	32.3	25.2	40.9
Egypt	North Sinai	El Arish 1	65913	2015	22.9	17.3	28.9
Egypt	North Sinai	El Arish 2	65914	2000	40.2	31.5	50.0
Egypt	North Sinai	El Arish 2	65914	2005	37.1	29.1	46.5
Egypt	North Sinai	El Arish 2	65914	2010	32.3	25.2	40.7
Egypt	North Sinai	El Arish 2	65914	2015	23.2	17.6	29.3
Egypt	North Sinai	El Arish 3	65922	2000	41.4	32.6	51.7
Egypt	North Sinai	El Arish 3	65922	2005	37.9	29.8	47.8
Egypt	North Sinai	El Arish 3	65922	2010	33.0	25.9	41.4
Egypt	North Sinai	El Arish 3	65922	2015	23.1	17.7	29.2
Egypt	North Sinai	El Arish 4	65925	2000	39.9	31.7	49.0
Egypt	North Sinai	El Arish 4	65925	2005	36.9	29.4	45.8
Egypt	North Sinai	El Arish 4	65925	2010	32.2	25.3	40.0
Egypt	North Sinai	El Arish 4	65925	2015	23.3	17.8	29.3
Egypt	North Sinai	Nakhl	65927	2000	44.2	35.7	54.6
Egypt	North Sinai	Nakhl	65927	2005	40.7	32.6	51.0
Egypt	North Sinai	Nakhl	65927	2010	35.5	28.5	44.3
Egypt	North Sinai	Nakhl	65927	2015	25.2	20.0	31.2
Egypt	North Sinai	Qasima	65928	2000	45.9	36.2	58.4
Egypt	North Sinai	Qasima	65928	2005	41.8	32.4	53.6
Egypt	North Sinai	Qasima	65928	2010	36.1	28.1	46.2
Egypt	North Sinai	Qasima	65928	2015	25.5	19.4	32.5
Egypt	North Sinai	Rafah	65929	2000	44.3	35.2	55.7
Egypt	North Sinai	Rafah	65929	2005	39.8	31.2	50.7
Egypt	North Sinai	Rafah	65929	2010	34.2	26.3	43.5
Egypt	North Sinai	Rafah	65929	2015	24.2	18.3	30.7
Egypt	North Sinai	Rummana	65931	2000	36.0	30.9	42.2
Egypt	North Sinai	Rummana	65931	2005	31.9	27.2	37.2
Egypt	North Sinai	Rummana	65931	2010	28.1	23.5	33.3
Egypt	North Sinai	Rummana	65931	2015	19.9	16.4	23.8
Egypt	North Sinai	Shaykh Zuwayd	65932	2000	43.2	34.2	53.9
Egypt	North Sinai	Shaykh Zuwayd	65932	2005	39.2	30.9	49.3
Egypt	North Sinai	Shaykh Zuwayd	65932	2010	34.0	26.6	42.8
Egypt	North Sinai	Shaykh Zuwayd	65932	2015	23.7	18.2	29.8

Admin 0	Admin 1	Admin 2	GAUL Code	Year	Under-5 mortality (per 1,000 livebirths)		
					Estimate	Lower bound, 95% UI	Upper bound, 95% UI
Egypt	Port Said	Al-Arab	65933	2000	29.6	25.9	33.8
Egypt	Port Said	Al-Arab	65933	2005	27.4	23.7	31.3
Egypt	Port Said	Al-Arab	65933	2010	25.7	21.9	29.8
Egypt	Port Said	Al-Arab	65933	2015	18.5	15.6	21.8
Egypt	Port Said	Al-Dawahy	65935	2000	30.5	26.8	34.5
Egypt	Port Said	Al-Dawahy	65935	2005	27.7	24.1	31.6
Egypt	Port Said	Al-Dawahy	65935	2010	25.7	22.1	29.7
Egypt	Port Said	Al-Dawahy	65935	2015	18.8	15.9	22.0
Egypt	Port Said	Al-Ganoub	65939	2000	31.0	27.3	34.9
Egypt	Port Said	Al-Ganoub	65939	2005	27.9	24.3	31.7
Egypt	Port Said	Al-Ganoub	65939	2010	25.9	22.3	29.6
Egypt	Port Said	Al-Ganoub	65939	2015	18.7	15.9	21.8
Egypt	Port Said	Al-Ganoub 2	65940	2000	34.9	30.4	39.5
Egypt	Port Said	Al-Ganoub 2	65940	2005	30.5	26.5	34.8
Egypt	Port Said	Al-Ganoub 2	65940	2010	27.0	23.3	31.0
Egypt	Port Said	Al-Ganoub 2	65940	2015	19.5	16.6	22.7
Egypt	Port Said	Al-Manasra	65942	2000	31.1	27.5	35.1
Egypt	Port Said	Al-Manasra	65942	2005	27.4	24.1	30.9
Egypt	Port Said	Al-Manasra	65942	2010	25.6	22.3	29.2
Egypt	Port Said	Al-Manasra	65942	2015	18.1	15.5	21.1
Egypt	Port Said	Al-Munakh	65944	2000	30.2	26.4	34.2
Egypt	Port Said	Al-Munakh	65944	2005	27.4	23.7	31.3
Egypt	Port Said	Al-Munakh	65944	2010	25.5	21.9	29.4
Egypt	Port Said	Al-Munakh	65944	2015	18.8	15.9	22.3
Egypt	Port Said	Al-Sharq	65946	2000	29.6	25.9	33.8
Egypt	Port Said	Al-Sharq	65946	2005	27.4	23.7	31.3
Egypt	Port Said	Al-Sharq	65946	2010	25.7	21.9	29.8
Egypt	Port Said	Al-Sharq	65946	2015	18.5	15.6	21.8
Egypt	Port Said	Al-Zohour	65949	2000	30.2	26.4	34.2
Egypt	Port Said	Al-Zohour	65949	2005	27.4	23.7	31.3
Egypt	Port Said	Al-Zohour	65949	2010	25.5	21.9	29.4
Egypt	Port Said	Al-Zohour	65949	2015	18.8	15.9	22.3
Egypt	Port Said	Mubark-Sharq Tafrea	65951	2000	33.1	29.0	37.5
Egypt	Port Said	Mubark-Sharq Tafrea	65951	2005	29.6	25.7	33.6
Egypt	Port Said	Mubark-Sharq Tafrea	65951	2010	26.5	22.9	30.4
Egypt	Port Said	Mubark-Sharq Tafrea	65951	2015	18.8	15.9	22.0
Egypt	Port Said	Police Department Port Said Port	65952	2000	29.6	25.9	33.8
Egypt	Port Said	Police Department Port Said Port	65952	2005	27.4	23.7	31.3
Egypt	Port Said	Police Department Port Said Port	65952	2010	25.7	21.9	29.8
Egypt	Port Said	Police Department Port Said Port	65952	2015	18.5	15.6	21.8
Egypt	Port Said	Port Fuad	65953	2000	30.1	26.3	34.2
Egypt	Port Said	Port Fuad	65953	2005	27.6	23.9	31.6
Egypt	Port Said	Port Fuad	65953	2010	25.6	21.9	29.5
Egypt	Port Said	Port Fuad	65953	2015	18.6	15.7	21.9
Egypt	Port Said	Port Fuad 2	65958	2000	30.2	26.4	34.3
Egypt	Port Said	Port Fuad 2	65958	2005	27.7	24.0	31.7
Egypt	Port Said	Port Fuad 2	65958	2010	25.7	22.0	29.6
Egypt	Port Said	Port Fuad 2	65958	2015	18.6	15.7	21.9
Egypt	Qena	Abu Tisht	65959	2000	65.8	59.4	72.8
Egypt	Qena	Abu Tisht	65959	2005	49.6	44.5	55.3
Egypt	Qena	Abu Tisht	65959	2010	36.8	32.3	41.7
Egypt	Qena	Abu Tisht	65959	2015	27.7	23.6	32.2
Egypt	Qena	Al Waqf	65963	2000	59.7	53.7	66.2
Egypt	Qena	Al Waqf	65963	2005	47.1	42.0	52.4
Egypt	Qena	Al Waqf	65963	2010	34.9	30.6	39.5
Egypt	Qena	Al Waqf	65963	2015	25.4	21.7	29.3
Egypt	Qena	Armant	65966	2000	55.3	48.9	61.9
Egypt	Qena	Armant	65966	2005	46.2	41.0	51.8
Egypt	Qena	Armant	65966	2010	34.4	29.9	39.2
Egypt	Qena	Armant	65966	2015	24.4	20.8	28.4
Egypt	Qena	Dishna	65973	2000	62.0	55.5	68.9
Egypt	Qena	Dishna	65973	2005	48.1	42.8	53.8
Egypt	Qena	Dishna	65973	2010	35.7	31.2	40.4
Egypt	Qena	Dishna	65973	2015	25.8	22.0	29.8
Egypt	Qena	Farshut	65974	2000	64.8	57.8	72.5
Egypt	Qena	Farshut	65974	2005	50.0	44.4	55.9
Egypt	Qena	Farshut	65974	2010	37.0	32.3	41.9
Egypt	Qena	Farshut	65974	2015	27.4	23.3	32.0
Egypt	Qena	Isna	65976	2000	58.5	51.4	66.5
Egypt	Qena	Isna	65976	2005	50.0	43.9	56.6
Egypt	Qena	Isna	65976	2010	35.8	30.9	40.8
Egypt	Qena	Isna	65976	2015	25.4	21.8	29.7
Egypt	Qena	Nag Hammadi	65977	2000	63.0	56.6	69.7
Egypt	Qena	Nag Hammadi	65977	2005	48.9	43.6	54.3
Egypt	Qena	Nag Hammadi	65977	2010	36.1	31.6	40.7
Egypt	Qena	Nag Hammadi	65977	2015	26.3	22.4	30.8
Egypt	Qena	Naqada	65978	2000	56.9	50.9	63.8
Egypt	Qena	Naqada	65978	2005	46.2	41.4	52.0
Egypt	Qena	Naqada	65978	2010	34.3	30.1	38.8
Egypt	Qena	Naqada	65978	2015	24.5	21.0	28.4
Egypt	Qena	Qift	65979	2000	58.8	52.0	66.9
Egypt	Qena	Qift	65979	2005	46.3	40.8	52.7
Egypt	Qena	Qift	65979	2010	33.7	29.2	38.8
Egypt	Qena	Qift	65979	2015	24.4	20.5	28.4
Egypt	Qena	Qina	65980	2000	59.9	53.1	67.7

Admin 0	Admin 1	Admin 2	GAUL Code	Year	Under-5 mortality (per 1,000 livebirths)		
					Estimate	Lower bound, 95% UI	Upper bound, 95% UI
Egypt	Qena	Qina	65980	2005	47.6	41.9	54.2
Egypt	Qena	Qina	65980	2010	35.5	30.9	40.7
Egypt	Qena	Qina	65980	2015	25.2	21.3	29.5
Egypt	Qena	Qina City	65981	2000	62.1	54.4	71.5
Egypt	Qena	Qina City	65981	2005	48.9	42.6	56.4
Egypt	Qena	Qina City	65981	2010	37.4	32.1	43.5
Egypt	Qena	Qina City	65981	2015	25.2	21.2	29.7
Egypt	Qena	Qus	65983	2000	56.4	50.2	63.3
Egypt	Qena	Qus	65983	2005	45.6	40.7	51.4
Egypt	Qena	Qus	65983	2010	33.6	29.3	38.1
Egypt	Qena	Qus	65983	2015	24.2	20.6	28.0
Egypt	Qena	Zemam Out	65984	2000	62.2	57.3	67.4
Egypt	Qena	Zemam Out	65984	2005	49.2	45.0	53.5
Egypt	Qena	Zemam Out	65984	2010	36.5	32.5	40.5
Egypt	Qena	Zemam Out	65984	2015	26.1	22.5	29.6
Egypt	Red Sea	Hurghada 1	65985	2000	46.5	36.8	57.8
Egypt	Red Sea	Hurghada 1	65985	2005	43.0	33.6	54.2
Egypt	Red Sea	Hurghada 1	65985	2010	39.6	31.2	50.2
Egypt	Red Sea	Hurghada 1	65985	2015	27.6	21.5	34.6
Egypt	Red Sea	Hurghada 2	65987	2000	46.7	37.4	57.8
Egypt	Red Sea	Hurghada 2	65987	2005	42.4	33.3	52.4
Egypt	Red Sea	Hurghada 2	65987	2010	38.1	29.8	47.0
Egypt	Red Sea	Hurghada 2	65987	2015	26.5	20.7	32.9
Egypt	Red Sea	Marsa Alam	65989	2000	54.5	41.8	68.4
Egypt	Red Sea	Marsa Alam	65989	2005	53.0	40.9	66.7
Egypt	Red Sea	Marsa Alam	65989	2010	45.4	35.0	57.0
Egypt	Red Sea	Marsa Alam	65989	2015	33.8	26.0	42.7
Egypt	Red Sea	Qusir	65990	2000	49.6	38.7	62.6
Egypt	Red Sea	Qusir	65990	2005	47.2	37.1	59.5
Egypt	Red Sea	Qusir	65990	2010	42.0	32.5	53.6
Egypt	Red Sea	Qusir	65990	2015	29.3	22.6	36.8
Egypt	Red Sea	Ras Gharib	65994	2000	43.3	34.1	54.5
Egypt	Red Sea	Ras Gharib	65994	2005	40.2	31.4	50.3
Egypt	Red Sea	Ras Gharib	65994	2010	35.0	27.5	44.3
Egypt	Red Sea	Ras Gharib	65994	2015	25.5	19.6	32.3
Egypt	Red Sea	Safaga	65995	2000	48.0	38.0	59.7
Egypt	Red Sea	Safaga	65995	2005	44.3	34.8	55.4
Egypt	Red Sea	Safaga	65995	2010	40.4	32.1	50.7
Egypt	Red Sea	Safaga	65995	2015	27.9	22.1	35.1
Egypt	Red Sea	Shallatin	65998	2000	56.6	44.4	71.9
Egypt	Red Sea	Shallatin	65998	2005	53.6	41.3	67.8
Egypt	Red Sea	Shallatin	65998	2010	45.5	34.7	58.4
Egypt	Red Sea	Shallatin	65998	2015	34.7	26.5	44.7
Egypt	Shrkia	10 Ramadan 1	65999	2000	35.8	32.0	40.1
Egypt	Shrkia	10 Ramadan 1	65999	2005	32.2	28.4	36.6
Egypt	Shrkia	10 Ramadan 1	65999	2010	27.7	24.3	31.6
Egypt	Shrkia	10 Ramadan 1	65999	2015	21.3	18.3	25.0
Egypt	Shrkia	10 Ramadan 2	66001	2000	36.3	32.5	40.8
Egypt	Shrkia	10 Ramadan 2	66001	2005	32.6	29.0	36.8
Egypt	Shrkia	10 Ramadan 2	66001	2010	28.0	24.7	32.0
Egypt	Shrkia	10 Ramadan 2	66001	2015	21.3	18.4	24.9
Egypt	Shrkia	Abu Hammad	66002	2000	37.0	33.5	40.9
Egypt	Shrkia	Abu Hammad	66002	2005	30.0	27.2	33.3
Egypt	Shrkia	Abu Hammad	66002	2010	25.4	22.5	28.5
Egypt	Shrkia	Abu Hammad	66002	2015	19.7	17.1	22.5
Egypt	Shrkia	Abu Kabir	66004	2000	35.4	32.2	38.9
Egypt	Shrkia	Abu Kabir	66004	2005	28.5	25.8	31.3
Egypt	Shrkia	Abu Kabir	66004	2010	24.6	21.8	27.5
Egypt	Shrkia	Abu Kabir	66004	2015	19.2	16.7	22.2
Egypt	Shrkia	Al-Husayniya	66006	2000	35.2	31.6	39.0
Egypt	Shrkia	Al-Husayniya	66006	2005	28.9	25.8	32.1
Egypt	Shrkia	Al-Husayniya	66006	2010	24.7	21.8	27.8
Egypt	Shrkia	Al-Husayniya	66006	2015	19.0	16.4	21.9
Egypt	Shrkia	Al-Qanayat	66007	2000	35.5	32.2	39.1
Egypt	Shrkia	Al-Qanayat	66007	2005	28.4	25.7	31.4
Egypt	Shrkia	Al-Qanayat	66007	2010	24.1	21.4	27.0
Egypt	Shrkia	Al-Qanayat	66007	2015	18.8	16.4	21.5
Egypt	Shrkia	Al-Salhiyya	66009	2000	36.7	33.0	41.1
Egypt	Shrkia	Al-Salhiyya	66009	2005	31.3	27.8	34.9
Egypt	Shrkia	Al-Salhiyya	66009	2010	26.3	23.2	29.5
Egypt	Shrkia	Al-Salhiyya	66009	2015	19.7	17.0	22.8
Egypt	Shrkia	Awlad Saqr	66010	2000	33.4	30.1	36.9
Egypt	Shrkia	Awlad Saqr	66010	2005	27.0	24.3	29.6
Egypt	Shrkia	Awlad Saqr	66010	2010	23.4	20.7	26.2
Egypt	Shrkia	Awlad Saqr	66010	2015	18.0	15.6	20.6
Egypt	Shrkia	Bilbis	66012	2000	37.3	34.1	40.9
Egypt	Shrkia	Bilbis	66012	2005	30.4	27.6	33.5
Egypt	Shrkia	Bilbis	66012	2010	25.4	22.8	28.3
Egypt	Shrkia	Bilbis	66012	2015	19.7	17.3	22.5
Egypt	Shrkia	Dyarb Nigm	66013	2000	34.3	31.3	37.5
Egypt	Shrkia	Dyarb Nigm	66013	2005	27.4	24.9	30.3
Egypt	Shrkia	Dyarb Nigm	66013	2010	23.2	20.7	26.0
Egypt	Shrkia	Dyarb Nigm	66013	2015	18.1	15.8	20.7
Egypt	Shrkia	El-Ibrahimiya	66014	2000	35.1	32.0	38.4
Egypt	Shrkia	El-Ibrahimiya	66014	2005	28.0	25.4	30.7

Admin 0	Admin 1	Admin 2	GAUL Code	Year	Under-5 mortality (per 1,000 livebirths)		
					Estimate	Lower bound, 95% UI	Upper bound, 95% UI
Egypt	Shrkia	El-Ibrahimiya	66014	2010	24.1	21.4	26.9
Egypt	Shrkia	El-Ibrahimiya	66014	2015	18.9	16.5	21.6
Egypt	Shrkia	Faqus	66015	2000	35.7	32.2	39.7
Egypt	Shrkia	Faqus	66015	2005	29.0	26.1	32.1
Egypt	Shrkia	Faqus	66015	2010	25.0	22.2	27.9
Egypt	Shrkia	Faqus	66015	2015	19.2	16.7	22.3
Egypt	Shrkia	Hihya	66016	2000	36.2	33.0	39.7
Egypt	Shrkia	Hihya	66016	2005	28.9	26.2	31.8
Egypt	Shrkia	Hihya	66016	2010	24.8	21.9	27.7
Egypt	Shrkia	Hihya	66016	2015	19.3	16.9	22.0
Egypt	Shrkia	Kafr Saqr	66017	2000	34.4	31.4	37.8
Egypt	Shrkia	Kafr Saqr	66017	2005	27.5	25.1	30.3
Egypt	Shrkia	Kafr Saqr	66017	2010	23.8	21.2	26.7
Egypt	Shrkia	Kafr Saqr	66017	2015	18.5	16.2	21.3
Egypt	Shrkia	Mashtul Al-Suq	66018	2000	37.9	34.9	41.2
Egypt	Shrkia	Mashtul Al-Suq	66018	2005	30.3	27.7	33.0
Egypt	Shrkia	Mashtul Al-Suq	66018	2010	24.5	22.1	27.2
Egypt	Shrkia	Mashtul Al-Suq	66018	2015	19.3	17.0	21.8
Egypt	Shrkia	Minya al-Qamh	66019	2000	36.8	33.5	40.4
Egypt	Shrkia	Minya al-Qamh	66019	2005	29.3	26.6	32.2
Egypt	Shrkia	Minya al-Qamh	66019	2010	24.5	22.0	27.3
Egypt	Shrkia	Minya al-Qamh	66019	2015	18.9	16.6	21.4
Egypt	Shrkia	Qurin	66020	2000	36.5	32.9	40.6
Egypt	Shrkia	Qurin	66020	2005	29.8	26.8	33.0
Egypt	Shrkia	Qurin	66020	2010	25.6	22.6	29.0
Egypt	Shrkia	Qurin	66020	2015	19.8	17.1	22.9
Egypt	Shrkia	Zaqaziq	66022	2000	36.3	32.9	39.8
Egypt	Shrkia	Zaqaziq	66022	2005	29.0	26.2	32.0
Egypt	Shrkia	Zaqaziq	66022	2010	24.6	21.9	27.5
Egypt	Shrkia	Zaqaziq	66022	2015	19.1	16.7	21.8
Egypt	Shrkia	Zaqaziq 1	66025	2000	35.5	32.2	39.1
Egypt	Shrkia	Zaqaziq 1	66025	2005	28.9	26.1	32.0
Egypt	Shrkia	Zaqaziq 1	66025	2010	24.8	22.0	27.7
Egypt	Shrkia	Zaqaziq 1	66025	2015	19.1	16.7	22.0
Egypt	Shrkia	Zaqaziq 2	66026	2000	35.5	32.2	39.1
Egypt	Shrkia	Zaqaziq 2	66026	2005	28.9	26.1	32.0
Egypt	Shrkia	Zaqaziq 2	66026	2010	24.8	22.0	27.7
Egypt	Shrkia	Zaqaziq 2	66026	2015	19.1	16.7	22.0
Egypt	Shrkia	Zemam Out	66027	2000	37.1	34.0	40.6
Egypt	Shrkia	Zemam Out	66027	2005	31.5	28.6	34.7
Egypt	Shrkia	Zemam Out	66027	2010	26.7	24.0	29.7
Egypt	Shrkia	Zemam Out	66027	2015	20.3	17.8	23.4
Egypt	South Sinai	Abu Radis	66028	2000	41.7	33.0	51.4
Egypt	South Sinai	Abu Radis	66028	2005	38.6	30.9	47.7
Egypt	South Sinai	Abu Radis	66028	2010	32.9	26.2	41.1
Egypt	South Sinai	Abu Radis	66028	2015	24.0	18.8	30.2
Egypt	South Sinai	Al-Tur	66029	2000	43.3	33.7	54.4
Egypt	South Sinai	Al-Tur	66029	2005	41.2	32.1	51.9
Egypt	South Sinai	Al-Tur	66029	2010	36.3	28.1	45.8
Egypt	South Sinai	Al-Tur	66029	2015	26.0	20.0	33.2
Egypt	South Sinai	Dahab	66030	2000	44.6	33.8	58.5
Egypt	South Sinai	Dahab	66030	2005	43.6	32.4	56.7
Egypt	South Sinai	Dahab	66030	2010	38.6	28.8	50.2
Egypt	South Sinai	Dahab	66030	2015	27.5	20.7	35.6
Egypt	South Sinai	Nuweiba	66032	2000	43.1	33.1	55.1
Egypt	South Sinai	Nuweiba	66032	2005	42.5	32.3	54.7
Egypt	South Sinai	Nuweiba	66032	2010	37.4	28.5	48.5
Egypt	South Sinai	Nuweiba	66032	2015	27.0	20.9	34.8
Egypt	South Sinai	Ras Sidr	66035	2000	37.8	31.6	44.6
Egypt	South Sinai	Ras Sidr	66035	2005	35.3	29.4	41.9
Egypt	South Sinai	Ras Sidr	66035	2010	30.0	25.0	35.7
Egypt	South Sinai	Ras Sidr	66035	2015	22.1	18.2	26.5
Egypt	South Sinai	Sant Katrin	66039	2000	45.4	35.2	58.2
Egypt	South Sinai	Sant Katrin	66039	2005	42.5	32.5	54.7
Egypt	South Sinai	Sant Katrin	66039	2010	36.6	28.1	47.3
Egypt	South Sinai	Sant Katrin	66039	2015	26.3	20.1	33.9
Egypt	South Sinai	Sharm el-Sheikh	66041	2000	44.9	33.8	58.1
Egypt	South Sinai	Sharm el-Sheikh	66041	2005	43.4	33.1	55.9
Egypt	South Sinai	Sharm el-Sheikh	66041	2010	39.0	29.7	49.5
Egypt	South Sinai	Sharm el-Sheikh	66041	2015	28.3	21.4	36.0
Egypt	South Sinai	Taba	66042	2000	43.5	32.7	56.5
Egypt	South Sinai	Taba	66042	2005	43.5	32.6	56.8
Egypt	South Sinai	Taba	66042	2010	38.8	29.2	50.9
Egypt	South Sinai	Taba	66042	2015	27.7	20.9	36.5
Egypt	Suez	Al-Arbiin	66043	2000	35.2	30.3	40.1
Egypt	Suez	Al-Arbiin	66043	2005	31.8	27.5	36.3
Egypt	Suez	Al-Arbiin	66043	2010	26.2	22.5	30.4
Egypt	Suez	Al-Arbiin	66043	2015	19.5	16.4	23.1
Egypt	Suez	Al-Ganayin	66044	2000	35.8	31.4	40.6
Egypt	Suez	Al-Ganayin	66044	2005	32.3	28.1	36.5
Egypt	Suez	Al-Ganayin	66044	2010	26.5	23.0	30.3
Egypt	Suez	Al-Ganayin	66044	2015	19.5	16.4	22.8
Egypt	Suez	Ataqa	66047	2000	38.2	33.0	43.7
Egypt	Suez	Ataqa	66047	2005	34.6	29.8	39.4
Egypt	Suez	Ataqa	66047	2010	28.8	24.8	33.1

Admin 0	Admin 1	Admin 2	GAUL Code	Year	Under-5 mortality (per 1,000 livebirths)		
					Estimate	Lower bound, 95% UI	Upper bound, 95% UI
Egypt	Suez	Ataqa	66047	2015	21.5	18.1	25.3
Egypt	Suez	Faysal	66048	2000	35.5	31.2	40.1
Egypt	Suez	Faysal	66048	2005	32.3	28.3	36.6
Egypt	Suez	Faysal	66048	2010	26.7	23.4	30.4
Egypt	Suez	Faysal	66048	2015	20.0	16.9	23.4
Egypt	Suez	Port Suez Police Department	66049	2000	36.6	31.4	41.8
Egypt	Suez	Port Suez Police Department	66049	2005	33.3	28.7	38.1
Egypt	Suez	Port Suez Police Department	66049	2010	27.7	23.7	32.2
Egypt	Suez	Port Suez Police Department	66049	2015	20.5	17.2	24.2
Egypt	Suez	Suez	66052	2000	35.7	30.9	40.7
Egypt	Suez	Suez	66052	2005	32.5	28.1	37.3
Egypt	Suez	Suez	66052	2010	27.2	23.5	31.4
Egypt	Suez	Suez	66052	2015	19.8	16.6	23.4
Egypt	Suhag	Akhmim	66054	2000	65.1	58.2	73.1
Egypt	Suhag	Akhmim	66054	2005	48.1	42.5	53.8
Egypt	Suhag	Akhmim	66054	2010	36.4	32.2	41.2
Egypt	Suhag	Akhmim	66054	2015	28.0	23.7	32.7
Egypt	Suhag	Akhmim City	66055	2000	64.3	58.1	71.8
Egypt	Suhag	Akhmim City	66055	2005	49.1	43.7	55.0
Egypt	Suhag	Akhmim City	66055	2010	37.7	33.3	42.7
Egypt	Suhag	Akhmim City	66055	2015	27.0	23.1	31.4
Egypt	Suhag	Al Usayrat	66062	2000	64.6	58.4	71.9
Egypt	Suhag	Al Usayrat	66062	2005	48.0	43.1	53.6
Egypt	Suhag	Al Usayrat	66062	2010	36.4	32.1	41.3
Egypt	Suhag	Al Usayrat	66062	2015	28.1	24.0	32.5
Egypt	Suhag	Al-Balyana	66056	2000	66.2	59.4	73.3
Egypt	Suhag	Al-Balyana	66056	2005	49.2	43.8	55.1
Egypt	Suhag	Al-Balyana	66056	2010	37.1	32.4	42.2
Egypt	Suhag	Al-Balyana	66056	2015	28.2	24.2	33.0
Egypt	Suhag	Al-Maragha	66059	2000	64.9	58.1	72.2
Egypt	Suhag	Al-Maragha	66059	2005	48.2	42.8	53.9
Egypt	Suhag	Al-Maragha	66059	2010	36.8	32.3	41.7
Egypt	Suhag	Al-Maragha	66059	2015	28.3	24.1	32.9
Egypt	Suhag	Al-Minshat	66061	2000	65.0	58.7	72.4
Egypt	Suhag	Al-Minshat	66061	2005	48.1	42.9	53.6
Egypt	Suhag	Al-Minshat	66061	2010	36.3	32.0	41.0
Egypt	Suhag	Al-Minshat	66061	2015	28.1	23.9	32.5
Egypt	Suhag	Dar al-Salam	66065	2000	67.8	60.9	74.4
Egypt	Suhag	Dar al-Salam	66065	2005	49.9	44.7	55.8
Egypt	Suhag	Dar al-Salam	66065	2010	37.4	32.8	42.2
Egypt	Suhag	Dar al-Salam	66065	2015	28.1	24.0	32.6
Egypt	Suhag	Girga	66066	2000	64.6	58.3	71.4
Egypt	Suhag	Girga	66066	2005	48.3	43.2	54.0
Egypt	Suhag	Girga	66066	2010	37.0	32.7	42.1
Egypt	Suhag	Girga	66066	2015	28.2	24.1	32.8
Egypt	Suhag	Guhayna Al-Gharbiyya	66067	2000	63.7	57.1	71.3
Egypt	Suhag	Guhayna Al-Gharbiyya	66067	2005	47.7	42.4	53.5
Egypt	Suhag	Guhayna Al-Gharbiyya	66067	2010	36.8	32.4	41.7
Egypt	Suhag	Guhayna Al-Gharbiyya	66067	2015	28.3	24.2	33.0
Egypt	Suhag	Kawther	66069	2000	64.3	57.6	72.1
Egypt	Suhag	Kawther	66069	2005	47.6	42.1	53.3
Egypt	Suhag	Kawther	66069	2010	36.1	31.9	40.9
Egypt	Suhag	Kawther	66069	2015	27.9	23.6	32.4
Egypt	Suhag	Kesm Tahta	66070	2000	64.5	57.5	72.7
Egypt	Suhag	Kesm Tahta	66070	2005	47.8	42.6	53.9
Egypt	Suhag	Kesm Tahta	66070	2010	36.7	32.2	41.7
Egypt	Suhag	Kesm Tahta	66070	2015	28.1	24.0	32.9
Egypt	Suhag	Saqulta	66072	2000	65.8	58.8	73.6
Egypt	Suhag	Saqulta	66072	2005	48.4	42.9	54.1
Egypt	Suhag	Saqulta	66072	2010	36.7	32.3	41.8
Egypt	Suhag	Saqulta	66072	2015	28.2	23.9	33.1
Egypt	Suhag	Suhag	66073	2000	64.6	57.9	71.6
Egypt	Suhag	Suhag	66073	2005	48.0	42.6	53.6
Egypt	Suhag	Suhag	66073	2010	36.5	32.2	41.5
Egypt	Suhag	Suhag	66073	2015	28.2	23.8	32.7
Egypt	Suhag	Suhag City	66085	2000	63.0	56.5	70.0
Egypt	Suhag	Suhag City	66085	2005	48.8	43.5	54.6
Egypt	Suhag	Suhag City	66085	2010	38.4	33.7	43.4
Egypt	Suhag	Suhag City	66085	2015	27.4	23.3	32.0
Egypt	Suhag	Suhag-2	66079	2000	63.6	57.0	70.9
Egypt	Suhag	Suhag-2	66079	2005	47.4	41.9	53.3
Egypt	Suhag	Suhag-2	66079	2010	36.2	32.0	41.2
Egypt	Suhag	Suhag-2	66079	2015	28.0	23.7	32.5
Egypt	Suhag	Tahta	66090	2000	65.3	58.3	73.4
Egypt	Suhag	Tahta	66090	2005	48.3	43.0	54.3
Egypt	Suhag	Tahta	66090	2010	37.1	32.5	42.2
Egypt	Suhag	Tahta	66090	2015	28.4	24.3	33.1
Egypt	Suhag	Tama	66091	2000	65.1	57.8	73.2
Egypt	Suhag	Tama	66091	2005	48.0	42.6	54.2
Egypt	Suhag	Tama	66091	2010	36.8	31.9	42.1
Egypt	Suhag	Tama	66091	2015	28.2	24.0	33.0
Egypt	Suhag	Zemam Out	66093	2000	64.8	58.8	71.1
Egypt	Suhag	Zemam Out	66093	2005	49.0	44.3	53.9
Egypt	Suhag	Zemam Out	66093	2010	38.1	33.8	42.7
Egypt	Suhag	Zemam Out	66093	2015	28.3	24.4	32.7

Admin 0	Admin 1	Admin 2	GAUL Code	Year	Under-5 mortality (per 1,000 livebirths)		
					Estimate	Lower bound, 95% UI	Upper bound, 95% UI
Equatorial Guinea	Bioko Norte	Bioko-Norte	15829	2000	101.1	84.4	120.3
Equatorial Guinea	Bioko Norte	Bioko-Norte	15829	2005	96.7	80.5	115.9
Equatorial Guinea	Bioko Norte	Bioko-Norte	15829	2010	85.4	70.4	104.2
Equatorial Guinea	Bioko Norte	Bioko-Norte	15829	2015	56.3	46.5	67.9
Equatorial Guinea	Bioko Sur	Bioko-Sur	15830	2000	106.0	87.6	127.3
Equatorial Guinea	Bioko Sur	Bioko-Sur	15830	2005	100.0	81.6	120.7
Equatorial Guinea	Bioko Sur	Bioko-Sur	15830	2010	89.5	72.5	108.7
Equatorial Guinea	Bioko Sur	Bioko-Sur	15830	2015	58.9	47.6	72.0
Equatorial Guinea	Centro Sur	Centro Sur	15831	2000	153.2	130.1	179.6
Equatorial Guinea	Centro Sur	Centro Sur	15831	2005	110.3	94.4	129.8
Equatorial Guinea	Centro Sur	Centro Sur	15831	2010	87.0	73.9	103.4
Equatorial Guinea	Centro Sur	Centro Sur	15831	2015	63.0	52.3	76.3
Equatorial Guinea	Kientem	Kientem	15832	2000	179.1	156.4	206.7
Equatorial Guinea	Kientem	Kientem	15832	2005	124.6	108.8	143.9
Equatorial Guinea	Kientem	Kientem	15832	2010	98.5	84.5	114.8
Equatorial Guinea	Kientem	Kientem	15832	2015	71.6	60.4	84.8
Equatorial Guinea	Litoral	Litoral	15833	2000	150.3	127.1	178.0
Equatorial Guinea	Litoral	Litoral	15833	2005	108.9	91.5	130.2
Equatorial Guinea	Litoral	Litoral	15833	2010	82.4	69.1	98.4
Equatorial Guinea	Litoral	Litoral	15833	2015	60.0	49.9	72.8
Equatorial Guinea	Welenzas	Welenzas	15834	2000	162.2	140.5	187.8
Equatorial Guinea	Welenzas	Welenzas	15834	2005	115.3	99.8	133.8
Equatorial Guinea	Welenzas	Welenzas	15834	2010	91.4	78.2	107.1
Equatorial Guinea	Welenzas	Welenzas	15834	2015	66.2	55.1	79.0
Eritrea	Anseba	Adi Tekeliezan	15835	2000	83.6	72.9	93.8
Eritrea	Anseba	Adi Tekeliezan	15835	2005	69.9	59.5	81.0
Eritrea	Anseba	Adi Tekeliezan	15835	2010	60.1	50.8	71.5
Eritrea	Anseba	Adi Tekeliezan	15835	2015	49.4	40.9	59.3
Eritrea	Anseba	Asmat	15836	2000	76.6	66.4	88.3
Eritrea	Anseba	Asmat	15836	2005	67.7	57.6	79.4
Eritrea	Anseba	Asmat	15836	2010	57.9	48.9	68.7
Eritrea	Anseba	Asmat	15836	2015	49.5	41.3	59.1
Eritrea	Anseba	Elabered	15837	2000	82.9	72.2	93.6
Eritrea	Anseba	Elabered	15837	2005	69.5	59.7	81.4
Eritrea	Anseba	Elabered	15837	2010	58.4	49.4	69.4
Eritrea	Anseba	Elabered	15837	2015	49.0	40.3	58.9
Eritrea	Anseba	Geleb	15838	2000	88.3	76.8	99.7
Eritrea	Anseba	Geleb	15838	2005	74.4	63.7	86.4
Eritrea	Anseba	Geleb	15838	2010	61.2	51.5	73.0
Eritrea	Anseba	Geleb	15838	2015	50.9	42.1	60.9
Eritrea	Anseba	Habero	15839	2000	82.2	71.0	94.9
Eritrea	Anseba	Habero	15839	2005	72.0	61.2	84.1
Eritrea	Anseba	Habero	15839	2010	61.3	51.2	72.9
Eritrea	Anseba	Habero	15839	2015	51.5	42.4	62.2
Eritrea	Anseba	Hagaz	15840	2000	83.5	72.7	94.4
Eritrea	Anseba	Hagaz	15840	2005	68.8	59.0	80.8
Eritrea	Anseba	Hagaz	15840	2010	59.0	49.7	70.3
Eritrea	Anseba	Hagaz	15840	2015	49.9	41.4	60.2
Eritrea	Anseba	Halhal	15841	2000	80.5	69.7	92.9
Eritrea	Anseba	Halhal	15841	2005	69.1	59.0	80.6
Eritrea	Anseba	Halhal	15841	2010	57.9	48.7	68.8
Eritrea	Anseba	Halhal	15841	2015	48.2	40.1	57.5
Eritrea	Anseba	Hamelmalo	15842	2000	85.0	72.8	96.8
Eritrea	Anseba	Hamelmalo	15842	2005	71.2	60.4	83.4
Eritrea	Anseba	Hamelmalo	15842	2010	59.1	49.6	70.3
Eritrea	Anseba	Hamelmalo	15842	2015	49.6	41.0	59.9
Eritrea	Anseba	Keren	15843	2000	76.7	65.5	87.6
Eritrea	Anseba	Keren	15843	2005	65.3	55.4	77.4
Eritrea	Anseba	Keren	15843	2010	55.9	46.8	67.0
Eritrea	Anseba	Keren	15843	2015	47.4	38.9	57.7
Eritrea	Anseba	Kerkebet	15844	2000	74.7	64.9	87.1
Eritrea	Anseba	Kerkebet	15844	2005	64.5	55.0	75.7
Eritrea	Anseba	Kerkebet	15844	2010	56.3	47.8	66.7
Eritrea	Anseba	Kerkebet	15844	2015	46.8	39.0	56.4
Eritrea	Anseba	Sel'a	15845	2000	71.6	62.7	82.4
Eritrea	Anseba	Sel'a	15845	2005	65.7	56.9	75.3
Eritrea	Anseba	Sel'a	15845	2010	55.6	47.9	64.4
Eritrea	Anseba	Sel'a	15845	2015	46.5	39.5	54.8
Eritrea	Archipelagos	Dahlak	15846	2000	113.0	88.9	144.7
Eritrea	Archipelagos	Dahlak	15846	2005	91.8	71.8	117.7
Eritrea	Archipelagos	Dahlak	15846	2010	84.0	65.5	109.8
Eritrea	Archipelagos	Dahlak	15846	2015	68.6	53.3	89.2
Eritrea	Debub	Adi Keih	15847	2000	103.6	91.2	117.1
Eritrea	Debub	Adi Keih	15847	2005	80.5	69.8	92.5
Eritrea	Debub	Adi Keih	15847	2010	69.6	59.7	81.6
Eritrea	Debub	Adi Keih	15847	2015	57.9	48.4	68.7
Eritrea	Debub	Adi Kuala	15848	2000	105.0	93.9	118.1
Eritrea	Debub	Adi Kuala	15848	2005	80.1	70.1	90.6
Eritrea	Debub	Adi Kuala	15848	2010	67.8	58.4	78.3
Eritrea	Debub	Adi Kuala	15848	2015	56.2	47.6	66.2
Eritrea	Debub	Areza	15849	2000	101.4	90.5	113.5
Eritrea	Debub	Areza	15849	2005	79.0	69.2	90.3
Eritrea	Debub	Areza	15849	2010	66.5	57.4	76.3
Eritrea	Debub	Areza	15849	2015	55.7	46.8	65.3
Eritrea	Debub	Dbarwa	15850	2000	93.3	83.3	104.6

Admin 0	Admin 1	Admin 2	GAUL Code	Year	Under-5 mortality (per 1,000 livebirths)		
					Estimate	Lower bound, 95% UI	Upper bound, 95% UI
Eritrea	Debub	Dbarwa	15850	2005	74.0	64.1	85.3
Eritrea	Debub	Dbarwa	15850	2010	64.2	55.0	75.2
Eritrea	Debub	Dbarwa	15850	2015	53.1	44.9	62.5
Eritrea	Debub	Dekemhare	15851	2000	91.8	81.5	102.2
Eritrea	Debub	Dekemhare	15851	2005	73.6	63.8	84.6
Eritrea	Debub	Dekemhare	15851	2010	64.9	55.5	75.9
Eritrea	Debub	Dekemhare	15851	2015	53.4	45.0	63.8
Eritrea	Debub	Emni Haili	15852	2000	104.2	93.1	116.8
Eritrea	Debub	Emni Haili	15852	2005	80.2	70.1	91.2
Eritrea	Debub	Emni Haili	15852	2010	67.6	58.0	78.2
Eritrea	Debub	Emni Haili	15852	2015	56.2	47.4	66.7
Eritrea	Debub	Mai Aini	15853	2000	102.1	91.3	114.4
Eritrea	Debub	Mai Aini	15853	2005	79.1	69.0	90.0
Eritrea	Debub	Mai Aini	15853	2010	68.5	59.3	79.3
Eritrea	Debub	Mai Aini	15853	2015	57.1	48.6	67.1
Eritrea	Debub	Mai Mne	15854	2000	109.0	97.4	122.6
Eritrea	Debub	Mai Mne	15854	2005	83.0	72.7	94.2
Eritrea	Debub	Mai Mne	15854	2010	70.4	60.6	81.0
Eritrea	Debub	Mai Mne	15854	2015	58.1	49.4	68.1
Eritrea	Debub	Mendefera	15855	2000	96.7	86.3	107.6
Eritrea	Debub	Mendefera	15855	2005	75.6	65.8	86.6
Eritrea	Debub	Mendefera	15855	2010	64.9	55.7	74.8
Eritrea	Debub	Mendefera	15855	2015	54.6	46.4	64.3
Eritrea	Debub	Segeneiti	15856	2000	99.1	87.1	112.6
Eritrea	Debub	Segeneiti	15856	2005	79.0	68.7	91.4
Eritrea	Debub	Segeneiti	15856	2010	68.1	58.0	79.7
Eritrea	Debub	Segeneiti	15856	2015	56.5	47.3	67.4
Eritrea	Debub	Senafe	15857	2000	111.1	98.2	125.6
Eritrea	Debub	Senafe	15857	2005	84.0	72.8	96.0
Eritrea	Debub	Senafe	15857	2010	71.5	61.5	83.0
Eritrea	Debub	Senafe	15857	2015	58.7	49.4	68.9
Eritrea	Debub	Tsorona	15858	2000	106.1	94.5	119.9
Eritrea	Debub	Tsorona	15858	2005	81.1	71.0	92.4
Eritrea	Debub	Tsorona	15858	2010	69.9	60.2	80.5
Eritrea	Debub	Tsorona	15858	2015	58.0	49.2	67.8
Eritrea	Debubawi Keih Bahri	Ara'eta	15859	2000	130.9	112.1	154.0
Eritrea	Debubawi Keih Bahri	Ara'eta	15859	2005	104.4	87.2	124.1
Eritrea	Debubawi Keih Bahri	Ara'eta	15859	2010	94.6	78.0	112.8
Eritrea	Debubawi Keih Bahri	Ara'eta	15859	2015	74.9	60.9	88.8
Eritrea	Debubawi Keih Bahri	Assab	15860	2000	114.9	95.5	135.3
Eritrea	Debubawi Keih Bahri	Assab	15860	2005	93.0	75.9	112.5
Eritrea	Debubawi Keih Bahri	Assab	15860	2010	85.4	69.1	103.1
Eritrea	Debubawi Keih Bahri	Assab	15860	2015	70.0	55.9	86.1
Eritrea	Debubawi Keih Bahri	Central Srs	15861	2000	135.1	114.6	160.2
Eritrea	Debubawi Keih Bahri	Central Srs	15861	2005	106.8	89.0	128.2
Eritrea	Debubawi Keih Bahri	Central Srs	15861	2010	96.4	78.7	116.6
Eritrea	Debubawi Keih Bahri	Central Srs	15861	2015	76.9	62.2	93.5
Eritrea	Debubawi Keih Bahri	Southern Srs	15862	2000	130.7	114.7	148.7
Eritrea	Debubawi Keih Bahri	Southern Srs	15862	2005	102.9	88.0	119.2
Eritrea	Debubawi Keih Bahri	Southern Srs	15862	2010	92.6	77.7	108.7
Eritrea	Debubawi Keih Bahri	Southern Srs	15862	2015	74.7	61.7	89.2
Eritrea	Gash Barka	Akurdet	15863	2000	86.7	74.1	101.7
Eritrea	Gash Barka	Akurdet	15863	2005	70.1	59.8	82.8
Eritrea	Gash Barka	Akurdet	15863	2010	60.9	50.4	72.5
Eritrea	Gash Barka	Akurdet	15863	2015	51.4	42.4	62.0
Eritrea	Gash Barka	Barentu	15864	2000	102.9	87.8	120.8
Eritrea	Gash Barka	Barentu	15864	2005	78.8	66.7	93.4
Eritrea	Gash Barka	Barentu	15864	2010	67.2	55.8	79.9
Eritrea	Gash Barka	Barentu	15864	2015	56.2	46.6	67.4
Eritrea	Gash Barka	Dge	15865	2000	85.3	73.4	98.6
Eritrea	Gash Barka	Dge	15865	2005	70.3	59.5	82.0
Eritrea	Gash Barka	Dge	15865	2010	61.2	51.5	72.6
Eritrea	Gash Barka	Dge	15865	2015	50.7	41.9	60.5
Eritrea	Gash Barka	Forto	15866	2000	80.6	69.6	92.1
Eritrea	Gash Barka	Forto	15866	2005	70.9	61.0	82.0
Eritrea	Gash Barka	Forto	15866	2010	61.7	52.8	71.6
Eritrea	Gash Barka	Forto	15866	2015	51.2	43.1	60.8
Eritrea	Gash Barka	Gonei	15867	2000	104.4	90.2	121.0
Eritrea	Gash Barka	Gonei	15867	2005	83.7	71.7	98.0
Eritrea	Gash Barka	Gonei	15867	2010	69.9	59.0	82.3
Eritrea	Gash Barka	Gonei	15867	2015	57.8	48.2	69.1
Eritrea	Gash Barka	Haikota	15868	2000	101.5	86.7	116.7
Eritrea	Gash Barka	Haikota	15868	2005	84.2	71.8	97.8
Eritrea	Gash Barka	Haikota	15868	2010	70.8	59.7	83.5
Eritrea	Gash Barka	Haikota	15868	2015	58.1	48.5	69.7
Eritrea	Gash Barka	La'elay Gash	15869	2000	118.8	104.3	136.4
Eritrea	Gash Barka	La'elay Gash	15869	2005	91.7	79.7	105.1
Eritrea	Gash Barka	La'elay Gash	15869	2010	76.4	64.9	88.7
Eritrea	Gash Barka	La'elay Gash	15869	2015	62.7	52.9	73.7
Eritrea	Gash Barka	Logo Anseba	15870	2000	83.8	74.5	94.7
Eritrea	Gash Barka	Logo Anseba	15870	2005	69.4	59.7	80.8
Eritrea	Gash Barka	Logo Anseba	15870	2010	60.2	51.2	71.1
Eritrea	Gash Barka	Logo Anseba	15870	2015	49.7	41.3	59.3
Eritrea	Gash Barka	Mensura	15871	2000	90.6	80.1	102.3
Eritrea	Gash Barka	Mensura	15871	2005	72.2	62.7	83.4

Admin 0	Admin 1	Admin 2	GAUL Code	Year	Under-5 mortality (per 1,000 livebirths)		
					Estimate	Lower bound, 95% UI	Upper bound, 95% UI
Eritrea	Gash Barka	Mensura	15871	2010	62.8	53.3	73.7
Eritrea	Gash Barka	Mensura	15871	2015	52.7	44.1	62.3
Eritrea	Gash Barka	Mogolo	15872	2000	99.0	86.0	115.1
Eritrea	Gash Barka	Mogolo	15872	2005	78.6	66.6	93.2
Eritrea	Gash Barka	Mogolo	15872	2010	66.3	55.7	78.1
Eritrea	Gash Barka	Mogolo	15872	2015	55.1	45.8	65.3
Eritrea	Gash Barka	Molqi	15873	2000	101.0	89.6	113.8
Eritrea	Gash Barka	Molqi	15873	2005	79.2	69.2	91.5
Eritrea	Gash Barka	Molqi	15873	2010	67.1	57.7	78.1
Eritrea	Gash Barka	Molqi	15873	2015	55.9	47.3	65.5
Eritrea	Gash Barka	Omhajer	15874	2000	117.0	102.1	136.8
Eritrea	Gash Barka	Omhajer	15874	2005	96.5	83.5	111.6
Eritrea	Gash Barka	Omhajer	15874	2010	81.7	70.3	95.0
Eritrea	Gash Barka	Omhajer	15874	2015	67.2	57.2	79.1
Eritrea	Gash Barka	Shambqo	15875	2000	107.3	92.5	123.6
Eritrea	Gash Barka	Shambqo	15875	2005	83.2	70.5	96.4
Eritrea	Gash Barka	Shambqo	15875	2010	70.1	58.8	82.7
Eritrea	Gash Barka	Shambqo	15875	2015	58.0	48.2	69.1
Eritrea	Gash Barka	Tesseney	15876	2000	99.3	85.0	114.6
Eritrea	Gash Barka	Tesseney	15876	2005	84.9	72.5	98.9
Eritrea	Gash Barka	Tesseney	15876	2010	72.5	62.0	84.8
Eritrea	Gash Barka	Tesseney	15876	2015	60.5	51.2	72.1
Eritrea	Maekel	Berik	15877	2000	75.9	67.4	85.2
Eritrea	Maekel	Berik	15877	2005	64.1	55.2	73.8
Eritrea	Maekel	Berik	15877	2010	58.6	49.9	69.3
Eritrea	Maekel	Berik	15877	2015	48.0	39.8	57.9
Eritrea	Maekel	Gala Nefhi	15878	2000	81.8	73.7	91.3
Eritrea	Maekel	Gala Nefhi	15878	2005	67.3	58.5	77.2
Eritrea	Maekel	Gala Nefhi	15878	2010	60.6	52.1	71.4
Eritrea	Maekel	Gala Nefhi	15878	2015	49.6	41.2	58.9
Eritrea	Maekel	Northern Asmara	15879	2000	68.9	60.8	77.2
Eritrea	Maekel	Northern Asmara	15879	2005	60.3	51.7	69.4
Eritrea	Maekel	Northern Asmara	15879	2010	57.6	49.2	68.2
Eritrea	Maekel	Northern Asmara	15879	2015	47.5	39.0	57.0
Eritrea	Maekel	Northern Merab	15880	2000	69.1	60.9	77.5
Eritrea	Maekel	Northern Merab	15880	2005	60.4	51.8	69.5
Eritrea	Maekel	Northern Merab	15880	2010	57.7	49.3	68.3
Eritrea	Maekel	Northern Merab	15880	2015	47.5	39.0	57.0
Eritrea	Maekel	Serejeqa	15881	2000	78.6	69.5	88.1
Eritrea	Maekel	Serejeqa	15881	2005	67.1	57.5	77.0
Eritrea	Maekel	Serejeqa	15881	2010	59.7	50.7	70.4
Eritrea	Maekel	Serejeqa	15881	2015	48.8	40.4	58.5
Eritrea	Maekel	Southern Asmara	15882	2000	71.2	63.2	79.8
Eritrea	Maekel	Southern Asmara	15882	2005	62.1	53.5	71.5
Eritrea	Maekel	Southern Asmara	15882	2010	58.6	50.3	69.2
Eritrea	Maekel	Southern Asmara	15882	2015	48.0	39.8	57.5
Eritrea	Maekel	Southern Merab	15883	2000	70.7	62.8	79.0
Eritrea	Maekel	Southern Merab	15883	2005	60.7	52.3	69.9
Eritrea	Maekel	Southern Merab	15883	2010	57.9	49.6	68.6
Eritrea	Maekel	Southern Merab	15883	2015	48.0	39.6	57.7
Eritrea	Semenawi Keih Bahri	Adobhe	15884	2000	89.3	76.0	105.3
Eritrea	Semenawi Keih Bahri	Adobhe	15884	2005	79.6	67.2	94.0
Eritrea	Semenawi Keih Bahri	Adobhe	15884	2010	65.2	54.5	77.3
Eritrea	Semenawi Keih Bahri	Adobhe	15884	2015	54.5	45.2	66.3
Eritrea	Semenawi Keih Bahri	Afabet	15885	2000	94.8	80.3	110.1
Eritrea	Semenawi Keih Bahri	Afabet	15885	2005	80.1	66.8	94.4
Eritrea	Semenawi Keih Bahri	Afabet	15885	2010	68.5	56.9	82.3
Eritrea	Semenawi Keih Bahri	Afabet	15885	2015	55.8	45.9	67.3
Eritrea	Semenawi Keih Bahri	Foro	15886	2000	110.9	96.0	128.7
Eritrea	Semenawi Keih Bahri	Foro	15886	2005	86.4	72.5	101.6
Eritrea	Semenawi Keih Bahri	Foro	15886	2010	75.8	62.4	89.5
Eritrea	Semenawi Keih Bahri	Foro	15886	2015	61.7	50.5	74.3
Eritrea	Semenawi Keih Bahri	Gel'alo	15887	2000	127.4	109.4	147.1
Eritrea	Semenawi Keih Bahri	Gel'alo	15887	2005	97.7	83.0	114.0
Eritrea	Semenawi Keih Bahri	Gel'alo	15887	2010	88.0	74.4	103.7
Eritrea	Semenawi Keih Bahri	Gel'alo	15887	2015	70.1	58.4	84.0
Eritrea	Semenawi Keih Bahri	Gindae	15888	2000	90.9	79.0	103.1
Eritrea	Semenawi Keih Bahri	Gindae	15888	2005	74.9	63.5	86.4
Eritrea	Semenawi Keih Bahri	Gindae	15888	2010	66.0	55.4	76.9
Eritrea	Semenawi Keih Bahri	Gindae	15888	2015	54.2	44.6	64.8
Eritrea	Semenawi Keih Bahri	Massawa	15889	2000	96.5	79.4	116.5
Eritrea	Semenawi Keih Bahri	Massawa	15889	2005	77.0	62.6	93.9
Eritrea	Semenawi Keih Bahri	Massawa	15889	2010	69.5	56.4	85.5
Eritrea	Semenawi Keih Bahri	Massawa	15889	2015	56.8	45.6	70.2
Eritrea	Semenawi Keih Bahri	Nakfa	15890	2000	89.5	75.6	104.2
Eritrea	Semenawi Keih Bahri	Nakfa	15890	2005	77.5	64.8	91.0
Eritrea	Semenawi Keih Bahri	Nakfa	15890	2010	63.8	53.1	76.1
Eritrea	Semenawi Keih Bahri	Nakfa	15890	2015	52.5	43.2	63.4
Eritrea	Semenawi Keih Bahri	Quarura	15891	2000	98.0	82.2	116.0
Eritrea	Semenawi Keih Bahri	Quarura	15891	2005	84.7	70.7	101.4
Eritrea	Semenawi Keih Bahri	Quarura	15891	2010	71.0	58.4	85.5
Eritrea	Semenawi Keih Bahri	Quarura	15891	2015	57.5	46.8	69.9
Eritrea	Semenawi Keih Bahri	Sheb	15892	2000	94.6	80.2	109.6
Eritrea	Semenawi Keih Bahri	Sheb	15892	2005	77.4	64.1	91.6
Eritrea	Semenawi Keih Bahri	Sheb	15892	2010	67.5	56.3	80.7

Admin 0	Admin 1	Admin 2	GAUL Code	Year	Under-5 mortality (per 1,000 livebirths)		
					Estimate	Lower bound, 95% UI	Upper bound, 95% UI
Eritrea	Semenawi Keih Bahri	Sheb	15892	2015	55.5	45.2	67.6
Ethiopia	Addis Ababa	Region 14	149277	2000	69.6	62.0	77.8
Ethiopia	Addis Ababa	Region 14	149277	2005	61.9	54.7	69.9
Ethiopia	Addis Ababa	Region 14	149277	2010	49.2	42.5	56.6
Ethiopia	Addis Ababa	Region 14	149277	2015	33.3	28.2	39.2
Ethiopia	Afar	Zone 1	40788	2000	132.9	119.3	148.2
Ethiopia	Afar	Zone 1	40788	2005	106.8	95.4	119.8
Ethiopia	Afar	Zone 1	40788	2010	78.2	68.2	88.6
Ethiopia	Afar	Zone 1	40788	2015	52.0	44.5	60.2
Ethiopia	Afar	Zone 2	40789	2000	128.6	115.7	142.4
Ethiopia	Afar	Zone 2	40789	2005	102.0	91.1	113.7
Ethiopia	Afar	Zone 2	40789	2010	76.8	67.8	86.7
Ethiopia	Afar	Zone 2	40789	2015	50.1	43.5	58.2
Ethiopia	Afar	Zone 3	40792	2000	147.5	133.9	162.0
Ethiopia	Afar	Zone 3	40792	2005	116.6	104.9	128.9
Ethiopia	Afar	Zone 3	40792	2010	83.0	74.2	92.7
Ethiopia	Afar	Zone 3	40792	2015	56.0	48.2	63.6
Ethiopia	Afar	Zone 4	40790	2000	141.8	127.1	157.8
Ethiopia	Afar	Zone 4	40790	2005	110.7	99.0	122.9
Ethiopia	Afar	Zone 4	40790	2010	81.2	70.9	91.3
Ethiopia	Afar	Zone 4	40790	2015	53.1	45.6	60.8
Ethiopia	Afar	Zone 5	40791	2000	149.4	135.1	164.7
Ethiopia	Afar	Zone 5	40791	2005	117.2	105.6	130.3
Ethiopia	Afar	Zone 5	40791	2010	83.2	73.0	94.2
Ethiopia	Afar	Zone 5	40791	2015	55.9	47.6	64.6
Ethiopia	Amhara	Awi/Agew	149278	2000	155.8	140.4	173.3
Ethiopia	Amhara	Awi/Agew	149278	2005	120.1	108.0	134.3
Ethiopia	Amhara	Awi/Agew	149278	2010	81.6	71.7	92.4
Ethiopia	Amhara	Awi/Agew	149278	2015	53.5	46.0	61.9
Ethiopia	Amhara	East Gojam	40796	2000	152.3	137.8	167.6
Ethiopia	Amhara	East Gojam	40796	2005	118.4	106.5	131.2
Ethiopia	Amhara	East Gojam	40796	2010	78.9	70.1	89.0
Ethiopia	Amhara	East Gojam	40796	2015	51.6	44.5	59.6
Ethiopia	Amhara	No Name	149279	2000	142.7	125.9	160.5
Ethiopia	Amhara	No Name	149279	2005	108.1	94.4	121.6
Ethiopia	Amhara	No Name	149279	2010	74.7	64.7	86.2
Ethiopia	Amhara	No Name	149279	2015	48.0	40.4	56.6
Ethiopia	Amhara	North Gonder	149280	2000	144.3	130.4	159.4
Ethiopia	Amhara	North Gonder	149280	2005	113.6	102.2	126.4
Ethiopia	Amhara	North Gonder	149280	2010	78.7	69.8	87.8
Ethiopia	Amhara	North Gonder	149280	2015	52.2	45.5	59.2
Ethiopia	Amhara	North Shewa(R3)	47682	2000	147.4	133.9	162.0
Ethiopia	Amhara	North Shewa(R3)	47682	2005	112.4	101.4	124.0
Ethiopia	Amhara	North Shewa(R3)	47682	2010	79.8	71.2	89.8
Ethiopia	Amhara	North Shewa(R3)	47682	2015	53.0	45.9	60.6
Ethiopia	Amhara	North Wollo	40799	2000	140.3	126.7	155.4
Ethiopia	Amhara	North Wollo	40799	2005	110.0	99.2	122.7
Ethiopia	Amhara	North Wollo	40799	2010	76.9	67.8	86.6
Ethiopia	Amhara	North Wollo	40799	2015	50.5	43.3	58.7
Ethiopia	Amhara	Oromia	40800	2000	147.5	133.6	162.3
Ethiopia	Amhara	Oromia	40800	2005	113.2	101.5	125.7
Ethiopia	Amhara	Oromia	40800	2010	80.8	70.4	92.2
Ethiopia	Amhara	Oromia	40800	2015	54.0	45.9	62.4
Ethiopia	Amhara	South Gonder	40801	2000	149.4	134.7	165.3
Ethiopia	Amhara	South Gonder	40801	2005	124.8	111.8	139.2
Ethiopia	Amhara	South Gonder	40801	2010	80.7	71.2	91.2
Ethiopia	Amhara	South Gonder	40801	2015	51.9	44.6	59.8
Ethiopia	Amhara	South Wollo	149281	2000	145.2	132.9	157.7
Ethiopia	Amhara	South Wollo	149281	2005	113.4	102.7	125.3
Ethiopia	Amhara	South Wollo	149281	2010	78.6	69.9	88.7
Ethiopia	Amhara	South Wollo	149281	2015	52.1	44.9	60.0
Ethiopia	Amhara	Special Woreda	149282	2000	142.1	127.3	157.8
Ethiopia	Amhara	Special Woreda	149282	2005	110.6	98.0	124.8
Ethiopia	Amhara	Special Woreda	149282	2010	79.3	68.5	91.4
Ethiopia	Amhara	Special Woreda	149282	2015	53.2	44.7	62.4
Ethiopia	Amhara	Wag Himra	40803	2000	138.4	123.4	154.1
Ethiopia	Amhara	Wag Himra	40803	2005	107.0	95.9	120.4
Ethiopia	Amhara	Wag Himra	40803	2010	75.9	66.5	86.3
Ethiopia	Amhara	Wag Himra	40803	2015	49.5	42.4	57.2
Ethiopia	Amhara	West Gojam	40804	2000	158.4	143.1	174.3
Ethiopia	Amhara	West Gojam	40804	2005	123.3	111.2	136.3
Ethiopia	Amhara	West Gojam	40804	2010	83.3	73.9	93.9
Ethiopia	Amhara	West Gojam	40804	2015	54.3	46.7	62.8
Ethiopia	Beneshangul Gumu	Asosa	40805	2000	184.8	170.7	199.0
Ethiopia	Beneshangul Gumu	Asosa	40805	2005	151.5	139.0	163.9
Ethiopia	Beneshangul Gumu	Asosa	40805	2010	100.1	90.3	110.7
Ethiopia	Beneshangul Gumu	Asosa	40805	2015	63.7	55.6	72.2
Ethiopia	Beneshangul Gumu	Kemashi	149283	2000	164.9	150.1	180.0
Ethiopia	Beneshangul Gumu	Kemashi	149283	2005	131.7	120.3	144.2
Ethiopia	Beneshangul Gumu	Kemashi	149283	2010	88.7	79.5	99.2
Ethiopia	Beneshangul Gumu	Kemashi	149283	2015	57.0	49.6	65.5
Ethiopia	Beneshangul Gumu	Metekel	40807	2000	166.1	153.1	180.1
Ethiopia	Beneshangul Gumu	Metekel	40807	2005	134.9	123.8	147.6
Ethiopia	Beneshangul Gumu	Metekel	40807	2010	88.9	79.6	98.8
Ethiopia	Beneshangul Gumu	Metekel	40807	2015	58.0	50.9	66.4

Admin 0	Admin 1	Admin 2	GAUL Code	Year	Under-5 mortality (per 1,000 livebirths)		
					Estimate	Lower bound, 95% UI	Upper bound, 95% UI
Ethiopia	Dire Dawa	Dire Dawa	40809	2000	114.0	103.8	125.0
Ethiopia	Dire Dawa	Dire Dawa	40809	2005	95.3	86.5	105.1
Ethiopia	Dire Dawa	Dire Dawa	40809	2010	68.8	61.4	77.2
Ethiopia	Dire Dawa	Dire Dawa	40809	2015	46.0	39.5	53.4
Ethiopia	Gambela	Agnuak	149284	2000	128.1	116.5	140.7
Ethiopia	Gambela	Agnuak	149284	2005	105.5	95.2	116.9
Ethiopia	Gambela	Agnuak	149284	2010	76.1	67.9	85.2
Ethiopia	Gambela	Agnuak	149284	2015	49.1	42.6	55.8
Ethiopia	Gambela	Majang	149285	2000	158.7	141.9	179.0
Ethiopia	Gambela	Majang	149285	2005	125.9	112.5	140.7
Ethiopia	Gambela	Majang	149285	2010	86.6	75.3	98.7
Ethiopia	Gambela	Majang	149285	2015	57.4	48.7	66.7
Ethiopia	Gambela	Nuer	149286	2000	106.8	96.1	118.5
Ethiopia	Gambela	Nuer	149286	2005	92.7	83.1	103.4
Ethiopia	Gambela	Nuer	149286	2010	69.0	61.2	78.0
Ethiopia	Gambela	Nuer	149286	2015	44.1	38.2	50.9
Ethiopia	Hareri	Hareri	40814	2000	114.6	105.1	125.0
Ethiopia	Hareri	Hareri	40814	2005	99.2	91.3	107.7
Ethiopia	Hareri	Hareri	40814	2010	69.4	61.9	77.1
Ethiopia	Hareri	Hareri	40814	2015	47.7	41.2	54.7
Ethiopia	Oromia	Arsi	149287	2000	131.0	117.6	147.6
Ethiopia	Oromia	Arsi	149287	2005	99.3	88.6	111.4
Ethiopia	Oromia	Arsi	149287	2010	72.2	63.0	81.1
Ethiopia	Oromia	Arsi	149287	2015	48.0	41.0	55.6
Ethiopia	Oromia	Bale	149288	2000	123.1	106.8	141.2
Ethiopia	Oromia	Bale	149288	2005	99.3	85.9	114.6
Ethiopia	Oromia	Bale	149288	2010	73.2	62.6	84.6
Ethiopia	Oromia	Bale	149288	2015	48.7	41.3	56.9
Ethiopia	Oromia	Borena	47688	2000	106.3	95.1	118.9
Ethiopia	Oromia	Borena	47688	2005	82.8	74.0	92.1
Ethiopia	Oromia	Borena	47688	2010	59.3	52.5	68.0
Ethiopia	Oromia	Borena	47688	2015	39.4	34.3	45.3
Ethiopia	Oromia	East Harerge	47689	2000	153.6	142.2	166.5
Ethiopia	Oromia	East Harerge	47689	2005	122.8	113.7	132.8
Ethiopia	Oromia	East Harerge	47689	2010	85.3	77.3	94.2
Ethiopia	Oromia	East Harerge	47689	2015	56.1	49.2	63.9
Ethiopia	Oromia	East Shewa	149289	2000	128.3	114.9	142.3
Ethiopia	Oromia	East Shewa	149289	2005	94.5	84.7	105.4
Ethiopia	Oromia	East Shewa	149289	2010	66.1	57.9	74.5
Ethiopia	Oromia	East Shewa	149289	2015	44.0	38.1	50.9
Ethiopia	Oromia	East Wellega	149290	2000	141.5	125.4	157.6
Ethiopia	Oromia	East Wellega	149290	2005	103.9	92.5	116.6
Ethiopia	Oromia	East Wellega	149290	2010	72.3	63.3	82.6
Ethiopia	Oromia	East Wellega	149290	2015	46.7	40.0	54.1
Ethiopia	Oromia	Guji	47691	2000	118.4	104.2	133.1
Ethiopia	Oromia	Guji	47691	2005	92.1	81.7	103.4
Ethiopia	Oromia	Guji	47691	2010	66.5	58.1	76.2
Ethiopia	Oromia	Guji	47691	2015	43.0	37.1	49.9
Ethiopia	Oromia	Horo Guduru	149291	2000	134.4	118.7	153.1
Ethiopia	Oromia	Horo Guduru	149291	2005	98.8	87.3	113.2
Ethiopia	Oromia	Horo Guduru	149291	2010	68.4	59.7	78.7
Ethiopia	Oromia	Horo Guduru	149291	2015	44.0	37.4	51.9
Ethiopia	Oromia	Ilubabor	149292	2000	142.7	126.7	159.9
Ethiopia	Oromia	Ilubabor	149292	2005	107.2	95.5	120.4
Ethiopia	Oromia	Ilubabor	149292	2010	76.1	66.3	86.8
Ethiopia	Oromia	Ilubabor	149292	2015	48.6	41.4	56.4
Ethiopia	Oromia	Jimma	40822	2000	141.1	126.6	156.2
Ethiopia	Oromia	Jimma	40822	2005	103.4	92.2	115.3
Ethiopia	Oromia	Jimma	40822	2010	72.1	63.1	81.3
Ethiopia	Oromia	Jimma	40822	2015	47.4	40.6	54.5
Ethiopia	Oromia	Kelem Wellega	149293	2000	143.9	127.2	162.0
Ethiopia	Oromia	Kelem Wellega	149293	2005	111.4	97.6	125.8
Ethiopia	Oromia	Kelem Wellega	149293	2010	79.9	69.3	91.5
Ethiopia	Oromia	Kelem Wellega	149293	2015	52.0	44.1	60.5
Ethiopia	Oromia	North Shewa(R4)	47692	2000	148.8	134.0	163.9
Ethiopia	Oromia	North Shewa(R4)	47692	2005	109.0	97.6	121.0
Ethiopia	Oromia	North Shewa(R4)	47692	2010	75.3	66.6	84.8
Ethiopia	Oromia	North Shewa(R4)	47692	2015	49.6	42.8	57.4
Ethiopia	Oromia	South West Shewa	47693	2000	120.3	107.2	134.0
Ethiopia	Oromia	South West Shewa	47693	2005	87.7	78.3	98.3
Ethiopia	Oromia	South West Shewa	47693	2010	61.6	53.7	70.4
Ethiopia	Oromia	South West Shewa	47693	2015	41.0	35.1	47.7
Ethiopia	Oromia	West Arsi	149294	2000	129.2	116.2	143.5
Ethiopia	Oromia	West Arsi	149294	2005	97.9	87.6	108.7
Ethiopia	Oromia	West Arsi	149294	2010	70.4	61.9	78.7
Ethiopia	Oromia	West Arsi	149294	2015	46.6	40.2	54.2
Ethiopia	Oromia	West Harerge	40824	2000	158.9	142.4	177.4
Ethiopia	Oromia	West Harerge	40824	2005	123.8	110.2	138.0
Ethiopia	Oromia	West Harerge	40824	2010	87.7	76.4	98.8
Ethiopia	Oromia	West Harerge	40824	2015	58.1	49.6	66.6
Ethiopia	Oromia	West Shewa	47694	2000	119.4	107.6	132.2
Ethiopia	Oromia	West Shewa	47694	2005	87.1	78.5	97.1
Ethiopia	Oromia	West Shewa	47694	2010	61.1	53.4	69.1
Ethiopia	Oromia	West Shewa	47694	2015	40.2	34.8	46.9
Ethiopia	Oromia	West Wellega	149295	2000	159.0	142.2	175.9

Admin 0	Admin 1	Admin 2	GAUL Code	Year	Under-5 mortality (per 1,000 livebirths)		
					Estimate	Lower bound, 95% UI	Upper bound, 95% UI
Ethiopia	Oromia	West Wellega	149295	2005	121.6	107.8	135.5
Ethiopia	Oromia	West Wellega	149295	2010	84.9	74.7	95.8
Ethiopia	Oromia	West Wellega	149295	2015	55.5	47.7	64.2
Ethiopia	SNNPR	Alaba	47695	2000	134.3	121.4	149.2
Ethiopia	SNNPR	Alaba	47695	2005	99.8	88.4	110.2
Ethiopia	SNNPR	Alaba	47695	2010	69.1	60.6	78.3
Ethiopia	SNNPR	Alaba	47695	2015	47.0	40.0	54.4
Ethiopia	SNNPR	Basketo	47696	2000	184.0	158.8	210.3
Ethiopia	SNNPR	Basketo	47696	2005	142.4	122.7	165.1
Ethiopia	SNNPR	Basketo	47696	2010	96.3	81.4	114.0
Ethiopia	SNNPR	Basketo	47696	2015	62.8	52.1	75.1
Ethiopia	SNNPR	Bench Maji	149296	2000	170.1	151.3	192.1
Ethiopia	SNNPR	Bench Maji	149296	2005	135.2	119.2	154.8
Ethiopia	SNNPR	Bench Maji	149296	2010	92.1	79.8	106.8
Ethiopia	SNNPR	Bench Maji	149296	2015	60.5	50.8	71.3
Ethiopia	SNNPR	Dawro	47698	2000	156.5	139.5	175.3
Ethiopia	SNNPR	Dawro	47698	2005	117.4	103.9	133.0
Ethiopia	SNNPR	Dawro	47698	2010	80.0	69.7	92.0
Ethiopia	SNNPR	Dawro	47698	2015	52.8	45.1	62.0
Ethiopia	SNNPR	Gamo Gofa	47699	2000	161.8	145.4	179.6
Ethiopia	SNNPR	Gamo Gofa	47699	2005	122.9	109.8	136.4
Ethiopia	SNNPR	Gamo Gofa	47699	2010	84.6	73.9	96.5
Ethiopia	SNNPR	Gamo Gofa	47699	2015	55.7	47.1	64.6
Ethiopia	SNNPR	Gedio	40831	2000	134.5	119.2	151.7
Ethiopia	SNNPR	Gedio	40831	2005	102.6	91.1	116.4
Ethiopia	SNNPR	Gedio	40831	2010	70.6	61.3	81.7
Ethiopia	SNNPR	Gedio	40831	2015	45.5	38.5	53.3
Ethiopia	SNNPR	Gurage	47700	2000	129.9	117.0	144.2
Ethiopia	SNNPR	Gurage	47700	2005	95.2	85.1	106.8
Ethiopia	SNNPR	Gurage	47700	2010	65.2	57.1	73.9
Ethiopia	SNNPR	Gurage	47700	2015	43.8	37.6	50.7
Ethiopia	SNNPR	Hadiya	40833	2000	133.4	121.0	147.7
Ethiopia	SNNPR	Hadiya	40833	2005	98.4	88.9	109.1
Ethiopia	SNNPR	Hadiya	40833	2010	67.3	59.7	76.2
Ethiopia	SNNPR	Hadiya	40833	2015	45.2	39.1	52.8
Ethiopia	SNNPR	KT	47702	2000	137.3	125.1	151.5
Ethiopia	SNNPR	KT	47702	2005	100.2	90.7	111.0
Ethiopia	SNNPR	KT	47702	2010	68.4	60.7	77.8
Ethiopia	SNNPR	KT	47702	2015	45.9	39.7	53.5
Ethiopia	SNNPR	Keffa	47701	2000	171.4	154.4	189.5
Ethiopia	SNNPR	Keffa	47701	2005	131.7	118.1	146.9
Ethiopia	SNNPR	Keffa	47701	2010	88.9	78.5	100.4
Ethiopia	SNNPR	Keffa	47701	2015	58.0	49.8	66.8
Ethiopia	SNNPR	Konta	47703	2000	170.3	149.4	192.0
Ethiopia	SNNPR	Konta	47703	2005	130.7	114.7	148.4
Ethiopia	SNNPR	Konta	47703	2010	89.1	77.4	103.3
Ethiopia	SNNPR	Konta	47703	2015	58.1	49.4	67.5
Ethiopia	SNNPR	Segen Peoples'	149297	2000	131.4	114.4	151.1
Ethiopia	SNNPR	Segen Peoples'	149297	2005	102.1	88.9	117.6
Ethiopia	SNNPR	Segen Peoples'	149297	2010	70.9	60.3	83.3
Ethiopia	SNNPR	Segen Peoples'	149297	2015	47.3	39.5	56.0
Ethiopia	SNNPR	Selti	47705	2000	135.2	121.9	150.1
Ethiopia	SNNPR	Selti	47705	2005	99.8	88.8	111.8
Ethiopia	SNNPR	Selti	47705	2010	68.6	60.2	78.1
Ethiopia	SNNPR	Selti	47705	2015	46.2	39.5	54.5
Ethiopia	SNNPR	Sheka	47704	2000	161.7	145.7	180.3
Ethiopia	SNNPR	Sheka	47704	2005	126.1	113.5	140.7
Ethiopia	SNNPR	Sheka	47704	2010	85.9	75.4	97.4
Ethiopia	SNNPR	Sheka	47704	2015	56.1	48.2	64.8
Ethiopia	SNNPR	Sidama	40838	2000	132.3	120.8	145.8
Ethiopia	SNNPR	Sidama	40838	2005	99.9	90.7	110.2
Ethiopia	SNNPR	Sidama	40838	2010	69.2	61.4	77.5
Ethiopia	SNNPR	Sidama	40838	2015	46.1	39.7	53.1
Ethiopia	SNNPR	South Omo	40839	2000	156.4	136.1	177.9
Ethiopia	SNNPR	South Omo	40839	2005	122.1	106.9	140.9
Ethiopia	SNNPR	South Omo	40839	2010	84.0	72.4	97.8
Ethiopia	SNNPR	South Omo	40839	2015	55.3	46.5	65.2
Ethiopia	SNNPR	Wolayita	47706	2000	144.7	131.4	159.6
Ethiopia	SNNPR	Wolayita	47706	2005	106.8	96.6	117.7
Ethiopia	SNNPR	Wolayita	47706	2010	73.0	64.5	82.0
Ethiopia	SNNPR	Wolayita	47706	2015	49.1	42.3	56.8
Ethiopia	SNNPR	Yem	40840	2000	127.7	112.7	145.0
Ethiopia	SNNPR	Yem	40840	2005	93.7	81.9	106.2
Ethiopia	SNNPR	Yem	40840	2010	64.8	55.7	74.4
Ethiopia	SNNPR	Yem	40840	2015	43.3	36.6	50.9
Ethiopia	Somali	Afder	40841	2000	108.1	92.9	124.7
Ethiopia	Somali	Afder	40841	2005	97.3	83.6	112.6
Ethiopia	Somali	Afder	40841	2010	69.4	59.4	81.1
Ethiopia	Somali	Afder	40841	2015	45.3	38.3	53.4
Ethiopia	Somali	Doolo	40849	2000	105.8	90.3	123.7
Ethiopia	Somali	Doolo	40849	2005	102.0	86.9	119.0
Ethiopia	Somali	Doolo	40849	2010	74.3	62.8	87.7
Ethiopia	Somali	Doolo	40849	2015	44.8	37.3	54.0
Ethiopia	Somali	Fafan	40845	2000	115.7	105.7	126.3
Ethiopia	Somali	Fafan	40845	2005	98.9	90.4	108.2

Admin 0	Admin 1	Admin 2	GAUL Code	Year	Under-5 mortality (per 1,000 livebirths)		
					Estimate	Lower bound, 95% UI	Upper bound, 95% UI
Ethiopia	Somali	Fafan	40845	2010	67.8	60.5	75.5
Ethiopia	Somali	Fafan	40845	2015	43.7	37.7	50.1
Ethiopia	Somali	Jarar	40842	2000	106.2	91.1	122.4
Ethiopia	Somali	Jarar	40842	2005	98.1	83.5	113.7
Ethiopia	Somali	Jarar	40842	2010	68.5	58.3	80.5
Ethiopia	Somali	Jarar	40842	2015	41.6	34.9	49.5
Ethiopia	Somali	Korahe	47708	2000	103.0	83.1	126.8
Ethiopia	Somali	Korahe	47708	2005	95.1	77.0	117.6
Ethiopia	Somali	Korahe	47708	2010	68.5	55.4	85.1
Ethiopia	Somali	Korahe	47708	2015	45.0	36.0	56.3
Ethiopia	Somali	Liben	47709	2000	89.9	79.4	100.8
Ethiopia	Somali	Liben	47709	2005	76.6	67.4	85.4
Ethiopia	Somali	Liben	47709	2010	55.1	47.8	62.3
Ethiopia	Somali	Liben	47709	2015	37.6	32.0	43.8
Ethiopia	Somali	Nogob	47707	2000	122.3	99.7	147.0
Ethiopia	Somali	Nogob	47707	2005	104.7	86.2	126.6
Ethiopia	Somali	Nogob	47707	2010	74.6	60.7	89.5
Ethiopia	Somali	Nogob	47707	2015	48.6	38.4	59.1
Ethiopia	Somali	Shabelle	40844	2000	104.8	86.9	126.0
Ethiopia	Somali	Shabelle	40844	2005	94.9	78.0	113.1
Ethiopia	Somali	Shabelle	40844	2010	69.0	57.3	83.2
Ethiopia	Somali	Shabelle	40844	2015	44.9	36.7	55.0
Ethiopia	Somali	Siti	40848	2000	120.1	109.5	131.4
Ethiopia	Somali	Siti	40848	2005	98.4	89.8	107.2
Ethiopia	Somali	Siti	40848	2010	70.8	63.5	78.5
Ethiopia	Somali	Siti	40848	2015	45.8	39.7	52.1
Ethiopia	Tigray	Central	40850	2000	116.7	106.3	127.7
Ethiopia	Tigray	Central	40850	2005	87.7	78.8	96.5
Ethiopia	Tigray	Central	40850	2010	64.8	57.5	72.1
Ethiopia	Tigray	Central	40850	2015	43.1	37.6	49.1
Ethiopia	Tigray	Eastern	40851	2000	116.3	106.1	127.9
Ethiopia	Tigray	Eastern	40851	2005	87.7	78.1	96.9
Ethiopia	Tigray	Eastern	40851	2010	64.9	57.7	73.3
Ethiopia	Tigray	Eastern	40851	2015	43.6	37.6	50.2
Ethiopia	Tigray	North Western	47710	2000	122.9	110.6	137.0
Ethiopia	Tigray	North Western	47710	2005	94.8	84.7	105.6
Ethiopia	Tigray	North Western	47710	2010	68.7	60.2	77.9
Ethiopia	Tigray	North Western	47710	2015	45.7	39.5	53.4
Ethiopia	Tigray	Southern	149298	2000	125.8	114.1	137.6
Ethiopia	Tigray	Southern	149298	2005	94.2	84.9	103.2
Ethiopia	Tigray	Southern	149298	2010	69.5	61.6	77.9
Ethiopia	Tigray	Southern	149298	2015	45.6	39.6	52.1
Ethiopia	Tigray	Western	47711	2000	132.1	117.8	148.3
Ethiopia	Tigray	Western	47711	2005	105.5	93.4	119.1
Ethiopia	Tigray	Western	47711	2010	75.9	66.4	85.7
Ethiopia	Tigray	Western	47711	2015	51.0	43.8	58.8
Gabon	Estuaire	Komo	16357	2000	70.7	62.3	81.2
Gabon	Estuaire	Komo	16357	2005	64.8	56.6	74.8
Gabon	Estuaire	Komo	16357	2010	55.4	48.4	63.3
Gabon	Estuaire	Komo	16357	2015	47.7	40.2	55.3
Gabon	Estuaire	Komo-Mondah	16358	2000	68.5	61.1	77.0
Gabon	Estuaire	Komo-Mondah	16358	2005	63.9	57.0	72.0
Gabon	Estuaire	Komo-Mondah	16358	2010	55.2	48.9	62.3
Gabon	Estuaire	Komo-Mondah	16358	2015	45.2	38.7	52.5
Gabon	Estuaire	Libreville	16359	2000	62.1	54.7	69.9
Gabon	Estuaire	Libreville	16359	2005	60.1	53.2	67.9
Gabon	Estuaire	Libreville	16359	2010	54.1	47.1	61.9
Gabon	Estuaire	Libreville	16359	2015	43.8	36.8	52.0
Gabon	Estuaire	Noya	16360	2000	76.9	65.6	89.5
Gabon	Estuaire	Noya	16360	2005	74.0	63.3	86.0
Gabon	Estuaire	Noya	16360	2010	61.6	52.4	71.6
Gabon	Estuaire	Noya	16360	2015	51.9	43.4	62.2
Gabon	Haut-Ogooue	Bayi-Brikolo	16361	2000	97.3	83.6	112.6
Gabon	Haut-Ogooue	Bayi-Brikolo	16361	2005	93.8	81.5	108.6
Gabon	Haut-Ogooue	Bayi-Brikolo	16361	2010	72.6	62.4	84.3
Gabon	Haut-Ogooue	Bayi-Brikolo	16361	2015	56.6	47.9	66.7
Gabon	Haut-Ogooue	Djoue	16362	2000	86.5	74.9	99.8
Gabon	Haut-Ogooue	Djoue	16362	2005	84.9	73.8	98.2
Gabon	Haut-Ogooue	Djoue	16362	2010	66.0	57.0	77.1
Gabon	Haut-Ogooue	Djoue	16362	2015	52.0	44.4	61.2
Gabon	Haut-Ogooue	Djouori-Agnili	16363	2000	78.8	68.0	91.8
Gabon	Haut-Ogooue	Djouori-Agnili	16363	2005	70.0	60.5	80.4
Gabon	Haut-Ogooue	Djouori-Agnili	16363	2010	55.4	47.4	64.8
Gabon	Haut-Ogooue	Djouori-Agnili	16363	2015	45.8	38.8	53.6
Gabon	Haut-Ogooue	Lebombi-Leyou	16364	2000	66.4	56.8	77.2
Gabon	Haut-Ogooue	Lebombi-Leyou	16364	2005	62.2	53.1	72.1
Gabon	Haut-Ogooue	Lebombi-Leyou	16364	2010	49.8	42.6	58.2
Gabon	Haut-Ogooue	Lebombi-Leyou	16364	2015	41.7	34.8	49.5
Gabon	Haut-Ogooue	Lekabi-Lewolo	16365	2000	81.2	68.6	94.9
Gabon	Haut-Ogooue	Lekabi-Lewolo	16365	2005	73.0	61.9	84.8
Gabon	Haut-Ogooue	Lekabi-Lewolo	16365	2010	57.6	48.8	67.3
Gabon	Haut-Ogooue	Lekabi-Lewolo	16365	2015	46.7	39.2	55.2
Gabon	Haut-Ogooue	Lekoko	16366	2000	81.4	70.7	94.3
Gabon	Haut-Ogooue	Lekoko	16366	2005	74.1	64.1	85.4
Gabon	Haut-Ogooue	Lekoko	16366	2010	58.4	49.8	68.2

Admin 0	Admin 1	Admin 2	GAUL Code	Year	Under-5 mortality (per 1,000 livebirths)		
					Estimate	Lower bound, 95% UI	Upper bound, 95% UI
Gabon	Haut-Ogooue	Lekoko	16366	2015	49.6	42.2	58.6
Gabon	Haut-Ogooue	Lekoni-Lekori	16367	2000	82.7	70.8	96.9
Gabon	Haut-Ogooue	Lekoni-Lekori	16367	2005	76.6	65.5	89.3
Gabon	Haut-Ogooue	Lekoni-Lekori	16367	2010	60.0	51.1	69.9
Gabon	Haut-Ogooue	Lekoni-Lekori	16367	2015	47.3	40.1	55.9
Gabon	Haut-Ogooue	Ogooue-Letili	16368	2000	90.4	76.4	105.6
Gabon	Haut-Ogooue	Ogooue-Letili	16368	2005	79.6	67.8	91.9
Gabon	Haut-Ogooue	Ogooue-Letili	16368	2010	62.6	53.1	73.2
Gabon	Haut-Ogooue	Ogooue-Letili	16368	2015	52.9	44.0	63.0
Gabon	Haut-Ogooue	Passa	16369	2000	67.7	57.7	77.9
Gabon	Haut-Ogooue	Passa	16369	2005	60.7	51.5	70.6
Gabon	Haut-Ogooue	Passa	16369	2010	48.1	40.6	56.3
Gabon	Haut-Ogooue	Passa	16369	2015	40.5	33.9	48.1
Gabon	Haut-Ogooue	Plateaux	16370	2000	83.9	73.7	96.9
Gabon	Haut-Ogooue	Plateaux	16370	2005	74.5	65.5	85.1
Gabon	Haut-Ogooue	Plateaux	16370	2010	59.4	51.3	68.2
Gabon	Haut-Ogooue	Plateaux	16370	2015	48.9	41.7	57.0
Gabon	Haut-Ogooue	Sebe-Brikolo	16371	2000	89.1	76.9	102.2
Gabon	Haut-Ogooue	Sebe-Brikolo	16371	2005	84.1	72.9	96.2
Gabon	Haut-Ogooue	Sebe-Brikolo	16371	2010	67.1	58.1	77.4
Gabon	Haut-Ogooue	Sebe-Brikolo	16371	2015	54.5	46.6	63.4
Gabon	Moyen-Ogooue	Abanga-Bigne	16372	2000	72.8	63.2	84.4
Gabon	Moyen-Ogooue	Abanga-Bigne	16372	2005	64.2	55.7	74.2
Gabon	Moyen-Ogooue	Abanga-Bigne	16372	2010	55.2	47.5	63.6
Gabon	Moyen-Ogooue	Abanga-Bigne	16372	2015	48.3	40.5	56.5
Gabon	Moyen-Ogooue	Ogooue et Lacs	16373	2000	66.2	57.0	77.3
Gabon	Moyen-Ogooue	Ogooue et Lacs	16373	2005	59.1	50.9	67.8
Gabon	Moyen-Ogooue	Ogooue et Lacs	16373	2010	49.3	42.4	57.5
Gabon	Moyen-Ogooue	Ogooue et Lacs	16373	2015	42.5	35.9	50.4
Gabon	Ngounie	Boumi-Louetsi	16374	2000	76.1	66.0	88.4
Gabon	Ngounie	Boumi-Louetsi	16374	2005	66.6	57.5	77.2
Gabon	Ngounie	Boumi-Louetsi	16374	2010	57.0	48.7	66.2
Gabon	Ngounie	Boumi-Louetsi	16374	2015	48.6	40.7	57.4
Gabon	Ngounie	Dola	16375	2000	68.7	59.8	78.8
Gabon	Ngounie	Dola	16375	2005	61.1	52.9	69.7
Gabon	Ngounie	Dola	16375	2010	50.5	43.5	58.1
Gabon	Ngounie	Dola	16375	2015	42.8	36.3	50.7
Gabon	Ngounie	Douya-Onoye	16376	2000	71.8	61.8	83.7
Gabon	Ngounie	Douya-Onoye	16376	2005	62.8	54.0	73.1
Gabon	Ngounie	Douya-Onoye	16376	2010	52.6	45.0	61.5
Gabon	Ngounie	Douya-Onoye	16376	2015	45.2	37.9	53.8
Gabon	Ngounie	Louetsi-Bibaka	16377	2000	79.7	68.5	93.7
Gabon	Ngounie	Louetsi-Bibaka	16377	2005	72.3	62.0	85.6
Gabon	Ngounie	Louetsi-Bibaka	16377	2010	59.7	50.7	70.6
Gabon	Ngounie	Louetsi-Bibaka	16377	2015	51.4	42.7	61.2
Gabon	Ngounie	Louetsi-Wano	16378	2000	69.7	59.5	80.4
Gabon	Ngounie	Louetsi-Wano	16378	2005	59.6	51.1	69.0
Gabon	Ngounie	Louetsi-Wano	16378	2010	50.4	43.0	58.6
Gabon	Ngounie	Louetsi-Wano	16378	2015	43.0	35.9	51.4
Gabon	Ngounie	Mougalaba	16379	2000	70.6	59.9	82.2
Gabon	Ngounie	Mougalaba	16379	2005	62.8	53.5	73.4
Gabon	Ngounie	Mougalaba	16379	2010	50.8	43.0	59.5
Gabon	Ngounie	Mougalaba	16379	2015	45.5	38.3	53.7
Gabon	Ngounie	Ndolou	16380	2000	68.4	57.9	79.8
Gabon	Ngounie	Ndolou	16380	2005	60.7	51.0	71.3
Gabon	Ngounie	Ndolou	16380	2010	49.3	41.2	58.4
Gabon	Ngounie	Ndolou	16380	2015	44.5	37.5	53.1
Gabon	Ngounie	Ogoulou	16381	2000	78.2	67.3	90.0
Gabon	Ngounie	Ogoulou	16381	2005	68.3	59.0	79.1
Gabon	Ngounie	Ogoulou	16381	2010	58.3	50.0	67.3
Gabon	Ngounie	Ogoulou	16381	2015	51.1	42.7	60.5
Gabon	Ngounie	Tsamba-Magotsi	16382	2000	73.6	62.9	85.6
Gabon	Ngounie	Tsamba-Magotsi	16382	2005	65.4	55.8	75.7
Gabon	Ngounie	Tsamba-Magotsi	16382	2010	53.1	45.2	62.2
Gabon	Ngounie	Tsamba-Magotsi	16382	2015	46.7	38.8	55.0
Gabon	Nyanga	Basse-Banio	16383	2000	65.4	56.0	75.9
Gabon	Nyanga	Basse-Banio	16383	2005	61.7	52.9	70.8
Gabon	Nyanga	Basse-Banio	16383	2010	48.3	41.2	56.1
Gabon	Nyanga	Basse-Banio	16383	2015	42.4	36.0	49.9
Gabon	Nyanga	Douigni	16384	2000	67.7	58.0	79.2
Gabon	Nyanga	Douigni	16384	2005	61.1	52.0	71.0
Gabon	Nyanga	Douigni	16384	2010	48.8	41.7	56.8
Gabon	Nyanga	Douigni	16384	2015	42.7	36.0	50.6
Gabon	Nyanga	Doutsila	16385	2000	68.6	59.6	78.9
Gabon	Nyanga	Doutsila	16385	2005	63.6	55.3	72.5
Gabon	Nyanga	Doutsila	16385	2010	50.2	43.5	57.6
Gabon	Nyanga	Doutsila	16385	2015	41.9	35.7	48.8
Gabon	Nyanga	Haute-Banio	16386	2000	72.3	59.3	86.8
Gabon	Nyanga	Haute-Banio	16386	2005	67.9	56.0	81.5
Gabon	Nyanga	Haute-Banio	16386	2010	52.5	43.4	62.8
Gabon	Nyanga	Haute-Banio	16386	2015	43.4	35.5	52.3
Gabon	Nyanga	Mongo	16387	2000	72.3	61.8	85.0
Gabon	Nyanga	Mongo	16387	2005	68.3	58.5	79.7
Gabon	Nyanga	Mongo	16387	2010	52.6	45.0	61.5
Gabon	Nyanga	Mongo	16387	2015	43.8	36.8	51.1

Admin 0	Admin 1	Admin 2	GAUL Code	Year	Under-5 mortality (per 1,000 livebirths)		
					Estimate	Lower bound, 95% UI	Upper bound, 95% UI
Gabon	Nyanga	Mougoutsi	16388	2000	64.3	55.3	74.1
Gabon	Nyanga	Mougoutsi	16388	2005	59.6	51.7	68.6
Gabon	Nyanga	Mougoutsi	16388	2010	46.9	40.3	54.1
Gabon	Nyanga	Mougoutsi	16388	2015	40.3	34.3	47.3
Gabon	Ogooue-Ivindo	Ivindo	16389	2000	98.8	86.4	112.1
Gabon	Ogooue-Ivindo	Ivindo	16389	2005	92.3	80.9	103.9
Gabon	Ogooue-Ivindo	Ivindo	16389	2010	75.2	65.8	85.7
Gabon	Ogooue-Ivindo	Ivindo	16389	2015	64.2	55.0	75.0
Gabon	Ogooue-Ivindo	Lope	16390	2000	75.2	64.2	88.3
Gabon	Ogooue-Ivindo	Lope	16390	2005	67.6	57.8	78.8
Gabon	Ogooue-Ivindo	Lope	16390	2010	56.9	48.5	66.1
Gabon	Ogooue-Ivindo	Lope	16390	2015	50.0	41.6	59.2
Gabon	Ogooue-Ivindo	Mvoung	16391	2000	89.4	75.4	102.9
Gabon	Ogooue-Ivindo	Mvoung	16391	2005	80.3	68.6	92.4
Gabon	Ogooue-Ivindo	Mvoung	16391	2010	67.5	57.6	78.6
Gabon	Ogooue-Ivindo	Mvoung	16391	2015	60.6	50.7	71.4
Gabon	Ogooue-Ivindo	Zadie	16392	2000	121.4	106.1	138.1
Gabon	Ogooue-Ivindo	Zadie	16392	2005	112.7	97.2	128.5
Gabon	Ogooue-Ivindo	Zadie	16392	2010	88.9	77.0	102.8
Gabon	Ogooue-Ivindo	Zadie	16392	2015	79.5	66.4	92.0
Gabon	Ogooue-Maritime	Bendje	16397	2000	51.4	42.5	62.0
Gabon	Ogooue-Maritime	Bendje	16397	2005	50.6	41.8	60.7
Gabon	Ogooue-Maritime	Bendje	16397	2010	43.9	35.8	53.0
Gabon	Ogooue-Maritime	Bendje	16397	2015	36.5	29.2	44.5
Gabon	Ogooue-Maritime	Etimboue	16398	2000	62.7	52.6	73.9
Gabon	Ogooue-Maritime	Etimboue	16398	2005	55.6	46.8	65.6
Gabon	Ogooue-Maritime	Etimboue	16398	2010	45.6	38.1	54.4
Gabon	Ogooue-Maritime	Etimboue	16398	2015	42.0	35.2	50.5
Gabon	Ogooue-Maritime	Ndougou	16399	2000	62.5	52.3	73.2
Gabon	Ogooue-Maritime	Ndougou	16399	2005	56.6	47.6	66.4
Gabon	Ogooue-Maritime	Ndougou	16399	2010	45.3	37.6	53.5
Gabon	Ogooue-Maritime	Ndougou	16399	2015	41.8	34.9	49.8
Gabon	Ogooue-lolo	Lolo-Bouenguidi	16393	2000	73.2	63.7	83.8
Gabon	Ogooue-lolo	Lolo-Bouenguidi	16393	2005	64.7	56.4	73.7
Gabon	Ogooue-lolo	Lolo-Bouenguidi	16393	2010	54.8	47.2	63.4
Gabon	Ogooue-lolo	Lolo-Bouenguidi	16393	2015	47.3	39.9	55.4
Gabon	Ogooue-lolo	Lombo-Bouenguidi	16394	2000	66.6	57.2	78.1
Gabon	Ogooue-lolo	Lombo-Bouenguidi	16394	2005	58.8	50.6	67.9
Gabon	Ogooue-lolo	Lombo-Bouenguidi	16394	2010	49.4	41.8	58.4
Gabon	Ogooue-lolo	Lombo-Bouenguidi	16394	2015	42.0	34.6	50.2
Gabon	Ogooue-lolo	Mouloundou	16395	2000	74.4	64.8	85.3
Gabon	Ogooue-lolo	Mouloundou	16395	2005	67.0	58.5	76.7
Gabon	Ogooue-lolo	Mouloundou	16395	2010	56.2	48.0	65.6
Gabon	Ogooue-lolo	Mouloundou	16395	2015	47.8	40.3	56.5
Gabon	Ogooue-lolo	Offoue-Onoye	16396	2000	75.9	64.4	88.9
Gabon	Ogooue-lolo	Offoue-Onoye	16396	2005	66.5	55.8	77.8
Gabon	Ogooue-lolo	Offoue-Onoye	16396	2010	57.0	47.5	67.3
Gabon	Ogooue-lolo	Offoue-Onoye	16396	2015	48.7	40.0	58.5
Gabon	Woleu-Ntem	Haut-Komo	16400	2000	81.0	68.5	94.9
Gabon	Woleu-Ntem	Haut-Komo	16400	2005	77.3	65.5	91.0
Gabon	Woleu-Ntem	Haut-Komo	16400	2010	64.1	53.5	75.4
Gabon	Woleu-Ntem	Haut-Komo	16400	2015	55.3	45.7	66.1
Gabon	Woleu-Ntem	Haut-Ntem	16401	2000	112.4	97.7	128.4
Gabon	Woleu-Ntem	Haut-Ntem	16401	2005	103.1	89.6	119.3
Gabon	Woleu-Ntem	Haut-Ntem	16401	2010	86.5	74.3	98.7
Gabon	Woleu-Ntem	Haut-Ntem	16401	2015	76.4	65.3	89.3
Gabon	Woleu-Ntem	Ntem	16402	2000	109.0	96.2	122.9
Gabon	Woleu-Ntem	Ntem	16402	2005	98.2	86.2	111.5
Gabon	Woleu-Ntem	Ntem	16402	2010	81.4	69.7	94.0
Gabon	Woleu-Ntem	Ntem	16402	2015	68.6	58.0	80.8
Gabon	Woleu-Ntem	Okano	16403	2000	81.1	69.2	94.4
Gabon	Woleu-Ntem	Okano	16403	2005	75.3	64.3	88.0
Gabon	Woleu-Ntem	Okano	16403	2010	62.7	53.7	73.0
Gabon	Woleu-Ntem	Okano	16403	2015	54.8	46.3	65.0
Gabon	Woleu-Ntem	Woleu	16404	2000	89.2	77.5	101.7
Gabon	Woleu-Ntem	Woleu	16404	2005	83.0	71.7	94.8
Gabon	Woleu-Ntem	Woleu	16404	2010	71.8	60.9	83.2
Gabon	Woleu-Ntem	Woleu	16404	2015	61.3	51.5	71.7
Gambia	Central River	Fulladu West	16406	2000	103.6	96.6	110.5
Gambia	Central River	Fulladu West	16406	2005	80.2	75.0	85.4
Gambia	Central River	Fulladu West	16406	2010	64.5	60.0	69.7
Gambia	Central River	Fulladu West	16406	2015	57.0	51.4	62.8
Gambia	Central River	Janjanbureh	16407	2000	99.5	92.1	106.8
Gambia	Central River	Janjanbureh	16407	2005	76.5	71.0	82.2
Gambia	Central River	Janjanbureh	16407	2010	61.2	56.3	66.4
Gambia	Central River	Janjanbureh	16407	2015	56.1	50.1	62.1
Gambia	Central River	Lower Saloum	16408	2000	117.9	110.5	125.1
Gambia	Central River	Lower Saloum	16408	2005	90.0	84.3	95.6
Gambia	Central River	Lower Saloum	16408	2010	65.7	61.0	70.3
Gambia	Central River	Lower Saloum	16408	2015	56.6	51.1	62.9
Gambia	Central River	Niamina Dankunku	16409	2000	102.6	96.4	109.2
Gambia	Central River	Niamina Dankunku	16409	2005	80.0	75.2	85.1
Gambia	Central River	Niamina Dankunku	16409	2010	61.1	56.8	65.4
Gambia	Central River	Niamina Dankunku	16409	2015	52.6	47.4	58.3
Gambia	Central River	Niamina East	16410	2000	99.4	93.4	106.5

Admin 0	Admin 1	Admin 2	GAUL Code	Year	Under-5 mortality (per 1,000 livebirths)		
					Estimate	Lower bound, 95% UI	Upper bound, 95% UI
Gambia	Central River	Niamina East	16410	2005	76.9	72.1	82.3
Gambia	Central River	Niamina East	16410	2010	62.2	57.8	67.0
Gambia	Central River	Niamina East	16410	2015	53.7	48.5	59.7
Gambia	Central River	Niamina West	16411	2000	115.0	107.9	122.8
Gambia	Central River	Niamina West	16411	2005	87.3	81.8	92.9
Gambia	Central River	Niamina West	16411	2010	66.0	61.0	70.8
Gambia	Central River	Niamina West	16411	2015	55.7	50.2	62.0
Gambia	Central River	Niani	16412	2000	112.0	104.7	119.4
Gambia	Central River	Niani	16412	2005	84.4	78.7	90.3
Gambia	Central River	Niani	16412	2010	69.8	64.7	75.2
Gambia	Central River	Niani	16412	2015	58.6	52.5	65.0
Gambia	Central River	Nianija	16413	2000	110.2	103.4	117.7
Gambia	Central River	Nianija	16413	2005	83.2	78.2	88.8
Gambia	Central River	Nianija	16413	2010	67.3	62.4	72.4
Gambia	Central River	Nianija	16413	2015	56.7	50.8	63.0
Gambia	Central River	Sami	16414	2000	118.9	110.2	127.5
Gambia	Central River	Sami	16414	2005	92.6	85.9	99.2
Gambia	Central River	Sami	16414	2010	74.5	68.8	80.5
Gambia	Central River	Sami	16414	2015	63.5	56.9	70.4
Gambia	Central River	Upper Saloum	16415	2000	115.4	108.5	122.5
Gambia	Central River	Upper Saloum	16415	2005	87.3	82.2	92.7
Gambia	Central River	Upper Saloum	16415	2010	66.6	61.9	71.1
Gambia	Central River	Upper Saloum	16415	2015	56.9	51.4	63.0
Gambia	Kanifing Municipal Council	Banjul	16405	2000	57.1	52.7	61.7
Gambia	Kanifing Municipal Council	Banjul	16405	2005	54.9	51.0	59.0
Gambia	Kanifing Municipal Council	Banjul	16405	2010	50.4	46.2	55.0
Gambia	Kanifing Municipal Council	Banjul	16405	2015	41.0	36.5	45.9
Gambia	Kanifing Municipal Council	Kombo Saint Mary	16416	2000	54.4	50.0	59.1
Gambia	Kanifing Municipal Council	Kombo Saint Mary	16416	2005	54.7	50.5	59.2
Gambia	Kanifing Municipal Council	Kombo Saint Mary	16416	2010	50.3	46.1	55.1
Gambia	Kanifing Municipal Council	Kombo Saint Mary	16416	2015	42.5	37.9	47.6
Gambia	Lower River	Jarra Central	16417	2000	112.9	105.6	120.5
Gambia	Lower River	Jarra Central	16417	2005	89.2	83.4	94.9
Gambia	Lower River	Jarra Central	16417	2010	64.5	59.8	69.0
Gambia	Lower River	Jarra Central	16417	2015	55.1	49.8	60.7
Gambia	Lower River	Jarra East	16418	2000	108.5	101.5	116.1
Gambia	Lower River	Jarra East	16418	2005	83.8	78.4	89.5
Gambia	Lower River	Jarra East	16418	2010	63.5	58.6	68.1
Gambia	Lower River	Jarra East	16418	2015	54.9	49.6	60.7
Gambia	Lower River	Jarra West	16419	2000	110.8	104.1	118.2
Gambia	Lower River	Jarra West	16419	2005	87.5	82.2	93.0
Gambia	Lower River	Jarra West	16419	2010	61.6	57.3	66.0
Gambia	Lower River	Jarra West	16419	2015	53.2	47.8	58.5
Gambia	Lower River	Kiang Central	16420	2000	102.9	96.6	109.6
Gambia	Lower River	Kiang Central	16420	2005	81.7	76.8	87.0
Gambia	Lower River	Kiang Central	16420	2010	61.7	57.4	66.3
Gambia	Lower River	Kiang Central	16420	2015	51.2	46.4	56.6
Gambia	Lower River	Kiang East	16421	2000	110.2	103.6	117.2
Gambia	Lower River	Kiang East	16421	2005	86.4	81.2	91.9
Gambia	Lower River	Kiang East	16421	2010	62.6	58.1	67.0
Gambia	Lower River	Kiang East	16421	2015	53.5	48.2	58.9
Gambia	Lower River	Kiang West	16422	2000	93.0	86.3	99.8
Gambia	Lower River	Kiang West	16422	2005	75.9	71.0	81.2
Gambia	Lower River	Kiang West	16422	2010	59.7	55.3	64.5
Gambia	Lower River	Kiang West	16422	2015	49.1	44.3	54.4
Gambia	North Bank	Central Baddibu	16423	2000	105.2	99.0	112.0
Gambia	North Bank	Central Baddibu	16423	2005	81.0	76.3	85.7
Gambia	North Bank	Central Baddibu	16423	2010	63.2	59.1	67.7
Gambia	North Bank	Central Baddibu	16423	2015	53.8	48.9	59.4
Gambia	North Bank	Jokadu	16424	2000	92.4	85.7	99.6
Gambia	North Bank	Jokadu	16424	2005	77.3	71.8	83.1
Gambia	North Bank	Jokadu	16424	2010	62.5	57.9	67.4
Gambia	North Bank	Jokadu	16424	2015	50.8	45.4	56.8
Gambia	North Bank	Lower Baddibu	16425	2000	97.8	91.0	104.8
Gambia	North Bank	Lower Baddibu	16425	2005	79.0	73.9	84.4
Gambia	North Bank	Lower Baddibu	16425	2010	62.9	58.5	67.8
Gambia	North Bank	Lower Baddibu	16425	2015	51.2	46.1	56.9
Gambia	North Bank	Lower Niumi	16426	2000	79.3	73.9	84.9
Gambia	North Bank	Lower Niumi	16426	2005	69.6	65.2	74.3
Gambia	North Bank	Lower Niumi	16426	2010	57.8	53.8	62.1
Gambia	North Bank	Lower Niumi	16426	2015	48.8	44.0	54.1
Gambia	North Bank	Upper Baddibu	16427	2000	114.1	108.2	120.8
Gambia	North Bank	Upper Baddibu	16427	2005	87.1	82.5	92.0
Gambia	North Bank	Upper Baddibu	16427	2010	63.9	59.8	68.1
Gambia	North Bank	Upper Baddibu	16427	2015	54.7	49.6	60.3
Gambia	North Bank	Upper Niumi	16428	2000	84.2	78.4	90.3
Gambia	North Bank	Upper Niumi	16428	2005	73.5	68.6	78.5
Gambia	North Bank	Upper Niumi	16428	2010	60.8	56.6	65.4
Gambia	North Bank	Upper Niumi	16428	2015	51.0	45.6	56.7
Gambia	Upper River	Fulladu East	16429	2000	141.0	132.5	150.3
Gambia	Upper River	Fulladu East	16429	2005	108.3	101.7	115.3
Gambia	Upper River	Fulladu East	16429	2010	86.1	79.9	92.6
Gambia	Upper River	Fulladu East	16429	2015	75.5	67.6	83.5
Gambia	Upper River	Kantora	16430	2000	139.2	130.2	150.2
Gambia	Upper River	Kantora	16430	2005	104.4	97.4	112.1

Admin 0	Admin 1	Admin 2	GAUL Code	Year	Under-5 mortality (per 1,000 livebirths)		
					Estimate	Lower bound, 95% UI	Upper bound, 95% UI
Gambia	Upper River	Kantora	16430	2010	85.1	78.3	92.7
Gambia	Upper River	Kantora	16430	2015	75.8	67.9	84.7
Gambia	Upper River	Sandu	16431	2000	135.7	127.2	145.6
Gambia	Upper River	Sandu	16431	2005	105.5	98.4	112.8
Gambia	Upper River	Sandu	16431	2010	83.7	77.2	90.6
Gambia	Upper River	Sandu	16431	2015	71.6	64.1	79.2
Gambia	Upper River	Wuli	16432	2000	127.1	118.6	137.0
Gambia	Upper River	Wuli	16432	2005	99.7	93.0	106.6
Gambia	Upper River	Wuli	16432	2010	80.6	74.5	87.4
Gambia	Upper River	Wuli	16432	2015	72.0	64.2	79.8
Gambia	West Coast	Foni Bintang-karenai	16433	2000	85.0	78.3	91.1
Gambia	West Coast	Foni Bintang-karenai	16433	2005	71.9	67.2	77.0
Gambia	West Coast	Foni Bintang-karenai	16433	2010	59.1	54.7	63.6
Gambia	West Coast	Foni Bintang-karenai	16433	2015	49.8	44.5	55.0
Gambia	West Coast	Foni Bondali	16434	2000	103.7	95.1	113.2
Gambia	West Coast	Foni Bondali	16434	2005	82.0	76.0	88.6
Gambia	West Coast	Foni Bondali	16434	2010	64.0	58.6	69.9
Gambia	West Coast	Foni Bondali	16434	2015	53.9	47.9	60.4
Gambia	West Coast	Foni Brefet	16435	2000	78.5	72.7	84.3
Gambia	West Coast	Foni Brefet	16435	2005	68.9	64.3	73.7
Gambia	West Coast	Foni Brefet	16435	2010	57.9	53.5	62.3
Gambia	West Coast	Foni Brefet	16435	2015	48.9	44.0	54.2
Gambia	West Coast	Foni Jarrol	16436	2000	111.6	103.2	120.7
Gambia	West Coast	Foni Jarrol	16436	2005	87.6	81.7	94.2
Gambia	West Coast	Foni Jarrol	16436	2010	67.2	61.8	72.9
Gambia	West Coast	Foni Jarrol	16436	2015	57.4	51.4	64.1
Gambia	West Coast	Foni Kansala	16437	2000	92.9	85.6	100.2
Gambia	West Coast	Foni Kansala	16437	2005	75.5	70.4	80.9
Gambia	West Coast	Foni Kansala	16437	2010	59.5	54.7	64.4
Gambia	West Coast	Foni Kansala	16437	2015	50.4	45.3	56.1
Gambia	West Coast	Kombo Central	16438	2000	64.5	59.3	70.0
Gambia	West Coast	Kombo Central	16438	2005	60.6	56.3	65.3
Gambia	West Coast	Kombo Central	16438	2010	53.4	49.0	58.3
Gambia	West Coast	Kombo Central	16438	2015	45.3	40.2	50.6
Gambia	West Coast	Kombo East	16439	2000	73.6	68.3	79.1
Gambia	West Coast	Kombo East	16439	2005	66.3	62.1	71.0
Gambia	West Coast	Kombo East	16439	2010	56.5	52.0	61.0
Gambia	West Coast	Kombo East	16439	2015	47.7	42.6	52.8
Gambia	West Coast	Kombo North	16440	2000	59.5	54.6	64.8
Gambia	West Coast	Kombo North	16440	2005	57.0	52.6	61.6
Gambia	West Coast	Kombo North	16440	2010	51.8	47.5	56.8
Gambia	West Coast	Kombo North	16440	2015	43.7	38.9	49.0
Gambia	West Coast	Kombo South	16441	2000	65.8	60.4	71.7
Gambia	West Coast	Kombo South	16441	2005	60.9	56.4	65.9
Gambia	West Coast	Kombo South	16441	2010	53.5	48.9	58.9
Gambia	West Coast	Kombo South	16441	2015	45.5	40.5	51.2
Ghana	Ashanti	Adansi North	190578	2000	101.2	94.0	109.4
Ghana	Ashanti	Adansi North	190578	2005	88.0	81.8	94.9
Ghana	Ashanti	Adansi North	190578	2010	72.8	66.9	79.2
Ghana	Ashanti	Adansi North	190578	2015	52.9	46.8	59.1
Ghana	Ashanti	Adansi South	190577	2000	104.1	96.3	112.2
Ghana	Ashanti	Adansi South	190577	2005	89.8	83.4	96.6
Ghana	Ashanti	Adansi South	190577	2010	74.0	68.0	80.3
Ghana	Ashanti	Adansi South	190577	2015	55.6	49.0	61.6
Ghana	Ashanti	Afigya Kwabre	190567	2000	90.0	84.0	95.9
Ghana	Ashanti	Afigya Kwabre	190567	2005	83.0	77.6	89.1
Ghana	Ashanti	Afigya Kwabre	190567	2010	69.2	64.2	74.7
Ghana	Ashanti	Afigya Kwabre	190567	2015	48.6	43.3	54.3
Ghana	Ashanti	Afigya Sekyere	190569	2000	91.8	84.5	99.2
Ghana	Ashanti	Afigya Sekyere	190569	2005	84.8	78.2	91.8
Ghana	Ashanti	Afigya Sekyere	190569	2010	70.1	64.3	76.5
Ghana	Ashanti	Afigya Sekyere	190569	2015	49.2	43.7	55.3
Ghana	Ashanti	Ahafo Ano North	16567	2000	97.2	89.9	105.7
Ghana	Ashanti	Ahafo Ano North	16567	2005	86.4	79.3	93.6
Ghana	Ashanti	Ahafo Ano North	16567	2010	71.0	64.7	77.6
Ghana	Ashanti	Ahafo Ano North	16567	2015	52.6	46.4	59.2
Ghana	Ashanti	Ahafo Ano South	16568	2000	105.3	98.2	113.2
Ghana	Ashanti	Ahafo Ano South	16568	2005	90.1	83.5	96.7
Ghana	Ashanti	Ahafo Ano South	16568	2010	73.4	67.4	79.4
Ghana	Ashanti	Ahafo Ano South	16568	2015	55.2	49.1	62.2
Ghana	Ashanti	Amansie Central	190606	2000	101.5	94.4	108.8
Ghana	Ashanti	Amansie Central	190606	2005	88.6	82.6	95.2
Ghana	Ashanti	Amansie Central	190606	2010	73.4	67.5	79.7
Ghana	Ashanti	Amansie Central	190606	2015	53.6	47.5	59.7
Ghana	Ashanti	Amansie West	16570	2000	104.7	96.3	113.9
Ghana	Ashanti	Amansie West	16570	2005	89.4	82.2	96.7
Ghana	Ashanti	Amansie West	16570	2010	73.2	67.1	79.6
Ghana	Ashanti	Amansie West	16570	2015	54.6	48.5	61.3
Ghana	Ashanti	Asante Akim Central Municipal	190718	2000	86.3	78.8	93.5
Ghana	Ashanti	Asante Akim Central Municipal	190718	2005	80.0	73.7	86.9
Ghana	Ashanti	Asante Akim Central Municipal	190718	2010	69.3	63.3	75.8
Ghana	Ashanti	Asante Akim Central Municipal	190718	2015	49.6	43.8	55.6
Ghana	Ashanti	Asante Akim North	190717	2000	88.0	80.4	96.0
Ghana	Ashanti	Asante Akim North	190717	2005	80.6	73.9	87.7
Ghana	Ashanti	Asante Akim North	190717	2010	69.2	63.1	75.7

Admin 0	Admin 1	Admin 2	GAUL Code	Year	Under-5 mortality (per 1,000 livebirths)		
					Estimate	Lower bound, 95% UI	Upper bound, 95% UI
Ghana	Ashanti	Asante Akim North	190717	2015	49.9	44.0	56.3
Ghana	Ashanti	Asante Akim South	16572	2000	92.4	85.5	99.4
Ghana	Ashanti	Asante Akim South	16572	2005	82.8	76.7	88.9
Ghana	Ashanti	Asante Akim South	16572	2010	70.0	64.6	76.1
Ghana	Ashanti	Asante Akim South	16572	2015	50.9	45.3	56.6
Ghana	Ashanti	Asokore Mampong Municipal	190719	2000	80.3	74.6	85.9
Ghana	Ashanti	Asokore Mampong Municipal	190719	2005	76.4	71.2	82.0
Ghana	Ashanti	Asokore Mampong Municipal	190719	2010	67.6	62.4	73.5
Ghana	Ashanti	Asokore Mampong Municipal	190719	2015	47.2	41.6	52.8
Ghana	Ashanti	Atwima Kwanwoma	190568	2000	88.0	82.0	94.5
Ghana	Ashanti	Atwima Kwanwoma	190568	2005	81.5	75.4	87.4
Ghana	Ashanti	Atwima Kwanwoma	190568	2010	68.9	63.6	74.8
Ghana	Ashanti	Atwima Kwanwoma	190568	2015	48.7	43.2	54.2
Ghana	Ashanti	Atwima Mponua	190576	2000	103.4	95.4	111.3
Ghana	Ashanti	Atwima Mponua	190576	2005	89.1	81.9	96.4
Ghana	Ashanti	Atwima Mponua	190576	2010	73.3	66.8	80.0
Ghana	Ashanti	Atwima Mponua	190576	2015	55.1	49.2	62.1
Ghana	Ashanti	Atwima Nwabiagya	190575	2000	89.4	83.2	95.7
Ghana	Ashanti	Atwima Nwabiagya	190575	2005	81.6	75.6	87.6
Ghana	Ashanti	Atwima Nwabiagya	190575	2010	68.9	63.6	74.5
Ghana	Ashanti	Atwima Nwabiagya	190575	2015	48.5	43.3	54.2
Ghana	Ashanti	Bekwai Municipal	190580	2000	96.7	90.2	103.7
Ghana	Ashanti	Bekwai Municipal	190580	2005	86.4	80.5	92.7
Ghana	Ashanti	Bekwai Municipal	190580	2010	71.9	66.5	77.9
Ghana	Ashanti	Bekwai Municipal	190580	2015	51.1	45.3	57.0
Ghana	Ashanti	Bosome Freho	190579	2000	100.9	93.5	108.3
Ghana	Ashanti	Bosome Freho	190579	2005	87.6	81.3	94.3
Ghana	Ashanti	Bosome Freho	190579	2010	72.7	67.0	79.1
Ghana	Ashanti	Bosome Freho	190579	2015	52.9	46.9	58.7
Ghana	Ashanti	Bosomtwe /Atwima / Kwanwoma	190581	2000	91.8	85.2	98.6
Ghana	Ashanti	Bosomtwe /Atwima / Kwanwoma	190581	2005	83.8	77.9	90.4
Ghana	Ashanti	Bosomtwe /Atwima / Kwanwoma	190581	2010	71.2	65.7	77.9
Ghana	Ashanti	Bosomtwe /Atwima / Kwanwoma	190581	2015	50.0	44.3	56.0
Ghana	Ashanti	Ejisu Juaben	16576	2000	87.6	81.2	93.6
Ghana	Ashanti	Ejisu Juaben	16576	2005	81.8	76.2	87.9
Ghana	Ashanti	Ejisu Juaben	16576	2010	69.5	64.2	75.5
Ghana	Ashanti	Ejisu Juaben	16576	2015	48.8	43.3	54.4
Ghana	Ashanti	Ejura Sekye Dumase	16575	2000	104.7	95.3	115.4
Ghana	Ashanti	Ejura Sekye Dumase	16575	2005	91.2	83.3	99.6
Ghana	Ashanti	Ejura Sekye Dumase	16575	2010	73.1	66.3	80.8
Ghana	Ashanti	Ejura Sekye Dumase	16575	2015	54.8	48.1	62.0
Ghana	Ashanti	Kma	190720	2000	75.7	70.3	81.0
Ghana	Ashanti	Kma	190720	2005	73.2	68.0	78.5
Ghana	Ashanti	Kma	190720	2010	65.9	60.9	71.7
Ghana	Ashanti	Kma	190720	2015	45.9	40.5	51.5
Ghana	Ashanti	Kwabre	16578	2000	80.8	75.2	86.4
Ghana	Ashanti	Kwabre	16578	2005	76.9	71.6	82.4
Ghana	Ashanti	Kwabre	16578	2010	67.7	62.4	73.5
Ghana	Ashanti	Kwabre	16578	2015	47.1	41.7	52.8
Ghana	Ashanti	Mampong Municipal	190582	2000	96.0	87.6	104.6
Ghana	Ashanti	Mampong Municipal	190582	2005	87.6	80.2	95.2
Ghana	Ashanti	Mampong Municipal	190582	2010	71.6	65.6	78.6
Ghana	Ashanti	Mampong Municipal	190582	2015	51.3	45.3	57.9
Ghana	Ashanti	Obuasi Municipal	190574	2000	92.2	85.1	100.4
Ghana	Ashanti	Obuasi Municipal	190574	2005	82.9	76.4	90.3
Ghana	Ashanti	Obuasi Municipal	190574	2010	70.7	64.4	77.7
Ghana	Ashanti	Obuasi Municipal	190574	2015	51.5	45.4	57.7
Ghana	Ashanti	Offinso Municipal	190573	2000	94.0	87.7	101.4
Ghana	Ashanti	Offinso Municipal	190573	2005	83.9	78.3	90.6
Ghana	Ashanti	Offinso Municipal	190573	2010	69.5	64.2	75.3
Ghana	Ashanti	Offinso Municipal	190573	2015	49.8	44.3	55.7
Ghana	Ashanti	Offinso North	190572	2000	97.9	90.4	106.1
Ghana	Ashanti	Offinso North	190572	2005	86.7	80.0	93.3
Ghana	Ashanti	Offinso North	190572	2010	70.9	64.7	78.1
Ghana	Ashanti	Offinso North	190572	2015	52.4	46.0	59.0
Ghana	Ashanti	Sekyere Afram Plains	190715	2000	86.5	79.3	93.8
Ghana	Ashanti	Sekyere Afram Plains	190715	2005	81.3	74.8	88.3
Ghana	Ashanti	Sekyere Afram Plains	190715	2010	69.5	63.9	75.9
Ghana	Ashanti	Sekyere Afram Plains	190715	2015	49.3	43.6	55.6
Ghana	Ashanti	Sekyere Afram Plains North	190716	2000	97.8	87.3	108.5
Ghana	Ashanti	Sekyere Afram Plains North	190716	2005	87.1	78.5	96.5
Ghana	Ashanti	Sekyere Afram Plains North	190716	2010	71.8	64.3	80.0
Ghana	Ashanti	Sekyere Afram Plains North	190716	2015	55.6	48.7	62.9
Ghana	Ashanti	Sekyere Central	190571	2000	94.5	86.3	103.2
Ghana	Ashanti	Sekyere Central	190571	2005	86.6	79.2	94.1
Ghana	Ashanti	Sekyere Central	190571	2010	71.4	65.3	78.1
Ghana	Ashanti	Sekyere Central	190571	2015	52.2	46.4	58.7
Ghana	Ashanti	Sekyere East	190570	2000	91.1	83.9	98.2
Ghana	Ashanti	Sekyere East	190570	2005	84.1	77.7	91.4
Ghana	Ashanti	Sekyere East	190570	2010	70.8	64.9	77.3
Ghana	Ashanti	Sekyere East	190570	2015	49.7	44.0	55.6
Ghana	Brong Ahafo	Asunafo North	190558	2000	98.1	90.2	106.6
Ghana	Brong Ahafo	Asunafo North	190558	2005	88.6	81.4	96.2
Ghana	Brong Ahafo	Asunafo North	190558	2010	71.9	65.6	78.6
Ghana	Brong Ahafo	Asunafo North	190558	2015	54.8	48.3	61.6

Admin 0	Admin 1	Admin 2	GAUL Code	Year	Under-5 mortality (per 1,000 livebirths)		
					Estimate	Lower bound, 95% UI	Upper bound, 95% UI
Ghana	Brong Ahafo	Asunafo South	190609	2000	99.8	92.3	109.5
Ghana	Brong Ahafo	Asunafo South	190609	2005	87.9	80.6	95.6
Ghana	Brong Ahafo	Asunafo South	190609	2010	72.1	65.2	79.1
Ghana	Brong Ahafo	Asunafo South	190609	2015	55.3	48.4	61.7
Ghana	Brong Ahafo	Asutifi North	190708	2000	92.0	84.8	99.3
Ghana	Brong Ahafo	Asutifi North	190708	2005	83.9	77.1	91.2
Ghana	Brong Ahafo	Asutifi North	190708	2010	68.8	62.6	75.0
Ghana	Brong Ahafo	Asutifi North	190708	2015	50.3	44.3	56.8
Ghana	Brong Ahafo	Asutifi South	190709	2000	96.5	88.6	105.2
Ghana	Brong Ahafo	Asutifi South	190709	2005	86.0	78.5	93.9
Ghana	Brong Ahafo	Asutifi South	190709	2010	70.6	63.9	77.6
Ghana	Brong Ahafo	Asutifi South	190709	2015	53.0	46.7	60.3
Ghana	Brong Ahafo	Atebubu Amantin	190614	2000	111.1	100.1	123.5
Ghana	Brong Ahafo	Atebubu Amantin	190614	2005	96.6	87.3	106.9
Ghana	Brong Ahafo	Atebubu Amantin	190614	2010	74.3	66.6	83.0
Ghana	Brong Ahafo	Atebubu Amantin	190614	2015	57.3	50.2	65.4
Ghana	Brong Ahafo	Banda	190712	2000	115.6	104.0	128.5
Ghana	Brong Ahafo	Banda	190712	2005	104.2	93.3	115.3
Ghana	Brong Ahafo	Banda	190712	2010	79.8	71.3	89.8
Ghana	Brong Ahafo	Banda	190712	2015	63.2	55.2	72.3
Ghana	Brong Ahafo	Berekum	16585	2000	87.4	79.6	95.1
Ghana	Brong Ahafo	Berekum	16585	2005	82.7	74.9	90.7
Ghana	Brong Ahafo	Berekum	16585	2010	66.7	60.1	73.9
Ghana	Brong Ahafo	Berekum	16585	2015	48.9	42.7	56.0
Ghana	Brong Ahafo	Dormaa East	190555	2000	90.7	82.4	98.9
Ghana	Brong Ahafo	Dormaa East	190555	2005	85.5	77.4	94.3
Ghana	Brong Ahafo	Dormaa East	190555	2010	67.9	61.5	74.9
Ghana	Brong Ahafo	Dormaa East	190555	2015	50.6	44.3	57.7
Ghana	Brong Ahafo	Dormaa Municipal	190683	2000	98.3	89.1	107.4
Ghana	Brong Ahafo	Dormaa Municipal	190683	2005	91.3	82.9	100.4
Ghana	Brong Ahafo	Dormaa Municipal	190683	2010	71.7	64.9	79.2
Ghana	Brong Ahafo	Dormaa Municipal	190683	2015	55.1	48.3	62.3
Ghana	Brong Ahafo	Dormaa West	190684	2000	102.3	94.1	110.8
Ghana	Brong Ahafo	Dormaa West	190684	2005	95.0	86.5	104.1
Ghana	Brong Ahafo	Dormaa West	190684	2010	75.8	68.7	83.4
Ghana	Brong Ahafo	Dormaa West	190684	2015	59.0	51.4	66.7
Ghana	Brong Ahafo	Jaman North	190713	2000	104.7	95.2	115.8
Ghana	Brong Ahafo	Jaman North	190713	2005	96.4	87.0	106.2
Ghana	Brong Ahafo	Jaman North	190713	2010	74.1	66.3	83.0
Ghana	Brong Ahafo	Jaman North	190713	2015	58.5	50.8	66.8
Ghana	Brong Ahafo	Jaman South	190556	2000	102.2	93.0	111.8
Ghana	Brong Ahafo	Jaman South	190556	2005	94.2	85.6	103.4
Ghana	Brong Ahafo	Jaman South	190556	2010	71.8	64.8	80.3
Ghana	Brong Ahafo	Jaman South	190556	2015	55.8	48.7	63.0
Ghana	Brong Ahafo	Kintampo North	190613	2000	118.3	106.8	131.1
Ghana	Brong Ahafo	Kintampo North	190613	2005	103.8	93.8	114.3
Ghana	Brong Ahafo	Kintampo North	190613	2010	79.9	71.6	89.1
Ghana	Brong Ahafo	Kintampo North	190613	2015	61.6	54.5	70.7
Ghana	Brong Ahafo	Kintampo South	190560	2000	117.5	107.4	129.8
Ghana	Brong Ahafo	Kintampo South	190560	2005	101.9	93.2	112.4
Ghana	Brong Ahafo	Kintampo South	190560	2010	79.1	71.5	88.4
Ghana	Brong Ahafo	Kintampo South	190560	2015	60.6	53.3	69.0
Ghana	Brong Ahafo	Nkoranza North	190559	2000	111.7	102.1	123.1
Ghana	Brong Ahafo	Nkoranza North	190559	2005	96.3	88.2	105.4
Ghana	Brong Ahafo	Nkoranza North	190559	2010	76.0	68.8	84.1
Ghana	Brong Ahafo	Nkoranza North	190559	2015	58.5	51.4	66.1
Ghana	Brong Ahafo	Nkoranza South	190610	2000	104.6	95.8	114.3
Ghana	Brong Ahafo	Nkoranza South	190610	2005	91.1	83.7	98.9
Ghana	Brong Ahafo	Nkoranza South	190610	2010	73.2	66.6	80.3
Ghana	Brong Ahafo	Nkoranza South	190610	2015	56.0	49.1	63.0
Ghana	Brong Ahafo	Pru	190619	2000	114.0	103.3	126.0
Ghana	Brong Ahafo	Pru	190619	2005	99.1	90.1	108.3
Ghana	Brong Ahafo	Pru	190619	2010	76.1	68.7	84.7
Ghana	Brong Ahafo	Pru	190619	2015	59.9	52.6	68.1
Ghana	Brong Ahafo	Sene East	190706	2000	109.2	98.0	120.5
Ghana	Brong Ahafo	Sene East	190706	2005	90.4	81.4	99.9
Ghana	Brong Ahafo	Sene East	190706	2010	72.3	64.7	80.9
Ghana	Brong Ahafo	Sene East	190706	2015	59.8	51.8	67.7
Ghana	Brong Ahafo	Sene West	190707	2000	112.4	100.2	125.8
Ghana	Brong Ahafo	Sene West	190707	2005	96.4	85.6	107.9
Ghana	Brong Ahafo	Sene West	190707	2010	74.1	65.5	83.1
Ghana	Brong Ahafo	Sene West	190707	2015	60.4	52.5	69.4
Ghana	Brong Ahafo	Sunyani Municipal	190630	2000	87.2	80.2	94.3
Ghana	Brong Ahafo	Sunyani Municipal	190630	2005	82.2	75.3	89.0
Ghana	Brong Ahafo	Sunyani Municipal	190630	2010	68.1	61.9	74.5
Ghana	Brong Ahafo	Sunyani Municipal	190630	2015	49.5	43.5	56.2
Ghana	Brong Ahafo	Sunyani West	190557	2000	84.7	77.6	92.1
Ghana	Brong Ahafo	Sunyani West	190557	2005	79.8	73.1	87.2
Ghana	Brong Ahafo	Sunyani West	190557	2010	67.0	60.9	74.1
Ghana	Brong Ahafo	Sunyani West	190557	2015	48.2	42.2	55.3
Ghana	Brong Ahafo	Tain	190714	2000	105.1	96.5	114.8
Ghana	Brong Ahafo	Tain	190714	2005	94.1	85.7	103.1
Ghana	Brong Ahafo	Tain	190714	2010	73.3	66.2	81.3
Ghana	Brong Ahafo	Tain	190714	2015	57.3	50.5	65.1
Ghana	Brong Ahafo	Tano North	190561	2000	89.9	82.7	97.1

Admin 0	Admin 1	Admin 2	GAUL Code	Year	Under-5 mortality (per 1,000 livebirths)		
					Estimate	Lower bound, 95% UI	Upper bound, 95% UI
Ghana	Brong Ahafo	Tano North	190561	2005	82.4	75.6	89.0
Ghana	Brong Ahafo	Tano North	190561	2010	68.8	62.3	75.9
Ghana	Brong Ahafo	Tano North	190561	2015	50.1	44.0	56.4
Ghana	Brong Ahafo	Tano South	190629	2000	93.8	86.7	101.2
Ghana	Brong Ahafo	Tano South	190629	2005	83.9	77.2	91.1
Ghana	Brong Ahafo	Tano South	190629	2010	69.8	63.6	76.5
Ghana	Brong Ahafo	Tano South	190629	2015	50.9	44.9	57.3
Ghana	Brong Ahafo	Techiman Municipal	190710	2000	100.2	92.0	109.8
Ghana	Brong Ahafo	Techiman Municipal	190710	2005	88.3	81.1	95.7
Ghana	Brong Ahafo	Techiman Municipal	190710	2010	71.4	64.8	78.8
Ghana	Brong Ahafo	Techiman Municipal	190710	2015	53.6	46.7	60.6
Ghana	Brong Ahafo	Techiman North	190711	2000	106.5	97.5	116.9
Ghana	Brong Ahafo	Techiman North	190711	2005	93.3	85.4	101.6
Ghana	Brong Ahafo	Techiman North	190711	2010	74.0	67.1	81.7
Ghana	Brong Ahafo	Techiman North	190711	2015	56.7	49.3	64.3
Ghana	Brong Ahafo	Wenchi	190628	2000	106.2	96.5	117.2
Ghana	Brong Ahafo	Wenchi	190628	2005	93.1	84.6	102.4
Ghana	Brong Ahafo	Wenchi	190628	2010	74.1	66.8	81.9
Ghana	Brong Ahafo	Wenchi	190628	2015	56.3	49.0	64.4
Ghana	Central	Abura / Asebu / Kwamankese	16595	2000	110.8	102.1	119.5
Ghana	Central	Abura / Asebu / Kwamankese	16595	2005	94.4	87.2	102.7
Ghana	Central	Abura / Asebu / Kwamankese	16595	2010	78.1	71.6	84.9
Ghana	Central	Abura / Asebu / Kwamankese	16595	2015	61.9	55.0	69.4
Ghana	Central	Agona East	190585	2000	106.8	99.5	114.5
Ghana	Central	Agona East	190585	2005	89.4	83.3	95.2
Ghana	Central	Agona East	190585	2010	76.5	71.0	82.7
Ghana	Central	Agona East	190585	2015	58.1	51.7	64.8
Ghana	Central	Agona West	190584	2000	103.8	97.1	111.2
Ghana	Central	Agona West	190584	2005	89.2	83.2	95.2
Ghana	Central	Agona West	190584	2010	77.6	72.2	83.7
Ghana	Central	Agona West	190584	2015	58.1	51.8	64.7
Ghana	Central	Ajumako-Enyan-Esiam	16597	2000	115.2	106.6	124.6
Ghana	Central	Ajumako-Enyan-Esiam	16597	2005	97.2	90.0	104.9
Ghana	Central	Ajumako-Enyan-Esiam	16597	2010	80.9	74.3	88.0
Ghana	Central	Ajumako-Enyan-Esiam	16597	2015	65.0	57.5	73.3
Ghana	Central	Asikuma / Odoben / Brakwa	16598	2000	111.2	103.5	119.6
Ghana	Central	Asikuma / Odoben / Brakwa	16598	2005	95.0	88.4	101.6
Ghana	Central	Asikuma / Odoben / Brakwa	16598	2010	79.7	73.5	86.0
Ghana	Central	Asikuma / Odoben / Brakwa	16598	2015	63.3	56.4	70.6
Ghana	Central	Assin North	190586	2000	109.0	100.9	117.5
Ghana	Central	Assin North	190586	2005	92.5	85.5	99.8
Ghana	Central	Assin North	190586	2010	76.2	69.8	82.6
Ghana	Central	Assin North	190586	2015	59.9	52.7	66.9
Ghana	Central	Assin South	190587	2000	115.3	106.3	124.8
Ghana	Central	Assin South	190587	2005	96.8	89.5	105.1
Ghana	Central	Assin South	190587	2010	79.7	73.2	86.8
Ghana	Central	Assin South	190587	2015	64.2	56.7	71.9
Ghana	Central	Awutu Senya East Municipal	190652	2000	94.2	87.9	101.1
Ghana	Central	Awutu Senya East Municipal	190652	2005	78.9	73.9	84.1
Ghana	Central	Awutu Senya East Municipal	190652	2010	69.6	64.3	75.2
Ghana	Central	Awutu Senya East Municipal	190652	2015	51.6	46.0	57.6
Ghana	Central	Awutu Senya West	190651	2000	102.8	96.0	110.1
Ghana	Central	Awutu Senya West	190651	2005	83.6	78.2	89.1
Ghana	Central	Awutu Senya West	190651	2010	72.2	67.1	78.3
Ghana	Central	Awutu Senya West	190651	2015	54.6	48.8	61.2
Ghana	Central	Cape Coast Metro	16601	2000	101.1	92.2	110.3
Ghana	Central	Cape Coast Metro	16601	2005	89.5	81.6	98.3
Ghana	Central	Cape Coast Metro	16601	2010	75.0	67.9	82.4
Ghana	Central	Cape Coast Metro	16601	2015	57.7	50.9	65.4
Ghana	Central	Effutu	190588	2000	97.3	89.9	105.3
Ghana	Central	Effutu	190588	2005	82.5	76.1	88.6
Ghana	Central	Effutu	190588	2010	73.5	67.6	79.5
Ghana	Central	Effutu	190588	2015	55.4	49.0	61.9
Ghana	Central	Ekumfi	190648	2000	116.9	106.5	128.3
Ghana	Central	Ekumfi	190648	2005	98.1	89.7	107.4
Ghana	Central	Ekumfi	190648	2010	81.4	73.8	89.3
Ghana	Central	Ekumfi	190648	2015	67.1	59.1	76.0
Ghana	Central	Gomoa East	190589	2000	101.6	94.7	109.3
Ghana	Central	Gomoa East	190589	2005	84.3	78.6	89.9
Ghana	Central	Gomoa East	190589	2010	74.4	69.1	80.3
Ghana	Central	Gomoa East	190589	2015	56.3	49.9	62.8
Ghana	Central	Gomoa West	190583	2000	111.7	103.4	120.3
Ghana	Central	Gomoa West	190583	2005	94.9	88.1	102.2
Ghana	Central	Gomoa West	190583	2010	80.3	73.8	86.5
Ghana	Central	Gomoa West	190583	2015	63.7	56.3	71.4
Ghana	Central	Komenda Edna Eguafo / Abirem	16602	2000	105.0	95.4	115.2
Ghana	Central	Komenda Edna Eguafo / Abirem	16602	2005	93.2	84.9	102.5
Ghana	Central	Komenda Edna Eguafo / Abirem	16602	2010	76.0	68.9	83.8
Ghana	Central	Komenda Edna Eguafo / Abirem	16602	2015	60.8	53.6	68.9
Ghana	Central	Mfantsiman	190647	2000	113.8	105.0	123.1
Ghana	Central	Mfantsiman	190647	2005	95.2	87.9	103.6
Ghana	Central	Mfantsiman	190647	2010	78.8	72.3	85.7
Ghana	Central	Mfantsiman	190647	2015	63.3	56.2	71.2
Ghana	Central	Twifo Ati-Morkwa	190650	2000	108.0	99.4	117.6
Ghana	Central	Twifo Ati-Morkwa	190650	2005	92.4	85.4	100.2

Admin 0	Admin 1	Admin 2	GAUL Code	Year	Under-5 mortality (per 1,000 livebirths)		
					Estimate	Lower bound, 95% UI	Upper bound, 95% UI
Ghana	Central	Twifo Ati-Morkwa	190650	2010	75.6	69.0	82.5
Ghana	Central	Twifo Ati-Morkwa	190650	2015	60.3	53.3	67.6
Ghana	Central	Twifo Lower Denkyira	190649	2000	112.5	103.9	121.8
Ghana	Central	Twifo Lower Denkyira	190649	2005	96.5	89.3	105.0
Ghana	Central	Twifo Lower Denkyira	190649	2010	78.3	71.8	85.7
Ghana	Central	Twifo Lower Denkyira	190649	2015	63.1	55.6	70.4
Ghana	Central	Upper Denkyira East	190605	2000	100.9	92.5	109.7
Ghana	Central	Upper Denkyira East	190605	2005	88.0	80.7	95.2
Ghana	Central	Upper Denkyira East	190605	2010	72.7	66.0	79.7
Ghana	Central	Upper Denkyira East	190605	2015	55.7	49.1	62.8
Ghana	Central	Upper Denkyira West	190607	2000	103.4	95.2	111.0
Ghana	Central	Upper Denkyira West	190607	2005	88.7	81.7	95.7
Ghana	Central	Upper Denkyira West	190607	2010	73.2	66.6	79.4
Ghana	Central	Upper Denkyira West	190607	2015	55.8	49.6	62.7
Ghana	Eastern	Akwapem North	16607	2000	85.2	79.3	91.2
Ghana	Eastern	Akwapem North	16607	2005	75.4	70.0	80.6
Ghana	Eastern	Akwapem North	16607	2010	63.2	58.4	68.6
Ghana	Eastern	Akwapem North	16607	2015	46.4	41.3	52.1
Ghana	Eastern	Akwapem South	190673	2000	87.1	81.2	93.5
Ghana	Eastern	Akwapem South	190673	2005	75.3	69.9	80.9
Ghana	Eastern	Akwapem South	190673	2010	64.2	59.1	69.7
Ghana	Eastern	Akwapem South	190673	2015	47.2	41.9	53.1
Ghana	Eastern	Akyem Mansa	190596	2000	103.7	95.1	112.8
Ghana	Eastern	Akyem Mansa	190596	2005	88.1	81.1	95.8
Ghana	Eastern	Akyem Mansa	190596	2010	72.3	66.4	79.2
Ghana	Eastern	Akyem Mansa	190596	2015	53.9	47.4	60.9
Ghana	Eastern	Asuogyaman	16609	2000	88.2	81.4	95.9
Ghana	Eastern	Asuogyaman	16609	2005	76.8	70.6	83.3
Ghana	Eastern	Asuogyaman	16609	2010	63.5	57.8	69.7
Ghana	Eastern	Asuogyaman	16609	2015	47.6	41.9	53.8
Ghana	Eastern	Atiwa	190601	2000	84.4	78.0	91.1
Ghana	Eastern	Atiwa	190601	2005	76.1	70.6	82.4
Ghana	Eastern	Atiwa	190601	2010	65.1	60.0	70.7
Ghana	Eastern	Atiwa	190601	2015	47.3	42.0	53.2
Ghana	Eastern	Ayensuano	190679	2000	95.1	88.2	102.2
Ghana	Eastern	Ayensuano	190679	2005	81.4	75.4	87.6
Ghana	Eastern	Ayensuano	190679	2010	67.9	62.5	74.0
Ghana	Eastern	Ayensuano	190679	2015	50.2	44.6	56.5
Ghana	Eastern	Birim Municipal	190598	2000	101.1	93.7	108.5
Ghana	Eastern	Birim Municipal	190598	2005	88.0	81.6	94.4
Ghana	Eastern	Birim Municipal	190598	2010	74.5	68.8	80.8
Ghana	Eastern	Birim Municipal	190598	2015	54.8	48.5	61.5
Ghana	Eastern	Birim North	190602	2000	90.3	83.7	97.8
Ghana	Eastern	Birim North	190602	2005	80.4	74.7	86.5
Ghana	Eastern	Birim North	190602	2010	68.3	63.2	74.1
Ghana	Eastern	Birim North	190602	2015	49.3	43.9	55.3
Ghana	Eastern	Birim South	190600	2000	103.7	95.9	112.5
Ghana	Eastern	Birim South	190600	2005	89.4	82.8	96.1
Ghana	Eastern	Birim South	190600	2010	75.0	68.9	81.7
Ghana	Eastern	Birim South	190600	2015	55.7	49.0	62.6
Ghana	Eastern	Denkyembour	190674	2000	96.7	89.8	103.7
Ghana	Eastern	Denkyembour	190674	2005	83.0	77.1	89.1
Ghana	Eastern	Denkyembour	190674	2010	69.9	64.6	76.1
Ghana	Eastern	Denkyembour	190674	2015	51.7	45.7	58.2
Ghana	Eastern	East Akim	190680	2000	82.2	75.6	89.1
Ghana	Eastern	East Akim	190680	2005	74.5	68.9	80.9
Ghana	Eastern	East Akim	190680	2010	64.2	58.6	70.2
Ghana	Eastern	East Akim	190680	2015	46.3	41.0	52.2
Ghana	Eastern	Fanteakwa	16613	2000	86.5	79.6	93.9
Ghana	Eastern	Fanteakwa	16613	2005	75.7	69.8	82.2
Ghana	Eastern	Fanteakwa	16613	2010	64.5	59.2	70.4
Ghana	Eastern	Fanteakwa	16613	2015	48.5	42.7	54.8
Ghana	Eastern	Kwaebibirem	190675	2000	93.1	86.4	100.4
Ghana	Eastern	Kwaebibirem	190675	2005	81.6	76.2	88.1
Ghana	Eastern	Kwaebibirem	190675	2010	68.6	63.3	74.7
Ghana	Eastern	Kwaebibirem	190675	2015	50.0	44.3	56.2
Ghana	Eastern	Kwahu Afram Plains North	190677	2000	93.9	85.6	103.0
Ghana	Eastern	Kwahu Afram Plains North	190677	2005	77.2	70.2	85.2
Ghana	Eastern	Kwahu Afram Plains North	190677	2010	67.7	61.6	74.9
Ghana	Eastern	Kwahu Afram Plains North	190677	2015	55.3	48.5	62.1
Ghana	Eastern	Kwahu Afram Plains South	190676	2000	96.0	86.9	106.2
Ghana	Eastern	Kwahu Afram Plains South	190676	2005	80.4	72.8	89.3
Ghana	Eastern	Kwahu Afram Plains South	190676	2010	68.3	61.3	76.0
Ghana	Eastern	Kwahu Afram Plains South	190676	2015	55.1	47.9	62.6
Ghana	Eastern	Kwahu East	190603	2000	80.9	73.8	88.4
Ghana	Eastern	Kwahu East	190603	2005	74.3	68.1	81.3
Ghana	Eastern	Kwahu East	190603	2010	65.2	59.5	71.7
Ghana	Eastern	Kwahu East	190603	2015	47.4	41.6	53.7
Ghana	Eastern	Kwahu South	190597	2000	81.1	73.8	89.1
Ghana	Eastern	Kwahu South	190597	2005	72.4	66.0	79.2
Ghana	Eastern	Kwahu South	190597	2010	64.0	58.0	70.4
Ghana	Eastern	Kwahu South	190597	2015	47.1	41.3	53.4
Ghana	Eastern	Kwahu West	190604	2000	81.0	74.6	88.5
Ghana	Eastern	Kwahu West	190604	2005	74.0	67.9	80.5
Ghana	Eastern	Kwahu West	190604	2010	64.5	59.0	70.8

Admin 0	Admin 1	Admin 2	GAUL Code	Year	Under-5 mortality (per 1,000 livebirths) Estimate	Lower bound, 95% UI	Upper bound, 95% UI
Ghana	Eastern	Kwahu West	190604	2015	46.8	41.3	52.8
Ghana	Eastern	Lower Manya	190612	2000	90.7	83.9	98.5
Ghana	Eastern	Lower Manya	190612	2005	78.3	71.8	84.6
Ghana	Eastern	Lower Manya	190612	2010	62.4	57.2	68.0
Ghana	Eastern	Lower Manya	190612	2015	46.7	41.2	52.8
Ghana	Eastern	New Juaben Municipal	16617	2000	76.9	71.4	82.6
Ghana	Eastern	New Juaben Municipal	16617	2005	68.8	63.7	74.0
Ghana	Eastern	New Juaben Municipal	16617	2010	61.1	56.2	66.2
Ghana	Eastern	New Juaben Municipal	16617	2015	44.3	39.1	49.8
Ghana	Eastern	Nsawam Adoagyiri	190672	2000	86.0	80.3	92.2
Ghana	Eastern	Nsawam Adoagyiri	190672	2005	73.7	68.3	79.1
Ghana	Eastern	Nsawam Adoagyiri	190672	2010	64.0	59.0	69.7
Ghana	Eastern	Nsawam Adoagyiri	190672	2015	47.5	42.1	53.6
Ghana	Eastern	Suhum Municipal	190678	2000	88.5	82.1	95.3
Ghana	Eastern	Suhum Municipal	190678	2005	78.4	72.6	84.5
Ghana	Eastern	Suhum Municipal	190678	2010	66.0	60.6	71.7
Ghana	Eastern	Suhum Municipal	190678	2015	48.2	42.8	54.1
Ghana	Eastern	Upper Manya	190599	2000	92.0	84.9	99.7
Ghana	Eastern	Upper Manya	190599	2005	79.5	72.8	86.1
Ghana	Eastern	Upper Manya	190599	2010	65.2	59.3	71.2
Ghana	Eastern	Upper Manya	190599	2015	49.4	43.5	55.7
Ghana	Eastern	Upper West Akim	190681	2000	99.3	92.2	106.5
Ghana	Eastern	Upper West Akim	190681	2005	83.9	78.3	89.6
Ghana	Eastern	Upper West Akim	190681	2010	71.0	65.7	77.1
Ghana	Eastern	Upper West Akim	190681	2015	52.6	46.9	58.8
Ghana	Eastern	West Akim	190682	2000	97.8	90.4	105.1
Ghana	Eastern	West Akim	190682	2005	84.8	78.6	90.9
Ghana	Eastern	West Akim	190682	2010	71.7	66.3	77.9
Ghana	Eastern	West Akim	190682	2015	52.2	46.3	58.5
Ghana	Eastern	Yilo Krobo	16620	2000	86.4	80.0	93.2
Ghana	Eastern	Yilo Krobo	16620	2005	75.8	70.1	81.3
Ghana	Eastern	Yilo Krobo	16620	2010	62.7	57.7	68.0
Ghana	Eastern	Yilo Krobo	16620	2015	46.2	40.8	52.1
Ghana	Greater Accra	Accra Metropolis	190655	2000	66.5	61.4	71.7
Ghana	Greater Accra	Accra Metropolis	190655	2005	62.4	57.8	67.3
Ghana	Greater Accra	Accra Metropolis	190655	2010	57.0	52.1	62.3
Ghana	Greater Accra	Accra Metropolis	190655	2015	43.3	37.9	49.0
Ghana	Greater Accra	Ada East	190631	2000	88.2	79.6	97.4
Ghana	Greater Accra	Ada East	190631	2005	82.3	74.3	90.9
Ghana	Greater Accra	Ada East	190631	2010	64.3	57.5	71.8
Ghana	Greater Accra	Ada East	190631	2015	48.1	42.0	55.0
Ghana	Greater Accra	Ada West	190633	2000	89.2	80.3	99.0
Ghana	Greater Accra	Ada West	190633	2005	82.3	74.2	90.9
Ghana	Greater Accra	Ada West	190633	2010	63.6	56.9	70.7
Ghana	Greater Accra	Ada West	190633	2015	49.2	42.8	56.1
Ghana	Greater Accra	Adenta	190539	2000	66.9	62.1	72.0
Ghana	Greater Accra	Adenta	190539	2005	63.0	58.4	67.7
Ghana	Greater Accra	Adenta	190539	2010	56.5	52.1	61.3
Ghana	Greater Accra	Adenta	190539	2015	42.9	37.8	48.4
Ghana	Greater Accra	Ashaiman	190723	2000	69.1	63.8	74.8
Ghana	Greater Accra	Ashaiman	190723	2005	64.6	59.7	69.6
Ghana	Greater Accra	Ashaiman	190723	2010	56.5	51.8	61.9
Ghana	Greater Accra	Ashaiman	190723	2015	43.2	38.0	48.7
Ghana	Greater Accra	Ga Central Municipal	190658	2000	69.0	63.8	74.4
Ghana	Greater Accra	Ga Central Municipal	190658	2005	64.7	60.2	69.6
Ghana	Greater Accra	Ga Central Municipal	190658	2010	58.8	53.9	63.9
Ghana	Greater Accra	Ga Central Municipal	190658	2015	43.9	38.7	49.5
Ghana	Greater Accra	Ga East	190654	2000	68.7	63.9	74.0
Ghana	Greater Accra	Ga East	190654	2005	63.7	59.2	68.3
Ghana	Greater Accra	Ga East	190654	2010	57.5	52.9	62.6
Ghana	Greater Accra	Ga East	190654	2015	43.4	38.3	49.2
Ghana	Greater Accra	Ga South	190656	2000	81.1	76.0	86.8
Ghana	Greater Accra	Ga South	190656	2005	72.0	67.4	76.6
Ghana	Greater Accra	Ga South	190656	2010	64.4	59.7	69.7
Ghana	Greater Accra	Ga South	190656	2015	47.7	42.6	53.4
Ghana	Greater Accra	Ga West	190657	2000	76.7	71.8	82.2
Ghana	Greater Accra	Ga West	190657	2005	69.3	64.7	74.1
Ghana	Greater Accra	Ga West	190657	2010	61.1	56.4	66.2
Ghana	Greater Accra	Ga West	190657	2015	45.7	40.6	51.5
Ghana	Greater Accra	Kpone Katamanso	190724	2000	74.6	69.3	80.3
Ghana	Greater Accra	Kpone Katamanso	190724	2005	69.2	64.1	74.3
Ghana	Greater Accra	Kpone Katamanso	190724	2010	59.9	55.2	65.3
Ghana	Greater Accra	Kpone Katamanso	190724	2015	44.5	39.5	50.1
Ghana	Greater Accra	La Dade Kotopon	190635	2000	66.1	61.1	71.5
Ghana	Greater Accra	La Dade Kotopon	190635	2005	63.1	58.3	67.8
Ghana	Greater Accra	La Dade Kotopon	190635	2010	57.3	52.6	62.7
Ghana	Greater Accra	La Dade Kotopon	190635	2015	43.4	38.0	48.8
Ghana	Greater Accra	La Nkwantanang Madina	190653	2000	71.2	66.3	76.5
Ghana	Greater Accra	La Nkwantanang Madina	190653	2005	65.5	60.9	70.1
Ghana	Greater Accra	La Nkwantanang Madina	190653	2010	58.1	53.6	63.1
Ghana	Greater Accra	La Nkwantanang Madina	190653	2015	43.6	38.8	49.2
Ghana	Greater Accra	Ledzokuku / Krowor	190540	2000	64.8	59.7	70.3
Ghana	Greater Accra	Ledzokuku / Krowor	190540	2005	61.8	57.0	66.7
Ghana	Greater Accra	Ledzokuku / Krowor	190540	2010	55.8	51.0	61.0
Ghana	Greater Accra	Ledzokuku / Krowor	190540	2015	42.4	37.1	47.8

Admin 0	Admin 1	Admin 2	GAUL Code	Year	Under-5 mortality (per 1,000 livebirths)		
					Estimate	Lower bound, 95% UI	Upper bound, 95% UI
Ghana	Greater Accra	Ningo Prampram	190634	2000	84.2	77.7	91.3
Ghana	Greater Accra	Ningo Prampram	190634	2005	76.7	70.4	82.6
Ghana	Greater Accra	Ningo Prampram	190634	2010	62.3	57.1	68.1
Ghana	Greater Accra	Ningo Prampram	190634	2015	47.2	42.1	53.2
Ghana	Greater Accra	Shai Osu Doku	190632	2000	89.5	83.2	96.8
Ghana	Greater Accra	Shai Osu Doku	190632	2005	78.9	73.0	84.8
Ghana	Greater Accra	Shai Osu Doku	190632	2010	62.3	57.3	67.7
Ghana	Greater Accra	Shai Osu Doku	190632	2015	47.4	42.1	53.2
Ghana	Greater Accra	Tema Metropolis	190722	2000	68.0	62.9	73.6
Ghana	Greater Accra	Tema Metropolis	190722	2005	64.7	59.7	69.8
Ghana	Greater Accra	Tema Metropolis	190722	2010	57.2	52.4	62.6
Ghana	Greater Accra	Tema Metropolis	190722	2015	43.3	38.2	48.8
Ghana	Northern	Bole	190611	2000	141.8	126.9	157.2
Ghana	Northern	Bole	190611	2005	130.1	116.5	144.7
Ghana	Northern	Bole	190611	2010	97.3	87.0	109.7
Ghana	Northern	Bole	190611	2015	75.8	66.2	87.3
Ghana	Northern	Bunkpurugu Yonyo	190552	2000	135.2	124.9	145.6
Ghana	Northern	Bunkpurugu Yonyo	190552	2005	124.4	116.0	133.7
Ghana	Northern	Bunkpurugu Yonyo	190552	2010	97.3	89.8	105.5
Ghana	Northern	Bunkpurugu Yonyo	190552	2015	78.3	70.2	87.5
Ghana	Northern	Chereponi	190541	2000	146.9	135.0	159.8
Ghana	Northern	Chereponi	190541	2005	137.2	126.2	148.6
Ghana	Northern	Chereponi	190541	2010	106.3	97.2	115.9
Ghana	Northern	Chereponi	190541	2015	94.6	84.5	105.8
Ghana	Northern	East Gonja	190621	2000	127.1	116.9	138.7
Ghana	Northern	East Gonja	190621	2005	112.7	103.7	122.8
Ghana	Northern	East Gonja	190621	2010	86.0	78.2	94.6
Ghana	Northern	East Gonja	190621	2015	73.7	65.0	83.6
Ghana	Northern	Gonja Central	190618	2000	134.2	122.3	146.6
Ghana	Northern	Gonja Central	190618	2005	118.4	107.4	129.1
Ghana	Northern	Gonja Central	190618	2010	91.5	82.6	101.4
Ghana	Northern	Gonja Central	190618	2015	76.9	67.4	86.8
Ghana	Northern	Gushiegu	190625	2000	152.7	141.2	166.0
Ghana	Northern	Gushiegu	190625	2005	137.6	127.5	149.1
Ghana	Northern	Gushiegu	190625	2010	106.4	97.4	116.6
Ghana	Northern	Gushiegu	190625	2015	95.5	84.8	106.3
Ghana	Northern	Karaga	190620	2000	155.1	143.3	168.1
Ghana	Northern	Karaga	190620	2005	137.5	127.2	148.4
Ghana	Northern	Karaga	190620	2010	107.0	97.9	116.5
Ghana	Northern	Karaga	190620	2015	92.8	82.6	103.9
Ghana	Northern	Kpandai	190615	2000	129.0	119.1	141.4
Ghana	Northern	Kpandai	190615	2005	112.8	102.9	123.7
Ghana	Northern	Kpandai	190615	2010	85.7	78.2	94.2
Ghana	Northern	Kpandai	190615	2015	78.3	69.0	88.8
Ghana	Northern	Kumbumgu	190701	2000	152.6	140.6	165.3
Ghana	Northern	Kumbumgu	190701	2005	131.1	120.2	141.8
Ghana	Northern	Kumbumgu	190701	2010	101.0	92.1	110.8
Ghana	Northern	Kumbumgu	190701	2015	85.1	75.5	95.9
Ghana	Northern	Mamprugu Moagduri	190703	2000	145.5	132.2	161.1
Ghana	Northern	Mamprugu Moagduri	190703	2005	122.8	110.5	136.0
Ghana	Northern	Mamprugu Moagduri	190703	2010	99.3	88.8	110.7
Ghana	Northern	Mamprugu Moagduri	190703	2015	79.6	69.3	91.8
Ghana	Northern	Mamprusi East	190554	2000	141.1	130.5	152.4
Ghana	Northern	Mamprusi East	190554	2005	125.6	116.6	136.1
Ghana	Northern	Mamprusi East	190554	2010	99.6	90.7	108.4
Ghana	Northern	Mamprusi East	190554	2015	78.9	70.4	89.1
Ghana	Northern	Mion	190698	2000	145.9	133.7	158.6
Ghana	Northern	Mion	190698	2005	129.5	118.8	141.2
Ghana	Northern	Mion	190698	2010	96.8	88.2	106.4
Ghana	Northern	Mion	190698	2015	91.6	80.6	103.7
Ghana	Northern	Nanumba North	190626	2000	136.9	126.0	149.2
Ghana	Northern	Nanumba North	190626	2005	122.1	112.1	133.3
Ghana	Northern	Nanumba North	190626	2010	90.8	82.3	98.8
Ghana	Northern	Nanumba North	190626	2015	85.1	74.8	95.4
Ghana	Northern	Nanumba South	190627	2000	137.1	126.2	149.7
Ghana	Northern	Nanumba South	190627	2005	123.0	112.6	135.1
Ghana	Northern	Nanumba South	190627	2010	91.5	83.2	100.1
Ghana	Northern	Nanumba South	190627	2015	85.7	75.7	96.6
Ghana	Northern	North Gonja	190696	2000	148.9	135.0	164.6
Ghana	Northern	North Gonja	190696	2005	129.0	116.7	141.8
Ghana	Northern	North Gonja	190696	2010	101.6	91.2	113.3
Ghana	Northern	North Gonja	190696	2015	86.7	75.9	98.6
Ghana	Northern	Saboba	190624	2000	151.9	139.5	165.2
Ghana	Northern	Saboba	190624	2005	141.5	130.5	153.0
Ghana	Northern	Saboba	190624	2010	107.4	98.1	117.3
Ghana	Northern	Saboba	190624	2015	101.8	90.1	114.4
Ghana	Northern	Sagnerigu	190726	2000	131.4	121.0	143.0
Ghana	Northern	Sagnerigu	190726	2005	111.1	102.1	120.7
Ghana	Northern	Sagnerigu	190726	2010	85.0	77.0	93.4
Ghana	Northern	Sagnerigu	190726	2015	70.2	61.9	78.8
Ghana	Northern	Savelugu Nanton	16632	2000	152.2	141.1	164.8
Ghana	Northern	Savelugu Nanton	16632	2005	131.1	120.6	141.4
Ghana	Northern	Savelugu Nanton	16632	2010	99.1	90.7	108.5
Ghana	Northern	Savelugu Nanton	16632	2015	84.2	75.0	95.0
Ghana	Northern	Sawla/Tuna/Kalba	190549	2000	152.1	137.8	167.5

Admin 0	Admin 1	Admin 2	GAUL Code	Year	Under-5 mortality (per 1,000 livebirths)		
					Estimate	Lower bound, 95% UI	Upper bound, 95% UI
Ghana	Northern	Sawla/Tuna/Kalba	190549	2005	139.7	126.9	152.8
Ghana	Northern	Sawla/Tuna/Kalba	190549	2010	108.0	96.3	119.6
Ghana	Northern	Sawla/Tuna/Kalba	190549	2015	84.9	74.1	97.7
Ghana	Northern	Tamale North Sub Metro	190725	2000	126.5	115.7	137.9
Ghana	Northern	Tamale North Sub Metro	190725	2005	106.8	98.2	116.1
Ghana	Northern	Tamale North Sub Metro	190725	2010	82.6	74.5	90.4
Ghana	Northern	Tamale North Sub Metro	190725	2015	69.1	60.8	77.7
Ghana	Northern	Tatale	190705	2000	150.9	139.0	163.7
Ghana	Northern	Tatale	190705	2005	138.4	126.8	149.5
Ghana	Northern	Tatale	190705	2010	104.3	95.0	113.7
Ghana	Northern	Tatale	190705	2015	96.3	85.0	107.9
Ghana	Northern	Tolon	190700	2000	149.9	138.3	163.0
Ghana	Northern	Tolon	190700	2005	129.7	119.2	140.2
Ghana	Northern	Tolon	190700	2010	100.1	91.0	110.0
Ghana	Northern	Tolon	190700	2015	86.3	76.0	97.5
Ghana	Northern	West Gonja	190697	2000	137.6	122.0	154.0
Ghana	Northern	West Gonja	190697	2005	121.5	107.7	135.4
Ghana	Northern	West Gonja	190697	2010	93.3	81.8	104.5
Ghana	Northern	West Gonja	190697	2015	76.6	66.2	88.5
Ghana	Northern	West Mamprusi	190702	2000	148.0	135.9	160.0
Ghana	Northern	West Mamprusi	190702	2005	126.8	116.0	137.9
Ghana	Northern	West Mamprusi	190702	2010	101.1	91.7	110.9
Ghana	Northern	West Mamprusi	190702	2015	78.9	70.1	89.6
Ghana	Northern	Yendi Municipal	190699	2000	141.8	130.2	155.0
Ghana	Northern	Yendi Municipal	190699	2005	125.1	115.0	137.2
Ghana	Northern	Yendi Municipal	190699	2010	92.1	83.9	100.9
Ghana	Northern	Yendi Municipal	190699	2015	86.5	76.3	97.4
Ghana	Northern	Zabzugu	190704	2000	147.0	135.6	159.1
Ghana	Northern	Zabzugu	190704	2005	134.8	124.3	146.1
Ghana	Northern	Zabzugu	190704	2010	99.9	90.7	109.2
Ghana	Northern	Zabzugu	190704	2015	93.8	82.9	105.4
Ghana	Upper East	Bawku Municipal	190695	2000	110.2	102.2	118.1
Ghana	Upper East	Bawku Municipal	190695	2005	96.7	89.5	104.1
Ghana	Upper East	Bawku Municipal	190695	2010	74.5	68.0	81.5
Ghana	Upper East	Bawku Municipal	190695	2015	54.5	48.5	60.8
Ghana	Upper East	Bawku West	16640	2000	120.5	111.2	129.8
Ghana	Upper East	Bawku West	16640	2005	104.6	97.3	113.0
Ghana	Upper East	Bawku West	16640	2010	83.7	76.5	91.4
Ghana	Upper East	Bawku West	16640	2015	60.3	53.8	67.5
Ghana	Upper East	Binduri	190693	2000	115.0	107.1	123.1
Ghana	Upper East	Binduri	190693	2005	101.0	94.2	108.1
Ghana	Upper East	Binduri	190693	2010	79.9	73.2	86.9
Ghana	Upper East	Binduri	190693	2015	57.8	51.6	64.3
Ghana	Upper East	Bolgatanga Municipal	190546	2000	122.0	114.0	130.0
Ghana	Upper East	Bolgatanga Municipal	190546	2005	101.1	94.7	108.0
Ghana	Upper East	Bolgatanga Municipal	190546	2010	78.6	72.6	84.9
Ghana	Upper East	Bolgatanga Municipal	190546	2015	58.1	52.4	65.2
Ghana	Upper East	Bongo	16642	2000	124.8	116.6	133.2
Ghana	Upper East	Bongo	16642	2005	104.6	97.6	111.7
Ghana	Upper East	Bongo	16642	2010	80.9	74.4	87.6
Ghana	Upper East	Bongo	16642	2015	59.3	53.4	66.6
Ghana	Upper East	Builsa North	190692	2000	131.5	121.0	143.9
Ghana	Upper East	Builsa North	190692	2005	110.0	100.4	120.3
Ghana	Upper East	Builsa North	190692	2010	86.6	78.3	95.5
Ghana	Upper East	Builsa North	190692	2015	64.1	56.9	72.3
Ghana	Upper East	Builsa South	190691	2000	132.7	120.6	147.1
Ghana	Upper East	Builsa South	190691	2005	112.8	102.3	124.7
Ghana	Upper East	Builsa South	190691	2010	88.9	79.5	98.2
Ghana	Upper East	Builsa South	190691	2015	67.6	59.3	77.0
Ghana	Upper East	Garu Tempane	190553	2000	118.3	110.7	125.6
Ghana	Upper East	Garu Tempane	190553	2005	106.0	99.2	112.9
Ghana	Upper East	Garu Tempane	190553	2010	83.0	76.7	89.3
Ghana	Upper East	Garu Tempane	190553	2015	61.3	55.1	67.7
Ghana	Upper East	Kasena Nankana East	190551	2000	124.1	115.4	133.4
Ghana	Upper East	Kasena Nankana East	190551	2005	103.1	96.0	111.0
Ghana	Upper East	Kasena Nankana East	190551	2010	81.1	74.0	88.2
Ghana	Upper East	Kasena Nankana East	190551	2015	59.2	52.6	66.5
Ghana	Upper East	Kasena Nankana West	190547	2000	130.4	121.8	139.7
Ghana	Upper East	Kasena Nankana West	190547	2005	108.7	101.3	116.5
Ghana	Upper East	Kasena Nankana West	190547	2010	84.9	77.8	92.0
Ghana	Upper East	Kasena Nankana West	190547	2015	62.3	55.6	69.8
Ghana	Upper East	Nabdam	190690	2000	125.2	117.0	133.5
Ghana	Upper East	Nabdam	190690	2005	105.6	98.6	113.3
Ghana	Upper East	Nabdam	190690	2010	82.3	75.8	88.8
Ghana	Upper East	Nabdam	190690	2015	60.9	54.8	68.3
Ghana	Upper East	Pusiga	190694	2000	117.3	109.2	125.0
Ghana	Upper East	Pusiga	190694	2005	105.8	98.6	113.4
Ghana	Upper East	Pusiga	190694	2010	81.6	75.0	88.5
Ghana	Upper East	Pusiga	190694	2015	60.7	54.3	67.3
Ghana	Upper East	Talensi	190689	2000	126.8	118.3	136.0
Ghana	Upper East	Talensi	190689	2005	106.0	99.0	114.0
Ghana	Upper East	Talensi	190689	2010	83.3	76.5	90.1
Ghana	Upper East	Talensi	190689	2015	60.7	54.6	68.0
Ghana	Upper West	Daffiama Bussie	190686	2000	142.1	131.1	155.1
Ghana	Upper West	Daffiama Bussie	190686	2005	121.3	111.7	131.2

Admin 0	Admin 1	Admin 2	GAUL Code	Year	Under-5 mortality (per 1,000 livebirths) Estimate	Lower bound, 95% UI	Upper bound, 95% UI
Ghana	Upper West	Daffiama Bussie	190686	2010	92.5	83.9	101.3
Ghana	Upper West	Daffiama Bussie	190686	2015	73.4	65.2	82.7
Ghana	Upper West	Jirapa	190543	2000	148.8	139.2	159.1
Ghana	Upper West	Jirapa	190543	2005	129.7	121.4	138.8
Ghana	Upper West	Jirapa	190543	2010	98.6	90.7	107.0
Ghana	Upper West	Jirapa	190543	2015	78.3	70.4	87.8
Ghana	Upper West	Lambussie Karni	190545	2000	146.7	136.8	157.1
Ghana	Upper West	Lambussie Karni	190545	2005	127.5	118.7	136.8
Ghana	Upper West	Lambussie Karni	190545	2010	98.7	90.6	107.4
Ghana	Upper West	Lambussie Karni	190545	2015	78.7	70.3	88.7
Ghana	Upper West	Lawra	190687	2000	156.0	146.3	166.8
Ghana	Upper West	Lawra	190687	2005	137.0	128.0	147.0
Ghana	Upper West	Lawra	190687	2010	105.2	96.8	114.3
Ghana	Upper West	Lawra	190687	2015	81.2	72.6	90.6
Ghana	Upper West	Nadowli-Kaleo	190685	2000	151.5	141.7	161.0
Ghana	Upper West	Nadowli-Kaleo	190685	2005	131.0	123.3	139.7
Ghana	Upper West	Nadowli-Kaleo	190685	2010	99.0	90.8	107.3
Ghana	Upper West	Nadowli-Kaleo	190685	2015	77.3	69.0	86.8
Ghana	Upper West	Nandom	190688	2000	151.0	140.6	161.4
Ghana	Upper West	Nandom	190688	2005	131.1	122.3	140.7
Ghana	Upper West	Nandom	190688	2010	101.8	93.6	111.3
Ghana	Upper West	Nandom	190688	2015	80.7	72.3	91.0
Ghana	Upper West	Sissala West	190544	2000	143.7	132.7	155.7
Ghana	Upper West	Sissala West	190544	2005	123.9	114.4	133.4
Ghana	Upper West	Sissala West	190544	2010	96.4	87.9	105.8
Ghana	Upper West	Sissala West	190544	2015	75.5	67.2	84.8
Ghana	Upper West	Sissala East	190550	2000	135.4	124.0	147.4
Ghana	Upper West	Sissala East	190550	2005	115.9	105.8	126.2
Ghana	Upper West	Sissala East	190550	2010	88.3	79.6	96.9
Ghana	Upper West	Sissala East	190550	2015	69.3	61.5	78.5
Ghana	Upper West	Wa East	190623	2000	138.6	126.7	151.5
Ghana	Upper West	Wa East	190623	2005	119.0	108.7	130.1
Ghana	Upper West	Wa East	190623	2010	91.7	83.4	101.0
Ghana	Upper West	Wa East	190623	2015	73.4	64.7	83.5
Ghana	Upper West	Wa Municipal	190542	2000	140.1	129.5	151.9
Ghana	Upper West	Wa Municipal	190542	2005	119.1	110.1	129.2
Ghana	Upper West	Wa Municipal	190542	2010	90.9	82.7	99.9
Ghana	Upper West	Wa Municipal	190542	2015	70.4	62.1	80.6
Ghana	Upper West	Wa West	190548	2000	153.1	143.0	164.0
Ghana	Upper West	Wa West	190548	2005	137.8	128.2	148.0
Ghana	Upper West	Wa West	190548	2010	105.1	96.2	114.0
Ghana	Upper West	Wa West	190548	2015	80.4	71.0	90.7
Ghana	Volta	Adaklu	190662	2000	89.4	81.8	97.4
Ghana	Volta	Adaklu	190662	2005	81.3	74.6	88.5
Ghana	Volta	Adaklu	190662	2010	66.6	60.5	73.4
Ghana	Volta	Adaklu	190662	2015	52.3	45.8	59.1
Ghana	Volta	Afadzato South	190727	2000	77.4	70.8	84.3
Ghana	Volta	Afadzato South	190727	2005	68.6	63.3	74.8
Ghana	Volta	Afadzato South	190727	2010	63.8	58.3	69.7
Ghana	Volta	Afadzato South	190727	2015	49.2	43.5	55.7
Ghana	Volta	Agotime Ziope	190661	2000	92.8	84.8	101.0
Ghana	Volta	Agotime Ziope	190661	2005	82.5	75.8	89.8
Ghana	Volta	Agotime Ziope	190661	2010	66.4	60.6	73.0
Ghana	Volta	Agotime Ziope	190661	2015	54.2	47.7	61.1
Ghana	Volta	Akatsi North	190664	2000	99.7	91.6	108.6
Ghana	Volta	Akatsi North	190664	2005	86.1	79.4	93.4
Ghana	Volta	Akatsi North	190664	2010	67.2	61.5	73.8
Ghana	Volta	Akatsi North	190664	2015	54.8	48.2	61.3
Ghana	Volta	Akatsi South	190663	2000	98.6	90.3	107.4
Ghana	Volta	Akatsi South	190663	2005	86.1	79.1	93.7
Ghana	Volta	Akatsi South	190663	2010	66.9	60.8	73.5
Ghana	Volta	Akatsi South	190663	2015	51.3	44.9	57.7
Ghana	Volta	Biakoye	190617	2000	85.8	78.2	93.9
Ghana	Volta	Biakoye	190617	2005	73.1	66.7	80.2
Ghana	Volta	Biakoye	190617	2010	65.1	58.7	71.8
Ghana	Volta	Biakoye	190617	2015	52.5	46.1	59.4
Ghana	Volta	Central Tongu	190671	2000	95.6	88.0	103.6
Ghana	Volta	Central Tongu	190671	2005	84.5	77.8	91.3
Ghana	Volta	Central Tongu	190671	2010	66.6	60.7	73.2
Ghana	Volta	Central Tongu	190671	2015	51.8	45.4	58.6
Ghana	Volta	Ho Municipal	190665	2000	80.1	73.5	87.1
Ghana	Volta	Ho Municipal	190665	2005	73.6	67.4	80.2
Ghana	Volta	Ho Municipal	190665	2010	64.5	58.8	70.8
Ghana	Volta	Ho Municipal	190665	2015	50.1	44.0	56.8
Ghana	Volta	Ho West	190667	2000	79.1	72.8	86.0
Ghana	Volta	Ho West	190667	2005	71.5	65.8	77.3
Ghana	Volta	Ho West	190667	2010	64.2	58.6	70.2
Ghana	Volta	Ho West	190667	2015	48.9	43.2	55.3
Ghana	Volta	Hohoe Municipal	190666	2000	80.5	73.8	88.3
Ghana	Volta	Hohoe Municipal	190666	2005	71.5	65.2	78.4
Ghana	Volta	Hohoe Municipal	190666	2010	64.6	58.6	71.2
Ghana	Volta	Hohoe Municipal	190666	2015	51.5	45.7	58.7
Ghana	Volta	Jasikan	190564	2000	90.9	82.8	99.6
Ghana	Volta	Jasikan	190564	2005	78.8	71.9	86.6
Ghana	Volta	Jasikan	190564	2010	67.6	61.3	74.5

Admin 0	Admin 1	Admin 2	GAUL Code	Year	Under-5 mortality (per 1,000 livebirths)		
					Estimate	Lower bound, 95% UI	Upper bound, 95% UI
Ghana	Volta	Jasikan	190564	2015	56.0	49.4	63.6
Ghana	Volta	Kadjebi	16654	2000	100.4	91.6	110.2
Ghana	Volta	Kadjebi	16654	2005	87.2	78.9	96.1
Ghana	Volta	Kadjebi	16654	2010	71.5	64.1	79.5
Ghana	Volta	Kadjebi	16654	2015	64.7	56.9	73.2
Ghana	Volta	Keta Municipal	33108	2000	90.1	82.0	98.6
Ghana	Volta	Keta Municipal	33108	2005	81.2	73.8	89.0
Ghana	Volta	Keta Municipal	33108	2010	64.1	57.9	70.9
Ghana	Volta	Keta Municipal	33108	2015	46.1	40.1	52.9
Ghana	Volta	Ketu North	190562	2000	96.3	88.9	104.7
Ghana	Volta	Ketu North	190562	2005	83.9	77.4	90.7
Ghana	Volta	Ketu North	190562	2010	65.4	60.2	71.8
Ghana	Volta	Ketu North	190562	2015	50.2	44.2	56.5
Ghana	Volta	Ketu South	190563	2000	85.7	79.1	93.2
Ghana	Volta	Ketu South	190563	2005	76.8	70.8	83.3
Ghana	Volta	Ketu South	190563	2010	61.1	56.1	67.1
Ghana	Volta	Ketu South	190563	2015	45.1	39.8	51.1
Ghana	Volta	Kpando Municipal	190669	2000	77.1	69.8	85.1
Ghana	Volta	Kpando Municipal	190669	2005	67.8	61.4	74.5
Ghana	Volta	Kpando Municipal	190669	2010	63.8	57.6	70.6
Ghana	Volta	Kpando Municipal	190669	2015	49.4	43.5	55.8
Ghana	Volta	Krachi East	190565	2000	103.3	94.0	113.1
Ghana	Volta	Krachi East	190565	2005	85.9	77.6	94.1
Ghana	Volta	Krachi East	190565	2010	69.1	62.4	76.3
Ghana	Volta	Krachi East	190565	2015	59.7	52.2	67.7
Ghana	Volta	Krachi Nchumuru	190660	2000	112.3	102.3	123.3
Ghana	Volta	Krachi Nchumuru	190660	2005	95.5	86.4	105.2
Ghana	Volta	Krachi Nchumuru	190660	2010	72.5	65.4	80.7
Ghana	Volta	Krachi Nchumuru	190660	2015	63.4	55.4	72.5
Ghana	Volta	Krachi West	190659	2000	107.9	97.3	118.9
Ghana	Volta	Krachi West	190659	2005	90.2	81.1	99.3
Ghana	Volta	Krachi West	190659	2010	70.5	62.9	79.0
Ghana	Volta	Krachi West	190659	2015	60.1	52.3	68.0
Ghana	Volta	Nkwanta North	190616	2000	123.1	113.2	134.7
Ghana	Volta	Nkwanta North	190616	2005	109.2	100.4	119.2
Ghana	Volta	Nkwanta North	190616	2010	83.9	76.8	92.2
Ghana	Volta	Nkwanta North	190616	2015	78.6	69.2	88.5
Ghana	Volta	Nkwanta South	190566	2000	114.9	105.0	125.8
Ghana	Volta	Nkwanta South	190566	2005	98.4	90.1	107.8
Ghana	Volta	Nkwanta South	190566	2010	76.5	69.6	84.2
Ghana	Volta	Nkwanta South	190566	2015	70.2	62.0	79.5
Ghana	Volta	North Dayi	190668	2000	80.2	73.2	88.0
Ghana	Volta	North Dayi	190668	2005	70.7	64.3	77.1
Ghana	Volta	North Dayi	190668	2010	64.7	58.5	71.3
Ghana	Volta	North Dayi	190668	2015	49.8	43.7	56.3
Ghana	Volta	North Tongu	190670	2000	95.5	88.3	103.6
Ghana	Volta	North Tongu	190670	2005	82.5	76.0	89.2
Ghana	Volta	North Tongu	190670	2010	64.7	59.0	70.3
Ghana	Volta	North Tongu	190670	2015	49.3	43.3	55.7
Ghana	Volta	South Dayi	190622	2000	83.8	76.4	91.9
Ghana	Volta	South Dayi	190622	2005	73.9	67.4	80.9
Ghana	Volta	South Dayi	190622	2010	64.8	58.6	71.7
Ghana	Volta	South Dayi	190622	2015	49.1	42.9	55.5
Ghana	Volta	South Tongu	16661	2000	94.8	87.1	103.4
Ghana	Volta	South Tongu	16661	2005	85.0	77.8	92.7
Ghana	Volta	South Tongu	16661	2010	66.0	60.0	73.0
Ghana	Volta	South Tongu	16661	2015	49.0	42.8	55.5
Ghana	Western	Ahanta West	16662	2000	103.4	92.4	114.6
Ghana	Western	Ahanta West	16662	2005	90.5	81.2	100.2
Ghana	Western	Ahanta West	16662	2010	71.3	63.8	80.1
Ghana	Western	Ahanta West	16662	2015	57.5	50.0	65.3
Ghana	Western	Aowin	190636	2000	109.6	99.6	120.5
Ghana	Western	Aowin	190636	2005	97.3	88.2	107.1
Ghana	Western	Aowin	190636	2010	74.3	66.6	82.8
Ghana	Western	Aowin	190636	2015	60.0	52.6	68.8
Ghana	Western	Bia East	190638	2000	106.9	97.7	116.3
Ghana	Western	Bia East	190638	2005	96.6	87.7	106.3
Ghana	Western	Bia East	190638	2010	77.0	69.6	85.1
Ghana	Western	Bia East	190638	2015	60.5	52.9	68.4
Ghana	Western	Bia West	190639	2000	113.1	103.5	123.6
Ghana	Western	Bia West	190639	2005	99.8	90.9	109.8
Ghana	Western	Bia West	190639	2010	78.1	70.6	86.6
Ghana	Western	Bia West	190639	2015	62.6	54.9	70.7
Ghana	Western	Bodi	190640	2000	104.6	95.5	115.3
Ghana	Western	Bodi	190640	2005	93.1	85.2	102.0
Ghana	Western	Bodi	190640	2010	73.8	66.0	81.7
Ghana	Western	Bodi	190640	2015	58.7	51.5	66.6
Ghana	Western	Ellembelle	190590	2000	104.4	93.3	116.5
Ghana	Western	Ellembelle	190590	2005	92.8	83.7	103.0
Ghana	Western	Ellembelle	190590	2010	71.0	62.9	79.7
Ghana	Western	Ellembelle	190590	2015	58.8	51.0	67.7
Ghana	Western	Jomoro	16665	2000	107.7	95.4	120.3
Ghana	Western	Jomoro	16665	2005	97.9	86.8	110.0
Ghana	Western	Jomoro	16665	2010	73.6	65.0	83.3
Ghana	Western	Jomoro	16665	2015	62.2	53.6	71.7

Admin 0	Admin 1	Admin 2	GAUL Code	Year	Under-5 mortality (per 1,000 livebirths)		
					Estimate	Lower bound, 95% UI	Upper bound, 95% UI
Ghana	Western	Juabeso	190641	2000	107.7	98.6	118.2
Ghana	Western	Juabeso	190641	2005	95.2	86.8	104.5
Ghana	Western	Juabeso	190641	2010	74.8	67.5	83.2
Ghana	Western	Juabeso	190641	2015	59.5	52.0	67.4
Ghana	Western	Mpohor	190644	2000	102.7	93.4	112.4
Ghana	Western	Mpohor	190644	2005	91.7	83.7	99.9
Ghana	Western	Mpohor	190644	2010	71.5	64.5	78.6
Ghana	Western	Mpohor	190644	2015	57.6	50.4	64.9
Ghana	Western	Nzema East	190592	2000	105.2	94.8	116.6
Ghana	Western	Nzema East	190592	2005	93.3	84.6	103.2
Ghana	Western	Nzema East	190592	2010	71.5	63.8	79.5
Ghana	Western	Nzema East	190592	2015	59.3	51.7	67.7
Ghana	Western	Prestea / Huni Valley	190591	2000	98.8	90.2	108.1
Ghana	Western	Prestea / Huni Valley	190591	2005	88.2	81.2	96.0
Ghana	Western	Prestea / Huni Valley	190591	2010	69.5	63.0	76.1
Ghana	Western	Prestea / Huni Valley	190591	2015	55.8	48.9	63.1
Ghana	Western	Sefwi Akontombra	190594	2000	103.7	94.9	113.7
Ghana	Western	Sefwi Akontombra	190594	2005	91.5	83.8	99.9
Ghana	Western	Sefwi Akontombra	190594	2010	72.5	65.4	79.8
Ghana	Western	Sefwi Akontombra	190594	2015	57.5	50.6	64.9
Ghana	Western	Sefwi Bibiani-Anhwiaso Bekwai	16664	2000	97.6	89.7	106.1
Ghana	Western	Sefwi Bibiani-Anhwiaso Bekwai	16664	2005	85.6	78.6	93.0
Ghana	Western	Sefwi Bibiani-Anhwiaso Bekwai	16664	2010	70.4	63.9	77.3
Ghana	Western	Sefwi Bibiani-Anhwiaso Bekwai	16664	2015	52.0	45.8	58.6
Ghana	Western	Sefwi-Wiawso	190608	2000	99.3	91.4	108.5
Ghana	Western	Sefwi-Wiawso	190608	2005	87.7	80.3	94.8
Ghana	Western	Sefwi-Wiawso	190608	2010	71.1	64.2	77.9
Ghana	Western	Sefwi-Wiawso	190608	2015	55.3	48.6	62.0
Ghana	Western	Sekondi Takoradi	190595	2000	96.1	86.9	105.4
Ghana	Western	Sekondi Takoradi	190595	2005	86.6	78.1	95.2
Ghana	Western	Sekondi Takoradi	190595	2010	69.9	62.7	77.3
Ghana	Western	Sekondi Takoradi	190595	2015	55.9	48.7	63.4
Ghana	Western	Shama	190642	2000	95.4	87.0	104.5
Ghana	Western	Shama	190642	2005	86.6	78.1	95.2
Ghana	Western	Shama	190642	2010	70.4	63.4	77.8
Ghana	Western	Shama	190642	2015	56.3	49.2	63.7
Ghana	Western	Suaman	190637	2000	109.0	98.3	121.1
Ghana	Western	Suaman	190637	2005	97.6	88.3	108.2
Ghana	Western	Suaman	190637	2010	74.9	66.7	83.8
Ghana	Western	Suaman	190637	2015	60.2	52.3	68.7
Ghana	Western	Tarkwa Nsuaem	190593	2000	95.1	86.7	104.7
Ghana	Western	Tarkwa Nsuaem	190593	2005	86.6	79.2	95.1
Ghana	Western	Tarkwa Nsuaem	190593	2010	68.0	60.8	75.1
Ghana	Western	Tarkwa Nsuaem	190593	2015	55.4	48.2	63.0
Ghana	Western	Wassa Amenfi Central	190646	2000	102.9	94.1	111.5
Ghana	Western	Wassa Amenfi Central	190646	2005	89.9	82.4	97.3
Ghana	Western	Wassa Amenfi Central	190646	2010	71.7	65.1	78.5
Ghana	Western	Wassa Amenfi Central	190646	2015	56.4	49.7	63.2
Ghana	Western	Wassa Amenfi East	190721	2000	103.3	93.7	112.9
Ghana	Western	Wassa Amenfi East	190721	2005	89.6	81.8	97.7
Ghana	Western	Wassa Amenfi East	190721	2010	72.6	65.9	79.7
Ghana	Western	Wassa Amenfi East	190721	2015	56.2	49.5	63.2
Ghana	Western	Wassa Amenfi West	190645	2000	103.4	94.0	113.5
Ghana	Western	Wassa Amenfi West	190645	2005	90.8	82.5	99.4
Ghana	Western	Wassa Amenfi West	190645	2010	71.0	64.2	78.4
Ghana	Western	Wassa Amenfi West	190645	2015	56.8	50.1	64.5
Ghana	Western	Wassa East	190643	2000	108.5	100.4	117.7
Ghana	Western	Wassa East	190643	2005	94.2	87.2	102.0
Ghana	Western	Wassa East	190643	2010	75.4	68.7	82.0
Ghana	Western	Wassa East	190643	2015	60.6	53.6	68.1
Guinea	Boke	Boffa	40708	2000	148.4	134.8	163.1
Guinea	Boke	Boffa	40708	2005	126.1	113.9	139.7
Guinea	Boke	Boffa	40708	2010	104.0	92.6	115.3
Guinea	Boke	Boffa	40708	2015	83.4	72.9	94.5
Guinea	Boke	Boke	40709	2000	154.0	140.4	169.6
Guinea	Boke	Boke	40709	2005	130.1	119.2	143.1
Guinea	Boke	Boke	40709	2010	103.6	93.9	113.8
Guinea	Boke	Boke	40709	2015	84.0	73.8	94.4
Guinea	Boke	Gaoual	40710	2000	189.4	173.4	208.0
Guinea	Boke	Gaoual	40710	2005	167.1	152.1	183.6
Guinea	Boke	Gaoual	40710	2010	132.3	119.7	146.5
Guinea	Boke	Gaoual	40710	2015	106.4	94.1	119.2
Guinea	Boke	Koundara	40711	2000	196.5	177.6	216.1
Guinea	Boke	Koundara	40711	2005	172.3	157.0	190.6
Guinea	Boke	Koundara	40711	2010	134.4	120.5	149.0
Guinea	Boke	Koundara	40711	2015	101.0	89.2	114.2
Guinea	Conakry	Dixinn	40712	2000	89.8	80.8	98.8
Guinea	Conakry	Dixinn	40712	2005	86.7	77.8	95.7
Guinea	Conakry	Dixinn	40712	2010	71.2	63.2	79.1
Guinea	Conakry	Dixinn	40712	2015	49.9	43.1	57.4
Guinea	Conakry	Matam	40714	2000	89.8	80.8	98.8
Guinea	Conakry	Matam	40714	2005	86.7	77.8	95.7
Guinea	Conakry	Matam	40714	2010	71.2	63.2	79.1
Guinea	Conakry	Matam	40714	2015	49.9	43.1	57.4
Guinea	Conakry	Matoto	40715	2000	92.2	83.2	101.1

Admin 0	Admin 1	Admin 2	GAUL Code	Year	Under-5 mortality (per 1,000 livebirths)		
					Estimate	Lower bound, 95% UI	Upper bound, 95% UI
Guinea	Conakry	Matoto	40715	2005	86.9	78.3	95.9
Guinea	Conakry	Matoto	40715	2010	72.4	64.6	80.4
Guinea	Conakry	Matoto	40715	2015	51.0	44.4	58.1
Guinea	Conakry	Ratoma	40716	2000	98.6	89.6	107.7
Guinea	Conakry	Ratoma	40716	2005	91.8	83.3	100.9
Guinea	Conakry	Ratoma	40716	2010	75.4	67.6	83.2
Guinea	Conakry	Ratoma	40716	2015	52.7	46.1	59.8
Guinea	Faranah	Dabola	40717	2000	189.0	172.5	206.7
Guinea	Faranah	Dabola	40717	2005	170.1	154.4	186.7
Guinea	Faranah	Dabola	40717	2010	148.8	133.8	165.7
Guinea	Faranah	Dabola	40717	2015	124.0	107.6	141.5
Guinea	Faranah	Dinguiraye	40718	2000	204.8	185.7	223.9
Guinea	Faranah	Dinguiraye	40718	2005	184.1	167.6	202.0
Guinea	Faranah	Dinguiraye	40718	2010	158.1	142.2	176.3
Guinea	Faranah	Dinguiraye	40718	2015	135.3	118.3	153.9
Guinea	Faranah	Faranah	40719	2000	205.6	189.6	221.3
Guinea	Faranah	Faranah	40719	2005	179.4	164.9	194.7
Guinea	Faranah	Faranah	40719	2010	161.5	145.8	177.5
Guinea	Faranah	Faranah	40719	2015	128.9	113.5	145.3
Guinea	Faranah	Kissidougou	40720	2000	204.5	189.3	221.5
Guinea	Faranah	Kissidougou	40720	2005	169.4	156.0	184.2
Guinea	Faranah	Kissidougou	40720	2010	146.8	132.6	162.9
Guinea	Faranah	Kissidougou	40720	2015	125.3	109.0	142.0
Guinea	Kankan	Kankan	40721	2000	198.5	182.5	214.7
Guinea	Kankan	Kankan	40721	2005	187.5	172.0	204.1
Guinea	Kankan	Kankan	40721	2010	165.3	149.8	181.7
Guinea	Kankan	Kankan	40721	2015	137.3	121.1	155.2
Guinea	Kankan	Kerouane	40722	2000	213.9	195.8	232.0
Guinea	Kankan	Kerouane	40722	2005	185.3	169.1	202.8
Guinea	Kankan	Kerouane	40722	2010	151.4	135.4	168.4
Guinea	Kankan	Kerouane	40722	2015	126.3	111.4	143.3
Guinea	Kankan	Kouroussa	40723	2000	217.1	199.4	235.9
Guinea	Kankan	Kouroussa	40723	2005	200.0	183.5	218.9
Guinea	Kankan	Kouroussa	40723	2010	174.9	159.3	194.2
Guinea	Kankan	Kouroussa	40723	2015	147.6	130.2	166.3
Guinea	Kankan	Mandiana	40724	2000	205.6	189.8	221.7
Guinea	Kankan	Mandiana	40724	2005	196.3	181.2	212.6
Guinea	Kankan	Mandiana	40724	2010	175.2	159.5	191.4
Guinea	Kankan	Mandiana	40724	2015	145.7	127.5	164.0
Guinea	Kankan	Siguiri	40725	2000	207.4	191.3	224.4
Guinea	Kankan	Siguiri	40725	2005	195.0	179.6	211.3
Guinea	Kankan	Siguiri	40725	2010	170.1	155.3	185.4
Guinea	Kankan	Siguiri	40725	2015	146.1	130.0	163.4
Guinea	Kindia	Coyah	40726	2000	147.2	135.0	160.3
Guinea	Kindia	Coyah	40726	2005	127.5	116.8	139.6
Guinea	Kindia	Coyah	40726	2010	104.5	94.9	115.5
Guinea	Kindia	Coyah	40726	2015	87.1	77.1	99.1
Guinea	Kindia	Dubreka	40727	2000	160.4	146.6	174.2
Guinea	Kindia	Dubreka	40727	2005	137.4	125.1	149.6
Guinea	Kindia	Dubreka	40727	2010	114.7	103.5	126.4
Guinea	Kindia	Dubreka	40727	2015	96.5	84.7	108.7
Guinea	Kindia	Forecariah	40728	2000	163.2	151.0	175.3
Guinea	Kindia	Forecariah	40728	2005	144.9	134.6	155.7
Guinea	Kindia	Forecariah	40728	2010	121.5	111.3	132.2
Guinea	Kindia	Forecariah	40728	2015	104.4	92.8	116.4
Guinea	Kindia	Fria	40729	2000	148.6	134.7	163.8
Guinea	Kindia	Fria	40729	2005	124.6	112.7	137.6
Guinea	Kindia	Fria	40729	2010	104.1	93.8	115.8
Guinea	Kindia	Fria	40729	2015	88.5	77.0	100.8
Guinea	Kindia	Kindia	40730	2000	159.1	146.4	172.5
Guinea	Kindia	Kindia	40730	2005	139.0	127.1	151.5
Guinea	Kindia	Kindia	40730	2010	121.7	110.3	133.6
Guinea	Kindia	Kindia	40730	2015	105.8	93.0	118.5
Guinea	Kindia	Telemele	40731	2000	184.5	169.1	200.5
Guinea	Kindia	Telemele	40731	2005	156.8	143.7	171.7
Guinea	Kindia	Telemele	40731	2010	129.6	117.7	143.0
Guinea	Kindia	Telemele	40731	2015	110.2	97.2	124.3
Guinea	Labe	Koubia	40732	2000	187.6	172.1	204.3
Guinea	Labe	Koubia	40732	2005	159.1	145.5	173.3
Guinea	Labe	Koubia	40732	2010	131.6	119.1	144.7
Guinea	Labe	Koubia	40732	2015	110.8	97.6	125.6
Guinea	Labe	Labe	40733	2000	152.1	141.0	163.9
Guinea	Labe	Labe	40733	2005	131.7	121.3	142.5
Guinea	Labe	Labe	40733	2010	108.3	98.7	118.4
Guinea	Labe	Labe	40733	2015	91.1	80.7	102.7
Guinea	Labe	Lelouma	40734	2000	180.1	166.0	194.9
Guinea	Labe	Lelouma	40734	2005	152.7	139.4	166.3
Guinea	Labe	Lelouma	40734	2010	123.7	112.3	135.2
Guinea	Labe	Lelouma	40734	2015	103.4	91.3	116.3
Guinea	Labe	Mali	40735	2000	209.2	194.1	226.8
Guinea	Labe	Mali	40735	2005	171.4	158.2	186.1
Guinea	Labe	Mali	40735	2010	139.1	127.0	152.5
Guinea	Labe	Mali	40735	2015	113.9	101.4	128.1
Guinea	Labe	Tougue	40736	2000	176.9	160.4	195.3
Guinea	Labe	Tougue	40736	2005	154.4	140.0	170.4

Admin 0	Admin 1	Admin 2	GAUL Code	Year	Under-5 mortality (per 1,000 livebirths)		
					Estimate	Lower bound, 95% UI	Upper bound, 95% UI
Guinea	Labe	Tougue	40736	2010	131.4	117.8	147.1
Guinea	Labe	Tougue	40736	2015	110.6	97.2	126.2
Guinea	Mamou	Dalaba	40737	2000	149.5	138.0	161.8
Guinea	Mamou	Dalaba	40737	2005	134.5	123.5	145.6
Guinea	Mamou	Dalaba	40737	2010	113.1	102.9	123.6
Guinea	Mamou	Dalaba	40737	2015	94.3	83.5	104.7
Guinea	Mamou	Mamou	40738	2000	163.1	151.3	175.0
Guinea	Mamou	Mamou	40738	2005	147.2	136.3	160.0
Guinea	Mamou	Mamou	40738	2010	126.6	115.2	138.7
Guinea	Mamou	Mamou	40738	2015	104.0	92.5	115.5
Guinea	Mamou	Pita	40739	2000	158.7	146.6	171.0
Guinea	Mamou	Pita	40739	2005	139.5	128.9	150.6
Guinea	Mamou	Pita	40739	2010	115.8	105.4	126.0
Guinea	Mamou	Pita	40739	2015	98.0	86.8	110.0
Guinea	Nzerekore	Beyla	40740	2000	208.1	189.8	228.0
Guinea	Nzerekore	Beyla	40740	2005	177.0	161.2	195.4
Guinea	Nzerekore	Beyla	40740	2010	146.6	131.7	162.7
Guinea	Nzerekore	Beyla	40740	2015	119.6	104.7	136.9
Guinea	Nzerekore	Gueckedou	40741	2000	195.8	182.8	208.5
Guinea	Nzerekore	Gueckedou	40741	2005	158.0	148.1	169.0
Guinea	Nzerekore	Gueckedou	40741	2010	137.0	125.9	148.7
Guinea	Nzerekore	Gueckedou	40741	2015	117.5	105.2	130.6
Guinea	Nzerekore	Lola	40742	2000	184.7	167.4	203.6
Guinea	Nzerekore	Lola	40742	2005	147.1	133.0	162.7
Guinea	Nzerekore	Lola	40742	2010	117.3	104.7	132.1
Guinea	Nzerekore	Lola	40742	2015	95.1	82.7	110.3
Guinea	Nzerekore	Macenta	40743	2000	187.9	173.7	203.7
Guinea	Nzerekore	Macenta	40743	2005	147.8	135.8	160.9
Guinea	Nzerekore	Macenta	40743	2010	116.8	105.9	130.0
Guinea	Nzerekore	Macenta	40743	2015	94.6	83.7	106.6
Guinea	Nzerekore	Nzerekore	40744	2000	154.1	139.9	168.5
Guinea	Nzerekore	Nzerekore	40744	2005	120.7	109.8	133.0
Guinea	Nzerekore	Nzerekore	40744	2010	96.0	86.2	106.6
Guinea	Nzerekore	Nzerekore	40744	2015	78.2	67.4	90.0
Guinea	Nzerekore	Yomou	40745	2000	149.2	138.0	161.5
Guinea	Nzerekore	Yomou	40745	2005	116.2	106.8	125.9
Guinea	Nzerekore	Yomou	40745	2010	95.5	86.3	104.4
Guinea	Nzerekore	Yomou	40745	2015	77.2	67.6	86.8
Guinea-Bissau	Bafata	Bafata	17068	2000	216.3	200.8	234.1
Guinea-Bissau	Bafata	Bafata	17068	2005	183.2	171.5	196.8
Guinea-Bissau	Bafata	Bafata	17068	2010	136.1	125.8	147.6
Guinea-Bissau	Bafata	Bafata	17068	2015	111.5	100.2	123.1
Guinea-Bissau	Bafata	Bambadinca	17069	2000	190.4	176.9	204.4
Guinea-Bissau	Bafata	Bambadinca	17069	2005	159.6	149.0	170.8
Guinea-Bissau	Bafata	Bambadinca	17069	2010	119.6	110.6	129.2
Guinea-Bissau	Bafata	Bambadinca	17069	2015	98.3	88.5	108.7
Guinea-Bissau	Bafata	Contuboel	17070	2000	214.5	200.5	229.6
Guinea-Bissau	Bafata	Contuboel	17070	2005	186.9	175.0	199.0
Guinea-Bissau	Bafata	Contuboel	17070	2010	135.7	125.6	146.8
Guinea-Bissau	Bafata	Contuboel	17070	2015	106.6	96.2	117.0
Guinea-Bissau	Bafata	Galomaro/cosse	17071	2000	213.5	196.5	232.1
Guinea-Bissau	Bafata	Galomaro/cosse	17071	2005	181.7	168.3	196.8
Guinea-Bissau	Bafata	Galomaro/cosse	17071	2010	139.5	127.9	151.8
Guinea-Bissau	Bafata	Galomaro/cosse	17071	2015	114.2	102.2	126.9
Guinea-Bissau	Bafata	Gamamudo/ganadu	17072	2000	210.7	197.4	225.5
Guinea-Bissau	Bafata	Gamamudo/ganadu	17072	2005	177.9	167.1	189.1
Guinea-Bissau	Bafata	Gamamudo/ganadu	17072	2010	129.9	120.3	139.8
Guinea-Bissau	Bafata	Gamamudo/ganadu	17072	2015	105.4	95.5	115.7
Guinea-Bissau	Bafata	Xitole	17073	2000	188.9	173.2	206.7
Guinea-Bissau	Bafata	Xitole	17073	2005	160.6	148.0	174.9
Guinea-Bissau	Bafata	Xitole	17073	2010	125.3	115.1	136.6
Guinea-Bissau	Bafata	Xitole	17073	2015	102.3	92.1	114.5
Guinea-Bissau	Biombo	Prabis	17074	2000	128.8	118.1	140.6
Guinea-Bissau	Biombo	Prabis	17074	2005	100.3	92.1	108.8
Guinea-Bissau	Biombo	Prabis	17074	2010	79.7	72.7	86.8
Guinea-Bissau	Biombo	Prabis	17074	2015	59.9	53.4	66.9
Guinea-Bissau	Biombo	Quinhamel	17075	2000	129.0	117.6	140.6
Guinea-Bissau	Biombo	Quinhamel	17075	2005	98.8	90.2	107.8
Guinea-Bissau	Biombo	Quinhamel	17075	2010	78.3	71.0	85.6
Guinea-Bissau	Biombo	Quinhamel	17075	2015	58.8	52.2	65.5
Guinea-Bissau	Biombo	Safim	17076	2000	120.7	111.1	131.0
Guinea-Bissau	Biombo	Safim	17076	2005	90.6	83.7	97.9
Guinea-Bissau	Biombo	Safim	17076	2010	72.7	66.9	78.4
Guinea-Bissau	Biombo	Safim	17076	2015	55.2	49.5	61.2
Guinea-Bissau	Bolama/bijagos	Bolama	17077	2000	129.1	117.5	140.7
Guinea-Bissau	Bolama/bijagos	Bolama	17077	2005	100.9	92.2	110.3
Guinea-Bissau	Bolama/bijagos	Bolama	17077	2010	79.5	72.0	87.7
Guinea-Bissau	Bolama/bijagos	Bolama	17077	2015	64.3	57.0	72.1
Guinea-Bissau	Bolama/bijagos	Bubaque	17078	2000	112.7	95.6	130.4
Guinea-Bissau	Bolama/bijagos	Bubaque	17078	2005	83.8	72.1	97.1
Guinea-Bissau	Bolama/bijagos	Bubaque	17078	2010	72.1	61.9	84.2
Guinea-Bissau	Bolama/bijagos	Bubaque	17078	2015	55.9	47.0	65.9
Guinea-Bissau	Bolama/bijagos	Caravela	17079	2000	114.2	97.6	131.9
Guinea-Bissau	Bolama/bijagos	Caravela	17079	2005	84.7	73.2	97.7
Guinea-Bissau	Bolama/bijagos	Caravela	17079	2010	73.1	63.2	85.1

Admin 0	Admin 1	Admin 2	GAUL Code	Year	Under-5 mortality (per 1,000 livebirths)		
					Estimate	Lower bound, 95% UI	Upper bound, 95% UI
Guinea-Bissau	Bolama/bijagos	Caravela	17079	2015	55.7	47.3	64.9
Guinea-Bissau	Bolama/bijagos	Uno	17080	2000	101.3	83.8	121.8
Guinea-Bissau	Bolama/bijagos	Uno	17080	2005	83.0	68.1	99.7
Guinea-Bissau	Bolama/bijagos	Uno	17080	2010	73.3	60.6	87.5
Guinea-Bissau	Bolama/bijagos	Uno	17080	2015	57.1	46.6	68.3
Guinea-Bissau	Cacheu	Bigene	17081	2000	168.3	157.9	179.5
Guinea-Bissau	Cacheu	Bigene	17081	2005	128.5	120.2	137.2
Guinea-Bissau	Cacheu	Bigene	17081	2010	93.8	87.0	100.5
Guinea-Bissau	Cacheu	Bigene	17081	2015	69.9	63.1	76.8
Guinea-Bissau	Cacheu	Bula	17082	2000	150.0	139.4	160.8
Guinea-Bissau	Cacheu	Bula	17082	2005	114.2	106.3	122.7
Guinea-Bissau	Cacheu	Bula	17082	2010	84.6	78.1	91.0
Guinea-Bissau	Cacheu	Bula	17082	2015	64.6	58.4	71.2
Guinea-Bissau	Cacheu	Cacheu/calequisse	17083	2000	142.6	129.8	155.7
Guinea-Bissau	Cacheu	Cacheu/calequisse	17083	2005	107.7	98.3	117.6
Guinea-Bissau	Cacheu	Cacheu/calequisse	17083	2010	83.9	75.9	92.0
Guinea-Bissau	Cacheu	Cacheu/calequisse	17083	2015	64.1	56.7	71.9
Guinea-Bissau	Cacheu	Caio	17084	2000	132.6	117.9	147.2
Guinea-Bissau	Cacheu	Caio	17084	2005	101.6	90.9	114.0
Guinea-Bissau	Cacheu	Caio	17084	2010	81.7	72.7	91.3
Guinea-Bissau	Cacheu	Caio	17084	2015	61.8	54.2	69.8
Guinea-Bissau	Cacheu	Canchungo	17085	2000	139.8	126.8	153.3
Guinea-Bissau	Cacheu	Canchungo	17085	2005	105.8	96.4	115.5
Guinea-Bissau	Cacheu	Canchungo	17085	2010	82.2	74.3	90.4
Guinea-Bissau	Cacheu	Canchungo	17085	2015	63.2	56.1	71.0
Guinea-Bissau	Cacheu	Sao Domingos	17086	2000	134.1	122.9	144.9
Guinea-Bissau	Cacheu	Sao Domingos	17086	2005	101.7	94.2	110.2
Guinea-Bissau	Cacheu	Sao Domingos	17086	2010	81.9	74.6	89.4
Guinea-Bissau	Cacheu	Sao Domingos	17086	2015	61.9	55.2	69.6
Guinea-Bissau	Gabu	Boe	17087	2000	193.5	176.1	213.1
Guinea-Bissau	Gabu	Boe	17087	2005	177.9	161.5	196.7
Guinea-Bissau	Gabu	Boe	17087	2010	145.3	131.4	160.5
Guinea-Bissau	Gabu	Boe	17087	2015	111.0	98.5	125.0
Guinea-Bissau	Gabu	Gabu	17088	2000	216.9	200.0	234.8
Guinea-Bissau	Gabu	Gabu	17088	2005	194.6	181.1	210.0
Guinea-Bissau	Gabu	Gabu	17088	2010	153.7	142.2	166.4
Guinea-Bissau	Gabu	Gabu	17088	2015	111.9	101.0	123.8
Guinea-Bissau	Gabu	Pirada	17089	2000	219.6	205.0	235.2
Guinea-Bissau	Gabu	Pirada	17089	2005	201.8	189.0	216.8
Guinea-Bissau	Gabu	Pirada	17089	2010	158.5	147.2	171.3
Guinea-Bissau	Gabu	Pirada	17089	2015	109.9	99.2	121.9
Guinea-Bissau	Gabu	Pitche	17090	2000	209.5	192.5	228.0
Guinea-Bissau	Gabu	Pitche	17090	2005	193.6	178.3	209.7
Guinea-Bissau	Gabu	Pitche	17090	2010	154.3	142.0	168.5
Guinea-Bissau	Gabu	Pitche	17090	2015	103.5	92.6	115.4
Guinea-Bissau	Gabu	Sonaco	17091	2000	227.0	211.9	243.6
Guinea-Bissau	Gabu	Sonaco	17091	2005	202.3	189.2	216.3
Guinea-Bissau	Gabu	Sonaco	17091	2010	154.9	143.9	167.1
Guinea-Bissau	Gabu	Sonaco	17091	2015	113.7	102.8	125.4
Guinea-Bissau	Oio	Bissora	17092	2000	161.5	151.2	172.2
Guinea-Bissau	Oio	Bissora	17092	2005	126.8	119.1	135.4
Guinea-Bissau	Oio	Bissora	17092	2010	93.7	87.0	100.4
Guinea-Bissau	Oio	Bissora	17092	2015	69.2	62.3	75.9
Guinea-Bissau	Oio	Farim	17093	2000	186.1	176.0	197.2
Guinea-Bissau	Oio	Farim	17093	2005	148.9	140.1	157.6
Guinea-Bissau	Oio	Farim	17093	2010	104.2	97.5	111.3
Guinea-Bissau	Oio	Farim	17093	2015	78.1	70.8	85.9
Guinea-Bissau	Oio	Mansaba	17094	2000	177.0	165.6	189.9
Guinea-Bissau	Oio	Mansaba	17094	2005	143.0	133.9	153.2
Guinea-Bissau	Oio	Mansaba	17094	2010	103.3	95.5	110.7
Guinea-Bissau	Oio	Mansaba	17094	2015	80.1	72.3	88.7
Guinea-Bissau	Oio	Mansoa	17095	2000	154.5	143.9	166.4
Guinea-Bissau	Oio	Mansoa	17095	2005	125.3	116.5	135.4
Guinea-Bissau	Oio	Mansoa	17095	2010	93.8	86.7	101.3
Guinea-Bissau	Oio	Mansoa	17095	2015	73.5	66.2	82.0
Guinea-Bissau	Oio	Nhacra	17096	2000	138.7	129.1	148.9
Guinea-Bissau	Oio	Nhacra	17096	2005	109.9	102.6	118.2
Guinea-Bissau	Oio	Nhacra	17096	2010	83.7	77.6	90.0
Guinea-Bissau	Oio	Nhacra	17096	2015	64.1	57.8	71.1
Guinea-Bissau	Quinara	Buba	17097	2000	153.8	141.9	166.5
Guinea-Bissau	Quinara	Buba	17097	2005	128.4	119.1	138.2
Guinea-Bissau	Quinara	Buba	17097	2010	100.2	92.2	108.7
Guinea-Bissau	Quinara	Buba	17097	2015	82.9	74.1	92.4
Guinea-Bissau	Quinara	Empada	17098	2000	136.2	124.4	147.4
Guinea-Bissau	Quinara	Empada	17098	2005	109.7	100.9	118.7
Guinea-Bissau	Quinara	Empada	17098	2010	87.1	79.6	94.6
Guinea-Bissau	Quinara	Empada	17098	2015	73.0	65.3	81.0
Guinea-Bissau	Quinara	Fulacunda	17099	2000	151.1	140.1	162.2
Guinea-Bissau	Quinara	Fulacunda	17099	2005	125.2	116.7	134.4
Guinea-Bissau	Quinara	Fulacunda	17099	2010	95.8	88.5	103.3
Guinea-Bissau	Quinara	Fulacunda	17099	2015	77.3	69.8	85.9
Guinea-Bissau	Quinara	Tite	17100	2000	135.4	125.6	145.5
Guinea-Bissau	Quinara	Tite	17100	2005	108.3	100.5	117.0
Guinea-Bissau	Quinara	Tite	17100	2010	82.3	75.3	88.7
Guinea-Bissau	Quinara	Tite	17100	2015	65.8	59.0	73.1

Admin 0	Admin 1	Admin 2	GAUL Code	Year	Under-5 mortality (per 1,000 livebirths)		
					Estimate	Lower bound, 95% UI	Upper bound, 95% UI
Guinea-Bissau	Sector Autonomo De Bissau	Sector Autonomo De Bissau	17101	2000	96.7	89.2	104.7
Guinea-Bissau	Sector Autonomo De Bissau	Sector Autonomo De Bissau	17101	2005	79.6	73.6	85.8
Guinea-Bissau	Sector Autonomo De Bissau	Sector Autonomo De Bissau	17101	2010	66.2	60.9	71.5
Guinea-Bissau	Sector Autonomo De Bissau	Sector Autonomo De Bissau	17101	2015	51.0	45.7	56.7
Guinea-Bissau	Tombali	Bedanda	17102	2000	133.9	121.0	146.8
Guinea-Bissau	Tombali	Bedanda	17102	2005	112.1	102.0	123.2
Guinea-Bissau	Tombali	Bedanda	17102	2010	91.3	82.7	100.3
Guinea-Bissau	Tombali	Bedanda	17102	2015	77.0	68.1	86.1
Guinea-Bissau	Tombali	Cacine	17103	2000	129.9	116.7	144.6
Guinea-Bissau	Tombali	Cacine	17103	2005	108.4	97.4	119.9
Guinea-Bissau	Tombali	Cacine	17103	2010	91.5	82.0	101.5
Guinea-Bissau	Tombali	Cacine	17103	2015	75.8	66.7	85.1
Guinea-Bissau	Tombali	Catio	17104	2000	128.9	116.3	141.9
Guinea-Bissau	Tombali	Catio	17104	2005	104.6	94.8	115.6
Guinea-Bissau	Tombali	Catio	17104	2010	85.6	77.3	94.5
Guinea-Bissau	Tombali	Catio	17104	2015	72.3	63.7	81.5
Guinea-Bissau	Tombali	Komo	17105	2000	114.7	101.0	129.3
Guinea-Bissau	Tombali	Komo	17105	2005	91.4	80.8	103.0
Guinea-Bissau	Tombali	Komo	17105	2010	78.1	68.7	88.3
Guinea-Bissau	Tombali	Komo	17105	2015	61.9	53.6	71.2
Guinea-Bissau	Tombali	Quebo	17106	2000	162.9	148.8	177.5
Guinea-Bissau	Tombali	Quebo	17106	2005	137.5	126.5	150.0
Guinea-Bissau	Tombali	Quebo	17106	2010	109.1	99.7	119.8
Guinea-Bissau	Tombali	Quebo	17106	2015	90.0	80.1	100.6
Kenya	Central	Kiambu	51333	2000	63.3	60.0	66.8
Kenya	Central	Kiambu	51333	2005	54.3	50.9	57.8
Kenya	Central	Kiambu	51333	2010	46.2	42.4	50.0
Kenya	Central	Kiambu	51333	2015	36.4	32.7	40.8
Kenya	Central	Kirinyaga	51334	2000	54.9	51.8	58.2
Kenya	Central	Kirinyaga	51334	2005	46.3	43.4	49.5
Kenya	Central	Kirinyaga	51334	2010	40.2	37.0	43.8
Kenya	Central	Kirinyaga	51334	2015	32.0	28.4	35.6
Kenya	Central	Maragua	51335	2000	62.2	58.8	65.7
Kenya	Central	Maragua	51335	2005	52.5	49.2	56.2
Kenya	Central	Maragua	51335	2010	44.3	40.4	48.3
Kenya	Central	Maragua	51335	2015	35.1	31.2	39.2
Kenya	Central	Muranga	51336	2000	57.7	54.5	60.9
Kenya	Central	Muranga	51336	2005	48.8	45.8	52.2
Kenya	Central	Muranga	51336	2010	41.9	38.4	45.7
Kenya	Central	Muranga	51336	2015	33.1	29.6	36.8
Kenya	Central	Nyandarua	51337	2000	59.9	56.2	63.7
Kenya	Central	Nyandarua	51337	2005	50.8	47.2	54.3
Kenya	Central	Nyandarua	51337	2010	42.8	39.1	46.6
Kenya	Central	Nyandarua	51337	2015	34.5	30.6	38.7
Kenya	Central	Nyeri	51338	2000	52.3	49.0	55.7
Kenya	Central	Nyeri	51338	2005	44.7	41.6	47.9
Kenya	Central	Nyeri	51338	2010	39.0	35.7	42.6
Kenya	Central	Nyeri	51338	2015	30.5	27.3	34.1
Kenya	Central	Thika	51339	2000	63.4	60.4	66.5
Kenya	Central	Thika	51339	2005	54.5	51.2	58.2
Kenya	Central	Thika	51339	2010	46.4	42.4	50.2
Kenya	Central	Thika	51339	2015	36.9	33.0	41.2
Kenya	Coast	Kilifi	51340	2000	107.9	101.2	115.0
Kenya	Coast	Kilifi	51340	2005	80.7	74.7	86.9
Kenya	Coast	Kilifi	51340	2010	57.5	52.5	63.5
Kenya	Coast	Kilifi	51340	2015	54.8	48.9	61.9
Kenya	Coast	Kwale	51341	2000	114.9	108.6	121.5
Kenya	Coast	Kwale	51341	2005	91.0	85.0	97.4
Kenya	Coast	Kwale	51341	2010	60.7	55.8	65.8
Kenya	Coast	Kwale	51341	2015	52.3	46.5	58.5
Kenya	Coast	Lamu	51342	2000	100.9	90.6	111.8
Kenya	Coast	Lamu	51342	2005	78.6	70.4	88.2
Kenya	Coast	Lamu	51342	2010	59.0	51.6	66.9
Kenya	Coast	Lamu	51342	2015	51.8	44.8	60.0
Kenya	Coast	Malindi	51343	2000	107.8	99.9	116.9
Kenya	Coast	Malindi	51343	2005	78.6	72.1	86.2
Kenya	Coast	Malindi	51343	2010	55.9	50.1	62.7
Kenya	Coast	Malindi	51343	2015	55.2	48.4	63.0
Kenya	Coast	Mombasa	51344	2000	73.4	68.6	78.2
Kenya	Coast	Mombasa	51344	2005	67.4	62.5	72.5
Kenya	Coast	Mombasa	51344	2010	53.7	48.7	59.2
Kenya	Coast	Mombasa	51344	2015	45.0	39.6	51.1
Kenya	Coast	Taita Taveta	51345	2000	73.2	66.8	79.7
Kenya	Coast	Taita Taveta	51345	2005	62.6	57.2	68.3
Kenya	Coast	Taita Taveta	51345	2010	47.9	43.2	52.7
Kenya	Coast	Taita Taveta	51345	2015	40.2	35.3	45.3
Kenya	Coast	Tana River	51346	2000	106.7	100.0	114.3
Kenya	Coast	Tana River	51346	2005	80.7	75.3	86.9
Kenya	Coast	Tana River	51346	2010	57.9	53.1	63.1
Kenya	Coast	Tana River	51346	2015	50.6	45.2	56.3
Kenya	Eastern	Embu	51347	2000	57.5	54.0	61.1
Kenya	Eastern	Embu	51347	2005	46.7	43.5	49.9
Kenya	Eastern	Embu	51347	2010	39.6	36.1	43.2
Kenya	Eastern	Embu	51347	2015	32.1	28.5	35.9
Kenya	Eastern	Isiolo	51348	2000	66.9	62.4	71.4

Admin 0	Admin 1	Admin 2	GAUL Code	Year	Under-5 mortality (per 1,000 livebirths)		
					Estimate	Lower bound, 95% UI	Upper bound, 95% UI
Kenya	Eastern	Isiolo	51348	2005	52.2	48.5	56.0
Kenya	Eastern	Isiolo	51348	2010	41.0	37.3	44.5
Kenya	Eastern	Isiolo	51348	2015	32.6	29.1	36.6
Kenya	Eastern	Kitui	51349	2000	86.8	81.0	92.2
Kenya	Eastern	Kitui	51349	2005	69.2	64.2	74.6
Kenya	Eastern	Kitui	51349	2010	54.6	50.0	59.5
Kenya	Eastern	Kitui	51349	2015	46.7	41.4	52.3
Kenya	Eastern	Machakos	51350	2000	69.3	65.8	73.1
Kenya	Eastern	Machakos	51350	2005	57.8	54.2	61.7
Kenya	Eastern	Machakos	51350	2010	47.9	43.9	51.7
Kenya	Eastern	Machakos	51350	2015	39.6	35.6	43.9
Kenya	Eastern	Makueni	51351	2000	71.4	67.1	76.0
Kenya	Eastern	Makueni	51351	2005	60.1	56.0	64.6
Kenya	Eastern	Makueni	51351	2010	48.1	44.0	52.3
Kenya	Eastern	Makueni	51351	2015	40.6	36.3	45.5
Kenya	Eastern	Marsabit	51352	2000	59.0	53.4	64.6
Kenya	Eastern	Marsabit	51352	2005	48.2	43.7	52.9
Kenya	Eastern	Marsabit	51352	2010	36.9	33.2	41.1
Kenya	Eastern	Marsabit	51352	2015	29.6	25.7	33.5
Kenya	Eastern	Mbeere	51353	2000	64.8	60.9	68.9
Kenya	Eastern	Mbeere	51353	2005	52.7	49.4	56.4
Kenya	Eastern	Mbeere	51353	2010	44.3	40.8	48.3
Kenya	Eastern	Mbeere	51353	2015	36.6	32.8	41.0
Kenya	Eastern	Meru Central	51354	2000	56.2	52.8	59.4
Kenya	Eastern	Meru Central	51354	2005	45.8	42.9	49.1
Kenya	Eastern	Meru Central	51354	2010	37.8	34.5	41.4
Kenya	Eastern	Meru Central	51354	2015	30.5	27.3	34.3
Kenya	Eastern	Meru North	51355	2000	64.8	60.3	69.4
Kenya	Eastern	Meru North	51355	2005	51.1	47.3	54.8
Kenya	Eastern	Meru North	51355	2010	38.6	34.8	42.1
Kenya	Eastern	Meru North	51355	2015	32.0	28.3	36.1
Kenya	Eastern	Meru South	51356	2000	61.4	57.7	65.4
Kenya	Eastern	Meru South	51356	2005	48.8	45.6	52.2
Kenya	Eastern	Meru South	51356	2010	40.2	36.7	43.8
Kenya	Eastern	Meru South	51356	2015	32.8	29.3	37.0
Kenya	Eastern	Moyale	51357	2000	67.6	60.9	74.6
Kenya	Eastern	Moyale	51357	2005	57.5	51.6	63.9
Kenya	Eastern	Moyale	51357	2010	43.2	37.8	49.0
Kenya	Eastern	Moyale	51357	2015	36.2	30.6	42.0
Kenya	Eastern	Mwingi	51358	2000	85.6	80.3	91.7
Kenya	Eastern	Mwingi	51358	2005	65.9	61.6	70.8
Kenya	Eastern	Mwingi	51358	2010	52.1	48.0	56.8
Kenya	Eastern	Mwingi	51358	2015	44.4	39.5	50.0
Kenya	Eastern	Tharaka	51359	2000	71.7	67.1	76.5
Kenya	Eastern	Tharaka	51359	2005	55.3	51.6	59.3
Kenya	Eastern	Tharaka	51359	2010	43.0	39.2	46.9
Kenya	Eastern	Tharaka	51359	2015	36.2	32.2	41.0
Kenya	Ilemi triangle	Ilemi Triangle	61034	2000	95.6	82.6	110.7
Kenya	Ilemi triangle	Ilemi Triangle	61034	2005	84.2	72.1	97.8
Kenya	Ilemi triangle	Ilemi Triangle	61034	2010	67.0	56.7	78.5
Kenya	Ilemi triangle	Ilemi Triangle	61034	2015	55.3	45.9	65.2
Kenya	Nairobi	Nairobi	51360	2000	64.0	60.4	67.7
Kenya	Nairobi	Nairobi	51360	2005	55.6	52.0	59.6
Kenya	Nairobi	Nairobi	51360	2010	47.9	43.8	52.3
Kenya	Nairobi	Nairobi	51360	2015	39.0	34.6	44.0
Kenya	North Eastern	Garissa	51361	2000	91.3	84.7	99.2
Kenya	North Eastern	Garissa	51361	2005	74.8	68.3	82.0
Kenya	North Eastern	Garissa	51361	2010	56.7	51.4	62.6
Kenya	North Eastern	Garissa	51361	2015	43.4	37.9	49.2
Kenya	North Eastern	Ijara	51362	2000	108.7	98.1	121.4
Kenya	North Eastern	Ijara	51362	2005	86.4	77.4	96.8
Kenya	North Eastern	Ijara	51362	2010	64.6	57.3	73.2
Kenya	North Eastern	Ijara	51362	2015	52.6	45.8	61.3
Kenya	North Eastern	Mandera	51363	2000	81.8	75.4	88.8
Kenya	North Eastern	Mandera	51363	2005	71.7	65.8	78.1
Kenya	North Eastern	Mandera	51363	2010	52.9	47.2	58.5
Kenya	North Eastern	Mandera	51363	2015	45.6	39.8	51.7
Kenya	North Eastern	Wajir	51364	2000	83.0	77.6	89.3
Kenya	North Eastern	Wajir	51364	2005	71.1	66.2	76.5
Kenya	North Eastern	Wajir	51364	2010	52.5	47.7	57.8
Kenya	North Eastern	Wajir	51364	2015	42.3	37.6	47.3
Kenya	Nyanza	Bondo	51365	2000	175.9	168.1	183.4
Kenya	Nyanza	Bondo	51365	2005	146.7	138.7	154.5
Kenya	Nyanza	Bondo	51365	2010	108.4	101.4	115.4
Kenya	Nyanza	Bondo	51365	2015	72.2	65.4	79.0
Kenya	Nyanza	Central Kisii	51366	2000	102.7	98.8	106.9
Kenya	Nyanza	Central Kisii	51366	2005	83.8	79.7	88.0
Kenya	Nyanza	Central Kisii	51366	2010	60.7	57.1	64.4
Kenya	Nyanza	Central Kisii	51366	2015	51.6	47.3	56.5
Kenya	Nyanza	Gucha	51367	2000	103.3	98.9	108.3
Kenya	Nyanza	Gucha	51367	2005	85.4	81.1	90.4
Kenya	Nyanza	Gucha	51367	2010	62.7	58.9	67.2
Kenya	Nyanza	Gucha	51367	2015	53.8	49.0	58.9
Kenya	Nyanza	Homa Bay	51368	2000	178.5	170.9	186.8
Kenya	Nyanza	Homa Bay	51368	2005	144.2	137.2	151.4

Admin 0	Admin 1	Admin 2	GAUL Code	Year	Under-5 mortality (per 1,000 livebirths)		
					Estimate	Lower bound, 95% UI	Upper bound, 95% UI
Kenya	Nyanza	Homa Bay	51368	2010	102.3	96.5	108.5
Kenya	Nyanza	Homa Bay	51368	2015	79.0	71.8	86.5
Kenya	Nyanza	Kisumu	51369	2000	134.6	129.3	140.4
Kenya	Nyanza	Kisumu	51369	2005	115.4	109.8	121.4
Kenya	Nyanza	Kisumu	51369	2010	85.0	79.9	90.6
Kenya	Nyanza	Kisumu	51369	2015	62.0	56.3	68.0
Kenya	Nyanza	Kuria	51370	2000	138.3	131.2	146.0
Kenya	Nyanza	Kuria	51370	2005	115.2	108.0	122.4
Kenya	Nyanza	Kuria	51370	2010	78.6	72.9	84.0
Kenya	Nyanza	Kuria	51370	2015	66.2	59.7	72.7
Kenya	Nyanza	Migori	51371	2000	175.0	167.5	183.1
Kenya	Nyanza	Migori	51371	2005	142.0	134.5	149.8
Kenya	Nyanza	Migori	51371	2010	99.8	93.6	106.2
Kenya	Nyanza	Migori	51371	2015	79.4	71.9	87.2
Kenya	Nyanza	Nyamira	51372	2000	89.1	85.5	93.0
Kenya	Nyanza	Nyamira	51372	2005	72.0	68.2	75.8
Kenya	Nyanza	Nyamira	51372	2010	53.3	49.9	56.7
Kenya	Nyanza	Nyamira	51372	2015	46.4	42.5	50.7
Kenya	Nyanza	Nyando	51373	2000	111.6	106.6	116.7
Kenya	Nyanza	Nyando	51373	2005	90.9	85.9	96.1
Kenya	Nyanza	Nyando	51373	2010	67.4	63.2	72.1
Kenya	Nyanza	Nyando	51373	2015	54.4	49.8	59.6
Kenya	Nyanza	Rachuonyo	51374	2000	132.3	126.9	137.8
Kenya	Nyanza	Rachuonyo	51374	2005	107.5	102.1	112.9
Kenya	Nyanza	Rachuonyo	51374	2010	76.8	72.5	81.1
Kenya	Nyanza	Rachuonyo	51374	2015	61.1	56.0	66.8
Kenya	Nyanza	Siaya	51375	2000	160.7	154.5	166.8
Kenya	Nyanza	Siaya	51375	2005	134.9	129.1	141.3
Kenya	Nyanza	Siaya	51375	2010	99.1	92.9	105.2
Kenya	Nyanza	Siaya	51375	2015	68.0	61.8	74.1
Kenya	Nyanza	Suba	51376	2000	202.6	191.3	214.8
Kenya	Nyanza	Suba	51376	2005	168.2	157.4	178.8
Kenya	Nyanza	Suba	51376	2010	121.4	112.8	130.2
Kenya	Nyanza	Suba	51376	2015	84.1	75.3	93.2
Kenya	Rift Valley	Baringo	51377	2000	65.2	60.7	70.2
Kenya	Rift Valley	Baringo	51377	2005	52.6	48.6	56.6
Kenya	Rift Valley	Baringo	51377	2010	42.3	38.5	46.1
Kenya	Rift Valley	Baringo	51377	2015	37.1	33.2	41.1
Kenya	Rift Valley	Bomet	51378	2000	67.9	63.8	72.4
Kenya	Rift Valley	Bomet	51378	2005	55.4	51.7	59.4
Kenya	Rift Valley	Bomet	51378	2010	43.8	40.0	47.4
Kenya	Rift Valley	Bomet	51378	2015	38.7	34.8	43.1
Kenya	Rift Valley	Buret	51379	2000	75.3	71.3	79.6
Kenya	Rift Valley	Buret	51379	2005	60.2	56.3	64.2
Kenya	Rift Valley	Buret	51379	2010	46.4	42.9	50.0
Kenya	Rift Valley	Buret	51379	2015	41.3	37.4	45.5
Kenya	Rift Valley	Kajiado	51380	2000	67.0	63.3	70.8
Kenya	Rift Valley	Kajiado	51380	2005	55.4	51.8	59.1
Kenya	Rift Valley	Kajiado	51380	2010	44.4	41.0	47.9
Kenya	Rift Valley	Kajiado	51380	2015	36.3	32.8	40.2
Kenya	Rift Valley	Keiyo	51381	2000	63.0	59.0	67.3
Kenya	Rift Valley	Keiyo	51381	2005	53.1	49.2	57.3
Kenya	Rift Valley	Keiyo	51381	2010	43.5	40.1	47.5
Kenya	Rift Valley	Keiyo	51381	2015	37.6	33.9	41.7
Kenya	Rift Valley	Kericho	51382	2000	81.8	77.5	86.1
Kenya	Rift Valley	Kericho	51382	2005	66.3	62.4	70.4
Kenya	Rift Valley	Kericho	51382	2010	51.1	47.7	54.7
Kenya	Rift Valley	Kericho	51382	2015	44.1	40.1	48.2
Kenya	Rift Valley	Koibatek	51383	2000	67.0	62.7	71.6
Kenya	Rift Valley	Koibatek	51383	2005	55.6	51.6	59.7
Kenya	Rift Valley	Koibatek	51383	2010	45.7	42.1	49.6
Kenya	Rift Valley	Koibatek	51383	2015	38.6	34.7	42.6
Kenya	Rift Valley	Laikipia	51384	2000	56.4	52.6	60.1
Kenya	Rift Valley	Laikipia	51384	2005	46.1	42.7	49.8
Kenya	Rift Valley	Laikipia	51384	2010	38.5	35.4	42.0
Kenya	Rift Valley	Laikipia	51384	2015	32.2	28.6	35.8
Kenya	Rift Valley	Marakwet	51385	2000	64.9	60.0	70.0
Kenya	Rift Valley	Marakwet	51385	2005	53.2	48.9	57.8
Kenya	Rift Valley	Marakwet	51385	2010	42.2	38.5	46.5
Kenya	Rift Valley	Marakwet	51385	2015	37.6	33.5	41.9
Kenya	Rift Valley	Nakuru	51386	2000	63.4	59.6	67.0
Kenya	Rift Valley	Nakuru	51386	2005	53.8	50.3	57.4
Kenya	Rift Valley	Nakuru	51386	2010	44.6	41.2	48.5
Kenya	Rift Valley	Nakuru	51386	2015	37.1	33.6	41.4
Kenya	Rift Valley	Nandi North	68769	2000	86.7	82.4	91.3
Kenya	Rift Valley	Nandi North	68769	2005	68.0	64.2	72.3
Kenya	Rift Valley	Nandi North	68769	2010	51.8	48.2	55.7
Kenya	Rift Valley	Nandi North	68769	2015	43.9	39.5	48.0
Kenya	Rift Valley	Nandi South	68770	2000	97.1	93.0	101.9
Kenya	Rift Valley	Nandi South	68770	2005	77.4	73.4	81.8
Kenya	Rift Valley	Nandi South	68770	2010	57.5	53.9	61.3
Kenya	Rift Valley	Nandi South	68770	2015	47.6	43.3	51.9
Kenya	Rift Valley	Narok	51388	2000	70.3	65.7	75.0
Kenya	Rift Valley	Narok	51388	2005	56.7	52.8	60.7
Kenya	Rift Valley	Narok	51388	2010	42.8	39.3	46.3

Admin 0	Admin 1	Admin 2	GAUL Code	Year	Under-5 mortality (per 1,000 livebirths)		
					Estimate	Lower bound, 95% UI	Upper bound, 95% UI
Kenya	Rift Valley	Narok	51388	2015	37.5	33.9	41.9
Kenya	Rift Valley	Samburu	51389	2000	67.4	61.4	73.8
Kenya	Rift Valley	Samburu	51389	2005	50.9	46.2	56.1
Kenya	Rift Valley	Samburu	51389	2010	38.0	34.2	42.3
Kenya	Rift Valley	Samburu	51389	2015	33.6	29.5	38.1
Kenya	Rift Valley	Trans Mara	51390	2000	87.7	83.0	92.7
Kenya	Rift Valley	Trans Mara	51390	2005	73.7	69.3	78.4
Kenya	Rift Valley	Trans Mara	51390	2010	53.5	49.9	57.6
Kenya	Rift Valley	Trans Mara	51390	2015	47.4	43.2	51.9
Kenya	Rift Valley	Trans Nzoia	51391	2000	77.3	72.8	81.7
Kenya	Rift Valley	Trans Nzoia	51391	2005	59.5	55.8	63.3
Kenya	Rift Valley	Trans Nzoia	51391	2010	46.5	43.2	50.2
Kenya	Rift Valley	Trans Nzoia	51391	2015	40.1	36.4	44.3
Kenya	Rift Valley	Turkana	51392	2000	86.3	79.8	92.9
Kenya	Rift Valley	Turkana	51392	2005	73.8	68.0	80.1
Kenya	Rift Valley	Turkana	51392	2010	57.5	52.4	63.2
Kenya	Rift Valley	Turkana	51392	2015	46.8	41.6	52.6
Kenya	Rift Valley	Uasin Gishu	51393	2000	66.1	62.6	69.7
Kenya	Rift Valley	Uasin Gishu	51393	2005	55.1	51.8	58.6
Kenya	Rift Valley	Uasin Gishu	51393	2010	45.2	42.1	48.8
Kenya	Rift Valley	Uasin Gishu	51393	2015	38.5	34.7	42.3
Kenya	Rift Valley	West Pokot	51394	2000	90.7	84.2	97.0
Kenya	Rift Valley	West Pokot	51394	2005	70.0	65.1	75.6
Kenya	Rift Valley	West Pokot	51394	2010	48.7	44.5	52.9
Kenya	Rift Valley	West Pokot	51394	2015	46.1	41.5	50.9
Kenya	Western	Bungoma	51395	2000	102.7	98.4	107.1
Kenya	Western	Bungoma	51395	2005	81.8	77.5	86.2
Kenya	Western	Bungoma	51395	2010	62.4	58.4	66.5
Kenya	Western	Bungoma	51395	2015	48.7	44.3	53.3
Kenya	Western	Busia	51396	2000	152.7	146.3	159.3
Kenya	Western	Busia	51396	2005	124.1	118.0	130.3
Kenya	Western	Busia	51396	2010	89.9	84.6	95.2
Kenya	Western	Busia	51396	2015	63.6	57.8	69.2
Kenya	Western	Butere Mumias	51397	2000	133.7	128.4	139.5
Kenya	Western	Butere Mumias	51397	2005	109.1	104.0	114.6
Kenya	Western	Butere Mumias	51397	2010	81.6	76.7	86.8
Kenya	Western	Butere Mumias	51397	2015	59.5	54.3	65.1
Kenya	Western	Kakamega	51398	2000	108.6	104.3	113.1
Kenya	Western	Kakamega	51398	2005	86.4	82.0	91.0
Kenya	Western	Kakamega	51398	2010	66.8	62.9	71.0
Kenya	Western	Kakamega	51398	2015	51.4	46.6	56.2
Kenya	Western	Lugari	51399	2000	79.1	75.1	82.9
Kenya	Western	Lugari	51399	2005	61.5	57.9	65.2
Kenya	Western	Lugari	51399	2010	48.9	45.5	52.7
Kenya	Western	Lugari	51399	2015	41.0	37.1	45.3
Kenya	Western	Mt Elgon	51400	2000	98.8	93.9	103.8
Kenya	Western	Mt Elgon	51400	2005	76.8	72.5	81.4
Kenya	Western	Mt Elgon	51400	2010	58.5	54.5	62.7
Kenya	Western	Mt Elgon	51400	2015	46.2	41.7	50.8
Kenya	Western	Teso	51401	2000	134.1	128.4	139.7
Kenya	Western	Teso	51401	2005	107.5	102.3	112.6
Kenya	Western	Teso	51401	2010	77.8	73.3	82.4
Kenya	Western	Teso	51401	2015	57.8	52.8	63.3
Kenya	Western	Vihiga	51402	2000	126.9	122.5	131.9
Kenya	Western	Vihiga	51402	2005	107.0	102.2	111.9
Kenya	Western	Vihiga	51402	2010	79.1	74.7	84.1
Kenya	Western	Vihiga	51402	2015	58.4	53.5	63.8
Lesotho	Berea	'Makhoroana	18817	2000	96.8	88.3	106.0
Lesotho	Berea	'Makhoroana	18817	2005	107.9	98.6	118.3
Lesotho	Berea	'Makhoroana	18817	2010	87.5	79.2	97.1
Lesotho	Berea	'Makhoroana	18817	2015	69.9	61.5	78.9
Lesotho	Berea	'Mamathe	18818	2000	92.4	85.3	100.5
Lesotho	Berea	'Mamathe	18818	2005	103.8	95.2	112.6
Lesotho	Berea	'Mamathe	18818	2010	85.4	77.9	94.1
Lesotho	Berea	'Mamathe	18818	2015	68.3	61.1	77.0
Lesotho	Berea	Bela-Bela	18819	2000	93.4	85.1	102.3
Lesotho	Berea	Bela-Bela	18819	2005	105.0	96.1	114.7
Lesotho	Berea	Bela-Bela	18819	2010	85.5	77.2	94.9
Lesotho	Berea	Bela-Bela	18819	2015	68.7	60.8	78.1
Lesotho	Berea	CPA	18820	2000	108.5	99.2	119.0
Lesotho	Berea	CPA	18820	2005	116.5	106.4	127.3
Lesotho	Berea	CPA	18820	2010	93.9	85.0	104.2
Lesotho	Berea	CPA	18820	2015	73.6	65.1	82.3
Lesotho	Berea	Foso	18821	2000	87.4	79.2	96.3
Lesotho	Berea	Foso	18821	2005	97.4	88.5	107.4
Lesotho	Berea	Foso	18821	2010	82.7	74.7	90.9
Lesotho	Berea	Foso	18821	2015	65.7	58.0	75.0
Lesotho	Berea	Khokhoba (I)	18822	2000	102.5	92.9	112.6
Lesotho	Berea	Khokhoba (I)	18822	2005	113.6	103.3	125.4
Lesotho	Berea	Khokhoba (I)	18822	2010	90.8	81.9	100.4
Lesotho	Berea	Khokhoba (I)	18822	2015	71.3	62.8	80.2
Lesotho	Berea	Khokhoba (II	18823	2000	107.8	97.8	118.7
Lesotho	Berea	Khokhoba (II	18823	2005	116.6	105.7	128.6
Lesotho	Berea	Khokhoba (II	18823	2010	93.9	84.7	104.8
Lesotho	Berea	Khokhoba (II	18823	2015	73.4	64.6	82.4

Admin 0	Admin 1	Admin 2	GAUL Code	Year	Under-5 mortality (per 1,000 livebirths)		
					Estimate	Lower bound, 95% UI	Upper bound, 95% UI
Lesotho	Berea	Koali	18824	2000	91.8	84.7	99.8
Lesotho	Berea	Koali	18824	2005	103.7	95.5	113.1
Lesotho	Berea	Koali	18824	2010	85.5	78.2	94.2
Lesotho	Berea	Koali	18824	2015	68.5	61.1	77.3
Lesotho	Berea	Kolojane	18825	2000	94.3	86.1	103.4
Lesotho	Berea	Kolojane	18825	2005	105.5	96.3	114.9
Lesotho	Berea	Kolojane	18825	2010	85.7	77.3	95.0
Lesotho	Berea	Kolojane	18825	2015	68.5	60.5	78.0
Lesotho	Berea	Lekokoaneng	18826	2000	89.2	81.9	97.8
Lesotho	Berea	Lekokoaneng	18826	2005	100.5	91.8	109.7
Lesotho	Berea	Lekokoaneng	18826	2010	84.2	76.5	92.8
Lesotho	Berea	Lekokoaneng	18826	2015	66.7	59.3	75.4
Lesotho	Berea	Majoe-Mats'o	18827	2000	101.5	92.3	111.2
Lesotho	Berea	Majoe-Mats'o	18827	2005	111.5	102.0	121.9
Lesotho	Berea	Majoe-Mats'o	18827	2010	89.8	81.3	99.3
Lesotho	Berea	Majoe-Mats'o	18827	2015	71.2	63.3	80.3
Lesotho	Berea	Mapoteng	18828	2000	92.8	85.0	101.0
Lesotho	Berea	Mapoteng	18828	2005	104.1	95.5	113.5
Lesotho	Berea	Mapoteng	18828	2010	85.6	77.5	95.0
Lesotho	Berea	Mapoteng	18828	2015	68.8	61.1	77.6
Lesotho	Berea	Maqhaka	18829	2000	90.6	82.7	99.1
Lesotho	Berea	Maqhaka	18829	2005	102.5	93.9	112.2
Lesotho	Berea	Maqhaka	18829	2010	85.5	77.9	94.1
Lesotho	Berea	Maqhaka	18829	2015	67.7	60.4	76.8
Lesotho	Berea	Mika	18830	2000	97.4	89.4	106.4
Lesotho	Berea	Mika	18830	2005	109.5	99.9	119.8
Lesotho	Berea	Mika	18830	2010	87.9	79.7	97.4
Lesotho	Berea	Mika	18830	2015	69.9	62.1	78.2
Lesotho	Berea	Mohatlane	18831	2000	93.1	85.8	101.2
Lesotho	Berea	Mohatlane	18831	2005	105.0	96.6	114.5
Lesotho	Berea	Mohatlane	18831	2010	86.1	78.6	94.7
Lesotho	Berea	Mohatlane	18831	2015	69.0	61.5	77.3
Lesotho	Berea	Mokhachane	18832	2000	93.8	85.5	102.5
Lesotho	Berea	Mokhachane	18832	2005	105.5	96.5	114.9
Lesotho	Berea	Mokhachane	18832	2010	85.9	77.8	95.1
Lesotho	Berea	Mokhachane	18832	2015	69.1	61.4	78.1
Lesotho	Berea	Mokhameleli	18833	2000	102.0	92.9	111.9
Lesotho	Berea	Mokhameleli	18833	2005	113.0	102.6	124.4
Lesotho	Berea	Mokhameleli	18833	2010	90.5	81.8	100.1
Lesotho	Berea	Mokhameleli	18833	2015	71.2	62.8	80.3
Lesotho	Berea	Mokomahatsi	18834	2000	91.9	83.9	100.4
Lesotho	Berea	Mokomahatsi	18834	2005	102.2	93.2	111.6
Lesotho	Berea	Mokomahatsi	18834	2010	84.5	76.5	93.5
Lesotho	Berea	Mokomahatsi	18834	2015	67.5	60.0	76.9
Lesotho	Berea	Pulane	18835	2000	98.7	90.1	107.6
Lesotho	Berea	Pulane	18835	2005	110.8	101.4	121.5
Lesotho	Berea	Pulane	18835	2010	88.7	80.4	97.7
Lesotho	Berea	Pulane	18835	2015	70.4	62.5	78.8
Lesotho	Berea	Sebetia (I)	18836	2000	101.2	92.3	110.9
Lesotho	Berea	Sebetia (I)	18836	2005	112.6	103.0	123.5
Lesotho	Berea	Sebetia (I)	18836	2010	90.0	81.3	100.2
Lesotho	Berea	Sebetia (I)	18836	2015	71.2	63.0	80.1
Lesotho	Berea	Sebetia (II)	18837	2000	90.6	82.6	99.4
Lesotho	Berea	Sebetia (II)	18837	2005	101.7	92.8	111.7
Lesotho	Berea	Sebetia (II)	18837	2010	84.7	76.7	93.4
Lesotho	Berea	Sebetia (II)	18837	2015	68.3	60.7	77.4
Lesotho	Berea	Thuathe	18838	2000	109.2	100.3	119.6
Lesotho	Berea	Thuathe	18838	2005	116.4	106.2	126.7
Lesotho	Berea	Thuathe	18838	2010	93.6	85.1	104.1
Lesotho	Berea	Thuathe	18838	2015	76.2	67.6	85.9
Lesotho	Berea	Thupa-Kubu	18839	2000	93.6	86.1	101.4
Lesotho	Berea	Thupa-Kubu	18839	2005	106.2	97.9	116.5
Lesotho	Berea	Thupa-Kubu	18839	2010	86.5	78.9	94.8
Lesotho	Berea	Thupa-Kubu	18839	2015	69.3	61.9	78.0
Lesotho	Berea	Urban	18840	2000	87.9	80.9	96.0
Lesotho	Berea	Urban	18840	2005	96.9	88.9	105.6
Lesotho	Berea	Urban	18840	2010	82.1	74.8	90.9
Lesotho	Berea	Urban	18840	2015	65.8	58.8	74.3
Lesotho	Butha Buthe	Boiketsiso	18841	2000	89.7	80.0	100.1
Lesotho	Butha Buthe	Boiketsiso	18841	2005	95.5	85.0	107.6
Lesotho	Butha Buthe	Boiketsiso	18841	2010	78.2	69.2	88.8
Lesotho	Butha Buthe	Boiketsiso	18841	2015	61.7	53.0	72.0
Lesotho	Butha Buthe	CPA	18842	2000	100.3	90.3	110.5
Lesotho	Butha Buthe	CPA	18842	2005	105.2	95.7	116.6
Lesotho	Butha Buthe	CPA	18842	2010	85.9	77.0	95.6
Lesotho	Butha Buthe	CPA	18842	2015	69.7	60.7	79.6
Lesotho	Butha Buthe	Chaba	18843	2000	89.4	80.3	98.8
Lesotho	Butha Buthe	Chaba	18843	2005	97.0	87.5	107.1
Lesotho	Butha Buthe	Chaba	18843	2010	79.0	71.0	88.6
Lesotho	Butha Buthe	Chaba	18843	2015	63.8	55.4	73.5
Lesotho	Butha Buthe	Khukhune	18844	2000	92.3	83.2	101.6
Lesotho	Butha Buthe	Khukhune	18844	2005	99.5	90.5	109.5
Lesotho	Butha Buthe	Khukhune	18844	2010	81.5	73.5	90.8
Lesotho	Butha Buthe	Khukhune	18844	2015	65.2	56.6	74.4
Lesotho	Butha Buthe	Lephats'oane	18845	2000	103.5	93.9	114.2

Admin 0	Admin 1	Admin 2	GAUL Code	Year	Under-5 mortality (per 1,000 livebirths)		
					Estimate	Lower bound, 95% UI	Upper bound, 95% UI
Lesotho	Butha Buthe	Lephats'oane	18845	2005	108.5	98.7	119.0
Lesotho	Butha Buthe	Lephats'oane	18845	2010	87.5	78.4	97.4
Lesotho	Butha Buthe	Lephats'oane	18845	2015	73.2	64.2	84.1
Lesotho	Butha Buthe	Likhutlong	18846	2000	91.5	82.9	100.1
Lesotho	Butha Buthe	Likhutlong	18846	2005	100.2	90.9	109.2
Lesotho	Butha Buthe	Likhutlong	18846	2010	81.5	73.5	90.1
Lesotho	Butha Buthe	Likhutlong	18846	2015	65.4	57.4	74.3
Lesotho	Butha Buthe	Linakeng	18847	2000	89.7	81.4	98.2
Lesotho	Butha Buthe	Linakeng	18847	2005	98.6	89.7	107.9
Lesotho	Butha Buthe	Linakeng	18847	2010	80.2	72.5	88.5
Lesotho	Butha Buthe	Linakeng	18847	2015	65.6	57.5	74.9
Lesotho	Butha Buthe	Liphakoeng	18848	2000	103.3	94.1	113.3
Lesotho	Butha Buthe	Liphakoeng	18848	2005	108.1	98.5	118.4
Lesotho	Butha Buthe	Liphakoeng	18848	2010	88.6	80.1	98.6
Lesotho	Butha Buthe	Liphakoeng	18848	2015	72.3	64.1	82.5
Lesotho	Butha Buthe	Liqobong	18849	2000	92.1	82.0	103.1
Lesotho	Butha Buthe	Liqobong	18849	2005	96.3	85.6	108.6
Lesotho	Butha Buthe	Liqobong	18849	2010	78.4	68.9	89.1
Lesotho	Butha Buthe	Liqobong	18849	2015	63.1	54.2	73.6
Lesotho	Butha Buthe	Maama	18850	2000	91.4	81.9	101.7
Lesotho	Butha Buthe	Maama	18850	2005	97.7	87.7	108.9
Lesotho	Butha Buthe	Maama	18850	2010	80.0	71.4	90.3
Lesotho	Butha Buthe	Maama	18850	2015	63.5	54.8	74.1
Lesotho	Butha Buthe	Makhunoane	18851	2000	86.3	77.3	95.8
Lesotho	Butha Buthe	Makhunoane	18851	2005	93.8	84.2	104.1
Lesotho	Butha Buthe	Makhunoane	18851	2010	77.0	68.5	86.8
Lesotho	Butha Buthe	Makhunoane	18851	2015	62.1	53.6	72.1
Lesotho	Butha Buthe	Mopeli	18852	2000	85.2	76.8	93.5
Lesotho	Butha Buthe	Mopeli	18852	2005	91.9	83.1	100.7
Lesotho	Butha Buthe	Mopeli	18852	2010	77.2	69.2	85.5
Lesotho	Butha Buthe	Mopeli	18852	2015	64.1	55.9	73.0
Lesotho	Butha Buthe	Qalo	18853	2000	86.4	78.0	94.6
Lesotho	Butha Buthe	Qalo	18853	2005	94.1	85.1	103.0
Lesotho	Butha Buthe	Qalo	18853	2010	78.9	71.1	87.5
Lesotho	Butha Buthe	Qalo	18853	2015	64.2	56.1	73.0
Lesotho	Butha Buthe	Qholaqhoe	18854	2000	86.0	77.3	94.9
Lesotho	Butha Buthe	Qholaqhoe	18854	2005	93.2	84.1	102.6
Lesotho	Butha Buthe	Qholaqhoe	18854	2010	77.0	68.8	85.8
Lesotho	Butha Buthe	Qholaqhoe	18854	2015	62.4	54.2	71.7
Lesotho	Butha Buthe	Sekubu	18855	2000	89.3	81.1	97.8
Lesotho	Butha Buthe	Sekubu	18855	2005	97.7	88.6	106.5
Lesotho	Butha Buthe	Sekubu	18855	2010	80.2	72.5	88.7
Lesotho	Butha Buthe	Sekubu	18855	2015	65.6	57.4	74.7
Lesotho	Butha Buthe	Tsime	18856	2000	90.9	82.4	99.5
Lesotho	Butha Buthe	Tsime	18856	2005	99.3	90.4	108.3
Lesotho	Butha Buthe	Tsime	18856	2010	80.8	73.1	89.3
Lesotho	Butha Buthe	Tsime	18856	2015	65.2	57.2	74.3
Lesotho	Butha Buthe	Tumane	18857	2000	86.4	77.7	95.5
Lesotho	Butha Buthe	Tumane	18857	2005	93.8	84.6	103.2
Lesotho	Butha Buthe	Tumane	18857	2010	77.2	69.2	86.4
Lesotho	Butha Buthe	Tumane	18857	2015	62.7	54.4	72.1
Lesotho	Butha Buthe	Urban	18858	2000	85.4	76.9	93.7
Lesotho	Butha Buthe	Urban	18858	2005	91.9	83.1	100.7
Lesotho	Butha Buthe	Urban	18858	2010	77.2	69.2	85.4
Lesotho	Butha Buthe	Urban	18858	2015	64.1	56.0	73.0
Lesotho	Leribe	CPA	18859	2000	103.4	94.6	112.9
Lesotho	Leribe	CPA	18859	2005	110.7	101.6	120.3
Lesotho	Leribe	CPA	18859	2010	89.5	81.4	98.6
Lesotho	Leribe	CPA	18859	2015	72.1	64.2	82.3
Lesotho	Leribe	Fobane	18860	2000	92.2	84.9	100.3
Lesotho	Leribe	Fobane	18860	2005	103.6	94.8	112.7
Lesotho	Leribe	Fobane	18860	2010	85.3	77.5	94.6
Lesotho	Leribe	Fobane	18860	2015	68.1	60.6	77.3
Lesotho	Leribe	Ha Mots'eare	18861	2000	90.0	81.5	98.5
Lesotho	Leribe	Ha Mots'eare	18861	2005	99.6	90.6	108.7
Lesotho	Leribe	Ha Mots'eare	18861	2010	81.6	73.6	90.1
Lesotho	Leribe	Ha Mots'eare	18861	2015	66.8	58.9	75.7
Lesotho	Leribe	Hleoheng	18862	2000	89.6	81.8	98.4
Lesotho	Leribe	Hleoheng	18862	2005	99.0	90.0	108.7
Lesotho	Leribe	Hleoheng	18862	2010	82.7	74.5	92.2
Lesotho	Leribe	Hleoheng	18862	2015	66.4	58.8	75.9
Lesotho	Leribe	Kolonyama	18863	2000	88.8	81.2	97.7
Lesotho	Leribe	Kolonyama	18863	2005	98.3	89.3	107.5
Lesotho	Leribe	Kolonyama	18863	2010	83.0	75.4	91.9
Lesotho	Leribe	Kolonyama	18863	2015	65.7	58.0	74.7
Lesotho	Leribe	Kosetabole	18864	2000	105.0	96.2	114.5
Lesotho	Leribe	Kosetabole	18864	2005	112.9	103.9	122.5
Lesotho	Leribe	Kosetabole	18864	2010	91.7	83.6	101.3
Lesotho	Leribe	Kosetabole	18864	2015	76.5	67.8	86.7
Lesotho	Leribe	Lejone	18865	2000	103.7	94.7	113.6
Lesotho	Leribe	Lejone	18865	2005	110.2	101.2	120.2
Lesotho	Leribe	Lejone	18865	2010	89.8	81.5	99.4
Lesotho	Leribe	Lejone	18865	2015	72.5	64.4	82.5
Lesotho	Leribe	Leribe	18866	2000	89.6	81.6	98.3
Lesotho	Leribe	Leribe	18866	2005	98.3	89.4	107.4

Admin 0	Admin 1	Admin 2	GAUL Code	Year	Under-5 mortality (per 1,000 livebirths) Estimate	Lower bound, 95% UI	Upper bound, 95% UI
Lesotho	Leribe	Leribe	18866	2010	81.7	73.9	90.4
Lesotho	Leribe	Leribe	18866	2015	65.8	57.7	74.7
Lesotho	Leribe	Likhakeng	18867	2000	91.5	83.5	100.4
Lesotho	Leribe	Likhakeng	18867	2005	101.1	91.6	110.6
Lesotho	Leribe	Likhakeng	18867	2010	83.5	75.4	92.3
Lesotho	Leribe	Likhakeng	18867	2015	67.3	59.4	76.2
Lesotho	Leribe	Lipetu	18868	2000	97.6	88.9	107.2
Lesotho	Leribe	Lipetu	18868	2005	108.6	98.9	119.0
Lesotho	Leribe	Lipetu	18868	2010	87.4	78.9	96.8
Lesotho	Leribe	Lipetu	18868	2015	69.4	61.5	78.1
Lesotho	Leribe	Mahobong	18869	2000	91.8	83.4	101.2
Lesotho	Leribe	Mahobong	18869	2005	102.5	92.8	112.5
Lesotho	Leribe	Mahobong	18869	2010	83.6	75.2	92.7
Lesotho	Leribe	Mahobong	18869	2015	68.4	60.0	77.5
Lesotho	Leribe	Matlakeng	18870	2000	85.3	77.1	93.9
Lesotho	Leribe	Matlakeng	18870	2005	92.8	83.8	102.0
Lesotho	Leribe	Matlakeng	18870	2010	77.9	69.9	86.7
Lesotho	Leribe	Matlakeng	18870	2015	64.1	56.2	72.8
Lesotho	Leribe	Matlameng	18871	2000	95.8	87.3	105.1
Lesotho	Leribe	Matlameng	18871	2005	105.0	95.8	114.7
Lesotho	Leribe	Matlameng	18871	2010	85.2	77.0	94.5
Lesotho	Leribe	Matlameng	18871	2015	68.0	60.4	76.9
Lesotho	Leribe	Mohobollo	18872	2000	89.4	81.3	98.2
Lesotho	Leribe	Mohobollo	18872	2005	99.0	90.1	108.0
Lesotho	Leribe	Mohobollo	18872	2010	81.5	73.6	90.0
Lesotho	Leribe	Mohobollo	18872	2015	66.2	58.4	75.0
Lesotho	Leribe	Mphorosane	18873	2000	104.0	94.6	114.0
Lesotho	Leribe	Mphorosane	18873	2005	110.2	100.8	120.2
Lesotho	Leribe	Mphorosane	18873	2010	89.5	80.6	99.6
Lesotho	Leribe	Mphorosane	18873	2015	75.5	66.7	86.5
Lesotho	Leribe	Nqechane	18874	2000	85.8	77.5	94.0
Lesotho	Leribe	Nqechane	18874	2005	94.6	85.5	103.7
Lesotho	Leribe	Nqechane	18874	2010	80.0	71.9	89.4
Lesotho	Leribe	Nqechane	18874	2015	64.6	56.8	73.4
Lesotho	Leribe	Peka	18875	2000	89.3	81.6	97.9
Lesotho	Leribe	Peka	18875	2005	99.4	90.0	108.5
Lesotho	Leribe	Peka	18875	2010	83.1	75.2	92.3
Lesotho	Leribe	Peka	18875	2015	66.6	59.1	75.9
Lesotho	Leribe	Pela Ts'oeu	18876	2000	94.4	85.9	102.9
Lesotho	Leribe	Pela Ts'oeu	18876	2005	102.8	93.7	112.0
Lesotho	Leribe	Pela Ts'oeu	18876	2010	83.5	75.8	92.2
Lesotho	Leribe	Pela Ts'oeu	18876	2015	66.9	59.1	75.7
Lesotho	Leribe	Pitseng	18877	2000	97.7	89.1	107.3
Lesotho	Leribe	Pitseng	18877	2005	107.5	98.0	117.4
Lesotho	Leribe	Pitseng	18877	2010	87.0	78.8	96.5
Lesotho	Leribe	Pitseng	18877	2015	68.8	61.1	77.8
Lesotho	Leribe	Seshote	18878	2000	104.1	94.8	113.9
Lesotho	Leribe	Seshote	18878	2005	110.6	100.9	121.1
Lesotho	Leribe	Seshote	18878	2010	89.4	80.8	98.9
Lesotho	Leribe	Seshote	18878	2015	76.2	67.1	87.7
Lesotho	Leribe	Thaba Phats'oa	18879	2000	96.5	87.8	106.3
Lesotho	Leribe	Thaba Phats'oa	18879	2005	107.0	97.2	117.3
Lesotho	Leribe	Thaba Phats'oa	18879	2010	86.5	78.0	95.8
Lesotho	Leribe	Thaba Phats'oa	18879	2015	69.1	61.2	78.2
Lesotho	Leribe	Tsikoane	18880	2000	89.7	81.7	98.4
Lesotho	Leribe	Tsikoane	18880	2005	99.2	90.1	108.5
Lesotho	Leribe	Tsikoane	18880	2010	82.7	74.5	91.9
Lesotho	Leribe	Tsikoane	18880	2015	66.6	58.9	75.8
Lesotho	Leribe	Urban	18881	2000	86.9	79.0	95.9
Lesotho	Leribe	Urban	18881	2005	94.8	86.0	103.9
Lesotho	Leribe	Urban	18881	2010	79.7	71.8	89.2
Lesotho	Leribe	Urban	18881	2015	64.7	56.9	73.9
Lesotho	Mafeteng	BOLEKA	18882	2000	92.9	84.1	101.9
Lesotho	Mafeteng	BOLEKA	18882	2005	106.6	96.8	118.3
Lesotho	Mafeteng	BOLEKA	18882	2010	87.3	78.8	96.6
Lesotho	Mafeteng	BOLEKA	18882	2015	69.1	60.9	78.2
Lesotho	Mafeteng	KOKOME	18883	2000	98.6	89.4	107.9
Lesotho	Mafeteng	KOKOME	18883	2005	113.2	103.3	124.2
Lesotho	Mafeteng	KOKOME	18883	2010	90.8	82.5	100.5
Lesotho	Mafeteng	KOKOME	18883	2015	72.9	64.3	81.9
Lesotho	Mafeteng	KOLO	18884	2000	90.1	81.1	98.8
Lesotho	Mafeteng	KOLO	18884	2005	102.6	92.9	113.1
Lesotho	Mafeteng	KOLO	18884	2010	85.4	76.8	94.9
Lesotho	Mafeteng	KOLO	18884	2015	67.3	59.0	76.3
Lesotho	Mafeteng	LEKHATJE	18885	2000	108.7	98.8	118.9
Lesotho	Mafeteng	LEKHATJE	18885	2005	120.9	110.6	132.6
Lesotho	Mafeteng	LEKHATJE	18885	2010	95.9	86.9	105.8
Lesotho	Mafeteng	LEKHATJE	18885	2015	76.4	67.8	86.2
Lesotho	Mafeteng	LIKHOELE	18886	2000	91.5	82.3	100.3
Lesotho	Mafeteng	LIKHOELE	18886	2005	101.3	92.1	111.6
Lesotho	Mafeteng	LIKHOELE	18886	2010	84.5	76.0	94.1
Lesotho	Mafeteng	LIKHOELE	18886	2015	67.5	59.0	76.4
Lesotho	Mafeteng	LITSOENENG	18887	2000	86.2	75.9	97.2
Lesotho	Mafeteng	LITSOENENG	18887	2005	96.0	84.6	108.9
Lesotho	Mafeteng	LITSOENENG	18887	2010	81.4	72.0	92.7

Admin 0	Admin 1	Admin 2	GAUL Code	Year	Under-5 mortality (per 1,000 livebirths)		
					Estimate	Lower bound, 95% UI	Upper bound, 95% UI
Lesotho	Mafeteng	LITSOENENG	18887	2015	63.9	55.1	73.4
Lesotho	Mafeteng	MALEALEA	18888	2000	101.2	92.1	110.7
Lesotho	Mafeteng	MALEALEA	18888	2005	115.1	105.0	126.7
Lesotho	Mafeteng	MALEALEA	18888	2010	91.8	83.2	101.5
Lesotho	Mafeteng	MALEALEA	18888	2015	73.2	64.8	82.4
Lesotho	Mafeteng	MALUMENG	18889	2000	98.5	89.1	107.9
Lesotho	Mafeteng	MALUMENG	18889	2005	111.8	101.6	123.1
Lesotho	Mafeteng	MALUMENG	18889	2010	90.1	81.4	99.5
Lesotho	Mafeteng	MALUMENG	18889	2015	71.6	62.9	81.1
Lesotho	Mafeteng	MASEMOUSU	18890	2000	106.4	96.6	116.6
Lesotho	Mafeteng	MASEMOUSU	18890	2005	119.2	109.1	131.1
Lesotho	Mafeteng	MASEMOUSU	18890	2010	94.5	85.4	104.8
Lesotho	Mafeteng	MASEMOUSU	18890	2015	75.6	67.0	85.4
Lesotho	Mafeteng	MATELILE	18891	2000	98.8	89.8	107.8
Lesotho	Mafeteng	MATELILE	18891	2005	112.5	102.6	124.4
Lesotho	Mafeteng	MATELILE	18891	2010	90.4	81.7	99.8
Lesotho	Mafeteng	MATELILE	18891	2015	72.5	64.0	81.6
Lesotho	Mafeteng	MATHEBE	18892	2000	93.1	84.3	101.9
Lesotho	Mafeteng	MATHEBE	18892	2005	105.8	95.9	117.1
Lesotho	Mafeteng	MATHEBE	18892	2010	86.9	77.9	96.5
Lesotho	Mafeteng	MATHEBE	18892	2015	68.8	60.5	77.9
Lesotho	Mafeteng	Maboloka	18893	2000	94.5	84.9	103.9
Lesotho	Mafeteng	Maboloka	18893	2005	106.3	96.4	117.1
Lesotho	Mafeteng	Maboloka	18893	2010	87.4	78.4	97.3
Lesotho	Mafeteng	Maboloka	18893	2015	69.4	60.9	78.9
Lesotho	Mafeteng	QALABANE	18894	2000	89.7	80.0	99.5
Lesotho	Mafeteng	QALABANE	18894	2005	100.1	89.7	111.0
Lesotho	Mafeteng	QALABANE	18894	2010	84.2	75.2	94.5
Lesotho	Mafeteng	QALABANE	18894	2015	66.3	57.6	75.5
Lesotho	Mafeteng	SEBELEKOANE	18895	2000	99.9	91.4	108.9
Lesotho	Mafeteng	SEBELEKOANE	18895	2005	113.6	104.5	124.6
Lesotho	Mafeteng	SEBELEKOANE	18895	2010	90.7	82.4	99.7
Lesotho	Mafeteng	SEBELEKOANE	18895	2015	72.8	64.6	81.4
Lesotho	Mafeteng	TAJANE	18896	2000	95.8	86.8	104.9
Lesotho	Mafeteng	TAJANE	18896	2005	109.6	99.9	121.7
Lesotho	Mafeteng	TAJANE	18896	2010	88.7	80.2	98.1
Lesotho	Mafeteng	TAJANE	18896	2015	70.4	62.2	79.3
Lesotho	Mafeteng	TEBANG	18897	2000	89.9	80.1	99.4
Lesotho	Mafeteng	TEBANG	18897	2005	101.8	91.3	113.0
Lesotho	Mafeteng	TEBANG	18897	2010	84.7	75.6	94.7
Lesotho	Mafeteng	TEBANG	18897	2015	66.9	58.6	76.4
Lesotho	Mafeteng	THABA-CHITJA	18898	2000	98.5	87.9	108.4
Lesotho	Mafeteng	THABA-CHITJA	18898	2005	112.2	101.6	123.7
Lesotho	Mafeteng	THABA-CHITJA	18898	2010	90.7	81.7	100.7
Lesotho	Mafeteng	THABA-CHITJA	18898	2015	72.2	63.6	82.0
Lesotho	Mafeteng	THABA-TSOEU	18899	2000	96.5	87.2	105.8
Lesotho	Mafeteng	THABA-TSOEU	18899	2005	110.2	100.0	122.6
Lesotho	Mafeteng	THABA-TSOEU	18899	2010	89.5	80.8	99.1
Lesotho	Mafeteng	THABA-TSOEU	18899	2015	70.5	62.3	79.9
Lesotho	Mafeteng	THABANA-MORENA	18900	2000	97.8	88.2	107.0
Lesotho	Mafeteng	THABANA-MORENA	18900	2005	111.9	101.7	122.8
Lesotho	Mafeteng	THABANA-MORENA	18900	2010	90.1	81.3	99.8
Lesotho	Mafeteng	THABANA-MORENA	18900	2015	71.8	63.4	81.3
Lesotho	Mafeteng	TSAKHOLO	18901	2000	87.3	77.3	97.6
Lesotho	Mafeteng	TSAKHOLO	18901	2005	97.8	87.0	109.7
Lesotho	Mafeteng	TSAKHOLO	18901	2010	82.5	72.9	93.0
Lesotho	Mafeteng	TSAKHOLO	18901	2015	65.3	56.5	74.9
Lesotho	Mafeteng	URBAN	18902	2000	87.7	78.8	96.7
Lesotho	Mafeteng	URBAN	18902	2005	97.1	87.7	107.0
Lesotho	Mafeteng	URBAN	18902	2010	82.4	73.7	92.3
Lesotho	Mafeteng	URBAN	18902	2015	66.5	58.0	75.3
Lesotho	Maseru	CPA	18903	2000	108.6	99.5	118.1
Lesotho	Maseru	CPA	18903	2005	119.5	109.8	129.9
Lesotho	Maseru	CPA	18903	2010	95.8	86.6	105.0
Lesotho	Maseru	CPA	18903	2015	74.5	66.2	82.9
Lesotho	Maseru	Likalaneng	18904	2000	101.1	92.3	109.9
Lesotho	Maseru	Likalaneng	18904	2005	114.4	105.3	125.0
Lesotho	Maseru	Likalaneng	18904	2010	91.5	82.7	101.2
Lesotho	Maseru	Likalaneng	18904	2015	71.6	63.5	80.4
Lesotho	Maseru	Makhaleng	18905	2000	103.1	94.3	112.3
Lesotho	Maseru	Makhaleng	18905	2005	115.2	105.6	125.9
Lesotho	Maseru	Makhaleng	18905	2010	92.1	83.3	101.3
Lesotho	Maseru	Makhaleng	18905	2015	71.8	63.5	80.6
Lesotho	Maseru	Matsieng	18906	2000	93.2	84.9	101.2
Lesotho	Maseru	Matsieng	18906	2005	106.4	97.7	117.2
Lesotho	Maseru	Matsieng	18906	2010	86.4	78.9	94.8
Lesotho	Maseru	Matsieng	18906	2015	69.3	61.4	77.6
Lesotho	Maseru	Mazenod	18907	2000	90.2	82.7	98.5
Lesotho	Maseru	Mazenod	18907	2005	102.4	93.9	111.7
Lesotho	Maseru	Mazenod	18907	2010	85.4	77.8	94.0
Lesotho	Maseru	Mazenod	18907	2015	68.1	60.7	76.3
Lesotho	Maseru	Mochochoko	18908	2000	105.5	97.2	114.9
Lesotho	Maseru	Mochochoko	18908	2005	116.9	107.7	127.6
Lesotho	Maseru	Mochochoko	18908	2010	93.8	85.0	103.2
Lesotho	Maseru	Mochochoko	18908	2015	72.9	64.9	81.6

Admin 0	Admin 1	Admin 2	GAUL Code	Year	Under-5 mortality (per 1,000 livebirths)		
					Estimate	Lower bound, 95% UI	Upper bound, 95% UI
Lesotho	Maseru	Moeaneng	18909	2000	108.1	99.2	117.8
Lesotho	Maseru	Moeaneng	18909	2005	119.2	109.5	129.7
Lesotho	Maseru	Moeaneng	18909	2010	95.4	86.5	104.8
Lesotho	Maseru	Moeaneng	18909	2015	76.3	67.8	85.2
Lesotho	Maseru	Mofoka	18910	2000	94.6	86.7	102.4
Lesotho	Maseru	Mofoka	18910	2005	108.3	99.5	118.6
Lesotho	Maseru	Mofoka	18910	2010	87.6	79.7	95.7
Lesotho	Maseru	Mofoka	18910	2015	70.2	62.4	78.4
Lesotho	Maseru	Mphatsoenyane	18911	2000	111.6	101.7	122.4
Lesotho	Maseru	Mphatsoenyane	18911	2005	122.5	111.4	134.1
Lesotho	Maseru	Mphatsoenyane	18911	2010	98.2	88.4	108.2
Lesotho	Maseru	Mphatsoenyane	18911	2015	78.1	69.0	87.6
Lesotho	Maseru	Nyakosoba	18912	2000	99.1	89.9	108.2
Lesotho	Maseru	Nyakosoba	18912	2005	111.8	101.0	123.1
Lesotho	Maseru	Nyakosoba	18912	2010	89.1	80.3	98.1
Lesotho	Maseru	Nyakosoba	18912	2015	70.6	62.0	79.3
Lesotho	Maseru	Pontseng	18913	2000	104.4	94.6	114.6
Lesotho	Maseru	Pontseng	18913	2005	115.4	104.6	126.7
Lesotho	Maseru	Pontseng	18913	2010	92.6	83.3	102.8
Lesotho	Maseru	Pontseng	18913	2015	71.7	63.5	80.6
Lesotho	Maseru	Popa	18914	2000	91.5	83.7	99.3
Lesotho	Maseru	Popa	18914	2005	104.4	95.7	114.1
Lesotho	Maseru	Popa	18914	2010	85.9	78.3	94.1
Lesotho	Maseru	Popa	18914	2015	69.3	61.5	77.8
Lesotho	Maseru	Ramabanta	18915	2000	102.1	92.8	111.2
Lesotho	Maseru	Ramabanta	18915	2005	115.4	105.6	126.0
Lesotho	Maseru	Ramabanta	18915	2010	92.1	83.0	101.2
Lesotho	Maseru	Ramabanta	18915	2015	72.8	64.3	81.5
Lesotho	Maseru	Rapoleboea	18916	2000	104.2	94.8	113.4
Lesotho	Maseru	Rapoleboea	18916	2005	116.4	106.3	127.1
Lesotho	Maseru	Rapoleboea	18916	2010	93.0	83.8	102.2
Lesotho	Maseru	Rapoleboea	18916	2015	73.2	64.5	81.7
Lesotho	Maseru	Ratau	18917	2000	94.2	86.5	102.4
Lesotho	Maseru	Ratau	18917	2005	107.7	99.1	117.8
Lesotho	Maseru	Ratau	18917	2010	86.9	78.8	95.1
Lesotho	Maseru	Ratau	18917	2015	69.4	61.4	77.8
Lesotho	Maseru	Ribaneng	18918	2000	104.7	95.6	114.0
Lesotho	Maseru	Ribaneng	18918	2005	117.8	108.2	128.6
Lesotho	Maseru	Ribaneng	18918	2010	93.9	85.4	103.2
Lesotho	Maseru	Ribaneng	18918	2015	74.7	66.1	83.8
Lesotho	Maseru	Rothe	18919	2000	90.9	82.9	99.1
Lesotho	Maseru	Rothe	18919	2005	104.3	95.3	114.5
Lesotho	Maseru	Rothe	18919	2010	86.3	78.4	95.3
Lesotho	Maseru	Rothe	18919	2015	68.1	60.6	76.4
Lesotho	Maseru	Semonkong	18920	2000	109.3	100.0	119.9
Lesotho	Maseru	Semonkong	18920	2005	120.7	110.3	132.1
Lesotho	Maseru	Semonkong	18920	2010	96.8	87.8	106.6
Lesotho	Maseru	Semonkong	18920	2015	78.4	69.2	87.8
Lesotho	Maseru	Serooeng	18921	2000	99.0	89.2	108.7
Lesotho	Maseru	Serooeng	18921	2005	112.2	101.3	123.7
Lesotho	Maseru	Serooeng	18921	2010	89.4	80.0	98.5
Lesotho	Maseru	Serooeng	18921	2015	71.2	62.4	80.1
Lesotho	Maseru	Setibing	18922	2000	102.2	93.0	111.6
Lesotho	Maseru	Setibing	18922	2005	113.9	103.8	125.2
Lesotho	Maseru	Setibing	18922	2010	90.6	81.8	99.8
Lesotho	Maseru	Setibing	18922	2015	70.5	62.3	78.9
Lesotho	Maseru	Setleketseng	18923	2000	98.8	90.0	107.5
Lesotho	Maseru	Setleketseng	18923	2005	111.9	102.6	122.6
Lesotho	Maseru	Setleketseng	18923	2010	89.3	81.1	97.9
Lesotho	Maseru	Setleketseng	18923	2015	71.4	63.2	79.8
Lesotho	Maseru	Thaba-Bosiu	18924	2000	91.6	84.1	99.5
Lesotho	Maseru	Thaba-Bosiu	18924	2005	104.7	96.3	114.6
Lesotho	Maseru	Thaba-Bosiu	18924	2010	86.1	78.4	94.2
Lesotho	Maseru	Thaba-Bosiu	18924	2015	68.7	61.2	77.2
Lesotho	Maseru	Urban	18925	2000	84.9	77.2	93.1
Lesotho	Maseru	Urban	18925	2005	95.7	87.3	105.4
Lesotho	Maseru	Urban	18925	2010	80.5	72.8	88.7
Lesotho	Maseru	Urban	18925	2015	64.5	56.8	73.1
Lesotho	Mohale's Hoek	CPA	18926	2000	113.1	105.2	121.6
Lesotho	Mohale's Hoek	CPA	18926	2005	120.4	111.8	129.1
Lesotho	Mohale's Hoek	CPA	18926	2010	96.6	89.0	105.3
Lesotho	Mohale's Hoek	CPA	18926	2015	74.6	67.0	82.9
Lesotho	Mohale's Hoek	Ha Anone	18927	2000	107.6	97.8	119.3
Lesotho	Mohale's Hoek	Ha Anone	18927	2005	119.4	108.0	132.2
Lesotho	Mohale's Hoek	Ha Anone	18927	2010	94.2	84.8	104.8
Lesotho	Mohale's Hoek	Ha Anone	18927	2015	73.5	64.4	83.5
Lesotho	Mohale's Hoek	Ha Leronti	18928	2000	107.7	99.2	117.3
Lesotho	Mohale's Hoek	Ha Leronti	18928	2005	119.6	109.9	130.3
Lesotho	Mohale's Hoek	Ha Leronti	18928	2010	95.6	86.7	105.2
Lesotho	Mohale's Hoek	Ha Leronti	18928	2015	74.5	65.5	83.8
Lesotho	Mohale's Hoek	Ha Teke	18929	2000	107.5	97.7	118.2
Lesotho	Mohale's Hoek	Ha Teke	18929	2005	119.6	109.1	132.1
Lesotho	Mohale's Hoek	Ha Teke	18929	2010	95.0	85.9	105.4
Lesotho	Mohale's Hoek	Ha Teke	18929	2015	74.2	65.5	83.4
Lesotho	Mohale's Hoek	Ha Thabo	18930	2000	111.9	102.0	122.9

Admin 0	Admin 1	Admin 2	GAUL Code	Year	Under-5 mortality (per 1,000 livebirths)		
					Estimate	Lower bound, 95% UI	Upper bound, 95% UI
Lesotho	Mohale's Hoek	Ha Thabo	18930	2005	123.4	112.3	135.2
Lesotho	Mohale's Hoek	Ha Thabo	18930	2010	97.7	88.4	108.7
Lesotho	Mohale's Hoek	Ha Thabo	18930	2015	76.2	66.7	85.9
Lesotho	Mohale's Hoek	Hloahloeng	18931	2000	104.3	96.0	113.9
Lesotho	Mohale's Hoek	Hloahloeng	18931	2005	116.1	106.5	126.5
Lesotho	Mohale's Hoek	Hloahloeng	18931	2010	92.7	84.3	102.2
Lesotho	Mohale's Hoek	Hloahloeng	18931	2015	72.6	63.8	81.4
Lesotho	Mohale's Hoek	Ketane	18932	2000	115.7	105.1	126.4
Lesotho	Mohale's Hoek	Ketane	18932	2005	125.1	113.7	136.7
Lesotho	Mohale's Hoek	Ketane	18932	2010	99.6	90.1	109.9
Lesotho	Mohale's Hoek	Ketane	18932	2015	78.0	68.9	87.7
Lesotho	Mohale's Hoek	Likhutloaneng	18933	2000	108.0	99.0	118.0
Lesotho	Mohale's Hoek	Likhutloaneng	18933	2005	120.9	110.6	132.1
Lesotho	Mohale's Hoek	Likhutloaneng	18933	2010	95.9	87.1	105.8
Lesotho	Mohale's Hoek	Likhutloaneng	18933	2015	74.7	65.6	84.4
Lesotho	Mohale's Hoek	Mafikalisiu	18934	2000	117.3	106.1	130.0
Lesotho	Mohale's Hoek	Mafikalisiu	18934	2005	126.1	113.5	139.6
Lesotho	Mohale's Hoek	Mafikalisiu	18934	2010	99.5	89.4	111.4
Lesotho	Mohale's Hoek	Mafikalisiu	18934	2015	77.6	68.3	87.9
Lesotho	Mohale's Hoek	Mekaling	18935	2000	103.8	94.1	115.2
Lesotho	Mohale's Hoek	Mekaling	18935	2005	115.3	104.0	127.3
Lesotho	Mohale's Hoek	Mekaling	18935	2010	92.0	83.1	103.2
Lesotho	Mohale's Hoek	Mekaling	18935	2015	71.3	62.6	81.5
Lesotho	Mohale's Hoek	Mohloka Kobo	18936	2000	103.3	93.8	114.1
Lesotho	Mohale's Hoek	Mohloka Kobo	18936	2005	115.6	104.9	127.3
Lesotho	Mohale's Hoek	Mohloka Kobo	18936	2010	91.9	83.2	101.7
Lesotho	Mohale's Hoek	Mohloka Kobo	18936	2015	72.8	64.3	82.2
Lesotho	Mohale's Hoek	Morifi	18937	2000	99.5	89.4	109.9
Lesotho	Mohale's Hoek	Morifi	18937	2005	110.7	99.4	122.9
Lesotho	Mohale's Hoek	Morifi	18937	2010	89.5	80.8	100.1
Lesotho	Mohale's Hoek	Morifi	18937	2015	70.4	62.0	80.5
Lesotho	Mohale's Hoek	Motsekuoa	18938	2000	105.0	96.2	114.7
Lesotho	Mohale's Hoek	Motsekuoa	18938	2005	116.2	106.5	126.1
Lesotho	Mohale's Hoek	Motsekuoa	18938	2010	93.5	85.0	103.1
Lesotho	Mohale's Hoek	Motsekuoa	18938	2015	73.0	64.5	82.0
Lesotho	Mohale's Hoek	Phamong	18939	2000	109.9	99.4	122.4
Lesotho	Mohale's Hoek	Phamong	18939	2005	121.6	110.0	135.3
Lesotho	Mohale's Hoek	Phamong	18939	2010	95.7	86.0	107.4
Lesotho	Mohale's Hoek	Phamong	18939	2015	74.2	65.2	84.5
Lesotho	Mohale's Hoek	Pontseng	18940	2000	101.3	92.4	111.1
Lesotho	Mohale's Hoek	Pontseng	18940	2005	115.4	105.8	126.1
Lesotho	Mohale's Hoek	Pontseng	18940	2010	92.9	84.5	102.7
Lesotho	Mohale's Hoek	Pontseng	18940	2015	73.6	65.1	82.6
Lesotho	Mohale's Hoek	Qabane	18941	2000	107.6	98.2	117.7
Lesotho	Mohale's Hoek	Qabane	18941	2005	115.1	105.4	125.9
Lesotho	Mohale's Hoek	Qabane	18941	2010	93.3	84.5	103.2
Lesotho	Mohale's Hoek	Qabane	18941	2015	72.0	63.4	81.1
Lesotho	Mohale's Hoek	Qalakheng	18942	2000	100.7	90.5	110.7
Lesotho	Mohale's Hoek	Qalakheng	18942	2005	112.5	102.0	124.2
Lesotho	Mohale's Hoek	Qalakheng	18942	2010	90.7	82.0	100.8
Lesotho	Mohale's Hoek	Qalakheng	18942	2015	71.6	63.2	80.8
Lesotho	Mohale's Hoek	Qhalasi	18943	2000	98.9	87.4	110.1
Lesotho	Mohale's Hoek	Qhalasi	18943	2005	110.3	98.8	123.0
Lesotho	Mohale's Hoek	Qhalasi	18943	2010	89.8	80.2	100.6
Lesotho	Mohale's Hoek	Qhalasi	18943	2015	70.5	61.9	80.5
Lesotho	Mohale's Hoek	Qhobong	18944	2000	117.4	106.2	130.4
Lesotho	Mohale's Hoek	Qhobong	18944	2005	125.7	113.8	139.3
Lesotho	Mohale's Hoek	Qhobong	18944	2010	99.0	89.1	110.8
Lesotho	Mohale's Hoek	Qhobong	18944	2015	77.3	67.9	87.7
Lesotho	Mohale's Hoek	Setofolo	18945	2000	104.1	95.4	114.0
Lesotho	Mohale's Hoek	Setofolo	18945	2005	114.8	104.9	125.7
Lesotho	Mohale's Hoek	Setofolo	18945	2010	92.4	83.8	102.5
Lesotho	Mohale's Hoek	Setofolo	18945	2015	71.1	62.5	80.1
Lesotho	Mohale's Hoek	Silooe	18946	2000	97.9	87.2	108.4
Lesotho	Mohale's Hoek	Silooe	18946	2005	108.6	97.6	120.2
Lesotho	Mohale's Hoek	Silooe	18946	2010	88.7	79.4	99.1
Lesotho	Mohale's Hoek	Silooe	18946	2015	69.9	61.3	79.5
Lesotho	Mohale's Hoek	Thaba-Tsoeu	18947	2000	109.3	99.6	120.1
Lesotho	Mohale's Hoek	Thaba-Tsoeu	18947	2005	120.1	110.0	132.0
Lesotho	Mohale's Hoek	Thaba-Tsoeu	18947	2010	96.0	87.2	105.9
Lesotho	Mohale's Hoek	Thaba-Tsoeu	18947	2015	74.7	66.1	83.6
Lesotho	Mohale's Hoek	Urban	18948	2000	97.1	87.4	106.8
Lesotho	Mohale's Hoek	Urban	18948	2005	106.9	96.9	118.2
Lesotho	Mohale's Hoek	Urban	18948	2010	88.3	80.0	97.8
Lesotho	Mohale's Hoek	Urban	18948	2015	70.8	62.5	80.1
Lesotho	Mokhotlong	'Meta	18949	2000	104.2	94.8	115.5
Lesotho	Mokhotlong	'Meta	18949	2005	106.1	96.2	116.7
Lesotho	Mokhotlong	'Meta	18949	2010	86.9	78.0	97.2
Lesotho	Mokhotlong	'Meta	18949	2015	67.3	58.5	77.8
Lesotho	Mokhotlong	Bobatsi	18950	2000	105.0	96.1	115.0
Lesotho	Mokhotlong	Bobatsi	18950	2005	108.4	99.2	118.7
Lesotho	Mokhotlong	Bobatsi	18950	2010	88.5	79.3	97.9
Lesotho	Mokhotlong	Bobatsi	18950	2015	67.6	59.1	76.9
Lesotho	Mokhotlong	CPA	18951	2000	105.2	96.4	115.0
Lesotho	Mokhotlong	CPA	18951	2005	106.8	97.9	116.9

Admin 0	Admin 1	Admin 2	GAUL Code	Year	Under-5 mortality (per 1,000 livebirths)		
					Estimate	Lower bound, 95% UI	Upper bound, 95% UI
Lesotho	Mokhotlong	CPA	18951	2010	86.3	78.2	95.5
Lesotho	Mokhotlong	CPA	18951	2015	67.1	58.5	76.7
Lesotho	Mokhotlong	Linakeng	18952	2000	107.6	97.9	118.3
Lesotho	Mokhotlong	Linakeng	18952	2005	111.4	101.3	122.0
Lesotho	Mokhotlong	Linakeng	18952	2010	90.6	81.2	100.0
Lesotho	Mokhotlong	Linakeng	18952	2015	69.2	60.8	78.5
Lesotho	Mokhotlong	Liphakoeng	18953	2000	102.0	92.2	112.8
Lesotho	Mokhotlong	Liphakoeng	18953	2005	104.8	95.1	115.7
Lesotho	Mokhotlong	Liphakoeng	18953	2010	86.2	76.6	96.5
Lesotho	Mokhotlong	Liphakoeng	18953	2015	65.5	56.9	75.3
Lesotho	Mokhotlong	Malingoaneng	18954	2000	106.9	97.9	117.2
Lesotho	Mokhotlong	Malingoaneng	18954	2005	110.0	100.8	120.5
Lesotho	Mokhotlong	Malingoaneng	18954	2010	89.9	81.0	99.3
Lesotho	Mokhotlong	Malingoaneng	18954	2015	69.2	60.6	79.4
Lesotho	Mokhotlong	Maluba-Lube	18955	2000	102.3	93.3	112.2
Lesotho	Mokhotlong	Maluba-Lube	18955	2005	106.8	98.1	117.1
Lesotho	Mokhotlong	Maluba-Lube	18955	2010	87.4	78.5	96.6
Lesotho	Mokhotlong	Maluba-Lube	18955	2015	67.5	59.3	77.0
Lesotho	Mokhotlong	Mapholaneng	18956	2000	105.5	96.4	116.8
Lesotho	Mokhotlong	Mapholaneng	18956	2005	107.7	97.8	118.6
Lesotho	Mokhotlong	Mapholaneng	18956	2010	88.2	79.3	97.7
Lesotho	Mokhotlong	Mapholaneng	18956	2015	68.4	59.7	78.8
Lesotho	Mokhotlong	Mateanong	18957	2000	105.7	94.7	117.2
Lesotho	Mokhotlong	Mateanong	18957	2005	107.5	96.9	119.4
Lesotho	Mokhotlong	Mateanong	18957	2010	86.6	76.8	96.8
Lesotho	Mokhotlong	Mateanong	18957	2015	68.9	59.5	79.6
Lesotho	Mokhotlong	Matsoku	18958	2000	108.6	98.8	119.2
Lesotho	Mokhotlong	Matsoku	18958	2005	112.3	102.1	123.6
Lesotho	Mokhotlong	Matsoku	18958	2010	92.1	83.0	102.4
Lesotho	Mokhotlong	Matsoku	18958	2015	76.8	67.0	88.2
Lesotho	Mokhotlong	Meno	18959	2000	106.9	97.5	117.3
Lesotho	Mokhotlong	Meno	18959	2005	110.9	101.2	121.3
Lesotho	Mokhotlong	Meno	18959	2010	90.4	81.4	100.5
Lesotho	Mokhotlong	Meno	18959	2015	75.0	65.7	86.1
Lesotho	Mokhotlong	Moeketsane	18960	2000	104.0	95.1	113.6
Lesotho	Mokhotlong	Moeketsane	18960	2005	108.7	99.5	118.5
Lesotho	Mokhotlong	Moeketsane	18960	2010	88.7	79.9	98.3
Lesotho	Mokhotlong	Moeketsane	18960	2015	67.8	59.4	77.0
Lesotho	Mokhotlong	Molikaliko	18961	2000	107.6	97.5	119.4
Lesotho	Mokhotlong	Molikaliko	18961	2005	107.0	97.0	117.9
Lesotho	Mokhotlong	Molikaliko	18961	2010	86.0	76.9	96.4
Lesotho	Mokhotlong	Molikaliko	18961	2015	66.2	57.5	76.1
Lesotho	Mokhotlong	Moshemong	18962	2000	99.6	89.8	110.5
Lesotho	Mokhotlong	Moshemong	18962	2005	102.3	92.4	113.7
Lesotho	Mokhotlong	Moshemong	18962	2010	83.2	73.7	93.6
Lesotho	Mokhotlong	Moshemong	18962	2015	65.5	56.6	75.4
Lesotho	Mokhotlong	Motsitseng	18963	2000	105.5	94.9	117.2
Lesotho	Mokhotlong	Motsitseng	18963	2005	105.9	96.2	117.2
Lesotho	Mokhotlong	Motsitseng	18963	2010	85.9	76.8	96.0
Lesotho	Mokhotlong	Motsitseng	18963	2015	66.8	57.6	77.2
Lesotho	Mokhotlong	Pae-Lea-Itlhatsoa	18964	2000	106.0	96.0	117.9
Lesotho	Mokhotlong	Pae-Lea-Itlhatsoa	18964	2005	106.3	95.7	117.8
Lesotho	Mokhotlong	Pae-Lea-Itlhatsoa	18964	2010	86.7	77.2	97.5
Lesotho	Mokhotlong	Pae-Lea-Itlhatsoa	18964	2015	67.1	58.6	77.8
Lesotho	Mokhotlong	Popa	18965	2000	103.7	94.1	115.3
Lesotho	Mokhotlong	Popa	18965	2005	106.3	96.4	117.6
Lesotho	Mokhotlong	Popa	18965	2010	86.9	77.3	97.1
Lesotho	Mokhotlong	Popa	18965	2015	66.9	58.4	76.8
Lesotho	Mokhotlong	Rafolatsane	18966	2000	103.5	94.1	114.2
Lesotho	Mokhotlong	Rafolatsane	18966	2005	106.7	97.1	117.8
Lesotho	Mokhotlong	Rafolatsane	18966	2010	87.4	78.2	97.4
Lesotho	Mokhotlong	Rafolatsane	18966	2015	67.2	58.7	76.8
Lesotho	Mokhotlong	Sehong-hong	18967	2000	104.8	94.9	115.6
Lesotho	Mokhotlong	Sehong-hong	18967	2005	107.1	97.5	117.7
Lesotho	Mokhotlong	Sehong-hong	18967	2010	87.6	78.1	97.6
Lesotho	Mokhotlong	Sehong-hong	18967	2015	67.1	58.5	76.9
Lesotho	Mokhotlong	Taung	18968	2000	108.7	99.3	118.6
Lesotho	Mokhotlong	Taung	18968	2005	112.1	102.8	122.2
Lesotho	Mokhotlong	Taung	18968	2010	91.4	82.6	100.7
Lesotho	Mokhotlong	Taung	18968	2015	70.1	61.6	80.4
Lesotho	Mokhotlong	Tlhanyaku	18969	2000	108.3	98.0	120.1
Lesotho	Mokhotlong	Tlhanyaku	18969	2005	107.4	97.4	118.2
Lesotho	Mokhotlong	Tlhanyaku	18969	2010	85.9	76.3	95.9
Lesotho	Mokhotlong	Tlhanyaku	18969	2015	65.7	56.7	75.6
Lesotho	Mokhotlong	Urban	18970	2000	101.3	91.0	112.9
Lesotho	Mokhotlong	Urban	18970	2005	104.0	94.0	115.5
Lesotho	Mokhotlong	Urban	18970	2010	84.8	75.0	95.2
Lesotho	Mokhotlong	Urban	18970	2015	65.5	56.9	75.4
Lesotho	Qacha's Nek	'Melikane	18971	2000	103.8	93.0	114.0
Lesotho	Qacha's Nek	'Melikane	18971	2005	110.2	99.8	121.1
Lesotho	Qacha's Nek	'Melikane	18971	2010	89.3	79.7	100.1
Lesotho	Qacha's Nek	'Melikane	18971	2015	68.5	59.8	78.3
Lesotho	Qacha's Nek	Ha Sekake	18972	2000	103.3	94.5	113.3
Lesotho	Qacha's Nek	Ha Sekake	18972	2005	116.0	106.3	127.4
Lesotho	Qacha's Nek	Ha Sekake	18972	2010	92.4	83.5	102.3

Admin 0	Admin 1	Admin 2	GAUL Code	Year	Under-5 mortality (per 1,000 livebirths)		
					Estimate	Lower bound, 95% UI	Upper bound, 95% UI
Lesotho	Qacha's Nek	Ha Sekake	18972	2015	72.4	63.3	81.4
Lesotho	Qacha's Nek	Ha Thuube	18973	2000	102.0	91.2	112.4
Lesotho	Qacha's Nek	Ha Thuube	18973	2005	108.4	97.5	119.7
Lesotho	Qacha's Nek	Ha Thuube	18973	2010	88.5	79.2	99.6
Lesotho	Qacha's Nek	Ha Thuube	18973	2015	67.2	58.7	76.7
Lesotho	Qacha's Nek	Lebakeng	18974	2000	104.4	94.8	114.0
Lesotho	Qacha's Nek	Lebakeng	18974	2005	111.9	102.1	122.1
Lesotho	Qacha's Nek	Lebakeng	18974	2010	90.6	81.7	100.4
Lesotho	Qacha's Nek	Lebakeng	18974	2015	69.1	60.9	78.2
Lesotho	Qacha's Nek	Leseling	18975	2000	100.7	91.1	110.4
Lesotho	Qacha's Nek	Leseling	18975	2005	110.5	99.6	121.6
Lesotho	Qacha's Nek	Leseling	18975	2010	89.4	80.3	99.3
Lesotho	Qacha's Nek	Leseling	18975	2015	68.8	60.0	78.3
Lesotho	Qacha's Nek	Linakeng	18976	2000	104.4	94.9	114.6
Lesotho	Qacha's Nek	Linakeng	18976	2005	113.6	103.1	124.8
Lesotho	Qacha's Nek	Linakeng	18976	2010	92.6	83.8	103.0
Lesotho	Qacha's Nek	Linakeng	18976	2015	69.5	61.1	78.7
Lesotho	Qacha's Nek	Makhoareng	18977	2000	96.8	86.6	107.0
Lesotho	Qacha's Nek	Makhoareng	18977	2005	104.3	93.8	115.8
Lesotho	Qacha's Nek	Makhoareng	18977	2010	84.7	75.8	95.8
Lesotho	Qacha's Nek	Makhoareng	18977	2015	65.0	56.6	74.7
Lesotho	Qacha's Nek	Matebeng	18978	2000	103.5	93.2	114.0
Lesotho	Qacha's Nek	Matebeng	18978	2005	109.4	99.1	119.7
Lesotho	Qacha's Nek	Matebeng	18978	2010	88.6	79.5	99.1
Lesotho	Qacha's Nek	Matebeng	18978	2015	68.0	59.4	77.4
Lesotho	Qacha's Nek	Matlali	18979	2000	97.7	87.3	108.0
Lesotho	Qacha's Nek	Matlali	18979	2005	108.7	97.0	120.8
Lesotho	Qacha's Nek	Matlali	18979	2010	87.8	78.0	98.9
Lesotho	Qacha's Nek	Matlali	18979	2015	67.8	58.9	78.2
Lesotho	Qacha's Nek	Mohlapiso	18980	2000	103.0	93.3	113.4
Lesotho	Qacha's Nek	Mohlapiso	18980	2005	112.0	100.9	123.5
Lesotho	Qacha's Nek	Mohlapiso	18980	2010	90.7	81.6	101.3
Lesotho	Qacha's Nek	Mohlapiso	18980	2015	69.0	60.3	78.4
Lesotho	Qacha's Nek	Mosaqane	18981	2000	97.5	87.1	108.8
Lesotho	Qacha's Nek	Mosaqane	18981	2005	106.7	95.0	119.5
Lesotho	Qacha's Nek	Mosaqane	18981	2010	86.3	76.4	97.9
Lesotho	Qacha's Nek	Mosaqane	18981	2015	65.7	57.1	76.0
Lesotho	Qacha's Nek	Mosenekeng	18982	2000	101.0	90.6	110.6
Lesotho	Qacha's Nek	Mosenekeng	18982	2005	109.2	99.1	119.7
Lesotho	Qacha's Nek	Mosenekeng	18982	2010	88.1	79.0	98.5
Lesotho	Qacha's Nek	Mosenekeng	18982	2015	68.3	59.5	77.4
Lesotho	Qacha's Nek	Moshebi	18983	2000	98.9	88.5	110.3
Lesotho	Qacha's Nek	Moshebi	18983	2005	103.6	92.6	114.7
Lesotho	Qacha's Nek	Moshebi	18983	2010	85.3	75.8	96.3
Lesotho	Qacha's Nek	Moshebi	18983	2015	64.2	55.4	73.9
Lesotho	Qacha's Nek	Mosuoe	18984	2000	98.2	87.8	108.4
Lesotho	Qacha's Nek	Mosuoe	18984	2005	108.8	97.6	120.8
Lesotho	Qacha's Nek	Mosuoe	18984	2010	87.8	78.2	98.5
Lesotho	Qacha's Nek	Mosuoe	18984	2015	67.9	59.1	78.1
Lesotho	Qacha's Nek	Pheellong	18985	2000	97.8	87.7	107.9
Lesotho	Qacha's Nek	Pheellong	18985	2005	108.8	97.7	120.6
Lesotho	Qacha's Nek	Pheellong	18985	2010	87.9	78.8	98.8
Lesotho	Qacha's Nek	Pheellong	18985	2015	68.3	59.4	78.1
Lesotho	Qacha's Nek	Rankakala	18986	2000	100.5	89.9	111.5
Lesotho	Qacha's Nek	Rankakala	18986	2005	108.1	97.0	120.3
Lesotho	Qacha's Nek	Rankakala	18986	2010	88.5	78.7	99.7
Lesotho	Qacha's Nek	Rankakala	18986	2015	67.8	59.1	78.1
Lesotho	Qacha's Nek	Sehlaba-Thebe	18987	2000	102.1	91.5	114.1
Lesotho	Qacha's Nek	Sehlaba-Thebe	18987	2005	107.1	96.1	118.8
Lesotho	Qacha's Nek	Sehlaba-Thebe	18987	2010	87.2	77.5	98.2
Lesotho	Qacha's Nek	Sehlaba-Thebe	18987	2015	65.8	56.7	75.5
Lesotho	Qacha's Nek	Sekhalabateng	18988	2000	111.4	101.0	123.0
Lesotho	Qacha's Nek	Sekhalabateng	18988	2005	120.9	110.1	133.5
Lesotho	Qacha's Nek	Sekhalabateng	18988	2010	97.4	87.4	108.6
Lesotho	Qacha's Nek	Sekhalabateng	18988	2015	73.8	64.2	83.4
Lesotho	Qacha's Nek	Tebellong	18989	2000	103.8	94.8	113.8
Lesotho	Qacha's Nek	Tebellong	18989	2005	114.6	104.7	125.7
Lesotho	Qacha's Nek	Tebellong	18989	2010	92.8	84.0	103.2
Lesotho	Qacha's Nek	Tebellong	18989	2015	70.5	61.6	79.5
Lesotho	Qacha's Nek	Tsatsa le Meno	18990	2000	102.4	92.1	113.0
Lesotho	Qacha's Nek	Tsatsa le Meno	18990	2005	107.6	96.9	118.6
Lesotho	Qacha's Nek	Tsatsa le Meno	18990	2010	87.9	78.7	99.3
Lesotho	Qacha's Nek	Tsatsa le Meno	18990	2015	66.0	57.2	75.6
Lesotho	Qacha's Nek	Urban	18991	2000	93.8	83.5	104.6
Lesotho	Qacha's Nek	Urban	18991	2005	104.3	92.8	117.4
Lesotho	Qacha's Nek	Urban	18991	2010	84.6	74.9	96.3
Lesotho	Qacha's Nek	Urban	18991	2015	66.3	57.4	76.8
Lesotho	Qacha's Nek	White Hill	18992	2000	100.4	90.9	110.5
Lesotho	Qacha's Nek	White Hill	18992	2005	112.6	102.0	124.7
Lesotho	Qacha's Nek	White Hill	18992	2010	90.3	81.5	100.6
Lesotho	Qacha's Nek	White Hill	18992	2015	69.6	60.6	78.8
Lesotho	Quthing	Metsi-Masooana (66)	19037	2000	111.1	100.5	122.7
Lesotho	Quthing	Metsi-Masooana (66)	19037	2005	121.5	110.0	134.2
Lesotho	Quthing	Metsi-Masooana (66)	19037	2010	96.2	86.0	107.4
Lesotho	Quthing	Metsi-Masooana (66)	19037	2015	75.0	65.5	85.2

Admin 0	Admin 1	Admin 2	GAUL Code	Year	Under-5 mortality (per 1,000 livebirths)		
					Estimate	Lower bound, 95% UI	Upper bound, 95% UI
Lesotho	Quthing	Moyeni (63)	19038	2000	101.1	91.0	113.2
Lesotho	Quthing	Moyeni (63)	19038	2005	113.3	101.4	125.9
Lesotho	Quthing	Moyeni (63)	19038	2010	91.2	81.9	103.2
Lesotho	Quthing	Moyeni (63)	19038	2015	70.8	62.0	81.7
Lesotho	Quthing	Name Unknown	18993	2000	102.7	91.2	117.1
Lesotho	Quthing	Name Unknown	18993	2005	114.4	102.0	128.4
Lesotho	Quthing	Name Unknown	18993	2010	90.8	79.7	103.5
Lesotho	Quthing	Name Unknown	18993	2015	70.5	60.8	82.2
Lesotho	Quthing	Name Unknown	18994	2000	107.3	97.3	119.1
Lesotho	Quthing	Name Unknown	18994	2005	119.6	108.7	132.7
Lesotho	Quthing	Name Unknown	18994	2010	94.2	84.6	105.2
Lesotho	Quthing	Name Unknown	18994	2015	73.6	64.3	82.9
Lesotho	Quthing	Name Unknown	18995	2000	114.7	103.1	126.7
Lesotho	Quthing	Name Unknown	18995	2005	123.2	110.7	137.0
Lesotho	Quthing	Name Unknown	18995	2010	98.8	88.4	110.9
Lesotho	Quthing	Name Unknown	18995	2015	77.2	66.4	87.7
Lesotho	Quthing	Name Unknown	18996	2000	106.6	96.1	119.2
Lesotho	Quthing	Name Unknown	18996	2005	118.4	106.7	131.4
Lesotho	Quthing	Name Unknown	18996	2010	92.8	83.0	104.5
Lesotho	Quthing	Name Unknown	18996	2015	73.8	64.3	84.7
Lesotho	Quthing	Name Unknown	18997	2000	111.7	100.6	123.8
Lesotho	Quthing	Name Unknown	18997	2005	121.6	109.8	135.8
Lesotho	Quthing	Name Unknown	18997	2010	98.3	87.9	110.2
Lesotho	Quthing	Name Unknown	18997	2015	75.3	65.0	85.4
Lesotho	Quthing	Name Unknown	18998	2000	112.1	99.4	126.8
Lesotho	Quthing	Name Unknown	18998	2005	120.7	108.0	135.2
Lesotho	Quthing	Name Unknown	18998	2010	96.0	85.0	109.6
Lesotho	Quthing	Name Unknown	18998	2015	72.4	62.6	83.7
Lesotho	Quthing	Name Unknown	18999	2000	104.0	92.0	118.6
Lesotho	Quthing	Name Unknown	18999	2005	116.8	103.3	132.8
Lesotho	Quthing	Name Unknown	18999	2010	92.2	81.7	106.1
Lesotho	Quthing	Name Unknown	18999	2015	72.2	62.7	84.1
Lesotho	Quthing	Name Unknown	19000	2000	111.4	99.2	126.2
Lesotho	Quthing	Name Unknown	19000	2005	120.0	106.9	134.7
Lesotho	Quthing	Name Unknown	19000	2010	95.8	84.5	109.3
Lesotho	Quthing	Name Unknown	19000	2015	71.2	61.5	82.8
Lesotho	Quthing	Name Unknown	19001	2000	111.7	100.5	123.5
Lesotho	Quthing	Name Unknown	19001	2005	124.1	112.6	137.4
Lesotho	Quthing	Name Unknown	19001	2010	98.6	88.7	110.1
Lesotho	Quthing	Name Unknown	19001	2015	77.3	67.0	87.7
Lesotho	Quthing	Name Unknown	19002	2000	110.7	99.5	123.1
Lesotho	Quthing	Name Unknown	19002	2005	120.2	108.0	133.6
Lesotho	Quthing	Name Unknown	19002	2010	95.8	85.2	107.7
Lesotho	Quthing	Name Unknown	19002	2015	73.6	64.1	84.1
Lesotho	Quthing	Name Unknown	19003	2000	107.8	97.0	119.5
Lesotho	Quthing	Name Unknown	19003	2005	121.6	109.7	135.0
Lesotho	Quthing	Name Unknown	19003	2010	96.6	86.0	108.1
Lesotho	Quthing	Name Unknown	19003	2015	75.9	65.7	86.2
Lesotho	Quthing	Name Unknown	19005	2000	97.7	86.9	111.5
Lesotho	Quthing	Name Unknown	19005	2005	107.9	95.8	122.5
Lesotho	Quthing	Name Unknown	19005	2010	88.4	78.4	101.5
Lesotho	Quthing	Name Unknown	19005	2015	68.8	59.8	80.5
Lesotho	Quthing	Name Unknown	19007	2000	101.0	91.0	113.1
Lesotho	Quthing	Name Unknown	19007	2005	115.7	103.9	128.5
Lesotho	Quthing	Name Unknown	19007	2010	92.6	83.0	104.5
Lesotho	Quthing	Name Unknown	19007	2015	72.1	62.8	83.3
Lesotho	Quthing	Name Unknown	19008	2000	113.6	101.2	126.1
Lesotho	Quthing	Name Unknown	19008	2005	121.5	108.1	135.3
Lesotho	Quthing	Name Unknown	19008	2010	97.7	86.7	109.8
Lesotho	Quthing	Name Unknown	19008	2015	76.7	65.7	87.8
Lesotho	Quthing	Name Unknown	19010	2000	103.3	92.5	115.7
Lesotho	Quthing	Name Unknown	19010	2005	116.5	104.8	129.8
Lesotho	Quthing	Name Unknown	19010	2010	92.2	82.4	104.6
Lesotho	Quthing	Name Unknown	19010	2015	73.1	63.4	84.0
Lesotho	Quthing	Name Unknown	19011	2000	106.4	97.2	116.4
Lesotho	Quthing	Name Unknown	19011	2005	120.9	110.6	132.5
Lesotho	Quthing	Name Unknown	19011	2010	94.8	85.9	105.2
Lesotho	Quthing	Name Unknown	19011	2015	75.0	65.4	84.6
Lesotho	Quthing	Name Unknown	19013	2000	103.2	91.7	117.0
Lesotho	Quthing	Name Unknown	19013	2005	116.6	103.8	131.6
Lesotho	Quthing	Name Unknown	19013	2010	92.4	82.0	105.7
Lesotho	Quthing	Name Unknown	19013	2015	72.1	62.6	83.9
Lesotho	Quthing	Name Unknown	19014	2000	108.8	97.6	121.2
Lesotho	Quthing	Name Unknown	19014	2005	119.1	107.4	131.9
Lesotho	Quthing	Name Unknown	19014	2010	94.5	84.3	106.8
Lesotho	Quthing	Name Unknown	19014	2015	73.2	63.9	83.3
Lesotho	Quthing	Name Unknown	19015	2000	105.6	95.6	116.9
Lesotho	Quthing	Name Unknown	19015	2005	118.5	107.7	131.1
Lesotho	Quthing	Name Unknown	19015	2010	93.5	83.7	105.1
Lesotho	Quthing	Name Unknown	19015	2015	73.3	64.1	82.9
Lesotho	Quthing	Name Unknown	19016	2000	100.7	90.1	113.5
Lesotho	Quthing	Name Unknown	19016	2005	114.4	102.4	127.8
Lesotho	Quthing	Name Unknown	19016	2010	90.1	80.3	102.4
Lesotho	Quthing	Name Unknown	19016	2015	72.0	62.5	83.4
Lesotho	Quthing	Name Unknown	19017	2000	111.4	100.0	123.9

Admin 0	Admin 1	Admin 2	GAUL Code	Year	Under-5 mortality (per 1,000 livebirths)		
					Estimate	Lower bound, 95% UI	Upper bound, 95% UI
Lesotho	Quthing	Name Unknown	19017	2005	122.0	109.9	136.8
Lesotho	Quthing	Name Unknown	19017	2010	98.6	87.7	111.0
Lesotho	Quthing	Name Unknown	19017	2015	76.7	66.1	87.2
Lesotho	Quthing	Name Unknown	19018	2000	114.1	104.1	126.0
Lesotho	Quthing	Name Unknown	19018	2005	124.4	113.3	137.6
Lesotho	Quthing	Name Unknown	19018	2010	98.7	88.8	109.8
Lesotho	Quthing	Name Unknown	19018	2015	75.8	66.0	85.5
Lesotho	Quthing	Name Unknown	19019	2000	113.4	103.5	125.3
Lesotho	Quthing	Name Unknown	19019	2005	124.9	113.8	137.7
Lesotho	Quthing	Name Unknown	19019	2010	98.5	88.8	109.4
Lesotho	Quthing	Name Unknown	19019	2015	76.9	66.9	86.9
Lesotho	Quthing	Name Unknown	19020	2000	106.5	96.5	117.3
Lesotho	Quthing	Name Unknown	19020	2005	121.3	110.1	134.1
Lesotho	Quthing	Name Unknown	19020	2010	96.5	86.5	107.6
Lesotho	Quthing	Name Unknown	19020	2015	74.6	64.6	84.3
Lesotho	Quthing	Name Unknown	19022	2000	116.2	104.6	128.7
Lesotho	Quthing	Name Unknown	19022	2005	124.7	112.6	138.7
Lesotho	Quthing	Name Unknown	19022	2010	99.4	88.7	111.7
Lesotho	Quthing	Name Unknown	19022	2015	76.1	65.7	86.4
Lesotho	Quthing	Name Unknown	19023	2000	99.2	88.7	112.0
Lesotho	Quthing	Name Unknown	19023	2005	112.7	100.6	125.8
Lesotho	Quthing	Name Unknown	19023	2010	90.7	81.0	103.3
Lesotho	Quthing	Name Unknown	19023	2015	70.8	61.6	82.4
Lesotho	Quthing	Name Unknown	19024	2000	107.9	95.8	122.5
Lesotho	Quthing	Name Unknown	19024	2005	117.1	104.0	131.9
Lesotho	Quthing	Name Unknown	19024	2010	93.6	82.1	106.9
Lesotho	Quthing	Name Unknown	19024	2015	71.5	61.5	82.8
Lesotho	Quthing	Name Unknown	19025	2000	105.9	94.4	119.2
Lesotho	Quthing	Name Unknown	19025	2005	117.9	105.7	131.7
Lesotho	Quthing	Name Unknown	19025	2010	93.3	83.0	106.1
Lesotho	Quthing	Name Unknown	19025	2015	72.9	63.4	83.9
Lesotho	Quthing	Name Unknown	19026	2000	100.6	89.2	114.6
Lesotho	Quthing	Name Unknown	19026	2005	113.0	100.1	127.6
Lesotho	Quthing	Name Unknown	19026	2010	89.3	79.0	102.5
Lesotho	Quthing	Name Unknown	19026	2015	69.8	60.3	81.4
Lesotho	Quthing	Name Unknown	19027	2000	107.4	96.7	119.4
Lesotho	Quthing	Name Unknown	19027	2005	121.1	109.1	135.1
Lesotho	Quthing	Name Unknown	19027	2010	97.4	86.6	109.5
Lesotho	Quthing	Name Unknown	19027	2015	75.4	65.0	85.6
Lesotho	Quthing	Name Unknown	19028	2000	109.4	98.7	121.3
Lesotho	Quthing	Name Unknown	19028	2005	120.7	109.4	133.8
Lesotho	Quthing	Name Unknown	19028	2010	94.6	84.6	106.9
Lesotho	Quthing	Name Unknown	19028	2015	74.7	65.4	84.9
Lesotho	Quthing	Name Unknown	19029	2000	100.3	89.7	113.7
Lesotho	Quthing	Name Unknown	19029	2005	114.2	101.9	128.1
Lesotho	Quthing	Name Unknown	19029	2010	90.6	80.7	103.4
Lesotho	Quthing	Name Unknown	19029	2015	70.6	61.3	82.2
Lesotho	Quthing	Name Unknown	19030	2000	112.4	99.7	126.5
Lesotho	Quthing	Name Unknown	19030	2005	120.6	107.6	135.1
Lesotho	Quthing	Name Unknown	19030	2010	96.5	85.3	109.9
Lesotho	Quthing	Name Unknown	19030	2015	73.1	63.2	84.4
Lesotho	Quthing	Name Unknown	19032	2000	114.4	102.5	127.5
Lesotho	Quthing	Name Unknown	19032	2005	123.4	110.5	137.8
Lesotho	Quthing	Name Unknown	19032	2010	98.6	87.6	111.1
Lesotho	Quthing	Name Unknown	19032	2015	76.5	65.6	86.9
Lesotho	Quthing	Name Unknown	19034	2000	107.8	97.8	119.3
Lesotho	Quthing	Name Unknown	19034	2005	121.1	109.8	133.9
Lesotho	Quthing	Name Unknown	19034	2010	95.2	85.6	106.0
Lesotho	Quthing	Name Unknown	19034	2015	74.7	65.3	84.4
Lesotho	Quthing	Name Unknown	19004	2000	101.7	90.2	115.5
Lesotho	Quthing	Name Unknown	19006	2000	108.6	98.2	120.6
Lesotho	Quthing	Name Unknown	19009	2000	97.7	86.9	111.5
Lesotho	Quthing	Name Unknown	19012	2000	113.8	102.7	125.5
Lesotho	Quthing	Name Unknown	19021	2000	101.9	92.4	112.7
Lesotho	Quthing	Name Unknown	19031	2000	126.9	113.5	143.5
Lesotho	Quthing	Name Unknown	19033	2000	107.4	96.7	119.4
Lesotho	Quthing	Name Unknown	19035	2000	105.7	95.7	117.3
Lesotho	Quthing	Name Unknown	19036	2000	99.2	88.7	112.0
Lesotho	Quthing	Name Unknown	19004	2005	115.9	102.9	131.4
Lesotho	Quthing	Name Unknown	19006	2005	119.7	108.6	132.7
Lesotho	Quthing	Name Unknown	19009	2005	107.9	95.8	122.5
Lesotho	Quthing	Name Unknown	19012	2005	121.9	110.5	135.0
Lesotho	Quthing	Name Unknown	19021	2005	117.1	106.5	129.5
Lesotho	Quthing	Name Unknown	19031	2005	135.6	121.1	151.8
Lesotho	Quthing	Name Unknown	19033	2005	121.1	109.1	135.1
Lesotho	Quthing	Name Unknown	19035	2005	118.8	107.7	131.6
Lesotho	Quthing	Name Unknown	19036	2005	112.7	100.6	125.8
Lesotho	Quthing	Name Unknown	19004	2010	92.1	81.8	105.5
Lesotho	Quthing	Name Unknown	19006	2010	93.7	83.9	105.5
Lesotho	Quthing	Name Unknown	19009	2010	88.4	78.4	101.5
Lesotho	Quthing	Name Unknown	19012	2010	98.7	88.3	110.4
Lesotho	Quthing	Name Unknown	19021	2010	93.2	83.4	104.9
Lesotho	Quthing	Name Unknown	19031	2010	110.4	98.9	126.0
Lesotho	Quthing	Name Unknown	19033	2010	97.4	86.6	109.5
Lesotho	Quthing	Name Unknown	19035	2010	93.8	84.3	105.2

Admin 0	Admin 1	Admin 2	GAUL Code	Year	Under-5 mortality (per 1,000 livebirths)		
					Estimate	Lower bound, 95% UI	Upper bound, 95% UI
Lesotho	Quthing	Name Unknown	19036	2010	90.7	81.0	103.3
Lesotho	Quthing	Name Unknown	19004	2015	71.9	62.5	84.1
Lesotho	Quthing	Name Unknown	19006	2015	73.8	64.5	83.4
Lesotho	Quthing	Name Unknown	19009	2015	68.8	59.8	80.5
Lesotho	Quthing	Name Unknown	19012	2015	74.6	64.6	84.4
Lesotho	Quthing	Name Unknown	19021	2015	72.8	63.6	82.4
Lesotho	Quthing	Name Unknown	19031	2015	79.0	68.8	91.4
Lesotho	Quthing	Name Unknown	19033	2015	75.4	65.0	85.6
Lesotho	Quthing	Name Unknown	19035	2015	73.2	63.9	82.6
Lesotho	Quthing	Name Unknown	19036	2015	70.8	61.6	82.4
Lesotho	Quthing	Qhoali (67)	19039	2000	104.3	95.1	114.3
Lesotho	Quthing	Qhoali (67)	19039	2005	119.0	108.8	130.9
Lesotho	Quthing	Qhoali (67)	19039	2010	94.2	85.1	104.7
Lesotho	Quthing	Qhoali (67)	19039	2015	74.2	64.7	83.7
Lesotho	Quthing	Sebapala (64)	19040	2000	106.0	95.8	117.8
Lesotho	Quthing	Sebapala (64)	19040	2005	118.6	107.1	131.5
Lesotho	Quthing	Sebapala (64)	19040	2010	93.4	83.6	104.9
Lesotho	Quthing	Sebapala (64)	19040	2015	73.3	64.2	83.6
Lesotho	Quthing	Tele (62)	19041	2000	106.1	94.3	121.0
Lesotho	Quthing	Tele (62)	19041	2005	115.4	102.2	129.6
Lesotho	Quthing	Tele (62)	19041	2010	92.2	80.8	105.2
Lesotho	Quthing	Tele (62)	19041	2015	70.4	60.6	81.9
Lesotho	Quthing	Tsatsane (65)	19042	2000	108.0	95.2	121.2
Lesotho	Quthing	Tsatsane (65)	19042	2005	116.5	103.2	130.9
Lesotho	Quthing	Tsatsane (65)	19042	2010	93.8	82.4	106.8
Lesotho	Quthing	Tsatsane (65)	19042	2015	71.5	61.2	82.5
Lesotho	Thaba Tseka	Bobete	19043	2000	105.3	96.8	114.1
Lesotho	Thaba Tseka	Bobete	19043	2005	110.3	101.1	120.0
Lesotho	Thaba Tseka	Bobete	19043	2010	89.6	81.5	98.8
Lesotho	Thaba Tseka	Bobete	19043	2015	69.5	61.1	78.3
Lesotho	Thaba Tseka	Bokhoasa	19044	2000	104.8	95.5	115.0
Lesotho	Thaba Tseka	Bokhoasa	19044	2005	109.2	99.6	119.1
Lesotho	Thaba Tseka	Bokhoasa	19044	2010	88.3	79.5	97.9
Lesotho	Thaba Tseka	Bokhoasa	19044	2015	68.8	60.7	77.6
Lesotho	Thaba Tseka	Bokong	19045	2000	108.2	99.5	117.7
Lesotho	Thaba Tseka	Bokong	19045	2005	115.1	106.5	125.0
Lesotho	Thaba Tseka	Bokong	19045	2010	93.8	85.4	103.3
Lesotho	Thaba Tseka	Bokong	19045	2015	74.3	66.1	83.2
Lesotho	Thaba Tseka	C P A	19046	2000	105.6	94.7	117.3
Lesotho	Thaba Tseka	C P A	19046	2005	108.8	98.1	120.6
Lesotho	Thaba Tseka	C P A	19046	2010	87.9	78.3	98.6
Lesotho	Thaba Tseka	C P A	19046	2015	67.0	58.2	76.9
Lesotho	Thaba Tseka	Khohlontso	19047	2000	110.8	101.9	121.1
Lesotho	Thaba Tseka	Khohlontso	19047	2005	117.0	108.0	126.9
Lesotho	Thaba Tseka	Khohlontso	19047	2010	94.6	85.8	104.7
Lesotho	Thaba Tseka	Khohlontso	19047	2015	72.9	64.9	82.0
Lesotho	Thaba Tseka	Khutlo-Semetsi	19048	2000	109.3	99.7	119.8
Lesotho	Thaba Tseka	Khutlo-Semetsi	19048	2005	117.8	108.2	128.5
Lesotho	Thaba Tseka	Khutlo-Semetsi	19048	2010	95.1	85.5	105.3
Lesotho	Thaba Tseka	Khutlo-Semetsi	19048	2015	73.9	65.4	83.3
Lesotho	Thaba Tseka	Lesobeng	19049	2000	106.3	97.6	115.9
Lesotho	Thaba Tseka	Lesobeng	19049	2005	114.3	105.2	123.9
Lesotho	Thaba Tseka	Lesobeng	19049	2010	92.0	83.7	101.3
Lesotho	Thaba Tseka	Lesobeng	19049	2015	73.0	64.9	82.3
Lesotho	Thaba Tseka	Linakeng	19050	2000	103.9	94.9	113.9
Lesotho	Thaba Tseka	Linakeng	19050	2005	108.4	99.3	117.7
Lesotho	Thaba Tseka	Linakeng	19050	2010	88.1	79.6	97.8
Lesotho	Thaba Tseka	Linakeng	19050	2015	68.7	60.5	77.7
Lesotho	Thaba Tseka	Maboloka	19051	2000	110.9	101.5	121.2
Lesotho	Thaba Tseka	Maboloka	19051	2005	116.3	106.7	127.2
Lesotho	Thaba Tseka	Maboloka	19051	2010	93.7	84.7	104.4
Lesotho	Thaba Tseka	Maboloka	19051	2015	71.9	63.8	81.5
Lesotho	Thaba Tseka	Mahlong	19052	2000	108.2	98.5	118.5
Lesotho	Thaba Tseka	Mahlong	19052	2005	117.3	107.7	128.0
Lesotho	Thaba Tseka	Mahlong	19052	2010	94.7	86.0	104.5
Lesotho	Thaba Tseka	Mahlong	19052	2015	72.3	64.0	81.6
Lesotho	Thaba Tseka	Makopoi	19053	2000	108.4	98.9	118.7
Lesotho	Thaba Tseka	Makopoi	19053	2005	117.2	107.4	128.5
Lesotho	Thaba Tseka	Makopoi	19053	2010	94.7	86.0	104.7
Lesotho	Thaba Tseka	Makopoi	19053	2015	71.3	63.3	80.4
Lesotho	Thaba Tseka	Manamaneng	19054	2000	107.1	96.8	118.2
Lesotho	Thaba Tseka	Manamaneng	19054	2005	110.4	100.1	122.0
Lesotho	Thaba Tseka	Manamaneng	19054	2010	89.2	79.6	99.3
Lesotho	Thaba Tseka	Manamaneng	19054	2015	69.7	60.9	79.3
Lesotho	Thaba Tseka	Mantsonyane	19055	2000	110.2	100.5	120.9
Lesotho	Thaba Tseka	Mantsonyane	19055	2005	116.8	107.6	127.7
Lesotho	Thaba Tseka	Mantsonyane	19055	2010	94.7	86.1	105.2
Lesotho	Thaba Tseka	Mantsonyane	19055	2015	70.9	62.8	80.1
Lesotho	Thaba Tseka	Mashai	19056	2000	106.4	96.4	117.6
Lesotho	Thaba Tseka	Mashai	19056	2005	109.7	99.4	120.7
Lesotho	Thaba Tseka	Mashai	19056	2010	88.8	79.7	99.0
Lesotho	Thaba Tseka	Mashai	19056	2015	69.0	60.5	78.5
Lesotho	Thaba Tseka	Methalaneng	19057	2000	108.3	99.1	118.8
Lesotho	Thaba Tseka	Methalaneng	19057	2005	117.4	107.8	127.9
Lesotho	Thaba Tseka	Methalaneng	19057	2010	94.6	85.6	104.4

Admin 0	Admin 1	Admin 2	GAUL Code	Year	Under-5 mortality (per 1,000 livebirths)		
					Estimate	Lower bound, 95% UI	Upper bound, 95% UI
Lesotho	Thaba Tseka	Methalaneng	19057	2015	73.4	65.1	82.8
Lesotho	Thaba Tseka	Mohlanapeng	19058	2000	106.4	97.4	115.8
Lesotho	Thaba Tseka	Mohlanapeng	19058	2005	112.0	102.3	122.2
Lesotho	Thaba Tseka	Mohlanapeng	19058	2010	90.7	82.2	100.4
Lesotho	Thaba Tseka	Mohlanapeng	19058	2015	69.5	61.0	78.4
Lesotho	Thaba Tseka	SEHONGHONG	19059	2000	105.6	95.7	116.1
Lesotho	Thaba Tseka	SEHONGHONG	19059	2005	110.2	100.5	120.3
Lesotho	Thaba Tseka	SEHONGHONG	19059	2010	89.1	79.9	99.1
Lesotho	Thaba Tseka	SEHONGHONG	19059	2015	68.2	59.8	77.8
Lesotho	Thaba Tseka	Semenanyane	19060	2000	109.1	99.5	119.6
Lesotho	Thaba Tseka	Semenanyane	19060	2005	113.0	103.5	123.9
Lesotho	Thaba Tseka	Semenanyane	19060	2010	91.6	82.7	101.4
Lesotho	Thaba Tseka	Semenanyane	19060	2015	73.7	64.7	84.4
Lesotho	Thaba Tseka	Suoane	19061	2000	113.0	104.1	123.3
Lesotho	Thaba Tseka	Suoane	19061	2005	117.1	108.2	127.0
Lesotho	Thaba Tseka	Suoane	19061	2010	95.2	87.1	104.9
Lesotho	Thaba Tseka	Suoane	19061	2015	75.2	67.0	84.6
Lesotho	Thaba Tseka	Takalatsa	19062	2000	112.9	101.9	124.6
Lesotho	Thaba Tseka	Takalatsa	19062	2005	118.9	107.9	131.6
Lesotho	Thaba Tseka	Takalatsa	19062	2010	95.7	85.7	107.3
Lesotho	Thaba Tseka	Takalatsa	19062	2015	76.9	67.8	87.7
Lesotho	Thaba Tseka	Tsoelike	19063	2000	112.6	103.5	123.0
Lesotho	Thaba Tseka	Tsoelike	19063	2005	118.0	108.7	128.3
Lesotho	Thaba Tseka	Tsoelike	19063	2010	95.7	86.9	106.0
Lesotho	Thaba Tseka	Tsoelike	19063	2015	73.8	65.8	82.8
Liberia	Bomi	Dowein	147291	2000	216.5	202.6	229.7
Liberia	Bomi	Dowein	147291	2005	154.1	144.4	165.1
Liberia	Bomi	Dowein	147291	2010	115.3	106.9	124.2
Liberia	Bomi	Dowein	147291	2015	90.2	79.9	100.9
Liberia	Bomi	Klay	147220	2000	215.4	202.2	228.6
Liberia	Bomi	Klay	147220	2005	151.8	142.1	161.8
Liberia	Bomi	Klay	147220	2010	114.4	106.3	122.5
Liberia	Bomi	Klay	147220	2015	90.2	80.4	100.5
Liberia	Bomi	Senjeh	147256	2000	207.9	194.7	221.1
Liberia	Bomi	Senjeh	147256	2005	141.0	131.9	150.1
Liberia	Bomi	Senjeh	147256	2010	108.8	100.5	118.0
Liberia	Bomi	Senjeh	147256	2015	86.1	76.8	96.1
Liberia	Bomi	Suehn Mecca	147260	2000	203.5	192.2	214.4
Liberia	Bomi	Suehn Mecca	147260	2005	142.6	135.1	150.8
Liberia	Bomi	Suehn Mecca	147260	2010	107.4	100.2	114.4
Liberia	Bomi	Suehn Mecca	147260	2015	84.1	75.6	93.5
Liberia	Bong	Boinsen	147163	2000	177.3	161.5	193.3
Liberia	Bong	Boinsen	147163	2005	127.1	116.2	138.5
Liberia	Bong	Boinsen	147163	2010	96.0	87.2	104.6
Liberia	Bong	Boinsen	147163	2015	72.1	63.3	81.6
Liberia	Bong	Fuamah	147192	2000	194.4	179.4	207.9
Liberia	Bong	Fuamah	147192	2005	129.1	119.3	139.2
Liberia	Bong	Fuamah	147192	2010	99.6	91.5	108.0
Liberia	Bong	Fuamah	147192	2015	79.2	70.1	88.9
Liberia	Bong	Jorquelleh	147215	2000	167.5	155.0	180.8
Liberia	Bong	Jorquelleh	147215	2005	116.3	107.8	125.2
Liberia	Bong	Jorquelleh	147215	2010	89.3	82.0	97.3
Liberia	Bong	Jorquelleh	147215	2015	66.9	59.2	75.6
Liberia	Bong	Kokoyah	147221	2000	192.7	175.4	210.7
Liberia	Bong	Kokoyah	147221	2005	137.1	124.6	149.9
Liberia	Bong	Kokoyah	147221	2010	103.9	93.6	114.1
Liberia	Bong	Kokoyah	147221	2015	80.6	70.3	91.9
Liberia	Bong	Kpaai	147224	2000	165.2	151.5	178.4
Liberia	Bong	Kpaai	147224	2005	117.4	108.3	127.1
Liberia	Bong	Kpaai	147224	2010	90.4	82.4	99.0
Liberia	Bong	Kpaai	147224	2015	66.5	58.1	75.8
Liberia	Bong	Panta	147241	2000	152.4	140.8	165.3
Liberia	Bong	Panta	147241	2005	108.3	99.0	118.1
Liberia	Bong	Panta	147241	2010	84.0	76.0	92.3
Liberia	Bong	Panta	147241	2015	62.9	55.3	71.2
Liberia	Bong	Salala	147248	2000	185.6	173.2	199.0
Liberia	Bong	Salala	147248	2005	123.7	115.1	132.6
Liberia	Bong	Salala	147248	2010	95.5	88.1	103.0
Liberia	Bong	Salala	147248	2015	77.9	69.1	87.0
Liberia	Bong	Sanoyeah	147251	2000	182.9	169.6	196.4
Liberia	Bong	Sanoyeah	147251	2005	125.8	116.8	135.3
Liberia	Bong	Sanoyeah	147251	2010	96.9	88.9	105.3
Liberia	Bong	Sanoyeah	147251	2015	73.9	65.7	83.2
Liberia	Bong	Suakoko	147259	2000	171.4	159.8	184.1
Liberia	Bong	Suakoko	147259	2005	118.3	109.9	127.2
Liberia	Bong	Suakoko	147259	2010	91.8	84.1	99.6
Liberia	Bong	Suakoko	147259	2015	68.1	60.5	76.7
Liberia	Bong	Tukpahblee	147265	2000	184.2	167.4	200.5
Liberia	Bong	Tukpahblee	147265	2005	131.7	120.0	144.2
Liberia	Bong	Tukpahblee	147265	2010	100.1	90.1	110.3
Liberia	Bong	Tukpahblee	147265	2015	76.5	67.0	87.2
Liberia	Bong	Yeallequelleh	147278	2000	183.5	169.9	198.5
Liberia	Bong	Yeallequelleh	147278	2005	127.9	117.9	138.3
Liberia	Bong	Yeallequelleh	147278	2010	99.4	90.6	107.9
Liberia	Bong	Yeallequelleh	147278	2015	75.9	67.1	85.4

Admin 0	Admin 1	Admin 2	GAUL Code	Year	Under-5 mortality (per 1,000 livebirths)		
					Estimate	Lower bound, 95% UI	Upper bound, 95% UI
Liberia	Bong	Zota	147281	2000	159.5	147.8	172.3
Liberia	Bong	Zota	147281	2005	111.7	102.9	120.3
Liberia	Bong	Zota	147281	2010	86.4	78.4	94.3
Liberia	Bong	Zota	147281	2015	62.0	54.5	70.2
Liberia	Gbarpolu	Belleh	147159	2000	180.0	164.5	197.5
Liberia	Gbarpolu	Belleh	147159	2005	125.7	114.9	137.8
Liberia	Gbarpolu	Belleh	147159	2010	95.0	86.0	105.3
Liberia	Gbarpolu	Belleh	147159	2015	69.3	60.8	79.5
Liberia	Gbarpolu	Bokomu	147164	2000	188.7	174.0	203.8
Liberia	Gbarpolu	Bokomu	147164	2005	128.0	118.7	138.1
Liberia	Gbarpolu	Bokomu	147164	2010	97.9	90.1	107.1
Liberia	Gbarpolu	Bokomu	147164	2015	72.0	63.9	81.2
Liberia	Gbarpolu	Bopolu	147167	2000	198.9	185.2	212.7
Liberia	Gbarpolu	Bopolu	147167	2005	135.2	126.1	144.9
Liberia	Gbarpolu	Bopolu	147167	2010	104.6	96.5	112.7
Liberia	Gbarpolu	Bopolu	147167	2015	80.8	72.1	90.4
Liberia	Gbarpolu	Gbarma	147197	2000	212.9	199.3	228.0
Liberia	Gbarpolu	Gbarma	147197	2005	146.4	136.6	156.2
Liberia	Gbarpolu	Gbarma	147197	2010	113.5	105.0	122.3
Liberia	Gbarpolu	Gbarma	147197	2015	90.8	81.1	101.3
Liberia	Gbarpolu	Gounwolaila	147208	2000	176.9	162.5	191.9
Liberia	Gbarpolu	Gounwolaila	147208	2005	120.9	111.6	131.3
Liberia	Gbarpolu	Gounwolaila	147208	2010	93.3	85.3	102.4
Liberia	Gbarpolu	Gounwolaila	147208	2015	68.4	60.3	77.1
Liberia	Gbarpolu	Kongba	147282	2000	219.1	201.8	236.6
Liberia	Gbarpolu	Kongba	147282	2005	160.1	148.7	172.8
Liberia	Gbarpolu	Kongba	147282	2010	122.8	112.3	133.5
Liberia	Gbarpolu	Kongba	147282	2015	94.7	83.3	106.8
Liberia	Grand Bassa	Commonwealth B	147175	2000	172.9	157.0	188.4
Liberia	Grand Bassa	Commonwealth B	147175	2005	121.2	110.5	132.6
Liberia	Grand Bassa	Commonwealth B	147175	2010	91.4	81.6	101.0
Liberia	Grand Bassa	Commonwealth B	147175	2015	73.3	63.5	84.1
Liberia	Grand Bassa	District No. 1	147177	2000	202.1	188.8	214.6
Liberia	Grand Bassa	District No. 1	147177	2005	140.0	131.1	149.1
Liberia	Grand Bassa	District No. 1	147177	2010	106.0	98.1	114.3
Liberia	Grand Bassa	District No. 1	147177	2015	86.8	77.7	97.1
Liberia	Grand Bassa	District No. 2	147178	2000	196.0	182.2	209.5
Liberia	Grand Bassa	District No. 2	147178	2005	138.1	128.6	148.3
Liberia	Grand Bassa	District No. 2	147178	2010	105.7	97.3	114.4
Liberia	Grand Bassa	District No. 2	147178	2015	84.3	74.7	94.7
Liberia	Grand Bassa	District No. 3	147179	2000	193.3	178.9	208.7
Liberia	Grand Bassa	District No. 3	147179	2005	138.7	128.7	150.1
Liberia	Grand Bassa	District No. 3	147179	2010	104.2	95.8	113.2
Liberia	Grand Bassa	District No. 3	147179	2015	82.5	73.3	92.9
Liberia	Grand Bassa	District No. 4	147180	2000	187.9	173.3	202.6
Liberia	Grand Bassa	District No. 4	147180	2005	135.8	126.0	146.5
Liberia	Grand Bassa	District No. 4	147180	2010	103.5	94.2	112.5
Liberia	Grand Bassa	District No. 4	147180	2015	85.8	75.4	97.5
Liberia	Grand Bassa	Neekreen	147234	2000	186.6	171.9	201.6
Liberia	Grand Bassa	Neekreen	147234	2005	131.4	121.3	142.9
Liberia	Grand Bassa	Neekreen	147234	2010	100.3	90.6	109.8
Liberia	Grand Bassa	Neekreen	147234	2015	81.9	71.6	93.2
Liberia	Grand Bassa	Owensgrove	147240	2000	190.9	175.5	207.0
Liberia	Grand Bassa	Owensgrove	147240	2005	132.1	122.1	142.4
Liberia	Grand Bassa	Owensgrove	147240	2010	97.9	89.4	107.5
Liberia	Grand Bassa	Owensgrove	147240	2015	79.0	70.0	89.1
Liberia	Grand Bassa	St. John River City	147257	2000	199.5	184.5	215.0
Liberia	Grand Bassa	St. John River City	147257	2005	141.3	130.9	152.7
Liberia	Grand Bassa	St. John River City	147257	2010	108.5	99.0	119.0
Liberia	Grand Bassa	St. John River City	147257	2015	89.4	78.7	101.3
Liberia	Grand Cape Mount	Commonwealth C	147176	2000	221.3	204.3	240.1
Liberia	Grand Cape Mount	Commonwealth C	147176	2005	155.6	143.5	169.2
Liberia	Grand Cape Mount	Commonwealth C	147176	2010	123.3	112.4	135.3
Liberia	Grand Cape Mount	Commonwealth C	147176	2015	97.0	85.8	109.5
Liberia	Grand Cape Mount	Garwula	147195	2000	231.3	218.0	245.5
Liberia	Grand Cape Mount	Garwula	147195	2005	164.4	154.3	175.2
Liberia	Grand Cape Mount	Garwula	147195	2010	127.6	118.2	137.7
Liberia	Grand Cape Mount	Garwula	147195	2015	102.2	91.5	113.8
Liberia	Grand Cape Mount	Golakonneh	147207	2000	233.1	220.6	247.4
Liberia	Grand Cape Mount	Golakonneh	147207	2005	165.8	156.4	175.4
Liberia	Grand Cape Mount	Golakonneh	147207	2010	129.3	120.6	138.6
Liberia	Grand Cape Mount	Golakonneh	147207	2015	102.9	92.7	115.0
Liberia	Grand Cape Mount	Porkpa	147244	2000	258.6	242.7	275.2
Liberia	Grand Cape Mount	Porkpa	147244	2005	190.8	179.2	202.7
Liberia	Grand Cape Mount	Porkpa	147244	2010	148.3	137.1	160.3
Liberia	Grand Cape Mount	Porkpa	147244	2015	120.0	107.9	134.5
Liberia	Grand Cape Mount	Tewor	147262	2000	258.2	242.3	275.8
Liberia	Grand Cape Mount	Tewor	147262	2005	189.9	176.8	203.2
Liberia	Grand Cape Mount	Tewor	147262	2010	146.8	135.5	159.4
Liberia	Grand Cape Mount	Tewor	147262	2015	119.5	106.5	134.4
Liberia	Grand Gedeh	B'hai	147156	2000	167.0	152.5	182.3
Liberia	Grand Gedeh	B'hai	147156	2005	124.1	112.8	134.9
Liberia	Grand Gedeh	B'hai	147156	2010	90.3	81.7	99.7
Liberia	Grand Gedeh	B'hai	147156	2015	65.9	57.5	75.0
Liberia	Grand Gedeh	Cavala	147171	2000	166.2	152.6	180.3

Admin 0	Admin 1	Admin 2	GAUL Code	Year	Under-5 mortality (per 1,000 livebirths)		
					Estimate	Lower bound, 95% UI	Upper bound, 95% UI
Liberia	Grand Gedeh	Cavala	147171	2005	127.8	117.2	138.9
Liberia	Grand Gedeh	Cavala	147171	2010	93.7	84.5	103.0
Liberia	Grand Gedeh	Cavala	147171	2015	70.0	61.2	80.0
Liberia	Grand Gedeh	Gbao	147196	2000	164.4	151.2	178.9
Liberia	Grand Gedeh	Gbao	147196	2005	125.5	115.6	136.4
Liberia	Grand Gedeh	Gbao	147196	2010	93.0	84.0	102.2
Liberia	Grand Gedeh	Gbao	147196	2015	71.0	62.1	80.7
Liberia	Grand Gedeh	Gboe-Ploe	147201	2000	164.5	150.7	181.0
Liberia	Grand Gedeh	Gboe-Ploe	147201	2005	123.9	112.9	136.3
Liberia	Grand Gedeh	Gboe-Ploe	147201	2010	92.6	83.3	102.8
Liberia	Grand Gedeh	Gboe-Ploe	147201	2015	73.0	63.7	83.4
Liberia	Grand Gedeh	Glio-Twarbo	147206	2000	144.1	128.2	159.9
Liberia	Grand Gedeh	Glio-Twarbo	147206	2005	116.5	104.7	129.2
Liberia	Grand Gedeh	Glio-Twarbo	147206	2010	86.4	76.9	97.0
Liberia	Grand Gedeh	Glio-Twarbo	147206	2015	68.4	59.5	78.8
Liberia	Grand Gedeh	Konobo	147223	2000	153.4	140.5	167.6
Liberia	Grand Gedeh	Konobo	147223	2005	122.6	112.1	134.2
Liberia	Grand Gedeh	Konobo	147223	2010	90.5	81.5	100.4
Liberia	Grand Gedeh	Konobo	147223	2015	71.3	62.4	81.8
Liberia	Grand Gedeh	Putu	147245	2000	157.1	143.2	173.3
Liberia	Grand Gedeh	Putu	147245	2005	124.2	113.5	135.9
Liberia	Grand Gedeh	Putu	147245	2010	93.0	84.0	103.0
Liberia	Grand Gedeh	Putu	147245	2015	74.8	65.3	85.6
Liberia	Grand Gedeh	Tchien	147261	2000	155.4	142.6	169.1
Liberia	Grand Gedeh	Tchien	147261	2005	116.2	106.2	126.5
Liberia	Grand Gedeh	Tchien	147261	2010	84.5	76.2	93.3
Liberia	Grand Gedeh	Tchien	147261	2015	64.0	55.8	73.1
Liberia	Grand Kru	Barclayville	147157	2000	149.2	136.0	163.0
Liberia	Grand Kru	Barclayville	147157	2005	118.3	108.5	128.4
Liberia	Grand Kru	Barclayville	147157	2010	93.6	84.7	102.8
Liberia	Grand Kru	Barclayville	147157	2015	78.5	68.6	90.1
Liberia	Grand Kru	Bleebo	147160	2000	149.7	138.1	162.4
Liberia	Grand Kru	Bleebo	147160	2005	121.5	112.9	130.7
Liberia	Grand Kru	Bleebo	147160	2010	92.9	84.8	101.6
Liberia	Grand Kru	Bleebo	147160	2015	76.6	67.7	87.4
Liberia	Grand Kru	Bolloh	147166	2000	160.4	145.6	175.1
Liberia	Grand Kru	Bolloh	147166	2005	127.3	116.6	138.0
Liberia	Grand Kru	Bolloh	147166	2010	100.4	90.8	110.5
Liberia	Grand Kru	Bolloh	147166	2015	84.4	73.7	96.6
Liberia	Grand Kru	Buah	147168	2000	152.5	140.9	164.4
Liberia	Grand Kru	Buah	147168	2005	121.7	113.4	130.9
Liberia	Grand Kru	Buah	147168	2010	95.2	87.3	103.6
Liberia	Grand Kru	Buah	147168	2015	79.9	70.2	91.3
Liberia	Grand Kru	Dorbor	147183	2000	158.7	145.6	172.2
Liberia	Grand Kru	Dorbor	147183	2005	126.2	116.6	135.9
Liberia	Grand Kru	Dorbor	147183	2010	98.5	89.5	107.0
Liberia	Grand Kru	Dorbor	147183	2015	83.8	73.6	95.8
Liberia	Grand Kru	Dweh	147185	2000	159.5	147.0	172.1
Liberia	Grand Kru	Dweh	147185	2005	126.0	118.0	135.9
Liberia	Grand Kru	Dweh	147185	2010	96.9	88.9	105.4
Liberia	Grand Kru	Dweh	147185	2015	82.8	72.8	94.1
Liberia	Grand Kru	Felo-Jekwi	147186	2000	150.4	136.3	164.6
Liberia	Grand Kru	Felo-Jekwi	147186	2005	118.6	108.4	129.2
Liberia	Grand Kru	Felo-Jekwi	147186	2010	93.7	84.3	104.0
Liberia	Grand Kru	Felo-Jekwi	147186	2015	78.5	68.3	89.8
Liberia	Grand Kru	Fenetoe	147188	2000	157.1	142.3	171.3
Liberia	Grand Kru	Fenetoe	147188	2005	125.0	114.3	135.7
Liberia	Grand Kru	Fenetoe	147188	2010	97.0	87.7	107.4
Liberia	Grand Kru	Fenetoe	147188	2015	80.9	70.8	93.2
Liberia	Grand Kru	Forpoh	147190	2000	164.4	149.7	178.7
Liberia	Grand Kru	Forpoh	147190	2005	128.6	118.1	139.3
Liberia	Grand Kru	Forpoh	147190	2010	100.5	91.7	110.4
Liberia	Grand Kru	Forpoh	147190	2015	85.9	75.1	98.1
Liberia	Grand Kru	Garraway	147194	2000	143.8	131.7	156.8
Liberia	Grand Kru	Garraway	147194	2005	116.3	107.2	125.9
Liberia	Grand Kru	Garraway	147194	2010	89.6	81.3	98.6
Liberia	Grand Kru	Garraway	147194	2015	74.8	65.3	85.5
Liberia	Grand Kru	Gee	147203	2000	155.0	143.1	167.1
Liberia	Grand Kru	Gee	147203	2005	124.5	116.2	133.5
Liberia	Grand Kru	Gee	147203	2010	95.1	87.3	103.4
Liberia	Grand Kru	Gee	147203	2015	78.9	69.5	89.7
Liberia	Grand Kru	Grand Cess Wedabo	147209	2000	146.6	133.5	160.6
Liberia	Grand Kru	Grand Cess Wedabo	147209	2005	116.7	107.0	127.0
Liberia	Grand Kru	Grand Cess Wedabo	147209	2010	91.2	82.5	100.3
Liberia	Grand Kru	Grand Cess Wedabo	147209	2015	75.7	66.2	86.6
Liberia	Grand Kru	Kpi	147227	2000	156.5	144.7	168.5
Liberia	Grand Kru	Kpi	147227	2005	125.4	117.2	134.2
Liberia	Grand Kru	Kpi	147227	2010	96.7	88.9	105.0
Liberia	Grand Kru	Kpi	147227	2015	81.7	72.1	92.9
Liberia	Grand Kru	Lower Jloh	147230	2000	159.4	144.4	174.8
Liberia	Grand Kru	Lower Jloh	147230	2005	127.3	116.0	139.2
Liberia	Grand Kru	Lower Jloh	147230	2010	101.8	91.5	113.3
Liberia	Grand Kru	Lower Jloh	147230	2015	85.5	74.9	97.9
Liberia	Grand Kru	Nrokwia-Wesldow	147236	2000	154.5	140.6	168.0
Liberia	Grand Kru	Nrokwia-Wesldow	147236	2005	123.2	113.1	133.4

Admin 0	Admin 1	Admin 2	GAUL Code	Year	Under-5 mortality (per 1,000 livebirths)		
					Estimate	Lower bound, 95% UI	Upper bound, 95% UI
Liberia	Grand Kru	Nrokwia-Wesldow	147236	2010	96.6	87.3	106.2
Liberia	Grand Kru	Nrokwia-Wesldow	147236	2015	80.6	70.5	92.7
Liberia	Grand Kru	Trenbo	147264	2000	145.4	133.8	157.9
Liberia	Grand Kru	Trenbo	147264	2005	117.3	108.6	126.4
Liberia	Grand Kru	Trenbo	147264	2010	90.4	82.3	98.7
Liberia	Grand Kru	Trenbo	147264	2015	75.8	66.7	86.3
Liberia	Grand Kru	Upper Jloh	147268	2000	154.5	139.8	169.2
Liberia	Grand Kru	Upper Jloh	147268	2005	122.4	111.6	133.7
Liberia	Grand Kru	Upper Jloh	147268	2010	96.7	86.7	107.7
Liberia	Grand Kru	Upper Jloh	147268	2015	81.4	71.0	93.9
Liberia	Grand Kru	Wlogba	147274	2000	162.4	149.3	175.3
Liberia	Grand Kru	Wlogba	147274	2005	127.5	118.5	137.1
Liberia	Grand Kru	Wlogba	147274	2010	99.5	91.1	108.3
Liberia	Grand Kru	Wlogba	147274	2015	86.3	75.8	98.3
Liberia	Lofa	Foya	147191	2000	188.3	175.0	203.0
Liberia	Lofa	Foya	147191	2005	136.0	126.8	146.1
Liberia	Lofa	Foya	147191	2010	103.6	95.0	112.8
Liberia	Lofa	Foya	147191	2015	84.5	75.2	94.5
Liberia	Lofa	Kolahun	147222	2000	190.6	176.4	205.7
Liberia	Lofa	Kolahun	147222	2005	134.9	125.2	145.1
Liberia	Lofa	Kolahun	147222	2010	101.0	92.7	110.3
Liberia	Lofa	Kolahun	147222	2015	77.7	69.0	86.9
Liberia	Lofa	Quardu Boundi	147247	2000	180.0	161.0	199.1
Liberia	Lofa	Quardu Boundi	147247	2005	125.5	112.9	139.9
Liberia	Lofa	Quardu Boundi	147247	2010	90.1	79.9	100.8
Liberia	Lofa	Quardu Boundi	147247	2015	70.6	61.7	80.3
Liberia	Lofa	Salayea	147249	2000	159.1	146.8	173.0
Liberia	Lofa	Salayea	147249	2005	111.5	102.0	121.6
Liberia	Lofa	Salayea	147249	2010	85.2	77.2	93.7
Liberia	Lofa	Salayea	147249	2015	61.3	53.8	69.4
Liberia	Lofa	Vahun	147269	2000	218.6	202.2	236.3
Liberia	Lofa	Vahun	147269	2005	163.7	151.8	176.4
Liberia	Lofa	Vahun	147269	2010	126.6	116.2	137.9
Liberia	Lofa	Vahun	147269	2015	99.0	87.3	111.5
Liberia	Lofa	Voinjama	147270	2000	182.1	166.2	198.2
Liberia	Lofa	Voinjama	147270	2005	125.1	115.2	136.3
Liberia	Lofa	Voinjama	147270	2010	91.9	83.4	101.5
Liberia	Lofa	Voinjama	147270	2015	71.2	62.9	80.3
Liberia	Lofa	Zorzor	147280	2000	170.0	156.4	185.3
Liberia	Lofa	Zorzor	147280	2005	118.1	108.3	129.3
Liberia	Lofa	Zorzor	147280	2010	88.2	79.6	96.6
Liberia	Lofa	Zorzor	147280	2015	64.2	56.4	72.5
Liberia	Margibi	Firestone	147189	2000	181.2	170.0	191.9
Liberia	Margibi	Firestone	147189	2005	123.9	116.4	131.8
Liberia	Margibi	Firestone	147189	2010	93.9	86.8	100.9
Liberia	Margibi	Firestone	147189	2015	74.4	66.8	82.8
Liberia	Margibi	Gibi	147204	2000	192.9	179.2	207.5
Liberia	Margibi	Gibi	147204	2005	129.4	120.4	138.8
Liberia	Margibi	Gibi	147204	2010	99.3	91.7	107.8
Liberia	Margibi	Gibi	147204	2015	81.2	72.1	91.0
Liberia	Margibi	Kakata	147217	2000	178.6	167.4	188.9
Liberia	Margibi	Kakata	147217	2005	118.2	110.7	126.1
Liberia	Margibi	Kakata	147217	2010	91.2	84.3	98.0
Liberia	Margibi	Kakata	147217	2015	72.0	64.0	80.0
Liberia	Margibi	Mambah Kaba	147231	2000	184.4	173.8	195.5
Liberia	Margibi	Mambah Kaba	147231	2005	127.4	120.2	135.2
Liberia	Margibi	Mambah Kaba	147231	2010	95.4	88.4	103.0
Liberia	Margibi	Mambah Kaba	147231	2015	73.8	65.7	82.9
Liberia	Maryland	Gwelekpoken	147211	2000	147.7	135.9	159.9
Liberia	Maryland	Gwelekpoken	147211	2005	120.0	111.6	129.6
Liberia	Maryland	Gwelekpoken	147211	2010	91.3	83.3	99.6
Liberia	Maryland	Gwelekpoken	147211	2015	74.1	65.0	84.0
Liberia	Maryland	Harper	147285	2000	125.5	113.9	139.2
Liberia	Maryland	Harper	147285	2005	102.8	93.6	112.7
Liberia	Maryland	Harper	147285	2010	77.9	69.5	86.2
Liberia	Maryland	Harper	147285	2015	62.0	53.5	71.3
Liberia	Maryland	Karluway No. 1	147218	2000	142.5	132.3	153.3
Liberia	Maryland	Karluway No. 1	147218	2005	115.8	107.8	124.9
Liberia	Maryland	Karluway No. 1	147218	2010	86.7	79.4	95.5
Liberia	Maryland	Karluway No. 1	147218	2015	70.4	61.7	80.3
Liberia	Maryland	Karluway No. 2	147219	2000	139.0	128.0	150.4
Liberia	Maryland	Karluway No. 2	147219	2005	112.9	104.9	122.3
Liberia	Maryland	Karluway No. 2	147219	2010	83.9	76.4	92.6
Liberia	Maryland	Karluway No. 2	147219	2015	69.5	60.5	79.4
Liberia	Maryland	Nyorken	147239	2000	147.6	134.9	159.6
Liberia	Maryland	Nyorken	147239	2005	120.8	111.9	131.0
Liberia	Maryland	Nyorken	147239	2010	90.7	82.5	99.7
Liberia	Maryland	Nyorken	147239	2015	73.6	64.4	83.8
Liberia	Maryland	Pleebo/Sodoken	147243	2000	132.7	121.9	144.3
Liberia	Maryland	Pleebo/Sodoken	147243	2005	108.6	100.3	118.6
Liberia	Maryland	Pleebo/Sodoken	147243	2010	80.5	72.6	88.9
Liberia	Maryland	Pleebo/Sodoken	147243	2015	65.9	57.2	75.6
Liberia	Maryland	Whojah	147273	2000	148.0	136.2	159.7
Liberia	Maryland	Whojah	147273	2005	120.2	112.2	129.6
Liberia	Maryland	Whojah	147273	2010	92.0	84.2	100.3

Admin 0	Admin 1	Admin 2	GAUL Code	Year	Under-5 mortality (per 1,000 livebirths)		
					Estimate	Lower bound, 95% UI	Upper bound, 95% UI
Liberia	Maryland	Whojah	147273	2015	75.7	66.5	85.7
Liberia	Montserrado	Careysburg	147170	2000	166.2	157.0	175.2
Liberia	Montserrado	Careysburg	147170	2005	119.1	112.9	126.2
Liberia	Montserrado	Careysburg	147170	2010	90.9	84.4	97.2
Liberia	Montserrado	Careysburg	147170	2015	69.8	62.4	78.1
Liberia	Montserrado	Commonwealth	147174	2000	167.6	158.5	177.0
Liberia	Montserrado	Commonwealth	147174	2005	120.9	114.0	127.7
Liberia	Montserrado	Commonwealth	147174	2010	91.0	84.7	97.8
Liberia	Montserrado	Commonwealth	147174	2015	69.4	61.7	77.6
Liberia	Montserrado	Greater Monrovia	147290	2000	156.5	147.4	166.1
Liberia	Montserrado	Greater Monrovia	147290	2005	113.1	106.2	119.8
Liberia	Montserrado	Greater Monrovia	147290	2010	86.0	79.8	93.0
Liberia	Montserrado	Greater Monrovia	147290	2015	63.4	56.2	71.3
Liberia	Montserrado	St. Paul River	147258	2000	172.1	162.9	180.7
Liberia	Montserrado	St. Paul River	147258	2005	124.8	118.1	132.0
Liberia	Montserrado	St. Paul River	147258	2010	94.3	87.7	100.6
Liberia	Montserrado	St. Paul River	147258	2015	70.8	62.7	79.0
Liberia	Montserrado	Todee	147263	2000	167.0	156.4	177.3
Liberia	Montserrado	Todee	147263	2005	116.1	109.0	123.8
Liberia	Montserrado	Todee	147263	2010	90.2	82.8	96.9
Liberia	Montserrado	Todee	147263	2015	69.2	61.7	76.8
Liberia	Nimba	Boe and Quilla	147162	2000	162.7	149.2	177.5
Liberia	Nimba	Boe and Quilla	147162	2005	116.3	106.9	126.7
Liberia	Nimba	Boe and Quilla	147162	2010	82.8	75.2	90.9
Liberia	Nimba	Boe and Quilla	147162	2015	62.3	54.7	70.7
Liberia	Nimba	Buu-Yao	147169	2000	161.5	147.0	176.9
Liberia	Nimba	Buu-Yao	147169	2005	115.8	105.6	127.3
Liberia	Nimba	Buu-Yao	147169	2010	82.2	73.8	91.0
Liberia	Nimba	Buu-Yao	147169	2015	63.4	54.8	72.6
Liberia	Nimba	Doe	147181	2000	166.1	151.2	181.8
Liberia	Nimba	Doe	147181	2005	119.4	109.2	129.5
Liberia	Nimba	Doe	147181	2010	85.0	77.2	93.5
Liberia	Nimba	Doe	147181	2015	64.0	55.8	72.2
Liberia	Nimba	Garr-Bain	147193	2000	133.2	122.0	144.8
Liberia	Nimba	Garr-Bain	147193	2005	94.0	86.4	102.5
Liberia	Nimba	Garr-Bain	147193	2010	71.1	64.0	77.9
Liberia	Nimba	Garr-Bain	147193	2015	54.2	47.6	61.2
Liberia	Nimba	Gbehlay-Geh	147199	2000	152.8	139.5	167.8
Liberia	Nimba	Gbehlay-Geh	147199	2005	108.9	99.7	119.9
Liberia	Nimba	Gbehlay-Geh	147199	2010	79.1	70.6	87.5
Liberia	Nimba	Gbehlay-Geh	147199	2015	63.5	55.3	72.6
Liberia	Nimba	Gbi & Doru	147200	2000	183.3	168.0	200.2
Liberia	Nimba	Gbi & Doru	147200	2005	133.6	122.4	145.8
Liberia	Nimba	Gbi & Doru	147200	2010	99.2	90.0	109.1
Liberia	Nimba	Gbi & Doru	147200	2015	79.3	69.3	89.2
Liberia	Nimba	Gbor	147202	2000	144.7	132.6	157.5
Liberia	Nimba	Gbor	147202	2005	102.6	94.5	112.2
Liberia	Nimba	Gbor	147202	2010	75.3	67.3	82.4
Liberia	Nimba	Gbor	147202	2015	58.8	51.4	66.9
Liberia	Nimba	Kparblee	147225	2000	173.4	158.5	188.4
Liberia	Nimba	Kparblee	147225	2005	124.0	113.5	134.8
Liberia	Nimba	Kparblee	147225	2010	87.8	79.5	96.6
Liberia	Nimba	Kparblee	147225	2015	65.8	57.3	74.7
Liberia	Nimba	Leewehpea-Mahn	147229	2000	143.2	131.7	155.3
Liberia	Nimba	Leewehpea-Mahn	147229	2005	102.0	93.9	111.0
Liberia	Nimba	Leewehpea-Mahn	147229	2010	75.9	68.6	83.4
Liberia	Nimba	Leewehpea-Mahn	147229	2015	57.2	50.4	64.4
Liberia	Nimba	Meinpea-Mahn	147232	2000	153.6	141.9	166.0
Liberia	Nimba	Meinpea-Mahn	147232	2005	108.9	100.7	117.4
Liberia	Nimba	Meinpea-Mahn	147232	2010	82.0	75.0	89.4
Liberia	Nimba	Meinpea-Mahn	147232	2015	61.6	54.2	69.9
Liberia	Nimba	Sanniquellie Mahn	147250	2000	134.7	123.5	147.1
Liberia	Nimba	Sanniquellie Mahn	147250	2005	95.5	86.7	104.2
Liberia	Nimba	Sanniquellie Mahn	147250	2010	70.9	63.3	78.3
Liberia	Nimba	Sanniquellie Mahn	147250	2015	54.5	47.3	62.0
Liberia	Nimba	Twan River	147267	2000	154.7	141.2	169.6
Liberia	Nimba	Twan River	147267	2005	110.0	100.8	121.0
Liberia	Nimba	Twan River	147267	2010	79.0	70.9	87.1
Liberia	Nimba	Twan River	147267	2015	63.3	55.2	72.6
Liberia	Nimba	Wee-Gbehyi-Mahn	147272	2000	152.7	140.1	166.7
Liberia	Nimba	Wee-Gbehyi-Mahn	147272	2005	109.2	100.5	118.4
Liberia	Nimba	Wee-Gbehyi-Mahn	147272	2010	79.1	72.0	86.5
Liberia	Nimba	Wee-Gbehyi-Mahn	147272	2015	58.3	51.2	65.8
Liberia	Nimba	Yarmein	147275	2000	155.2	142.0	170.1
Liberia	Nimba	Yarmein	147275	2005	110.2	100.5	120.4
Liberia	Nimba	Yarmein	147275	2010	80.7	72.7	89.0
Liberia	Nimba	Yarmein	147275	2015	64.2	56.4	73.0
Liberia	Nimba	Yarpea Mahn	147276	2000	144.1	132.4	157.3
Liberia	Nimba	Yarpea Mahn	147276	2005	102.0	93.6	111.5
Liberia	Nimba	Yarpea Mahn	147276	2010	75.1	67.5	82.2
Liberia	Nimba	Yarpea Mahn	147276	2015	58.5	51.2	66.4
Liberia	Nimba	Yarwein Mehnsonnoh	147277	2000	171.7	156.8	187.4
Liberia	Nimba	Yarwein Mehnsonnoh	147277	2005	122.8	113.0	132.8
Liberia	Nimba	Yarwein Mehnsonnoh	147277	2010	89.5	81.7	97.8
Liberia	Nimba	Yarwein Mehnsonnoh	147277	2015	68.2	59.9	76.6

Admin 0	Admin 1	Admin 2	GAUL Code	Year	Under-5 mortality (per 1,000 livebirths)		
					Estimate	Lower bound, 95% UI	Upper bound, 95% UI
Liberia	Nimba	Zoe-Gbao	147279	2000	148.5	136.5	162.2
Liberia	Nimba	Zoe-Gbao	147279	2005	106.0	97.8	115.6
Liberia	Nimba	Zoe-Gbao	147279	2010	76.5	69.1	83.9
Liberia	Nimba	Zoe-Gbao	147279	2015	58.6	51.3	66.3
Liberia	River Gee	Chedepo	147173	2000	169.5	155.7	184.6
Liberia	River Gee	Chedepo	147173	2005	131.7	122.4	142.1
Liberia	River Gee	Chedepo	147173	2010	99.4	90.1	109.6
Liberia	River Gee	Chedepo	147173	2015	83.4	73.2	95.5
Liberia	River Gee	Gbeapo	147198	2000	163.8	151.3	177.6
Liberia	River Gee	Gbeapo	147198	2005	128.7	119.8	138.4
Liberia	River Gee	Gbeapo	147198	2010	97.1	88.7	106.6
Liberia	River Gee	Gbeapo	147198	2015	81.5	71.5	93.0
Liberia	River Gee	Glaro	147205	2000	156.6	140.6	173.8
Liberia	River Gee	Glaro	147205	2005	124.2	112.1	137.8
Liberia	River Gee	Glaro	147205	2010	93.3	83.3	104.7
Liberia	River Gee	Glaro	147205	2015	80.4	69.3	93.0
Liberia	River Gee	Karforh	147283	2000	170.9	156.9	185.6
Liberia	River Gee	Karforh	147283	2005	131.8	122.2	142.1
Liberia	River Gee	Karforh	147283	2010	100.6	91.8	110.2
Liberia	River Gee	Karforh	147283	2015	86.1	75.4	97.6
Liberia	River Gee	Nanee	147233	2000	167.4	154.0	180.5
Liberia	River Gee	Nanee	147233	2005	129.8	120.6	139.9
Liberia	River Gee	Nanee	147233	2010	100.3	91.8	109.3
Liberia	River Gee	Nanee	147233	2015	87.3	76.7	99.0
Liberia	River Gee	Nyenawliken	147237	2000	161.4	149.3	173.4
Liberia	River Gee	Nyenawliken	147237	2005	127.2	119.3	136.2
Liberia	River Gee	Nyenawliken	147237	2010	96.7	88.8	105.1
Liberia	River Gee	Nyenawliken	147237	2015	81.3	71.5	91.8
Liberia	River Gee	Nyenebo	147238	2000	149.8	138.9	161.9
Liberia	River Gee	Nyenebo	147238	2005	120.6	112.1	130.3
Liberia	River Gee	Nyenebo	147238	2010	90.6	82.6	100.0
Liberia	River Gee	Nyenebo	147238	2015	76.5	66.9	87.3
Liberia	River Gee	Potupo	147284	2000	161.6	149.6	174.7
Liberia	River Gee	Potupo	147284	2005	127.4	118.7	137.0
Liberia	River Gee	Potupo	147284	2010	96.0	87.9	105.0
Liberia	River Gee	Potupo	147284	2015	82.5	72.6	94.1
Liberia	River Gee	Sarbo	147254	2000	154.0	140.6	167.4
Liberia	River Gee	Sarbo	147254	2005	123.0	113.8	133.3
Liberia	River Gee	Sarbo	147254	2010	92.0	83.8	101.1
Liberia	River Gee	Sarbo	147254	2015	78.1	68.5	89.1
Liberia	River Gee	Tuobo	147266	2000	151.7	140.0	164.4
Liberia	River Gee	Tuobo	147266	2005	121.5	112.6	131.3
Liberia	River Gee	Tuobo	147266	2010	91.0	82.1	100.2
Liberia	River Gee	Tuobo	147266	2015	76.9	67.3	87.8
Liberia	Rivercess	Beawor	147158	2000	169.6	153.9	186.2
Liberia	Rivercess	Beawor	147158	2005	128.8	117.1	142.0
Liberia	Rivercess	Beawor	147158	2010	100.5	90.9	111.5
Liberia	Rivercess	Beawor	147158	2015	81.2	71.5	92.1
Liberia	Rivercess	Central Rivercess	147172	2000	176.0	161.5	190.3
Liberia	Rivercess	Central Rivercess	147172	2005	130.8	121.0	142.0
Liberia	Rivercess	Central Rivercess	147172	2010	99.9	91.2	109.4
Liberia	Rivercess	Central Rivercess	147172	2015	81.5	72.1	91.6
Liberia	Rivercess	Doedain	147182	2000	185.9	170.7	202.0
Liberia	Rivercess	Doedain	147182	2005	135.3	124.3	146.4
Liberia	Rivercess	Doedain	147182	2010	100.2	91.6	109.5
Liberia	Rivercess	Doedain	147182	2015	78.8	70.0	88.5
Liberia	Rivercess	Fen River	147187	2000	172.2	157.9	189.0
Liberia	Rivercess	Fen River	147187	2005	126.8	115.9	138.7
Liberia	Rivercess	Fen River	147187	2010	96.9	87.5	106.5
Liberia	Rivercess	Fen River	147187	2015	81.3	71.4	92.7
Liberia	Rivercess	Jo River	147214	2000	189.8	174.6	204.6
Liberia	Rivercess	Jo River	147214	2005	137.4	126.7	148.9
Liberia	Rivercess	Jo River	147214	2010	103.4	94.9	112.2
Liberia	Rivercess	Jo River	147214	2015	83.4	74.2	93.4
Liberia	Rivercess	Norwein	147235	2000	182.7	168.0	197.6
Liberia	Rivercess	Norwein	147235	2005	133.4	123.3	144.9
Liberia	Rivercess	Norwein	147235	2010	100.6	91.9	109.8
Liberia	Rivercess	Norwein	147235	2015	84.2	74.3	95.2
Liberia	Rivercess	Sam Gbalor	147288	2000	165.5	150.9	181.6
Liberia	Rivercess	Sam Gbalor	147288	2005	125.8	114.5	138.2
Liberia	Rivercess	Sam Gbalor	147288	2010	99.4	89.8	109.7
Liberia	Rivercess	Sam Gbalor	147288	2015	80.0	70.0	91.1
Liberia	Rivercess	Zarflahn	147289	2000	168.4	154.1	184.5
Liberia	Rivercess	Zarflahn	147289	2005	124.9	114.0	136.7
Liberia	Rivercess	Zarflahn	147289	2010	96.9	87.6	106.5
Liberia	Rivercess	Zarflahn	147289	2015	80.1	69.9	91.6
Liberia	Sinoe	Bodae	147161	2000	165.9	150.4	181.2
Liberia	Sinoe	Bodae	147161	2005	129.9	118.4	141.4
Liberia	Sinoe	Bodae	147161	2010	102.0	92.2	112.7
Liberia	Sinoe	Bodae	147161	2015	87.4	76.3	100.0
Liberia	Sinoe	Bokon	147165	2000	162.7	147.2	177.8
Liberia	Sinoe	Bokon	147165	2005	129.2	118.2	139.9
Liberia	Sinoe	Bokon	147165	2010	102.4	92.5	113.0
Liberia	Sinoe	Bokon	147165	2015	86.2	75.6	97.9
Liberia	Sinoe	Butaw	147286	2000	149.9	135.9	164.7

Admin 0	Admin 1	Admin 2	GAUL Code	Year	Under-5 mortality (per 1,000 livebirths)		
					Estimate	Lower bound, 95% UI	Upper bound, 95% UI
Liberia	Sinoe	Butaw	147286	2005	112.4	101.9	123.4
Liberia	Sinoe	Butaw	147286	2010	93.5	83.5	103.9
Liberia	Sinoe	Butaw	147286	2015	76.1	65.8	86.5
Liberia	Sinoe	Dugbe River	147184	2000	160.7	145.4	176.7
Liberia	Sinoe	Dugbe River	147184	2005	128.2	117.4	139.9
Liberia	Sinoe	Dugbe River	147184	2010	103.9	93.4	115.6
Liberia	Sinoe	Dugbe River	147184	2015	85.5	74.6	97.3
Liberia	Sinoe	Greenville	147210	2000	131.9	119.4	145.1
Liberia	Sinoe	Greenville	147210	2005	98.2	88.7	107.8
Liberia	Sinoe	Greenville	147210	2010	84.2	75.2	93.3
Liberia	Sinoe	Greenville	147210	2015	65.0	56.6	74.0
Liberia	Sinoe	Jaedae	147212	2000	166.3	150.9	181.0
Liberia	Sinoe	Jaedae	147212	2005	131.1	120.0	142.3
Liberia	Sinoe	Jaedae	147212	2010	106.1	96.1	116.5
Liberia	Sinoe	Jaedae	147212	2015	89.5	78.5	101.7
Liberia	Sinoe	Jeadepo	147213	2000	171.0	156.6	185.2
Liberia	Sinoe	Jeadepo	147213	2005	132.9	122.2	144.0
Liberia	Sinoe	Jeadepo	147213	2010	104.8	95.0	114.7
Liberia	Sinoe	Jeadepo	147213	2015	88.5	77.7	100.8
Liberia	Sinoe	Juarzon	147216	2000	160.2	144.6	177.0
Liberia	Sinoe	Juarzon	147216	2005	124.5	113.2	136.9
Liberia	Sinoe	Juarzon	147216	2010	99.6	89.2	111.0
Liberia	Sinoe	Juarzon	147216	2015	81.4	70.7	92.7
Liberia	Sinoe	Kpayan	147226	2000	161.6	147.8	175.3
Liberia	Sinoe	Kpayan	147226	2005	127.9	117.7	138.4
Liberia	Sinoe	Kpayan	147226	2010	104.9	94.7	115.6
Liberia	Sinoe	Kpayan	147226	2015	84.6	74.2	96.1
Liberia	Sinoe	Kulu Shaw Boe	147228	2000	168.0	153.6	184.2
Liberia	Sinoe	Kulu Shaw Boe	147228	2005	129.7	118.5	142.0
Liberia	Sinoe	Kulu Shaw Boe	147228	2010	102.2	91.9	113.3
Liberia	Sinoe	Kulu Shaw Boe	147228	2015	82.9	72.4	93.9
Liberia	Sinoe	Plahn Nyarn	147242	2000	159.0	143.4	175.9
Liberia	Sinoe	Plahn Nyarn	147242	2005	124.0	112.1	137.2
Liberia	Sinoe	Plahn Nyarn	147242	2010	99.9	89.0	112.3
Liberia	Sinoe	Plahn Nyarn	147242	2015	81.1	70.4	92.9
Liberia	Sinoe	Pynes Town	147246	2000	152.1	138.4	167.7
Liberia	Sinoe	Pynes Town	147246	2005	119.8	109.2	132.0
Liberia	Sinoe	Pynes Town	147246	2010	89.9	80.6	99.6
Liberia	Sinoe	Pynes Town	147246	2015	72.1	62.7	82.0
Liberia	Sinoe	Sanquin Dist No. 1	147252	2000	168.7	153.2	185.8
Liberia	Sinoe	Sanquin Dist No. 1	147252	2005	130.6	118.5	142.9
Liberia	Sinoe	Sanquin Dist No. 1	147252	2010	102.5	92.4	113.8
Liberia	Sinoe	Sanquin Dist No. 1	147252	2015	83.5	73.3	94.1
Liberia	Sinoe	Sanquin Dist No. 3	147253	2000	153.7	139.9	168.5
Liberia	Sinoe	Sanquin Dist No. 3	147253	2005	114.5	104.6	126.0
Liberia	Sinoe	Sanquin Dist No. 3	147253	2010	93.6	84.4	103.3
Liberia	Sinoe	Sanquin Dist No. 3	147253	2015	75.4	65.8	85.5
Liberia	Sinoe	Sanquin Dist No.2	147287	2000	149.6	136.4	164.5
Liberia	Sinoe	Sanquin Dist No.2	147287	2005	112.1	102.3	122.8
Liberia	Sinoe	Sanquin Dist No.2	147287	2010	93.5	83.6	103.8
Liberia	Sinoe	Sanquin Dist No.2	147287	2015	75.5	65.1	85.6
Liberia	Sinoe	Seekon	147255	2000	172.2	157.7	187.9
Liberia	Sinoe	Seekon	147255	2005	132.9	121.7	145.5
Liberia	Sinoe	Seekon	147255	2010	103.4	93.1	114.1
Liberia	Sinoe	Seekon	147255	2015	84.3	73.7	95.4
Liberia	Sinoe	Wedjah	147271	2000	163.5	148.6	178.7
Liberia	Sinoe	Wedjah	147271	2005	127.0	116.7	138.3
Liberia	Sinoe	Wedjah	147271	2010	102.5	92.1	113.3
Liberia	Sinoe	Wedjah	147271	2015	82.7	72.8	93.8
Madagascar	Alaotra Mangoro	Ambatondrazaka	154509	2000	112.9	101.1	125.8
Madagascar	Alaotra Mangoro	Ambatondrazaka	154509	2005	98.2	87.9	109.4
Madagascar	Alaotra Mangoro	Ambatondrazaka	154509	2010	87.6	77.5	98.2
Madagascar	Alaotra Mangoro	Ambatondrazaka	154509	2015	74.7	64.7	85.4
Madagascar	Alaotra Mangoro	Amparafaravola	154510	2000	118.8	105.9	132.1
Madagascar	Alaotra Mangoro	Amparafaravola	154510	2005	100.0	89.6	111.1
Madagascar	Alaotra Mangoro	Amparafaravola	154510	2010	88.0	77.7	99.3
Madagascar	Alaotra Mangoro	Amparafaravola	154510	2015	75.4	65.3	85.7
Madagascar	Alaotra Mangoro	Andilamena	154511	2000	117.1	102.7	133.6
Madagascar	Alaotra Mangoro	Andilamena	154511	2005	96.0	84.5	109.5
Madagascar	Alaotra Mangoro	Andilamena	154511	2010	87.5	76.1	100.3
Madagascar	Alaotra Mangoro	Andilamena	154511	2015	75.4	64.5	87.4
Madagascar	Alaotra Mangoro	Anosibe-An'ala	154512	2000	107.1	95.1	120.9
Madagascar	Alaotra Mangoro	Anosibe-An'ala	154512	2005	106.1	94.3	120.5
Madagascar	Alaotra Mangoro	Anosibe-An'ala	154512	2010	106.1	93.9	120.9
Madagascar	Alaotra Mangoro	Anosibe-An'ala	154512	2015	87.7	75.8	100.6
Madagascar	Alaotra Mangoro	Moramanga	154513	2000	103.8	93.4	116.9
Madagascar	Alaotra Mangoro	Moramanga	154513	2005	100.7	90.5	112.5
Madagascar	Alaotra Mangoro	Moramanga	154513	2010	94.6	84.3	106.5
Madagascar	Alaotra Mangoro	Moramanga	154513	2015	76.7	66.3	87.7
Madagascar	Amoron I Mania	Ambatofinandrahana	154514	2000	110.4	97.9	123.6
Madagascar	Amoron I Mania	Ambatofinandrahana	154514	2005	100.4	90.0	112.7
Madagascar	Amoron I Mania	Ambatofinandrahana	154514	2010	94.2	83.6	106.6
Madagascar	Amoron I Mania	Ambatofinandrahana	154514	2015	82.3	71.5	95.6
Madagascar	Amoron I Mania	Ambositra	154515	2000	117.6	107.9	128.3
Madagascar	Amoron I Mania	Ambositra	154515	2005	109.4	100.2	119.1

Admin 0	Admin 1	Admin 2	GAUL Code	Year	Under-5 mortality (per 1,000 livebirths)		
					Estimate	Lower bound, 95% UI	Upper bound, 95% UI
Madagascar	Amoron I Mania	Ambositra	154515	2010	103.7	94.0	114.2
Madagascar	Amoron I Mania	Ambositra	154515	2015	89.1	78.2	100.6
Madagascar	Amoron I Mania	Fandriana	154516	2000	111.0	100.7	121.8
Madagascar	Amoron I Mania	Fandriana	154516	2005	102.2	92.6	112.7
Madagascar	Amoron I Mania	Fandriana	154516	2010	98.5	88.6	109.4
Madagascar	Amoron I Mania	Fandriana	154516	2015	85.3	74.5	97.6
Madagascar	Amoron I Mania	Manandriana	154517	2000	114.9	104.4	127.1
Madagascar	Amoron I Mania	Manandriana	154517	2005	106.6	97.3	117.0
Madagascar	Amoron I Mania	Manandriana	154517	2010	99.3	89.3	109.5
Madagascar	Amoron I Mania	Manandriana	154517	2015	87.8	76.7	99.6
Madagascar	Analamanga	Ambohidratrimo	154518	2000	81.0	73.8	88.9
Madagascar	Analamanga	Ambohidratrimo	154518	2005	76.5	69.8	84.1
Madagascar	Analamanga	Ambohidratrimo	154518	2010	78.4	70.7	86.9
Madagascar	Analamanga	Ambohidratrimo	154518	2015	71.1	62.1	80.9
Madagascar	Analamanga	Andramasina	154519	2000	87.4	78.9	96.9
Madagascar	Analamanga	Andramasina	154519	2005	85.8	77.2	95.5
Madagascar	Analamanga	Andramasina	154519	2010	87.0	77.7	97.5
Madagascar	Analamanga	Andramasina	154519	2015	73.3	63.7	83.7
Madagascar	Analamanga	Anjozorobe	154520	2000	96.1	86.2	108.2
Madagascar	Analamanga	Anjozorobe	154520	2005	94.1	84.3	105.6
Madagascar	Analamanga	Anjozorobe	154520	2010	89.1	78.5	99.8
Madagascar	Analamanga	Anjozorobe	154520	2015	80.3	68.7	91.5
Madagascar	Analamanga	Ankazobe	154521	2000	95.6	85.3	107.8
Madagascar	Analamanga	Ankazobe	154521	2005	92.5	81.8	104.0
Madagascar	Analamanga	Ankazobe	154521	2010	91.7	80.7	104.0
Madagascar	Analamanga	Ankazobe	154521	2015	84.5	72.5	97.2
Madagascar	Analamanga	Antananarivo Atsimondrano	154522	2000	74.3	68.1	81.6
Madagascar	Analamanga	Antananarivo Atsimondrano	154522	2005	70.7	64.8	77.1
Madagascar	Analamanga	Antananarivo Atsimondrano	154522	2010	72.5	65.3	80.4
Madagascar	Analamanga	Antananarivo Atsimondrano	154522	2015	65.0	57.2	73.9
Madagascar	Analamanga	Antananarivo Avaradrano	154523	2000	76.0	69.2	83.8
Madagascar	Analamanga	Antananarivo Avaradrano	154523	2005	72.8	66.5	79.5
Madagascar	Analamanga	Antananarivo Avaradrano	154523	2010	74.1	66.6	82.1
Madagascar	Analamanga	Antananarivo Avaradrano	154523	2015	66.2	57.8	75.8
Madagascar	Analamanga	Antananarivo I	123870	2000	69.1	63.1	75.9
Madagascar	Analamanga	Antananarivo I	123870	2005	67.7	62.0	73.8
Madagascar	Analamanga	Antananarivo I	123870	2010	69.1	62.3	76.7
Madagascar	Analamanga	Antananarivo I	123870	2015	64.4	56.4	73.7
Madagascar	Analamanga	Antananarivo II	123871	2000	71.9	65.4	78.9
Madagascar	Analamanga	Antananarivo II	123871	2005	69.4	63.4	75.8
Madagascar	Analamanga	Antananarivo II	123871	2010	70.3	63.2	78.2
Madagascar	Analamanga	Antananarivo II	123871	2015	63.8	55.6	73.3
Madagascar	Analamanga	Antananarivo III	123872	2000	69.1	63.1	75.9
Madagascar	Analamanga	Antananarivo III	123872	2005	67.7	62.0	73.8
Madagascar	Analamanga	Antananarivo III	123872	2010	69.1	62.3	76.7
Madagascar	Analamanga	Antananarivo III	123872	2015	64.4	56.4	73.7
Madagascar	Analamanga	Antananarivo IV	123873	2000	68.0	62.1	74.6
Madagascar	Analamanga	Antananarivo IV	123873	2005	67.0	61.6	73.2
Madagascar	Analamanga	Antananarivo IV	123873	2010	69.0	62.2	76.5
Madagascar	Analamanga	Antananarivo IV	123873	2015	63.4	55.4	72.6
Madagascar	Analamanga	Antananarivo V	123874	2000	70.5	64.3	77.7
Madagascar	Analamanga	Antananarivo V	123874	2005	68.2	62.1	74.2
Madagascar	Analamanga	Antananarivo V	123874	2010	69.9	63.0	77.8
Madagascar	Analamanga	Antananarivo V	123874	2015	64.7	56.6	74.1
Madagascar	Analamanga	Antananarivo VI	123875	2000	70.0	64.2	76.5
Madagascar	Analamanga	Antananarivo VI	123875	2005	68.9	63.2	74.9
Madagascar	Analamanga	Antananarivo VI	123875	2010	70.6	63.9	77.8
Madagascar	Analamanga	Antananarivo VI	123875	2015	63.7	55.8	72.5
Madagascar	Analamanga	Manjakandriana	154524	2000	83.1	75.7	91.7
Madagascar	Analamanga	Manjakandriana	154524	2005	81.0	73.7	89.1
Madagascar	Analamanga	Manjakandriana	154524	2010	80.5	72.3	89.5
Madagascar	Analamanga	Manjakandriana	154524	2015	69.5	60.7	79.5
Madagascar	Analanjirofo	Fenerive Est	154525	2000	121.7	106.1	137.4
Madagascar	Analanjirofo	Fenerive Est	154525	2005	99.0	87.2	112.2
Madagascar	Analanjirofo	Fenerive Est	154525	2010	87.3	76.0	99.9
Madagascar	Analanjirofo	Fenerive Est	154525	2015	71.8	61.3	83.6
Madagascar	Analanjirofo	Mananara-Avaratra	154526	2000	115.8	100.7	134.2
Madagascar	Analanjirofo	Mananara-Avaratra	154526	2005	95.3	82.3	109.9
Madagascar	Analanjirofo	Mananara-Avaratra	154526	2010	84.9	73.1	99.2
Madagascar	Analanjirofo	Mananara-Avaratra	154526	2015	70.8	59.4	83.7
Madagascar	Analanjirofo	Maroantsetra	154527	2000	105.7	92.4	120.2
Madagascar	Analanjirofo	Maroantsetra	154527	2005	88.0	77.0	99.9
Madagascar	Analanjirofo	Maroantsetra	154527	2010	77.0	67.0	88.2
Madagascar	Analanjirofo	Maroantsetra	154527	2015	68.5	57.5	80.1
Madagascar	Analanjirofo	Sainte Marie	154528	2000	114.8	96.0	138.6
Madagascar	Analanjirofo	Sainte Marie	154528	2005	94.7	79.4	113.5
Madagascar	Analanjirofo	Sainte Marie	154528	2010	80.8	67.5	96.2
Madagascar	Analanjirofo	Sainte Marie	154528	2015	69.4	56.2	83.5
Madagascar	Analanjirofo	Soanierana Ivongo	154529	2000	119.8	104.1	137.2
Madagascar	Analanjirofo	Soanierana Ivongo	154529	2005	98.5	85.7	113.4
Madagascar	Analanjirofo	Soanierana Ivongo	154529	2010	87.7	74.9	101.8
Madagascar	Analanjirofo	Soanierana Ivongo	154529	2015	71.1	59.3	83.7
Madagascar	Analanjirofo	Vavatenina	154530	2000	124.9	111.3	139.4
Madagascar	Analanjirofo	Vavatenina	154530	2005	101.6	90.0	113.9
Madagascar	Analanjirofo	Vavatenina	154530	2010	90.0	79.8	102.3

Admin 0	Admin 1	Admin 2	GAUL Code	Year	Under-5 mortality (per 1,000 livebirths)		
					Estimate	Lower bound, 95% UI	Upper bound, 95% UI
Madagascar	Analanjirofo	Vavatenina	154530	2015	73.9	63.6	86.0
Madagascar	Androy	Ambovombe-Androy	154531	2000	115.7	106.0	125.3
Madagascar	Androy	Ambovombe-Androy	154531	2005	98.7	91.2	107.1
Madagascar	Androy	Ambovombe-Androy	154531	2010	92.7	84.9	101.2
Madagascar	Androy	Ambovombe-Androy	154531	2015	79.1	70.1	89.7
Madagascar	Androy	Bekily	154532	2000	131.0	119.1	145.0
Madagascar	Androy	Bekily	154532	2005	106.2	97.0	116.5
Madagascar	Androy	Bekily	154532	2010	100.6	90.7	111.8
Madagascar	Androy	Bekily	154532	2015	89.6	78.9	102.9
Madagascar	Androy	Beloha	154533	2000	102.0	91.1	112.7
Madagascar	Androy	Beloha	154533	2005	86.1	77.8	94.8
Madagascar	Androy	Beloha	154533	2010	79.3	71.0	88.1
Madagascar	Androy	Beloha	154533	2015	68.0	58.7	77.8
Madagascar	Androy	Tsihombe	154534	2000	104.7	94.3	115.9
Madagascar	Androy	Tsihombe	154534	2005	90.0	81.9	99.3
Madagascar	Androy	Tsihombe	154534	2010	84.6	75.9	94.0
Madagascar	Androy	Tsihombe	154534	2015	71.8	62.7	81.6
Madagascar	Anosy	Amboasary-Atsimo	154535	2000	125.7	115.2	137.4
Madagascar	Anosy	Amboasary-Atsimo	154535	2005	112.2	102.8	122.2
Madagascar	Anosy	Amboasary-Atsimo	154535	2010	102.1	93.0	111.6
Madagascar	Anosy	Amboasary-Atsimo	154535	2015	91.9	81.1	104.7
Madagascar	Anosy	Betroka	154536	2000	139.1	123.8	155.4
Madagascar	Anosy	Betroka	154536	2005	119.9	107.0	134.3
Madagascar	Anosy	Betroka	154536	2010	110.0	97.2	124.7
Madagascar	Anosy	Betroka	154536	2015	97.3	84.5	111.8
Madagascar	Anosy	Taolagnaro	154537	2000	152.6	137.1	169.8
Madagascar	Anosy	Taolagnaro	154537	2005	139.6	126.2	154.5
Madagascar	Anosy	Taolagnaro	154537	2010	128.3	114.6	143.3
Madagascar	Anosy	Taolagnaro	154537	2015	117.0	101.7	134.0
Madagascar	Atsimo Andrefana	Ampanihy Ouest	154538	2000	111.6	101.1	123.6
Madagascar	Atsimo Andrefana	Ampanihy Ouest	154538	2005	97.0	88.4	106.2
Madagascar	Atsimo Andrefana	Ampanihy Ouest	154538	2010	84.7	76.8	93.7
Madagascar	Atsimo Andrefana	Ampanihy Ouest	154538	2015	77.2	67.8	87.5
Madagascar	Atsimo Andrefana	Ankazoabo	154539	2000	144.4	121.7	168.9
Madagascar	Atsimo Andrefana	Ankazoabo	154539	2005	116.7	98.8	136.5
Madagascar	Atsimo Andrefana	Ankazoabo	154539	2010	102.8	86.9	120.5
Madagascar	Atsimo Andrefana	Ankazoabo	154539	2015	97.4	80.9	115.5
Madagascar	Atsimo Andrefana	Benenitra	154540	2000	134.7	118.2	153.1
Madagascar	Atsimo Andrefana	Benenitra	154540	2005	112.9	99.9	127.0
Madagascar	Atsimo Andrefana	Benenitra	154540	2010	102.8	89.5	116.6
Madagascar	Atsimo Andrefana	Benenitra	154540	2015	97.5	82.6	113.4
Madagascar	Atsimo Andrefana	Beroroha	154541	2000	133.3	111.9	157.4
Madagascar	Atsimo Andrefana	Beroroha	154541	2005	113.7	95.2	135.9
Madagascar	Atsimo Andrefana	Beroroha	154541	2010	102.5	86.2	122.0
Madagascar	Atsimo Andrefana	Beroroha	154541	2015	94.5	79.1	114.4
Madagascar	Atsimo Andrefana	Betioky Atsimo	154542	2000	119.8	107.6	132.7
Madagascar	Atsimo Andrefana	Betioky Atsimo	154542	2005	99.7	90.3	110.9
Madagascar	Atsimo Andrefana	Betioky Atsimo	154542	2010	88.5	78.8	98.9
Madagascar	Atsimo Andrefana	Betioky Atsimo	154542	2015	85.4	74.2	98.1
Madagascar	Atsimo Andrefana	Morombe	154543	2000	129.7	110.5	152.2
Madagascar	Atsimo Andrefana	Morombe	154543	2005	103.3	88.3	122.0
Madagascar	Atsimo Andrefana	Morombe	154543	2010	91.5	77.6	108.2
Madagascar	Atsimo Andrefana	Morombe	154543	2015	83.9	69.6	100.4
Madagascar	Atsimo Andrefana	Sakaraha	154544	2000	133.4	115.9	150.7
Madagascar	Atsimo Andrefana	Sakaraha	154544	2005	107.0	94.6	121.4
Madagascar	Atsimo Andrefana	Sakaraha	154544	2010	97.3	84.6	111.3
Madagascar	Atsimo Andrefana	Sakaraha	154544	2015	89.0	76.7	104.0
Madagascar	Atsimo Andrefana	Toliary-I	154545	2000	85.3	74.4	97.2
Madagascar	Atsimo Andrefana	Toliary-I	154545	2005	74.1	64.0	84.2
Madagascar	Atsimo Andrefana	Toliary-I	154545	2010	72.5	63.1	83.8
Madagascar	Atsimo Andrefana	Toliary-I	154545	2015	67.3	56.9	79.3
Madagascar	Atsimo Andrefana	Toliary-II	154546	2000	114.8	102.5	127.6
Madagascar	Atsimo Andrefana	Toliary-II	154546	2005	93.5	83.9	102.9
Madagascar	Atsimo Andrefana	Toliary-II	154546	2010	82.7	73.8	92.6
Madagascar	Atsimo Andrefana	Toliary-II	154546	2015	75.2	65.3	86.7
Madagascar	Atsimo Atsinanana	Befotaka	154547	2000	154.1	137.2	173.7
Madagascar	Atsimo Atsinanana	Befotaka	154547	2005	137.3	121.7	154.4
Madagascar	Atsimo Atsinanana	Befotaka	154547	2010	131.6	117.2	149.1
Madagascar	Atsimo Atsinanana	Befotaka	154547	2015	118.8	102.3	137.9
Madagascar	Atsimo Atsinanana	Farafangana	154548	2000	174.2	158.5	190.4
Madagascar	Atsimo Atsinanana	Farafangana	154548	2005	152.4	139.8	167.1
Madagascar	Atsimo Atsinanana	Farafangana	154548	2010	136.5	123.7	150.8
Madagascar	Atsimo Atsinanana	Farafangana	154548	2015	122.9	108.1	139.2
Madagascar	Atsimo Atsinanana	Midongy-Atsimo	154549	2000	162.2	144.9	181.6
Madagascar	Atsimo Atsinanana	Midongy-Atsimo	154549	2005	142.1	127.2	158.5
Madagascar	Atsimo Atsinanana	Midongy-Atsimo	154549	2010	136.8	122.5	153.4
Madagascar	Atsimo Atsinanana	Midongy-Atsimo	154549	2015	117.0	101.5	135.2
Madagascar	Atsimo Atsinanana	Vangaindrano	154550	2000	174.6	159.4	192.8
Madagascar	Atsimo Atsinanana	Vangaindrano	154550	2005	156.7	143.8	169.8
Madagascar	Atsimo Atsinanana	Vangaindrano	154550	2010	146.5	133.7	162.5
Madagascar	Atsimo Atsinanana	Vangaindrano	154550	2015	129.0	113.3	149.0
Madagascar	Atsimo Atsinanana	Vondrozo	154551	2000	168.2	153.4	183.4
Madagascar	Atsimo Atsinanana	Vondrozo	154551	2005	145.0	133.0	158.0
Madagascar	Atsimo Atsinanana	Vondrozo	154551	2010	132.5	119.8	145.6
Madagascar	Atsimo Atsinanana	Vondrozo	154551	2015	118.5	104.8	133.7

Admin 0	Admin 1	Admin 2	GAUL Code	Year	Under-5 mortality (per 1,000 livebirths)		
					Estimate	Lower bound, 95% UI	Upper bound, 95% UI
Madagascar	Atsinanana	Antanambao Manampontsy	154552	2000	122.6	109.4	139.3
Madagascar	Atsinanana	Antanambao Manampontsy	154552	2005	112.4	99.4	128.3
Madagascar	Atsinanana	Antanambao Manampontsy	154552	2010	109.3	96.2	124.7
Madagascar	Atsinanana	Antanambao Manampontsy	154552	2015	89.0	76.5	103.6
Madagascar	Atsinanana	Brickaville	154553	2000	123.8	108.5	140.4
Madagascar	Atsinanana	Brickaville	154553	2005	109.9	96.5	125.4
Madagascar	Atsinanana	Brickaville	154553	2010	99.2	86.8	113.9
Madagascar	Atsinanana	Brickaville	154553	2015	79.7	67.5	91.7
Madagascar	Atsinanana	Mahanoro	154554	2000	139.2	122.8	155.8
Madagascar	Atsinanana	Mahanoro	154554	2005	126.3	111.7	143.3
Madagascar	Atsinanana	Mahanoro	154554	2010	123.9	108.5	141.0
Madagascar	Atsinanana	Mahanoro	154554	2015	104.6	89.5	120.5
Madagascar	Atsinanana	Marolambo	154555	2000	118.5	106.1	132.9
Madagascar	Atsinanana	Marolambo	154555	2005	115.1	103.5	129.8
Madagascar	Atsinanana	Marolambo	154555	2010	117.8	104.2	132.8
Madagascar	Atsinanana	Marolambo	154555	2015	101.2	87.6	116.2
Madagascar	Atsinanana	Toamasina I	154556	2000	96.8	83.8	111.0
Madagascar	Atsinanana	Toamasina I	154556	2005	90.8	79.1	103.3
Madagascar	Atsinanana	Toamasina I	154556	2010	82.0	70.9	94.6
Madagascar	Atsinanana	Toamasina I	154556	2015	73.1	61.7	85.5
Madagascar	Atsinanana	Toamasina II	154557	2000	129.0	115.5	142.6
Madagascar	Atsinanana	Toamasina II	154557	2005	107.4	95.7	119.8
Madagascar	Atsinanana	Toamasina II	154557	2010	94.4	83.3	106.8
Madagascar	Atsinanana	Toamasina II	154557	2015	76.8	66.2	87.9
Madagascar	Atsinanana	Vatomandry	154558	2000	128.5	112.5	146.6
Madagascar	Atsinanana	Vatomandry	154558	2005	113.9	99.6	129.7
Madagascar	Atsinanana	Vatomandry	154558	2010	106.3	92.3	122.3
Madagascar	Atsinanana	Vatomandry	154558	2015	85.2	72.9	99.1
Madagascar	Betsiboka	Kandreho	154559	2000	115.9	98.4	135.0
Madagascar	Betsiboka	Kandreho	154559	2005	97.4	83.3	113.0
Madagascar	Betsiboka	Kandreho	154559	2010	90.7	76.7	106.4
Madagascar	Betsiboka	Kandreho	154559	2015	87.9	73.5	104.7
Madagascar	Betsiboka	Maevatanana	154560	2000	119.2	104.9	133.7
Madagascar	Betsiboka	Maevatanana	154560	2005	104.1	92.6	117.7
Madagascar	Betsiboka	Maevatanana	154560	2010	98.3	86.1	111.4
Madagascar	Betsiboka	Maevatanana	154560	2015	87.7	75.2	101.7
Madagascar	Betsiboka	Tsaratanana	154561	2000	126.0	110.8	142.3
Madagascar	Betsiboka	Tsaratanana	154561	2005	107.7	95.6	121.5
Madagascar	Betsiboka	Tsaratanana	154561	2010	98.6	86.7	112.2
Madagascar	Betsiboka	Tsaratanana	154561	2015	86.4	73.7	100.6
Madagascar	Boeny	Ambato Boeni	154562	2000	121.1	107.5	136.3
Madagascar	Boeny	Ambato Boeni	154562	2005	101.6	90.3	115.0
Madagascar	Boeny	Ambato Boeni	154562	2010	93.7	82.0	106.3
Madagascar	Boeny	Ambato Boeni	154562	2015	83.7	72.3	97.1
Madagascar	Boeny	Mahajanga I	154563	2000	96.0	81.7	114.3
Madagascar	Boeny	Mahajanga I	154563	2005	87.9	74.5	103.0
Madagascar	Boeny	Mahajanga I	154563	2010	82.0	69.0	97.1
Madagascar	Boeny	Mahajanga I	154563	2015	75.2	62.2	91.2
Madagascar	Boeny	Mahajanga II	154564	2000	108.3	94.1	124.5
Madagascar	Boeny	Mahajanga II	154564	2005	94.4	82.5	108.0
Madagascar	Boeny	Mahajanga II	154564	2010	85.1	73.1	98.5
Madagascar	Boeny	Mahajanga II	154564	2015	75.2	62.8	89.6
Madagascar	Boeny	Marovoay	154565	2000	124.0	110.3	139.5
Madagascar	Boeny	Marovoay	154565	2005	104.0	93.4	116.5
Madagascar	Boeny	Marovoay	154565	2010	93.4	82.2	105.8
Madagascar	Boeny	Marovoay	154565	2015	80.9	69.2	93.3
Madagascar	Boeny	Mitsinjo	154566	2000	123.7	106.6	145.9
Madagascar	Boeny	Mitsinjo	154566	2005	104.5	90.3	121.6
Madagascar	Boeny	Mitsinjo	154566	2010	94.2	80.1	110.1
Madagascar	Boeny	Mitsinjo	154566	2015	84.3	70.3	99.6
Madagascar	Boeny	Soalala	154567	2000	121.7	102.5	143.5
Madagascar	Boeny	Soalala	154567	2005	101.8	86.3	119.1
Madagascar	Boeny	Soalala	154567	2010	91.8	76.9	108.1
Madagascar	Boeny	Soalala	154567	2015	88.5	73.9	105.3
Madagascar	Bongolava	Fenoarivobe	154568	2000	100.0	88.3	112.0
Madagascar	Bongolava	Fenoarivobe	154568	2005	92.5	82.2	105.0
Madagascar	Bongolava	Fenoarivobe	154568	2010	87.9	77.5	99.5
Madagascar	Bongolava	Fenoarivobe	154568	2015	82.3	70.7	95.2
Madagascar	Bongolava	Tsiroanomandidy	154569	2000	103.3	91.8	115.4
Madagascar	Bongolava	Tsiroanomandidy	154569	2005	93.1	83.9	104.1
Madagascar	Bongolava	Tsiroanomandidy	154569	2010	85.2	75.8	96.1
Madagascar	Bongolava	Tsiroanomandidy	154569	2015	78.6	68.0	90.2
Madagascar	Diana	Ambanja	154570	2000	102.3	88.0	117.5
Madagascar	Diana	Ambanja	154570	2005	85.9	74.1	98.6
Madagascar	Diana	Ambanja	154570	2010	76.3	65.6	87.7
Madagascar	Diana	Ambanja	154570	2015	71.9	61.1	85.1
Madagascar	Diana	Ambilobe	154571	2000	96.5	85.0	109.5
Madagascar	Diana	Ambilobe	154571	2005	80.9	71.2	92.2
Madagascar	Diana	Ambilobe	154571	2010	73.9	64.2	85.2
Madagascar	Diana	Ambilobe	154571	2015	70.1	60.2	82.5
Madagascar	Diana	Antsiranana I	154572	2000	90.3	73.4	111.1
Madagascar	Diana	Antsiranana I	154572	2005	75.4	61.6	92.0
Madagascar	Diana	Antsiranana I	154572	2010	72.3	59.1	87.5
Madagascar	Diana	Antsiranana I	154572	2015	65.2	52.2	80.4
Madagascar	Diana	Antsiranana II	154573	2000	101.3	85.5	121.5

Admin 0	Admin 1	Admin 2	GAUL Code	Year	Under-5 mortality (per 1,000 livebirths) Estimate	Lower bound, 95% UI	Upper bound, 95% UI
Madagascar	Diana	Antsiranana II	154573	2005	85.3	71.5	101.6
Madagascar	Diana	Antsiranana II	154573	2010	75.9	63.1	90.8
Madagascar	Diana	Antsiranana II	154573	2015	69.7	57.2	83.7
Madagascar	Diana	Nosy-Be	154574	2000	86.3	71.4	103.4
Madagascar	Diana	Nosy-Be	154574	2005	77.7	64.2	93.2
Madagascar	Diana	Nosy-Be	154574	2010	76.4	63.0	91.3
Madagascar	Diana	Nosy-Be	154574	2015	72.3	58.6	86.9
Madagascar	Haute Matsiatra	Ambalavao	154575	2000	133.2	119.8	146.8
Madagascar	Haute Matsiatra	Ambalavao	154575	2005	119.1	108.8	130.8
Madagascar	Haute Matsiatra	Ambalavao	154575	2010	112.7	102.0	124.4
Madagascar	Haute Matsiatra	Ambalavao	154575	2015	96.1	84.4	108.3
Madagascar	Haute Matsiatra	Ambohimahasoa	154576	2000	126.8	115.6	139.2
Madagascar	Haute Matsiatra	Ambohimahasoa	154576	2005	115.0	105.5	125.9
Madagascar	Haute Matsiatra	Ambohimahasoa	154576	2010	106.8	97.3	118.8
Madagascar	Haute Matsiatra	Ambohimahasoa	154576	2015	92.1	80.7	105.1
Madagascar	Haute Matsiatra	Fianarantsoa I	154577	2000	121.9	110.2	135.3
Madagascar	Haute Matsiatra	Fianarantsoa I	154577	2005	108.7	98.7	120.7
Madagascar	Haute Matsiatra	Fianarantsoa I	154577	2010	104.6	93.5	116.7
Madagascar	Haute Matsiatra	Fianarantsoa I	154577	2015	90.8	78.8	104.3
Madagascar	Haute Matsiatra	Ikalamavony	154578	2000	121.2	106.5	137.4
Madagascar	Haute Matsiatra	Ikalamavony	154578	2005	109.5	96.4	123.8
Madagascar	Haute Matsiatra	Ikalamavony	154578	2010	103.3	90.9	117.7
Madagascar	Haute Matsiatra	Ikalamavony	154578	2015	88.3	76.4	102.8
Madagascar	Haute Matsiatra	Isandra	123865	2000	122.4	110.3	136.2
Madagascar	Haute Matsiatra	Isandra	123865	2005	109.7	99.1	121.3
Madagascar	Haute Matsiatra	Isandra	123865	2010	103.9	93.4	116.2
Madagascar	Haute Matsiatra	Isandra	123865	2015	89.8	78.7	102.1
Madagascar	Haute Matsiatra	Lalangina	123866	2000	134.4	122.3	148.3
Madagascar	Haute Matsiatra	Lalangina	123866	2005	120.9	110.9	132.2
Madagascar	Haute Matsiatra	Lalangina	123866	2010	112.2	101.3	124.2
Madagascar	Haute Matsiatra	Lalangina	123866	2015	95.2	82.9	108.3
Madagascar	Haute Matsiatra	Vohibato	123867	2000	135.2	123.3	148.8
Madagascar	Haute Matsiatra	Vohibato	123867	2005	119.6	109.1	131.0
Madagascar	Haute Matsiatra	Vohibato	123867	2010	113.0	102.3	124.3
Madagascar	Haute Matsiatra	Vohibato	123867	2015	96.1	84.3	108.1
Madagascar	Ihorombe	Iakora	154579	2000	142.9	126.4	162.2
Madagascar	Ihorombe	Iakora	154579	2005	126.9	111.6	143.7
Madagascar	Ihorombe	Iakora	154579	2010	120.7	106.6	137.8
Madagascar	Ihorombe	Iakora	154579	2015	102.9	88.1	119.1
Madagascar	Ihorombe	Ihosy	154580	2000	128.9	114.6	143.8
Madagascar	Ihorombe	Ihosy	154580	2005	112.5	99.9	126.6
Madagascar	Ihorombe	Ihosy	154580	2010	103.5	92.5	116.9
Madagascar	Ihorombe	Ihosy	154580	2015	88.5	77.2	101.6
Madagascar	Ihorombe	Ivohibe	154581	2000	147.0	132.4	163.5
Madagascar	Ihorombe	Ivohibe	154581	2005	128.6	116.3	143.3
Madagascar	Ihorombe	Ivohibe	154581	2010	119.3	106.7	133.1
Madagascar	Ihorombe	Ivohibe	154581	2015	101.1	88.1	114.9
Madagascar	Itasy	Arivonimamo	154582	2000	90.3	82.5	98.2
Madagascar	Itasy	Arivonimamo	154582	2005	81.4	74.6	88.5
Madagascar	Itasy	Arivonimamo	154582	2010	83.6	76.0	92.4
Madagascar	Itasy	Arivonimamo	154582	2015	74.0	65.2	84.6
Madagascar	Itasy	Miarinarivo	154583	2000	93.0	84.4	102.6
Madagascar	Itasy	Miarinarivo	154583	2005	85.5	77.9	93.6
Madagascar	Itasy	Miarinarivo	154583	2010	86.8	78.1	96.0
Madagascar	Itasy	Miarinarivo	154583	2015	77.2	68.0	87.6
Madagascar	Itasy	Soavinandriana	154584	2000	98.5	88.6	109.5
Madagascar	Itasy	Soavinandriana	154584	2005	91.1	82.0	100.6
Madagascar	Itasy	Soavinandriana	154584	2010	87.9	78.4	98.7
Madagascar	Itasy	Soavinandriana	154584	2015	77.0	67.2	88.1
Madagascar	Melaky	Ambatomainty	154585	2000	111.8	94.4	130.9
Madagascar	Melaky	Ambatomainty	154585	2005	96.9	82.5	113.1
Madagascar	Melaky	Ambatomainty	154585	2010	87.1	73.6	102.5
Madagascar	Melaky	Ambatomainty	154585	2015	86.4	70.6	102.2
Madagascar	Melaky	Antsalova	154586	2000	116.7	100.1	135.2
Madagascar	Melaky	Antsalova	154586	2005	98.7	84.2	115.1
Madagascar	Melaky	Antsalova	154586	2010	87.4	74.5	102.3
Madagascar	Melaky	Antsalova	154586	2015	85.5	72.1	101.2
Madagascar	Melaky	Besalampy	154587	2000	118.8	99.9	139.9
Madagascar	Melaky	Besalampy	154587	2005	98.9	82.5	115.4
Madagascar	Melaky	Besalampy	154587	2010	89.4	74.1	104.8
Madagascar	Melaky	Besalampy	154587	2015	90.6	74.3	108.0
Madagascar	Melaky	Maintirano	154588	2000	112.0	96.1	129.1
Madagascar	Melaky	Maintirano	154588	2005	94.8	82.1	109.1
Madagascar	Melaky	Maintirano	154588	2010	83.1	71.1	97.2
Madagascar	Melaky	Maintirano	154588	2015	84.1	70.5	99.4
Madagascar	Melaky	Morafenobe	154589	2000	111.3	91.7	133.4
Madagascar	Melaky	Morafenobe	154589	2005	93.9	77.3	114.1
Madagascar	Melaky	Morafenobe	154589	2010	83.9	69.1	100.8
Madagascar	Melaky	Morafenobe	154589	2015	88.4	71.3	107.2
Madagascar	Menabe	Belo Sur Tsiribihina	154590	2000	118.6	101.5	137.3
Madagascar	Menabe	Belo Sur Tsiribihina	154590	2005	100.4	86.1	115.7
Madagascar	Menabe	Belo Sur Tsiribihina	154590	2010	86.8	73.6	100.3
Madagascar	Menabe	Belo Sur Tsiribihina	154590	2015	80.0	67.3	94.4
Madagascar	Menabe	Mahabo	154591	2000	130.1	111.7	151.9
Madagascar	Menabe	Mahabo	154591	2005	109.2	93.4	126.7

Admin 0	Admin 1	Admin 2	GAUL Code	Year	Under-5 mortality (per 1,000 livebirths) Estimate	Lower bound, 95% UI	Upper bound, 95% UI
Madagascar	Menabe	Mahabo	154591	2010	99.1	84.5	114.2
Madagascar	Menabe	Mahabo	154591	2015	94.7	80.2	110.2
Madagascar	Menabe	Manja	154592	2000	138.6	116.9	162.8
Madagascar	Menabe	Manja	154592	2005	111.6	95.0	132.3
Madagascar	Menabe	Manja	154592	2010	97.3	82.8	115.2
Madagascar	Menabe	Manja	154592	2015	90.5	75.2	107.3
Madagascar	Menabe	Miandrivazo	154593	2000	113.0	99.4	128.5
Madagascar	Menabe	Miandrivazo	154593	2005	97.4	85.8	110.6
Madagascar	Menabe	Miandrivazo	154593	2010	86.4	74.6	98.9
Madagascar	Menabe	Miandrivazo	154593	2015	82.4	70.5	95.5
Madagascar	Menabe	Morondava	154594	2000	119.0	100.6	139.6
Madagascar	Menabe	Morondava	154594	2005	100.6	85.1	117.8
Madagascar	Menabe	Morondava	154594	2010	87.3	74.3	103.5
Madagascar	Menabe	Morondava	154594	2015	79.3	66.5	94.6
Madagascar	Sava	Andapa	154595	2000	97.5	85.8	110.4
Madagascar	Sava	Andapa	154595	2005	82.5	72.9	93.3
Madagascar	Sava	Andapa	154595	2010	74.1	64.8	84.4
Madagascar	Sava	Andapa	154595	2015	70.5	60.2	82.3
Madagascar	Sava	Antalaha	154596	2000	97.5	84.5	112.6
Madagascar	Sava	Antalaha	154596	2005	85.6	74.2	98.9
Madagascar	Sava	Antalaha	154596	2010	75.0	64.5	86.6
Madagascar	Sava	Antalaha	154596	2015	68.0	56.9	80.4
Madagascar	Sava	Sambava	154597	2000	95.7	83.9	108.7
Madagascar	Sava	Sambava	154597	2005	82.4	71.8	94.0
Madagascar	Sava	Sambava	154597	2010	75.2	65.3	87.0
Madagascar	Sava	Sambava	154597	2015	70.3	60.5	81.6
Madagascar	Sava	Vohemar	154598	2000	98.6	85.7	112.5
Madagascar	Sava	Vohemar	154598	2005	84.1	72.7	95.9
Madagascar	Sava	Vohemar	154598	2010	77.1	66.6	89.1
Madagascar	Sava	Vohemar	154598	2015	73.5	62.6	86.0
Madagascar	Sofia	Analalava	154599	2000	103.7	90.7	118.6
Madagascar	Sofia	Analalava	154599	2005	86.3	75.0	97.4
Madagascar	Sofia	Analalava	154599	2010	77.9	67.2	88.7
Madagascar	Sofia	Analalava	154599	2015	74.3	62.8	86.4
Madagascar	Sofia	Antsohihy	154600	2000	100.0	86.7	114.8
Madagascar	Sofia	Antsohihy	154600	2005	85.3	74.2	97.7
Madagascar	Sofia	Antsohihy	154600	2010	78.0	66.9	90.6
Madagascar	Sofia	Antsohihy	154600	2015	74.8	62.4	88.6
Madagascar	Sofia	Bealanana	154601	2000	101.1	88.0	115.4
Madagascar	Sofia	Bealanana	154601	2005	81.7	71.5	93.4
Madagascar	Sofia	Bealanana	154601	2010	74.0	64.2	85.5
Madagascar	Sofia	Bealanana	154601	2015	73.4	62.4	85.3
Madagascar	Sofia	Befandriana Nord	154602	2000	103.5	91.1	116.6
Madagascar	Sofia	Befandriana Nord	154602	2005	85.8	75.9	96.8
Madagascar	Sofia	Befandriana Nord	154602	2010	75.5	65.5	86.1
Madagascar	Sofia	Befandriana Nord	154602	2015	73.4	61.8	85.7
Madagascar	Sofia	Mampikony	154603	2000	113.8	99.1	128.9
Madagascar	Sofia	Mampikony	154603	2005	94.5	83.4	107.5
Madagascar	Sofia	Mampikony	154603	2010	85.7	73.9	97.6
Madagascar	Sofia	Mampikony	154603	2015	77.3	65.4	89.8
Madagascar	Sofia	Mandritsara	154604	2000	112.0	97.4	127.2
Madagascar	Sofia	Mandritsara	154604	2005	92.1	80.1	104.3
Madagascar	Sofia	Mandritsara	154604	2010	81.6	70.0	93.0
Madagascar	Sofia	Mandritsara	154604	2015	73.4	62.7	85.1
Madagascar	Sofia	Port-Berge (Boriziny-Vaovao)	154605	2000	108.3	95.5	121.7
Madagascar	Sofia	Port-Berge (Boriziny-Vaovao)	154605	2005	90.5	80.4	101.3
Madagascar	Sofia	Port-Berge (Boriziny-Vaovao)	154605	2010	80.0	69.6	90.5
Madagascar	Sofia	Port-Berge (Boriziny-Vaovao)	154605	2015	74.4	62.6	86.4
Madagascar	Vakinankaratra	Ambatolampy	154606	2000	96.3	86.6	106.6
Madagascar	Vakinankaratra	Ambatolampy	154606	2005	89.2	79.9	99.3
Madagascar	Vakinankaratra	Ambatolampy	154606	2010	89.9	79.6	100.9
Madagascar	Vakinankaratra	Ambatolampy	154606	2015	76.8	66.7	87.8
Madagascar	Vakinankaratra	Antanifotsy	154607	2000	102.1	91.3	112.7
Madagascar	Vakinankaratra	Antanifotsy	154607	2005	94.4	84.4	104.6
Madagascar	Vakinankaratra	Antanifotsy	154607	2010	95.0	84.6	106.7
Madagascar	Vakinankaratra	Antanifotsy	154607	2015	81.3	70.2	94.0
Madagascar	Vakinankaratra	Antsirabe I	154608	2000	91.3	81.8	101.8
Madagascar	Vakinankaratra	Antsirabe I	154608	2005	83.6	75.0	93.3
Madagascar	Vakinankaratra	Antsirabe I	154608	2010	85.2	75.1	96.1
Madagascar	Vakinankaratra	Antsirabe I	154608	2015	74.2	64.4	86.6
Madagascar	Vakinankaratra	Antsirabe II	154609	2000	99.5	90.5	109.6
Madagascar	Vakinankaratra	Antsirabe II	154609	2005	89.7	81.4	98.7
Madagascar	Vakinankaratra	Antsirabe II	154609	2010	89.5	80.2	100.2
Madagascar	Vakinankaratra	Antsirabe II	154609	2015	78.7	68.3	91.0
Madagascar	Vakinankaratra	Betafo	123868	2000	98.6	88.5	110.5
Madagascar	Vakinankaratra	Betafo	123868	2005	89.9	80.8	100.3
Madagascar	Vakinankaratra	Betafo	123868	2010	89.8	79.8	101.2
Madagascar	Vakinankaratra	Betafo	123868	2015	78.8	68.2	90.7
Madagascar	Vakinankaratra	Faratsiho	154610	2000	93.9	84.9	103.5
Madagascar	Vakinankaratra	Faratsiho	154610	2005	86.1	77.8	94.6
Madagascar	Vakinankaratra	Faratsiho	154610	2010	86.2	76.8	96.5
Madagascar	Vakinankaratra	Faratsiho	154610	2015	75.3	66.1	86.6
Madagascar	Vakinankaratra	Mandoto	123869	2000	111.5	97.6	127.2
Madagascar	Vakinankaratra	Mandoto	123869	2005	99.9	87.6	112.9
Madagascar	Vakinankaratra	Mandoto	123869	2010	92.9	80.8	106.7

Admin 0	Admin 1	Admin 2	GAUL Code	Year	Under-5 mortality (per 1,000 livebirths)		
					Estimate	Lower bound, 95% UI	Upper bound, 95% UI
Madagascar	Vakinankaratra	Mandoto	123869	2015	83.1	71.0	96.6
Madagascar	Vatovavy Fitovinany	Ifanadiana	154611	2000	160.6	144.6	177.6
Madagascar	Vatovavy Fitovinany	Ifanadiana	154611	2005	143.7	130.9	158.5
Madagascar	Vatovavy Fitovinany	Ifanadiana	154611	2010	134.0	120.0	150.6
Madagascar	Vatovavy Fitovinany	Ifanadiana	154611	2015	108.0	94.1	124.4
Madagascar	Vatovavy Fitovinany	Ikongo	154612	2000	164.9	150.7	179.9
Madagascar	Vatovavy Fitovinany	Ikongo	154612	2005	144.9	132.6	157.8
Madagascar	Vatovavy Fitovinany	Ikongo	154612	2010	134.8	121.8	148.7
Madagascar	Vatovavy Fitovinany	Ikongo	154612	2015	115.7	101.8	130.9
Madagascar	Vatovavy Fitovinany	Manakara Atsimo	154613	2000	178.3	161.0	196.8
Madagascar	Vatovavy Fitovinany	Manakara Atsimo	154613	2005	153.1	137.6	169.7
Madagascar	Vatovavy Fitovinany	Manakara Atsimo	154613	2010	140.0	125.0	156.0
Madagascar	Vatovavy Fitovinany	Manakara Atsimo	154613	2015	122.7	107.5	140.0
Madagascar	Vatovavy Fitovinany	Mananjary	154614	2000	180.3	160.7	201.6
Madagascar	Vatovavy Fitovinany	Mananjary	154614	2005	157.2	140.7	175.3
Madagascar	Vatovavy Fitovinany	Mananjary	154614	2010	141.9	125.2	161.3
Madagascar	Vatovavy Fitovinany	Mananjary	154614	2015	113.8	98.6	133.5
Madagascar	Vatovavy Fitovinany	Nosy-Varika	154615	2000	168.4	149.9	187.4
Madagascar	Vatovavy Fitovinany	Nosy-Varika	154615	2005	149.4	132.2	166.0
Madagascar	Vatovavy Fitovinany	Nosy-Varika	154615	2010	142.1	124.8	160.7
Madagascar	Vatovavy Fitovinany	Nosy-Varika	154615	2015	125.4	108.7	145.9
Madagascar	Vatovavy Fitovinany	Vohipeno	154616	2000	177.4	161.1	194.3
Madagascar	Vatovavy Fitovinany	Vohipeno	154616	2005	150.8	137.3	166.8
Madagascar	Vatovavy Fitovinany	Vohipeno	154616	2010	135.9	122.0	150.8
Madagascar	Vatovavy Fitovinany	Vohipeno	154616	2015	125.5	109.6	142.9
Malawi	Central Region	Dedza	19307	2000	185.7	178.6	193.0
Malawi	Central Region	Dedza	19307	2005	143.0	136.9	149.1
Malawi	Central Region	Dedza	19307	2010	109.1	103.4	115.0
Malawi	Central Region	Dedza	19307	2015	80.0	74.3	86.7
Malawi	Central Region	Dowa	19308	2000	166.9	161.2	172.9
Malawi	Central Region	Dowa	19308	2005	123.4	118.9	128.4
Malawi	Central Region	Dowa	19308	2010	98.8	94.2	103.6
Malawi	Central Region	Dowa	19308	2015	75.3	69.8	81.1
Malawi	Central Region	Kasungu	19309	2000	170.6	163.8	177.6
Malawi	Central Region	Kasungu	19309	2005	126.7	120.9	132.3
Malawi	Central Region	Kasungu	19309	2010	105.3	100.0	110.5
Malawi	Central Region	Kasungu	19309	2015	83.0	76.6	90.0
Malawi	Central Region	Lilongwe	19310	2000	162.5	157.0	168.5
Malawi	Central Region	Lilongwe	19310	2005	127.6	122.5	133.1
Malawi	Central Region	Lilongwe	19310	2010	102.5	98.0	107.5
Malawi	Central Region	Lilongwe	19310	2015	76.8	71.0	82.9
Malawi	Central Region	Mchinji	19311	2000	184.7	176.3	193.2
Malawi	Central Region	Mchinji	19311	2005	136.9	130.5	143.8
Malawi	Central Region	Mchinji	19311	2010	115.5	109.1	121.8
Malawi	Central Region	Mchinji	19311	2015	96.1	88.3	104.6
Malawi	Central Region	Nkhotakota	19312	2000	156.5	148.9	164.2
Malawi	Central Region	Nkhotakota	19312	2005	115.6	109.9	121.6
Malawi	Central Region	Nkhotakota	19312	2010	90.4	85.3	95.8
Malawi	Central Region	Nkhotakota	19312	2015	67.2	61.3	72.9
Malawi	Central Region	Ntcheu	19313	2000	166.5	159.4	173.8
Malawi	Central Region	Ntcheu	19313	2005	133.3	127.5	139.2
Malawi	Central Region	Ntcheu	19313	2010	100.1	94.8	105.9
Malawi	Central Region	Ntcheu	19313	2015	73.5	67.7	79.1
Malawi	Central Region	Ntchisi	19314	2000	165.0	158.0	173.3
Malawi	Central Region	Ntchisi	19314	2005	121.9	116.4	127.4
Malawi	Central Region	Ntchisi	19314	2010	96.5	91.6	101.8
Malawi	Central Region	Ntchisi	19314	2015	73.6	67.7	79.3
Malawi	Central Region	Salima	19315	2000	171.8	164.6	179.3
Malawi	Central Region	Salima	19315	2005	128.8	123.0	134.8
Malawi	Central Region	Salima	19315	2010	98.5	93.0	104.1
Malawi	Central Region	Salima	19315	2015	75.4	69.4	81.7
Malawi	Northern Region	Chitipa	19316	2000	130.8	123.4	138.9
Malawi	Northern Region	Chitipa	19316	2005	100.4	94.5	106.2
Malawi	Northern Region	Chitipa	19316	2010	77.7	72.3	83.1
Malawi	Northern Region	Chitipa	19316	2015	67.9	60.8	75.2
Malawi	Northern Region	Karonga	19317	2000	117.9	111.6	125.1
Malawi	Northern Region	Karonga	19317	2005	94.7	89.1	100.8
Malawi	Northern Region	Karonga	19317	2010	75.6	70.5	80.8
Malawi	Northern Region	Karonga	19317	2015	64.2	57.6	70.7
Malawi	Northern Region	Likoma	42172	2000	125.0	111.9	139.8
Malawi	Northern Region	Likoma	42172	2005	97.5	87.0	107.9
Malawi	Northern Region	Likoma	42172	2010	74.5	66.3	83.6
Malawi	Northern Region	Likoma	42172	2015	59.4	52.3	67.3
Malawi	Northern Region	Mzimba	19318	2000	142.9	136.9	149.6
Malawi	Northern Region	Mzimba	19318	2005	110.5	105.4	116.1
Malawi	Northern Region	Mzimba	19318	2010	89.9	84.7	95.1
Malawi	Northern Region	Mzimba	19318	2015	70.9	65.0	77.0
Malawi	Northern Region	Nkhata Bay	42173	2000	130.4	123.1	138.0
Malawi	Northern Region	Nkhata Bay	42173	2005	102.6	96.6	109.0
Malawi	Northern Region	Nkhata Bay	42173	2010	83.1	77.7	88.9
Malawi	Northern Region	Nkhata Bay	42173	2015	62.7	56.8	68.8
Malawi	Northern Region	Rumphi	19320	2000	118.9	112.0	126.3
Malawi	Northern Region	Rumphi	19320	2005	94.1	88.6	100.0
Malawi	Northern Region	Rumphi	19320	2010	78.0	72.3	83.7
Malawi	Northern Region	Rumphi	19320	2015	65.0	58.7	71.6

Admin 0	Admin 1	Admin 2	GAUL Code	Year	Under-5 mortality (per 1,000 livebirths)		
					Estimate	Lower bound, 95% UI	Upper bound, 95% UI
Malawi	Southern Region	Balaka	42174	2000	165.2	158.1	172.4
Malawi	Southern Region	Balaka	42174	2005	128.7	123.2	134.6
Malawi	Southern Region	Balaka	42174	2010	96.8	91.7	101.7
Malawi	Southern Region	Balaka	42174	2015	71.0	65.5	76.6
Malawi	Southern Region	Blantyre	19321	2000	137.7	133.0	142.9
Malawi	Southern Region	Blantyre	19321	2005	111.9	107.7	116.3
Malawi	Southern Region	Blantyre	19321	2010	92.9	89.0	97.5
Malawi	Southern Region	Blantyre	19321	2015	70.7	65.4	76.1
Malawi	Southern Region	Chikwawa	19322	2000	186.6	179.0	194.3
Malawi	Southern Region	Chikwawa	19322	2005	125.5	119.2	131.5
Malawi	Southern Region	Chikwawa	19322	2010	97.8	92.0	103.6
Malawi	Southern Region	Chikwawa	19322	2015	75.2	69.2	81.7
Malawi	Southern Region	Chiradzulu	19323	2000	173.2	167.7	179.1
Malawi	Southern Region	Chiradzulu	19323	2005	128.7	123.9	133.1
Malawi	Southern Region	Chiradzulu	19323	2010	104.4	100.2	109.1
Malawi	Southern Region	Chiradzulu	19323	2015	76.5	71.1	82.5
Malawi	Southern Region	Machinga	42175	2000	162.4	155.4	169.5
Malawi	Southern Region	Machinga	42175	2005	123.0	117.3	128.9
Malawi	Southern Region	Machinga	42175	2010	93.2	88.2	98.9
Malawi	Southern Region	Machinga	42175	2015	70.5	64.5	76.6
Malawi	Southern Region	Mangochi	19325	2000	169.4	162.2	176.3
Malawi	Southern Region	Mangochi	19325	2005	132.5	126.9	138.5
Malawi	Southern Region	Mangochi	19325	2010	100.2	94.8	105.9
Malawi	Southern Region	Mangochi	19325	2015	75.0	69.0	80.9
Malawi	Southern Region	Mulanje	42176	2000	191.9	185.1	198.5
Malawi	Southern Region	Mulanje	42176	2005	144.5	138.6	150.3
Malawi	Southern Region	Mulanje	42176	2010	116.3	110.6	121.8
Malawi	Southern Region	Mulanje	42176	2015	83.8	77.6	90.9
Malawi	Southern Region	Mwanza	65271	2000	161.0	153.5	169.5
Malawi	Southern Region	Mwanza	65271	2005	133.8	127.2	140.6
Malawi	Southern Region	Mwanza	65271	2010	97.5	91.9	103.6
Malawi	Southern Region	Mwanza	65271	2015	77.4	70.8	84.8
Malawi	Southern Region	Neno	65270	2000	161.0	153.4	168.6
Malawi	Southern Region	Neno	65270	2005	131.1	125.4	137.2
Malawi	Southern Region	Neno	65270	2010	96.5	91.6	101.8
Malawi	Southern Region	Neno	65270	2015	74.3	68.3	80.3
Malawi	Southern Region	Nsanje	19328	2000	196.2	187.4	205.5
Malawi	Southern Region	Nsanje	19328	2005	140.4	133.3	148.0
Malawi	Southern Region	Nsanje	19328	2010	100.4	94.5	107.3
Malawi	Southern Region	Nsanje	19328	2015	79.7	72.8	86.7
Malawi	Southern Region	Phalombe	42177	2000	187.1	179.0	195.5
Malawi	Southern Region	Phalombe	42177	2005	144.5	138.1	151.0
Malawi	Southern Region	Phalombe	42177	2010	115.0	108.5	121.5
Malawi	Southern Region	Phalombe	42177	2015	85.3	78.7	92.6
Malawi	Southern Region	Thyolo	19329	2000	195.6	189.2	201.9
Malawi	Southern Region	Thyolo	19329	2005	130.1	124.9	135.1
Malawi	Southern Region	Thyolo	19329	2010	104.2	99.3	109.2
Malawi	Southern Region	Thyolo	19329	2015	76.6	70.6	82.7
Malawi	Southern Region	Zomba	19330	2000	165.8	160.0	172.1
Malawi	Southern Region	Zomba	19330	2005	125.4	120.4	130.6
Malawi	Southern Region	Zomba	19330	2010	101.1	96.6	106.2
Malawi	Southern Region	Zomba	19330	2015	74.3	68.9	79.9
Mali	Bamako	Bamako	81878	2000	111.6	105.7	118.2
Mali	Bamako	Bamako	81878	2005	97.5	92.1	103.3
Mali	Bamako	Bamako	81878	2010	83.0	77.4	89.1
Mali	Bamako	Bamako	81878	2015	71.9	65.4	79.1
Mali	Gao	Ansongo	19372	2000	170.7	153.5	188.6
Mali	Gao	Ansongo	19372	2005	164.7	147.6	184.2
Mali	Gao	Ansongo	19372	2010	162.3	143.2	182.4
Mali	Gao	Ansongo	19372	2015	137.1	118.7	157.7
Mali	Gao	Bourem	19373	2000	142.9	127.0	159.5
Mali	Gao	Bourem	19373	2005	128.2	113.8	142.5
Mali	Gao	Bourem	19373	2010	116.0	101.5	130.6
Mali	Gao	Bourem	19373	2015	101.3	87.0	117.8
Mali	Gao	Gao	19374	2000	139.5	126.8	154.3
Mali	Gao	Gao	19374	2005	127.3	114.8	141.4
Mali	Gao	Gao	19374	2010	124.6	110.1	140.0
Mali	Gao	Gao	19374	2015	111.0	96.2	128.7
Mali	Gao	Menaka	19375	2000	136.1	118.9	153.3
Mali	Gao	Menaka	19375	2005	126.7	111.4	143.6
Mali	Gao	Menaka	19375	2010	131.4	114.8	150.7
Mali	Gao	Menaka	19375	2015	120.9	103.7	141.5
Mali	Kayes	Bafoulabe	19376	2000	223.4	208.9	239.0
Mali	Kayes	Bafoulabe	19376	2005	181.0	167.4	194.7
Mali	Kayes	Bafoulabe	19376	2010	161.8	148.1	175.7
Mali	Kayes	Bafoulabe	19376	2015	146.9	131.2	164.1
Mali	Kayes	Diema	19377	2000	223.0	207.5	238.1
Mali	Kayes	Diema	19377	2005	177.8	164.9	190.1
Mali	Kayes	Diema	19377	2010	152.4	139.4	165.2
Mali	Kayes	Diema	19377	2015	135.9	121.5	152.5
Mali	Kayes	Kayes	19378	2000	167.2	156.1	178.7
Mali	Kayes	Kayes	19378	2005	127.8	119.2	137.6
Mali	Kayes	Kayes	19378	2010	105.8	97.7	114.6
Mali	Kayes	Kayes	19378	2015	93.8	83.9	104.6
Mali	Kayes	Kenieba	19379	2000	274.7	255.6	294.3

Admin 0	Admin 1	Admin 2	GAUL Code	Year	Under-5 mortality (per 1,000 livebirths)		
					Estimate	Lower bound, 95% UI	Upper bound, 95% UI
Mali	Kayes	Kenieba	19379	2005	227.4	210.2	245.2
Mali	Kayes	Kenieba	19379	2010	193.1	176.2	211.5
Mali	Kayes	Kenieba	19379	2015	171.2	151.6	191.4
Mali	Kayes	Kita	19380	2000	222.0	209.7	236.7
Mali	Kayes	Kita	19380	2005	194.8	182.1	208.0
Mali	Kayes	Kita	19380	2010	177.4	165.1	191.5
Mali	Kayes	Kita	19380	2015	160.4	144.3	176.9
Mali	Kayes	Nioro	19381	2000	202.2	187.8	217.8
Mali	Kayes	Nioro	19381	2005	154.7	144.0	166.7
Mali	Kayes	Nioro	19381	2010	133.2	121.4	146.0
Mali	Kayes	Nioro	19381	2015	116.9	103.8	131.3
Mali	Kayes	Yelimane	19382	2000	160.9	147.0	174.9
Mali	Kayes	Yelimane	19382	2005	123.9	114.0	134.1
Mali	Kayes	Yelimane	19382	2010	105.7	96.2	115.5
Mali	Kayes	Yelimane	19382	2015	94.3	82.7	106.8
Mali	Kidal	Abeibara	19383	2000	98.3	78.8	120.8
Mali	Kidal	Abeibara	19383	2005	87.8	70.1	107.2
Mali	Kidal	Abeibara	19383	2010	84.6	67.1	103.0
Mali	Kidal	Abeibara	19383	2015	84.6	66.5	104.9
Mali	Kidal	Kidal	19384	2000	100.0	84.9	115.5
Mali	Kidal	Kidal	19384	2005	85.7	72.1	99.8
Mali	Kidal	Kidal	19384	2010	75.7	62.6	88.8
Mali	Kidal	Kidal	19384	2015	77.7	63.5	93.8
Mali	Kidal	Tessalit	19385	2000	102.4	82.6	123.7
Mali	Kidal	Tessalit	19385	2005	90.1	71.8	111.5
Mali	Kidal	Tessalit	19385	2010	84.6	68.0	104.6
Mali	Kidal	Tessalit	19385	2015	81.1	64.2	100.2
Mali	Kidal	Tin-Essako	19386	2000	102.5	84.3	124.5
Mali	Kidal	Tin-Essako	19386	2005	91.8	73.9	112.6
Mali	Kidal	Tin-Essako	19386	2010	88.2	71.2	108.7
Mali	Kidal	Tin-Essako	19386	2015	87.8	70.5	110.7
Mali	Koulikoro	Banamba	19387	2000	206.2	192.7	221.1
Mali	Koulikoro	Banamba	19387	2005	179.9	166.8	193.6
Mali	Koulikoro	Banamba	19387	2010	146.9	135.3	159.2
Mali	Koulikoro	Banamba	19387	2015	130.6	117.2	146.0
Mali	Koulikoro	Dioila	19388	2000	201.9	191.3	214.3
Mali	Koulikoro	Dioila	19388	2005	181.3	171.3	192.3
Mali	Koulikoro	Dioila	19388	2010	152.3	142.7	162.9
Mali	Koulikoro	Dioila	19388	2015	133.7	121.0	146.9
Mali	Koulikoro	Kangaba	19389	2000	193.1	179.1	209.3
Mali	Koulikoro	Kangaba	19389	2005	174.8	161.0	189.2
Mali	Koulikoro	Kangaba	19389	2010	152.0	139.1	166.7
Mali	Koulikoro	Kangaba	19389	2015	138.5	122.6	155.7
Mali	Koulikoro	Kati	19390	2000	155.7	149.1	163.1
Mali	Koulikoro	Kati	19390	2005	131.9	125.5	138.7
Mali	Koulikoro	Kati	19390	2010	110.5	103.9	117.0
Mali	Koulikoro	Kati	19390	2015	101.2	92.5	110.3
Mali	Koulikoro	Kolokani	19391	2000	192.4	179.0	206.1
Mali	Koulikoro	Kolokani	19391	2005	176.9	163.9	190.8
Mali	Koulikoro	Kolokani	19391	2010	148.6	136.1	162.1
Mali	Koulikoro	Kolokani	19391	2015	132.5	118.5	147.5
Mali	Koulikoro	Koulikoro	19392	2000	186.6	175.6	198.4
Mali	Koulikoro	Koulikoro	19392	2005	163.7	153.3	174.7
Mali	Koulikoro	Koulikoro	19392	2010	132.0	122.7	141.7
Mali	Koulikoro	Koulikoro	19392	2015	120.2	109.3	133.7
Mali	Koulikoro	Nara	19393	2000	203.3	190.0	218.0
Mali	Koulikoro	Nara	19393	2005	168.0	155.5	181.6
Mali	Koulikoro	Nara	19393	2010	146.7	134.9	160.5
Mali	Koulikoro	Nara	19393	2015	130.9	116.8	147.0
Mali	Mopti	Bandiagara	19394	2000	197.5	187.8	208.2
Mali	Mopti	Bandiagara	19394	2005	163.5	154.5	173.3
Mali	Mopti	Bandiagara	19394	2010	144.6	135.5	154.3
Mali	Mopti	Bandiagara	19394	2015	124.8	112.6	138.3
Mali	Mopti	Bankass	19395	2000	197.5	184.3	211.1
Mali	Mopti	Bankass	19395	2005	170.0	158.2	182.4
Mali	Mopti	Bankass	19395	2010	140.2	129.5	151.3
Mali	Mopti	Bankass	19395	2015	120.6	107.8	133.4
Mali	Mopti	Djenne	19396	2000	207.7	194.1	222.2
Mali	Mopti	Djenne	19396	2005	176.3	164.6	188.4
Mali	Mopti	Djenne	19396	2010	144.8	134.0	156.2
Mali	Mopti	Djenne	19396	2015	122.5	109.8	136.6
Mali	Mopti	Douentza	19397	2000	206.2	192.7	219.9
Mali	Mopti	Douentza	19397	2005	170.1	159.4	182.1
Mali	Mopti	Douentza	19397	2010	155.2	142.7	168.4
Mali	Mopti	Douentza	19397	2015	133.8	120.1	149.3
Mali	Mopti	Koro	19398	2000	193.2	182.2	204.8
Mali	Mopti	Koro	19398	2005	162.9	152.6	173.7
Mali	Mopti	Koro	19398	2010	143.0	132.8	153.7
Mali	Mopti	Koro	19398	2015	122.8	110.5	135.7
Mali	Mopti	Mopti	19399	2000	212.0	201.4	224.4
Mali	Mopti	Mopti	19399	2005	178.4	167.6	189.4
Mali	Mopti	Mopti	19399	2010	149.8	139.0	160.7
Mali	Mopti	Mopti	19399	2015	131.4	118.6	145.6
Mali	Mopti	Tenenkou	19400	2000	239.1	223.2	255.8
Mali	Mopti	Tenenkou	19400	2005	201.4	186.4	217.6

Admin 0	Admin 1	Admin 2	GAUL Code	Year	Under-5 mortality (per 1,000 livebirths)		
					Estimate	Lower bound, 95% UI	Upper bound, 95% UI
Mali	Mopti	Tenenkou	19400	2010	163.5	149.5	178.0
Mali	Mopti	Tenenkou	19400	2015	147.8	131.7	165.2
Mali	Mopti	Youwarou	19401	2000	253.3	235.7	271.5
Mali	Mopti	Youwarou	19401	2005	207.2	191.9	224.7
Mali	Mopti	Youwarou	19401	2010	184.4	167.3	202.7
Mali	Mopti	Youwarou	19401	2015	161.0	142.2	181.9
Mali	Segou	Baroueli	19402	2000	216.8	204.2	230.8
Mali	Segou	Baroueli	19402	2005	189.5	177.4	202.1
Mali	Segou	Baroueli	19402	2010	154.5	143.6	165.4
Mali	Segou	Baroueli	19402	2015	134.8	121.3	148.6
Mali	Segou	Bla	19403	2000	225.0	213.3	237.2
Mali	Segou	Bla	19403	2005	207.2	195.3	219.8
Mali	Segou	Bla	19403	2010	172.5	161.2	183.6
Mali	Segou	Bla	19403	2015	143.4	129.8	157.9
Mali	Segou	Macina	19404	2000	239.0	224.8	254.1
Mali	Segou	Macina	19404	2005	203.8	190.8	218.8
Mali	Segou	Macina	19404	2010	163.9	151.5	178.0
Mali	Segou	Macina	19404	2015	144.9	129.8	160.9
Mali	Segou	Niono	19405	2000	205.8	190.3	221.8
Mali	Segou	Niono	19405	2005	175.2	160.7	189.8
Mali	Segou	Niono	19405	2010	140.0	127.7	153.3
Mali	Segou	Niono	19405	2015	133.1	118.3	149.1
Mali	Segou	San	19406	2000	198.5	187.7	210.2
Mali	Segou	San	19406	2005	177.6	167.3	188.6
Mali	Segou	San	19406	2010	150.0	140.1	161.3
Mali	Segou	San	19406	2015	126.3	114.6	140.3
Mali	Segou	Segou	19407	2000	227.0	215.2	239.3
Mali	Segou	Segou	19407	2005	196.4	184.7	208.3
Mali	Segou	Segou	19407	2010	157.4	146.7	168.3
Mali	Segou	Segou	19407	2015	138.7	126.1	153.0
Mali	Segou	Tominian	19408	2000	187.1	176.5	198.4
Mali	Segou	Tominian	19408	2005	169.7	160.2	180.4
Mali	Segou	Tominian	19408	2010	141.6	132.0	153.0
Mali	Segou	Tominian	19408	2015	119.8	108.4	132.4
Mali	Sikasso	Bougouni	19409	2000	202.2	191.7	213.1
Mali	Sikasso	Bougouni	19409	2005	179.8	169.8	190.1
Mali	Sikasso	Bougouni	19409	2010	156.8	145.5	167.6
Mali	Sikasso	Bougouni	19409	2015	133.5	120.5	148.2
Mali	Sikasso	Kadiolo	19410	2000	220.4	205.6	235.8
Mali	Sikasso	Kadiolo	19410	2005	207.8	193.0	223.3
Mali	Sikasso	Kadiolo	19410	2010	174.4	160.5	189.2
Mali	Sikasso	Kadiolo	19410	2015	150.5	135.4	168.4
Mali	Sikasso	Kolondieba	19411	2000	220.4	207.5	235.3
Mali	Sikasso	Kolondieba	19411	2005	196.1	182.3	210.2
Mali	Sikasso	Kolondieba	19411	2010	166.1	153.1	179.6
Mali	Sikasso	Kolondieba	19411	2015	139.3	124.7	157.2
Mali	Sikasso	Koutiala	19412	2000	224.2	211.7	237.7
Mali	Sikasso	Koutiala	19412	2005	199.5	187.0	212.4
Mali	Sikasso	Koutiala	19412	2010	165.0	154.0	176.6
Mali	Sikasso	Koutiala	19412	2015	139.0	126.0	153.4
Mali	Sikasso	Sikasso	19413	2000	208.5	197.4	219.3
Mali	Sikasso	Sikasso	19413	2005	187.6	177.0	198.8
Mali	Sikasso	Sikasso	19413	2010	152.9	142.7	163.1
Mali	Sikasso	Sikasso	19413	2015	133.1	120.2	146.8
Mali	Sikasso	Yanfolila	19414	2000	198.6	185.9	212.8
Mali	Sikasso	Yanfolila	19414	2005	180.5	168.4	193.9
Mali	Sikasso	Yanfolila	19414	2010	157.1	144.3	170.0
Mali	Sikasso	Yanfolila	19414	2015	135.9	120.4	152.2
Mali	Sikasso	Yorosso	19415	2000	206.9	194.1	221.8
Mali	Sikasso	Yorosso	19415	2005	188.6	176.9	202.3
Mali	Sikasso	Yorosso	19415	2010	159.6	147.7	173.4
Mali	Sikasso	Yorosso	19415	2015	136.1	123.1	151.3
Mali	Tombouctou	Dire	19416	2000	223.5	205.8	243.4
Mali	Tombouctou	Dire	19416	2005	187.6	170.3	204.9
Mali	Tombouctou	Dire	19416	2010	167.9	149.8	186.6
Mali	Tombouctou	Dire	19416	2015	146.9	126.7	168.8
Mali	Tombouctou	Goundam	19417	2000	215.8	198.5	233.5
Mali	Tombouctou	Goundam	19417	2005	183.4	166.7	200.5
Mali	Tombouctou	Goundam	19417	2010	161.5	144.7	179.4
Mali	Tombouctou	Goundam	19417	2015	143.4	124.9	163.0
Mali	Tombouctou	Gourma-Rharous	19418	2000	183.8	170.1	199.9
Mali	Tombouctou	Gourma-Rharous	19418	2005	156.8	144.3	171.4
Mali	Tombouctou	Gourma-Rharous	19418	2010	144.8	131.8	159.9
Mali	Tombouctou	Gourma-Rharous	19418	2015	128.0	114.5	144.5
Mali	Tombouctou	Niafunke	19419	2000	237.3	221.2	253.8
Mali	Tombouctou	Niafunke	19419	2005	200.7	185.4	216.2
Mali	Tombouctou	Niafunke	19419	2010	185.9	169.3	202.7
Mali	Tombouctou	Niafunke	19419	2015	161.3	142.2	181.8
Mali	Tombouctou	Tombouctou	19420	2000	158.1	143.4	174.8
Mali	Tombouctou	Tombouctou	19420	2005	138.2	124.1	153.7
Mali	Tombouctou	Tombouctou	19420	2010	123.9	109.4	140.0
Mali	Tombouctou	Tombouctou	19420	2015	112.8	97.6	130.1
Mauritania	Adrar	Aoujeft	19490	2000	87.4	76.6	99.5
Mauritania	Adrar	Aoujeft	19490	2005	88.8	78.1	101.1
Mauritania	Adrar	Aoujeft	19490	2010	72.1	62.3	83.8

Admin 0	Admin 1	Admin 2	GAUL Code	Year	Under-5 mortality (per 1,000 livebirths)		
					Estimate	Lower bound, 95% UI	Upper bound, 95% UI
Mauritania	Adrar	Aoujeft	19490	2015	58.1	49.0	67.8
Mauritania	Adrar	Atar	19491	2000	89.9	78.9	103.0
Mauritania	Adrar	Atar	19491	2005	90.1	78.7	102.4
Mauritania	Adrar	Atar	19491	2010	73.6	63.3	84.6
Mauritania	Adrar	Atar	19491	2015	59.2	50.3	68.7
Mauritania	Adrar	Chinguity	123633	2000	81.9	71.0	94.5
Mauritania	Adrar	Chinguity	123633	2005	84.4	74.0	96.4
Mauritania	Adrar	Chinguity	123633	2010	69.3	60.1	79.5
Mauritania	Adrar	Chinguity	123633	2015	56.7	48.8	65.9
Mauritania	Assaba	Barkeol	19494	2000	92.7	85.0	100.9
Mauritania	Assaba	Barkeol	19494	2005	80.2	73.4	88.0
Mauritania	Assaba	Barkeol	19494	2010	67.8	60.7	75.8
Mauritania	Assaba	Barkeol	19494	2015	56.7	49.3	64.5
Mauritania	Assaba	Boumdeid	19495	2000	78.8	68.7	90.3
Mauritania	Assaba	Boumdeid	19495	2005	69.5	60.8	79.7
Mauritania	Assaba	Boumdeid	19495	2010	60.0	51.7	69.4
Mauritania	Assaba	Boumdeid	19495	2015	49.4	41.9	58.0
Mauritania	Assaba	Guerrou	19496	2000	87.4	78.4	97.0
Mauritania	Assaba	Guerrou	19496	2005	76.1	68.5	84.6
Mauritania	Assaba	Guerrou	19496	2010	64.6	56.8	73.0
Mauritania	Assaba	Guerrou	19496	2015	52.7	44.9	61.2
Mauritania	Assaba	Kankossa	19497	2000	90.0	81.8	98.1
Mauritania	Assaba	Kankossa	19497	2005	81.1	73.5	88.5
Mauritania	Assaba	Kankossa	19497	2010	65.3	58.5	72.4
Mauritania	Assaba	Kankossa	19497	2015	55.5	48.3	63.2
Mauritania	Assaba	Kiffa	19498	2000	82.8	74.7	90.8
Mauritania	Assaba	Kiffa	19498	2005	73.6	66.2	80.9
Mauritania	Assaba	Kiffa	19498	2010	62.0	55.1	69.5
Mauritania	Assaba	Kiffa	19498	2015	50.7	43.8	58.1
Mauritania	Brakna	Aleg	19499	2000	98.1	89.0	107.8
Mauritania	Brakna	Aleg	19499	2005	83.2	75.7	91.1
Mauritania	Brakna	Aleg	19499	2010	63.2	56.6	70.8
Mauritania	Brakna	Aleg	19499	2015	50.0	43.6	57.0
Mauritania	Brakna	Bababe	19500	2000	103.3	94.4	112.8
Mauritania	Brakna	Bababe	19500	2005	85.4	77.6	93.0
Mauritania	Brakna	Bababe	19500	2010	61.8	55.5	69.2
Mauritania	Brakna	Bababe	19500	2015	46.3	39.8	53.0
Mauritania	Brakna	Boghe	19501	2000	108.4	100.1	117.3
Mauritania	Brakna	Boghe	19501	2005	83.2	76.8	90.0
Mauritania	Brakna	Boghe	19501	2010	57.8	52.4	64.0
Mauritania	Brakna	Boghe	19501	2015	41.8	36.5	48.1
Mauritania	Brakna	M'Bagne	19502	2000	107.7	98.8	116.0
Mauritania	Brakna	M'Bagne	19502	2005	90.2	82.6	97.5
Mauritania	Brakna	M'Bagne	19502	2010	64.8	58.5	72.3
Mauritania	Brakna	M'Bagne	19502	2015	49.7	43.3	56.6
Mauritania	Brakna	Maghta Lahjar	19503	2000	96.3	85.9	108.6
Mauritania	Brakna	Maghta Lahjar	19503	2005	84.3	74.6	95.2
Mauritania	Brakna	Maghta Lahjar	19503	2010	68.3	59.0	78.3
Mauritania	Brakna	Maghta Lahjar	19503	2015	55.4	47.5	64.1
Mauritania	Dakhlet-Nouadhibou	Nouadhibou	19504	2000	78.3	68.2	88.4
Mauritania	Dakhlet-Nouadhibou	Nouadhibou	19504	2005	76.8	67.1	87.2
Mauritania	Dakhlet-Nouadhibou	Nouadhibou	19504	2010	61.6	53.4	70.5
Mauritania	Dakhlet-Nouadhibou	Nouadhibou	19504	2015	49.3	42.0	57.1
Mauritania	Gorgol	Kaedi	19505	2000	108.0	100.4	115.8
Mauritania	Gorgol	Kaedi	19505	2005	89.5	83.4	95.9
Mauritania	Gorgol	Kaedi	19505	2010	68.6	62.6	75.1
Mauritania	Gorgol	Kaedi	19505	2015	56.9	50.4	64.3
Mauritania	Gorgol	M'Bout	19506	2000	102.3	94.2	110.9
Mauritania	Gorgol	M'Bout	19506	2005	84.4	77.5	91.1
Mauritania	Gorgol	M'Bout	19506	2010	70.1	63.4	77.1
Mauritania	Gorgol	M'Bout	19506	2015	59.1	51.9	67.5
Mauritania	Gorgol	Maghama	19507	2000	104.6	97.3	112.2
Mauritania	Gorgol	Maghama	19507	2005	86.2	79.8	92.7
Mauritania	Gorgol	Maghama	19507	2010	67.6	61.6	74.4
Mauritania	Gorgol	Maghama	19507	2015	56.5	49.8	63.7
Mauritania	Gorgol	Monguel	19508	2000	105.0	95.3	114.8
Mauritania	Gorgol	Monguel	19508	2005	87.4	79.7	95.6
Mauritania	Gorgol	Monguel	19508	2010	70.7	63.3	79.6
Mauritania	Gorgol	Monguel	19508	2015	59.5	52.0	68.2
Mauritania	Guidimakha	Ould Yenge	19509	2000	101.8	93.2	111.3
Mauritania	Guidimakha	Ould Yenge	19509	2005	86.6	79.5	94.3
Mauritania	Guidimakha	Ould Yenge	19509	2010	70.4	63.4	78.5
Mauritania	Guidimakha	Ould Yenge	19509	2015	59.8	52.4	69.0
Mauritania	Guidimakha	Selibaby	19510	2000	110.4	103.8	118.6
Mauritania	Guidimakha	Selibaby	19510	2005	94.2	88.7	100.8
Mauritania	Guidimakha	Selibaby	19510	2010	73.0	67.4	79.2
Mauritania	Guidimakha	Selibaby	19510	2015	60.1	53.9	67.2
Mauritania	Hodh Ech Chargi	Amourj	19511	2000	114.9	103.8	127.0
Mauritania	Hodh Ech Chargi	Amourj	19511	2005	107.9	97.7	120.5
Mauritania	Hodh Ech Chargi	Amourj	19511	2010	91.3	81.6	102.1
Mauritania	Hodh Ech Chargi	Amourj	19511	2015	82.7	72.2	94.3
Mauritania	Hodh Ech Chargi	Bassikounou	19512	2000	115.5	102.3	131.5
Mauritania	Hodh Ech Chargi	Bassikounou	19512	2005	113.3	99.8	128.7
Mauritania	Hodh Ech Chargi	Bassikounou	19512	2010	91.9	80.6	105.2
Mauritania	Hodh Ech Chargi	Bassikounou	19512	2015	85.1	73.2	99.2

Admin 0	Admin 1	Admin 2	GAUL Code	Year	Under-5 mortality (per 1,000 livebirths)		
					Estimate	Lower bound, 95% UI	Upper bound, 95% UI
Mauritania	Hodh Ech Chargi	Djigueni	19513	2000	101.4	90.6	112.6
Mauritania	Hodh Ech Chargi	Djigueni	19513	2005	90.8	82.0	101.8
Mauritania	Hodh Ech Chargi	Djigueni	19513	2010	80.8	71.3	90.8
Mauritania	Hodh Ech Chargi	Djigueni	19513	2015	69.8	59.7	80.7
Mauritania	Hodh Ech Chargi	Nema	19514	2000	98.9	88.7	110.6
Mauritania	Hodh Ech Chargi	Nema	19514	2005	91.4	81.6	102.2
Mauritania	Hodh Ech Chargi	Nema	19514	2010	79.8	69.6	90.8
Mauritania	Hodh Ech Chargi	Nema	19514	2015	66.0	56.9	76.7
Mauritania	Hodh Ech Chargi	Oualata	19515	2000	95.5	83.3	109.6
Mauritania	Hodh Ech Chargi	Oualata	19515	2005	92.3	79.6	106.5
Mauritania	Hodh Ech Chargi	Oualata	19515	2010	77.5	66.3	89.4
Mauritania	Hodh Ech Chargi	Oualata	19515	2015	63.9	54.1	74.4
Mauritania	Hodh Ech Chargi	Timbedra	19516	2000	98.5	88.9	108.9
Mauritania	Hodh Ech Chargi	Timbedra	19516	2005	90.0	80.4	99.8
Mauritania	Hodh Ech Chargi	Timbedra	19516	2010	81.4	72.5	91.2
Mauritania	Hodh Ech Chargi	Timbedra	19516	2015	70.2	60.8	80.9
Mauritania	Hodh El Gharbi	Aioun	19517	2000	69.7	61.4	78.2
Mauritania	Hodh El Gharbi	Aioun	19517	2005	64.5	56.5	73.1
Mauritania	Hodh El Gharbi	Aioun	19517	2010	55.3	47.7	63.2
Mauritania	Hodh El Gharbi	Aioun	19517	2015	45.6	38.7	53.1
Mauritania	Hodh El Gharbi	Kobeni	19518	2000	92.5	84.4	101.2
Mauritania	Hodh El Gharbi	Kobeni	19518	2005	83.5	76.0	92.0
Mauritania	Hodh El Gharbi	Kobeni	19518	2010	72.1	64.1	79.6
Mauritania	Hodh El Gharbi	Kobeni	19518	2015	59.0	51.4	67.5
Mauritania	Hodh El Gharbi	Tamchekett	19519	2000	71.2	62.8	80.8
Mauritania	Hodh El Gharbi	Tamchekett	19519	2005	64.9	57.2	73.9
Mauritania	Hodh El Gharbi	Tamchekett	19519	2010	54.1	47.2	62.0
Mauritania	Hodh El Gharbi	Tamchekett	19519	2015	45.6	38.7	53.3
Mauritania	Hodh El Gharbi	Tintane	19520	2000	81.7	74.3	89.8
Mauritania	Hodh El Gharbi	Tintane	19520	2005	74.5	67.9	81.5
Mauritania	Hodh El Gharbi	Tintane	19520	2010	62.5	55.9	68.9
Mauritania	Hodh El Gharbi	Tintane	19520	2015	53.4	46.7	60.5
Mauritania	Inchiri	Akjoujt	19521	2000	84.9	72.3	98.2
Mauritania	Inchiri	Akjoujt	19521	2005	84.6	72.2	97.7
Mauritania	Inchiri	Akjoujt	19521	2010	71.1	60.2	83.6
Mauritania	Inchiri	Akjoujt	19521	2015	58.1	48.4	68.2
Mauritania	Nouakchott	Nouakchott	19522	2000	81.1	74.3	88.8
Mauritania	Nouakchott	Nouakchott	19522	2005	76.1	68.9	83.5
Mauritania	Nouakchott	Nouakchott	19522	2010	63.2	55.9	70.9
Mauritania	Nouakchott	Nouakchott	19522	2015	49.1	42.2	56.8
Mauritania	Tagant	Moudjeria	19523	2000	74.9	66.8	83.5
Mauritania	Tagant	Moudjeria	19523	2005	71.8	63.9	81.0
Mauritania	Tagant	Moudjeria	19523	2010	61.3	53.5	70.3
Mauritania	Tagant	Moudjeria	19523	2015	49.1	42.4	57.4
Mauritania	Tagant	Tichitt	19524	2000	67.1	57.0	77.9
Mauritania	Tagant	Tichitt	19524	2005	65.0	55.5	74.9
Mauritania	Tagant	Tichitt	19524	2010	55.4	46.6	64.7
Mauritania	Tagant	Tichitt	19524	2015	46.8	38.7	55.2
Mauritania	Tagant	Tidjikja	19525	2000	71.1	62.2	81.5
Mauritania	Tagant	Tidjikja	19525	2005	70.8	61.4	80.3
Mauritania	Tagant	Tidjikja	19525	2010	60.3	52.1	69.8
Mauritania	Tagant	Tidjikja	19525	2015	49.0	41.6	57.6
Mauritania	Tiris-Zemmour	Bir Mogrein	19526	2000	61.6	50.2	74.9
Mauritania	Tiris-Zemmour	Bir Mogrein	19526	2005	64.7	52.8	78.3
Mauritania	Tiris-Zemmour	Bir Mogrein	19526	2010	56.1	46.3	67.7
Mauritania	Tiris-Zemmour	Bir Mogrein	19526	2015	48.0	38.9	58.5
Mauritania	Tiris-Zemmour	F'Derik	19527	2000	81.3	69.5	95.3
Mauritania	Tiris-Zemmour	F'Derik	19527	2005	83.7	71.3	97.4
Mauritania	Tiris-Zemmour	F'Derik	19527	2010	69.6	57.8	81.8
Mauritania	Tiris-Zemmour	F'Derik	19527	2015	58.8	48.0	70.8
Mauritania	Tiris-Zemmour	Zoueratt	19528	2000	81.8	70.0	95.8
Mauritania	Tiris-Zemmour	Zoueratt	19528	2005	83.1	70.6	97.0
Mauritania	Tiris-Zemmour	Zoueratt	19528	2010	69.1	57.2	82.1
Mauritania	Tiris-Zemmour	Zoueratt	19528	2015	59.0	48.2	71.4
Mauritania	Trarza	Boutilimit	19529	2000	83.0	73.7	92.2
Mauritania	Trarza	Boutilimit	19529	2005	76.8	68.1	85.8
Mauritania	Trarza	Boutilimit	19529	2010	62.5	55.0	70.6
Mauritania	Trarza	Boutilimit	19529	2015	48.1	41.0	55.6
Mauritania	Trarza	Keur Macene	19530	2000	80.0	70.8	90.4
Mauritania	Trarza	Keur Macene	19530	2005	69.7	62.2	78.4
Mauritania	Trarza	Keur Macene	19530	2010	55.0	48.6	62.4
Mauritania	Trarza	Keur Macene	19530	2015	39.9	34.2	46.1
Mauritania	Trarza	Mederdra	19531	2000	80.1	71.7	89.8
Mauritania	Trarza	Mederdra	19531	2005	70.9	63.4	79.0
Mauritania	Trarza	Mederdra	19531	2010	56.8	50.2	64.0
Mauritania	Trarza	Mederdra	19531	2015	42.3	36.3	49.2
Mauritania	Trarza	Ouad Naga	19532	2000	82.8	76.2	90.4
Mauritania	Trarza	Ouad Naga	19532	2005	77.9	70.8	85.1
Mauritania	Trarza	Ouad Naga	19532	2010	65.0	57.8	72.6
Mauritania	Trarza	Ouad Naga	19532	2015	50.2	43.4	57.5
Mauritania	Trarza	R'kiz	19533	2000	92.9	84.2	101.5
Mauritania	Trarza	R'kiz	19533	2005	76.6	70.1	83.7
Mauritania	Trarza	R'kiz	19533	2010	55.8	49.9	62.4
Mauritania	Trarza	R'kiz	19533	2015	40.1	34.5	45.8
Mauritania	Trarza	Rosso	19534	2000	85.2	78.0	93.0

Admin 0	Admin 1	Admin 2	GAUL Code	Year	Under-5 mortality (per 1,000 livebirths)		
					Estimate	Lower bound, 95% UI	Upper bound, 95% UI
Mauritania	Trarza	Rosso	19534	2005	71.6	65.4	78.2
Mauritania	Trarza	Rosso	19534	2010	54.1	48.6	60.4
Mauritania	Trarza	Rosso	19534	2015	39.1	33.8	44.9
Morocco	Chaouia - Ouardigha	Ben Slimane	147339	2000	36.7	31.7	41.9
Morocco	Chaouia - Ouardigha	Ben Slimane	147339	2005	29.7	26.2	33.9
Morocco	Chaouia - Ouardigha	Ben Slimane	147339	2010	25.9	22.1	30.3
Morocco	Chaouia - Ouardigha	Ben Slimane	147339	2015	20.8	17.2	24.7
Morocco	Chaouia - Ouardigha	Khouribga	21805	2000	42.4	36.6	49.4
Morocco	Chaouia - Ouardigha	Khouribga	21805	2005	34.0	29.4	39.6
Morocco	Chaouia - Ouardigha	Khouribga	21805	2010	28.4	24.1	33.9
Morocco	Chaouia - Ouardigha	Khouribga	21805	2015	23.3	19.4	28.4
Morocco	Chaouia - Ouardigha	Settat	21806	2000	41.3	36.2	47.2
Morocco	Chaouia - Ouardigha	Settat	21806	2005	32.8	28.6	37.4
Morocco	Chaouia - Ouardigha	Settat	21806	2010	27.4	23.4	31.7
Morocco	Chaouia - Ouardigha	Settat	21806	2015	22.2	18.7	26.6
Morocco	Doukkala - Abda	El Jadida	21804	2000	42.7	36.1	50.3
Morocco	Doukkala - Abda	El Jadida	21804	2005	34.4	29.1	40.1
Morocco	Doukkala - Abda	El Jadida	21804	2010	27.2	22.6	32.3
Morocco	Doukkala - Abda	El Jadida	21804	2015	22.0	17.9	26.7
Morocco	Doukkala - Abda	Safi	21832	2000	46.2	39.1	54.9
Morocco	Doukkala - Abda	Safi	21832	2005	37.8	31.5	45.1
Morocco	Doukkala - Abda	Safi	21832	2010	29.6	24.3	35.4
Morocco	Doukkala - Abda	Safi	21832	2015	23.8	19.3	28.9
Morocco	Fès - Boulemane	Boulemane	21788	2000	55.6	46.8	65.6
Morocco	Fès - Boulemane	Boulemane	21788	2005	44.0	36.9	52.0
Morocco	Fès - Boulemane	Boulemane	21788	2010	33.6	27.8	40.5
Morocco	Fès - Boulemane	Boulemane	21788	2015	26.2	21.6	32.3
Morocco	Fès - Boulemane	Fès	147340	2000	45.6	39.2	52.2
Morocco	Fès - Boulemane	Fès	147340	2005	37.9	32.9	43.7
Morocco	Fès - Boulemane	Fès	147340	2010	30.5	25.6	35.7
Morocco	Fès - Boulemane	Fès	147340	2015	24.4	20.1	29.6
Morocco	Fès - Boulemane	Sefrou	147341	2000	48.8	42.0	56.4
Morocco	Fès - Boulemane	Sefrou	147341	2005	39.5	34.1	45.8
Morocco	Fès - Boulemane	Sefrou	147341	2010	31.3	26.3	37.1
Morocco	Fès - Boulemane	Sefrou	147341	2015	24.8	20.4	30.0
Morocco	Fès - Boulemane	Zouagha-Moulay Yacoub	147342	2000	47.2	40.9	54.0
Morocco	Fès - Boulemane	Zouagha-Moulay Yacoub	147342	2005	38.7	33.5	44.3
Morocco	Fès - Boulemane	Zouagha-Moulay Yacoub	147342	2010	30.4	25.5	35.6
Morocco	Fès - Boulemane	Zouagha-Moulay Yacoub	147342	2015	24.2	19.9	29.4
Morocco	Gharb - Chrarda - Béni Hssen	Kénitra	21809	2000	43.4	37.5	50.1
Morocco	Gharb - Chrarda - Béni Hssen	Kénitra	21809	2005	34.4	29.6	39.4
Morocco	Gharb - Chrarda - Béni Hssen	Kénitra	21809	2010	27.3	23.1	31.9
Morocco	Gharb - Chrarda - Béni Hssen	Kénitra	21809	2015	22.1	18.2	26.3
Morocco	Gharb - Chrarda - Béni Hssen	Sidi Kacem	21814	2000	50.4	44.2	57.3
Morocco	Gharb - Chrarda - Béni Hssen	Sidi Kacem	21814	2005	39.9	34.5	45.6
Morocco	Gharb - Chrarda - Béni Hssen	Sidi Kacem	21814	2010	30.1	25.4	35.0
Morocco	Gharb - Chrarda - Béni Hssen	Sidi Kacem	21814	2015	24.1	19.7	28.8
Morocco	Grand Casablanca	Casablanca	147343	2000	32.6	27.9	37.7
Morocco	Grand Casablanca	Casablanca	147343	2005	27.8	23.9	32.2
Morocco	Grand Casablanca	Casablanca	147343	2010	24.5	20.2	28.9
Morocco	Grand Casablanca	Casablanca	147343	2015	19.9	16.4	24.2
Morocco	Grand Casablanca	Mohammedia	147344	2000	32.4	27.9	37.1
Morocco	Grand Casablanca	Mohammedia	147344	2005	27.6	24.1	31.8
Morocco	Grand Casablanca	Mohammedia	147344	2010	24.3	20.4	28.5
Morocco	Grand Casablanca	Mohammedia	147344	2015	19.3	16.0	23.4
Morocco	Guelmim - Es-Semara	Assa-Zag	147345	2000	53.4	42.3	66.2
Morocco	Guelmim - Es-Semara	Assa-Zag	147345	2005	43.9	34.5	54.5
Morocco	Guelmim - Es-Semara	Assa-Zag	147345	2010	35.7	28.2	44.5
Morocco	Guelmim - Es-Semara	Assa-Zag	147345	2015	28.0	21.9	35.0
Morocco	Guelmim - Es-Semara	Guelmim	147346	2000	48.5	39.4	59.0
Morocco	Guelmim - Es-Semara	Guelmim	147346	2005	39.0	31.5	47.5
Morocco	Guelmim - Es-Semara	Guelmim	147346	2010	30.9	24.8	38.0
Morocco	Guelmim - Es-Semara	Guelmim	147346	2015	24.4	19.1	30.3
Morocco	Guelmim - Es-Semara	Tan-Tan	21825	2000	51.4	40.7	64.3
Morocco	Guelmim - Es-Semara	Tan-Tan	21825	2005	42.4	33.8	53.4
Morocco	Guelmim - Es-Semara	Tan-Tan	21825	2010	36.1	28.7	45.7
Morocco	Guelmim - Es-Semara	Tan-Tan	21825	2015	28.2	21.9	36.1
Morocco	Guelmim - Es-Semara	Tata	21827	2000	62.0	51.5	73.9
Morocco	Guelmim - Es-Semara	Tata	21827	2005	50.3	42.0	60.2
Morocco	Guelmim - Es-Semara	Tata	21827	2010	36.6	30.4	44.4
Morocco	Guelmim - Es-Semara	Tata	21827	2015	28.7	23.6	35.2
Morocco	Laâyoune - Boujdour - Sakia El Hamra	Laâyoune	21823	2000	54.1	42.3	68.8
Morocco	Laâyoune - Boujdour - Sakia El Hamra	Laâyoune	21823	2005	46.2	36.5	58.9
Morocco	Laâyoune - Boujdour - Sakia El Hamra	Laâyoune	21823	2010	41.1	32.2	51.5
Morocco	Laâyoune - Boujdour - Sakia El Hamra	Laâyoune	21823	2015	31.0	24.1	39.6
Morocco	Marrakech - Tensift - Al Haouz	Al Haouz	147347	2000	55.7	47.9	65.1
Morocco	Marrakech - Tensift - Al Haouz	Al Haouz	147347	2005	45.7	39.0	53.3
Morocco	Marrakech - Tensift - Al Haouz	Al Haouz	147347	2010	32.9	27.5	39.5
Morocco	Marrakech - Tensift - Al Haouz	Al Haouz	147347	2015	26.4	21.7	31.8
Morocco	Marrakech - Tensift - Al Haouz	Chichaoua	147348	2000	56.3	48.0	65.6
Morocco	Marrakech - Tensift - Al Haouz	Chichaoua	147348	2005	47.0	39.8	55.3
Morocco	Marrakech - Tensift - Al Haouz	Chichaoua	147348	2010	33.1	27.4	39.5
Morocco	Marrakech - Tensift - Al Haouz	Chichaoua	147348	2015	25.8	21.1	31.6
Morocco	Marrakech - Tensift - Al Haouz	El Kelaâ des Sraghna	21829	2000	47.5	41.5	54.3
Morocco	Marrakech - Tensift - Al Haouz	El Kelaâ des Sraghna	21829	2005	38.7	33.5	44.2

Admin 0	Admin 1	Admin 2	GAUL Code	Year	Under-5 mortality (per 1,000 livebirths)		
					Estimate	Lower bound, 95% UI	Upper bound, 95% UI
Morocco	Marrakech - Tensift - Al Haouz	El Kelaâ des Sraghna	21829	2010	28.3	24.0	33.5
Morocco	Marrakech - Tensift - Al Haouz	El Kelaâ des Sraghna	21829	2015	22.8	19.0	27.3
Morocco	Marrakech - Tensift - Al Haouz	Essaouira	21830	2000	52.3	44.0	62.5
Morocco	Marrakech - Tensift - Al Haouz	Essaouira	21830	2005	43.5	36.4	52.4
Morocco	Marrakech - Tensift - Al Haouz	Essaouira	21830	2010	31.6	25.8	38.3
Morocco	Marrakech - Tensift - Al Haouz	Essaouira	21830	2015	25.2	20.5	30.6
Morocco	Marrakech - Tensift - Al Haouz	Marrakech	147349	2000	44.5	38.1	51.7
Morocco	Marrakech - Tensift - Al Haouz	Marrakech	147349	2005	37.4	31.8	43.8
Morocco	Marrakech - Tensift - Al Haouz	Marrakech	147349	2010	29.3	24.1	35.1
Morocco	Marrakech - Tensift - Al Haouz	Marrakech	147349	2015	23.2	18.8	28.2
Morocco	Meknès - Tafilalet	El Hajeb	147350	2000	46.9	40.5	53.6
Morocco	Meknès - Tafilalet	El Hajeb	147350	2005	38.1	32.7	43.6
Morocco	Meknès - Tafilalet	El Hajeb	147350	2010	29.4	24.9	34.3
Morocco	Meknès - Tafilalet	El Hajeb	147350	2015	23.8	19.7	28.4
Morocco	Meknès - Tafilalet	Errachidia	21792	2000	60.2	50.2	71.9
Morocco	Meknès - Tafilalet	Errachidia	21792	2005	47.5	39.5	57.0
Morocco	Meknès - Tafilalet	Errachidia	21792	2010	36.7	30.2	44.9
Morocco	Meknès - Tafilalet	Errachidia	21792	2015	28.9	23.5	35.5
Morocco	Meknès - Tafilalet	Ifrane	21793	2000	48.0	40.5	56.2
Morocco	Meknès - Tafilalet	Ifrane	21793	2005	38.0	32.3	44.7
Morocco	Meknès - Tafilalet	Ifrane	21793	2010	30.5	25.5	36.4
Morocco	Meknès - Tafilalet	Ifrane	21793	2015	24.7	20.3	30.2
Morocco	Meknès - Tafilalet	Khénifra	21794	2000	49.3	42.0	57.8
Morocco	Meknès - Tafilalet	Khénifra	21794	2005	39.0	33.3	45.5
Morocco	Meknès - Tafilalet	Khénifra	21794	2010	30.9	25.6	36.3
Morocco	Meknès - Tafilalet	Khénifra	21794	2015	25.3	20.8	30.1
Morocco	Meknès - Tafilalet	Meknès	147351	2000	43.5	37.4	49.5
Morocco	Meknès - Tafilalet	Meknès	147351	2005	36.0	30.7	41.2
Morocco	Meknès - Tafilalet	Meknès	147351	2010	29.0	24.4	33.6
Morocco	Meknès - Tafilalet	Meknès	147351	2015	23.2	18.9	27.5
Morocco	Oriental	Berkane Taourirt	147352	2000	44.7	37.6	52.4
Morocco	Oriental	Berkane Taourirt	147352	2005	34.3	28.8	40.4
Morocco	Oriental	Berkane Taourirt	147352	2010	28.6	23.5	34.4
Morocco	Oriental	Berkane Taourirt	147352	2015	22.4	18.0	27.1
Morocco	Oriental	Figuig	21818	2000	62.6	50.7	76.4
Morocco	Oriental	Figuig	21818	2005	49.5	40.1	60.3
Morocco	Oriental	Figuig	21818	2010	38.9	31.5	47.7
Morocco	Oriental	Figuig	21818	2015	29.5	23.5	36.2
Morocco	Oriental	Jerada	147353	2000	48.6	40.8	57.3
Morocco	Oriental	Jerada	147353	2005	37.3	31.5	44.1
Morocco	Oriental	Jerada	147353	2010	31.2	25.7	37.5
Morocco	Oriental	Jerada	147353	2015	24.3	19.5	29.7
Morocco	Oriental	Nador	21819	2000	46.8	39.4	55.3
Morocco	Oriental	Nador	21819	2005	36.1	29.9	42.7
Morocco	Oriental	Nador	21819	2010	29.4	24.0	35.7
Morocco	Oriental	Nador	21819	2015	23.1	18.4	28.2
Morocco	Oriental	Oujda Angad	147354	2000	42.1	34.4	50.4
Morocco	Oriental	Oujda Angad	147354	2005	33.2	26.7	39.9
Morocco	Oriental	Oujda Angad	147354	2010	29.3	23.4	35.4
Morocco	Oriental	Oujda Angad	147354	2015	22.9	17.9	28.4
Morocco	Rabat - Salé - Zemmour - Zaer	Khémisset	21810	2000	41.1	35.7	47.1
Morocco	Rabat - Salé - Zemmour - Zaer	Khémisset	21810	2005	33.8	29.2	38.7
Morocco	Rabat - Salé - Zemmour - Zaer	Khémisset	21810	2010	26.9	22.7	31.5
Morocco	Rabat - Salé - Zemmour - Zaer	Khémisset	21810	2015	22.3	18.4	26.5
Morocco	Rabat - Salé - Zemmour - Zaer	Rabat	21812	2000	31.6	27.2	36.4
Morocco	Rabat - Salé - Zemmour - Zaer	Rabat	21812	2005	27.2	23.4	31.4
Morocco	Rabat - Salé - Zemmour - Zaer	Rabat	21812	2010	24.6	20.6	29.1
Morocco	Rabat - Salé - Zemmour - Zaer	Rabat	21812	2015	19.5	15.9	23.4
Morocco	Rabat - Salé - Zemmour - Zaer	Salé	21813	2000	33.6	29.1	38.8
Morocco	Rabat - Salé - Zemmour - Zaer	Salé	21813	2005	28.5	24.4	32.7
Morocco	Rabat - Salé - Zemmour - Zaer	Salé	21813	2010	25.2	21.0	29.6
Morocco	Rabat - Salé - Zemmour - Zaer	Salé	21813	2015	20.4	16.8	24.4
Morocco	Rabat - Salé - Zemmour - Zaer	Skhirate-Témara	147355	2000	32.7	28.0	38.0
Morocco	Rabat - Salé - Zemmour - Zaer	Skhirate-Témara	147355	2005	27.7	23.8	32.0
Morocco	Rabat - Salé - Zemmour - Zaer	Skhirate-Témara	147355	2010	24.6	20.7	29.0
Morocco	Rabat - Salé - Zemmour - Zaer	Skhirate-Témara	147355	2015	19.7	16.1	23.7
Morocco	Souss - Massa - Draâ	Agadir-Ida ou Tanane	147356	2000	44.2	37.0	52.7
Morocco	Souss - Massa - Draâ	Agadir-Ida ou Tanane	147356	2005	37.7	31.7	44.7
Morocco	Souss - Massa - Draâ	Agadir-Ida ou Tanane	147356	2010	29.2	23.8	35.4
Morocco	Souss - Massa - Draâ	Agadir-Ida ou Tanane	147356	2015	23.2	18.8	28.8
Morocco	Souss - Massa - Draâ	Chtouka-Aït Baha	147357	2000	49.3	41.8	58.3
Morocco	Souss - Massa - Draâ	Chtouka-Aït Baha	147357	2005	40.7	34.5	47.8
Morocco	Souss - Massa - Draâ	Chtouka-Aït Baha	147357	2010	29.0	23.9	34.8
Morocco	Souss - Massa - Draâ	Chtouka-Aït Baha	147357	2015	23.2	18.8	28.4
Morocco	Souss - Massa - Draâ	Inezgane-Aït Melloul	147358	2000	45.3	38.0	54.0
Morocco	Souss - Massa - Draâ	Inezgane-Aït Melloul	147358	2005	37.7	31.6	44.4
Morocco	Souss - Massa - Draâ	Inezgane-Aït Melloul	147358	2010	28.4	23.2	34.4
Morocco	Souss - Massa - Draâ	Inezgane-Aït Melloul	147358	2015	22.5	18.3	27.9
Morocco	Souss - Massa - Draâ	Ouarzazate	147359	2000	62.6	53.2	72.9
Morocco	Souss - Massa - Draâ	Ouarzazate	147359	2005	50.4	42.8	58.9
Morocco	Souss - Massa - Draâ	Ouarzazate	147359	2010	36.3	30.2	42.6
Morocco	Souss - Massa - Draâ	Ouarzazate	147359	2015	28.6	23.6	34.0
Morocco	Souss - Massa - Draâ	Taroudannt	21826	2000	57.1	48.5	67.3
Morocco	Souss - Massa - Draâ	Taroudannt	21826	2005	47.2	40.1	55.4
Morocco	Souss - Massa - Draâ	Taroudannt	21826	2010	33.2	27.8	40.2

Admin 0	Admin 1	Admin 2	GAUL Code	Year	Under-5 mortality (per 1,000 livebirths)		
					Estimate	Lower bound, 95% UI	Upper bound, 95% UI
Morocco	Souss - Massa - Draâ	Taroudannt	21826	2015	26.1	21.4	31.7
Morocco	Souss - Massa - Draâ	Tiznit	21828	2000	50.2	41.9	59.8
Morocco	Souss - Massa - Draâ	Tiznit	21828	2005	41.6	34.9	49.4
Morocco	Souss - Massa - Draâ	Tiznit	21828	2010	31.1	25.5	37.6
Morocco	Souss - Massa - Draâ	Tiznit	21828	2015	24.8	19.9	30.5
Morocco	Souss - Massa - Draâ	Zagora	147360	2000	64.5	52.3	78.4
Morocco	Souss - Massa - Draâ	Zagora	147360	2005	51.1	41.6	61.9
Morocco	Souss - Massa - Draâ	Zagora	147360	2010	37.0	29.7	45.0
Morocco	Souss - Massa - Draâ	Zagora	147360	2015	29.5	23.5	35.9
Morocco	Tadla - Azilal	Azilal	21799	2000	55.5	47.6	64.4
Morocco	Tadla - Azilal	Azilal	21799	2005	44.4	37.6	51.3
Morocco	Tadla - Azilal	Azilal	21799	2010	32.2	26.7	38.1
Morocco	Tadla - Azilal	Azilal	21799	2015	26.0	21.4	31.3
Morocco	Tadla - Azilal	Béni Mellal	21802	2000	46.4	39.8	54.1
Morocco	Tadla - Azilal	Béni Mellal	21802	2005	36.8	31.2	42.8
Morocco	Tadla - Azilal	Béni Mellal	21802	2010	27.9	23.3	33.1
Morocco	Tadla - Azilal	Béni Mellal	21802	2015	23.5	19.3	28.4
Morocco	Tanger - Tétouan	Chefchaouen	21808	2000	57.4	49.6	66.3
Morocco	Tanger - Tétouan	Chefchaouen	21808	2005	46.0	39.6	53.1
Morocco	Tanger - Tétouan	Chefchaouen	21808	2010	34.5	28.8	40.6
Morocco	Tanger - Tétouan	Chefchaouen	21808	2015	27.0	22.0	32.1
Morocco	Tanger - Tétouan	Fahs Anjra	147361	2000	50.6	42.5	59.9
Morocco	Tanger - Tétouan	Fahs Anjra	147361	2005	39.3	32.9	46.8
Morocco	Tanger - Tétouan	Fahs Anjra	147361	2010	33.5	27.5	40.6
Morocco	Tanger - Tétouan	Fahs Anjra	147361	2015	26.5	21.2	32.8
Morocco	Tanger - Tétouan	Larache	21811	2000	51.2	43.2	60.7
Morocco	Tanger - Tétouan	Larache	21811	2005	39.8	33.4	46.9
Morocco	Tanger - Tétouan	Larache	21811	2010	31.8	26.0	38.4
Morocco	Tanger - Tétouan	Larache	21811	2015	25.0	19.9	30.5
Morocco	Tanger - Tétouan	Tanger-Assilah	147362	2000	49.0	41.0	58.3
Morocco	Tanger - Tétouan	Tanger-Assilah	147362	2005	38.7	32.2	46.1
Morocco	Tanger - Tétouan	Tanger-Assilah	147362	2010	33.2	27.1	40.1
Morocco	Tanger - Tétouan	Tanger-Assilah	147362	2015	25.9	20.8	32.1
Morocco	Tanger - Tétouan	Tétouan	21817	2000	52.1	43.7	61.3
Morocco	Tanger - Tétouan	Tétouan	21817	2005	40.5	33.7	47.8
Morocco	Tanger - Tétouan	Tétouan	21817	2010	33.7	26.9	40.6
Morocco	Tanger - Tétouan	Tétouan	21817	2015	26.2	20.9	31.9
Morocco	Taza - Al Hoceima - Taounate	Al Hoceïma	21787	2000	53.0	45.6	60.9
Morocco	Taza - Al Hoceima - Taounate	Al Hoceïma	21787	2005	41.5	35.6	47.7
Morocco	Taza - Al Hoceima - Taounate	Al Hoceïma	21787	2010	32.3	27.4	37.4
Morocco	Taza - Al Hoceima - Taounate	Al Hoceïma	21787	2015	25.4	21.1	30.2
Morocco	Taza - Al Hoceima - Taounate	Taounate	21790	2000	55.3	48.2	63.5
Morocco	Taza - Al Hoceima - Taounate	Taounate	21790	2005	44.8	39.1	51.3
Morocco	Taza - Al Hoceima - Taounate	Taounate	21790	2010	32.6	27.9	38.0
Morocco	Taza - Al Hoceima - Taounate	Taounate	21790	2015	25.9	21.5	30.9
Morocco	Taza - Al Hoceima - Taounate	Taza	21791	2000	52.0	44.3	60.2
Morocco	Taza - Al Hoceima - Taounate	Taza	21791	2005	41.1	35.2	47.8
Morocco	Taza - Al Hoceima - Taounate	Taza	21791	2010	31.2	26.3	36.7
Morocco	Taza - Al Hoceima - Taounate	Taza	21791	2015	24.8	20.5	30.1
Mozambique	Cabo Delgado	Ancuabe	21833	2000	198.8	180.8	216.4
Mozambique	Cabo Delgado	Ancuabe	21833	2005	150.6	137.4	164.4
Mozambique	Cabo Delgado	Ancuabe	21833	2010	114.5	101.9	128.0
Mozambique	Cabo Delgado	Ancuabe	21833	2015	95.7	83.0	109.6
Mozambique	Cabo Delgado	Balama	21834	2000	193.8	176.1	213.4
Mozambique	Cabo Delgado	Balama	21834	2005	145.0	130.7	160.2
Mozambique	Cabo Delgado	Balama	21834	2010	118.7	104.8	133.1
Mozambique	Cabo Delgado	Balama	21834	2015	94.0	81.2	107.8
Mozambique	Cabo Delgado	Chiure	21835	2000	201.2	186.8	217.8
Mozambique	Cabo Delgado	Chiure	21835	2005	148.8	137.1	161.4
Mozambique	Cabo Delgado	Chiure	21835	2010	114.4	103.2	126.6
Mozambique	Cabo Delgado	Chiure	21835	2015	96.5	84.2	109.5
Mozambique	Cabo Delgado	Cidade de Pemba	65247	2000	156.2	139.8	173.6
Mozambique	Cabo Delgado	Cidade de Pemba	65247	2005	113.9	101.7	127.0
Mozambique	Cabo Delgado	Cidade de Pemba	65247	2010	79.6	69.6	90.3
Mozambique	Cabo Delgado	Cidade de Pemba	65247	2015	73.2	62.7	85.2
Mozambique	Cabo Delgado	Ibo	21836	2000	169.0	147.0	195.6
Mozambique	Cabo Delgado	Ibo	21836	2005	130.6	112.5	150.9
Mozambique	Cabo Delgado	Ibo	21836	2010	94.0	79.3	110.1
Mozambique	Cabo Delgado	Ibo	21836	2015	83.3	69.1	98.8
Mozambique	Cabo Delgado	Macomia	21837	2000	179.6	161.5	200.3
Mozambique	Cabo Delgado	Macomia	21837	2005	143.4	127.8	159.5
Mozambique	Cabo Delgado	Macomia	21837	2010	108.2	94.9	122.3
Mozambique	Cabo Delgado	Macomia	21837	2015	92.1	78.8	106.2
Mozambique	Cabo Delgado	Mecufi	21838	2000	184.9	167.6	204.3
Mozambique	Cabo Delgado	Mecufi	21838	2005	136.9	122.8	151.2
Mozambique	Cabo Delgado	Mecufi	21838	2010	101.0	89.0	114.2
Mozambique	Cabo Delgado	Mecufi	21838	2015	88.4	75.3	102.4
Mozambique	Cabo Delgado	Meluco	21839	2000	192.5	171.6	213.2
Mozambique	Cabo Delgado	Meluco	21839	2005	149.2	132.8	166.7
Mozambique	Cabo Delgado	Meluco	21839	2010	112.4	98.6	127.6
Mozambique	Cabo Delgado	Meluco	21839	2015	93.4	80.1	107.6
Mozambique	Cabo Delgado	Mocimboa da Praia	21840	2000	162.2	145.0	180.9
Mozambique	Cabo Delgado	Mocimboa da Praia	21840	2005	130.7	116.7	146.9
Mozambique	Cabo Delgado	Mocimboa da Praia	21840	2010	95.0	82.5	107.7
Mozambique	Cabo Delgado	Mocimboa da Praia	21840	2015	81.9	69.5	94.8

Admin 0	Admin 1	Admin 2	GAUL Code	Year	Under-5 mortality (per 1,000 livebirths)		
					Estimate	Lower bound, 95% UI	Upper bound, 95% UI
Mozambique	Cabo Delgado	Montepuez	21841	2000	191.4	174.1	207.6
Mozambique	Cabo Delgado	Montepuez	21841	2005	147.2	134.4	160.1
Mozambique	Cabo Delgado	Montepuez	21841	2010	110.3	98.7	122.4
Mozambique	Cabo Delgado	Montepuez	21841	2015	88.8	76.8	101.2
Mozambique	Cabo Delgado	Mueda	21842	2000	165.2	150.1	180.8
Mozambique	Cabo Delgado	Mueda	21842	2005	133.6	120.6	147.3
Mozambique	Cabo Delgado	Mueda	21842	2010	97.2	86.0	108.9
Mozambique	Cabo Delgado	Mueda	21842	2015	78.4	68.2	89.7
Mozambique	Cabo Delgado	Muidumbe	21843	2000	171.9	154.3	190.6
Mozambique	Cabo Delgado	Muidumbe	21843	2005	138.5	124.2	153.5
Mozambique	Cabo Delgado	Muidumbe	21843	2010	100.8	88.2	113.7
Mozambique	Cabo Delgado	Muidumbe	21843	2015	85.8	73.5	99.9
Mozambique	Cabo Delgado	Namuno	21844	2000	204.7	188.2	222.7
Mozambique	Cabo Delgado	Namuno	21844	2005	150.0	137.7	163.9
Mozambique	Cabo Delgado	Namuno	21844	2010	123.5	110.4	138.2
Mozambique	Cabo Delgado	Namuno	21844	2015	99.2	86.2	114.1
Mozambique	Cabo Delgado	Nangade	21845	2000	161.4	146.7	177.7
Mozambique	Cabo Delgado	Nangade	21845	2005	130.7	118.2	144.1
Mozambique	Cabo Delgado	Nangade	21845	2010	95.0	84.1	106.5
Mozambique	Cabo Delgado	Nangade	21845	2015	79.9	69.2	91.6
Mozambique	Cabo Delgado	Palma	21846	2000	160.0	141.6	180.2
Mozambique	Cabo Delgado	Palma	21846	2005	128.0	113.7	144.1
Mozambique	Cabo Delgado	Palma	21846	2010	96.6	84.2	109.9
Mozambique	Cabo Delgado	Palma	21846	2015	83.5	71.2	97.1
Mozambique	Cabo Delgado	Pemba	21847	2000	186.9	168.8	205.8
Mozambique	Cabo Delgado	Pemba	21847	2005	141.4	128.3	156.2
Mozambique	Cabo Delgado	Pemba	21847	2010	102.3	89.7	115.5
Mozambique	Cabo Delgado	Pemba	21847	2015	90.2	77.1	104.5
Mozambique	Cabo Delgado	Quissanga	21848	2000	187.9	169.4	209.1
Mozambique	Cabo Delgado	Quissanga	21848	2005	143.1	128.6	159.0
Mozambique	Cabo Delgado	Quissanga	21848	2010	106.9	93.6	122.6
Mozambique	Cabo Delgado	Quissanga	21848	2015	92.3	78.9	108.0
Mozambique	Gaza	Bilene	21849	2000	144.4	133.3	155.7
Mozambique	Gaza	Bilene	21849	2005	122.5	113.0	132.2
Mozambique	Gaza	Bilene	21849	2010	94.5	85.0	104.4
Mozambique	Gaza	Bilene	21849	2015	80.8	70.5	91.3
Mozambique	Gaza	Chibuto	21850	2000	141.6	130.6	152.8
Mozambique	Gaza	Chibuto	21850	2005	120.0	111.0	129.7
Mozambique	Gaza	Chibuto	21850	2010	91.1	81.7	100.9
Mozambique	Gaza	Chibuto	21850	2015	78.1	68.3	88.7
Mozambique	Gaza	Chicualacuala	21851	2000	101.9	88.1	117.0
Mozambique	Gaza	Chicualacuala	21851	2005	89.7	78.1	103.4
Mozambique	Gaza	Chicualacuala	21851	2010	75.4	64.6	88.0
Mozambique	Gaza	Chicualacuala	21851	2015	60.5	51.5	71.3
Mozambique	Gaza	Chigubo	21852	2000	130.3	115.5	147.3
Mozambique	Gaza	Chigubo	21852	2005	115.8	102.3	131.3
Mozambique	Gaza	Chigubo	21852	2010	90.1	78.1	102.6
Mozambique	Gaza	Chigubo	21852	2015	70.4	60.0	82.3
Mozambique	Gaza	Chokwe	21853	2000	142.4	130.4	154.3
Mozambique	Gaza	Chokwe	21853	2005	117.0	107.0	126.8
Mozambique	Gaza	Chokwe	21853	2010	90.5	80.9	100.5
Mozambique	Gaza	Chokwe	21853	2015	78.3	68.1	89.3
Mozambique	Gaza	Cidade de Xai-Xai	65248	2000	116.7	106.6	128.2
Mozambique	Gaza	Cidade de Xai-Xai	65248	2005	103.5	94.3	113.4
Mozambique	Gaza	Cidade de Xai-Xai	65248	2010	85.0	75.9	95.4
Mozambique	Gaza	Cidade de Xai-Xai	65248	2015	67.8	58.9	78.3
Mozambique	Gaza	Guija	21854	2000	151.1	138.2	165.3
Mozambique	Gaza	Guija	21854	2005	123.9	112.2	135.7
Mozambique	Gaza	Guija	21854	2010	94.6	84.2	105.9
Mozambique	Gaza	Guija	21854	2015	82.7	72.0	94.2
Mozambique	Gaza	Mabalane	21855	2000	132.4	115.5	152.1
Mozambique	Gaza	Mabalane	21855	2005	113.4	99.0	129.9
Mozambique	Gaza	Mabalane	21855	2010	87.6	75.1	101.8
Mozambique	Gaza	Mabalane	21855	2015	72.9	62.0	85.9
Mozambique	Gaza	Mandlakaze	21856	2000	133.1	121.5	145.9
Mozambique	Gaza	Mandlakaze	21856	2005	113.7	104.5	123.9
Mozambique	Gaza	Mandlakaze	21856	2010	88.5	79.8	98.3
Mozambique	Gaza	Mandlakaze	21856	2015	73.0	63.8	83.1
Mozambique	Gaza	Massangena	21857	2000	114.7	98.5	132.5
Mozambique	Gaza	Massangena	21857	2005	104.4	90.6	120.7
Mozambique	Gaza	Massangena	21857	2010	80.7	68.7	93.9
Mozambique	Gaza	Massangena	21857	2015	63.6	54.1	75.1
Mozambique	Gaza	Massingir	21858	2000	119.3	102.8	136.8
Mozambique	Gaza	Massingir	21858	2005	98.7	85.6	113.9
Mozambique	Gaza	Massingir	21858	2010	79.7	67.8	92.8
Mozambique	Gaza	Massingir	21858	2015	67.8	57.5	79.7
Mozambique	Gaza	Xai-xai	21859	2000	136.0	125.8	147.3
Mozambique	Gaza	Xai-xai	21859	2005	116.4	107.5	125.9
Mozambique	Gaza	Xai-xai	21859	2010	90.3	81.4	99.7
Mozambique	Gaza	Xai-xai	21859	2015	76.8	67.1	87.5
Mozambique	Inhambane	Cidade de Inhambane	65249	2000	112.8	101.3	125.5
Mozambique	Inhambane	Cidade de Inhambane	65249	2005	98.7	89.1	108.7
Mozambique	Inhambane	Cidade de Inhambane	65249	2010	87.1	76.9	97.0
Mozambique	Inhambane	Cidade de Inhambane	65249	2015	66.3	56.9	76.6
Mozambique	Inhambane	Funhalouro	21860	2000	129.2	114.0	145.7

Admin 0	Admin 1	Admin 2	GAUL Code	Year	Under-5 mortality (per 1,000 livebirths)		
					Estimate	Lower bound, 95% UI	Upper bound, 95% UI
Mozambique	Inhambane	Funhalouro	21860	2005	117.0	102.9	132.3
Mozambique	Inhambane	Funhalouro	21860	2010	92.9	81.0	105.6
Mozambique	Inhambane	Funhalouro	21860	2015	70.7	60.0	82.4
Mozambique	Inhambane	Govuro	21861	2000	145.8	128.0	166.2
Mozambique	Inhambane	Govuro	21861	2005	123.2	108.1	139.8
Mozambique	Inhambane	Govuro	21861	2010	95.7	82.9	109.9
Mozambique	Inhambane	Govuro	21861	2015	77.7	66.7	91.5
Mozambique	Inhambane	Homoine	21862	2000	129.5	117.1	143.2
Mozambique	Inhambane	Homoine	21862	2005	114.5	104.4	125.6
Mozambique	Inhambane	Homoine	21862	2010	96.6	85.5	108.3
Mozambique	Inhambane	Homoine	21862	2015	75.3	64.6	86.3
Mozambique	Inhambane	Inharrime	21863	2000	132.5	120.1	146.4
Mozambique	Inhambane	Inharrime	21863	2005	113.6	102.8	124.7
Mozambique	Inhambane	Inharrime	21863	2010	93.5	83.0	104.5
Mozambique	Inhambane	Inharrime	21863	2015	73.6	63.7	84.5
Mozambique	Inhambane	Inhassoro	21864	2000	137.9	121.5	154.9
Mozambique	Inhambane	Inhassoro	21864	2005	117.5	103.9	133.9
Mozambique	Inhambane	Inhassoro	21864	2010	94.4	81.9	107.8
Mozambique	Inhambane	Inhassoro	21864	2015	77.1	65.9	90.5
Mozambique	Inhambane	Jangamo	21865	2000	128.0	116.2	141.7
Mozambique	Inhambane	Jangamo	21865	2005	109.8	98.8	121.4
Mozambique	Inhambane	Jangamo	21865	2010	92.5	81.4	104.3
Mozambique	Inhambane	Jangamo	21865	2015	75.0	64.4	86.8
Mozambique	Inhambane	Mabote	21866	2000	126.0	111.0	143.3
Mozambique	Inhambane	Mabote	21866	2005	111.5	98.0	127.1
Mozambique	Inhambane	Mabote	21866	2010	85.6	74.3	97.8
Mozambique	Inhambane	Mabote	21866	2015	66.6	56.8	77.9
Mozambique	Inhambane	Massinga	21867	2000	123.2	111.1	135.7
Mozambique	Inhambane	Massinga	21867	2005	109.3	99.6	120.3
Mozambique	Inhambane	Massinga	21867	2010	89.6	80.3	100.8
Mozambique	Inhambane	Massinga	21867	2015	71.0	61.2	81.5
Mozambique	Inhambane	Maxixe	65250	2000	123.6	112.3	135.9
Mozambique	Inhambane	Maxixe	65250	2005	108.4	98.8	118.4
Mozambique	Inhambane	Maxixe	65250	2010	92.9	82.2	103.6
Mozambique	Inhambane	Maxixe	65250	2015	72.3	62.3	83.0
Mozambique	Inhambane	Morrumbene	21868	2000	127.6	115.8	141.0
Mozambique	Inhambane	Morrumbene	21868	2005	112.7	102.7	123.8
Mozambique	Inhambane	Morrumbene	21868	2010	93.7	83.8	105.0
Mozambique	Inhambane	Morrumbene	21868	2015	73.3	63.2	84.0
Mozambique	Inhambane	Panda	21869	2000	134.8	121.2	150.6
Mozambique	Inhambane	Panda	21869	2005	119.5	107.6	132.7
Mozambique	Inhambane	Panda	21869	2010	96.8	85.2	109.6
Mozambique	Inhambane	Panda	21869	2015	74.5	63.9	85.3
Mozambique	Inhambane	Vilankulo	21870	2000	127.1	113.1	142.7
Mozambique	Inhambane	Vilankulo	21870	2005	111.3	98.6	125.4
Mozambique	Inhambane	Vilankulo	21870	2010	91.8	80.5	104.9
Mozambique	Inhambane	Vilankulo	21870	2015	74.9	63.9	87.4
Mozambique	Inhambane	Zavala	21871	2000	129.0	115.4	142.7
Mozambique	Inhambane	Zavala	21871	2005	109.5	98.6	122.0
Mozambique	Inhambane	Zavala	21871	2010	87.7	77.6	99.4
Mozambique	Inhambane	Zavala	21871	2015	71.2	61.4	81.9
Mozambique	Lago niassa	Lago niassa	65267	2000	137.9	128.4	148.6
Mozambique	Lago niassa	Lago niassa	65267	2005	100.1	92.8	108.1
Mozambique	Lago niassa	Lago niassa	65267	2010	80.7	74.3	87.2
Mozambique	Lago niassa	Lago niassa	65267	2015	57.7	51.9	63.8
Mozambique	Manica	Barue	21872	2000	156.1	142.7	170.4
Mozambique	Manica	Barue	21872	2005	132.9	121.0	145.3
Mozambique	Manica	Barue	21872	2010	102.7	91.1	113.6
Mozambique	Manica	Barue	21872	2015	80.0	69.9	91.0
Mozambique	Manica	Cidade de Chimoio	65251	2000	141.0	130.4	152.0
Mozambique	Manica	Cidade de Chimoio	65251	2005	129.9	120.3	140.2
Mozambique	Manica	Cidade de Chimoio	65251	2010	107.4	97.8	118.2
Mozambique	Manica	Cidade de Chimoio	65251	2015	83.7	74.1	94.7
Mozambique	Manica	Gondola	21873	2000	169.8	157.8	181.2
Mozambique	Manica	Gondola	21873	2005	146.4	136.8	156.6
Mozambique	Manica	Gondola	21873	2010	115.9	106.3	126.3
Mozambique	Manica	Gondola	21873	2015	92.6	82.3	104.0
Mozambique	Manica	Guro	21874	2000	176.9	159.7	195.3
Mozambique	Manica	Guro	21874	2005	137.2	124.4	152.4
Mozambique	Manica	Guro	21874	2010	106.3	94.8	119.2
Mozambique	Manica	Guro	21874	2015	82.6	72.1	94.3
Mozambique	Manica	Machaze	21875	2000	134.7	121.0	149.9
Mozambique	Manica	Machaze	21875	2005	121.4	109.3	135.6
Mozambique	Manica	Machaze	21875	2010	89.5	80.1	100.5
Mozambique	Manica	Machaze	21875	2015	68.4	60.2	78.4
Mozambique	Manica	Macossa	21876	2000	176.0	160.1	193.9
Mozambique	Manica	Macossa	21876	2005	142.6	128.8	157.7
Mozambique	Manica	Macossa	21876	2010	112.7	100.3	127.0
Mozambique	Manica	Macossa	21876	2015	84.2	73.8	96.4
Mozambique	Manica	Manica	21877	2000	136.9	127.5	146.9
Mozambique	Manica	Manica	21877	2005	135.9	126.8	146.1
Mozambique	Manica	Manica	21877	2010	111.8	101.8	122.1
Mozambique	Manica	Manica	21877	2015	86.1	76.6	96.9
Mozambique	Manica	Mossurize	21878	2000	136.7	124.1	149.2
Mozambique	Manica	Mossurize	21878	2005	130.9	119.9	143.7

Admin 0	Admin 1	Admin 2	GAUL Code	Year	Under-5 mortality (per 1,000 livebirths)		
					Estimate	Lower bound, 95% UI	Upper bound, 95% UI
Mozambique	Manica	Mossurize	21878	2010	94.8	85.7	105.3
Mozambique	Manica	Mossurize	21878	2015	71.3	62.9	81.4
Mozambique	Manica	Sussundenga	21879	2000	151.4	138.9	164.8
Mozambique	Manica	Sussundenga	21879	2005	149.1	137.0	162.2
Mozambique	Manica	Sussundenga	21879	2010	115.4	104.8	127.4
Mozambique	Manica	Sussundenga	21879	2015	87.7	77.5	99.8
Mozambique	Manica	Tambara	21880	2000	177.1	160.3	194.8
Mozambique	Manica	Tambara	21880	2005	130.4	117.2	143.8
Mozambique	Manica	Tambara	21880	2010	108.7	96.5	122.1
Mozambique	Manica	Tambara	21880	2015	78.8	69.0	89.6
Mozambique	Maputo	Boane	21882	2000	103.2	97.0	110.2
Mozambique	Maputo	Boane	21882	2005	86.4	81.0	92.4
Mozambique	Maputo	Boane	21882	2010	72.9	67.0	79.6
Mozambique	Maputo	Boane	21882	2015	55.0	48.3	62.7
Mozambique	Maputo	Cidade da Matola	65252	2000	104.3	98.4	110.6
Mozambique	Maputo	Cidade da Matola	65252	2005	87.4	82.1	92.9
Mozambique	Maputo	Cidade da Matola	65252	2010	72.4	66.4	78.7
Mozambique	Maputo	Cidade da Matola	65252	2015	55.7	49.0	63.2
Mozambique	Maputo	Cidade de Maputo	41374	2000	99.4	93.6	105.3
Mozambique	Maputo	Cidade de Maputo	41374	2005	83.0	77.8	88.2
Mozambique	Maputo	Cidade de Maputo	41374	2010	71.3	65.3	78.1
Mozambique	Maputo	Cidade de Maputo	41374	2015	56.5	49.8	64.1
Mozambique	Maputo	Magude	21883	2000	129.6	117.8	143.1
Mozambique	Maputo	Magude	21883	2005	111.1	100.7	122.1
Mozambique	Maputo	Magude	21883	2010	89.0	79.1	100.0
Mozambique	Maputo	Magude	21883	2015	73.8	63.3	85.0
Mozambique	Maputo	Manhica	41375	2000	136.4	124.7	150.1
Mozambique	Maputo	Manhica	41375	2005	112.0	101.6	122.5
Mozambique	Maputo	Manhica	41375	2010	83.8	74.5	94.2
Mozambique	Maputo	Manhica	41375	2015	69.6	59.8	80.4
Mozambique	Maputo	Manhica	41376	2000	131.9	122.1	143.4
Mozambique	Maputo	Manhica	41376	2005	110.5	102.5	119.8
Mozambique	Maputo	Manhica	41376	2010	83.4	75.3	92.3
Mozambique	Maputo	Manhica	41376	2015	69.0	59.8	78.6
Mozambique	Maputo	Marracuene	21885	2000	112.3	105.5	120.2
Mozambique	Maputo	Marracuene	21885	2005	92.1	86.0	98.2
Mozambique	Maputo	Marracuene	21885	2010	73.7	67.4	80.7
Mozambique	Maputo	Marracuene	21885	2015	58.9	51.6	66.8
Mozambique	Maputo	Matutuine	21886	2000	105.9	95.9	117.3
Mozambique	Maputo	Matutuine	21886	2005	86.6	78.2	95.6
Mozambique	Maputo	Matutuine	21886	2010	81.7	72.4	92.2
Mozambique	Maputo	Matutuine	21886	2015	59.2	51.5	67.8
Mozambique	Maputo	Moamba	21887	2000	116.6	108.3	125.3
Mozambique	Maputo	Moamba	21887	2005	97.9	91.0	105.1
Mozambique	Maputo	Moamba	21887	2010	78.5	71.6	86.4
Mozambique	Maputo	Moamba	21887	2015	59.3	51.9	67.1
Mozambique	Maputo	Namaacha	21888	2000	93.1	85.4	102.0
Mozambique	Maputo	Namaacha	21888	2005	90.3	82.7	98.3
Mozambique	Maputo	Namaacha	21888	2010	78.2	70.8	86.5
Mozambique	Maputo	Namaacha	21888	2015	58.4	51.0	66.3
Mozambique	Nampula	Angoche	21889	2000	170.7	154.0	188.9
Mozambique	Nampula	Angoche	21889	2005	142.6	129.1	157.9
Mozambique	Nampula	Angoche	21889	2010	116.4	103.2	132.4
Mozambique	Nampula	Angoche	21889	2015	94.3	81.3	109.8
Mozambique	Nampula	Cidade de Nampula	65259	2000	140.3	128.0	152.7
Mozambique	Nampula	Cidade de Nampula	65259	2005	111.4	101.7	122.7
Mozambique	Nampula	Cidade de Nampula	65259	2010	85.2	75.9	96.2
Mozambique	Nampula	Cidade de Nampula	65259	2015	70.6	60.9	82.3
Mozambique	Nampula	Erati	21890	2000	188.2	174.0	204.5
Mozambique	Nampula	Erati	21890	2005	143.1	131.2	157.0
Mozambique	Nampula	Erati	21890	2010	112.6	100.6	125.6
Mozambique	Nampula	Erati	21890	2015	92.3	79.9	105.3
Mozambique	Nampula	Ilha de Mocambique	65260	2000	158.9	140.4	178.7
Mozambique	Nampula	Ilha de Mocambique	65260	2005	131.5	116.3	150.3
Mozambique	Nampula	Ilha de Mocambique	65260	2010	105.4	90.4	122.4
Mozambique	Nampula	Ilha de Mocambique	65260	2015	85.2	72.2	101.5
Mozambique	Nampula	Lalaua	21891	2000	179.0	160.7	199.8
Mozambique	Nampula	Lalaua	21891	2005	137.2	121.7	152.9
Mozambique	Nampula	Lalaua	21891	2010	110.1	95.6	125.2
Mozambique	Nampula	Lalaua	21891	2015	86.5	74.3	99.4
Mozambique	Nampula	Malema	21892	2000	173.0	157.9	190.5
Mozambique	Nampula	Malema	21892	2005	136.8	124.0	152.0
Mozambique	Nampula	Malema	21892	2010	107.0	94.7	120.5
Mozambique	Nampula	Malema	21892	2015	83.7	73.1	94.9
Mozambique	Nampula	Meconta	21893	2000	164.8	153.1	178.5
Mozambique	Nampula	Meconta	21893	2005	133.6	123.6	144.4
Mozambique	Nampula	Meconta	21893	2010	104.0	92.8	116.5
Mozambique	Nampula	Meconta	21893	2015	86.1	74.3	98.1
Mozambique	Nampula	Mecuburi	21894	2000	186.6	169.0	205.1
Mozambique	Nampula	Mecuburi	21894	2005	142.5	129.1	156.7
Mozambique	Nampula	Mecuburi	21894	2010	111.9	99.2	125.0
Mozambique	Nampula	Mecuburi	21894	2015	89.7	77.4	102.6
Mozambique	Nampula	Memba	21895	2000	172.2	156.7	188.9
Mozambique	Nampula	Memba	21895	2005	133.2	120.6	147.0
Mozambique	Nampula	Memba	21895	2010	105.6	93.1	118.4

Admin 0	Admin 1	Admin 2	GAUL Code	Year	Under-5 mortality (per 1,000 livebirths)		
					Estimate	Lower bound, 95% UI	Upper bound, 95% UI
Mozambique	Nampula	Memba	21895	2015	89.3	76.9	102.8
Mozambique	Nampula	Mogincual	21896	2000	171.0	153.9	190.3
Mozambique	Nampula	Mogincual	21896	2005	140.5	127.4	156.2
Mozambique	Nampula	Mogincual	21896	2010	114.4	101.1	130.1
Mozambique	Nampula	Mogincual	21896	2015	94.3	80.1	108.9
Mozambique	Nampula	Mogovolas	21897	2000	168.5	155.6	183.4
Mozambique	Nampula	Mogovolas	21897	2005	138.9	127.5	151.4
Mozambique	Nampula	Mogovolas	21897	2010	112.5	100.0	125.9
Mozambique	Nampula	Mogovolas	21897	2015	90.7	78.8	104.2
Mozambique	Nampula	Moma	21898	2000	169.7	153.7	188.0
Mozambique	Nampula	Moma	21898	2005	141.2	127.2	155.6
Mozambique	Nampula	Moma	21898	2010	116.6	101.6	131.1
Mozambique	Nampula	Moma	21898	2015	94.0	81.1	108.4
Mozambique	Nampula	Monapo	21899	2000	170.5	156.3	185.6
Mozambique	Nampula	Monapo	21899	2005	136.6	125.3	149.1
Mozambique	Nampula	Monapo	21899	2010	109.5	97.2	122.6
Mozambique	Nampula	Monapo	21899	2015	91.1	78.2	104.0
Mozambique	Nampula	Mossuril	21900	2000	164.3	148.6	182.2
Mozambique	Nampula	Mossuril	21900	2005	133.5	120.2	149.2
Mozambique	Nampula	Mossuril	21900	2010	105.3	92.7	119.7
Mozambique	Nampula	Mossuril	21900	2015	89.3	76.8	104.4
Mozambique	Nampula	Muecate	21901	2000	178.1	163.1	193.4
Mozambique	Nampula	Muecate	21901	2005	138.8	127.3	151.9
Mozambique	Nampula	Muecate	21901	2010	107.7	95.8	120.6
Mozambique	Nampula	Muecate	21901	2015	88.0	76.2	100.5
Mozambique	Nampula	Murrupula	21902	2000	177.0	159.5	195.6
Mozambique	Nampula	Murrupula	21902	2005	141.6	127.5	156.5
Mozambique	Nampula	Murrupula	21902	2010	110.0	97.5	126.0
Mozambique	Nampula	Murrupula	21902	2015	89.3	77.0	103.1
Mozambique	Nampula	Nacala	65261	2000	138.5	123.5	154.7
Mozambique	Nampula	Nacala	65261	2005	109.1	97.0	121.7
Mozambique	Nampula	Nacala	65261	2010	81.0	70.8	92.2
Mozambique	Nampula	Nacala	65261	2015	70.6	60.4	82.3
Mozambique	Nampula	Nacala-A-Velha	21903	2000	166.9	152.0	184.3
Mozambique	Nampula	Nacala-A-Velha	21903	2005	131.9	119.3	145.6
Mozambique	Nampula	Nacala-A-Velha	21903	2010	104.0	91.5	117.8
Mozambique	Nampula	Nacala-A-Velha	21903	2015	88.7	76.2	102.7
Mozambique	Nampula	Nacaroa	21904	2000	176.2	160.6	193.8
Mozambique	Nampula	Nacaroa	21904	2005	137.6	124.9	151.3
Mozambique	Nampula	Nacaroa	21904	2010	109.5	97.6	123.7
Mozambique	Nampula	Nacaroa	21904	2015	90.4	77.7	103.7
Mozambique	Nampula	Nampula	21905	2000	169.2	156.0	182.7
Mozambique	Nampula	Nampula	21905	2005	135.3	124.8	147.5
Mozambique	Nampula	Nampula	21905	2010	102.8	91.9	114.7
Mozambique	Nampula	Nampula	21905	2015	84.5	73.4	96.9
Mozambique	Nampula	Ribaue	21906	2000	179.2	162.0	198.3
Mozambique	Nampula	Ribaue	21906	2005	141.1	126.6	155.9
Mozambique	Nampula	Ribaue	21906	2010	108.0	95.6	122.2
Mozambique	Nampula	Ribaue	21906	2015	85.7	74.5	99.4
Mozambique	Niassa	Cidade de Lichinga	65262	2000	133.2	120.5	147.2
Mozambique	Niassa	Cidade de Lichinga	65262	2005	96.0	86.5	106.0
Mozambique	Niassa	Cidade de Lichinga	65262	2010	74.6	67.0	83.3
Mozambique	Niassa	Cidade de Lichinga	65262	2015	55.9	48.9	63.6
Mozambique	Niassa	Cuamba	21907	2000	146.2	133.4	159.4
Mozambique	Niassa	Cuamba	21907	2005	112.5	102.5	123.0
Mozambique	Niassa	Cuamba	21907	2010	87.9	79.2	97.3
Mozambique	Niassa	Cuamba	21907	2015	69.2	61.2	78.7
Mozambique	Niassa	Lago	21908	2000	135.1	123.3	149.0
Mozambique	Niassa	Lago	21908	2005	99.0	90.8	109.0
Mozambique	Niassa	Lago	21908	2010	79.2	71.8	87.7
Mozambique	Niassa	Lago	21908	2015	59.1	52.7	66.2
Mozambique	Niassa	Lichinga	21909	2000	148.8	137.7	160.4
Mozambique	Niassa	Lichinga	21909	2005	112.3	103.5	121.7
Mozambique	Niassa	Lichinga	21909	2010	90.6	82.9	99.5
Mozambique	Niassa	Lichinga	21909	2015	65.4	58.9	73.6
Mozambique	Niassa	Majune	21910	2000	150.1	132.4	168.3
Mozambique	Niassa	Majune	21910	2005	115.9	101.4	131.0
Mozambique	Niassa	Majune	21910	2010	96.1	83.4	109.3
Mozambique	Niassa	Majune	21910	2015	74.4	63.9	86.1
Mozambique	Niassa	Mandimba	21911	2000	151.2	138.8	165.1
Mozambique	Niassa	Mandimba	21911	2005	114.5	104.7	125.4
Mozambique	Niassa	Mandimba	21911	2010	94.3	85.5	104.2
Mozambique	Niassa	Mandimba	21911	2015	69.8	62.1	78.4
Mozambique	Niassa	Marrupa	21912	2000	154.6	137.2	173.9
Mozambique	Niassa	Marrupa	21912	2005	116.2	103.4	131.2
Mozambique	Niassa	Marrupa	21912	2010	97.2	84.9	110.4
Mozambique	Niassa	Marrupa	21912	2015	78.1	67.4	90.6
Mozambique	Niassa	Maua	21913	2000	150.8	135.1	169.6
Mozambique	Niassa	Maua	21913	2005	115.6	102.8	129.9
Mozambique	Niassa	Maua	21913	2010	96.2	84.8	109.0
Mozambique	Niassa	Maua	21913	2015	74.1	64.6	84.8
Mozambique	Niassa	Mavago	21914	2000	155.8	135.5	181.1
Mozambique	Niassa	Mavago	21914	2005	123.1	107.4	142.2
Mozambique	Niassa	Mavago	21914	2010	99.5	85.4	117.3
Mozambique	Niassa	Mavago	21914	2015	78.5	66.2	93.1

Admin 0	Admin 1	Admin 2	GAUL Code	Year	Under-5 mortality (per 1,000 livebirths)		
					Estimate	Lower bound, 95% UI	Upper bound, 95% UI
Mozambique	Niassa	Mecanhelas	21915	2000	154.1	143.4	165.1
Mozambique	Niassa	Mecanhelas	21915	2005	114.5	106.6	123.2
Mozambique	Niassa	Mecanhelas	21915	2010	91.9	84.5	99.9
Mozambique	Niassa	Mecanhelas	21915	2015	69.2	62.1	76.3
Mozambique	Niassa	Mecula	21916	2000	160.3	142.0	180.1
Mozambique	Niassa	Mecula	21916	2005	130.0	114.8	146.6
Mozambique	Niassa	Mecula	21916	2010	100.6	87.6	114.3
Mozambique	Niassa	Mecula	21916	2015	78.8	68.1	90.4
Mozambique	Niassa	Metarica	21917	2000	153.0	138.0	170.4
Mozambique	Niassa	Metarica	21917	2005	119.5	107.6	133.2
Mozambique	Niassa	Metarica	21917	2010	95.9	85.0	108.2
Mozambique	Niassa	Metarica	21917	2015	74.1	65.1	84.1
Mozambique	Niassa	Muembe	21918	2000	150.7	133.1	169.6
Mozambique	Niassa	Muembe	21918	2005	117.1	103.5	132.9
Mozambique	Niassa	Muembe	21918	2010	96.0	83.7	110.3
Mozambique	Niassa	Muembe	21918	2015	72.8	63.0	84.5
Mozambique	Niassa	Ngauma	21919	2000	156.0	143.1	169.7
Mozambique	Niassa	Ngauma	21919	2005	118.6	108.2	129.7
Mozambique	Niassa	Ngauma	21919	2010	98.5	89.1	109.3
Mozambique	Niassa	Ngauma	21919	2015	71.5	63.6	81.3
Mozambique	Niassa	Nipepe	21920	2000	171.6	152.0	191.4
Mozambique	Niassa	Nipepe	21920	2005	130.3	114.6	145.9
Mozambique	Niassa	Nipepe	21920	2010	108.1	94.3	123.5
Mozambique	Niassa	Nipepe	21920	2015	84.8	72.9	97.8
Mozambique	Niassa	Sanga	21921	2000	146.2	131.3	163.5
Mozambique	Niassa	Sanga	21921	2005	113.0	101.7	126.1
Mozambique	Niassa	Sanga	21921	2010	90.6	81.0	101.9
Mozambique	Niassa	Sanga	21921	2015	67.8	59.7	77.2
Mozambique	Sofala	Buzi	21922	2000	162.5	149.2	176.6
Mozambique	Sofala	Buzi	21922	2005	135.4	124.4	146.8
Mozambique	Sofala	Buzi	21922	2010	100.7	90.5	111.5
Mozambique	Sofala	Buzi	21922	2015	81.2	70.9	91.6
Mozambique	Sofala	Caia	21923	2000	164.5	151.4	178.9
Mozambique	Sofala	Caia	21923	2005	124.4	113.4	135.5
Mozambique	Sofala	Caia	21923	2010	101.5	91.0	113.3
Mozambique	Sofala	Caia	21923	2015	79.3	69.8	89.9
Mozambique	Sofala	Chemba	21924	2000	168.0	153.7	183.3
Mozambique	Sofala	Chemba	21924	2005	120.6	110.4	132.1
Mozambique	Sofala	Chemba	21924	2010	98.9	89.4	109.4
Mozambique	Sofala	Chemba	21924	2015	73.1	64.7	82.0
Mozambique	Sofala	Cheringoma	21925	2000	173.0	157.1	190.5
Mozambique	Sofala	Cheringoma	21925	2005	135.2	121.7	149.6
Mozambique	Sofala	Cheringoma	21925	2010	106.7	94.7	121.2
Mozambique	Sofala	Cheringoma	21925	2015	85.9	74.7	98.6
Mozambique	Sofala	Chibabava	21926	2000	149.9	136.2	164.3
Mozambique	Sofala	Chibabava	21926	2005	131.9	119.6	144.2
Mozambique	Sofala	Chibabava	21926	2010	98.4	88.5	109.0
Mozambique	Sofala	Chibabava	21926	2015	76.3	67.1	86.6
Mozambique	Sofala	Cidade da Beira	65263	2000	139.8	127.5	153.4
Mozambique	Sofala	Cidade da Beira	65263	2005	110.1	100.3	120.5
Mozambique	Sofala	Cidade da Beira	65263	2010	89.6	79.2	100.3
Mozambique	Sofala	Cidade da Beira	65263	2015	71.2	61.4	82.0
Mozambique	Sofala	Dondo	21927	2000	157.6	144.3	171.7
Mozambique	Sofala	Dondo	21927	2005	127.1	115.8	138.9
Mozambique	Sofala	Dondo	21927	2010	98.6	88.4	109.7
Mozambique	Sofala	Dondo	21927	2015	80.7	70.2	92.9
Mozambique	Sofala	Gorongosa	21928	2000	171.9	157.6	187.1
Mozambique	Sofala	Gorongosa	21928	2005	139.1	126.9	152.0
Mozambique	Sofala	Gorongosa	21928	2010	109.1	98.5	121.5
Mozambique	Sofala	Gorongosa	21928	2015	85.4	74.9	96.5
Mozambique	Sofala	Machanga	21929	2000	151.0	133.7	170.6
Mozambique	Sofala	Machanga	21929	2005	127.1	113.3	144.2
Mozambique	Sofala	Machanga	21929	2010	95.6	84.2	108.8
Mozambique	Sofala	Machanga	21929	2015	77.7	66.8	90.9
Mozambique	Sofala	Maringue	21930	2000	167.6	153.9	184.0
Mozambique	Sofala	Maringue	21930	2005	129.3	118.4	142.2
Mozambique	Sofala	Maringue	21930	2010	104.8	93.9	116.2
Mozambique	Sofala	Maringue	21930	2015	78.4	69.4	89.3
Mozambique	Sofala	Marromeu	21931	2000	179.5	162.5	197.5
Mozambique	Sofala	Marromeu	21931	2005	139.6	125.7	155.3
Mozambique	Sofala	Marromeu	21931	2010	113.8	100.6	128.7
Mozambique	Sofala	Marromeu	21931	2015	94.0	81.3	108.2
Mozambique	Sofala	Muanza	21932	2000	174.0	157.1	192.5
Mozambique	Sofala	Muanza	21932	2005	137.2	123.3	151.9
Mozambique	Sofala	Muanza	21932	2010	107.4	94.7	120.3
Mozambique	Sofala	Muanza	21932	2015	87.5	75.8	100.3
Mozambique	Sofala	Nhamatanda	21933	2000	160.1	148.6	173.0
Mozambique	Sofala	Nhamatanda	21933	2005	135.8	126.2	147.6
Mozambique	Sofala	Nhamatanda	21933	2010	106.4	96.0	117.3
Mozambique	Sofala	Nhamatanda	21933	2015	85.9	75.6	97.6
Mozambique	Tete	Angonia	21934	2000	168.1	158.7	177.9
Mozambique	Tete	Angonia	21934	2005	132.6	125.4	140.2
Mozambique	Tete	Angonia	21934	2010	108.3	101.4	115.9
Mozambique	Tete	Angonia	21934	2015	75.5	68.9	82.5
Mozambique	Tete	Cahora Bassa	21935	2000	142.2	128.9	156.8

Admin 0	Admin 1	Admin 2	GAUL Code	Year	Under-5 mortality (per 1,000 livebirths)		
					Estimate	Lower bound, 95% UI	Upper bound, 95% UI
Mozambique	Tete	Cahora Bassa	21935	2005	118.6	107.1	131.9
Mozambique	Tete	Cahora Bassa	21935	2010	97.8	87.2	109.2
Mozambique	Tete	Cahora Bassa	21935	2015	77.8	68.2	89.3
Mozambique	Tete	Changara	21936	2000	159.8	146.7	174.2
Mozambique	Tete	Changara	21936	2005	126.3	116.0	138.2
Mozambique	Tete	Changara	21936	2010	101.9	91.7	113.7
Mozambique	Tete	Changara	21936	2015	80.0	70.1	91.3
Mozambique	Tete	Chifunde	21937	2000	175.7	161.8	190.6
Mozambique	Tete	Chifunde	21937	2005	142.6	131.3	155.3
Mozambique	Tete	Chifunde	21937	2010	121.1	110.2	133.3
Mozambique	Tete	Chifunde	21937	2015	92.1	81.8	103.3
Mozambique	Tete	Chiuta	21938	2000	174.4	159.1	191.7
Mozambique	Tete	Chiuta	21938	2005	143.0	130.3	156.5
Mozambique	Tete	Chiuta	21938	2010	117.3	105.8	130.2
Mozambique	Tete	Chiuta	21938	2015	87.6	77.0	99.8
Mozambique	Tete	Cidade de Tete	65264	2000	141.0	129.4	155.9
Mozambique	Tete	Cidade de Tete	65264	2005	107.6	97.9	118.6
Mozambique	Tete	Cidade de Tete	65264	2010	88.2	78.7	98.3
Mozambique	Tete	Cidade de Tete	65264	2015	68.6	59.2	78.9
Mozambique	Tete	Luenha	65265	2000	161.0	144.0	178.0
Mozambique	Tete	Luenha	65265	2005	127.2	113.5	142.6
Mozambique	Tete	Luenha	65265	2010	98.6	87.2	111.8
Mozambique	Tete	Luenha	65265	2015	78.3	67.7	89.5
Mozambique	Tete	Macanga	21939	2000	182.8	169.4	197.5
Mozambique	Tete	Macanga	21939	2005	147.3	136.2	158.4
Mozambique	Tete	Macanga	21939	2010	125.1	114.3	136.6
Mozambique	Tete	Macanga	21939	2015	89.0	79.6	99.1
Mozambique	Tete	Magoe	21940	2000	137.5	125.0	152.8
Mozambique	Tete	Magoe	21940	2005	115.6	105.1	127.3
Mozambique	Tete	Magoe	21940	2010	98.4	87.9	109.4
Mozambique	Tete	Magoe	21940	2015	81.4	71.6	92.3
Mozambique	Tete	Maravia	21941	2000	173.7	157.3	190.2
Mozambique	Tete	Maravia	21941	2005	142.1	129.4	156.1
Mozambique	Tete	Maravia	21941	2010	120.2	108.0	133.2
Mozambique	Tete	Maravia	21941	2015	97.4	86.1	110.8
Mozambique	Tete	Moatize	21942	2000	162.7	152.2	174.6
Mozambique	Tete	Moatize	21942	2005	127.3	118.5	136.7
Mozambique	Tete	Moatize	21942	2010	101.7	93.1	110.5
Mozambique	Tete	Moatize	21942	2015	77.5	69.5	86.1
Mozambique	Tete	Mutarara	21943	2000	170.5	160.2	181.0
Mozambique	Tete	Mutarara	21943	2005	121.0	113.8	128.6
Mozambique	Tete	Mutarara	21943	2010	100.1	93.2	107.6
Mozambique	Tete	Mutarara	21943	2015	73.7	67.0	80.6
Mozambique	Tete	Tsangano	21944	2000	160.9	151.3	170.8
Mozambique	Tete	Tsangano	21944	2005	127.3	119.8	135.3
Mozambique	Tete	Tsangano	21944	2010	100.9	93.9	109.0
Mozambique	Tete	Tsangano	21944	2015	72.5	65.8	79.8
Mozambique	Tete	Zumbu	21945	2000	168.3	151.1	187.4
Mozambique	Tete	Zumbu	21945	2005	137.6	123.3	153.0
Mozambique	Tete	Zumbu	21945	2010	115.0	101.1	129.2
Mozambique	Tete	Zumbu	21945	2015	100.2	86.6	114.3
Mozambique	Zambezia	Alto Molocue	21955	2000	183.5	167.5	201.5
Mozambique	Zambezia	Alto Molocue	21955	2005	145.4	132.2	160.1
Mozambique	Zambezia	Alto Molocue	21955	2010	116.5	103.3	131.4
Mozambique	Zambezia	Alto Molocue	21955	2015	93.1	81.4	106.3
Mozambique	Zambezia	Chinde	21946	2000	194.2	175.7	215.4
Mozambique	Zambezia	Chinde	21946	2005	154.8	138.3	173.7
Mozambique	Zambezia	Chinde	21946	2010	124.6	108.3	139.8
Mozambique	Zambezia	Chinde	21946	2015	105.5	90.6	121.2
Mozambique	Zambezia	Cidade de Quelimane	65266	2000	162.8	147.2	179.3
Mozambique	Zambezia	Cidade de Quelimane	65266	2005	132.6	119.7	147.3
Mozambique	Zambezia	Cidade de Quelimane	65266	2010	103.0	90.7	116.0
Mozambique	Zambezia	Cidade de Quelimane	65266	2015	88.6	76.2	102.6
Mozambique	Zambezia	Gile	21947	2000	181.8	164.7	200.0
Mozambique	Zambezia	Gile	21947	2005	146.7	133.0	162.6
Mozambique	Zambezia	Gile	21947	2010	115.9	102.9	131.9
Mozambique	Zambezia	Gile	21947	2015	93.6	80.9	108.4
Mozambique	Zambezia	Gurue	21948	2000	184.7	168.5	201.2
Mozambique	Zambezia	Gurue	21948	2005	144.9	132.1	158.4
Mozambique	Zambezia	Gurue	21948	2010	115.8	103.4	128.4
Mozambique	Zambezia	Gurue	21948	2015	91.8	80.3	103.6
Mozambique	Zambezia	Ile	21949	2000	197.1	181.6	214.6
Mozambique	Zambezia	Ile	21949	2005	157.9	145.2	172.8
Mozambique	Zambezia	Ile	21949	2010	128.7	115.6	143.3
Mozambique	Zambezia	Ile	21949	2015	102.2	89.1	116.6
Mozambique	Zambezia	Inhassunge	21950	2000	189.0	170.8	209.2
Mozambique	Zambezia	Inhassunge	21950	2005	152.2	137.0	169.5
Mozambique	Zambezia	Inhassunge	21950	2010	112.8	99.1	127.5
Mozambique	Zambezia	Inhassunge	21950	2015	99.4	85.8	115.3
Mozambique	Zambezia	Lugela	21951	2000	204.4	186.2	222.1
Mozambique	Zambezia	Lugela	21951	2005	162.6	149.7	177.2
Mozambique	Zambezia	Lugela	21951	2010	133.5	121.0	148.7
Mozambique	Zambezia	Lugela	21951	2015	104.1	92.8	117.0
Mozambique	Zambezia	Maganja da Costa	21952	2000	197.8	180.2	215.5
Mozambique	Zambezia	Maganja da Costa	21952	2005	161.6	147.2	177.7

Admin 0	Admin 1	Admin 2	GAUL Code	Year	Under-5 mortality (per 1,000 livebirths)		
					Estimate	Lower bound, 95% UI	Upper bound, 95% UI
Mozambique	Zambezia	Maganja da Costa	21952	2010	135.6	121.0	152.1
Mozambique	Zambezia	Maganja da Costa	21952	2015	110.8	95.9	127.1
Mozambique	Zambezia	Milange	21953	2000	187.4	176.6	198.0
Mozambique	Zambezia	Milange	21953	2005	145.6	137.2	154.0
Mozambique	Zambezia	Milange	21953	2010	121.1	112.7	129.2
Mozambique	Zambezia	Milange	21953	2015	88.0	80.2	96.3
Mozambique	Zambezia	Mocuba	21954	2000	202.0	185.1	219.0
Mozambique	Zambezia	Mocuba	21954	2005	162.0	149.6	176.1
Mozambique	Zambezia	Mocuba	21954	2010	130.5	117.8	144.3
Mozambique	Zambezia	Mocuba	21954	2015	107.1	94.3	119.8
Mozambique	Zambezia	Mopeia	21956	2000	188.2	171.5	206.9
Mozambique	Zambezia	Mopeia	21956	2005	149.6	136.8	164.6
Mozambique	Zambezia	Mopeia	21956	2010	118.5	105.6	132.2
Mozambique	Zambezia	Mopeia	21956	2015	98.7	86.2	112.0
Mozambique	Zambezia	Morrumbala	21957	2000	191.3	177.9	204.4
Mozambique	Zambezia	Morrumbala	21957	2005	145.2	135.2	156.3
Mozambique	Zambezia	Morrumbala	21957	2010	116.5	106.5	127.4
Mozambique	Zambezia	Morrumbala	21957	2015	90.2	80.6	100.1
Mozambique	Zambezia	Namacurra	21958	2000	199.2	182.1	218.4
Mozambique	Zambezia	Namacurra	21958	2005	161.3	147.0	176.9
Mozambique	Zambezia	Namacurra	21958	2010	123.6	110.4	139.1
Mozambique	Zambezia	Namacurra	21958	2015	107.2	93.8	122.9
Mozambique	Zambezia	Namarroi	21959	2000	201.3	185.3	217.7
Mozambique	Zambezia	Namarroi	21959	2005	158.2	144.6	171.3
Mozambique	Zambezia	Namarroi	21959	2010	131.1	118.1	144.5
Mozambique	Zambezia	Namarroi	21959	2015	101.0	89.0	113.6
Mozambique	Zambezia	Nicoadala	21960	2000	194.2	177.7	212.0
Mozambique	Zambezia	Nicoadala	21960	2005	157.3	142.9	172.7
Mozambique	Zambezia	Nicoadala	21960	2010	117.5	104.9	131.6
Mozambique	Zambezia	Nicoadala	21960	2015	103.1	89.9	117.9
Mozambique	Zambezia	Pebane	21961	2000	178.2	159.6	198.2
Mozambique	Zambezia	Pebane	21961	2005	148.8	133.6	165.9
Mozambique	Zambezia	Pebane	21961	2010	123.6	108.2	140.2
Mozambique	Zambezia	Pebane	21961	2015	101.2	87.4	117.4
Namibia	Caprivi	Kabe	42154	2000	82.5	72.6	93.4
Namibia	Caprivi	Kabe	42154	2005	77.5	68.4	87.6
Namibia	Caprivi	Kabe	42154	2010	68.5	59.7	78.0
Namibia	Caprivi	Kabe	42154	2015	49.5	42.1	57.8
Namibia	Caprivi	Katima Muliro Rural	42155	2000	79.9	70.3	91.0
Namibia	Caprivi	Katima Muliro Rural	42155	2005	77.3	68.1	87.5
Namibia	Caprivi	Katima Muliro Rural	42155	2010	68.9	60.0	79.0
Namibia	Caprivi	Katima Muliro Rural	42155	2015	50.3	42.4	58.9
Namibia	Caprivi	Katima Muliro Urban	42156	2000	79.4	69.4	90.8
Namibia	Caprivi	Katima Muliro Urban	42156	2005	76.7	67.2	87.6
Namibia	Caprivi	Katima Muliro Urban	42156	2010	67.6	58.5	77.9
Namibia	Caprivi	Katima Muliro Urban	42156	2015	49.5	41.5	58.4
Namibia	Caprivi	Kongola	22250	2000	89.3	76.7	102.8
Namibia	Caprivi	Kongola	22250	2005	82.6	70.8	95.5
Namibia	Caprivi	Kongola	22250	2010	71.6	61.0	82.8
Namibia	Caprivi	Kongola	22250	2015	53.2	44.7	63.1
Namibia	Caprivi	Linyandi	22251	2000	82.6	70.5	95.8
Namibia	Caprivi	Linyandi	22251	2005	77.9	66.1	90.1
Namibia	Caprivi	Linyandi	22251	2010	69.1	58.1	81.5
Namibia	Caprivi	Linyandi	22251	2015	51.3	42.2	61.5
Namibia	Caprivi	Sibinda	22252	2000	80.0	69.4	91.9
Namibia	Caprivi	Sibinda	22252	2005	77.0	66.7	88.7
Namibia	Caprivi	Sibinda	22252	2010	68.9	59.4	80.0
Namibia	Caprivi	Sibinda	22252	2015	50.9	42.9	60.4
Namibia	Erongo	Arandis	22253	2000	44.2	36.1	53.9
Namibia	Erongo	Arandis	22253	2005	49.0	39.6	60.1
Namibia	Erongo	Arandis	22253	2010	44.1	35.8	54.2
Namibia	Erongo	Arandis	22253	2015	32.0	25.3	39.1
Namibia	Erongo	Daures	22254	2000	49.5	40.2	61.0
Namibia	Erongo	Daures	22254	2005	51.9	42.3	64.0
Namibia	Erongo	Daures	22254	2010	45.5	37.6	55.5
Namibia	Erongo	Daures	22254	2015	33.7	27.3	41.5
Namibia	Erongo	Karibib	22255	2000	50.0	41.1	60.8
Namibia	Erongo	Karibib	22255	2005	52.9	43.3	64.0
Namibia	Erongo	Karibib	22255	2010	46.1	37.7	55.6
Namibia	Erongo	Karibib	22255	2015	34.5	27.4	42.7
Namibia	Erongo	Omaruru	22256	2000	51.3	41.2	62.8
Namibia	Erongo	Omaruru	22256	2005	54.1	43.9	67.3
Namibia	Erongo	Omaruru	22256	2010	46.4	37.6	57.7
Namibia	Erongo	Omaruru	22256	2015	34.5	27.5	42.7
Namibia	Erongo	Swakopmund	22257	2000	44.5	36.0	54.2
Namibia	Erongo	Swakopmund	22257	2005	48.6	39.5	58.8
Namibia	Erongo	Swakopmund	22257	2010	43.7	35.0	53.9
Namibia	Erongo	Swakopmund	22257	2015	31.3	24.7	38.8
Namibia	Erongo	Walvisbay Rural	22258	2000	45.7	37.4	55.8
Namibia	Erongo	Walvisbay Rural	22258	2005	50.1	40.9	61.9
Namibia	Erongo	Walvisbay Rural	22258	2010	45.3	36.6	55.5
Namibia	Erongo	Walvisbay Rural	22258	2015	32.3	25.7	40.0
Namibia	Erongo	Walvisbay Urban	22259	2000	44.8	36.1	55.4
Namibia	Erongo	Walvisbay Urban	22259	2005	48.9	39.4	61.2
Namibia	Erongo	Walvisbay Urban	22259	2010	44.4	35.6	54.8

Admin 0	Admin 1	Admin 2	GAUL Code	Year	Under-5 mortality (per 1,000 livebirths)		
					Estimate	Lower bound, 95% UI	Upper bound, 95% UI
Namibia	Erongo	Walvisbay Urban	22259	2015	32.2	25.1	40.5
Namibia	Hardap	Gibeon	22260	2000	50.8	41.8	62.4
Namibia	Hardap	Gibeon	22260	2005	53.6	43.6	65.8
Namibia	Hardap	Gibeon	22260	2010	46.5	37.8	56.8
Namibia	Hardap	Gibeon	22260	2015	35.8	29.0	44.3
Namibia	Hardap	Mariental Rural	22261	2000	50.8	42.8	60.7
Namibia	Hardap	Mariental Rural	22261	2005	51.6	43.3	61.5
Namibia	Hardap	Mariental Rural	22261	2010	44.8	37.5	54.2
Namibia	Hardap	Mariental Rural	22261	2015	36.2	30.1	44.3
Namibia	Hardap	Mariental Urban	22262	2000	49.2	39.5	61.2
Namibia	Hardap	Mariental Urban	22262	2005	50.9	41.3	63.1
Namibia	Hardap	Mariental Urban	22262	2010	43.9	35.5	54.9
Namibia	Hardap	Mariental Urban	22262	2015	34.5	27.5	43.7
Namibia	Hardap	Rehoboth East	22263	2000	49.0	40.8	58.2
Namibia	Hardap	Rehoboth East	22263	2005	49.8	41.3	59.5
Namibia	Hardap	Rehoboth East	22263	2010	42.8	35.2	52.2
Namibia	Hardap	Rehoboth East	22263	2015	32.3	25.5	39.3
Namibia	Hardap	Rehoboth Rural	22264	2000	51.1	42.1	62.1
Namibia	Hardap	Rehoboth Rural	22264	2005	52.8	42.8	63.4
Namibia	Hardap	Rehoboth Rural	22264	2010	45.6	37.2	55.8
Namibia	Hardap	Rehoboth Rural	22264	2015	34.2	27.2	41.7
Namibia	Hardap	Rehoboth West	22265	2000	48.3	40.0	57.3
Namibia	Hardap	Rehoboth West	22265	2005	49.6	40.9	59.0
Namibia	Hardap	Rehoboth West	22265	2010	42.7	34.9	52.2
Namibia	Hardap	Rehoboth West	22265	2015	32.0	25.3	39.3
Namibia	Karas	Berseba	22266	2000	47.0	38.6	56.5
Namibia	Karas	Berseba	22266	2005	50.3	40.9	60.5
Namibia	Karas	Berseba	22266	2010	44.6	36.2	54.2
Namibia	Karas	Berseba	22266	2015	35.2	28.5	43.2
Namibia	Karas	Karas	22267	2000	49.8	40.2	60.0
Namibia	Karas	Karas	22267	2005	53.4	43.2	64.4
Namibia	Karas	Karas	22267	2010	47.4	38.7	57.2
Namibia	Karas	Karas	22267	2015	36.0	29.2	44.1
Namibia	Karas	Keetmanshoop Rural	22268	2000	49.6	40.7	60.3
Namibia	Karas	Keetmanshoop Rural	22268	2005	53.2	43.7	65.2
Namibia	Karas	Keetmanshoop Rural	22268	2010	46.8	38.6	57.6
Namibia	Karas	Keetmanshoop Rural	22268	2015	37.6	30.7	46.0
Namibia	Karas	Keetmanshoop Urban	22269	2000	42.5	33.3	53.5
Namibia	Karas	Keetmanshoop Urban	22269	2005	45.2	35.1	57.2
Namibia	Karas	Keetmanshoop Urban	22269	2010	40.3	31.7	51.2
Namibia	Karas	Keetmanshoop Urban	22269	2015	32.9	25.8	42.3
Namibia	Karas	Luderitz	22270	2000	46.5	37.0	57.5
Namibia	Karas	Luderitz	22270	2005	51.1	40.9	62.9
Namibia	Karas	Luderitz	22270	2010	46.9	37.4	58.6
Namibia	Karas	Luderitz	22270	2015	34.3	27.6	42.8
Namibia	Karas	Oranjemund	22271	2000	44.6	35.2	56.4
Namibia	Karas	Oranjemund	22271	2005	49.4	38.4	62.6
Namibia	Karas	Oranjemund	22271	2010	46.0	35.9	58.4
Namibia	Karas	Oranjemund	22271	2015	33.7	26.2	42.8
Namibia	Kavango	Kahenge	22272	2000	76.2	65.3	88.8
Namibia	Kavango	Kahenge	22272	2005	76.5	65.2	88.5
Namibia	Kavango	Kahenge	22272	2010	59.4	50.3	69.8
Namibia	Kavango	Kahenge	22272	2015	45.6	37.4	53.9
Namibia	Kavango	Kapako	22273	2000	72.6	62.5	84.8
Namibia	Kavango	Kapako	22273	2005	72.1	62.0	83.6
Namibia	Kavango	Kapako	22273	2010	58.2	49.7	68.1
Namibia	Kavango	Kapako	22273	2015	45.1	37.9	53.7
Namibia	Kavango	Mashare	22274	2000	74.7	63.8	87.2
Namibia	Kavango	Mashare	22274	2005	72.2	61.4	84.4
Namibia	Kavango	Mashare	22274	2010	58.4	49.1	69.0
Namibia	Kavango	Mashare	22274	2015	45.4	37.3	53.9
Namibia	Kavango	Mpungu	22275	2000	82.1	69.5	97.3
Namibia	Kavango	Mpungu	22275	2005	82.9	70.4	98.4
Namibia	Kavango	Mpungu	22275	2010	63.8	53.8	77.4
Namibia	Kavango	Mpungu	22275	2015	47.9	39.6	58.8
Namibia	Kavango	Mukwe	22276	2000	89.9	75.0	107.7
Namibia	Kavango	Mukwe	22276	2005	80.0	66.3	94.5
Namibia	Kavango	Mukwe	22276	2010	63.1	51.6	76.1
Namibia	Kavango	Mukwe	22276	2015	49.1	39.4	59.7
Namibia	Kavango	Ndiyona	22277	2000	75.9	63.2	89.7
Namibia	Kavango	Ndiyona	22277	2005	72.0	59.6	85.3
Namibia	Kavango	Ndiyona	22277	2010	57.5	47.5	68.6
Namibia	Kavango	Ndiyona	22277	2015	45.6	36.8	54.6
Namibia	Kavango	Rundu Rural East	42157	2000	72.7	62.0	84.8
Namibia	Kavango	Rundu Rural East	42157	2005	72.1	61.7	84.0
Namibia	Kavango	Rundu Rural East	42157	2010	58.5	49.7	68.4
Namibia	Kavango	Rundu Rural East	42157	2015	45.3	37.6	53.8
Namibia	Kavango	Rundu Rural West	42158	2000	71.0	61.0	82.6
Namibia	Kavango	Rundu Rural West	42158	2005	70.5	60.5	82.0
Namibia	Kavango	Rundu Rural West	42158	2010	57.1	48.8	66.3
Namibia	Kavango	Rundu Rural West	42158	2015	44.2	37.1	52.5
Namibia	Kavango	Rundu Urban	22279	2000	73.0	62.2	85.8
Namibia	Kavango	Rundu Urban	22279	2005	76.3	64.9	89.2
Namibia	Kavango	Rundu Urban	22279	2010	66.5	56.5	77.9
Namibia	Kavango	Rundu Urban	22279	2015	41.6	34.3	50.0

Admin 0	Admin 1	Admin 2	GAUL Code	Year	Under-5 mortality (per 1,000 livebirths)		
					Estimate	Lower bound, 95% UI	Upper bound, 95% UI
Namibia	Khomas	Katutura Central	22281	2000	41.8	35.5	48.7
Namibia	Khomas	Katutura Central	22281	2005	44.5	38.2	52.2
Namibia	Khomas	Katutura Central	22281	2010	39.3	33.3	46.7
Namibia	Khomas	Katutura Central	22281	2015	29.6	24.4	35.7
Namibia	Khomas	Katutura East	22282	2000	41.8	35.5	48.7
Namibia	Khomas	Katutura East	22282	2005	44.5	38.2	52.2
Namibia	Khomas	Katutura East	22282	2010	39.3	33.3	46.7
Namibia	Khomas	Katutura East	22282	2015	29.6	24.4	35.7
Namibia	Khomas	Khomasdal North	22283	2000	42.9	36.6	49.9
Namibia	Khomas	Khomasdal North	22283	2005	45.1	38.6	53.1
Namibia	Khomas	Khomasdal North	22283	2010	39.9	33.8	47.4
Namibia	Khomas	Khomasdal North	22283	2015	30.3	24.9	36.4
Namibia	Khomas	Moses Garoeb	42170	2000	45.2	38.6	52.5
Namibia	Khomas	Moses Garoeb	42170	2005	45.6	39.2	53.7
Namibia	Khomas	Moses Garoeb	42170	2010	40.4	34.1	47.9
Namibia	Khomas	Moses Garoeb	42170	2015	30.6	25.4	36.8
Namibia	Khomas	Soweto	22284	2000	41.8	35.5	48.7
Namibia	Khomas	Soweto	22284	2005	44.5	38.2	52.2
Namibia	Khomas	Soweto	22284	2010	39.3	33.3	46.7
Namibia	Khomas	Soweto	22284	2015	29.6	24.4	35.7
Namibia	Khomas	Tobias Hainyeko	42169	2000	45.2	38.6	52.5
Namibia	Khomas	Tobias Hainyeko	42169	2005	45.6	39.2	53.7
Namibia	Khomas	Tobias Hainyeko	42169	2010	40.4	34.1	47.9
Namibia	Khomas	Tobias Hainyeko	42169	2015	30.6	25.4	36.8
Namibia	Khomas	Wanaheda	22285	2000	42.7	36.4	49.8
Namibia	Khomas	Wanaheda	22285	2005	44.6	38.2	52.5
Namibia	Khomas	Wanaheda	22285	2010	39.6	33.4	47.1
Namibia	Khomas	Wanaheda	22285	2015	29.8	24.6	35.9
Namibia	Khomas	Windhoek East	22286	2000	42.6	36.5	49.3
Namibia	Khomas	Windhoek East	22286	2005	44.8	38.6	52.3
Namibia	Khomas	Windhoek East	22286	2010	40.2	34.2	47.4
Namibia	Khomas	Windhoek East	22286	2015	30.0	24.9	36.1
Namibia	Khomas	Windhoek Rural	22287	2000	47.1	41.1	53.6
Namibia	Khomas	Windhoek Rural	22287	2005	49.1	43.0	56.0
Namibia	Khomas	Windhoek Rural	22287	2010	43.0	37.3	49.7
Namibia	Khomas	Windhoek Rural	22287	2015	32.3	27.3	38.1
Namibia	Khomas	Windhoek West	22288	2000	41.9	35.9	48.6
Namibia	Khomas	Windhoek West	22288	2005	44.4	38.2	52.1
Namibia	Khomas	Windhoek West	22288	2010	39.6	33.7	46.8
Namibia	Khomas	Windhoek West	22288	2015	29.8	24.6	35.9
Namibia	Kunene	Epupa	22289	2000	72.4	60.9	86.3
Namibia	Kunene	Epupa	22289	2005	67.0	56.8	79.9
Namibia	Kunene	Epupa	22289	2010	55.5	46.4	65.8
Namibia	Kunene	Epupa	22289	2015	42.2	35.0	50.9
Namibia	Kunene	Kamanjab	22290	2000	56.9	47.1	68.1
Namibia	Kunene	Kamanjab	22290	2005	57.9	48.3	69.1
Namibia	Kunene	Kamanjab	22290	2010	50.4	41.9	60.5
Namibia	Kunene	Kamanjab	22290	2015	36.1	29.5	43.5
Namibia	Kunene	Khorixas	22291	2000	52.2	42.2	62.6
Namibia	Kunene	Khorixas	22291	2005	53.7	44.0	64.4
Namibia	Kunene	Khorixas	22291	2010	47.6	38.5	57.9
Namibia	Kunene	Khorixas	22291	2015	34.3	27.5	42.1
Namibia	Kunene	Opuwo	22292	2000	66.4	55.1	78.5
Namibia	Kunene	Opuwo	22292	2005	61.8	51.4	73.1
Namibia	Kunene	Opuwo	22292	2010	51.7	43.0	60.9
Namibia	Kunene	Opuwo	22292	2015	39.5	32.8	47.9
Namibia	Kunene	Outjo	22293	2000	53.7	44.2	65.3
Namibia	Kunene	Outjo	22293	2005	56.0	46.1	68.4
Namibia	Kunene	Outjo	22293	2010	49.0	40.7	59.5
Namibia	Kunene	Outjo	22293	2015	34.2	28.2	41.6
Namibia	Kunene	Sesfontein	22294	2000	59.3	48.3	71.5
Namibia	Kunene	Sesfontein	22294	2005	57.1	47.0	69.0
Namibia	Kunene	Sesfontein	22294	2010	49.6	40.8	60.3
Namibia	Kunene	Sesfontein	22294	2015	36.7	30.1	44.5
Namibia	Ohangwena	Eenhana	42159	2000	77.8	68.4	89.3
Namibia	Ohangwena	Eenhana	42159	2005	79.7	69.8	91.3
Namibia	Ohangwena	Eenhana	42159	2010	64.2	55.5	74.0
Namibia	Ohangwena	Eenhana	42159	2015	48.7	40.9	57.4
Namibia	Ohangwena	Endola	42160	2000	73.4	65.8	81.4
Namibia	Ohangwena	Endola	42160	2005	76.8	68.4	85.5
Namibia	Ohangwena	Endola	42160	2010	62.6	55.1	70.5
Namibia	Ohangwena	Endola	42160	2015	45.6	38.9	52.8
Namibia	Ohangwena	Engela	22297	2000	74.2	65.9	83.0
Namibia	Ohangwena	Engela	22297	2005	78.8	69.6	88.3
Namibia	Ohangwena	Engela	22297	2010	63.5	55.6	72.1
Namibia	Ohangwena	Engela	22297	2015	46.5	39.6	53.8
Namibia	Ohangwena	Epembe	22298	2000	83.6	72.8	96.5
Namibia	Ohangwena	Epembe	22298	2005	83.6	71.9	95.8
Namibia	Ohangwena	Epembe	22298	2010	66.3	56.6	77.7
Namibia	Ohangwena	Epembe	22298	2015	51.3	42.6	61.0
Namibia	Ohangwena	Ohangwena	22299	2000	75.9	67.2	85.6
Namibia	Ohangwena	Ohangwena	22299	2005	80.1	70.5	90.2
Namibia	Ohangwena	Ohangwena	22299	2010	63.9	55.7	73.0
Namibia	Ohangwena	Ohangwena	22299	2015	47.0	39.7	54.6
Namibia	Ohangwena	Okongo	22300	2000	90.4	75.8	107.0

Admin 0	Admin 1	Admin 2	GAUL Code	Year	Under-5 mortality (per 1,000 livebirths)		
					Estimate	Lower bound, 95% UI	Upper bound, 95% UI
Namibia	Ohangwena	Okongo	22300	2005	88.0	74.0	103.8
Namibia	Ohangwena	Okongo	22300	2010	69.5	57.9	82.8
Namibia	Ohangwena	Okongo	22300	2015	53.3	43.6	64.4
Namibia	Ohangwena	Omulonga	42161	2000	74.4	66.7	83.2
Namibia	Ohangwena	Omulonga	42161	2005	78.0	69.2	86.7
Namibia	Ohangwena	Omulonga	42161	2010	63.4	55.6	71.6
Namibia	Ohangwena	Omulonga	42161	2015	46.9	39.9	54.3
Namibia	Ohangwena	Omundaungilo	22301	2000	84.9	73.1	98.4
Namibia	Ohangwena	Omundaungilo	22301	2005	85.7	73.5	99.0
Namibia	Ohangwena	Omundaungilo	22301	2010	67.4	57.2	79.0
Namibia	Ohangwena	Omundaungilo	22301	2015	51.6	42.7	61.1
Namibia	Ohangwena	Ondobe	42162	2000	79.0	69.7	89.8
Namibia	Ohangwena	Ondobe	42162	2005	82.7	72.5	93.8
Namibia	Ohangwena	Ondobe	42162	2010	65.7	57.2	75.2
Namibia	Ohangwena	Ondobe	42162	2015	50.1	42.3	58.5
Namibia	Ohangwena	Ongenga	22303	2000	73.4	65.4	82.4
Namibia	Ohangwena	Ongenga	22303	2005	79.0	70.2	88.0
Namibia	Ohangwena	Ongenga	22303	2010	63.4	55.3	72.1
Namibia	Ohangwena	Ongenga	22303	2015	46.4	39.5	53.9
Namibia	Ohangwena	Oshikango	42163	2000	78.6	69.4	89.0
Namibia	Ohangwena	Oshikango	42163	2005	82.9	72.3	94.0
Namibia	Ohangwena	Oshikango	42163	2010	65.2	56.5	74.9
Namibia	Ohangwena	Oshikango	42163	2015	48.6	40.9	56.6
Namibia	Omaheke	Aminius	22305	2000	60.2	50.2	72.5
Namibia	Omaheke	Aminius	22305	2005	55.2	45.6	66.0
Namibia	Omaheke	Aminius	22305	2010	47.9	39.8	58.2
Namibia	Omaheke	Aminius	22305	2015	39.0	31.6	47.3
Namibia	Omaheke	Epukiro	42164	2000	64.7	52.9	77.8
Namibia	Omaheke	Epukiro	42164	2005	58.7	48.4	70.9
Namibia	Omaheke	Epukiro	42164	2010	50.8	41.3	60.8
Namibia	Omaheke	Epukiro	42164	2015	38.9	31.4	47.4
Namibia	Omaheke	Gobabis	22306	2000	65.0	54.6	77.5
Namibia	Omaheke	Gobabis	22306	2005	57.6	48.4	68.6
Namibia	Omaheke	Gobabis	22306	2010	49.3	41.3	58.8
Namibia	Omaheke	Gobabis	22306	2015	39.6	32.8	48.5
Namibia	Omaheke	Kalahari	22307	2000	67.5	56.3	80.0
Namibia	Omaheke	Kalahari	22307	2005	58.3	49.0	68.3
Namibia	Omaheke	Kalahari	22307	2010	50.1	41.7	59.3
Namibia	Omaheke	Kalahari	22307	2015	40.8	34.1	49.2
Namibia	Omaheke	Otjinene	42165	2000	60.5	49.4	73.3
Namibia	Omaheke	Otjinene	42165	2005	54.9	45.2	66.5
Namibia	Omaheke	Otjinene	42165	2010	47.4	38.3	57.5
Namibia	Omaheke	Otjinene	42165	2015	36.7	29.4	45.4
Namibia	Omaheke	Otjombinde	22309	2000	69.7	58.0	84.5
Namibia	Omaheke	Otjombinde	22309	2005	63.2	52.3	76.2
Namibia	Omaheke	Otjombinde	22309	2010	54.5	45.0	66.3
Namibia	Omaheke	Otjombinde	22309	2015	41.3	34.0	50.5
Namibia	Omaheke	Steinhausen	22310	2000	64.6	54.4	76.3
Namibia	Omaheke	Steinhausen	22310	2005	57.3	48.9	66.9
Namibia	Omaheke	Steinhausen	22310	2010	49.4	41.5	59.1
Namibia	Omaheke	Steinhausen	22310	2015	38.5	32.2	46.2
Namibia	Omusati	Anamulenge	22311	2000	64.5	55.5	74.3
Namibia	Omusati	Anamulenge	22311	2005	68.2	59.3	78.2
Namibia	Omusati	Anamulenge	22311	2010	58.7	50.2	68.3
Namibia	Omusati	Anamulenge	22311	2015	40.8	34.1	48.1
Namibia	Omusati	Elim	42166	2000	62.8	54.8	71.4
Namibia	Omusati	Elim	42166	2005	66.6	58.0	75.5
Namibia	Omusati	Elim	42166	2010	56.7	49.3	65.4
Namibia	Omusati	Elim	42166	2015	40.7	34.5	47.8
Namibia	Omusati	Etayi	22313	2000	68.3	60.3	76.3
Namibia	Omusati	Etayi	22313	2005	72.1	63.7	80.7
Namibia	Omusati	Etayi	22313	2010	59.6	51.8	67.7
Namibia	Omusati	Etayi	22313	2015	43.3	36.9	50.5
Namibia	Omusati	Ogongo	22314	2000	62.7	54.4	71.6
Namibia	Omusati	Ogongo	22314	2005	65.5	56.3	74.7
Namibia	Omusati	Ogongo	22314	2010	56.5	48.3	65.9
Namibia	Omusati	Ogongo	22314	2015	40.1	33.9	47.2
Namibia	Omusati	Okahao	42167	2000	59.5	50.9	70.0
Namibia	Omusati	Okahao	42167	2005	60.9	52.3	70.6
Namibia	Omusati	Okahao	42167	2010	52.9	44.9	61.8
Namibia	Omusati	Okahao	42167	2015	37.9	31.8	44.6
Namibia	Omusati	Okalongo	22316	2000	67.1	58.7	76.1
Namibia	Omusati	Okalongo	22316	2005	71.9	62.9	81.4
Namibia	Omusati	Okalongo	22316	2010	60.3	51.7	69.6
Namibia	Omusati	Okalongo	22316	2015	43.0	36.3	50.1
Namibia	Omusati	Onesi	22317	2000	63.5	54.3	74.2
Namibia	Omusati	Onesi	22317	2005	64.4	55.3	74.9
Namibia	Omusati	Onesi	22317	2010	56.6	48.3	67.2
Namibia	Omusati	Onesi	22317	2015	39.9	33.6	47.6
Namibia	Omusati	Oshikuku	22318	2000	63.9	55.7	72.3
Namibia	Omusati	Oshikuku	22318	2005	67.0	58.3	75.7
Namibia	Omusati	Oshikuku	22318	2010	56.9	49.1	65.4
Namibia	Omusati	Oshikuku	22318	2015	40.9	34.7	48.0
Namibia	Omusati	Otamanzi	42168	2000	60.5	52.2	70.3
Namibia	Omusati	Otamanzi	42168	2005	63.1	54.3	72.7

Admin 0	Admin 1	Admin 2	GAUL Code	Year	Under-5 mortality (per 1,000 livebirths)		
					Estimate	Lower bound, 95% UI	Upper bound, 95% UI
Namibia	Omusati	Otamanzi	42168	2010	54.5	46.4	63.9
Namibia	Omusati	Otamanzi	42168	2015	39.4	32.9	46.9
Namibia	Omusati	Outapi	22319	2000	63.8	55.1	73.0
Namibia	Omusati	Outapi	22319	2005	65.8	56.9	75.8
Namibia	Omusati	Outapi	22319	2010	57.3	49.0	66.9
Namibia	Omusati	Outapi	22319	2015	40.0	33.8	47.3
Namibia	Omusati	Ruacana	22320	2000	65.2	56.0	75.8
Namibia	Omusati	Ruacana	22320	2005	63.9	55.4	74.2
Namibia	Omusati	Ruacana	22320	2010	54.8	47.4	64.8
Namibia	Omusati	Ruacana	22320	2015	40.1	33.9	47.6
Namibia	Omusati	Tsandi	22321	2000	60.7	52.3	70.3
Namibia	Omusati	Tsandi	22321	2005	62.8	53.9	72.6
Namibia	Omusati	Tsandi	22321	2010	54.8	46.3	64.2
Namibia	Omusati	Tsandi	22321	2015	39.1	32.7	46.6
Namibia	Oshana	Okaku	22322	2000	67.0	60.0	74.4
Namibia	Oshana	Okaku	22322	2005	71.5	63.9	79.0
Namibia	Oshana	Okaku	22322	2010	59.3	52.1	66.8
Namibia	Oshana	Okaku	22322	2015	44.2	37.4	51.1
Namibia	Oshana	Okatana	22323	2000	63.2	56.1	70.9
Namibia	Oshana	Okatana	22323	2005	67.7	59.9	75.4
Namibia	Oshana	Okatana	22323	2010	56.7	49.9	64.3
Namibia	Oshana	Okatana	22323	2015	41.3	35.1	48.2
Namibia	Oshana	Okatyali	22324	2000	62.8	55.0	71.1
Namibia	Oshana	Okatyali	22324	2005	67.3	58.9	76.0
Namibia	Oshana	Okatyali	22324	2010	56.9	49.5	65.3
Namibia	Oshana	Okatyali	22324	2015	42.2	35.8	49.6
Namibia	Oshana	Ompundja	22325	2000	61.4	53.8	70.0
Namibia	Oshana	Ompundja	22325	2005	65.8	57.9	74.7
Namibia	Oshana	Ompundja	22325	2010	55.9	48.4	64.4
Namibia	Oshana	Ompundja	22325	2015	40.9	34.4	48.1
Namibia	Oshana	Ondangwa	22326	2000	62.8	56.1	69.7
Namibia	Oshana	Ondangwa	22326	2005	66.8	59.3	74.6
Namibia	Oshana	Ondangwa	22326	2010	55.6	48.7	62.9
Namibia	Oshana	Ondangwa	22326	2015	41.0	34.7	47.7
Namibia	Oshana	Ongwediva	22327	2000	62.8	56.0	69.8
Namibia	Oshana	Ongwediva	22327	2005	66.9	60.0	74.3
Namibia	Oshana	Ongwediva	22327	2010	55.4	48.6	62.6
Namibia	Oshana	Ongwediva	22327	2015	40.9	34.7	47.5
Namibia	Oshana	Oshakati East	22328	2000	61.6	54.7	68.9
Namibia	Oshana	Oshakati East	22328	2005	65.2	58.0	73.0
Namibia	Oshana	Oshakati East	22328	2010	54.4	47.6	61.7
Namibia	Oshana	Oshakati East	22328	2015	40.0	33.9	46.7
Namibia	Oshana	Oshakati West	22329	2000	60.1	53.2	67.7
Namibia	Oshana	Oshakati West	22329	2005	63.6	56.2	71.5
Namibia	Oshana	Oshakati West	22329	2010	52.9	46.3	60.1
Namibia	Oshana	Oshakati West	22329	2015	38.9	32.8	45.5
Namibia	Oshana	Uukwiyu	22330	2000	63.3	56.0	71.0
Namibia	Oshana	Uukwiyu	22330	2005	67.8	59.7	76.2
Namibia	Oshana	Uukwiyu	22330	2010	57.5	49.8	65.8
Namibia	Oshana	Uukwiyu	22330	2015	42.6	35.9	49.8
Namibia	Oshana	Uuvudhiya	22331	2000	58.1	49.7	68.4
Namibia	Oshana	Uuvudhiya	22331	2005	60.4	51.6	70.8
Namibia	Oshana	Uuvudhiya	22331	2010	52.0	43.6	61.8
Namibia	Oshana	Uuvudhiya	22331	2015	37.8	31.2	45.2
Namibia	Oshikoto	Engodi	22332	2000	79.1	67.4	91.2
Namibia	Oshikoto	Engodi	22332	2005	77.8	66.4	89.7
Namibia	Oshikoto	Engodi	22332	2010	62.5	53.2	72.4
Namibia	Oshikoto	Engodi	22332	2015	46.8	38.9	55.2
Namibia	Oshikoto	Guinas	22333	2000	69.4	58.7	81.7
Namibia	Oshikoto	Guinas	22333	2005	68.9	58.1	81.5
Namibia	Oshikoto	Guinas	22333	2010	56.8	47.7	67.6
Namibia	Oshikoto	Guinas	22333	2015	39.9	32.9	47.5
Namibia	Oshikoto	Okankolo	22334	2000	80.7	69.8	92.3
Namibia	Oshikoto	Okankolo	22334	2005	79.7	68.8	91.1
Namibia	Oshikoto	Okankolo	22334	2010	63.6	54.5	73.9
Namibia	Oshikoto	Okankolo	22334	2015	48.2	40.0	57.3
Namibia	Oshikoto	Olukonda	22335	2000	66.0	58.4	73.7
Namibia	Oshikoto	Olukonda	22335	2005	68.6	60.7	77.1
Namibia	Oshikoto	Olukonda	22335	2010	57.6	50.2	65.6
Namibia	Oshikoto	Olukonda	22335	2015	41.9	35.7	48.9
Namibia	Oshikoto	Omuntele	22336	2000	69.5	60.2	79.9
Namibia	Oshikoto	Omuntele	22336	2005	71.3	62.0	82.2
Namibia	Oshikoto	Omuntele	22336	2010	59.9	51.3	69.7
Namibia	Oshikoto	Omuntele	22336	2015	45.0	37.9	53.2
Namibia	Oshikoto	Omuthiyagwipundi	22337	2000	67.5	57.3	78.0
Namibia	Oshikoto	Omuthiyagwipundi	22337	2005	68.8	58.8	79.4
Namibia	Oshikoto	Omuthiyagwipundi	22337	2010	57.8	49.1	67.2
Namibia	Oshikoto	Omuthiyagwipundi	22337	2015	41.6	35.1	49.3
Namibia	Oshikoto	Onayena	22338	2000	69.0	61.0	77.5
Namibia	Oshikoto	Onayena	22338	2005	71.2	62.7	80.5
Namibia	Oshikoto	Onayena	22338	2010	59.7	51.6	68.2
Namibia	Oshikoto	Onayena	22338	2015	44.1	36.9	51.6
Namibia	Oshikoto	Oniipa	22339	2000	68.4	61.2	75.8
Namibia	Oshikoto	Oniipa	22339	2005	71.1	63.2	78.9
Namibia	Oshikoto	Oniipa	22339	2010	59.3	51.8	67.0

Admin 0	Admin 1	Admin 2	GAUL Code	Year	Under-5 mortality (per 1,000 livebirths)		
					Estimate	Lower bound, 95% UI	Upper bound, 95% UI
Namibia	Oshikoto	Oniipa	22339	2015	43.7	37.0	50.6
Namibia	Oshikoto	Onyaanya	22340	2000	72.3	62.7	82.2
Namibia	Oshikoto	Onyaanya	22340	2005	73.4	63.6	84.4
Namibia	Oshikoto	Onyaanya	22340	2010	61.1	52.3	70.8
Namibia	Oshikoto	Onyaanya	22340	2015	45.5	38.1	53.6
Namibia	Oshikoto	Tsumeb	22341	2000	58.8	48.6	70.2
Namibia	Oshikoto	Tsumeb	22341	2005	61.3	51.1	74.5
Namibia	Oshikoto	Tsumeb	22341	2010	51.8	42.5	62.6
Namibia	Oshikoto	Tsumeb	22341	2015	35.7	28.7	43.8
Namibia	Otjozondjupa	Grootfontein	22342	2000	60.1	50.0	71.2
Namibia	Otjozondjupa	Grootfontein	22342	2005	59.6	49.9	71.0
Namibia	Otjozondjupa	Grootfontein	22342	2010	49.1	40.8	58.6
Namibia	Otjozondjupa	Grootfontein	22342	2015	36.2	29.9	43.5
Namibia	Otjozondjupa	Okahandja	22343	2000	54.1	45.3	64.9
Namibia	Otjozondjupa	Okahandja	22343	2005	54.0	45.1	65.2
Namibia	Otjozondjupa	Okahandja	22343	2010	46.5	38.1	55.9
Namibia	Otjozondjupa	Okahandja	22343	2015	34.0	27.5	41.2
Namibia	Otjozondjupa	Okakarara	22344	2000	57.4	48.4	68.4
Namibia	Otjozondjupa	Okakarara	22344	2005	55.9	46.6	66.0
Namibia	Otjozondjupa	Okakarara	22344	2010	47.4	39.4	57.1
Namibia	Otjozondjupa	Okakarara	22344	2015	35.2	29.2	42.5
Namibia	Otjozondjupa	Omatako	22345	2000	56.7	48.4	65.9
Namibia	Otjozondjupa	Omatako	22345	2005	55.3	47.5	64.3
Namibia	Otjozondjupa	Omatako	22345	2010	47.8	41.2	56.7
Namibia	Otjozondjupa	Omatako	22345	2015	34.8	29.3	41.5
Namibia	Otjozondjupa	Otavi	22346	2000	55.8	46.4	66.7
Namibia	Otjozondjupa	Otavi	22346	2005	57.7	48.3	68.7
Namibia	Otjozondjupa	Otavi	22346	2010	49.2	41.3	58.9
Namibia	Otjozondjupa	Otavi	22346	2015	34.8	28.8	42.1
Namibia	Otjozondjupa	Otjiwarongo	22347	2000	50.3	41.7	60.6
Namibia	Otjozondjupa	Otjiwarongo	22347	2005	52.3	43.3	63.1
Namibia	Otjozondjupa	Otjiwarongo	22347	2010	45.9	37.8	55.9
Namibia	Otjozondjupa	Otjiwarongo	22347	2015	33.5	27.2	41.1
Namibia	Otjozondjupa	Tsumkwe	22348	2000	65.0	53.5	78.0
Namibia	Otjozondjupa	Tsumkwe	22348	2005	62.7	51.6	74.9
Namibia	Otjozondjupa	Tsumkwe	22348	2010	52.0	42.4	62.6
Namibia	Otjozondjupa	Tsumkwe	22348	2015	39.7	32.2	48.0
Niger	Agadez	Arlit	22551	2000	92.0	77.6	109.5
Niger	Agadez	Arlit	22551	2005	79.2	66.4	93.7
Niger	Agadez	Arlit	22551	2010	70.0	57.8	83.7
Niger	Agadez	Arlit	22551	2015	63.2	52.0	76.2
Niger	Agadez	Bilma	22552	2000	99.1	80.5	122.3
Niger	Agadez	Bilma	22552	2005	88.6	72.0	109.7
Niger	Agadez	Bilma	22552	2010	84.7	69.2	103.5
Niger	Agadez	Bilma	22552	2015	69.5	56.0	85.9
Niger	Agadez	Tchighozerine	22553	2000	97.4	86.0	109.2
Niger	Agadez	Tchighozerine	22553	2005	83.9	74.1	94.1
Niger	Agadez	Tchighozerine	22553	2010	75.1	65.8	85.0
Niger	Agadez	Tchighozerine	22553	2015	67.9	58.4	78.5
Niger	Diffa	Diffa	22554	2000	147.6	131.9	165.9
Niger	Diffa	Diffa	22554	2005	115.3	102.1	131.0
Niger	Diffa	Diffa	22554	2010	107.0	93.3	122.4
Niger	Diffa	Diffa	22554	2015	85.1	72.6	99.5
Niger	Diffa	Maine-soroa	22555	2000	163.6	146.5	183.3
Niger	Diffa	Maine-soroa	22555	2005	126.7	111.4	142.8
Niger	Diffa	Maine-soroa	22555	2010	118.7	103.6	135.1
Niger	Diffa	Maine-soroa	22555	2015	96.0	81.8	111.3
Niger	Diffa	N'guigmi	22556	2000	123.5	108.5	139.7
Niger	Diffa	N'guigmi	22556	2005	97.1	84.3	111.5
Niger	Diffa	N'guigmi	22556	2010	91.6	79.4	106.0
Niger	Diffa	N'guigmi	22556	2015	74.8	63.9	87.4
Niger	Dosso	Birni N'gaoure	22557	2000	229.1	213.1	245.0
Niger	Dosso	Birni N'gaoure	22557	2005	193.8	179.9	209.2
Niger	Dosso	Birni N'gaoure	22557	2010	159.8	146.6	174.6
Niger	Dosso	Birni N'gaoure	22557	2015	139.4	123.4	156.5
Niger	Dosso	Dogondoutchi	22558	2000	222.8	209.2	237.1
Niger	Dosso	Dogondoutchi	22558	2005	192.3	180.9	205.2
Niger	Dosso	Dogondoutchi	22558	2010	156.7	144.8	169.5
Niger	Dosso	Dogondoutchi	22558	2015	131.3	116.9	146.8
Niger	Dosso	Dosso	22559	2000	231.2	216.2	247.0
Niger	Dosso	Dosso	22559	2005	197.7	184.9	211.2
Niger	Dosso	Dosso	22559	2010	162.4	149.1	175.9
Niger	Dosso	Dosso	22559	2015	141.8	126.2	158.9
Niger	Dosso	Gaya	22560	2000	222.6	207.7	239.0
Niger	Dosso	Gaya	22560	2005	190.1	177.2	204.9
Niger	Dosso	Gaya	22560	2010	163.0	151.1	177.7
Niger	Dosso	Gaya	22560	2015	136.0	120.7	152.0
Niger	Dosso	Loga	22561	2000	225.2	209.2	243.1
Niger	Dosso	Loga	22561	2005	194.5	179.8	209.9
Niger	Dosso	Loga	22561	2010	158.0	144.6	172.3
Niger	Dosso	Loga	22561	2015	136.9	120.4	154.1
Niger	Maradi	Aguie	22562	2000	274.3	257.4	290.5
Niger	Maradi	Aguie	22562	2005	208.8	195.4	222.9
Niger	Maradi	Aguie	22562	2010	157.2	146.0	169.6
Niger	Maradi	Aguie	22562	2015	127.2	114.0	142.7

Admin 0	Admin 1	Admin 2	GAUL Code	Year	Under-5 mortality (per 1,000 livebirths)		
					Estimate	Lower bound, 95% UI	Upper bound, 95% UI
Niger	Maradi	Dakoro	22563	2000	246.6	229.7	266.7
Niger	Maradi	Dakoro	22563	2005	181.6	167.7	196.4
Niger	Maradi	Dakoro	22563	2010	127.9	116.0	139.9
Niger	Maradi	Dakoro	22563	2015	108.2	95.4	121.6
Niger	Maradi	Guidan Roumji	22564	2000	262.4	246.9	278.9
Niger	Maradi	Guidan Roumji	22564	2005	194.8	183.4	206.8
Niger	Maradi	Guidan Roumji	22564	2010	137.9	127.6	148.9
Niger	Maradi	Guidan Roumji	22564	2015	117.4	105.2	131.2
Niger	Maradi	Madarounfa	22565	2000	269.3	252.8	287.7
Niger	Maradi	Madarounfa	22565	2005	201.5	189.2	214.2
Niger	Maradi	Madarounfa	22565	2010	146.8	135.5	157.8
Niger	Maradi	Madarounfa	22565	2015	120.8	108.1	135.2
Niger	Maradi	Mayahi	22566	2000	253.9	236.8	271.0
Niger	Maradi	Mayahi	22566	2005	189.3	175.3	202.5
Niger	Maradi	Mayahi	22566	2010	133.0	122.1	144.5
Niger	Maradi	Mayahi	22566	2015	113.0	99.4	127.7
Niger	Maradi	Tessaoua	22567	2000	255.8	239.6	272.7
Niger	Maradi	Tessaoua	22567	2005	194.4	182.2	208.3
Niger	Maradi	Tessaoua	22567	2010	142.0	131.7	153.4
Niger	Maradi	Tessaoua	22567	2015	119.0	106.1	133.3
Niger	Niamey	Commune 1	22568	2000	122.6	112.6	132.2
Niger	Niamey	Commune 1	22568	2005	106.7	98.9	114.9
Niger	Niamey	Commune 1	22568	2010	94.2	85.1	103.0
Niger	Niamey	Commune 1	22568	2015	77.1	67.8	86.7
Niger	Niamey	Commune 2	22569	2000	128.5	118.7	138.3
Niger	Niamey	Commune 2	22569	2005	111.0	103.0	119.3
Niger	Niamey	Commune 2	22569	2010	97.7	88.6	107.0
Niger	Niamey	Commune 2	22569	2015	80.4	71.0	90.6
Niger	Niamey	Commune 3	22570	2000	122.5	112.9	132.0
Niger	Niamey	Commune 3	22570	2005	108.6	100.6	117.0
Niger	Niamey	Commune 3	22570	2010	95.4	86.4	104.6
Niger	Niamey	Commune 3	22570	2015	77.2	67.8	87.0
Niger	Tahoua	Birni N'konni	22571	2000	243.6	227.9	260.9
Niger	Tahoua	Birni N'konni	22571	2005	197.1	183.5	211.7
Niger	Tahoua	Birni N'konni	22571	2010	141.3	130.0	153.7
Niger	Tahoua	Birni N'konni	22571	2015	123.9	109.7	139.4
Niger	Tahoua	Bouza	22572	2000	224.5	208.1	241.7
Niger	Tahoua	Bouza	22572	2005	171.0	157.7	184.9
Niger	Tahoua	Bouza	22572	2010	119.6	108.5	132.1
Niger	Tahoua	Bouza	22572	2015	110.2	97.0	124.9
Niger	Tahoua	Illela	22573	2000	227.1	210.8	243.0
Niger	Tahoua	Illela	22573	2005	178.5	165.0	192.1
Niger	Tahoua	Illela	22573	2010	128.8	117.7	141.0
Niger	Tahoua	Illela	22573	2015	116.8	102.9	131.2
Niger	Tahoua	Keita	22574	2000	219.9	201.9	237.5
Niger	Tahoua	Keita	22574	2005	164.9	151.4	178.6
Niger	Tahoua	Keita	22574	2010	116.0	104.6	128.8
Niger	Tahoua	Keita	22574	2015	107.4	93.9	122.2
Niger	Tahoua	Madaoua	22575	2000	238.7	221.9	255.7
Niger	Tahoua	Madaoua	22575	2005	187.3	173.8	201.5
Niger	Tahoua	Madaoua	22575	2010	131.1	119.5	143.9
Niger	Tahoua	Madaoua	22575	2015	118.3	105.4	133.3
Niger	Tahoua	Tahoua	22576	2000	219.0	201.1	237.9
Niger	Tahoua	Tahoua	22576	2005	168.7	153.7	184.5
Niger	Tahoua	Tahoua	22576	2010	121.2	108.2	135.8
Niger	Tahoua	Tahoua	22576	2015	110.7	96.7	126.9
Niger	Tahoua	Tchin Tabaradene	22577	2000	199.9	179.1	222.5
Niger	Tahoua	Tchin Tabaradene	22577	2005	156.4	140.8	174.4
Niger	Tahoua	Tchin Tabaradene	22577	2010	113.9	101.0	127.9
Niger	Tahoua	Tchin Tabaradene	22577	2015	101.2	88.3	115.8
Niger	Tillaberi	Filingue	22578	2000	200.5	185.0	217.0
Niger	Tillaberi	Filingue	22578	2005	179.0	165.4	194.3
Niger	Tillaberi	Filingue	22578	2010	162.4	148.3	178.5
Niger	Tillaberi	Filingue	22578	2015	122.8	108.2	138.0
Niger	Tillaberi	Kollo	22579	2000	208.8	196.3	221.8
Niger	Tillaberi	Kollo	22579	2005	174.2	162.8	185.8
Niger	Tillaberi	Kollo	22579	2010	155.6	143.5	168.9
Niger	Tillaberi	Kollo	22579	2015	117.6	104.7	130.6
Niger	Tillaberi	Ouallam	22580	2000	201.8	184.0	221.2
Niger	Tillaberi	Ouallam	22580	2005	179.3	163.2	196.4
Niger	Tillaberi	Ouallam	22580	2010	163.4	148.0	181.7
Niger	Tillaberi	Ouallam	22580	2015	121.1	105.7	136.7
Niger	Tillaberi	Say	22581	2000	229.3	213.0	247.3
Niger	Tillaberi	Say	22581	2005	189.6	175.9	203.8
Niger	Tillaberi	Say	22581	2010	164.2	150.9	177.8
Niger	Tillaberi	Say	22581	2015	127.9	114.2	142.4
Niger	Tillaberi	Tera	22582	2000	225.5	209.9	240.9
Niger	Tillaberi	Tera	22582	2005	194.6	180.0	207.8
Niger	Tillaberi	Tera	22582	2010	174.5	160.2	189.6
Niger	Tillaberi	Tera	22582	2015	131.8	117.3	148.2
Niger	Tillaberi	Tillaberi	22583	2000	202.6	185.2	220.1
Niger	Tillaberi	Tillaberi	22583	2005	179.5	164.4	195.8
Niger	Tillaberi	Tillaberi	22583	2010	163.5	147.9	181.4
Niger	Tillaberi	Tillaberi	22583	2015	121.1	106.0	139.1
Niger	Zinder	Goure	22584	2000	224.7	202.8	248.4

Admin 0	Admin 1	Admin 2	GAUL Code	Year	Under-5 mortality (per 1,000 livebirths)		
					Estimate	Lower bound, 95% UI	Upper bound, 95% UI
Niger	Zinder	Goure	22584	2005	176.7	159.0	196.4
Niger	Zinder	Goure	22584	2010	143.6	127.5	162.0
Niger	Zinder	Goure	22584	2015	108.6	94.7	124.8
Niger	Zinder	Magaria	22585	2000	262.7	246.5	279.6
Niger	Zinder	Magaria	22585	2005	202.7	190.6	216.3
Niger	Zinder	Magaria	22585	2010	161.0	149.1	174.2
Niger	Zinder	Magaria	22585	2015	127.1	113.8	141.2
Niger	Zinder	Matamey	22586	2000	257.4	239.0	277.4
Niger	Zinder	Matamey	22586	2005	197.9	183.5	213.7
Niger	Zinder	Matamey	22586	2010	155.0	141.7	169.6
Niger	Zinder	Matamey	22586	2015	124.8	110.4	140.1
Niger	Zinder	Miria	22587	2000	243.9	227.2	261.1
Niger	Zinder	Miria	22587	2005	189.7	176.6	204.2
Niger	Zinder	Miria	22587	2010	144.9	132.0	157.4
Niger	Zinder	Miria	22587	2015	112.7	99.2	127.5
Niger	Zinder	Tanout	22588	2000	226.5	205.0	249.5
Niger	Zinder	Tanout	22588	2005	178.5	160.6	197.3
Niger	Zinder	Tanout	22588	2010	134.1	119.6	150.6
Niger	Zinder	Tanout	22588	2015	101.7	88.7	117.6
Nigeria	Abia	Aba North	191374	2000	134.0	123.3	145.8
Nigeria	Abia	Aba North	191374	2005	128.5	117.7	138.9
Nigeria	Abia	Aba North	191374	2010	103.6	93.7	113.7
Nigeria	Abia	Aba North	191374	2015	75.4	66.7	85.9
Nigeria	Abia	Aba South	191375	2000	135.4	124.3	147.2
Nigeria	Abia	Aba South	191375	2005	130.3	119.3	141.2
Nigeria	Abia	Aba South	191375	2010	105.1	95.2	115.4
Nigeria	Abia	Aba South	191375	2015	76.6	67.8	87.3
Nigeria	Abia	Arochukwu	22592	2000	143.0	131.4	155.2
Nigeria	Abia	Arochukwu	22592	2005	140.4	129.1	152.0
Nigeria	Abia	Arochukwu	22592	2010	116.1	105.9	128.1
Nigeria	Abia	Arochukwu	22592	2015	87.1	77.1	98.8
Nigeria	Abia	Bende	22593	2000	148.8	137.1	161.5
Nigeria	Abia	Bende	22593	2005	143.9	132.6	155.8
Nigeria	Abia	Bende	22593	2010	119.0	108.7	130.0
Nigeria	Abia	Bende	22593	2015	87.3	77.7	98.8
Nigeria	Abia	Ikwuano	22594	2000	140.9	128.7	152.8
Nigeria	Abia	Ikwuano	22594	2005	139.2	127.5	151.6
Nigeria	Abia	Ikwuano	22594	2010	112.8	102.8	124.1
Nigeria	Abia	Ikwuano	22594	2015	83.5	73.9	95.0
Nigeria	Abia	Isiala Ngwa North	191358	2000	141.3	130.9	152.1
Nigeria	Abia	Isiala Ngwa North	191358	2005	138.2	128.0	148.2
Nigeria	Abia	Isiala Ngwa North	191358	2010	113.7	105.0	123.6
Nigeria	Abia	Isiala Ngwa North	191358	2015	82.0	73.2	92.5
Nigeria	Abia	Isiala Ngwa South	191361	2000	142.2	132.0	152.8
Nigeria	Abia	Isiala Ngwa South	191361	2005	139.2	129.0	148.9
Nigeria	Abia	Isiala Ngwa South	191361	2010	113.7	104.7	123.2
Nigeria	Abia	Isiala Ngwa South	191361	2015	82.8	73.6	92.8
Nigeria	Abia	Isuikwuato	191336	2000	151.6	139.9	164.3
Nigeria	Abia	Isuikwuato	191336	2005	144.6	133.1	157.7
Nigeria	Abia	Isuikwuato	191336	2010	119.8	109.2	131.4
Nigeria	Abia	Isuikwuato	191336	2015	86.9	76.9	98.0
Nigeria	Abia	Oboma Ngwa	191363	2000	136.7	126.2	148.1
Nigeria	Abia	Oboma Ngwa	191363	2005	133.1	122.4	143.4
Nigeria	Abia	Oboma Ngwa	191363	2010	108.2	98.8	118.4
Nigeria	Abia	Oboma Ngwa	191363	2015	79.8	70.4	90.2
Nigeria	Abia	Ohafia	22598	2000	150.9	139.4	163.0
Nigeria	Abia	Ohafia	22598	2005	145.5	134.6	156.7
Nigeria	Abia	Ohafia	22598	2010	121.0	111.0	131.9
Nigeria	Abia	Ohafia	22598	2015	90.4	80.1	102.0
Nigeria	Abia	Osisioma Ngwa	22602	2000	140.3	129.5	151.6
Nigeria	Abia	Osisioma Ngwa	22602	2005	136.6	125.4	147.6
Nigeria	Abia	Osisioma Ngwa	22602	2010	110.9	101.3	121.2
Nigeria	Abia	Osisioma Ngwa	22602	2015	80.7	71.6	91.1
Nigeria	Abia	Ugwunagbo	191380	2000	140.8	129.2	153.4
Nigeria	Abia	Ugwunagbo	191380	2005	138.5	126.9	149.9
Nigeria	Abia	Ugwunagbo	191380	2010	111.4	100.7	122.3
Nigeria	Abia	Ugwunagbo	191380	2015	82.1	72.3	93.7
Nigeria	Abia	Ukwa East	22601	2000	139.4	127.4	152.2
Nigeria	Abia	Ukwa East	22601	2005	136.6	125.5	148.9
Nigeria	Abia	Ukwa East	22601	2010	109.1	99.2	120.2
Nigeria	Abia	Ukwa East	22601	2015	82.3	72.7	93.1
Nigeria	Abia	Ukwa West	22602	2000	140.3	128.6	152.5
Nigeria	Abia	Ukwa West	22602	2005	135.9	124.6	147.4
Nigeria	Abia	Ukwa West	22602	2010	109.8	99.6	120.6
Nigeria	Abia	Ukwa West	22602	2015	81.0	71.4	92.3
Nigeria	Abia	Umu-Nneochi	191331	2000	150.1	139.8	161.6
Nigeria	Abia	Umu-Nneochi	191331	2005	141.7	131.6	152.5
Nigeria	Abia	Umu-Nneochi	191331	2010	119.5	110.1	130.2
Nigeria	Abia	Umu-Nneochi	191331	2015	86.2	76.4	96.4
Nigeria	Abia	Umuahia North	191348	2000	139.1	128.5	150.9
Nigeria	Abia	Umuahia North	191348	2005	133.9	123.8	144.9
Nigeria	Abia	Umuahia North	191348	2010	110.7	101.6	120.7
Nigeria	Abia	Umuahia North	191348	2015	81.5	72.3	92.1
Nigeria	Abia	Umuahia South	191355	2000	140.9	130.0	152.5
Nigeria	Abia	Umuahia South	191355	2005	136.8	126.3	147.6

Admin 0	Admin 1	Admin 2	GAUL Code	Year	Under-5 mortality (per 1,000 livebirths)		
					Estimate	Lower bound, 95% UI	Upper bound, 95% UI
Nigeria	Abia	Umuahia South	191355	2010	113.1	104.0	123.5
Nigeria	Abia	Umuahia South	191355	2015	81.6	72.7	92.4
Nigeria	Abuja	Abaji	22775	2000	126.9	116.3	138.9
Nigeria	Abuja	Abaji	22775	2005	110.1	100.2	120.6
Nigeria	Abuja	Abaji	22775	2010	96.5	87.1	106.8
Nigeria	Abuja	Abaji	22775	2015	82.5	72.0	93.7
Nigeria	Abuja	Abuja Municipal	191147	2000	117.4	106.8	127.6
Nigeria	Abuja	Abuja Municipal	191147	2005	104.2	95.7	113.3
Nigeria	Abuja	Abuja Municipal	191147	2010	91.0	83.1	100.7
Nigeria	Abuja	Abuja Municipal	191147	2015	73.4	64.5	83.3
Nigeria	Abuja	Bwari	191137	2000	123.4	112.6	134.2
Nigeria	Abuja	Bwari	191137	2005	108.5	99.5	118.1
Nigeria	Abuja	Bwari	191137	2010	94.4	86.0	104.9
Nigeria	Abuja	Bwari	191137	2015	77.0	68.0	87.7
Nigeria	Abuja	Gwagwalada	191142	2000	123.2	111.9	135.1
Nigeria	Abuja	Gwagwalada	191142	2005	108.1	97.9	118.5
Nigeria	Abuja	Gwagwalada	191142	2010	95.2	85.5	105.3
Nigeria	Abuja	Gwagwalada	191142	2015	79.6	69.2	90.9
Nigeria	Abuja	Kuje	22778	2000	124.9	113.0	137.7
Nigeria	Abuja	Kuje	22778	2005	109.4	99.2	120.0
Nigeria	Abuja	Kuje	22778	2010	94.1	84.0	105.2
Nigeria	Abuja	Kuje	22778	2015	77.1	67.3	87.6
Nigeria	Abuja	Kwali	191156	2000	124.7	113.0	137.4
Nigeria	Abuja	Kwali	191156	2005	109.0	98.1	120.8
Nigeria	Abuja	Kwali	191156	2010	94.5	84.1	105.1
Nigeria	Abuja	Kwali	191156	2015	78.1	67.5	89.2
Nigeria	Adamawa	Demsa	191123	2000	221.8	203.8	241.8
Nigeria	Adamawa	Demsa	191123	2005	195.8	179.8	213.8
Nigeria	Adamawa	Demsa	191123	2010	159.4	144.1	176.8
Nigeria	Adamawa	Demsa	191123	2015	132.7	118.3	148.9
Nigeria	Adamawa	Fufore	22604	2000	227.3	210.6	244.7
Nigeria	Adamawa	Fufore	22604	2005	199.9	184.4	215.9
Nigeria	Adamawa	Fufore	22604	2010	156.3	142.2	171.0
Nigeria	Adamawa	Fufore	22604	2015	138.7	123.7	153.8
Nigeria	Adamawa	Ganye	191163	2000	209.7	186.9	233.2
Nigeria	Adamawa	Ganye	191163	2005	180.9	161.1	200.9
Nigeria	Adamawa	Ganye	191163	2010	144.7	128.4	162.2
Nigeria	Adamawa	Ganye	191163	2015	132.2	114.8	151.1
Nigeria	Adamawa	Girie	22606	2000	241.0	219.9	264.8
Nigeria	Adamawa	Girie	22606	2005	210.2	191.9	230.1
Nigeria	Adamawa	Girie	22606	2010	171.4	154.1	189.9
Nigeria	Adamawa	Girie	22606	2015	141.9	125.3	162.1
Nigeria	Adamawa	Gombi	191126	2000	231.2	211.3	253.2
Nigeria	Adamawa	Gombi	191126	2005	205.0	186.7	225.2
Nigeria	Adamawa	Gombi	191126	2010	162.3	146.2	180.2
Nigeria	Adamawa	Gombi	191126	2015	139.2	122.4	157.2
Nigeria	Adamawa	Guyuk	22607	2000	240.2	220.0	260.7
Nigeria	Adamawa	Guyuk	22607	2005	215.2	197.2	234.6
Nigeria	Adamawa	Guyuk	22607	2010	175.0	157.4	194.0
Nigeria	Adamawa	Guyuk	22607	2015	141.7	124.8	158.6
Nigeria	Adamawa	Hong	22608	2000	224.7	206.2	244.5
Nigeria	Adamawa	Hong	22608	2005	199.0	183.0	215.2
Nigeria	Adamawa	Hong	22608	2010	161.8	147.5	177.5
Nigeria	Adamawa	Hong	22608	2015	139.8	123.5	157.5
Nigeria	Adamawa	Jada	22609	2000	219.6	198.9	240.9
Nigeria	Adamawa	Jada	22609	2005	189.8	172.1	208.6
Nigeria	Adamawa	Jada	22609	2010	151.1	134.3	168.9
Nigeria	Adamawa	Jada	22609	2015	135.9	118.8	155.8
Nigeria	Adamawa	Lamurde	191121	2000	233.1	215.4	254.2
Nigeria	Adamawa	Lamurde	191121	2005	205.0	190.1	221.4
Nigeria	Adamawa	Lamurde	191121	2010	168.9	154.8	184.2
Nigeria	Adamawa	Lamurde	191121	2015	138.0	123.1	153.9
Nigeria	Adamawa	Madagali	22610	2000	193.8	176.5	211.7
Nigeria	Adamawa	Madagali	22610	2005	176.1	161.1	192.0
Nigeria	Adamawa	Madagali	22610	2010	148.9	133.8	165.1
Nigeria	Adamawa	Madagali	22610	2015	132.6	117.0	150.1
Nigeria	Adamawa	Maiha	22611	2000	232.3	213.7	251.7
Nigeria	Adamawa	Maiha	22611	2005	211.1	195.5	229.1
Nigeria	Adamawa	Maiha	22611	2010	167.9	153.1	183.7
Nigeria	Adamawa	Maiha	22611	2015	153.2	135.0	173.5
Nigeria	Adamawa	Mayo-Belwa	22612	2000	226.9	206.5	248.1
Nigeria	Adamawa	Mayo-Belwa	22612	2005	195.3	176.9	214.7
Nigeria	Adamawa	Mayo-Belwa	22612	2010	156.5	140.4	173.9
Nigeria	Adamawa	Mayo-Belwa	22612	2015	139.0	123.0	157.1
Nigeria	Adamawa	Michika	22613	2000	185.1	168.0	203.3
Nigeria	Adamawa	Michika	22613	2005	169.1	153.1	186.5
Nigeria	Adamawa	Michika	22613	2010	140.0	126.3	156.0
Nigeria	Adamawa	Michika	22613	2015	120.9	106.5	137.2
Nigeria	Adamawa	Mubi North	191101	2000	205.6	188.6	223.1
Nigeria	Adamawa	Mubi North	191101	2005	189.0	174.7	205.2
Nigeria	Adamawa	Mubi North	191101	2010	152.9	139.7	166.7
Nigeria	Adamawa	Mubi North	191101	2015	135.3	120.2	151.8
Nigeria	Adamawa	Mubi South	191106	2000	219.2	202.3	235.9
Nigeria	Adamawa	Mubi South	191106	2005	201.0	186.5	216.6
Nigeria	Adamawa	Mubi South	191106	2010	161.0	147.0	175.5

Admin 0	Admin 1	Admin 2	GAUL Code	Year	Under-5 mortality (per 1,000 livebirths)		
					Estimate	Lower bound, 95% UI	Upper bound, 95% UI
Nigeria	Adamawa	Mubi South	191106	2015	146.9	129.8	165.3
Nigeria	Adamawa	Numan	191125	2000	235.3	217.8	256.7
Nigeria	Adamawa	Numan	191125	2005	206.4	190.3	222.1
Nigeria	Adamawa	Numan	191125	2010	168.1	152.7	184.6
Nigeria	Adamawa	Numan	191125	2015	138.2	123.0	155.0
Nigeria	Adamawa	Shelleng	22616	2000	248.1	226.7	270.8
Nigeria	Adamawa	Shelleng	22616	2005	219.3	201.0	238.9
Nigeria	Adamawa	Shelleng	22616	2010	178.9	161.6	197.7
Nigeria	Adamawa	Shelleng	22616	2015	147.5	130.2	166.8
Nigeria	Adamawa	Song	191111	2000	242.5	225.1	261.5
Nigeria	Adamawa	Song	191111	2005	215.2	198.9	233.3
Nigeria	Adamawa	Song	191111	2010	172.0	157.3	189.1
Nigeria	Adamawa	Song	191111	2015	147.7	131.4	166.2
Nigeria	Adamawa	Teungo	191174	2000	221.7	197.7	249.4
Nigeria	Adamawa	Teungo	191174	2005	191.5	171.0	214.9
Nigeria	Adamawa	Teungo	191174	2010	154.5	135.6	175.2
Nigeria	Adamawa	Teungo	191174	2015	144.6	124.4	165.8
Nigeria	Adamawa	Yola North	191139	2000	199.7	182.2	217.7
Nigeria	Adamawa	Yola North	191139	2005	173.6	159.4	189.9
Nigeria	Adamawa	Yola North	191139	2010	143.5	129.9	159.3
Nigeria	Adamawa	Yola North	191139	2015	118.2	104.4	133.6
Nigeria	Adamawa	Yola South	191141	2000	207.0	189.2	226.0
Nigeria	Adamawa	Yola South	191141	2005	180.0	164.3	197.2
Nigeria	Adamawa	Yola South	191141	2010	146.2	132.0	162.2
Nigeria	Adamawa	Yola South	191141	2015	123.7	109.7	139.5
Nigeria	Akwa Ibom	Abak	22619	2000	134.9	123.9	147.9
Nigeria	Akwa Ibom	Abak	22619	2005	133.7	123.1	144.9
Nigeria	Akwa Ibom	Abak	22619	2010	107.5	98.1	118.0
Nigeria	Akwa Ibom	Abak	22619	2015	83.5	73.8	94.3
Nigeria	Akwa Ibom	Eastern Obolo	191403	2000	132.6	118.2	148.0
Nigeria	Akwa Ibom	Eastern Obolo	191403	2005	127.9	114.2	142.5
Nigeria	Akwa Ibom	Eastern Obolo	191403	2010	102.6	90.7	116.1
Nigeria	Akwa Ibom	Eastern Obolo	191403	2015	81.8	70.8	94.0
Nigeria	Akwa Ibom	Eket	22620	2000	127.6	115.0	141.6
Nigeria	Akwa Ibom	Eket	22620	2005	126.6	113.8	140.3
Nigeria	Akwa Ibom	Eket	22620	2010	103.8	92.2	116.5
Nigeria	Akwa Ibom	Eket	22620	2015	84.3	72.4	96.0
Nigeria	Akwa Ibom	Esit Eket	191397	2000	130.7	116.6	145.4
Nigeria	Akwa Ibom	Esit Eket	191397	2005	129.7	115.2	145.3
Nigeria	Akwa Ibom	Esit Eket	191397	2010	108.4	95.2	123.0
Nigeria	Akwa Ibom	Esit Eket	191397	2015	88.7	76.4	102.4
Nigeria	Akwa Ibom	Essien Udim	22622	2000	138.0	126.6	150.4
Nigeria	Akwa Ibom	Essien Udim	22622	2005	136.8	126.5	147.6
Nigeria	Akwa Ibom	Essien Udim	22622	2010	110.1	101.0	120.5
Nigeria	Akwa Ibom	Essien Udim	22622	2015	82.9	73.3	93.5
Nigeria	Akwa Ibom	Etim Ekpo	191381	2000	139.8	127.8	152.5
Nigeria	Akwa Ibom	Etim Ekpo	191381	2005	137.8	127.3	149.0
Nigeria	Akwa Ibom	Etim Ekpo	191381	2010	109.7	99.9	120.4
Nigeria	Akwa Ibom	Etim Ekpo	191381	2015	84.0	74.3	94.7
Nigeria	Akwa Ibom	Etinan	22624	2000	133.8	122.0	146.0
Nigeria	Akwa Ibom	Etinan	22624	2005	131.9	120.4	144.1
Nigeria	Akwa Ibom	Etinan	22624	2010	107.4	96.9	118.0
Nigeria	Akwa Ibom	Etinan	22624	2015	85.8	75.4	96.9
Nigeria	Akwa Ibom	Ibeno	191402	2000	126.3	112.5	140.8
Nigeria	Akwa Ibom	Ibeno	191402	2005	125.2	111.3	139.9
Nigeria	Akwa Ibom	Ibeno	191402	2010	104.6	92.2	119.2
Nigeria	Akwa Ibom	Ibeno	191402	2015	83.0	71.4	95.7
Nigeria	Akwa Ibom	Ibesikpo Asutan	191382	2000	133.4	122.0	145.3
Nigeria	Akwa Ibom	Ibesikpo Asutan	191382	2005	131.9	120.8	143.6
Nigeria	Akwa Ibom	Ibesikpo Asutan	191382	2010	108.8	98.4	120.1
Nigeria	Akwa Ibom	Ibesikpo Asutan	191382	2015	88.2	77.7	99.2
Nigeria	Akwa Ibom	Ibiono Ibom	191360	2000	135.9	124.7	148.7
Nigeria	Akwa Ibom	Ibiono Ibom	191360	2005	134.3	123.7	146.1
Nigeria	Akwa Ibom	Ibiono Ibom	191360	2010	109.8	99.8	121.7
Nigeria	Akwa Ibom	Ibiono Ibom	191360	2015	85.5	75.8	96.9
Nigeria	Akwa Ibom	Ika	191379	2000	141.8	129.0	154.3
Nigeria	Akwa Ibom	Ika	191379	2005	139.1	128.1	150.6
Nigeria	Akwa Ibom	Ika	191379	2010	110.9	100.6	122.0
Nigeria	Akwa Ibom	Ika	191379	2015	83.8	74.2	95.1
Nigeria	Akwa Ibom	Ikono	22625	2000	137.7	126.2	149.9
Nigeria	Akwa Ibom	Ikono	22625	2005	136.2	125.3	148.3
Nigeria	Akwa Ibom	Ikono	22625	2010	110.5	100.7	121.9
Nigeria	Akwa Ibom	Ikono	22625	2015	84.8	75.3	95.8
Nigeria	Akwa Ibom	Ikot Abasi	191396	2000	133.1	119.6	147.6
Nigeria	Akwa Ibom	Ikot Abasi	191396	2005	129.1	116.1	143.4
Nigeria	Akwa Ibom	Ikot Abasi	191396	2010	103.1	91.8	115.1
Nigeria	Akwa Ibom	Ikot Abasi	191396	2015	82.3	71.2	94.4
Nigeria	Akwa Ibom	Ikot Ekpene	191367	2000	134.2	122.3	146.7
Nigeria	Akwa Ibom	Ikot Ekpene	191367	2005	131.7	120.6	143.3
Nigeria	Akwa Ibom	Ikot Ekpene	191367	2010	105.6	96.3	116.7
Nigeria	Akwa Ibom	Ikot Ekpene	191367	2015	80.6	70.7	91.5
Nigeria	Akwa Ibom	Ini	22628	2000	144.4	132.4	155.7
Nigeria	Akwa Ibom	Ini	22628	2005	141.8	131.2	153.5
Nigeria	Akwa Ibom	Ini	22628	2010	114.5	104.8	125.4
Nigeria	Akwa Ibom	Ini	22628	2015	86.9	77.0	98.1

Admin 0	Admin 1	Admin 2	GAUL Code	Year	Under-5 mortality (per 1,000 livebirths)		
					Estimate	Lower bound, 95% UI	Upper bound, 95% UI
Nigeria	Akwa Ibom	Itu	191366	2000	134.0	122.3	146.5
Nigeria	Akwa Ibom	Itu	191366	2005	132.1	121.0	144.4
Nigeria	Akwa Ibom	Itu	191366	2010	109.0	98.7	121.2
Nigeria	Akwa Ibom	Itu	191366	2015	84.1	74.7	95.4
Nigeria	Akwa Ibom	Mbo	22630	2000	133.5	118.8	150.1
Nigeria	Akwa Ibom	Mbo	22630	2005	132.0	116.8	148.5
Nigeria	Akwa Ibom	Mbo	22630	2010	111.7	97.7	127.0
Nigeria	Akwa Ibom	Mbo	22630	2015	91.4	78.2	105.6
Nigeria	Akwa Ibom	Mkpat Enin	22631	2000	134.2	121.6	148.0
Nigeria	Akwa Ibom	Mkpat Enin	22631	2005	131.4	119.4	144.2
Nigeria	Akwa Ibom	Mkpat Enin	22631	2010	106.6	95.8	118.8
Nigeria	Akwa Ibom	Mkpat Enin	22631	2015	85.2	74.2	96.8
Nigeria	Akwa Ibom	Nsit Atai	191385	2000	135.2	122.1	147.9
Nigeria	Akwa Ibom	Nsit Atai	191385	2005	134.0	121.5	147.0
Nigeria	Akwa Ibom	Nsit Atai	191385	2010	111.9	100.4	124.9
Nigeria	Akwa Ibom	Nsit Atai	191385	2015	91.9	80.3	103.7
Nigeria	Akwa Ibom	Nsit Ibom	22632	2000	134.8	123.0	147.1
Nigeria	Akwa Ibom	Nsit Ibom	22632	2005	133.0	121.6	144.6
Nigeria	Akwa Ibom	Nsit Ibom	22632	2010	108.9	98.6	119.6
Nigeria	Akwa Ibom	Nsit Ibom	22632	2015	87.7	77.7	98.9
Nigeria	Akwa Ibom	Nsit Ubium	22633	2000	133.0	120.3	146.3
Nigeria	Akwa Ibom	Nsit Ubium	22633	2005	131.8	119.3	144.6
Nigeria	Akwa Ibom	Nsit Ubium	22633	2010	108.5	97.0	120.9
Nigeria	Akwa Ibom	Nsit Ubium	22633	2015	89.3	77.4	101.2
Nigeria	Akwa Ibom	Obot Akara	191362	2000	140.8	128.8	152.7
Nigeria	Akwa Ibom	Obot Akara	191362	2005	138.9	127.3	149.9
Nigeria	Akwa Ibom	Obot Akara	191362	2010	111.5	101.6	122.5
Nigeria	Akwa Ibom	Obot Akara	191362	2015	83.7	73.8	94.9
Nigeria	Akwa Ibom	Okobo	22634	2000	134.4	121.4	147.8
Nigeria	Akwa Ibom	Okobo	22634	2005	133.4	120.4	146.6
Nigeria	Akwa Ibom	Okobo	22634	2010	112.7	100.8	125.8
Nigeria	Akwa Ibom	Okobo	22634	2015	92.5	80.1	105.2
Nigeria	Akwa Ibom	Onna	22635	2000	131.4	118.7	145.2
Nigeria	Akwa Ibom	Onna	22635	2005	129.3	116.6	143.4
Nigeria	Akwa Ibom	Onna	22635	2010	105.6	94.0	118.2
Nigeria	Akwa Ibom	Onna	22635	2015	84.6	73.0	96.5
Nigeria	Akwa Ibom	Oron	191425	2000	130.4	117.0	144.6
Nigeria	Akwa Ibom	Oron	191425	2005	129.5	115.6	143.6
Nigeria	Akwa Ibom	Oron	191425	2010	108.6	96.7	121.9
Nigeria	Akwa Ibom	Oron	191425	2015	89.0	76.5	101.8
Nigeria	Akwa Ibom	Oruk Anam	22637	2000	137.0	125.2	149.9
Nigeria	Akwa Ibom	Oruk Anam	22637	2005	134.5	124.1	146.2
Nigeria	Akwa Ibom	Oruk Anam	22637	2010	108.1	98.1	118.8
Nigeria	Akwa Ibom	Oruk Anam	22637	2015	86.2	75.6	96.8
Nigeria	Akwa Ibom	Udung Uko	191391	2000	133.3	118.6	149.9
Nigeria	Akwa Ibom	Udung Uko	191391	2005	132.6	117.4	149.4
Nigeria	Akwa Ibom	Udung Uko	191391	2010	113.2	99.6	128.2
Nigeria	Akwa Ibom	Udung Uko	191391	2015	93.3	79.8	107.2
Nigeria	Akwa Ibom	Ukanafun	22638	2000	139.0	126.7	152.4
Nigeria	Akwa Ibom	Ukanafun	22638	2005	136.6	125.6	148.5
Nigeria	Akwa Ibom	Ukanafun	22638	2010	108.8	99.2	119.5
Nigeria	Akwa Ibom	Ukanafun	22638	2015	84.7	74.6	95.2
Nigeria	Akwa Ibom	Uruan	22640	2000	134.5	123.0	146.0
Nigeria	Akwa Ibom	Uruan	22640	2005	133.2	122.3	145.3
Nigeria	Akwa Ibom	Uruan	22640	2010	110.8	100.9	122.9
Nigeria	Akwa Ibom	Uruan	22640	2015	90.1	79.6	101.8
Nigeria	Akwa Ibom	Urue Offong /Oruko	22641	2000	133.4	119.0	149.3
Nigeria	Akwa Ibom	Urue Offong /Oruko	22641	2005	132.7	117.8	148.2
Nigeria	Akwa Ibom	Urue Offong /Oruko	22641	2010	112.9	99.4	127.9
Nigeria	Akwa Ibom	Urue Offong /Oruko	22641	2015	92.4	79.4	106.2
Nigeria	Akwa Ibom	Uyo	191377	2000	131.5	120.9	143.4
Nigeria	Akwa Ibom	Uyo	191377	2005	130.1	119.3	141.5
Nigeria	Akwa Ibom	Uyo	191377	2010	105.7	95.9	117.0
Nigeria	Akwa Ibom	Uyo	191377	2015	83.4	73.7	94.5
Nigeria	Anambra	Aguata	22643	2000	129.2	119.5	139.9
Nigeria	Anambra	Aguata	22643	2005	120.9	112.1	130.6
Nigeria	Anambra	Aguata	22643	2010	104.5	95.9	113.3
Nigeria	Anambra	Aguata	22643	2015	76.5	68.0	85.7
Nigeria	Anambra	Anambra East	191304	2000	113.8	104.4	124.3
Nigeria	Anambra	Anambra East	191304	2005	110.0	101.1	119.9
Nigeria	Anambra	Anambra East	191304	2010	96.6	87.8	105.9
Nigeria	Anambra	Anambra East	191304	2015	71.0	62.9	80.4
Nigeria	Anambra	Anambra West	22644	2000	131.3	120.0	144.8
Nigeria	Anambra	Anambra West	22644	2005	122.1	111.0	133.9
Nigeria	Anambra	Anambra West	22644	2010	108.4	98.4	119.7
Nigeria	Anambra	Anambra West	22644	2015	81.4	72.0	91.8
Nigeria	Anambra	Anaocha	22645	2000	127.3	117.5	137.8
Nigeria	Anambra	Anaocha	22645	2005	118.9	110.2	128.9
Nigeria	Anambra	Anaocha	22645	2010	103.9	95.1	112.7
Nigeria	Anambra	Anaocha	22645	2015	76.4	68.0	85.2
Nigeria	Anambra	Awka North	22646	2000	126.7	116.3	137.8
Nigeria	Anambra	Awka North	22646	2005	121.7	111.7	132.0
Nigeria	Anambra	Awka North	22646	2010	108.0	98.6	118.7
Nigeria	Anambra	Awka North	22646	2015	77.1	67.7	87.4
Nigeria	Anambra	Awka South	22647	2000	123.4	113.8	133.6

Admin 0	Admin 1	Admin 2	GAUL Code	Year	Under-5 mortality (per 1,000 livebirths) Estimate	Lower bound, 95% UI	Upper bound, 95% UI
Nigeria	Anambra	Awka South	22647	2005	118.4	108.8	128.0
Nigeria	Anambra	Awka South	22647	2010	105.1	96.3	115.3
Nigeria	Anambra	Awka South	22647	2015	76.0	67.2	85.2
Nigeria	Anambra	Ayamelum	191283	2000	132.6	120.4	146.6
Nigeria	Anambra	Ayamelum	191283	2005	125.1	113.5	138.3
Nigeria	Anambra	Ayamelum	191283	2010	111.2	100.7	123.0
Nigeria	Anambra	Ayamelum	191283	2015	81.3	71.6	92.7
Nigeria	Anambra	Dunukofia	191317	2000	121.0	111.6	131.6
Nigeria	Anambra	Dunukofia	191317	2005	115.3	106.2	124.6
Nigeria	Anambra	Dunukofia	191317	2010	100.8	92.0	109.9
Nigeria	Anambra	Dunukofia	191317	2015	73.5	65.1	82.8
Nigeria	Anambra	Ekwusigo	191328	2000	124.2	114.7	134.6
Nigeria	Anambra	Ekwusigo	191328	2005	114.8	106.4	124.0
Nigeria	Anambra	Ekwusigo	191328	2010	99.4	91.1	107.0
Nigeria	Anambra	Ekwusigo	191328	2015	72.2	64.3	80.7
Nigeria	Anambra	Idemili North	191321	2000	119.5	110.2	129.8
Nigeria	Anambra	Idemili North	191321	2005	112.1	103.6	121.2
Nigeria	Anambra	Idemili North	191321	2010	98.2	90.1	106.3
Nigeria	Anambra	Idemili North	191321	2015	72.3	64.4	81.3
Nigeria	Anambra	Idemili South	191326	2000	120.6	111.3	130.5
Nigeria	Anambra	Idemili South	191326	2005	112.6	104.3	121.7
Nigeria	Anambra	Idemili South	191326	2010	98.6	90.6	106.4
Nigeria	Anambra	Idemili South	191326	2015	71.7	63.8	80.4
Nigeria	Anambra	Ihiala	22649	2000	131.8	121.9	142.6
Nigeria	Anambra	Ihiala	22649	2005	122.1	113.4	132.1
Nigeria	Anambra	Ihiala	22649	2010	104.4	95.8	113.8
Nigeria	Anambra	Ihiala	22649	2015	75.9	67.2	84.5
Nigeria	Anambra	Njikoka	191319	2000	123.3	113.8	133.8
Nigeria	Anambra	Njikoka	191319	2005	117.2	107.9	126.5
Nigeria	Anambra	Njikoka	191319	2010	103.0	94.3	112.5
Nigeria	Anambra	Njikoka	191319	2015	74.4	65.8	83.8
Nigeria	Anambra	Nnewi North	191329	2000	121.8	112.8	131.8
Nigeria	Anambra	Nnewi North	191329	2005	113.5	105.4	122.4
Nigeria	Anambra	Nnewi North	191329	2010	98.6	90.7	105.9
Nigeria	Anambra	Nnewi North	191329	2015	72.3	64.6	81.1
Nigeria	Anambra	Nnewi South	22652	2000	129.9	120.7	140.3
Nigeria	Anambra	Nnewi South	22652	2005	120.0	111.9	129.4
Nigeria	Anambra	Nnewi South	22652	2010	102.8	94.6	110.7
Nigeria	Anambra	Nnewi South	22652	2015	75.3	67.0	83.9
Nigeria	Anambra	Ogbaru	22653	2000	128.8	118.7	139.9
Nigeria	Anambra	Ogbaru	22653	2005	117.7	108.5	126.9
Nigeria	Anambra	Ogbaru	22653	2010	104.1	95.0	113.2
Nigeria	Anambra	Ogbaru	22653	2015	74.9	66.7	83.9
Nigeria	Anambra	Onitsha North	191323	2000	109.1	100.0	119.2
Nigeria	Anambra	Onitsha North	191323	2005	103.7	95.2	113.3
Nigeria	Anambra	Onitsha North	191323	2010	91.1	82.6	99.2
Nigeria	Anambra	Onitsha North	191323	2015	68.7	60.9	77.6
Nigeria	Anambra	Onitsha South	191325	2000	122.9	113.1	134.1
Nigeria	Anambra	Onitsha South	191325	2005	113.8	104.8	123.7
Nigeria	Anambra	Onitsha South	191325	2010	99.3	90.3	107.6
Nigeria	Anambra	Onitsha South	191325	2015	72.3	64.2	81.3
Nigeria	Anambra	Orumba North	22655	2000	132.6	122.5	143.3
Nigeria	Anambra	Orumba North	22655	2005	125.1	115.4	135.8
Nigeria	Anambra	Orumba North	22655	2010	108.3	99.2	118.1
Nigeria	Anambra	Orumba North	22655	2015	78.3	69.3	87.6
Nigeria	Anambra	Orumba South	22656	2000	141.5	131.2	152.4
Nigeria	Anambra	Orumba South	22656	2005	131.7	122.4	142.3
Nigeria	Anambra	Orumba South	22656	2010	112.7	103.5	122.6
Nigeria	Anambra	Orumba South	22656	2015	81.9	72.4	92.0
Nigeria	Anambra	Oyi	191316	2000	111.3	102.1	121.6
Nigeria	Anambra	Oyi	191316	2005	107.6	98.7	117.1
Nigeria	Anambra	Oyi	191316	2010	94.6	86.2	103.5
Nigeria	Anambra	Oyi	191316	2015	70.7	62.8	79.8
Nigeria	Bauchi	Alkaleri	191104	2000	267.3	245.8	290.7
Nigeria	Bauchi	Alkaleri	191104	2005	228.4	209.0	248.1
Nigeria	Bauchi	Alkaleri	191104	2010	190.6	173.5	210.0
Nigeria	Bauchi	Alkaleri	191104	2015	177.1	156.8	198.5
Nigeria	Bauchi	Bauchi	22661	2000	252.5	229.3	278.3
Nigeria	Bauchi	Bauchi	22661	2005	219.3	198.9	241.1
Nigeria	Bauchi	Bauchi	22661	2010	182.4	164.9	201.9
Nigeria	Bauchi	Bauchi	22661	2015	163.1	142.7	185.8
Nigeria	Bauchi	Bogoro	191120	2000	250.2	225.2	276.8
Nigeria	Bauchi	Bogoro	191120	2005	223.3	200.0	248.6
Nigeria	Bauchi	Bogoro	191120	2010	194.1	172.7	218.1
Nigeria	Bauchi	Bogoro	191120	2015	168.4	147.0	191.5
Nigeria	Bauchi	Damban	191054	2000	236.3	218.8	254.3
Nigeria	Bauchi	Damban	191054	2005	197.0	182.2	212.5
Nigeria	Bauchi	Damban	191054	2010	174.3	159.3	190.6
Nigeria	Bauchi	Damban	191054	2015	158.0	140.3	178.1
Nigeria	Bauchi	Darazo	22663	2000	260.2	239.5	280.4
Nigeria	Bauchi	Darazo	22663	2005	218.1	200.3	236.1
Nigeria	Bauchi	Darazo	22663	2010	190.4	174.1	208.3
Nigeria	Bauchi	Darazo	22663	2015	174.3	155.2	196.5
Nigeria	Bauchi	Dass	22664	2000	277.8	250.2	306.4
Nigeria	Bauchi	Dass	22664	2005	237.7	214.1	264.3

Admin 0	Admin 1	Admin 2	GAUL Code	Year	Under-5 mortality (per 1,000 livebirths)		
					Estimate	Lower bound, 95% UI	Upper bound, 95% UI
Nigeria	Bauchi	Dass	22664	2010	199.9	179.1	222.8
Nigeria	Bauchi	Dass	22664	2015	176.0	154.1	200.6
Nigeria	Bauchi	Gamawa	22666	2000	241.8	224.0	259.8
Nigeria	Bauchi	Gamawa	22666	2005	206.8	192.0	223.3
Nigeria	Bauchi	Gamawa	22666	2010	181.9	166.9	198.1
Nigeria	Bauchi	Gamawa	22666	2015	163.9	145.7	183.5
Nigeria	Bauchi	Ganjuwa	22667	2000	286.1	264.7	308.8
Nigeria	Bauchi	Ganjuwa	22667	2005	247.1	228.4	266.0
Nigeria	Bauchi	Ganjuwa	22667	2010	216.0	198.4	236.0
Nigeria	Bauchi	Ganjuwa	22667	2015	193.4	172.5	217.4
Nigeria	Bauchi	Giade	191068	2000	273.3	252.2	294.8
Nigeria	Bauchi	Giade	191068	2005	233.5	214.9	252.7
Nigeria	Bauchi	Giade	191068	2010	204.7	187.6	222.7
Nigeria	Bauchi	Giade	191068	2015	185.3	164.3	208.2
Nigeria	Bauchi	Itas/Gadau	22669	2000	295.7	276.6	315.8
Nigeria	Bauchi	Itas/Gadau	22669	2005	254.1	237.3	270.8
Nigeria	Bauchi	Itas/Gadau	22669	2010	217.9	201.0	234.9
Nigeria	Bauchi	Itas/Gadau	22669	2015	194.0	173.9	217.0
Nigeria	Bauchi	Jama'are	22670	2000	288.8	268.3	309.3
Nigeria	Bauchi	Jama'are	22670	2005	246.0	229.5	263.6
Nigeria	Bauchi	Jama'are	22670	2010	216.6	200.0	234.3
Nigeria	Bauchi	Jama'are	22670	2015	191.1	170.4	214.3
Nigeria	Bauchi	Katagum	191051	2000	265.9	247.9	285.1
Nigeria	Bauchi	Katagum	191051	2005	222.7	208.5	238.9
Nigeria	Bauchi	Katagum	191051	2010	189.0	174.7	204.0
Nigeria	Bauchi	Katagum	191051	2015	180.5	161.2	202.7
Nigeria	Bauchi	Kirfi	191096	2000	276.1	249.2	304.8
Nigeria	Bauchi	Kirfi	191096	2005	232.9	209.4	259.2
Nigeria	Bauchi	Kirfi	191096	2010	201.7	180.7	226.8
Nigeria	Bauchi	Kirfi	191096	2015	184.8	162.0	210.0
Nigeria	Bauchi	Misau	191070	2000	249.8	230.6	269.0
Nigeria	Bauchi	Misau	191070	2005	206.1	189.8	221.5
Nigeria	Bauchi	Misau	191070	2010	176.9	162.2	193.4
Nigeria	Bauchi	Misau	191070	2015	164.6	145.4	186.6
Nigeria	Bauchi	Ningi	191081	2000	319.2	295.1	342.3
Nigeria	Bauchi	Ningi	191081	2005	271.2	250.2	294.0
Nigeria	Bauchi	Ningi	191081	2010	235.4	214.1	257.1
Nigeria	Bauchi	Ningi	191081	2015	214.6	190.2	240.6
Nigeria	Bauchi	Shira	191067	2000	280.8	261.1	300.3
Nigeria	Bauchi	Shira	191067	2005	239.0	220.6	255.9
Nigeria	Bauchi	Shira	191067	2010	211.7	194.8	228.0
Nigeria	Bauchi	Shira	191067	2015	190.6	169.3	213.3
Nigeria	Bauchi	Tafawa-Balewa	191107	2000	262.1	238.6	285.8
Nigeria	Bauchi	Tafawa-Balewa	191107	2005	229.1	208.7	251.2
Nigeria	Bauchi	Tafawa-Balewa	191107	2010	195.5	176.4	217.4
Nigeria	Bauchi	Tafawa-Balewa	191107	2015	173.5	152.7	195.9
Nigeria	Bauchi	Toro	22678	2000	283.9	260.2	308.3
Nigeria	Bauchi	Toro	22678	2005	238.9	220.4	259.8
Nigeria	Bauchi	Toro	22678	2010	207.8	189.7	227.1
Nigeria	Bauchi	Toro	22678	2015	192.2	171.2	215.6
Nigeria	Bauchi	Warji	191083	2000	300.0	278.3	321.4
Nigeria	Bauchi	Warji	191083	2005	254.6	236.2	274.0
Nigeria	Bauchi	Warji	191083	2010	228.9	210.0	247.6
Nigeria	Bauchi	Warji	191083	2015	202.0	178.5	226.3
Nigeria	Bauchi	Zaki	22680	2000	256.7	238.8	275.7
Nigeria	Bauchi	Zaki	22680	2005	223.9	207.8	242.2
Nigeria	Bauchi	Zaki	22680	2010	192.6	177.1	208.8
Nigeria	Bauchi	Zaki	22680	2015	171.5	152.6	192.9
Nigeria	Bayelsa	Brass	191393	2000	165.6	150.4	183.5
Nigeria	Bayelsa	Brass	191393	2005	149.7	135.0	164.7
Nigeria	Bayelsa	Brass	191393	2010	128.4	114.9	142.6
Nigeria	Bayelsa	Brass	191393	2015	98.2	86.2	111.0
Nigeria	Bayelsa	Ekeremor	23062	2000	173.9	158.4	192.1
Nigeria	Bayelsa	Ekeremor	23062	2005	156.9	142.4	172.7
Nigeria	Bayelsa	Ekeremor	23062	2010	140.2	125.9	155.1
Nigeria	Bayelsa	Ekeremor	23062	2015	115.8	101.9	131.2
Nigeria	Bayelsa	Kolokuma/Opokuma	191370	2000	165.3	151.2	180.5
Nigeria	Bayelsa	Kolokuma/Opokuma	191370	2005	145.7	133.0	158.3
Nigeria	Bayelsa	Kolokuma/Opokuma	191370	2010	126.7	115.3	140.2
Nigeria	Bayelsa	Kolokuma/Opokuma	191370	2015	92.5	81.9	104.1
Nigeria	Bayelsa	Nembe	191398	2000	167.9	151.9	185.6
Nigeria	Bayelsa	Nembe	191398	2005	153.6	138.4	169.9
Nigeria	Bayelsa	Nembe	191398	2010	132.2	118.0	148.0
Nigeria	Bayelsa	Nembe	191398	2015	101.2	88.6	114.8
Nigeria	Bayelsa	Ogbia	191383	2000	172.6	157.6	188.5
Nigeria	Bayelsa	Ogbia	191383	2005	154.5	140.4	168.7
Nigeria	Bayelsa	Ogbia	191383	2010	132.4	119.7	145.9
Nigeria	Bayelsa	Ogbia	191383	2015	97.9	86.3	110.7
Nigeria	Bayelsa	Sagbama	23073	2000	166.1	153.1	179.5
Nigeria	Bayelsa	Sagbama	23073	2005	146.2	135.5	157.5
Nigeria	Bayelsa	Sagbama	23073	2010	129.0	117.8	141.5
Nigeria	Bayelsa	Sagbama	23073	2015	96.9	86.5	108.3
Nigeria	Bayelsa	Southern Ijaw	23074	2000	180.1	164.3	197.1
Nigeria	Bayelsa	Southern Ijaw	23074	2005	161.5	147.7	176.8
Nigeria	Bayelsa	Southern Ijaw	23074	2010	142.9	129.8	156.9

Admin 0	Admin 1	Admin 2	GAUL Code	Year	Under-5 mortality (per 1,000 livebirths) Estimate	Lower bound, 95% UI	Upper bound, 95% UI
Nigeria	Bayelsa	Southern Ijaw	23074	2015	113.7	100.5	127.9
Nigeria	Bayelsa	Yenegoa	191359	2000	161.0	148.2	174.2
Nigeria	Bayelsa	Yenegoa	191359	2005	141.8	129.9	153.9
Nigeria	Bayelsa	Yenegoa	191359	2010	123.6	112.9	135.7
Nigeria	Bayelsa	Yenegoa	191359	2015	89.5	79.2	100.4
Nigeria	Benue	Ado	22681	2000	190.0	172.5	206.7
Nigeria	Benue	Ado	22681	2005	164.6	150.7	180.1
Nigeria	Benue	Ado	22681	2010	143.0	130.7	157.3
Nigeria	Benue	Ado	22681	2015	114.4	100.6	130.0
Nigeria	Benue	Agatu	191192	2000	158.8	142.8	176.8
Nigeria	Benue	Agatu	191192	2005	134.7	121.4	149.4
Nigeria	Benue	Agatu	191192	2010	119.7	106.7	133.4
Nigeria	Benue	Agatu	191192	2015	100.6	87.1	115.8
Nigeria	Benue	Apa	191204	2000	160.1	144.7	178.6
Nigeria	Benue	Apa	191204	2005	137.6	123.6	152.7
Nigeria	Benue	Apa	191204	2010	121.3	108.0	135.9
Nigeria	Benue	Apa	191204	2015	100.3	87.1	115.2
Nigeria	Benue	Bukuru	22683	2000	146.4	132.7	160.5
Nigeria	Benue	Bukuru	22683	2005	129.1	116.8	141.9
Nigeria	Benue	Bukuru	22683	2010	113.1	101.6	125.3
Nigeria	Benue	Bukuru	22683	2015	98.7	86.7	113.1
Nigeria	Benue	Gboko	191221	2000	139.2	126.1	153.4
Nigeria	Benue	Gboko	191221	2005	126.0	113.2	139.5
Nigeria	Benue	Gboko	191221	2010	111.2	99.7	123.9
Nigeria	Benue	Gboko	191221	2015	92.6	81.0	106.7
Nigeria	Benue	Guma	22685	2000	165.5	149.3	182.7
Nigeria	Benue	Guma	22685	2005	142.9	129.0	158.9
Nigeria	Benue	Guma	22685	2010	124.3	110.9	138.5
Nigeria	Benue	Guma	22685	2015	110.6	95.6	127.2
Nigeria	Benue	Gwer East	22686	2000	164.4	149.2	180.8
Nigeria	Benue	Gwer East	22686	2005	141.6	129.3	155.6
Nigeria	Benue	Gwer East	22686	2010	125.2	112.6	138.2
Nigeria	Benue	Gwer East	22686	2015	105.5	93.1	119.6
Nigeria	Benue	Gwer West	22687	2000	161.3	146.5	178.1
Nigeria	Benue	Gwer West	22687	2005	139.8	127.2	153.2
Nigeria	Benue	Gwer West	22687	2010	123.8	110.9	136.6
Nigeria	Benue	Gwer West	22687	2015	102.9	90.1	117.0
Nigeria	Benue	Katsina- Ala	191220	2000	154.8	139.5	171.2
Nigeria	Benue	Katsina- Ala	191220	2005	135.6	122.5	149.8
Nigeria	Benue	Katsina- Ala	191220	2010	118.7	106.2	131.9
Nigeria	Benue	Katsina- Ala	191220	2015	104.8	91.5	119.2
Nigeria	Benue	Konshisha	22689	2000	153.8	140.5	167.7
Nigeria	Benue	Konshisha	22689	2005	134.2	122.2	147.1
Nigeria	Benue	Konshisha	22689	2010	119.1	107.4	131.9
Nigeria	Benue	Konshisha	22689	2015	101.5	89.5	115.0
Nigeria	Benue	Kwande	22690	2000	141.9	127.5	157.6
Nigeria	Benue	Kwande	22690	2005	129.3	117.3	142.9
Nigeria	Benue	Kwande	22690	2010	110.9	98.9	124.0
Nigeria	Benue	Kwande	22690	2015	95.2	83.4	109.1
Nigeria	Benue	Logo	191197	2000	160.5	144.9	177.1
Nigeria	Benue	Logo	191197	2005	140.0	126.0	153.7
Nigeria	Benue	Logo	191197	2010	121.3	107.6	134.9
Nigeria	Benue	Logo	191197	2015	108.3	94.6	123.4
Nigeria	Benue	Markurdi	22691	2000	139.9	124.0	157.2
Nigeria	Benue	Markurdi	22691	2005	125.1	111.5	141.3
Nigeria	Benue	Markurdi	22691	2010	110.6	97.5	124.7
Nigeria	Benue	Markurdi	22691	2015	89.4	77.3	104.2
Nigeria	Benue	Obi	191261	2000	170.5	154.0	188.6
Nigeria	Benue	Obi	191261	2005	146.9	133.6	161.5
Nigeria	Benue	Obi	191261	2010	128.2	114.7	141.7
Nigeria	Benue	Obi	191261	2015	104.9	91.8	119.4
Nigeria	Benue	Ogbadigbo	22692	2000	164.8	151.9	179.4
Nigeria	Benue	Ogbadigbo	22692	2005	144.0	132.3	156.9
Nigeria	Benue	Ogbadigbo	22692	2010	126.7	114.9	139.6
Nigeria	Benue	Ogbadigbo	22692	2015	97.7	86.7	110.7
Nigeria	Benue	Ohimini	191246	2000	163.1	148.3	180.1
Nigeria	Benue	Ohimini	191246	2005	141.6	128.4	156.4
Nigeria	Benue	Ohimini	191246	2010	123.2	110.2	136.0
Nigeria	Benue	Ohimini	191246	2015	97.5	85.1	111.3
Nigeria	Benue	Oju	191267	2000	170.0	154.4	187.4
Nigeria	Benue	Oju	191267	2005	147.5	134.4	162.6
Nigeria	Benue	Oju	191267	2010	129.0	116.1	142.4
Nigeria	Benue	Oju	191267	2015	105.9	93.2	120.4
Nigeria	Benue	Okpokwu	22694	2000	171.0	157.2	186.6
Nigeria	Benue	Okpokwu	22694	2005	147.3	135.2	160.4
Nigeria	Benue	Okpokwu	22694	2010	128.1	116.8	140.3
Nigeria	Benue	Okpokwu	22694	2015	100.2	88.5	113.9
Nigeria	Benue	Oturkpo	191218	2000	158.5	142.9	175.7
Nigeria	Benue	Oturkpo	191218	2005	137.6	125.0	151.9
Nigeria	Benue	Oturkpo	191218	2010	121.2	108.7	134.1
Nigeria	Benue	Oturkpo	191218	2015	95.5	83.6	108.7
Nigeria	Benue	Tarka	191210	2000	156.0	140.1	174.1
Nigeria	Benue	Tarka	191210	2005	135.6	120.9	151.2
Nigeria	Benue	Tarka	191210	2010	118.9	104.6	132.8
Nigeria	Benue	Tarka	191210	2015	104.4	90.2	120.5

Admin 0	Admin 1	Admin 2	GAUL Code	Year	Under-5 mortality (per 1,000 livebirths)		
					Estimate	Lower bound, 95% UI	Upper bound, 95% UI
Nigeria	Benue	Ukum	22696	2000	162.6	146.9	179.8
Nigeria	Benue	Ukum	22696	2005	142.0	128.4	156.5
Nigeria	Benue	Ukum	22696	2010	123.3	109.4	136.8
Nigeria	Benue	Ukum	22696	2015	110.6	96.4	125.0
Nigeria	Benue	Ushongo	22697	2000	142.4	128.4	156.8
Nigeria	Benue	Ushongo	22697	2005	125.8	113.1	138.4
Nigeria	Benue	Ushongo	22697	2010	111.1	99.6	123.2
Nigeria	Benue	Ushongo	22697	2015	95.7	84.0	109.4
Nigeria	Benue	Vandeikya	22698	2000	139.6	125.1	154.5
Nigeria	Benue	Vandeikya	22698	2005	124.3	111.1	138.4
Nigeria	Benue	Vandeikya	22698	2010	109.7	97.7	122.9
Nigeria	Benue	Vandeikya	22698	2015	93.6	81.6	107.7
Nigeria	Borno	Abadam	190950	2000	156.8	138.5	177.4
Nigeria	Borno	Abadam	190950	2005	132.3	116.0	150.9
Nigeria	Borno	Abadam	190950	2010	114.9	99.9	133.0
Nigeria	Borno	Abadam	190950	2015	107.3	90.8	126.8
Nigeria	Borno	Askira/Uba	22699	2000	217.4	198.1	237.0
Nigeria	Borno	Askira/Uba	22699	2005	186.4	170.2	203.3
Nigeria	Borno	Askira/Uba	22699	2010	148.6	134.3	163.2
Nigeria	Borno	Askira/Uba	22699	2015	128.7	113.4	144.6
Nigeria	Borno	Bama	22700	2000	239.0	217.5	261.2
Nigeria	Borno	Bama	22700	2005	205.0	186.7	224.7
Nigeria	Borno	Bama	22700	2010	174.0	156.9	192.6
Nigeria	Borno	Bama	22700	2015	161.6	142.6	183.0
Nigeria	Borno	Bayo	191093	2000	281.3	254.3	308.8
Nigeria	Borno	Bayo	191093	2005	234.4	213.2	255.9
Nigeria	Borno	Bayo	191093	2010	189.9	170.6	210.5
Nigeria	Borno	Bayo	191093	2015	167.0	145.7	191.5
Nigeria	Borno	Biu	191089	2000	250.1	225.1	278.8
Nigeria	Borno	Biu	191089	2005	209.9	189.2	232.7
Nigeria	Borno	Biu	191089	2010	172.0	153.6	194.5
Nigeria	Borno	Biu	191089	2015	145.2	125.7	167.7
Nigeria	Borno	Chibok	191091	2000	226.5	203.1	251.7
Nigeria	Borno	Chibok	191091	2005	188.8	171.0	208.5
Nigeria	Borno	Chibok	191091	2010	150.3	133.8	167.0
Nigeria	Borno	Chibok	191091	2015	132.0	115.3	152.4
Nigeria	Borno	Damboa	191073	2000	230.0	209.7	251.9
Nigeria	Borno	Damboa	191073	2005	193.1	176.1	212.2
Nigeria	Borno	Damboa	191073	2010	157.7	141.8	174.3
Nigeria	Borno	Damboa	191073	2015	136.5	120.4	155.0
Nigeria	Borno	Dikwa	22703	2000	222.1	197.7	249.2
Nigeria	Borno	Dikwa	22703	2005	190.1	169.1	213.2
Nigeria	Borno	Dikwa	22703	2010	151.5	133.6	172.0
Nigeria	Borno	Dikwa	22703	2015	140.3	121.3	162.1
Nigeria	Borno	Gubio	190969	2000	217.5	191.9	245.1
Nigeria	Borno	Gubio	190969	2005	184.6	163.6	208.9
Nigeria	Borno	Gubio	190969	2010	160.2	138.9	181.0
Nigeria	Borno	Gubio	190969	2015	137.2	117.5	160.6
Nigeria	Borno	Guzamala	190958	2000	191.3	169.4	215.1
Nigeria	Borno	Guzamala	190958	2005	160.7	142.6	181.7
Nigeria	Borno	Guzamala	190958	2010	137.1	119.9	157.1
Nigeria	Borno	Guzamala	190958	2015	127.1	108.4	150.4
Nigeria	Borno	Gwoza	22704	2000	220.1	199.0	241.6
Nigeria	Borno	Gwoza	22704	2005	192.4	174.6	210.4
Nigeria	Borno	Gwoza	22704	2010	162.7	145.0	180.6
Nigeria	Borno	Gwoza	22704	2015	144.1	126.9	165.0
Nigeria	Borno	Hawul	191097	2000	262.5	237.7	292.3
Nigeria	Borno	Hawul	191097	2005	219.6	198.6	242.8
Nigeria	Borno	Hawul	191097	2010	175.3	157.4	196.7
Nigeria	Borno	Hawul	191097	2015	147.1	128.4	168.4
Nigeria	Borno	Jere	191026	2000	194.6	176.3	214.8
Nigeria	Borno	Jere	191026	2005	163.1	148.6	179.5
Nigeria	Borno	Jere	191026	2010	129.9	116.3	145.4
Nigeria	Borno	Jere	191026	2015	103.7	90.0	118.2
Nigeria	Borno	Kaga	191041	2000	248.6	222.3	277.6
Nigeria	Borno	Kaga	191041	2005	207.7	185.6	232.3
Nigeria	Borno	Kaga	191041	2010	177.2	154.7	200.7
Nigeria	Borno	Kaga	191041	2015	149.3	127.7	173.1
Nigeria	Borno	Kala/Balge	191010	2000	217.3	194.8	241.6
Nigeria	Borno	Kala/Balge	191010	2005	190.2	170.4	210.9
Nigeria	Borno	Kala/Balge	191010	2010	159.2	141.1	179.7
Nigeria	Borno	Kala/Balge	191010	2015	158.9	138.3	180.8
Nigeria	Borno	Konduga	191024	2000	187.8	171.4	205.8
Nigeria	Borno	Konduga	191024	2005	153.9	140.9	168.5
Nigeria	Borno	Konduga	191024	2010	123.7	110.6	137.8
Nigeria	Borno	Konduga	191024	2015	102.5	88.8	116.8
Nigeria	Borno	Kukawa	190954	2000	175.1	155.1	197.3
Nigeria	Borno	Kukawa	190954	2005	147.0	129.7	166.0
Nigeria	Borno	Kukawa	190954	2010	124.0	108.0	141.1
Nigeria	Borno	Kukawa	190954	2015	113.5	97.2	133.4
Nigeria	Borno	Kwaya Kusar	191099	2000	279.3	252.3	305.7
Nigeria	Borno	Kwaya Kusar	191099	2005	234.2	211.3	257.2
Nigeria	Borno	Kwaya Kusar	191099	2010	190.6	170.9	212.3
Nigeria	Borno	Kwaya Kusar	191099	2015	161.7	140.8	186.2
Nigeria	Borno	Lake chad	190951	2000	155.2	138.9	173.3

Admin 0	Admin 1	Admin 2	GAUL Code	Year	Under-5 mortality (per 1,000 livebirths)		
					Estimate	Lower bound, 95% UI	Upper bound, 95% UI
Nigeria	Borno	Lake chad	190951	2005	137.3	123.0	153.1
Nigeria	Borno	Lake chad	190951	2010	118.6	104.9	133.3
Nigeria	Borno	Lake chad	190951	2015	107.2	94.1	123.3
Nigeria	Borno	Mafa	191014	2000	210.7	189.7	232.5
Nigeria	Borno	Mafa	191014	2005	178.0	160.7	197.7
Nigeria	Borno	Mafa	191014	2010	143.1	126.5	160.4
Nigeria	Borno	Mafa	191014	2015	121.0	104.9	138.9
Nigeria	Borno	Magumeri	190998	2000	230.3	205.8	256.8
Nigeria	Borno	Magumeri	190998	2005	193.3	172.8	216.2
Nigeria	Borno	Magumeri	190998	2010	163.6	143.7	184.8
Nigeria	Borno	Magumeri	190998	2015	138.1	117.9	160.0
Nigeria	Borno	Maiduguri	191040	2000	163.2	145.6	182.2
Nigeria	Borno	Maiduguri	191040	2005	131.8	118.9	147.1
Nigeria	Borno	Maiduguri	191040	2010	107.6	95.0	122.2
Nigeria	Borno	Maiduguri	191040	2015	88.3	75.8	102.6
Nigeria	Borno	Marte	191406	2000	199.2	178.4	222.2
Nigeria	Borno	Marte	191406	2005	167.7	150.4	187.4
Nigeria	Borno	Marte	191406	2010	132.1	117.2	148.5
Nigeria	Borno	Marte	191406	2015	117.5	101.6	135.7
Nigeria	Borno	Mobbar	191407	2000	183.8	163.6	205.8
Nigeria	Borno	Mobbar	191407	2005	157.3	138.2	177.5
Nigeria	Borno	Mobbar	191407	2010	138.2	119.0	157.8
Nigeria	Borno	Mobbar	191407	2015	120.2	101.8	140.4
Nigeria	Borno	Monguno	190987	2000	201.3	177.0	226.0
Nigeria	Borno	Monguno	190987	2005	168.1	148.2	190.9
Nigeria	Borno	Monguno	190987	2010	139.8	121.1	158.8
Nigeria	Borno	Monguno	190987	2015	126.0	106.6	147.9
Nigeria	Borno	Ngala	191006	2000	196.4	173.6	221.6
Nigeria	Borno	Ngala	191006	2005	165.8	147.6	184.6
Nigeria	Borno	Ngala	191006	2010	127.3	111.6	143.8
Nigeria	Borno	Ngala	191006	2015	127.0	109.3	145.8
Nigeria	Borno	Nganzai	190976	2000	211.4	189.6	234.6
Nigeria	Borno	Nganzai	190976	2005	177.0	156.7	199.8
Nigeria	Borno	Nganzai	190976	2010	149.5	130.6	171.2
Nigeria	Borno	Nganzai	190976	2015	131.0	111.4	153.2
Nigeria	Borno	Shani	191102	2000	279.5	252.9	308.1
Nigeria	Borno	Shani	191102	2005	236.6	213.7	261.2
Nigeria	Borno	Shani	191102	2010	187.5	167.3	209.2
Nigeria	Borno	Shani	191102	2015	156.6	136.4	179.9
Nigeria	Cross River	Abi	22713	2000	165.1	152.4	178.7
Nigeria	Cross River	Abi	22713	2005	156.6	144.7	169.8
Nigeria	Cross River	Abi	22713	2010	132.6	120.9	145.5
Nigeria	Cross River	Abi	22713	2015	97.6	85.9	110.3
Nigeria	Cross River	Akamkpa	22714	2000	143.5	130.4	159.0
Nigeria	Cross River	Akamkpa	22714	2005	140.1	126.8	153.6
Nigeria	Cross River	Akamkpa	22714	2010	120.1	108.4	133.3
Nigeria	Cross River	Akamkpa	22714	2015	94.8	82.5	108.1
Nigeria	Cross River	Akpabuyo	191376	2000	135.8	121.9	151.1
Nigeria	Cross River	Akpabuyo	191376	2005	133.9	120.0	148.6
Nigeria	Cross River	Akpabuyo	191376	2010	113.3	99.8	127.7
Nigeria	Cross River	Akpabuyo	191376	2015	91.7	79.5	106.2
Nigeria	Cross River	Bakassi	191417	2000	143.0	127.1	160.3
Nigeria	Cross River	Bakassi	191417	2005	134.8	119.6	151.4
Nigeria	Cross River	Bakassi	191417	2010	113.0	98.7	128.2
Nigeria	Cross River	Bakassi	191417	2015	91.1	78.3	105.1
Nigeria	Cross River	Bekwarra	191280	2000	122.7	110.6	136.4
Nigeria	Cross River	Bekwarra	191280	2005	115.6	103.7	128.9
Nigeria	Cross River	Bekwarra	191280	2010	101.8	90.7	114.8
Nigeria	Cross River	Bekwarra	191280	2015	79.2	68.6	91.9
Nigeria	Cross River	Biase	22716	2000	155.0	141.8	170.4
Nigeria	Cross River	Biase	22716	2005	150.6	137.1	165.3
Nigeria	Cross River	Biase	22716	2010	127.3	115.2	141.0
Nigeria	Cross River	Biase	22716	2015	96.9	84.8	109.6
Nigeria	Cross River	Boki	22717	2000	121.5	110.1	133.7
Nigeria	Cross River	Boki	22717	2005	118.5	107.6	131.0
Nigeria	Cross River	Boki	22717	2010	103.6	92.3	115.6
Nigeria	Cross River	Boki	22717	2015	78.5	67.8	89.9
Nigeria	Cross River	Calabar Municipal	191378	2000	127.8	115.1	141.9
Nigeria	Cross River	Calabar Municipal	191378	2005	127.4	114.4	140.9
Nigeria	Cross River	Calabar Municipal	191378	2010	107.3	95.1	120.6
Nigeria	Cross River	Calabar Municipal	191378	2015	86.1	74.6	99.4
Nigeria	Cross River	Calabar South	191405	2000	136.2	123.4	149.6
Nigeria	Cross River	Calabar South	191405	2005	133.6	120.6	146.9
Nigeria	Cross River	Calabar South	191405	2010	112.3	100.7	125.0
Nigeria	Cross River	Calabar South	191405	2015	89.1	77.5	101.9
Nigeria	Cross River	Etung	191330	2000	130.4	115.8	145.3
Nigeria	Cross River	Etung	191330	2005	125.8	111.4	140.7
Nigeria	Cross River	Etung	191330	2010	109.7	96.7	124.1
Nigeria	Cross River	Etung	191330	2015	83.7	72.2	97.4
Nigeria	Cross River	Ikom	191311	2000	129.5	117.6	142.6
Nigeria	Cross River	Ikom	191311	2005	126.3	113.9	139.2
Nigeria	Cross River	Ikom	191311	2010	109.9	98.3	121.8
Nigeria	Cross River	Ikom	191311	2015	82.0	71.5	93.9
Nigeria	Cross River	Obanliku	22720	2000	111.0	98.8	123.4
Nigeria	Cross River	Obanliku	22720	2005	110.5	99.1	123.5

Admin 0	Admin 1	Admin 2	GAUL Code	Year	Under-5 mortality (per 1,000 livebirths) Estimate	Lower bound, 95% UI	Upper bound, 95% UI
Nigeria	Cross River	Obanliku	22720	2010	96.1	85.2	108.5
Nigeria	Cross River	Obanliku	22720	2015	74.0	64.2	85.6
Nigeria	Cross River	Obubra	22721	2000	153.9	142.1	166.4
Nigeria	Cross River	Obubra	22721	2005	147.2	136.2	158.9
Nigeria	Cross River	Obubra	22721	2010	127.1	115.9	139.0
Nigeria	Cross River	Obubra	22721	2015	94.4	83.2	106.9
Nigeria	Cross River	Obudu	22722	2000	115.4	103.4	128.1
Nigeria	Cross River	Obudu	22722	2005	113.0	101.4	125.4
Nigeria	Cross River	Obudu	22722	2010	98.6	87.4	111.4
Nigeria	Cross River	Obudu	22722	2015	76.3	66.0	88.6
Nigeria	Cross River	Odukpani	191357	2000	139.9	127.5	153.8
Nigeria	Cross River	Odukpani	191357	2005	138.1	126.9	150.9
Nigeria	Cross River	Odukpani	191357	2010	116.8	105.8	129.5
Nigeria	Cross River	Odukpani	191357	2015	91.8	81.2	103.7
Nigeria	Cross River	Ogoja	191286	2000	128.3	117.1	140.5
Nigeria	Cross River	Ogoja	191286	2005	123.9	112.9	136.0
Nigeria	Cross River	Ogoja	191286	2010	108.2	97.2	120.0
Nigeria	Cross River	Ogoja	191286	2015	82.3	71.6	94.4
Nigeria	Cross River	Yakurr	22725	2000	158.2	145.2	172.5
Nigeria	Cross River	Yakurr	22725	2005	152.7	139.9	165.8
Nigeria	Cross River	Yakurr	22725	2010	129.8	117.9	142.5
Nigeria	Cross River	Yakurr	22725	2015	97.1	85.4	110.2
Nigeria	Cross River	Yala	22726	2000	136.9	126.1	148.8
Nigeria	Cross River	Yala	22726	2005	127.7	117.5	139.4
Nigeria	Cross River	Yala	22726	2010	112.3	101.7	123.7
Nigeria	Cross River	Yala	22726	2015	85.7	75.4	97.5
Nigeria	Delta	Aniocha North	22727	2000	123.8	113.6	135.6
Nigeria	Delta	Aniocha North	22727	2005	114.0	104.8	124.7
Nigeria	Delta	Aniocha North	22727	2010	101.2	92.2	111.7
Nigeria	Delta	Aniocha North	22727	2015	76.1	67.3	85.4
Nigeria	Delta	Aniocha South	22728	2000	121.1	110.6	133.7
Nigeria	Delta	Aniocha South	22728	2005	110.8	101.0	121.9
Nigeria	Delta	Aniocha South	22728	2010	100.0	90.2	110.4
Nigeria	Delta	Aniocha South	22728	2015	74.3	65.6	83.8
Nigeria	Delta	Bomadi	191418	2000	162.5	149.0	177.2
Nigeria	Delta	Bomadi	191418	2005	144.9	133.7	157.5
Nigeria	Delta	Bomadi	191418	2010	127.9	117.0	139.8
Nigeria	Delta	Bomadi	191418	2015	100.6	89.2	113.7
Nigeria	Delta	Burutu	22730	2000	157.9	143.3	173.7
Nigeria	Delta	Burutu	22730	2005	140.8	128.5	155.0
Nigeria	Delta	Burutu	22730	2010	127.1	114.9	140.1
Nigeria	Delta	Burutu	22730	2015	106.0	93.2	119.9
Nigeria	Delta	Ethiope East	191420	2000	117.3	106.2	129.5
Nigeria	Delta	Ethiope East	191420	2005	102.9	93.6	112.6
Nigeria	Delta	Ethiope East	191420	2010	99.5	89.3	108.9
Nigeria	Delta	Ethiope East	191420	2015	74.5	65.1	84.5
Nigeria	Delta	Ethiope West	22732	2000	107.3	95.9	119.7
Nigeria	Delta	Ethiope West	22732	2005	98.5	89.3	108.9
Nigeria	Delta	Ethiope West	22732	2010	92.5	83.0	102.2
Nigeria	Delta	Ethiope West	22732	2015	72.1	62.5	83.1
Nigeria	Delta	Ika North East	22733	2000	116.9	105.5	129.6
Nigeria	Delta	Ika North East	22733	2005	106.8	96.6	118.5
Nigeria	Delta	Ika North East	22733	2010	98.4	87.9	109.7
Nigeria	Delta	Ika North East	22733	2015	73.6	64.2	84.3
Nigeria	Delta	Ika South	22734	2000	113.3	102.3	125.2
Nigeria	Delta	Ika South	22734	2005	103.1	93.0	114.1
Nigeria	Delta	Ika South	22734	2010	96.5	86.6	107.2
Nigeria	Delta	Ika South	22734	2015	72.0	62.6	82.0
Nigeria	Delta	Isoko North	191421	2000	128.1	116.2	140.7
Nigeria	Delta	Isoko North	191421	2005	110.8	100.9	121.3
Nigeria	Delta	Isoko North	191421	2010	106.0	96.0	116.0
Nigeria	Delta	Isoko North	191421	2015	78.2	68.4	88.6
Nigeria	Delta	Isoko South	191353	2000	135.4	123.4	148.4
Nigeria	Delta	Isoko South	191353	2005	117.6	108.1	128.0
Nigeria	Delta	Isoko South	191353	2010	110.1	99.6	121.0
Nigeria	Delta	Isoko South	191353	2015	80.8	71.4	90.8
Nigeria	Delta	Ndokwa East	22737	2000	133.7	122.8	145.8
Nigeria	Delta	Ndokwa East	22737	2005	118.6	109.0	128.4
Nigeria	Delta	Ndokwa East	22737	2010	109.0	99.2	118.5
Nigeria	Delta	Ndokwa East	22737	2015	78.5	69.9	87.9
Nigeria	Delta	Ndokwa West	191332	2000	123.1	111.4	136.3
Nigeria	Delta	Ndokwa West	191332	2005	108.3	97.9	119.2
Nigeria	Delta	Ndokwa West	191332	2010	103.6	93.1	114.5
Nigeria	Delta	Ndokwa West	191332	2015	76.2	66.9	86.9
Nigeria	Delta	Okpe	191341	2000	120.7	109.3	133.5
Nigeria	Delta	Okpe	191341	2005	107.5	98.0	117.6
Nigeria	Delta	Okpe	191341	2010	100.8	90.7	111.0
Nigeria	Delta	Okpe	191341	2015	78.6	69.0	89.1
Nigeria	Delta	Oshimili North	191301	2000	123.7	114.5	135.1
Nigeria	Delta	Oshimili North	191301	2005	114.9	105.7	125.1
Nigeria	Delta	Oshimili North	191301	2010	101.5	92.5	111.0
Nigeria	Delta	Oshimili North	191301	2015	75.2	66.5	84.7
Nigeria	Delta	Oshimili South	191318	2000	114.6	105.1	125.5
Nigeria	Delta	Oshimili South	191318	2005	108.1	99.1	118.4
Nigeria	Delta	Oshimili South	191318	2010	95.1	86.4	104.0

Admin 0	Admin 1	Admin 2	GAUL Code	Year	Under-5 mortality (per 1,000 livebirths)		
					Estimate	Lower bound, 95% UI	Upper bound, 95% UI
Nigeria	Delta	Oshimili South	191318	2015	71.1	63.0	80.0
Nigeria	Delta	Patani	191364	2000	156.9	144.3	171.3
Nigeria	Delta	Patani	191364	2005	137.6	127.0	149.0
Nigeria	Delta	Patani	191364	2010	123.5	112.0	135.9
Nigeria	Delta	Patani	191364	2015	93.0	83.0	104.2
Nigeria	Delta	Sapele	22741	2000	111.6	99.8	124.5
Nigeria	Delta	Sapele	22741	2005	102.1	92.8	113.1
Nigeria	Delta	Sapele	22741	2010	95.4	85.9	105.9
Nigeria	Delta	Sapele	22741	2015	75.6	65.7	87.1
Nigeria	Delta	Udu	191352	2000	127.2	114.8	140.7
Nigeria	Delta	Udu	191352	2005	111.6	101.8	122.1
Nigeria	Delta	Udu	191352	2010	105.4	94.7	116.5
Nigeria	Delta	Udu	191352	2015	81.5	71.0	92.6
Nigeria	Delta	Ughelli North	191343	2000	124.7	113.4	136.8
Nigeria	Delta	Ughelli North	191343	2005	108.8	99.3	118.4
Nigeria	Delta	Ughelli North	191343	2010	103.7	93.9	113.3
Nigeria	Delta	Ughelli North	191343	2015	78.2	68.7	88.3
Nigeria	Delta	Ughelli South	191350	2000	141.3	128.8	154.6
Nigeria	Delta	Ughelli South	191350	2005	124.0	113.9	135.2
Nigeria	Delta	Ughelli South	191350	2010	113.9	103.4	125.6
Nigeria	Delta	Ughelli South	191350	2015	89.1	78.4	101.1
Nigeria	Delta	Ukwuani	191334	2000	117.2	105.8	130.0
Nigeria	Delta	Ukwuani	191334	2005	103.7	93.7	114.5
Nigeria	Delta	Ukwuani	191334	2010	99.1	88.6	109.7
Nigeria	Delta	Ukwuani	191334	2015	73.3	64.0	83.7
Nigeria	Delta	Uvwie	191349	2000	113.1	101.8	125.6
Nigeria	Delta	Uvwie	191349	2005	102.2	92.9	111.8
Nigeria	Delta	Uvwie	191349	2010	97.8	87.7	108.1
Nigeria	Delta	Uvwie	191349	2015	76.1	66.2	86.8
Nigeria	Delta	Warri North	191324	2000	122.9	108.5	137.0
Nigeria	Delta	Warri North	191324	2005	114.7	102.4	127.9
Nigeria	Delta	Warri North	191324	2010	105.8	94.2	117.6
Nigeria	Delta	Warri North	191324	2015	89.2	77.6	102.1
Nigeria	Delta	Warri South	191340	2000	116.8	105.6	129.2
Nigeria	Delta	Warri South	191340	2005	106.8	97.1	116.4
Nigeria	Delta	Warri South	191340	2010	100.4	90.8	110.9
Nigeria	Delta	Warri South	191340	2015	79.9	69.9	91.0
Nigeria	Delta	Warri South-West	191339	2000	132.7	119.8	146.5
Nigeria	Delta	Warri South-West	191339	2005	120.0	109.2	131.6
Nigeria	Delta	Warri South-West	191339	2010	111.7	100.5	123.3
Nigeria	Delta	Warri South-West	191339	2015	91.4	80.3	103.9
Nigeria	Ebonyi	Abakaliki	191314	2000	163.9	153.1	174.8
Nigeria	Ebonyi	Abakaliki	191314	2005	150.9	140.8	161.4
Nigeria	Ebonyi	Abakaliki	191314	2010	130.8	119.5	142.8
Nigeria	Ebonyi	Abakaliki	191314	2015	97.5	86.7	110.1
Nigeria	Ebonyi	Afikpo North	22590	2000	177.9	164.6	192.2
Nigeria	Ebonyi	Afikpo North	22590	2005	161.9	150.0	174.7
Nigeria	Ebonyi	Afikpo North	22590	2010	133.9	122.6	146.7
Nigeria	Ebonyi	Afikpo North	22590	2015	100.7	89.1	113.4
Nigeria	Ebonyi	Afikpo South	22591	2000	180.3	167.9	194.1
Nigeria	Ebonyi	Afikpo South	22591	2005	164.4	152.6	177.0
Nigeria	Ebonyi	Afikpo South	22591	2010	134.1	123.4	146.5
Nigeria	Ebonyi	Afikpo South	22591	2015	101.1	89.6	113.9
Nigeria	Ebonyi	Ebonyi	191282	2000	172.2	159.9	184.3
Nigeria	Ebonyi	Ebonyi	191282	2005	156.9	145.5	168.4
Nigeria	Ebonyi	Ebonyi	191282	2010	134.5	123.1	146.7
Nigeria	Ebonyi	Ebonyi	191282	2015	100.6	89.4	113.5
Nigeria	Ebonyi	Ezza North	191312	2000	185.6	172.5	198.9
Nigeria	Ebonyi	Ezza North	191312	2005	166.6	155.2	179.2
Nigeria	Ebonyi	Ezza North	191312	2010	139.4	128.2	151.9
Nigeria	Ebonyi	Ezza North	191312	2015	106.4	94.0	119.4
Nigeria	Ebonyi	Ezza South	191320	2000	183.4	171.3	197.0
Nigeria	Ebonyi	Ezza South	191320	2005	166.0	154.8	178.2
Nigeria	Ebonyi	Ezza South	191320	2010	139.3	127.8	151.8
Nigeria	Ebonyi	Ezza South	191320	2015	104.6	92.4	117.3
Nigeria	Ebonyi	Ikwo	22765	2000	178.1	165.8	190.6
Nigeria	Ebonyi	Ikwo	22765	2005	162.6	151.9	174.6
Nigeria	Ebonyi	Ikwo	22765	2010	138.0	126.5	150.8
Nigeria	Ebonyi	Ikwo	22765	2015	103.6	91.4	116.8
Nigeria	Ebonyi	Ishielu	22766	2000	186.4	173.0	200.7
Nigeria	Ebonyi	Ishielu	22766	2005	167.0	155.1	179.9
Nigeria	Ebonyi	Ishielu	22766	2010	139.3	128.5	151.2
Nigeria	Ebonyi	Ishielu	22766	2015	106.6	94.2	119.4
Nigeria	Ebonyi	Ivo	191333	2000	182.6	168.8	197.6
Nigeria	Ebonyi	Ivo	191333	2005	162.8	150.2	176.3
Nigeria	Ebonyi	Ivo	191333	2010	132.9	121.4	145.6
Nigeria	Ebonyi	Ivo	191333	2015	99.0	87.8	111.4
Nigeria	Ebonyi	Izzi	22768	2000	172.3	158.7	186.1
Nigeria	Ebonyi	Izzi	22768	2005	155.3	143.6	168.8
Nigeria	Ebonyi	Izzi	22768	2010	133.6	121.8	147.3
Nigeria	Ebonyi	Izzi	22768	2015	103.5	91.8	117.1
Nigeria	Ebonyi	Ohaozara	191327	2000	188.7	175.9	202.7
Nigeria	Ebonyi	Ohaozara	191327	2005	169.0	157.3	181.8
Nigeria	Ebonyi	Ohaozara	191327	2010	138.2	127.4	151.4
Nigeria	Ebonyi	Ohaozara	191327	2015	104.2	92.4	117.1

Admin 0	Admin 1	Admin 2	GAUL Code	Year	Under-5 mortality (per 1,000 livebirths)		
					Estimate	Lower bound, 95% UI	Upper bound, 95% UI
Nigeria	Ebonyi	Ohaukwu	22771	2000	181.6	167.5	195.5
Nigeria	Ebonyi	Ohaukwu	22771	2005	163.0	150.2	176.6
Nigeria	Ebonyi	Ohaukwu	22771	2010	137.8	125.9	150.0
Nigeria	Ebonyi	Ohaukwu	22771	2015	104.5	92.0	117.4
Nigeria	Ebonyi	Onicha	22600	2000	191.0	178.5	204.2
Nigeria	Ebonyi	Onicha	22600	2005	170.7	159.1	182.4
Nigeria	Ebonyi	Onicha	22600	2010	139.6	128.6	151.8
Nigeria	Ebonyi	Onicha	22600	2015	105.9	94.4	118.0
Nigeria	Edo	Akoko-Edo	22746	2000	112.1	102.3	123.5
Nigeria	Edo	Akoko-Edo	22746	2005	103.1	93.4	114.0
Nigeria	Edo	Akoko-Edo	22746	2010	89.0	80.0	98.8
Nigeria	Edo	Akoko-Edo	22746	2015	66.9	58.8	77.0
Nigeria	Edo	Egor	191308	2000	95.3	85.9	106.0
Nigeria	Edo	Egor	191308	2005	89.6	80.9	99.4
Nigeria	Edo	Egor	191308	2010	84.6	76.1	94.5
Nigeria	Edo	Egor	191308	2015	64.6	56.5	74.2
Nigeria	Edo	Esan Central	191279	2000	118.8	107.2	131.9
Nigeria	Edo	Esan Central	191279	2005	111.1	99.9	123.4
Nigeria	Edo	Esan Central	191279	2010	99.0	88.9	110.4
Nigeria	Edo	Esan Central	191279	2015	73.8	64.3	84.5
Nigeria	Edo	Esan North-East	22748	2000	116.1	104.2	130.0
Nigeria	Edo	Esan North-East	22748	2005	108.2	96.7	120.5
Nigeria	Edo	Esan North-East	22748	2010	98.0	87.7	110.2
Nigeria	Edo	Esan North-East	22748	2015	73.6	64.0	84.6
Nigeria	Edo	Esan South-East	22749	2000	124.6	112.5	137.6
Nigeria	Edo	Esan South-East	22749	2005	114.6	103.6	125.7
Nigeria	Edo	Esan South-East	22749	2010	102.1	91.8	112.9
Nigeria	Edo	Esan South-East	22749	2015	77.0	67.5	87.4
Nigeria	Edo	Esan West	22750	2000	111.0	100.9	122.4
Nigeria	Edo	Esan West	22750	2005	104.1	94.2	115.1
Nigeria	Edo	Esan West	22750	2010	94.2	85.0	104.4
Nigeria	Edo	Esan West	22750	2015	70.7	61.8	80.3
Nigeria	Edo	Etsako Central	191257	2000	124.2	112.7	136.3
Nigeria	Edo	Etsako Central	191257	2005	114.9	104.4	125.8
Nigeria	Edo	Etsako Central	191257	2010	101.3	91.5	112.1
Nigeria	Edo	Etsako Central	191257	2015	75.9	67.0	86.3
Nigeria	Edo	Etsako East	22751	2000	115.6	104.8	127.9
Nigeria	Edo	Etsako East	22751	2005	109.1	99.2	120.3
Nigeria	Edo	Etsako East	22751	2010	96.5	87.0	107.4
Nigeria	Edo	Etsako East	22751	2015	72.0	63.4	82.2
Nigeria	Edo	Etsako West	191255	2000	115.3	103.7	127.5
Nigeria	Edo	Etsako West	191255	2005	107.7	97.6	119.3
Nigeria	Edo	Etsako West	191255	2010	95.5	86.4	106.4
Nigeria	Edo	Etsako West	191255	2015	71.2	62.3	81.7
Nigeria	Edo	Igueben	191289	2000	116.4	105.5	128.7
Nigeria	Edo	Igueben	191289	2005	108.0	97.8	119.0
Nigeria	Edo	Igueben	191289	2010	97.7	87.4	108.0
Nigeria	Edo	Igueben	191289	2015	73.2	63.7	82.9
Nigeria	Edo	Ikpoba-Okha	191309	2000	95.8	86.2	105.9
Nigeria	Edo	Ikpoba-Okha	191309	2005	90.5	82.0	99.6
Nigeria	Edo	Ikpoba-Okha	191309	2010	85.2	76.3	94.8
Nigeria	Edo	Ikpoba-Okha	191309	2015	65.4	57.3	75.1
Nigeria	Edo	Oredo	191313	2000	107.4	96.6	119.1
Nigeria	Edo	Oredo	191313	2005	99.4	90.2	109.9
Nigeria	Edo	Oredo	191313	2010	89.9	81.1	100.1
Nigeria	Edo	Oredo	191313	2015	70.3	61.6	80.7
Nigeria	Edo	Orhionmwon	191310	2000	116.3	106.2	128.1
Nigeria	Edo	Orhionmwon	191310	2005	106.4	97.2	116.1
Nigeria	Edo	Orhionmwon	191310	2010	98.0	88.6	107.1
Nigeria	Edo	Orhionmwon	191310	2015	73.7	64.8	83.3
Nigeria	Edo	Ovia North-East	191277	2000	115.9	105.4	126.9
Nigeria	Edo	Ovia North-East	191277	2005	107.5	98.1	118.1
Nigeria	Edo	Ovia North-East	191277	2010	95.9	87.1	106.1
Nigeria	Edo	Ovia North-East	191277	2015	74.8	65.8	85.1
Nigeria	Edo	Ovia South-West	191276	2000	122.8	110.9	134.8
Nigeria	Edo	Ovia South-West	191276	2005	113.1	102.9	124.7
Nigeria	Edo	Ovia South-West	191276	2010	101.4	91.7	112.2
Nigeria	Edo	Ovia South-West	191276	2015	83.3	73.2	94.8
Nigeria	Edo	Owan East	191252	2000	118.0	107.3	130.2
Nigeria	Edo	Owan East	191252	2005	108.4	98.4	119.8
Nigeria	Edo	Owan East	191252	2010	94.4	85.1	105.0
Nigeria	Edo	Owan East	191252	2015	70.0	61.4	80.7
Nigeria	Edo	Owan West	191263	2000	117.1	105.2	130.3
Nigeria	Edo	Owan West	191263	2005	108.2	96.8	121.0
Nigeria	Edo	Owan West	191263	2010	94.6	84.6	106.2
Nigeria	Edo	Owan West	191263	2015	69.8	60.6	80.2
Nigeria	Edo	Uhunmwonde	191281	2000	107.9	98.1	119.0
Nigeria	Edo	Uhunmwonde	191281	2005	99.9	90.8	109.9
Nigeria	Edo	Uhunmwonde	191281	2010	91.3	82.1	100.7
Nigeria	Edo	Uhunmwonde	191281	2015	69.1	61.0	78.6
Nigeria	Ekiti	Ado-Ekiti	22967	2000	95.0	86.1	105.0
Nigeria	Ekiti	Ado-Ekiti	22967	2005	92.5	83.8	102.1
Nigeria	Ekiti	Ado-Ekiti	22967	2010	78.7	70.5	87.7
Nigeria	Ekiti	Ado-Ekiti	22967	2015	61.2	53.3	69.7
Nigeria	Ekiti	Efon	191202	2000	93.6	85.6	102.5

Admin 0	Admin 1	Admin 2	GAUL Code	Year	Under-5 mortality (per 1,000 livebirths)		
					Estimate	Lower bound, 95% UI	Upper bound, 95% UI
Nigeria	Ekiti	Efon	191202	2005	90.5	82.6	98.9
Nigeria	Ekiti	Efon	191202	2010	77.2	69.7	85.6
Nigeria	Ekiti	Efon	191202	2015	59.4	52.6	67.5
Nigeria	Ekiti	Ekiti East	191200	2000	98.3	88.8	109.8
Nigeria	Ekiti	Ekiti East	191200	2005	94.0	84.7	104.0
Nigeria	Ekiti	Ekiti East	191200	2010	81.0	71.9	90.4
Nigeria	Ekiti	Ekiti East	191200	2015	61.6	53.7	70.7
Nigeria	Ekiti	Ekiti South-West	22973	2000	99.4	90.8	108.9
Nigeria	Ekiti	Ekiti South-West	22973	2005	96.1	87.8	104.8
Nigeria	Ekiti	Ekiti South-West	22973	2010	81.2	73.3	89.4
Nigeria	Ekiti	Ekiti South-West	22973	2015	62.2	54.8	70.6
Nigeria	Ekiti	Ekiti West	191199	2000	94.5	86.4	103.1
Nigeria	Ekiti	Ekiti West	191199	2005	91.2	83.5	99.3
Nigeria	Ekiti	Ekiti West	191199	2010	78.0	70.7	86.0
Nigeria	Ekiti	Ekiti West	191199	2015	60.0	53.3	68.5
Nigeria	Ekiti	Emure	191224	2000	105.7	96.7	115.8
Nigeria	Ekiti	Emure	191224	2005	101.1	92.2	110.9
Nigeria	Ekiti	Emure	191224	2010	85.8	77.0	94.3
Nigeria	Ekiti	Emure	191224	2015	64.7	57.0	74.1
Nigeria	Ekiti	Gboyin	191209	2000	103.9	94.8	114.3
Nigeria	Ekiti	Gboyin	191209	2005	99.3	90.6	108.4
Nigeria	Ekiti	Gboyin	191209	2010	84.0	75.6	92.5
Nigeria	Ekiti	Gboyin	191209	2015	63.5	55.8	72.3
Nigeria	Ekiti	Ido-Osi	22977	2000	93.6	84.7	103.2
Nigeria	Ekiti	Ido-Osi	22977	2005	90.2	81.5	99.6
Nigeria	Ekiti	Ido-Osi	22977	2010	77.2	68.9	85.7
Nigeria	Ekiti	Ido-Osi	22977	2015	59.6	52.3	68.1
Nigeria	Ekiti	Ijero	22979	2000	91.6	82.7	101.0
Nigeria	Ekiti	Ijero	22979	2005	87.2	78.9	95.9
Nigeria	Ekiti	Ijero	22979	2010	74.9	67.2	83.3
Nigeria	Ekiti	Ijero	22979	2015	57.9	50.9	66.4
Nigeria	Ekiti	Ikere	22981	2000	98.0	89.2	107.9
Nigeria	Ekiti	Ikere	22981	2005	95.7	87.2	105.4
Nigeria	Ekiti	Ikere	22981	2010	80.7	72.7	89.1
Nigeria	Ekiti	Ikere	22981	2015	62.4	54.4	71.1
Nigeria	Ekiti	Ikole	22982	2000	98.2	89.2	109.3
Nigeria	Ekiti	Ikole	22982	2005	94.4	85.3	104.2
Nigeria	Ekiti	Ikole	22982	2010	80.9	72.2	89.8
Nigeria	Ekiti	Ikole	22982	2015	62.3	54.3	71.4
Nigeria	Ekiti	Ilejemeje	191194	2000	92.2	83.1	102.5
Nigeria	Ekiti	Ilejemeje	191194	2005	88.8	79.6	98.6
Nigeria	Ekiti	Ilejemeje	191194	2010	76.1	67.5	85.1
Nigeria	Ekiti	Ilejemeje	191194	2015	59.4	52.0	68.2
Nigeria	Ekiti	Irepodun/Ifelodun	191203	2000	97.8	89.0	107.0
Nigeria	Ekiti	Irepodun/Ifelodun	191203	2005	94.5	86.0	103.4
Nigeria	Ekiti	Irepodun/Ifelodun	191203	2010	79.6	71.5	88.6
Nigeria	Ekiti	Irepodun/Ifelodun	191203	2015	61.2	53.9	69.3
Nigeria	Ekiti	Ise /Orun	191413	2000	105.4	96.9	115.5
Nigeria	Ekiti	Ise /Orun	191413	2005	101.0	92.2	110.4
Nigeria	Ekiti	Ise /Orun	191413	2010	85.4	76.9	93.8
Nigeria	Ekiti	Ise /Orun	191413	2015	64.3	56.4	73.1
Nigeria	Ekiti	Moba	191191	2000	89.1	79.5	99.5
Nigeria	Ekiti	Moba	191191	2005	85.8	76.5	95.8
Nigeria	Ekiti	Moba	191191	2010	75.0	66.3	84.3
Nigeria	Ekiti	Moba	191191	2015	59.1	51.5	68.2
Nigeria	Ekiti	Oye	22991	2000	95.5	86.8	105.7
Nigeria	Ekiti	Oye	22991	2005	91.9	83.0	101.0
Nigeria	Ekiti	Oye	22991	2010	78.6	70.1	87.0
Nigeria	Ekiti	Oye	22991	2015	61.0	53.6	69.3
Nigeria	Enugu	Aninri	191322	2000	164.3	151.9	177.5
Nigeria	Enugu	Aninri	191322	2005	155.5	143.8	167.9
Nigeria	Enugu	Aninri	191322	2010	132.0	121.5	144.6
Nigeria	Enugu	Aninri	191322	2015	95.5	84.5	107.2
Nigeria	Enugu	Awgu	191315	2000	151.5	139.5	163.6
Nigeria	Enugu	Awgu	191315	2005	144.1	133.1	155.8
Nigeria	Enugu	Awgu	191315	2010	124.3	113.9	136.1
Nigeria	Enugu	Awgu	191315	2015	89.0	78.4	100.5
Nigeria	Enugu	Enugu East	191292	2000	126.0	116.2	136.4
Nigeria	Enugu	Enugu East	191292	2005	128.8	118.1	139.5
Nigeria	Enugu	Enugu East	191292	2010	119.2	109.4	129.9
Nigeria	Enugu	Enugu East	191292	2015	85.4	75.5	96.0
Nigeria	Enugu	Enugu North	191302	2000	121.2	111.8	131.2
Nigeria	Enugu	Enugu North	191302	2005	126.9	116.4	137.1
Nigeria	Enugu	Enugu North	191302	2010	117.8	107.7	128.4
Nigeria	Enugu	Enugu North	191302	2015	84.4	74.4	95.3
Nigeria	Enugu	Enugu South	191306	2000	130.1	119.4	140.7
Nigeria	Enugu	Enugu South	191306	2005	131.6	120.4	143.1
Nigeria	Enugu	Enugu South	191306	2010	120.7	109.7	131.6
Nigeria	Enugu	Enugu South	191306	2015	85.6	75.1	96.8
Nigeria	Enugu	Ezeagu	22761	2000	134.2	123.7	145.6
Nigeria	Enugu	Ezeagu	22761	2005	130.6	120.3	141.0
Nigeria	Enugu	Ezeagu	22761	2010	116.3	106.5	126.9
Nigeria	Enugu	Ezeagu	22761	2015	83.0	73.4	93.9
Nigeria	Enugu	Igbo-Etiti	22763	2000	125.5	114.7	137.6
Nigeria	Enugu	Igbo-Etiti	22763	2005	124.6	113.7	136.2

Admin 0	Admin 1	Admin 2	GAUL Code	Year	Under-5 mortality (per 1,000 livebirths)		
					Estimate	Lower bound, 95% UI	Upper bound, 95% UI
Nigeria	Enugu	Igbo-Etiti	22763	2010	115.9	105.3	127.7
Nigeria	Enugu	Igbo-Etiti	22763	2015	82.9	72.7	95.4
Nigeria	Enugu	Igbo-eze North	191258	2000	128.6	116.7	141.4
Nigeria	Enugu	Igbo-eze North	191258	2005	119.4	108.9	131.4
Nigeria	Enugu	Igbo-eze North	191258	2010	109.5	98.6	121.3
Nigeria	Enugu	Igbo-eze North	191258	2015	80.3	70.5	91.3
Nigeria	Enugu	Igbo-eze South	191264	2000	126.8	115.4	139.4
Nigeria	Enugu	Igbo-eze South	191264	2005	118.6	108.0	130.1
Nigeria	Enugu	Igbo-eze South	191264	2010	109.7	98.9	121.6
Nigeria	Enugu	Igbo-eze South	191264	2015	81.0	71.0	92.2
Nigeria	Enugu	Isi-Uzo	191275	2000	152.8	140.3	166.0
Nigeria	Enugu	Isi-Uzo	191275	2005	140.1	128.0	153.2
Nigeria	Enugu	Isi-Uzo	191275	2010	125.6	114.2	138.1
Nigeria	Enugu	Isi-Uzo	191275	2015	91.8	80.5	103.0
Nigeria	Enugu	Nkanu East	191294	2000	158.2	146.4	169.7
Nigeria	Enugu	Nkanu East	191294	2005	150.4	139.2	161.6
Nigeria	Enugu	Nkanu East	191294	2010	130.7	119.9	142.4
Nigeria	Enugu	Nkanu East	191294	2015	94.2	83.4	106.0
Nigeria	Enugu	Nkanu West	191307	2000	144.3	132.3	155.9
Nigeria	Enugu	Nkanu West	191307	2005	141.3	129.4	153.4
Nigeria	Enugu	Nkanu West	191307	2010	125.5	113.7	137.5
Nigeria	Enugu	Nkanu West	191307	2015	89.7	78.5	101.5
Nigeria	Enugu	Nsukka	22770	2000	116.0	105.9	127.7
Nigeria	Enugu	Nsukka	22770	2005	115.5	105.3	126.4
Nigeria	Enugu	Nsukka	22770	2010	109.4	99.1	121.0
Nigeria	Enugu	Nsukka	22770	2015	79.0	69.1	90.5
Nigeria	Enugu	Oji-River	22772	2000	138.9	128.9	149.5
Nigeria	Enugu	Oji-River	22772	2005	132.5	122.8	142.5
Nigeria	Enugu	Oji-River	22772	2010	115.2	106.6	125.4
Nigeria	Enugu	Oji-River	22772	2015	82.2	72.4	92.0
Nigeria	Enugu	Udenu	191270	2000	140.5	128.7	153.5
Nigeria	Enugu	Udenu	191270	2005	129.0	118.1	141.2
Nigeria	Enugu	Udenu	191270	2010	116.8	106.0	128.5
Nigeria	Enugu	Udenu	191270	2015	86.2	76.0	97.7
Nigeria	Enugu	Udi	22773	2000	128.9	118.8	140.1
Nigeria	Enugu	Udi	22773	2005	129.1	118.6	139.5
Nigeria	Enugu	Udi	22773	2010	118.0	108.2	128.8
Nigeria	Enugu	Udi	22773	2015	83.4	73.5	94.3
Nigeria	Enugu	Uzo-Uwani	22774	2000	132.7	121.4	147.1
Nigeria	Enugu	Uzo-Uwani	22774	2005	125.1	113.7	137.9
Nigeria	Enugu	Uzo-Uwani	22774	2010	112.6	101.7	125.0
Nigeria	Enugu	Uzo-Uwani	22774	2015	82.8	72.8	94.4
Nigeria	Gombe	Akko	22658	2000	246.1	229.7	263.5
Nigeria	Gombe	Akko	22658	2005	208.8	194.2	223.7
Nigeria	Gombe	Akko	22658	2010	170.6	157.1	184.4
Nigeria	Gombe	Akko	22658	2015	152.6	136.4	170.8
Nigeria	Gombe	Balanga	22660	2000	266.1	246.1	287.1
Nigeria	Gombe	Balanga	22660	2005	229.6	213.0	247.5
Nigeria	Gombe	Balanga	22660	2010	181.7	166.8	199.4
Nigeria	Gombe	Balanga	22660	2015	150.1	133.1	167.7
Nigeria	Gombe	Billiri	22662	2000	244.3	225.3	263.3
Nigeria	Gombe	Billiri	22662	2005	209.2	192.7	226.8
Nigeria	Gombe	Billiri	22662	2010	171.1	156.7	187.0
Nigeria	Gombe	Billiri	22662	2015	147.0	131.1	164.5
Nigeria	Gombe	Dukku	22665	2000	267.1	245.5	290.2
Nigeria	Gombe	Dukku	22665	2005	222.0	204.2	240.9
Nigeria	Gombe	Dukku	22665	2010	190.2	173.3	208.9
Nigeria	Gombe	Dukku	22665	2015	172.5	153.3	192.2
Nigeria	Gombe	Funakaye	191090	2000	233.2	215.1	254.3
Nigeria	Gombe	Funakaye	191090	2005	196.0	179.9	213.3
Nigeria	Gombe	Funakaye	191090	2010	170.0	154.5	186.8
Nigeria	Gombe	Funakaye	191090	2015	154.3	136.0	174.8
Nigeria	Gombe	Gombe	191103	2000	250.4	232.4	269.0
Nigeria	Gombe	Gombe	191103	2005	210.4	196.5	226.4
Nigeria	Gombe	Gombe	191103	2010	168.7	155.3	183.0
Nigeria	Gombe	Gombe	191103	2015	151.5	135.0	169.6
Nigeria	Gombe	Kaltungo	191110	2000	251.7	234.2	270.4
Nigeria	Gombe	Kaltungo	191110	2005	215.8	199.9	232.8
Nigeria	Gombe	Kaltungo	191110	2010	174.4	160.2	190.1
Nigeria	Gombe	Kaltungo	191110	2015	146.7	131.2	163.7
Nigeria	Gombe	Kwami	191094	2000	264.1	246.2	285.2
Nigeria	Gombe	Kwami	191094	2005	218.9	203.9	235.1
Nigeria	Gombe	Kwami	191094	2010	184.4	169.7	199.9
Nigeria	Gombe	Kwami	191094	2015	171.6	152.0	191.8
Nigeria	Gombe	Nafada	191082	2000	244.8	225.7	263.2
Nigeria	Gombe	Nafada	191082	2005	204.9	187.6	221.1
Nigeria	Gombe	Nafada	191082	2010	181.3	165.4	198.5
Nigeria	Gombe	Nafada	191082	2015	159.5	141.4	180.2
Nigeria	Gombe	Shomgom	191115	2000	228.1	211.1	245.8
Nigeria	Gombe	Shomgom	191115	2005	199.2	184.2	217.3
Nigeria	Gombe	Shomgom	191115	2010	161.0	147.0	175.9
Nigeria	Gombe	Shomgom	191115	2015	138.4	123.7	154.7
Nigeria	Gombe	Yamaltu/Deba	22679	2000	267.2	247.4	288.6
Nigeria	Gombe	Yamaltu/Deba	22679	2005	224.4	209.5	241.6
Nigeria	Gombe	Yamaltu/Deba	22679	2010	184.8	169.5	200.3

Admin 0	Admin 1	Admin 2	GAUL Code	Year	Under-5 mortality (per 1,000 livebirths)		
					Estimate	Lower bound, 95% UI	Upper bound, 95% UI
Nigeria	Gombe	Yamaltu/Deba	22679	2015	162.6	144.1	184.1
Nigeria	Imo	Aboh-Mbaise	22779	2000	141.8	131.4	153.3
Nigeria	Imo	Aboh-Mbaise	22779	2005	137.6	127.6	149.0
Nigeria	Imo	Aboh-Mbaise	22779	2010	114.0	105.0	124.6
Nigeria	Imo	Aboh-Mbaise	22779	2015	81.4	72.4	92.1
Nigeria	Imo	Ahiazu-Mbaise	22780	2000	139.2	128.4	151.2
Nigeria	Imo	Ahiazu-Mbaise	22780	2005	134.6	123.5	146.7
Nigeria	Imo	Ahiazu-Mbaise	22780	2010	112.5	102.9	123.1
Nigeria	Imo	Ahiazu-Mbaise	22780	2015	81.2	72.1	91.8
Nigeria	Imo	Ehime-Mbano	22781	2000	139.9	129.2	151.8
Nigeria	Imo	Ehime-Mbano	22781	2005	134.3	123.4	146.3
Nigeria	Imo	Ehime-Mbano	22781	2010	111.8	102.1	122.7
Nigeria	Imo	Ehime-Mbano	22781	2015	81.3	72.2	91.7
Nigeria	Imo	Ezinihitte Mbaise	22782	2000	141.8	131.4	153.2
Nigeria	Imo	Ezinihitte Mbaise	22782	2005	137.4	127.1	148.7
Nigeria	Imo	Ezinihitte Mbaise	22782	2010	113.4	104.6	123.6
Nigeria	Imo	Ezinihitte Mbaise	22782	2015	81.8	72.5	92.3
Nigeria	Imo	Ideato North	22784	2000	138.8	128.1	150.6
Nigeria	Imo	Ideato North	22784	2005	129.6	120.1	140.1
Nigeria	Imo	Ideato North	22784	2010	109.4	99.9	118.6
Nigeria	Imo	Ideato North	22784	2015	79.7	70.7	89.5
Nigeria	Imo	Ideato South	22783	2000	140.2	129.8	152.8
Nigeria	Imo	Ideato South	22783	2005	132.4	122.4	143.5
Nigeria	Imo	Ideato South	22783	2010	109.9	100.5	119.5
Nigeria	Imo	Ideato South	22783	2015	80.3	71.1	90.3
Nigeria	Imo	Ihitte/Uboma	22785	2000	141.6	131.2	153.4
Nigeria	Imo	Ihitte/Uboma	22785	2005	135.8	125.2	147.7
Nigeria	Imo	Ihitte/Uboma	22785	2010	112.2	102.4	123.1
Nigeria	Imo	Ihitte/Uboma	22785	2015	81.7	72.7	92.4
Nigeria	Imo	Ikeduru	22786	2000	140.5	129.9	153.0
Nigeria	Imo	Ikeduru	22786	2005	136.0	125.6	147.5
Nigeria	Imo	Ikeduru	22786	2010	114.1	104.7	124.4
Nigeria	Imo	Ikeduru	22786	2015	81.1	72.0	91.4
Nigeria	Imo	Isiala Mbano	22787	2000	139.7	129.4	152.0
Nigeria	Imo	Isiala Mbano	22787	2005	133.8	123.6	145.4
Nigeria	Imo	Isiala Mbano	22787	2010	112.3	103.1	122.4
Nigeria	Imo	Isiala Mbano	22787	2015	80.6	71.8	90.8
Nigeria	Imo	Isu	191347	2000	140.5	130.2	152.4
Nigeria	Imo	Isu	191347	2005	133.5	123.5	144.3
Nigeria	Imo	Isu	191347	2010	112.5	103.3	122.8
Nigeria	Imo	Isu	191347	2015	80.7	71.4	90.7
Nigeria	Imo	Mbatoli	22789	2000	141.4	130.5	153.8
Nigeria	Imo	Mbatoli	22789	2005	135.9	125.4	147.2
Nigeria	Imo	Mbatoli	22789	2010	114.6	105.1	125.1
Nigeria	Imo	Mbatoli	22789	2015	81.4	72.0	91.9
Nigeria	Imo	Ngor-Okpala	22790	2000	145.5	134.7	156.8
Nigeria	Imo	Ngor-Okpala	22790	2005	142.7	132.3	154.4
Nigeria	Imo	Ngor-Okpala	22790	2010	116.2	107.2	126.7
Nigeria	Imo	Ngor-Okpala	22790	2015	82.5	73.2	92.9
Nigeria	Imo	Njaba	191345	2000	139.9	129.3	151.9
Nigeria	Imo	Njaba	191345	2005	132.5	122.4	143.4
Nigeria	Imo	Njaba	191345	2010	111.2	101.8	121.4
Nigeria	Imo	Njaba	191345	2015	80.6	71.2	90.5
Nigeria	Imo	Nkwerre	191344	2000	138.5	128.6	150.8
Nigeria	Imo	Nkwerre	191344	2005	131.8	121.9	143.1
Nigeria	Imo	Nkwerre	191344	2010	110.3	100.8	120.0
Nigeria	Imo	Nkwerre	191344	2015	80.5	71.4	90.3
Nigeria	Imo	Nwangele	191346	2000	139.1	129.5	151.6
Nigeria	Imo	Nwangele	191346	2005	132.2	122.4	143.5
Nigeria	Imo	Nwangele	191346	2010	110.9	101.9	120.8
Nigeria	Imo	Nwangele	191346	2015	80.2	71.3	90.1
Nigeria	Imo	Obowo	22792	2000	140.5	129.5	152.0
Nigeria	Imo	Obowo	22792	2005	136.1	125.5	148.0
Nigeria	Imo	Obowo	22792	2010	112.5	103.0	123.2
Nigeria	Imo	Obowo	22792	2015	81.6	72.5	92.4
Nigeria	Imo	Oguta	22793	2000	138.2	125.9	151.3
Nigeria	Imo	Oguta	22793	2005	130.2	119.3	141.9
Nigeria	Imo	Oguta	22793	2010	111.1	101.1	122.2
Nigeria	Imo	Oguta	22793	2015	78.8	69.2	88.7
Nigeria	Imo	Ohaji /Egbema	22794	2000	143.3	131.3	155.8
Nigeria	Imo	Ohaji /Egbema	22794	2005	134.9	124.8	145.7
Nigeria	Imo	Ohaji /Egbema	22794	2010	113.6	103.7	123.7
Nigeria	Imo	Ohaji /Egbema	22794	2015	79.2	70.1	89.3
Nigeria	Imo	Okigwe	191335	2000	145.7	135.4	156.6
Nigeria	Imo	Okigwe	191335	2005	137.4	127.4	148.2
Nigeria	Imo	Okigwe	191335	2010	114.5	105.3	124.2
Nigeria	Imo	Okigwe	191335	2015	83.3	74.1	93.2
Nigeria	Imo	Orlu	22796	2000	137.7	127.2	149.0
Nigeria	Imo	Orlu	22796	2005	128.6	119.0	139.4
Nigeria	Imo	Orlu	22796	2010	107.9	98.7	117.5
Nigeria	Imo	Orlu	22796	2015	78.9	69.8	88.8
Nigeria	Imo	Orsu	22797	2000	135.4	125.2	146.3
Nigeria	Imo	Orsu	22797	2005	125.2	116.7	135.2
Nigeria	Imo	Orsu	22797	2010	106.0	97.5	115.1
Nigeria	Imo	Orsu	22797	2015	77.4	68.8	86.4

Admin 0	Admin 1	Admin 2	GAUL Code	Year	Under-5 mortality (per 1,000 livebirths)		
					Estimate	Lower bound, 95% UI	Upper bound, 95% UI
Nigeria	Imo	Oru East	191342	2000	140.8	129.2	152.9
Nigeria	Imo	Oru East	191342	2005	133.0	122.8	144.3
Nigeria	Imo	Oru East	191342	2010	111.8	101.9	122.5
Nigeria	Imo	Oru East	191342	2015	80.3	70.9	90.5
Nigeria	Imo	Oru West	191338	2000	136.5	125.4	148.3
Nigeria	Imo	Oru West	191338	2005	127.3	117.7	137.7
Nigeria	Imo	Oru West	191338	2010	107.4	98.3	117.5
Nigeria	Imo	Oru West	191338	2015	77.4	68.5	87.0
Nigeria	Imo	Owerri Municipal	191356	2000	138.5	127.7	150.5
Nigeria	Imo	Owerri Municipal	191356	2005	133.2	122.9	144.7
Nigeria	Imo	Owerri Municipal	191356	2010	111.4	101.9	121.6
Nigeria	Imo	Owerri Municipal	191356	2015	79.2	70.0	89.5
Nigeria	Imo	Owerri North	191354	2000	141.2	130.2	153.1
Nigeria	Imo	Owerri North	191354	2005	136.8	126.4	149.1
Nigeria	Imo	Owerri North	191354	2010	114.3	105.1	124.7
Nigeria	Imo	Owerri North	191354	2015	80.9	71.7	91.6
Nigeria	Imo	Owerri West	191351	2000	142.6	131.6	155.4
Nigeria	Imo	Owerri West	191351	2005	137.3	126.7	149.0
Nigeria	Imo	Owerri West	191351	2010	114.7	104.7	125.4
Nigeria	Imo	Owerri West	191351	2015	81.2	71.6	91.6
Nigeria	Imo	Unuimo	191337	2000	142.6	132.6	154.3
Nigeria	Imo	Unuimo	191337	2005	135.5	125.6	146.8
Nigeria	Imo	Unuimo	191337	2010	112.9	103.7	122.8
Nigeria	Imo	Unuimo	191337	2015	82.1	73.0	92.3
Nigeria	Jigawa	Auyo	191007	2000	281.9	263.0	301.0
Nigeria	Jigawa	Auyo	191007	2005	250.4	232.6	268.6
Nigeria	Jigawa	Auyo	191007	2010	203.3	188.2	220.5
Nigeria	Jigawa	Auyo	191007	2015	173.7	155.1	196.4
Nigeria	Jigawa	Babura	22800	2000	285.3	265.5	307.0
Nigeria	Jigawa	Babura	22800	2005	252.9	235.4	271.8
Nigeria	Jigawa	Babura	22800	2010	210.4	193.7	228.1
Nigeria	Jigawa	Babura	22800	2015	184.9	164.8	207.1
Nigeria	Jigawa	Biriniwa	22801	2000	259.7	241.4	279.1
Nigeria	Jigawa	Biriniwa	22801	2005	232.0	214.6	250.8
Nigeria	Jigawa	Biriniwa	22801	2010	193.5	177.4	211.8
Nigeria	Jigawa	Biriniwa	22801	2015	168.0	149.2	191.1
Nigeria	Jigawa	Birnin Kudu	191060	2000	310.9	289.5	332.9
Nigeria	Jigawa	Birnin Kudu	191060	2005	262.1	243.9	281.7
Nigeria	Jigawa	Birnin Kudu	191060	2010	227.4	209.4	246.7
Nigeria	Jigawa	Birnin Kudu	191060	2015	201.4	179.6	225.6
Nigeria	Jigawa	Buji	191056	2000	318.5	296.6	340.2
Nigeria	Jigawa	Buji	191056	2005	272.2	254.5	291.8
Nigeria	Jigawa	Buji	191056	2010	235.9	217.1	255.3
Nigeria	Jigawa	Buji	191056	2015	202.1	180.8	225.5
Nigeria	Jigawa	Dutse	22803	2000	311.6	291.2	331.8
Nigeria	Jigawa	Dutse	22803	2005	262.7	244.5	281.5
Nigeria	Jigawa	Dutse	22803	2010	215.6	198.9	232.9
Nigeria	Jigawa	Dutse	22803	2015	197.0	176.3	220.9
Nigeria	Jigawa	Gagarawa	190996	2000	314.0	293.6	334.8
Nigeria	Jigawa	Gagarawa	190996	2005	279.4	260.5	299.5
Nigeria	Jigawa	Gagarawa	190996	2010	233.6	216.3	251.9
Nigeria	Jigawa	Gagarawa	190996	2015	200.7	180.5	225.1
Nigeria	Jigawa	Garki	22804	2000	304.3	285.7	324.6
Nigeria	Jigawa	Garki	22804	2005	266.1	249.6	284.4
Nigeria	Jigawa	Garki	22804	2010	218.4	203.4	236.1
Nigeria	Jigawa	Garki	22804	2015	195.0	174.8	216.3
Nigeria	Jigawa	Gumel	190991	2000	290.3	262.5	319.4
Nigeria	Jigawa	Gumel	190991	2005	249.0	225.5	274.6
Nigeria	Jigawa	Gumel	190991	2010	203.4	183.5	226.6
Nigeria	Jigawa	Gumel	190991	2015	179.5	157.9	205.3
Nigeria	Jigawa	Guri	190980	2000	219.1	202.3	237.4
Nigeria	Jigawa	Guri	190980	2005	194.5	179.3	211.0
Nigeria	Jigawa	Guri	190980	2010	172.8	158.0	189.0
Nigeria	Jigawa	Guri	190980	2015	147.2	130.2	166.3
Nigeria	Jigawa	Gwaram	22806	2000	289.0	268.1	309.5
Nigeria	Jigawa	Gwaram	22806	2005	247.6	229.3	265.6
Nigeria	Jigawa	Gwaram	22806	2010	218.0	200.7	235.3
Nigeria	Jigawa	Gwaram	22806	2015	191.6	170.7	214.2
Nigeria	Jigawa	Gwiwa	190978	2000	266.2	246.7	285.7
Nigeria	Jigawa	Gwiwa	190978	2005	241.3	223.0	260.1
Nigeria	Jigawa	Gwiwa	190978	2010	205.8	188.9	223.9
Nigeria	Jigawa	Gwiwa	190978	2015	173.5	153.9	195.8
Nigeria	Jigawa	Hadejia	191004	2000	246.3	228.3	264.5
Nigeria	Jigawa	Hadejia	191004	2005	209.9	194.1	226.5
Nigeria	Jigawa	Hadejia	191004	2010	171.5	157.2	187.6
Nigeria	Jigawa	Hadejia	191004	2015	162.1	144.0	184.0
Nigeria	Jigawa	Jahun	191025	2000	320.3	299.6	341.1
Nigeria	Jigawa	Jahun	191025	2005	286.2	268.3	306.2
Nigeria	Jigawa	Jahun	191025	2010	238.2	220.6	256.2
Nigeria	Jigawa	Jahun	191025	2015	202.3	179.9	225.4
Nigeria	Jigawa	Kafin Hausa	22809	2000	301.1	281.5	321.4
Nigeria	Jigawa	Kafin Hausa	22809	2005	269.6	250.5	290.0
Nigeria	Jigawa	Kafin Hausa	22809	2010	217.6	201.4	235.7
Nigeria	Jigawa	Kafin Hausa	22809	2015	190.5	170.4	214.7
Nigeria	Jigawa	Kaugama	190981	2000	295.6	275.6	316.8

Admin 0	Admin 1	Admin 2	GAUL Code	Year	Under-5 mortality (per 1,000 livebirths)		
					Estimate	Lower bound, 95% UI	Upper bound, 95% UI
Nigeria	Jigawa	Kaugama	190981	2005	263.7	244.8	282.5
Nigeria	Jigawa	Kaugama	190981	2010	217.1	199.7	236.4
Nigeria	Jigawa	Kaugama	190981	2015	188.6	168.3	212.6
Nigeria	Jigawa	Kazaure	190988	2000	244.7	223.5	266.6
Nigeria	Jigawa	Kazaure	190988	2005	211.6	194.0	230.4
Nigeria	Jigawa	Kazaure	190988	2010	176.3	159.3	194.1
Nigeria	Jigawa	Kazaure	190988	2015	154.3	134.7	175.0
Nigeria	Jigawa	Kiri Kasamma	190985	2000	238.5	220.2	257.4
Nigeria	Jigawa	Kiri Kasamma	190985	2005	210.5	193.5	228.3
Nigeria	Jigawa	Kiri Kasamma	190985	2010	178.9	163.7	195.4
Nigeria	Jigawa	Kiri Kasamma	190985	2015	154.5	136.8	175.8
Nigeria	Jigawa	Kiyawa	22812	2000	317.5	297.0	338.4
Nigeria	Jigawa	Kiyawa	22812	2005	277.4	258.1	297.6
Nigeria	Jigawa	Kiyawa	22812	2010	232.4	215.2	250.5
Nigeria	Jigawa	Kiyawa	22812	2015	202.6	180.6	227.0
Nigeria	Jigawa	Maigatari	191410	2000	290.2	268.5	313.6
Nigeria	Jigawa	Maigatari	191410	2005	256.2	237.1	277.9
Nigeria	Jigawa	Maigatari	191410	2010	211.7	193.1	231.1
Nigeria	Jigawa	Maigatari	191410	2015	181.0	161.0	204.3
Nigeria	Jigawa	Malam Maduri	22813	2000	268.8	249.7	288.6
Nigeria	Jigawa	Malam Maduri	22813	2005	235.8	218.0	254.0
Nigeria	Jigawa	Malam Maduri	22813	2010	192.3	176.3	210.8
Nigeria	Jigawa	Malam Maduri	22813	2015	173.0	153.1	196.2
Nigeria	Jigawa	Miga	191017	2000	309.3	288.6	332.1
Nigeria	Jigawa	Miga	191017	2005	276.8	257.7	297.7
Nigeria	Jigawa	Miga	191017	2010	227.0	208.8	247.3
Nigeria	Jigawa	Miga	191017	2015	195.8	174.4	219.4
Nigeria	Jigawa	Ringim	191018	2000	293.6	273.3	317.0
Nigeria	Jigawa	Ringim	191018	2005	250.3	232.2	269.7
Nigeria	Jigawa	Ringim	191018	2010	201.9	186.5	219.5
Nigeria	Jigawa	Ringim	191018	2015	182.8	163.5	204.4
Nigeria	Jigawa	Roni	190992	2000	262.8	243.5	282.8
Nigeria	Jigawa	Roni	190992	2005	230.4	213.0	249.1
Nigeria	Jigawa	Roni	190992	2010	191.9	175.3	209.9
Nigeria	Jigawa	Roni	190992	2015	161.1	142.5	181.6
Nigeria	Jigawa	Sule Tankarkar	191412	2000	286.8	266.5	306.3
Nigeria	Jigawa	Sule Tankarkar	191412	2005	252.3	234.7	269.7
Nigeria	Jigawa	Sule Tankarkar	191412	2010	211.3	195.8	228.8
Nigeria	Jigawa	Sule Tankarkar	191412	2015	184.0	164.6	205.2
Nigeria	Jigawa	Taura	191012	2000	305.5	286.9	325.9
Nigeria	Jigawa	Taura	191012	2005	269.7	253.3	287.5
Nigeria	Jigawa	Taura	191012	2010	224.3	207.9	242.6
Nigeria	Jigawa	Taura	191012	2015	194.4	174.5	216.8
Nigeria	Jigawa	Yankwashi	190979	2000	256.5	238.1	276.1
Nigeria	Jigawa	Yankwashi	190979	2005	229.3	212.7	247.3
Nigeria	Jigawa	Yankwashi	190979	2010	192.8	176.3	210.1
Nigeria	Jigawa	Yankwashi	190979	2015	154.0	134.2	174.4
Nigeria	Kaduna	Birnin Gwari	22817	2000	196.6	177.9	216.8
Nigeria	Kaduna	Birnin Gwari	22817	2005	167.0	151.7	184.8
Nigeria	Kaduna	Birnin Gwari	22817	2010	144.7	129.3	161.2
Nigeria	Kaduna	Birnin Gwari	22817	2015	139.9	122.0	159.3
Nigeria	Kaduna	Chikun	191092	2000	159.8	145.0	173.7
Nigeria	Kaduna	Chikun	191092	2005	136.3	124.2	149.3
Nigeria	Kaduna	Chikun	191092	2010	115.3	104.3	127.8
Nigeria	Kaduna	Chikun	191092	2015	109.6	97.1	123.9
Nigeria	Kaduna	Giwa	22820	2000	217.2	200.1	233.9
Nigeria	Kaduna	Giwa	22820	2005	179.3	165.8	193.3
Nigeria	Kaduna	Giwa	22820	2010	149.1	136.0	163.8
Nigeria	Kaduna	Giwa	22820	2015	140.5	124.4	157.4
Nigeria	Kaduna	Igabi	22821	2000	156.7	142.2	170.4
Nigeria	Kaduna	Igabi	22821	2005	133.8	121.5	146.1
Nigeria	Kaduna	Igabi	22821	2010	111.8	101.8	122.9
Nigeria	Kaduna	Igabi	22821	2015	108.3	95.5	122.3
Nigeria	Kaduna	Ikara	191075	2000	257.2	237.0	278.3
Nigeria	Kaduna	Ikara	191075	2005	210.1	193.9	227.8
Nigeria	Kaduna	Ikara	191075	2010	167.8	151.9	183.4
Nigeria	Kaduna	Ikara	191075	2015	163.2	144.6	183.3
Nigeria	Kaduna	Jaba	22823	2000	166.3	151.7	182.4
Nigeria	Kaduna	Jaba	22823	2005	148.7	135.7	163.5
Nigeria	Kaduna	Jaba	22823	2010	129.7	115.9	145.7
Nigeria	Kaduna	Jaba	22823	2015	110.8	97.6	126.2
Nigeria	Kaduna	Jema'a	191130	2000	168.2	154.2	184.1
Nigeria	Kaduna	Jema'a	191130	2005	151.4	138.0	166.1
Nigeria	Kaduna	Jema'a	191130	2010	133.3	120.0	148.5
Nigeria	Kaduna	Jema'a	191130	2015	114.6	101.6	130.7
Nigeria	Kaduna	Kachia	191108	2000	171.0	155.9	187.8
Nigeria	Kaduna	Kachia	191108	2005	146.8	134.4	161.0
Nigeria	Kaduna	Kachia	191108	2010	125.7	113.5	139.8
Nigeria	Kaduna	Kachia	191108	2015	112.5	100.2	127.9
Nigeria	Kaduna	Kaduna North	22819	2000	134.0	119.3	148.1
Nigeria	Kaduna	Kaduna North	22819	2005	120.5	107.4	133.5
Nigeria	Kaduna	Kaduna North	22819	2010	102.7	91.9	114.2
Nigeria	Kaduna	Kaduna North	22819	2015	95.1	82.5	108.8
Nigeria	Kaduna	Kaduna South	22832	2000	130.1	115.5	144.1
Nigeria	Kaduna	Kaduna South	22832	2005	116.8	103.9	129.5

Admin 0	Admin 1	Admin 2	GAUL Code	Year	Under-5 mortality (per 1,000 livebirths)		
					Estimate	Lower bound, 95% UI	Upper bound, 95% UI
Nigeria	Kaduna	Kaduna South	22832	2010	98.9	88.1	110.1
Nigeria	Kaduna	Kaduna South	22832	2015	91.4	78.9	104.7
Nigeria	Kaduna	Kagarko	191415	2000	162.3	148.7	177.1
Nigeria	Kaduna	Kagarko	191415	2005	140.7	129.3	153.3
Nigeria	Kaduna	Kagarko	191415	2010	120.1	109.0	133.7
Nigeria	Kaduna	Kagarko	191415	2015	103.4	92.0	116.9
Nigeria	Kaduna	Kajuru	191100	2000	176.8	159.8	196.6
Nigeria	Kaduna	Kajuru	191100	2005	149.0	134.2	165.0
Nigeria	Kaduna	Kajuru	191100	2010	126.9	114.4	142.6
Nigeria	Kaduna	Kajuru	191100	2015	117.9	104.1	134.4
Nigeria	Kaduna	Kaura	22826	2000	166.5	150.4	183.2
Nigeria	Kaduna	Kaura	22826	2005	150.7	135.4	166.2
Nigeria	Kaduna	Kaura	22826	2010	131.9	117.9	147.4
Nigeria	Kaduna	Kaura	22826	2015	115.1	101.3	131.8
Nigeria	Kaduna	Kauru	22827	2000	195.1	178.5	212.7
Nigeria	Kaduna	Kauru	22827	2005	164.0	149.6	178.3
Nigeria	Kaduna	Kauru	22827	2010	141.3	128.2	155.0
Nigeria	Kaduna	Kauru	22827	2015	134.4	119.3	151.4
Nigeria	Kaduna	Kubau	191087	2000	245.0	226.5	265.8
Nigeria	Kaduna	Kubau	191087	2005	201.3	185.9	219.1
Nigeria	Kaduna	Kubau	191087	2010	167.0	153.2	183.5
Nigeria	Kaduna	Kubau	191087	2015	166.3	147.3	186.6
Nigeria	Kaduna	Kudan	191080	2000	221.8	205.4	239.9
Nigeria	Kaduna	Kudan	191080	2005	180.0	167.1	194.5
Nigeria	Kaduna	Kudan	191080	2010	148.1	135.1	161.4
Nigeria	Kaduna	Kudan	191080	2015	140.8	124.7	157.1
Nigeria	Kaduna	Lere	22828	2000	212.0	194.3	233.3
Nigeria	Kaduna	Lere	22828	2005	178.7	163.2	196.1
Nigeria	Kaduna	Lere	22828	2010	154.4	139.7	169.9
Nigeria	Kaduna	Lere	22828	2015	144.0	127.7	162.1
Nigeria	Kaduna	Makarfi	191077	2000	236.5	218.8	255.0
Nigeria	Kaduna	Makarfi	191077	2005	189.9	176.5	205.2
Nigeria	Kaduna	Makarfi	191077	2010	154.5	141.5	168.5
Nigeria	Kaduna	Makarfi	191077	2015	145.1	129.9	161.9
Nigeria	Kaduna	Sabon Gari	22830	2000	186.2	169.8	204.6
Nigeria	Kaduna	Sabon Gari	22830	2005	154.7	141.3	168.5
Nigeria	Kaduna	Sabon Gari	22830	2010	130.9	118.0	143.7
Nigeria	Kaduna	Sabon Gari	22830	2015	129.2	113.9	146.0
Nigeria	Kaduna	Sanga	191133	2000	173.9	157.9	191.2
Nigeria	Kaduna	Sanga	191133	2005	155.8	141.3	172.4
Nigeria	Kaduna	Sanga	191133	2010	137.8	124.6	152.8
Nigeria	Kaduna	Sanga	191133	2015	120.4	105.9	136.7
Nigeria	Kaduna	Soba	22831	2000	222.0	205.2	241.9
Nigeria	Kaduna	Soba	22831	2005	180.2	166.8	195.0
Nigeria	Kaduna	Soba	22831	2010	148.8	135.6	162.8
Nigeria	Kaduna	Soba	22831	2015	157.3	138.9	176.8
Nigeria	Kaduna	Zangon Kataf	22833	2000	170.6	155.2	188.1
Nigeria	Kaduna	Zangon Kataf	22833	2005	152.3	138.2	167.6
Nigeria	Kaduna	Zangon Kataf	22833	2010	133.4	119.5	148.4
Nigeria	Kaduna	Zangon Kataf	22833	2015	117.7	103.6	134.4
Nigeria	Kaduna	Zaria	22834	2000	184.6	168.1	204.0
Nigeria	Kaduna	Zaria	22834	2005	154.3	140.2	169.0
Nigeria	Kaduna	Zaria	22834	2010	129.9	117.0	143.0
Nigeria	Kaduna	Zaria	22834	2015	134.4	118.2	152.1
Nigeria	Kano	Ajingi	191029	2000	302.1	283.5	321.5
Nigeria	Kano	Ajingi	191029	2005	256.4	240.5	272.6
Nigeria	Kano	Ajingi	191029	2010	192.0	179.1	205.6
Nigeria	Kano	Ajingi	191029	2015	176.1	158.0	195.1
Nigeria	Kano	Albasu	191059	2000	317.0	295.5	337.5
Nigeria	Kano	Albasu	191059	2005	271.1	252.5	289.7
Nigeria	Kano	Albasu	191059	2010	208.1	192.0	224.9
Nigeria	Kano	Albasu	191059	2015	186.4	166.7	208.7
Nigeria	Kano	Bagwai	22836	2000	280.8	261.7	300.7
Nigeria	Kano	Bagwai	22836	2005	235.1	219.7	251.4
Nigeria	Kano	Bagwai	22836	2010	183.7	169.0	197.9
Nigeria	Kano	Bagwai	22836	2015	157.2	140.6	175.0
Nigeria	Kano	Bebeji	191058	2000	286.9	265.0	310.2
Nigeria	Kano	Bebeji	191058	2005	242.9	223.5	263.5
Nigeria	Kano	Bebeji	191058	2010	178.1	162.2	193.6
Nigeria	Kano	Bebeji	191058	2015	163.5	145.0	183.6
Nigeria	Kano	Bichi	22838	2000	282.1	264.3	302.7
Nigeria	Kano	Bichi	22838	2005	237.6	221.7	254.0
Nigeria	Kano	Bichi	22838	2010	182.3	169.0	196.9
Nigeria	Kano	Bichi	22838	2015	158.9	142.5	176.9
Nigeria	Kano	Bunkure	22839	2000	283.5	264.4	301.2
Nigeria	Kano	Bunkure	22839	2005	238.1	222.4	253.8
Nigeria	Kano	Bunkure	22839	2010	181.2	167.6	194.8
Nigeria	Kano	Bunkure	22839	2015	162.9	146.5	182.1
Nigeria	Kano	Dala	191031	2000	219.7	207.1	233.1
Nigeria	Kano	Dala	191031	2005	176.5	166.5	188.0
Nigeria	Kano	Dala	191031	2010	140.2	130.9	149.6
Nigeria	Kano	Dala	191031	2015	148.2	131.7	165.9
Nigeria	Kano	Dambatta	190999	2000	263.8	246.3	282.0
Nigeria	Kano	Dambatta	190999	2005	222.5	207.7	238.7
Nigeria	Kano	Dambatta	190999	2010	175.0	162.1	188.7

Admin 0	Admin 1	Admin 2	GAUL Code	Year	Under-5 mortality (per 1,000 livebirths)		
					Estimate	Lower bound, 95% UI	Upper bound, 95% UI
Nigeria	Kano	Dambatta	190999	2015	155.9	138.3	173.4
Nigeria	Kano	Dawakin Kudu	191043	2000	277.2	261.4	292.5
Nigeria	Kano	Dawakin Kudu	191043	2005	229.4	216.0	242.4
Nigeria	Kano	Dawakin Kudu	191043	2010	171.7	159.9	183.1
Nigeria	Kano	Dawakin Kudu	191043	2015	165.3	149.1	182.9
Nigeria	Kano	Dawakin Tofa	22843	2000	272.2	257.2	288.4
Nigeria	Kano	Dawakin Tofa	22843	2005	228.5	215.3	241.4
Nigeria	Kano	Dawakin Tofa	22843	2010	169.3	158.1	180.8
Nigeria	Kano	Dawakin Tofa	22843	2015	164.7	147.5	182.7
Nigeria	Kano	Doguwa	191084	2000	279.2	254.5	307.1
Nigeria	Kano	Doguwa	191084	2005	231.1	210.1	254.7
Nigeria	Kano	Doguwa	191084	2010	189.0	169.3	209.4
Nigeria	Kano	Doguwa	191084	2015	181.4	158.4	205.0
Nigeria	Kano	Fagge	191033	2000	202.5	190.3	214.5
Nigeria	Kano	Fagge	191033	2005	160.1	150.2	170.6
Nigeria	Kano	Fagge	191033	2010	134.3	124.8	143.7
Nigeria	Kano	Fagge	191033	2015	132.5	118.1	147.5
Nigeria	Kano	Gabasawa	22844	2000	282.9	267.7	299.8
Nigeria	Kano	Gabasawa	22844	2005	235.4	222.2	249.0
Nigeria	Kano	Gabasawa	22844	2010	184.3	172.9	196.7
Nigeria	Kano	Gabasawa	22844	2015	164.4	148.7	180.9
Nigeria	Kano	Garko	191061	2000	305.7	286.9	325.5
Nigeria	Kano	Garko	191061	2005	258.0	240.5	275.7
Nigeria	Kano	Garko	191061	2010	198.2	182.9	213.5
Nigeria	Kano	Garko	191061	2015	179.1	160.4	199.5
Nigeria	Kano	Garum Mallam	191055	2000	274.8	252.9	298.0
Nigeria	Kano	Garum Mallam	191055	2005	230.6	212.0	250.3
Nigeria	Kano	Garum Mallam	191055	2010	173.1	158.5	188.5
Nigeria	Kano	Garum Mallam	191055	2015	157.2	139.8	175.4
Nigeria	Kano	Gaya	191046	2000	311.0	292.8	329.7
Nigeria	Kano	Gaya	191046	2005	266.3	250.0	281.7
Nigeria	Kano	Gaya	191046	2010	203.3	189.2	217.5
Nigeria	Kano	Gaya	191046	2015	180.2	162.3	200.0
Nigeria	Kano	Gezawa	22846	2000	257.8	242.6	272.9
Nigeria	Kano	Gezawa	22846	2005	214.7	201.7	228.0
Nigeria	Kano	Gezawa	22846	2010	163.2	152.4	174.5
Nigeria	Kano	Gezawa	22846	2015	159.5	142.5	177.1
Nigeria	Kano	Gwale	191035	2000	214.2	201.4	227.2
Nigeria	Kano	Gwale	191035	2005	171.0	161.1	181.8
Nigeria	Kano	Gwale	191035	2010	137.3	127.9	147.0
Nigeria	Kano	Gwale	191035	2015	139.1	123.9	155.3
Nigeria	Kano	Gwarzo	22847	2000	287.7	268.2	309.0
Nigeria	Kano	Gwarzo	22847	2005	242.5	225.8	259.4
Nigeria	Kano	Gwarzo	22847	2010	191.4	175.3	205.9
Nigeria	Kano	Gwarzo	22847	2015	164.6	147.7	182.2
Nigeria	Kano	Kabo	22848	2000	289.9	269.1	311.9
Nigeria	Kano	Kabo	22848	2005	246.4	228.1	263.6
Nigeria	Kano	Kabo	22848	2010	187.7	172.9	202.2
Nigeria	Kano	Kabo	22848	2015	163.3	146.0	181.9
Nigeria	Kano	Kano Municipal	191039	2000	234.7	220.9	249.0
Nigeria	Kano	Kano Municipal	191039	2005	187.8	177.0	199.9
Nigeria	Kano	Kano Municipal	191039	2010	146.0	136.3	156.4
Nigeria	Kano	Kano Municipal	191039	2015	149.1	132.6	166.4
Nigeria	Kano	Karaye	191047	2000	275.1	255.1	297.5
Nigeria	Kano	Karaye	191047	2005	230.6	213.8	249.2
Nigeria	Kano	Karaye	191047	2010	178.1	162.8	192.9
Nigeria	Kano	Karaye	191047	2015	157.5	141.2	175.4
Nigeria	Kano	Kibiya	191065	2000	304.0	282.8	326.4
Nigeria	Kano	Kibiya	191065	2005	256.3	237.0	275.7
Nigeria	Kano	Kibiya	191065	2010	198.0	180.9	214.9
Nigeria	Kano	Kibiya	191065	2015	176.2	157.4	197.4
Nigeria	Kano	Kiru	191052	2000	275.3	255.6	296.1
Nigeria	Kano	Kiru	191052	2005	233.4	216.7	250.8
Nigeria	Kano	Kiru	191052	2010	171.2	157.7	184.6
Nigeria	Kano	Kiru	191052	2015	160.4	143.3	179.2
Nigeria	Kano	Kumbotso	22851	2000	243.2	229.3	256.7
Nigeria	Kano	Kumbotso	22851	2005	197.3	186.1	209.8
Nigeria	Kano	Kumbotso	22851	2010	149.8	140.0	160.2
Nigeria	Kano	Kumbotso	22851	2015	158.4	141.3	177.2
Nigeria	Kano	Kunchi	191002	2000	282.0	261.8	304.1
Nigeria	Kano	Kunchi	191002	2005	243.2	225.4	262.3
Nigeria	Kano	Kunchi	191002	2010	195.2	178.9	213.8
Nigeria	Kano	Kunchi	191002	2015	163.6	145.7	184.2
Nigeria	Kano	Kura	191049	2000	265.9	249.3	282.7
Nigeria	Kano	Kura	191049	2005	215.1	201.3	229.7
Nigeria	Kano	Kura	191049	2010	159.6	148.1	171.1
Nigeria	Kano	Kura	191049	2015	150.7	135.3	166.8
Nigeria	Kano	Madobi	191042	2000	274.2	256.6	292.6
Nigeria	Kano	Madobi	191042	2005	230.2	214.8	245.5
Nigeria	Kano	Madobi	191042	2010	170.7	158.5	183.0
Nigeria	Kano	Madobi	191042	2015	156.5	140.4	173.7
Nigeria	Kano	Makoda	191000	2000	260.2	242.3	278.8
Nigeria	Kano	Makoda	191000	2005	221.6	206.0	237.4
Nigeria	Kano	Makoda	191000	2010	177.5	164.3	191.4
Nigeria	Kano	Makoda	191000	2015	156.3	138.1	174.4

Admin 0	Admin 1	Admin 2	GAUL Code	Year	Under-5 mortality (per 1,000 livebirths)		
					Estimate	Lower bound, 95% UI	Upper bound, 95% UI
Nigeria	Kano	Minjibir	22853	2000	269.1	252.3	286.5
Nigeria	Kano	Minjibir	22853	2005	225.7	211.8	240.4
Nigeria	Kano	Minjibir	22853	2010	172.8	160.9	185.6
Nigeria	Kano	Minjibir	22853	2015	159.7	142.2	177.2
Nigeria	Kano	Nassarawa	191030	2000	203.6	190.7	216.8
Nigeria	Kano	Nassarawa	191030	2005	162.9	153.0	174.0
Nigeria	Kano	Nassarawa	191030	2010	133.9	124.2	143.6
Nigeria	Kano	Nassarawa	191030	2015	128.2	113.9	142.9
Nigeria	Kano	Rano	191066	2000	296.7	274.8	319.7
Nigeria	Kano	Rano	191066	2005	248.3	229.2	267.6
Nigeria	Kano	Rano	191066	2010	190.9	174.3	207.6
Nigeria	Kano	Rano	191066	2015	171.5	152.3	192.3
Nigeria	Kano	Rimin Gado	191034	2000	291.0	273.1	310.5
Nigeria	Kano	Rimin Gado	191034	2005	251.2	235.1	266.9
Nigeria	Kano	Rimin Gado	191034	2010	190.0	176.4	203.9
Nigeria	Kano	Rimin Gado	191034	2015	163.7	147.3	181.1
Nigeria	Kano	Rogo	191064	2000	264.2	242.2	289.4
Nigeria	Kano	Rogo	191064	2005	222.1	203.9	242.2
Nigeria	Kano	Rogo	191064	2010	167.7	152.8	183.7
Nigeria	Kano	Rogo	191064	2015	152.7	135.7	171.2
Nigeria	Kano	Shanono	22857	2000	300.1	279.7	320.2
Nigeria	Kano	Shanono	22857	2005	262.5	244.6	279.9
Nigeria	Kano	Shanono	22857	2010	209.8	193.2	225.2
Nigeria	Kano	Shanono	22857	2015	172.7	155.4	191.6
Nigeria	Kano	Sumaila	22858	2000	317.7	295.8	341.4
Nigeria	Kano	Sumaila	22858	2005	272.9	253.1	294.5
Nigeria	Kano	Sumaila	22858	2010	214.7	196.4	233.8
Nigeria	Kano	Sumaila	22858	2015	191.5	170.2	215.2
Nigeria	Kano	Takai	22859	2000	320.1	297.5	343.1
Nigeria	Kano	Takai	22859	2005	271.0	250.7	292.0
Nigeria	Kano	Takai	22859	2010	215.6	197.8	234.6
Nigeria	Kano	Takai	22859	2015	189.0	168.3	212.0
Nigeria	Kano	Tarauni	191036	2000	204.0	191.5	216.6
Nigeria	Kano	Tarauni	191036	2005	162.1	152.3	172.6
Nigeria	Kano	Tarauni	191036	2010	134.7	125.1	144.3
Nigeria	Kano	Tarauni	191036	2015	142.4	126.4	159.2
Nigeria	Kano	Tofa	191027	2000	286.9	270.7	304.9
Nigeria	Kano	Tofa	191027	2005	241.2	227.4	255.3
Nigeria	Kano	Tofa	191027	2010	182.6	170.8	195.2
Nigeria	Kano	Tofa	191027	2015	160.9	144.7	178.2
Nigeria	Kano	Tsanyawa	191008	2000	294.8	273.2	316.7
Nigeria	Kano	Tsanyawa	191008	2005	257.1	239.0	276.5
Nigeria	Kano	Tsanyawa	191008	2010	201.5	185.4	217.6
Nigeria	Kano	Tsanyawa	191008	2015	171.2	152.9	191.6
Nigeria	Kano	Tundun Wada	191078	2000	297.0	272.4	321.5
Nigeria	Kano	Tundun Wada	191078	2005	251.6	230.8	273.2
Nigeria	Kano	Tundun Wada	191078	2010	194.9	176.6	214.0
Nigeria	Kano	Tundun Wada	191078	2015	179.6	158.1	201.4
Nigeria	Kano	Ungogo	22862	2000	213.9	200.7	227.4
Nigeria	Kano	Ungogo	22862	2005	171.6	161.1	182.7
Nigeria	Kano	Ungogo	22862	2010	137.0	127.3	146.6
Nigeria	Kano	Ungogo	22862	2015	132.0	117.7	147.0
Nigeria	Kano	Warawa	191038	2000	278.6	262.7	293.9
Nigeria	Kano	Warawa	191038	2005	232.9	219.4	246.8
Nigeria	Kano	Warawa	191038	2010	174.5	163.0	186.3
Nigeria	Kano	Warawa	191038	2015	182.4	163.2	203.0
Nigeria	Kano	Wudil	191044	2000	281.8	265.3	299.1
Nigeria	Kano	Wudil	191044	2005	229.3	214.5	243.2
Nigeria	Kano	Wudil	191044	2010	177.3	164.8	189.3
Nigeria	Kano	Wudil	191044	2015	166.4	150.1	184.8
Nigeria	Katsina	Bakori	191053	2000	269.5	250.1	290.1
Nigeria	Katsina	Bakori	191053	2005	239.6	222.2	257.5
Nigeria	Katsina	Bakori	191053	2010	212.0	195.1	229.8
Nigeria	Katsina	Bakori	191053	2015	164.9	146.1	183.4
Nigeria	Katsina	Batagarawa	190968	2000	292.4	273.1	313.1
Nigeria	Katsina	Batagarawa	190968	2005	260.1	243.8	277.2
Nigeria	Katsina	Batagarawa	190968	2010	208.8	193.5	225.3
Nigeria	Katsina	Batagarawa	190968	2015	170.7	152.6	192.1
Nigeria	Katsina	Batsari	22865	2000	310.8	286.1	339.1
Nigeria	Katsina	Batsari	22865	2005	274.6	253.0	297.5
Nigeria	Katsina	Batsari	22865	2010	218.5	198.7	240.3
Nigeria	Katsina	Batsari	22865	2015	174.6	154.1	197.7
Nigeria	Katsina	Baure	190975	2000	264.9	243.8	287.5
Nigeria	Katsina	Baure	190975	2005	232.9	214.4	253.7
Nigeria	Katsina	Baure	190975	2010	198.0	180.3	219.0
Nigeria	Katsina	Baure	190975	2015	159.8	139.9	181.5
Nigeria	Katsina	Bindawa	22866	2000	289.4	266.7	314.9
Nigeria	Katsina	Bindawa	22866	2005	255.8	235.5	277.8
Nigeria	Katsina	Bindawa	22866	2010	214.8	195.7	236.0
Nigeria	Katsina	Bindawa	22866	2015	165.2	145.8	187.4
Nigeria	Katsina	Charanchi	190986	2000	301.8	278.9	326.7
Nigeria	Katsina	Charanchi	190986	2005	266.1	246.7	284.8
Nigeria	Katsina	Charanchi	190986	2010	218.6	201.2	236.9
Nigeria	Katsina	Charanchi	190986	2015	174.4	155.1	195.9
Nigeria	Katsina	Dandume	191071	2000	256.6	234.8	279.4

Admin 0	Admin 1	Admin 2	GAUL Code	Year	Under-5 mortality (per 1,000 livebirths)		
					Estimate	Lower bound, 95% UI	Upper bound, 95% UI
Nigeria	Katsina	Dandume	191071	2005	227.8	209.4	247.3
Nigeria	Katsina	Dandume	191071	2010	207.9	188.8	228.7
Nigeria	Katsina	Dandume	191071	2015	161.8	142.7	182.8
Nigeria	Katsina	Danja	191074	2000	252.7	234.8	271.9
Nigeria	Katsina	Danja	191074	2005	217.3	201.7	234.2
Nigeria	Katsina	Danja	191074	2010	180.5	165.7	196.0
Nigeria	Katsina	Danja	191074	2015	153.2	136.4	170.8
Nigeria	Katsina	Danmusa	191015	2000	309.2	286.2	331.2
Nigeria	Katsina	Danmusa	191015	2005	275.0	256.4	294.6
Nigeria	Katsina	Danmusa	191015	2010	229.5	211.8	249.3
Nigeria	Katsina	Danmusa	191015	2015	181.4	162.7	203.9
Nigeria	Katsina	Daura	190963	2000	251.8	234.3	271.8
Nigeria	Katsina	Daura	190963	2005	228.5	212.5	244.7
Nigeria	Katsina	Daura	190963	2010	190.1	173.8	206.6
Nigeria	Katsina	Daura	190963	2015	165.5	146.5	187.1
Nigeria	Katsina	Dutsi	190970	2000	273.3	253.5	295.3
Nigeria	Katsina	Dutsi	190970	2005	245.9	227.7	266.7
Nigeria	Katsina	Dutsi	190970	2010	227.2	208.7	248.5
Nigeria	Katsina	Dutsi	190970	2015	184.5	163.8	206.8
Nigeria	Katsina	Dutsinma	190997	2000	290.2	269.7	310.4
Nigeria	Katsina	Dutsinma	190997	2005	251.0	233.1	268.5
Nigeria	Katsina	Dutsinma	190997	2010	204.9	189.2	222.1
Nigeria	Katsina	Dutsinma	190997	2015	170.6	151.2	192.2
Nigeria	Katsina	Faskari	191045	2000	270.0	247.7	292.8
Nigeria	Katsina	Faskari	191045	2005	245.5	226.3	265.8
Nigeria	Katsina	Faskari	191045	2010	213.4	194.1	232.7
Nigeria	Katsina	Faskari	191045	2015	173.1	152.7	193.7
Nigeria	Katsina	Funtua	191069	2000	241.6	222.6	262.3
Nigeria	Katsina	Funtua	191069	2005	202.7	186.9	219.6
Nigeria	Katsina	Funtua	191069	2010	172.8	157.7	189.0
Nigeria	Katsina	Funtua	191069	2015	151.5	133.9	169.1
Nigeria	Katsina	Ingawa	22871	2000	284.5	264.3	305.6
Nigeria	Katsina	Ingawa	22871	2005	255.3	237.2	273.8
Nigeria	Katsina	Ingawa	22871	2010	219.8	202.1	238.7
Nigeria	Katsina	Ingawa	22871	2015	173.0	154.9	194.8
Nigeria	Katsina	Jibia	22872	2000	267.3	248.4	287.6
Nigeria	Katsina	Jibia	22872	2005	233.5	218.1	250.5
Nigeria	Katsina	Jibia	22872	2010	191.0	176.0	207.5
Nigeria	Katsina	Jibia	22872	2015	159.0	141.7	178.9
Nigeria	Katsina	Kafur	191048	2000	279.3	259.0	299.5
Nigeria	Katsina	Kafur	191048	2005	239.1	222.8	257.2
Nigeria	Katsina	Kafur	191048	2010	204.5	188.3	221.5
Nigeria	Katsina	Kafur	191048	2015	164.2	146.4	183.6
Nigeria	Katsina	Kaita	22873	2000	293.5	272.0	315.0
Nigeria	Katsina	Kaita	22873	2005	263.8	245.4	284.5
Nigeria	Katsina	Kaita	22873	2010	224.1	205.1	244.6
Nigeria	Katsina	Kaita	22873	2015	172.3	153.1	195.0
Nigeria	Katsina	Kankara	22874	2000	289.8	268.6	313.9
Nigeria	Katsina	Kankara	22874	2005	256.8	238.8	276.6
Nigeria	Katsina	Kankara	22874	2010	226.7	208.4	247.6
Nigeria	Katsina	Kankara	22874	2015	175.4	156.1	196.5
Nigeria	Katsina	Kankia	190995	2000	300.3	277.5	323.4
Nigeria	Katsina	Kankia	190995	2005	264.7	246.4	283.7
Nigeria	Katsina	Kankia	190995	2010	218.7	201.6	235.5
Nigeria	Katsina	Kankia	190995	2015	173.9	155.0	194.8
Nigeria	Katsina	Katsina	190965	2000	261.0	243.2	280.8
Nigeria	Katsina	Katsina	190965	2005	225.6	210.6	242.5
Nigeria	Katsina	Katsina	190965	2010	188.4	174.3	204.5
Nigeria	Katsina	Katsina	190965	2015	159.6	142.1	180.0
Nigeria	Katsina	Kurfi	190977	2000	306.0	285.0	327.4
Nigeria	Katsina	Kurfi	190977	2005	269.1	251.8	286.6
Nigeria	Katsina	Kurfi	190977	2010	215.7	199.2	232.8
Nigeria	Katsina	Kurfi	190977	2015	173.8	155.4	194.2
Nigeria	Katsina	Kusada	191001	2000	296.2	274.8	319.0
Nigeria	Katsina	Kusada	191001	2005	263.4	245.4	283.9
Nigeria	Katsina	Kusada	191001	2010	219.3	201.1	237.9
Nigeria	Katsina	Kusada	191001	2015	170.1	152.4	191.2
Nigeria	Katsina	Mai'Adua	190955	2000	265.7	248.0	285.2
Nigeria	Katsina	Mai'Adua	190955	2005	240.0	223.2	257.4
Nigeria	Katsina	Mai'Adua	190955	2010	203.2	186.6	221.9
Nigeria	Katsina	Mai'Adua	190955	2015	179.0	159.0	201.2
Nigeria	Katsina	Malumfashi	191037	2000	283.5	260.0	308.0
Nigeria	Katsina	Malumfashi	191037	2005	242.5	223.7	262.3
Nigeria	Katsina	Malumfashi	191037	2010	205.9	187.4	225.8
Nigeria	Katsina	Malumfashi	191037	2015	165.3	146.1	186.9
Nigeria	Katsina	Mani	22878	2000	285.1	264.8	305.1
Nigeria	Katsina	Mani	22878	2005	257.4	239.5	275.6
Nigeria	Katsina	Mani	22878	2010	222.6	204.8	241.3
Nigeria	Katsina	Mani	22878	2015	173.4	154.5	195.1
Nigeria	Katsina	Mashi	190953	2000	283.9	267.2	301.5
Nigeria	Katsina	Mashi	190953	2005	262.8	247.6	278.9
Nigeria	Katsina	Mashi	190953	2010	225.0	209.1	242.5
Nigeria	Katsina	Mashi	190953	2015	180.2	160.8	202.0
Nigeria	Katsina	Matazu	191011	2000	318.5	294.7	345.8
Nigeria	Katsina	Matazu	191011	2005	280.3	259.7	303.0

Admin 0	Admin 1	Admin 2	GAUL Code	Year	Under-5 mortality (per 1,000 livebirths)		
					Estimate	Lower bound, 95% UI	Upper bound, 95% UI
Nigeria	Katsina	Matazu	191011	2010	241.4	221.4	263.4
Nigeria	Katsina	Matazu	191011	2015	182.9	162.4	205.1
Nigeria	Katsina	Musawa	191020	2000	306.6	285.1	329.4
Nigeria	Katsina	Musawa	191020	2005	273.3	254.3	293.7
Nigeria	Katsina	Musawa	191020	2010	235.0	216.8	254.6
Nigeria	Katsina	Musawa	191020	2015	178.0	158.8	197.5
Nigeria	Katsina	Rimi	190967	2000	293.5	272.8	315.5
Nigeria	Katsina	Rimi	190967	2005	259.7	242.4	277.9
Nigeria	Katsina	Rimi	190967	2010	214.8	197.3	232.9
Nigeria	Katsina	Rimi	190967	2015	166.8	149.1	188.3
Nigeria	Katsina	Sabuwa	191076	2000	246.9	226.6	270.1
Nigeria	Katsina	Sabuwa	191076	2005	220.6	202.6	241.4
Nigeria	Katsina	Sabuwa	191076	2010	198.8	180.7	221.4
Nigeria	Katsina	Sabuwa	191076	2015	162.3	143.2	183.9
Nigeria	Katsina	Safana	190993	2000	315.0	294.5	335.4
Nigeria	Katsina	Safana	190993	2005	279.9	263.2	298.2
Nigeria	Katsina	Safana	190993	2010	225.8	209.0	243.7
Nigeria	Katsina	Safana	190993	2015	181.7	162.7	203.8
Nigeria	Katsina	Sandamu	190972	2000	258.8	241.9	277.3
Nigeria	Katsina	Sandamu	190972	2005	233.5	216.8	250.7
Nigeria	Katsina	Sandamu	190972	2010	197.5	181.4	214.6
Nigeria	Katsina	Sandamu	190972	2015	175.0	155.1	196.4
Nigeria	Katsina	Zango	190964	2000	252.2	235.0	270.4
Nigeria	Katsina	Zango	190964	2005	224.0	207.8	239.8
Nigeria	Katsina	Zango	190964	2010	190.5	175.1	207.2
Nigeria	Katsina	Zango	190964	2015	165.2	146.4	186.0
Nigeria	Kebbi	Aleiro	191009	2000	211.4	194.4	229.2
Nigeria	Kebbi	Aleiro	191009	2005	200.4	184.8	218.7
Nigeria	Kebbi	Aleiro	191009	2010	184.9	169.9	201.7
Nigeria	Kebbi	Aleiro	191009	2015	157.1	138.3	176.8
Nigeria	Kebbi	Arewa Dandi	22884	2000	234.2	219.3	251.4
Nigeria	Kebbi	Arewa Dandi	22884	2005	217.1	202.7	233.1
Nigeria	Kebbi	Arewa Dandi	22884	2010	193.5	178.2	209.7
Nigeria	Kebbi	Arewa Dandi	22884	2015	168.4	150.2	188.5
Nigeria	Kebbi	Argungu	190983	2000	210.7	193.7	228.3
Nigeria	Kebbi	Argungu	190983	2005	196.7	180.4	214.5
Nigeria	Kebbi	Argungu	190983	2010	173.0	158.4	188.0
Nigeria	Kebbi	Argungu	190983	2015	151.0	133.7	168.7
Nigeria	Kebbi	Augie	190959	2000	231.2	212.2	250.3
Nigeria	Kebbi	Augie	190959	2005	222.5	203.8	240.9
Nigeria	Kebbi	Augie	190959	2010	182.7	166.7	198.4
Nigeria	Kebbi	Augie	190959	2015	158.7	140.4	177.2
Nigeria	Kebbi	Bagudo	22886	2000	192.0	175.0	210.0
Nigeria	Kebbi	Bagudo	22886	2005	179.9	165.1	196.1
Nigeria	Kebbi	Bagudo	22886	2010	169.8	154.0	186.3
Nigeria	Kebbi	Bagudo	22886	2015	154.4	136.0	175.1
Nigeria	Kebbi	Birnin-Kebbi	22887	2000	203.8	187.6	220.4
Nigeria	Kebbi	Birnin-Kebbi	22887	2005	187.0	173.2	202.8
Nigeria	Kebbi	Birnin-Kebbi	22887	2010	170.4	155.8	186.0
Nigeria	Kebbi	Birnin-Kebbi	22887	2015	154.3	136.2	173.9
Nigeria	Kebbi	Bunza	191022	2000	237.6	215.9	261.0
Nigeria	Kebbi	Bunza	191022	2005	222.8	202.3	244.2
Nigeria	Kebbi	Bunza	191022	2010	206.5	187.2	228.3
Nigeria	Kebbi	Bunza	191022	2015	184.5	161.8	208.7
Nigeria	Kebbi	Dandi	22889	2000	215.2	196.8	235.2
Nigeria	Kebbi	Dandi	22889	2005	200.6	184.7	220.0
Nigeria	Kebbi	Dandi	22889	2010	190.9	173.9	211.5
Nigeria	Kebbi	Dandi	22889	2015	164.6	144.1	186.5
Nigeria	Kebbi	Danko Wasagu	191419	2000	204.1	186.6	224.2
Nigeria	Kebbi	Danko Wasagu	191419	2005	183.3	167.4	200.8
Nigeria	Kebbi	Danko Wasagu	191419	2010	170.1	153.9	186.8
Nigeria	Kebbi	Danko Wasagu	191419	2015	150.3	131.7	171.0
Nigeria	Kebbi	Fakai	191062	2000	206.0	186.6	227.6
Nigeria	Kebbi	Fakai	191062	2005	191.2	174.0	210.3
Nigeria	Kebbi	Fakai	191062	2010	178.8	160.9	199.0
Nigeria	Kebbi	Fakai	191062	2015	155.9	136.7	179.3
Nigeria	Kebbi	Gwandu	22890	2000	219.8	202.4	238.1
Nigeria	Kebbi	Gwandu	22890	2005	213.1	196.0	231.9
Nigeria	Kebbi	Gwandu	22890	2010	186.8	171.0	204.6
Nigeria	Kebbi	Gwandu	22890	2015	158.2	140.8	176.3
Nigeria	Kebbi	Jega	191021	2000	226.9	207.5	248.2
Nigeria	Kebbi	Jega	191021	2005	217.8	199.1	239.4
Nigeria	Kebbi	Jega	191021	2010	198.4	180.9	217.6
Nigeria	Kebbi	Jega	191021	2015	174.3	154.0	195.9
Nigeria	Kebbi	Kalgo	191005	2000	232.1	211.3	255.2
Nigeria	Kebbi	Kalgo	191005	2005	214.5	196.0	234.5
Nigeria	Kebbi	Kalgo	191005	2010	199.6	181.2	220.0
Nigeria	Kebbi	Kalgo	191005	2015	172.6	152.2	195.9
Nigeria	Kebbi	Koko/Besse	22892	2000	216.9	196.9	240.3
Nigeria	Kebbi	Koko/Besse	22892	2005	203.1	184.6	223.8
Nigeria	Kebbi	Koko/Besse	22892	2010	197.8	178.7	220.0
Nigeria	Kebbi	Koko/Besse	22892	2015	167.0	146.6	192.0
Nigeria	Kebbi	Maiyama	22893	2000	236.2	213.8	261.1
Nigeria	Kebbi	Maiyama	22893	2005	225.5	205.1	250.1
Nigeria	Kebbi	Maiyama	22893	2010	211.0	189.7	234.3

Admin 0	Admin 1	Admin 2	GAUL Code	Year	Under-5 mortality (per 1,000 livebirths)		
					Estimate	Lower bound, 95% UI	Upper bound, 95% UI
Nigeria	Kebbi	Maiyama	22893	2015	179.2	157.3	204.2
Nigeria	Kebbi	Ngaski	22894	2000	196.4	174.5	220.1
Nigeria	Kebbi	Ngaski	22894	2005	176.8	158.7	196.8
Nigeria	Kebbi	Ngaski	22894	2010	172.8	153.6	195.1
Nigeria	Kebbi	Ngaski	22894	2015	160.2	139.9	185.2
Nigeria	Kebbi	Sakaba	191079	2000	179.8	161.5	201.1
Nigeria	Kebbi	Sakaba	191079	2005	161.0	145.2	179.1
Nigeria	Kebbi	Sakaba	191079	2010	153.2	136.6	171.5
Nigeria	Kebbi	Sakaba	191079	2015	140.2	121.2	162.0
Nigeria	Kebbi	Shanga	191072	2000	206.4	186.8	226.4
Nigeria	Kebbi	Shanga	191072	2005	190.9	173.6	209.2
Nigeria	Kebbi	Shanga	191072	2010	184.6	166.2	204.9
Nigeria	Kebbi	Shanga	191072	2015	163.0	142.6	187.6
Nigeria	Kebbi	Suru	22896	2000	228.6	208.4	251.6
Nigeria	Kebbi	Suru	22896	2005	213.2	194.9	234.1
Nigeria	Kebbi	Suru	22896	2010	204.0	185.0	226.6
Nigeria	Kebbi	Suru	22896	2015	172.4	151.0	196.4
Nigeria	Kebbi	Yauri	191086	2000	196.6	175.2	217.5
Nigeria	Kebbi	Yauri	191086	2005	179.3	161.6	197.6
Nigeria	Kebbi	Yauri	191086	2010	177.6	158.5	198.7
Nigeria	Kebbi	Yauri	191086	2015	161.4	139.6	185.4
Nigeria	Kebbi	Zuru	191057	2000	178.5	161.3	198.0
Nigeria	Kebbi	Zuru	191057	2005	162.9	147.5	180.6
Nigeria	Kebbi	Zuru	191057	2010	151.4	135.1	168.4
Nigeria	Kebbi	Zuru	191057	2015	135.6	118.7	155.7
Nigeria	Kogi	Adavi	22899	2000	111.1	100.7	123.0
Nigeria	Kogi	Adavi	22899	2005	100.6	90.8	111.2
Nigeria	Kogi	Adavi	22899	2010	87.3	78.4	97.0
Nigeria	Kogi	Adavi	22899	2015	67.5	58.9	77.2
Nigeria	Kogi	Ajaokuta	191205	2000	124.9	111.6	139.4
Nigeria	Kogi	Ajaokuta	191205	2005	111.7	99.8	124.6
Nigeria	Kogi	Ajaokuta	191205	2010	98.3	86.5	111.3
Nigeria	Kogi	Ajaokuta	191205	2015	75.7	65.5	87.0
Nigeria	Kogi	Ankpa	191216	2000	144.8	131.3	160.3
Nigeria	Kogi	Ankpa	191216	2005	127.7	115.6	141.3
Nigeria	Kogi	Ankpa	191216	2010	112.3	100.2	125.4
Nigeria	Kogi	Ankpa	191216	2015	88.3	77.0	100.8
Nigeria	Kogi	Bassa	22902	2000	132.0	119.2	146.6
Nigeria	Kogi	Bassa	22902	2005	114.9	103.5	127.6
Nigeria	Kogi	Bassa	22902	2010	101.6	90.3	113.4
Nigeria	Kogi	Bassa	22902	2015	80.1	69.6	90.9
Nigeria	Kogi	Dekina	22903	2000	137.5	123.1	153.1
Nigeria	Kogi	Dekina	22903	2005	121.6	109.2	134.6
Nigeria	Kogi	Dekina	22903	2010	107.5	95.7	119.8
Nigeria	Kogi	Dekina	22903	2015	85.2	74.3	97.8
Nigeria	Kogi	Ibaji	191265	2000	160.4	144.4	177.9
Nigeria	Kogi	Ibaji	191265	2005	141.5	127.3	156.3
Nigeria	Kogi	Ibaji	191265	2010	122.9	110.5	137.0
Nigeria	Kogi	Ibaji	191265	2015	96.7	84.9	109.2
Nigeria	Kogi	Idah	191260	2000	143.3	127.5	160.6
Nigeria	Kogi	Idah	191260	2005	127.4	113.8	143.0
Nigeria	Kogi	Idah	191260	2010	112.9	100.1	127.6
Nigeria	Kogi	Idah	191260	2015	85.1	73.9	98.5
Nigeria	Kogi	Igalamela-Odolu	191251	2000	138.8	125.3	154.4
Nigeria	Kogi	Igalamela-Odolu	191251	2005	125.7	113.0	139.2
Nigeria	Kogi	Igalamela-Odolu	191251	2010	111.5	100.0	124.2
Nigeria	Kogi	Igalamela-Odolu	191251	2015	84.4	74.4	96.4
Nigeria	Kogi	Ijumu	22904	2000	115.9	104.0	128.6
Nigeria	Kogi	Ijumu	22904	2005	104.8	93.3	116.5
Nigeria	Kogi	Ijumu	22904	2010	88.9	79.2	99.1
Nigeria	Kogi	Ijumu	22904	2015	71.0	62.1	81.4
Nigeria	Kogi	Kabba /Bunu	22905	2000	116.9	105.5	129.3
Nigeria	Kogi	Kabba /Bunu	22905	2005	104.2	94.1	115.1
Nigeria	Kogi	Kabba /Bunu	22905	2010	90.5	80.9	100.5
Nigeria	Kogi	Kabba /Bunu	22905	2015	72.7	63.8	83.4
Nigeria	Kogi	Koton-Karfe	22906	2000	132.0	118.4	147.6
Nigeria	Kogi	Koton-Karfe	22906	2005	111.8	99.6	125.8
Nigeria	Kogi	Koton-Karfe	22906	2010	98.2	86.0	110.8
Nigeria	Kogi	Koton-Karfe	22906	2015	80.3	68.9	92.7
Nigeria	Kogi	Lokoja	22907	2000	124.0	112.3	138.8
Nigeria	Kogi	Lokoja	22907	2005	107.7	97.6	119.5
Nigeria	Kogi	Lokoja	22907	2010	94.4	84.6	104.8
Nigeria	Kogi	Lokoja	22907	2015	78.0	67.8	89.0
Nigeria	Kogi	Mopa-Muro	191178	2000	113.5	101.1	127.6
Nigeria	Kogi	Mopa-Muro	191178	2005	102.5	91.2	115.1
Nigeria	Kogi	Mopa-Muro	191178	2010	89.6	78.9	101.0
Nigeria	Kogi	Mopa-Muro	191178	2015	72.5	63.1	83.4
Nigeria	Kogi	Ofu	22908	2000	141.4	128.2	157.0
Nigeria	Kogi	Ofu	22908	2005	126.0	113.6	138.6
Nigeria	Kogi	Ofu	22908	2010	111.3	99.4	123.9
Nigeria	Kogi	Ofu	22908	2015	85.9	75.6	98.1
Nigeria	Kogi	Ogori/ Magongo	191228	2000	124.3	112.8	137.6
Nigeria	Kogi	Ogori/ Magongo	191228	2005	108.9	98.5	120.4
Nigeria	Kogi	Ogori/ Magongo	191228	2010	92.6	83.0	103.3
Nigeria	Kogi	Ogori/ Magongo	191228	2015	70.7	62.0	81.2

Admin 0	Admin 1	Admin 2	GAUL Code	Year	Under-5 mortality (per 1,000 livebirths)		
					Estimate	Lower bound, 95% UI	Upper bound, 95% UI
Nigeria	Kogi	Okehi	22909	2000	116.8	106.2	128.9
Nigeria	Kogi	Okehi	22909	2005	103.7	93.5	114.2
Nigeria	Kogi	Okehi	22909	2010	89.5	80.3	99.5
Nigeria	Kogi	Okehi	22909	2015	69.8	60.9	79.5
Nigeria	Kogi	Okene	191223	2000	118.8	108.0	131.1
Nigeria	Kogi	Okene	191223	2005	106.7	96.7	117.7
Nigeria	Kogi	Okene	191223	2010	92.5	83.4	103.1
Nigeria	Kogi	Okene	191223	2015	71.5	62.9	81.5
Nigeria	Kogi	Olamaboro	22911	2000	153.2	140.5	168.0
Nigeria	Kogi	Olamaboro	22911	2005	135.2	123.8	147.2
Nigeria	Kogi	Olamaboro	22911	2010	119.1	107.9	130.9
Nigeria	Kogi	Olamaboro	22911	2015	91.2	79.7	103.1
Nigeria	Kogi	Omala	191189	2000	146.4	131.8	162.2
Nigeria	Kogi	Omala	191189	2005	126.5	113.8	140.1
Nigeria	Kogi	Omala	191189	2010	112.3	100.5	125.6
Nigeria	Kogi	Omala	191189	2015	91.5	79.8	105.6
Nigeria	Kogi	Yagba East	191173	2000	112.3	100.2	125.9
Nigeria	Kogi	Yagba East	191173	2005	101.6	90.8	113.5
Nigeria	Kogi	Yagba East	191173	2010	89.1	78.6	99.9
Nigeria	Kogi	Yagba East	191173	2015	73.3	63.6	84.2
Nigeria	Kogi	Yagba West	191169	2000	109.7	96.7	124.4
Nigeria	Kogi	Yagba West	191169	2005	99.2	87.9	112.3
Nigeria	Kogi	Yagba West	191169	2010	88.1	77.4	100.1
Nigeria	Kogi	Yagba West	191169	2015	73.7	64.3	86.5
Nigeria	Kwara	Asa	22914	2000	92.4	83.4	102.8
Nigeria	Kwara	Asa	22914	2005	84.7	76.1	94.1
Nigeria	Kwara	Asa	22914	2010	77.4	68.6	86.8
Nigeria	Kwara	Asa	22914	2015	66.5	58.0	76.7
Nigeria	Kwara	Baruten	22915	2000	118.7	107.5	130.7
Nigeria	Kwara	Baruten	22915	2005	102.8	93.2	113.0
Nigeria	Kwara	Baruten	22915	2010	93.9	83.5	103.8
Nigeria	Kwara	Baruten	22915	2015	91.9	80.6	104.3
Nigeria	Kwara	Edu	191411	2000	114.1	101.2	129.0
Nigeria	Kwara	Edu	191411	2005	99.8	88.5	112.2
Nigeria	Kwara	Edu	191411	2010	93.2	82.3	105.5
Nigeria	Kwara	Edu	191411	2015	85.1	73.7	98.4
Nigeria	Kwara	Ekiti	191184	2000	106.4	95.2	118.2
Nigeria	Kwara	Ekiti	191184	2005	96.9	86.9	107.4
Nigeria	Kwara	Ekiti	191184	2010	84.4	75.2	94.5
Nigeria	Kwara	Ekiti	191184	2015	69.6	61.0	79.9
Nigeria	Kwara	Ifelodun	22917	2000	106.0	94.4	117.6
Nigeria	Kwara	Ifelodun	22917	2005	94.7	84.9	105.2
Nigeria	Kwara	Ifelodun	22917	2010	85.0	75.6	95.2
Nigeria	Kwara	Ifelodun	22917	2015	74.3	65.6	85.3
Nigeria	Kwara	Ilorin East	191162	2000	93.4	83.9	104.7
Nigeria	Kwara	Ilorin East	191162	2005	84.5	75.8	94.0
Nigeria	Kwara	Ilorin East	191162	2010	78.0	69.4	88.0
Nigeria	Kwara	Ilorin East	191162	2015	66.0	57.1	76.1
Nigeria	Kwara	Ilorin South	191170	2000	90.8	81.6	101.5
Nigeria	Kwara	Ilorin South	191170	2005	83.7	75.3	93.0
Nigeria	Kwara	Ilorin South	191170	2010	77.2	68.6	86.9
Nigeria	Kwara	Ilorin South	191170	2015	65.3	56.6	75.2
Nigeria	Kwara	Ilorin West	191168	2000	87.6	78.5	98.2
Nigeria	Kwara	Ilorin West	191168	2005	81.1	72.5	90.5
Nigeria	Kwara	Ilorin West	191168	2010	75.6	66.9	85.1
Nigeria	Kwara	Ilorin West	191168	2015	64.2	55.7	74.4
Nigeria	Kwara	Irepodun	191176	2000	100.9	91.1	110.6
Nigeria	Kwara	Irepodun	191176	2005	92.4	83.5	101.7
Nigeria	Kwara	Irepodun	191176	2010	80.8	71.8	90.8
Nigeria	Kwara	Irepodun	191176	2015	67.6	59.2	78.0
Nigeria	Kwara	Isin	191177	2000	100.1	89.5	111.0
Nigeria	Kwara	Isin	191177	2005	91.9	82.6	102.2
Nigeria	Kwara	Isin	191177	2010	81.1	71.6	91.1
Nigeria	Kwara	Isin	191177	2015	68.0	59.2	78.5
Nigeria	Kwara	Kaiama	22921	2000	129.2	113.8	146.1
Nigeria	Kwara	Kaiama	22921	2005	115.7	102.5	129.9
Nigeria	Kwara	Kaiama	22921	2010	109.5	95.4	125.3
Nigeria	Kwara	Kaiama	22921	2015	101.4	87.1	117.7
Nigeria	Kwara	Moro	22922	2000	111.9	99.6	124.9
Nigeria	Kwara	Moro	22922	2005	99.6	89.4	111.6
Nigeria	Kwara	Moro	22922	2010	91.1	81.1	103.0
Nigeria	Kwara	Moro	22922	2015	82.3	71.8	94.7
Nigeria	Kwara	Offa	22923	2000	90.8	81.3	100.3
Nigeria	Kwara	Offa	22923	2005	84.0	75.7	93.3
Nigeria	Kwara	Offa	22923	2010	75.5	67.1	84.9
Nigeria	Kwara	Offa	22923	2015	63.8	55.6	73.7
Nigeria	Kwara	Oke-Ero	191179	2000	105.3	93.8	117.3
Nigeria	Kwara	Oke-Ero	191179	2005	96.3	86.4	107.0
Nigeria	Kwara	Oke-Ero	191179	2010	83.4	74.1	93.7
Nigeria	Kwara	Oke-Ero	191179	2015	69.5	60.4	79.5
Nigeria	Kwara	Oyun	22924	2000	98.6	88.8	108.3
Nigeria	Kwara	Oyun	22924	2005	90.4	81.7	100.0
Nigeria	Kwara	Oyun	22924	2010	78.9	70.3	88.0
Nigeria	Kwara	Oyun	22924	2015	65.9	57.7	75.5
Nigeria	Kwara	Pategi	191157	2000	120.1	106.3	136.3

Admin 0	Admin 1	Admin 2	GAUL Code	Year	Under-5 mortality (per 1,000 livebirths)		
					Estimate	Lower bound, 95% UI	Upper bound, 95% UI
Nigeria	Kwara	Pategi	191157	2005	103.3	91.6	116.3
Nigeria	Kwara	Pategi	191157	2010	94.7	82.8	107.1
Nigeria	Kwara	Pategi	191157	2015	85.8	74.6	99.4
Nigeria	Lagos	Agege	191287	2000	86.3	79.2	93.8
Nigeria	Lagos	Agege	191287	2005	85.7	79.3	92.6
Nigeria	Lagos	Agege	191287	2010	80.3	73.5	86.9
Nigeria	Lagos	Agege	191287	2015	65.8	58.0	74.3
Nigeria	Lagos	Ajeromi/ Ifelodun	191303	2000	86.9	79.8	94.7
Nigeria	Lagos	Ajeromi/ Ifelodun	191303	2005	86.7	79.9	93.8
Nigeria	Lagos	Ajeromi/ Ifelodun	191303	2010	82.9	75.9	90.2
Nigeria	Lagos	Ajeromi/ Ifelodun	191303	2015	67.5	59.4	76.8
Nigeria	Lagos	Alimosho	191285	2000	87.5	80.6	95.1
Nigeria	Lagos	Alimosho	191285	2005	90.3	83.5	97.4
Nigeria	Lagos	Alimosho	191285	2010	85.2	78.2	92.1
Nigeria	Lagos	Alimosho	191285	2015	67.5	59.7	76.5
Nigeria	Lagos	Amuwo Odofin	191300	2000	87.6	80.6	95.1
Nigeria	Lagos	Amuwo Odofin	191300	2005	88.7	82.0	95.8
Nigeria	Lagos	Amuwo Odofin	191300	2010	84.1	77.3	91.3
Nigeria	Lagos	Amuwo Odofin	191300	2015	67.9	59.6	76.9
Nigeria	Lagos	Apapa	191416	2000	87.3	80.1	95.5
Nigeria	Lagos	Apapa	191416	2005	85.6	78.6	92.8
Nigeria	Lagos	Apapa	191416	2010	82.0	74.7	89.7
Nigeria	Lagos	Apapa	191416	2015	67.4	59.2	77.0
Nigeria	Lagos	Badagry	22926	2000	94.4	86.8	102.5
Nigeria	Lagos	Badagry	22926	2005	96.8	89.3	105.0
Nigeria	Lagos	Badagry	22926	2010	88.1	81.1	95.9
Nigeria	Lagos	Badagry	22926	2015	74.0	65.8	83.3
Nigeria	Lagos	Epe	22927	2000	105.1	95.0	115.6
Nigeria	Lagos	Epe	22927	2005	107.0	97.1	119.3
Nigeria	Lagos	Epe	22927	2010	94.8	84.6	105.0
Nigeria	Lagos	Epe	22927	2015	76.4	67.1	87.0
Nigeria	Lagos	Eti-Osa	22928	2000	89.6	81.8	98.2
Nigeria	Lagos	Eti-Osa	22928	2005	90.0	82.3	98.5
Nigeria	Lagos	Eti-Osa	22928	2010	86.5	78.7	94.9
Nigeria	Lagos	Eti-Osa	22928	2015	68.5	60.1	78.0
Nigeria	Lagos	Ibeju Lekki	22929	2000	98.8	88.9	109.5
Nigeria	Lagos	Ibeju Lekki	22929	2005	104.2	94.2	116.8
Nigeria	Lagos	Ibeju Lekki	22929	2010	94.9	85.0	105.6
Nigeria	Lagos	Ibeju Lekki	22929	2015	76.3	66.6	86.7
Nigeria	Lagos	Ifako/Ijaye	191284	2000	87.8	80.3	95.5
Nigeria	Lagos	Ifako/Ijaye	191284	2005	91.6	84.8	99.1
Nigeria	Lagos	Ifako/Ijaye	191284	2010	85.3	77.9	92.6
Nigeria	Lagos	Ifako/Ijaye	191284	2015	67.8	59.8	76.5
Nigeria	Lagos	Ikeja	191288	2000	85.7	78.6	93.0
Nigeria	Lagos	Ikeja	191288	2005	86.1	79.6	92.9
Nigeria	Lagos	Ikeja	191288	2010	80.9	74.1	87.8
Nigeria	Lagos	Ikeja	191288	2015	65.9	58.1	74.5
Nigeria	Lagos	Ikorodu	22931	2000	89.1	81.6	97.1
Nigeria	Lagos	Ikorodu	22931	2005	93.4	86.1	101.5
Nigeria	Lagos	Ikorodu	22931	2010	87.5	79.6	95.2
Nigeria	Lagos	Ikorodu	22931	2015	68.7	60.4	77.9
Nigeria	Lagos	Kosofe	191291	2000	86.0	78.9	93.3
Nigeria	Lagos	Kosofe	191291	2005	88.0	81.5	95.1
Nigeria	Lagos	Kosofe	191291	2010	83.6	76.2	90.6
Nigeria	Lagos	Kosofe	191291	2015	66.9	58.9	75.8
Nigeria	Lagos	Lagos Island	191293	2000	87.5	80.1	95.3
Nigeria	Lagos	Lagos Island	191293	2005	88.5	81.6	95.9
Nigeria	Lagos	Lagos Island	191293	2010	84.6	77.4	92.1
Nigeria	Lagos	Lagos Island	191293	2015	66.6	58.3	75.7
Nigeria	Lagos	Lagos Mainland	191299	2000	86.2	79.1	93.8
Nigeria	Lagos	Lagos Mainland	191299	2005	86.2	79.5	93.3
Nigeria	Lagos	Lagos Mainland	191299	2010	82.1	74.9	89.1
Nigeria	Lagos	Lagos Mainland	191299	2015	66.0	57.8	75.1
Nigeria	Lagos	Mushin	191297	2000	85.7	78.7	92.9
Nigeria	Lagos	Mushin	191297	2005	84.4	78.0	91.3
Nigeria	Lagos	Mushin	191297	2010	80.5	73.6	87.3
Nigeria	Lagos	Mushin	191297	2015	65.7	57.8	74.5
Nigeria	Lagos	Ojo	191298	2000	88.4	81.3	95.8
Nigeria	Lagos	Ojo	191298	2005	88.9	81.9	96.6
Nigeria	Lagos	Ojo	191298	2010	83.4	76.8	90.6
Nigeria	Lagos	Ojo	191298	2015	68.2	60.3	77.1
Nigeria	Lagos	Oshodi/Isolo	191295	2000	86.6	79.5	94.0
Nigeria	Lagos	Oshodi/Isolo	191295	2005	86.3	79.8	93.2
Nigeria	Lagos	Oshodi/Isolo	191295	2010	81.4	74.8	88.1
Nigeria	Lagos	Oshodi/Isolo	191295	2015	66.7	58.8	75.8
Nigeria	Lagos	Shomolu	191296	2000	85.7	78.7	93.0
Nigeria	Lagos	Shomolu	191296	2005	85.9	79.4	92.9
Nigeria	Lagos	Shomolu	191296	2010	81.5	74.5	88.3
Nigeria	Lagos	Shomolu	191296	2015	66.2	58.1	75.0
Nigeria	Lagos	Surulere	23036	2000	86.4	79.2	94.0
Nigeria	Lagos	Surulere	23036	2005	84.7	78.3	91.7
Nigeria	Lagos	Surulere	23036	2010	80.7	73.7	87.6
Nigeria	Lagos	Surulere	23036	2015	66.4	58.4	75.4
Nigeria	Nassarawa	Akwanga	191146	2000	170.8	155.9	187.2
Nigeria	Nassarawa	Akwanga	191146	2005	150.9	137.3	165.9

Admin 0	Admin 1	Admin 2	GAUL Code	Year	Under-5 mortality (per 1,000 livebirths)		
					Estimate	Lower bound, 95% UI	Upper bound, 95% UI
Nigeria	Nassarawa	Akwanga	191146	2010	134.2	120.9	148.5
Nigeria	Nassarawa	Akwanga	191146	2015	114.5	100.7	129.7
Nigeria	Nassarawa	Awe	191171	2000	179.9	162.2	199.0
Nigeria	Nassarawa	Awe	191171	2005	158.2	144.3	173.9
Nigeria	Nassarawa	Awe	191171	2010	137.7	123.6	152.3
Nigeria	Nassarawa	Awe	191171	2015	127.0	110.7	144.6
Nigeria	Nassarawa	Doma	23041	2000	169.4	153.5	186.5
Nigeria	Nassarawa	Doma	23041	2005	144.6	131.3	160.2
Nigeria	Nassarawa	Doma	23041	2010	128.3	114.9	142.2
Nigeria	Nassarawa	Doma	23041	2015	113.8	99.1	129.8
Nigeria	Nassarawa	Karu	191136	2000	149.1	136.7	163.0
Nigeria	Nassarawa	Karu	191136	2005	127.8	117.4	139.3
Nigeria	Nassarawa	Karu	191136	2010	110.2	100.0	121.9
Nigeria	Nassarawa	Karu	191136	2015	92.8	82.3	105.1
Nigeria	Nassarawa	Keana	191180	2000	173.0	154.0	191.9
Nigeria	Nassarawa	Keana	191180	2005	149.4	135.1	166.5
Nigeria	Nassarawa	Keana	191180	2010	130.8	116.2	146.3
Nigeria	Nassarawa	Keana	191180	2015	119.6	103.3	137.8
Nigeria	Nassarawa	Keffi	191158	2000	148.9	133.9	165.5
Nigeria	Nassarawa	Keffi	191158	2005	126.8	113.9	141.4
Nigeria	Nassarawa	Keffi	191158	2010	111.5	99.1	124.2
Nigeria	Nassarawa	Keffi	191158	2015	91.8	79.9	104.4
Nigeria	Nassarawa	Kokona	191145	2000	168.2	153.1	185.1
Nigeria	Nassarawa	Kokona	191145	2005	145.2	132.5	160.0
Nigeria	Nassarawa	Kokona	191145	2010	125.9	113.3	138.3
Nigeria	Nassarawa	Kokona	191145	2015	109.8	96.3	123.7
Nigeria	Nassarawa	Lafia	191152	2000	168.9	154.6	184.6
Nigeria	Nassarawa	Lafia	191152	2005	147.4	135.4	160.5
Nigeria	Nassarawa	Lafia	191152	2010	131.2	118.8	144.1
Nigeria	Nassarawa	Lafia	191152	2015	115.6	102.2	130.7
Nigeria	Nassarawa	Nassarawa	191161	2000	153.2	138.8	167.4
Nigeria	Nassarawa	Nassarawa	191161	2005	129.3	117.6	142.0
Nigeria	Nassarawa	Nassarawa	191161	2010	114.6	102.7	126.6
Nigeria	Nassarawa	Nassarawa	191161	2015	99.6	87.3	112.6
Nigeria	Nassarawa	Nassarawa Egon	23049	2000	173.7	159.2	189.7
Nigeria	Nassarawa	Nassarawa Egon	23049	2005	151.5	138.9	165.3
Nigeria	Nassarawa	Nassarawa Egon	23049	2010	133.6	121.4	146.9
Nigeria	Nassarawa	Nassarawa Egon	23049	2015	120.6	106.4	136.4
Nigeria	Nassarawa	Obi	191172	2000	172.2	155.9	189.6
Nigeria	Nassarawa	Obi	191172	2005	149.0	135.3	164.0
Nigeria	Nassarawa	Obi	191172	2010	132.7	118.5	147.8
Nigeria	Nassarawa	Obi	191172	2015	121.9	106.5	138.7
Nigeria	Nassarawa	Toto	191165	2000	139.5	124.8	155.9
Nigeria	Nassarawa	Toto	191165	2005	117.9	104.4	131.5
Nigeria	Nassarawa	Toto	191165	2010	104.6	92.6	117.1
Nigeria	Nassarawa	Toto	191165	2015	85.5	73.9	97.4
Nigeria	Nassarawa	Wamba	191143	2000	175.9	160.7	192.0
Nigeria	Nassarawa	Wamba	191143	2005	156.8	142.7	171.7
Nigeria	Nassarawa	Wamba	191143	2010	139.1	126.2	153.8
Nigeria	Nassarawa	Wamba	191143	2015	127.1	112.4	144.8
Nigeria	Niger	Agaie	22937	2000	155.0	139.6	172.4
Nigeria	Niger	Agaie	22937	2005	131.9	119.4	146.2
Nigeria	Niger	Agaie	22937	2010	114.3	102.0	127.2
Nigeria	Niger	Agaie	22937	2015	103.5	89.3	117.8
Nigeria	Niger	Agwara	22938	2000	188.9	167.0	211.9
Nigeria	Niger	Agwara	22938	2005	173.9	154.9	193.1
Nigeria	Niger	Agwara	22938	2010	165.6	146.8	187.1
Nigeria	Niger	Agwara	22938	2015	154.4	133.8	178.7
Nigeria	Niger	Bida	191150	2000	135.6	120.9	151.2
Nigeria	Niger	Bida	191150	2005	120.5	107.8	134.8
Nigeria	Niger	Bida	191150	2010	108.2	96.0	122.8
Nigeria	Niger	Bida	191150	2015	98.4	85.1	113.5
Nigeria	Niger	Borgu	22940	2000	168.4	152.9	186.4
Nigeria	Niger	Borgu	22940	2005	152.3	136.9	166.9
Nigeria	Niger	Borgu	22940	2010	144.9	129.1	161.7
Nigeria	Niger	Borgu	22940	2015	136.3	118.5	155.6
Nigeria	Niger	Bosso	191118	2000	140.2	124.7	156.5
Nigeria	Niger	Bosso	191118	2005	120.0	107.6	134.0
Nigeria	Niger	Bosso	191118	2010	106.2	93.2	120.3
Nigeria	Niger	Bosso	191118	2015	96.8	83.7	112.0
Nigeria	Niger	Chanchaga	191124	2000	128.5	113.1	145.0
Nigeria	Niger	Chanchaga	191124	2005	109.4	96.9	123.7
Nigeria	Niger	Chanchaga	191124	2010	98.8	85.9	113.2
Nigeria	Niger	Chanchaga	191124	2015	88.7	75.7	103.9
Nigeria	Niger	Edati	191414	2000	144.1	128.5	162.4
Nigeria	Niger	Edati	191414	2005	126.7	112.8	141.0
Nigeria	Niger	Edati	191414	2010	112.7	99.8	127.6
Nigeria	Niger	Edati	191414	2015	102.8	89.5	119.7
Nigeria	Niger	Gbako	191129	2000	149.1	134.3	165.0
Nigeria	Niger	Gbako	191129	2005	130.8	118.0	144.1
Nigeria	Niger	Gbako	191129	2010	118.6	106.1	133.3
Nigeria	Niger	Gbako	191129	2015	108.4	95.2	124.7
Nigeria	Niger	Gurara	191132	2000	154.7	140.7	169.1
Nigeria	Niger	Gurara	191132	2005	131.0	119.4	143.8
Nigeria	Niger	Gurara	191132	2010	110.0	99.1	122.6

Admin 0	Admin 1	Admin 2	GAUL Code	Year	Under-5 mortality (per 1,000 livebirths)		
					Estimate	Lower bound, 95% UI	Upper bound, 95% UI
Nigeria	Niger	Gurara	191132	2015	99.6	86.9	112.9
Nigeria	Niger	Katcha	22939	2000	156.0	141.6	172.4
Nigeria	Niger	Katcha	22939	2005	134.8	122.9	148.0
Nigeria	Niger	Katcha	22939	2010	118.5	106.5	131.6
Nigeria	Niger	Katcha	22939	2015	108.0	94.6	122.5
Nigeria	Niger	Kontagora	191095	2000	145.5	127.7	167.2
Nigeria	Niger	Kontagora	191095	2005	132.2	116.5	149.7
Nigeria	Niger	Kontagora	191095	2010	123.5	107.5	142.2
Nigeria	Niger	Kontagora	191095	2015	111.7	95.7	130.0
Nigeria	Niger	Lapai	22945	2000	150.6	137.4	165.9
Nigeria	Niger	Lapai	22945	2005	126.6	114.2	139.7
Nigeria	Niger	Lapai	22945	2010	108.6	96.7	120.6
Nigeria	Niger	Lapai	22945	2015	98.1	85.9	111.4
Nigeria	Niger	Lavun	191422	2000	151.6	137.1	167.7
Nigeria	Niger	Lavun	191422	2005	132.9	120.7	146.8
Nigeria	Niger	Lavun	191422	2010	120.0	107.6	134.6
Nigeria	Niger	Lavun	191422	2015	110.6	97.4	126.2
Nigeria	Niger	Magama	22947	2000	179.3	159.1	203.1
Nigeria	Niger	Magama	22947	2005	163.4	146.5	182.8
Nigeria	Niger	Magama	22947	2010	150.6	133.4	170.1
Nigeria	Niger	Magama	22947	2015	143.7	124.8	166.2
Nigeria	Niger	Mariga	191088	2000	173.8	156.8	192.9
Nigeria	Niger	Mariga	191088	2005	152.6	138.6	169.1
Nigeria	Niger	Mariga	191088	2010	140.5	125.5	157.2
Nigeria	Niger	Mariga	191088	2015	138.0	120.7	159.6
Nigeria	Niger	Mashegu	191105	2000	171.6	152.1	192.5
Nigeria	Niger	Mashegu	191105	2005	153.7	136.5	173.1
Nigeria	Niger	Mashegu	191105	2010	140.7	124.2	158.5
Nigeria	Niger	Mashegu	191105	2015	131.6	115.4	152.3
Nigeria	Niger	Mokwa	191127	2000	142.9	127.0	161.4
Nigeria	Niger	Mokwa	191127	2005	126.3	111.8	141.6
Nigeria	Niger	Mokwa	191127	2010	115.1	100.6	131.0
Nigeria	Niger	Mokwa	191127	2015	104.9	91.7	121.1
Nigeria	Niger	Muya	191109	2000	163.1	146.3	181.6
Nigeria	Niger	Muya	191109	2005	136.4	122.3	152.3
Nigeria	Niger	Muya	191109	2010	114.5	102.5	128.5
Nigeria	Niger	Muya	191109	2015	106.7	92.2	122.8
Nigeria	Niger	Paikoro	22949	2000	157.6	144.1	172.7
Nigeria	Niger	Paikoro	22949	2005	134.3	121.7	147.2
Nigeria	Niger	Paikoro	22949	2010	114.5	102.7	127.5
Nigeria	Niger	Paikoro	22949	2015	104.4	91.0	118.8
Nigeria	Niger	Rafi	22950	2000	162.1	144.7	181.3
Nigeria	Niger	Rafi	22950	2005	140.4	125.5	155.9
Nigeria	Niger	Rafi	22950	2010	123.6	109.4	139.7
Nigeria	Niger	Rafi	22950	2015	121.4	105.1	139.7
Nigeria	Niger	Rijau	22951	2000	174.1	157.3	192.3
Nigeria	Niger	Rijau	22951	2005	156.6	142.6	171.9
Nigeria	Niger	Rijau	22951	2010	146.1	131.3	161.9
Nigeria	Niger	Rijau	22951	2015	137.5	120.9	156.6
Nigeria	Niger	Shiroro	191098	2000	161.2	145.7	179.0
Nigeria	Niger	Shiroro	191098	2005	137.0	123.5	151.6
Nigeria	Niger	Shiroro	191098	2010	116.5	103.2	129.8
Nigeria	Niger	Shiroro	191098	2015	111.5	96.6	127.1
Nigeria	Niger	Suleja	191140	2000	141.3	128.2	154.1
Nigeria	Niger	Suleja	191140	2005	122.4	111.2	133.8
Nigeria	Niger	Suleja	191140	2010	103.2	93.1	114.3
Nigeria	Niger	Suleja	191140	2015	88.0	76.6	100.3
Nigeria	Niger	Tafa	191138	2000	136.1	123.2	148.0
Nigeria	Niger	Tafa	191138	2005	115.0	104.7	125.1
Nigeria	Niger	Tafa	191138	2010	98.8	89.3	109.7
Nigeria	Niger	Tafa	191138	2015	84.1	73.4	95.7
Nigeria	Niger	Wushishi	191114	2000	162.2	145.1	181.0
Nigeria	Niger	Wushishi	191114	2005	142.5	128.2	159.1
Nigeria	Niger	Wushishi	191114	2010	128.7	113.7	145.9
Nigeria	Niger	Wushishi	191114	2015	118.9	103.6	136.6
Nigeria	Ogun	Abeokuta North	191237	2000	116.3	106.0	128.8
Nigeria	Ogun	Abeokuta North	191237	2005	105.3	95.6	116.0
Nigeria	Ogun	Abeokuta North	191237	2010	90.2	81.8	99.4
Nigeria	Ogun	Abeokuta North	191237	2015	81.1	71.4	91.6
Nigeria	Ogun	Abeokuta South	191253	2000	98.8	88.5	110.3
Nigeria	Ogun	Abeokuta South	191253	2005	95.7	86.0	106.0
Nigeria	Ogun	Abeokuta South	191253	2010	82.9	74.6	92.1
Nigeria	Ogun	Abeokuta South	191253	2015	69.9	60.8	79.4
Nigeria	Ogun	Ado Odo/Ota	22956	2000	96.8	89.4	104.6
Nigeria	Ogun	Ado Odo/Ota	22956	2005	98.8	91.6	106.0
Nigeria	Ogun	Ado Odo/Ota	22956	2010	88.9	81.7	96.1
Nigeria	Ogun	Ado Odo/Ota	22956	2015	73.1	65.1	82.2
Nigeria	Ogun	Egbado /Yewa North	191234	2000	124.5	115.1	134.9
Nigeria	Ogun	Egbado /Yewa North	191234	2005	111.0	101.8	120.6
Nigeria	Ogun	Egbado /Yewa North	191234	2010	94.6	86.3	102.6
Nigeria	Ogun	Egbado /Yewa North	191234	2015	89.6	79.4	100.8
Nigeria	Ogun	Egbado /Yewa South	191268	2000	114.5	106.4	123.3
Nigeria	Ogun	Egbado /Yewa South	191268	2005	107.3	99.4	115.5
Nigeria	Ogun	Egbado /Yewa South	191268	2010	92.4	85.1	100.0
Nigeria	Ogun	Egbado /Yewa South	191268	2015	84.0	74.8	93.9

Admin 0	Admin 1	Admin 2	GAUL Code	Year	Under-5 mortality (per 1,000 livebirths)		
					Estimate	Lower bound, 95% UI	Upper bound, 95% UI
Nigeria	Ogun	Ewekoro	191259	2000	117.2	107.7	127.8
Nigeria	Ogun	Ewekoro	191259	2005	109.2	100.2	119.4
Nigeria	Ogun	Ewekoro	191259	2010	92.7	84.7	101.2
Nigeria	Ogun	Ewekoro	191259	2015	79.8	70.4	89.3
Nigeria	Ogun	Ifo	191274	2000	95.0	87.2	102.9
Nigeria	Ogun	Ifo	191274	2005	97.3	90.2	104.9
Nigeria	Ogun	Ifo	191274	2010	88.0	80.7	95.3
Nigeria	Ogun	Ifo	191274	2015	70.9	62.7	79.5
Nigeria	Ogun	Ijebu East	22960	2000	121.5	109.9	133.8
Nigeria	Ogun	Ijebu East	22960	2005	114.6	102.9	128.1
Nigeria	Ogun	Ijebu East	22960	2010	98.1	88.4	109.3
Nigeria	Ogun	Ijebu East	22960	2015	80.9	71.3	92.7
Nigeria	Ogun	Ijebu North	22961	2000	111.7	101.1	123.5
Nigeria	Ogun	Ijebu North	22961	2005	105.1	95.1	116.3
Nigeria	Ogun	Ijebu North	22961	2010	90.0	80.4	99.5
Nigeria	Ogun	Ijebu North	22961	2015	74.1	65.4	84.5
Nigeria	Ogun	Ijebu North-East	191271	2000	116.6	105.2	129.5
Nigeria	Ogun	Ijebu North-East	191271	2005	110.0	98.5	123.2
Nigeria	Ogun	Ijebu North-East	191271	2010	94.3	84.0	104.6
Nigeria	Ogun	Ijebu North-East	191271	2015	78.2	68.5	89.3
Nigeria	Ogun	Ijebu-Ode	191273	2000	106.7	96.4	118.3
Nigeria	Ogun	Ijebu-Ode	191273	2005	104.1	93.8	116.1
Nigeria	Ogun	Ijebu-Ode	191273	2010	91.1	81.4	101.5
Nigeria	Ogun	Ijebu-Ode	191273	2015	73.2	64.0	83.7
Nigeria	Ogun	Ikenne	191266	2000	108.0	98.8	118.0
Nigeria	Ogun	Ikenne	191266	2005	104.6	95.7	113.9
Nigeria	Ogun	Ikenne	191266	2010	89.0	80.0	97.4
Nigeria	Ogun	Ikenne	191266	2015	72.3	63.2	81.1
Nigeria	Ogun	Imeko-Afon	191404	2000	119.2	108.2	131.7
Nigeria	Ogun	Imeko-Afon	191404	2005	104.2	93.9	114.7
Nigeria	Ogun	Imeko-Afon	191404	2010	90.6	80.7	100.4
Nigeria	Ogun	Imeko-Afon	191404	2015	85.7	75.3	97.1
Nigeria	Ogun	Ipokia	191278	2000	120.7	113.2	128.7
Nigeria	Ogun	Ipokia	191278	2005	112.5	105.5	120.0
Nigeria	Ogun	Ipokia	191278	2010	96.1	89.2	103.4
Nigeria	Ogun	Ipokia	191278	2015	89.6	80.1	99.6
Nigeria	Ogun	Obafemi-Owode	22963	2000	114.3	104.2	125.1
Nigeria	Ogun	Obafemi-Owode	22963	2005	107.2	98.5	116.8
Nigeria	Ogun	Obafemi-Owode	22963	2010	90.8	82.5	99.6
Nigeria	Ogun	Obafemi-Owode	22963	2015	76.3	67.0	85.5
Nigeria	Ogun	Odeda	22964	2000	117.3	106.3	129.4
Nigeria	Ogun	Odeda	22964	2005	106.4	96.5	116.7
Nigeria	Ogun	Odeda	22964	2010	89.1	80.7	97.5
Nigeria	Ogun	Odeda	22964	2015	77.0	67.5	86.7
Nigeria	Ogun	Odogbolu	191272	2000	111.2	101.4	122.0
Nigeria	Ogun	Odogbolu	191272	2005	107.9	98.2	118.6
Nigeria	Ogun	Odogbolu	191272	2010	92.9	83.6	101.8
Nigeria	Ogun	Odogbolu	191272	2015	75.2	66.2	85.0
Nigeria	Ogun	Ogun Waterside	22965	2000	128.4	113.6	144.2
Nigeria	Ogun	Ogun Waterside	22965	2005	121.2	107.1	136.2
Nigeria	Ogun	Ogun Waterside	22965	2010	106.5	94.0	120.1
Nigeria	Ogun	Ogun Waterside	22965	2015	89.7	76.7	103.3
Nigeria	Ogun	Remo-North	191262	2000	113.5	103.4	124.0
Nigeria	Ogun	Remo-North	191262	2005	106.2	97.3	116.0
Nigeria	Ogun	Remo-North	191262	2010	90.4	81.0	99.3
Nigeria	Ogun	Remo-North	191262	2015	75.2	65.9	84.2
Nigeria	Ogun	Shagamu	191269	2000	100.4	92.0	109.3
Nigeria	Ogun	Shagamu	191269	2005	101.9	93.7	111.5
Nigeria	Ogun	Shagamu	191269	2010	89.6	81.1	97.7
Nigeria	Ogun	Shagamu	191269	2015	71.9	62.9	80.7
Nigeria	Ondo	Akoko North-East	22968	2000	104.1	94.7	114.5
Nigeria	Ondo	Akoko North-East	22968	2005	98.4	89.4	107.7
Nigeria	Ondo	Akoko North-East	22968	2010	84.1	75.5	92.7
Nigeria	Ondo	Akoko North-East	22968	2015	63.5	55.6	72.7
Nigeria	Ondo	Akoko North-West	22969	2000	103.7	93.8	114.8
Nigeria	Ondo	Akoko North-West	22969	2005	98.1	88.9	108.3
Nigeria	Ondo	Akoko North-West	22969	2010	84.0	75.1	93.2
Nigeria	Ondo	Akoko North-West	22969	2015	63.6	55.5	72.8
Nigeria	Ondo	Akoko South-East	191225	2000	108.6	98.0	120.7
Nigeria	Ondo	Akoko South-East	191225	2005	101.6	91.7	112.0
Nigeria	Ondo	Akoko South-East	191225	2010	87.4	78.2	96.8
Nigeria	Ondo	Akoko South-East	191225	2015	65.4	57.2	75.4
Nigeria	Ondo	Akoko South-West	191229	2000	109.7	99.6	120.1
Nigeria	Ondo	Akoko South-West	191229	2005	102.9	93.7	113.3
Nigeria	Ondo	Akoko South-West	191229	2010	88.3	78.9	97.4
Nigeria	Ondo	Akoko South-West	191229	2015	66.1	57.8	75.6
Nigeria	Ondo	Akure North	191236	2000	105.6	96.1	116.7
Nigeria	Ondo	Akure North	191236	2005	101.1	91.2	112.1
Nigeria	Ondo	Akure North	191236	2010	86.8	77.8	96.5
Nigeria	Ondo	Akure North	191236	2015	65.7	57.0	75.3
Nigeria	Ondo	Akure South	191245	2000	99.7	90.2	110.0
Nigeria	Ondo	Akure South	191245	2005	96.7	87.1	106.9
Nigeria	Ondo	Akure South	191245	2010	83.2	74.1	92.6
Nigeria	Ondo	Akure South	191245	2015	63.6	55.1	73.0
Nigeria	Ondo	Ese-Odo	191305	2000	130.4	115.5	146.0

Admin 0	Admin 1	Admin 2	GAUL Code	Year	Under-5 mortality (per 1,000 livebirths)		
					Estimate	Lower bound, 95% UI	Upper bound, 95% UI
Nigeria	Ondo	Ese-Odo	191305	2005	121.1	107.9	135.4
Nigeria	Ondo	Ese-Odo	191305	2010	109.3	96.7	122.0
Nigeria	Ondo	Ese-Odo	191305	2015	93.7	81.9	106.8
Nigeria	Ondo	Idanre	22976	2000	115.6	105.3	126.4
Nigeria	Ondo	Idanre	22976	2005	108.7	98.2	119.6
Nigeria	Ondo	Idanre	22976	2010	94.3	84.9	104.6
Nigeria	Ondo	Idanre	22976	2015	72.8	63.4	83.3
Nigeria	Ondo	Ifedore	22978	2000	105.6	96.0	116.1
Nigeria	Ondo	Ifedore	22978	2005	100.6	91.1	110.4
Nigeria	Ondo	Ifedore	22978	2010	85.2	76.6	94.2
Nigeria	Ondo	Ifedore	22978	2015	65.4	57.3	74.5
Nigeria	Ondo	Ilaje	191290	2000	128.3	114.3	144.5
Nigeria	Ondo	Ilaje	191290	2005	121.3	107.9	135.9
Nigeria	Ondo	Ilaje	191290	2010	110.2	97.3	123.9
Nigeria	Ondo	Ilaje	191290	2015	94.7	81.9	108.0
Nigeria	Ondo	Ile-Oluji/Okeigbo	191240	2000	110.2	99.2	121.3
Nigeria	Ondo	Ile-Oluji/Okeigbo	191240	2005	104.1	93.3	114.9
Nigeria	Ondo	Ile-Oluji/Okeigbo	191240	2010	88.7	79.4	98.5
Nigeria	Ondo	Ile-Oluji/Okeigbo	191240	2015	69.9	61.3	79.7
Nigeria	Ondo	Irele	22985	2000	125.6	113.3	140.4
Nigeria	Ondo	Irele	22985	2005	117.0	105.2	130.4
Nigeria	Ondo	Irele	22985	2010	104.6	92.7	116.8
Nigeria	Ondo	Irele	22985	2015	87.1	75.9	99.0
Nigeria	Ondo	Odigbo	22987	2000	119.3	108.1	131.9
Nigeria	Ondo	Odigbo	22987	2005	113.7	102.5	125.7
Nigeria	Ondo	Odigbo	22987	2010	99.2	89.3	109.7
Nigeria	Ondo	Odigbo	22987	2015	80.3	70.3	91.3
Nigeria	Ondo	Okitipupa	22980	2000	120.0	107.5	135.1
Nigeria	Ondo	Okitipupa	22980	2005	113.6	101.5	126.9
Nigeria	Ondo	Okitipupa	22980	2010	102.1	90.1	113.9
Nigeria	Ondo	Okitipupa	22980	2015	85.0	73.1	98.3
Nigeria	Ondo	Ondo East	191254	2000	113.6	102.5	124.8
Nigeria	Ondo	Ondo East	191254	2005	107.6	96.1	118.4
Nigeria	Ondo	Ondo East	191254	2010	92.3	82.5	102.3
Nigeria	Ondo	Ondo East	191254	2015	72.3	63.1	82.7
Nigeria	Ondo	Ondo West	191256	2000	101.7	91.7	112.2
Nigeria	Ondo	Ondo West	191256	2005	97.2	86.6	107.0
Nigeria	Ondo	Ondo West	191256	2010	85.8	76.8	95.6
Nigeria	Ondo	Ondo West	191256	2015	66.9	58.0	77.2
Nigeria	Ondo	Ose	191239	2000	116.8	106.2	128.4
Nigeria	Ondo	Ose	191239	2005	108.1	98.2	119.2
Nigeria	Ondo	Ose	191239	2010	93.9	84.4	103.5
Nigeria	Ondo	Ose	191239	2015	69.9	61.3	80.7
Nigeria	Ondo	Owo	191249	2000	112.5	100.6	124.6
Nigeria	Ondo	Owo	191249	2005	106.3	95.2	119.3
Nigeria	Ondo	Owo	191249	2010	91.6	81.3	102.5
Nigeria	Ondo	Owo	191249	2015	68.4	59.3	79.3
Nigeria	Osun	Aiyedaade	22993	2000	89.3	80.1	98.3
Nigeria	Osun	Aiyedaade	22993	2005	83.9	75.9	92.8
Nigeria	Osun	Aiyedaade	22993	2010	73.1	65.2	82.2
Nigeria	Osun	Aiyedaade	22993	2015	58.7	51.3	68.1
Nigeria	Osun	Aiyedire	22994	2000	91.0	82.3	100.0
Nigeria	Osun	Aiyedire	22994	2005	86.1	78.3	94.9
Nigeria	Osun	Aiyedire	22994	2010	75.1	67.4	83.6
Nigeria	Osun	Aiyedire	22994	2015	61.2	54.0	70.0
Nigeria	Osun	Atakumosa East	191222	2000	100.7	91.3	110.4
Nigeria	Osun	Atakumosa East	191222	2005	96.3	86.6	105.7
Nigeria	Osun	Atakumosa East	191222	2010	83.7	75.3	92.9
Nigeria	Osun	Atakumosa East	191222	2015	65.2	57.0	74.3
Nigeria	Osun	Atakumosa West	191208	2000	95.4	87.1	104.1
Nigeria	Osun	Atakumosa West	191208	2005	90.3	82.8	98.8
Nigeria	Osun	Atakumosa West	191208	2010	77.9	70.5	86.5
Nigeria	Osun	Atakumosa West	191208	2015	60.4	53.3	68.3
Nigeria	Osun	Boluwaduro	191193	2000	86.4	78.1	94.8
Nigeria	Osun	Boluwaduro	191193	2005	83.0	75.4	91.2
Nigeria	Osun	Boluwaduro	191193	2010	73.1	65.6	81.2
Nigeria	Osun	Boluwaduro	191193	2015	57.9	51.0	66.5
Nigeria	Osun	Boripe	22995	2000	84.9	76.8	92.8
Nigeria	Osun	Boripe	22995	2005	81.3	73.9	89.4
Nigeria	Osun	Boripe	22995	2010	72.1	65.0	80.0
Nigeria	Osun	Boripe	22995	2015	56.8	50.0	65.3
Nigeria	Osun	Ede North	191206	2000	88.8	80.0	97.2
Nigeria	Osun	Ede North	191206	2005	83.8	75.7	91.5
Nigeria	Osun	Ede North	191206	2010	72.2	64.3	80.4
Nigeria	Osun	Ede North	191206	2015	57.8	51.2	65.8
Nigeria	Osun	Ede South	191211	2000	89.6	80.7	97.8
Nigeria	Osun	Ede South	191211	2005	84.3	76.3	92.3
Nigeria	Osun	Ede South	191211	2010	73.3	65.7	81.8
Nigeria	Osun	Ede South	191211	2015	58.4	51.6	66.8
Nigeria	Osun	Egbedore	191198	2000	82.7	74.4	90.9
Nigeria	Osun	Egbedore	191198	2005	79.0	70.7	86.4
Nigeria	Osun	Egbedore	191198	2010	70.1	62.6	78.4
Nigeria	Osun	Egbedore	191198	2015	55.6	48.9	64.0
Nigeria	Osun	Ejigbo	22998	2000	85.2	76.6	94.0
Nigeria	Osun	Ejigbo	22998	2005	80.3	72.9	88.3

Admin 0	Admin 1	Admin 2	GAUL Code	Year	Under-5 mortality (per 1,000 livebirths)		
					Estimate	Lower bound, 95% UI	Upper bound, 95% UI
Nigeria	Osun	Ejigbo	22998	2010	71.6	64.0	79.9
Nigeria	Osun	Ejigbo	22998	2015	58.9	51.7	67.4
Nigeria	Osun	Ife Central	191226	2000	91.2	82.6	100.2
Nigeria	Osun	Ife Central	191226	2005	86.7	78.7	95.1
Nigeria	Osun	Ife Central	191226	2010	74.6	67.0	83.7
Nigeria	Osun	Ife Central	191226	2015	58.7	51.8	67.1
Nigeria	Osun	Ife East	191232	2000	98.3	88.7	107.8
Nigeria	Osun	Ife East	191232	2005	93.4	85.2	102.5
Nigeria	Osun	Ife East	191232	2010	80.7	72.7	90.4
Nigeria	Osun	Ife East	191232	2015	62.2	54.8	70.8
Nigeria	Osun	Ife North	23001	2000	86.6	78.5	94.9
Nigeria	Osun	Ife North	23001	2005	82.8	75.1	90.8
Nigeria	Osun	Ife North	23001	2010	72.5	65.2	81.1
Nigeria	Osun	Ife North	23001	2015	56.4	49.8	64.5
Nigeria	Osun	Ife South	23002	2000	103.8	93.9	114.1
Nigeria	Osun	Ife South	23002	2005	98.2	88.2	108.3
Nigeria	Osun	Ife South	23002	2010	85.0	76.5	95.1
Nigeria	Osun	Ife South	23002	2015	67.3	59.2	76.9
Nigeria	Osun	Ifedayo	191190	2000	90.2	81.1	99.8
Nigeria	Osun	Ifedayo	191190	2005	86.5	77.5	96.4
Nigeria	Osun	Ifedayo	191190	2010	75.9	67.5	85.1
Nigeria	Osun	Ifedayo	191190	2015	59.8	52.2	68.9
Nigeria	Osun	Ifelodun	22917	2000	81.7	73.9	89.4
Nigeria	Osun	Ifelodun	22917	2005	78.9	71.8	86.8
Nigeria	Osun	Ifelodun	22917	2010	70.3	63.1	78.1
Nigeria	Osun	Ifelodun	22917	2015	55.2	48.6	63.7
Nigeria	Osun	Ila	191187	2000	86.7	78.4	95.2
Nigeria	Osun	Ila	191187	2005	83.3	75.1	92.1
Nigeria	Osun	Ila	191187	2010	73.5	65.8	82.0
Nigeria	Osun	Ila	191187	2015	58.1	50.8	66.9
Nigeria	Osun	Ilesha East	191215	2000	87.8	79.7	96.6
Nigeria	Osun	Ilesha East	191215	2005	85.0	77.0	93.7
Nigeria	Osun	Ilesha East	191215	2010	73.3	65.5	81.5
Nigeria	Osun	Ilesha East	191215	2015	57.3	50.4	65.3
Nigeria	Osun	Ilesha West	191214	2000	84.7	76.9	93.1
Nigeria	Osun	Ilesha West	191214	2005	82.2	74.6	90.0
Nigeria	Osun	Ilesha West	191214	2010	71.9	64.6	79.8
Nigeria	Osun	Ilesha West	191214	2015	56.5	49.9	64.0
Nigeria	Osun	Irepodun	191196	2000	83.9	75.2	92.3
Nigeria	Osun	Irepodun	191196	2005	79.6	71.2	87.8
Nigeria	Osun	Irepodun	191196	2010	70.3	62.5	78.7
Nigeria	Osun	Irepodun	191196	2015	56.0	49.1	64.6
Nigeria	Osun	Irewole	191230	2000	89.7	81.2	98.6
Nigeria	Osun	Irewole	191230	2005	85.1	77.0	93.9
Nigeria	Osun	Irewole	191230	2010	74.4	66.6	83.1
Nigeria	Osun	Irewole	191230	2015	60.8	53.5	69.4
Nigeria	Osun	Isokan	191248	2000	100.5	91.1	109.9
Nigeria	Osun	Isokan	191248	2005	94.9	86.1	104.3
Nigeria	Osun	Isokan	191248	2010	81.2	73.0	90.5
Nigeria	Osun	Isokan	191248	2015	66.2	58.4	75.3
Nigeria	Osun	Iwo	23006	2000	85.4	77.5	94.1
Nigeria	Osun	Iwo	23006	2005	80.9	73.4	89.0
Nigeria	Osun	Iwo	23006	2010	71.4	64.1	78.7
Nigeria	Osun	Iwo	23006	2015	58.6	51.1	66.9
Nigeria	Osun	Obokun	23007	2000	90.6	82.3	98.9
Nigeria	Osun	Obokun	23007	2005	86.4	78.9	94.6
Nigeria	Osun	Obokun	23007	2010	74.9	67.3	82.8
Nigeria	Osun	Obokun	23007	2015	58.5	51.8	67.0
Nigeria	Osun	Odo-Otin	23008	2000	85.2	76.5	93.7
Nigeria	Osun	Odo-Otin	23008	2005	80.8	73.0	89.1
Nigeria	Osun	Odo-Otin	23008	2010	71.6	64.1	80.1
Nigeria	Osun	Odo-Otin	23008	2015	57.0	49.9	65.9
Nigeria	Osun	Ola-Oluwa	23009	2000	88.8	80.8	97.6
Nigeria	Osun	Ola-Oluwa	23009	2005	83.5	76.0	91.5
Nigeria	Osun	Ola-Oluwa	23009	2010	73.7	65.7	82.0
Nigeria	Osun	Ola-Oluwa	23009	2015	61.0	53.3	69.5
Nigeria	Osun	Olorunda	23010	2000	85.1	76.4	93.1
Nigeria	Osun	Olorunda	23010	2005	80.8	73.4	88.7
Nigeria	Osun	Olorunda	23010	2010	71.0	63.7	79.1
Nigeria	Osun	Olorunda	23010	2015	56.0	49.5	64.6
Nigeria	Osun	Oriade	23011	2000	96.3	88.2	105.2
Nigeria	Osun	Oriade	23011	2005	92.2	84.1	101.0
Nigeria	Osun	Oriade	23011	2010	78.9	71.3	87.1
Nigeria	Osun	Oriade	23011	2015	60.8	53.6	69.1
Nigeria	Osun	Orolu	22920	2000	82.2	73.7	90.4
Nigeria	Osun	Orolu	22920	2005	78.2	69.9	86.2
Nigeria	Osun	Orolu	22920	2010	69.6	61.8	77.6
Nigeria	Osun	Orolu	22920	2015	55.4	48.6	63.9
Nigeria	Osun	Osogbo	23012	2000	80.8	72.4	88.8
Nigeria	Osun	Osogbo	23012	2005	77.6	69.8	85.0
Nigeria	Osun	Osogbo	23012	2010	69.0	61.4	77.2
Nigeria	Osun	Osogbo	23012	2015	54.8	48.2	62.9
Nigeria	Oyo	Afijio	23013	2000	90.7	81.1	101.9
Nigeria	Oyo	Afijio	23013	2005	85.4	76.3	96.1
Nigeria	Oyo	Afijio	23013	2010	75.2	66.3	84.6

Admin 0	Admin 1	Admin 2	GAUL Code	Year	Under-5 mortality (per 1,000 livebirths)		
					Estimate	Lower bound, 95% UI	Upper bound, 95% UI
Nigeria	Oyo	Afijio	23013	2015	63.2	54.3	73.2
Nigeria	Oyo	Akinyele	191213	2000	92.8	84.5	102.3
Nigeria	Oyo	Akinyele	191213	2005	88.0	80.3	96.2
Nigeria	Oyo	Akinyele	191213	2010	76.6	69.1	84.2
Nigeria	Oyo	Akinyele	191213	2015	63.5	55.5	72.0
Nigeria	Oyo	Atiba	191166	2000	92.8	83.0	104.5
Nigeria	Oyo	Atiba	191166	2005	86.5	77.3	96.5
Nigeria	Oyo	Atiba	191166	2010	77.5	68.4	86.9
Nigeria	Oyo	Atiba	191166	2015	66.9	58.0	76.4
Nigeria	Oyo	Atisbo	191167	2000	103.5	92.8	115.2
Nigeria	Oyo	Atisbo	191167	2005	91.2	82.0	101.4
Nigeria	Oyo	Atisbo	191167	2010	82.2	73.4	92.2
Nigeria	Oyo	Atisbo	191167	2015	74.6	64.9	85.2
Nigeria	Oyo	Egbeda	191231	2000	93.2	85.3	101.9
Nigeria	Oyo	Egbeda	191231	2005	88.2	80.7	96.1
Nigeria	Oyo	Egbeda	191231	2010	76.5	69.3	84.3
Nigeria	Oyo	Egbeda	191231	2015	63.2	55.6	71.8
Nigeria	Oyo	Ibadan North	191233	2000	89.9	81.4	99.6
Nigeria	Oyo	Ibadan North	191233	2005	85.9	78.0	94.0
Nigeria	Oyo	Ibadan North	191233	2010	75.5	67.9	83.1
Nigeria	Oyo	Ibadan North	191233	2015	62.2	54.6	70.4
Nigeria	Oyo	Ibadan North-East	191238	2000	91.1	82.5	100.7
Nigeria	Oyo	Ibadan North-East	191238	2005	86.5	78.6	94.4
Nigeria	Oyo	Ibadan North-East	191238	2010	75.4	68.1	82.9
Nigeria	Oyo	Ibadan North-East	191238	2015	62.2	54.7	70.4
Nigeria	Oyo	Ibadan North-West	191235	2000	90.5	81.7	100.0
Nigeria	Oyo	Ibadan North-West	191235	2005	86.7	78.5	94.8
Nigeria	Oyo	Ibadan North-West	191235	2010	75.5	67.9	83.1
Nigeria	Oyo	Ibadan North-West	191235	2015	62.3	54.8	70.5
Nigeria	Oyo	Ibadan South-East	191244	2000	90.9	82.3	100.6
Nigeria	Oyo	Ibadan South-East	191244	2005	86.9	78.8	94.9
Nigeria	Oyo	Ibadan South-East	191244	2010	75.7	68.1	83.3
Nigeria	Oyo	Ibadan South-East	191244	2015	62.1	54.8	70.2
Nigeria	Oyo	Ibadan South-West	191242	2000	91.9	83.2	101.7
Nigeria	Oyo	Ibadan South-West	191242	2005	87.6	79.2	95.7
Nigeria	Oyo	Ibadan South-West	191242	2010	76.2	68.6	83.9
Nigeria	Oyo	Ibadan South-West	191242	2015	62.8	55.2	71.0
Nigeria	Oyo	Ibarapa Central	191227	2000	106.4	95.3	119.7
Nigeria	Oyo	Ibarapa Central	191227	2005	96.5	85.9	108.2
Nigeria	Oyo	Ibarapa Central	191227	2010	84.8	75.5	95.2
Nigeria	Oyo	Ibarapa Central	191227	2015	72.6	63.0	83.4
Nigeria	Oyo	Ibarapa East	23021	2000	105.8	94.2	119.1
Nigeria	Oyo	Ibarapa East	23021	2005	95.8	85.5	107.7
Nigeria	Oyo	Ibarapa East	23021	2010	83.1	74.0	94.2
Nigeria	Oyo	Ibarapa East	23021	2015	71.0	61.7	81.4
Nigeria	Oyo	Ibarapa North	191201	2000	108.7	97.3	120.7
Nigeria	Oyo	Ibarapa North	191201	2005	96.4	85.8	107.5
Nigeria	Oyo	Ibarapa North	191201	2010	84.9	75.2	95.3
Nigeria	Oyo	Ibarapa North	191201	2015	75.6	65.5	86.6
Nigeria	Oyo	Ido	191207	2000	101.1	91.9	112.1
Nigeria	Oyo	Ido	191207	2005	94.3	85.4	103.1
Nigeria	Oyo	Ido	191207	2010	80.8	72.7	88.9
Nigeria	Oyo	Ido	191207	2015	67.8	59.9	76.5
Nigeria	Oyo	Irepo	191144	2000	102.4	90.2	117.4
Nigeria	Oyo	Irepo	191144	2005	90.8	80.4	103.4
Nigeria	Oyo	Irepo	191144	2010	86.0	75.2	98.3
Nigeria	Oyo	Irepo	191144	2015	77.5	66.5	90.1
Nigeria	Oyo	Iseyin	191188	2000	92.9	82.4	104.3
Nigeria	Oyo	Iseyin	191188	2005	85.6	75.9	96.2
Nigeria	Oyo	Iseyin	191188	2010	76.7	68.2	86.5
Nigeria	Oyo	Iseyin	191188	2015	65.5	56.5	75.4
Nigeria	Oyo	Itesiwaju	191175	2000	97.7	86.8	109.6
Nigeria	Oyo	Itesiwaju	191175	2005	88.3	78.1	99.3
Nigeria	Oyo	Itesiwaju	191175	2010	79.1	69.8	89.9
Nigeria	Oyo	Itesiwaju	191175	2015	69.2	59.5	79.3
Nigeria	Oyo	Iwajowa	191182	2000	107.8	96.0	120.7
Nigeria	Oyo	Iwajowa	191182	2005	94.1	83.0	105.9
Nigeria	Oyo	Iwajowa	191182	2010	83.9	73.8	95.5
Nigeria	Oyo	Iwajowa	191182	2015	76.6	66.6	87.8
Nigeria	Oyo	Kajola	191183	2000	96.6	85.2	109.5
Nigeria	Oyo	Kajola	191183	2005	86.6	75.8	97.9
Nigeria	Oyo	Kajola	191183	2010	78.6	68.7	89.6
Nigeria	Oyo	Kajola	191183	2015	68.0	57.9	78.9
Nigeria	Oyo	Lagelu	191219	2000	95.1	87.2	104.0
Nigeria	Oyo	Lagelu	191219	2005	89.7	82.2	97.5
Nigeria	Oyo	Lagelu	191219	2010	77.1	69.8	84.8
Nigeria	Oyo	Lagelu	191219	2015	64.2	56.3	73.1
Nigeria	Oyo	Ogbomosho North	191181	2000	80.7	71.8	90.6
Nigeria	Oyo	Ogbomosho North	191181	2005	76.9	68.6	86.3
Nigeria	Oyo	Ogbomosho North	191181	2010	71.2	62.7	80.8
Nigeria	Oyo	Ogbomosho North	191181	2015	57.6	49.7	67.7
Nigeria	Oyo	Ogbomosho South	191186	2000	84.1	75.0	94.2
Nigeria	Oyo	Ogbomosho South	191186	2005	78.8	70.6	88.3
Nigeria	Oyo	Ogbomosho South	191186	2010	72.1	63.7	81.4
Nigeria	Oyo	Ogbomosho South	191186	2015	58.7	51.0	68.3

Admin 0	Admin 1	Admin 2	GAUL Code	Year	Under-5 mortality (per 1,000 livebirths)		
					Estimate	Lower bound, 95% UI	Upper bound, 95% UI
Nigeria	Oyo	Ogo Oluwa	23030	2000	89.7	80.9	99.5
Nigeria	Oyo	Ogo Oluwa	23030	2005	84.2	76.0	93.1
Nigeria	Oyo	Ogo Oluwa	23030	2010	74.6	66.5	83.6
Nigeria	Oyo	Ogo Oluwa	23030	2015	62.2	54.5	71.3
Nigeria	Oyo	Olorunsogo	191154	2000	101.1	89.3	115.4
Nigeria	Oyo	Olorunsogo	191154	2005	91.0	81.5	103.6
Nigeria	Oyo	Olorunsogo	191154	2010	83.8	74.4	95.3
Nigeria	Oyo	Olorunsogo	191154	2015	74.8	64.7	86.5
Nigeria	Oyo	Oluyole	191247	2000	104.4	94.8	115.1
Nigeria	Oyo	Oluyole	191247	2005	97.3	88.4	106.2
Nigeria	Oyo	Oluyole	191247	2010	83.0	74.8	91.4
Nigeria	Oyo	Oluyole	191247	2015	69.2	61.1	78.2
Nigeria	Oyo	Ona Ara	191243	2000	99.9	91.2	109.0
Nigeria	Oyo	Ona Ara	191243	2005	93.7	85.3	101.9
Nigeria	Oyo	Ona Ara	191243	2010	80.2	72.2	88.2
Nigeria	Oyo	Ona Ara	191243	2015	66.1	58.3	74.5
Nigeria	Oyo	Orelope	23033	2000	98.7	86.5	113.3
Nigeria	Oyo	Orelope	23033	2005	87.9	77.8	100.0
Nigeria	Oyo	Orelope	23033	2010	81.8	71.8	94.3
Nigeria	Oyo	Orelope	23033	2015	74.5	64.1	86.8
Nigeria	Oyo	Ori-Ire	23034	2000	94.8	85.0	105.6
Nigeria	Oyo	Ori-Ire	23034	2005	86.9	78.3	96.9
Nigeria	Oyo	Ori-Ire	23034	2010	78.5	69.7	88.0
Nigeria	Oyo	Ori-Ire	23034	2015	67.3	58.8	77.4
Nigeria	Oyo	Oyo East	191195	2000	85.3	75.8	96.6
Nigeria	Oyo	Oyo East	191195	2005	80.6	71.1	91.4
Nigeria	Oyo	Oyo East	191195	2010	72.9	63.7	83.0
Nigeria	Oyo	Oyo East	191195	2015	61.0	52.3	71.4
Nigeria	Oyo	Oyo West	191185	2000	82.2	73.0	93.3
Nigeria	Oyo	Oyo West	191185	2005	78.2	68.6	88.9
Nigeria	Oyo	Oyo West	191185	2010	71.2	62.1	81.0
Nigeria	Oyo	Oyo West	191185	2015	58.8	50.1	69.1
Nigeria	Oyo	Saki East	191155	2000	92.7	81.9	104.6
Nigeria	Oyo	Saki East	191155	2005	83.2	74.2	93.9
Nigeria	Oyo	Saki East	191155	2010	77.2	67.9	87.7
Nigeria	Oyo	Saki East	191155	2015	68.3	58.8	79.2
Nigeria	Oyo	Saki West	191159	2000	96.6	85.5	109.5
Nigeria	Oyo	Saki West	191159	2005	86.6	76.8	98.0
Nigeria	Oyo	Saki West	191159	2010	79.7	70.4	91.1
Nigeria	Oyo	Saki West	191159	2015	71.4	61.5	82.8
Nigeria	Oyo	Surulere	23036	2000	89.3	80.5	98.4
Nigeria	Oyo	Surulere	23036	2005	83.3	75.3	91.8
Nigeria	Oyo	Surulere	23036	2010	74.6	66.9	83.6
Nigeria	Oyo	Surulere	23036	2015	61.4	53.8	70.4
Nigeria	Plateau	Barkin Ladi	191117	2000	154.4	141.1	168.3
Nigeria	Plateau	Barkin Ladi	191117	2005	142.8	130.7	157.0
Nigeria	Plateau	Barkin Ladi	191117	2010	127.1	115.0	140.7
Nigeria	Plateau	Barkin Ladi	191117	2015	106.9	94.8	121.7
Nigeria	Plateau	Bassa	23040	2000	180.3	163.3	198.0
Nigeria	Plateau	Bassa	23040	2005	163.0	149.1	178.3
Nigeria	Plateau	Bassa	23040	2010	142.2	128.7	157.5
Nigeria	Plateau	Bassa	23040	2015	125.0	110.2	141.9
Nigeria	Plateau	Bokkos	191134	2000	153.2	138.7	168.9
Nigeria	Plateau	Bokkos	191134	2005	144.6	131.6	159.3
Nigeria	Plateau	Bokkos	191134	2010	129.4	116.5	143.1
Nigeria	Plateau	Bokkos	191134	2015	113.3	99.9	129.7
Nigeria	Plateau	Jos East	191113	2000	173.7	157.4	190.8
Nigeria	Plateau	Jos East	191113	2005	157.7	144.0	173.1
Nigeria	Plateau	Jos East	191113	2010	138.9	125.7	153.6
Nigeria	Plateau	Jos East	191113	2015	117.8	103.8	133.8
Nigeria	Plateau	Jos North	191112	2000	151.4	136.8	166.4
Nigeria	Plateau	Jos North	191112	2005	140.3	127.9	155.3
Nigeria	Plateau	Jos North	191112	2010	126.0	113.1	141.1
Nigeria	Plateau	Jos North	191112	2015	105.2	92.5	120.7
Nigeria	Plateau	Jos South	191116	2000	148.5	135.1	162.8
Nigeria	Plateau	Jos South	191116	2005	138.8	126.7	152.3
Nigeria	Plateau	Jos South	191116	2010	123.3	111.3	136.9
Nigeria	Plateau	Jos South	191116	2015	103.6	91.9	118.7
Nigeria	Plateau	Kanam	23043	2000	213.5	193.7	234.1
Nigeria	Plateau	Kanam	23043	2005	194.4	178.2	213.2
Nigeria	Plateau	Kanam	23043	2010	167.9	153.1	187.0
Nigeria	Plateau	Kanam	23043	2015	147.3	129.5	166.9
Nigeria	Plateau	Kanke	191131	2000	183.6	167.0	200.8
Nigeria	Plateau	Kanke	191131	2005	170.2	154.9	185.6
Nigeria	Plateau	Kanke	191131	2010	153.1	138.1	169.3
Nigeria	Plateau	Kanke	191131	2015	133.4	116.5	152.1
Nigeria	Plateau	Langtang North	23046	2000	172.6	155.4	190.8
Nigeria	Plateau	Langtang North	23046	2005	160.8	144.9	177.2
Nigeria	Plateau	Langtang North	23046	2010	145.2	129.8	162.2
Nigeria	Plateau	Langtang North	23046	2015	125.7	110.0	144.1
Nigeria	Plateau	Langtang South	23047	2000	181.4	163.9	199.7
Nigeria	Plateau	Langtang South	23047	2005	165.1	149.5	182.2
Nigeria	Plateau	Langtang South	23047	2010	143.4	128.2	160.6
Nigeria	Plateau	Langtang South	23047	2015	133.4	116.9	151.3
Nigeria	Plateau	Mangu	191119	2000	156.3	142.4	172.0

Admin 0	Admin 1	Admin 2	GAUL Code	Year	Under-5 mortality (per 1,000 livebirths) Estimate	Lower bound, 95% UI	Upper bound, 95% UI
Nigeria	Plateau	Mangu	191119	2005	146.9	134.2	161.8
Nigeria	Plateau	Mangu	191119	2010	132.0	119.0	146.2
Nigeria	Plateau	Mangu	191119	2015	112.6	98.8	129.1
Nigeria	Plateau	Mikang	191148	2000	168.2	152.5	185.3
Nigeria	Plateau	Mikang	191148	2005	154.3	140.3	168.3
Nigeria	Plateau	Mikang	191148	2010	142.0	128.6	157.8
Nigeria	Plateau	Mikang	191148	2015	126.5	111.3	143.9
Nigeria	Plateau	Pankshin	191128	2000	160.0	144.8	174.9
Nigeria	Plateau	Pankshin	191128	2005	149.1	135.6	163.0
Nigeria	Plateau	Pankshin	191128	2010	136.8	122.8	151.7
Nigeria	Plateau	Pankshin	191128	2015	117.8	103.0	134.1
Nigeria	Plateau	Qua'an Pan	23051	2000	161.3	145.7	177.5
Nigeria	Plateau	Qua'an Pan	23051	2005	145.3	131.5	159.2
Nigeria	Plateau	Qua'an Pan	23051	2010	132.3	118.2	146.7
Nigeria	Plateau	Qua'an Pan	23051	2015	121.7	106.5	138.5
Nigeria	Plateau	Riyom	191122	2000	156.9	142.2	172.3
Nigeria	Plateau	Riyom	191122	2005	145.0	131.4	159.9
Nigeria	Plateau	Riyom	191122	2010	127.4	114.9	141.8
Nigeria	Plateau	Riyom	191122	2015	110.6	97.9	125.7
Nigeria	Plateau	Shendam	191151	2000	170.6	153.9	186.6
Nigeria	Plateau	Shendam	191151	2005	153.3	139.8	167.1
Nigeria	Plateau	Shendam	191151	2010	138.3	124.8	153.0
Nigeria	Plateau	Shendam	191151	2015	127.3	111.2	143.8
Nigeria	Plateau	Wase	23053	2000	195.4	178.2	215.7
Nigeria	Plateau	Wase	23053	2005	177.3	161.8	193.9
Nigeria	Plateau	Wase	23053	2010	153.3	138.2	168.7
Nigeria	Plateau	Wase	23053	2015	138.1	121.6	155.2
Nigeria	Rivers	Abua /Odual	23054	2000	159.2	145.0	174.0
Nigeria	Rivers	Abua /Odual	23054	2005	143.4	130.8	157.1
Nigeria	Rivers	Abua /Odual	23054	2010	119.4	107.6	132.4
Nigeria	Rivers	Abua /Odual	23054	2015	88.2	77.4	100.0
Nigeria	Rivers	Ahoada East	191371	2000	151.3	139.6	164.7
Nigeria	Rivers	Ahoada East	191371	2005	136.3	125.3	148.2
Nigeria	Rivers	Ahoada East	191371	2010	115.3	104.5	126.7
Nigeria	Rivers	Ahoada East	191371	2015	83.1	73.5	94.1
Nigeria	Rivers	Ahoada West	191369	2000	152.4	140.7	165.4
Nigeria	Rivers	Ahoada West	191369	2005	137.3	126.7	149.5
Nigeria	Rivers	Ahoada West	191369	2010	117.9	107.1	129.7
Nigeria	Rivers	Ahoada West	191369	2015	85.1	74.7	95.9
Nigeria	Rivers	Akuku-Toru	191394	2000	153.2	138.3	170.2
Nigeria	Rivers	Akuku-Toru	191394	2005	140.4	125.9	156.1
Nigeria	Rivers	Akuku-Toru	191394	2010	117.5	104.2	130.9
Nigeria	Rivers	Akuku-Toru	191394	2015	89.0	77.1	100.8
Nigeria	Rivers	Andoni	191400	2000	135.8	119.5	153.4
Nigeria	Rivers	Andoni	191400	2005	129.5	113.9	146.7
Nigeria	Rivers	Andoni	191400	2010	102.2	89.1	117.0
Nigeria	Rivers	Andoni	191400	2015	81.5	70.3	94.2
Nigeria	Rivers	Asari-Toru	23058	2000	147.4	133.4	162.6
Nigeria	Rivers	Asari-Toru	23058	2005	133.8	121.2	147.2
Nigeria	Rivers	Asari-Toru	23058	2010	109.4	98.2	121.8
Nigeria	Rivers	Asari-Toru	23058	2015	80.7	70.3	91.4
Nigeria	Rivers	Bonny	191399	2000	137.0	122.2	152.8
Nigeria	Rivers	Bonny	191399	2005	128.3	114.4	143.6
Nigeria	Rivers	Bonny	191399	2010	102.8	90.5	116.8
Nigeria	Rivers	Bonny	191399	2015	80.6	70.1	92.8
Nigeria	Rivers	Degema	191386	2000	146.8	132.8	161.7
Nigeria	Rivers	Degema	191386	2005	134.8	121.9	148.6
Nigeria	Rivers	Degema	191386	2010	111.1	99.6	123.6
Nigeria	Rivers	Degema	191386	2015	83.6	73.1	94.5
Nigeria	Rivers	Eleme	191387	2000	136.5	124.8	149.6
Nigeria	Rivers	Eleme	191387	2005	127.8	116.0	140.4
Nigeria	Rivers	Eleme	191387	2010	102.9	92.2	114.1
Nigeria	Rivers	Eleme	191387	2015	78.4	69.1	89.3
Nigeria	Rivers	Emuoha	191365	2000	151.9	139.6	166.3
Nigeria	Rivers	Emuoha	191365	2005	138.6	126.8	150.9
Nigeria	Rivers	Emuoha	191365	2010	114.5	103.4	127.2
Nigeria	Rivers	Emuoha	191365	2015	84.1	73.8	95.0
Nigeria	Rivers	Etche	191372	2000	146.9	135.3	159.8
Nigeria	Rivers	Etche	191372	2005	140.6	129.1	153.5
Nigeria	Rivers	Etche	191372	2010	112.7	101.7	124.3
Nigeria	Rivers	Etche	191372	2015	82.2	72.2	93.4
Nigeria	Rivers	Gokana	23065	2000	134.8	121.5	148.8
Nigeria	Rivers	Gokana	23065	2005	127.2	114.2	141.4
Nigeria	Rivers	Gokana	23065	2010	101.9	90.7	114.3
Nigeria	Rivers	Gokana	23065	2015	79.4	69.7	91.2
Nigeria	Rivers	Ikwerre	191368	2000	149.3	137.5	162.1
Nigeria	Rivers	Ikwerre	191368	2005	137.9	126.8	149.7
Nigeria	Rivers	Ikwerre	191368	2010	111.7	100.8	122.7
Nigeria	Rivers	Ikwerre	191368	2015	81.2	71.6	91.3
Nigeria	Rivers	Khana	191388	2000	133.7	120.7	148.1
Nigeria	Rivers	Khana	191388	2005	128.6	116.1	142.6
Nigeria	Rivers	Khana	191388	2010	102.4	91.3	115.0
Nigeria	Rivers	Khana	191388	2015	79.9	69.9	91.8
Nigeria	Rivers	Obio/Akpor	23069	2000	136.9	125.5	149.4
Nigeria	Rivers	Obio/Akpor	23069	2005	125.9	115.7	136.8

Admin 0	Admin 1	Admin 2	GAUL Code	Year	Under-5 mortality (per 1,000 livebirths)		
					Estimate	Lower bound, 95% UI	Upper bound, 95% UI
Nigeria	Rivers	Obio/Akpor	23069	2010	101.9	91.8	112.2
Nigeria	Rivers	Obio/Akpor	23069	2015	76.4	67.4	86.4
Nigeria	Rivers	Ogba/ Egbema/ Ndoni	23070	2000	138.2	126.4	150.5
Nigeria	Rivers	Ogba/ Egbema/ Ndoni	23070	2005	125.2	114.9	136.5
Nigeria	Rivers	Ogba/ Egbema/ Ndoni	23070	2010	110.3	100.5	120.9
Nigeria	Rivers	Ogba/ Egbema/ Ndoni	23070	2015	77.5	68.6	87.6
Nigeria	Rivers	Ogu /Bolo	191395	2000	136.6	123.5	150.4
Nigeria	Rivers	Ogu /Bolo	191395	2005	128.2	115.4	142.0
Nigeria	Rivers	Ogu /Bolo	191395	2010	102.6	91.6	114.9
Nigeria	Rivers	Ogu /Bolo	191395	2015	79.1	69.4	90.6
Nigeria	Rivers	Okrika	191392	2000	135.8	124.0	149.1
Nigeria	Rivers	Okrika	191392	2005	126.5	114.3	139.5
Nigeria	Rivers	Okrika	191392	2010	102.5	91.7	113.9
Nigeria	Rivers	Okrika	191392	2015	77.8	68.5	88.4
Nigeria	Rivers	Omumma	191373	2000	145.1	134.1	157.2
Nigeria	Rivers	Omumma	191373	2005	141.0	129.4	153.4
Nigeria	Rivers	Omumma	191373	2010	113.0	102.3	123.8
Nigeria	Rivers	Omumma	191373	2015	82.2	72.5	93.6
Nigeria	Rivers	Opobo /Nkoro	191401	2000	132.9	117.8	149.6
Nigeria	Rivers	Opobo /Nkoro	191401	2005	127.9	113.3	143.8
Nigeria	Rivers	Opobo /Nkoro	191401	2010	101.8	89.2	116.3
Nigeria	Rivers	Opobo /Nkoro	191401	2015	80.1	69.5	92.6
Nigeria	Rivers	Oyigbo	191384	2000	137.9	126.7	150.2
Nigeria	Rivers	Oyigbo	191384	2005	131.3	120.6	143.3
Nigeria	Rivers	Oyigbo	191384	2010	104.8	95.0	114.8
Nigeria	Rivers	Oyigbo	191384	2015	78.8	69.7	89.4
Nigeria	Rivers	Port Harcourt	191389	2000	135.0	123.3	148.6
Nigeria	Rivers	Port Harcourt	191389	2005	123.6	112.4	135.3
Nigeria	Rivers	Port Harcourt	191389	2010	100.1	89.7	110.8
Nigeria	Rivers	Port Harcourt	191389	2015	75.0	66.0	85.0
Nigeria	Rivers	Tai	191390	2000	136.9	124.4	149.9
Nigeria	Rivers	Tai	191390	2005	129.9	117.6	143.5
Nigeria	Rivers	Tai	191390	2010	103.8	93.3	114.9
Nigeria	Rivers	Tai	191390	2015	79.8	70.3	91.1
Nigeria	Sokoto	Binji	23078	2000	271.5	249.4	297.3
Nigeria	Sokoto	Binji	23078	2005	265.4	243.1	288.2
Nigeria	Sokoto	Binji	23078	2010	204.8	186.3	224.2
Nigeria	Sokoto	Binji	23078	2015	172.5	151.0	194.5
Nigeria	Sokoto	Bodinga	23079	2000	271.7	250.9	294.7
Nigeria	Sokoto	Bodinga	23079	2005	267.1	247.5	288.8
Nigeria	Sokoto	Bodinga	23079	2010	205.4	186.8	224.8
Nigeria	Sokoto	Bodinga	23079	2015	176.1	156.2	198.8
Nigeria	Sokoto	Dange-Shuni	190971	2000	291.0	268.9	315.2
Nigeria	Sokoto	Dange-Shuni	190971	2005	286.7	265.7	309.0
Nigeria	Sokoto	Dange-Shuni	190971	2010	217.9	199.7	238.0
Nigeria	Sokoto	Dange-Shuni	190971	2015	182.4	162.5	206.2
Nigeria	Sokoto	Gada	23082	2000	267.9	248.4	288.0
Nigeria	Sokoto	Gada	23082	2005	250.9	234.4	268.8
Nigeria	Sokoto	Gada	23082	2010	200.7	184.4	218.4
Nigeria	Sokoto	Gada	23082	2015	173.8	153.5	195.7
Nigeria	Sokoto	Goronyo	23083	2000	285.5	261.8	309.0
Nigeria	Sokoto	Goronyo	23083	2005	266.4	246.6	287.9
Nigeria	Sokoto	Goronyo	23083	2010	209.6	190.8	229.4
Nigeria	Sokoto	Goronyo	23083	2015	176.1	156.8	198.0
Nigeria	Sokoto	Gudu	191409	2000	248.7	228.5	272.7
Nigeria	Sokoto	Gudu	191409	2005	238.2	218.5	260.0
Nigeria	Sokoto	Gudu	191409	2010	192.3	174.7	213.6
Nigeria	Sokoto	Gudu	191409	2015	169.7	148.8	192.2
Nigeria	Sokoto	Gwadabawa	23086	2000	277.0	257.3	298.1
Nigeria	Sokoto	Gwadabawa	23086	2005	267.8	250.1	287.7
Nigeria	Sokoto	Gwadabawa	23086	2010	207.5	190.4	226.3
Nigeria	Sokoto	Gwadabawa	23086	2015	175.3	155.5	197.3
Nigeria	Sokoto	Illela	23087	2000	274.8	253.6	295.8
Nigeria	Sokoto	Illela	23087	2005	249.3	230.3	268.6
Nigeria	Sokoto	Illela	23087	2010	199.2	180.3	217.5
Nigeria	Sokoto	Illela	23087	2015	172.5	151.6	195.9
Nigeria	Sokoto	Isa	191408	2000	278.9	256.2	303.2
Nigeria	Sokoto	Isa	191408	2005	251.2	230.6	273.3
Nigeria	Sokoto	Isa	191408	2010	200.6	181.3	219.1
Nigeria	Sokoto	Isa	191408	2015	169.8	151.0	191.0
Nigeria	Sokoto	Kebbe	191023	2000	224.9	204.0	246.1
Nigeria	Sokoto	Kebbe	191023	2005	220.1	199.5	239.2
Nigeria	Sokoto	Kebbe	191023	2010	193.5	173.9	213.6
Nigeria	Sokoto	Kebbe	191023	2015	160.5	142.1	181.0
Nigeria	Sokoto	Kware	23090	2000	276.2	257.0	295.5
Nigeria	Sokoto	Kware	23090	2005	271.2	253.7	289.5
Nigeria	Sokoto	Kware	23090	2010	207.0	190.5	224.4
Nigeria	Sokoto	Kware	23090	2015	174.8	155.6	195.4
Nigeria	Sokoto	Rabah	23092	2000	287.4	261.3	316.6
Nigeria	Sokoto	Rabah	23092	2005	278.7	253.9	305.3
Nigeria	Sokoto	Rabah	23092	2010	218.8	198.1	243.3
Nigeria	Sokoto	Rabah	23092	2015	181.9	161.0	205.2
Nigeria	Sokoto	Sabon Birni	23093	2000	272.4	250.6	294.8
Nigeria	Sokoto	Sabon Birni	23093	2005	245.9	226.8	267.4
Nigeria	Sokoto	Sabon Birni	23093	2010	191.3	172.8	211.1

Admin 0	Admin 1	Admin 2	GAUL Code	Year	Under-5 mortality (per 1,000 livebirths)		
					Estimate	Lower bound, 95% UI	Upper bound, 95% UI
Nigeria	Sokoto	Sabon Birni	23093	2015	163.0	144.3	185.9
Nigeria	Sokoto	Shagari	190984	2000	261.2	241.9	282.8
Nigeria	Sokoto	Shagari	190984	2005	260.8	242.1	280.7
Nigeria	Sokoto	Shagari	190984	2010	205.3	188.8	221.8
Nigeria	Sokoto	Shagari	190984	2015	173.7	154.1	194.8
Nigeria	Sokoto	Silame	23094	2000	257.5	238.0	279.3
Nigeria	Sokoto	Silame	23094	2005	251.4	231.8	270.5
Nigeria	Sokoto	Silame	23094	2010	196.6	179.3	213.1
Nigeria	Sokoto	Silame	23094	2015	166.7	146.7	186.4
Nigeria	Sokoto	Sokoto North	190962	2000	244.3	227.5	261.4
Nigeria	Sokoto	Sokoto North	190962	2005	235.9	221.0	252.1
Nigeria	Sokoto	Sokoto North	190962	2010	183.8	169.0	199.3
Nigeria	Sokoto	Sokoto North	190962	2015	166.4	147.5	187.2
Nigeria	Sokoto	Sokoto South	190966	2000	263.4	244.7	282.2
Nigeria	Sokoto	Sokoto South	190966	2005	257.8	241.0	276.4
Nigeria	Sokoto	Sokoto South	190966	2010	195.2	179.3	211.7
Nigeria	Sokoto	Sokoto South	190966	2015	173.0	154.1	194.6
Nigeria	Sokoto	Tambuwal	23097	2000	237.3	219.7	256.4
Nigeria	Sokoto	Tambuwal	23097	2005	235.9	219.4	252.8
Nigeria	Sokoto	Tambuwal	23097	2010	199.7	183.7	216.0
Nigeria	Sokoto	Tambuwal	23097	2015	162.8	145.2	181.2
Nigeria	Sokoto	Tangaza	190949	2000	278.5	256.8	302.1
Nigeria	Sokoto	Tangaza	190949	2005	275.1	255.0	296.2
Nigeria	Sokoto	Tangaza	190949	2010	209.9	192.2	229.2
Nigeria	Sokoto	Tangaza	190949	2015	177.5	155.8	200.0
Nigeria	Sokoto	Tureta	190989	2000	278.1	252.1	306.1
Nigeria	Sokoto	Tureta	190989	2005	278.2	253.1	306.5
Nigeria	Sokoto	Tureta	190989	2010	217.8	195.9	242.0
Nigeria	Sokoto	Tureta	190989	2015	184.4	160.8	209.3
Nigeria	Sokoto	Wamako	23100	2000	240.6	224.6	256.3
Nigeria	Sokoto	Wamako	23100	2005	229.8	216.0	244.8
Nigeria	Sokoto	Wamako	23100	2010	184.4	170.0	200.0
Nigeria	Sokoto	Wamako	23100	2015	168.7	150.2	189.1
Nigeria	Sokoto	Wurno	23101	2000	276.7	254.8	300.4
Nigeria	Sokoto	Wurno	23101	2005	268.3	248.7	290.2
Nigeria	Sokoto	Wurno	23101	2010	206.8	188.9	227.2
Nigeria	Sokoto	Wurno	23101	2015	174.4	154.9	196.3
Nigeria	Sokoto	Yabo	190974	2000	259.1	240.2	279.5
Nigeria	Sokoto	Yabo	190974	2005	257.5	238.5	275.7
Nigeria	Sokoto	Yabo	190974	2010	201.1	183.9	217.3
Nigeria	Sokoto	Yabo	190974	2015	170.1	151.1	191.1
Nigeria	Taraba	Ardo-Kola	23108	2000	215.5	195.9	237.9
Nigeria	Taraba	Ardo-Kola	23108	2005	184.0	167.9	201.7
Nigeria	Taraba	Ardo-Kola	23108	2010	147.0	132.5	163.5
Nigeria	Taraba	Ardo-Kola	23108	2015	135.8	118.9	154.0
Nigeria	Taraba	Bali	191164	2000	237.1	217.1	258.7
Nigeria	Taraba	Bali	191164	2005	204.2	187.3	223.4
Nigeria	Taraba	Bali	191164	2010	165.1	148.7	182.0
Nigeria	Taraba	Bali	191164	2015	159.3	140.3	180.2
Nigeria	Taraba	Donga	23105	2000	190.2	170.9	210.4
Nigeria	Taraba	Donga	23105	2005	169.1	152.8	187.7
Nigeria	Taraba	Donga	23105	2010	140.2	125.4	156.9
Nigeria	Taraba	Donga	23105	2015	130.4	114.2	148.9
Nigeria	Taraba	Gashaka	23106	2000	234.1	208.8	260.3
Nigeria	Taraba	Gashaka	23106	2005	201.7	179.4	225.0
Nigeria	Taraba	Gashaka	23106	2010	166.5	145.7	187.7
Nigeria	Taraba	Gashaka	23106	2015	156.8	135.0	180.3
Nigeria	Taraba	Gassol	191160	2000	228.4	208.6	249.9
Nigeria	Taraba	Gassol	191160	2005	199.0	182.2	217.1
Nigeria	Taraba	Gassol	191160	2010	161.2	146.4	176.8
Nigeria	Taraba	Gassol	191160	2015	156.2	137.9	175.8
Nigeria	Taraba	Ibi	23107	2000	197.9	181.0	215.5
Nigeria	Taraba	Ibi	23107	2005	174.1	160.1	190.0
Nigeria	Taraba	Ibi	23107	2010	145.6	131.9	160.0
Nigeria	Taraba	Ibi	23107	2015	136.3	120.2	153.4
Nigeria	Taraba	Jalingo	191153	2000	218.6	198.3	242.8
Nigeria	Taraba	Jalingo	191153	2005	184.0	167.8	201.9
Nigeria	Taraba	Jalingo	191153	2010	147.3	132.3	164.0
Nigeria	Taraba	Jalingo	191153	2015	131.8	115.6	149.2
Nigeria	Taraba	Karim Lamido	23109	2000	222.9	205.7	241.2
Nigeria	Taraba	Karim Lamido	23109	2005	193.2	179.5	207.9
Nigeria	Taraba	Karim Lamido	23109	2010	156.0	142.6	169.8
Nigeria	Taraba	Karim Lamido	23109	2015	139.2	123.9	156.1
Nigeria	Taraba	Kurmi	191212	2000	183.7	163.4	206.9
Nigeria	Taraba	Kurmi	191212	2005	166.0	147.3	186.0
Nigeria	Taraba	Kurmi	191212	2010	137.9	120.1	156.7
Nigeria	Taraba	Kurmi	191212	2015	126.2	108.6	145.7
Nigeria	Taraba	Lau	191135	2000	232.0	211.8	251.6
Nigeria	Taraba	Lau	191135	2005	200.0	184.5	217.4
Nigeria	Taraba	Lau	191135	2010	160.4	145.5	176.1
Nigeria	Taraba	Lau	191135	2015	141.0	126.0	158.9
Nigeria	Taraba	Sardauna	191241	2000	182.4	163.9	202.4
Nigeria	Taraba	Sardauna	191241	2005	163.7	147.7	182.0
Nigeria	Taraba	Sardauna	191241	2010	134.9	120.1	151.5
Nigeria	Taraba	Sardauna	191241	2015	121.1	105.6	138.9

Admin 0	Admin 1	Admin 2	GAUL Code	Year	Under-5 mortality (per 1,000 livebirths)		
					Estimate	Lower bound, 95% UI	Upper bound, 95% UI
Nigeria	Taraba	Takum	191217	2000	166.6	149.5	184.0
Nigeria	Taraba	Takum	191217	2005	149.3	133.9	165.5
Nigeria	Taraba	Takum	191217	2010	126.8	112.9	143.9
Nigeria	Taraba	Takum	191217	2015	112.5	97.6	129.2
Nigeria	Taraba	Ussa	191250	2000	154.2	136.5	171.5
Nigeria	Taraba	Ussa	191250	2005	142.1	127.5	158.1
Nigeria	Taraba	Ussa	191250	2010	118.5	105.5	134.0
Nigeria	Taraba	Ussa	191250	2015	104.6	90.9	120.9
Nigeria	Taraba	Wukari	23113	2000	178.5	162.8	196.0
Nigeria	Taraba	Wukari	23113	2005	157.1	142.5	172.2
Nigeria	Taraba	Wukari	23113	2010	131.8	118.9	146.0
Nigeria	Taraba	Wukari	23113	2015	121.8	107.1	137.2
Nigeria	Taraba	Yorro	191149	2000	226.6	208.0	246.4
Nigeria	Taraba	Yorro	191149	2005	193.3	177.8	210.0
Nigeria	Taraba	Yorro	191149	2010	152.6	138.8	168.3
Nigeria	Taraba	Yorro	191149	2015	137.4	121.5	154.4
Nigeria	Taraba	Zing	23115	2000	230.4	210.8	251.5
Nigeria	Taraba	Zing	23115	2005	195.7	178.6	213.1
Nigeria	Taraba	Zing	23115	2010	155.5	140.0	171.9
Nigeria	Taraba	Zing	23115	2015	137.8	120.9	155.3
Nigeria	Yobe	Bade	191423	2000	198.4	181.5	216.8
Nigeria	Yobe	Bade	191423	2005	171.5	157.1	187.7
Nigeria	Yobe	Bade	191423	2010	154.6	140.2	170.7
Nigeria	Yobe	Bade	191423	2015	131.0	115.0	148.8
Nigeria	Yobe	Borsari	190973	2000	196.6	177.0	217.7
Nigeria	Yobe	Borsari	190973	2005	169.9	153.8	188.3
Nigeria	Yobe	Borsari	190973	2010	150.6	134.9	168.1
Nigeria	Yobe	Borsari	190973	2015	128.4	112.6	146.4
Nigeria	Yobe	Damaturu	191032	2000	236.9	213.4	265.0
Nigeria	Yobe	Damaturu	191032	2005	200.6	179.4	223.9
Nigeria	Yobe	Damaturu	191032	2010	169.7	150.1	188.2
Nigeria	Yobe	Damaturu	191032	2015	146.8	126.0	169.0
Nigeria	Yobe	Fika	23118	2000	238.4	221.1	256.1
Nigeria	Yobe	Fika	23118	2005	199.9	183.8	217.0
Nigeria	Yobe	Fika	23118	2010	175.9	160.3	193.5
Nigeria	Yobe	Fika	23118	2015	152.2	134.1	173.1
Nigeria	Yobe	Fune	23119	2000	235.1	215.8	255.8
Nigeria	Yobe	Fune	23119	2005	197.8	180.3	216.3
Nigeria	Yobe	Fune	23119	2010	174.7	158.7	192.9
Nigeria	Yobe	Fune	23119	2015	148.6	130.9	168.1
Nigeria	Yobe	Geidam	23120	2000	200.3	177.4	229.0
Nigeria	Yobe	Geidam	23120	2005	173.3	152.3	198.5
Nigeria	Yobe	Geidam	23120	2010	152.3	132.5	175.7
Nigeria	Yobe	Geidam	23120	2015	129.0	111.0	152.3
Nigeria	Yobe	Gujba	191063	2000	263.6	242.2	288.2
Nigeria	Yobe	Gujba	191063	2005	219.6	201.4	240.9
Nigeria	Yobe	Gujba	191063	2010	188.3	170.6	208.6
Nigeria	Yobe	Gujba	191063	2015	160.8	140.1	183.4
Nigeria	Yobe	Gulani	191085	2000	261.3	239.8	284.8
Nigeria	Yobe	Gulani	191085	2005	220.0	201.3	240.2
Nigeria	Yobe	Gulani	191085	2010	189.8	171.8	209.8
Nigeria	Yobe	Gulani	191085	2015	164.9	144.1	187.8
Nigeria	Yobe	Jakusko	190982	2000	209.4	193.6	226.9
Nigeria	Yobe	Jakusko	190982	2005	179.5	165.3	194.1
Nigeria	Yobe	Jakusko	190982	2010	159.1	145.0	174.4
Nigeria	Yobe	Jakusko	190982	2015	137.0	121.1	153.7
Nigeria	Yobe	Karasuwa	190961	2000	195.0	175.9	214.5
Nigeria	Yobe	Karasuwa	190961	2005	170.5	155.3	188.4
Nigeria	Yobe	Karasuwa	190961	2010	155.0	139.6	172.4
Nigeria	Yobe	Karasuwa	190961	2015	131.6	114.7	151.0
Nigeria	Yobe	Machina	190956	2000	244.7	224.6	268.0
Nigeria	Yobe	Machina	190956	2005	214.8	196.0	237.5
Nigeria	Yobe	Machina	190956	2010	177.5	159.5	197.7
Nigeria	Yobe	Machina	190956	2015	155.3	135.6	177.4
Nigeria	Yobe	Nangere	191028	2000	213.5	193.9	235.0
Nigeria	Yobe	Nangere	191028	2005	180.4	162.6	198.6
Nigeria	Yobe	Nangere	191028	2010	159.1	142.3	177.8
Nigeria	Yobe	Nangere	191028	2015	142.9	125.1	164.6
Nigeria	Yobe	Nguru	190960	2000	195.2	177.2	213.3
Nigeria	Yobe	Nguru	190960	2005	168.3	153.6	183.5
Nigeria	Yobe	Nguru	190960	2010	149.2	134.7	165.1
Nigeria	Yobe	Nguru	190960	2015	130.4	114.1	149.2
Nigeria	Yobe	Potiskum	191050	2000	213.7	193.5	235.8
Nigeria	Yobe	Potiskum	191050	2005	181.5	162.9	200.8
Nigeria	Yobe	Potiskum	191050	2010	159.9	142.0	179.1
Nigeria	Yobe	Potiskum	191050	2015	141.1	123.2	163.2
Nigeria	Yobe	Tarmuwa	191003	2000	235.2	209.1	263.6
Nigeria	Yobe	Tarmuwa	191003	2005	200.4	178.2	223.3
Nigeria	Yobe	Tarmuwa	191003	2010	172.9	152.0	193.9
Nigeria	Yobe	Tarmuwa	191003	2015	148.3	127.4	170.5
Nigeria	Yobe	Yunusari	23124	2000	183.2	163.5	205.7
Nigeria	Yobe	Yunusari	23124	2005	156.4	138.2	175.8
Nigeria	Yobe	Yunusari	23124	2010	140.0	122.8	159.9
Nigeria	Yobe	Yunusari	23124	2015	118.4	101.5	136.9
Nigeria	Yobe	Yusufari	190952	2000	199.5	181.1	218.4

Admin 0	Admin 1	Admin 2	GAUL Code	Year	Under-5 mortality (per 1,000 livebirths) Estimate	Lower bound, 95% UI	Upper bound, 95% UI
Nigeria	Yobe	Yusufari	190952	2005	172.5	157.6	189.6
Nigeria	Yobe	Yusufari	190952	2010	156.6	141.0	175.1
Nigeria	Yobe	Yusufari	190952	2015	132.8	115.9	151.5
Nigeria	Zamfara	Anka	191013	2000	261.3	239.2	284.9
Nigeria	Zamfara	Anka	191013	2005	240.0	219.9	262.2
Nigeria	Zamfara	Anka	191013	2010	213.2	192.8	234.4
Nigeria	Zamfara	Anka	191013	2015	181.9	160.2	204.4
Nigeria	Zamfara	Bakura	23077	2000	264.4	241.1	289.4
Nigeria	Zamfara	Bakura	23077	2005	246.4	225.8	269.4
Nigeria	Zamfara	Bakura	23077	2010	212.2	192.3	233.5
Nigeria	Zamfara	Bakura	23077	2015	173.8	153.5	196.2
Nigeria	Zamfara	Birnin-Magaji/Kiyaw	190994	2000	322.4	300.7	343.4
Nigeria	Zamfara	Birnin-Magaji/Kiyaw	190994	2005	294.6	275.8	314.5
Nigeria	Zamfara	Birnin-Magaji/Kiyaw	190994	2010	249.7	230.9	269.2
Nigeria	Zamfara	Birnin-Magaji/Kiyaw	190994	2015	195.0	174.6	217.4
Nigeria	Zamfara	Bukkuyum	191016	2000	246.5	225.5	269.4
Nigeria	Zamfara	Bukkuyum	191016	2005	230.5	210.4	251.5
Nigeria	Zamfara	Bukkuyum	191016	2010	209.5	189.8	228.8
Nigeria	Zamfara	Bukkuyum	191016	2015	175.0	154.7	195.8
Nigeria	Zamfara	Bungudu	23080	2000	283.8	266.0	302.8
Nigeria	Zamfara	Bungudu	23080	2005	261.0	244.3	279.1
Nigeria	Zamfara	Bungudu	23080	2010	234.8	216.8	253.8
Nigeria	Zamfara	Bungudu	23080	2015	185.3	165.1	206.5
Nigeria	Zamfara	Gummi	191019	2000	229.4	209.1	251.3
Nigeria	Zamfara	Gummi	191019	2005	221.8	201.9	242.1
Nigeria	Zamfara	Gummi	191019	2010	195.2	176.0	215.7
Nigeria	Zamfara	Gummi	191019	2015	165.5	145.6	187.6
Nigeria	Zamfara	Gusau	23085	2000	256.6	240.7	274.3
Nigeria	Zamfara	Gusau	23085	2005	231.4	217.0	247.7
Nigeria	Zamfara	Gusau	23085	2010	201.0	185.4	217.1
Nigeria	Zamfara	Gusau	23085	2015	170.1	151.8	189.1
Nigeria	Zamfara	Kaura Namoda	190990	2000	318.0	296.8	340.3
Nigeria	Zamfara	Kaura Namoda	190990	2005	289.3	269.6	310.7
Nigeria	Zamfara	Kaura Namoda	190990	2010	248.3	228.2	268.4
Nigeria	Zamfara	Kaura Namoda	190990	2015	197.1	175.6	221.3
Nigeria	Zamfara	Maradun	23091	2000	288.7	265.8	311.3
Nigeria	Zamfara	Maradun	23091	2005	270.3	249.0	292.3
Nigeria	Zamfara	Maradun	23091	2010	226.6	205.5	247.4
Nigeria	Zamfara	Maradun	23091	2015	181.9	162.6	205.7
Nigeria	Zamfara	Maru	191424	2000	250.4	232.6	271.2
Nigeria	Zamfara	Maru	191424	2005	223.3	207.5	241.7
Nigeria	Zamfara	Maru	191424	2010	202.6	186.3	219.5
Nigeria	Zamfara	Maru	191424	2015	176.4	157.1	197.4
Nigeria	Zamfara	Shinkafi	190957	2000	288.1	266.3	310.1
Nigeria	Zamfara	Shinkafi	190957	2005	257.4	237.5	277.3
Nigeria	Zamfara	Shinkafi	190957	2010	212.1	193.2	230.7
Nigeria	Zamfara	Shinkafi	190957	2015	173.2	154.6	195.4
Nigeria	Zamfara	Talata-Mafara	23096	2000	267.3	241.9	295.4
Nigeria	Zamfara	Talata-Mafara	23096	2005	248.0	225.7	273.7
Nigeria	Zamfara	Talata-Mafara	23096	2010	223.4	200.8	248.4
Nigeria	Zamfara	Talata-Mafara	23096	2015	179.6	158.0	204.8
Nigeria	Zamfara	Tsafe	23099	2000	274.4	252.2	298.1
Nigeria	Zamfara	Tsafe	23099	2005	251.9	232.6	273.2
Nigeria	Zamfara	Tsafe	23099	2010	229.0	208.8	250.3
Nigeria	Zamfara	Tsafe	23099	2015	178.2	156.9	200.4
Nigeria	Zamfara	Zurmi	23103	2000	323.9	304.4	344.1
Nigeria	Zamfara	Zurmi	23103	2005	294.2	277.2	312.1
Nigeria	Zamfara	Zurmi	23103	2010	241.7	224.4	260.1
Nigeria	Zamfara	Zurmi	23103	2015	191.4	172.0	214.2
Rwanda	East/Iburasirazuba	Bugesera	21974	2000	190.2	181.4	199.5
Rwanda	East/Iburasirazuba	Bugesera	21974	2005	127.3	121.2	133.6
Rwanda	East/Iburasirazuba	Bugesera	21974	2010	80.0	75.6	84.7
Rwanda	East/Iburasirazuba	Bugesera	21974	2015	67.6	62.2	73.7
Rwanda	East/Iburasirazuba	Gatsibo	21978	2000	191.7	183.4	200.7
Rwanda	East/Iburasirazuba	Gatsibo	21978	2005	125.6	119.5	131.2
Rwanda	East/Iburasirazuba	Gatsibo	21978	2010	73.2	68.7	77.8
Rwanda	East/Iburasirazuba	Gatsibo	21978	2015	56.0	51.3	61.2
Rwanda	East/Iburasirazuba	Kayonza	21984	2000	194.9	184.9	204.7
Rwanda	East/Iburasirazuba	Kayonza	21984	2005	128.2	121.4	136.1
Rwanda	East/Iburasirazuba	Kayonza	21984	2010	75.9	71.0	81.2
Rwanda	East/Iburasirazuba	Kayonza	21984	2015	58.7	53.5	64.3
Rwanda	East/Iburasirazuba	Kirehe	21986	2000	203.1	191.6	214.8
Rwanda	East/Iburasirazuba	Kirehe	21986	2005	134.5	125.8	143.0
Rwanda	East/Iburasirazuba	Kirehe	21986	2010	84.9	78.7	90.9
Rwanda	East/Iburasirazuba	Kirehe	21986	2015	66.9	60.1	73.5
Rwanda	East/Iburasirazuba	Ngoma	21989	2000	212.3	202.4	223.2
Rwanda	East/Iburasirazuba	Ngoma	21989	2005	141.1	133.5	148.9
Rwanda	East/Iburasirazuba	Ngoma	21989	2010	84.6	79.4	90.3
Rwanda	East/Iburasirazuba	Ngoma	21989	2015	67.0	61.0	73.4
Rwanda	East/Iburasirazuba	Nyagatare	21992	2000	179.2	170.6	187.3
Rwanda	East/Iburasirazuba	Nyagatare	21992	2005	123.0	116.8	129.4
Rwanda	East/Iburasirazuba	Nyagatare	21992	2010	73.3	68.7	78.5
Rwanda	East/Iburasirazuba	Nyagatare	21992	2015	54.1	48.9	59.2
Rwanda	East/Iburasirazuba	Rwamagana	22003	2000	175.5	167.7	184.7
Rwanda	East/Iburasirazuba	Rwamagana	22003	2005	120.3	114.5	126.7

Admin 0	Admin 1	Admin 2	GAUL Code	Year	Under-5 mortality (per 1,000 livebirths)		
					Estimate	Lower bound, 95% UI	Upper bound, 95% UI
Rwanda	East/Iburasirazuba	Rwamagana	22003	2010	72.1	67.8	76.9
Rwanda	East/Iburasirazuba	Rwamagana	22003	2015	55.9	51.1	61.0
Rwanda	Kigali City/Umujyi wa Kigali	Gasabo	21977	2000	127.3	122.3	132.5
Rwanda	Kigali City/Umujyi wa Kigali	Gasabo	21977	2005	92.2	88.2	96.4
Rwanda	Kigali City/Umujyi wa Kigali	Gasabo	21977	2010	60.7	57.3	64.4
Rwanda	Kigali City/Umujyi wa Kigali	Gasabo	21977	2015	47.6	43.9	51.9
Rwanda	Kigali City/Umujyi wa Kigali	Kicukiro	21985	2000	108.2	103.2	113.0
Rwanda	Kigali City/Umujyi wa Kigali	Kicukiro	21985	2005	86.0	82.1	90.3
Rwanda	Kigali City/Umujyi wa Kigali	Kicukiro	21985	2010	59.5	56.0	63.2
Rwanda	Kigali City/Umujyi wa Kigali	Kicukiro	21985	2015	47.9	44.1	52.4
Rwanda	Kigali City/Umujyi wa Kigali	Nyarugenge	21996	2000	103.0	98.5	107.7
Rwanda	Kigali City/Umujyi wa Kigali	Nyarugenge	21996	2005	82.1	78.1	86.2
Rwanda	Kigali City/Umujyi wa Kigali	Nyarugenge	21996	2010	57.6	54.1	61.2
Rwanda	Kigali City/Umujyi wa Kigali	Nyarugenge	21996	2015	45.7	42.0	50.1
Rwanda	North/Amajyaruguru	Burera	21975	2000	159.0	152.7	165.1
Rwanda	North/Amajyaruguru	Burera	21975	2005	104.3	99.2	109.7
Rwanda	North/Amajyaruguru	Burera	21975	2010	63.7	59.8	67.8
Rwanda	North/Amajyaruguru	Burera	21975	2015	47.0	42.6	51.4
Rwanda	North/Amajyaruguru	Gakenke	21976	2000	154.8	148.7	160.8
Rwanda	North/Amajyaruguru	Gakenke	21976	2005	101.5	96.9	106.2
Rwanda	North/Amajyaruguru	Gakenke	21976	2010	62.9	59.3	66.8
Rwanda	North/Amajyaruguru	Gakenke	21976	2015	47.6	43.7	52.0
Rwanda	North/Amajyaruguru	Gicumbi	21979	2000	167.9	161.3	174.9
Rwanda	North/Amajyaruguru	Gicumbi	21979	2005	108.6	103.6	113.7
Rwanda	North/Amajyaruguru	Gicumbi	21979	2010	65.0	61.1	69.0
Rwanda	North/Amajyaruguru	Gicumbi	21979	2015	49.5	45.1	53.9
Rwanda	North/Amajyaruguru	Musanze	21988	2000	159.7	151.6	167.9
Rwanda	North/Amajyaruguru	Musanze	21988	2005	106.2	100.7	112.4
Rwanda	North/Amajyaruguru	Musanze	21988	2010	64.6	60.4	69.0
Rwanda	North/Amajyaruguru	Musanze	21988	2015	48.0	43.7	52.7
Rwanda	North/Amajyaruguru	Rulindo	22000	2000	153.8	147.7	160.2
Rwanda	North/Amajyaruguru	Rulindo	22000	2005	99.8	95.2	104.4
Rwanda	North/Amajyaruguru	Rulindo	22000	2010	61.7	58.1	65.6
Rwanda	North/Amajyaruguru	Rulindo	22000	2015	47.4	43.4	51.7
Rwanda	South/Amajyepfo	Gisagara	21980	2000	178.6	171.1	186.2
Rwanda	South/Amajyepfo	Gisagara	21980	2005	126.0	120.9	131.8
Rwanda	South/Amajyepfo	Gisagara	21980	2010	85.0	80.3	89.6
Rwanda	South/Amajyepfo	Gisagara	21980	2015	66.9	61.7	72.6
Rwanda	South/Amajyepfo	Huye	21981	2000	144.8	138.8	151.4
Rwanda	South/Amajyepfo	Huye	21981	2005	106.1	101.4	110.8
Rwanda	South/Amajyepfo	Huye	21981	2010	72.3	68.1	76.2
Rwanda	South/Amajyepfo	Huye	21981	2015	56.2	52.0	61.0
Rwanda	South/Amajyepfo	Kamonyi	21982	2000	131.9	126.3	137.2
Rwanda	South/Amajyepfo	Kamonyi	21982	2005	93.4	89.4	97.4
Rwanda	South/Amajyepfo	Kamonyi	21982	2010	61.8	58.4	65.4
Rwanda	South/Amajyepfo	Kamonyi	21982	2015	48.5	44.9	52.7
Rwanda	South/Amajyepfo	Muhanga	21987	2000	132.3	126.8	137.8
Rwanda	South/Amajyepfo	Muhanga	21987	2005	92.7	88.6	97.0
Rwanda	South/Amajyepfo	Muhanga	21987	2010	60.6	57.2	64.1
Rwanda	South/Amajyepfo	Muhanga	21987	2015	47.1	43.3	51.0
Rwanda	South/Amajyepfo	Nyamagabe	21993	2000	139.4	133.5	145.6
Rwanda	South/Amajyepfo	Nyamagabe	21993	2005	97.7	92.9	102.5
Rwanda	South/Amajyepfo	Nyamagabe	21993	2010	66.2	62.2	70.2
Rwanda	South/Amajyepfo	Nyamagabe	21993	2015	50.9	46.8	55.8
Rwanda	South/Amajyepfo	Nyanza	21995	2000	154.2	146.6	161.3
Rwanda	South/Amajyepfo	Nyanza	21995	2005	107.5	102.6	112.8
Rwanda	South/Amajyepfo	Nyanza	21995	2010	71.1	67.1	75.3
Rwanda	South/Amajyepfo	Nyanza	21995	2015	57.9	53.5	63.0
Rwanda	South/Amajyepfo	Nyaruguru	21997	2000	162.7	155.4	170.4
Rwanda	South/Amajyepfo	Nyaruguru	21997	2005	115.0	109.3	120.7
Rwanda	South/Amajyepfo	Nyaruguru	21997	2010	80.1	75.5	84.9
Rwanda	South/Amajyepfo	Nyaruguru	21997	2015	61.9	57.0	67.5
Rwanda	South/Amajyepfo	Ruhango	21999	2000	140.6	134.6	146.9
Rwanda	South/Amajyepfo	Ruhango	21999	2005	99.2	94.7	103.7
Rwanda	South/Amajyepfo	Ruhango	21999	2010	65.5	61.8	69.4
Rwanda	South/Amajyepfo	Ruhango	21999	2015	52.0	47.8	56.3
Rwanda	West/Iburengerazuba	Karongi	21983	2000	134.0	127.6	140.2
Rwanda	West/Iburengerazuba	Karongi	21983	2005	92.7	87.6	97.7
Rwanda	West/Iburengerazuba	Karongi	21983	2010	60.9	57.1	64.9
Rwanda	West/Iburengerazuba	Karongi	21983	2015	47.0	42.9	51.4
Rwanda	West/Iburengerazuba	Ngororero	21990	2000	146.0	140.0	151.7
Rwanda	West/Iburengerazuba	Ngororero	21990	2005	98.1	93.6	102.8
Rwanda	West/Iburengerazuba	Ngororero	21990	2010	62.9	59.2	66.8
Rwanda	West/Iburengerazuba	Ngororero	21990	2015	48.2	44.2	52.3
Rwanda	West/Iburengerazuba	Nyabihu	21991	2000	158.7	150.9	166.6
Rwanda	West/Iburengerazuba	Nyabihu	21991	2005	104.1	98.8	109.9
Rwanda	West/Iburengerazuba	Nyabihu	21991	2010	66.2	62.0	70.8
Rwanda	West/Iburengerazuba	Nyabihu	21991	2015	48.8	44.4	53.6
Rwanda	West/Iburengerazuba	Nyamasheke	21994	2000	130.9	123.6	138.6
Rwanda	West/Iburengerazuba	Nyamasheke	21994	2005	92.7	87.6	98.2
Rwanda	West/Iburengerazuba	Nyamasheke	21994	2010	62.5	58.2	66.9
Rwanda	West/Iburengerazuba	Nyamasheke	21994	2015	48.3	43.8	53.6
Rwanda	West/Iburengerazuba	Rubavu	21998	2000	152.2	144.1	160.4
Rwanda	West/Iburengerazuba	Rubavu	21998	2005	103.0	97.2	109.5
Rwanda	West/Iburengerazuba	Rubavu	21998	2010	63.0	58.4	67.4

Admin 0	Admin 1	Admin 2	GAUL Code	Year	Under-5 mortality (per 1,000 livebirths) Estimate	Lower bound, 95% UI	Upper bound, 95% UI
Rwanda	West/Iburengerazuba	Rubavu	21998	2015	48.3	43.9	53.0
Rwanda	West/Iburengerazuba	Rusizi	22001	2000	138.8	130.6	147.1
Rwanda	West/Iburengerazuba	Rusizi	22001	2005	102.5	96.7	108.7
Rwanda	West/Iburengerazuba	Rusizi	22001	2010	70.6	65.7	75.9
Rwanda	West/Iburengerazuba	Rusizi	22001	2015	54.8	49.8	60.2
Rwanda	West/Iburengerazuba	Rutsiro	22002	2000	144.5	137.1	151.7
Rwanda	West/Iburengerazuba	Rutsiro	22002	2005	97.3	92.2	102.7
Rwanda	West/Iburengerazuba	Rutsiro	22002	2010	62.1	57.8	66.1
Rwanda	West/Iburengerazuba	Rutsiro	22002	2015	47.3	43.2	52.0
Senegal	Dakar	Dakar	1382	2000	56.5	51.8	61.5
Senegal	Dakar	Dakar	1382	2005	52.3	47.9	57.2
Senegal	Dakar	Dakar	1382	2010	45.9	41.2	50.8
Senegal	Dakar	Dakar	1382	2015	37.3	32.7	42.5
Senegal	Dakar	Rufisque	25321	2000	64.7	60.0	69.8
Senegal	Dakar	Rufisque	25321	2005	56.9	52.3	61.4
Senegal	Dakar	Rufisque	25321	2010	48.2	43.9	52.7
Senegal	Dakar	Rufisque	25321	2015	38.3	33.9	43.4
Senegal	Diourbel	Bambey	25322	2000	113.7	107.4	120.5
Senegal	Diourbel	Bambey	25322	2005	87.3	81.9	93.2
Senegal	Diourbel	Bambey	25322	2010	63.8	59.1	68.9
Senegal	Diourbel	Bambey	25322	2015	48.0	43.4	53.2
Senegal	Diourbel	Diourbel	25323	2000	121.4	114.7	128.8
Senegal	Diourbel	Diourbel	25323	2005	92.8	86.9	98.7
Senegal	Diourbel	Diourbel	25323	2010	68.1	63.2	73.3
Senegal	Diourbel	Diourbel	25323	2015	52.2	47.3	57.6
Senegal	Diourbel	Mbacke	47596	2000	136.6	129.0	144.1
Senegal	Diourbel	Mbacke	47596	2005	104.6	98.3	111.2
Senegal	Diourbel	Mbacke	47596	2010	76.3	70.6	81.9
Senegal	Diourbel	Mbacke	47596	2015	57.7	52.1	63.9
Senegal	Fatick	Fatick	25325	2000	114.9	108.6	121.7
Senegal	Fatick	Fatick	25325	2005	86.1	80.9	91.4
Senegal	Fatick	Fatick	25325	2010	63.9	59.5	68.6
Senegal	Fatick	Fatick	25325	2015	48.5	43.8	53.2
Senegal	Fatick	Foundiougne	25326	2000	110.2	103.8	117.4
Senegal	Fatick	Foundiougne	25326	2005	88.5	83.1	94.1
Senegal	Fatick	Foundiougne	25326	2010	66.0	61.5	70.4
Senegal	Fatick	Foundiougne	25326	2015	49.6	45.0	54.8
Senegal	Fatick	Gossas	1383	2000	142.5	135.3	150.2
Senegal	Fatick	Gossas	1383	2005	105.7	99.3	112.2
Senegal	Fatick	Gossas	1383	2010	77.5	72.3	83.1
Senegal	Fatick	Gossas	1383	2015	57.1	51.7	63.1
Senegal	Kaffrine	Birkilane	1380	2000	148.7	141.1	156.4
Senegal	Kaffrine	Birkilane	1380	2005	109.2	103.6	115.3
Senegal	Kaffrine	Birkilane	1380	2010	76.5	72.1	81.8
Senegal	Kaffrine	Birkilane	1380	2015	56.2	51.1	62.1
Senegal	Kaffrine	Kaffrine	1396	2000	148.6	140.2	157.1
Senegal	Kaffrine	Kaffrine	1396	2005	111.7	105.6	118.4
Senegal	Kaffrine	Kaffrine	1396	2010	76.2	70.9	81.2
Senegal	Kaffrine	Kaffrine	1396	2015	58.2	52.3	64.5
Senegal	Kaffrine	Koungueul	1486	2000	145.8	136.6	156.1
Senegal	Kaffrine	Koungueul	1486	2005	112.7	105.4	120.7
Senegal	Kaffrine	Koungueul	1486	2010	88.5	81.6	95.8
Senegal	Kaffrine	Koungueul	1486	2015	64.4	57.7	71.6
Senegal	Kaffrine	Maleme hodar	1497	2000	145.2	136.6	154.2
Senegal	Kaffrine	Maleme hodar	1497	2005	110.9	103.9	117.5
Senegal	Kaffrine	Maleme hodar	1497	2010	83.4	77.6	89.3
Senegal	Kaffrine	Maleme hodar	1497	2015	61.6	55.5	68.3
Senegal	Kaolack	Guinguineo	1395	2000	130.9	123.9	138.7
Senegal	Kaolack	Guinguineo	1395	2005	95.8	90.1	101.9
Senegal	Kaolack	Guinguineo	1395	2010	70.9	66.2	76.1
Senegal	Kaolack	Guinguineo	1395	2015	51.9	47.1	57.0
Senegal	Kaolack	Kaolack	25329	2000	118.6	113.0	125.1
Senegal	Kaolack	Kaolack	25329	2005	89.4	84.7	94.3
Senegal	Kaolack	Kaolack	25329	2010	65.7	61.8	69.9
Senegal	Kaolack	Kaolack	25329	2015	48.7	44.4	53.3
Senegal	Kaolack	Nioro du rip	25330	2000	144.9	138.3	152.7
Senegal	Kaolack	Nioro du rip	25330	2005	105.3	100.0	110.7
Senegal	Kaolack	Nioro du rip	25330	2010	68.9	65.1	73.2
Senegal	Kaolack	Nioro du rip	25330	2015	53.4	48.6	58.7
Senegal	Kedougou	Kedougou	1397	2000	187.8	174.5	201.6
Senegal	Kedougou	Kedougou	1397	2005	152.8	141.9	164.0
Senegal	Kedougou	Kedougou	1397	2010	121.2	111.2	132.2
Senegal	Kedougou	Kedougou	1397	2015	98.2	86.8	110.1
Senegal	Kedougou	Salemata	1510	2000	178.0	162.7	194.6
Senegal	Kedougou	Salemata	1510	2005	149.8	137.3	164.1
Senegal	Kedougou	Salemata	1510	2010	119.1	107.2	131.8
Senegal	Kedougou	Salemata	1510	2015	96.4	84.9	109.5
Senegal	Kedougou	Saraya	1538	2000	209.5	193.5	226.9
Senegal	Kedougou	Saraya	1538	2005	171.5	159.1	184.6
Senegal	Kedougou	Saraya	1538	2010	138.5	126.6	151.1
Senegal	Kedougou	Saraya	1538	2015	117.8	104.9	132.6
Senegal	Kolda	Kolda	1408	2000	186.1	176.1	196.3
Senegal	Kolda	Kolda	1408	2005	149.5	141.4	157.5
Senegal	Kolda	Kolda	1408	2010	99.3	93.1	106.3
Senegal	Kolda	Kolda	1408	2015	85.4	77.5	93.2

Admin 0	Admin 1	Admin 2	GAUL Code	Year	Under-5 mortality (per 1,000 livebirths)		
					Estimate	Lower bound, 95% UI	Upper bound, 95% UI
Senegal	Kolda	Medina yoro foula	1499	2000	163.3	154.3	172.6
Senegal	Kolda	Medina yoro foula	1499	2005	126.6	119.2	133.5
Senegal	Kolda	Medina yoro foula	1499	2010	87.0	81.0	93.0
Senegal	Kolda	Medina yoro foula	1499	2015	74.1	67.2	81.5
Senegal	Kolda	Velingara	25333	2000	184.0	173.8	194.5
Senegal	Kolda	Velingara	25333	2005	150.5	142.3	158.8
Senegal	Kolda	Velingara	25333	2010	111.0	103.4	119.1
Senegal	Kolda	Velingara	25333	2015	92.0	83.7	102.2
Senegal	Louga	Kebemer	25334	2000	108.1	101.6	115.2
Senegal	Louga	Kebemer	25334	2005	85.6	80.4	91.0
Senegal	Louga	Kebemer	25334	2010	64.9	59.8	69.7
Senegal	Louga	Kebemer	25334	2015	48.7	43.8	53.9
Senegal	Louga	Linguere	47594	2000	124.4	114.9	134.7
Senegal	Louga	Linguere	47594	2005	95.7	88.4	103.4
Senegal	Louga	Linguere	47594	2010	72.9	66.9	79.7
Senegal	Louga	Linguere	47594	2015	52.2	46.4	58.9
Senegal	Louga	Louga	25336	2000	95.8	89.6	103.2
Senegal	Louga	Louga	25336	2005	80.0	74.8	85.8
Senegal	Louga	Louga	25336	2010	62.0	56.9	67.3
Senegal	Louga	Louga	25336	2015	46.2	41.5	51.7
Senegal	Matam	Kanel	47593	2000	111.7	103.7	119.8
Senegal	Matam	Kanel	47593	2005	93.4	86.9	100.4
Senegal	Matam	Kanel	47593	2010	75.6	69.2	82.3
Senegal	Matam	Kanel	47593	2015	59.6	52.7	66.7
Senegal	Matam	Matam	47595	2000	110.5	103.2	117.9
Senegal	Matam	Matam	47595	2005	89.2	83.3	95.1
Senegal	Matam	Matam	47595	2010	72.6	67.0	79.0
Senegal	Matam	Matam	47595	2015	56.4	49.5	63.2
Senegal	Matam	Ranerou	47599	2000	128.9	117.4	140.7
Senegal	Matam	Ranerou	47599	2005	107.0	97.6	116.6
Senegal	Matam	Ranerou	47599	2010	87.9	79.4	97.3
Senegal	Matam	Ranerou	47599	2015	60.7	53.8	69.0
Senegal	Saint louis	Dagana	47590	2000	91.6	84.8	99.0
Senegal	Saint louis	Dagana	47590	2005	74.5	69.0	80.4
Senegal	Saint louis	Dagana	47590	2010	60.5	55.3	67.0
Senegal	Saint louis	Dagana	47590	2015	44.6	39.0	50.8
Senegal	Saint louis	Podor	47598	2000	118.2	110.4	126.2
Senegal	Saint louis	Podor	47598	2005	88.6	82.8	94.6
Senegal	Saint louis	Podor	47598	2010	67.1	61.8	73.5
Senegal	Saint louis	Podor	47598	2015	48.7	43.0	54.9
Senegal	Saint louis	Saint louis	47600	2000	71.6	64.9	79.0
Senegal	Saint louis	Saint louis	47600	2005	63.8	58.0	70.0
Senegal	Saint louis	Saint louis	47600	2010	55.0	49.3	61.6
Senegal	Saint louis	Saint louis	47600	2015	41.9	36.5	47.8
Senegal	Sedhiou	Bounkiling	1381	2000	160.0	149.4	169.9
Senegal	Sedhiou	Bounkiling	1381	2005	124.4	116.9	132.4
Senegal	Sedhiou	Bounkiling	1381	2010	81.5	75.9	87.8
Senegal	Sedhiou	Bounkiling	1381	2015	63.5	57.4	70.2
Senegal	Sedhiou	Goudomp	1394	2000	175.4	165.6	185.5
Senegal	Sedhiou	Goudomp	1394	2005	129.0	122.0	136.3
Senegal	Sedhiou	Goudomp	1394	2010	85.0	79.2	90.6
Senegal	Sedhiou	Goudomp	1394	2015	68.2	61.9	75.1
Senegal	Sedhiou	Sedhiou	1609	2000	171.7	162.1	181.5
Senegal	Sedhiou	Sedhiou	1609	2005	128.2	121.2	135.8
Senegal	Sedhiou	Sedhiou	1609	2010	84.1	78.9	89.7
Senegal	Sedhiou	Sedhiou	1609	2015	65.5	59.4	71.9
Senegal	Tambacounda	Bakel	1379	2000	131.4	122.5	141.3
Senegal	Tambacounda	Bakel	1379	2005	105.8	98.5	114.3
Senegal	Tambacounda	Bakel	1379	2010	85.7	78.7	93.1
Senegal	Tambacounda	Bakel	1379	2015	72.2	64.4	81.0
Senegal	Tambacounda	Goudiry	1384	2000	155.2	142.9	169.4
Senegal	Tambacounda	Goudiry	1384	2005	124.4	114.6	135.4
Senegal	Tambacounda	Goudiry	1384	2010	101.5	92.1	111.4
Senegal	Tambacounda	Goudiry	1384	2015	83.2	73.8	93.1
Senegal	Tambacounda	Koupentoum	1488	2000	162.6	151.1	174.3
Senegal	Tambacounda	Koupentoum	1488	2005	132.0	122.9	141.7
Senegal	Tambacounda	Koupentoum	1488	2010	109.7	100.7	118.7
Senegal	Tambacounda	Koupentoum	1488	2015	80.9	71.7	89.6
Senegal	Tambacounda	Tambacounda	1649	2000	167.9	158.8	177.9
Senegal	Tambacounda	Tambacounda	1649	2005	130.1	122.4	138.3
Senegal	Tambacounda	Tambacounda	1649	2010	99.1	92.3	106.2
Senegal	Tambacounda	Tambacounda	1649	2015	82.7	74.5	90.7
Senegal	Thies	Mbour	25343	2000	80.9	75.3	86.7
Senegal	Thies	Mbour	25343	2005	66.7	62.3	72.0
Senegal	Thies	Mbour	25343	2010	53.7	49.6	58.3
Senegal	Thies	Mbour	25343	2015	41.8	37.7	46.6
Senegal	Thies	Thies	25344	2000	79.0	74.0	84.1
Senegal	Thies	Thies	25344	2005	65.1	60.6	69.5
Senegal	Thies	Thies	25344	2010	52.2	47.9	56.5
Senegal	Thies	Thies	25344	2015	40.8	36.4	45.4
Senegal	Thies	Tivaoune	25345	2000	92.8	87.0	98.9
Senegal	Thies	Tivaoune	25345	2005	74.8	69.6	79.9
Senegal	Thies	Tivaoune	25345	2010	58.1	53.3	62.9
Senegal	Thies	Tivaoune	25345	2015	44.1	39.5	48.8
Senegal	Ziguinchor	Bignona	25346	2000	110.9	103.6	118.2

Admin 0	Admin 1	Admin 2	GAUL Code	Year	Under-5 mortality (per 1,000 livebirths)		
					Estimate	Lower bound, 95% UI	Upper bound, 95% UI
Senegal	Ziguinchor	Bignona	25346	2005	90.4	85.1	96.9
Senegal	Ziguinchor	Bignona	25346	2010	66.7	61.8	72.1
Senegal	Ziguinchor	Bignona	25346	2015	50.5	45.3	56.0
Senegal	Ziguinchor	Oussouye	25347	2000	99.5	87.8	111.1
Senegal	Ziguinchor	Oussouye	25347	2005	82.5	73.6	92.5
Senegal	Ziguinchor	Oussouye	25347	2010	63.1	55.6	70.9
Senegal	Ziguinchor	Oussouye	25347	2015	49.3	42.9	57.0
Senegal	Ziguinchor	Ziguinchor	25348	2000	104.5	96.3	112.9
Senegal	Ziguinchor	Ziguinchor	25348	2005	85.6	79.4	92.6
Senegal	Ziguinchor	Ziguinchor	25348	2010	62.9	57.6	68.9
Senegal	Ziguinchor	Ziguinchor	25348	2015	49.5	44.2	55.7
Sierra Leone	Eastern	Kailahun	25406	2000	232.4	220.6	244.8
Sierra Leone	Eastern	Kailahun	25406	2005	191.7	182.1	202.0
Sierra Leone	Eastern	Kailahun	25406	2010	155.5	145.3	166.3
Sierra Leone	Eastern	Kailahun	25406	2015	127.9	115.8	142.0
Sierra Leone	Eastern	Kenema	25407	2000	254.0	242.5	265.8
Sierra Leone	Eastern	Kenema	25407	2005	213.1	203.3	223.7
Sierra Leone	Eastern	Kenema	25407	2010	170.8	160.1	180.9
Sierra Leone	Eastern	Kenema	25407	2015	141.1	127.8	155.4
Sierra Leone	Eastern	Kono	25408	2000	226.0	213.2	238.2
Sierra Leone	Eastern	Kono	25408	2005	189.5	179.5	200.1
Sierra Leone	Eastern	Kono	25408	2010	163.8	152.8	175.5
Sierra Leone	Eastern	Kono	25408	2015	140.4	127.2	155.5
Sierra Leone	Northern	Bombali	25409	2000	167.3	157.0	177.1
Sierra Leone	Northern	Bombali	25409	2005	147.6	138.6	156.8
Sierra Leone	Northern	Bombali	25409	2010	129.5	120.5	139.2
Sierra Leone	Northern	Bombali	25409	2015	106.7	97.4	118.4
Sierra Leone	Northern	Kambia	25410	2000	178.8	167.1	190.0
Sierra Leone	Northern	Kambia	25410	2005	160.7	150.0	170.9
Sierra Leone	Northern	Kambia	25410	2010	129.5	120.4	139.2
Sierra Leone	Northern	Kambia	25410	2015	106.7	96.4	117.9
Sierra Leone	Northern	Koinadugu	25411	2000	231.3	218.3	245.0
Sierra Leone	Northern	Koinadugu	25411	2005	202.3	191.3	213.8
Sierra Leone	Northern	Koinadugu	25411	2010	166.9	156.0	178.7
Sierra Leone	Northern	Koinadugu	25411	2015	132.0	119.7	145.0
Sierra Leone	Northern	Port Loko	25412	2000	206.8	196.7	217.1
Sierra Leone	Northern	Port Loko	25412	2005	189.7	180.6	199.6
Sierra Leone	Northern	Port Loko	25412	2010	154.1	144.0	163.4
Sierra Leone	Northern	Port Loko	25412	2015	120.9	110.2	132.4
Sierra Leone	Northern	Tonkolili	25413	2000	235.8	225.3	247.6
Sierra Leone	Northern	Tonkolili	25413	2005	203.4	193.7	213.1
Sierra Leone	Northern	Tonkolili	25413	2010	177.5	167.1	188.5
Sierra Leone	Northern	Tonkolili	25413	2015	143.7	131.4	157.2
Sierra Leone	Southern	Bo	25414	2000	227.4	216.1	238.9
Sierra Leone	Southern	Bo	25414	2005	191.1	181.7	201.1
Sierra Leone	Southern	Bo	25414	2010	151.2	142.1	160.4
Sierra Leone	Southern	Bo	25414	2015	125.0	113.1	137.9
Sierra Leone	Southern	Bonthe	25415	2000	209.7	195.6	223.9
Sierra Leone	Southern	Bonthe	25415	2005	176.6	164.9	188.5
Sierra Leone	Southern	Bonthe	25415	2010	144.2	133.2	155.8
Sierra Leone	Southern	Bonthe	25415	2015	120.1	108.2	133.0
Sierra Leone	Southern	Moyamba	25416	2000	248.4	235.4	261.5
Sierra Leone	Southern	Moyamba	25416	2005	215.3	203.5	227.4
Sierra Leone	Southern	Moyamba	25416	2010	177.9	167.3	189.7
Sierra Leone	Southern	Moyamba	25416	2015	144.8	130.7	159.7
Sierra Leone	Southern	Pujehun	25417	2000	272.0	257.6	288.2
Sierra Leone	Southern	Pujehun	25417	2005	226.3	214.3	238.3
Sierra Leone	Southern	Pujehun	25417	2010	181.7	169.8	194.7
Sierra Leone	Southern	Pujehun	25417	2015	151.7	137.3	168.7
Sierra Leone	Western Area	Western Area Rur	111380	2000	170.6	159.1	182.9
Sierra Leone	Western Area	Western Area Rur	111380	2005	164.0	152.5	175.8
Sierra Leone	Western Area	Western Area Rur	111380	2010	133.4	123.2	143.2
Sierra Leone	Western Area	Western Area Rur	111380	2015	96.6	85.5	107.7
Sierra Leone	Western Area	Western Area Urb	111381	2000	134.4	124.4	145.7
Sierra Leone	Western Area	Western Area Urb	111381	2005	133.3	123.1	144.3
Sierra Leone	Western Area	Western Area Urb	111381	2010	113.2	103.6	122.8
Sierra Leone	Western Area	Western Area Urb	111381	2015	79.2	69.8	89.4
Somalia	Awdal	Baki	25653	2000	108.8	96.9	123.2
Somalia	Awdal	Baki	25653	2005	96.2	84.9	107.1
Somalia	Awdal	Baki	25653	2010	97.2	85.1	110.0
Somalia	Awdal	Baki	25653	2015	73.1	62.3	84.7
Somalia	Awdal	Borama	25654	2000	101.8	91.3	113.6
Somalia	Awdal	Borama	25654	2005	89.7	80.5	99.8
Somalia	Awdal	Borama	25654	2010	89.7	78.7	101.5
Somalia	Awdal	Borama	25654	2015	68.9	58.2	80.3
Somalia	Awdal	Lughaye	25655	2000	112.0	96.8	129.5
Somalia	Awdal	Lughaye	25655	2005	96.1	83.4	110.3
Somalia	Awdal	Lughaye	25655	2010	93.9	80.3	108.6
Somalia	Awdal	Lughaye	25655	2015	73.3	61.1	87.6
Somalia	Awdal	Zeylac	25656	2000	104.1	92.4	117.1
Somalia	Awdal	Zeylac	25656	2005	87.1	76.8	98.2
Somalia	Awdal	Zeylac	25656	2010	86.9	74.9	99.3
Somalia	Awdal	Zeylac	25656	2015	68.7	57.6	80.4
Somalia	Bakool	Ceel Barde	25657	2000	125.0	102.3	153.9
Somalia	Bakool	Ceel Barde	25657	2005	111.5	90.8	136.5

Admin 0	Admin 1	Admin 2	GAUL Code	Year	Under-5 mortality (per 1,000 livebirths)		
					Estimate	Lower bound, 95% UI	Upper bound, 95% UI
Somalia	Bakool	Ceel Barde	25657	2010	109.0	89.3	135.6
Somalia	Bakool	Ceel Barde	25657	2015	88.5	70.1	110.3
Somalia	Bakool	Rab Dhuure	25658	2000	134.7	111.4	163.6
Somalia	Bakool	Rab Dhuure	25658	2005	125.7	103.4	152.1
Somalia	Bakool	Rab Dhuure	25658	2010	113.2	92.8	136.7
Somalia	Bakool	Rab Dhuure	25658	2015	89.8	72.3	110.1
Somalia	Bakool	Tayeeglow	25659	2000	130.8	109.3	155.4
Somalia	Bakool	Tayeeglow	25659	2005	130.3	108.5	156.0
Somalia	Bakool	Tayeeglow	25659	2010	117.5	97.0	143.6
Somalia	Bakool	Tayeeglow	25659	2015	98.8	79.1	121.7
Somalia	Bakool	Waajid	25660	2000	135.3	112.5	162.2
Somalia	Bakool	Waajid	25660	2005	128.8	106.5	154.1
Somalia	Bakool	Waajid	25660	2010	112.4	91.3	135.7
Somalia	Bakool	Waajid	25660	2015	93.0	74.8	114.4
Somalia	Bakool	Xudur	25661	2000	127.3	105.7	153.4
Somalia	Bakool	Xudur	25661	2005	117.9	98.0	140.9
Somalia	Bakool	Xudur	25661	2010	111.5	91.0	135.0
Somalia	Bakool	Xudur	25661	2015	90.6	72.3	111.4
Somalia	Banadir	Banadir	25672	2000	203.9	185.5	222.8
Somalia	Banadir	Banadir	25672	2005	167.4	151.3	185.0
Somalia	Banadir	Banadir	25672	2010	161.0	139.6	183.2
Somalia	Banadir	Banadir	25672	2015	123.9	105.0	143.7
Somalia	Bari	Bandarbeyla	25662	2000	116.5	93.8	143.5
Somalia	Bari	Bandarbeyla	25662	2005	107.0	85.5	132.3
Somalia	Bari	Bandarbeyla	25662	2010	118.7	93.9	147.7
Somalia	Bari	Bandarbeyla	25662	2015	90.9	71.2	112.5
Somalia	Bari	Bossaso	25663	2000	123.4	104.0	146.3
Somalia	Bari	Bossaso	25663	2005	109.4	91.6	129.6
Somalia	Bari	Bossaso	25663	2010	118.5	97.8	140.8
Somalia	Bari	Bossaso	25663	2015	98.6	79.5	122.3
Somalia	Bari	Caluula	25664	2000	117.5	91.9	147.7
Somalia	Bari	Caluula	25664	2005	103.7	82.1	129.6
Somalia	Bari	Caluula	25664	2010	115.8	91.3	144.6
Somalia	Bari	Caluula	25664	2015	86.9	67.2	109.5
Somalia	Bari	Iskushuban	25665	2000	117.2	96.4	141.5
Somalia	Bari	Iskushuban	25665	2005	108.3	88.9	130.8
Somalia	Bari	Iskushuban	25665	2010	119.7	96.7	145.0
Somalia	Bari	Iskushuban	25665	2015	93.6	75.1	114.8
Somalia	Bari	Qandala	25666	2000	119.6	97.6	143.5
Somalia	Bari	Qandala	25666	2005	107.1	88.6	126.5
Somalia	Bari	Qandala	25666	2010	119.8	97.2	143.4
Somalia	Bari	Qandala	25666	2015	91.3	73.1	111.5
Somalia	Bari	Qardho	25667	2000	118.9	96.9	143.3
Somalia	Bari	Qardho	25667	2005	108.9	88.7	131.5
Somalia	Bari	Qardho	25667	2010	116.1	94.3	142.7
Somalia	Bari	Qardho	25667	2015	88.8	71.2	111.1
Somalia	Bay	Baydhaba	25668	2000	184.1	159.2	209.8
Somalia	Bay	Baydhaba	25668	2005	162.3	140.3	185.0
Somalia	Bay	Baydhaba	25668	2010	128.6	108.6	150.4
Somalia	Bay	Baydhaba	25668	2015	99.6	82.8	119.5
Somalia	Bay	Buur Hakaba	25669	2000	209.2	181.1	240.2
Somalia	Bay	Buur Hakaba	25669	2005	168.3	146.7	192.0
Somalia	Bay	Buur Hakaba	25669	2010	146.4	125.1	171.4
Somalia	Bay	Buur Hakaba	25669	2015	114.2	96.0	137.3
Somalia	Bay	Diinsoor	25670	2000	163.0	137.1	191.5
Somalia	Bay	Diinsoor	25670	2005	147.6	124.6	174.5
Somalia	Bay	Diinsoor	25670	2010	122.2	102.9	144.6
Somalia	Bay	Diinsoor	25670	2015	106.7	88.2	127.2
Somalia	Bay	Qansax Dheere	25671	2000	166.5	140.4	195.8
Somalia	Bay	Qansax Dheere	25671	2005	162.1	136.8	191.0
Somalia	Bay	Qansax Dheere	25671	2010	122.8	102.4	147.5
Somalia	Bay	Qansax Dheere	25671	2015	102.4	83.7	122.7
Somalia	Galgaduud	Cabudwaaq	25673	2000	145.1	119.4	174.3
Somalia	Galgaduud	Cabudwaaq	25673	2005	125.7	103.9	151.8
Somalia	Galgaduud	Cabudwaaq	25673	2010	123.9	101.4	151.5
Somalia	Galgaduud	Cabudwaaq	25673	2015	88.6	70.7	109.2
Somalia	Galgaduud	Cadaado	25674	2000	148.2	121.0	179.6
Somalia	Galgaduud	Cadaado	25674	2005	125.9	104.4	152.2
Somalia	Galgaduud	Cadaado	25674	2010	125.5	101.7	153.9
Somalia	Galgaduud	Cadaado	25674	2015	88.8	70.3	109.1
Somalia	Galgaduud	Ceel Buur	25675	2000	147.7	121.4	177.7
Somalia	Galgaduud	Ceel Buur	25675	2005	126.1	104.7	151.2
Somalia	Galgaduud	Ceel Buur	25675	2010	126.1	102.5	153.1
Somalia	Galgaduud	Ceel Buur	25675	2015	88.0	69.9	107.9
Somalia	Galgaduud	Ceel Dheer	25676	2000	147.8	120.5	177.9
Somalia	Galgaduud	Ceel Dheer	25676	2005	123.5	101.7	150.0
Somalia	Galgaduud	Ceel Dheer	25676	2010	124.3	100.9	150.9
Somalia	Galgaduud	Ceel Dheer	25676	2015	87.0	69.1	107.2
Somalia	Galgaduud	Dhuusamarreeb	25677	2000	149.1	121.4	183.3
Somalia	Galgaduud	Dhuusamarreeb	25677	2005	126.3	101.8	153.6
Somalia	Galgaduud	Dhuusamarreeb	25677	2010	123.9	98.9	154.2
Somalia	Galgaduud	Dhuusamarreeb	25677	2015	88.9	70.0	111.1
Somalia	Gedo	Baardheere	25678	2000	118.7	100.7	139.8
Somalia	Gedo	Baardheere	25678	2005	109.7	92.5	128.1
Somalia	Gedo	Baardheere	25678	2010	101.4	84.6	120.3

Admin 0	Admin 1	Admin 2	GAUL Code	Year	Under-5 mortality (per 1,000 livebirths)		
					Estimate	Lower bound, 95% UI	Upper bound, 95% UI
Somalia	Gedo	Baardheere	25678	2015	90.4	74.3	109.0
Somalia	Gedo	Belet Xaawo	25679	2000	99.8	87.6	111.8
Somalia	Gedo	Belet Xaawo	25679	2005	91.1	80.7	101.6
Somalia	Gedo	Belet Xaawo	25679	2010	88.8	77.3	101.3
Somalia	Gedo	Belet Xaawo	25679	2015	73.5	62.4	86.3
Somalia	Gedo	Ceel Waaq	25680	2000	84.2	74.2	95.8
Somalia	Gedo	Ceel Waaq	25680	2005	73.6	64.6	84.1
Somalia	Gedo	Ceel Waaq	25680	2010	75.9	65.3	87.6
Somalia	Gedo	Ceel Waaq	25680	2015	63.6	53.6	75.3
Somalia	Gedo	Doolow	25681	2000	116.2	102.6	131.8
Somalia	Gedo	Doolow	25681	2005	104.6	91.7	117.9
Somalia	Gedo	Doolow	25681	2010	102.2	88.2	118.2
Somalia	Gedo	Doolow	25681	2015	78.5	66.3	91.6
Somalia	Gedo	Garbahaarey	25682	2000	118.0	100.5	139.0
Somalia	Gedo	Garbahaarey	25682	2005	109.7	93.2	128.2
Somalia	Gedo	Garbahaarey	25682	2010	101.6	85.9	120.2
Somalia	Gedo	Garbahaarey	25682	2015	83.5	69.2	99.4
Somalia	Gedo	Luuq	25683	2000	131.1	110.8	154.0
Somalia	Gedo	Luuq	25683	2005	120.1	101.0	140.3
Somalia	Gedo	Luuq	25683	2010	111.4	92.0	132.8
Somalia	Gedo	Luuq	25683	2015	87.9	71.9	106.2
Somalia	Hiraan	Belet Weyne	25684	2000	118.3	97.9	140.3
Somalia	Hiraan	Belet Weyne	25684	2005	109.3	89.9	131.1
Somalia	Hiraan	Belet Weyne	25684	2010	113.2	92.3	139.4
Somalia	Hiraan	Belet Weyne	25684	2015	86.7	69.3	108.0
Somalia	Hiraan	Bulo Burto	25685	2000	125.8	104.4	148.5
Somalia	Hiraan	Bulo Burto	25685	2005	114.5	95.3	134.1
Somalia	Hiraan	Bulo Burto	25685	2010	114.9	94.9	138.6
Somalia	Hiraan	Bulo Burto	25685	2015	88.2	70.4	108.1
Somalia	Hiraan	Jalalaqsi	25686	2000	138.8	115.2	164.8
Somalia	Hiraan	Jalalaqsi	25686	2005	120.9	100.7	143.3
Somalia	Hiraan	Jalalaqsi	25686	2010	119.4	97.9	143.6
Somalia	Hiraan	Jalalaqsi	25686	2015	93.0	73.8	113.5
Somalia	Juba Dhexe	Bu'aale	25698	2000	141.9	118.1	171.8
Somalia	Juba Dhexe	Bu'aale	25698	2005	128.2	107.6	153.1
Somalia	Juba Dhexe	Bu'aale	25698	2010	127.6	104.6	155.0
Somalia	Juba Dhexe	Bu'aale	25698	2015	98.6	80.5	119.7
Somalia	Juba Dhexe	Jilib	25699	2000	168.1	141.2	198.5
Somalia	Juba Dhexe	Jilib	25699	2005	141.3	119.8	168.3
Somalia	Juba Dhexe	Jilib	25699	2010	140.4	116.4	172.0
Somalia	Juba Dhexe	Jilib	25699	2015	108.1	87.9	132.6
Somalia	Juba Dhexe	Saakow	25700	2000	137.7	114.5	165.0
Somalia	Juba Dhexe	Saakow	25700	2005	122.5	101.3	145.8
Somalia	Juba Dhexe	Saakow	25700	2010	120.2	98.8	145.2
Somalia	Juba Dhexe	Saakow	25700	2015	93.3	75.5	114.1
Somalia	Juba Hoose	Afmadow	25687	2000	141.3	121.9	164.4
Somalia	Juba Hoose	Afmadow	25687	2005	122.5	106.1	142.6
Somalia	Juba Hoose	Afmadow	25687	2010	113.0	96.1	133.2
Somalia	Juba Hoose	Afmadow	25687	2015	89.2	74.1	105.4
Somalia	Juba Hoose	Badhaadhe	25688	2000	141.9	121.4	165.4
Somalia	Juba Hoose	Badhaadhe	25688	2005	118.3	100.9	137.6
Somalia	Juba Hoose	Badhaadhe	25688	2010	118.3	100.5	138.5
Somalia	Juba Hoose	Badhaadhe	25688	2015	90.4	75.3	107.9
Somalia	Juba Hoose	Jamaame	25689	2000	176.2	146.7	208.5
Somalia	Juba Hoose	Jamaame	25689	2005	141.9	118.6	168.8
Somalia	Juba Hoose	Jamaame	25689	2010	135.7	113.0	163.2
Somalia	Juba Hoose	Jamaame	25689	2015	109.5	87.0	134.9
Somalia	Juba Hoose	Kismaayo	25690	2000	163.9	139.7	195.5
Somalia	Juba Hoose	Kismaayo	25690	2005	134.8	114.1	159.6
Somalia	Juba Hoose	Kismaayo	25690	2010	131.1	110.6	153.5
Somalia	Juba Hoose	Kismaayo	25690	2015	101.7	82.8	122.0
Somalia	Mudug	Gaalkacyo	25705	2000	135.6	115.5	157.8
Somalia	Mudug	Gaalkacyo	25705	2005	120.8	102.1	142.5
Somalia	Mudug	Gaalkacyo	25705	2010	123.6	103.3	149.7
Somalia	Mudug	Gaalkacyo	25705	2015	93.1	75.0	113.2
Somalia	Mudug	Galdogob	25706	2000	132.1	111.3	155.3
Somalia	Mudug	Galdogob	25706	2005	122.3	103.8	144.5
Somalia	Mudug	Galdogob	25706	2010	127.4	106.0	152.9
Somalia	Mudug	Galdogob	25706	2015	93.5	76.5	112.8
Somalia	Mudug	Hobyo	25707	2000	134.4	111.9	159.5
Somalia	Mudug	Hobyo	25707	2005	119.2	100.2	142.0
Somalia	Mudug	Hobyo	25707	2010	124.9	102.3	149.9
Somalia	Mudug	Hobyo	25707	2015	91.9	74.2	112.6
Somalia	Mudug	Jariiban	25708	2000	123.8	101.2	150.3
Somalia	Mudug	Jariiban	25708	2005	112.0	91.2	135.6
Somalia	Mudug	Jariiban	25708	2010	119.4	96.8	144.8
Somalia	Mudug	Jariiban	25708	2015	86.0	68.2	107.5
Somalia	Mudug	Xarardheere	25709	2000	137.3	111.2	167.1
Somalia	Mudug	Xarardheere	25709	2005	120.9	98.3	146.7
Somalia	Mudug	Xarardheere	25709	2010	126.9	101.9	156.2
Somalia	Mudug	Xarardheere	25709	2015	94.8	75.3	116.4
Somalia	Nugaal	Burtinle	25710	2000	145.4	121.2	171.1
Somalia	Nugaal	Burtinle	25710	2005	125.2	104.4	148.3
Somalia	Nugaal	Burtinle	25710	2010	123.9	102.5	146.9
Somalia	Nugaal	Burtinle	25710	2015	86.9	70.8	106.7

Admin 0	Admin 1	Admin 2	GAUL Code	Year	Under-5 mortality (per 1,000 livebirths)		
					Estimate	Lower bound, 95% UI	Upper bound, 95% UI
Somalia	Nugaal	Eyl	25711	2000	138.1	113.1	166.7
Somalia	Nugaal	Eyl	25711	2005	117.2	94.4	141.3
Somalia	Nugaal	Eyl	25711	2010	121.1	98.6	148.5
Somalia	Nugaal	Eyl	25711	2015	89.7	72.3	110.6
Somalia	Nugaal	Garoowe	25712	2000	147.2	122.1	175.7
Somalia	Nugaal	Garoowe	25712	2005	123.7	102.2	148.4
Somalia	Nugaal	Garoowe	25712	2010	122.1	99.8	148.1
Somalia	Nugaal	Garoowe	25712	2015	87.5	70.1	108.0
Somalia	Sanaag	Ceel Afweyn	25714	2000	127.6	110.5	145.4
Somalia	Sanaag	Ceel Afweyn	25714	2005	117.5	102.6	135.5
Somalia	Sanaag	Ceel Afweyn	25714	2010	128.4	110.2	149.1
Somalia	Sanaag	Ceel Afweyn	25714	2015	86.2	72.2	102.0
Somalia	Sanaag	Ceerigaabo	25715	2000	120.7	105.0	137.6
Somalia	Sanaag	Ceerigaabo	25715	2005	106.8	93.0	121.4
Somalia	Sanaag	Ceerigaabo	25715	2010	115.6	99.3	132.7
Somalia	Sanaag	Ceerigaabo	25715	2015	81.9	68.2	96.4
Somalia	Sanaag	Laasqoray	25713	2000	120.6	102.3	141.8
Somalia	Sanaag	Laasqoray	25713	2005	107.4	90.5	127.3
Somalia	Sanaag	Laasqoray	25713	2010	117.6	98.3	139.5
Somalia	Sanaag	Laasqoray	25713	2015	85.7	70.5	103.8
Somalia	Shabelle Dhexe	Adan Yabaal	25701	2000	131.8	106.9	158.6
Somalia	Shabelle Dhexe	Adan Yabaal	25701	2005	116.8	96.0	138.8
Somalia	Shabelle Dhexe	Adan Yabaal	25701	2010	118.8	96.5	145.1
Somalia	Shabelle Dhexe	Adan Yabaal	25701	2015	87.5	69.9	108.0
Somalia	Shabelle Dhexe	Balcad	25702	2000	179.4	160.9	200.6
Somalia	Shabelle Dhexe	Balcad	25702	2005	152.8	136.2	171.0
Somalia	Shabelle Dhexe	Balcad	25702	2010	143.1	124.7	164.8
Somalia	Shabelle Dhexe	Balcad	25702	2015	123.0	104.7	143.6
Somalia	Shabelle Dhexe	Cadale	25703	2000	142.7	117.8	170.7
Somalia	Shabelle Dhexe	Cadale	25703	2005	122.1	99.9	147.2
Somalia	Shabelle Dhexe	Cadale	25703	2010	121.7	99.3	148.2
Somalia	Shabelle Dhexe	Cadale	25703	2015	90.8	72.8	110.6
Somalia	Shabelle Dhexe	Jowhar	25704	2000	156.5	135.8	180.9
Somalia	Shabelle Dhexe	Jowhar	25704	2005	135.3	116.8	154.8
Somalia	Shabelle Dhexe	Jowhar	25704	2010	128.2	109.1	151.2
Somalia	Shabelle Dhexe	Jowhar	25704	2015	106.9	88.9	128.1
Somalia	Shabelle Hoose	Afgooye	25691	2000	197.9	180.3	215.3
Somalia	Shabelle Hoose	Afgooye	25691	2005	166.4	150.7	184.0
Somalia	Shabelle Hoose	Afgooye	25691	2010	157.4	136.9	178.4
Somalia	Shabelle Hoose	Afgooye	25691	2015	125.1	106.5	144.9
Somalia	Shabelle Hoose	Baraawe	25692	2000	196.3	167.4	229.7
Somalia	Shabelle Hoose	Baraawe	25692	2005	163.8	139.7	191.8
Somalia	Shabelle Hoose	Baraawe	25692	2010	154.0	127.6	184.7
Somalia	Shabelle Hoose	Baraawe	25692	2015	128.2	104.3	157.5
Somalia	Shabelle Hoose	Kurtunwaarey	25693	2000	208.2	178.0	242.3
Somalia	Shabelle Hoose	Kurtunwaarey	25693	2005	174.3	150.0	204.3
Somalia	Shabelle Hoose	Kurtunwaarey	25693	2010	159.0	132.2	189.0
Somalia	Shabelle Hoose	Kurtunwaarey	25693	2015	146.0	118.6	179.9
Somalia	Shabelle Hoose	Marka	25694	2000	218.5	190.6	249.0
Somalia	Shabelle Hoose	Marka	25694	2005	179.9	156.9	205.3
Somalia	Shabelle Hoose	Marka	25694	2010	158.9	134.7	184.7
Somalia	Shabelle Hoose	Marka	25694	2015	145.9	122.3	176.0
Somalia	Shabelle Hoose	Qoryooley	25695	2000	224.8	196.7	255.5
Somalia	Shabelle Hoose	Qoryooley	25695	2005	186.7	164.6	212.6
Somalia	Shabelle Hoose	Qoryooley	25695	2010	161.8	138.2	187.4
Somalia	Shabelle Hoose	Qoryooley	25695	2015	155.7	130.2	186.4
Somalia	Shabelle Hoose	Sablaale	25696	2000	183.5	153.3	217.1
Somalia	Shabelle Hoose	Sablaale	25696	2005	156.2	130.6	184.6
Somalia	Shabelle Hoose	Sablaale	25696	2010	148.2	122.2	179.0
Somalia	Shabelle Hoose	Sablaale	25696	2015	134.3	108.6	166.0
Somalia	Shabelle Hoose	Wanla Weyn	25697	2000	191.7	166.4	220.0
Somalia	Shabelle Hoose	Wanla Weyn	25697	2005	169.4	147.8	193.3
Somalia	Shabelle Hoose	Wanla Weyn	25697	2010	151.1	128.0	175.5
Somalia	Shabelle Hoose	Wanla Weyn	25697	2015	136.1	113.9	162.0
Somalia	Sool	Caynabo	25716	2000	146.1	126.2	169.0
Somalia	Sool	Caynabo	25716	2005	134.7	116.5	154.1
Somalia	Sool	Caynabo	25716	2010	136.0	117.1	157.3
Somalia	Sool	Caynabo	25716	2015	92.3	76.9	109.0
Somalia	Sool	Laas Caanood	25717	2000	138.7	117.1	161.9
Somalia	Sool	Laas Caanood	25717	2005	124.3	105.8	142.5
Somalia	Sool	Laas Caanood	25717	2010	126.6	106.7	149.7
Somalia	Sool	Laas Caanood	25717	2015	89.7	74.2	108.2
Somalia	Sool	Taleex	25718	2000	127.3	104.8	153.5
Somalia	Sool	Taleex	25718	2005	117.1	96.0	139.8
Somalia	Sool	Taleex	25718	2010	118.3	96.2	143.3
Somalia	Sool	Taleex	25718	2015	89.0	71.9	108.7
Somalia	Sool	Xudun	25719	2000	132.2	110.7	155.6
Somalia	Sool	Xudun	25719	2005	121.0	102.6	143.0
Somalia	Sool	Xudun	25719	2010	122.8	103.5	146.5
Somalia	Sool	Xudun	25719	2015	84.3	69.3	102.6
Somalia	Togdheer	Burco	25720	2000	148.4	133.1	165.4
Somalia	Togdheer	Burco	25720	2005	140.9	127.6	157.1
Somalia	Togdheer	Burco	25720	2010	148.0	130.9	166.8
Somalia	Togdheer	Burco	25720	2015	99.7	84.7	115.6
Somalia	Togdheer	Buuhoodle	25721	2000	132.6	113.3	155.4

Admin 0	Admin 1	Admin 2	GAUL Code	Year	Under-5 mortality (per 1,000 livebirths)		
					Estimate	Lower bound, 95% UI	Upper bound, 95% UI
Somalia	Togdheer	Buuhoodle	25721	2005	125.8	107.3	145.4
Somalia	Togdheer	Buuhoodle	25721	2010	132.5	111.5	154.8
Somalia	Togdheer	Buuhoodle	25721	2015	91.6	75.8	109.9
Somalia	Togdheer	Owdweyne	25722	2000	143.9	127.7	159.5
Somalia	Togdheer	Owdweyne	25722	2005	134.5	121.6	147.4
Somalia	Togdheer	Owdweyne	25722	2010	137.4	121.1	153.3
Somalia	Togdheer	Owdweyne	25722	2015	94.8	81.3	109.4
Somalia	Togdheer	Sheikh	25723	2000	146.4	127.6	170.8
Somalia	Togdheer	Sheikh	25723	2005	134.3	117.0	154.8
Somalia	Togdheer	Sheikh	25723	2010	143.1	124.7	165.3
Somalia	Togdheer	Sheikh	25723	2015	97.4	82.1	115.7
Somalia	Woqooyi Galbeed	Berbera	25724	2000	125.6	106.6	147.7
Somalia	Woqooyi Galbeed	Berbera	25724	2005	117.7	100.4	136.0
Somalia	Woqooyi Galbeed	Berbera	25724	2010	133.6	113.7	156.6
Somalia	Woqooyi Galbeed	Berbera	25724	2015	91.0	74.6	110.8
Somalia	Woqooyi Galbeed	Gebiley	25725	2000	112.2	101.7	123.9
Somalia	Woqooyi Galbeed	Gebiley	25725	2005	99.2	90.2	108.6
Somalia	Woqooyi Galbeed	Gebiley	25725	2010	102.4	90.9	114.6
Somalia	Woqooyi Galbeed	Gebiley	25725	2015	75.5	65.1	87.8
Somalia	Woqooyi Galbeed	Hargeysa	25726	2000	111.5	101.6	121.1
Somalia	Woqooyi Galbeed	Hargeysa	25726	2005	99.8	92.1	108.3
Somalia	Woqooyi Galbeed	Hargeysa	25726	2010	102.9	92.7	113.2
Somalia	Woqooyi Galbeed	Hargeysa	25726	2015	75.2	65.0	86.3
South Africa	Eastern Cape	Alfred Nzo District Municipality	77353	2000	114.0	103.7	125.4
South Africa	Eastern Cape	Alfred Nzo District Municipality	77353	2005	114.3	103.0	126.2
South Africa	Eastern Cape	Alfred Nzo District Municipality	77353	2010	91.6	81.2	103.2
South Africa	Eastern Cape	Alfred Nzo District Municipality	77353	2015	68.7	60.0	80.0
South Africa	Eastern Cape	Amathole District Municipality	77320	2000	79.2	71.2	88.4
South Africa	Eastern Cape	Amathole District Municipality	77320	2005	80.4	70.6	90.9
South Africa	Eastern Cape	Amathole District Municipality	77320	2010	58.4	50.0	67.8
South Africa	Eastern Cape	Amathole District Municipality	77320	2015	51.1	43.4	59.6
South Africa	Eastern Cape	Buffalo City Metropolitan Municipality	77316	2000	58.8	50.2	68.5
South Africa	Eastern Cape	Buffalo City Metropolitan Municipality	77316	2005	63.8	54.1	74.5
South Africa	Eastern Cape	Buffalo City Metropolitan Municipality	77316	2010	45.8	37.7	56.1
South Africa	Eastern Cape	Buffalo City Metropolitan Municipality	77316	2015	42.1	34.6	51.5
South Africa	Eastern Cape	Cacadu District Municipality	77319	2000	61.9	54.5	69.9
South Africa	Eastern Cape	Cacadu District Municipality	77319	2005	63.1	55.4	71.5
South Africa	Eastern Cape	Cacadu District Municipality	77319	2010	50.4	43.5	58.2
South Africa	Eastern Cape	Cacadu District Municipality	77319	2015	40.3	34.3	46.9
South Africa	Eastern Cape	Chris Hani District Municipality	77321	2000	81.6	72.7	91.0
South Africa	Eastern Cape	Chris Hani District Municipality	77321	2005	82.2	72.5	91.9
South Africa	Eastern Cape	Chris Hani District Municipality	77321	2010	68.9	59.5	78.9
South Africa	Eastern Cape	Chris Hani District Municipality	77321	2015	51.2	43.4	59.6
South Africa	Eastern Cape	Joe Gqabi District Municipality	77322	2000	105.4	95.7	116.4
South Africa	Eastern Cape	Joe Gqabi District Municipality	77322	2005	110.9	100.1	121.9
South Africa	Eastern Cape	Joe Gqabi District Municipality	77322	2010	91.4	80.8	102.7
South Africa	Eastern Cape	Joe Gqabi District Municipality	77322	2015	66.6	58.0	75.9
South Africa	Eastern Cape	Nelson Mandela Bay Metropolitan Municip	77366	2000	53.7	44.3	64.8
South Africa	Eastern Cape	Nelson Mandela Bay Metropolitan Municip	77366	2005	58.0	47.5	69.3
South Africa	Eastern Cape	Nelson Mandela Bay Metropolitan Municip	77366	2010	40.4	32.6	50.4
South Africa	Eastern Cape	Nelson Mandela Bay Metropolitan Municip	77366	2015	38.6	31.1	48.0
South Africa	Eastern Cape	O.R.Tambo District Municipality	77323	2000	111.8	100.0	124.7
South Africa	Eastern Cape	O.R.Tambo District Municipality	77323	2005	107.5	95.1	120.8
South Africa	Eastern Cape	O.R.Tambo District Municipality	77323	2010	80.4	69.7	92.2
South Africa	Eastern Cape	O.R.Tambo District Municipality	77323	2015	63.1	53.0	73.8
South Africa	Free State	Fezile Dabi District Municipality	77328	2000	77.1	69.1	85.6
South Africa	Free State	Fezile Dabi District Municipality	77328	2005	79.7	70.8	89.4
South Africa	Free State	Fezile Dabi District Municipality	77328	2010	68.5	59.6	77.8
South Africa	Free State	Fezile Dabi District Municipality	77328	2015	49.5	42.2	58.0
South Africa	Free State	Lejweleputswa District Municipality	77325	2000	83.1	73.1	94.1
South Africa	Free State	Lejweleputswa District Municipality	77325	2005	85.7	75.1	98.3
South Africa	Free State	Lejweleputswa District Municipality	77325	2010	73.5	63.5	85.9
South Africa	Free State	Lejweleputswa District Municipality	77325	2015	55.0	46.7	65.1
South Africa	Free State	Mangaung Metropolitan Municipality	77365	2000	81.2	70.2	94.3
South Africa	Free State	Mangaung Metropolitan Municipality	77365	2005	88.3	76.4	101.8
South Africa	Free State	Mangaung Metropolitan Municipality	77365	2010	76.1	64.6	89.1
South Africa	Free State	Mangaung Metropolitan Municipality	77365	2015	56.6	47.3	67.4
South Africa	Free State	Thabo Mofutsanyane District Municipality	77326	2000	97.9	89.6	106.4
South Africa	Free State	Thabo Mofutsanyane District Municipality	77326	2005	103.0	94.5	112.3
South Africa	Free State	Thabo Mofutsanyane District Municipality	77326	2010	84.3	76.1	92.9
South Africa	Free State	Thabo Mofutsanyane District Municipality	77326	2015	62.7	55.1	70.7
South Africa	Free State	Xhariep District Municipality	77324	2000	90.4	79.9	102.2
South Africa	Free State	Xhariep District Municipality	77324	2005	93.7	83.2	105.2
South Africa	Free State	Xhariep District Municipality	77324	2010	78.5	68.6	90.0
South Africa	Free State	Xhariep District Municipality	77324	2015	59.0	50.7	67.7
South Africa	Gauteng	City of Johannesburg Metropolitan Munici	77364	2000	54.8	48.2	61.5
South Africa	Gauteng	City of Johannesburg Metropolitan Munici	77364	2005	60.2	53.0	68.4
South Africa	Gauteng	City of Johannesburg Metropolitan Munici	77364	2010	53.4	45.4	62.5
South Africa	Gauteng	City of Johannesburg Metropolitan Munici	77364	2015	39.3	33.2	46.7
South Africa	Gauteng	City of Tshwane Metropolitan Municipality	77367	2000	56.8	50.1	63.8
South Africa	Gauteng	City of Tshwane Metropolitan Municipality	77367	2005	62.1	54.6	70.3
South Africa	Gauteng	City of Tshwane Metropolitan Municipality	77367	2010	54.8	46.8	63.7
South Africa	Gauteng	City of Tshwane Metropolitan Municipality	77367	2015	39.3	33.3	46.3
South Africa	Gauteng	Ekurhuleni Metropolitan Municipality	77362	2000	58.7	52.1	65.8
South Africa	Gauteng	Ekurhuleni Metropolitan Municipality	77362	2005	63.1	55.6	70.9

Admin 0	Admin 1	Admin 2	GAUL Code	Year	Under-5 mortality (per 1,000 livebirths)		
					Estimate	Lower bound, 95% UI	Upper bound, 95% UI
South Africa	Gauteng	Ekurhuleni Metropolitan Municipality	77362	2010	55.3	47.1	64.0
South Africa	Gauteng	Ekurhuleni Metropolitan Municipality	77362	2015	39.9	33.6	47.4
South Africa	Gauteng	Sedibeng District Municipality	77351	2000	63.0	56.2	70.5
South Africa	Gauteng	Sedibeng District Municipality	77351	2005	67.0	59.0	75.5
South Africa	Gauteng	Sedibeng District Municipality	77351	2010	58.5	50.3	67.7
South Africa	Gauteng	Sedibeng District Municipality	77351	2015	42.4	35.4	50.3
South Africa	Gauteng	West Rand District Municipality	77356	2000	60.2	53.8	67.2
South Africa	Gauteng	West Rand District Municipality	77356	2005	63.6	56.0	71.9
South Africa	Gauteng	West Rand District Municipality	77356	2010	56.0	47.8	64.6
South Africa	Gauteng	West Rand District Municipality	77356	2015	40.7	33.9	47.9
South Africa	KwaZulu-Natal	Amajuba District Municipality	77333	2000	96.3	84.1	110.0
South Africa	KwaZulu-Natal	Amajuba District Municipality	77333	2005	97.3	84.7	112.3
South Africa	KwaZulu-Natal	Amajuba District Municipality	77333	2010	79.5	68.3	91.5
South Africa	KwaZulu-Natal	Amajuba District Municipality	77333	2015	59.3	49.7	69.5
South Africa	KwaZulu-Natal	Sisonke District Municipality	77352	2000	101.9	91.3	113.2
South Africa	KwaZulu-Natal	Sisonke District Municipality	77352	2005	103.3	92.0	115.9
South Africa	KwaZulu-Natal	Sisonke District Municipality	77352	2010	84.7	74.7	96.4
South Africa	KwaZulu-Natal	Sisonke District Municipality	77352	2015	63.1	54.6	73.4
South Africa	KwaZulu-Natal	Ugu District Municipality	77329	2000	96.1	84.9	106.9
South Africa	KwaZulu-Natal	Ugu District Municipality	77329	2005	94.4	82.8	106.5
South Africa	KwaZulu-Natal	Ugu District Municipality	77329	2010	64.5	55.2	74.3
South Africa	KwaZulu-Natal	Ugu District Municipality	77329	2015	58.5	49.1	68.6
South Africa	KwaZulu-Natal	Umgungundlovu District Municipality	77330	2000	85.0	76.4	93.8
South Africa	KwaZulu-Natal	Umgungundlovu District Municipality	77330	2005	88.9	79.7	100.1
South Africa	KwaZulu-Natal	Umgungundlovu District Municipality	77330	2010	66.0	58.1	75.8
South Africa	KwaZulu-Natal	Umgungundlovu District Municipality	77330	2015	55.5	47.7	65.1
South Africa	KwaZulu-Natal	Umkhanyakude District Municipality	77335	2000	111.9	100.4	125.3
South Africa	KwaZulu-Natal	Umkhanyakude District Municipality	77335	2005	99.7	89.0	111.0
South Africa	KwaZulu-Natal	Umkhanyakude District Municipality	77335	2010	82.1	71.9	93.3
South Africa	KwaZulu-Natal	Umkhanyakude District Municipality	77335	2015	57.0	49.4	65.5
South Africa	KwaZulu-Natal	Umzinyathi District Municipality	77332	2000	96.9	87.0	108.6
South Africa	KwaZulu-Natal	Umzinyathi District Municipality	77332	2005	95.4	84.7	107.5
South Africa	KwaZulu-Natal	Umzinyathi District Municipality	77332	2010	72.8	63.6	83.0
South Africa	KwaZulu-Natal	Umzinyathi District Municipality	77332	2015	57.4	49.2	66.7
South Africa	KwaZulu-Natal	Uthukela District Municipality	77331	2000	99.8	88.8	111.1
South Africa	KwaZulu-Natal	Uthukela District Municipality	77331	2005	100.8	89.9	113.8
South Africa	KwaZulu-Natal	Uthukela District Municipality	77331	2010	72.7	63.4	82.1
South Africa	KwaZulu-Natal	Uthukela District Municipality	77331	2015	60.0	51.2	69.6
South Africa	KwaZulu-Natal	Uthungulu District Municipality	77336	2000	93.1	82.3	104.8
South Africa	KwaZulu-Natal	Uthungulu District Municipality	77336	2005	87.1	76.5	98.7
South Africa	KwaZulu-Natal	Uthungulu District Municipality	77336	2010	72.5	62.3	83.5
South Africa	KwaZulu-Natal	Uthungulu District Municipality	77336	2015	53.2	45.0	62.4
South Africa	KwaZulu-Natal	Zululand District Municipality	77334	2000	109.6	99.2	120.8
South Africa	KwaZulu-Natal	Zululand District Municipality	77334	2005	101.4	91.9	112.0
South Africa	KwaZulu-Natal	Zululand District Municipality	77334	2010	84.9	75.6	95.4
South Africa	KwaZulu-Natal	Zululand District Municipality	77334	2015	58.9	51.5	67.3
South Africa	KwaZulu-Natal	eThekwini Metropolitan Municipality	77363	2000	73.0	64.6	82.0
South Africa	KwaZulu-Natal	eThekwini Metropolitan Municipality	77363	2005	76.6	67.3	86.6
South Africa	KwaZulu-Natal	eThekwini Metropolitan Municipality	77363	2010	58.5	50.6	67.8
South Africa	KwaZulu-Natal	eThekwini Metropolitan Municipality	77363	2015	49.8	41.8	59.5
South Africa	KwaZulu-Natal	iLembe District Municipality	77337	2000	86.5	77.1	97.3
South Africa	KwaZulu-Natal	iLembe District Municipality	77337	2005	84.0	73.6	95.6
South Africa	KwaZulu-Natal	iLembe District Municipality	77337	2010	69.6	60.0	80.5
South Africa	KwaZulu-Natal	iLembe District Municipality	77337	2015	53.4	45.0	63.0
South Africa	Limpopo	Capricorn District Municipality	77344	2000	66.7	58.3	75.7
South Africa	Limpopo	Capricorn District Municipality	77344	2005	67.4	59.1	77.8
South Africa	Limpopo	Capricorn District Municipality	77344	2010	58.8	50.5	69.2
South Africa	Limpopo	Capricorn District Municipality	77344	2015	43.2	36.0	51.6
South Africa	Limpopo	Mopani District Municipality	77342	2000	73.0	64.4	82.0
South Africa	Limpopo	Mopani District Municipality	77342	2005	72.3	63.4	81.4
South Africa	Limpopo	Mopani District Municipality	77342	2010	59.3	51.5	68.4
South Africa	Limpopo	Mopani District Municipality	77342	2015	44.4	37.5	52.3
South Africa	Limpopo	Sekhukhune District Municipality	77355	2000	70.2	62.0	79.5
South Africa	Limpopo	Sekhukhune District Municipality	77355	2005	68.0	59.3	77.1
South Africa	Limpopo	Sekhukhune District Municipality	77355	2010	57.1	49.3	65.3
South Africa	Limpopo	Sekhukhune District Municipality	77355	2015	41.5	34.8	49.1
South Africa	Limpopo	Vhembe District Municipality	77343	2000	67.3	59.7	75.9
South Africa	Limpopo	Vhembe District Municipality	77343	2005	69.0	61.4	77.9
South Africa	Limpopo	Vhembe District Municipality	77343	2010	60.2	52.3	68.8
South Africa	Limpopo	Vhembe District Municipality	77343	2015	45.3	38.6	53.5
South Africa	Limpopo	Waterberg District Municipality	77345	2000	70.0	61.9	79.2
South Africa	Limpopo	Waterberg District Municipality	77345	2005	71.2	63.0	81.1
South Africa	Limpopo	Waterberg District Municipality	77345	2010	60.3	52.4	69.7
South Africa	Limpopo	Waterberg District Municipality	77345	2015	43.9	37.4	51.6
South Africa	Mpumalanga	Ehlanzeni District Municipality	77341	2000	87.1	79.5	96.7
South Africa	Mpumalanga	Ehlanzeni District Municipality	77341	2005	86.1	78.4	94.9
South Africa	Mpumalanga	Ehlanzeni District Municipality	77341	2010	68.7	61.3	76.9
South Africa	Mpumalanga	Ehlanzeni District Municipality	77341	2015	49.0	42.7	56.3
South Africa	Mpumalanga	Gert Sibande District Municipality	77339	2000	95.1	87.2	104.5
South Africa	Mpumalanga	Gert Sibande District Municipality	77339	2005	93.6	85.0	103.4
South Africa	Mpumalanga	Gert Sibande District Municipality	77339	2010	74.5	67.0	83.2
South Africa	Mpumalanga	Gert Sibande District Municipality	77339	2015	54.4	47.9	61.7
South Africa	Mpumalanga	Nkangala District Municipality	77340	2000	70.7	62.8	78.4
South Africa	Mpumalanga	Nkangala District Municipality	77340	2005	70.9	63.2	79.5
South Africa	Mpumalanga	Nkangala District Municipality	77340	2010	59.7	52.1	68.4

Admin 0	Admin 1	Admin 2	GAUL Code	Year	Under-5 mortality (per 1,000 livebirths)		
					Estimate	Lower bound, 95% UI	Upper bound, 95% UI
South Africa	Mpumalanga	Nkangala District Municipality	77340	2015	42.8	36.3	50.2
South Africa	North West	Bojanala District Municipality	77346	2000	62.9	55.5	70.4
South Africa	North West	Bojanala District Municipality	77346	2005	64.8	56.5	72.7
South Africa	North West	Bojanala District Municipality	77346	2010	55.9	47.8	64.2
South Africa	North West	Bojanala District Municipality	77346	2015	40.9	34.2	47.8
South Africa	North West	Dr Kenneth Kaunda District Municipality	77350	2000	73.8	64.8	82.9
South Africa	North West	Dr Kenneth Kaunda District Municipality	77350	2005	76.4	66.0	86.9
South Africa	North West	Dr Kenneth Kaunda District Municipality	77350	2010	65.7	56.0	76.8
South Africa	North West	Dr Kenneth Kaunda District Municipality	77350	2015	48.5	40.6	57.4
South Africa	North West	Dr Ruth Segomotsi Mompati District Munic	77348	2000	90.6	80.0	102.0
South Africa	North West	Dr Ruth Segomotsi Mompati District Munic	77348	2005	86.0	75.8	97.1
South Africa	North West	Dr Ruth Segomotsi Mompati District Munic	77348	2010	70.5	60.9	81.8
South Africa	North West	Dr Ruth Segomotsi Mompati District Munic	77348	2015	54.7	46.3	64.1
South Africa	North West	Ngaka Modiri Molema District Municipality	77347	2000	78.2	69.5	88.0
South Africa	North West	Ngaka Modiri Molema District Municipality	77347	2005	75.7	67.1	85.9
South Africa	North West	Ngaka Modiri Molema District Municipality	77347	2010	64.1	55.7	73.3
South Africa	North West	Ngaka Modiri Molema District Municipality	77347	2015	49.1	41.1	57.3
South Africa	Northern Cape	Frances Baard District Municipality	77361	2000	78.1	67.3	90.8
South Africa	Northern Cape	Frances Baard District Municipality	77361	2005	78.0	66.4	91.6
South Africa	Northern Cape	Frances Baard District Municipality	77361	2010	65.8	55.2	78.8
South Africa	Northern Cape	Frances Baard District Municipality	77361	2015	50.7	42.0	61.5
South Africa	Northern Cape	John Taolo Gaetsewe District Municipality	77354	2000	84.1	70.9	99.8
South Africa	Northern Cape	John Taolo Gaetsewe District Municipality	77354	2005	81.8	68.6	98.0
South Africa	Northern Cape	John Taolo Gaetsewe District Municipality	77354	2010	67.0	55.3	80.5
South Africa	Northern Cape	John Taolo Gaetsewe District Municipality	77354	2015	52.6	42.9	63.0
South Africa	Northern Cape	Namakwa District Municipality	77358	2000	67.7	57.1	78.9
South Africa	Northern Cape	Namakwa District Municipality	77358	2005	70.1	59.3	82.0
South Africa	Northern Cape	Namakwa District Municipality	77358	2010	58.9	49.9	69.5
South Africa	Northern Cape	Namakwa District Municipality	77358	2015	42.1	35.3	50.4
South Africa	Northern Cape	Pixley ka Seme District Municipality	77359	2000	78.7	67.3	92.3
South Africa	Northern Cape	Pixley ka Seme District Municipality	77359	2005	77.5	66.5	90.0
South Africa	Northern Cape	Pixley ka Seme District Municipality	77359	2010	64.4	54.8	76.1
South Africa	Northern Cape	Pixley ka Seme District Municipality	77359	2015	47.9	40.3	57.0
South Africa	Northern Cape	Siyanda District Municipality	77360	2000	76.3	64.1	89.7
South Africa	Northern Cape	Siyanda District Municipality	77360	2005	76.0	63.8	90.0
South Africa	Northern Cape	Siyanda District Municipality	77360	2010	62.7	51.7	73.7
South Africa	Northern Cape	Siyanda District Municipality	77360	2015	46.1	38.0	55.6
South Africa	Western Cape	Cape Winelands District Municipality	77327	2000	52.4	45.7	59.8
South Africa	Western Cape	Cape Winelands District Municipality	77327	2005	52.8	45.5	60.7
South Africa	Western Cape	Cape Winelands District Municipality	77327	2010	45.4	38.4	53.8
South Africa	Western Cape	Cape Winelands District Municipality	77327	2015	32.3	26.7	38.7
South Africa	Western Cape	Central Karoo District Municipality	77357	2000	66.0	55.3	79.8
South Africa	Western Cape	Central Karoo District Municipality	77357	2005	65.6	54.5	78.5
South Africa	Western Cape	Central Karoo District Municipality	77357	2010	55.6	45.8	67.0
South Africa	Western Cape	Central Karoo District Municipality	77357	2015	39.8	31.9	48.8
South Africa	Western Cape	City of Cape Town Metropolitan Municipal	77317	2000	47.3	40.2	55.2
South Africa	Western Cape	City of Cape Town Metropolitan Municipal	77317	2005	50.1	42.5	59.4
South Africa	Western Cape	City of Cape Town Metropolitan Municipal	77317	2010	43.9	36.2	53.3
South Africa	Western Cape	City of Cape Town Metropolitan Municipal	77317	2015	31.6	25.2	39.0
South Africa	Western Cape	Eden District Municipality	77349	2000	61.1	52.1	72.4
South Africa	Western Cape	Eden District Municipality	77349	2005	61.5	52.3	72.2
South Africa	Western Cape	Eden District Municipality	77349	2010	53.2	44.8	63.9
South Africa	Western Cape	Eden District Municipality	77349	2015	38.2	31.0	46.6
South Africa	Western Cape	Overberg District Municipality	77338	2000	54.5	46.9	63.2
South Africa	Western Cape	Overberg District Municipality	77338	2005	54.7	46.7	64.0
South Africa	Western Cape	Overberg District Municipality	77338	2010	47.5	39.7	56.3
South Africa	Western Cape	Overberg District Municipality	77338	2015	33.8	27.5	40.6
South Africa	Western Cape	West Coast District Municipality	77318	2000	55.7	47.8	64.2
South Africa	Western Cape	West Coast District Municipality	77318	2005	56.6	48.5	65.3
South Africa	Western Cape	West Coast District Municipality	77318	2010	49.0	41.3	57.9
South Africa	Western Cape	West Coast District Municipality	77318	2015	34.8	28.9	41.6
South Sudan	Central Equatoria	Bahr Al Jabal	37033	2000	157.7	144.2	171.4
South Sudan	Central Equatoria	Bahr Al Jabal	37033	2005	134.0	121.2	146.9
South Sudan	Central Equatoria	Bahr Al Jabal	37033	2010	111.1	99.4	123.9
South Sudan	Central Equatoria	Bahr Al Jabal	37033	2015	100.1	87.2	114.1
South Sudan	Central Equatoria	Kajo Kaji	37034	2000	142.0	130.2	154.8
South Sudan	Central Equatoria	Kajo Kaji	37034	2005	123.1	112.3	134.8
South Sudan	Central Equatoria	Kajo Kaji	37034	2010	101.5	91.5	111.9
South Sudan	Central Equatoria	Kajo Kaji	37034	2015	92.3	80.5	105.5
South Sudan	Central Equatoria	Nahr Yei	37035	2000	152.1	139.4	165.1
South Sudan	Central Equatoria	Nahr Yei	37035	2005	130.2	120.2	141.8
South Sudan	Central Equatoria	Nahr Yei	37035	2010	105.2	95.2	115.4
South Sudan	Central Equatoria	Nahr Yei	37035	2015	97.4	85.3	110.3
South Sudan	Central Equatoria	Terkaka	37036	2000	147.0	133.1	162.5
South Sudan	Central Equatoria	Terkaka	37036	2005	128.9	116.3	142.1
South Sudan	Central Equatoria	Terkaka	37036	2010	106.5	94.8	118.5
South Sudan	Central Equatoria	Terkaka	37036	2015	95.5	82.7	109.5
South Sudan	Eastern Equatoria	Amatonge	37042	2000	138.9	126.6	151.3
South Sudan	Eastern Equatoria	Amatonge	37042	2005	121.5	111.2	132.8
South Sudan	Eastern Equatoria	Amatonge	37042	2010	108.0	97.3	119.7
South Sudan	Eastern Equatoria	Amatonge	37042	2015	92.4	81.8	105.0
South Sudan	Eastern Equatoria	Kapoeta	37043	2000	117.5	106.6	130.7
South Sudan	Eastern Equatoria	Kapoeta	37043	2005	101.7	91.7	112.1
South Sudan	Eastern Equatoria	Kapoeta	37043	2010	92.9	82.3	104.2
South Sudan	Eastern Equatoria	Kapoeta	37043	2015	81.7	71.4	93.3

Admin 0	Admin 1	Admin 2	GAUL Code	Year	Under-5 mortality (per 1,000 livebirths)		
					Estimate	Lower bound, 95% UI	Upper bound, 95% UI
South Sudan	Eastern Equatoria	Magwi	37044	2000	138.9	127.2	152.1
South Sudan	Eastern Equatoria	Magwi	37044	2005	120.6	109.1	132.0
South Sudan	Eastern Equatoria	Magwi	37044	2010	104.0	94.1	114.8
South Sudan	Eastern Equatoria	Magwi	37044	2015	94.5	83.0	108.4
South Sudan	Eastern Equatoria	Shokodom	37045	2000	123.0	110.5	135.9
South Sudan	Eastern Equatoria	Shokodom	37045	2005	107.8	96.0	119.7
South Sudan	Eastern Equatoria	Shokodom	37045	2010	101.5	90.4	113.6
South Sudan	Eastern Equatoria	Shokodom	37045	2015	85.8	74.5	98.1
South Sudan	El Buheyrat	Aliab	37022	2000	140.9	126.4	158.4
South Sudan	El Buheyrat	Aliab	37022	2005	120.9	107.2	135.0
South Sudan	El Buheyrat	Aliab	37022	2010	105.5	92.2	118.9
South Sudan	El Buheyrat	Aliab	37022	2015	93.0	78.6	107.8
South Sudan	El Buheyrat	Rumbek	37023	2000	122.9	112.6	134.4
South Sudan	El Buheyrat	Rumbek	37023	2005	100.7	92.3	109.7
South Sudan	El Buheyrat	Rumbek	37023	2010	93.6	83.6	103.9
South Sudan	El Buheyrat	Rumbek	37023	2015	89.9	77.6	103.0
South Sudan	El Buheyrat	Shobet	37024	2000	132.9	121.0	146.1
South Sudan	El Buheyrat	Shobet	37024	2005	108.7	98.6	120.2
South Sudan	El Buheyrat	Shobet	37024	2010	100.8	89.8	112.9
South Sudan	El Buheyrat	Shobet	37024	2015	97.6	84.4	113.5
South Sudan	El Buheyrat	Yerol	37025	2000	125.2	113.0	139.2
South Sudan	El Buheyrat	Yerol	37025	2005	105.9	95.1	117.0
South Sudan	El Buheyrat	Yerol	37025	2010	95.5	84.1	107.2
South Sudan	El Buheyrat	Yerol	37025	2015	89.4	77.4	103.5
South Sudan	Jonglei	Akobo	37051	2000	121.9	108.7	135.8
South Sudan	Jonglei	Akobo	37051	2005	101.1	90.2	112.9
South Sudan	Jonglei	Akobo	37051	2010	89.0	78.5	101.0
South Sudan	Jonglei	Akobo	37051	2015	78.4	67.3	91.1
South Sudan	Jonglei	Ayod	37052	2000	116.3	103.6	129.4
South Sudan	Jonglei	Ayod	37052	2005	102.6	91.2	114.9
South Sudan	Jonglei	Ayod	37052	2010	92.0	80.8	104.9
South Sudan	Jonglei	Ayod	37052	2015	87.9	75.5	102.6
South Sudan	Jonglei	Bor	37053	2000	139.8	123.4	158.6
South Sudan	Jonglei	Bor	37053	2005	118.2	103.3	134.2
South Sudan	Jonglei	Bor	37053	2010	102.0	88.4	116.0
South Sudan	Jonglei	Bor	37053	2015	90.2	76.8	105.3
South Sudan	Jonglei	Fam Al Zaraf	37054	2000	123.8	109.2	140.3
South Sudan	Jonglei	Fam Al Zaraf	37054	2005	110.2	96.6	124.8
South Sudan	Jonglei	Fam Al Zaraf	37054	2010	95.2	82.7	108.9
South Sudan	Jonglei	Fam Al Zaraf	37054	2015	92.3	77.6	109.3
South Sudan	Jonglei	Nahr Atiem	37055	2000	124.4	109.5	140.2
South Sudan	Jonglei	Nahr Atiem	37055	2005	105.6	93.3	119.0
South Sudan	Jonglei	Nahr Atiem	37055	2010	95.1	82.6	108.4
South Sudan	Jonglei	Nahr Atiem	37055	2015	85.3	73.0	99.1
South Sudan	Jonglei	Pibor	37056	2000	141.2	126.4	156.6
South Sudan	Jonglei	Pibor	37056	2005	116.4	103.5	129.2
South Sudan	Jonglei	Pibor	37056	2010	97.1	85.6	109.9
South Sudan	Jonglei	Pibor	37056	2015	84.1	72.8	96.9
South Sudan	Jonglei	Wat	37057	2000	116.5	102.5	131.7
South Sudan	Jonglei	Wat	37057	2005	99.1	87.5	112.2
South Sudan	Jonglei	Wat	37057	2010	88.2	77.8	101.8
South Sudan	Jonglei	Wat	37057	2015	79.5	68.1	92.9
South Sudan	Northern Bahr El Ghazal	Aryat	37076	2000	174.8	158.0	191.8
South Sudan	Northern Bahr El Ghazal	Aryat	37076	2005	154.7	139.7	170.4
South Sudan	Northern Bahr El Ghazal	Aryat	37076	2010	141.1	125.7	158.1
South Sudan	Northern Bahr El Ghazal	Aryat	37076	2015	119.4	102.5	137.4
South Sudan	Northern Bahr El Ghazal	Aweil	37077	2000	183.7	170.7	197.9
South Sudan	Northern Bahr El Ghazal	Aweil	37077	2005	157.1	145.1	170.1
South Sudan	Northern Bahr El Ghazal	Aweil	37077	2010	143.9	130.4	157.4
South Sudan	Northern Bahr El Ghazal	Aweil	37077	2015	120.8	106.2	135.6
South Sudan	Northern Bahr El Ghazal	Malek	37078	2000	193.2	176.4	211.1
South Sudan	Northern Bahr El Ghazal	Malek	37078	2005	160.8	145.8	175.4
South Sudan	Northern Bahr El Ghazal	Malek	37078	2010	145.7	131.3	161.0
South Sudan	Northern Bahr El Ghazal	Malek	37078	2015	124.8	109.0	143.1
South Sudan	Northern Bahr El Ghazal	Wanjuk	37079	2000	185.0	171.0	199.7
South Sudan	Northern Bahr El Ghazal	Wanjuk	37079	2005	158.9	146.1	172.4
South Sudan	Northern Bahr El Ghazal	Wanjuk	37079	2010	143.2	130.0	158.5
South Sudan	Northern Bahr El Ghazal	Wanjuk	37079	2015	121.6	106.6	138.9
South Sudan	Unity	Al Leiri	37115	2000	126.3	114.5	138.5
South Sudan	Unity	Al Leiri	37115	2005	108.1	97.7	119.7
South Sudan	Unity	Al Leiri	37115	2010	103.9	91.8	117.1
South Sudan	Unity	Al Leiri	37115	2015	97.3	83.6	112.5
South Sudan	Unity	Al Mayom	37116	2000	139.7	126.2	153.9
South Sudan	Unity	Al Mayom	37116	2005	118.7	105.9	131.6
South Sudan	Unity	Al Mayom	37116	2010	109.9	97.4	123.6
South Sudan	Unity	Al Mayom	37116	2015	106.7	91.9	122.7
South Sudan	Unity	Faring	37117	2000	132.6	117.3	149.9
South Sudan	Unity	Faring	37117	2005	118.7	105.2	133.5
South Sudan	Unity	Faring	37117	2010	106.8	93.2	122.8
South Sudan	Unity	Faring	37117	2015	96.0	81.2	112.6
South Sudan	Unity	Rabkona	37118	2000	130.8	118.4	144.2
South Sudan	Unity	Rabkona	37118	2005	113.3	102.3	124.2
South Sudan	Unity	Rabkona	37118	2010	106.1	94.4	118.1
South Sudan	Unity	Rabkona	37118	2015	100.1	85.7	115.5
South Sudan	Upper Nile	Al Mabien	37119	2000	165.5	147.9	185.8

Admin 0	Admin 1	Admin 2	GAUL Code	Year	Under-5 mortality (per 1,000 livebirths)		
					Estimate	Lower bound, 95% UI	Upper bound, 95% UI
South Sudan	Upper Nile	Al Mabien	37119	2005	139.2	124.0	155.9
South Sudan	Upper Nile	Al Mabien	37119	2010	115.8	101.7	131.6
South Sudan	Upper Nile	Al Mabien	37119	2015	94.2	80.6	109.3
South Sudan	Upper Nile	Al Renk	37120	2000	141.5	127.3	158.5
South Sudan	Upper Nile	Al Renk	37120	2005	126.2	113.5	142.1
South Sudan	Upper Nile	Al Renk	37120	2010	98.3	87.1	110.7
South Sudan	Upper Nile	Al Renk	37120	2015	84.1	72.6	96.5
South Sudan	Upper Nile	Baleit	37121	2000	137.8	122.6	153.3
South Sudan	Upper Nile	Baleit	37121	2005	116.9	104.5	130.6
South Sudan	Upper Nile	Baleit	37121	2010	99.1	87.4	111.7
South Sudan	Upper Nile	Baleit	37121	2015	88.4	76.0	102.4
South Sudan	Upper Nile	Fashooda	37122	2000	143.7	129.5	159.2
South Sudan	Upper Nile	Fashooda	37122	2005	128.5	115.7	143.0
South Sudan	Upper Nile	Fashooda	37122	2010	103.7	93.0	114.6
South Sudan	Upper Nile	Fashooda	37122	2015	90.1	78.9	102.6
South Sudan	Upper Nile	Malut	37123	2000	151.0	134.5	169.6
South Sudan	Upper Nile	Malut	37123	2005	132.0	116.6	148.0
South Sudan	Upper Nile	Malut	37123	2010	108.4	94.9	122.3
South Sudan	Upper Nile	Malut	37123	2015	92.8	79.4	107.7
South Sudan	Upper Nile	Mayot	37124	2000	133.3	119.5	146.6
South Sudan	Upper Nile	Mayot	37124	2005	106.7	96.0	117.7
South Sudan	Upper Nile	Mayot	37124	2010	90.9	80.9	101.5
South Sudan	Upper Nile	Mayot	37124	2015	78.8	68.4	90.4
South Sudan	Upper Nile	Sobat	37125	2000	123.4	110.5	137.2
South Sudan	Upper Nile	Sobat	37125	2005	101.6	91.8	112.3
South Sudan	Upper Nile	Sobat	37125	2010	90.1	80.2	100.6
South Sudan	Upper Nile	Sobat	37125	2015	79.6	68.9	92.1
South Sudan	Upper Nile	Tonga	37126	2000	143.7	127.4	162.4
South Sudan	Upper Nile	Tonga	37126	2005	121.2	106.8	136.9
South Sudan	Upper Nile	Tonga	37126	2010	99.9	86.6	114.8
South Sudan	Upper Nile	Tonga	37126	2015	94.1	79.9	110.7
South Sudan	Warab	Gogrial	37127	2000	174.9	161.4	188.4
South Sudan	Warab	Gogrial	37127	2005	147.9	135.9	160.4
South Sudan	Warab	Gogrial	37127	2010	128.8	116.2	141.7
South Sudan	Warab	Gogrial	37127	2015	118.5	104.0	134.9
South Sudan	Warab	Nahr Lol	37128	2000	162.0	148.0	176.4
South Sudan	Warab	Nahr Lol	37128	2005	139.4	127.6	152.1
South Sudan	Warab	Nahr Lol	37128	2010	123.3	111.0	137.6
South Sudan	Warab	Nahr Lol	37128	2015	114.6	100.3	130.3
South Sudan	Warab	Tonj	37129	2000	147.5	135.2	159.8
South Sudan	Warab	Tonj	37129	2005	123.6	112.2	134.9
South Sudan	Warab	Tonj	37129	2010	112.7	101.6	124.6
South Sudan	Warab	Tonj	37129	2015	105.6	92.8	121.0
South Sudan	Warab	Warab	37130	2000	158.3	146.5	171.5
South Sudan	Warab	Warab	37130	2005	134.3	123.2	146.5
South Sudan	Warab	Warab	37130	2010	120.7	108.5	134.2
South Sudan	Warab	Warab	37130	2015	113.5	99.1	128.5
South Sudan	Western Bahr El Ghazal	Nahr Jur	37131	2000	152.4	138.2	167.5
South Sudan	Western Bahr El Ghazal	Nahr Jur	37131	2005	125.7	113.9	138.9
South Sudan	Western Bahr El Ghazal	Nahr Jur	37131	2010	117.3	103.7	131.6
South Sudan	Western Bahr El Ghazal	Nahr Jur	37131	2015	104.7	90.9	120.2
South Sudan	Western Bahr El Ghazal	Raja	37132	2000	145.5	129.0	163.6
South Sudan	Western Bahr El Ghazal	Raja	37132	2005	131.6	116.9	147.0
South Sudan	Western Bahr El Ghazal	Raja	37132	2010	111.2	99.0	124.5
South Sudan	Western Bahr El Ghazal	Raja	37132	2015	101.8	88.8	116.6
South Sudan	Western Bahr El Ghazal	Wau	37133	2000	152.7	140.6	166.3
South Sudan	Western Bahr El Ghazal	Wau	37133	2005	131.2	119.2	142.8
South Sudan	Western Bahr El Ghazal	Wau	37133	2010	118.2	105.8	130.7
South Sudan	Western Bahr El Ghazal	Wau	37133	2015	103.8	91.4	117.3
South Sudan	Western Equatoria	Meridi	37141	2000	145.9	131.8	161.4
South Sudan	Western Equatoria	Meridi	37141	2005	130.8	118.3	144.8
South Sudan	Western Equatoria	Meridi	37141	2010	108.8	96.1	122.4
South Sudan	Western Equatoria	Meridi	37141	2015	103.2	89.3	118.8
South Sudan	Western Equatoria	Mundri	37142	2000	140.4	127.2	154.8
South Sudan	Western Equatoria	Mundri	37142	2005	126.1	114.6	138.6
South Sudan	Western Equatoria	Mundri	37142	2010	104.6	93.4	117.1
South Sudan	Western Equatoria	Mundri	37142	2015	96.7	84.4	111.0
South Sudan	Western Equatoria	Tombura	37143	2000	161.5	144.3	178.5
South Sudan	Western Equatoria	Tombura	37143	2005	144.7	129.3	161.5
South Sudan	Western Equatoria	Tombura	37143	2010	118.4	104.1	134.9
South Sudan	Western Equatoria	Tombura	37143	2015	110.5	95.2	127.0
South Sudan	Western Equatoria	Yambio	37144	2000	155.7	139.0	172.8
South Sudan	Western Equatoria	Yambio	37144	2005	140.0	125.6	155.1
South Sudan	Western Equatoria	Yambio	37144	2010	115.5	102.1	129.9
South Sudan	Western Equatoria	Yambio	37144	2015	108.9	94.5	124.9
Sudan	Abyei	Abyei	125	2000	128.4	114.3	143.3
Sudan	Abyei	Abyei	125	2005	106.2	94.5	118.0
Sudan	Abyei	Abyei	125	2010	110.0	96.4	124.4
Sudan	Abyei	Abyei	125	2015	101.5	87.4	116.5
Sudan	Al Jazeera	Al Hasaheisa	37026	2000	81.2	74.0	89.0
Sudan	Al Jazeera	Al Hasaheisa	37026	2005	61.4	56.1	67.3
Sudan	Al Jazeera	Al Hasaheisa	37026	2010	58.1	52.7	63.9
Sudan	Al Jazeera	Al Hasaheisa	37026	2015	54.4	48.2	61.6
Sudan	Al Jazeera	Al Kamlin	37027	2000	84.3	76.6	92.9
Sudan	Al Jazeera	Al Kamlin	37027	2005	63.5	58.3	69.4

Admin 0	Admin 1	Admin 2	GAUL Code	Year	Under-5 mortality (per 1,000 livebirths)		
					Estimate	Lower bound, 95% UI	Upper bound, 95% UI
Sudan	Al Jazeera	Al Kamlin	37027	2010	60.3	54.5	66.3
Sudan	Al Jazeera	Al Kamlin	37027	2015	56.2	49.3	63.1
Sudan	Al Jazeera	Al Managil	37028	2000	79.7	72.0	87.2
Sudan	Al Jazeera	Al Managil	37028	2005	61.9	55.8	68.0
Sudan	Al Jazeera	Al Managil	37028	2010	59.4	53.0	65.5
Sudan	Al Jazeera	Al Managil	37028	2015	53.6	47.1	61.3
Sudan	Al Jazeera	East Al Jazeera	37029	2000	85.8	77.3	94.2
Sudan	Al Jazeera	East Al Jazeera	37029	2005	64.7	58.6	71.2
Sudan	Al Jazeera	East Al Jazeera	37029	2010	60.7	54.8	67.7
Sudan	Al Jazeera	East Al Jazeera	37029	2015	55.4	48.6	63.0
Sudan	Al Jazeera	North Al Jazeera	37030	2000	88.5	80.1	97.9
Sudan	Al Jazeera	North Al Jazeera	37030	2005	66.5	60.4	73.2
Sudan	Al Jazeera	North Al Jazeera	37030	2010	62.7	56.5	69.3
Sudan	Al Jazeera	North Al Jazeera	37030	2015	57.3	50.5	64.6
Sudan	Al Jazeera	South Al Jazeera	37031	2000	84.0	76.4	92.1
Sudan	Al Jazeera	South Al Jazeera	37031	2005	64.6	58.4	71.1
Sudan	Al Jazeera	South Al Jazeera	37031	2010	61.6	55.5	68.4
Sudan	Al Jazeera	South Al Jazeera	37031	2015	53.5	47.2	61.5
Sudan	Al Jazeera	Um Al Gura	37032	2000	90.1	82.1	98.8
Sudan	Al Jazeera	Um Al Gura	37032	2005	68.9	62.9	75.3
Sudan	Al Jazeera	Um Al Gura	37032	2010	64.2	58.3	71.1
Sudan	Al Jazeera	Um Al Gura	37032	2015	57.8	50.9	65.0
Sudan	Blue Nile	Ad Damazin	37037	2000	197.8	183.0	214.5
Sudan	Blue Nile	Ad Damazin	37037	2005	145.4	134.8	157.6
Sudan	Blue Nile	Ad Damazin	37037	2010	119.8	109.0	132.0
Sudan	Blue Nile	Ad Damazin	37037	2015	101.0	89.3	114.9
Sudan	Blue Nile	Al Kurumuk	37038	2000	181.9	167.4	198.1
Sudan	Blue Nile	Al Kurumuk	37038	2005	143.5	132.4	155.7
Sudan	Blue Nile	Al Kurumuk	37038	2010	127.7	116.5	141.1
Sudan	Blue Nile	Al Kurumuk	37038	2015	100.3	87.7	113.9
Sudan	Blue Nile	Al Roseires	37039	2000	181.4	168.8	194.3
Sudan	Blue Nile	Al Roseires	37039	2005	146.1	136.5	156.7
Sudan	Blue Nile	Al Roseires	37039	2010	120.4	110.7	130.7
Sudan	Blue Nile	Al Roseires	37039	2015	96.7	86.0	108.9
Sudan	Blue Nile	Baw	37040	2000	182.4	167.9	197.6
Sudan	Blue Nile	Baw	37040	2005	149.0	137.8	161.1
Sudan	Blue Nile	Baw	37040	2010	126.9	115.5	139.7
Sudan	Blue Nile	Baw	37040	2015	101.9	90.3	115.3
Sudan	Blue Nile	Geissan	37041	2000	193.1	178.9	208.0
Sudan	Blue Nile	Geissan	37041	2005	169.9	157.0	183.4
Sudan	Blue Nile	Geissan	37041	2010	142.2	129.6	156.7
Sudan	Blue Nile	Geissan	37041	2015	101.7	89.7	114.9
Sudan	Gadaref	Al Faw	37046	2000	113.9	104.4	123.4
Sudan	Gadaref	Al Faw	37046	2005	87.4	80.4	94.4
Sudan	Gadaref	Al Faw	37046	2010	83.4	76.2	91.2
Sudan	Gadaref	Al Faw	37046	2015	73.9	65.4	82.8
Sudan	Gadaref	Al Fushqa	68804	2000	122.9	109.6	136.1
Sudan	Gadaref	Al Fushqa	68804	2005	96.8	86.9	107.6
Sudan	Gadaref	Al Fushqa	68804	2010	92.1	81.4	103.1
Sudan	Gadaref	Al Fushqa	68804	2015	84.7	73.6	98.0
Sudan	Gadaref	Al Gadaref	37048	2000	126.7	112.9	142.2
Sudan	Gadaref	Al Gadaref	37048	2005	98.9	87.5	110.8
Sudan	Gadaref	Al Gadaref	37048	2010	91.0	80.2	103.0
Sudan	Gadaref	Al Gadaref	37048	2015	79.8	69.2	93.1
Sudan	Gadaref	Al Galabat	37049	2000	133.4	121.0	146.3
Sudan	Gadaref	Al Galabat	37049	2005	103.0	93.5	113.6
Sudan	Gadaref	Al Galabat	37049	2010	97.3	87.0	109.2
Sudan	Gadaref	Al Galabat	37049	2015	91.5	79.8	104.9
Sudan	Gadaref	Al Rahd	37050	2000	123.4	112.9	133.5
Sudan	Gadaref	Al Rahd	37050	2005	95.5	87.4	103.3
Sudan	Gadaref	Al Rahd	37050	2010	86.4	78.5	94.9
Sudan	Gadaref	Al Rahd	37050	2015	75.2	66.5	85.3
Sudan	Kassala	Al Gash	37058	2000	72.4	65.5	79.6
Sudan	Kassala	Al Gash	37058	2005	62.8	57.1	69.7
Sudan	Kassala	Al Gash	37058	2010	62.2	55.7	69.6
Sudan	Kassala	Al Gash	37058	2015	58.4	50.3	67.0
Sudan	Kassala	Butana	37062	2000	93.4	85.5	102.5
Sudan	Kassala	Butana	37062	2005	76.2	69.2	83.7
Sudan	Kassala	Butana	37062	2010	74.9	67.4	82.9
Sudan	Kassala	Butana	37062	2015	68.1	59.8	77.5
Sudan	Kassala	Hamashkorieb	37059	2000	58.4	51.5	65.7
Sudan	Kassala	Hamashkorieb	37059	2005	52.2	46.0	58.9
Sudan	Kassala	Hamashkorieb	37059	2010	52.1	45.0	59.1
Sudan	Kassala	Hamashkorieb	37059	2015	49.1	41.0	56.8
Sudan	Kassala	Kassala	37060	2000	83.0	72.9	94.3
Sudan	Kassala	Kassala	37060	2005	69.2	60.5	78.7
Sudan	Kassala	Kassala	37060	2010	68.5	60.3	78.7
Sudan	Kassala	Kassala	37060	2015	64.7	55.1	75.4
Sudan	Kassala	Nahr Atbara	37061	2000	85.0	76.7	94.3
Sudan	Kassala	Nahr Atbara	37061	2005	70.1	63.0	77.5
Sudan	Kassala	Nahr Atbara	37061	2010	69.4	61.7	77.5
Sudan	Kassala	Nahr Atbara	37061	2015	64.4	55.4	74.4
Sudan	Kassala	Seteet	68803	2000	108.7	97.6	120.3
Sudan	Kassala	Seteet	68803	2005	87.2	78.7	96.4
Sudan	Kassala	Seteet	68803	2010	84.3	75.0	94.0

Admin 0	Admin 1	Admin 2	GAUL Code	Year	Under-5 mortality (per 1,000 livebirths)		
					Estimate	Lower bound, 95% UI	Upper bound, 95% UI
Sudan	Kassala	Seteet	68803	2015	76.7	66.7	88.1
Sudan	Khartoum	Jabal Aulia	37063	2000	81.8	75.2	89.0
Sudan	Khartoum	Jabal Aulia	37063	2005	63.1	58.2	68.7
Sudan	Khartoum	Jabal Aulia	37063	2010	61.3	55.3	67.4
Sudan	Khartoum	Jabal Aulia	37063	2015	50.9	44.7	57.8
Sudan	Khartoum	Karary	37064	2000	76.2	68.8	84.1
Sudan	Khartoum	Karary	37064	2005	62.8	56.8	68.9
Sudan	Khartoum	Karary	37064	2010	62.0	55.2	69.5
Sudan	Khartoum	Karary	37064	2015	49.9	43.2	57.5
Sudan	Khartoum	Khartoum	37065	2000	77.1	70.3	84.0
Sudan	Khartoum	Khartoum	37065	2005	62.0	57.0	67.5
Sudan	Khartoum	Khartoum	37065	2010	60.9	54.9	67.2
Sudan	Khartoum	Khartoum	37065	2015	48.3	42.2	55.1
Sudan	Khartoum	Khartoum Bahri	37066	2000	77.1	70.3	84.6
Sudan	Khartoum	Khartoum Bahri	37066	2005	62.6	57.3	68.6
Sudan	Khartoum	Khartoum Bahri	37066	2010	61.7	55.4	68.3
Sudan	Khartoum	Khartoum Bahri	37066	2015	48.9	42.6	56.2
Sudan	Khartoum	Sharg En Nile	37067	2000	82.7	75.5	90.5
Sudan	Khartoum	Sharg En Nile	37067	2005	64.4	59.2	70.2
Sudan	Khartoum	Sharg En Nile	37067	2010	62.4	56.2	68.5
Sudan	Khartoum	Sharg En Nile	37067	2015	53.2	46.7	60.1
Sudan	Khartoum	Um Badda	37068	2000	78.2	71.3	85.4
Sudan	Khartoum	Um Badda	37068	2005	63.0	57.5	68.7
Sudan	Khartoum	Um Badda	37068	2010	62.0	55.6	68.1
Sudan	Khartoum	Um Badda	37068	2015	50.3	43.7	57.3
Sudan	Khartoum	Um Durman	37069	2000	77.6	70.3	85.4
Sudan	Khartoum	Um Durman	37069	2005	63.2	57.6	69.6
Sudan	Khartoum	Um Durman	37069	2010	62.6	55.9	69.3
Sudan	Khartoum	Um Durman	37069	2015	50.1	43.5	57.7
Sudan	Nile	Abu Hamad	37070	2000	69.5	59.7	80.5
Sudan	Nile	Abu Hamad	37070	2005	61.4	53.6	70.5
Sudan	Nile	Abu Hamad	37070	2010	60.6	52.8	70.2
Sudan	Nile	Abu Hamad	37070	2015	51.4	43.9	60.3
Sudan	Nile	Ad Damer	37071	2000	70.5	61.3	80.3
Sudan	Nile	Ad Damer	37071	2005	58.6	50.5	66.8
Sudan	Nile	Ad Damer	37071	2010	55.3	48.0	63.2
Sudan	Nile	Ad Damer	37071	2015	46.7	39.3	54.9
Sudan	Nile	Al Matammah	37072	2000	75.6	67.0	84.8
Sudan	Nile	Al Matammah	37072	2005	62.0	55.5	68.9
Sudan	Nile	Al Matammah	37072	2010	60.2	53.6	67.4
Sudan	Nile	Al Matammah	37072	2015	49.4	42.8	57.0
Sudan	Nile	Atbara	37073	2000	69.0	60.4	78.2
Sudan	Nile	Atbara	37073	2005	58.5	51.4	66.2
Sudan	Nile	Atbara	37073	2010	56.2	48.9	63.8
Sudan	Nile	Atbara	37073	2015	48.1	40.6	56.1
Sudan	Nile	Barbar	37074	2000	70.9	60.5	81.8
Sudan	Nile	Barbar	37074	2005	59.3	50.3	69.1
Sudan	Nile	Barbar	37074	2010	56.8	48.1	66.1
Sudan	Nile	Barbar	37074	2015	48.0	40.2	57.5
Sudan	Nile	Shendi	37075	2000	76.1	68.0	84.4
Sudan	Nile	Shendi	37075	2005	62.2	55.8	68.9
Sudan	Nile	Shendi	37075	2010	60.3	53.7	67.0
Sudan	Nile	Shendi	37075	2015	50.9	44.1	58.3
Sudan	Northern	Addabah	37090	2000	69.5	61.0	79.0
Sudan	Northern	Addabah	37090	2005	61.4	53.8	69.7
Sudan	Northern	Addabah	37090	2010	60.6	52.9	69.1
Sudan	Northern	Addabah	37090	2015	48.3	41.5	56.3
Sudan	Northern	Dongola	37091	2000	66.9	59.6	75.0
Sudan	Northern	Dongola	37091	2005	60.5	54.0	68.2
Sudan	Northern	Dongola	37091	2010	59.1	52.2	66.5
Sudan	Northern	Dongola	37091	2015	47.1	40.7	54.5
Sudan	Northern	Merawi	37092	2000	69.1	59.1	80.6
Sudan	Northern	Merawi	37092	2005	61.1	52.4	71.1
Sudan	Northern	Merawi	37092	2010	59.3	50.0	69.8
Sudan	Northern	Merawi	37092	2015	46.4	38.4	55.9
Sudan	Northern	Wadi Halfa	37093	2000	60.5	53.1	69.4
Sudan	Northern	Wadi Halfa	37093	2005	58.6	51.6	66.5
Sudan	Northern	Wadi Halfa	37093	2010	59.4	51.9	67.8
Sudan	Northern	Wadi Halfa	37093	2015	47.8	41.3	55.0
Sudan	Northern Darfur	Al Fasher	37080	2000	82.5	74.2	91.4
Sudan	Northern Darfur	Al Fasher	37080	2005	74.8	67.7	82.1
Sudan	Northern Darfur	Al Fasher	37080	2010	73.4	65.7	82.2
Sudan	Northern Darfur	Al Fasher	37080	2015	72.5	63.3	82.4
Sudan	Northern Darfur	Kabkabiya	37081	2000	81.2	73.0	90.0
Sudan	Northern Darfur	Kabkabiya	37081	2005	74.0	66.9	82.0
Sudan	Northern Darfur	Kabkabiya	37081	2010	71.1	63.5	79.7
Sudan	Northern Darfur	Kabkabiya	37081	2015	70.0	61.3	80.0
Sudan	Northern Darfur	Kutum	37082	2000	71.9	63.6	80.9
Sudan	Northern Darfur	Kutum	37082	2005	65.3	58.0	73.5
Sudan	Northern Darfur	Kutum	37082	2010	64.4	56.7	72.6
Sudan	Northern Darfur	Kutum	37082	2015	62.2	53.6	71.4
Sudan	Northern Darfur	Mellit	37083	2000	69.5	62.4	76.5
Sudan	Northern Darfur	Mellit	37083	2005	64.3	57.6	71.2
Sudan	Northern Darfur	Mellit	37083	2010	65.3	58.4	73.0
Sudan	Northern Darfur	Mellit	37083	2015	59.8	52.8	67.2

Admin 0	Admin 1	Admin 2	GAUL Code	Year	Under-5 mortality (per 1,000 livebirths)		
					Estimate	Lower bound, 95% UI	Upper bound, 95% UI
Sudan	Northern Darfur	Um Kadada	37084	2000	83.1	74.5	93.6
Sudan	Northern Darfur	Um Kadada	37084	2005	77.5	70.1	85.6
Sudan	Northern Darfur	Um Kadada	37084	2010	76.3	68.2	84.7
Sudan	Northern Darfur	Um Kadada	37084	2015	73.5	64.6	83.9
Sudan	Northern Kordofan	Bara	37085	2000	82.9	74.9	92.7
Sudan	Northern Kordofan	Bara	37085	2005	69.5	62.2	77.2
Sudan	Northern Kordofan	Bara	37085	2010	67.4	59.5	76.1
Sudan	Northern Kordofan	Bara	37085	2015	58.0	50.4	66.8
Sudan	Northern Kordofan	En Nuhud	37147	2000	87.2	78.5	96.8
Sudan	Northern Kordofan	En Nuhud	37147	2005	78.9	71.7	87.4
Sudan	Northern Kordofan	En Nuhud	37147	2010	73.8	66.1	82.6
Sudan	Northern Kordofan	En Nuhud	37147	2015	70.0	61.2	80.0
Sudan	Northern Kordofan	Ghebeish	37148	2000	91.8	82.7	102.8
Sudan	Northern Kordofan	Ghebeish	37148	2005	83.6	75.8	92.6
Sudan	Northern Kordofan	Ghebeish	37148	2010	79.5	71.1	88.7
Sudan	Northern Kordofan	Ghebeish	37148	2015	77.5	68.0	88.1
Sudan	Northern Kordofan	Jebrat Al Sheikh	37086	2000	85.4	75.8	96.0
Sudan	Northern Kordofan	Jebrat Al Sheikh	37086	2005	67.2	59.8	75.1
Sudan	Northern Kordofan	Jebrat Al Sheikh	37086	2010	66.6	58.9	75.9
Sudan	Northern Kordofan	Jebrat Al Sheikh	37086	2015	57.4	49.9	66.3
Sudan	Northern Kordofan	Sheikan	37087	2000	90.9	80.2	102.9
Sudan	Northern Kordofan	Sheikan	37087	2005	75.4	66.2	85.6
Sudan	Northern Kordofan	Sheikan	37087	2010	70.4	61.2	80.6
Sudan	Northern Kordofan	Sheikan	37087	2015	63.3	53.7	74.0
Sudan	Northern Kordofan	Sowdari	37088	2000	75.5	67.0	85.3
Sudan	Northern Kordofan	Sowdari	37088	2005	66.4	58.7	74.7
Sudan	Northern Kordofan	Sowdari	37088	2010	65.3	57.3	73.9
Sudan	Northern Kordofan	Sowdari	37088	2015	57.0	49.3	66.2
Sudan	Northern Kordofan	Um Rawaba	37089	2000	97.5	88.1	107.5
Sudan	Northern Kordofan	Um Rawaba	37089	2005	78.7	71.5	87.2
Sudan	Northern Kordofan	Um Rawaba	37089	2010	73.3	65.9	81.3
Sudan	Northern Kordofan	Um Rawaba	37089	2015	63.1	55.5	72.1
Sudan	Red Sea	Halayeb	40771	2000	70.2	59.4	83.0
Sudan	Red Sea	Halayeb	40771	2005	62.5	53.3	73.4
Sudan	Red Sea	Halayeb	40771	2010	61.6	52.1	72.4
Sudan	Red Sea	Halayeb	40771	2015	54.2	45.2	65.1
Sudan	Red Sea	Port Sudan	37095	2000	90.4	79.7	102.6
Sudan	Red Sea	Port Sudan	37095	2005	76.3	67.8	85.8
Sudan	Red Sea	Port Sudan	37095	2010	71.2	62.3	81.1
Sudan	Red Sea	Port Sudan	37095	2015	64.7	54.8	75.5
Sudan	Red Sea	Sinkat	37096	2000	72.5	64.7	81.4
Sudan	Red Sea	Sinkat	37096	2005	64.6	57.9	72.7
Sudan	Red Sea	Sinkat	37096	2010	63.0	56.1	71.3
Sudan	Red Sea	Sinkat	37096	2015	54.6	47.3	62.9
Sudan	Red Sea	Tokar	37097	2000	74.9	66.3	83.7
Sudan	Red Sea	Tokar	37097	2005	67.3	59.5	75.5
Sudan	Red Sea	Tokar	37097	2010	65.0	56.5	73.9
Sudan	Red Sea	Tokar	37097	2015	56.6	48.1	65.3
Sudan	Sennar	Ad Dinder	37098	2000	117.6	108.5	126.8
Sudan	Sennar	Ad Dinder	37098	2005	95.2	88.1	103.1
Sudan	Sennar	Ad Dinder	37098	2010	86.0	78.6	93.7
Sudan	Sennar	Ad Dinder	37098	2015	72.6	64.2	81.7
Sudan	Sennar	Sennar	37099	2000	94.7	87.5	102.9
Sudan	Sennar	Sennar	37099	2005	74.5	69.2	80.9
Sudan	Sennar	Sennar	37099	2010	68.1	62.4	74.6
Sudan	Sennar	Sennar	37099	2015	58.4	51.5	65.7
Sudan	Sennar	Singa	37100	2000	104.2	96.1	112.8
Sudan	Sennar	Singa	37100	2005	84.9	78.4	92.0
Sudan	Sennar	Singa	37100	2010	77.0	70.3	84.1
Sudan	Sennar	Singa	37100	2015	65.2	57.7	73.7
Sudan	Southern Darfur	Adayala	37101	2000	96.4	84.8	108.5
Sudan	Southern Darfur	Adayala	37101	2005	85.8	76.0	96.1
Sudan	Southern Darfur	Adayala	37101	2010	86.0	76.3	97.4
Sudan	Southern Darfur	Adayala	37101	2015	83.9	72.6	96.2
Sudan	Southern Darfur	Al Deain	37102	2000	103.8	95.6	112.9
Sudan	Southern Darfur	Al Deain	37102	2005	91.1	84.0	98.9
Sudan	Southern Darfur	Al Deain	37102	2010	91.6	83.9	100.4
Sudan	Southern Darfur	Al Deain	37102	2015	89.5	79.8	100.0
Sudan	Southern Darfur	Buram	37103	2000	100.5	90.7	111.6
Sudan	Southern Darfur	Buram	37103	2005	86.0	77.7	94.3
Sudan	Southern Darfur	Buram	37103	2010	81.4	73.2	90.4
Sudan	Southern Darfur	Buram	37103	2015	78.8	69.8	89.2
Sudan	Southern Darfur	Edd al Fursan	37104	2000	90.3	81.1	99.3
Sudan	Southern Darfur	Edd al Fursan	37104	2005	80.1	72.3	88.5
Sudan	Southern Darfur	Edd al Fursan	37104	2010	72.8	65.0	80.9
Sudan	Southern Darfur	Edd al Fursan	37104	2015	70.9	61.7	80.9
Sudan	Southern Darfur	Kas	37105	2000	89.8	80.9	99.5
Sudan	Southern Darfur	Kas	37105	2005	81.8	73.4	90.1
Sudan	Southern Darfur	Kas	37105	2010	76.0	67.6	84.5
Sudan	Southern Darfur	Kas	37105	2015	72.7	63.0	82.7
Sudan	Southern Darfur	Nyala	37106	2000	93.3	84.6	102.2
Sudan	Southern Darfur	Nyala	37106	2005	81.5	73.8	89.7
Sudan	Southern Darfur	Nyala	37106	2010	76.8	69.3	84.6
Sudan	Southern Darfur	Nyala	37106	2015	73.2	64.2	83.6
Sudan	Southern Darfur	Rehed Al Birdi	37107	2000	98.8	87.6	111.9

Admin 0	Admin 1	Admin 2	GAUL Code	Year	Under-5 mortality (per 1,000 livebirths)		
					Estimate	Lower bound, 95% UI	Upper bound, 95% UI
Sudan	Southern Darfur	Rehed Al Birdi	37107	2005	85.2	75.9	96.2
Sudan	Southern Darfur	Rehed Al Birdi	37107	2010	75.8	67.2	85.9
Sudan	Southern Darfur	Rehed Al Birdi	37107	2015	76.0	66.1	88.3
Sudan	Southern Darfur	Shearia	37108	2000	93.1	84.5	102.6
Sudan	Southern Darfur	Shearia	37108	2005	84.3	76.4	92.8
Sudan	Southern Darfur	Shearia	37108	2010	82.5	73.5	91.1
Sudan	Southern Darfur	Shearia	37108	2015	79.2	69.1	90.6
Sudan	Southern Darfur	Tulus	37109	2000	87.3	76.6	98.8
Sudan	Southern Darfur	Tulus	37109	2005	76.1	67.4	85.4
Sudan	Southern Darfur	Tulus	37109	2010	70.7	61.7	80.7
Sudan	Southern Darfur	Tulus	37109	2015	67.6	57.8	78.8
Sudan	Southern Kordofan	Abu Jubaiyah	37110	2000	134.7	120.4	149.9
Sudan	Southern Kordofan	Abu Jubaiyah	37110	2005	112.2	100.7	124.2
Sudan	Southern Kordofan	Abu Jubaiyah	37110	2010	100.0	89.6	111.7
Sudan	Southern Kordofan	Abu Jubaiyah	37110	2015	85.6	74.3	98.1
Sudan	Southern Kordofan	Abyei	4151	2000	112.2	100.4	125.1
Sudan	Southern Kordofan	Abyei	4151	2005	93.9	85.0	103.9
Sudan	Southern Kordofan	Abyei	4151	2010	93.8	83.6	104.2
Sudan	Southern Kordofan	Abyei	4151	2015	88.4	77.3	100.4
Sudan	Southern Kordofan	As Salam	37146	2000	108.7	97.0	123.6
Sudan	Southern Kordofan	As Salam	37146	2005	94.2	84.2	106.3
Sudan	Southern Kordofan	As Salam	37146	2010	88.4	77.6	100.1
Sudan	Southern Kordofan	As Salam	37146	2015	80.2	69.8	92.3
Sudan	Southern Kordofan	Delling	37111	2000	123.4	110.6	137.7
Sudan	Southern Kordofan	Delling	37111	2005	107.8	95.9	119.5
Sudan	Southern Kordofan	Delling	37111	2010	92.8	82.7	104.0
Sudan	Southern Kordofan	Delling	37111	2015	80.6	69.8	93.0
Sudan	Southern Kordofan	Kadugli	37112	2000	133.8	121.7	146.6
Sudan	Southern Kordofan	Kadugli	37112	2005	110.5	101.1	120.5
Sudan	Southern Kordofan	Kadugli	37112	2010	101.5	91.9	112.4
Sudan	Southern Kordofan	Kadugli	37112	2015	90.8	80.0	103.1
Sudan	Southern Kordofan	Lagawa	37149	2000	118.8	108.1	130.5
Sudan	Southern Kordofan	Lagawa	37149	2005	100.5	91.9	109.9
Sudan	Southern Kordofan	Lagawa	37149	2010	95.3	86.1	105.7
Sudan	Southern Kordofan	Lagawa	37149	2015	84.7	74.4	95.8
Sudan	Southern Kordofan	Rashad	37113	2000	123.1	110.0	137.1
Sudan	Southern Kordofan	Rashad	37113	2005	102.3	92.3	113.3
Sudan	Southern Kordofan	Rashad	37113	2010	91.3	81.5	101.5
Sudan	Southern Kordofan	Rashad	37113	2015	76.4	66.4	87.5
Sudan	Southern Kordofan	Talodi	37114	2000	129.6	116.0	145.4
Sudan	Southern Kordofan	Talodi	37114	2005	109.2	97.8	121.2
Sudan	Southern Kordofan	Talodi	37114	2010	100.7	89.7	113.5
Sudan	Southern Kordofan	Talodi	37114	2015	87.4	75.9	99.7
Sudan	Western Darfur	Al Geneina	37134	2000	103.4	94.4	113.9
Sudan	Western Darfur	Al Geneina	37134	2005	92.5	85.1	99.8
Sudan	Western Darfur	Al Geneina	37134	2010	81.9	74.7	89.1
Sudan	Western Darfur	Al Geneina	37134	2015	79.1	70.8	88.4
Sudan	Western Darfur	Habillah	37135	2000	115.7	105.6	126.9
Sudan	Western Darfur	Habillah	37135	2005	104.5	96.2	113.9
Sudan	Western Darfur	Habillah	37135	2010	92.9	84.7	101.7
Sudan	Western Darfur	Habillah	37135	2015	84.7	75.2	95.1
Sudan	Western Darfur	Jebel Marra	37136	2000	91.5	82.0	101.8
Sudan	Western Darfur	Jebel Marra	37136	2005	84.2	75.0	93.4
Sudan	Western Darfur	Jebel Marra	37136	2010	78.3	69.5	87.2
Sudan	Western Darfur	Jebel Marra	37136	2015	76.9	66.9	88.2
Sudan	Western Darfur	Kulbus	37137	2000	87.0	79.1	95.6
Sudan	Western Darfur	Kulbus	37137	2005	77.2	70.4	84.2
Sudan	Western Darfur	Kulbus	37137	2010	71.7	65.0	79.1
Sudan	Western Darfur	Kulbus	37137	2015	70.0	61.8	78.9
Sudan	Western Darfur	Mukjar	37138	2000	107.9	97.0	119.1
Sudan	Western Darfur	Mukjar	37138	2005	97.8	88.1	107.8
Sudan	Western Darfur	Mukjar	37138	2010	84.7	76.3	94.0
Sudan	Western Darfur	Mukjar	37138	2015	83.0	72.2	94.0
Sudan	Western Darfur	Wadi Salih	37139	2000	113.6	103.6	124.5
Sudan	Western Darfur	Wadi Salih	37139	2005	104.0	94.4	113.6
Sudan	Western Darfur	Wadi Salih	37139	2010	92.3	83.2	101.9
Sudan	Western Darfur	Wadi Salih	37139	2015	86.1	75.9	96.7
Sudan	Western Darfur	Zallingi	37140	2000	104.7	94.6	115.3
Sudan	Western Darfur	Zallingi	37140	2005	92.6	83.9	101.5
Sudan	Western Darfur	Zallingi	37140	2010	83.5	74.7	91.8
Sudan	Western Darfur	Zallingi	37140	2015	82.0	72.2	92.5
Sudan	White Nile	Ad Douiem	37150	2000	89.0	80.9	97.1
Sudan	White Nile	Ad Douiem	37150	2005	69.9	62.9	77.0
Sudan	White Nile	Ad Douiem	37150	2010	66.5	59.9	73.9
Sudan	White Nile	Ad Douiem	37150	2015	56.7	49.3	64.8
Sudan	White Nile	Al Gutaina	37151	2000	90.3	83.2	97.2
Sudan	White Nile	Al Gutaina	37151	2005	67.6	62.7	72.9
Sudan	White Nile	Al Gutaina	37151	2010	65.4	59.4	71.3
Sudan	White Nile	Al Gutaina	37151	2015	57.0	50.5	64.4
Sudan	White Nile	Al Jabalian	37152	2000	95.0	85.8	103.8
Sudan	White Nile	Al Jabalian	37152	2005	76.9	69.8	84.1
Sudan	White Nile	Al Jabalian	37152	2010	69.3	62.3	76.3
Sudan	White Nile	Al Jabalian	37152	2015	59.4	51.5	68.0
Sudan	White Nile	Kosti	37153	2000	102.5	93.6	112.0
Sudan	White Nile	Kosti	37153	2005	80.1	73.2	87.6

Admin 0	Admin 1	Admin 2	GAUL Code	Year	Under-5 mortality (per 1,000 livebirths)		
					Estimate	Lower bound, 95% UI	Upper bound, 95% UI
Sudan	White Nile	Kosti	37153	2010	73.5	66.6	80.8
Sudan	White Nile	Kosti	37153	2015	63.7	56.0	71.9
Swaziland	Hhohho	Hhukwini	25943	2000	84.9	77.9	92.3
Swaziland	Hhohho	Hhukwini	25943	2005	94.3	86.5	102.8
Swaziland	Hhohho	Hhukwini	25943	2010	69.9	63.4	77.0
Swaziland	Hhohho	Hhukwini	25943	2015	50.9	44.6	58.3
Swaziland	Hhohho	Lobamba	25944	2000	83.4	76.4	91.2
Swaziland	Hhohho	Lobamba	25944	2005	91.7	83.7	100.5
Swaziland	Hhohho	Lobamba	25944	2010	69.9	63.1	77.4
Swaziland	Hhohho	Lobamba	25944	2015	49.5	43.2	56.6
Swaziland	Hhohho	Madlangampisi	25945	2000	85.3	77.2	93.5
Swaziland	Hhohho	Madlangampisi	25945	2005	94.5	85.7	104.1
Swaziland	Hhohho	Madlangampisi	25945	2010	70.8	63.8	78.8
Swaziland	Hhohho	Madlangampisi	25945	2015	50.2	43.7	57.9
Swaziland	Hhohho	Maphalaleni	25946	2000	85.0	78.2	91.7
Swaziland	Hhohho	Maphalaleni	25946	2005	95.3	88.1	102.8
Swaziland	Hhohho	Maphalaleni	25946	2010	71.8	65.8	78.7
Swaziland	Hhohho	Maphalaleni	25946	2015	52.1	45.9	59.2
Swaziland	Hhohho	Mayiwane	25947	2000	81.6	73.1	90.4
Swaziland	Hhohho	Mayiwane	25947	2005	84.6	75.9	93.5
Swaziland	Hhohho	Mayiwane	25947	2010	57.4	51.4	64.5
Swaziland	Hhohho	Mayiwane	25947	2015	47.4	41.0	54.8
Swaziland	Hhohho	Mbabane	25948	2000	80.3	72.9	88.1
Swaziland	Hhohho	Mbabane	25948	2005	89.1	80.8	98.1
Swaziland	Hhohho	Mbabane	25948	2010	68.9	61.9	76.8
Swaziland	Hhohho	Mbabane	25948	2015	48.2	42.1	55.6
Swaziland	Hhohho	Mhlangatane	25949	2000	83.5	75.2	92.2
Swaziland	Hhohho	Mhlangatane	25949	2005	89.6	80.1	99.9
Swaziland	Hhohho	Mhlangatane	25949	2010	60.3	54.1	67.5
Swaziland	Hhohho	Mhlangatane	25949	2015	48.4	41.9	55.4
Swaziland	Hhohho	Motjane	25950	2000	81.8	74.4	89.6
Swaziland	Hhohho	Motjane	25950	2005	87.4	79.1	96.6
Swaziland	Hhohho	Motjane	25950	2010	61.5	55.3	68.3
Swaziland	Hhohho	Motjane	25950	2015	49.5	42.9	56.6
Swaziland	Hhohho	Ndzingeni	25951	2000	83.7	75.5	92.2
Swaziland	Hhohho	Ndzingeni	25951	2005	90.6	82.0	99.7
Swaziland	Hhohho	Ndzingeni	25951	2010	63.1	57.1	70.6
Swaziland	Hhohho	Ndzingeni	25951	2015	49.7	43.1	57.2
Swaziland	Hhohho	Nkaba	25952	2000	79.9	73.1	86.9
Swaziland	Hhohho	Nkaba	25952	2005	84.7	77.9	92.6
Swaziland	Hhohho	Nkaba	25952	2010	60.7	54.8	66.9
Swaziland	Hhohho	Nkaba	25952	2015	49.7	43.6	56.9
Swaziland	Hhohho	Ntfonjeni	25953	2000	76.7	68.3	86.1
Swaziland	Hhohho	Ntfonjeni	25953	2005	77.8	69.5	86.9
Swaziland	Hhohho	Ntfonjeni	25953	2010	55.4	49.0	62.5
Swaziland	Hhohho	Ntfonjeni	25953	2015	46.1	39.5	54.2
Swaziland	Hhohho	Pigg's Peak	25954	2000	78.8	71.9	86.9
Swaziland	Hhohho	Pigg's Peak	25954	2005	82.1	74.8	90.3
Swaziland	Hhohho	Pigg's Peak	25954	2010	57.7	52.0	64.1
Swaziland	Hhohho	Pigg's Peak	25954	2015	48.1	41.7	55.3
Swaziland	Hhohho	Timpisini	25955	2000	73.8	65.7	82.7
Swaziland	Hhohho	Timpisini	25955	2005	74.8	66.9	83.8
Swaziland	Hhohho	Timpisini	25955	2010	53.9	47.5	60.9
Swaziland	Hhohho	Timpisini	25955	2015	44.6	38.2	52.0
Swaziland	Lubombo	Dvokodvweni	25956	2000	96.3	88.0	105.2
Swaziland	Lubombo	Dvokodvweni	25956	2005	107.5	98.3	116.9
Swaziland	Lubombo	Dvokodvweni	25956	2010	81.2	73.6	89.8
Swaziland	Lubombo	Dvokodvweni	25956	2015	56.0	49.1	64.1
Swaziland	Lubombo	Hlane	25957	2000	92.0	84.1	100.5
Swaziland	Lubombo	Hlane	25957	2005	101.7	93.5	110.8
Swaziland	Lubombo	Hlane	25957	2010	76.8	69.8	84.3
Swaziland	Lubombo	Hlane	25957	2015	53.2	46.7	60.6
Swaziland	Lubombo	Lomahasha	25958	2000	101.5	91.3	113.4
Swaziland	Lubombo	Lomahasha	25958	2005	101.4	91.0	112.9
Swaziland	Lubombo	Lomahasha	25958	2010	70.1	62.5	79.0
Swaziland	Lubombo	Lomahasha	25958	2015	52.9	45.5	61.9
Swaziland	Lubombo	Lubuli	25959	2000	100.7	90.8	111.1
Swaziland	Lubombo	Lubuli	25959	2005	102.3	92.4	114.2
Swaziland	Lubombo	Lubuli	25959	2010	80.4	71.2	89.5
Swaziland	Lubombo	Lubuli	25959	2015	57.0	49.7	65.7
Swaziland	Lubombo	Lugongolweni	25960	2000	103.1	92.0	115.4
Swaziland	Lubombo	Lugongolweni	25960	2005	109.2	97.6	122.3
Swaziland	Lubombo	Lugongolweni	25960	2010	84.5	74.5	95.7
Swaziland	Lubombo	Lugongolweni	25960	2015	58.0	50.5	68.0
Swaziland	Lubombo	Mhlume	25961	2000	88.7	80.5	97.8
Swaziland	Lubombo	Mhlume	25961	2005	96.2	87.3	105.9
Swaziland	Lubombo	Mhlume	25961	2010	70.1	62.9	77.5
Swaziland	Lubombo	Mhlume	25961	2015	49.5	42.8	56.8
Swaziland	Lubombo	Mpolonjeni	25962	2000	104.6	94.7	114.3
Swaziland	Lubombo	Mpolonjeni	25962	2005	109.7	100.4	120.1
Swaziland	Lubombo	Mpolonjeni	25962	2010	84.0	75.6	93.5
Swaziland	Lubombo	Mpolonjeni	25962	2015	57.4	50.4	66.3
Swaziland	Lubombo	Nkilongo	25963	2000	98.2	88.2	108.2
Swaziland	Lubombo	Nkilongo	25963	2005	103.9	93.9	115.2
Swaziland	Lubombo	Nkilongo	25963	2010	79.5	70.5	88.9

Admin 0	Admin 1	Admin 2	GAUL Code	Year	Under-5 mortality (per 1,000 livebirths)		
					Estimate	Lower bound, 95% UI	Upper bound, 95% UI
Swaziland	Lubombo	Nkilongo	25963	2015	54.7	47.8	63.2
Swaziland	Lubombo	Siphofaneni	25964	2000	99.6	91.7	108.0
Swaziland	Lubombo	Siphofaneni	25964	2005	110.3	101.8	119.9
Swaziland	Lubombo	Siphofaneni	25964	2010	81.0	73.6	89.0
Swaziland	Lubombo	Siphofaneni	25964	2015	56.7	50.0	64.3
Swaziland	Lubombo	Sithobela	25965	2000	103.5	94.9	112.9
Swaziland	Lubombo	Sithobela	25965	2005	111.3	102.2	121.4
Swaziland	Lubombo	Sithobela	25965	2010	85.2	77.2	94.3
Swaziland	Lubombo	Sithobela	25965	2015	58.8	51.8	67.5
Swaziland	Lubombo	Tikhuba	25966	2000	116.5	104.6	128.3
Swaziland	Lubombo	Tikhuba	25966	2005	110.6	99.5	122.5
Swaziland	Lubombo	Tikhuba	25966	2010	86.5	77.1	96.6
Swaziland	Lubombo	Tikhuba	25966	2015	58.0	50.6	67.1
Swaziland	Manzini	Kukhanyeni	25967	2000	90.1	82.9	97.8
Swaziland	Manzini	Kukhanyeni	25967	2005	99.9	92.0	108.6
Swaziland	Manzini	Kukhanyeni	25967	2010	76.4	69.5	83.8
Swaziland	Manzini	Kukhanyeni	25967	2015	52.6	45.8	59.9
Swaziland	Manzini	Kwaluseni	25968	2000	89.3	82.4	96.6
Swaziland	Manzini	Kwaluseni	25968	2005	101.0	93.1	108.8
Swaziland	Manzini	Kwaluseni	25968	2010	73.9	67.3	81.2
Swaziland	Manzini	Kwaluseni	25968	2015	50.7	44.6	57.6
Swaziland	Manzini	Lamgabhi	25969	2000	90.1	82.3	98.4
Swaziland	Manzini	Lamgabhi	25969	2005	95.9	87.4	104.8
Swaziland	Manzini	Lamgabhi	25969	2010	73.4	66.4	81.3
Swaziland	Manzini	Lamgabhi	25969	2015	52.6	46.0	60.1
Swaziland	Manzini	Ludzeludze	25970	2000	88.3	81.5	95.6
Swaziland	Manzini	Ludzeludze	25970	2005	96.5	89.4	104.4
Swaziland	Manzini	Ludzeludze	25970	2010	73.7	67.1	80.9
Swaziland	Manzini	Ludzeludze	25970	2015	50.9	44.7	57.8
Swaziland	Manzini	Mafutseni	25971	2000	95.2	86.9	103.8
Swaziland	Manzini	Mafutseni	25971	2005	107.2	97.9	117.1
Swaziland	Manzini	Mafutseni	25971	2010	79.8	72.5	87.6
Swaziland	Manzini	Mafutseni	25971	2015	53.3	46.2	61.0
Swaziland	Manzini	Mahlangatja	25972	2000	102.2	93.2	112.1
Swaziland	Manzini	Mahlangatja	25972	2005	110.8	100.9	121.3
Swaziland	Manzini	Mahlangatja	25972	2010	85.5	77.4	94.3
Swaziland	Manzini	Mahlangatja	25972	2015	58.8	51.4	67.0
Swaziland	Manzini	Mahlanya	25973	2000	87.3	80.2	94.8
Swaziland	Manzini	Mahlanya	25973	2005	94.4	86.8	102.0
Swaziland	Manzini	Mahlanya	25973	2010	71.4	65.0	78.3
Swaziland	Manzini	Mahlanya	25973	2015	49.0	42.9	55.7
Swaziland	Manzini	Mangcongco	25974	2000	92.2	82.4	103.2
Swaziland	Manzini	Mangcongco	25974	2005	89.7	80.2	101.4
Swaziland	Manzini	Mangcongco	25974	2010	68.0	60.2	76.6
Swaziland	Manzini	Mangcongco	25974	2015	54.3	46.4	63.1
Swaziland	Manzini	Manzini	25975	2000	89.5	82.5	96.9
Swaziland	Manzini	Manzini	25975	2005	101.9	94.4	110.3
Swaziland	Manzini	Manzini	25975	2010	74.7	68.1	82.0
Swaziland	Manzini	Manzini	25975	2015	50.8	44.6	57.9
Swaziland	Manzini	Mhlambanyatsi	25976	2000	88.2	80.7	96.4
Swaziland	Manzini	Mhlambanyatsi	25976	2005	89.7	82.0	98.3
Swaziland	Manzini	Mhlambanyatsi	25976	2010	68.5	61.9	76.0
Swaziland	Manzini	Mhlambanyatsi	25976	2015	51.6	45.0	59.1
Swaziland	Manzini	Mkhiweni	25977	2000	88.2	80.9	96.1
Swaziland	Manzini	Mkhiweni	25977	2005	100.6	91.9	110.5
Swaziland	Manzini	Mkhiweni	25977	2010	74.8	67.7	82.3
Swaziland	Manzini	Mkhiweni	25977	2015	51.7	45.0	59.3
Swaziland	Manzini	Mthongwaneni	25978	2000	97.5	89.7	105.6
Swaziland	Manzini	Mthongwaneni	25978	2005	109.1	100.6	118.6
Swaziland	Manzini	Mthongwaneni	25978	2010	79.2	72.0	87.1
Swaziland	Manzini	Mthongwaneni	25978	2015	54.6	47.8	62.1
Swaziland	Manzini	Ngwempisi	25979	2000	95.8	86.4	106.5
Swaziland	Manzini	Ngwempisi	25979	2005	100.7	90.4	112.6
Swaziland	Manzini	Ngwempisi	25979	2010	79.2	70.4	88.4
Swaziland	Manzini	Ngwempisi	25979	2015	56.2	48.8	64.9
Swaziland	Manzini	Nhlambeni	25980	2000	97.3	89.2	105.8
Swaziland	Manzini	Nhlambeni	25980	2005	108.3	99.1	117.9
Swaziland	Manzini	Nhlambeni	25980	2010	78.8	71.3	86.5
Swaziland	Manzini	Nhlambeni	25980	2015	54.1	47.4	61.8
Swaziland	Manzini	Ntondozi	25981	2000	94.1	86.5	102.4
Swaziland	Manzini	Ntondozi	25981	2005	102.6	94.1	111.7
Swaziland	Manzini	Ntondozi	25981	2010	77.3	70.3	84.4
Swaziland	Manzini	Ntondozi	25981	2015	53.7	47.2	61.1
Swaziland	Shiselweni	Gege	25982	2000	100.8	92.0	111.1
Swaziland	Shiselweni	Gege	25982	2005	104.0	94.6	114.7
Swaziland	Shiselweni	Gege	25982	2010	82.6	74.1	90.9
Swaziland	Shiselweni	Gege	25982	2015	58.1	51.2	66.4
Swaziland	Shiselweni	Hosea	25983	2000	104.8	94.9	114.9
Swaziland	Shiselweni	Hosea	25983	2005	109.5	99.1	120.5
Swaziland	Shiselweni	Hosea	25983	2010	85.7	77.0	95.4
Swaziland	Shiselweni	Hosea	25983	2015	58.8	51.7	67.4
Swaziland	Shiselweni	Kubuta	25984	2000	105.7	96.2	115.5
Swaziland	Shiselweni	Kubuta	25984	2005	115.0	104.7	125.8
Swaziland	Shiselweni	Kubuta	25984	2010	87.8	79.3	97.4
Swaziland	Shiselweni	Kubuta	25984	2015	60.5	53.1	69.4

Admin 0	Admin 1	Admin 2	GAUL Code	Year	Under-5 mortality (per 1,000 livebirths) Estimate	Lower bound, 95% UI	Upper bound, 95% UI
Swaziland	Shiselweni	Lavumisa	25985	2000	98.8	88.0	110.9
Swaziland	Shiselweni	Lavumisa	25985	2005	92.9	82.2	104.9
Swaziland	Shiselweni	Lavumisa	25985	2010	72.3	63.5	82.1
Swaziland	Shiselweni	Lavumisa	25985	2015	55.6	47.8	64.5
Swaziland	Shiselweni	Maseyisini	25986	2000	101.8	92.0	112.8
Swaziland	Shiselweni	Maseyisini	25986	2005	105.5	94.5	117.3
Swaziland	Shiselweni	Maseyisini	25986	2010	82.4	73.7	91.5
Swaziland	Shiselweni	Maseyisini	25986	2015	58.3	50.7	67.1
Swaziland	Shiselweni	Matsanjeni	25987	2000	101.2	91.3	111.6
Swaziland	Shiselweni	Matsanjeni	25987	2005	101.4	91.4	113.3
Swaziland	Shiselweni	Matsanjeni	25987	2010	79.4	70.3	88.8
Swaziland	Shiselweni	Matsanjeni	25987	2015	57.6	50.0	65.8
Swaziland	Shiselweni	Mbangweni	25988	2000	105.7	96.3	116.0
Swaziland	Shiselweni	Mbangweni	25988	2005	110.1	99.7	120.9
Swaziland	Shiselweni	Mbangweni	25988	2010	85.6	77.4	95.0
Swaziland	Shiselweni	Mbangweni	25988	2015	59.3	52.1	67.5
Swaziland	Shiselweni	Mtsambama	25989	2000	104.9	96.2	114.4
Swaziland	Shiselweni	Mtsambama	25989	2005	112.1	102.7	121.8
Swaziland	Shiselweni	Mtsambama	25989	2010	87.2	79.2	96.1
Swaziland	Shiselweni	Mtsambama	25989	2015	59.4	52.4	67.7
Swaziland	Shiselweni	Ngudzeni	25990	2000	105.9	96.0	115.6
Swaziland	Shiselweni	Ngudzeni	25990	2005	112.4	101.9	122.9
Swaziland	Shiselweni	Ngudzeni	25990	2010	87.2	79.0	97.0
Swaziland	Shiselweni	Ngudzeni	25990	2015	59.6	52.5	68.2
Swaziland	Shiselweni	Nkwene	25991	2000	106.2	97.0	116.5
Swaziland	Shiselweni	Nkwene	25991	2005	113.8	103.5	124.8
Swaziland	Shiselweni	Nkwene	25991	2010	87.8	79.5	97.0
Swaziland	Shiselweni	Nkwene	25991	2015	60.0	52.6	68.8
Swaziland	Shiselweni	Sandleni	25992	2000	105.3	95.8	115.1
Swaziland	Shiselweni	Sandleni	25992	2005	112.1	102.2	122.4
Swaziland	Shiselweni	Sandleni	25992	2010	86.7	78.7	95.7
Swaziland	Shiselweni	Sandleni	25992	2015	59.3	52.6	67.6
Swaziland	Shiselweni	Shiselweni 1	25993	2000	99.7	90.0	109.7
Swaziland	Shiselweni	Shiselweni 1	25993	2005	101.1	91.3	111.1
Swaziland	Shiselweni	Shiselweni 1	25993	2010	80.4	71.9	89.7
Swaziland	Shiselweni	Shiselweni 1	25993	2015	57.3	50.2	65.6
Swaziland	Shiselweni	Sigwe	25994	2000	102.9	93.8	112.3
Swaziland	Shiselweni	Sigwe	25994	2005	108.9	99.3	118.8
Swaziland	Shiselweni	Sigwe	25994	2010	84.8	76.5	93.9
Swaziland	Shiselweni	Sigwe	25994	2015	58.5	51.6	66.9
Swaziland	Shiselweni	Zombodze	25995	2000	101.4	91.8	111.1
Swaziland	Shiselweni	Zombodze	25995	2005	105.9	95.9	116.4
Swaziland	Shiselweni	Zombodze	25995	2010	83.7	75.0	92.9
Swaziland	Shiselweni	Zombodze	25995	2015	58.7	51.2	67.1
Togo	Centrale	Blitta	65275	2000	133.8	121.9	147.4
Togo	Centrale	Blitta	65275	2005	116.5	106.5	127.3
Togo	Centrale	Blitta	65275	2010	100.4	90.6	111.0
Togo	Centrale	Blitta	65275	2015	86.1	75.7	97.6
Togo	Centrale	Sotouboua	65285	2000	138.4	126.2	150.7
Togo	Centrale	Sotouboua	65285	2005	125.1	115.1	135.8
Togo	Centrale	Sotouboua	65285	2010	105.7	96.5	115.2
Togo	Centrale	Sotouboua	65285	2015	87.1	77.3	98.8
Togo	Centrale	Tchamba	27386	2000	138.7	127.8	150.8
Togo	Centrale	Tchamba	27386	2005	123.6	114.3	134.8
Togo	Centrale	Tchamba	27386	2010	107.3	98.2	117.7
Togo	Centrale	Tchamba	27386	2015	86.6	77.5	97.6
Togo	Centrale	Tchaoudjo	27387	2000	138.1	124.9	152.1
Togo	Centrale	Tchaoudjo	27387	2005	124.2	113.3	135.9
Togo	Centrale	Tchaoudjo	27387	2010	104.5	95.1	115.2
Togo	Centrale	Tchaoudjo	27387	2015	79.1	70.3	89.3
Togo	Kara	Assoli	27388	2000	139.6	128.4	151.6
Togo	Kara	Assoli	27388	2005	128.1	117.8	138.7
Togo	Kara	Assoli	27388	2010	107.6	98.2	117.9
Togo	Kara	Assoli	27388	2015	80.2	70.9	89.7
Togo	Kara	Bassar	65274	2000	154.7	141.2	168.9
Togo	Kara	Bassar	65274	2005	145.1	132.5	158.5
Togo	Kara	Bassar	65274	2010	115.5	104.9	126.5
Togo	Kara	Bassar	65274	2015	94.7	83.9	106.6
Togo	Kara	Binah	27390	2000	132.1	122.2	142.8
Togo	Kara	Binah	27390	2005	118.5	109.4	128.3
Togo	Kara	Binah	27390	2010	99.9	91.4	109.4
Togo	Kara	Binah	27390	2015	79.7	70.5	89.7
Togo	Kara	Dankpen	65276	2000	162.4	149.8	177.4
Togo	Kara	Dankpen	65276	2005	154.7	142.2	168.0
Togo	Kara	Dankpen	65276	2010	122.5	111.8	134.2
Togo	Kara	Dankpen	65276	2015	108.4	95.9	122.3
Togo	Kara	Doufelgou	27391	2000	136.8	126.7	148.3
Togo	Kara	Doufelgou	27391	2005	124.1	114.7	134.3
Togo	Kara	Doufelgou	27391	2010	102.4	93.5	112.0
Togo	Kara	Doufelgou	27391	2015	82.9	73.8	93.0
Togo	Kara	Keran	27392	2000	141.6	130.7	153.8
Togo	Kara	Keran	27392	2005	127.3	116.3	138.7
Togo	Kara	Keran	27392	2010	107.2	97.5	116.9
Togo	Kara	Keran	27392	2015	93.8	82.8	106.1
Togo	Kara	Kozah	27393	2000	132.9	121.4	145.1

Admin 0	Admin 1	Admin 2	GAUL Code	Year	Under-5 mortality (per 1,000 livebirths)		
					Estimate	Lower bound, 95% UI	Upper bound, 95% UI
Togo	Kara	Kozah	27393	2005	122.5	111.9	133.2
Togo	Kara	Kozah	27393	2010	101.4	92.1	111.2
Togo	Kara	Kozah	27393	2015	78.0	69.0	87.9
Togo	Maritime	Ave	65273	2000	109.9	101.7	119.1
Togo	Maritime	Ave	65273	2005	94.6	87.6	101.8
Togo	Maritime	Ave	65273	2010	78.4	72.3	85.4
Togo	Maritime	Ave	65273	2015	60.5	53.6	67.8
Togo	Maritime	Golfe	27394	2000	88.2	81.9	95.5
Togo	Maritime	Golfe	27394	2005	80.6	74.4	87.1
Togo	Maritime	Golfe	27394	2010	68.9	63.2	75.0
Togo	Maritime	Golfe	27394	2015	48.0	42.6	54.0
Togo	Maritime	Lacs	27395	2000	105.4	98.5	113.2
Togo	Maritime	Lacs	27395	2005	86.9	80.9	93.5
Togo	Maritime	Lacs	27395	2010	74.8	68.9	80.8
Togo	Maritime	Lacs	27395	2015	65.0	57.8	72.4
Togo	Maritime	Vo	27396	2000	105.1	97.8	113.5
Togo	Maritime	Vo	27396	2005	86.7	80.1	93.8
Togo	Maritime	Vo	27396	2010	73.6	67.5	80.1
Togo	Maritime	Vo	27396	2015	62.4	55.3	69.8
Togo	Maritime	Yoto	27397	2000	111.7	104.0	119.8
Togo	Maritime	Yoto	27397	2005	93.1	85.7	99.8
Togo	Maritime	Yoto	27397	2010	77.8	71.5	83.9
Togo	Maritime	Yoto	27397	2015	71.5	63.3	79.4
Togo	Maritime	Zio	65288	2000	112.2	104.3	120.6
Togo	Maritime	Zio	65288	2005	94.2	87.3	101.3
Togo	Maritime	Zio	65288	2010	78.0	72.0	84.2
Togo	Maritime	Zio	65288	2015	63.8	56.9	71.0
Togo	Plateaux	Agou	65272	2000	107.5	97.8	118.4
Togo	Plateaux	Agou	65272	2005	93.3	85.4	102.3
Togo	Plateaux	Agou	65272	2010	81.1	73.7	89.3
Togo	Plateaux	Agou	65272	2015	67.0	59.4	75.9
Togo	Plateaux	Amou	27399	2000	127.5	115.3	140.8
Togo	Plateaux	Amou	27399	2005	110.1	100.2	120.7
Togo	Plateaux	Amou	27399	2010	92.9	83.7	102.5
Togo	Plateaux	Amou	27399	2015	82.5	72.5	94.0
Togo	Plateaux	Danyi	65277	2000	103.0	93.3	114.0
Togo	Plateaux	Danyi	65277	2005	90.0	81.8	99.1
Togo	Plateaux	Danyi	65277	2010	80.9	72.7	89.5
Togo	Plateaux	Danyi	65277	2015	65.1	57.6	73.5
Togo	Plateaux	Est-Mono	65278	2000	139.3	126.3	153.2
Togo	Plateaux	Est-Mono	65278	2005	121.9	111.1	133.5
Togo	Plateaux	Est-Mono	65278	2010	105.8	95.8	116.8
Togo	Plateaux	Est-Mono	65278	2015	93.0	82.0	105.2
Togo	Plateaux	Haho	65279	2000	125.9	115.4	137.5
Togo	Plateaux	Haho	65279	2005	106.9	98.4	116.3
Togo	Plateaux	Haho	65279	2010	88.2	80.4	96.4
Togo	Plateaux	Haho	65279	2015	81.3	72.2	91.1
Togo	Plateaux	Kloto	65280	2000	97.6	89.7	106.8
Togo	Plateaux	Kloto	65280	2005	85.1	78.5	92.6
Togo	Plateaux	Kloto	65280	2010	78.5	71.7	86.0
Togo	Plateaux	Kloto	65280	2015	61.7	55.2	69.5
Togo	Plateaux	Moyen-Mono	65282	2000	139.8	129.2	151.1
Togo	Plateaux	Moyen-Mono	65282	2005	117.2	108.4	126.8
Togo	Plateaux	Moyen-Mono	65282	2010	99.2	90.2	108.3
Togo	Plateaux	Moyen-Mono	65282	2015	96.0	85.2	107.8
Togo	Plateaux	Ogou	65283	2000	138.5	126.5	151.1
Togo	Plateaux	Ogou	65283	2005	119.5	109.7	130.2
Togo	Plateaux	Ogou	65283	2010	101.0	92.1	110.5
Togo	Plateaux	Ogou	65283	2015	93.1	82.5	105.0
Togo	Plateaux	Wawa	27403	2000	117.9	107.0	129.3
Togo	Plateaux	Wawa	27403	2005	102.1	92.9	112.1
Togo	Plateaux	Wawa	27403	2010	89.6	80.6	99.0
Togo	Plateaux	Wawa	27403	2015	76.4	67.2	86.3
Togo	Savanes	Kpendjal	65281	2000	162.1	150.7	174.0
Togo	Savanes	Kpendjal	65281	2005	148.6	137.9	159.8
Togo	Savanes	Kpendjal	65281	2010	125.5	115.0	136.3
Togo	Savanes	Kpendjal	65281	2015	95.3	85.6	106.0
Togo	Savanes	Oti	65284	2000	156.0	145.1	167.8
Togo	Savanes	Oti	65284	2005	144.2	133.2	154.4
Togo	Savanes	Oti	65284	2010	121.2	112.1	130.4
Togo	Savanes	Oti	65284	2015	100.0	90.3	110.8
Togo	Savanes	Tandjouare	65286	2000	155.3	144.1	167.6
Togo	Savanes	Tandjouare	65286	2005	143.2	133.7	153.6
Togo	Savanes	Tandjouare	65286	2010	118.0	108.9	127.7
Togo	Savanes	Tandjouare	65286	2015	89.6	80.3	99.5
Togo	Savanes	Tone	65287	2000	151.1	141.0	161.6
Togo	Savanes	Tone	65287	2005	139.1	129.9	148.7
Togo	Savanes	Tone	65287	2010	113.1	104.7	121.6
Togo	Savanes	Tone	65287	2015	84.8	76.5	93.8
Uganda	Abim	Labwor	28455	2000	153.6	140.3	168.7
Uganda	Abim	Labwor	28455	2005	129.6	118.4	142.0
Uganda	Abim	Labwor	28455	2010	104.5	94.4	115.0
Uganda	Abim	Labwor	28455	2015	82.0	72.1	92.5
Uganda	Adjumani	Adjumani	28381	2000	144.1	133.2	156.4
Uganda	Adjumani	Adjumani	28381	2005	122.8	113.4	132.6

Admin 0	Admin 1	Admin 2	GAUL Code	Year	Under-5 mortality (per 1,000 livebirths) Estimate	Lower bound, 95% UI	Upper bound, 95% UI
Uganda	Adjumani	Adjumani	28381	2010	102.4	93.4	111.9
Uganda	Adjumani	Adjumani	28381	2015	81.0	72.2	91.4
Uganda	Agago	Agago	28449	2000	162.2	148.6	177.3
Uganda	Agago	Agago	28449	2005	135.6	124.6	147.8
Uganda	Agago	Agago	28449	2010	108.3	98.3	118.5
Uganda	Agago	Agago	28449	2015	85.6	75.3	95.6
Uganda	Alebtong	Moroto	28463	2000	141.0	131.8	150.5
Uganda	Alebtong	Moroto	28463	2005	120.0	112.6	128.4
Uganda	Alebtong	Moroto	28463	2010	96.7	90.0	103.7
Uganda	Alebtong	Moroto	28463	2015	77.9	69.7	85.9
Uganda	Amolatar	Kyoga	28461	2000	122.2	113.1	132.3
Uganda	Amolatar	Kyoga	28461	2005	100.4	93.4	108.2
Uganda	Amolatar	Kyoga	28461	2010	85.0	78.8	92.5
Uganda	Amolatar	Kyoga	28461	2015	68.5	61.0	76.6
Uganda	Amudat	Upe	28495	2000	129.5	119.9	139.3
Uganda	Amudat	Upe	28495	2005	104.5	96.7	113.5
Uganda	Amudat	Upe	28495	2010	77.6	70.6	85.4
Uganda	Amudat	Upe	28495	2015	66.4	58.8	74.1
Uganda	Amuria	Amuria	28441	2000	129.1	119.8	139.1
Uganda	Amuria	Amuria	28441	2005	109.0	101.2	116.9
Uganda	Amuria	Amuria	28441	2010	88.7	82.4	95.5
Uganda	Amuria	Amuria	28441	2015	71.1	63.6	79.3
Uganda	Amuria	Kapelebyong	28442	2000	138.4	127.5	150.3
Uganda	Amuria	Kapelebyong	28442	2005	117.6	108.4	127.5
Uganda	Amuria	Kapelebyong	28442	2010	94.9	87.5	103.5
Uganda	Amuria	Kapelebyong	28442	2015	74.1	66.1	83.1
Uganda	Amuru	Kilak	28405	2000	157.2	145.5	169.2
Uganda	Amuru	Kilak	28405	2005	133.2	123.2	143.7
Uganda	Amuru	Kilak	28405	2010	108.5	99.1	117.9
Uganda	Amuru	Kilak	28405	2015	88.9	78.8	100.8
Uganda	Apac	Kwania	28383	2000	128.5	120.3	138.0
Uganda	Apac	Kwania	28383	2005	106.5	99.2	114.0
Uganda	Apac	Kwania	28383	2010	89.1	82.6	95.9
Uganda	Apac	Kwania	28383	2015	73.0	65.3	81.4
Uganda	Apac	Maruzi	28384	2000	129.0	120.1	138.7
Uganda	Apac	Maruzi	28384	2005	107.4	100.1	115.1
Uganda	Apac	Maruzi	28384	2010	89.9	83.0	96.8
Uganda	Apac	Maruzi	28384	2015	73.5	65.6	82.0
Uganda	Arua	Arua Municipality	28387	2000	151.9	141.4	163.7
Uganda	Arua	Arua Municipality	28387	2005	127.2	118.2	137.6
Uganda	Arua	Arua Municipality	28387	2010	101.3	92.4	111.1
Uganda	Arua	Arua Municipality	28387	2015	76.1	67.8	85.6
Uganda	Arua	Ayivu	28388	2000	163.6	152.6	175.4
Uganda	Arua	Ayivu	28388	2005	135.8	126.3	146.7
Uganda	Arua	Ayivu	28388	2010	105.8	96.6	115.8
Uganda	Arua	Ayivu	28388	2015	80.3	71.4	89.9
Uganda	Arua	Madi-okollo	28390	2000	168.7	157.3	180.3
Uganda	Arua	Madi-okollo	28390	2005	141.8	132.5	153.2
Uganda	Arua	Madi-okollo	28390	2010	109.9	101.4	119.6
Uganda	Arua	Madi-okollo	28390	2015	87.3	78.6	98.7
Uganda	Arua	Terego	28392	2000	162.9	153.1	172.5
Uganda	Arua	Terego	28392	2005	135.5	127.4	144.1
Uganda	Arua	Terego	28392	2010	107.3	99.4	115.6
Uganda	Arua	Terego	28392	2015	85.4	76.5	94.7
Uganda	Arua	Vurra	28393	2000	170.3	160.1	181.0
Uganda	Arua	Vurra	28393	2005	142.2	132.8	152.0
Uganda	Arua	Vurra	28393	2010	109.0	100.5	118.5
Uganda	Arua	Vurra	28393	2015	85.4	76.4	95.7
Uganda	Budaka	Budaka	28520	2000	123.2	116.4	130.3
Uganda	Budaka	Budaka	28520	2005	101.5	96.1	107.3
Uganda	Budaka	Budaka	28520	2010	83.1	77.8	88.8
Uganda	Budaka	Budaka	28520	2015	65.3	59.2	72.0
Uganda	Bududa	Manjiya	28480	2000	116.2	109.1	123.9
Uganda	Bududa	Manjiya	28480	2005	93.1	87.2	99.4
Uganda	Bududa	Manjiya	28480	2010	76.9	71.2	82.8
Uganda	Bududa	Manjiya	28480	2015	58.8	53.3	65.1
Uganda	Bugiri	Bukooli	28394	2000	155.4	146.9	164.3
Uganda	Bugiri	Bukooli	28394	2005	127.8	120.1	135.1
Uganda	Bugiri	Bukooli	28394	2010	100.1	93.4	107.1
Uganda	Bugiri	Bukooli	28394	2015	74.6	67.5	82.2
Uganda	Buhweju	Buhweju	28397	2000	151.1	140.9	160.4
Uganda	Buhweju	Buhweju	28397	2005	120.5	111.9	129.5
Uganda	Buhweju	Buhweju	28397	2010	84.4	77.6	92.2
Uganda	Buhweju	Buhweju	28397	2015	63.8	57.2	71.4
Uganda	Buikwe	Buyikwe	28510	2000	111.1	104.1	118.9
Uganda	Buikwe	Buyikwe	28510	2005	100.3	94.1	106.9
Uganda	Buikwe	Buyikwe	28510	2010	85.9	80.1	92.4
Uganda	Buikwe	Buyikwe	28510	2015	56.7	50.7	62.9
Uganda	Bukedea	Bukedea	28456	2000	114.7	108.4	121.4
Uganda	Bukedea	Bukedea	28456	2005	94.9	89.4	100.9
Uganda	Bukedea	Bukedea	28456	2010	78.2	72.9	83.9
Uganda	Bukedea	Bukedea	28456	2015	63.2	57.2	70.0
Uganda	Bukomansimbi	Bukomansimbi	28468	2000	138.4	129.4	147.4
Uganda	Bukomansimbi	Bukomansimbi	28468	2005	116.5	108.7	124.5
Uganda	Bukomansimbi	Bukomansimbi	28468	2010	100.1	92.9	108.5

Admin 0	Admin 1	Admin 2	GAUL Code	Year	Under-5 mortality (per 1,000 livebirths)		
					Estimate	Lower bound, 95% UI	Upper bound, 95% UI
Uganda	Bukomansimbi	Bukomansimbi	28468	2015	70.6	63.5	79.2
Uganda	Bukwo	Kongasis	42203	2000	94.6	86.9	102.3
Uganda	Bukwo	Kongasis	42203	2005	73.7	67.3	80.2
Uganda	Bukwo	Kongasis	42203	2010	58.7	53.4	64.5
Uganda	Bukwo	Kongasis	42203	2015	48.8	43.5	54.6
Uganda	Bulambuli	Bulambuli	28478	2000	109.6	102.8	116.8
Uganda	Bulambuli	Bulambuli	28478	2005	89.1	83.0	95.4
Uganda	Bulambuli	Bulambuli	28478	2010	73.8	68.0	79.5
Uganda	Bulambuli	Bulambuli	28478	2015	58.5	52.7	65.2
Uganda	Buliisa	Buliisa	28473	2000	157.6	143.3	172.8
Uganda	Buliisa	Buliisa	28473	2005	133.1	121.2	145.2
Uganda	Buliisa	Buliisa	28473	2010	105.1	95.7	116.2
Uganda	Buliisa	Buliisa	28473	2015	82.0	71.9	92.6
Uganda	Bundibugyo	Bwamba	28395	2000	153.3	141.0	167.3
Uganda	Bundibugyo	Bwamba	28395	2005	126.4	115.7	137.4
Uganda	Bundibugyo	Bwamba	28395	2010	91.6	82.9	101.1
Uganda	Bundibugyo	Bwamba	28395	2015	70.8	62.3	79.6
Uganda	Bushenyi	Igara	28399	2000	144.4	134.9	154.1
Uganda	Bushenyi	Igara	28399	2005	115.8	107.8	124.3
Uganda	Bushenyi	Igara	28399	2010	80.8	74.3	88.3
Uganda	Bushenyi	Igara	28399	2015	61.9	55.3	69.2
Uganda	Busia	Samia-bugwe	28402	2000	160.0	152.8	168.1
Uganda	Busia	Samia-bugwe	28402	2005	131.3	124.5	138.6
Uganda	Busia	Samia-bugwe	28402	2010	103.2	96.7	110.1
Uganda	Busia	Samia-bugwe	28402	2015	73.7	66.7	81.0
Uganda	Butaleja	Bunyole	28539	2000	133.8	125.9	141.8
Uganda	Butaleja	Bunyole	28539	2005	109.7	103.5	116.1
Uganda	Butaleja	Bunyole	28539	2010	88.9	83.4	95.2
Uganda	Butaleja	Bunyole	28539	2015	68.7	62.3	75.7
Uganda	Butambala	Butambala	28499	2000	105.4	98.3	114.0
Uganda	Butambala	Butambala	28499	2005	95.0	88.2	102.2
Uganda	Butambala	Butambala	28499	2010	82.1	75.7	89.1
Uganda	Butambala	Butambala	28499	2015	59.2	53.4	66.4
Uganda	Buvuma	Buvuma Islands	28509	2000	137.7	126.8	148.9
Uganda	Buvuma	Buvuma Islands	28509	2005	117.0	107.6	126.5
Uganda	Buvuma	Buvuma Islands	28509	2010	98.4	90.3	106.7
Uganda	Buvuma	Buvuma Islands	28509	2015	62.6	55.9	69.8
Uganda	Buyende	Budiope	28432	2000	130.2	121.3	139.4
Uganda	Buyende	Budiope	28432	2005	110.8	103.5	118.4
Uganda	Buyende	Budiope	28432	2010	90.1	83.8	96.6
Uganda	Buyende	Budiope	28432	2015	70.6	63.8	78.5
Uganda	Dokolo	Dokolo	28459	2000	126.8	117.9	136.2
Uganda	Dokolo	Dokolo	28459	2005	105.0	98.6	112.5
Uganda	Dokolo	Dokolo	28459	2010	87.1	80.9	94.2
Uganda	Dokolo	Dokolo	28459	2015	70.6	63.3	78.1
Uganda	Gomba	Gomba	28501	2000	115.4	107.6	123.7
Uganda	Gomba	Gomba	28501	2005	101.8	95.4	109.1
Uganda	Gomba	Gomba	28501	2010	87.8	81.4	94.8
Uganda	Gomba	Gomba	28501	2015	64.5	57.8	71.9
Uganda	Gulu	Aswa	28403	2000	157.2	145.2	169.4
Uganda	Gulu	Aswa	28403	2005	131.2	121.7	142.2
Uganda	Gulu	Aswa	28403	2010	105.2	96.1	115.1
Uganda	Gulu	Aswa	28403	2015	86.4	76.1	97.5
Uganda	Gulu	Gulu Municipality	28404	2000	144.9	133.0	157.8
Uganda	Gulu	Gulu Municipality	28404	2005	119.7	109.9	131.0
Uganda	Gulu	Gulu Municipality	28404	2010	100.0	91.0	110.1
Uganda	Gulu	Gulu Municipality	28404	2015	80.7	70.7	91.5
Uganda	Gulu	Omoro	28407	2000	158.5	147.8	169.7
Uganda	Gulu	Omoro	28407	2005	132.6	123.6	141.9
Uganda	Gulu	Omoro	28407	2010	105.9	98.0	115.0
Uganda	Gulu	Omoro	28407	2015	87.6	77.9	97.8
Uganda	Hoima	Bugahya	28408	2000	135.8	123.8	148.5
Uganda	Hoima	Bugahya	28408	2005	116.5	106.2	127.4
Uganda	Hoima	Bugahya	28408	2010	94.2	86.1	103.4
Uganda	Hoima	Bugahya	28408	2015	73.6	65.2	82.9
Uganda	Hoima	Buhaguzi	28409	2000	141.8	131.5	152.6
Uganda	Hoima	Buhaguzi	28409	2005	122.0	113.0	131.7
Uganda	Hoima	Buhaguzi	28409	2010	97.8	89.5	105.7
Uganda	Hoima	Buhaguzi	28409	2015	77.9	69.4	86.8
Uganda	Ibanda	Ibanda	28483	2000	164.8	154.8	175.4
Uganda	Ibanda	Ibanda	28483	2005	135.6	127.0	145.4
Uganda	Ibanda	Ibanda	28483	2010	98.0	90.8	106.5
Uganda	Ibanda	Ibanda	28483	2015	73.7	66.6	82.1
Uganda	Iganga	Bugweri	28410	2000	144.1	136.6	152.1
Uganda	Iganga	Bugweri	28410	2005	120.8	114.2	127.1
Uganda	Iganga	Bugweri	28410	2010	96.1	90.0	102.6
Uganda	Iganga	Bugweri	28410	2015	71.6	64.9	78.8
Uganda	Iganga	Kigulu	28413	2000	136.6	129.1	145.0
Uganda	Iganga	Kigulu	28413	2005	115.3	108.5	122.1
Uganda	Iganga	Kigulu	28413	2010	92.6	86.6	99.3
Uganda	Iganga	Kigulu	28413	2015	67.8	61.0	74.8
Uganda	Isingiro	Bukanga	28482	2000	169.5	156.3	183.2
Uganda	Isingiro	Bukanga	28482	2005	138.6	127.6	149.3
Uganda	Isingiro	Bukanga	28482	2010	101.7	93.2	111.5
Uganda	Isingiro	Bukanga	28482	2015	69.4	61.2	78.2

Admin 0	Admin 1	Admin 2	GAUL Code	Year	Under-5 mortality (per 1,000 livebirths)		
					Estimate	Lower bound, 95% UI	Upper bound, 95% UI
Uganda	Isingiro	Isingiro	28484	2000	164.7	154.5	176.6
Uganda	Isingiro	Isingiro	28484	2005	131.3	122.3	141.2
Uganda	Isingiro	Isingiro	28484	2010	91.2	84.3	99.1
Uganda	Isingiro	Isingiro	28484	2015	65.1	58.2	72.8
Uganda	Jinja	Butembe	28415	2000	110.0	103.7	116.5
Uganda	Jinja	Butembe	28415	2005	100.3	94.7	106.3
Uganda	Jinja	Butembe	28415	2010	86.1	80.6	92.1
Uganda	Jinja	Butembe	28415	2015	57.3	51.7	63.3
Uganda	Jinja	Jinja Municipality	28416	2000	96.6	90.4	103.0
Uganda	Jinja	Jinja Municipality	28416	2005	93.1	87.3	99.1
Uganda	Jinja	Jinja Municipality	28416	2010	83.3	77.4	89.7
Uganda	Jinja	Jinja Municipality	28416	2015	55.0	49.1	61.0
Uganda	Jinja	Kagoma	28417	2000	115.2	109.0	121.9
Uganda	Jinja	Kagoma	28417	2005	103.7	98.1	109.4
Uganda	Jinja	Kagoma	28417	2010	86.7	81.4	92.7
Uganda	Jinja	Kagoma	28417	2015	58.7	52.9	64.5
Uganda	Kaabong	Dodoth	28453	2000	145.6	134.0	157.9
Uganda	Kaabong	Dodoth	28453	2005	120.0	109.6	130.9
Uganda	Kaabong	Dodoth	28453	2010	95.8	87.0	104.9
Uganda	Kaabong	Dodoth	28453	2015	77.1	67.9	86.9
Uganda	Kabale	Kabale Municipality	28418	2000	132.5	124.9	139.7
Uganda	Kabale	Kabale Municipality	28418	2005	98.7	92.5	105.1
Uganda	Kabale	Kabale Municipality	28418	2010	68.8	63.9	74.1
Uganda	Kabale	Kabale Municipality	28418	2015	52.4	47.2	58.1
Uganda	Kabale	Ndorwa	28419	2000	158.8	150.8	166.6
Uganda	Kabale	Ndorwa	28419	2005	110.2	103.7	116.8
Uganda	Kabale	Ndorwa	28419	2010	73.9	68.9	79.3
Uganda	Kabale	Ndorwa	28419	2015	55.7	50.3	61.5
Uganda	Kabale	Rubanda	28420	2000	146.0	137.8	153.5
Uganda	Kabale	Rubanda	28420	2005	105.1	98.8	111.3
Uganda	Kabale	Rubanda	28420	2010	71.0	65.7	76.3
Uganda	Kabale	Rubanda	28420	2015	51.8	46.8	57.6
Uganda	Kabale	Rukiga	28421	2000	150.9	143.1	159.2
Uganda	Kabale	Rukiga	28421	2005	111.5	104.8	118.1
Uganda	Kabale	Rukiga	28421	2010	75.5	70.2	81.5
Uganda	Kabale	Rukiga	28421	2015	55.0	49.5	61.3
Uganda	Kabarole	Bunyangabu	28422	2000	145.8	135.9	155.8
Uganda	Kabarole	Bunyangabu	28422	2005	119.9	110.8	128.8
Uganda	Kabarole	Bunyangabu	28422	2010	87.7	80.2	95.8
Uganda	Kabarole	Bunyangabu	28422	2015	68.9	61.0	77.5
Uganda	Kabarole	Burahya	28423	2000	153.3	142.5	165.0
Uganda	Kabarole	Burahya	28423	2005	126.4	117.0	135.8
Uganda	Kabarole	Burahya	28423	2010	92.0	84.3	100.5
Uganda	Kabarole	Burahya	28423	2015	72.0	63.9	80.8
Uganda	Kabarole	Fort Portal Municipality	28424	2000	135.8	124.7	147.1
Uganda	Kabarole	Fort Portal Municipality	28424	2005	115.7	105.8	125.6
Uganda	Kabarole	Fort Portal Municipality	28424	2010	86.9	79.0	96.1
Uganda	Kabarole	Fort Portal Municipality	28424	2015	67.4	59.5	76.1
Uganda	Kaberamaido	Kaberamaido	28533	2000	121.4	111.8	131.0
Uganda	Kaberamaido	Kaberamaido	28533	2005	100.8	93.9	109.0
Uganda	Kaberamaido	Kaberamaido	28533	2010	83.3	77.1	90.0
Uganda	Kaberamaido	Kaberamaido	28533	2015	67.6	60.5	75.5
Uganda	Kaberamaido	Kalaki	28534	2000	121.9	113.0	131.1
Uganda	Kaberamaido	Kalaki	28534	2005	102.0	95.4	109.8
Uganda	Kaberamaido	Kalaki	28534	2010	83.8	77.9	90.7
Uganda	Kaberamaido	Kalaki	28534	2015	68.2	61.6	75.8
Uganda	Kalangala	Bujumba	28429	2000	128.6	116.9	141.7
Uganda	Kalangala	Bujumba	28429	2005	105.5	95.6	116.5
Uganda	Kalangala	Bujumba	28429	2010	84.7	76.5	93.8
Uganda	Kalangala	Bujumba	28429	2015	61.8	54.8	70.0
Uganda	Kalangala	Kyamuswa	28430	2000	130.5	117.2	145.1
Uganda	Kalangala	Kyamuswa	28430	2005	107.9	96.7	120.0
Uganda	Kalangala	Kyamuswa	28430	2010	87.7	78.3	97.9
Uganda	Kalangala	Kyamuswa	28430	2015	60.5	53.4	69.3
Uganda	Kaliro	Bulamogi	28434	2000	133.5	124.4	142.5
Uganda	Kaliro	Bulamogi	28434	2005	112.9	106.2	120.2
Uganda	Kaliro	Bulamogi	28434	2010	91.4	85.2	98.2
Uganda	Kaliro	Bulamogi	28434	2015	71.3	64.4	79.2
Uganda	Kalungu	Kalungu	28470	2000	126.9	118.1	135.9
Uganda	Kalungu	Kalungu	28470	2005	107.4	99.5	116.0
Uganda	Kalungu	Kalungu	28470	2010	92.1	85.0	99.8
Uganda	Kalungu	Kalungu	28470	2015	65.9	59.1	73.9
Uganda	Kampala	Kampala	149623	2000	80.6	76.0	85.4
Uganda	Kampala	Kampala	149623	2005	77.8	73.5	82.2
Uganda	Kampala	Kampala	149623	2010	70.3	65.9	75.1
Uganda	Kampala	Kampala	149623	2015	51.0	45.7	56.5
Uganda	Kampala	Central Kampala	42202	2000	78.8	74.4	83.5
Uganda	Kampala	Central Kampala	42202	2005	76.7	72.4	80.9
Uganda	Kampala	Central Kampala	42202	2010	69.3	64.9	74.0
Uganda	Kampala	Central Kampala	42202	2015	50.8	45.4	56.2
Uganda	Kampala	Kawempe	42213	2000	79.3	75.0	84.1
Uganda	Kampala	Kawempe	42213	2005	76.0	72.0	80.3
Uganda	Kampala	Kawempe	42213	2010	68.5	64.3	73.1
Uganda	Kampala	Kawempe	42213	2015	50.3	45.1	55.8
Uganda	Kampala	Makerere University	42214	2000	79.3	75.0	84.1

Admin 0	Admin 1	Admin 2	GAUL Code	Year	Under-5 mortality (per 1,000 livebirths)		
					Estimate	Lower bound, 95% UI	Upper bound, 95% UI
Uganda	Kampala	Makerere University	42214	2005	76.0	72.0	80.3
Uganda	Kampala	Makerere University	42214	2010	68.5	64.3	73.1
Uganda	Kampala	Makerere University	42214	2015	50.3	45.1	55.8
Uganda	Kampala	Nakawa	42215	2000	80.4	76.1	85.0
Uganda	Kampala	Nakawa	42215	2005	78.7	74.4	82.8
Uganda	Kampala	Nakawa	42215	2010	70.8	66.4	75.4
Uganda	Kampala	Nakawa	42215	2015	51.1	45.8	56.5
Uganda	Kampala	Rubaga	42216	2000	81.4	77.1	86.3
Uganda	Kampala	Rubaga	42216	2005	77.9	73.7	82.2
Uganda	Kampala	Rubaga	42216	2010	69.9	65.5	74.6
Uganda	Kampala	Rubaga	42216	2015	50.7	45.6	56.2
Uganda	Kamuli	Bugabula	28433	2000	128.9	121.7	137.0
Uganda	Kamuli	Bugabula	28433	2005	111.1	103.8	118.0
Uganda	Kamuli	Bugabula	28433	2010	90.4	84.3	97.3
Uganda	Kamuli	Bugabula	28433	2015	67.9	61.3	75.0
Uganda	Kamuli	Buzaaya	28435	2000	122.3	115.3	129.3
Uganda	Kamuli	Buzaaya	28435	2005	106.6	100.1	112.9
Uganda	Kamuli	Buzaaya	28435	2010	88.0	82.2	94.7
Uganda	Kamuli	Buzaaya	28435	2015	62.0	56.0	68.5
Uganda	Kamwenge	Kibale	28425	2000	167.8	156.5	180.5
Uganda	Kamwenge	Kibale	28425	2005	140.2	130.7	151.0
Uganda	Kamwenge	Kibale	28425	2010	103.6	95.0	113.0
Uganda	Kamwenge	Kibale	28425	2015	80.7	71.9	90.3
Uganda	Kamwenge	Kitagwenda	28426	2000	165.2	153.0	177.6
Uganda	Kamwenge	Kitagwenda	28426	2005	135.5	124.5	146.6
Uganda	Kamwenge	Kitagwenda	28426	2010	97.9	89.7	107.6
Uganda	Kamwenge	Kitagwenda	28426	2015	75.4	67.6	84.6
Uganda	Kanungu	Kinkiizi	28528	2000	135.8	125.1	147.7
Uganda	Kanungu	Kinkiizi	28528	2005	104.6	96.6	113.5
Uganda	Kanungu	Kinkiizi	28528	2010	71.3	64.7	78.2
Uganda	Kanungu	Kinkiizi	28528	2015	53.4	47.3	60.2
Uganda	Kapchorwa	Tingey	42205	2000	108.2	100.7	116.2
Uganda	Kapchorwa	Tingey	42205	2005	86.6	80.0	93.4
Uganda	Kapchorwa	Tingey	42205	2010	70.6	65.0	76.5
Uganda	Kapchorwa	Tingey	42205	2015	55.8	49.7	62.2
Uganda	Kasese	Bukonjo	42206	2000	121.1	111.1	131.2
Uganda	Kasese	Bukonjo	42206	2005	98.5	90.4	107.9
Uganda	Kasese	Bukonjo	42206	2010	73.2	66.4	80.8
Uganda	Kasese	Bukonjo	42206	2015	54.9	48.6	62.2
Uganda	Kasese	Busongora	42207	2000	129.8	120.2	139.6
Uganda	Kasese	Busongora	42207	2005	108.5	100.2	117.1
Uganda	Kasese	Busongora	42207	2010	80.6	73.9	88.0
Uganda	Kasese	Busongora	42207	2015	62.0	55.3	69.4
Uganda	Katakwi	Usuk	28443	2000	130.3	120.5	140.6
Uganda	Katakwi	Usuk	28443	2005	109.6	101.3	118.1
Uganda	Katakwi	Usuk	28443	2010	88.4	81.2	95.7
Uganda	Katakwi	Usuk	28443	2015	68.6	61.1	76.7
Uganda	Kayunga	Bbaale	28508	2000	119.6	112.4	126.9
Uganda	Kayunga	Bbaale	28508	2005	102.2	96.1	108.5
Uganda	Kayunga	Bbaale	28508	2010	86.1	80.1	92.4
Uganda	Kayunga	Bbaale	28508	2015	61.2	55.0	68.1
Uganda	Kayunga	Ntenjeru	28513	2000	114.2	107.6	121.2
Uganda	Kayunga	Ntenjeru	28513	2005	100.5	94.6	106.9
Uganda	Kayunga	Ntenjeru	28513	2010	84.7	78.9	91.1
Uganda	Kayunga	Ntenjeru	28513	2015	57.5	51.8	63.7
Uganda	Kibaale	Bugangaizi	28444	2000	138.0	127.6	148.7
Uganda	Kibaale	Bugangaizi	28444	2005	116.4	108.1	125.0
Uganda	Kibaale	Bugangaizi	28444	2010	92.8	85.4	101.0
Uganda	Kibaale	Bugangaizi	28444	2015	74.3	66.6	83.3
Uganda	Kibaale	Buyaga	28445	2000	151.5	140.2	163.5
Uganda	Kibaale	Buyaga	28445	2005	125.8	116.5	136.1
Uganda	Kibaale	Buyaga	28445	2010	98.8	90.9	107.4
Uganda	Kibaale	Buyaga	28445	2015	79.1	71.1	88.4
Uganda	Kibaale	Buyanja	28446	2000	149.0	137.2	160.7
Uganda	Kibaale	Buyanja	28446	2005	124.1	114.7	134.4
Uganda	Kibaale	Buyanja	28446	2010	97.1	88.9	105.5
Uganda	Kibaale	Buyanja	28446	2015	77.9	70.0	87.6
Uganda	Kibuku	Kibuku	28522	2000	128.0	120.4	135.6
Uganda	Kibuku	Kibuku	28522	2005	106.6	100.7	112.7
Uganda	Kibuku	Kibuku	28522	2010	86.1	80.3	92.2
Uganda	Kibuku	Kibuku	28522	2015	69.1	62.4	76.2
Uganda	Kiruhura	Kazo	28486	2000	167.8	156.5	180.6
Uganda	Kiruhura	Kazo	28486	2005	141.8	131.7	153.0
Uganda	Kiruhura	Kazo	28486	2010	106.8	98.6	116.3
Uganda	Kiruhura	Kazo	28486	2015	79.0	71.2	87.9
Uganda	Kiruhura	Nyabushozi	28488	2000	167.7	156.2	179.9
Uganda	Kiruhura	Nyabushozi	28488	2005	138.9	129.4	150.1
Uganda	Kiruhura	Nyabushozi	28488	2010	103.9	96.1	113.1
Uganda	Kiruhura	Nyabushozi	28488	2015	75.6	68.4	84.3
Uganda	Kiryandongo	Kibanda	28475	2000	138.0	126.2	149.2
Uganda	Kiryandongo	Kibanda	28475	2005	114.1	104.7	124.0
Uganda	Kiryandongo	Kibanda	28475	2010	94.3	86.5	103.0
Uganda	Kiryandongo	Kibanda	28475	2015	75.4	67.0	85.2
Uganda	Kisoro	Bufumbira	28448	2000	165.4	155.1	175.6
Uganda	Kisoro	Bufumbira	28448	2005	116.1	108.6	123.9

Admin 0	Admin 1	Admin 2	GAUL Code	Year	Under-5 mortality (per 1,000 livebirths)		
					Estimate	Lower bound, 95% UI	Upper bound, 95% UI
Uganda	Kisoro	Bufumbira	28448	2010	76.3	70.5	82.1
Uganda	Kisoro	Bufumbira	28448	2015	55.1	49.5	61.1
Uganda	Kitgum	Chua	28451	2000	168.5	152.9	182.5
Uganda	Kitgum	Chua	28451	2005	138.1	125.7	151.6
Uganda	Kitgum	Chua	28451	2010	110.6	100.0	122.1
Uganda	Kitgum	Chua	28451	2015	88.3	78.0	100.4
Uganda	Koboko	Koboko	28389	2000	157.6	145.6	170.0
Uganda	Koboko	Koboko	28389	2005	133.0	122.7	143.7
Uganda	Koboko	Koboko	28389	2010	106.4	97.2	116.9
Uganda	Koboko	Koboko	28389	2015	83.8	73.7	95.3
Uganda	Kole	Kole	28382	2000	142.4	133.8	151.7
Uganda	Kole	Kole	28382	2005	119.8	112.8	127.3
Uganda	Kole	Kole	28382	2010	98.3	91.6	105.2
Uganda	Kole	Kole	28382	2015	79.8	71.6	88.1
Uganda	Kotido	Jie	28454	2000	136.8	125.4	148.9
Uganda	Kotido	Jie	28454	2005	115.3	104.6	125.8
Uganda	Kotido	Jie	28454	2010	96.6	87.4	106.5
Uganda	Kotido	Jie	28454	2015	74.8	65.5	84.9
Uganda	Kumi	Kumi	28457	2000	116.3	109.2	123.8
Uganda	Kumi	Kumi	28457	2005	97.8	91.9	104.1
Uganda	Kumi	Kumi	28457	2010	80.0	74.6	85.6
Uganda	Kumi	Kumi	28457	2015	65.2	58.8	72.0
Uganda	Kween	Kween	42204	2000	104.5	96.4	112.5
Uganda	Kween	Kween	42204	2005	82.4	75.8	89.2
Uganda	Kween	Kween	42204	2010	65.8	60.1	71.5
Uganda	Kween	Kween	42204	2015	53.1	47.5	59.3
Uganda	Kyankwanzi	Kiboga	28447	2000	125.5	115.4	135.7
Uganda	Kyankwanzi	Kiboga	28447	2005	105.1	97.2	113.3
Uganda	Kyankwanzi	Kiboga	28447	2010	85.6	78.4	93.5
Uganda	Kyankwanzi	Kiboga	28447	2015	68.6	61.4	76.8
Uganda	Kyegegwa	Kyaka	28427	2000	159.7	146.7	173.8
Uganda	Kyegegwa	Kyaka	28427	2005	132.4	122.5	143.2
Uganda	Kyegegwa	Kyaka	28427	2010	104.6	95.7	114.3
Uganda	Kyegegwa	Kyaka	28427	2015	82.2	73.2	91.8
Uganda	Kyenjojo	Mwenge	28428	2000	160.2	149.2	172.2
Uganda	Kyenjojo	Mwenge	28428	2005	133.4	124.6	143.2
Uganda	Kyenjojo	Mwenge	28428	2010	99.7	91.8	108.8
Uganda	Kyenjojo	Mwenge	28428	2015	79.2	70.6	88.5
Uganda	Lamwo	Lamwo	28452	2000	164.5	148.9	180.5
Uganda	Lamwo	Lamwo	28452	2005	135.4	122.4	149.5
Uganda	Lamwo	Lamwo	28452	2010	112.9	101.4	125.1
Uganda	Lamwo	Lamwo	28452	2015	90.3	79.6	102.4
Uganda	Lira	Erute	28460	2000	144.1	134.4	153.8
Uganda	Lira	Erute	28460	2005	121.4	113.5	129.0
Uganda	Lira	Erute	28460	2010	98.7	92.0	105.5
Uganda	Lira	Erute	28460	2015	80.1	71.5	88.5
Uganda	Lira	Lira Municipality	28462	2000	127.4	118.3	137.5
Uganda	Lira	Lira Municipality	28462	2005	107.5	100.1	115.3
Uganda	Lira	Lira Municipality	28462	2010	93.0	86.1	100.1
Uganda	Lira	Lira Municipality	28462	2015	73.0	64.8	81.0
Uganda	Luuka	Luuka	28414	2000	133.5	126.6	140.8
Uganda	Luuka	Luuka	28414	2005	114.0	108.1	120.3
Uganda	Luuka	Luuka	28414	2010	92.1	86.5	98.5
Uganda	Luuka	Luuka	28414	2015	67.8	61.4	74.7
Uganda	Luwero	Katikamu	28465	2000	104.9	97.7	113.4
Uganda	Luwero	Katikamu	28465	2005	91.2	85.0	98.7
Uganda	Luwero	Katikamu	28465	2010	76.9	70.9	83.6
Uganda	Luwero	Katikamu	28465	2015	54.9	49.2	61.9
Uganda	Luwero	Wabusana (bamunanika)	28467	2000	108.7	102.0	116.1
Uganda	Luwero	Wabusana (bamunanika)	28467	2005	94.5	88.6	101.1
Uganda	Luwero	Wabusana (bamunanika)	28467	2010	79.2	73.3	85.3
Uganda	Luwero	Wabusana (bamunanika)	28467	2015	56.1	50.5	62.6
Uganda	Lwengo	Bukoto	149624	2000	157.5	147.9	167.7
Uganda	Lwengo	Bukoto	149624	2005	128.0	120.2	136.6
Uganda	Lwengo	Bukoto	149624	2010	105.7	97.9	114.1
Uganda	Lwengo	Bukoto	149624	2015	73.8	66.3	82.4
Uganda	Lyantonde	Kabula	28524	2000	170.2	158.4	182.2
Uganda	Lyantonde	Kabula	28524	2005	138.4	129.7	149.1
Uganda	Lyantonde	Kabula	28524	2010	111.2	103.0	120.4
Uganda	Lyantonde	Kabula	28524	2015	78.7	71.1	88.0
Uganda	Manafwa	Bubulo	28476	2000	123.7	117.2	131.3
Uganda	Manafwa	Bubulo	28476	2005	99.4	93.9	105.3
Uganda	Manafwa	Bubulo	28476	2010	81.6	76.2	87.6
Uganda	Manafwa	Bubulo	28476	2015	61.9	56.1	68.2
Uganda	Maracha	Maracha	28391	2000	163.3	152.6	174.3
Uganda	Maracha	Maracha	28391	2005	136.6	128.0	146.2
Uganda	Maracha	Maracha	28391	2010	106.9	98.4	116.2
Uganda	Maracha	Maracha	28391	2015	83.8	74.4	93.6
Uganda	Masaka	Bukoto	28469	2000	134.0	125.0	145.0
Uganda	Masaka	Bukoto	28469	2005	111.1	102.2	120.4
Uganda	Masaka	Bukoto	28469	2010	94.6	86.7	103.5
Uganda	Masaka	Bukoto	28469	2015	66.7	59.9	74.7
Uganda	Masaka	Masaka Municipality	28471	2000	118.5	110.0	128.1
Uganda	Masaka	Masaka Municipality	28471	2005	103.1	94.7	111.7
Uganda	Masaka	Masaka Municipality	28471	2010	89.1	81.4	97.4

Admin 0	Admin 1	Admin 2	GAUL Code	Year	Under-5 mortality (per 1,000 livebirths) Estimate	Lower bound, 95% UI	Upper bound, 95% UI
Uganda	Masaka	Masaka Municipality	28471	2015	62.7	56.0	70.6
Uganda	Masindi	Bujenje	28472	2000	136.0	124.2	148.8
Uganda	Masindi	Bujenje	28472	2005	114.6	104.2	124.9
Uganda	Masindi	Bujenje	28472	2010	92.0	83.8	100.3
Uganda	Masindi	Bujenje	28472	2015	72.5	63.9	81.6
Uganda	Masindi	Buruli M	42208	2000	136.9	123.4	151.7
Uganda	Masindi	Buruli M	42208	2005	113.4	102.4	125.1
Uganda	Masindi	Buruli M	42208	2010	91.3	82.4	100.7
Uganda	Masindi	Buruli M	42208	2015	72.2	63.5	81.8
Uganda	Mayuge	Bunya	28411	2000	144.6	135.9	153.0
Uganda	Mayuge	Bunya	28411	2005	122.3	115.0	129.7
Uganda	Mayuge	Bunya	28411	2010	98.3	91.7	105.0
Uganda	Mayuge	Bunya	28411	2015	68.4	61.9	75.6
Uganda	Mbale	Bungokho	28479	2000	118.3	112.0	125.1
Uganda	Mbale	Bungokho	28479	2005	96.4	91.0	102.0
Uganda	Mbale	Bungokho	28479	2010	79.3	73.9	85.2
Uganda	Mbale	Bungokho	28479	2015	61.7	56.0	68.0
Uganda	Mbale	Mbale Municipality	28481	2000	109.2	103.2	115.7
Uganda	Mbale	Mbale Municipality	28481	2005	91.6	86.3	97.1
Uganda	Mbale	Mbale Municipality	28481	2010	76.2	70.9	81.8
Uganda	Mbale	Mbale Municipality	28481	2015	58.8	53.4	65.1
Uganda	Mbarara	Kashari	28485	2000	159.1	149.2	170.1
Uganda	Mbarara	Kashari	28485	2005	128.2	119.2	137.8
Uganda	Mbarara	Kashari	28485	2010	90.4	83.6	98.1
Uganda	Mbarara	Kashari	28485	2015	67.4	60.6	75.3
Uganda	Mbarara	Mbarara Municipality	28487	2000	140.0	130.0	151.2
Uganda	Mbarara	Mbarara Municipality	28487	2005	111.2	102.6	120.6
Uganda	Mbarara	Mbarara Municipality	28487	2010	82.8	75.8	90.4
Uganda	Mbarara	Mbarara Municipality	28487	2015	62.3	55.8	69.9
Uganda	Mbarara	Rwampara	28489	2000	158.0	148.9	167.6
Uganda	Mbarara	Rwampara	28489	2005	123.0	115.5	131.1
Uganda	Mbarara	Rwampara	28489	2010	83.8	77.6	90.6
Uganda	Mbarara	Rwampara	28489	2015	62.5	56.1	69.4
Uganda	Mitooma	Ruhinda	28400	2000	142.7	133.2	152.5
Uganda	Mitooma	Ruhinda	28400	2005	113.4	105.7	121.4
Uganda	Mitooma	Ruhinda	28400	2010	78.7	72.6	85.5
Uganda	Mitooma	Ruhinda	28400	2015	60.0	53.8	66.9
Uganda	Mityana	Busujju	28504	2000	112.3	104.9	120.7
Uganda	Mityana	Busujju	28504	2005	97.7	91.5	104.5
Uganda	Mityana	Busujju	28504	2010	82.3	76.3	88.4
Uganda	Mityana	Busujju	28504	2015	61.6	55.3	68.6
Uganda	Mityana	Mityana	28507	2000	115.9	107.6	125.4
Uganda	Mityana	Mityana	28507	2005	97.2	90.1	104.3
Uganda	Mityana	Mityana	28507	2010	80.4	73.9	87.2
Uganda	Mityana	Mityana	28507	2015	61.2	54.7	68.6
Uganda	Moroto	Matheniko	28492	2000	149.2	137.1	162.7
Uganda	Moroto	Matheniko	28492	2005	122.5	112.1	133.5
Uganda	Moroto	Matheniko	28492	2010	97.5	88.3	107.5
Uganda	Moroto	Matheniko	28492	2015	79.8	69.1	91.1
Uganda	Moroto	Moroto Municipality	28493	2000	151.5	138.1	166.4
Uganda	Moroto	Moroto Municipality	28493	2005	123.6	111.7	136.0
Uganda	Moroto	Moroto Municipality	28493	2010	96.0	86.2	106.5
Uganda	Moroto	Moroto Municipality	28493	2015	79.1	68.2	90.8
Uganda	Moyo	Obongi	28496	2000	145.9	134.3	157.8
Uganda	Moyo	Obongi	28496	2005	122.7	113.6	133.1
Uganda	Moyo	Obongi	28496	2010	101.5	92.9	110.7
Uganda	Moyo	Obongi	28496	2015	78.6	69.8	88.2
Uganda	Moyo	West Moyo	28497	2000	141.7	130.4	153.9
Uganda	Moyo	West Moyo	28497	2005	120.6	110.9	130.9
Uganda	Moyo	West Moyo	28497	2010	102.2	92.9	112.5
Uganda	Moyo	West Moyo	28497	2015	80.2	70.6	90.8
Uganda	Mpigi	Mawokota	28503	2000	106.1	99.4	114.1
Uganda	Mpigi	Mawokota	28503	2005	94.9	88.5	101.7
Uganda	Mpigi	Mawokota	28503	2010	80.8	75.1	87.5
Uganda	Mpigi	Mawokota	28503	2015	57.9	52.3	64.7
Uganda	Mubende	Buwekula	28505	2000	142.0	130.9	153.3
Uganda	Mubende	Buwekula	28505	2005	119.2	110.7	129.0
Uganda	Mubende	Buwekula	28505	2010	97.8	89.8	106.6
Uganda	Mubende	Buwekula	28505	2015	76.2	68.3	85.4
Uganda	Mubende	Kassanda	28506	2000	123.8	114.0	134.2
Uganda	Mubende	Kassanda	28506	2005	104.0	96.0	112.1
Uganda	Mubende	Kassanda	28506	2010	86.4	79.2	94.0
Uganda	Mubende	Kassanda	28506	2015	67.2	60.0	75.3
Uganda	Mukono	Mukono	28511	2000	106.7	101.0	112.7
Uganda	Mukono	Mukono	28511	2005	95.6	90.4	100.8
Uganda	Mukono	Mukono	28511	2010	80.6	75.4	85.7
Uganda	Mukono	Mukono	28511	2015	55.7	50.0	61.4
Uganda	Mukono	Nakifuma	28512	2000	109.4	103.3	115.7
Uganda	Mukono	Nakifuma	28512	2005	96.6	91.2	102.4
Uganda	Mukono	Nakifuma	28512	2010	80.9	75.6	86.7
Uganda	Mukono	Nakifuma	28512	2015	55.5	50.0	61.5
Uganda	Nakapiripirit	Kadani (chekwii)	28491	2000	140.8	128.2	153.5
Uganda	Nakapiripirit	Kadani (chekwii)	28491	2005	114.4	104.5	125.7
Uganda	Nakapiripirit	Kadani (chekwii)	28491	2010	87.5	78.5	97.6
Uganda	Nakapiripirit	Kadani (chekwii)	28491	2015	71.4	62.5	81.0

Admin 0	Admin 1	Admin 2	GAUL Code	Year	Under-5 mortality (per 1,000 livebirths)		
					Estimate	Lower bound, 95% UI	Upper bound, 95% UI
Uganda	Nakapiripirit	Pian	28494	2000	152.9	139.9	166.5
Uganda	Nakapiripirit	Pian	28494	2005	126.0	115.2	137.9
Uganda	Nakapiripirit	Pian	28494	2010	99.8	90.3	110.7
Uganda	Nakapiripirit	Pian	28494	2015	79.1	69.3	90.0
Uganda	Nakaseke	Nakaseke	28466	2000	111.0	103.7	119.1
Uganda	Nakaseke	Nakaseke	28466	2005	94.2	87.3	101.3
Uganda	Nakaseke	Nakaseke	28466	2010	78.9	72.7	86.1
Uganda	Nakaseke	Nakaseke	28466	2015	58.8	52.9	65.8
Uganda	Nakasongola	Buruli	42209	2000	111.9	102.7	122.7
Uganda	Nakasongola	Buruli	42209	2005	94.2	87.0	102.2
Uganda	Nakasongola	Buruli	42209	2010	82.0	75.1	89.3
Uganda	Nakasongola	Buruli	42209	2015	60.9	54.0	68.5
Uganda	Namayingo	Bukooli	149625	2000	177.0	166.7	187.8
Uganda	Namayingo	Bukooli	149625	2005	143.4	134.6	153.1
Uganda	Namayingo	Bukooli	149625	2010	113.3	105.2	121.7
Uganda	Namayingo	Bukooli	149625	2015	77.4	69.0	85.6
Uganda	Namutumba	Busiki	28412	2000	137.0	128.9	145.5
Uganda	Namutumba	Busiki	28412	2005	115.2	108.7	122.2
Uganda	Namutumba	Busiki	28412	2010	92.6	86.8	98.8
Uganda	Namutumba	Busiki	28412	2015	71.6	65.1	79.4
Uganda	Napak	Bokora	28490	2000	157.3	145.2	169.2
Uganda	Napak	Bokora	28490	2005	129.5	119.0	140.1
Uganda	Napak	Bokora	28490	2010	105.0	95.7	114.7
Uganda	Napak	Bokora	28490	2015	84.3	74.1	95.3
Uganda	Nebbi	Jonam	28514	2000	169.4	155.2	184.5
Uganda	Nebbi	Jonam	28514	2005	144.4	132.5	157.0
Uganda	Nebbi	Jonam	28514	2010	110.3	100.1	121.2
Uganda	Nebbi	Jonam	28514	2015	87.6	78.3	99.5
Uganda	Nebbi	Padyere	28516	2000	165.7	154.3	177.6
Uganda	Nebbi	Padyere	28516	2005	141.1	131.5	151.8
Uganda	Nebbi	Padyere	28516	2010	106.4	98.1	116.2
Uganda	Nebbi	Padyere	28516	2015	83.4	74.9	93.1
Uganda	Ngora	Ngora	28458	2000	116.6	109.6	124.0
Uganda	Ngora	Ngora	28458	2005	97.7	91.9	104.3
Uganda	Ngora	Ngora	28458	2010	79.5	74.1	85.1
Uganda	Ngora	Ngora	28458	2015	64.4	58.3	71.0
Uganda	Ntoroko	Ntoroko	28396	2000	159.9	146.9	174.3
Uganda	Ntoroko	Ntoroko	28396	2005	133.4	122.0	144.2
Uganda	Ntoroko	Ntoroko	28396	2010	100.2	90.9	109.7
Uganda	Ntoroko	Ntoroko	28396	2015	76.9	68.0	86.2
Uganda	Ntungamo	Kajara	28517	2000	144.3	134.8	153.9
Uganda	Ntungamo	Kajara	28517	2005	111.9	104.0	120.3
Uganda	Ntungamo	Kajara	28517	2010	75.6	69.7	81.9
Uganda	Ntungamo	Kajara	28517	2015	58.0	52.0	64.9
Uganda	Ntungamo	Ruhaama	28518	2000	158.7	148.5	168.7
Uganda	Ntungamo	Ruhaama	28518	2005	122.7	114.6	131.1
Uganda	Ntungamo	Ruhaama	28518	2010	82.1	75.9	88.8
Uganda	Ntungamo	Ruhaama	28518	2015	60.3	54.1	67.0
Uganda	Ntungamo	Rushenyi	28519	2000	150.3	142.0	158.7
Uganda	Ntungamo	Rushenyi	28519	2005	113.9	107.2	120.8
Uganda	Ntungamo	Rushenyi	28519	2010	76.8	71.2	82.8
Uganda	Ntungamo	Rushenyi	28519	2015	57.2	51.5	63.6
Uganda	Nwoya	Nwoya	28406	2000	161.6	148.7	175.5
Uganda	Nwoya	Nwoya	28406	2005	137.0	126.1	148.9
Uganda	Nwoya	Nwoya	28406	2010	109.9	100.1	119.6
Uganda	Nwoya	Nwoya	28406	2015	89.7	79.7	101.4
Uganda	Otuke	Otuke	28464	2000	151.0	139.8	163.0
Uganda	Otuke	Otuke	28464	2005	129.5	120.2	140.3
Uganda	Otuke	Otuke	28464	2010	104.2	95.9	112.6
Uganda	Otuke	Otuke	28464	2015	82.4	73.4	91.9
Uganda	Oyam	Oyam	28385	2000	147.2	137.9	156.8
Uganda	Oyam	Oyam	28385	2005	123.9	116.3	132.2
Uganda	Oyam	Oyam	28385	2010	101.2	94.1	109.1
Uganda	Oyam	Oyam	28385	2015	83.0	74.3	92.8
Uganda	Pader	Aruu	28450	2000	165.5	152.7	179.4
Uganda	Pader	Aruu	28450	2005	138.8	128.1	150.2
Uganda	Pader	Aruu	28450	2010	111.2	102.2	121.2
Uganda	Pader	Aruu	28450	2015	89.8	80.0	100.9
Uganda	Pallisa	Butebo	28521	2000	118.9	111.6	126.1
Uganda	Pallisa	Butebo	28521	2005	98.7	92.7	105.0
Uganda	Pallisa	Butebo	28521	2010	80.8	75.2	86.9
Uganda	Pallisa	Butebo	28521	2015	65.3	59.0	72.5
Uganda	Pallisa	Pallisa	28523	2000	121.4	113.3	129.1
Uganda	Pallisa	Pallisa	28523	2005	101.7	95.2	108.1
Uganda	Pallisa	Pallisa	28523	2010	83.1	77.1	89.3
Uganda	Pallisa	Pallisa	28523	2015	66.6	59.8	74.0
Uganda	Rakai	Kakuuto	28525	2000	162.9	150.9	175.5
Uganda	Rakai	Kakuuto	28525	2005	132.2	122.2	142.7
Uganda	Rakai	Kakuuto	28525	2010	106.0	97.1	115.8
Uganda	Rakai	Kakuuto	28525	2015	72.2	64.6	81.8
Uganda	Rakai	Kooki	28526	2000	169.9	157.6	183.1
Uganda	Rakai	Kooki	28526	2005	137.3	127.9	148.3
Uganda	Rakai	Kooki	28526	2010	108.8	100.4	118.8
Uganda	Rakai	Kooki	28526	2015	75.3	67.3	84.8
Uganda	Rakai	Kyotera	28527	2000	153.9	143.2	165.4

Admin 0	Admin 1	Admin 2	GAUL Code	Year	Under-5 mortality (per 1,000 livebirths) Estimate	Lower bound, 95% UI	Upper bound, 95% UI
Uganda	Rakai	Kyotera	28527	2005	124.3	115.2	133.5
Uganda	Rakai	Kyotera	28527	2010	102.4	94.2	111.5
Uganda	Rakai	Kyotera	28527	2015	71.7	64.4	80.3
Uganda	Rubirizi	Bunyaruguru	28398	2000	147.2	136.5	157.5
Uganda	Rubirizi	Bunyaruguru	28398	2005	119.9	110.9	128.8
Uganda	Rubirizi	Bunyaruguru	28398	2010	86.3	79.2	94.6
Uganda	Rubirizi	Bunyaruguru	28398	2015	65.3	58.6	72.5
Uganda	Rukungiri	Rubabo	28529	2000	138.5	129.8	147.3
Uganda	Rukungiri	Rubabo	28529	2005	105.4	98.7	112.4
Uganda	Rukungiri	Rubabo	28529	2010	72.0	66.7	77.7
Uganda	Rukungiri	Rubabo	28529	2015	54.0	48.4	60.4
Uganda	Rukungiri	Rujumbura	28530	2000	138.1	128.5	148.5
Uganda	Rukungiri	Rujumbura	28530	2005	108.9	100.9	117.4
Uganda	Rukungiri	Rujumbura	28530	2010	75.0	69.0	81.2
Uganda	Rukungiri	Rujumbura	28530	2015	57.6	51.5	64.4
Uganda	Serere	Kasilo	28535	2000	120.4	111.7	129.0
Uganda	Serere	Kasilo	28535	2005	100.6	93.4	107.9
Uganda	Serere	Kasilo	28535	2010	82.1	75.9	88.7
Uganda	Serere	Kasilo	28535	2015	65.4	58.8	72.4
Uganda	Serere	Serere	28536	2000	116.8	108.9	124.7
Uganda	Serere	Serere	28536	2005	97.8	91.6	105.1
Uganda	Serere	Serere	28536	2010	79.6	73.6	85.8
Uganda	Serere	Serere	28536	2015	64.8	58.4	71.2
Uganda	Sheema	Sheema	28401	2000	151.1	142.6	160.0
Uganda	Sheema	Sheema	28401	2005	119.3	112.1	126.9
Uganda	Sheema	Sheema	28401	2010	82.7	76.7	89.3
Uganda	Sheema	Sheema	28401	2015	63.3	56.8	70.3
Uganda	Sironko	Budadiri	28477	2000	111.6	105.1	117.9
Uganda	Sironko	Budadiri	28477	2005	90.6	84.7	96.4
Uganda	Sironko	Budadiri	28477	2010	75.1	69.4	80.6
Uganda	Sironko	Budadiri	28477	2015	59.0	53.6	65.6
Uganda	Soroti	Soroti	28537	2000	118.4	109.8	126.5
Uganda	Soroti	Soroti	28537	2005	98.9	92.4	106.0
Uganda	Soroti	Soroti	28537	2010	81.1	75.2	87.3
Uganda	Soroti	Soroti	28537	2015	65.7	59.2	72.7
Uganda	Soroti	Soroti Municipality	28538	2000	108.4	100.0	116.6
Uganda	Soroti	Soroti Municipality	28538	2005	90.8	84.2	98.1
Uganda	Soroti	Soroti Municipality	28538	2010	77.3	71.1	83.6
Uganda	Soroti	Soroti Municipality	28538	2015	61.0	54.8	68.0
Uganda	Ssembabule	Lwemiyaga	28531	2000	160.3	147.2	175.2
Uganda	Ssembabule	Lwemiyaga	28531	2005	132.7	121.7	144.9
Uganda	Ssembabule	Lwemiyaga	28531	2010	110.4	100.3	121.8
Uganda	Ssembabule	Lwemiyaga	28531	2015	81.1	72.4	92.0
Uganda	Ssembabule	Mawogola	28532	2000	156.6	145.9	167.7
Uganda	Ssembabule	Mawogola	28532	2005	130.0	121.5	140.2
Uganda	Ssembabule	Mawogola	28532	2010	110.0	101.7	119.5
Uganda	Ssembabule	Mawogola	28532	2015	76.9	68.8	86.1
Uganda	Tororo	Kisoko (west Budama)	28540	2000	146.8	138.6	155.2
Uganda	Tororo	Kisoko (west Budama)	28540	2005	119.2	112.3	126.1
Uganda	Tororo	Kisoko (west Budama)	28540	2010	93.5	87.7	99.8
Uganda	Tororo	Kisoko (west Budama)	28540	2015	72.2	65.2	79.5
Uganda	Tororo	Tororo	28541	2000	136.7	130.0	143.5
Uganda	Tororo	Tororo	28541	2005	110.6	104.9	116.4
Uganda	Tororo	Tororo	28541	2010	89.1	83.7	94.8
Uganda	Tororo	Tororo	28541	2015	66.6	60.5	73.0
Uganda	Tororo	Tororo Municipality	28542	2000	131.8	125.5	138.5
Uganda	Tororo	Tororo Municipality	28542	2005	109.3	103.7	115.3
Uganda	Tororo	Tororo Municipality	28542	2010	87.7	82.3	93.7
Uganda	Tororo	Tororo Municipality	28542	2015	65.9	59.9	72.4
Uganda	Wakiso	Busiro	42210	2000	101.4	96.4	106.8
Uganda	Wakiso	Busiro	42210	2005	90.1	85.5	94.9
Uganda	Wakiso	Busiro	42210	2010	77.5	73.0	82.6
Uganda	Wakiso	Busiro	42210	2015	54.6	49.5	60.4
Uganda	Wakiso	Entebbe Municipality	42211	2000	100.7	93.5	108.9
Uganda	Wakiso	Entebbe Municipality	42211	2005	88.6	82.0	95.5
Uganda	Wakiso	Entebbe Municipality	42211	2010	77.1	70.6	84.1
Uganda	Wakiso	Entebbe Municipality	42211	2015	53.7	47.9	60.2
Uganda	Wakiso	Kyadondo	42212	2000	91.5	86.9	96.4
Uganda	Wakiso	Kyadondo	42212	2005	83.7	79.4	88.1
Uganda	Wakiso	Kyadondo	42212	2010	74.7	70.3	79.4
Uganda	Wakiso	Kyadondo	42212	2015	52.7	47.5	58.2
Uganda	Yumbe	Aringa	28386	2000	153.2	143.2	163.5
Uganda	Yumbe	Aringa	28386	2005	129.6	121.2	138.9
Uganda	Yumbe	Aringa	28386	2010	105.2	96.9	114.1
Uganda	Yumbe	Aringa	28386	2015	83.9	74.6	94.5
Uganda	Zombo	Okoro	28515	2000	166.2	154.1	178.9
Uganda	Zombo	Okoro	28515	2005	139.8	129.7	151.0
Uganda	Zombo	Okoro	28515	2010	106.1	97.2	115.3
Uganda	Zombo	Okoro	28515	2015	83.2	74.2	93.7
United Republic of Tanzania	Arusha	Arusha	115082	2000	56.5	51.2	61.9
United Republic of Tanzania	Arusha	Arusha	115082	2005	50.2	45.4	55.4
United Republic of Tanzania	Arusha	Arusha	115082	2010	44.0	39.3	49.1
United Republic of Tanzania	Arusha	Arusha	115082	2015	36.6	31.8	42.1
United Republic of Tanzania	Arusha	Arusha Urban	48384	2000	54.5	49.2	60.0
United Republic of Tanzania	Arusha	Arusha Urban	48384	2005	49.2	44.2	54.5

Admin 0	Admin 1	Admin 2	GAUL Code	Year	Under-5 mortality (per 1,000 livebirths)		
					Estimate	Lower bound, 95% UI	Upper bound, 95% UI
United Republic of Tanzania	Arusha	Arusha Urban	48384	2010	43.7	38.9	48.9
United Republic of Tanzania	Arusha	Arusha Urban	48384	2015	36.1	31.3	41.7
United Republic of Tanzania	Arusha	Karatu	115068	2000	73.3	66.5	80.9
United Republic of Tanzania	Arusha	Karatu	115068	2005	58.0	52.2	64.4
United Republic of Tanzania	Arusha	Karatu	115068	2010	48.5	43.1	54.0
United Republic of Tanzania	Arusha	Karatu	115068	2015	40.8	35.5	46.9
United Republic of Tanzania	Arusha	Longido	115121	2000	76.2	68.5	84.0
United Republic of Tanzania	Arusha	Longido	115121	2005	55.8	50.4	61.6
United Republic of Tanzania	Arusha	Longido	115121	2010	45.1	40.4	50.2
United Republic of Tanzania	Arusha	Longido	115121	2015	38.3	33.3	43.6
United Republic of Tanzania	Arusha	Meru	115047	2000	59.0	53.6	64.5
United Republic of Tanzania	Arusha	Meru	115047	2005	52.1	47.7	56.9
United Republic of Tanzania	Arusha	Meru	115047	2010	45.4	40.7	50.2
United Republic of Tanzania	Arusha	Meru	115047	2015	37.4	32.7	42.6
United Republic of Tanzania	Arusha	Monduli	115037	2000	74.6	67.3	81.9
United Republic of Tanzania	Arusha	Monduli	115037	2005	56.7	50.9	62.4
United Republic of Tanzania	Arusha	Monduli	115037	2010	47.8	42.7	52.9
United Republic of Tanzania	Arusha	Monduli	115037	2015	40.3	34.8	45.8
United Republic of Tanzania	Arusha	Ngorongoro	190510	2000	88.4	79.7	97.6
United Republic of Tanzania	Arusha	Ngorongoro	190510	2005	66.0	59.6	72.8
United Republic of Tanzania	Arusha	Ngorongoro	190510	2010	50.1	44.7	55.8
United Republic of Tanzania	Arusha	Ngorongoro	190510	2015	41.5	36.3	47.0
United Republic of Tanzania	Dar-es-salaam	Ilala	48388	2000	98.8	91.7	105.9
United Republic of Tanzania	Dar-es-salaam	Ilala	48388	2005	91.0	83.9	98.0
United Republic of Tanzania	Dar-es-salaam	Ilala	48388	2010	76.0	70.2	82.3
United Republic of Tanzania	Dar-es-salaam	Ilala	48388	2015	60.0	53.4	67.0
United Republic of Tanzania	Dar-es-salaam	Kinondoni	48389	2000	96.1	89.2	103.1
United Republic of Tanzania	Dar-es-salaam	Kinondoni	48389	2005	87.3	80.8	93.8
United Republic of Tanzania	Dar-es-salaam	Kinondoni	48389	2010	73.7	67.8	79.6
United Republic of Tanzania	Dar-es-salaam	Kinondoni	48389	2015	58.8	52.4	65.5
United Republic of Tanzania	Dar-es-salaam	Temeke	48390	2000	103.2	95.4	110.8
United Republic of Tanzania	Dar-es-salaam	Temeke	48390	2005	92.2	85.4	100.0
United Republic of Tanzania	Dar-es-salaam	Temeke	48390	2010	77.1	71.2	84.1
United Republic of Tanzania	Dar-es-salaam	Temeke	48390	2015	60.1	53.4	67.3
United Republic of Tanzania	Dodoma	Bahi	115083	2000	150.1	137.0	164.4
United Republic of Tanzania	Dodoma	Bahi	115083	2005	115.7	104.6	126.4
United Republic of Tanzania	Dodoma	Bahi	115083	2010	90.8	82.1	100.4
United Republic of Tanzania	Dodoma	Bahi	115083	2015	71.2	62.5	80.8
United Republic of Tanzania	Dodoma	Chamwino	115066	2000	148.9	137.3	160.7
United Republic of Tanzania	Dodoma	Chamwino	115066	2005	115.8	106.8	124.7
United Republic of Tanzania	Dodoma	Chamwino	115066	2010	90.5	82.5	98.6
United Republic of Tanzania	Dodoma	Chamwino	115066	2015	70.4	62.4	79.0
United Republic of Tanzania	Dodoma	Chemba	115095	2000	121.1	108.8	133.4
United Republic of Tanzania	Dodoma	Chemba	115095	2005	94.3	85.3	103.9
United Republic of Tanzania	Dodoma	Chemba	115095	2010	74.5	66.8	82.6
United Republic of Tanzania	Dodoma	Chemba	115095	2015	58.1	50.9	65.3
United Republic of Tanzania	Dodoma	Dodoma Urban	48392	2000	130.1	117.9	143.4
United Republic of Tanzania	Dodoma	Dodoma Urban	48392	2005	108.5	97.8	119.6
United Republic of Tanzania	Dodoma	Dodoma Urban	48392	2010	86.6	77.4	96.7
United Republic of Tanzania	Dodoma	Dodoma Urban	48392	2015	67.8	58.9	77.4
United Republic of Tanzania	Dodoma	Kondoa	115036	2000	94.8	85.1	105.1
United Republic of Tanzania	Dodoma	Kondoa	115036	2005	76.4	69.0	84.9
United Republic of Tanzania	Dodoma	Kondoa	115036	2010	62.1	55.3	69.5
United Republic of Tanzania	Dodoma	Kondoa	115036	2015	49.6	43.0	56.9
United Republic of Tanzania	Dodoma	Kongwa	48394	2000	137.1	125.1	150.0
United Republic of Tanzania	Dodoma	Kongwa	48394	2005	105.1	95.0	115.1
United Republic of Tanzania	Dodoma	Kongwa	48394	2010	78.5	70.0	86.9
United Republic of Tanzania	Dodoma	Kongwa	48394	2015	63.6	55.4	72.4
United Republic of Tanzania	Dodoma	Mpwapwa	48395	2000	141.4	127.8	156.6
United Republic of Tanzania	Dodoma	Mpwapwa	48395	2005	110.3	99.7	121.7
United Republic of Tanzania	Dodoma	Mpwapwa	48395	2010	86.5	77.7	96.5
United Republic of Tanzania	Dodoma	Mpwapwa	48395	2015	68.6	60.1	78.3
United Republic of Tanzania	Geita	Bukombe	115065	2000	128.3	117.2	140.7
United Republic of Tanzania	Geita	Bukombe	115065	2005	104.1	95.3	113.7
United Republic of Tanzania	Geita	Bukombe	115065	2010	83.6	75.3	92.3
United Republic of Tanzania	Geita	Bukombe	115065	2015	70.2	62.1	78.9
United Republic of Tanzania	Geita	Chato	115076	2000	126.4	116.3	137.1
United Republic of Tanzania	Geita	Chato	115076	2005	102.3	94.4	110.3
United Republic of Tanzania	Geita	Chato	115076	2010	78.8	72.1	86.0
United Republic of Tanzania	Geita	Chato	115076	2015	61.5	54.7	68.7
United Republic of Tanzania	Geita	Geita	115030	2000	124.7	115.2	134.8
United Republic of Tanzania	Geita	Geita	115030	2005	102.7	94.8	111.0
United Republic of Tanzania	Geita	Geita	115030	2010	80.4	73.7	87.0
United Republic of Tanzania	Geita	Geita	115030	2015	62.9	56.2	70.2
United Republic of Tanzania	Geita	Mbogwe	115059	2000	128.0	116.8	139.8
United Republic of Tanzania	Geita	Mbogwe	115059	2005	107.0	97.5	117.4
United Republic of Tanzania	Geita	Mbogwe	115059	2010	85.7	77.9	94.5
United Republic of Tanzania	Geita	Mbogwe	115059	2015	68.7	60.6	77.0
United Republic of Tanzania	Geita	Nyang'wale	115051	2000	124.8	114.1	135.4
United Republic of Tanzania	Geita	Nyang'wale	115051	2005	104.0	95.4	113.1
United Republic of Tanzania	Geita	Nyang'wale	115051	2010	82.8	75.5	90.4
United Republic of Tanzania	Geita	Nyang'wale	115051	2015	63.3	56.0	71.4
United Republic of Tanzania	Iringa	Iringa Rural	115032	2000	128.8	117.4	141.4
United Republic of Tanzania	Iringa	Iringa Rural	115032	2005	98.3	89.2	107.9
United Republic of Tanzania	Iringa	Iringa Rural	115032	2010	80.6	72.4	89.4

Admin 0	Admin 1	Admin 2	GAUL Code	Year	Under-5 mortality (per 1,000 livebirths)		
					Estimate	Lower bound, 95% UI	Upper bound, 95% UI
United Republic of Tanzania	Iringa	Iringa Rural	115032	2015	62.5	54.5	70.8
United Republic of Tanzania	Iringa	Iringa Urban	48397	2000	99.7	89.9	110.1
United Republic of Tanzania	Iringa	Iringa Urban	48397	2005	87.9	79.7	97.6
United Republic of Tanzania	Iringa	Iringa Urban	48397	2010	73.7	65.8	82.7
United Republic of Tanzania	Iringa	Iringa Urban	48397	2015	58.6	50.6	67.4
United Republic of Tanzania	Iringa	Kilolo	115069	2000	124.9	113.3	136.5
United Republic of Tanzania	Iringa	Kilolo	115069	2005	100.6	91.3	110.7
United Republic of Tanzania	Iringa	Kilolo	115069	2010	83.6	75.2	92.4
United Republic of Tanzania	Iringa	Kilolo	115069	2015	63.9	55.3	72.0
United Republic of Tanzania	Iringa	Mafinga Township Authority	115079	2000	117.5	105.0	131.6
United Republic of Tanzania	Iringa	Mafinga Township Authority	115079	2005	91.2	81.7	101.5
United Republic of Tanzania	Iringa	Mafinga Township Authority	115079	2010	75.7	66.8	85.0
United Republic of Tanzania	Iringa	Mafinga Township Authority	115079	2015	57.2	49.1	65.9
United Republic of Tanzania	Iringa	Mufindi	115048	2000	122.5	111.3	134.3
United Republic of Tanzania	Iringa	Mufindi	115048	2005	94.8	86.4	103.9
United Republic of Tanzania	Iringa	Mufindi	115048	2010	78.3	70.7	86.1
United Republic of Tanzania	Iringa	Mufindi	115048	2015	59.1	51.9	67.3
United Republic of Tanzania	Kagera	Biharamulo	115064	2000	146.2	134.8	158.3
United Republic of Tanzania	Kagera	Biharamulo	115064	2005	112.2	103.6	121.1
United Republic of Tanzania	Kagera	Biharamulo	115064	2010	84.9	77.9	92.7
United Republic of Tanzania	Kagera	Biharamulo	115064	2015	67.5	60.4	75.1
United Republic of Tanzania	Kagera	Bukoba Rural	48404	2000	134.8	123.7	146.3
United Republic of Tanzania	Kagera	Bukoba Rural	48404	2005	107.4	98.5	117.1
United Republic of Tanzania	Kagera	Bukoba Rural	48404	2010	80.5	73.5	88.6
United Republic of Tanzania	Kagera	Bukoba Rural	48404	2015	61.1	54.1	68.8
United Republic of Tanzania	Kagera	Bukoba Urban	48405	2000	113.4	102.0	125.6
United Republic of Tanzania	Kagera	Bukoba Urban	48405	2005	93.1	84.2	102.8
United Republic of Tanzania	Kagera	Bukoba Urban	48405	2010	72.1	64.9	80.7
United Republic of Tanzania	Kagera	Bukoba Urban	48405	2015	57.5	50.2	66.0
United Republic of Tanzania	Kagera	Karagwe	115034	2000	156.8	145.7	169.0
United Republic of Tanzania	Kagera	Karagwe	115034	2005	120.7	112.4	129.4
United Republic of Tanzania	Kagera	Karagwe	115034	2010	85.6	79.2	92.5
United Republic of Tanzania	Kagera	Karagwe	115034	2015	63.2	56.9	70.1
United Republic of Tanzania	Kagera	Kyerwa	115107	2000	174.5	162.4	187.4
United Republic of Tanzania	Kagera	Kyerwa	115107	2005	136.4	126.8	147.1
United Republic of Tanzania	Kagera	Kyerwa	115107	2010	92.4	85.4	100.2
United Republic of Tanzania	Kagera	Kyerwa	115107	2015	66.2	59.0	74.0
United Republic of Tanzania	Kagera	Missenyi	115102	2000	147.3	136.0	159.3
United Republic of Tanzania	Kagera	Missenyi	115102	2005	120.8	111.5	130.5
United Republic of Tanzania	Kagera	Missenyi	115102	2010	90.4	83.1	99.0
United Republic of Tanzania	Kagera	Missenyi	115102	2015	65.7	58.6	74.2
United Republic of Tanzania	Kagera	Muleba	48407	2000	133.5	122.4	145.6
United Republic of Tanzania	Kagera	Muleba	48407	2005	103.9	95.3	113.5
United Republic of Tanzania	Kagera	Muleba	48407	2010	77.1	70.3	84.9
United Republic of Tanzania	Kagera	Muleba	48407	2015	59.3	52.3	66.6
United Republic of Tanzania	Kagera	Ngara	48408	2000	198.4	186.4	210.9
United Republic of Tanzania	Kagera	Ngara	48408	2005	142.6	133.8	152.0
United Republic of Tanzania	Kagera	Ngara	48408	2010	103.6	96.5	111.2
United Republic of Tanzania	Kagera	Ngara	48408	2015	84.5	76.5	92.5
United Republic of Tanzania	Kaskazini Pemba	Micheweni	48462	2000	116.0	107.2	125.6
United Republic of Tanzania	Kaskazini Pemba	Micheweni	48462	2005	82.9	75.5	90.4
United Republic of Tanzania	Kaskazini Pemba	Micheweni	48462	2010	58.0	52.4	64.3
United Republic of Tanzania	Kaskazini Pemba	Micheweni	48462	2015	50.1	43.6	56.6
United Republic of Tanzania	Kaskazini Pemba	Wete	48463	2000	110.2	102.7	118.1
United Republic of Tanzania	Kaskazini Pemba	Wete	48463	2005	77.8	71.8	83.8
United Republic of Tanzania	Kaskazini Pemba	Wete	48463	2010	56.0	51.0	61.3
United Republic of Tanzania	Kaskazini Pemba	Wete	48463	2015	47.7	42.1	53.6
United Republic of Tanzania	Kaskazini Unguja	Kaskazini A	48507	2000	105.8	98.1	114.3
United Republic of Tanzania	Kaskazini Unguja	Kaskazini A	48507	2005	71.8	66.1	78.0
United Republic of Tanzania	Kaskazini Unguja	Kaskazini A	48507	2010	59.7	54.4	65.2
United Republic of Tanzania	Kaskazini Unguja	Kaskazini A	48507	2015	48.9	43.1	55.0
United Republic of Tanzania	Kaskazini Unguja	Kaskazini B	48508	2000	100.6	94.1	107.7
United Republic of Tanzania	Kaskazini Unguja	Kaskazini B	48508	2005	70.8	65.7	76.0
United Republic of Tanzania	Kaskazini Unguja	Kaskazini B	48508	2010	59.3	54.4	64.1
United Republic of Tanzania	Kaskazini Unguja	Kaskazini B	48508	2015	48.5	43.1	54.0
United Republic of Tanzania	Katavi	Mlele	115117	2000	136.8	125.3	148.0
United Republic of Tanzania	Katavi	Mlele	115117	2005	113.0	103.6	122.5
United Republic of Tanzania	Katavi	Mlele	115117	2010	84.2	76.6	92.6
United Republic of Tanzania	Katavi	Mlele	115117	2015	67.8	60.5	75.9
United Republic of Tanzania	Katavi	Mpanda Rural	115116	2000	146.1	132.1	162.0
United Republic of Tanzania	Katavi	Mpanda Rural	115116	2005	117.8	105.8	130.3
United Republic of Tanzania	Katavi	Mpanda Rural	115116	2010	90.5	81.1	101.1
United Republic of Tanzania	Katavi	Mpanda Rural	115116	2015	77.4	68.5	88.0
United Republic of Tanzania	Katavi	Mpanda Urban	115118	2000	132.5	118.9	147.1
United Republic of Tanzania	Katavi	Mpanda Urban	115118	2005	109.3	97.7	122.3
United Republic of Tanzania	Katavi	Mpanda Urban	115118	2010	80.6	71.3	90.9
United Republic of Tanzania	Katavi	Mpanda Urban	115118	2015	67.1	58.8	77.9
United Republic of Tanzania	Kigoma	Buhigwe	115084	2000	124.5	115.6	133.7
United Republic of Tanzania	Kigoma	Buhigwe	115084	2005	97.4	90.5	104.7
United Republic of Tanzania	Kigoma	Buhigwe	115084	2010	73.9	68.1	79.7
United Republic of Tanzania	Kigoma	Buhigwe	115084	2015	60.5	53.9	67.5
United Republic of Tanzania	Kigoma	Kakonko	115096	2000	141.2	130.3	151.8
United Republic of Tanzania	Kigoma	Kakonko	115096	2005	109.9	101.7	118.5
United Republic of Tanzania	Kigoma	Kakonko	115096	2010	86.7	79.4	93.8
United Republic of Tanzania	Kigoma	Kakonko	115096	2015	71.2	63.6	78.8

Admin 0	Admin 1	Admin 2	GAUL Code	Year	Under-5 mortality (per 1,000 livebirths) Estimate	Lower bound, 95% UI	Upper bound, 95% UI
United Republic of Tanzania	Kigoma	Kasulu	115041	2000	120.6	110.8	131.5
United Republic of Tanzania	Kigoma	Kasulu	115041	2005	96.7	88.9	104.8
United Republic of Tanzania	Kigoma	Kasulu	115041	2010	75.6	69.4	83.0
United Republic of Tanzania	Kigoma	Kasulu	115041	2015	63.6	56.5	71.3
United Republic of Tanzania	Kigoma	Kasulu Township Authority	115106	2000	117.4	106.5	129.3
United Republic of Tanzania	Kigoma	Kasulu Township Authority	115106	2005	93.2	84.4	102.4
United Republic of Tanzania	Kigoma	Kasulu Township Authority	115106	2010	71.7	64.7	79.9
United Republic of Tanzania	Kigoma	Kasulu Township Authority	115106	2015	59.4	52.0	67.7
United Republic of Tanzania	Kigoma	Kibondo	115035	2000	126.3	115.9	136.6
United Republic of Tanzania	Kigoma	Kibondo	115035	2005	100.4	92.7	108.6
United Republic of Tanzania	Kigoma	Kibondo	115035	2010	81.6	75.0	88.8
United Republic of Tanzania	Kigoma	Kibondo	115035	2015	69.2	62.5	77.1
United Republic of Tanzania	Kigoma	Kigoma Municipal-Ujiji	115112	2000	117.1	105.5	130.8
United Republic of Tanzania	Kigoma	Kigoma Municipal-Ujiji	115112	2005	101.6	91.4	112.7
United Republic of Tanzania	Kigoma	Kigoma Municipal-Ujiji	115112	2010	78.0	69.6	86.9
United Republic of Tanzania	Kigoma	Kigoma Municipal-Ujiji	115112	2015	62.1	54.0	70.7
United Republic of Tanzania	Kigoma	Kigoma Rural	115055	2000	132.6	121.0	145.3
United Republic of Tanzania	Kigoma	Kigoma Rural	115055	2005	106.1	97.2	115.9
United Republic of Tanzania	Kigoma	Kigoma Rural	115055	2010	80.9	73.1	88.5
United Republic of Tanzania	Kigoma	Kigoma Rural	115055	2015	65.5	58.0	73.9
United Republic of Tanzania	Kigoma	Uvinza	115081	2000	137.8	126.1	151.5
United Republic of Tanzania	Kigoma	Uvinza	115081	2005	113.2	103.3	124.4
United Republic of Tanzania	Kigoma	Uvinza	115081	2010	87.9	79.4	97.0
United Republic of Tanzania	Kigoma	Uvinza	115081	2015	74.4	66.2	83.6
United Republic of Tanzania	Kilimanjaro	Hai	115077	2000	55.9	50.7	61.2
United Republic of Tanzania	Kilimanjaro	Hai	115077	2005	50.7	46.3	55.5
United Republic of Tanzania	Kilimanjaro	Hai	115077	2010	45.0	40.2	50.2
United Republic of Tanzania	Kilimanjaro	Hai	115077	2015	36.3	31.4	41.5
United Republic of Tanzania	Kilimanjaro	Moshi	48414	2000	55.3	50.8	60.1
United Republic of Tanzania	Kilimanjaro	Moshi	48414	2005	50.8	46.4	55.5
United Republic of Tanzania	Kilimanjaro	Moshi	48414	2010	45.2	40.6	50.1
United Republic of Tanzania	Kilimanjaro	Moshi	48414	2015	36.2	31.6	41.5
United Republic of Tanzania	Kilimanjaro	Moshi Municipal	48415	2000	50.3	46.0	54.9
United Republic of Tanzania	Kilimanjaro	Moshi Municipal	48415	2005	48.1	43.8	53.0
United Republic of Tanzania	Kilimanjaro	Moshi Municipal	48415	2010	43.1	38.5	48.0
United Republic of Tanzania	Kilimanjaro	Moshi Municipal	48415	2015	34.7	30.1	39.7
United Republic of Tanzania	Kilimanjaro	Mwanga	190511	2000	60.1	54.0	66.9
United Republic of Tanzania	Kilimanjaro	Mwanga	190511	2005	56.3	50.6	62.8
United Republic of Tanzania	Kilimanjaro	Mwanga	190511	2010	49.5	44.1	55.6
United Republic of Tanzania	Kilimanjaro	Mwanga	190511	2015	40.3	34.9	46.2
United Republic of Tanzania	Kilimanjaro	Rombo	48417	2000	62.2	57.0	67.4
United Republic of Tanzania	Kilimanjaro	Rombo	48417	2005	55.2	50.3	60.2
United Republic of Tanzania	Kilimanjaro	Rombo	48417	2010	47.8	43.0	53.1
United Republic of Tanzania	Kilimanjaro	Rombo	48417	2015	38.8	33.7	44.2
United Republic of Tanzania	Kilimanjaro	Same	48418	2000	71.1	64.0	79.1
United Republic of Tanzania	Kilimanjaro	Same	48418	2005	65.4	58.6	72.7
United Republic of Tanzania	Kilimanjaro	Same	48418	2010	56.5	50.2	63.6
United Republic of Tanzania	Kilimanjaro	Same	48418	2015	47.0	40.5	53.6
United Republic of Tanzania	Kilimanjaro	Siha	115104	2000	57.6	52.0	63.0
United Republic of Tanzania	Kilimanjaro	Siha	115104	2005	51.4	46.5	56.3
United Republic of Tanzania	Kilimanjaro	Siha	115104	2010	44.7	39.8	49.5
United Republic of Tanzania	Kilimanjaro	Siha	115104	2015	36.8	31.7	42.0
United Republic of Tanzania	Kusini Pemba	Chake Chake	48464	2000	103.2	96.0	111.0
United Republic of Tanzania	Kusini Pemba	Chake Chake	48464	2005	71.5	66.1	77.2
United Republic of Tanzania	Kusini Pemba	Chake Chake	48464	2010	53.9	48.9	59.0
United Republic of Tanzania	Kusini Pemba	Chake Chake	48464	2015	46.1	40.5	52.1
United Republic of Tanzania	Kusini Pemba	Mkoani	48465	2000	107.0	98.9	115.6
United Republic of Tanzania	Kusini Pemba	Mkoani	48465	2005	71.3	65.5	77.4
United Republic of Tanzania	Kusini Pemba	Mkoani	48465	2010	54.4	49.2	59.7
United Republic of Tanzania	Kusini Pemba	Mkoani	48465	2015	46.9	41.2	53.6
United Republic of Tanzania	Kusini Unguja	Kati	48509	2000	95.8	89.4	102.2
United Republic of Tanzania	Kusini Unguja	Kati	48509	2005	69.6	64.6	74.6
United Republic of Tanzania	Kusini Unguja	Kati	48509	2010	60.9	56.1	66.2
United Republic of Tanzania	Kusini Unguja	Kati	48509	2015	49.0	43.6	54.6
United Republic of Tanzania	Kusini Unguja	Kusini	48510	2000	90.5	82.7	98.1
United Republic of Tanzania	Kusini Unguja	Kusini	48510	2005	70.4	64.2	76.9
United Republic of Tanzania	Kusini Unguja	Kusini	48510	2010	63.4	57.5	70.0
United Republic of Tanzania	Kusini Unguja	Kusini	48510	2015	50.3	44.1	56.9
United Republic of Tanzania	Lindi	Kilwa	48419	2000	163.0	145.4	182.6
United Republic of Tanzania	Lindi	Kilwa	48419	2005	124.4	110.3	139.5
United Republic of Tanzania	Lindi	Kilwa	48419	2010	87.5	76.6	98.8
United Republic of Tanzania	Lindi	Kilwa	48419	2015	66.4	57.3	76.2
United Republic of Tanzania	Lindi	Lindi Rural	48420	2000	153.8	141.6	167.0
United Republic of Tanzania	Lindi	Lindi Rural	48420	2005	127.4	117.2	137.9
United Republic of Tanzania	Lindi	Lindi Rural	48420	2010	86.5	78.3	94.5
United Republic of Tanzania	Lindi	Lindi Rural	48420	2015	65.3	57.2	73.8
United Republic of Tanzania	Lindi	Lindi Urban	48421	2000	145.2	130.8	161.3
United Republic of Tanzania	Lindi	Lindi Urban	48421	2005	119.5	107.7	132.6
United Republic of Tanzania	Lindi	Lindi Urban	48421	2010	81.7	72.7	91.3
United Republic of Tanzania	Lindi	Lindi Urban	48421	2015	63.4	54.6	72.9
United Republic of Tanzania	Lindi	Liwale	48422	2000	165.9	148.1	186.2
United Republic of Tanzania	Lindi	Liwale	48422	2005	134.3	119.7	150.9
United Republic of Tanzania	Lindi	Liwale	48422	2010	95.4	83.6	107.8
United Republic of Tanzania	Lindi	Liwale	48422	2015	69.8	60.4	80.4
United Republic of Tanzania	Lindi	Nachingwea	48423	2000	152.2	139.6	165.3

Admin 0	Admin 1	Admin 2	GAUL Code	Year	Under-5 mortality (per 1,000 livebirths)		
					Estimate	Lower bound, 95% UI	Upper bound, 95% UI
United Republic of Tanzania	Lindi	Nachingwea	48423	2005	127.0	115.4	139.9
United Republic of Tanzania	Lindi	Nachingwea	48423	2010	90.0	80.9	100.3
United Republic of Tanzania	Lindi	Nachingwea	48423	2015	63.1	54.6	71.5
United Republic of Tanzania	Lindi	Ruangwa	48424	2000	153.9	141.4	168.8
United Republic of Tanzania	Lindi	Ruangwa	48424	2005	127.7	115.9	140.1
United Republic of Tanzania	Lindi	Ruangwa	48424	2010	89.4	80.3	99.8
United Republic of Tanzania	Lindi	Ruangwa	48424	2015	64.4	56.0	73.3
United Republic of Tanzania	Manyara	Babati	190512	2000	74.0	67.3	81.1
United Republic of Tanzania	Manyara	Babati	190512	2005	59.7	54.4	65.6
United Republic of Tanzania	Manyara	Babati	190512	2010	50.3	45.1	55.9
United Republic of Tanzania	Manyara	Babati	190512	2015	42.4	37.2	48.4
United Republic of Tanzania	Manyara	Babati Urban	115120	2000	70.6	63.1	78.5
United Republic of Tanzania	Manyara	Babati Urban	115120	2005	59.1	53.1	66.1
United Republic of Tanzania	Manyara	Babati Urban	115120	2010	49.8	44.0	56.1
United Republic of Tanzania	Manyara	Babati Urban	115120	2015	41.4	35.7	48.0
United Republic of Tanzania	Manyara	Hanang	190513	2000	88.9	81.1	96.6
United Republic of Tanzania	Manyara	Hanang	190513	2005	69.9	63.7	76.2
United Republic of Tanzania	Manyara	Hanang	190513	2010	55.9	50.7	61.7
United Republic of Tanzania	Manyara	Hanang	190513	2015	46.3	40.9	52.5
United Republic of Tanzania	Manyara	Kiteto	48427	2000	124.8	112.4	136.7
United Republic of Tanzania	Manyara	Kiteto	48427	2005	95.0	86.5	104.6
United Republic of Tanzania	Manyara	Kiteto	48427	2010	70.7	62.4	78.6
United Republic of Tanzania	Manyara	Kiteto	48427	2015	57.3	50.2	65.1
United Republic of Tanzania	Manyara	Mbulu	115119	2000	73.3	66.9	80.1
United Republic of Tanzania	Manyara	Mbulu	115119	2005	59.5	54.0	65.8
United Republic of Tanzania	Manyara	Mbulu	115119	2010	49.1	43.9	54.5
United Republic of Tanzania	Manyara	Mbulu	115119	2015	41.3	35.9	47.3
United Republic of Tanzania	Manyara	Simanjiro	48429	2000	78.3	70.9	86.3
United Republic of Tanzania	Manyara	Simanjiro	48429	2005	64.1	58.4	70.6
United Republic of Tanzania	Manyara	Simanjiro	48429	2010	53.3	48.0	59.2
United Republic of Tanzania	Manyara	Simanjiro	48429	2015	44.6	39.2	50.8
United Republic of Tanzania	Mara	Bunda	48430	2000	150.0	138.3	162.1
United Republic of Tanzania	Mara	Bunda	48430	2005	120.3	110.8	130.5
United Republic of Tanzania	Mara	Bunda	48430	2010	87.7	80.1	95.8
United Republic of Tanzania	Mara	Bunda	48430	2015	68.8	60.9	77.7
United Republic of Tanzania	Mara	Butiama	115094	2000	159.2	147.7	171.3
United Republic of Tanzania	Mara	Butiama	115094	2005	131.8	121.9	141.8
United Republic of Tanzania	Mara	Butiama	115094	2010	97.7	89.5	106.6
United Republic of Tanzania	Mara	Butiama	115094	2015	72.3	64.8	80.5
United Republic of Tanzania	Mara	Musoma	115061	2000	148.3	135.6	160.7
United Republic of Tanzania	Mara	Musoma	115061	2005	120.9	109.6	132.5
United Republic of Tanzania	Mara	Musoma	115061	2010	89.9	81.6	99.4
United Republic of Tanzania	Mara	Musoma	115061	2015	69.1	60.8	77.9
United Republic of Tanzania	Mara	Musoma Municipal	48432	2000	141.3	128.9	154.5
United Republic of Tanzania	Mara	Musoma Municipal	48432	2005	122.8	112.2	133.8
United Republic of Tanzania	Mara	Musoma Municipal	48432	2010	93.8	84.5	103.0
United Republic of Tanzania	Mara	Musoma Municipal	48432	2015	69.7	61.7	78.3
United Republic of Tanzania	Mara	Rorya	115093	2000	200.8	187.1	215.8
United Republic of Tanzania	Mara	Rorya	115093	2005	167.5	155.1	180.6
United Republic of Tanzania	Mara	Rorya	115093	2010	125.2	114.8	136.0
United Republic of Tanzania	Mara	Rorya	115093	2015	89.3	80.2	99.2
United Republic of Tanzania	Mara	Serengeti	48433	2000	129.8	119.3	141.4
United Republic of Tanzania	Mara	Serengeti	48433	2005	107.7	98.7	117.6
United Republic of Tanzania	Mara	Serengeti	48433	2010	77.0	70.2	84.4
United Republic of Tanzania	Mara	Serengeti	48433	2015	60.8	54.2	68.0
United Republic of Tanzania	Mara	Tarime	115039	2000	164.3	154.1	175.4
United Republic of Tanzania	Mara	Tarime	115039	2005	136.0	126.4	145.9
United Republic of Tanzania	Mara	Tarime	115039	2010	98.4	91.0	106.6
United Republic of Tanzania	Mara	Tarime	115039	2015	74.8	67.1	82.8
United Republic of Tanzania	Mbeya	Chunya	48435	2000	148.0	132.5	164.8
United Republic of Tanzania	Mbeya	Chunya	48435	2005	117.1	104.4	130.0
United Republic of Tanzania	Mbeya	Chunya	48435	2010	84.5	75.4	94.7
United Republic of Tanzania	Mbeya	Chunya	48435	2015	69.8	60.7	79.8
United Republic of Tanzania	Mbeya	Ileje	48436	2000	117.5	109.4	125.6
United Republic of Tanzania	Mbeya	Ileje	48436	2005	92.5	86.4	99.0
United Republic of Tanzania	Mbeya	Ileje	48436	2010	73.9	68.2	80.3
United Republic of Tanzania	Mbeya	Ileje	48436	2015	59.5	53.3	66.6
United Republic of Tanzania	Mbeya	Kyela	48437	2000	113.6	105.7	122.2
United Republic of Tanzania	Mbeya	Kyela	48437	2005	91.5	84.8	98.5
United Republic of Tanzania	Mbeya	Kyela	48437	2010	71.1	65.4	77.0
United Republic of Tanzania	Mbeya	Kyela	48437	2015	55.4	49.7	62.0
United Republic of Tanzania	Mbeya	Mbarali	115101	2000	136.7	122.3	151.7
United Republic of Tanzania	Mbeya	Mbarali	115101	2005	105.9	95.2	116.5
United Republic of Tanzania	Mbeya	Mbarali	115101	2010	82.0	73.8	92.2
United Republic of Tanzania	Mbeya	Mbarali	115101	2015	65.1	56.8	73.7
United Republic of Tanzania	Mbeya	Mbeya Rural	115046	2000	118.8	110.1	128.2
United Republic of Tanzania	Mbeya	Mbeya Rural	115046	2005	98.0	90.4	106.0
United Republic of Tanzania	Mbeya	Mbeya Rural	115046	2010	77.2	70.7	84.5
United Republic of Tanzania	Mbeya	Mbeya Rural	115046	2015	62.3	54.9	69.8
United Republic of Tanzania	Mbeya	Mbeya Urban	48440	2000	106.6	97.2	116.4
United Republic of Tanzania	Mbeya	Mbeya Urban	48440	2005	92.0	83.6	100.8
United Republic of Tanzania	Mbeya	Mbeya Urban	48440	2010	75.4	68.1	83.2
United Republic of Tanzania	Mbeya	Mbeya Urban	48440	2015	60.8	53.1	69.2
United Republic of Tanzania	Mbeya	Mbozi	115088	2000	139.3	127.7	151.1
United Republic of Tanzania	Mbeya	Mbozi	115088	2005	106.0	97.9	115.3

Admin 0	Admin 1	Admin 2	GAUL Code	Year	Under-5 mortality (per 1,000 livebirths)		
					Estimate	Lower bound, 95% UI	Upper bound, 95% UI
United Republic of Tanzania	Mbeya	Mbozi	115088	2010	81.8	74.3	90.0
United Republic of Tanzania	Mbeya	Mbozi	115088	2015	67.0	59.2	75.9
United Republic of Tanzania	Mbeya	Momba	115109	2000	153.6	139.9	167.5
United Republic of Tanzania	Mbeya	Momba	115109	2005	119.5	109.3	130.3
United Republic of Tanzania	Mbeya	Momba	115109	2010	89.8	81.2	99.0
United Republic of Tanzania	Mbeya	Momba	115109	2015	74.2	65.3	84.0
United Republic of Tanzania	Mbeya	Rungwe	48442	2000	122.1	112.8	131.9
United Republic of Tanzania	Mbeya	Rungwe	48442	2005	99.3	91.5	107.8
United Republic of Tanzania	Mbeya	Rungwe	48442	2010	77.9	71.0	84.9
United Republic of Tanzania	Mbeya	Rungwe	48442	2015	60.8	53.6	68.0
United Republic of Tanzania	Mbeya	Tunduma	115111	2000	138.0	124.9	152.0
United Republic of Tanzania	Mbeya	Tunduma	115111	2005	102.8	93.4	112.9
United Republic of Tanzania	Mbeya	Tunduma	115111	2010	77.0	69.3	85.3
United Republic of Tanzania	Mbeya	Tunduma	115111	2015	64.7	56.2	73.7
United Republic of Tanzania	Mjini Magharibi	Magharibi	48512	2000	86.7	80.8	92.9
United Republic of Tanzania	Mjini Magharibi	Magharibi	48512	2005	65.6	60.7	70.6
United Republic of Tanzania	Mjini Magharibi	Magharibi	48512	2010	59.3	54.5	64.1
United Republic of Tanzania	Mjini Magharibi	Magharibi	48512	2015	47.5	42.1	53.3
United Republic of Tanzania	Mjini Magharibi	Mjini	48511	2000	84.0	78.2	90.3
United Republic of Tanzania	Mjini Magharibi	Mjini	48511	2005	64.3	59.5	69.3
United Republic of Tanzania	Mjini Magharibi	Mjini	48511	2010	58.7	53.9	63.5
United Republic of Tanzania	Mjini Magharibi	Mjini	48511	2015	46.7	41.3	52.7
United Republic of Tanzania	Morogoro	Gairo	115115	2000	129.6	118.1	142.5
United Republic of Tanzania	Morogoro	Gairo	115115	2005	99.3	90.1	109.8
United Republic of Tanzania	Morogoro	Gairo	115115	2010	73.3	65.7	81.3
United Republic of Tanzania	Morogoro	Gairo	115115	2015	60.6	53.5	69.4
United Republic of Tanzania	Morogoro	Kilombero	48443	2000	131.5	120.1	143.9
United Republic of Tanzania	Morogoro	Kilombero	48443	2005	104.1	94.8	113.7
United Republic of Tanzania	Morogoro	Kilombero	48443	2010	84.1	75.9	92.9
United Republic of Tanzania	Morogoro	Kilombero	48443	2015	63.4	56.1	71.3
United Republic of Tanzania	Morogoro	Kilosa	115114	2000	138.4	126.9	151.4
United Republic of Tanzania	Morogoro	Kilosa	115114	2005	107.9	98.7	117.8
United Republic of Tanzania	Morogoro	Kilosa	115114	2010	82.7	75.3	91.4
United Republic of Tanzania	Morogoro	Kilosa	115114	2015	65.7	58.4	74.5
United Republic of Tanzania	Morogoro	Morogoro	115113	2000	142.1	129.6	155.1
United Republic of Tanzania	Morogoro	Morogoro	115113	2005	112.7	102.2	122.5
United Republic of Tanzania	Morogoro	Morogoro	115113	2010	88.1	80.0	97.0
United Republic of Tanzania	Morogoro	Morogoro	115113	2015	69.5	61.5	78.4
United Republic of Tanzania	Morogoro	Morogoro Urban	48446	2000	116.7	105.8	129.6
United Republic of Tanzania	Morogoro	Morogoro Urban	48446	2005	95.7	86.5	106.4
United Republic of Tanzania	Morogoro	Morogoro Urban	48446	2010	75.7	67.6	84.3
United Republic of Tanzania	Morogoro	Morogoro Urban	48446	2015	61.1	53.3	70.0
United Republic of Tanzania	Morogoro	Mvomero	115090	2000	137.0	126.2	149.3
United Republic of Tanzania	Morogoro	Mvomero	115090	2005	107.1	97.9	117.1
United Republic of Tanzania	Morogoro	Mvomero	115090	2010	80.6	73.4	88.7
United Republic of Tanzania	Morogoro	Mvomero	115090	2015	65.5	58.3	73.9
United Republic of Tanzania	Morogoro	Ulanga	48448	2000	144.5	129.1	162.8
United Republic of Tanzania	Morogoro	Ulanga	48448	2005	119.1	106.0	134.4
United Republic of Tanzania	Morogoro	Ulanga	48448	2010	93.6	82.7	106.1
United Republic of Tanzania	Morogoro	Ulanga	48448	2015	68.2	59.3	78.4
United Republic of Tanzania	Mtwara	Masasi	115057	2000	150.6	139.4	162.1
United Republic of Tanzania	Mtwara	Masasi	115057	2005	124.2	114.6	135.0
United Republic of Tanzania	Mtwara	Masasi	115057	2010	86.1	78.1	95.1
United Republic of Tanzania	Mtwara	Masasi	115057	2015	62.1	54.2	70.3
United Republic of Tanzania	Mtwara	Masasi Township Authority	115100	2000	145.7	132.0	159.7
United Republic of Tanzania	Mtwara	Masasi Township Authority	115100	2005	119.6	107.9	132.5
United Republic of Tanzania	Mtwara	Masasi Township Authority	115100	2010	83.7	74.7	93.8
United Republic of Tanzania	Mtwara	Masasi Township Authority	115100	2015	60.1	51.8	68.5
United Republic of Tanzania	Mtwara	Mtwara Rural	48450	2000	153.9	141.0	167.7
United Republic of Tanzania	Mtwara	Mtwara Rural	48450	2005	125.3	113.5	137.2
United Republic of Tanzania	Mtwara	Mtwara Rural	48450	2010	82.8	74.1	92.1
United Republic of Tanzania	Mtwara	Mtwara Rural	48450	2015	66.4	57.9	76.5
United Republic of Tanzania	Mtwara	Mtwara Urban	48451	2000	128.2	113.2	142.7
United Republic of Tanzania	Mtwara	Mtwara Urban	48451	2005	103.5	91.1	116.3
United Republic of Tanzania	Mtwara	Mtwara Urban	48451	2010	71.9	63.3	81.9
United Republic of Tanzania	Mtwara	Mtwara Urban	48451	2015	59.2	50.4	69.4
United Republic of Tanzania	Mtwara	Nanyumbu	115091	2000	154.8	139.4	170.7
United Republic of Tanzania	Mtwara	Nanyumbu	115091	2005	128.9	115.6	142.5
United Republic of Tanzania	Mtwara	Nanyumbu	115091	2010	88.9	78.6	99.7
United Republic of Tanzania	Mtwara	Nanyumbu	115091	2015	63.4	54.5	72.7
United Republic of Tanzania	Mtwara	Newala	115050	2000	150.7	138.4	163.6
United Republic of Tanzania	Mtwara	Newala	115050	2005	125.3	114.8	135.8
United Republic of Tanzania	Mtwara	Newala	115050	2010	85.7	77.2	94.4
United Republic of Tanzania	Mtwara	Newala	115050	2015	62.7	54.7	71.9
United Republic of Tanzania	Mtwara	Tandahimba	115073	2000	155.4	142.2	169.3
United Republic of Tanzania	Mtwara	Tandahimba	115073	2005	127.9	117.1	139.6
United Republic of Tanzania	Mtwara	Tandahimba	115073	2010	84.5	76.0	93.7
United Republic of Tanzania	Mtwara	Tandahimba	115073	2015	64.4	56.3	73.7
United Republic of Tanzania	Mwanza	Administrative unit not available	484720002	2000	121.9	112.5	131.7
United Republic of Tanzania	Mwanza	Administrative unit not available	484720002	2005	100.9	92.9	109.5
United Republic of Tanzania	Mwanza	Administrative unit not available	484720002	2010	80.0	73.3	87.1
United Republic of Tanzania	Mwanza	Administrative unit not available	484720002	2015	59.3	52.8	65.8
United Republic of Tanzania	Mwanza	Ilemela	115086	2000	107.1	97.5	116.3
United Republic of Tanzania	Mwanza	Ilemela	115086	2005	91.0	82.8	100.2
United Republic of Tanzania	Mwanza	Ilemela	115086	2010	74.4	67.4	81.7

Admin 0	Admin 1	Admin 2	GAUL Code	Year	Under-5 mortality (per 1,000 livebirths)		
					Estimate	Lower bound, 95% UI	Upper bound, 95% UI
United Republic of Tanzania	Mwanza	Ilemela	115086	2015	56.1	49.0	63.2
United Republic of Tanzania	Mwanza	Kwimba	115070	2000	127.6	118.0	137.1
United Republic of Tanzania	Mwanza	Kwimba	115070	2005	102.3	94.9	110.5
United Republic of Tanzania	Mwanza	Kwimba	115070	2010	80.2	73.7	86.7
United Republic of Tanzania	Mwanza	Kwimba	115070	2015	59.7	53.2	66.4
United Republic of Tanzania	Mwanza	Magu	115045	2000	130.4	120.7	139.9
United Republic of Tanzania	Mwanza	Magu	115045	2005	104.7	96.7	112.6
United Republic of Tanzania	Mwanza	Magu	115045	2010	82.2	75.3	89.0
United Republic of Tanzania	Mwanza	Magu	115045	2015	62.4	55.3	70.1
United Republic of Tanzania	Mwanza	Misungwi	115103	2000	121.1	111.1	131.0
United Republic of Tanzania	Mwanza	Misungwi	115103	2005	99.3	90.9	107.7
United Republic of Tanzania	Mwanza	Misungwi	115103	2010	80.5	73.8	87.4
United Republic of Tanzania	Mwanza	Misungwi	115103	2015	60.8	53.8	68.3
United Republic of Tanzania	Mwanza	Nyamagana	115062	2000	101.8	92.7	110.5
United Republic of Tanzania	Mwanza	Nyamagana	115062	2005	87.9	79.9	96.1
United Republic of Tanzania	Mwanza	Nyamagana	115062	2010	72.6	65.9	79.7
United Republic of Tanzania	Mwanza	Nyamagana	115062	2015	54.3	47.6	61.2
United Republic of Tanzania	Mwanza	Sengerema	48460	2000	119.4	109.8	129.0
United Republic of Tanzania	Mwanza	Sengerema	48460	2005	97.2	89.7	105.2
United Republic of Tanzania	Mwanza	Sengerema	48460	2010	78.6	71.9	86.1
United Republic of Tanzania	Mwanza	Sengerema	48460	2015	61.4	54.4	68.2
United Republic of Tanzania	Mwanza	Ukerewe	48461	2000	131.5	117.2	146.7
United Republic of Tanzania	Mwanza	Ukerewe	48461	2005	109.6	98.1	122.7
United Republic of Tanzania	Mwanza	Ukerewe	48461	2010	84.2	74.7	95.3
United Republic of Tanzania	Mwanza	Ukerewe	48461	2015	64.6	56.5	74.2
United Republic of Tanzania	Njombe	Ludewa	48399	2000	110.1	99.7	120.6
United Republic of Tanzania	Njombe	Ludewa	48399	2005	90.1	81.6	99.3
United Republic of Tanzania	Njombe	Ludewa	48399	2010	72.2	64.9	80.1
United Republic of Tanzania	Njombe	Ludewa	48399	2015	54.1	47.4	61.0
United Republic of Tanzania	Njombe	Makambako Township Authority	115087	2000	116.0	105.5	129.0
United Republic of Tanzania	Njombe	Makambako Township Authority	115087	2005	92.7	84.0	102.7
United Republic of Tanzania	Njombe	Makambako Township Authority	115087	2010	75.2	67.1	84.1
United Republic of Tanzania	Njombe	Makambako Township Authority	115087	2015	57.4	49.5	65.7
United Republic of Tanzania	Njombe	Makete	48400	2000	120.7	110.7	131.3
United Republic of Tanzania	Njombe	Makete	48400	2005	99.4	91.1	108.2
United Republic of Tanzania	Njombe	Makete	48400	2010	77.7	70.4	85.2
United Republic of Tanzania	Njombe	Makete	48400	2015	59.9	52.9	67.1
United Republic of Tanzania	Njombe	Njombe Rural	115072	2000	112.4	102.0	122.7
United Republic of Tanzania	Njombe	Njombe Rural	115072	2005	95.1	86.0	105.0
United Republic of Tanzania	Njombe	Njombe Rural	115072	2010	77.2	69.1	85.9
United Republic of Tanzania	Njombe	Njombe Rural	115072	2015	57.0	49.6	65.3
United Republic of Tanzania	Njombe	Njombe Urban	115038	2000	108.0	97.3	118.1
United Republic of Tanzania	Njombe	Njombe Urban	115038	2005	92.0	82.9	101.7
United Republic of Tanzania	Njombe	Njombe Urban	115038	2010	73.8	66.1	81.5
United Republic of Tanzania	Njombe	Njombe Urban	115038	2015	54.8	48.3	62.6
United Republic of Tanzania	Njombe	Wanging'ombe	115054	2000	120.5	109.7	132.4
United Republic of Tanzania	Njombe	Wanging'ombe	115054	2005	96.5	88.0	105.7
United Republic of Tanzania	Njombe	Wanging'ombe	115054	2010	76.9	69.6	85.0
United Republic of Tanzania	Njombe	Wanging'ombe	115054	2015	58.5	51.2	66.1
United Republic of Tanzania	Pwani	Bagamoyo	48466	2000	134.8	124.1	147.0
United Republic of Tanzania	Pwani	Bagamoyo	48466	2005	108.2	99.5	117.8
United Republic of Tanzania	Pwani	Bagamoyo	48466	2010	82.3	75.3	89.9
United Republic of Tanzania	Pwani	Bagamoyo	48466	2015	64.0	57.2	71.9
United Republic of Tanzania	Pwani	Kibaha	115042	2000	128.3	117.1	139.3
United Republic of Tanzania	Pwani	Kibaha	115042	2005	103.6	94.6	112.7
United Republic of Tanzania	Pwani	Kibaha	115042	2010	81.5	74.7	89.4
United Republic of Tanzania	Pwani	Kibaha	115042	2015	62.7	55.6	71.5
United Republic of Tanzania	Pwani	Kibaha Urban	115098	2000	118.0	109.3	127.9
United Republic of Tanzania	Pwani	Kibaha Urban	115098	2005	96.6	88.8	104.7
United Republic of Tanzania	Pwani	Kibaha Urban	115098	2010	77.4	71.2	84.0
United Republic of Tanzania	Pwani	Kibaha Urban	115098	2015	59.3	52.8	67.3
United Republic of Tanzania	Pwani	Kisarawe	115056	2000	131.6	121.1	142.6
United Republic of Tanzania	Pwani	Kisarawe	115056	2005	104.9	96.3	113.4
United Republic of Tanzania	Pwani	Kisarawe	115056	2010	84.6	77.5	92.3
United Republic of Tanzania	Pwani	Kisarawe	115056	2015	64.3	57.4	72.9
United Republic of Tanzania	Pwani	Mafia	48469	2000	123.4	104.4	142.9
United Republic of Tanzania	Pwani	Mafia	48469	2005	93.1	78.8	107.9
United Republic of Tanzania	Pwani	Mafia	48469	2010	79.5	67.1	92.3
United Republic of Tanzania	Pwani	Mafia	48469	2015	64.3	53.6	76.0
United Republic of Tanzania	Pwani	Mkuranga	115071	2000	145.0	133.3	157.7
United Republic of Tanzania	Pwani	Mkuranga	115071	2005	112.4	102.9	122.0
United Republic of Tanzania	Pwani	Mkuranga	115071	2010	86.7	78.8	95.4
United Republic of Tanzania	Pwani	Mkuranga	115071	2015	66.4	58.8	74.9
United Republic of Tanzania	Pwani	Rufiji	48471	2000	150.2	134.7	166.7
United Republic of Tanzania	Pwani	Rufiji	48471	2005	109.2	98.0	121.5
United Republic of Tanzania	Pwani	Rufiji	48471	2010	85.6	76.0	96.1
United Republic of Tanzania	Pwani	Rufiji	48471	2015	67.3	58.9	76.8
United Republic of Tanzania	Rukwa	Kalambo	115033	2000	172.4	158.6	186.7
United Republic of Tanzania	Rukwa	Kalambo	115033	2005	132.4	122.2	143.6
United Republic of Tanzania	Rukwa	Kalambo	115033	2010	97.7	89.0	107.0
United Republic of Tanzania	Rukwa	Kalambo	115033	2015	79.2	70.1	89.5
United Republic of Tanzania	Rukwa	Nkasi	48474	2000	158.0	143.5	172.4
United Republic of Tanzania	Rukwa	Nkasi	48474	2005	128.3	117.5	141.1
United Republic of Tanzania	Rukwa	Nkasi	48474	2010	94.7	85.8	104.3
United Republic of Tanzania	Rukwa	Nkasi	48474	2015	74.9	65.9	85.3

Admin 0	Admin 1	Admin 2	GAUL Code	Year	Under-5 mortality (per 1,000 livebirths)		
					Estimate	Lower bound, 95% UI	Upper bound, 95% UI
United Republic of Tanzania	Rukwa	Sumbawanga Rural	115053	2000	156.5	144.0	170.3
United Republic of Tanzania	Rukwa	Sumbawanga Rural	115053	2005	122.5	112.7	133.0
United Republic of Tanzania	Rukwa	Sumbawanga Rural	115053	2010	91.5	83.0	100.4
United Republic of Tanzania	Rukwa	Sumbawanga Rural	115053	2015	73.1	64.7	82.5
United Republic of Tanzania	Rukwa	Sumbawanga Urban	48476	2000	136.5	123.2	150.2
United Republic of Tanzania	Rukwa	Sumbawanga Urban	48476	2005	109.7	99.9	120.3
United Republic of Tanzania	Rukwa	Sumbawanga Urban	48476	2010	86.3	77.7	96.4
United Republic of Tanzania	Rukwa	Sumbawanga Urban	48476	2015	68.8	59.9	79.5
United Republic of Tanzania	Ruvuma	Mbinga	115058	2000	111.3	101.8	121.6
United Republic of Tanzania	Ruvuma	Mbinga	115058	2005	92.3	84.4	100.5
United Republic of Tanzania	Ruvuma	Mbinga	115058	2010	71.2	64.8	78.8
United Republic of Tanzania	Ruvuma	Mbinga	115058	2015	50.9	44.7	57.3
United Republic of Tanzania	Ruvuma	Namtumbo	48478	2000	152.3	136.6	167.6
United Republic of Tanzania	Ruvuma	Namtumbo	48478	2005	127.1	114.0	140.6
United Republic of Tanzania	Ruvuma	Namtumbo	48478	2010	93.6	83.4	104.8
United Republic of Tanzania	Ruvuma	Namtumbo	48478	2015	66.1	57.7	76.3
United Republic of Tanzania	Ruvuma	Nyasa	115092	2000	110.4	100.7	120.8
United Republic of Tanzania	Ruvuma	Nyasa	115092	2005	91.9	83.8	100.4
United Republic of Tanzania	Ruvuma	Nyasa	115092	2010	72.1	65.6	79.9
United Republic of Tanzania	Ruvuma	Nyasa	115092	2015	51.1	45.2	57.4
United Republic of Tanzania	Ruvuma	Songea Rural	48479	2000	124.5	113.9	135.4
United Republic of Tanzania	Ruvuma	Songea Rural	48479	2005	106.1	96.5	115.9
United Republic of Tanzania	Ruvuma	Songea Rural	48479	2010	81.0	73.0	89.4
United Republic of Tanzania	Ruvuma	Songea Rural	48479	2015	57.1	50.3	65.1
United Republic of Tanzania	Ruvuma	Songea Urban	48480	2000	124.8	112.1	138.7
United Republic of Tanzania	Ruvuma	Songea Urban	48480	2005	105.4	94.4	116.8
United Republic of Tanzania	Ruvuma	Songea Urban	48480	2010	77.7	68.4	88.2
United Republic of Tanzania	Ruvuma	Songea Urban	48480	2015	57.0	49.0	66.4
United Republic of Tanzania	Ruvuma	Tunduru	48481	2000	165.7	148.6	184.1
United Republic of Tanzania	Ruvuma	Tunduru	48481	2005	139.8	125.4	155.3
United Republic of Tanzania	Ruvuma	Tunduru	48481	2010	96.8	85.5	108.4
United Republic of Tanzania	Ruvuma	Tunduru	48481	2015	68.4	58.2	78.7
United Republic of Tanzania	Shinyanga	Kahama	115067	2000	127.3	117.8	136.9
United Republic of Tanzania	Shinyanga	Kahama	115067	2005	107.4	99.7	115.8
United Republic of Tanzania	Shinyanga	Kahama	115067	2010	84.7	78.1	92.0
United Republic of Tanzania	Shinyanga	Kahama	115067	2015	64.0	56.9	71.6
United Republic of Tanzania	Shinyanga	Kahama Township Authority	115080	2000	126.2	113.9	138.4
United Republic of Tanzania	Shinyanga	Kahama Township Authority	115080	2005	104.3	95.0	114.7
United Republic of Tanzania	Shinyanga	Kahama Township Authority	115080	2010	81.8	73.9	89.8
United Republic of Tanzania	Shinyanga	Kahama Township Authority	115080	2015	61.2	53.1	69.6
United Republic of Tanzania	Shinyanga	Kishapu	115043	2000	115.0	105.8	125.1
United Republic of Tanzania	Shinyanga	Kishapu	115043	2005	96.2	88.5	104.4
United Republic of Tanzania	Shinyanga	Kishapu	115043	2010	73.6	67.1	80.5
United Republic of Tanzania	Shinyanga	Kishapu	115043	2015	55.7	49.1	62.7
United Republic of Tanzania	Shinyanga	Shinyanga Rural	115063	2000	116.0	106.4	125.4
United Republic of Tanzania	Shinyanga	Shinyanga Rural	115063	2005	96.6	89.3	104.4
United Republic of Tanzania	Shinyanga	Shinyanga Rural	115063	2010	75.9	69.2	83.0
United Republic of Tanzania	Shinyanga	Shinyanga Rural	115063	2015	56.6	50.2	63.4
United Republic of Tanzania	Shinyanga	Shinyanga Urban	48489	2000	104.2	94.5	114.2
United Republic of Tanzania	Shinyanga	Shinyanga Urban	48489	2005	89.8	81.4	98.6
United Republic of Tanzania	Shinyanga	Shinyanga Urban	48489	2010	70.1	63.3	77.3
United Republic of Tanzania	Shinyanga	Shinyanga Urban	48489	2015	53.5	46.9	60.5
United Republic of Tanzania	Simiyu	Bariadi	115029	2000	148.2	137.6	160.2
United Republic of Tanzania	Simiyu	Bariadi	115029	2005	121.9	112.7	132.3
United Republic of Tanzania	Simiyu	Bariadi	115029	2010	83.9	76.8	91.3
United Republic of Tanzania	Simiyu	Bariadi	115029	2015	64.5	57.2	72.7
United Republic of Tanzania	Simiyu	Busega	115075	2000	145.3	133.1	159.1
United Republic of Tanzania	Simiyu	Busega	115075	2005	115.7	105.9	127.1
United Republic of Tanzania	Simiyu	Busega	115075	2010	85.0	77.2	93.9
United Republic of Tanzania	Simiyu	Busega	115075	2015	65.6	57.6	74.6
United Republic of Tanzania	Simiyu	Itilima	115040	2000	137.5	127.2	149.2
United Republic of Tanzania	Simiyu	Itilima	115040	2005	114.9	105.9	124.5
United Republic of Tanzania	Simiyu	Itilima	115040	2010	79.5	72.7	86.7
United Republic of Tanzania	Simiyu	Itilima	115040	2015	60.9	53.9	68.2
United Republic of Tanzania	Simiyu	Maswa	48486	2000	128.1	118.7	139.3
United Republic of Tanzania	Simiyu	Maswa	48486	2005	107.5	99.2	116.2
United Republic of Tanzania	Simiyu	Maswa	48486	2010	79.0	72.1	86.3
United Republic of Tanzania	Simiyu	Maswa	48486	2015	58.4	51.5	65.3
United Republic of Tanzania	Simiyu	Meatu	48487	2000	125.4	114.6	137.6
United Republic of Tanzania	Simiyu	Meatu	48487	2005	104.9	95.9	115.2
United Republic of Tanzania	Simiyu	Meatu	48487	2010	75.6	68.5	82.8
United Republic of Tanzania	Simiyu	Meatu	48487	2015	57.4	50.1	65.1
United Republic of Tanzania	Singida	Ikungi	115078	2000	107.3	98.1	117.9
United Republic of Tanzania	Singida	Ikungi	115078	2005	84.3	76.7	92.7
United Republic of Tanzania	Singida	Ikungi	115078	2010	67.6	60.8	74.9
United Republic of Tanzania	Singida	Ikungi	115078	2015	52.0	45.5	58.8
United Republic of Tanzania	Singida	Iramba	190514	2000	99.9	90.5	110.3
United Republic of Tanzania	Singida	Iramba	190514	2005	79.4	71.9	87.4
United Republic of Tanzania	Singida	Iramba	190514	2010	65.1	58.6	72.1
United Republic of Tanzania	Singida	Iramba	190514	2015	50.6	44.3	57.5
United Republic of Tanzania	Singida	Manyoni	48491	2000	136.5	123.3	150.4
United Republic of Tanzania	Singida	Manyoni	48491	2005	104.5	93.9	115.1
United Republic of Tanzania	Singida	Manyoni	48491	2010	81.5	73.0	90.2
United Republic of Tanzania	Singida	Manyoni	48491	2015	63.3	55.8	71.4
United Republic of Tanzania	Singida	Mkalama	115089	2000	89.9	81.6	98.1

Admin 0	Admin 1	Admin 2	GAUL Code	Year	Under-5 mortality (per 1,000 livebirths)		
					Estimate	Lower bound, 95% UI	Upper bound, 95% UI
United Republic of Tanzania	Singida	Mkalama	115089	2005	73.9	67.0	81.2
United Republic of Tanzania	Singida	Mkalama	115089	2010	60.6	54.7	67.0
United Republic of Tanzania	Singida	Mkalama	115089	2015	48.5	42.7	55.3
United Republic of Tanzania	Singida	Singida	115052	2000	93.4	85.8	102.4
United Republic of Tanzania	Singida	Singida	115052	2005	75.6	69.0	82.4
United Republic of Tanzania	Singida	Singida	115052	2010	61.1	55.4	67.1
United Republic of Tanzania	Singida	Singida	115052	2015	48.7	42.7	54.9
United Republic of Tanzania	Singida	Singida Urban	48493	2000	87.3	79.4	96.9
United Republic of Tanzania	Singida	Singida Urban	48493	2005	72.2	65.4	79.5
United Republic of Tanzania	Singida	Singida Urban	48493	2010	59.7	53.3	66.5
United Republic of Tanzania	Singida	Singida Urban	48493	2015	47.2	40.8	53.4
United Republic of Tanzania	Tabora	Kaliua	190515	2000	122.7	107.6	140.2
United Republic of Tanzania	Tabora	Kaliua	190515	2005	102.2	89.2	116.8
United Republic of Tanzania	Tabora	Kaliua	190515	2010	83.1	71.9	94.9
United Republic of Tanzania	Tabora	Kaliua	190515	2015	69.2	58.9	81.0
United Republic of Tanzania	Tabora	Igunga	48494	2000	115.2	105.0	126.5
United Republic of Tanzania	Tabora	Igunga	48494	2005	90.1	82.5	98.2
United Republic of Tanzania	Tabora	Igunga	48494	2010	69.1	62.8	76.2
United Republic of Tanzania	Tabora	Igunga	48494	2015	52.9	46.5	60.2
United Republic of Tanzania	Tabora	Kaliua	115097	2000	127.8	116.0	139.4
United Republic of Tanzania	Tabora	Kaliua	115097	2005	105.4	95.7	116.2
United Republic of Tanzania	Tabora	Kaliua	115097	2010	83.1	75.3	91.7
United Republic of Tanzania	Tabora	Kaliua	115097	2015	64.0	56.5	72.2
United Republic of Tanzania	Tabora	Nzega	48495	2000	120.9	111.0	131.5
United Republic of Tanzania	Tabora	Nzega	48495	2005	96.8	88.7	104.8
United Republic of Tanzania	Tabora	Nzega	48495	2010	74.2	67.5	81.1
United Republic of Tanzania	Tabora	Nzega	48495	2015	55.2	48.8	62.4
United Republic of Tanzania	Tabora	Sikonge	48496	2000	128.0	114.0	143.1
United Republic of Tanzania	Tabora	Sikonge	48496	2005	102.1	90.9	114.3
United Republic of Tanzania	Tabora	Sikonge	48496	2010	77.4	67.9	87.4
United Republic of Tanzania	Tabora	Sikonge	48496	2015	58.8	50.6	67.4
United Republic of Tanzania	Tabora	Tabora Urban	48497	2000	112.1	100.4	124.9
United Republic of Tanzania	Tabora	Tabora Urban	48497	2005	95.1	84.8	105.9
United Republic of Tanzania	Tabora	Tabora Urban	48497	2010	74.6	66.4	84.5
United Republic of Tanzania	Tabora	Tabora Urban	48497	2015	56.5	48.9	65.1
United Republic of Tanzania	Tabora	Urambo	115074	2000	129.4	115.4	143.9
United Republic of Tanzania	Tabora	Urambo	115074	2005	103.1	91.7	114.4
United Republic of Tanzania	Tabora	Urambo	115074	2010	80.2	71.2	90.3
United Republic of Tanzania	Tabora	Urambo	115074	2015	60.5	52.8	69.3
United Republic of Tanzania	Tabora	Uyui	48499	2000	127.7	117.5	139.5
United Republic of Tanzania	Tabora	Uyui	48499	2005	101.5	92.7	110.4
United Republic of Tanzania	Tabora	Uyui	48499	2010	77.5	70.3	85.3
United Republic of Tanzania	Tabora	Uyui	48499	2015	57.1	50.2	63.9
United Republic of Tanzania	Tanga	Handeni	115085	2000	147.4	134.8	161.9
United Republic of Tanzania	Tanga	Handeni	115085	2005	114.2	104.7	125.5
United Republic of Tanzania	Tanga	Handeni	115085	2010	82.5	74.9	90.0
United Republic of Tanzania	Tanga	Handeni	115085	2015	65.1	57.5	73.0
United Republic of Tanzania	Tanga	Handeni Township Authority	115110	2000	136.7	122.3	153.3
United Republic of Tanzania	Tanga	Handeni Township Authority	115110	2005	102.7	91.4	113.8
United Republic of Tanzania	Tanga	Handeni Township Authority	115110	2010	74.1	65.6	83.3
United Republic of Tanzania	Tanga	Handeni Township Authority	115110	2015	61.4	53.3	70.4
United Republic of Tanzania	Tanga	Kilindi	115099	2000	133.4	120.2	147.9
United Republic of Tanzania	Tanga	Kilindi	115099	2005	100.0	90.2	110.8
United Republic of Tanzania	Tanga	Kilindi	115099	2010	72.4	65.1	80.7
United Republic of Tanzania	Tanga	Kilindi	115099	2015	60.8	53.3	70.0
United Republic of Tanzania	Tanga	Korogwe	115044	2000	111.3	102.4	121.1
United Republic of Tanzania	Tanga	Korogwe	115044	2005	88.3	81.3	96.0
United Republic of Tanzania	Tanga	Korogwe	115044	2010	67.3	61.1	74.1
United Republic of Tanzania	Tanga	Korogwe	115044	2015	56.5	50.0	63.9
United Republic of Tanzania	Tanga	Korogwe Township Authority	115108	2000	125.3	113.6	138.6
United Republic of Tanzania	Tanga	Korogwe Township Authority	115108	2005	99.9	90.6	109.7
United Republic of Tanzania	Tanga	Korogwe Township Authority	115108	2010	73.2	65.3	81.3
United Republic of Tanzania	Tanga	Korogwe Township Authority	115108	2015	58.4	51.0	66.4
United Republic of Tanzania	Tanga	Lushoto	48503	2000	108.0	99.0	118.0
United Republic of Tanzania	Tanga	Lushoto	48503	2005	85.3	77.8	93.9
United Republic of Tanzania	Tanga	Lushoto	48503	2010	66.1	59.4	73.3
United Republic of Tanzania	Tanga	Lushoto	48503	2015	55.7	48.8	63.6
United Republic of Tanzania	Tanga	Mkinga	115105	2000	120.8	111.4	130.8
United Republic of Tanzania	Tanga	Mkinga	115105	2005	100.0	91.1	109.4
United Republic of Tanzania	Tanga	Mkinga	115105	2010	70.9	64.6	77.7
United Republic of Tanzania	Tanga	Mkinga	115105	2015	57.2	50.3	64.7
United Republic of Tanzania	Tanga	Muheza	115060	2000	130.2	119.6	142.3
United Republic of Tanzania	Tanga	Muheza	115060	2005	106.7	97.7	116.7
United Republic of Tanzania	Tanga	Muheza	115060	2010	77.4	69.7	85.1
United Republic of Tanzania	Tanga	Muheza	115060	2015	59.5	52.0	66.7
United Republic of Tanzania	Tanga	Pangani	48505	2000	122.0	110.4	134.3
United Republic of Tanzania	Tanga	Pangani	48505	2005	98.5	89.3	108.5
United Republic of Tanzania	Tanga	Pangani	48505	2010	74.1	66.4	81.8
United Republic of Tanzania	Tanga	Pangani	48505	2015	56.8	49.6	64.1
United Republic of Tanzania	Tanga	Tanga	48506	2000	104.0	93.7	115.9
United Republic of Tanzania	Tanga	Tanga	48506	2005	88.4	79.3	98.5
United Republic of Tanzania	Tanga	Tanga	48506	2010	65.7	58.4	73.2
United Republic of Tanzania	Tanga	Tanga	48506	2015	53.2	45.8	60.9
Zambia	Central	Chibombo	65174	2000	140.2	130.1	149.9
Zambia	Central	Chibombo	65174	2005	110.7	102.2	119.2

Admin 0	Admin 1	Admin 2	GAUL Code	Year	Under-5 mortality (per 1,000 livebirths)		
					Estimate	Lower bound, 95% UI	Upper bound, 95% UI
Zambia	Central	Chibombo	65174	2010	76.0	69.6	82.8
Zambia	Central	Chibombo	65174	2015	59.3	52.6	66.3
Zambia	Central	Kabwe	65175	2000	124.9	112.5	137.3
Zambia	Central	Kabwe	65175	2005	96.9	86.4	106.8
Zambia	Central	Kabwe	65175	2010	67.5	60.2	75.3
Zambia	Central	Kabwe	65175	2015	53.1	46.2	60.9
Zambia	Central	Kapiri-Mposhi	65176	2000	145.6	132.2	161.4
Zambia	Central	Kapiri-Mposhi	65176	2005	115.0	103.9	127.0
Zambia	Central	Kapiri-Mposhi	65176	2010	80.4	71.8	89.5
Zambia	Central	Kapiri-Mposhi	65176	2015	60.3	52.5	69.3
Zambia	Central	Mkushi	65177	2000	154.9	141.7	169.8
Zambia	Central	Mkushi	65177	2005	129.8	118.2	143.5
Zambia	Central	Mkushi	65177	2010	95.4	85.9	106.2
Zambia	Central	Mkushi	65177	2015	68.6	60.4	77.8
Zambia	Central	Mumbwa	65178	2000	146.0	131.7	161.7
Zambia	Central	Mumbwa	65178	2005	111.8	99.7	124.2
Zambia	Central	Mumbwa	65178	2010	81.2	72.1	91.2
Zambia	Central	Mumbwa	65178	2015	63.4	54.7	72.3
Zambia	Central	Serenje	65179	2000	159.0	144.7	174.2
Zambia	Central	Serenje	65179	2005	132.5	119.8	145.5
Zambia	Central	Serenje	65179	2010	101.6	91.0	113.2
Zambia	Central	Serenje	65179	2015	76.7	67.1	87.2
Zambia	Copperbelt	Chililabombwe	65180	2000	112.6	101.9	125.2
Zambia	Copperbelt	Chililabombwe	65180	2005	93.0	83.7	103.1
Zambia	Copperbelt	Chililabombwe	65180	2010	74.5	65.8	84.0
Zambia	Copperbelt	Chililabombwe	65180	2015	57.1	49.2	66.1
Zambia	Copperbelt	Chingola	65181	2000	107.5	98.2	119.2
Zambia	Copperbelt	Chingola	65181	2005	89.8	81.4	99.1
Zambia	Copperbelt	Chingola	65181	2010	72.0	64.6	80.3
Zambia	Copperbelt	Chingola	65181	2015	54.3	46.9	62.5
Zambia	Copperbelt	Kalulushi	65182	2000	118.0	109.5	127.0
Zambia	Copperbelt	Kalulushi	65182	2005	96.2	88.4	105.2
Zambia	Copperbelt	Kalulushi	65182	2010	74.9	67.9	82.3
Zambia	Copperbelt	Kalulushi	65182	2015	54.5	47.8	61.8
Zambia	Copperbelt	Kitwe	65183	2000	106.4	98.5	114.5
Zambia	Copperbelt	Kitwe	65183	2005	90.0	83.0	98.1
Zambia	Copperbelt	Kitwe	65183	2010	72.7	66.1	80.0
Zambia	Copperbelt	Kitwe	65183	2015	53.4	46.7	60.8
Zambia	Copperbelt	Luanshya	65184	2000	115.6	106.9	126.3
Zambia	Copperbelt	Luanshya	65184	2005	98.3	90.5	106.9
Zambia	Copperbelt	Luanshya	65184	2010	74.9	67.9	82.3
Zambia	Copperbelt	Luanshya	65184	2015	54.7	48.3	61.7
Zambia	Copperbelt	Lufwanyama	65185	2000	132.4	119.8	146.6
Zambia	Copperbelt	Lufwanyama	65185	2005	108.2	97.2	120.2
Zambia	Copperbelt	Lufwanyama	65185	2010	83.7	74.4	93.7
Zambia	Copperbelt	Lufwanyama	65185	2015	59.3	51.3	67.9
Zambia	Copperbelt	Masaiti	65186	2000	137.2	126.2	148.3
Zambia	Copperbelt	Masaiti	65186	2005	115.0	106.2	125.2
Zambia	Copperbelt	Masaiti	65186	2010	86.9	79.0	95.8
Zambia	Copperbelt	Masaiti	65186	2015	61.2	54.0	68.5
Zambia	Copperbelt	Mpongwe	65187	2000	136.7	123.1	151.1
Zambia	Copperbelt	Mpongwe	65187	2005	114.1	102.5	126.1
Zambia	Copperbelt	Mpongwe	65187	2010	85.2	76.1	95.1
Zambia	Copperbelt	Mpongwe	65187	2015	61.3	53.0	70.0
Zambia	Copperbelt	Mufulira	65188	2000	104.3	95.4	114.8
Zambia	Copperbelt	Mufulira	65188	2005	90.0	81.7	99.3
Zambia	Copperbelt	Mufulira	65188	2010	73.4	66.0	81.4
Zambia	Copperbelt	Mufulira	65188	2015	54.9	48.0	62.9
Zambia	Copperbelt	Ndola	65189	2000	115.0	104.8	125.9
Zambia	Copperbelt	Ndola	65189	2005	98.3	89.9	107.3
Zambia	Copperbelt	Ndola	65189	2010	76.9	69.7	85.3
Zambia	Copperbelt	Ndola	65189	2015	56.5	49.4	63.8
Zambia	Eastern	Chadiza	65190	2000	172.2	160.5	185.7
Zambia	Eastern	Chadiza	65190	2005	135.7	125.8	146.5
Zambia	Eastern	Chadiza	65190	2010	101.3	93.0	110.4
Zambia	Eastern	Chadiza	65190	2015	72.1	64.9	80.2
Zambia	Eastern	Chama	65191	2000	170.4	156.5	185.2
Zambia	Eastern	Chama	65191	2005	129.7	119.1	141.0
Zambia	Eastern	Chama	65191	2010	86.7	78.7	95.2
Zambia	Eastern	Chama	65191	2015	64.0	57.3	72.0
Zambia	Eastern	Chipata	65192	2000	169.4	159.2	180.8
Zambia	Eastern	Chipata	65192	2005	126.9	118.6	135.7
Zambia	Eastern	Chipata	65192	2010	99.5	92.0	107.4
Zambia	Eastern	Chipata	65192	2015	73.2	66.3	80.6
Zambia	Eastern	Katete	65193	2000	174.8	161.5	190.2
Zambia	Eastern	Katete	65193	2005	136.8	125.7	149.5
Zambia	Eastern	Katete	65193	2010	104.5	95.1	114.5
Zambia	Eastern	Katete	65193	2015	81.7	72.8	92.0
Zambia	Eastern	Lundazi	65194	2000	180.0	168.3	192.8
Zambia	Eastern	Lundazi	65194	2005	138.5	128.4	149.4
Zambia	Eastern	Lundazi	65194	2010	97.9	90.4	106.7
Zambia	Eastern	Lundazi	65194	2015	70.7	63.9	78.3
Zambia	Eastern	Mambwe	65195	2000	171.6	154.6	191.1
Zambia	Eastern	Mambwe	65195	2005	133.3	119.8	148.5
Zambia	Eastern	Mambwe	65195	2010	101.0	90.0	112.3

Admin 0	Admin 1	Admin 2	GAUL Code	Year	Under-5 mortality (per 1,000 livebirths)		
					Estimate	Lower bound, 95% UI	Upper bound, 95% UI
Zambia	Eastern	Mambwe	65195	2015	79.2	69.5	89.6
Zambia	Eastern	Nyimba	65196	2000	173.4	155.5	193.1
Zambia	Eastern	Nyimba	65196	2005	141.1	125.8	157.6
Zambia	Eastern	Nyimba	65196	2010	105.6	92.9	118.7
Zambia	Eastern	Nyimba	65196	2015	85.0	73.4	97.6
Zambia	Eastern	Petauke	65197	2000	178.0	163.3	194.6
Zambia	Eastern	Petauke	65197	2005	140.4	127.3	154.2
Zambia	Eastern	Petauke	65197	2010	107.8	96.7	119.1
Zambia	Eastern	Petauke	65197	2015	86.0	75.7	96.8
Zambia	Luapula	Chienge	65198	2000	229.3	205.0	254.5
Zambia	Luapula	Chienge	65198	2005	187.5	167.7	209.5
Zambia	Luapula	Chienge	65198	2010	145.8	128.7	163.9
Zambia	Luapula	Chienge	65198	2015	116.9	101.3	134.1
Zambia	Luapula	Kawambwa	65199	2000	169.9	155.0	185.8
Zambia	Luapula	Kawambwa	65199	2005	130.0	118.8	142.3
Zambia	Luapula	Kawambwa	65199	2010	96.3	87.1	106.7
Zambia	Luapula	Kawambwa	65199	2015	72.6	64.0	82.0
Zambia	Luapula	Mansa	65200	2000	178.5	163.0	195.1
Zambia	Luapula	Mansa	65200	2005	124.1	112.8	137.0
Zambia	Luapula	Mansa	65200	2010	97.0	87.0	108.8
Zambia	Luapula	Mansa	65200	2015	70.1	61.2	79.9
Zambia	Luapula	Milenge	65201	2000	174.8	155.9	195.0
Zambia	Luapula	Milenge	65201	2005	129.3	115.6	145.2
Zambia	Luapula	Milenge	65201	2010	99.6	88.0	112.8
Zambia	Luapula	Milenge	65201	2015	76.1	65.7	87.1
Zambia	Luapula	Mwense	65202	2000	179.7	163.2	198.2
Zambia	Luapula	Mwense	65202	2005	134.3	122.2	148.6
Zambia	Luapula	Mwense	65202	2010	99.6	89.3	110.4
Zambia	Luapula	Mwense	65202	2015	74.0	65.1	83.7
Zambia	Luapula	Nchelenge	65203	2000	207.8	187.7	227.8
Zambia	Luapula	Nchelenge	65203	2005	161.2	145.4	179.0
Zambia	Luapula	Nchelenge	65203	2010	124.2	110.6	139.6
Zambia	Luapula	Nchelenge	65203	2015	95.7	82.9	109.7
Zambia	Luapula	Samfya	65204	2000	180.2	164.9	196.6
Zambia	Luapula	Samfya	65204	2005	130.9	119.6	143.7
Zambia	Luapula	Samfya	65204	2010	100.4	91.3	110.8
Zambia	Luapula	Samfya	65204	2015	73.5	65.0	82.3
Zambia	Lusaka	Chongwe	65205	2000	116.9	106.7	127.7
Zambia	Lusaka	Chongwe	65205	2005	104.0	95.0	113.6
Zambia	Lusaka	Chongwe	65205	2010	79.3	71.7	87.3
Zambia	Lusaka	Chongwe	65205	2015	58.2	51.1	65.9
Zambia	Lusaka	Kafue	65206	2000	112.0	104.3	120.2
Zambia	Lusaka	Kafue	65206	2005	99.9	92.4	107.3
Zambia	Lusaka	Kafue	65206	2010	74.4	67.9	80.5
Zambia	Lusaka	Kafue	65206	2015	56.9	50.3	63.7
Zambia	Lusaka	Luangwa	65207	2000	130.8	115.2	150.1
Zambia	Lusaka	Luangwa	65207	2005	112.3	98.5	128.3
Zambia	Lusaka	Luangwa	65207	2010	86.6	74.7	100.1
Zambia	Lusaka	Luangwa	65207	2015	65.1	54.5	75.9
Zambia	Lusaka	Lusaka	65208	2000	105.9	98.4	114.2
Zambia	Lusaka	Lusaka	65208	2005	95.1	87.5	103.0
Zambia	Lusaka	Lusaka	65208	2010	72.9	66.0	79.4
Zambia	Lusaka	Lusaka	65208	2015	55.9	49.1	62.7
Zambia	North-Western	Chavuma	65221	2000	121.1	104.3	139.4
Zambia	North-Western	Chavuma	65221	2005	95.0	81.5	110.0
Zambia	North-Western	Chavuma	65221	2010	71.5	60.5	82.8
Zambia	North-Western	Chavuma	65221	2015	57.8	48.3	68.2
Zambia	North-Western	Kabompo	65222	2000	128.4	114.3	143.8
Zambia	North-Western	Kabompo	65222	2005	97.7	86.4	109.0
Zambia	North-Western	Kabompo	65222	2010	72.6	63.6	81.7
Zambia	North-Western	Kabompo	65222	2015	55.9	48.5	64.5
Zambia	North-Western	Kasempa	65223	2000	139.8	124.1	156.3
Zambia	North-Western	Kasempa	65223	2005	108.6	95.9	123.0
Zambia	North-Western	Kasempa	65223	2010	79.1	69.4	90.2
Zambia	North-Western	Kasempa	65223	2015	59.2	51.0	68.7
Zambia	North-Western	Mufumbwe	65224	2000	139.1	123.5	155.5
Zambia	North-Western	Mufumbwe	65224	2005	108.1	95.7	121.7
Zambia	North-Western	Mufumbwe	65224	2010	78.9	69.4	89.0
Zambia	North-Western	Mufumbwe	65224	2015	61.9	53.5	71.6
Zambia	North-Western	Mwinilunga	65225	2000	129.4	115.1	145.8
Zambia	North-Western	Mwinilunga	65225	2005	103.2	91.6	115.3
Zambia	North-Western	Mwinilunga	65225	2010	71.8	62.8	81.4
Zambia	North-Western	Mwinilunga	65225	2015	55.6	48.0	63.9
Zambia	North-Western	Solwezi	65226	2000	140.4	129.5	151.9
Zambia	North-Western	Solwezi	65226	2005	107.3	98.3	116.7
Zambia	North-Western	Solwezi	65226	2010	79.2	71.2	87.7
Zambia	North-Western	Solwezi	65226	2015	56.6	50.1	63.8
Zambia	North-Western	Zambezi	65227	2000	127.9	114.5	143.0
Zambia	North-Western	Zambezi	65227	2005	96.1	85.7	107.8
Zambia	North-Western	Zambezi	65227	2010	74.9	65.5	85.2
Zambia	North-Western	Zambezi	65227	2015	59.3	51.0	69.1
Zambia	Northern	Chilubi	65209	2000	187.3	167.0	208.2
Zambia	Northern	Chilubi	65209	2005	140.2	124.9	156.9
Zambia	Northern	Chilubi	65209	2010	104.9	92.6	117.4
Zambia	Northern	Chilubi	65209	2015	76.3	66.4	87.0

Admin 0	Admin 1	Admin 2	GAUL Code	Year	Under-5 mortality (per 1,000 livebirths)		
					Estimate	Lower bound, 95% UI	Upper bound, 95% UI
Zambia	Northern	Chinsali	65210	2000	168.0	153.6	183.3
Zambia	Northern	Chinsali	65210	2005	118.1	107.3	129.2
Zambia	Northern	Chinsali	65210	2010	86.4	77.9	96.1
Zambia	Northern	Chinsali	65210	2015	68.3	59.7	77.4
Zambia	Northern	Isoka	65211	2000	146.8	135.5	159.0
Zambia	Northern	Isoka	65211	2005	106.9	98.6	115.4
Zambia	Northern	Isoka	65211	2010	76.0	69.3	83.3
Zambia	Northern	Isoka	65211	2015	56.4	50.1	63.0
Zambia	Northern	Kaputa	65212	2000	210.2	189.3	232.0
Zambia	Northern	Kaputa	65212	2005	168.5	152.4	185.2
Zambia	Northern	Kaputa	65212	2010	126.2	112.2	139.8
Zambia	Northern	Kaputa	65212	2015	102.1	89.1	115.1
Zambia	Northern	Kasama	65213	2000	161.2	145.7	176.4
Zambia	Northern	Kasama	65213	2005	115.7	105.4	127.2
Zambia	Northern	Kasama	65213	2010	81.2	72.9	89.7
Zambia	Northern	Kasama	65213	2015	64.1	55.6	73.5
Zambia	Northern	Luwingu	65214	2000	170.9	156.5	188.2
Zambia	Northern	Luwingu	65214	2005	127.7	116.3	140.6
Zambia	Northern	Luwingu	65214	2010	93.7	84.2	103.5
Zambia	Northern	Luwingu	65214	2015	68.7	60.0	77.4
Zambia	Northern	Mbala	65215	2000	181.7	167.8	196.3
Zambia	Northern	Mbala	65215	2005	140.7	128.9	152.9
Zambia	Northern	Mbala	65215	2010	96.2	87.9	105.3
Zambia	Northern	Mbala	65215	2015	74.7	66.1	84.7
Zambia	Northern	Mpika	65216	2000	163.4	149.1	179.6
Zambia	Northern	Mpika	65216	2005	125.2	114.2	138.0
Zambia	Northern	Mpika	65216	2010	89.1	80.7	98.9
Zambia	Northern	Mpika	65216	2015	67.7	60.1	76.8
Zambia	Northern	Mporokoso	65217	2000	162.8	147.3	179.9
Zambia	Northern	Mporokoso	65217	2005	120.6	108.8	133.1
Zambia	Northern	Mporokoso	65217	2010	84.4	75.7	94.0
Zambia	Northern	Mporokoso	65217	2015	65.6	56.9	74.9
Zambia	Northern	Mpulungu	65218	2000	181.4	164.2	199.8
Zambia	Northern	Mpulungu	65218	2005	143.8	128.8	158.7
Zambia	Northern	Mpulungu	65218	2010	99.2	88.9	110.6
Zambia	Northern	Mpulungu	65218	2015	77.4	67.9	88.4
Zambia	Northern	Mungwi	65219	2000	170.3	155.3	185.7
Zambia	Northern	Mungwi	65219	2005	127.0	116.3	138.8
Zambia	Northern	Mungwi	65219	2010	90.0	81.5	99.5
Zambia	Northern	Mungwi	65219	2015	69.9	61.1	79.3
Zambia	Northern	Nakonde	65220	2000	161.2	146.7	175.9
Zambia	Northern	Nakonde	65220	2005	121.3	110.1	132.2
Zambia	Northern	Nakonde	65220	2010	83.1	75.1	91.7
Zambia	Northern	Nakonde	65220	2015	64.6	56.9	72.9
Zambia	Southern	Choma	65228	2000	121.4	109.7	133.2
Zambia	Southern	Choma	65228	2005	95.4	86.7	105.2
Zambia	Southern	Choma	65228	2010	73.4	66.3	81.4
Zambia	Southern	Choma	65228	2015	54.1	47.0	61.1
Zambia	Southern	Gwembe	65229	2000	126.1	113.1	140.4
Zambia	Southern	Gwembe	65229	2005	102.2	91.8	114.5
Zambia	Southern	Gwembe	65229	2010	80.6	71.8	90.8
Zambia	Southern	Gwembe	65229	2015	57.8	50.2	65.5
Zambia	Southern	Itezhi-tezhi	65230	2000	136.8	121.1	153.9
Zambia	Southern	Itezhi-tezhi	65230	2005	108.8	95.3	122.9
Zambia	Southern	Itezhi-tezhi	65230	2010	86.4	75.6	97.6
Zambia	Southern	Itezhi-tezhi	65230	2015	67.4	57.6	77.3
Zambia	Southern	Kalomo	65231	2000	116.8	105.7	129.1
Zambia	Southern	Kalomo	65231	2005	95.9	87.1	106.2
Zambia	Southern	Kalomo	65231	2010	72.4	64.9	80.6
Zambia	Southern	Kalomo	65231	2015	53.3	46.3	60.2
Zambia	Southern	Kazungula	65232	2000	124.7	111.1	139.2
Zambia	Southern	Kazungula	65232	2005	106.5	95.9	118.8
Zambia	Southern	Kazungula	65232	2010	80.3	71.5	90.1
Zambia	Southern	Kazungula	65232	2015	57.8	50.1	66.8
Zambia	Southern	Livingstone	65233	2000	89.4	78.5	101.5
Zambia	Southern	Livingstone	65233	2005	87.1	76.9	98.6
Zambia	Southern	Livingstone	65233	2010	68.2	59.4	77.7
Zambia	Southern	Livingstone	65233	2015	48.7	41.3	57.3
Zambia	Southern	Mazabuka	65234	2000	128.3	117.7	140.3
Zambia	Southern	Mazabuka	65234	2005	104.0	95.6	114.0
Zambia	Southern	Mazabuka	65234	2010	75.1	67.9	82.9
Zambia	Southern	Mazabuka	65234	2015	55.6	48.6	63.2
Zambia	Southern	Monze	65235	2000	129.7	118.4	142.2
Zambia	Southern	Monze	65235	2005	102.8	93.2	113.9
Zambia	Southern	Monze	65235	2010	75.8	67.9	84.4
Zambia	Southern	Monze	65235	2015	56.5	48.9	64.3
Zambia	Southern	Namwala	65236	2000	124.4	111.4	139.2
Zambia	Southern	Namwala	65236	2005	100.2	89.3	112.2
Zambia	Southern	Namwala	65236	2010	79.1	69.7	88.9
Zambia	Southern	Namwala	65236	2015	60.9	52.3	70.0
Zambia	Southern	Siavonga	65237	2000	109.9	98.3	122.5
Zambia	Southern	Siavonga	65237	2005	97.8	87.2	108.3
Zambia	Southern	Siavonga	65237	2010	79.3	70.6	89.0
Zambia	Southern	Siavonga	65237	2015	57.5	49.7	65.8
Zambia	Southern	Sinazongwe	65238	2000	109.1	97.1	123.4

Admin 0	Admin 1	Admin 2	GAUL Code	Year	Under-5 mortality (per 1,000 livebirths)		
					Estimate	Lower bound, 95% UI	Upper bound, 95% UI
Zambia	Southern	Sinazongwe	65238	2005	96.5	85.8	109.3
Zambia	Southern	Sinazongwe	65238	2010	79.5	70.4	90.8
Zambia	Southern	Sinazongwe	65238	2015	54.2	46.9	62.7
Zambia	Western	Kalabo	65239	2000	157.3	141.2	175.5
Zambia	Western	Kalabo	65239	2005	120.9	108.3	134.6
Zambia	Western	Kalabo	65239	2010	92.5	81.5	104.0
Zambia	Western	Kalabo	65239	2015	74.1	63.5	85.0
Zambia	Western	Kaoma	65240	2000	153.1	137.1	170.6
Zambia	Western	Kaoma	65240	2005	117.5	104.8	131.2
Zambia	Western	Kaoma	65240	2010	91.2	80.1	102.5
Zambia	Western	Kaoma	65240	2015	72.3	61.6	83.2
Zambia	Western	Lukulu	65241	2000	138.8	124.5	154.3
Zambia	Western	Lukulu	65241	2005	102.5	91.8	114.2
Zambia	Western	Lukulu	65241	2010	83.6	74.2	93.6
Zambia	Western	Lukulu	65241	2015	66.4	57.4	76.2
Zambia	Western	Mongu	65242	2000	157.4	143.8	173.0
Zambia	Western	Mongu	65242	2005	120.0	108.6	133.5
Zambia	Western	Mongu	65242	2010	91.3	82.0	101.7
Zambia	Western	Mongu	65242	2015	73.4	63.9	83.3
Zambia	Western	Senanga	65243	2000	167.1	152.1	183.7
Zambia	Western	Senanga	65243	2005	131.1	118.0	144.6
Zambia	Western	Senanga	65243	2010	93.6	83.4	104.6
Zambia	Western	Senanga	65243	2015	73.9	64.6	85.2
Zambia	Western	Sesheke	65244	2000	131.3	117.6	146.1
Zambia	Western	Sesheke	65244	2005	113.4	101.2	127.1
Zambia	Western	Sesheke	65244	2010	88.3	78.2	99.3
Zambia	Western	Sesheke	65244	2015	64.7	55.5	74.0
Zambia	Western	Shang'ombo	65245	2000	156.8	138.7	177.1
Zambia	Western	Shang'ombo	65245	2005	128.4	113.7	144.5
Zambia	Western	Shang'ombo	65245	2010	91.9	80.4	104.9
Zambia	Western	Shang'ombo	65245	2015	71.5	61.5	82.5
Zimbabwe	Bulawayo	Bulawayo	33051	2000	54.2	48.7	59.9
Zimbabwe	Bulawayo	Bulawayo	33051	2005	61.2	55.5	67.5
Zimbabwe	Bulawayo	Bulawayo	33051	2010	58.9	53.0	65.2
Zimbabwe	Bulawayo	Bulawayo	33051	2015	44.3	39.2	50.5
Zimbabwe	Harare	Chitungwiza	68807	2000	61.5	56.2	66.9
Zimbabwe	Harare	Chitungwiza	68807	2005	72.7	67.2	79.1
Zimbabwe	Harare	Chitungwiza	68807	2010	74.6	68.3	80.9
Zimbabwe	Harare	Chitungwiza	68807	2015	53.6	47.4	60.3
Zimbabwe	Harare	Harare	68809	2000	63.4	58.4	68.2
Zimbabwe	Harare	Harare	68809	2005	74.6	69.2	80.0
Zimbabwe	Harare	Harare	68809	2010	76.7	70.9	82.5
Zimbabwe	Harare	Harare	68809	2015	55.6	49.7	62.2
Zimbabwe	Manicaland	Buhera	33053	2000	83.9	75.8	92.2
Zimbabwe	Manicaland	Buhera	33053	2005	92.5	83.7	101.6
Zimbabwe	Manicaland	Buhera	33053	2010	88.9	80.1	98.1
Zimbabwe	Manicaland	Buhera	33053	2015	67.1	59.2	75.8
Zimbabwe	Manicaland	Chimanimani	33054	2000	110.5	99.7	122.7
Zimbabwe	Manicaland	Chimanimani	33054	2005	110.4	100.1	121.8
Zimbabwe	Manicaland	Chimanimani	33054	2010	97.4	87.4	108.4
Zimbabwe	Manicaland	Chimanimani	33054	2015	70.6	61.7	81.0
Zimbabwe	Manicaland	Chipinge	33055	2000	102.8	93.2	112.8
Zimbabwe	Manicaland	Chipinge	33055	2005	98.0	89.8	107.5
Zimbabwe	Manicaland	Chipinge	33055	2010	85.2	76.8	94.5
Zimbabwe	Manicaland	Chipinge	33055	2015	62.7	55.2	71.9
Zimbabwe	Manicaland	Makoni	33056	2000	82.5	75.3	90.2
Zimbabwe	Manicaland	Makoni	33056	2005	90.5	82.3	99.1
Zimbabwe	Manicaland	Makoni	33056	2010	85.6	77.8	94.5
Zimbabwe	Manicaland	Makoni	33056	2015	64.2	56.9	72.3
Zimbabwe	Manicaland	Mutare	61704	2000	96.1	88.1	104.2
Zimbabwe	Manicaland	Mutare	61704	2005	103.3	95.4	112.3
Zimbabwe	Manicaland	Mutare	61704	2010	95.8	87.0	104.6
Zimbabwe	Manicaland	Mutare	61704	2015	70.8	63.0	79.6
Zimbabwe	Manicaland	Mutasa	33058	2000	107.9	98.3	117.8
Zimbabwe	Manicaland	Mutasa	33058	2005	112.6	103.2	123.1
Zimbabwe	Manicaland	Mutasa	33058	2010	99.1	89.8	109.3
Zimbabwe	Manicaland	Mutasa	33058	2015	73.8	64.8	83.7
Zimbabwe	Manicaland	Nyanga	33059	2000	101.8	92.5	111.6
Zimbabwe	Manicaland	Nyanga	33059	2005	100.0	90.5	109.3
Zimbabwe	Manicaland	Nyanga	33059	2010	89.5	80.2	99.1
Zimbabwe	Manicaland	Nyanga	33059	2015	66.0	57.7	75.1
Zimbabwe	Mashonaland Central	Bindura	33060	2000	79.5	72.1	87.3
Zimbabwe	Mashonaland Central	Bindura	33060	2005	85.7	78.0	93.6
Zimbabwe	Mashonaland Central	Bindura	33060	2010	85.7	78.1	93.9
Zimbabwe	Mashonaland Central	Bindura	33060	2015	62.8	55.8	70.2
Zimbabwe	Mashonaland Central	Centenary	33061	2000	94.3	85.5	104.0
Zimbabwe	Mashonaland Central	Centenary	33061	2005	95.3	86.0	105.1
Zimbabwe	Mashonaland Central	Centenary	33061	2010	89.3	80.2	98.1
Zimbabwe	Mashonaland Central	Centenary	33061	2015	68.9	61.0	77.5
Zimbabwe	Mashonaland Central	Guruve	68808	2000	86.3	77.4	95.8
Zimbabwe	Mashonaland Central	Guruve	68808	2005	88.6	79.4	98.2
Zimbabwe	Mashonaland Central	Guruve	68808	2010	84.3	75.7	93.2
Zimbabwe	Mashonaland Central	Guruve	68808	2015	64.8	57.4	73.9
Zimbabwe	Mashonaland Central	Mazowe	33063	2000	79.1	72.8	86.4
Zimbabwe	Mashonaland Central	Mazowe	33063	2005	85.8	79.1	93.0

Admin 0	Admin 1	Admin 2	GAUL Code	Year	Under-5 mortality (per 1,000 livebirths)		
					Estimate	Lower bound, 95% UI	Upper bound, 95% UI
Zimbabwe	Mashonaland Central	Mazowe	33063	2010	84.4	77.6	91.4
Zimbabwe	Mashonaland Central	Mazowe	33063	2015	62.6	56.3	69.9
Zimbabwe	Mashonaland Central	Mbire	68811	2000	98.0	88.0	109.5
Zimbabwe	Mashonaland Central	Mbire	68811	2005	94.1	84.9	105.0
Zimbabwe	Mashonaland Central	Mbire	68811	2010	87.6	78.5	98.5
Zimbabwe	Mashonaland Central	Mbire	68811	2015	68.3	59.9	78.0
Zimbabwe	Mashonaland Central	Mount Darwin	33064	2000	92.9	84.1	102.3
Zimbabwe	Mashonaland Central	Mount Darwin	33064	2005	93.7	85.2	103.4
Zimbabwe	Mashonaland Central	Mount Darwin	33064	2010	90.2	81.4	98.9
Zimbabwe	Mashonaland Central	Mount Darwin	33064	2015	68.8	61.3	77.5
Zimbabwe	Mashonaland Central	Rushinga	33065	2000	93.4	83.3	103.7
Zimbabwe	Mashonaland Central	Rushinga	33065	2005	90.4	81.5	101.4
Zimbabwe	Mashonaland Central	Rushinga	33065	2010	85.7	76.7	96.0
Zimbabwe	Mashonaland Central	Rushinga	33065	2015	65.3	57.0	74.7
Zimbabwe	Mashonaland Central	Shamva	33066	2000	84.5	76.9	93.0
Zimbabwe	Mashonaland Central	Shamva	33066	2005	89.1	81.1	98.1
Zimbabwe	Mashonaland Central	Shamva	33066	2010	87.9	80.0	96.5
Zimbabwe	Mashonaland Central	Shamva	33066	2015	65.6	58.4	73.7
Zimbabwe	Mashonaland East	Chikomba	40749	2000	76.3	68.7	83.9
Zimbabwe	Mashonaland East	Chikomba	40749	2005	85.8	76.9	95.6
Zimbabwe	Mashonaland East	Chikomba	40749	2010	83.6	74.7	92.7
Zimbabwe	Mashonaland East	Chikomba	40749	2015	63.1	55.6	71.4
Zimbabwe	Mashonaland East	Goromonzi	33067	2000	74.2	68.6	80.3
Zimbabwe	Mashonaland East	Goromonzi	33067	2005	82.4	76.4	88.2
Zimbabwe	Mashonaland East	Goromonzi	33067	2010	82.2	76.1	88.8
Zimbabwe	Mashonaland East	Goromonzi	33067	2015	59.5	53.5	65.9
Zimbabwe	Mashonaland East	Hwedza	33068	2000	77.4	69.5	85.6
Zimbabwe	Mashonaland East	Hwedza	33068	2005	86.8	78.5	96.4
Zimbabwe	Mashonaland East	Hwedza	33068	2010	84.6	76.1	93.8
Zimbabwe	Mashonaland East	Hwedza	33068	2015	63.4	55.4	71.6
Zimbabwe	Mashonaland East	Marondera	33069	2000	72.6	65.5	80.2
Zimbabwe	Mashonaland East	Marondera	33069	2005	81.3	73.6	89.1
Zimbabwe	Mashonaland East	Marondera	33069	2010	79.6	72.0	87.6
Zimbabwe	Mashonaland East	Marondera	33069	2015	58.5	50.9	65.7
Zimbabwe	Mashonaland East	Mudzi	33070	2000	98.9	89.5	109.0
Zimbabwe	Mashonaland East	Mudzi	33070	2005	91.8	82.8	101.4
Zimbabwe	Mashonaland East	Mudzi	33070	2010	84.6	76.0	94.2
Zimbabwe	Mashonaland East	Mudzi	33070	2015	63.8	55.7	72.3
Zimbabwe	Mashonaland East	Murehwa	33071	2000	79.6	72.1	88.1
Zimbabwe	Mashonaland East	Murehwa	33071	2005	86.5	78.1	94.7
Zimbabwe	Mashonaland East	Murehwa	33071	2010	83.3	75.5	91.8
Zimbabwe	Mashonaland East	Murehwa	33071	2015	62.3	55.3	70.1
Zimbabwe	Mashonaland East	Mutoko	33072	2000	85.1	76.7	94.1
Zimbabwe	Mashonaland East	Mutoko	33072	2005	87.0	78.5	95.8
Zimbabwe	Mashonaland East	Mutoko	33072	2010	83.6	74.8	92.4
Zimbabwe	Mashonaland East	Mutoko	33072	2015	62.6	54.9	70.9
Zimbabwe	Mashonaland East	Seke	33073	2000	73.1	66.7	80.0
Zimbabwe	Mashonaland East	Seke	33073	2005	82.3	75.4	89.7
Zimbabwe	Mashonaland East	Seke	33073	2010	81.5	74.2	88.8
Zimbabwe	Mashonaland East	Seke	33073	2015	60.2	52.8	67.1
Zimbabwe	Mashonaland East	Uzumba-Maramba-Pfungwe	33074	2000	86.8	78.6	95.4
Zimbabwe	Mashonaland East	Uzumba-Maramba-Pfungwe	33074	2005	88.6	80.5	97.1
Zimbabwe	Mashonaland East	Uzumba-Maramba-Pfungwe	33074	2010	85.4	77.6	94.2
Zimbabwe	Mashonaland East	Uzumba-Maramba-Pfungwe	33074	2015	64.0	56.8	72.2
Zimbabwe	Mashonaland West	Chegutu	33075	2000	76.7	69.8	84.1
Zimbabwe	Mashonaland West	Chegutu	33075	2005	85.8	78.5	93.1
Zimbabwe	Mashonaland West	Chegutu	33075	2010	83.8	76.1	91.5
Zimbabwe	Mashonaland West	Chegutu	33075	2015	62.7	55.8	70.6
Zimbabwe	Mashonaland West	Hurungwe	33076	2000	96.4	87.5	105.6
Zimbabwe	Mashonaland West	Hurungwe	33076	2005	94.8	86.4	103.9
Zimbabwe	Mashonaland West	Hurungwe	33076	2010	88.1	79.7	96.3
Zimbabwe	Mashonaland West	Hurungwe	33076	2015	67.6	59.8	76.0
Zimbabwe	Mashonaland West	Kadoma	33077	2000	81.5	73.9	90.0
Zimbabwe	Mashonaland West	Kadoma	33077	2005	89.2	81.4	97.4
Zimbabwe	Mashonaland West	Kadoma	33077	2010	86.7	78.4	96.0
Zimbabwe	Mashonaland West	Kadoma	33077	2015	65.4	58.2	73.5
Zimbabwe	Mashonaland West	Kariba	33078	2000	97.0	86.6	108.2
Zimbabwe	Mashonaland West	Kariba	33078	2005	90.7	81.3	100.8
Zimbabwe	Mashonaland West	Kariba	33078	2010	89.1	79.6	100.0
Zimbabwe	Mashonaland West	Kariba	33078	2015	67.2	58.5	76.7
Zimbabwe	Mashonaland West	Makonde	33079	2000	87.2	79.1	95.4
Zimbabwe	Mashonaland West	Makonde	33079	2005	91.1	83.1	99.1
Zimbabwe	Mashonaland West	Makonde	33079	2010	85.9	78.4	94.0
Zimbabwe	Mashonaland West	Makonde	33079	2015	65.2	58.0	73.2
Zimbabwe	Mashonaland West	Zvimba	33080	2000	80.8	73.9	88.1
Zimbabwe	Mashonaland West	Zvimba	33080	2005	88.1	80.9	96.0
Zimbabwe	Mashonaland West	Zvimba	33080	2010	84.5	77.3	91.8
Zimbabwe	Mashonaland West	Zvimba	33080	2015	63.2	56.5	70.9
Zimbabwe	Masvingo	Bikita	33081	2000	85.2	76.9	94.9
Zimbabwe	Masvingo	Bikita	33081	2005	87.9	78.7	98.0
Zimbabwe	Masvingo	Bikita	33081	2010	84.8	76.1	94.5
Zimbabwe	Masvingo	Bikita	33081	2015	63.7	55.9	73.3
Zimbabwe	Masvingo	Chiredzi	33082	2000	84.1	75.9	93.3
Zimbabwe	Masvingo	Chiredzi	33082	2005	80.7	72.8	89.3
Zimbabwe	Masvingo	Chiredzi	33082	2010	75.5	67.7	84.2

Admin 0	Admin 1	Admin 2	GAUL Code	Year	Under-5 mortality (per 1,000 livebirths)		
					Estimate	Lower bound, 95% UI	Upper bound, 95% UI
Zimbabwe	Masvingo	Chiredzi	33082	2015	56.9	50.2	65.2
Zimbabwe	Masvingo	Chivi	33083	2000	71.5	63.7	80.1
Zimbabwe	Masvingo	Chivi	33083	2005	76.6	68.6	84.5
Zimbabwe	Masvingo	Chivi	33083	2010	75.9	68.1	84.4
Zimbabwe	Masvingo	Chivi	33083	2015	58.4	51.3	66.5
Zimbabwe	Masvingo	Gutu	33084	2000	79.2	71.3	88.1
Zimbabwe	Masvingo	Gutu	33084	2005	87.2	78.5	96.6
Zimbabwe	Masvingo	Gutu	33084	2010	85.4	76.6	94.5
Zimbabwe	Masvingo	Gutu	33084	2015	64.7	56.6	73.3
Zimbabwe	Masvingo	Masvingo	33085	2000	73.2	65.8	81.3
Zimbabwe	Masvingo	Masvingo	33085	2005	78.9	71.1	86.8
Zimbabwe	Masvingo	Masvingo	33085	2010	78.1	70.2	86.7
Zimbabwe	Masvingo	Masvingo	33085	2015	59.8	52.6	67.5
Zimbabwe	Masvingo	Mwenezi	33086	2000	70.4	62.5	79.3
Zimbabwe	Masvingo	Mwenezi	33086	2005	70.5	62.8	79.2
Zimbabwe	Masvingo	Mwenezi	33086	2010	68.3	60.8	76.5
Zimbabwe	Masvingo	Mwenezi	33086	2015	53.3	46.5	61.4
Zimbabwe	Masvingo	Zaka	33087	2000	79.3	71.0	89.6
Zimbabwe	Masvingo	Zaka	33087	2005	82.1	73.5	91.6
Zimbabwe	Masvingo	Zaka	33087	2010	80.0	71.3	89.3
Zimbabwe	Masvingo	Zaka	33087	2015	60.9	52.8	69.2
Zimbabwe	Matabeleland North	Binga	33088	2000	83.2	74.3	92.7
Zimbabwe	Matabeleland North	Binga	33088	2005	79.8	71.0	89.6
Zimbabwe	Matabeleland North	Binga	33088	2010	79.7	70.4	89.4
Zimbabwe	Matabeleland North	Binga	33088	2015	57.3	49.7	64.9
Zimbabwe	Matabeleland North	Bubi	33089	2000	62.8	54.8	72.1
Zimbabwe	Matabeleland North	Bubi	33089	2005	67.0	58.7	76.0
Zimbabwe	Matabeleland North	Bubi	33089	2010	61.8	54.1	69.9
Zimbabwe	Matabeleland North	Bubi	33089	2015	48.1	41.1	55.1
Zimbabwe	Matabeleland North	Hwange	61705	2000	75.9	67.2	85.3
Zimbabwe	Matabeleland North	Hwange	61705	2005	71.2	63.2	80.5
Zimbabwe	Matabeleland North	Hwange	61705	2010	69.1	61.1	78.7
Zimbabwe	Matabeleland North	Hwange	61705	2015	51.2	43.7	59.4
Zimbabwe	Matabeleland North	Lupane	33091	2000	66.3	58.0	74.5
Zimbabwe	Matabeleland North	Lupane	33091	2005	67.8	59.6	76.3
Zimbabwe	Matabeleland North	Lupane	33091	2010	63.6	55.7	71.8
Zimbabwe	Matabeleland North	Lupane	33091	2015	48.7	41.9	55.9
Zimbabwe	Matabeleland North	Nkayi	33092	2000	67.3	59.6	75.5
Zimbabwe	Matabeleland North	Nkayi	33092	2005	72.0	64.5	80.8
Zimbabwe	Matabeleland North	Nkayi	33092	2010	68.4	60.6	76.5
Zimbabwe	Matabeleland North	Nkayi	33092	2015	53.3	46.2	60.8
Zimbabwe	Matabeleland North	Tsholotsho	33093	2000	65.8	57.1	75.1
Zimbabwe	Matabeleland North	Tsholotsho	33093	2005	65.5	57.2	74.7
Zimbabwe	Matabeleland North	Tsholotsho	33093	2010	59.4	52.0	68.0
Zimbabwe	Matabeleland North	Tsholotsho	33093	2015	46.7	39.7	53.7
Zimbabwe	Matabeleland North	Umguza	33094	2000	60.7	54.7	66.8
Zimbabwe	Matabeleland North	Umguza	33094	2005	66.4	60.9	72.1
Zimbabwe	Matabeleland North	Umguza	33094	2010	60.9	55.5	66.9
Zimbabwe	Matabeleland North	Umguza	33094	2015	47.3	42.0	53.6
Zimbabwe	Matabeleland South	Beitbridge	33095	2000	63.2	55.8	72.0
Zimbabwe	Matabeleland South	Beitbridge	33095	2005	63.2	55.9	71.7
Zimbabwe	Matabeleland South	Beitbridge	33095	2010	60.6	52.9	69.0
Zimbabwe	Matabeleland South	Beitbridge	33095	2015	48.7	41.8	56.0
Zimbabwe	Matabeleland South	Bulilima	68806	2000	67.5	59.0	76.4
Zimbabwe	Matabeleland South	Bulilima	68806	2005	67.3	59.1	76.9
Zimbabwe	Matabeleland South	Bulilima	68806	2010	60.7	53.3	69.4
Zimbabwe	Matabeleland South	Bulilima	68806	2015	49.1	42.0	57.3
Zimbabwe	Matabeleland South	Gwanda	33097	2000	61.2	54.3	68.7
Zimbabwe	Matabeleland South	Gwanda	33097	2005	65.3	58.1	74.3
Zimbabwe	Matabeleland South	Gwanda	33097	2010	61.7	54.4	69.9
Zimbabwe	Matabeleland South	Gwanda	33097	2015	50.6	43.6	58.4
Zimbabwe	Matabeleland South	Insiza	33098	2000	62.2	55.7	69.7
Zimbabwe	Matabeleland South	Insiza	33098	2005	68.5	61.8	76.6
Zimbabwe	Matabeleland South	Insiza	33098	2010	65.7	58.9	73.3
Zimbabwe	Matabeleland South	Insiza	33098	2015	52.6	46.1	59.8
Zimbabwe	Matabeleland South	Mangwe	68810	2000	64.4	57.5	72.0
Zimbabwe	Matabeleland South	Mangwe	68810	2005	66.6	59.7	74.3
Zimbabwe	Matabeleland South	Mangwe	68810	2010	61.3	54.2	69.6
Zimbabwe	Matabeleland South	Mangwe	68810	2015	50.5	43.1	58.3
Zimbabwe	Matabeleland South	Matobo	33099	2000	62.0	55.8	68.6
Zimbabwe	Matabeleland South	Matobo	33099	2005	66.1	60.0	73.1
Zimbabwe	Matabeleland South	Matobo	33099	2010	61.4	55.2	68.2
Zimbabwe	Matabeleland South	Matobo	33099	2015	49.7	43.5	56.8
Zimbabwe	Matabeleland South	Umzingwane	33100	2000	61.0	54.0	68.0
Zimbabwe	Matabeleland South	Umzingwane	33100	2005	66.8	59.6	74.8
Zimbabwe	Matabeleland South	Umzingwane	33100	2010	62.3	55.4	70.3
Zimbabwe	Matabeleland South	Umzingwane	33100	2015	49.8	43.3	57.1
Zimbabwe	Midlands	Chirumhanzu	33101	2000	71.7	63.7	79.9
Zimbabwe	Midlands	Chirumhanzu	33101	2005	82.0	73.3	91.7
Zimbabwe	Midlands	Chirumhanzu	33101	2010	82.8	73.7	92.5
Zimbabwe	Midlands	Chirumhanzu	33101	2015	62.4	54.9	71.4
Zimbabwe	Midlands	Gokwe North	40750	2000	88.6	79.8	97.9
Zimbabwe	Midlands	Gokwe North	40750	2005	89.3	80.2	98.8
Zimbabwe	Midlands	Gokwe North	40750	2010	88.4	79.2	98.2
Zimbabwe	Midlands	Gokwe North	40750	2015	66.1	57.9	75.4

Admin 0	Admin 1	Admin 2	GAUL Code	Year	Under-5 mortality (per 1,000 livebirths)		
					Estimate	Lower bound, 95% UI	Upper bound, 95% UI
Zimbabwe	Midlands	Gokwe South	40751	2000	76.3	69.1	84.8
Zimbabwe	Midlands	Gokwe South	40751	2005	80.8	72.5	89.0
Zimbabwe	Midlands	Gokwe South	40751	2010	79.5	71.5	87.8
Zimbabwe	Midlands	Gokwe South	40751	2015	60.2	53.2	68.3
Zimbabwe	Midlands	Gweru	33103	2000	63.0	56.1	70.8
Zimbabwe	Midlands	Gweru	33103	2005	72.8	65.0	81.0
Zimbabwe	Midlands	Gweru	33103	2010	74.5	66.3	83.5
Zimbabwe	Midlands	Gweru	33103	2015	56.4	49.0	65.1
Zimbabwe	Midlands	Kwekwe	33104	2000	67.9	61.2	75.4
Zimbabwe	Midlands	Kwekwe	33104	2005	77.3	70.4	85.1
Zimbabwe	Midlands	Kwekwe	33104	2010	77.9	70.2	86.5
Zimbabwe	Midlands	Kwekwe	33104	2015	59.1	52.1	67.2
Zimbabwe	Midlands	Mberengwa	33105	2000	64.0	56.8	72.2
Zimbabwe	Midlands	Mberengwa	33105	2005	69.7	61.9	78.0
Zimbabwe	Midlands	Mberengwa	33105	2010	70.3	62.5	78.4
Zimbabwe	Midlands	Mberengwa	33105	2015	54.7	47.7	62.3
Zimbabwe	Midlands	Shurugwi	33106	2000	67.6	60.2	75.7
Zimbabwe	Midlands	Shurugwi	33106	2005	77.2	69.0	86.0
Zimbabwe	Midlands	Shurugwi	33106	2010	78.7	70.4	88.1
Zimbabwe	Midlands	Shurugwi	33106	2015	59.7	52.4	68.6
Zimbabwe	Midlands	Zvishavane	33107	2000	64.6	56.8	72.8
Zimbabwe	Midlands	Zvishavane	33107	2005	72.0	64.4	80.7
Zimbabwe	Midlands	Zvishavane	33107	2010	72.9	64.8	81.9
Zimbabwe	Midlands	Zvishavane	33107	2015	56.0	48.6	64.0

Supplementary results Table 3. **Neonatal mortality estimates for subnational administrative level 1 divisions in Africa, 2000, 2005, 2010, and 2015.** UI = uncertainty interval

Admin 0	Admin 1	GAUL Code	Year	Neonatal mortality (per 1,000 livebirths)		
				Estimate	Lower bound, 95% UI	Upper bound, 95% UI
Angola	Bengo	398	2000	41.1	34.2	50.0
Angola	Bengo	398	2005	33.4	28.0	40.1
Angola	Bengo	398	2010	27.3	22.7	32.8
Angola	Bengo	398	2015	21.2	17.0	26.7
Angola	Benguela	399	2000	39.3	31.3	48.2
Angola	Benguela	399	2005	35.1	28.3	42.9
Angola	Benguela	399	2010	29.7	23.4	37.1
Angola	Benguela	399	2015	22.3	17.0	28.7
Angola	Bie	400	2000	31.9	24.8	39.9
Angola	Bie	400	2005	26.9	21.0	33.3
Angola	Bie	400	2010	22.3	17.5	28.2
Angola	Bie	400	2015	16.8	12.6	21.6
Angola	Cabinda	401	2000	34.7	28.9	41.5
Angola	Cabinda	401	2005	29.1	24.1	34.7
Angola	Cabinda	401	2010	23.0	18.8	27.7
Angola	Cabinda	401	2015	18.8	14.7	23.4
Angola	Cuando Cubango	402	2000	31.9	26.1	38.4
Angola	Cuando Cubango	402	2005	27.2	22.5	33.1
Angola	Cuando Cubango	402	2010	22.9	18.4	28.3
Angola	Cuando Cubango	402	2015	17.6	13.9	22.3
Angola	Cuanza Sul	403	2000	40.4	32.6	49.5
Angola	Cuanza Sul	403	2005	36.0	28.8	44.1
Angola	Cuanza Sul	403	2010	26.3	20.9	32.9
Angola	Cuanza Sul	403	2015	20.6	15.8	26.3
Angola	Cunene	404	2000	30.6	25.5	36.9
Angola	Cunene	404	2005	27.7	22.8	33.3
Angola	Cunene	404	2010	22.8	18.7	27.8
Angola	Cunene	404	2015	17.6	13.9	22.1
Angola	Huambo	405	2000	37.3	29.5	46.1
Angola	Huambo	405	2005	32.4	26.2	40.0
Angola	Huambo	405	2010	24.8	19.7	31.2
Angola	Huambo	405	2015	19.2	14.8	25.1
Angola	Huila	406	2000	41.2	34.7	49.3
Angola	Huila	406	2005	36.2	30.9	42.4
Angola	Huila	406	2010	29.9	24.9	35.5
Angola	Huila	406	2015	22.5	18.0	27.3
Angola	Kuanza Norte	407	2000	43.5	35.0	53.4
Angola	Kuanza Norte	407	2005	35.0	28.2	42.2
Angola	Kuanza Norte	407	2010	26.0	20.4	32.3
Angola	Kuanza Norte	407	2015	20.6	15.8	26.5
Angola	Luanda	408	2000	32.2	27.0	37.9
Angola	Luanda	408	2005	29.6	25.0	34.5
Angola	Luanda	408	2010	27.2	22.3	32.3
Angola	Luanda	408	2015	20.1	15.8	25.2
Angola	Lunda Norte	409	2000	36.7	29.5	45.0
Angola	Lunda Norte	409	2005	29.7	24.2	35.8
Angola	Lunda Norte	409	2010	23.4	18.5	28.6
Angola	Lunda Norte	409	2015	18.2	14.3	22.7
Angola	Lunda Sul	410	2000	38.0	29.7	47.7
Angola	Lunda Sul	410	2005	31.8	25.0	40.0
Angola	Lunda Sul	410	2010	26.1	19.8	32.6
Angola	Lunda Sul	410	2015	20.4	15.3	26.3
Angola	Malanje	411	2000	39.2	31.4	47.9
Angola	Malanje	411	2005	31.1	25.1	37.6
Angola	Malanje	411	2010	24.5	19.7	30.7
Angola	Malanje	411	2015	19.0	14.7	24.4
Angola	Moxico	412	2000	36.0	28.4	44.4
Angola	Moxico	412	2005	30.2	24.1	37.1
Angola	Moxico	412	2010	23.9	18.5	29.7
Angola	Moxico	412	2015	19.4	14.8	24.8
Angola	Namibe	413	2000	36.8	29.0	44.8
Angola	Namibe	413	2005	32.7	26.3	39.8
Angola	Namibe	413	2010	28.3	22.8	34.6
Angola	Namibe	413	2015	21.3	16.7	27.2
Angola	Uige	414	2000	45.1	37.1	54.5

Admin 0	Admin 1	GAUL Code	Year	Neonatal mortality (per 1,000 livebirths)		
				Estimate	Lower bound, 95% UI	Upper bound, 95% UI
Angola	Uige	414	2005	33.5	27.8	39.8
Angola	Uige	414	2010	26.4	21.4	31.9
Angola	Uige	414	2015	20.3	15.8	25.5
Angola	Zaire	415	2000	41.0	33.1	50.0
Angola	Zaire	415	2005	34.8	28.3	42.1
Angola	Zaire	415	2010	28.0	22.8	34.4
Angola	Zaire	415	2015	21.2	16.7	26.7
Benin	Alibori	609	2000	40.0	35.8	44.5
Benin	Alibori	609	2005	36.9	32.9	41.2
Benin	Alibori	609	2010	31.7	27.4	36.3
Benin	Alibori	609	2015	27.4	22.6	33.1
Benin	Atakora	610	2000	34.2	30.8	37.7
Benin	Atakora	610	2005	32.1	28.9	35.7
Benin	Atakora	610	2010	26.1	23.0	29.7
Benin	Atakora	610	2015	23.4	19.4	27.9
Benin	Atlantique	611	2000	35.7	32.7	38.9
Benin	Atlantique	611	2005	32.0	29.3	35.1
Benin	Atlantique	611	2010	30.1	26.7	33.7
Benin	Atlantique	611	2015	26.8	22.2	32.2
Benin	Borgou	612	2000	36.5	32.4	40.9
Benin	Borgou	612	2005	30.9	27.4	34.7
Benin	Borgou	612	2010	26.8	23.5	30.9
Benin	Borgou	612	2015	23.3	19.3	28.1
Benin	Collines	613	2000	41.7	37.2	46.9
Benin	Collines	613	2005	37.1	32.7	41.4
Benin	Collines	613	2010	32.1	28.0	36.5
Benin	Collines	613	2015	27.8	23.0	33.2
Benin	Couffo	614	2000	38.6	35.3	42.2
Benin	Couffo	614	2005	33.1	30.1	36.3
Benin	Couffo	614	2010	29.9	26.6	33.5
Benin	Couffo	614	2015	27.5	23.1	32.4
Benin	Donga	615	2000	40.7	36.3	45.6
Benin	Donga	615	2005	36.5	32.3	40.6
Benin	Donga	615	2010	30.1	26.1	34.4
Benin	Donga	615	2015	26.1	21.4	31.3
Benin	Littoral	616	2000	31.3	28.0	34.9
Benin	Littoral	616	2005	29.3	26.4	32.8
Benin	Littoral	616	2010	28.9	25.3	32.9
Benin	Littoral	616	2015	27.4	22.5	33.4
Benin	Mono	617	2000	35.8	32.4	39.5
Benin	Mono	617	2005	31.5	28.3	34.9
Benin	Mono	617	2010	28.4	25.2	32.1
Benin	Mono	617	2015	25.7	21.4	30.9
Benin	Oueme	618	2000	37.6	34.3	41.3
Benin	Oueme	618	2005	34.3	31.4	37.4
Benin	Oueme	618	2010	32.8	29.0	36.8
Benin	Oueme	618	2015	28.1	23.4	33.8
Benin	Plateau	619	2000	42.7	38.9	46.7
Benin	Plateau	619	2005	37.5	34.2	41.1
Benin	Plateau	619	2010	33.4	29.6	37.4
Benin	Plateau	619	2015	28.5	24.2	33.9
Benin	Zou	620	2000	40.5	37.1	44.2
Benin	Zou	620	2005	34.9	31.7	38.3
Benin	Zou	620	2010	32.0	28.4	36.1
Benin	Zou	620	2015	28.4	23.8	34.1
Botswana	Central	654	2000	18.0	15.4	21.1
Botswana	Central	654	2005	16.4	14.1	19.3
Botswana	Central	654	2010	12.0	10.1	14.1
Botswana	Central	654	2015	7.8	6.3	9.5
Botswana	Chobe	655	2000	22.1	17.7	26.8
Botswana	Chobe	655	2005	19.5	15.8	24.1
Botswana	Chobe	655	2010	13.2	10.5	16.4
Botswana	Chobe	655	2015	8.7	6.7	11.2
Botswana	Ghanzi	656	2000	22.7	17.3	28.8
Botswana	Ghanzi	656	2005	19.8	15.1	24.9
Botswana	Ghanzi	656	2010	13.1	10.1	16.6
Botswana	Ghanzi	656	2015	8.6	6.5	11.1

Admin 0	Admin 1	GAUL Code	Year	Neonatal mortality (per 1,000 livebirths)		
				Estimate	Lower bound, 95% UI	Upper bound, 95% UI
Botswana	Kgalagadi	657	2000	19.5	15.2	24.6
Botswana	Kgalagadi	657	2005	17.3	13.4	21.8
Botswana	Kgalagadi	657	2010	11.9	9.1	15.2
Botswana	Kgalagadi	657	2015	8.0	6.0	10.4
Botswana	Kgatleng	658	2000	18.5	15.3	22.3
Botswana	Kgatleng	658	2005	16.6	13.4	20.3
Botswana	Kgatleng	658	2010	12.0	9.5	15.1
Botswana	Kgatleng	658	2015	7.8	6.0	10.0
Botswana	Kweneng	659	2000	19.5	16.0	23.3
Botswana	Kweneng	659	2005	17.4	14.3	21.1
Botswana	Kweneng	659	2010	12.3	9.8	15.2
Botswana	Kweneng	659	2015	8.0	6.2	10.1
Botswana	Ngamiland	660	2000	25.5	20.3	31.6
Botswana	Ngamiland	660	2005	22.1	17.7	27.4
Botswana	Ngamiland	660	2010	14.9	11.8	18.6
Botswana	Ngamiland	660	2015	9.6	7.3	12.3
Botswana	North East	661	2000	15.1	12.2	18.4
Botswana	North East	661	2005	14.0	11.5	17.0
Botswana	North East	661	2010	10.6	8.5	12.9
Botswana	North East	661	2015	7.1	5.4	8.9
Botswana	South-East	662	2000	17.7	14.7	21.0
Botswana	South-East	662	2005	16.2	13.3	19.4
Botswana	South-East	662	2010	11.6	9.3	14.3
Botswana	South-East	662	2015	7.7	5.9	9.8
Botswana	Southern	663	2000	18.9	15.9	22.4
Botswana	Southern	663	2005	16.9	14.1	20.2
Botswana	Southern	663	2010	12.0	9.7	14.6
Botswana	Southern	663	2015	7.8	6.2	9.8
Burkina Faso	Boucle Du Mouhoun	40276	2000	40.4	37.1	43.9
Burkina Faso	Boucle Du Mouhoun	40276	2005	35.9	32.9	39.1
Burkina Faso	Boucle Du Mouhoun	40276	2010	31.8	28.7	35.3
Burkina Faso	Boucle Du Mouhoun	40276	2015	29.0	25.1	33.4
Burkina Faso	Cascades	40277	2000	49.4	45.1	54.3
Burkina Faso	Cascades	40277	2005	45.8	41.6	50.3
Burkina Faso	Cascades	40277	2010	40.4	35.9	45.2
Burkina Faso	Cascades	40277	2015	34.8	29.3	41.1
Burkina Faso	Centre	40278	2000	32.0	28.5	35.7
Burkina Faso	Centre	40278	2005	29.1	26.1	32.6
Burkina Faso	Centre	40278	2010	29.5	26.0	33.7
Burkina Faso	Centre	40278	2015	25.4	21.1	30.1
Burkina Faso	Centre-est	40279	2000	37.3	34.4	40.7
Burkina Faso	Centre-est	40279	2005	33.9	31.2	36.8
Burkina Faso	Centre-est	40279	2010	30.2	27.2	33.7
Burkina Faso	Centre-est	40279	2015	27.3	23.3	31.9
Burkina Faso	Centre-nord	40280	2000	39.9	36.6	43.4
Burkina Faso	Centre-nord	40280	2005	35.9	33.0	38.8
Burkina Faso	Centre-nord	40280	2010	31.9	28.8	35.5
Burkina Faso	Centre-nord	40280	2015	30.4	25.8	35.2
Burkina Faso	Centre-ouest	40281	2000	39.1	36.1	42.5
Burkina Faso	Centre-ouest	40281	2005	35.6	32.8	38.9
Burkina Faso	Centre-ouest	40281	2010	31.7	28.5	35.4
Burkina Faso	Centre-ouest	40281	2015	29.4	25.2	34.6
Burkina Faso	Centre-sud	40282	2000	37.6	34.5	40.8
Burkina Faso	Centre-sud	40282	2005	34.0	31.1	37.1
Burkina Faso	Centre-sud	40282	2010	30.8	27.7	34.5
Burkina Faso	Centre-sud	40282	2015	27.1	23.3	31.5
Burkina Faso	Est	40283	2000	45.6	41.9	49.7
Burkina Faso	Est	40283	2005	40.8	37.5	44.6
Burkina Faso	Est	40283	2010	36.5	32.7	40.6
Burkina Faso	Est	40283	2015	32.7	27.9	37.9
Burkina Faso	Hauts-bassins	40284	2000	40.8	37.3	44.8
Burkina Faso	Hauts-bassins	40284	2005	38.5	35.2	42.2
Burkina Faso	Hauts-bassins	40284	2010	34.1	30.4	38.3
Burkina Faso	Hauts-bassins	40284	2015	30.7	25.8	35.9
Burkina Faso	Nord	40285	2000	40.8	37.1	44.4
Burkina Faso	Nord	40285	2005	35.7	32.4	39.1
Burkina Faso	Nord	40285	2010	31.9	28.4	35.9

Admin 0	Admin 1	GAUL Code	Year	Neonatal mortality (per 1,000 livebirths)		
				Estimate	Lower bound, 95% UI	Upper bound, 95% UI
Burkina Faso	Nord	40285	2015	29.8	25.1	35.4
Burkina Faso	Plateau Central	40286	2000	37.7	34.9	40.8
Burkina Faso	Plateau Central	40286	2005	33.5	31.0	36.2
Burkina Faso	Plateau Central	40286	2010	30.9	27.9	34.1
Burkina Faso	Plateau Central	40286	2015	28.5	24.5	32.8
Burkina Faso	Sahel	40287	2000	46.6	42.7	50.8
Burkina Faso	Sahel	40287	2005	41.2	37.7	44.8
Burkina Faso	Sahel	40287	2010	37.6	33.4	41.9
Burkina Faso	Sahel	40287	2015	34.7	29.4	40.5
Burkina Faso	Sud-ouest	40288	2000	43.3	39.4	47.4
Burkina Faso	Sud-ouest	40288	2005	40.8	37.1	44.7
Burkina Faso	Sud-ouest	40288	2010	35.4	31.5	39.7
Burkina Faso	Sud-ouest	40288	2015	31.4	26.4	37.2
Burundi	Bubanza	40542	2000	43.4	39.9	47.2
Burundi	Bubanza	40542	2005	36.7	33.5	39.8
Burundi	Bubanza	40542	2010	31.4	28.5	34.5
Burundi	Bubanza	40542	2015	28.3	24.5	32.7
Burundi	Bujumbura Mairie	40543	2000	35.5	31.6	39.9
Burundi	Bujumbura Mairie	40543	2005	33.3	29.7	37.4
Burundi	Bujumbura Mairie	40543	2010	29.3	26.0	32.9
Burundi	Bujumbura Mairie	40543	2015	28.7	24.2	33.6
Burundi	Bujumbura Rural	40544	2000	39.5	35.6	43.8
Burundi	Bujumbura Rural	40544	2005	35.3	31.9	39.3
Burundi	Bujumbura Rural	40544	2010	29.8	26.8	33.3
Burundi	Bujumbura Rural	40544	2015	27.5	23.5	32.0
Burundi	Bururi	40545	2000	38.4	34.3	42.8
Burundi	Bururi	40545	2005	34.4	31.1	38.3
Burundi	Bururi	40545	2010	29.1	25.9	32.6
Burundi	Bururi	40545	2015	26.1	22.0	30.5
Burundi	Cankuzo	40546	2000	41.4	36.9	46.3
Burundi	Cankuzo	40546	2005	35.5	31.7	39.5
Burundi	Cankuzo	40546	2010	30.0	26.4	33.6
Burundi	Cankuzo	40546	2015	28.2	23.8	33.1
Burundi	Cibitoke	40547	2000	44.3	40.6	48.7
Burundi	Cibitoke	40547	2005	36.5	33.3	39.7
Burundi	Cibitoke	40547	2010	31.2	28.1	34.4
Burundi	Cibitoke	40547	2015	29.2	25.0	33.9
Burundi	Gitega	40548	2000	42.0	38.3	45.8
Burundi	Gitega	40548	2005	36.5	33.5	39.7
Burundi	Gitega	40548	2010	31.3	28.5	34.4
Burundi	Gitega	40548	2015	27.8	24.1	32.1
Burundi	Karuzi	40549	2000	46.6	42.4	50.9
Burundi	Karuzi	40549	2005	39.0	35.7	42.3
Burundi	Karuzi	40549	2010	32.7	29.7	35.6
Burundi	Karuzi	40549	2015	29.6	25.6	34.1
Burundi	Kayanza	40550	2000	46.0	42.2	50.1
Burundi	Kayanza	40550	2005	38.3	35.3	41.1
Burundi	Kayanza	40550	2010	32.4	29.5	35.4
Burundi	Kayanza	40550	2015	28.2	24.4	32.5
Burundi	Kirundo	40551	2000	47.7	44.0	51.4
Burundi	Kirundo	40551	2005	37.2	34.2	40.1
Burundi	Kirundo	40551	2010	30.8	28.0	33.7
Burundi	Kirundo	40551	2015	29.5	25.6	33.9
Burundi	Makamba	40552	2000	35.8	31.3	40.5
Burundi	Makamba	40552	2005	33.0	29.2	37.2
Burundi	Makamba	40552	2010	27.6	24.2	31.4
Burundi	Makamba	40552	2015	24.8	20.7	29.6
Burundi	Muramvya	40553	2000	42.8	39.4	46.4
Burundi	Muramvya	40553	2005	36.9	34.1	40.0
Burundi	Muramvya	40553	2010	31.0	28.4	34.0
Burundi	Muramvya	40553	2015	27.6	24.1	31.7
Burundi	Muyinga	40554	2000	46.5	42.3	50.5
Burundi	Muyinga	40554	2005	37.5	34.3	41.1
Burundi	Muyinga	40554	2010	31.5	28.6	34.4
Burundi	Muyinga	40554	2015	29.5	25.4	33.8
Burundi	Mwaro	40555	2000	41.3	37.5	45.0
Burundi	Mwaro	40555	2005	36.0	32.7	39.3

Admin 0	Admin 1	GAUL Code	Year	Neonatal mortality (per 1,000 livebirths)		
				Estimate	Lower bound, 95% UI	Upper bound, 95% UI
Burundi	Mwaro	40555	2010	30.6	27.7	33.7
Burundi	Mwaro	40555	2015	27.3	23.5	31.6
Burundi	Ngozi	40556	2000	47.3	43.6	51.3
Burundi	Ngozi	40556	2005	39.0	35.9	42.1
Burundi	Ngozi	40556	2010	32.5	29.7	35.4
Burundi	Ngozi	40556	2015	29.4	25.6	33.8
Burundi	Rutana	40557	2000	38.5	34.7	42.6
Burundi	Rutana	40557	2005	34.9	31.5	38.5
Burundi	Rutana	40557	2010	29.5	26.4	33.0
Burundi	Rutana	40557	2015	26.6	22.6	31.0
Burundi	Ruyigi	40558	2000	42.0	37.6	46.6
Burundi	Ruyigi	40558	2005	36.8	33.1	40.7
Burundi	Ruyigi	40558	2010	31.1	27.8	34.9
Burundi	Ruyigi	40558	2015	28.3	24.0	33.2
Cameroon	Adamaoua	815	2000	43.8	38.5	49.5
Cameroon	Adamaoua	815	2005	37.8	32.9	42.7
Cameroon	Adamaoua	815	2010	34.0	28.5	39.3
Cameroon	Adamaoua	815	2015	31.3	25.3	37.8
Cameroon	Centre	816	2000	32.1	28.4	36.1
Cameroon	Centre	816	2005	30.5	27.0	34.0
Cameroon	Centre	816	2010	29.1	24.9	33.8
Cameroon	Centre	816	2015	26.2	21.0	32.0
Cameroon	Est	817	2000	36.5	31.8	41.7
Cameroon	Est	817	2005	33.7	29.2	38.9
Cameroon	Est	817	2010	29.1	24.5	34.6
Cameroon	Est	817	2015	28.5	23.4	34.9
Cameroon	Extrême - Nord	818	2000	39.0	35.5	42.6
Cameroon	Extrême - Nord	818	2005	37.2	33.7	40.8
Cameroon	Extrême - Nord	818	2010	35.1	31.5	39.1
Cameroon	Extrême - Nord	818	2015	32.7	28.1	37.8
Cameroon	Littoral	819	2000	34.1	29.7	38.6
Cameroon	Littoral	819	2005	31.7	27.6	35.9
Cameroon	Littoral	819	2010	30.3	25.7	35.3
Cameroon	Littoral	819	2015	25.6	20.7	31.9
Cameroon	Nord	820	2000	48.1	43.7	52.8
Cameroon	Nord	820	2005	42.7	38.8	47.1
Cameroon	Nord	820	2010	37.4	33.3	41.9
Cameroon	Nord	820	2015	33.2	28.0	38.8
Cameroon	Nord - Ouest	821	2000	32.7	28.9	36.6
Cameroon	Nord - Ouest	821	2005	31.9	28.2	35.8
Cameroon	Nord - Ouest	821	2010	28.2	24.5	32.7
Cameroon	Nord - Ouest	821	2015	25.9	21.3	31.2
Cameroon	Ouest	822	2000	30.7	27.2	34.8
Cameroon	Ouest	822	2005	28.6	25.4	32.1
Cameroon	Ouest	822	2010	26.9	22.9	31.4
Cameroon	Ouest	822	2015	24.4	20.0	29.8
Cameroon	Sud	823	2000	36.3	31.6	41.7
Cameroon	Sud	823	2005	33.9	29.2	39.1
Cameroon	Sud	823	2010	31.2	26.4	36.7
Cameroon	Sud	823	2015	29.5	23.8	35.9
Cameroon	Sud - Ouest	824	2000	37.2	33.3	41.4
Cameroon	Sud - Ouest	824	2005	34.3	30.7	38.1
Cameroon	Sud - Ouest	824	2010	30.8	26.9	35.4
Cameroon	Sud - Ouest	824	2015	27.3	22.4	33.3
Central African Republic	Bamingui-Bangoran	853	2000	43.1	33.4	56.1
Central African Republic	Bamingui-Bangoran	853	2005	41.6	31.7	54.4
Central African Republic	Bamingui-Bangoran	853	2010	40.4	30.7	53.1
Central African Republic	Bamingui-Bangoran	853	2015	38.0	28.5	50.7
Central African Republic	Bangui	67154	2000	41.2	31.6	52.0
Central African Republic	Bangui	67154	2005	42.9	33.7	53.9
Central African Republic	Bangui	67154	2010	40.9	30.6	52.4
Central African Republic	Bangui	67154	2015	41.0	30.1	54.4
Central African Republic	Basse-Kotto	854	2000	48.2	37.4	61.1
Central African Republic	Basse-Kotto	854	2005	47.6	37.2	59.0
Central African Republic	Basse-Kotto	854	2010	42.4	33.4	53.6
Central African Republic	Basse-Kotto	854	2015	40.8	31.1	53.1
Central African Republic	Haut-Mbomou	855	2000	48.9	38.2	63.8

Admin 0	Admin 1	GAUL Code	Year	Neonatal mortality (per 1,000 livebirths)		
				Estimate	Lower bound, 95% UI	Upper bound, 95% UI
Central African Republic	Haut-Mbomou	855	2005	45.1	34.8	58.2
Central African Republic	Haut-Mbomou	855	2010	47.9	36.1	62.1
Central African Republic	Haut-Mbomou	855	2015	47.8	35.1	62.9
Central African Republic	Haute-Kotto	856	2000	44.9	33.2	59.3
Central African Republic	Haute-Kotto	856	2005	43.1	31.8	56.6
Central African Republic	Haute-Kotto	856	2010	42.8	31.5	56.7
Central African Republic	Haute-Kotto	856	2015	41.5	29.9	55.6
Central African Republic	Kémo	857	2000	47.1	35.4	60.4
Central African Republic	Kémo	857	2005	46.4	34.7	59.8
Central African Republic	Kémo	857	2010	42.5	31.4	56.2
Central African Republic	Kémo	857	2015	40.9	29.3	54.2
Central African Republic	Lobaye	858	2000	47.0	37.7	58.3
Central African Republic	Lobaye	858	2005	44.6	36.1	55.4
Central African Republic	Lobaye	858	2010	41.1	32.8	52.0
Central African Republic	Lobaye	858	2015	37.6	28.9	48.1
Central African Republic	Mambéré-Kadéï	859	2000	47.3	37.6	58.3
Central African Republic	Mambéré-Kadéï	859	2005	44.0	35.1	54.3
Central African Republic	Mambéré-Kadéï	859	2010	42.5	33.2	54.0
Central African Republic	Mambéré-Kadéï	859	2015	37.6	28.3	48.8
Central African Republic	Mbomou	860	2000	47.8	36.6	61.0
Central African Republic	Mbomou	860	2005	46.1	35.2	58.2
Central African Republic	Mbomou	860	2010	43.7	33.5	56.4
Central African Republic	Mbomou	860	2015	42.8	31.6	55.6
Central African Republic	Nana-Gribizi	861	2000	43.6	33.1	56.0
Central African Republic	Nana-Gribizi	861	2005	42.5	32.3	55.5
Central African Republic	Nana-Gribizi	861	2010	40.8	31.0	54.3
Central African Republic	Nana-Gribizi	861	2015	37.7	27.9	50.5
Central African Republic	Nana-Mambéré	862	2000	47.4	38.1	58.3
Central African Republic	Nana-Mambéré	862	2005	45.2	35.8	56.3
Central African Republic	Nana-Mambéré	862	2010	44.8	34.6	55.4
Central African Republic	Nana-Mambéré	862	2015	39.8	30.0	50.7
Central African Republic	Ombella M'Poko	863	2000	45.7	36.1	56.2
Central African Republic	Ombella M'Poko	863	2005	46.3	37.1	56.8
Central African Republic	Ombella M'Poko	863	2010	42.2	32.8	52.9
Central African Republic	Ombella M'Poko	863	2015	41.0	31.0	53.3
Central African Republic	Ouaka	864	2000	47.5	36.7	60.7
Central African Republic	Ouaka	864	2005	46.2	35.8	58.3
Central African Republic	Ouaka	864	2010	42.4	32.6	55.7
Central African Republic	Ouaka	864	2015	40.8	30.3	54.5
Central African Republic	Ouham	865	2000	47.2	37.1	58.4
Central African Republic	Ouham	865	2005	46.3	36.2	57.5
Central African Republic	Ouham	865	2010	45.5	35.5	57.1
Central African Republic	Ouham	865	2015	40.5	30.5	51.5
Central African Republic	Ouham Pendé	866	2000	51.8	42.6	63.4
Central African Republic	Ouham Pendé	866	2005	52.4	42.4	62.8
Central African Republic	Ouham Pendé	866	2010	51.0	41.3	62.5
Central African Republic	Ouham Pendé	866	2015	45.2	35.3	56.8
Central African Republic	Sangha-Mbaéré	867	2000	46.2	36.3	57.8
Central African Republic	Sangha-Mbaéré	867	2005	41.7	33.2	52.1
Central African Republic	Sangha-Mbaéré	867	2010	39.6	30.4	50.6
Central African Republic	Sangha-Mbaéré	867	2015	37.1	27.5	48.3
Central African Republic	Vakaga	868	2000	44.2	35.3	54.9
Central African Republic	Vakaga	868	2005	42.4	34.0	53.2
Central African Republic	Vakaga	868	2010	43.4	34.2	54.4
Central African Republic	Vakaga	868	2015	41.8	31.9	51.7
Chad	Assongha	12926	2000	45.6	39.4	53.0
Chad	Assongha	12926	2005	41.7	36.3	48.4
Chad	Assongha	12926	2010	35.2	30.3	40.9
Chad	Assongha	12926	2015	30.9	25.5	37.8
Chad	Baguirmi	12910	2000	45.8	39.6	52.3
Chad	Baguirmi	12910	2005	44.1	38.2	49.8
Chad	Baguirmi	12910	2010	40.0	34.7	45.8
Chad	Baguirmi	12910	2015	35.4	30.1	41.6
Chad	Barh Koh	12923	2000	43.6	37.0	50.9
Chad	Barh Koh	12923	2005	38.2	32.7	44.2
Chad	Barh Koh	12923	2010	33.4	28.5	38.7
Chad	Barh Koh	12923	2015	29.6	24.4	35.6

Admin 0	Admin 1	GAUL Code	Year	Neonatal mortality (per 1,000 livebirths)		
				Estimate	Lower bound, 95% UI	Upper bound, 95% UI
Chad	Barl El Gazal	12914	2000	30.9	26.0	36.2
Chad	Barl El Gazal	12914	2005	27.6	23.4	32.3
Chad	Barl El Gazal	12914	2010	23.8	20.1	28.0
Chad	Barl El Gazal	12914	2015	22.6	18.3	27.4
Chad	Batha Est	12904	2000	36.7	30.5	44.5
Chad	Batha Est	12904	2005	32.3	26.8	38.7
Chad	Batha Est	12904	2010	26.9	22.1	32.5
Chad	Batha Est	12904	2015	24.5	19.3	30.4
Chad	Batha Ouest	12905	2000	34.2	29.2	40.8
Chad	Batha Ouest	12905	2005	31.1	26.6	36.3
Chad	Batha Ouest	12905	2010	26.4	22.4	31.4
Chad	Batha Ouest	12905	2015	24.4	20.0	29.7
Chad	Biltine	870	2000	37.6	32.2	44.3
Chad	Biltine	870	2005	33.5	28.8	38.9
Chad	Biltine	870	2010	28.8	24.8	33.6
Chad	Biltine	870	2015	26.2	21.9	31.4
Chad	Borkou	12907	2000	31.5	25.0	38.4
Chad	Borkou	12907	2005	29.0	23.4	35.3
Chad	Borkou	12907	2010	25.4	20.2	31.3
Chad	Borkou	12907	2015	24.3	19.2	30.2
Chad	Daraba	12911	2000	39.9	33.8	46.7
Chad	Daraba	12911	2005	37.2	31.8	43.4
Chad	Daraba	12911	2010	33.2	28.3	38.6
Chad	Daraba	12911	2015	29.4	24.2	35.1
Chad	Ennedi	12908	2000	34.8	28.5	42.4
Chad	Ennedi	12908	2005	31.1	25.4	37.7
Chad	Ennedi	12908	2010	27.8	22.0	33.7
Chad	Ennedi	12908	2015	26.0	20.3	32.2
Chad	Guera	873	2000	45.0	38.9	51.5
Chad	Guera	873	2005	40.3	35.3	46.0
Chad	Guera	873	2010	34.0	29.3	39.2
Chad	Guera	873	2015	30.1	25.4	35.9
Chad	Hadjer Lamis	12912	2000	39.2	34.9	43.8
Chad	Hadjer Lamis	12912	2005	37.5	33.3	42.0
Chad	Hadjer Lamis	12912	2010	34.5	30.4	39.0
Chad	Hadjer Lamis	12912	2015	35.4	29.8	41.6
Chad	Kabia	12920	2000	42.8	37.6	48.5
Chad	Kabia	12920	2005	42.1	37.3	47.5
Chad	Kabia	12920	2010	38.8	33.8	44.2
Chad	Kabia	12920	2015	35.3	30.0	41.4
Chad	Kanem	12915	2000	33.6	29.1	38.2
Chad	Kanem	12915	2005	30.1	26.5	34.2
Chad	Kanem	12915	2010	25.7	22.2	29.6
Chad	Kanem	12915	2015	24.2	20.5	28.7
Chad	Lac	875	2000	37.7	32.6	43.9
Chad	Lac	875	2005	33.5	28.8	39.2
Chad	Lac	875	2010	28.6	24.3	33.8
Chad	Lac	875	2015	27.3	22.5	33.2
Chad	Lac Iro	12924	2000	45.2	36.8	54.5
Chad	Lac Iro	12924	2005	39.0	32.1	47.2
Chad	Lac Iro	12924	2010	33.5	27.3	40.9
Chad	Lac Iro	12924	2015	29.9	23.8	37.4
Chad	Logone Occidental	876	2000	52.3	46.7	59.3
Chad	Logone Occidental	876	2005	49.8	44.4	55.2
Chad	Logone Occidental	876	2010	46.6	41.1	52.4
Chad	Logone Occidental	876	2015	39.2	33.3	46.0
Chad	Logone Oriental	12918	2000	53.6	47.4	60.6
Chad	Logone Oriental	12918	2005	49.8	43.7	55.9
Chad	Logone Oriental	12918	2010	46.1	40.9	51.8
Chad	Logone Oriental	12918	2015	38.4	32.7	44.7
Chad	Mandoul	12925	2000	47.4	41.3	54.4
Chad	Mandoul	12925	2005	42.6	37.4	48.3
Chad	Mandoul	12925	2010	37.4	32.8	42.7
Chad	Mandoul	12925	2015	32.4	27.2	38.6
Chad	Mayo-Boneye	12921	2000	42.2	36.8	48.1
Chad	Mayo-Boneye	12921	2005	41.4	36.2	46.8
Chad	Mayo-Boneye	12921	2010	37.6	32.4	42.9

Admin 0	Admin 1	GAUL Code	Year	Neonatal mortality (per 1,000 livebirths)		
				Estimate	Lower bound, 95% UI	Upper bound, 95% UI
Chad	Mayo-Boneye	12921	2015	34.7	29.2	41.2
Chad	Mayo-Dala	12922	2000	43.2	38.5	48.2
Chad	Mayo-Dala	12922	2005	41.0	36.4	45.7
Chad	Mayo-Dala	12922	2010	36.5	32.2	41.5
Chad	Mayo-Dala	12922	2015	33.7	28.6	39.9
Chad	Mont De Lam	12919	2000	59.2	50.4	69.2
Chad	Mont De Lam	12919	2005	54.8	46.8	63.9
Chad	Mont De Lam	12919	2010	48.5	40.7	57.1
Chad	Mont De Lam	12919	2015	40.8	33.2	49.8
Chad	Ouaddai	12927	2000	42.1	36.5	48.8
Chad	Ouaddai	12927	2005	37.3	32.5	42.6
Chad	Ouaddai	12927	2010	30.5	25.9	35.4
Chad	Ouaddai	12927	2015	27.7	22.7	33.0
Chad	Salamat	881	2000	43.2	36.7	50.3
Chad	Salamat	881	2005	37.2	31.8	43.0
Chad	Salamat	881	2010	30.8	26.0	35.9
Chad	Salamat	881	2015	27.8	22.6	33.7
Chad	Sila	12928	2000	44.7	38.0	52.0
Chad	Sila	12928	2005	38.7	33.2	44.7
Chad	Sila	12928	2010	32.7	27.9	37.9
Chad	Sila	12928	2015	29.5	24.0	35.2
Chad	Tandjile Est	12930	2000	47.1	40.7	54.2
Chad	Tandjile Est	12930	2005	44.3	38.3	50.7
Chad	Tandjile Est	12930	2010	41.8	35.7	48.5
Chad	Tandjile Est	12930	2015	34.6	29.0	41.1
Chad	Tandjile Ouest	12931	2000	47.5	41.4	53.9
Chad	Tandjile Ouest	12931	2005	45.7	40.3	51.5
Chad	Tandjile Ouest	12931	2010	43.6	38.0	49.7
Chad	Tandjile Ouest	12931	2015	36.5	31.1	43.1
Chad	Tibesti	12909	2000	32.5	22.8	45.9
Chad	Tibesti	12909	2005	29.7	20.7	42.1
Chad	Tibesti	12909	2010	25.6	18.1	35.8
Chad	Tibesti	12909	2015	24.9	17.5	35.1
Congo	Bouenza	970	2000	31.1	26.5	36.2
Congo	Bouenza	970	2005	28.5	24.4	33.2
Congo	Bouenza	970	2010	23.7	20.2	27.8
Congo	Bouenza	970	2015	20.5	16.5	25.0
Congo	Brazzaville	190432	2000	29.9	26.2	34.1
Congo	Brazzaville	190432	2005	26.8	23.4	30.5
Congo	Brazzaville	190432	2010	23.4	20.1	27.3
Congo	Brazzaville	190432	2015	19.5	15.6	23.7
Congo	Cuvette	971	2000	29.9	25.0	35.2
Congo	Cuvette	971	2005	28.6	24.1	34.0
Congo	Cuvette	971	2010	24.2	20.0	28.9
Congo	Cuvette	971	2015	21.3	17.2	26.3
Congo	Cuvette-Ouest	972	2000	28.1	23.6	33.3
Congo	Cuvette-Ouest	972	2005	26.9	22.9	32.2
Congo	Cuvette-Ouest	972	2010	22.6	18.8	27.1
Congo	Cuvette-Ouest	972	2015	20.9	16.7	26.0
Congo	Kouilou	190433	2000	23.7	20.2	27.6
Congo	Kouilou	190433	2005	23.0	19.6	27.0
Congo	Kouilou	190433	2010	19.1	16.1	22.8
Congo	Kouilou	190433	2015	18.1	14.4	22.3
Congo	Lekoumou	974	2000	26.9	22.9	31.6
Congo	Lekoumou	974	2005	24.8	21.1	28.9
Congo	Lekoumou	974	2010	20.2	16.8	24.0
Congo	Lekoumou	974	2015	19.6	15.7	24.4
Congo	Likouala	975	2000	31.6	26.2	37.5
Congo	Likouala	975	2005	30.1	25.3	35.8
Congo	Likouala	975	2010	24.3	20.4	29.0
Congo	Likouala	975	2015	22.6	18.1	28.0
Congo	Niari	976	2000	24.2	20.7	28.2
Congo	Niari	976	2005	23.5	20.3	27.1
Congo	Niari	976	2010	19.4	16.6	22.7
Congo	Niari	976	2015	18.1	14.8	21.9
Congo	Plateaux	977	2000	31.4	26.6	37.1
Congo	Plateaux	977	2005	28.7	24.4	34.0

Admin 0	Admin 1	GAUL Code	Year	Neonatal mortality (per 1,000 livebirths)		
				Estimate	Lower bound, 95% UI	Upper bound, 95% UI
Congo	Plateaux	977	2010	24.4	20.3	29.1
Congo	Plateaux	977	2015	21.7	17.4	27.0
Congo	Point-Noire	190434	2000	21.9	18.5	25.7
Congo	Point-Noire	190434	2005	21.4	17.9	25.3
Congo	Point-Noire	190434	2010	19.3	15.9	23.0
Congo	Point-Noire	190434	2015	18.1	14.4	22.6
Congo	Pool	190431	2000	33.3	29.4	37.6
Congo	Pool	190431	2005	29.1	25.5	32.9
Congo	Pool	190431	2010	24.0	20.7	27.5
Congo	Pool	190431	2015	20.4	16.7	24.4
Congo	Sangha	979	2000	33.0	27.8	39.2
Congo	Sangha	979	2005	31.7	26.7	37.0
Congo	Sangha	979	2010	26.0	21.7	31.0
Congo	Sangha	979	2015	25.5	20.5	31.2
Côte d'Ivoire	Bas Sassandra	190430	2000	39.7	34.0	45.8
Côte d'Ivoire	Bas Sassandra	190430	2005	35.4	30.5	40.8
Côte d'Ivoire	Bas Sassandra	190430	2010	32.9	27.7	38.4
Côte d'Ivoire	Bas Sassandra	190430	2015	30.3	24.5	37.1
Côte d'Ivoire	Comoe	16837	2000	41.7	37.0	47.3
Côte d'Ivoire	Comoe	16837	2005	40.1	35.7	45.0
Côte d'Ivoire	Comoe	16837	2010	37.1	32.3	42.4
Côte d'Ivoire	Comoe	16837	2015	32.7	27.1	39.7
Côte d'Ivoire	Denguele	1041	2000	66.4	58.0	75.9
Côte d'Ivoire	Denguele	1041	2005	60.4	52.3	68.6
Côte d'Ivoire	Denguele	1041	2010	52.4	44.5	60.8
Côte d'Ivoire	Denguele	1041	2015	45.1	36.6	55.1
Côte d'Ivoire	District autonome de Abidjan	16838	2000	36.8	31.1	43.6
Côte d'Ivoire	District autonome de Abidjan	16838	2005	38.1	32.4	45.2
Côte d'Ivoire	District autonome de Abidjan	16838	2010	37.6	31.2	45.3
Côte d'Ivoire	District autonome de Abidjan	16838	2015	33.1	25.7	41.9
Côte d'Ivoire	District autonome de Yamoussoukro	16839	2000	40.5	34.0	48.2
Côte d'Ivoire	District autonome de Yamoussoukro	16839	2005	35.1	29.2	41.6
Côte d'Ivoire	District autonome de Yamoussoukro	16839	2010	33.6	27.1	40.8
Côte d'Ivoire	District autonome de Yamoussoukro	16839	2015	30.6	23.9	38.4
Côte d'Ivoire	Gôh-Djiboua	16840	2000	39.3	32.8	45.6
Côte d'Ivoire	Gôh-Djiboua	16840	2005	35.0	29.5	41.1
Côte d'Ivoire	Gôh-Djiboua	16840	2010	32.7	27.3	39.1
Côte d'Ivoire	Gôh-Djiboua	16840	2015	30.1	23.6	37.3
Côte d'Ivoire	Lacs	16841	2000	45.0	39.2	51.7
Côte d'Ivoire	Lacs	16841	2005	41.9	36.6	47.4
Côte d'Ivoire	Lacs	16841	2010	38.1	32.6	44.2
Côte d'Ivoire	Lacs	16841	2015	33.8	27.3	41.1
Côte d'Ivoire	Lagunes	16842	2000	40.8	35.4	47.0
Côte d'Ivoire	Lagunes	16842	2005	40.0	34.5	45.5
Côte d'Ivoire	Lagunes	16842	2010	36.5	31.1	42.8
Côte d'Ivoire	Lagunes	16842	2015	33.0	26.5	40.7
Côte d'Ivoire	Montagnes	16843	2000	45.4	39.9	51.3
Côte d'Ivoire	Montagnes	16843	2005	38.7	33.8	44.0
Côte d'Ivoire	Montagnes	16843	2010	33.3	28.7	38.4
Côte d'Ivoire	Montagnes	16843	2015	30.9	25.1	37.9
Côte d'Ivoire	Sassandra-Marahoue	16844	2000	45.9	39.7	52.8
Côte d'Ivoire	Sassandra-Marahoue	16844	2005	39.6	34.3	45.5
Côte d'Ivoire	Sassandra-Marahoue	16844	2010	36.0	30.5	42.9
Côte d'Ivoire	Sassandra-Marahoue	16844	2015	32.3	26.2	39.6
Côte d'Ivoire	Savanes	1048	2000	67.1	60.3	74.8
Côte d'Ivoire	Savanes	1048	2005	64.5	57.8	72.1
Côte d'Ivoire	Savanes	1048	2010	57.2	50.5	65.0
Côte d'Ivoire	Savanes	1048	2015	48.9	40.9	58.5
Côte d'Ivoire	Vallee Du Bandama	1051	2000	43.5	37.6	50.1
Côte d'Ivoire	Vallee Du Bandama	1051	2005	41.4	36.0	47.3
Côte d'Ivoire	Vallee Du Bandama	1051	2010	36.9	31.4	43.0
Côte d'Ivoire	Vallee Du Bandama	1051	2015	32.6	26.6	39.5
Côte d'Ivoire	Woroba	16845	2000	61.6	54.7	69.4
Côte d'Ivoire	Woroba	16845	2005	55.4	48.6	62.6
Côte d'Ivoire	Woroba	16845	2010	47.3	41.0	54.2
Côte d'Ivoire	Woroba	16845	2015	40.7	33.3	48.4
Côte d'Ivoire	Zanzan	1053	2000	42.7	37.8	48.2

Admin 0	Admin 1	GAUL Code	Year	Neonatal mortality (per 1,000 livebirths)		
				Estimate	Lower bound, 95% UI	Upper bound, 95% UI
Côte d'Ivoire	Zanzan	1053	2005	42.6	37.5	48.3
Côte d'Ivoire	Zanzan	1053	2010	38.1	33.2	43.9
Côte d'Ivoire	Zanzan	1053	2015	33.4	27.3	40.1
Democratic Republic of the Congo	Bandundu	1066	2000	33.5	29.4	38.5
Democratic Republic of the Congo	Bandundu	1066	2005	31.6	27.6	35.9
Democratic Republic of the Congo	Bandundu	1066	2010	27.4	23.7	31.1
Democratic Republic of the Congo	Bandundu	1066	2015	23.5	19.5	28.0
Democratic Republic of the Congo	Bas-Congo	1067	2000	41.3	35.9	47.5
Democratic Republic of the Congo	Bas-Congo	1067	2005	35.8	31.5	40.6
Democratic Republic of the Congo	Bas-Congo	1067	2010	32.7	28.3	37.2
Democratic Republic of the Congo	Bas-Congo	1067	2015	27.2	22.3	32.3
Democratic Republic of the Congo	Equateur	1068	2000	39.6	35.0	44.6
Democratic Republic of the Congo	Equateur	1068	2005	38.0	34.0	42.3
Democratic Republic of the Congo	Equateur	1068	2010	30.9	27.2	34.7
Democratic Republic of the Congo	Equateur	1068	2015	27.2	23.5	31.5
Democratic Republic of the Congo	Kasai Occidental	1069	2000	33.6	29.1	38.5
Democratic Republic of the Congo	Kasai Occidental	1069	2005	31.3	27.4	35.4
Democratic Republic of the Congo	Kasai Occidental	1069	2010	27.9	24.1	32.4
Democratic Republic of the Congo	Kasai Occidental	1069	2015	24.1	20.1	28.7
Democratic Republic of the Congo	Kasai Oriental	1070	2000	31.0	27.0	35.5
Democratic Republic of the Congo	Kasai Oriental	1070	2005	31.1	27.5	35.4
Democratic Republic of the Congo	Kasai Oriental	1070	2010	27.9	24.0	32.2
Democratic Republic of the Congo	Kasai Oriental	1070	2015	25.7	21.5	30.6
Democratic Republic of the Congo	Katanga	1071	2000	41.4	37.3	46.0
Democratic Republic of the Congo	Katanga	1071	2005	37.5	34.0	41.7
Democratic Republic of the Congo	Katanga	1071	2010	35.3	31.6	39.4
Democratic Republic of the Congo	Katanga	1071	2015	30.8	26.8	35.3
Democratic Republic of the Congo	Kinshasa	1072	2000	32.9	29.0	37.4
Democratic Republic of the Congo	Kinshasa	1072	2005	28.9	25.4	32.6
Democratic Republic of the Congo	Kinshasa	1072	2010	27.8	23.8	32.2
Democratic Republic of the Congo	Kinshasa	1072	2015	22.0	17.5	26.6
Democratic Republic of the Congo	Maniema	1073	2000	38.9	33.5	44.4
Democratic Republic of the Congo	Maniema	1073	2005	36.4	31.6	41.5
Democratic Republic of the Congo	Maniema	1073	2010	31.8	27.1	36.8
Democratic Republic of the Congo	Maniema	1073	2015	29.2	24.2	34.8
Democratic Republic of the Congo	Nord-Kivu	1074	2000	34.4	30.6	38.2
Democratic Republic of the Congo	Nord-Kivu	1074	2005	27.6	24.7	30.6
Democratic Republic of the Congo	Nord-Kivu	1074	2010	26.3	23.3	29.6
Democratic Republic of the Congo	Nord-Kivu	1074	2015	21.4	18.2	24.9
Democratic Republic of the Congo	Orientale	1075	2000	40.6	35.9	46.0
Democratic Republic of the Congo	Orientale	1075	2005	35.9	32.0	41.1
Democratic Republic of the Congo	Orientale	1075	2010	33.2	28.7	37.9
Democratic Republic of the Congo	Orientale	1075	2015	29.8	25.4	35.1
Democratic Republic of the Congo	Sud-Kivu	1076	2000	39.1	35.1	43.6
Democratic Republic of the Congo	Sud-Kivu	1076	2005	35.0	31.7	38.6
Democratic Republic of the Congo	Sud-Kivu	1076	2010	32.8	29.4	36.3
Democratic Republic of the Congo	Sud-Kivu	1076	2015	27.2	23.5	31.4
Djibouti	Ali Sabieh	1093	2000	28.3	24.2	33.0
Djibouti	Ali Sabieh	1093	2005	25.1	21.0	29.8
Djibouti	Ali Sabieh	1093	2010	22.0	17.7	27.2
Djibouti	Ali Sabieh	1093	2015	17.8	13.8	22.7
Djibouti	Dikhil	1094	2000	26.6	22.6	30.8
Djibouti	Dikhil	1094	2005	23.9	20.0	28.2
Djibouti	Dikhil	1094	2010	20.8	16.8	25.4
Djibouti	Dikhil	1094	2015	17.7	13.6	22.4
Djibouti	Djibouti	1095	2000	30.9	26.6	35.6
Djibouti	Djibouti	1095	2005	27.5	22.9	32.7
Djibouti	Djibouti	1095	2010	23.5	18.8	29.0
Djibouti	Djibouti	1095	2015	20.1	15.5	25.5
Djibouti	Obock	1096	2000	31.8	25.8	39.0
Djibouti	Obock	1096	2005	28.1	22.2	34.9
Djibouti	Obock	1096	2010	24.0	18.4	30.7
Djibouti	Obock	1096	2015	20.5	15.3	26.9
Djibouti	Tadjourah	1097	2000	30.3	25.3	35.7
Djibouti	Tadjourah	1097	2005	26.5	21.9	32.0
Djibouti	Tadjourah	1097	2010	23.1	18.1	28.5
Djibouti	Tadjourah	1097	2015	19.9	15.0	25.5

Admin 0	Admin 1	GAUL Code	Year	Neonatal mortality (per 1,000 livebirths)		
				Estimate	Lower bound, 95% UI	Upper bound, 95% UI
Egypt	Alexandria	61510	2000	17.1	14.0	20.4
Egypt	Alexandria	61510	2005	14.2	11.5	17.3
Egypt	Alexandria	61510	2010	11.8	9.6	14.4
Egypt	Alexandria	61510	2015	9.0	7.1	11.3
Egypt	Assiut	61511	2000	25.4	22.0	29.0
Egypt	Assiut	61511	2005	21.5	18.6	24.8
Egypt	Assiut	61511	2010	17.5	14.8	20.7
Egypt	Assiut	61511	2015	12.5	10.1	15.2
Egypt	Aswan	61512	2000	24.6	20.6	28.9
Egypt	Aswan	61512	2005	22.7	19.0	26.8
Egypt	Aswan	61512	2010	18.8	15.2	22.8
Egypt	Aswan	61512	2015	13.7	10.9	17.0
Egypt	Behera	61513	2000	18.3	15.9	21.1
Egypt	Behera	61513	2005	14.0	12.2	16.2
Egypt	Behera	61513	2010	10.7	9.0	12.6
Egypt	Behera	61513	2015	8.2	6.7	10.1
Egypt	Beni Suef	61514	2000	22.9	20.2	25.8
Egypt	Beni Suef	61514	2005	18.1	15.9	20.6
Egypt	Beni Suef	61514	2010	14.0	12.0	16.4
Egypt	Beni Suef	61514	2015	11.3	9.2	13.4
Egypt	Cairo	61515	2000	19.0	16.9	21.0
Egypt	Cairo	61515	2005	16.1	14.1	18.1
Egypt	Cairo	61515	2010	12.8	11.2	14.7
Egypt	Cairo	61515	2015	10.2	8.5	12.1
Egypt	Dakahlia	61516	2000	17.2	15.2	19.3
Egypt	Dakahlia	61516	2005	13.4	11.7	15.1
Egypt	Dakahlia	61516	2010	10.4	9.0	12.2
Egypt	Dakahlia	61516	2015	8.3	6.9	10.0
Egypt	Damietta	61517	2000	16.2	13.8	18.9
Egypt	Damietta	61517	2005	12.9	11.0	15.0
Egypt	Damietta	61517	2010	10.7	8.9	12.6
Egypt	Damietta	61517	2015	8.2	6.6	10.0
Egypt	Fayoum	61518	2000	21.5	18.8	24.7
Egypt	Fayoum	61518	2005	16.9	14.8	19.2
Egypt	Fayoum	61518	2010	12.9	10.9	15.1
Egypt	Fayoum	61518	2015	10.3	8.4	12.6
Egypt	Gharbia	61519	2000	17.2	15.3	19.3
Egypt	Gharbia	61519	2005	13.3	11.8	15.2
Egypt	Gharbia	61519	2010	10.1	8.8	11.8
Egypt	Gharbia	61519	2015	8.1	6.7	9.7
Egypt	Giza	61520	2000	19.5	17.5	21.8
Egypt	Giza	61520	2005	16.0	14.1	17.8
Egypt	Giza	61520	2010	12.7	11.0	14.5
Egypt	Giza	61520	2015	10.0	8.3	11.9
Egypt	Hala'ib triangle	40776	2000	26.8	18.9	37.1
Egypt	Hala'ib triangle	40776	2005	23.7	16.8	32.5
Egypt	Hala'ib triangle	40776	2010	19.5	13.9	26.8
Egypt	Hala'ib triangle	40776	2015	15.6	10.8	21.8
Egypt	Ismailia	61521	2000	19.3	16.5	22.4
Egypt	Ismailia	61521	2005	16.0	13.6	18.7
Egypt	Ismailia	61521	2010	12.8	10.7	15.0
Egypt	Ismailia	61521	2015	9.8	7.9	11.9
Egypt	Kafr El-Shikh	61522	2000	16.9	14.4	19.5
Egypt	Kafr El-Shikh	61522	2005	13.0	11.1	15.1
Egypt	Kafr El-Shikh	61522	2010	10.1	8.5	11.9
Egypt	Kafr El-Shikh	61522	2015	7.8	6.4	9.7
Egypt	Kalyoubia	61523	2000	19.3	17.3	21.4
Egypt	Kalyoubia	61523	2005	15.6	13.9	17.3
Egypt	Kalyoubia	61523	2010	11.8	10.2	13.5
Egypt	Kalyoubia	61523	2015	9.5	8.0	11.2
Egypt	Luxor	61524	2000	22.4	19.1	26.1
Egypt	Luxor	61524	2005	20.1	16.9	23.5
Egypt	Luxor	61524	2010	15.9	13.1	18.8
Egypt	Luxor	61524	2015	11.4	9.2	14.1
Egypt	Matrouh	61525	2000	18.5	14.4	23.5
Egypt	Matrouh	61525	2005	15.8	12.1	20.1
Egypt	Matrouh	61525	2010	12.8	9.6	16.6

Admin 0	Admin 1	GAUL Code	Year	Neonatal mortality (per 1,000 livebirths)		
				Estimate	Lower bound, 95% UI	Upper bound, 95% UI
Egypt	Matrouh	61525	2015	9.7	7.2	12.6
Egypt	Menia	61526	2000	22.4	19.7	25.2
Egypt	Menia	61526	2005	18.6	16.1	21.6
Egypt	Menia	61526	2010	14.7	12.5	17.3
Egypt	Menia	61526	2015	11.1	8.9	13.4
Egypt	Menoufia	61527	2000	18.5	16.5	20.8
Egypt	Menoufia	61527	2005	14.4	12.8	16.2
Egypt	Menoufia	61527	2010	10.8	9.2	12.6
Egypt	Menoufia	61527	2015	8.5	7.1	10.3
Egypt	New Valley	61528	2000	22.8	19.1	27.1
Egypt	New Valley	61528	2005	21.1	17.7	25.4
Egypt	New Valley	61528	2010	18.6	15.5	22.5
Egypt	New Valley	61528	2015	13.4	11.0	16.3
Egypt	North Sinai	61529	2000	19.5	14.6	25.3
Egypt	North Sinai	61529	2005	17.1	12.9	22.3
Egypt	North Sinai	61529	2010	14.8	10.9	19.4
Egypt	North Sinai	61529	2015	10.9	8.0	14.6
Egypt	Port Said	61530	2000	15.8	13.1	18.9
Egypt	Port Said	61530	2005	13.4	11.1	16.1
Egypt	Port Said	61530	2010	11.5	9.4	14.0
Egypt	Port Said	61530	2015	9.2	7.3	11.5
Egypt	Qena	61531	2000	23.2	20.2	26.5
Egypt	Qena	61531	2005	20.0	17.3	22.7
Egypt	Qena	61531	2010	15.7	13.2	18.4
Egypt	Qena	61531	2015	11.5	9.3	13.8
Egypt	Red Sea	61532	2000	22.1	17.2	27.7
Egypt	Red Sea	61532	2005	20.2	15.8	25.3
Egypt	Red Sea	61532	2010	17.4	13.7	21.7
Egypt	Red Sea	61532	2015	13.4	10.4	17.2
Egypt	Shrkia	61533	2000	19.2	17.0	21.5
Egypt	Shrkia	61533	2005	15.0	13.3	16.9
Egypt	Shrkia	61533	2010	11.4	9.9	13.1
Egypt	Shrkia	61533	2015	9.5	7.8	11.4
Egypt	South Sinai	61534	2000	22.0	16.4	28.9
Egypt	South Sinai	61534	2005	19.7	14.4	26.0
Egypt	South Sinai	61534	2010	16.9	12.5	22.3
Egypt	South Sinai	61534	2015	13.0	9.5	17.3
Egypt	Suez	61535	2000	19.9	16.5	23.6
Egypt	Suez	61535	2005	17.0	14.1	20.4
Egypt	Suez	61535	2010	14.0	11.5	16.9
Egypt	Suez	61535	2015	10.5	8.3	13.0
Egypt	Suhag	61536	2000	25.8	22.2	29.3
Egypt	Suhag	61536	2005	21.6	18.7	24.8
Egypt	Suhag	61536	2010	17.4	14.7	20.2
Egypt	Suhag	61536	2015	13.0	10.5	15.8
Equatorial Guinea	Bioko Norte	1199	2000	33.1	25.8	42.1
Equatorial Guinea	Bioko Norte	1199	2005	28.7	22.1	36.7
Equatorial Guinea	Bioko Norte	1199	2010	27.0	20.6	34.5
Equatorial Guinea	Bioko Norte	1199	2015	20.3	15.3	26.8
Equatorial Guinea	Bioko Sur	1200	2000	33.8	25.7	43.6
Equatorial Guinea	Bioko Sur	1200	2005	29.1	22.2	38.1
Equatorial Guinea	Bioko Sur	1200	2010	27.2	20.7	36.0
Equatorial Guinea	Bioko Sur	1200	2015	20.7	15.4	27.6
Equatorial Guinea	Centro Sur	1201	2000	39.5	30.7	50.2
Equatorial Guinea	Centro Sur	1201	2005	31.2	24.1	39.7
Equatorial Guinea	Centro Sur	1201	2010	26.6	20.4	34.0
Equatorial Guinea	Centro Sur	1201	2015	21.8	16.0	28.8
Equatorial Guinea	Kientem	1202	2000	41.9	33.8	51.6
Equatorial Guinea	Kientem	1202	2005	32.6	26.1	40.3
Equatorial Guinea	Kientem	1202	2010	27.9	21.7	35.4
Equatorial Guinea	Kientem	1202	2015	22.7	17.1	29.5
Equatorial Guinea	Litoral	1203	2000	40.7	31.1	51.8
Equatorial Guinea	Litoral	1203	2005	33.2	25.1	42.3
Equatorial Guinea	Litoral	1203	2010	27.5	20.6	35.2
Equatorial Guinea	Litoral	1203	2015	21.8	15.8	29.0
Equatorial Guinea	Welenzas	1204	2000	40.2	31.8	50.0
Equatorial Guinea	Welenzas	1204	2005	31.6	24.9	39.3

Admin 0	Admin 1	GAUL Code	Year	Neonatal mortality (per 1,000 livebirths)		
				Estimate	Lower bound, 95% UI	Upper bound, 95% UI
Equatorial Guinea	Welenzas	1204	2010	27.0	20.8	33.9
Equatorial Guinea	Welenzas	1204	2015	22.3	16.6	29.1
Eritrea	Anseba	1205	2000	22.1	18.4	26.1
Eritrea	Anseba	1205	2005	20.9	17.1	25.5
Eritrea	Anseba	1205	2010	18.9	15.1	23.8
Eritrea	Anseba	1205	2015	17.2	13.4	22.1
Eritrea	Archipelagos	1206	2000	26.6	19.7	34.8
Eritrea	Archipelagos	1206	2005	22.8	16.7	30.3
Eritrea	Archipelagos	1206	2010	21.2	15.5	28.6
Eritrea	Archipelagos	1206	2015	18.8	13.2	25.8
Eritrea	Debub	1207	2000	26.9	23.1	30.8
Eritrea	Debub	1207	2005	23.3	19.3	27.8
Eritrea	Debub	1207	2010	21.8	17.4	26.9
Eritrea	Debub	1207	2015	18.9	14.5	24.0
Eritrea	Debubawi Keih Bahri	1208	2000	34.5	27.8	42.2
Eritrea	Debubawi Keih Bahri	1208	2005	29.1	23.0	35.8
Eritrea	Debubawi Keih Bahri	1208	2010	27.0	21.0	33.9
Eritrea	Debubawi Keih Bahri	1208	2015	24.2	18.4	31.1
Eritrea	Gash Barka	1209	2000	28.8	24.9	32.9
Eritrea	Gash Barka	1209	2005	25.5	21.6	29.8
Eritrea	Gash Barka	1209	2010	23.0	18.8	28.0
Eritrea	Gash Barka	1209	2015	20.2	15.8	25.3
Eritrea	Maekel	1210	2000	21.4	17.5	25.5
Eritrea	Maekel	1210	2005	19.5	15.6	24.2
Eritrea	Maekel	1210	2010	19.0	14.6	24.7
Eritrea	Maekel	1210	2015	16.9	12.5	22.3
Eritrea	Semenawi Keih Bahri	1211	2000	23.7	20.0	27.7
Eritrea	Semenawi Keih Bahri	1211	2005	21.5	17.8	25.9
Eritrea	Semenawi Keih Bahri	1211	2010	19.7	15.9	24.1
Eritrea	Semenawi Keih Bahri	1211	2015	17.8	13.9	22.5
Ethiopia	Addis Ababa	1227	2000	25.7	21.4	30.5
Ethiopia	Addis Ababa	1227	2005	23.5	19.5	28.3
Ethiopia	Addis Ababa	1227	2010	20.5	16.3	24.9
Ethiopia	Addis Ababa	1227	2015	16.6	12.6	21.3
Ethiopia	Afar	1228	2000	39.7	35.4	44.5
Ethiopia	Afar	1228	2005	33.4	29.4	37.8
Ethiopia	Afar	1228	2010	25.5	21.9	29.3
Ethiopia	Afar	1228	2015	20.3	16.7	24.2
Ethiopia	Amhara	1229	2000	51.5	47.0	57.0
Ethiopia	Amhara	1229	2005	43.0	38.8	47.7
Ethiopia	Amhara	1229	2010	32.7	28.6	36.9
Ethiopia	Amhara	1229	2015	24.2	20.4	28.5
Ethiopia	Beneshangul Gumu	1230	2000	55.1	49.8	61.5
Ethiopia	Beneshangul Gumu	1230	2005	47.3	42.4	53.0
Ethiopia	Beneshangul Gumu	1230	2010	35.3	30.6	40.4
Ethiopia	Beneshangul Gumu	1230	2015	25.3	20.8	30.2
Ethiopia	Dire Dawa	1231	2000	34.3	29.2	39.9
Ethiopia	Dire Dawa	1231	2005	31.7	27.1	36.7
Ethiopia	Dire Dawa	1231	2010	25.7	21.3	30.5
Ethiopia	Dire Dawa	1231	2015	19.1	14.9	24.0
Ethiopia	Gambela	1232	2000	42.5	37.0	48.4
Ethiopia	Gambela	1232	2005	36.9	31.9	42.4
Ethiopia	Gambela	1232	2010	29.8	24.9	35.1
Ethiopia	Gambela	1232	2015	21.0	16.9	25.8
Ethiopia	Hareri	1233	2000	35.6	30.7	41.0
Ethiopia	Hareri	1233	2005	33.2	28.5	38.3
Ethiopia	Hareri	1233	2010	26.9	22.4	31.9
Ethiopia	Hareri	1233	2015	20.4	16.1	25.5
Ethiopia	Oromia	47678	2000	40.6	37.6	43.9
Ethiopia	Oromia	47678	2005	34.5	31.7	37.5
Ethiopia	Oromia	47678	2010	27.3	24.4	30.5
Ethiopia	Oromia	47678	2015	19.8	17.2	23.1
Ethiopia	SNNPR	1236	2000	38.0	34.1	42.2
Ethiopia	SNNPR	1236	2005	31.8	28.5	35.4
Ethiopia	SNNPR	1236	2010	24.7	21.0	28.7
Ethiopia	SNNPR	1236	2015	18.5	15.2	22.6
Ethiopia	Somali	47679	2000	34.8	30.9	39.3

Admin 0	Admin 1	GAUL Code	Year	Neonatal mortality (per 1,000 livebirths)		
				Estimate	Lower bound, 95% UI	Upper bound, 95% UI
Ethiopia	Somali	47679	2005	32.6	29.0	36.7
Ethiopia	Somali	47679	2010	25.3	22.1	28.8
Ethiopia	Somali	47679	2015	18.7	15.8	21.9
Ethiopia	Tigray	1237	2000	44.7	40.2	49.8
Ethiopia	Tigray	1237	2005	35.3	31.3	39.6
Ethiopia	Tigray	1237	2010	27.8	23.9	32.4
Ethiopia	Tigray	1237	2015	20.9	17.2	25.1
Gabon	Estuaire	1275	2000	29.1	24.1	35.1
Gabon	Estuaire	1275	2005	28.3	23.5	33.9
Gabon	Estuaire	1275	2010	26.7	21.6	32.5
Gabon	Estuaire	1275	2015	22.3	17.1	28.6
Gabon	Haut-Ogooue	1276	2000	21.4	17.5	26.0
Gabon	Haut-Ogooue	1276	2005	20.9	17.1	25.8
Gabon	Haut-Ogooue	1276	2010	17.8	14.3	22.3
Gabon	Haut-Ogooue	1276	2015	16.4	12.7	20.7
Gabon	Moyen-Ogooue	1277	2000	21.4	17.1	26.3
Gabon	Moyen-Ogooue	1277	2005	20.7	16.8	25.2
Gabon	Moyen-Ogooue	1277	2010	18.8	15.0	23.4
Gabon	Moyen-Ogooue	1277	2015	17.6	13.3	22.8
Gabon	Ngounie	1278	2000	18.8	15.5	22.9
Gabon	Ngounie	1278	2005	18.1	14.9	21.8
Gabon	Ngounie	1278	2010	16.1	13.0	19.8
Gabon	Ngounie	1278	2015	15.8	12.3	20.3
Gabon	Nyanga	1279	2000	16.9	13.8	20.7
Gabon	Nyanga	1279	2005	16.8	13.5	20.7
Gabon	Nyanga	1279	2010	14.6	11.8	18.2
Gabon	Nyanga	1279	2015	14.3	11.0	18.4
Gabon	Ogooue-Ivindo	1280	2000	28.2	23.8	33.3
Gabon	Ogooue-Ivindo	1280	2005	27.4	23.1	32.3
Gabon	Ogooue-Ivindo	1280	2010	23.1	19.4	27.1
Gabon	Ogooue-Ivindo	1280	2015	21.9	17.4	26.9
Gabon	Ogooue-Maritime	1282	2000	23.6	17.8	30.5
Gabon	Ogooue-Maritime	1282	2005	23.3	17.8	30.2
Gabon	Ogooue-Maritime	1282	2010	21.8	16.3	28.2
Gabon	Ogooue-Maritime	1282	2015	19.1	14.1	25.2
Gabon	Ogooue-lolo	1281	2000	21.4	17.4	25.8
Gabon	Ogooue-lolo	1281	2005	20.8	17.0	25.2
Gabon	Ogooue-lolo	1281	2010	18.2	14.7	22.3
Gabon	Ogooue-lolo	1281	2015	17.3	13.3	21.8
Gabon	Woleu-Ntem	1283	2000	31.5	25.8	37.9
Gabon	Woleu-Ntem	1283	2005	30.7	25.1	36.5
Gabon	Woleu-Ntem	1283	2010	27.3	21.5	33.0
Gabon	Woleu-Ntem	1283	2015	25.5	19.4	32.1
Gambia	Central River	1285	2000	36.8	33.6	40.1
Gambia	Central River	1285	2005	29.6	27.2	32.1
Gambia	Central River	1285	2010	24.0	21.7	26.5
Gambia	Central River	1285	2015	22.0	19.0	25.5
Gambia	Kanifing Municipal Council	112869	2000	25.0	22.0	28.0
Gambia	Kanifing Municipal Council	112869	2005	24.5	21.7	27.3
Gambia	Kanifing Municipal Council	112869	2010	23.7	20.8	26.9
Gambia	Kanifing Municipal Council	112869	2015	21.3	17.6	25.6
Gambia	Lower River	1287	2000	36.2	33.1	39.6
Gambia	Lower River	1287	2005	30.2	27.8	32.7
Gambia	Lower River	1287	2010	24.6	22.2	27.2
Gambia	Lower River	1287	2015	22.3	18.8	25.8
Gambia	North Bank	1288	2000	33.5	30.8	36.4
Gambia	North Bank	1288	2005	29.2	27.0	31.6
Gambia	North Bank	1288	2010	25.2	22.9	27.7
Gambia	North Bank	1288	2015	22.2	18.8	25.7
Gambia	Upper River	1289	2000	43.8	39.7	48.4
Gambia	Upper River	1289	2005	35.9	32.7	39.3
Gambia	Upper River	1289	2010	28.8	25.7	32.4
Gambia	Upper River	1289	2015	25.8	22.1	29.8
Gambia	West Coast	1290	2000	27.3	24.5	29.9
Gambia	West Coast	1290	2005	26.0	23.5	28.7
Gambia	West Coast	1290	2010	24.4	21.7	27.3
Gambia	West Coast	1290	2015	21.7	18.3	25.8

Admin 0	Admin 1	GAUL Code	Year	Neonatal mortality (per 1,000 livebirths)		
				Estimate	Lower bound, 95% UI	Upper bound, 95% UI
Ghana	Ashanti	1324	2000	33.7	30.7	37.2
Ghana	Ashanti	1324	2005	32.5	29.5	35.7
Ghana	Ashanti	1324	2010	30.8	27.7	34.5
Ghana	Ashanti	1324	2015	24.7	20.8	29.2
Ghana	Brong Ahafo	1325	2000	36.8	33.4	40.2
Ghana	Brong Ahafo	1325	2005	34.8	31.7	38.0
Ghana	Brong Ahafo	1325	2010	30.6	27.5	33.9
Ghana	Brong Ahafo	1325	2015	25.6	21.6	29.8
Ghana	Central	1326	2000	37.4	34.1	41.3
Ghana	Central	1326	2005	33.6	30.6	36.7
Ghana	Central	1326	2010	30.5	27.5	34.0
Ghana	Central	1326	2015	25.9	22.1	30.4
Ghana	Eastern	1327	2000	33.1	30.2	36.2
Ghana	Eastern	1327	2005	30.6	28.0	33.5
Ghana	Eastern	1327	2010	28.4	25.5	31.3
Ghana	Eastern	1327	2015	23.6	20.1	27.7
Ghana	Greater Accra	1328	2000	29.8	26.7	33.2
Ghana	Greater Accra	1328	2005	28.2	25.3	31.2
Ghana	Greater Accra	1328	2010	27.6	24.2	31.3
Ghana	Greater Accra	1328	2015	22.8	19.1	27.3
Ghana	Northern	1329	2000	40.1	36.9	43.6
Ghana	Northern	1329	2005	38.0	35.0	41.2
Ghana	Northern	1329	2010	29.8	27.1	32.9
Ghana	Northern	1329	2015	26.6	22.7	30.6
Ghana	Upper East	1330	2000	37.0	33.7	40.9
Ghana	Upper East	1330	2005	34.6	31.6	37.8
Ghana	Upper East	1330	2010	27.2	24.2	30.3
Ghana	Upper East	1330	2015	23.3	19.7	27.1
Ghana	Upper West	1331	2000	42.6	38.8	46.5
Ghana	Upper West	1331	2005	40.4	36.7	44.2
Ghana	Upper West	1331	2010	32.2	28.9	35.9
Ghana	Upper West	1331	2015	27.1	23.0	31.8
Ghana	Volta	1332	2000	35.0	32.1	38.2
Ghana	Volta	1332	2005	32.7	29.8	35.4
Ghana	Volta	1332	2010	28.7	26.0	31.6
Ghana	Volta	1332	2015	25.0	21.5	29.3
Ghana	Western	1333	2000	38.1	34.5	41.9
Ghana	Western	1333	2005	35.6	32.2	39.2
Ghana	Western	1333	2010	30.5	27.1	34.1
Ghana	Western	1333	2015	26.0	21.8	30.6
Guinea	Boke	40700	2000	46.0	41.1	51.5
Guinea	Boke	40700	2005	44.1	39.4	49.6
Guinea	Boke	40700	2010	38.3	33.7	43.0
Guinea	Boke	40700	2015	32.5	27.6	38.1
Guinea	Conakry	40701	2000	32.9	28.6	37.9
Guinea	Conakry	40701	2005	33.1	28.5	38.1
Guinea	Conakry	40701	2010	32.7	27.7	38.9
Guinea	Conakry	40701	2015	27.2	21.8	33.3
Guinea	Faranah	40702	2000	56.4	51.4	61.6
Guinea	Faranah	40702	2005	49.1	44.7	53.9
Guinea	Faranah	40702	2010	43.2	38.4	48.5
Guinea	Faranah	40702	2015	37.7	32.0	44.2
Guinea	Kankan	40703	2000	53.6	48.7	58.8
Guinea	Kankan	40703	2005	49.0	44.0	54.2
Guinea	Kankan	40703	2010	43.2	38.4	48.7
Guinea	Kankan	40703	2015	37.8	32.0	45.2
Guinea	Kindia	40704	2000	42.4	38.4	46.9
Guinea	Kindia	40704	2005	39.1	35.0	43.2
Guinea	Kindia	40704	2010	35.4	31.2	40.4
Guinea	Kindia	40704	2015	32.4	27.1	38.4
Guinea	Labe	40705	2000	54.9	49.5	60.8
Guinea	Labe	40705	2005	49.7	44.4	55.4
Guinea	Labe	40705	2010	42.8	37.1	48.8
Guinea	Labe	40705	2015	36.7	30.3	43.3
Guinea	Mamou	40706	2000	50.6	45.3	56.4
Guinea	Mamou	40706	2005	45.6	40.5	51.0
Guinea	Mamou	40706	2010	40.3	35.4	45.9

Admin 0	Admin 1	GAUL Code	Year	Neonatal mortality (per 1,000 livebirths)		
				Estimate	Lower bound, 95% UI	Upper bound, 95% UI
Guinea	Mamou	40706	2015	35.5	29.5	42.6
Guinea	Nzerekore	40707	2000	47.8	43.7	51.9
Guinea	Nzerekore	40707	2005	39.6	36.0	43.1
Guinea	Nzerekore	40707	2010	34.6	30.8	38.4
Guinea	Nzerekore	40707	2015	31.4	26.7	36.5
Guinea-Bissau	Bafata	1385	2000	62.5	56.4	69.5
Guinea-Bissau	Bafata	1385	2005	55.5	49.9	61.0
Guinea-Bissau	Bafata	1385	2010	44.6	39.9	49.7
Guinea-Bissau	Bafata	1385	2015	37.1	31.5	43.2
Guinea-Bissau	Biombo	1386	2000	40.2	35.3	45.7
Guinea-Bissau	Biombo	1386	2005	33.7	29.7	38.0
Guinea-Bissau	Biombo	1386	2010	30.3	26.7	34.6
Guinea-Bissau	Biombo	1386	2015	26.1	21.9	30.8
Guinea-Bissau	Bolama/bijagos	1387	2000	38.9	32.3	45.7
Guinea-Bissau	Bolama/bijagos	1387	2005	32.9	27.5	38.9
Guinea-Bissau	Bolama/bijagos	1387	2010	30.6	25.3	36.3
Guinea-Bissau	Bolama/bijagos	1387	2015	26.1	21.2	32.1
Guinea-Bissau	Cacheu	1388	2000	41.3	37.0	46.0
Guinea-Bissau	Cacheu	1388	2005	35.2	31.7	38.9
Guinea-Bissau	Cacheu	1388	2010	31.0	27.7	34.8
Guinea-Bissau	Cacheu	1388	2015	26.0	22.2	30.4
Guinea-Bissau	Gabu	1389	2000	69.1	61.7	76.7
Guinea-Bissau	Gabu	1389	2005	63.4	56.4	70.5
Guinea-Bissau	Gabu	1389	2010	50.6	44.7	57.2
Guinea-Bissau	Gabu	1389	2015	39.6	33.3	46.5
Guinea-Bissau	Oio	1390	2000	50.1	45.2	55.3
Guinea-Bissau	Oio	1390	2005	42.4	38.4	46.5
Guinea-Bissau	Oio	1390	2010	34.7	31.6	38.7
Guinea-Bissau	Oio	1390	2015	28.6	24.5	32.9
Guinea-Bissau	Quinara	1391	2000	47.8	42.4	53.5
Guinea-Bissau	Quinara	1391	2005	41.4	36.8	46.3
Guinea-Bissau	Quinara	1391	2010	34.3	30.6	38.6
Guinea-Bissau	Quinara	1391	2015	30.2	25.5	35.2
Guinea-Bissau	Sector Autonomo De Bissau	1392	2000	36.0	32.1	40.6
Guinea-Bissau	Sector Autonomo De Bissau	1392	2005	32.2	28.8	35.9
Guinea-Bissau	Sector Autonomo De Bissau	1392	2010	29.6	26.4	33.4
Guinea-Bissau	Sector Autonomo De Bissau	1392	2015	26.5	22.5	31.1
Guinea-Bissau	Tombali	1393	2000	47.0	40.9	54.0
Guinea-Bissau	Tombali	1393	2005	41.2	36.1	47.0
Guinea-Bissau	Tombali	1393	2010	34.6	29.9	39.4
Guinea-Bissau	Tombali	1393	2015	31.1	25.8	36.8
Kenya	Central	51325	2000	24.4	23.0	25.8
Kenya	Central	51325	2005	22.6	20.8	24.3
Kenya	Central	51325	2010	21.3	19.1	23.7
Kenya	Central	51325	2015	17.5	15.1	20.2
Kenya	Coast	51326	2000	30.5	28.1	32.9
Kenya	Coast	51326	2005	28.6	26.2	31.2
Kenya	Coast	51326	2010	23.0	20.5	25.5
Kenya	Coast	51326	2015	20.7	17.7	24.1
Kenya	Eastern	51327	2000	25.0	23.6	26.4
Kenya	Eastern	51327	2005	23.1	21.7	24.7
Kenya	Eastern	51327	2010	20.4	18.6	22.3
Kenya	Eastern	51327	2015	17.9	15.8	20.3
Kenya	Ilemi triangle	61033	2000	30.9	24.5	37.8
Kenya	Ilemi triangle	61033	2005	30.5	24.1	37.3
Kenya	Ilemi triangle	61033	2010	27.0	21.1	33.9
Kenya	Ilemi triangle	61033	2015	22.8	17.3	29.3
Kenya	Nairobi	51328	2000	25.7	23.6	28.1
Kenya	Nairobi	51328	2005	24.6	22.3	27.4
Kenya	Nairobi	51328	2010	23.0	20.3	26.1
Kenya	Nairobi	51328	2015	19.5	16.4	23.1
Kenya	North Eastern	51329	2000	28.9	26.4	31.5
Kenya	North Eastern	51329	2005	28.4	25.8	31.0
Kenya	North Eastern	51329	2010	23.2	20.6	26.1
Kenya	North Eastern	51329	2015	18.2	15.5	21.2
Kenya	Nyanza	51330	2000	32.0	30.5	33.5
Kenya	Nyanza	51330	2005	28.6	26.8	30.4

Admin 0	Admin 1	GAUL Code	Year	Neonatal mortality (per 1,000 livebirths)		
				Estimate	Lower bound, 95% UI	Upper bound, 95% UI
Kenya	Nyanza	51330	2010	23.5	21.7	25.3
Kenya	Nyanza	51330	2015	20.2	17.9	22.7
Kenya	Rift Valley	51331	2000	26.1	25.0	27.3
Kenya	Rift Valley	51331	2005	23.4	22.1	24.8
Kenya	Rift Valley	51331	2010	20.3	19.0	21.8
Kenya	Rift Valley	51331	2015	18.0	16.2	20.0
Kenya	Western	51332	2000	29.1	27.5	30.7
Kenya	Western	51332	2005	25.9	24.2	27.6
Kenya	Western	51332	2010	21.9	20.2	23.8
Kenya	Western	51332	2015	18.9	16.6	21.2
Lesotho	Berea	1803	2000	36.7	32.4	41.4
Lesotho	Berea	1803	2005	38.5	34.1	43.2
Lesotho	Berea	1803	2010	34.3	29.7	38.8
Lesotho	Berea	1803	2015	28.4	23.7	33.8
Lesotho	Butha Buthe	1804	2000	37.1	31.9	43.0
Lesotho	Butha Buthe	1804	2005	37.8	32.5	43.5
Lesotho	Butha Buthe	1804	2010	32.7	27.7	38.3
Lesotho	Butha Buthe	1804	2015	28.4	23.1	34.3
Lesotho	Leribe	1805	2000	37.5	33.2	42.6
Lesotho	Leribe	1805	2005	38.7	34.1	43.5
Lesotho	Leribe	1805	2010	33.9	29.4	38.7
Lesotho	Leribe	1805	2015	29.1	24.4	34.4
Lesotho	Mafeteng	1806	2000	35.8	31.1	40.8
Lesotho	Mafeteng	1806	2005	38.2	33.5	43.5
Lesotho	Mafeteng	1806	2010	34.0	29.2	39.3
Lesotho	Mafeteng	1806	2015	28.3	23.2	34.0
Lesotho	Maseru	1807	2000	37.3	33.1	41.7
Lesotho	Maseru	1807	2005	39.3	35.3	44.0
Lesotho	Maseru	1807	2010	34.9	30.3	39.5
Lesotho	Maseru	1807	2015	29.0	24.3	34.2
Lesotho	Mohale's Hoek	1808	2000	38.7	34.3	43.3
Lesotho	Mohale's Hoek	1808	2005	40.3	36.0	45.3
Lesotho	Mohale's Hoek	1808	2010	35.2	31.1	40.1
Lesotho	Mohale's Hoek	1808	2015	29.6	24.8	35.0
Lesotho	Mokhotlong	1809	2000	40.6	35.3	46.7
Lesotho	Mokhotlong	1809	2005	40.3	35.1	45.6
Lesotho	Mokhotlong	1809	2010	34.4	29.2	39.8
Lesotho	Mokhotlong	1809	2015	29.3	24.1	35.3
Lesotho	Qacha's Nek	1810	2000	39.6	34.0	45.5
Lesotho	Qacha's Nek	1810	2005	40.7	35.2	47.0
Lesotho	Qacha's Nek	1810	2010	35.4	30.3	41.4
Lesotho	Qacha's Nek	1810	2015	28.6	23.5	34.4
Lesotho	Quthing	1811	2000	38.1	32.9	43.6
Lesotho	Quthing	1811	2005	39.3	34.1	45.1
Lesotho	Quthing	1811	2010	33.7	28.8	39.5
Lesotho	Quthing	1811	2015	28.7	23.4	34.5
Lesotho	Thaba Tseka	1812	2000	42.7	37.8	48.0
Lesotho	Thaba Tseka	1812	2005	43.2	38.3	48.2
Lesotho	Thaba Tseka	1812	2010	37.3	32.4	42.9
Lesotho	Thaba Tseka	1812	2015	31.0	26.2	36.7
Liberia	Bomi	1814	2000	46.6	42.0	51.1
Liberia	Bomi	1814	2005	35.8	32.2	39.3
Liberia	Bomi	1814	2010	31.4	28.0	35.0
Liberia	Bomi	1814	2015	26.4	21.9	31.2
Liberia	Bong	1815	2000	44.4	39.8	49.3
Liberia	Bong	1815	2005	33.2	29.8	36.5
Liberia	Bong	1815	2010	27.6	24.3	31.2
Liberia	Bong	1815	2015	22.3	18.8	26.5
Liberia	Gbarpolu	1816	2000	47.8	42.9	53.3
Liberia	Gbarpolu	1816	2005	35.8	32.5	39.7
Liberia	Gbarpolu	1816	2010	29.9	26.5	33.8
Liberia	Gbarpolu	1816	2015	25.0	21.2	29.7
Liberia	Grand Bassa	1817	2000	50.0	44.9	55.4
Liberia	Grand Bassa	1817	2005	38.9	34.9	43.1
Liberia	Grand Bassa	1817	2010	31.0	27.3	34.8
Liberia	Grand Bassa	1817	2015	25.2	20.8	30.4
Liberia	Grand Cape Mount	1818	2000	49.7	45.2	54.4

Admin 0	Admin 1	GAUL Code	Year	Neonatal mortality (per 1,000 livebirths)		
				Estimate	Lower bound, 95% UI	Upper bound, 95% UI
Liberia	Grand Cape Mount	1818	2005	37.9	34.4	41.5
Liberia	Grand Cape Mount	1818	2010	32.2	28.8	35.7
Liberia	Grand Cape Mount	1818	2015	27.6	22.9	32.4
Liberia	Grand Gedeh	1819	2000	43.9	38.8	49.6
Liberia	Grand Gedeh	1819	2005	35.5	31.7	40.0
Liberia	Grand Gedeh	1819	2010	28.7	24.9	33.0
Liberia	Grand Gedeh	1819	2015	23.5	19.1	28.3
Liberia	Grand Kru	1820	2000	41.9	36.9	47.6
Liberia	Grand Kru	1820	2005	35.0	31.2	39.2
Liberia	Grand Kru	1820	2010	29.2	25.3	33.6
Liberia	Grand Kru	1820	2015	23.5	19.3	28.7
Liberia	Lofa	1821	2000	45.9	41.2	51.4
Liberia	Lofa	1821	2005	35.1	31.5	39.0
Liberia	Lofa	1821	2010	28.4	24.9	32.1
Liberia	Lofa	1821	2015	23.8	20.1	28.3
Liberia	Margibi	1822	2000	45.9	41.5	50.4
Liberia	Margibi	1822	2005	35.0	31.7	38.2
Liberia	Margibi	1822	2010	30.2	27.0	33.8
Liberia	Margibi	1822	2015	25.6	21.5	30.4
Liberia	Maryland	1823	2000	41.0	35.9	46.4
Liberia	Maryland	1823	2005	34.7	30.8	38.8
Liberia	Maryland	1823	2010	30.0	26.0	34.3
Liberia	Maryland	1823	2015	23.9	19.4	29.1
Liberia	Montserrado	1824	2000	42.8	38.8	47.1
Liberia	Montserrado	1824	2005	35.0	31.7	38.5
Liberia	Montserrado	1824	2010	30.3	26.7	34.0
Liberia	Montserrado	1824	2015	25.8	21.4	31.0
Liberia	Nimba	1825	2000	40.2	35.6	45.1
Liberia	Nimba	1825	2005	31.0	27.6	34.6
Liberia	Nimba	1825	2010	24.9	21.9	28.2
Liberia	Nimba	1825	2015	20.6	16.9	24.8
Liberia	River Gee	1827	2000	44.6	39.4	50.3
Liberia	River Gee	1827	2005	36.9	32.9	41.3
Liberia	River Gee	1827	2010	30.6	26.7	35.2
Liberia	River Gee	1827	2015	24.2	19.8	29.5
Liberia	Rivercess	1826	2000	49.8	43.9	56.0
Liberia	Rivercess	1826	2005	39.3	34.5	44.0
Liberia	Rivercess	1826	2010	30.9	27.0	35.1
Liberia	Rivercess	1826	2015	24.9	20.5	30.2
Liberia	Sinoe	1828	2000	43.1	37.7	49.0
Liberia	Sinoe	1828	2005	34.7	30.7	39.2
Liberia	Sinoe	1828	2010	28.9	25.3	33.2
Liberia	Sinoe	1828	2015	23.1	18.8	28.1
Madagascar	Alaotra Mangoro	41750	2000	32.7	28.6	37.5
Madagascar	Alaotra Mangoro	41750	2005	30.2	26.4	34.1
Madagascar	Alaotra Mangoro	41750	2010	28.7	24.8	33.3
Madagascar	Alaotra Mangoro	41750	2015	25.7	21.2	30.6
Madagascar	Amoron I Mania	41751	2000	33.4	29.4	37.9
Madagascar	Amoron I Mania	41751	2005	31.9	28.0	36.3
Madagascar	Amoron I Mania	41751	2010	30.2	25.8	35.0
Madagascar	Amoron I Mania	41751	2015	27.6	22.5	33.2
Madagascar	Analamanga	41752	2000	29.7	26.1	33.9
Madagascar	Analamanga	41752	2005	28.8	25.4	32.4
Madagascar	Analamanga	41752	2010	28.7	24.7	32.9
Madagascar	Analamanga	41752	2015	27.3	22.4	32.7
Madagascar	Analanjirofo	41753	2000	32.7	27.1	38.4
Madagascar	Analanjirofo	41753	2005	29.7	25.4	34.6
Madagascar	Analanjirofo	41753	2010	27.4	22.8	32.5
Madagascar	Analanjirofo	41753	2015	24.1	19.6	29.7
Madagascar	Androy	41754	2000	34.2	29.9	38.7
Madagascar	Androy	41754	2005	30.9	27.6	34.9
Madagascar	Androy	41754	2010	31.0	27.1	35.3
Madagascar	Androy	41754	2015	28.8	23.7	34.4
Madagascar	Anosy	41755	2000	37.3	32.7	42.2
Madagascar	Anosy	41755	2005	33.8	30.0	38.3
Madagascar	Anosy	41755	2010	33.2	28.9	38.1
Madagascar	Anosy	41755	2015	31.6	26.4	38.0

Admin 0	Admin 1	GAUL Code	Year	Neonatal mortality (per 1,000 livebirths)		
				Estimate	Lower bound, 95% UI	Upper bound, 95% UI
Madagascar	Atsimo Andrefana	41756	2000	32.4	28.1	37.2
Madagascar	Atsimo Andrefana	41756	2005	28.7	25.2	32.6
Madagascar	Atsimo Andrefana	41756	2010	28.9	25.0	33.0
Madagascar	Atsimo Andrefana	41756	2015	27.6	23.2	32.8
Madagascar	Atsimo Atsinanana	41757	2000	41.2	35.7	46.9
Madagascar	Atsimo Atsinanana	41757	2005	37.4	32.7	42.1
Madagascar	Atsimo Atsinanana	41757	2010	35.4	30.7	40.7
Madagascar	Atsimo Atsinanana	41757	2015	33.8	28.1	40.6
Madagascar	Atsinanana	41758	2000	33.2	28.8	38.2
Madagascar	Atsinanana	41758	2005	31.7	27.5	36.1
Madagascar	Atsinanana	41758	2010	29.5	25.1	34.2
Madagascar	Atsinanana	41758	2015	26.3	21.7	31.5
Madagascar	Betsiboka	41759	2000	36.6	31.0	43.3
Madagascar	Betsiboka	41759	2005	33.5	28.5	39.5
Madagascar	Betsiboka	41759	2010	32.7	27.5	38.9
Madagascar	Betsiboka	41759	2015	29.2	23.8	35.4
Madagascar	Boeny	41760	2000	38.4	32.4	45.3
Madagascar	Boeny	41760	2005	34.6	29.5	40.9
Madagascar	Boeny	41760	2010	32.9	27.1	39.2
Madagascar	Boeny	41760	2015	29.4	23.5	36.4
Madagascar	Bongolava	41761	2000	33.4	27.7	39.1
Madagascar	Bongolava	41761	2005	31.3	26.5	36.6
Madagascar	Bongolava	41761	2010	29.5	24.8	35.1
Madagascar	Bongolava	41761	2015	28.1	22.7	34.6
Madagascar	Diana	41762	2000	30.5	24.9	37.0
Madagascar	Diana	41762	2005	28.3	23.3	34.3
Madagascar	Diana	41762	2010	27.1	21.8	33.3
Madagascar	Diana	41762	2015	24.9	19.6	32.2
Madagascar	Haute Matsiatra	41763	2000	37.3	32.9	42.6
Madagascar	Haute Matsiatra	41763	2005	33.9	29.8	38.3
Madagascar	Haute Matsiatra	41763	2010	33.4	28.5	38.7
Madagascar	Haute Matsiatra	41763	2015	29.8	24.3	35.8
Madagascar	Ihorombe	41764	2000	36.9	31.7	42.4
Madagascar	Ihorombe	41764	2005	33.4	28.9	38.6
Madagascar	Ihorombe	41764	2010	32.9	28.0	38.7
Madagascar	Ihorombe	41764	2015	30.6	25.0	37.0
Madagascar	Itasy	41765	2000	33.0	28.7	37.9
Madagascar	Itasy	41765	2005	31.1	27.2	35.1
Madagascar	Itasy	41765	2010	30.9	26.5	35.7
Madagascar	Itasy	41765	2015	28.1	22.9	33.7
Madagascar	Melaky	41766	2000	32.4	26.2	39.4
Madagascar	Melaky	41766	2005	29.2	23.8	35.4
Madagascar	Melaky	41766	2010	27.2	21.6	33.4
Madagascar	Melaky	41766	2015	26.0	20.3	32.7
Madagascar	Menabe	41767	2000	34.4	28.9	40.9
Madagascar	Menabe	41767	2005	31.8	26.3	37.7
Madagascar	Menabe	41767	2010	29.9	24.8	35.7
Madagascar	Menabe	41767	2015	28.3	22.8	34.7
Madagascar	Sava	41768	2000	29.2	23.9	35.4
Madagascar	Sava	41768	2005	28.2	23.4	33.6
Madagascar	Sava	41768	2010	25.9	21.4	31.2
Madagascar	Sava	41768	2015	24.4	19.1	30.5
Madagascar	Sofia	41769	2000	30.3	25.6	35.4
Madagascar	Sofia	41769	2005	28.2	24.1	32.8
Madagascar	Sofia	41769	2010	26.8	22.2	31.9
Madagascar	Sofia	41769	2015	25.5	20.2	31.2
Madagascar	Vakinankaratra	41770	2000	32.0	28.1	36.3
Madagascar	Vakinankaratra	41770	2005	30.1	26.3	34.0
Madagascar	Vakinankaratra	41770	2010	29.9	25.7	34.4
Madagascar	Vakinankaratra	41770	2015	26.9	21.9	32.4
Madagascar	Vatovavy Fitovinany	41771	2000	38.7	33.8	44.2
Madagascar	Vatovavy Fitovinany	41771	2005	35.8	31.5	40.9
Madagascar	Vatovavy Fitovinany	41771	2010	33.7	29.0	39.3
Madagascar	Vatovavy Fitovinany	41771	2015	30.9	25.5	37.2
Malawi	Area under National Administration	65268	2000	35.4	33.4	37.5
Malawi	Area under National Administration	65268	2005	30.7	29.0	32.5
Malawi	Area under National Administration	65268	2010	27.4	25.7	29.3

Admin 0	Admin 1	GAUL Code	Year	Neonatal mortality (per 1,000 livebirths)		
				Estimate	Lower bound, 95% UI	Upper bound, 95% UI
Malawi	Area under National Administration	65268	2015	23.3	21.2	25.6
Malawi	Central Region	1888	2000	38.3	36.6	40.2
Malawi	Central Region	1888	2005	33.4	31.8	35.1
Malawi	Central Region	1888	2010	30.0	28.2	31.8
Malawi	Central Region	1888	2015	26.1	23.5	28.8
Malawi	Northern Region	1889	2000	37.3	35.2	39.6
Malawi	Northern Region	1889	2005	33.7	31.7	36.0
Malawi	Northern Region	1889	2010	29.5	27.3	32.1
Malawi	Northern Region	1889	2015	24.3	21.6	27.4
Malawi	Southern Region	1890	2000	41.8	40.3	43.4
Malawi	Southern Region	1890	2005	33.6	32.2	35.1
Malawi	Southern Region	1890	2010	30.6	29.1	32.3
Malawi	Southern Region	1890	2015	26.1	23.6	28.7
Mali	Bamako	1926	2000	40.0	36.4	44.0
Mali	Bamako	1926	2005	36.6	33.2	40.3
Mali	Bamako	1926	2010	36.1	32.4	40.0
Mali	Bamako	1926	2015	33.5	28.5	39.3
Mali	Gao	1927	2000	44.2	38.9	50.9
Mali	Gao	1927	2005	41.5	35.8	47.8
Mali	Gao	1927	2010	41.4	34.8	49.3
Mali	Gao	1927	2015	39.5	32.2	48.7
Mali	Kayes	1928	2000	55.9	51.8	60.3
Mali	Kayes	1928	2005	49.8	46.0	53.6
Mali	Kayes	1928	2010	43.8	40.0	48.1
Mali	Kayes	1928	2015	40.0	34.5	46.0
Mali	Kidal	1929	2000	29.1	22.6	37.4
Mali	Kidal	1929	2005	27.1	20.4	35.1
Mali	Kidal	1929	2010	27.2	20.2	35.8
Mali	Kidal	1929	2015	27.3	20.0	36.0
Mali	Koulikoro	1930	2000	50.9	47.9	54.0
Mali	Koulikoro	1930	2005	45.0	42.2	48.2
Mali	Koulikoro	1930	2010	40.9	37.8	44.2
Mali	Koulikoro	1930	2015	38.0	33.4	43.2
Mali	Mopti	1931	2000	55.4	51.8	59.2
Mali	Mopti	1931	2005	50.3	46.8	54.1
Mali	Mopti	1931	2010	43.9	40.1	48.1
Mali	Mopti	1931	2015	40.4	35.0	46.4
Mali	Segou	1932	2000	55.8	52.2	59.7
Mali	Segou	1932	2005	50.1	46.6	53.9
Mali	Segou	1932	2010	43.1	39.2	47.0
Mali	Segou	1932	2015	39.6	34.4	45.6
Mali	Sikasso	1933	2000	60.0	56.6	63.6
Mali	Sikasso	1933	2005	53.9	50.4	57.5
Mali	Sikasso	1933	2010	47.7	44.0	51.6
Mali	Sikasso	1933	2015	42.3	36.7	48.0
Mali	Tombouctou	1934	2000	55.6	49.4	61.6
Mali	Tombouctou	1934	2005	51.1	45.0	57.5
Mali	Tombouctou	1934	2010	47.6	41.1	55.1
Mali	Tombouctou	1934	2015	44.9	36.8	54.4
Mauritania	Adrar	2004	2000	35.9	30.1	42.8
Mauritania	Adrar	2004	2005	36.7	30.5	43.4
Mauritania	Adrar	2004	2010	32.0	25.6	38.8
Mauritania	Adrar	2004	2015	30.5	23.7	38.5
Mauritania	Assaba	2005	2000	36.8	32.7	41.0
Mauritania	Assaba	2005	2005	33.3	29.8	37.1
Mauritania	Assaba	2005	2010	28.4	24.2	32.5
Mauritania	Assaba	2005	2015	25.0	20.6	30.1
Mauritania	Brakna	2006	2000	39.2	35.4	43.5
Mauritania	Brakna	2006	2005	36.0	32.4	39.6
Mauritania	Brakna	2006	2010	30.2	26.3	34.3
Mauritania	Brakna	2006	2015	24.5	19.9	29.1
Mauritania	Dakhlet-Nouadhibou	2007	2000	33.4	27.6	40.3
Mauritania	Dakhlet-Nouadhibou	2007	2005	33.6	27.3	40.8
Mauritania	Dakhlet-Nouadhibou	2007	2010	28.8	23.3	35.3
Mauritania	Dakhlet-Nouadhibou	2007	2015	25.6	19.9	32.5
Mauritania	Gorgol	2008	2000	39.8	36.4	43.6
Mauritania	Gorgol	2008	2005	35.4	32.1	38.8

Admin 0	Admin 1	GAUL Code	Year	Neonatal mortality (per 1,000 livebirths)		
				Estimate	Lower bound, 95% UI	Upper bound, 95% UI
Mauritania	Gorgol	2008	2010	29.6	25.9	33.4
Mauritania	Gorgol	2008	2015	25.3	21.0	29.8
Mauritania	Guidimakha	2009	2000	39.8	35.8	44.3
Mauritania	Guidimakha	2009	2005	35.2	31.8	38.9
Mauritania	Guidimakha	2009	2010	28.8	25.2	32.8
Mauritania	Guidimakha	2009	2015	24.4	20.3	29.1
Mauritania	Hodh Ech Chargi	2010	2000	40.8	35.9	46.1
Mauritania	Hodh Ech Chargi	2010	2005	35.9	31.6	41.1
Mauritania	Hodh Ech Chargi	2010	2010	31.0	26.5	36.0
Mauritania	Hodh Ech Chargi	2010	2015	27.6	22.6	33.0
Mauritania	Hodh El Gharbi	2011	2000	32.3	28.5	37.1
Mauritania	Hodh El Gharbi	2011	2005	29.0	25.6	33.0
Mauritania	Hodh El Gharbi	2011	2010	24.9	21.4	28.8
Mauritania	Hodh El Gharbi	2011	2015	22.1	18.2	26.4
Mauritania	Inchiri	2012	2000	35.5	28.3	44.3
Mauritania	Inchiri	2012	2005	36.1	28.5	45.0
Mauritania	Inchiri	2012	2010	31.9	24.8	40.6
Mauritania	Inchiri	2012	2015	29.9	22.4	38.9
Mauritania	Nouakchott	2013	2000	36.6	31.6	42.2
Mauritania	Nouakchott	2013	2005	36.2	31.0	41.6
Mauritania	Nouakchott	2013	2010	32.3	27.2	38.1
Mauritania	Nouakchott	2013	2015	27.3	21.8	33.6
Mauritania	Tagant	2014	2000	32.6	27.6	38.3
Mauritania	Tagant	2014	2005	31.5	26.5	37.4
Mauritania	Tagant	2014	2010	27.1	21.9	33.1
Mauritania	Tagant	2014	2015	25.6	20.2	31.6
Mauritania	Tiris-Zemmour	2015	2000	36.5	28.8	45.6
Mauritania	Tiris-Zemmour	2015	2005	36.3	28.4	46.0
Mauritania	Tiris-Zemmour	2015	2010	32.5	24.9	41.6
Mauritania	Tiris-Zemmour	2015	2015	31.7	23.7	41.9
Mauritania	Trarza	2016	2000	34.8	31.3	38.5
Mauritania	Trarza	2016	2005	33.6	30.4	37.4
Mauritania	Trarza	2016	2010	29.6	26.3	33.3
Mauritania	Trarza	2016	2015	23.8	20.0	28.4
Morocco	Chaouia - Ouardigha	147326	2000	23.0	19.1	27.5
Morocco	Chaouia - Ouardigha	147326	2005	19.6	16.3	23.4
Morocco	Chaouia - Ouardigha	147326	2010	17.4	14.0	21.4
Morocco	Chaouia - Ouardigha	147326	2015	14.4	11.2	18.5
Morocco	Doukkala - Abda	147327	2000	23.2	18.6	28.5
Morocco	Doukkala - Abda	147327	2005	20.3	16.1	25.0
Morocco	Doukkala - Abda	147327	2010	16.5	12.8	20.9
Morocco	Doukkala - Abda	147327	2015	13.8	10.4	18.1
Morocco	Fès - Boulemane	147328	2000	24.4	20.0	29.7
Morocco	Fès - Boulemane	147328	2005	20.9	17.2	25.0
Morocco	Fès - Boulemane	147328	2010	17.7	13.9	22.3
Morocco	Fès - Boulemane	147328	2015	14.8	11.1	19.0
Morocco	Gharb - Chrarda - Béni Hssen	147329	2000	24.5	20.1	29.4
Morocco	Gharb - Chrarda - Béni Hssen	147329	2005	20.1	16.6	24.3
Morocco	Gharb - Chrarda - Béni Hssen	147329	2010	16.6	13.1	20.8
Morocco	Gharb - Chrarda - Béni Hssen	147329	2015	14.0	10.4	18.1
Morocco	Grand Casablanca	147330	2000	20.2	15.9	25.3
Morocco	Grand Casablanca	147330	2005	17.8	14.2	22.4
Morocco	Grand Casablanca	147330	2010	16.0	12.3	20.5
Morocco	Grand Casablanca	147330	2015	13.5	10.1	18.1
Morocco	Guelmim - Es-Semara	147331	2000	25.2	19.9	31.8
Morocco	Guelmim - Es-Semara	147331	2005	22.0	17.2	27.7
Morocco	Guelmim - Es-Semara	147331	2010	18.4	14.3	23.5
Morocco	Guelmim - Es-Semara	147331	2015	15.5	11.7	20.2
Morocco	Laâyoune - Boujdour - Sakia El Hamra	147332	2000	27.6	18.6	39.7
Morocco	Laâyoune - Boujdour - Sakia El Hamra	147332	2005	25.8	17.4	37.1
Morocco	Laâyoune - Boujdour - Sakia El Hamra	147332	2010	22.9	15.6	33.2
Morocco	Laâyoune - Boujdour - Sakia El Hamra	147332	2015	18.4	12.6	26.3
Morocco	Marrakech - Tensift - Al Haouz	147333	2000	23.9	19.8	28.6
Morocco	Marrakech - Tensift - Al Haouz	147333	2005	20.8	16.9	25.1
Morocco	Marrakech - Tensift - Al Haouz	147333	2010	16.5	13.1	20.6
Morocco	Marrakech - Tensift - Al Haouz	147333	2015	13.8	10.6	17.9
Morocco	Meknès - Tafilalet	2107	2000	25.7	21.3	30.3

Admin 0	Admin 1	GAUL Code	Year	Neonatal mortality (per 1,000 livebirths)		
				Estimate	Lower bound, 95% UI	Upper bound, 95% UI
Morocco	Meknès - Tafilalet	2107	2005	21.7	18.0	26.0
Morocco	Meknès - Tafilalet	2107	2010	18.0	14.5	22.1
Morocco	Meknès - Tafilalet	2107	2015	15.3	11.8	19.1
Morocco	Oriental	2109	2000	24.1	19.0	29.7
Morocco	Oriental	2109	2005	20.0	15.8	24.8
Morocco	Oriental	2109	2010	17.5	13.5	21.9
Morocco	Oriental	2109	2015	14.6	11.0	18.8
Morocco	Rabat - Salé - Zemmour - Zaer	147334	2000	20.7	16.9	25.0
Morocco	Rabat - Salé - Zemmour - Zaer	147334	2005	17.9	14.6	21.8
Morocco	Rabat - Salé - Zemmour - Zaer	147334	2010	16.1	12.6	20.0
Morocco	Rabat - Salé - Zemmour - Zaer	147334	2015	13.5	10.2	17.5
Morocco	Souss - Massa - Draâ	147335	2000	24.6	20.5	29.8
Morocco	Souss - Massa - Draâ	147335	2005	21.5	17.8	25.9
Morocco	Souss - Massa - Draâ	147335	2010	17.0	13.8	20.8
Morocco	Souss - Massa - Draâ	147335	2015	14.3	11.2	17.9
Morocco	Tadla - Azilal	147336	2000	26.1	21.1	32.2
Morocco	Tadla - Azilal	147336	2005	21.5	17.1	26.5
Morocco	Tadla - Azilal	147336	2010	17.1	13.2	21.8
Morocco	Tadla - Azilal	147336	2015	14.7	11.1	19.2
Morocco	Tanger - Tétouan	147337	2000	27.1	21.9	33.1
Morocco	Tanger - Tétouan	147337	2005	22.6	18.2	27.9
Morocco	Tanger - Tétouan	147337	2010	19.3	14.6	24.7
Morocco	Tanger - Tétouan	147337	2015	15.7	11.7	20.7
Morocco	Taza - Al Hoceima - Taounate	147338	2000	26.7	21.9	32.4
Morocco	Taza - Al Hoceima - Taounate	147338	2005	22.4	18.3	26.7
Morocco	Taza - Al Hoceima - Taounate	147338	2010	17.9	14.3	22.0
Morocco	Taza - Al Hoceima - Taounate	147338	2015	15.0	11.6	19.2
Mozambique	Cabo Delgado	2112	2000	46.8	42.8	51.2
Mozambique	Cabo Delgado	2112	2005	38.2	35.0	41.8
Mozambique	Cabo Delgado	2112	2010	30.2	26.4	34.3
Mozambique	Cabo Delgado	2112	2015	26.6	22.3	31.6
Mozambique	Gaza	2113	2000	34.4	31.2	38.2
Mozambique	Gaza	2113	2005	32.0	28.9	35.5
Mozambique	Gaza	2113	2010	30.1	25.9	34.5
Mozambique	Gaza	2113	2015	24.3	20.1	29.1
Mozambique	Inhambane	2114	2000	33.0	29.4	37.3
Mozambique	Inhambane	2114	2005	30.7	27.2	34.3
Mozambique	Inhambane	2114	2010	28.5	24.4	33.3
Mozambique	Inhambane	2114	2015	23.2	18.8	28.1
Mozambique	Manica	2115	2000	36.2	33.1	39.7
Mozambique	Manica	2115	2005	33.8	31.0	36.9
Mozambique	Manica	2115	2010	30.7	27.4	34.4
Mozambique	Manica	2115	2015	26.7	23.1	31.3
Mozambique	Maputo	41373	2000	31.2	28.5	34.6
Mozambique	Maputo	41373	2005	28.7	26.0	31.6
Mozambique	Maputo	41373	2010	28.1	24.4	31.9
Mozambique	Maputo	41373	2015	22.1	18.3	26.7
Mozambique	Nampula	2118	2000	43.0	39.1	46.9
Mozambique	Nampula	2118	2005	35.8	32.7	39.3
Mozambique	Nampula	2118	2010	28.2	24.3	32.4
Mozambique	Nampula	2118	2015	24.5	20.3	29.4
Mozambique	Niassa	2119	2000	41.2	37.7	44.9
Mozambique	Niassa	2119	2005	33.6	30.9	36.7
Mozambique	Niassa	2119	2010	28.5	25.5	31.6
Mozambique	Niassa	2119	2015	24.7	21.4	28.4
Mozambique	Sofala	2120	2000	41.4	37.8	45.4
Mozambique	Sofala	2120	2005	35.7	32.7	39.2
Mozambique	Sofala	2120	2010	32.1	28.4	36.2
Mozambique	Sofala	2120	2015	27.3	23.3	32.2
Mozambique	Tete	2121	2000	43.2	40.4	46.1
Mozambique	Tete	2121	2005	37.2	34.5	39.9
Mozambique	Tete	2121	2010	33.1	30.3	36.1
Mozambique	Tete	2121	2015	28.3	25.0	31.9
Mozambique	Zambezia	2122	2000	50.0	46.3	54.1
Mozambique	Zambezia	2122	2005	40.3	37.2	43.6
Mozambique	Zambezia	2122	2010	33.7	30.3	37.4
Mozambique	Zambezia	2122	2015	29.6	25.4	34.2

Admin 0	Admin 1	GAUL Code	Year	Neonatal mortality (per 1,000 livebirths)		
				Estimate	Lower bound, 95% UI	Upper bound, 95% UI
Namibia	Caprivi	2137	2000	30.5	25.1	36.7
Namibia	Caprivi	2137	2005	31.0	25.5	36.8
Namibia	Caprivi	2137	2010	29.2	23.7	35.5
Namibia	Caprivi	2137	2015	23.2	17.8	29.3
Namibia	Erongo	2138	2000	16.7	12.9	21.2
Namibia	Erongo	2138	2005	18.0	14.0	22.8
Namibia	Erongo	2138	2010	18.1	13.9	23.6
Namibia	Erongo	2138	2015	14.9	11.0	19.8
Namibia	Hardap	2139	2000	18.0	14.3	22.5
Namibia	Hardap	2139	2005	19.5	15.4	24.3
Namibia	Hardap	2139	2010	18.6	14.5	23.5
Namibia	Hardap	2139	2015	15.4	11.6	20.0
Namibia	Karas	2140	2000	17.2	13.3	22.0
Namibia	Karas	2140	2005	19.1	14.8	24.1
Namibia	Karas	2140	2010	18.7	14.4	23.7
Namibia	Karas	2140	2015	15.8	12.1	20.7
Namibia	Kavango	2141	2000	23.7	19.2	28.7
Namibia	Kavango	2141	2005	24.2	19.7	29.2
Namibia	Kavango	2141	2010	22.4	18.1	27.3
Namibia	Kavango	2141	2015	18.5	14.6	23.4
Namibia	Khomas	2142	2000	18.2	14.2	22.7
Namibia	Khomas	2142	2005	19.2	15.0	24.0
Namibia	Khomas	2142	2010	19.0	14.6	24.2
Namibia	Khomas	2142	2015	15.3	11.6	19.6
Namibia	Kunene	2143	2000	21.3	17.6	26.0
Namibia	Kunene	2143	2005	21.7	17.8	26.3
Namibia	Kunene	2143	2010	20.5	16.6	25.1
Namibia	Kunene	2143	2015	16.6	13.2	20.4
Namibia	Ohangwena	2144	2000	23.9	20.1	28.3
Namibia	Ohangwena	2144	2005	24.7	20.8	29.1
Namibia	Ohangwena	2144	2010	21.9	17.9	26.8
Namibia	Ohangwena	2144	2015	18.4	14.6	23.6
Namibia	Omaheke	2145	2000	23.1	18.8	28.3
Namibia	Omaheke	2145	2005	23.8	19.2	29.0
Namibia	Omaheke	2145	2010	22.1	17.6	27.9
Namibia	Omaheke	2145	2015	18.1	14.2	23.3
Namibia	Omusati	2146	2000	22.6	18.8	27.1
Namibia	Omusati	2146	2005	23.2	19.4	27.8
Namibia	Omusati	2146	2010	21.1	17.1	26.3
Namibia	Omusati	2146	2015	17.3	13.6	21.8
Namibia	Oshana	2147	2000	22.6	18.9	27.0
Namibia	Oshana	2147	2005	23.3	19.4	27.7
Namibia	Oshana	2147	2010	21.2	17.2	26.2
Namibia	Oshana	2147	2015	17.8	13.8	22.8
Namibia	Oshikoto	2148	2000	22.0	18.6	25.9
Namibia	Oshikoto	2148	2005	22.9	19.2	27.3
Namibia	Oshikoto	2148	2010	21.2	17.5	25.9
Namibia	Oshikoto	2148	2015	17.3	13.6	21.9
Namibia	Otjozondjupa	2149	2000	19.5	15.9	23.3
Namibia	Otjozondjupa	2149	2005	20.3	16.7	24.0
Namibia	Otjozondjupa	2149	2010	19.2	15.8	23.6
Namibia	Otjozondjupa	2149	2015	15.7	12.5	19.3
Niger	Agadez	2202	2000	24.7	20.5	29.7
Niger	Agadez	2202	2005	22.8	18.9	27.7
Niger	Agadez	2202	2010	22.5	18.1	27.5
Niger	Agadez	2202	2015	18.9	14.7	23.8
Niger	Diffa	2203	2000	36.0	30.0	42.7
Niger	Diffa	2203	2005	29.5	24.7	35.3
Niger	Diffa	2203	2010	30.2	25.1	36.6
Niger	Diffa	2203	2015	23.9	19.1	29.7
Niger	Dosso	2204	2000	44.9	40.5	49.5
Niger	Dosso	2204	2005	39.8	36.0	43.7
Niger	Dosso	2204	2010	38.3	33.7	42.9
Niger	Dosso	2204	2015	31.3	26.1	37.0
Niger	Maradi	2205	2000	48.7	44.6	53.4
Niger	Maradi	2205	2005	40.2	36.6	44.1
Niger	Maradi	2205	2010	32.2	28.5	35.9

Admin 0	Admin 1	GAUL Code	Year	Neonatal mortality (per 1,000 livebirths)		
				Estimate	Lower bound, 95% UI	Upper bound, 95% UI
Niger	Maradi	2205	2015	28.0	23.6	33.0
Niger	Niamey	2206	2000	32.5	28.6	36.9
Niger	Niamey	2206	2005	30.2	26.5	34.3
Niger	Niamey	2206	2010	31.5	26.8	36.2
Niger	Niamey	2206	2015	26.8	21.8	32.8
Niger	Tahoua	2207	2000	41.6	37.3	46.5
Niger	Tahoua	2207	2005	35.3	31.5	39.2
Niger	Tahoua	2207	2010	30.2	26.5	34.1
Niger	Tahoua	2207	2015	26.6	21.9	31.6
Niger	Tillaberi	2208	2000	38.9	35.4	42.6
Niger	Tillaberi	2208	2005	34.0	30.6	37.5
Niger	Tillaberi	2208	2010	32.2	28.4	36.0
Niger	Tillaberi	2208	2015	28.1	23.7	33.0
Niger	Zinder	2209	2000	44.1	39.6	49.2
Niger	Zinder	2209	2005	36.9	33.1	41.0
Niger	Zinder	2209	2010	25.3	21.8	29.3
Niger	Zinder	2209	2015	26.2	22.0	31.2
Nigeria	Abia	65698	2000	44.6	40.3	49.5
Nigeria	Abia	65698	2005	42.6	38.3	47.0
Nigeria	Abia	65698	2010	38.2	34.0	43.4
Nigeria	Abia	65698	2015	30.9	25.9	36.7
Nigeria	Abuja	2221	2000	45.9	40.2	52.1
Nigeria	Abuja	2221	2005	43.5	38.3	49.5
Nigeria	Abuja	2221	2010	41.2	35.7	47.5
Nigeria	Abuja	2221	2015	35.3	29.0	42.4
Nigeria	Adamawa	2211	2000	59.6	54.4	65.5
Nigeria	Adamawa	2211	2005	57.7	52.6	63.2
Nigeria	Adamawa	2211	2010	48.2	43.3	53.9
Nigeria	Adamawa	2211	2015	46.5	39.7	53.8
Nigeria	Akwa Ibom	2212	2000	42.3	37.3	47.7
Nigeria	Akwa Ibom	2212	2005	41.2	36.4	46.1
Nigeria	Akwa Ibom	2212	2010	36.3	31.3	41.5
Nigeria	Akwa Ibom	2212	2015	31.5	25.8	37.7
Nigeria	Anambra	2213	2000	43.4	38.5	48.5
Nigeria	Anambra	2213	2005	38.7	34.4	43.0
Nigeria	Anambra	2213	2010	37.8	33.1	42.7
Nigeria	Anambra	2213	2015	30.0	24.8	35.5
Nigeria	Bauchi	65699	2000	63.5	58.2	68.8
Nigeria	Bauchi	65699	2005	59.0	54.3	63.8
Nigeria	Bauchi	65699	2010	52.0	46.9	56.8
Nigeria	Bauchi	65699	2015	48.1	40.9	55.7
Nigeria	Bayelsa	65700	2000	49.6	44.0	55.9
Nigeria	Bayelsa	65700	2005	45.6	40.6	50.9
Nigeria	Bayelsa	65700	2010	43.8	38.0	49.9
Nigeria	Bayelsa	65700	2015	38.4	31.8	45.2
Nigeria	Benue	2215	2000	51.8	46.9	57.5
Nigeria	Benue	2215	2005	47.1	42.4	52.0
Nigeria	Benue	2215	2010	42.2	37.3	47.4
Nigeria	Benue	2215	2015	38.8	32.6	46.0
Nigeria	Borno	2216	2000	47.6	42.5	52.6
Nigeria	Borno	2216	2005	45.2	40.8	50.3
Nigeria	Borno	2216	2010	39.5	35.0	44.4
Nigeria	Borno	2216	2015	37.8	32.0	44.9
Nigeria	Cross River	2217	2000	47.8	43.2	52.5
Nigeria	Cross River	2217	2005	45.6	41.4	50.1
Nigeria	Cross River	2217	2010	39.5	35.2	44.6
Nigeria	Cross River	2217	2015	34.6	29.3	40.9
Nigeria	Delta	2218	2000	44.4	39.5	49.9
Nigeria	Delta	2218	2005	39.6	35.1	44.1
Nigeria	Delta	2218	2010	40.2	35.5	45.2
Nigeria	Delta	2218	2015	34.2	28.8	40.3
Nigeria	Ebonyi	65701	2000	56.9	51.1	63.6
Nigeria	Ebonyi	65701	2005	51.0	46.1	56.5
Nigeria	Ebonyi	65701	2010	45.2	39.9	51.3
Nigeria	Ebonyi	65701	2015	37.7	31.5	44.9
Nigeria	Edo	2219	2000	41.9	36.8	47.3
Nigeria	Edo	2219	2005	37.7	33.8	42.2

Admin 0	Admin 1	GAUL Code	Year	Neonatal mortality (per 1,000 livebirths)		
				Estimate	Lower bound, 95% UI	Upper bound, 95% UI
Nigeria	Edo	2219	2010	36.7	32.1	41.8
Nigeria	Edo	2219	2015	32.0	26.8	38.7
Nigeria	Ekiti	65702	2000	36.5	32.2	41.2
Nigeria	Ekiti	65702	2005	34.7	30.6	39.1
Nigeria	Ekiti	65702	2010	33.0	28.5	38.0
Nigeria	Ekiti	65702	2015	28.7	23.9	34.6
Nigeria	Enugu	65703	2000	44.7	40.1	50.0
Nigeria	Enugu	65703	2005	39.8	35.7	44.5
Nigeria	Enugu	65703	2010	39.6	35.0	44.5
Nigeria	Enugu	65703	2015	31.5	26.4	37.3
Nigeria	Gombe	65704	2000	59.4	53.7	65.3
Nigeria	Gombe	65704	2005	56.2	51.2	61.6
Nigeria	Gombe	65704	2010	48.6	43.4	54.3
Nigeria	Gombe	65704	2015	46.0	39.0	53.6
Nigeria	Imo	2222	2000	45.2	40.4	50.4
Nigeria	Imo	2222	2005	42.4	37.8	47.1
Nigeria	Imo	2222	2010	38.9	34.0	44.0
Nigeria	Imo	2222	2015	30.6	25.7	36.7
Nigeria	Jigawa	2223	2000	60.6	55.8	65.7
Nigeria	Jigawa	2223	2005	55.7	51.8	60.5
Nigeria	Jigawa	2223	2010	48.4	43.9	53.1
Nigeria	Jigawa	2223	2015	43.4	37.5	50.1
Nigeria	Kaduna	2224	2000	49.4	44.6	54.3
Nigeria	Kaduna	2224	2005	45.8	41.3	50.3
Nigeria	Kaduna	2224	2010	41.4	37.1	46.1
Nigeria	Kaduna	2224	2015	39.7	33.6	46.2
Nigeria	Kano	2225	2000	62.3	57.2	67.6
Nigeria	Kano	2225	2005	56.9	52.2	61.9
Nigeria	Kano	2225	2010	47.6	43.0	52.5
Nigeria	Kano	2225	2015	45.2	38.7	52.9
Nigeria	Katsina	2226	2000	56.6	51.8	61.1
Nigeria	Katsina	2226	2005	52.5	48.5	57.0
Nigeria	Katsina	2226	2010	42.0	38.0	46.4
Nigeria	Katsina	2226	2015	39.4	33.7	45.6
Nigeria	Kebbi	2227	2000	50.9	46.1	56.0
Nigeria	Kebbi	2227	2005	47.9	43.6	52.5
Nigeria	Kebbi	2227	2010	44.2	39.4	49.6
Nigeria	Kebbi	2227	2015	41.5	35.0	48.7
Nigeria	Kogi	2228	2000	42.1	37.8	47.6
Nigeria	Kogi	2228	2005	38.1	34.1	42.5
Nigeria	Kogi	2228	2010	35.0	30.3	39.4
Nigeria	Kogi	2228	2015	31.1	26.0	37.0
Nigeria	Kwara	2229	2000	34.2	30.0	38.7
Nigeria	Kwara	2229	2005	32.4	28.8	36.7
Nigeria	Kwara	2229	2010	31.0	26.9	35.6
Nigeria	Kwara	2229	2015	29.7	25.0	35.5
Nigeria	Lagos	2230	2000	40.4	35.8	45.1
Nigeria	Lagos	2230	2005	40.5	36.0	45.2
Nigeria	Lagos	2230	2010	42.3	37.5	47.8
Nigeria	Lagos	2230	2015	36.8	30.5	43.4
Nigeria	Nassarawa	65705	2000	52.8	47.5	58.8
Nigeria	Nassarawa	65705	2005	48.8	43.5	54.1
Nigeria	Nassarawa	65705	2010	44.7	39.4	50.2
Nigeria	Nassarawa	65705	2015	41.8	34.9	49.4
Nigeria	Niger	2231	2000	40.6	36.6	44.9
Nigeria	Niger	2231	2005	37.1	33.5	41.1
Nigeria	Niger	2231	2010	34.8	30.7	39.2
Nigeria	Niger	2231	2015	33.9	28.6	39.5
Nigeria	Ogun	2232	2000	41.7	37.7	45.8
Nigeria	Ogun	2232	2005	40.7	36.8	44.9
Nigeria	Ogun	2232	2010	39.2	35.0	43.8
Nigeria	Ogun	2232	2015	35.7	30.0	41.7
Nigeria	Ondo	65706	2000	40.3	35.9	45.1
Nigeria	Ondo	65706	2005	37.3	33.3	41.3
Nigeria	Ondo	65706	2010	35.2	31.0	39.9
Nigeria	Ondo	65706	2015	32.1	27.1	38.4
Nigeria	Osun	2234	2000	33.3	29.4	37.2

Admin 0	Admin 1	GAUL Code	Year	Neonatal mortality (per 1,000 livebirths)		
				Estimate	Lower bound, 95% UI	Upper bound, 95% UI
Nigeria	Osun	2234	2005	31.8	28.2	35.6
Nigeria	Osun	2234	2010	31.0	27.0	35.3
Nigeria	Osun	2234	2015	27.2	22.6	32.4
Nigeria	Oyo	2235	2000	34.0	30.0	38.0
Nigeria	Oyo	2235	2005	32.2	28.7	35.8
Nigeria	Oyo	2235	2010	30.8	27.0	34.8
Nigeria	Oyo	2235	2015	29.1	24.3	34.6
Nigeria	Plateau	65707	2000	49.7	44.7	55.7
Nigeria	Plateau	65707	2005	49.2	44.2	54.7
Nigeria	Plateau	65707	2010	45.0	40.0	51.0
Nigeria	Plateau	65707	2015	42.7	36.0	50.6
Nigeria	Rivers	65708	2000	44.4	39.2	50.2
Nigeria	Rivers	65708	2005	42.2	37.7	47.3
Nigeria	Rivers	65708	2010	38.7	34.2	44.0
Nigeria	Rivers	65708	2015	32.3	26.9	38.1
Nigeria	Sokoto	65709	2000	54.3	49.2	60.0
Nigeria	Sokoto	65709	2005	49.9	45.5	54.8
Nigeria	Sokoto	65709	2010	43.8	39.0	49.1
Nigeria	Sokoto	65709	2015	39.6	33.5	46.3
Nigeria	Taraba	2239	2000	58.7	53.5	64.5
Nigeria	Taraba	2239	2005	55.6	50.5	60.7
Nigeria	Taraba	2239	2010	47.5	42.4	52.5
Nigeria	Taraba	2239	2015	44.6	38.2	51.9
Nigeria	Yobe	2240	2000	48.5	43.6	53.5
Nigeria	Yobe	2240	2005	45.2	41.0	49.8
Nigeria	Yobe	2240	2010	39.8	35.6	44.5
Nigeria	Yobe	2240	2015	36.6	30.8	43.1
Nigeria	Zamfara	65710	2000	55.9	50.4	61.5
Nigeria	Zamfara	65710	2005	51.5	47.1	56.3
Nigeria	Zamfara	65710	2010	45.8	41.0	51.1
Nigeria	Zamfara	65710	2015	40.7	34.7	47.6
Rwanda	East/Iburasirazuba	21969	2000	40.3	37.7	43.0
Rwanda	East/Iburasirazuba	21969	2005	30.2	28.1	32.4
Rwanda	East/Iburasirazuba	21969	2010	22.1	20.2	24.0
Rwanda	East/Iburasirazuba	21969	2015	18.6	16.2	21.2
Rwanda	Kigali City/Umujyi wa Kigali	21970	2000	33.7	31.3	36.1
Rwanda	Kigali City/Umujyi wa Kigali	21970	2005	28.1	26.0	30.1
Rwanda	Kigali City/Umujyi wa Kigali	21970	2010	21.6	19.8	23.7
Rwanda	Kigali City/Umujyi wa Kigali	21970	2015	18.9	16.3	21.8
Rwanda	North/Amajyaruguru	21971	2000	39.6	37.1	42.4
Rwanda	North/Amajyaruguru	21971	2005	29.3	27.3	31.3
Rwanda	North/Amajyaruguru	21971	2010	20.9	19.1	22.9
Rwanda	North/Amajyaruguru	21971	2015	17.6	15.2	20.2
Rwanda	South/Amajyepfo	21972	2000	40.6	38.4	43.0
Rwanda	South/Amajyepfo	21972	2005	32.8	30.9	34.8
Rwanda	South/Amajyepfo	21972	2010	24.5	22.7	26.4
Rwanda	South/Amajyepfo	21972	2015	20.3	17.8	23.0
Rwanda	West/Iburengerazuba	21973	2000	39.3	36.7	41.8
Rwanda	West/Iburengerazuba	21973	2005	31.1	29.1	33.3
Rwanda	West/Iburengerazuba	21973	2010	22.6	20.8	24.5
Rwanda	West/Iburengerazuba	21973	2015	18.9	16.4	21.6
Senegal	Dakar	2636	2000	23.1	20.2	26.3
Senegal	Dakar	2636	2005	21.2	18.6	24.1
Senegal	Dakar	2636	2010	20.2	17.4	23.2
Senegal	Dakar	2636	2015	17.8	14.4	21.4
Senegal	Diourbel	47585	2000	41.3	37.9	44.9
Senegal	Diourbel	47585	2005	35.4	32.5	38.6
Senegal	Diourbel	47585	2010	28.8	25.9	32.0
Senegal	Diourbel	47585	2015	23.4	19.9	27.2
Senegal	Fatick	47586	2000	35.5	32.8	38.3
Senegal	Fatick	47586	2005	30.7	28.5	33.3
Senegal	Fatick	47586	2010	25.6	23.5	27.9
Senegal	Fatick	47586	2015	20.9	18.2	23.8
Senegal	Kaffrine	1378	2000	37.8	34.7	41.0
Senegal	Kaffrine	1378	2005	32.2	29.6	34.9
Senegal	Kaffrine	1378	2010	23.6	21.3	26.0
Senegal	Kaffrine	1378	2015	19.8	17.0	22.9

Admin 0	Admin 1	GAUL Code	Year	Neonatal mortality (per 1,000 livebirths)		
				Estimate	Lower bound, 95% UI	Upper bound, 95% UI
Senegal	Kaolack	1373	2000	36.8	34.2	39.8
Senegal	Kaolack	1373	2005	31.1	28.9	33.7
Senegal	Kaolack	1373	2010	24.5	22.3	26.8
Senegal	Kaolack	1373	2015	20.1	17.2	23.0
Senegal	Kedougou	1374	2000	54.5	48.4	61.0
Senegal	Kedougou	1374	2005	47.2	42.3	52.2
Senegal	Kedougou	1374	2010	34.6	30.3	38.8
Senegal	Kedougou	1374	2015	31.1	25.7	36.7
Senegal	Kolda	1375	2000	50.2	46.4	53.9
Senegal	Kolda	1375	2005	43.0	39.7	46.3
Senegal	Kolda	1375	2010	30.9	28.0	34.0
Senegal	Kolda	1375	2015	26.7	23.1	30.6
Senegal	Louga	47587	2000	37.8	34.6	41.2
Senegal	Louga	47587	2005	33.5	30.8	36.5
Senegal	Louga	47587	2010	28.2	25.3	31.1
Senegal	Louga	47587	2015	22.5	19.1	26.3
Senegal	Matam	47588	2000	43.1	39.3	46.9
Senegal	Matam	47588	2005	37.2	34.0	40.6
Senegal	Matam	47588	2010	30.0	26.8	33.4
Senegal	Matam	47588	2015	24.4	20.7	28.6
Senegal	Saint louis	47589	2000	36.2	33.3	39.6
Senegal	Saint louis	47589	2005	31.3	28.8	33.9
Senegal	Saint louis	47589	2010	27.7	24.8	30.8
Senegal	Saint louis	47589	2015	21.4	18.0	25.3
Senegal	Sedhiou	1376	2000	42.1	38.3	46.0
Senegal	Sedhiou	1376	2005	34.9	31.8	37.9
Senegal	Sedhiou	1376	2010	26.3	23.7	28.9
Senegal	Sedhiou	1376	2015	22.5	19.4	25.8
Senegal	Tambacounda	1377	2000	42.7	39.2	46.5
Senegal	Tambacounda	1377	2005	36.6	33.7	39.9
Senegal	Tambacounda	1377	2010	27.7	24.9	30.7
Senegal	Tambacounda	1377	2015	23.5	20.4	26.8
Senegal	Thies	2644	2000	28.9	26.4	31.6
Senegal	Thies	2644	2005	25.5	23.3	27.9
Senegal	Thies	2644	2010	23.0	20.6	25.6
Senegal	Thies	2644	2015	19.1	16.3	22.2
Senegal	Ziguinchor	2645	2000	32.6	29.4	36.2
Senegal	Ziguinchor	2645	2005	28.8	25.9	31.7
Senegal	Ziguinchor	2645	2010	24.4	21.6	27.2
Senegal	Ziguinchor	2645	2015	20.8	17.7	24.3
Sierra Leone	Eastern	2654	2000	57.8	53.3	62.6
Sierra Leone	Eastern	2654	2005	49.9	46.2	53.6
Sierra Leone	Eastern	2654	2010	42.2	38.4	46.3
Sierra Leone	Eastern	2654	2015	36.4	31.4	42.3
Sierra Leone	Northern	2655	2000	52.8	49.2	56.5
Sierra Leone	Northern	2655	2005	47.8	44.8	51.2
Sierra Leone	Northern	2655	2010	41.5	38.0	45.4
Sierra Leone	Northern	2655	2015	35.4	30.6	41.0
Sierra Leone	Southern	2656	2000	57.3	52.9	61.6
Sierra Leone	Southern	2656	2005	50.4	46.7	54.5
Sierra Leone	Southern	2656	2010	43.8	40.1	48.0
Sierra Leone	Southern	2656	2015	37.3	32.2	43.2
Sierra Leone	Western Area	2657	2000	48.2	42.4	54.7
Sierra Leone	Western Area	2657	2005	48.5	43.2	54.6
Sierra Leone	Western Area	2657	2010	44.9	39.3	51.6
Sierra Leone	Western Area	2657	2015	38.3	31.8	45.8
Somalia	Awdal	2688	2000	35.8	30.5	41.5
Somalia	Awdal	2688	2005	32.0	27.6	37.2
Somalia	Awdal	2688	2010	30.6	25.6	36.6
Somalia	Awdal	2688	2015	27.9	22.0	34.9
Somalia	Bakool	2689	2000	35.8	28.1	45.9
Somalia	Bakool	2689	2005	32.9	25.6	41.8
Somalia	Bakool	2689	2010	30.0	22.5	39.3
Somalia	Bakool	2689	2015	28.2	20.8	37.6
Somalia	Banadir	2692	2000	47.5	38.9	56.5
Somalia	Banadir	2692	2005	40.5	33.1	48.5
Somalia	Banadir	2692	2010	37.5	29.5	47.4

Admin 0	Admin 1	GAUL Code	Year	Neonatal mortality (per 1,000 livebirths)		
				Estimate	Lower bound, 95% UI	Upper bound, 95% UI
Somalia	Banadir	2692	2015	34.1	25.5	43.9
Somalia	Bari	2690	2000	36.1	28.4	45.3
Somalia	Bari	2690	2005	32.7	26.0	40.9
Somalia	Bari	2690	2010	31.2	24.0	39.2
Somalia	Bari	2690	2015	29.7	22.1	38.5
Somalia	Bay	2691	2000	35.3	28.7	43.4
Somalia	Bay	2691	2005	33.7	27.1	41.5
Somalia	Bay	2691	2010	29.3	22.3	38.1
Somalia	Bay	2691	2015	27.9	20.9	36.8
Somalia	Galgaduud	2693	2000	40.1	31.5	50.1
Somalia	Galgaduud	2693	2005	36.7	28.6	46.7
Somalia	Galgaduud	2693	2010	34.3	26.1	44.6
Somalia	Galgaduud	2693	2015	29.0	21.8	38.1
Somalia	Gedo	2694	2000	30.7	25.5	36.6
Somalia	Gedo	2694	2005	27.3	22.5	32.6
Somalia	Gedo	2694	2010	24.9	20.1	30.7
Somalia	Gedo	2694	2015	23.7	18.7	30.1
Somalia	Hiraan	2695	2000	38.8	30.2	49.6
Somalia	Hiraan	2695	2005	36.6	28.3	46.7
Somalia	Hiraan	2695	2010	33.6	25.5	44.3
Somalia	Hiraan	2695	2015	29.5	21.8	39.8
Somalia	Juba Dhexe	2698	2000	36.6	28.8	46.3
Somalia	Juba Dhexe	2698	2005	32.0	25.2	40.1
Somalia	Juba Dhexe	2698	2010	23.2	17.8	30.3
Somalia	Juba Dhexe	2698	2015	27.2	20.2	35.6
Somalia	Juba Hoose	2696	2000	39.1	30.8	48.6
Somalia	Juba Hoose	2696	2005	36.1	28.5	45.0
Somalia	Juba Hoose	2696	2010	33.0	25.7	41.6
Somalia	Juba Hoose	2696	2015	28.6	21.6	37.3
Somalia	Mudug	2700	2000	36.1	28.6	44.2
Somalia	Mudug	2700	2005	33.9	26.7	41.8
Somalia	Mudug	2700	2010	31.6	24.6	39.7
Somalia	Mudug	2700	2015	28.9	21.6	37.5
Somalia	Nugaal	2701	2000	34.1	26.1	43.3
Somalia	Nugaal	2701	2005	30.5	23.9	38.7
Somalia	Nugaal	2701	2010	29.5	22.3	37.7
Somalia	Nugaal	2701	2015	25.0	18.4	32.7
Somalia	Sanaag	2702	2000	36.9	30.6	45.0
Somalia	Sanaag	2702	2005	33.7	28.3	40.9
Somalia	Sanaag	2702	2010	34.1	27.5	41.7
Somalia	Sanaag	2702	2015	28.2	21.5	35.8
Somalia	Shabelle Dhexe	2699	2000	42.8	34.8	51.9
Somalia	Shabelle Dhexe	2699	2005	38.0	30.7	46.6
Somalia	Shabelle Dhexe	2699	2010	34.0	26.3	43.2
Somalia	Shabelle Dhexe	2699	2015	30.7	22.7	39.4
Somalia	Shabelle Hoose	2697	2000	47.8	40.1	56.1
Somalia	Shabelle Hoose	2697	2005	42.5	35.6	50.8
Somalia	Shabelle Hoose	2697	2010	36.2	28.8	44.6
Somalia	Shabelle Hoose	2697	2015	34.2	26.1	43.4
Somalia	Sool	2703	2000	34.4	28.0	42.1
Somalia	Sool	2703	2005	33.6	27.3	40.4
Somalia	Sool	2703	2010	32.5	25.9	40.1
Somalia	Sool	2703	2015	26.9	20.9	34.2
Somalia	Togdheer	2704	2000	40.3	33.7	47.5
Somalia	Togdheer	2704	2005	38.5	32.9	44.9
Somalia	Togdheer	2704	2010	38.8	32.2	46.5
Somalia	Togdheer	2704	2015	31.7	24.8	39.6
Somalia	Woqooyi Galbeed	2705	2000	37.6	32.7	42.4
Somalia	Woqooyi Galbeed	2705	2005	35.2	31.2	39.5
Somalia	Woqooyi Galbeed	2705	2010	35.2	30.5	40.7
Somalia	Woqooyi Galbeed	2705	2015	29.9	24.1	36.5
South Africa	Eastern Cape	77310	2000	24.5	21.6	27.7
South Africa	Eastern Cape	77310	2005	23.5	20.4	26.8
South Africa	Eastern Cape	77310	2010	21.0	17.8	24.7
South Africa	Eastern Cape	77310	2015	17.5	14.6	20.9
South Africa	Free State	2707	2000	25.4	22.5	28.4
South Africa	Free State	2707	2005	25.1	22.2	28.4

Admin 0	Admin 1	GAUL Code	Year	Neonatal mortality (per 1,000 livebirths)		
				Estimate	Lower bound, 95% UI	Upper bound, 95% UI
South Africa	Free State	2707	2010	22.5	19.3	26.0
South Africa	Free State	2707	2015	18.8	15.7	22.3
South Africa	Gauteng	2708	2000	20.9	17.9	24.3
South Africa	Gauteng	2708	2005	20.7	17.3	24.7
South Africa	Gauteng	2708	2010	18.7	14.8	23.0
South Africa	Gauteng	2708	2015	15.1	11.7	19.2
South Africa	KwaZulu-Natal	77311	2000	25.6	22.7	28.7
South Africa	KwaZulu-Natal	77311	2005	24.2	21.1	27.4
South Africa	KwaZulu-Natal	77311	2010	21.7	18.6	25.3
South Africa	KwaZulu-Natal	77311	2015	17.8	14.7	21.5
South Africa	Limpopo	77312	2000	22.4	19.2	25.8
South Africa	Limpopo	77312	2005	21.5	18.2	25.1
South Africa	Limpopo	77312	2010	18.9	15.7	23.2
South Africa	Limpopo	77312	2015	15.8	12.6	19.8
South Africa	Mpumalanga	77313	2000	23.8	21.2	26.6
South Africa	Mpumalanga	77313	2005	22.0	19.5	24.8
South Africa	Mpumalanga	77313	2010	19.3	16.6	22.4
South Africa	Mpumalanga	77313	2015	15.7	12.8	18.6
South Africa	North West	77314	2000	22.7	20.0	25.5
South Africa	North West	77314	2005	22.6	19.5	25.9
South Africa	North West	77314	2010	19.5	16.4	23.1
South Africa	North West	77314	2015	16.2	13.1	19.8
South Africa	Northern Cape	77315	2000	22.6	19.1	26.6
South Africa	Northern Cape	77315	2005	22.3	18.5	26.6
South Africa	Northern Cape	77315	2010	18.9	15.5	22.7
South Africa	Northern Cape	77315	2015	15.9	12.7	19.7
South Africa	Western Cape	2714	2000	19.3	16.0	23.4
South Africa	Western Cape	2714	2005	19.2	15.6	23.7
South Africa	Western Cape	2714	2010	16.9	13.3	21.8
South Africa	Western Cape	2714	2015	13.4	10.3	17.5
South Sudan	Central Equatoria	2748	2000	43.5	39.2	48.5
South Sudan	Central Equatoria	2748	2005	42.1	37.8	46.6
South Sudan	Central Equatoria	2748	2010	37.2	32.4	42.5
South Sudan	Central Equatoria	2748	2015	36.8	30.2	44.0
South Sudan	Eastern Equatoria	2750	2000	34.3	30.5	38.2
South Sudan	Eastern Equatoria	2750	2005	34.2	30.5	38.2
South Sudan	Eastern Equatoria	2750	2010	32.9	28.7	37.4
South Sudan	Eastern Equatoria	2750	2015	33.1	27.6	39.4
South Sudan	El Buheyrat	2746	2000	34.5	30.4	39.1
South Sudan	El Buheyrat	2746	2005	30.0	26.7	33.7
South Sudan	El Buheyrat	2746	2010	28.5	24.2	33.0
South Sudan	El Buheyrat	2746	2015	28.3	23.2	34.3
South Sudan	Jonglei	2751	2000	33.2	29.3	37.1
South Sudan	Jonglei	2751	2005	30.2	26.6	34.0
South Sudan	Jonglei	2751	2010	28.4	24.4	32.7
South Sudan	Jonglei	2751	2015	26.7	22.3	31.8
South Sudan	Northern Bahr El Ghazal	2754	2000	56.5	51.1	62.4
South Sudan	Northern Bahr El Ghazal	2754	2005	49.8	44.6	55.6
South Sudan	Northern Bahr El Ghazal	2754	2010	49.5	43.1	56.5
South Sudan	Northern Bahr El Ghazal	2754	2015	40.8	33.3	49.5
South Sudan	Unity	2747	2000	35.6	31.5	40.2
South Sudan	Unity	2747	2005	30.5	26.7	34.7
South Sudan	Unity	2747	2010	29.8	25.5	34.7
South Sudan	Unity	2747	2015	28.7	23.3	34.8
South Sudan	Upper Nile	37021	2000	32.3	28.6	36.5
South Sudan	Upper Nile	37021	2005	30.0	26.8	33.6
South Sudan	Upper Nile	37021	2010	27.5	24.1	31.5
South Sudan	Upper Nile	37021	2015	25.4	21.3	30.1
South Sudan	Warab	2765	2000	43.6	39.3	48.2
South Sudan	Warab	2765	2005	37.4	33.5	41.8
South Sudan	Warab	2765	2010	34.6	30.2	39.4
South Sudan	Warab	2765	2015	32.9	27.2	39.3
South Sudan	Western Bahr El Ghazal	2766	2000	41.1	36.6	46.3
South Sudan	Western Bahr El Ghazal	2766	2005	36.6	32.4	41.5
South Sudan	Western Bahr El Ghazal	2766	2010	33.3	28.5	38.4
South Sudan	Western Bahr El Ghazal	2766	2015	31.4	25.6	38.1
South Sudan	Western Equatoria	2768	2000	41.6	36.9	47.2

Admin 0	Admin 1	GAUL Code	Year	Neonatal mortality (per 1,000 livebirths)		
				Estimate	Lower bound, 95% UI	Upper bound, 95% UI
South Sudan	Western Equatoria	2768	2005	39.8	35.1	45.0
South Sudan	Western Equatoria	2768	2010	33.1	28.2	38.1
South Sudan	Western Equatoria	2768	2015	33.6	27.9	40.3
Sudan	Abyei	124	2000	49.7	42.4	57.9
Sudan	Abyei	124	2005	40.7	34.8	47.6
Sudan	Abyei	124	2010	41.0	34.1	48.9
Sudan	Abyei	124	2015	36.5	29.0	45.1
Sudan	Al Jazeera	2745	2000	33.5	29.8	37.8
Sudan	Al Jazeera	2745	2005	27.9	24.8	31.3
Sudan	Al Jazeera	2745	2010	26.7	23.4	30.3
Sudan	Al Jazeera	2745	2015	26.0	21.9	30.8
Sudan	Blue Nile	2749	2000	49.5	44.5	55.0
Sudan	Blue Nile	2749	2005	42.5	38.4	46.9
Sudan	Blue Nile	2749	2010	38.1	33.7	43.0
Sudan	Blue Nile	2749	2015	31.4	26.4	36.9
Sudan	Gadaref	68805	2000	36.1	32.2	40.2
Sudan	Gadaref	68805	2005	31.2	28.1	34.5
Sudan	Gadaref	68805	2010	29.9	26.6	33.7
Sudan	Gadaref	68805	2015	27.4	23.2	32.0
Sudan	Kassala	68783	2000	30.4	27.0	34.6
Sudan	Kassala	68783	2005	27.6	24.6	30.9
Sudan	Kassala	68783	2010	26.5	23.1	30.4
Sudan	Kassala	68783	2015	25.8	21.4	30.9
Sudan	Khartoum	2753	2000	30.8	27.0	34.8
Sudan	Khartoum	2753	2005	27.9	24.8	31.3
Sudan	Khartoum	2753	2010	27.8	24.2	31.8
Sudan	Khartoum	2753	2015	24.8	20.6	29.6
Sudan	Nile	2761	2000	30.5	26.5	35.1
Sudan	Nile	2761	2005	28.2	24.5	31.9
Sudan	Nile	2761	2010	26.9	23.5	31.1
Sudan	Nile	2761	2015	24.5	20.6	29.4
Sudan	Northern	2755	2000	32.1	28.0	36.8
Sudan	Northern	2755	2005	30.3	26.4	34.5
Sudan	Northern	2755	2010	29.3	25.4	34.2
Sudan	Northern	2755	2015	25.2	21.0	29.8
Sudan	Northern Darfur	2757	2000	34.6	30.8	38.2
Sudan	Northern Darfur	2757	2005	32.3	29.1	35.7
Sudan	Northern Darfur	2757	2010	33.1	29.6	37.0
Sudan	Northern Darfur	2757	2015	31.9	27.3	36.9
Sudan	Northern Kordofan	68784	2000	34.9	31.5	38.6
Sudan	Northern Kordofan	68784	2005	31.0	28.0	34.2
Sudan	Northern Kordofan	68784	2010	30.5	27.2	34.6
Sudan	Northern Kordofan	68784	2015	29.2	25.2	34.0
Sudan	Red Sea	40772	2000	27.2	23.4	31.5
Sudan	Red Sea	40772	2005	25.1	21.9	29.0
Sudan	Red Sea	40772	2010	23.3	20.0	27.2
Sudan	Red Sea	40772	2015	23.6	19.3	28.4
Sudan	Sennar	2762	2000	36.2	32.4	40.6
Sudan	Sennar	2762	2005	30.2	27.1	33.9
Sudan	Sennar	2762	2010	28.4	25.0	31.9
Sudan	Sennar	2762	2015	25.6	21.4	30.1
Sudan	Southern Darfur	2764	2000	41.5	37.7	45.4
Sudan	Southern Darfur	2764	2005	37.0	33.7	40.5
Sudan	Southern Darfur	2764	2010	37.3	33.6	41.2
Sudan	Southern Darfur	2764	2015	34.8	30.1	40.0
Sudan	Southern Kordofan	4150	2000	41.8	37.8	46.2
Sudan	Southern Kordofan	4150	2005	34.8	31.5	38.2
Sudan	Southern Kordofan	4150	2010	34.8	31.0	38.8
Sudan	Southern Kordofan	4150	2015	31.3	26.6	36.3
Sudan	Western Darfur	2769	2000	36.6	32.6	40.5
Sudan	Western Darfur	2769	2005	33.7	30.7	37.0
Sudan	Western Darfur	2769	2010	32.0	28.7	35.3
Sudan	Western Darfur	2769	2015	29.0	24.6	33.6
Sudan	White Nile	2770	2000	36.0	32.8	39.5
Sudan	White Nile	2770	2005	30.1	27.3	33.3
Sudan	White Nile	2770	2010	29.3	26.1	32.6
Sudan	White Nile	2770	2015	27.2	23.2	31.9

Admin 0	Admin 1	GAUL Code	Year	Neonatal mortality (per 1,000 livebirths)		
				Estimate	Lower bound, 95% UI	Upper bound, 95% UI
Swaziland	Hhohho	2782	2000	20.8	18.3	23.8
Swaziland	Hhohho	2782	2005	22.2	19.6	25.4
Swaziland	Hhohho	2782	2010	19.5	16.6	22.9
Swaziland	Hhohho	2782	2015	16.2	13.2	19.7
Swaziland	Lubombo	2783	2000	21.9	19.3	24.6
Swaziland	Lubombo	2783	2005	23.2	20.5	26.1
Swaziland	Lubombo	2783	2010	20.5	18.0	23.8
Swaziland	Lubombo	2783	2015	16.9	14.0	20.3
Swaziland	Manzini	2784	2000	21.1	18.6	23.8
Swaziland	Manzini	2784	2005	22.6	19.8	25.5
Swaziland	Manzini	2784	2010	20.4	17.5	23.5
Swaziland	Manzini	2784	2015	16.4	13.5	19.8
Swaziland	Shiselweni	2785	2000	23.8	20.7	27.1
Swaziland	Shiselweni	2785	2005	25.1	21.8	28.5
Swaziland	Shiselweni	2785	2010	22.5	19.0	26.0
Swaziland	Shiselweni	2785	2015	18.5	15.0	22.5
Togo	Centrale	2970	2000	38.7	34.0	43.7
Togo	Centrale	2970	2005	35.4	31.2	40.0
Togo	Centrale	2970	2010	30.9	26.8	35.5
Togo	Centrale	2970	2015	27.5	22.7	33.0
Togo	Kara	2971	2000	35.8	31.8	40.0
Togo	Kara	2971	2005	33.6	29.8	37.5
Togo	Kara	2971	2010	28.2	24.7	32.1
Togo	Kara	2971	2015	24.9	20.6	29.7
Togo	Maritime	2972	2000	34.5	31.0	38.8
Togo	Maritime	2972	2005	31.8	28.7	35.2
Togo	Maritime	2972	2010	30.2	26.8	33.8
Togo	Maritime	2972	2015	25.5	21.2	30.2
Togo	Plateaux	2973	2000	37.6	33.7	42.0
Togo	Plateaux	2973	2005	34.7	31.1	38.7
Togo	Plateaux	2973	2010	32.3	28.8	36.2
Togo	Plateaux	2973	2015	29.2	24.6	34.6
Togo	Savanes	2974	2000	37.3	33.7	41.2
Togo	Savanes	2974	2005	35.6	31.8	39.4
Togo	Savanes	2974	2010	29.9	26.6	33.5
Togo	Savanes	2974	2015	25.8	21.8	30.6
Uganda	Abim	743	2000	34.8	30.1	39.9
Uganda	Abim	743	2005	32.1	28.1	36.9
Uganda	Abim	743	2010	28.7	24.8	33.0
Uganda	Abim	743	2015	23.8	19.4	28.6
Uganda	Adjumani	3104	2000	37.0	32.4	42.0
Uganda	Adjumani	3104	2005	36.0	31.2	40.5
Uganda	Adjumani	3104	2010	32.1	27.6	36.7
Uganda	Adjumani	3104	2015	28.3	23.1	34.0
Uganda	Agago	790	2000	36.2	31.5	41.3
Uganda	Agago	790	2005	33.2	29.0	37.6
Uganda	Agago	790	2010	29.7	25.5	34.4
Uganda	Agago	790	2015	25.5	20.4	30.9
Uganda	Alebtong	852	2000	34.2	30.6	38.4
Uganda	Alebtong	852	2005	31.8	28.7	35.1
Uganda	Alebtong	852	2010	28.7	25.4	32.3
Uganda	Alebtong	852	2015	25.1	20.8	29.8
Uganda	Amolatar	47067	2000	33.8	30.1	38.1
Uganda	Amolatar	47067	2005	31.3	27.8	35.3
Uganda	Amolatar	47067	2010	28.3	24.8	32.0
Uganda	Amolatar	47067	2015	24.8	20.6	29.2
Uganda	Amudat	744	2000	33.3	29.3	37.6
Uganda	Amudat	744	2005	29.6	25.7	33.8
Uganda	Amudat	744	2010	24.8	21.4	28.7
Uganda	Amudat	744	2015	21.8	18.2	25.8
Uganda	Amuria	47068	2000	31.7	27.9	35.9
Uganda	Amuria	47068	2005	29.6	26.1	33.3
Uganda	Amuria	47068	2010	27.2	23.9	31.0
Uganda	Amuria	47068	2015	23.6	19.7	27.7
Uganda	Amuru	745	2000	36.6	32.1	41.5
Uganda	Amuru	745	2005	34.9	30.2	39.5
Uganda	Amuru	745	2010	30.8	26.7	35.4

Admin 0	Admin 1	GAUL Code	Year	Neonatal mortality (per 1,000 livebirths)		
				Estimate	Lower bound, 95% UI	Upper bound, 95% UI
Uganda	Amuru	745	2015	27.2	22.4	33.1
Uganda	Apac	746	2000	34.6	30.7	38.8
Uganda	Apac	746	2005	32.3	28.9	36.1
Uganda	Apac	746	2010	28.9	25.5	32.6
Uganda	Apac	746	2015	25.5	21.1	30.0
Uganda	Arua	1057	2000	37.3	33.4	41.4
Uganda	Arua	1057	2005	35.4	31.8	39.0
Uganda	Arua	1057	2010	29.8	26.3	33.6
Uganda	Arua	1057	2015	26.7	22.0	31.7
Uganda	Budaka	747	2000	31.7	28.8	34.5
Uganda	Budaka	747	2005	29.3	26.8	32.0
Uganda	Budaka	747	2010	25.8	23.0	28.6
Uganda	Budaka	747	2015	22.4	19.2	26.0
Uganda	Bududa	1080	2000	30.4	27.7	33.1
Uganda	Bududa	1080	2005	27.5	25.1	30.2
Uganda	Bududa	1080	2010	24.3	21.9	26.8
Uganda	Bududa	1080	2015	22.1	18.9	25.7
Uganda	Bugiri	748	2000	36.2	33.3	39.3
Uganda	Bugiri	748	2005	33.2	30.3	36.1
Uganda	Bugiri	748	2010	27.8	25.1	30.7
Uganda	Bugiri	748	2015	24.1	20.6	27.7
Uganda	Buhweju	778	2000	40.7	35.8	45.7
Uganda	Buhweju	778	2005	33.4	29.1	37.7
Uganda	Buhweju	778	2010	27.5	23.6	31.8
Uganda	Buhweju	778	2015	22.6	18.7	27.3
Uganda	Buikwe	749	2000	33.3	30.1	36.7
Uganda	Buikwe	749	2005	31.9	29.0	35.4
Uganda	Buikwe	749	2010	29.7	26.7	33.4
Uganda	Buikwe	749	2015	22.9	19.5	26.9
Uganda	Bukedea	788	2000	30.4	27.5	33.6
Uganda	Bukedea	788	2005	28.0	25.3	30.9
Uganda	Bukedea	788	2010	25.1	22.4	28.1
Uganda	Bukedea	788	2015	22.3	18.9	26.0
Uganda	Bukomansimbi	750	2000	36.9	33.1	41.2
Uganda	Bukomansimbi	750	2005	33.3	29.8	37.0
Uganda	Bukomansimbi	750	2010	29.9	26.5	34.0
Uganda	Bukomansimbi	750	2015	24.9	20.7	29.6
Uganda	Bukwo	751	2000	28.4	24.9	32.0
Uganda	Bukwo	751	2005	25.3	22.2	28.4
Uganda	Bukwo	751	2010	22.8	19.8	26.2
Uganda	Bukwo	751	2015	20.6	17.2	24.7
Uganda	Bulambuli	752	2000	29.9	26.9	33.1
Uganda	Bulambuli	752	2005	27.4	24.8	30.3
Uganda	Bulambuli	752	2010	24.3	21.7	27.3
Uganda	Bulambuli	752	2015	22.4	19.0	26.2
Uganda	Buliisa	753	2000	37.2	32.1	42.9
Uganda	Buliisa	753	2005	34.8	30.3	40.0
Uganda	Buliisa	753	2010	31.1	26.8	35.9
Uganda	Buliisa	753	2015	25.7	21.0	31.3
Uganda	Bundibugyo	754	2000	40.1	34.9	45.7
Uganda	Bundibugyo	754	2005	34.8	30.1	39.8
Uganda	Bundibugyo	754	2010	28.6	24.4	33.0
Uganda	Bundibugyo	754	2015	25.0	20.5	29.9
Uganda	Bushenyi	755	2000	39.2	35.4	43.6
Uganda	Bushenyi	755	2005	32.0	28.5	36.1
Uganda	Bushenyi	755	2010	26.0	22.6	29.9
Uganda	Bushenyi	755	2015	21.7	17.9	26.1
Uganda	Busia	3110	2000	35.8	32.6	39.3
Uganda	Busia	3110	2005	32.5	29.3	35.9
Uganda	Busia	3110	2010	27.4	24.3	30.6
Uganda	Busia	3110	2015	23.2	19.6	27.0
Uganda	Butaleja	47071	2000	33.1	30.0	36.1
Uganda	Butaleja	47071	2005	30.5	27.6	33.3
Uganda	Butaleja	47071	2010	26.3	23.4	29.2
Uganda	Butaleja	47071	2015	22.9	19.4	26.4
Uganda	Butambala	757	2000	32.3	29.0	35.9
Uganda	Butambala	757	2005	30.2	27.3	33.4

Admin 0	Admin 1	GAUL Code	Year	Neonatal mortality (per 1,000 livebirths)		
				Estimate	Lower bound, 95% UI	Upper bound, 95% UI
Uganda	Butambala	757	2010	28.3	25.0	31.8
Uganda	Butambala	757	2015	22.9	19.3	27.0
Uganda	Buvuma	1082	2000	37.4	33.5	41.9
Uganda	Buvuma	1082	2005	34.7	30.9	38.7
Uganda	Buvuma	1082	2010	30.8	27.4	35.0
Uganda	Buvuma	1082	2015	24.4	20.6	28.5
Uganda	Buyende	756	2000	31.9	28.5	35.4
Uganda	Buyende	756	2005	30.4	27.3	33.6
Uganda	Buyende	756	2010	27.0	24.0	30.4
Uganda	Buyende	756	2015	23.8	20.3	28.2
Uganda	Dokolo	789	2000	34.1	30.7	38.4
Uganda	Dokolo	789	2005	31.5	28.3	34.8
Uganda	Dokolo	789	2010	28.5	25.2	32.0
Uganda	Dokolo	789	2015	24.9	20.8	29.4
Uganda	Gomba	1086	2000	33.7	30.2	37.4
Uganda	Gomba	1086	2005	31.3	28.0	35.1
Uganda	Gomba	1086	2010	28.7	25.3	32.4
Uganda	Gomba	1086	2015	23.8	19.8	28.0
Uganda	Gulu	1089	2000	35.9	31.5	40.5
Uganda	Gulu	1089	2005	33.7	29.7	38.0
Uganda	Gulu	1089	2010	30.2	26.3	34.2
Uganda	Gulu	1089	2015	26.5	21.7	32.0
Uganda	Hoima	3112	2000	36.3	32.1	41.2
Uganda	Hoima	3112	2005	33.8	29.8	38.2
Uganda	Hoima	3112	2010	30.1	26.2	34.2
Uganda	Hoima	3112	2015	25.2	20.8	30.1
Uganda	Ibanda	47072	2000	40.6	36.2	45.4
Uganda	Ibanda	47072	2005	34.7	30.7	38.7
Uganda	Ibanda	47072	2010	28.7	25.0	32.9
Uganda	Ibanda	47072	2015	24.5	20.4	29.0
Uganda	Iganga	758	2000	34.8	31.3	38.4
Uganda	Iganga	758	2005	32.8	29.7	36.3
Uganda	Iganga	758	2010	27.9	24.9	31.4
Uganda	Iganga	758	2015	23.7	20.3	27.6
Uganda	Isingiro	47073	2000	39.2	34.9	43.9
Uganda	Isingiro	47073	2005	33.0	29.3	37.1
Uganda	Isingiro	47073	2010	27.3	23.8	31.1
Uganda	Isingiro	47073	2015	22.3	18.5	26.2
Uganda	Jinja	3114	2000	31.7	28.8	34.8
Uganda	Jinja	3114	2005	31.3	28.7	34.4
Uganda	Jinja	3114	2010	28.1	25.3	31.4
Uganda	Jinja	3114	2015	22.9	19.6	26.6
Uganda	Kaabong	47074	2000	35.5	30.6	40.7
Uganda	Kaabong	47074	2005	32.4	27.9	37.4
Uganda	Kaabong	47074	2010	29.3	24.7	34.2
Uganda	Kaabong	47074	2015	23.9	19.4	29.3
Uganda	Kabale	3115	2000	37.8	34.4	41.1
Uganda	Kabale	3115	2005	29.7	27.2	32.4
Uganda	Kabale	3115	2010	24.6	22.1	27.3
Uganda	Kabale	3115	2015	20.1	17.0	23.2
Uganda	Kabarole	42180	2000	39.4	34.5	44.5
Uganda	Kabarole	42180	2005	34.5	30.3	39.4
Uganda	Kabarole	42180	2010	28.7	24.8	32.7
Uganda	Kabarole	42180	2015	25.1	20.9	29.9
Uganda	Kaberamaido	42181	2000	32.4	29.1	36.1
Uganda	Kaberamaido	42181	2005	30.4	27.4	33.7
Uganda	Kaberamaido	42181	2010	27.7	24.7	30.9
Uganda	Kaberamaido	42181	2015	24.2	20.4	28.4
Uganda	Kalangala	3117	2000	35.7	31.0	41.7
Uganda	Kalangala	3117	2005	32.5	27.7	37.7
Uganda	Kalangala	3117	2010	29.4	25.0	34.2
Uganda	Kalangala	3117	2015	24.6	20.2	29.7
Uganda	Kaliro	47075	2000	32.6	29.4	36.3
Uganda	Kaliro	47075	2005	30.7	27.7	33.7
Uganda	Kaliro	47075	2010	26.8	24.0	30.1
Uganda	Kaliro	47075	2015	23.7	20.2	27.7
Uganda	Kalungu	759	2000	35.7	32.1	40.1

Admin 0	Admin 1	GAUL Code	Year	Neonatal mortality (per 1,000 livebirths)		
				Estimate	Lower bound, 95% UI	Upper bound, 95% UI
Uganda	Kalungu	759	2005	32.4	28.9	36.2
Uganda	Kalungu	759	2010	29.4	25.8	33.4
Uganda	Kalungu	759	2015	24.5	20.4	29.0
Uganda	Kampala	42182	2000	28.3	25.9	31.0
Uganda	Kampala	42182	2005	28.3	25.9	31.0
Uganda	Kampala	42182	2010	28.0	25.1	31.1
Uganda	Kampala	42182	2015	22.5	19.2	26.4
Uganda	Kamuli	760	2000	32.0	28.9	35.2
Uganda	Kamuli	760	2005	30.7	27.9	34.1
Uganda	Kamuli	760	2010	27.0	24.0	30.4
Uganda	Kamuli	760	2015	23.0	19.7	27.0
Uganda	Kamwenge	42183	2000	40.2	36.1	44.8
Uganda	Kamwenge	42183	2005	34.8	31.0	38.9
Uganda	Kamwenge	42183	2010	28.7	25.1	32.5
Uganda	Kamwenge	42183	2015	25.3	21.2	29.9
Uganda	Kanungu	42184	2000	35.8	31.8	40.4
Uganda	Kanungu	42184	2005	28.8	25.2	32.4
Uganda	Kanungu	42184	2010	23.6	20.7	27.1
Uganda	Kanungu	42184	2015	19.5	16.3	23.2
Uganda	Kapchorwa	761	2000	29.6	26.4	32.9
Uganda	Kapchorwa	761	2005	26.9	24.1	30.0
Uganda	Kapchorwa	761	2010	23.9	21.1	26.9
Uganda	Kapchorwa	761	2015	21.7	18.4	25.6
Uganda	Kasese	3121	2000	36.8	32.5	41.3
Uganda	Kasese	3121	2005	31.3	27.6	35.3
Uganda	Kasese	3121	2010	26.3	22.7	30.1
Uganda	Kasese	3121	2015	22.2	18.5	26.6
Uganda	Katakwi	47078	2000	30.8	27.1	34.3
Uganda	Katakwi	47078	2005	28.6	25.3	31.8
Uganda	Katakwi	47078	2010	26.2	23.0	29.6
Uganda	Katakwi	47078	2015	22.6	19.0	26.5
Uganda	Kayunga	42185	2000	32.5	29.6	35.7
Uganda	Kayunga	42185	2005	30.7	28.1	34.0
Uganda	Kayunga	42185	2010	28.2	25.2	31.5
Uganda	Kayunga	42185	2015	22.5	19.2	26.4
Uganda	Kibaale	3123	2000	37.7	33.7	42.3
Uganda	Kibaale	3123	2005	34.0	30.1	38.0
Uganda	Kibaale	3123	2010	29.6	26.1	33.4
Uganda	Kibaale	3123	2015	25.8	21.7	30.6
Uganda	Kiboga	1234	2000	34.1	30.2	38.2
Uganda	Kiboga	1234	2005	31.3	27.8	35.1
Uganda	Kiboga	1234	2010	28.1	24.6	31.7
Uganda	Kiboga	1234	2015	23.4	19.7	27.8
Uganda	Kibuku	1235	2000	32.4	29.5	35.4
Uganda	Kibuku	1235	2005	30.1	27.4	33.0
Uganda	Kibuku	1235	2010	26.2	23.5	29.2
Uganda	Kibuku	1235	2015	23.5	20.1	27.2
Uganda	Kiruhura	47079	2000	40.0	35.5	44.7
Uganda	Kiruhura	47079	2005	35.3	31.4	39.5
Uganda	Kiruhura	47079	2010	29.4	25.6	33.7
Uganda	Kiruhura	47079	2015	25.0	21.0	29.6
Uganda	Kiryandongo	1241	2000	35.5	31.4	39.9
Uganda	Kiryandongo	1241	2005	32.8	29.0	37.0
Uganda	Kiryandongo	1241	2010	30.1	26.4	34.3
Uganda	Kiryandongo	1241	2015	25.4	20.9	30.4
Uganda	Kisoro	3125	2000	40.5	36.2	44.6
Uganda	Kisoro	3125	2005	31.0	27.7	34.3
Uganda	Kisoro	3125	2010	24.8	21.9	28.0
Uganda	Kisoro	3125	2015	20.1	16.7	23.6
Uganda	Kitgum	763	2000	37.7	32.6	43.9
Uganda	Kitgum	763	2005	34.4	29.4	39.7
Uganda	Kitgum	763	2010	30.8	26.3	36.2
Uganda	Kitgum	763	2015	27.3	22.0	33.6
Uganda	Koboko	47080	2000	40.5	35.8	45.6
Uganda	Koboko	47080	2005	39.2	34.6	43.8
Uganda	Koboko	47080	2010	32.8	28.2	38.0
Uganda	Koboko	47080	2015	29.5	24.0	35.5

Admin 0	Admin 1	GAUL Code	Year	Neonatal mortality (per 1,000 livebirths)		
				Estimate	Lower bound, 95% UI	Upper bound, 95% UI
Uganda	Kole	764	2000	34.9	31.2	39.0
Uganda	Kole	764	2005	32.7	29.5	36.1
Uganda	Kole	764	2010	29.3	25.9	32.8
Uganda	Kole	764	2015	25.9	21.4	30.6
Uganda	Kotido	765	2000	34.1	29.4	39.0
Uganda	Kotido	765	2005	31.4	27.3	36.1
Uganda	Kotido	765	2010	28.5	24.4	33.3
Uganda	Kotido	765	2015	22.7	18.4	27.6
Uganda	Kumi	1261	2000	30.4	27.3	33.8
Uganda	Kumi	1261	2005	28.3	25.4	31.4
Uganda	Kumi	1261	2010	25.6	22.6	28.6
Uganda	Kumi	1261	2015	22.7	19.2	26.6
Uganda	Kween	766	2000	29.3	25.9	32.5
Uganda	Kween	766	2005	26.4	23.4	29.6
Uganda	Kween	766	2010	23.4	20.6	26.4
Uganda	Kween	766	2015	21.5	18.1	25.6
Uganda	Kyankwanzi	1344	2000	35.7	31.5	40.1
Uganda	Kyankwanzi	1344	2005	32.9	28.8	37.6
Uganda	Kyankwanzi	1344	2010	29.3	25.6	33.3
Uganda	Kyankwanzi	1344	2015	24.5	20.2	29.4
Uganda	Kyegegwa	1361	2000	38.9	34.4	44.0
Uganda	Kyegegwa	1361	2005	34.6	30.3	39.4
Uganda	Kyegegwa	1361	2010	29.7	25.7	33.9
Uganda	Kyegegwa	1361	2015	26.1	21.8	31.2
Uganda	Kyenjojo	1362	2000	39.1	34.4	43.9
Uganda	Kyenjojo	1362	2005	34.8	30.5	39.7
Uganda	Kyenjojo	1362	2010	29.3	25.5	33.5
Uganda	Kyenjojo	1362	2015	25.8	21.4	30.8
Uganda	Lamwo	767	2000	39.2	33.6	45.1
Uganda	Lamwo	767	2005	36.8	31.7	42.7
Uganda	Lamwo	767	2010	33.2	28.3	39.2
Uganda	Lamwo	767	2015	29.9	24.2	37.0
Uganda	Lira	1370	2000	34.3	30.9	38.2
Uganda	Lira	1370	2005	32.3	29.2	35.6
Uganda	Lira	1370	2010	29.2	25.8	32.5
Uganda	Lira	1370	2015	25.6	21.3	30.4
Uganda	Luuka	768	2000	33.5	30.6	36.5
Uganda	Luuka	768	2005	31.8	29.1	34.7
Uganda	Luuka	768	2010	27.4	24.8	30.6
Uganda	Luuka	768	2015	23.4	20.0	27.3
Uganda	Luwero	47083	2000	31.5	28.6	35.1
Uganda	Luwero	47083	2005	29.8	26.9	33.0
Uganda	Luwero	47083	2010	27.7	24.5	31.1
Uganda	Luwero	47083	2015	21.9	18.6	26.1
Uganda	Lwengo	1366	2000	38.9	34.9	43.1
Uganda	Lwengo	1366	2005	34.3	30.9	38.1
Uganda	Lwengo	1366	2010	30.2	26.4	34.4
Uganda	Lwengo	1366	2015	25.3	21.1	29.7
Uganda	Lyantonde	1372	2000	39.2	35.1	44.0
Uganda	Lyantonde	1372	2005	34.4	30.7	38.1
Uganda	Lyantonde	1372	2010	30.0	26.2	34.1
Uganda	Lyantonde	1372	2015	25.1	20.9	29.6
Uganda	Manafwa	769	2000	31.2	28.6	34.0
Uganda	Manafwa	769	2005	28.2	25.7	30.8
Uganda	Manafwa	769	2010	24.8	22.2	27.3
Uganda	Manafwa	769	2015	22.1	18.8	25.7
Uganda	Maracha	780	2000	38.9	34.6	43.7
Uganda	Maracha	780	2005	37.0	33.0	41.2
Uganda	Maracha	780	2010	30.7	26.5	35.2
Uganda	Maracha	780	2015	27.9	22.8	33.5
Uganda	Masaka	770	2000	36.9	32.6	41.8
Uganda	Masaka	770	2005	33.2	29.1	37.3
Uganda	Masaka	770	2010	29.8	25.8	34.2
Uganda	Masaka	770	2015	24.7	20.3	29.7
Uganda	Masindi	771	2000	36.0	31.3	41.1
Uganda	Masindi	771	2005	33.3	28.9	38.3
Uganda	Masindi	771	2010	30.0	26.0	34.7

Admin 0	Admin 1	GAUL Code	Year	Neonatal mortality (per 1,000 livebirths)		
				Estimate	Lower bound, 95% UI	Upper bound, 95% UI
Uganda	Masindi	771	2015	24.9	20.6	30.3
Uganda	Mayuge	42189	2000	36.3	32.9	40.1
Uganda	Mayuge	42189	2005	34.0	30.9	37.3
Uganda	Mayuge	42189	2010	28.9	25.9	31.9
Uganda	Mayuge	42189	2015	24.2	20.7	28.0
Uganda	Mbale	47085	2000	30.6	27.8	33.5
Uganda	Mbale	47085	2005	28.3	25.7	31.2
Uganda	Mbale	47085	2010	25.1	22.4	27.9
Uganda	Mbale	47085	2015	22.1	18.7	25.7
Uganda	Mbarara	47086	2000	39.6	35.7	43.6
Uganda	Mbarara	47086	2005	33.0	29.6	36.7
Uganda	Mbarara	47086	2010	27.3	24.1	30.9
Uganda	Mbarara	47086	2015	22.7	19.0	26.6
Uganda	Mitooma	1360	2000	38.3	34.7	42.5
Uganda	Mitooma	1360	2005	31.0	27.8	34.6
Uganda	Mitooma	1360	2010	25.1	21.9	28.6
Uganda	Mitooma	1360	2015	21.0	17.3	24.8
Uganda	Mityana	47087	2000	32.8	29.7	36.3
Uganda	Mityana	47087	2005	30.4	27.3	33.6
Uganda	Mityana	47087	2010	27.5	24.3	30.7
Uganda	Mityana	47087	2015	22.4	19.0	26.5
Uganda	Moroto	772	2000	33.4	28.8	38.4
Uganda	Moroto	772	2005	30.3	26.3	35.1
Uganda	Moroto	772	2010	27.0	23.1	31.6
Uganda	Moroto	772	2015	22.1	18.2	27.1
Uganda	Moyo	3136	2000	39.6	34.9	44.3
Uganda	Moyo	3136	2005	38.3	33.6	43.0
Uganda	Moyo	3136	2010	34.1	29.4	38.8
Uganda	Moyo	3136	2015	29.9	24.4	35.9
Uganda	Mpigi	1363	2000	32.3	29.3	36.0
Uganda	Mpigi	1363	2005	30.1	27.5	33.1
Uganda	Mpigi	1363	2010	28.4	25.3	31.7
Uganda	Mpigi	1363	2015	22.7	19.4	26.8
Uganda	Mubende	47088	2000	35.5	31.9	39.6
Uganda	Mubende	47088	2005	32.4	29.0	35.9
Uganda	Mubende	47088	2010	28.7	25.3	32.2
Uganda	Mubende	47088	2015	24.5	20.7	28.9
Uganda	Mukono	1367	2000	31.9	29.6	34.6
Uganda	Mukono	1367	2005	30.4	28.1	33.1
Uganda	Mukono	1367	2010	28.3	25.7	31.1
Uganda	Mukono	1367	2015	22.2	19.0	25.8
Uganda	Nakapiripirit	773	2000	33.4	29.1	37.9
Uganda	Nakapiripirit	773	2005	30.2	26.1	34.6
Uganda	Nakapiripirit	773	2010	25.9	22.4	30.1
Uganda	Nakapiripirit	773	2015	21.9	18.1	26.2
Uganda	Nakaseke	47089	2000	32.3	28.9	36.2
Uganda	Nakaseke	47089	2005	30.3	27.0	33.9
Uganda	Nakaseke	47089	2010	27.7	24.7	31.4
Uganda	Nakaseke	47089	2015	22.2	18.9	26.5
Uganda	Nakasongola	42195	2000	32.6	28.6	37.1
Uganda	Nakasongola	42195	2005	30.7	26.8	35.0
Uganda	Nakasongola	42195	2010	28.8	24.9	32.9
Uganda	Nakasongola	42195	2015	23.3	19.2	27.6
Uganda	Namayingo	1368	2000	38.8	35.3	42.9
Uganda	Namayingo	1368	2005	34.5	31.1	38.1
Uganda	Namayingo	1368	2010	29.2	25.9	32.6
Uganda	Namayingo	1368	2015	24.6	21.0	28.5
Uganda	Namutumba	774	2000	33.6	30.6	37.2
Uganda	Namutumba	774	2005	31.6	28.8	34.6
Uganda	Namutumba	774	2010	27.0	24.3	30.2
Uganda	Namutumba	774	2015	23.7	20.3	27.6
Uganda	Napak	775	2000	33.9	29.6	38.6
Uganda	Napak	775	2005	30.7	26.7	35.6
Uganda	Napak	775	2010	27.6	23.7	31.8
Uganda	Napak	775	2015	22.7	18.9	27.4
Uganda	Nebbi	776	2000	36.3	32.3	41.1
Uganda	Nebbi	776	2005	35.1	31.0	39.3

Admin 0	Admin 1	GAUL Code	Year	Neonatal mortality (per 1,000 livebirths)		
				Estimate	Lower bound, 95% UI	Upper bound, 95% UI
Uganda	Nebbi	776	2010	29.7	26.0	34.0
Uganda	Nebbi	776	2015	25.9	21.5	31.2
Uganda	Ngora	777	2000	30.7	27.7	34.2
Uganda	Ngora	777	2005	28.6	25.8	31.6
Uganda	Ngora	777	2010	26.0	23.2	29.0
Uganda	Ngora	777	2015	22.9	19.5	26.7
Uganda	Ntoroko	779	2000	39.1	33.6	45.2
Uganda	Ntoroko	779	2005	34.7	30.0	40.3
Uganda	Ntoroko	779	2010	29.7	25.5	34.5
Uganda	Ntoroko	779	2015	26.0	21.5	31.5
Uganda	Ntungamo	3141	2000	38.5	35.2	42.3
Uganda	Ntungamo	3141	2005	30.8	27.9	33.9
Uganda	Ntungamo	3141	2010	25.3	22.4	28.3
Uganda	Ntungamo	3141	2015	20.8	17.6	24.5
Uganda	Nwoya	1364	2000	36.2	32.0	40.6
Uganda	Nwoya	1364	2005	34.6	30.4	39.1
Uganda	Nwoya	1364	2010	30.5	26.6	34.5
Uganda	Nwoya	1364	2015	26.4	21.6	31.5
Uganda	Otuke	781	2000	34.5	30.5	38.7
Uganda	Otuke	781	2005	32.3	29.0	35.9
Uganda	Otuke	781	2010	29.2	25.7	33.0
Uganda	Otuke	781	2015	25.1	20.6	29.6
Uganda	Oyam	782	2000	35.2	31.5	39.4
Uganda	Oyam	782	2005	33.1	29.8	36.7
Uganda	Oyam	782	2010	29.5	26.0	32.9
Uganda	Oyam	782	2015	26.2	21.7	31.1
Uganda	Pader	783	2000	36.5	31.9	41.3
Uganda	Pader	783	2005	33.8	29.8	38.1
Uganda	Pader	783	2010	30.2	26.1	34.7
Uganda	Pader	783	2015	26.5	21.8	31.8
Uganda	Pallisa	1369	2000	31.3	28.4	34.5
Uganda	Pallisa	1369	2005	29.2	26.4	32.1
Uganda	Pallisa	1369	2010	25.9	23.2	29.1
Uganda	Pallisa	1369	2015	23.0	19.7	26.8
Uganda	Rakai	1371	2000	39.2	35.1	43.8
Uganda	Rakai	1371	2005	34.6	30.8	38.6
Uganda	Rakai	1371	2010	29.7	25.6	33.8
Uganda	Rakai	1371	2015	24.8	20.7	29.1
Uganda	Rubirizi	784	2000	39.1	34.8	43.3
Uganda	Rubirizi	784	2005	32.4	28.6	36.2
Uganda	Rubirizi	784	2010	26.5	22.9	30.2
Uganda	Rubirizi	784	2015	22.4	18.5	27.1
Uganda	Rukungiri	42197	2000	37.0	33.4	40.7
Uganda	Rukungiri	42197	2005	29.8	26.8	32.8
Uganda	Rukungiri	42197	2010	24.3	21.5	27.4
Uganda	Rukungiri	42197	2015	20.2	17.0	23.8
Uganda	Serere	1365	2000	31.6	28.4	34.8
Uganda	Serere	1365	2005	29.6	26.7	32.5
Uganda	Serere	1365	2010	26.8	24.0	29.9
Uganda	Serere	1365	2015	23.4	20.0	27.2
Uganda	Sheema	762	2000	39.9	36.4	44.0
Uganda	Sheema	762	2005	32.5	29.2	36.0
Uganda	Sheema	762	2010	26.5	23.2	29.9
Uganda	Sheema	762	2015	22.1	18.5	26.0
Uganda	Sironko	785	2000	30.1	27.2	33.1
Uganda	Sironko	785	2005	27.5	24.8	30.6
Uganda	Sironko	785	2010	24.5	21.9	27.4
Uganda	Sironko	785	2015	22.1	18.7	26.0
Uganda	Soroti	786	2000	31.1	27.5	34.9
Uganda	Soroti	786	2005	29.1	25.9	32.6
Uganda	Soroti	786	2010	26.7	23.4	30.2
Uganda	Soroti	786	2015	23.3	19.5	27.0
Uganda	Ssembabule	3145	2000	38.2	34.1	42.6
Uganda	Ssembabule	3145	2005	34.2	30.6	38.3
Uganda	Ssembabule	3145	2010	30.3	26.8	34.4
Uganda	Ssembabule	3145	2015	25.7	21.4	30.2
Uganda	Tororo	47090	2000	33.7	30.8	36.7

Admin 0	Admin 1	GAUL Code	Year	Neonatal mortality (per 1,000 livebirths)		
				Estimate	Lower bound, 95% UI	Upper bound, 95% UI
Uganda	Tororo	47090	2005	30.7	27.9	33.4
Uganda	Tororo	47090	2010	26.1	23.4	28.8
Uganda	Tororo	47090	2015	22.9	19.4	26.4
Uganda	Wakiso	42200	2000	29.9	27.5	32.5
Uganda	Wakiso	42200	2005	28.9	26.6	31.4
Uganda	Wakiso	42200	2010	28.2	25.5	31.0
Uganda	Wakiso	42200	2015	22.1	18.9	25.9
Uganda	Yumbe	42201	2000	40.7	36.0	45.6
Uganda	Yumbe	42201	2005	39.1	34.6	43.6
Uganda	Yumbe	42201	2010	33.5	28.9	38.1
Uganda	Yumbe	42201	2015	30.0	24.4	36.3
Uganda	Zombo	787	2000	36.4	31.5	41.6
Uganda	Zombo	787	2005	34.9	30.5	39.9
Uganda	Zombo	787	2010	28.9	25.1	33.0
Uganda	Zombo	787	2015	26.2	21.6	31.4
United Republic of Tanzania	Arusha	48357	2000	26.4	23.5	29.5
United Republic of Tanzania	Arusha	48357	2005	23.6	21.0	26.4
United Republic of Tanzania	Arusha	48357	2010	21.3	18.6	24.4
United Republic of Tanzania	Arusha	48357	2015	17.9	15.0	21.3
United Republic of Tanzania	Dar-es-salaam	48358	2000	33.6	29.7	37.6
United Republic of Tanzania	Dar-es-salaam	48358	2005	31.5	27.9	35.6
United Republic of Tanzania	Dar-es-salaam	48358	2010	29.2	25.8	33.0
United Republic of Tanzania	Dar-es-salaam	48358	2015	25.1	21.0	29.8
United Republic of Tanzania	Dodoma	48359	2000	36.1	32.2	40.4
United Republic of Tanzania	Dodoma	48359	2005	31.4	27.7	35.4
United Republic of Tanzania	Dodoma	48359	2010	26.6	23.3	30.5
United Republic of Tanzania	Dodoma	48359	2015	24.0	20.1	28.6
United Republic of Tanzania	Geita	115002	2000	34.6	30.6	38.6
United Republic of Tanzania	Geita	115002	2005	30.6	27.5	34.0
United Republic of Tanzania	Geita	115002	2010	26.6	23.4	30.2
United Republic of Tanzania	Geita	115002	2015	22.6	18.8	26.9
United Republic of Tanzania	Iringa	115003	2000	38.0	33.4	43.2
United Republic of Tanzania	Iringa	115003	2005	32.8	28.6	37.3
United Republic of Tanzania	Iringa	115003	2010	28.0	24.2	32.2
United Republic of Tanzania	Iringa	115003	2015	24.7	20.4	29.8
United Republic of Tanzania	Kagera	115004	2000	37.3	34.3	40.7
United Republic of Tanzania	Kagera	115004	2005	31.3	28.8	34.0
United Republic of Tanzania	Kagera	115004	2010	26.2	23.8	28.8
United Republic of Tanzania	Kagera	115004	2015	21.8	18.9	25.1
United Republic of Tanzania	Kaskazini Pemba	48371	2000	30.7	27.2	34.3
United Republic of Tanzania	Kaskazini Pemba	48371	2005	26.6	23.3	30.2
United Republic of Tanzania	Kaskazini Pemba	48371	2010	22.5	19.4	25.5
United Republic of Tanzania	Kaskazini Pemba	48371	2015	18.5	15.2	22.4
United Republic of Tanzania	Kaskazini Unguja	48372	2000	33.0	29.4	37.0
United Republic of Tanzania	Kaskazini Unguja	48372	2005	28.0	24.9	31.4
United Republic of Tanzania	Kaskazini Unguja	48372	2010	25.6	22.6	29.1
United Republic of Tanzania	Kaskazini Unguja	48372	2015	20.8	17.2	24.8
United Republic of Tanzania	Katavi	115005	2000	36.4	32.0	41.4
United Republic of Tanzania	Katavi	115005	2005	33.1	29.0	37.6
United Republic of Tanzania	Katavi	115005	2010	28.3	24.5	32.3
United Republic of Tanzania	Katavi	115005	2015	24.3	20.3	28.6
United Republic of Tanzania	Kigoma	48362	2000	34.9	31.5	38.6
United Republic of Tanzania	Kigoma	48362	2005	30.8	27.7	34.1
United Republic of Tanzania	Kigoma	48362	2010	26.6	23.9	29.9
United Republic of Tanzania	Kigoma	48362	2015	22.4	19.2	26.2
United Republic of Tanzania	Kilimanjaro	48363	2000	23.5	20.9	26.5
United Republic of Tanzania	Kilimanjaro	48363	2005	22.6	20.1	25.4
United Republic of Tanzania	Kilimanjaro	48363	2010	21.2	18.5	24.2
United Republic of Tanzania	Kilimanjaro	48363	2015	17.4	14.3	20.8
United Republic of Tanzania	Kusini Pemba	48378	2000	29.9	26.2	33.7
United Republic of Tanzania	Kusini Pemba	48378	2005	25.3	22.1	28.6
United Republic of Tanzania	Kusini Pemba	48378	2010	22.1	18.9	25.5
United Republic of Tanzania	Kusini Pemba	48378	2015	18.4	15.0	22.5
United Republic of Tanzania	Kusini Unguja	48379	2000	31.4	28.0	35.1
United Republic of Tanzania	Kusini Unguja	48379	2005	27.9	25.0	31.2
United Republic of Tanzania	Kusini Unguja	48379	2010	26.6	23.5	30.2
United Republic of Tanzania	Kusini Unguja	48379	2015	21.3	18.0	25.2

Admin 0	Admin 1	GAUL Code	Year	Neonatal mortality (per 1,000 livebirths)		
				Estimate	Lower bound, 95% UI	Upper bound, 95% UI
United Republic of Tanzania	Lindi	48364	2000	41.7	36.9	46.9
United Republic of Tanzania	Lindi	48364	2005	35.5	31.4	39.8
United Republic of Tanzania	Lindi	48364	2010	28.1	24.5	31.9
United Republic of Tanzania	Lindi	48364	2015	25.8	21.5	30.5
United Republic of Tanzania	Manyara	48365	2000	27.8	24.8	31.0
United Republic of Tanzania	Manyara	48365	2005	25.2	22.5	28.0
United Republic of Tanzania	Manyara	48365	2010	22.3	19.7	25.1
United Republic of Tanzania	Manyara	48365	2015	19.3	16.3	22.6
United Republic of Tanzania	Mara	48366	2000	35.2	32.0	38.7
United Republic of Tanzania	Mara	48366	2005	30.8	27.8	33.8
United Republic of Tanzania	Mara	48366	2010	25.8	22.9	28.9
United Republic of Tanzania	Mara	48366	2015	21.1	17.9	24.5
United Republic of Tanzania	Mbeya	48367	2000	36.4	32.9	40.3
United Republic of Tanzania	Mbeya	48367	2005	32.8	29.5	36.3
United Republic of Tanzania	Mbeya	48367	2010	27.4	24.2	31.0
United Republic of Tanzania	Mbeya	48367	2015	24.7	20.8	28.8
United Republic of Tanzania	Mjini Magharibi	48382	2000	30.2	26.9	33.5
United Republic of Tanzania	Mjini Magharibi	48382	2005	27.1	24.2	30.1
United Republic of Tanzania	Mjini Magharibi	48382	2010	26.4	23.3	29.5
United Republic of Tanzania	Mjini Magharibi	48382	2015	21.4	17.9	25.3
United Republic of Tanzania	Morogoro	48368	2000	38.4	34.5	42.7
United Republic of Tanzania	Morogoro	48368	2005	33.0	29.5	36.9
United Republic of Tanzania	Morogoro	48368	2010	27.6	24.3	31.4
United Republic of Tanzania	Morogoro	48368	2015	24.8	21.0	29.8
United Republic of Tanzania	Mtwara	48369	2000	40.9	36.7	45.7
United Republic of Tanzania	Mtwara	48369	2005	34.7	30.6	38.8
United Republic of Tanzania	Mtwara	48369	2010	27.5	23.9	31.3
United Republic of Tanzania	Mtwara	48369	2015	25.2	20.7	29.8
United Republic of Tanzania	Mwanza	115006	2000	32.3	28.8	35.9
United Republic of Tanzania	Mwanza	115006	2005	29.0	25.9	32.1
United Republic of Tanzania	Mwanza	115006	2010	25.5	22.6	28.7
United Republic of Tanzania	Mwanza	115006	2015	21.0	17.8	24.7
United Republic of Tanzania	Njombe	115007	2000	36.2	32.0	40.5
United Republic of Tanzania	Njombe	115007	2005	31.9	28.4	36.0
United Republic of Tanzania	Njombe	115007	2010	26.8	23.4	30.3
United Republic of Tanzania	Njombe	115007	2015	23.8	19.9	28.4
United Republic of Tanzania	Pwani	48373	2000	36.8	33.2	40.6
United Republic of Tanzania	Pwani	48373	2005	31.9	28.8	35.4
United Republic of Tanzania	Pwani	48373	2010	27.8	24.9	31.3
United Republic of Tanzania	Pwani	48373	2015	24.3	20.7	28.7
United Republic of Tanzania	Rukwa	115008	2000	41.0	36.4	46.5
United Republic of Tanzania	Rukwa	115008	2005	37.4	33.1	41.9
United Republic of Tanzania	Rukwa	115008	2010	31.9	27.7	36.6
United Republic of Tanzania	Rukwa	115008	2015	26.6	21.9	31.9
United Republic of Tanzania	Ruvuma	48375	2000	39.7	35.3	44.2
United Republic of Tanzania	Ruvuma	48375	2005	34.4	30.5	38.5
United Republic of Tanzania	Ruvuma	48375	2010	28.2	24.6	32.2
United Republic of Tanzania	Ruvuma	48375	2015	25.0	20.9	29.5
United Republic of Tanzania	Shinyanga	115009	2000	32.5	29.0	36.3
United Republic of Tanzania	Shinyanga	115009	2005	29.8	26.7	33.4
United Republic of Tanzania	Shinyanga	115009	2010	26.2	23.1	29.5
United Republic of Tanzania	Shinyanga	115009	2015	21.3	18.1	24.9
United Republic of Tanzania	Simiyu	115010	2000	31.9	28.1	35.8
United Republic of Tanzania	Simiyu	115010	2005	28.3	25.2	31.6
United Republic of Tanzania	Simiyu	115010	2010	23.7	21.0	26.7
United Republic of Tanzania	Simiyu	115010	2015	19.6	16.6	23.1
United Republic of Tanzania	Singida	48377	2000	28.8	25.6	32.4
United Republic of Tanzania	Singida	48377	2005	26.4	23.3	29.8
United Republic of Tanzania	Singida	48377	2010	23.4	20.6	26.6
United Republic of Tanzania	Singida	48377	2015	19.7	16.4	23.4
United Republic of Tanzania	Tabora	48380	2000	32.0	28.4	35.9
United Republic of Tanzania	Tabora	48380	2005	29.2	26.1	32.4
United Republic of Tanzania	Tabora	48380	2010	26.2	23.2	29.8
United Republic of Tanzania	Tabora	48380	2015	20.9	17.7	24.6
United Republic of Tanzania	Tanga	48381	2000	32.7	29.2	36.7
United Republic of Tanzania	Tanga	48381	2005	29.4	26.3	33.0
United Republic of Tanzania	Tanga	48381	2010	24.3	21.5	27.4

Admin 0	Admin 1	GAUL Code	Year	Neonatal mortality (per 1,000 livebirths)		
				Estimate	Lower bound, 95% UI	Upper bound, 95% UI
United Republic of Tanzania	Tanga	48381	2015	21.4	17.7	25.4
Zambia	Central	3426	2000	33.5	30.3	37.2
Zambia	Central	3426	2005	29.4	26.4	32.8
Zambia	Central	3426	2010	25.3	22.3	28.5
Zambia	Central	3426	2015	21.3	18.2	24.8
Zambia	Copperbelt	3427	2000	29.0	25.6	32.4
Zambia	Copperbelt	3427	2005	26.5	23.5	29.7
Zambia	Copperbelt	3427	2010	24.1	21.0	27.6
Zambia	Copperbelt	3427	2015	20.3	16.9	24.3
Zambia	Eastern	3428	2000	38.1	35.0	41.4
Zambia	Eastern	3428	2005	33.6	30.9	36.6
Zambia	Eastern	3428	2010	28.8	26.2	31.7
Zambia	Eastern	3428	2015	25.8	22.8	29.4
Zambia	Luapula	3429	2000	34.4	30.7	38.6
Zambia	Luapula	3429	2005	30.0	26.9	33.4
Zambia	Luapula	3429	2010	24.9	22.0	28.1
Zambia	Luapula	3429	2015	21.7	18.5	25.8
Zambia	Lusaka	3430	2000	30.8	27.1	34.7
Zambia	Lusaka	3430	2005	28.6	25.2	32.3
Zambia	Lusaka	3430	2010	27.0	23.2	31.0
Zambia	Lusaka	3430	2015	22.2	18.4	26.6
Zambia	North-Western	3431	2000	31.3	27.7	34.8
Zambia	North-Western	3431	2005	26.8	23.8	30.3
Zambia	North-Western	3431	2010	22.4	19.4	25.6
Zambia	North-Western	3431	2015	18.9	15.8	22.5
Zambia	Northern	3432	2000	37.2	33.9	40.5
Zambia	Northern	3432	2005	32.2	29.5	35.1
Zambia	Northern	3432	2010	25.9	23.3	28.4
Zambia	Northern	3432	2015	22.3	19.4	25.7
Zambia	Southern	3433	2000	30.3	26.9	33.7
Zambia	Southern	3433	2005	27.6	24.6	31.0
Zambia	Southern	3433	2010	25.3	22.3	28.8
Zambia	Southern	3433	2015	20.8	17.4	24.5
Zambia	Western	3434	2000	38.5	34.1	43.3
Zambia	Western	3434	2005	33.3	29.5	37.4
Zambia	Western	3434	2010	28.3	24.9	32.4
Zambia	Western	3434	2015	23.7	19.8	28.0
Zimbabwe	Bulawayo	3435	2000	16.9	14.4	19.9
Zimbabwe	Bulawayo	3435	2005	20.4	17.5	23.6
Zimbabwe	Bulawayo	3435	2010	22.4	19.2	26.5
Zimbabwe	Bulawayo	3435	2015	19.0	15.5	23.4
Zimbabwe	Harare	3436	2000	22.7	20.0	25.7
Zimbabwe	Harare	3436	2005	26.5	23.8	29.5
Zimbabwe	Harare	3436	2010	29.9	26.6	33.5
Zimbabwe	Harare	3436	2015	24.8	21.0	29.2
Zimbabwe	Manicaland	3437	2000	27.1	24.4	30.1
Zimbabwe	Manicaland	3437	2005	29.6	26.8	32.6
Zimbabwe	Manicaland	3437	2010	29.7	26.5	33.1
Zimbabwe	Manicaland	3437	2015	26.0	22.6	29.9
Zimbabwe	Mashonaland Central	3438	2000	28.8	25.8	32.2
Zimbabwe	Mashonaland Central	3438	2005	31.2	28.0	34.4
Zimbabwe	Mashonaland Central	3438	2010	32.8	29.4	36.4
Zimbabwe	Mashonaland Central	3438	2015	27.9	23.8	32.5
Zimbabwe	Mashonaland East	69550	2000	26.1	23.5	28.8
Zimbabwe	Mashonaland East	69550	2005	28.7	26.1	31.4
Zimbabwe	Mashonaland East	69550	2010	30.7	27.6	33.9
Zimbabwe	Mashonaland East	69550	2015	26.3	22.7	30.2
Zimbabwe	Mashonaland West	3440	2000	27.2	24.4	30.4
Zimbabwe	Mashonaland West	3440	2005	30.9	27.9	34.1
Zimbabwe	Mashonaland West	3440	2010	32.4	28.9	36.1
Zimbabwe	Mashonaland West	3440	2015	27.3	23.6	31.4
Zimbabwe	Masvingo	3441	2000	22.0	19.3	25.2
Zimbabwe	Masvingo	3441	2005	24.3	21.6	27.3
Zimbabwe	Masvingo	3441	2010	25.7	22.5	29.1
Zimbabwe	Masvingo	3441	2015	22.7	19.3	26.7
Zimbabwe	Matabeleland North	69549	2000	19.6	17.3	22.2
Zimbabwe	Matabeleland North	69549	2005	22.4	19.9	25.3

Admin 0	Admin 1	GAUL Code	Year	Neonatal mortality (per 1,000 livebirths)		
				Estimate	Lower bound, 95% UI	Upper bound, 95% UI
Zimbabwe	Matabeleland North	69549	2010	22.2	19.4	25.3
Zimbabwe	Matabeleland North	69549	2015	19.7	16.7	23.1
Zimbabwe	Matabeleland South	3443	2000	18.5	16.3	21.1
Zimbabwe	Matabeleland South	3443	2005	21.5	19.0	24.1
Zimbabwe	Matabeleland South	3443	2010	21.8	19.3	24.9
Zimbabwe	Matabeleland South	3443	2015	19.9	16.9	23.3
Zimbabwe	Midlands	3444	2000	21.5	19.1	24.1
Zimbabwe	Midlands	3444	2005	25.4	22.9	28.2
Zimbabwe	Midlands	3444	2010	28.0	25.1	31.3
Zimbabwe	Midlands	3444	2015	24.2	20.8	27.9

Supplementary results Table 4. **Neonatal mortality estimates for subnational administrative levels 1 and 2 divisions in Africa, 2000, 2005, 2010, and 2015.** UI = uncertainty interval							
**Admin 0**	**Admin 1**	**Admin 2**	**GAUL Code**	**Year**	**Neonatal mortality (per 1,000 livebirths)**		
					**Estimate**	**Lower bound, 95% UI**	**Upper bound, 95% UI**
Angola	Bengo	Ambriz	4206	2000	39.4	31.0	49.8
Angola	Bengo	Ambriz	4206	2005	32.9	25.8	41.1
Angola	Bengo	Ambriz	4206	2010	28.0	21.7	35.3
Angola	Bengo	Ambriz	4206	2015	21.3	16.1	27.6
Angola	Bengo	Bula Atumba	4207	2000	44.2	34.2	55.8
Angola	Bengo	Bula Atumba	4207	2005	34.6	26.9	44.0
Angola	Bengo	Bula Atumba	4207	2010	26.2	20.0	33.6
Angola	Bengo	Bula Atumba	4207	2015	20.6	15.5	27.0
Angola	Bengo	Dande	4208	2000	39.6	32.5	48.3
Angola	Bengo	Dande	4208	2005	32.7	26.9	39.4
Angola	Bengo	Dande	4208	2010	27.8	22.6	33.9
Angola	Bengo	Dande	4208	2015	21.2	16.6	27.2
Angola	Bengo	Dembos	4209	2000	44.6	35.2	55.7
Angola	Bengo	Dembos	4209	2005	34.8	27.7	43.3
Angola	Bengo	Dembos	4209	2010	26.6	20.6	33.7
Angola	Bengo	Dembos	4209	2015	20.9	15.9	27.4
Angola	Bengo	Icolo E Bengo	4210	2000	37.8	30.9	45.7
Angola	Bengo	Icolo E Bengo	4210	2005	31.4	25.8	37.8
Angola	Bengo	Icolo E Bengo	4210	2010	27.3	21.9	33.6
Angola	Bengo	Icolo E Bengo	4210	2015	20.4	15.9	26.3
Angola	Bengo	Nambuangongo	4211	2000	42.2	33.3	52.9
Angola	Bengo	Nambuangongo	4211	2005	33.8	26.7	41.7
Angola	Bengo	Nambuangongo	4211	2010	27.4	21.5	34.3
Angola	Bengo	Nambuangongo	4211	2015	21.9	16.8	28.4
Angola	Bengo	Pango Aluquem	4212	2000	44.1	34.6	56.0
Angola	Bengo	Pango Aluquem	4212	2005	35.3	28.1	44.0
Angola	Bengo	Pango Aluquem	4212	2010	26.7	20.6	34.1
Angola	Bengo	Pango Aluquem	4212	2015	21.5	16.1	28.5
Angola	Bengo	Quissama	4213	2000	39.2	31.3	48.8
Angola	Bengo	Quissama	4213	2005	33.5	26.6	41.3
Angola	Bengo	Quissama	4213	2010	27.7	21.8	34.3
Angola	Bengo	Quissama	4213	2015	21.4	16.4	27.5
Angola	Benguela	Baia Farta	4214	2000	41.4	31.1	54.0
Angola	Benguela	Baia Farta	4214	2005	36.6	27.9	47.5
Angola	Benguela	Baia Farta	4214	2010	30.9	23.2	40.4
Angola	Benguela	Baia Farta	4214	2015	22.9	16.7	30.5
Angola	Benguela	Balombo	4215	2000	43.0	33.1	55.5
Angola	Benguela	Balombo	4215	2005	36.5	28.6	45.5
Angola	Benguela	Balombo	4215	2010	28.5	21.8	36.6
Angola	Benguela	Balombo	4215	2015	21.1	15.7	27.7
Angola	Benguela	Benguela	4216	2000	35.8	27.3	45.5
Angola	Benguela	Benguela	4216	2005	33.1	25.5	42.5
Angola	Benguela	Benguela	4216	2010	28.9	22.0	37.2
Angola	Benguela	Benguela	4216	2015	21.7	15.8	28.8
Angola	Benguela	Bocoio	4217	2000	42.4	33.4	53.1
Angola	Benguela	Bocoio	4217	2005	36.0	28.6	44.7
Angola	Benguela	Bocoio	4217	2010	29.3	22.8	36.8
Angola	Benguela	Bocoio	4217	2015	21.5	16.0	27.9
Angola	Benguela	Caimbambo	4218	2000	47.7	36.9	60.5
Angola	Benguela	Caimbambo	4218	2005	40.9	31.3	51.5
Angola	Benguela	Caimbambo	4218	2010	33.2	25.1	42.0
Angola	Benguela	Caimbambo	4218	2015	24.6	18.2	32.6
Angola	Benguela	Chongoroi	4219	2000	48.0	38.1	60.8
Angola	Benguela	Chongoroi	4219	2005	41.1	32.6	51.9
Angola	Benguela	Chongoroi	4219	2010	33.6	26.2	42.2
Angola	Benguela	Chongoroi	4219	2015	25.0	18.9	32.6
Angola	Benguela	Cubal	4220	2000	49.1	38.5	62.0
Angola	Benguela	Cubal	4220	2005	42.3	33.3	52.3
Angola	Benguela	Cubal	4220	2010	33.4	25.7	42.3
Angola	Benguela	Cubal	4220	2015	25.4	18.6	33.1
Angola	Benguela	Ganda	4221	2000	47.3	36.9	59.9
Angola	Benguela	Ganda	4221	2005	40.5	31.8	50.0
Angola	Benguela	Ganda	4221	2010	32.1	25.0	40.5
Angola	Benguela	Ganda	4221	2015	24.3	18.3	31.3
Angola	Benguela	Lobito	4222	2000	34.3	26.1	44.2
Angola	Benguela	Lobito	4222	2005	31.5	24.4	40.7
Angola	Benguela	Lobito	4222	2010	27.8	21.0	36.0
Angola	Benguela	Lobito	4222	2015	20.9	15.3	28.1
Angola	Bie	Andulo	4223	2000	34.7	25.8	44.3
Angola	Bie	Andulo	4223	2005	28.4	21.5	36.1
Angola	Bie	Andulo	4223	2010	22.8	16.9	29.4
Angola	Bie	Andulo	4223	2015	17.0	12.3	22.7
Angola	Bie	Camacupa	4224	2000	31.3	23.0	41.1
Angola	Bie	Camacupa	4224	2005	26.7	20.2	35.0
Angola	Bie	Camacupa	4224	2010	22.2	16.3	29.5
Angola	Bie	Camacupa	4224	2015	16.7	12.0	22.5
Angola	Bie	Catabola	4225	2000	31.2	23.2	40.7
Angola	Bie	Catabola	4225	2005	26.9	19.8	35.0
Angola	Bie	Catabola	4225	2010	22.1	16.3	29.1
Angola	Bie	Catabola	4225	2015	16.7	11.9	22.6
Angola	Bie	Chinguar	4226	2000	32.0	24.2	40.7
Angola	Bie	Chinguar	4226	2005	27.0	20.7	34.2

Admin 0	Admin 1	Admin 2	GAUL Code	Year	Neonatal mortality (per 1,000 livebirths)		
					Estimate	Lower bound, 95% UI	Upper bound, 95% UI
Angola	Bie	Chinguar	4226	2010	22.6	17.2	29.2
Angola	Bie	Chinguar	4226	2015	16.7	12.4	22.3
Angola	Bie	Chitembo	4227	2000	32.2	23.9	42.5
Angola	Bie	Chitembo	4227	2005	26.8	20.2	34.3
Angola	Bie	Chitembo	4227	2010	22.3	16.7	29.3
Angola	Bie	Chitembo	4227	2015	16.7	12.1	22.4
Angola	Bie	Cuemba	4228	2000	32.1	23.1	42.4
Angola	Bie	Cuemba	4228	2005	27.2	20.2	36.1
Angola	Bie	Cuemba	4228	2010	22.6	16.3	30.1
Angola	Bie	Cuemba	4228	2015	17.0	12.3	23.0
Angola	Bie	Cunhinga	4229	2000	31.6	23.3	40.9
Angola	Bie	Cunhinga	4229	2005	26.7	19.6	34.0
Angola	Bie	Cunhinga	4229	2010	22.0	16.3	28.7
Angola	Bie	Cunhinga	4229	2015	16.4	11.8	22.2
Angola	Bie	Kuito	4230	2000	30.3	22.7	39.8
Angola	Bie	Kuito	4230	2005	26.0	19.5	33.7
Angola	Bie	Kuito	4230	2010	21.9	16.3	28.9
Angola	Bie	Kuito	4230	2015	16.7	12.2	22.3
Angola	Bie	N'harea	4231	2000	33.1	24.6	43.9
Angola	Bie	N'harea	4231	2005	27.5	20.4	35.9
Angola	Bie	N'harea	4231	2010	22.3	16.4	29.4
Angola	Bie	N'harea	4231	2015	16.7	12.0	22.6
Angola	Cabinda	Belize	4232	2000	36.0	29.7	43.0
Angola	Cabinda	Belize	4232	2005	31.7	26.6	37.3
Angola	Cabinda	Belize	4232	2010	23.0	19.0	27.8
Angola	Cabinda	Belize	4232	2015	19.8	15.4	24.7
Angola	Cabinda	Buco-zau	4233	2000	34.9	29.3	41.5
Angola	Cabinda	Buco-zau	4233	2005	30.3	25.3	36.2
Angola	Cabinda	Buco-zau	4233	2010	22.4	18.3	26.9
Angola	Cabinda	Buco-zau	4233	2015	18.7	14.7	23.5
Angola	Cabinda	Cabinda	4234	2000	35.0	28.3	43.0
Angola	Cabinda	Cabinda	4234	2005	28.9	23.1	35.3
Angola	Cabinda	Cabinda	4234	2010	23.4	18.8	28.7
Angola	Cabinda	Cabinda	4234	2015	18.9	14.6	23.9
Angola	Cabinda	Cacongo (landana)	4235	2000	32.0	26.7	38.6
Angola	Cabinda	Cacongo (landana)	4235	2005	27.2	22.5	32.3
Angola	Cabinda	Cacongo (landana)	4235	2010	21.8	17.8	25.9
Angola	Cabinda	Cacongo (landana)	4235	2015	17.5	13.6	21.8
Angola	Cuando Cubango	Calai	4236	2000	28.5	22.2	36.1
Angola	Cuando Cubango	Calai	4236	2005	25.0	19.6	31.4
Angola	Cuando Cubango	Calai	4236	2010	22.2	17.2	28.2
Angola	Cuando Cubango	Calai	4236	2015	17.1	12.9	22.4
Angola	Cuando Cubango	Cuangar	4237	2000	27.5	21.6	35.0
Angola	Cuando Cubango	Cuangar	4237	2005	24.2	19.1	30.6
Angola	Cuando Cubango	Cuangar	4237	2010	20.8	16.1	26.4
Angola	Cuando Cubango	Cuangar	4237	2015	16.4	12.2	21.8
Angola	Cuando Cubango	Cuchi	4238	2000	33.0	23.6	44.0
Angola	Cuando Cubango	Cuchi	4238	2005	28.2	20.8	37.1
Angola	Cuando Cubango	Cuchi	4238	2010	23.1	16.7	30.9
Angola	Cuando Cubango	Cuchi	4238	2015	17.4	12.4	24.2
Angola	Cuando Cubango	Cuito Cuanavale	4239	2000	31.4	22.7	41.4
Angola	Cuando Cubango	Cuito Cuanavale	4239	2005	26.3	19.1	35.0
Angola	Cuando Cubango	Cuito Cuanavale	4239	2010	22.1	15.6	29.8
Angola	Cuando Cubango	Cuito Cuanavale	4239	2015	17.2	11.9	23.7
Angola	Cuando Cubango	Dirico	4240	2000	34.7	26.9	44.2
Angola	Cuando Cubango	Dirico	4240	2005	30.2	23.2	38.0
Angola	Cuando Cubango	Dirico	4240	2010	26.0	20.0	33.7
Angola	Cuando Cubango	Dirico	4240	2015	20.2	14.9	26.2
Angola	Cuando Cubango	Mavinga	4241	2000	35.4	27.2	45.3
Angola	Cuando Cubango	Mavinga	4241	2005	29.9	22.7	38.4
Angola	Cuando Cubango	Mavinga	4241	2010	25.6	19.4	33.1
Angola	Cuando Cubango	Mavinga	4241	2015	20.4	15.3	26.8
Angola	Cuando Cubango	Menongue	4242	2000	30.9	22.6	40.4
Angola	Cuando Cubango	Menongue	4242	2005	25.9	19.5	33.9
Angola	Cuando Cubango	Menongue	4242	2010	21.5	15.8	29.0
Angola	Cuando Cubango	Menongue	4242	2015	16.2	11.6	22.2
Angola	Cuando Cubango	Nankova	4243	2000	29.5	21.8	39.9
Angola	Cuando Cubango	Nankova	4243	2005	25.2	18.7	34.0
Angola	Cuando Cubango	Nankova	4243	2010	21.4	15.5	28.8
Angola	Cuando Cubango	Nankova	4243	2015	17.0	11.9	23.4
Angola	Cuando Cubango	Rivungo	4244	2000	43.8	34.6	54.4
Angola	Cuando Cubango	Rivungo	4244	2005	37.1	28.9	46.0
Angola	Cuando Cubango	Rivungo	4244	2010	31.0	24.0	39.2
Angola	Cuando Cubango	Rivungo	4244	2015	24.5	18.5	31.2
Angola	Cuanza Sul	Amboim	4245	2000	42.2	32.2	54.0
Angola	Cuanza Sul	Amboim	4245	2005	37.6	28.6	48.0
Angola	Cuanza Sul	Amboim	4245	2010	27.1	20.3	35.5
Angola	Cuanza Sul	Amboim	4245	2015	21.4	15.7	28.0
Angola	Cuanza Sul	Cassongue	4246	2000	39.6	30.5	51.2
Angola	Cuanza Sul	Cassongue	4246	2005	36.0	27.9	45.0
Angola	Cuanza Sul	Cassongue	4246	2010	25.5	19.6	32.5
Angola	Cuanza Sul	Cassongue	4246	2015	20.0	14.7	26.1
Angola	Cuanza Sul	Conda	4247	2000	41.7	31.9	53.4
Angola	Cuanza Sul	Conda	4247	2005	37.1	28.1	47.1
Angola	Cuanza Sul	Conda	4247	2010	27.3	20.6	35.6

Admin 0	Admin 1	Admin 2	GAUL Code	Year	Neonatal mortality (per 1,000 livebirths)		
					Estimate	Lower bound, 95% UI	Upper bound, 95% UI
Angola	Cuanza Sul	Conda	4247	2015	21.2	15.6	27.8
Angola	Cuanza Sul	Ebo	4248	2000	41.7	32.4	53.7
Angola	Cuanza Sul	Ebo	4248	2005	37.4	28.7	47.7
Angola	Cuanza Sul	Ebo	4248	2010	26.8	20.1	34.8
Angola	Cuanza Sul	Ebo	4248	2015	21.3	15.4	28.3
Angola	Cuanza Sul	Libolo	4249	2000	43.6	33.8	55.5
Angola	Cuanza Sul	Libolo	4249	2005	38.1	29.4	48.8
Angola	Cuanza Sul	Libolo	4249	2010	27.2	20.5	35.4
Angola	Cuanza Sul	Libolo	4249	2015	21.6	16.0	28.5
Angola	Cuanza Sul	Mussende	4250	2000	39.9	30.5	50.8
Angola	Cuanza Sul	Mussende	4250	2005	34.1	26.0	43.9
Angola	Cuanza Sul	Mussende	4250	2010	24.5	18.4	31.9
Angola	Cuanza Sul	Mussende	4250	2015	19.6	14.4	26.0
Angola	Cuanza Sul	Porto Amboim	4251	2000	37.4	28.3	48.6
Angola	Cuanza Sul	Porto Amboim	4251	2005	34.6	26.1	44.3
Angola	Cuanza Sul	Porto Amboim	4251	2010	26.3	19.7	34.5
Angola	Cuanza Sul	Porto Amboim	4251	2015	20.5	15.1	27.6
Angola	Cuanza Sul	Quibala	4252	2000	40.7	31.8	51.5
Angola	Cuanza Sul	Quibala	4252	2005	36.1	28.0	45.4
Angola	Cuanza Sul	Quibala	4252	2010	25.4	19.4	32.8
Angola	Cuanza Sul	Quibala	4252	2015	20.1	15.0	26.5
Angola	Cuanza Sul	Quilenda	4253	2000	43.3	33.6	55.8
Angola	Cuanza Sul	Quilenda	4253	2005	38.4	29.6	48.8
Angola	Cuanza Sul	Quilenda	4253	2010	27.8	21.0	35.8
Angola	Cuanza Sul	Quilenda	4253	2015	22.1	16.2	29.5
Angola	Cuanza Sul	Seles	4254	2000	40.7	31.4	52.0
Angola	Cuanza Sul	Seles	4254	2005	36.3	28.0	45.7
Angola	Cuanza Sul	Seles	4254	2010	27.0	20.6	34.3
Angola	Cuanza Sul	Seles	4254	2015	20.8	15.5	27.1
Angola	Cuanza Sul	Sumbe	4255	2000	37.7	29.1	49.1
Angola	Cuanza Sul	Sumbe	4255	2005	33.2	25.6	42.6
Angola	Cuanza Sul	Sumbe	4255	2010	26.4	20.0	34.5
Angola	Cuanza Sul	Sumbe	4255	2015	20.2	15.0	27.3
Angola	Cuanza Sul	Waku Kungu	4256	2000	39.1	30.0	51.1
Angola	Cuanza Sul	Waku Kungu	4256	2005	35.1	26.8	44.7
Angola	Cuanza Sul	Waku Kungu	4256	2010	24.8	18.9	31.8
Angola	Cuanza Sul	Waku Kungu	4256	2015	19.2	14.0	25.3
Angola	Cunene	Cahama	4257	2000	33.2	25.7	41.9
Angola	Cunene	Cahama	4257	2005	28.9	22.7	36.2
Angola	Cunene	Cahama	4257	2010	24.2	18.7	31.4
Angola	Cunene	Cahama	4257	2015	18.3	13.8	23.8
Angola	Cunene	Cuanhama	4258	2000	30.2	23.7	38.3
Angola	Cunene	Cuanhama	4258	2005	27.7	21.6	35.1
Angola	Cunene	Cuanhama	4258	2010	22.5	17.6	28.9
Angola	Cunene	Cuanhama	4258	2015	17.5	13.2	23.2
Angola	Cunene	Curoca	4259	2000	31.1	23.5	39.5
Angola	Cunene	Curoca	4259	2005	27.3	21.1	34.4
Angola	Cunene	Curoca	4259	2010	23.1	17.3	29.5
Angola	Cunene	Curoca	4259	2015	17.4	12.7	22.8
Angola	Cunene	Cuvelai	4260	2000	32.6	24.3	43.2
Angola	Cunene	Cuvelai	4260	2005	29.6	22.3	39.5
Angola	Cunene	Cuvelai	4260	2010	24.0	17.9	31.9
Angola	Cunene	Cuvelai	4260	2015	18.7	13.7	24.9
Angola	Cunene	Namacunde	4261	2000	29.5	23.7	36.6
Angola	Cunene	Namacunde	4261	2005	27.0	21.6	33.4
Angola	Cunene	Namacunde	4261	2010	22.2	17.5	28.3
Angola	Cunene	Namacunde	4261	2015	17.5	13.3	22.7
Angola	Cunene	Ombadja	4262	2000	29.8	23.7	37.4
Angola	Cunene	Ombadja	4262	2005	27.4	21.9	34.3
Angola	Cunene	Ombadja	4262	2010	22.7	17.8	29.2
Angola	Cunene	Ombadja	4262	2015	17.2	13.0	22.1
Angola	Huambo	Bailundo	4263	2000	35.0	27.2	44.1
Angola	Huambo	Bailundo	4263	2005	31.1	24.4	39.0
Angola	Huambo	Bailundo	4263	2010	22.8	17.3	28.8
Angola	Huambo	Bailundo	4263	2015	17.5	12.9	23.1
Angola	Huambo	Caala	4264	2000	39.0	30.5	48.9
Angola	Huambo	Caala	4264	2005	33.4	26.8	41.6
Angola	Huambo	Caala	4264	2010	26.0	20.3	33.4
Angola	Huambo	Caala	4264	2015	20.3	15.4	26.6
Angola	Huambo	Ekunha	4265	2000	38.2	29.5	48.7
Angola	Huambo	Ekunha	4265	2005	32.7	26.0	41.7
Angola	Huambo	Ekunha	4265	2010	24.8	19.1	32.1
Angola	Huambo	Ekunha	4265	2015	18.9	14.0	25.0
Angola	Huambo	Huambo	4266	2000	35.8	27.6	45.3
Angola	Huambo	Huambo	4266	2005	30.8	24.2	38.5
Angola	Huambo	Huambo	4266	2010	24.1	18.9	30.9
Angola	Huambo	Huambo	4266	2015	18.9	14.1	25.1
Angola	Huambo	Katchiungo	4267	2000	32.5	25.2	40.7
Angola	Huambo	Katchiungo	4267	2005	27.8	21.8	34.8
Angola	Huambo	Katchiungo	4267	2010	22.2	17.2	28.5
Angola	Huambo	Katchiungo	4267	2015	17.0	12.9	22.6
Angola	Huambo	Londuimbali	4268	2000	38.5	30.0	49.5
Angola	Huambo	Londuimbali	4268	2005	35.1	27.1	44.1
Angola	Huambo	Londuimbali	4268	2010	24.6	18.9	31.8
Angola	Huambo	Londuimbali	4268	2015	19.1	14.0	25.4

Admin 0	Admin 1	Admin 2	GAUL Code	Year	Neonatal mortality (per 1,000 livebirths)		
					Estimate	Lower bound, 95% UI	Upper bound, 95% UI
Angola	Huambo	Longonjo	4269	2000	42.5	33.1	53.8
Angola	Huambo	Longonjo	4269	2005	37.6	30.0	47.0
Angola	Huambo	Longonjo	4269	2010	27.6	21.5	35.6
Angola	Huambo	Longonjo	4269	2015	21.4	16.2	28.1
Angola	Huambo	Mungo	4270	2000	33.1	25.1	42.3
Angola	Huambo	Mungo	4270	2005	29.3	22.1	37.6
Angola	Huambo	Mungo	4270	2010	21.7	16.3	27.9
Angola	Huambo	Mungo	4270	2015	16.6	12.0	22.0
Angola	Huambo	Tchikala-tcholo.	4271	2000	34.9	27.7	43.5
Angola	Huambo	Tchikala-tcholo.	4271	2005	29.7	23.8	36.9
Angola	Huambo	Tchikala-tcholo.	4271	2010	23.4	18.2	29.8
Angola	Huambo	Tchikala-tcholo.	4271	2015	17.8	13.4	23.6
Angola	Huambo	Tchindjenje	4272	2000	43.8	34.0	55.4
Angola	Huambo	Tchindjenje	4272	2005	40.3	31.6	50.1
Angola	Huambo	Tchindjenje	4272	2010	28.5	22.0	36.5
Angola	Huambo	Tchindjenje	4272	2015	21.9	16.3	28.5
Angola	Huambo	Ukuma	4273	2000	42.3	32.5	53.9
Angola	Huambo	Ukuma	4273	2005	38.2	30.1	47.9
Angola	Huambo	Ukuma	4273	2010	27.3	20.8	35.3
Angola	Huambo	Ukuma	4273	2015	21.0	15.7	27.5
Angola	Huila	Caconda	4274	2000	45.0	35.3	57.4
Angola	Huila	Caconda	4274	2005	39.5	31.3	48.5
Angola	Huila	Caconda	4274	2010	31.8	24.7	40.3
Angola	Huila	Caconda	4274	2015	24.1	18.1	30.9
Angola	Huila	Cacula	4275	2000	40.7	32.4	51.3
Angola	Huila	Cacula	4275	2005	35.1	28.3	43.3
Angola	Huila	Cacula	4275	2010	29.9	23.6	37.2
Angola	Huila	Cacula	4275	2015	21.8	16.7	27.8
Angola	Huila	Caluquembe	4276	2000	46.4	37.0	57.6
Angola	Huila	Caluquembe	4276	2005	41.0	32.8	49.7
Angola	Huila	Caluquembe	4276	2010	33.0	25.6	41.4
Angola	Huila	Caluquembe	4276	2015	25.0	19.0	31.7
Angola	Huila	Chibia	4277	2000	39.6	31.4	49.0
Angola	Huila	Chibia	4277	2005	34.4	27.4	41.9
Angola	Huila	Chibia	4277	2010	28.2	21.9	34.8
Angola	Huila	Chibia	4277	2015	20.9	16.0	26.6
Angola	Huila	Chicomba	4278	2000	44.9	35.7	56.0
Angola	Huila	Chicomba	4278	2005	40.3	32.1	49.4
Angola	Huila	Chicomba	4278	2010	32.4	25.4	40.6
Angola	Huila	Chicomba	4278	2015	24.9	19.0	31.8
Angola	Huila	Chipindo	4279	2000	40.4	31.4	51.8
Angola	Huila	Chipindo	4279	2005	35.6	27.6	44.4
Angola	Huila	Chipindo	4279	2010	28.6	21.9	36.9
Angola	Huila	Chipindo	4279	2015	21.6	16.0	28.3
Angola	Huila	Gambos	4280	2000	38.2	30.2	47.5
Angola	Huila	Gambos	4280	2005	32.9	25.9	40.7
Angola	Huila	Gambos	4280	2010	26.9	20.9	33.9
Angola	Huila	Gambos	4280	2015	19.9	15.2	25.5
Angola	Huila	Humpata	4281	2000	35.6	27.5	44.7
Angola	Huila	Humpata	4281	2005	31.7	24.7	39.4
Angola	Huila	Humpata	4281	2010	27.6	21.3	34.7
Angola	Huila	Humpata	4281	2015	20.2	15.2	26.2
Angola	Huila	Jamba	4282	2000	38.1	28.4	49.8
Angola	Huila	Jamba	4282	2005	33.4	25.5	43.1
Angola	Huila	Jamba	4282	2010	27.6	20.5	35.8
Angola	Huila	Jamba	4282	2015	21.1	15.3	28.1
Angola	Huila	Kuvango	4283	2000	36.0	27.5	46.6
Angola	Huila	Kuvango	4283	2005	31.0	23.6	39.0
Angola	Huila	Kuvango	4283	2010	25.5	19.2	33.1
Angola	Huila	Kuvango	4283	2015	19.2	13.8	25.3
Angola	Huila	Lubango	4284	2000	35.6	27.8	44.5
Angola	Huila	Lubango	4284	2005	32.0	25.3	39.9
Angola	Huila	Lubango	4284	2010	27.6	21.4	34.8
Angola	Huila	Lubango	4284	2015	20.5	15.4	26.2
Angola	Huila	Matala	4285	2000	40.2	31.5	51.6
Angola	Huila	Matala	4285	2005	35.2	27.6	44.0
Angola	Huila	Matala	4285	2010	29.1	22.3	37.7
Angola	Huila	Matala	4285	2015	22.2	16.9	29.1
Angola	Huila	Quilengues	4286	2000	45.7	36.3	57.5
Angola	Huila	Quilengues	4286	2005	38.3	30.3	47.3
Angola	Huila	Quilengues	4286	2010	32.1	25.0	40.0
Angola	Huila	Quilengues	4286	2015	23.7	17.7	30.0
Angola	Huila	Quipungo	4287	2000	42.4	34.2	52.9
Angola	Huila	Quipungo	4287	2005	37.5	30.1	46.4
Angola	Huila	Quipungo	4287	2010	30.4	23.8	38.1
Angola	Huila	Quipungo	4287	2015	22.8	17.5	29.0
Angola	Kuanza Norte	Ambaca	4288	2000	42.5	33.8	53.3
Angola	Kuanza Norte	Ambaca	4288	2005	32.2	25.7	39.8
Angola	Kuanza Norte	Ambaca	4288	2010	24.6	19.3	30.9
Angola	Kuanza Norte	Ambaca	4288	2015	19.6	14.9	25.8
Angola	Kuanza Norte	Banga	4289	2000	43.6	34.0	54.8
Angola	Kuanza Norte	Banga	4289	2005	34.4	27.0	42.7
Angola	Kuanza Norte	Banga	4289	2010	25.7	20.0	32.8
Angola	Kuanza Norte	Banga	4289	2015	20.4	15.5	27.5
Angola	Kuanza Norte	Bolongongo	4290	2000	44.3	35.1	54.9

Admin 0	Admin 1	Admin 2	GAUL Code	Year	Neonatal mortality (per 1,000 livebirths)		
					Estimate	Lower bound, 95% UI	Upper bound, 95% UI
Angola	Kuanza Norte	Bolongongo	4290	2005	34.1	27.3	41.8
Angola	Kuanza Norte	Bolongongo	4290	2010	25.6	20.0	32.3
Angola	Kuanza Norte	Bolongongo	4290	2015	20.4	15.5	26.9
Angola	Kuanza Norte	Cambambe	4291	2000	43.3	34.2	54.7
Angola	Kuanza Norte	Cambambe	4291	2005	36.6	28.3	46.0
Angola	Kuanza Norte	Cambambe	4291	2010	27.4	21.0	35.4
Angola	Kuanza Norte	Cambambe	4291	2015	21.4	16.1	27.9
Angola	Kuanza Norte	Cazengo (ndalatando)	4292	2000	43.6	33.6	55.2
Angola	Kuanza Norte	Cazengo (ndalatando)	4292	2005	36.0	27.8	45.5
Angola	Kuanza Norte	Cazengo (ndalatando)	4292	2010	26.3	20.1	34.3
Angola	Kuanza Norte	Cazengo (ndalatando)	4292	2015	20.9	15.4	27.7
Angola	Kuanza Norte	Golungo Alto	4293	2000	45.0	34.6	56.8
Angola	Kuanza Norte	Golungo Alto	4293	2005	36.6	28.4	45.8
Angola	Kuanza Norte	Golungo Alto	4293	2010	26.8	20.2	34.1
Angola	Kuanza Norte	Golungo Alto	4293	2015	21.2	15.6	27.8
Angola	Kuanza Norte	Gonguembo	4294	2000	44.1	34.1	55.3
Angola	Kuanza Norte	Gonguembo	4294	2005	35.5	27.7	44.3
Angola	Kuanza Norte	Gonguembo	4294	2010	26.6	20.3	34.1
Angola	Kuanza Norte	Gonguembo	4294	2015	20.7	15.6	27.2
Angola	Kuanza Norte	Lucala	4295	2000	44.0	34.0	56.3
Angola	Kuanza Norte	Lucala	4295	2005	36.3	28.2	46.2
Angola	Kuanza Norte	Lucala	4295	2010	26.2	20.3	34.1
Angola	Kuanza Norte	Lucala	4295	2015	20.9	15.4	27.7
Angola	Kuanza Norte	Quiculungo	4296	2000	43.6	33.7	55.0
Angola	Kuanza Norte	Quiculungo	4296	2005	33.2	26.0	40.7
Angola	Kuanza Norte	Quiculungo	4296	2010	25.0	19.3	31.9
Angola	Kuanza Norte	Quiculungo	4296	2015	19.8	14.8	26.3
Angola	Kuanza Norte	Samba Caju	4297	2000	43.0	33.7	53.8
Angola	Kuanza Norte	Samba Caju	4297	2005	33.8	26.8	42.0
Angola	Kuanza Norte	Samba Caju	4297	2010	25.1	19.5	32.2
Angola	Kuanza Norte	Samba Caju	4297	2015	20.1	15.3	26.8
Angola	Luanda	Cacuaco	4298	2000	33.3	28.0	39.2
Angola	Luanda	Cacuaco	4298	2005	29.9	25.4	35.1
Angola	Luanda	Cacuaco	4298	2010	27.3	22.6	32.7
Angola	Luanda	Cacuaco	4298	2015	20.1	15.9	25.3
Angola	Luanda	Luanda	4299	2000	31.7	26.4	37.4
Angola	Luanda	Luanda	4299	2005	29.4	24.8	34.4
Angola	Luanda	Luanda	4299	2010	27.0	22.2	32.2
Angola	Luanda	Luanda	4299	2015	20.0	15.7	25.0
Angola	Luanda	Viana	4300	2000	32.7	27.3	38.3
Angola	Luanda	Viana	4300	2005	29.7	25.1	34.8
Angola	Luanda	Viana	4300	2010	27.3	22.5	32.7
Angola	Luanda	Viana	4300	2015	20.3	16.0	25.4
Angola	Lunda Norte	Cambulo	4301	2000	41.3	31.4	52.0
Angola	Lunda Norte	Cambulo	4301	2005	35.3	26.9	44.2
Angola	Lunda Norte	Cambulo	4301	2010	27.5	20.4	35.7
Angola	Lunda Norte	Cambulo	4301	2015	21.6	15.9	28.1
Angola	Lunda Norte	Capenda Camulemba	4302	2000	35.5	26.3	46.8
Angola	Lunda Norte	Capenda Camulemba	4302	2005	28.3	21.1	37.3
Angola	Lunda Norte	Capenda Camulemba	4302	2010	22.2	16.1	30.0
Angola	Lunda Norte	Capenda Camulemba	4302	2015	17.2	12.1	23.4
Angola	Lunda Norte	Caungula	4303	2000	34.7	25.6	45.9
Angola	Lunda Norte	Caungula	4303	2005	27.3	20.2	36.0
Angola	Lunda Norte	Caungula	4303	2010	21.7	15.7	29.1
Angola	Lunda Norte	Caungula	4303	2015	17.0	11.9	22.8
Angola	Lunda Norte	Chitato	4304	2000	37.1	27.6	48.4
Angola	Lunda Norte	Chitato	4304	2005	30.5	23.2	39.3
Angola	Lunda Norte	Chitato	4304	2010	24.6	18.1	32.1
Angola	Lunda Norte	Chitato	4304	2015	19.0	13.7	25.2
Angola	Lunda Norte	Cuango	4305	2000	35.5	26.6	47.3
Angola	Lunda Norte	Cuango	4305	2005	27.3	20.0	36.3
Angola	Lunda Norte	Cuango	4305	2010	21.3	15.4	28.3
Angola	Lunda Norte	Cuango	4305	2015	17.3	12.3	23.1
Angola	Lunda Norte	Cuilo	4306	2000	35.5	25.8	47.0
Angola	Lunda Norte	Cuilo	4306	2005	29.2	21.6	38.4
Angola	Lunda Norte	Cuilo	4306	2010	23.4	16.8	31.3
Angola	Lunda Norte	Cuilo	4306	2015	17.7	12.2	23.6
Angola	Lunda Norte	Lubalo	4307	2000	34.9	25.8	45.7
Angola	Lunda Norte	Lubalo	4307	2005	28.3	21.1	36.6
Angola	Lunda Norte	Lubalo	4307	2010	22.7	16.5	29.8
Angola	Lunda Norte	Lubalo	4307	2015	17.5	12.4	23.5
Angola	Lunda Norte	Lucapa	4308	2000	38.5	27.7	49.8
Angola	Lunda Norte	Lucapa	4308	2005	31.8	23.6	41.5
Angola	Lunda Norte	Lucapa	4308	2010	25.3	18.3	33.6
Angola	Lunda Norte	Lucapa	4308	2015	19.3	13.7	25.9
Angola	Lunda Norte	Xa-muteba	4309	2000	35.9	27.3	46.0
Angola	Lunda Norte	Xa-muteba	4309	2005	28.5	21.5	36.8
Angola	Lunda Norte	Xa-muteba	4309	2010	21.8	16.3	28.2
Angola	Lunda Norte	Xa-muteba	4309	2015	17.2	12.3	22.4
Angola	Lunda Sul	Cacolo	4310	2000	33.2	24.2	43.5
Angola	Lunda Sul	Cacolo	4310	2005	27.6	20.4	36.9
Angola	Lunda Sul	Cacolo	4310	2010	22.5	16.8	30.2
Angola	Lunda Sul	Cacolo	4310	2015	17.2	12.6	23.0
Angola	Lunda Sul	Dala	4311	2000	35.4	26.4	45.9
Angola	Lunda Sul	Dala	4311	2005	29.7	22.3	38.7

Admin 0	Admin 1	Admin 2	GAUL Code	Year	Neonatal mortality (per 1,000 livebirths)		
					Estimate	Lower bound, 95% UI	Upper bound, 95% UI
Angola	Lunda Sul	Dala	4311	2010	24.6	18.2	32.3
Angola	Lunda Sul	Dala	4311	2015	18.9	13.5	25.0
Angola	Lunda Sul	Muconda	4312	2000	43.8	33.6	55.8
Angola	Lunda Sul	Muconda	4312	2005	37.2	28.8	48.1
Angola	Lunda Sul	Muconda	4312	2010	30.1	23.0	38.9
Angola	Lunda Sul	Muconda	4312	2015	23.7	17.4	31.3
Angola	Lunda Sul	Saurimo	4313	2000	36.8	27.1	48.6
Angola	Lunda Sul	Saurimo	4313	2005	30.5	22.6	40.5
Angola	Lunda Sul	Saurimo	4313	2010	25.3	18.2	33.2
Angola	Lunda Sul	Saurimo	4313	2015	20.1	14.3	26.8
Angola	Malanje	Cacuso	4314	2000	43.5	33.6	55.1
Angola	Malanje	Cacuso	4314	2005	34.6	27.0	43.6
Angola	Malanje	Cacuso	4314	2010	26.8	20.7	34.2
Angola	Malanje	Cacuso	4314	2015	20.7	15.5	26.9
Angola	Malanje	Calandula	4315	2000	41.4	32.5	52.2
Angola	Malanje	Calandula	4315	2005	31.8	25.5	39.3
Angola	Malanje	Calandula	4315	2010	25.3	19.9	32.3
Angola	Malanje	Calandula	4315	2015	19.6	14.9	25.6
Angola	Malanje	Cambundi-catembo	4316	2000	35.8	27.0	47.4
Angola	Malanje	Cambundi-catembo	4316	2005	28.9	21.4	38.2
Angola	Malanje	Cambundi-catembo	4316	2010	22.9	16.8	30.7
Angola	Malanje	Cambundi-catembo	4316	2015	17.5	12.2	23.3
Angola	Malanje	Cangandala	4317	2000	39.3	29.7	50.7
Angola	Malanje	Cangandala	4317	2005	31.9	24.4	41.2
Angola	Malanje	Cangandala	4317	2010	24.9	18.8	33.0
Angola	Malanje	Cangandala	4317	2015	19.2	14.1	25.9
Angola	Malanje	Caombo	4318	2000	38.4	29.4	49.9
Angola	Malanje	Caombo	4318	2005	30.0	23.1	38.1
Angola	Malanje	Caombo	4318	2010	23.3	17.6	30.2
Angola	Malanje	Caombo	4318	2015	18.7	13.7	25.0
Angola	Malanje	Kiwaba N'zogi	4319	2000	39.8	30.8	50.9
Angola	Malanje	Kiwaba N'zogi	4319	2005	31.6	24.6	39.7
Angola	Malanje	Kiwaba N'zogi	4319	2010	24.5	18.8	31.5
Angola	Malanje	Kiwaba N'zogi	4319	2015	19.4	14.4	25.8
Angola	Malanje	Kunda Dia-baze	4320	2000	37.2	27.8	49.6
Angola	Malanje	Kunda Dia-baze	4320	2005	29.0	21.4	39.0
Angola	Malanje	Kunda Dia-baze	4320	2010	22.3	16.5	30.0
Angola	Malanje	Kunda Dia-baze	4320	2015	17.9	12.9	24.5
Angola	Malanje	Luquembo	4321	2000	33.9	25.2	44.4
Angola	Malanje	Luquembo	4321	2005	27.8	20.6	36.8
Angola	Malanje	Luquembo	4321	2010	22.4	16.4	30.0
Angola	Malanje	Luquembo	4321	2015	16.9	11.9	22.4
Angola	Malanje	Malanje	4322	2000	39.2	29.8	50.3
Angola	Malanje	Malanje	4322	2005	31.6	24.2	40.3
Angola	Malanje	Malanje	4322	2010	25.2	19.1	33.0
Angola	Malanje	Malanje	4322	2015	19.4	14.3	26.5
Angola	Malanje	Marimba	4323	2000	36.1	27.2	47.7
Angola	Malanje	Marimba	4323	2005	28.1	21.1	36.6
Angola	Malanje	Marimba	4323	2010	21.9	16.3	28.8
Angola	Malanje	Marimba	4323	2015	17.7	12.9	24.0
Angola	Malanje	Massango	4324	2000	37.5	28.3	48.6
Angola	Malanje	Massango	4324	2005	28.7	21.9	36.9
Angola	Malanje	Massango	4324	2010	22.7	17.4	29.5
Angola	Malanje	Massango	4324	2015	18.4	13.7	24.5
Angola	Malanje	Mucari	4325	2000	39.6	29.9	51.4
Angola	Malanje	Mucari	4325	2005	31.8	24.5	41.0
Angola	Malanje	Mucari	4325	2010	24.1	18.0	31.8
Angola	Malanje	Mucari	4325	2015	18.6	13.6	25.1
Angola	Malanje	Quela	4326	2000	38.1	29.1	49.2
Angola	Malanje	Quela	4326	2005	30.3	22.9	39.3
Angola	Malanje	Quela	4326	2010	23.0	17.2	30.1
Angola	Malanje	Quela	4326	2015	18.0	13.1	24.0
Angola	Malanje	Quirima	4327	2000	33.2	24.2	44.4
Angola	Malanje	Quirima	4327	2005	27.6	20.0	36.5
Angola	Malanje	Quirima	4327	2010	22.2	16.1	30.2
Angola	Malanje	Quirima	4327	2015	17.0	12.2	22.8
Angola	Moxico	Alto Zambeze	4328	2000	36.0	28.9	44.0
Angola	Moxico	Alto Zambeze	4328	2005	31.1	25.4	37.4
Angola	Moxico	Alto Zambeze	4328	2010	24.2	19.3	29.6
Angola	Moxico	Alto Zambeze	4328	2015	21.0	16.5	26.4
Angola	Moxico	Camanongue	4329	2000	35.0	25.4	46.5
Angola	Moxico	Camanongue	4329	2005	29.2	21.4	38.6
Angola	Moxico	Camanongue	4329	2010	23.1	16.4	30.5
Angola	Moxico	Camanongue	4329	2015	18.4	12.5	25.0
Angola	Moxico	Kameia Lumege	4330	2000	36.8	27.0	49.1
Angola	Moxico	Kameia Lumege	4330	2005	31.0	22.3	41.4
Angola	Moxico	Kameia Lumege	4330	2010	24.5	17.4	33.0
Angola	Moxico	Kameia Lumege	4330	2015	20.3	13.9	27.3
Angola	Moxico	Leua	4331	2000	36.1	25.7	48.7
Angola	Moxico	Leua	4331	2005	29.7	21.3	40.2
Angola	Moxico	Leua	4331	2010	23.5	16.5	31.7
Angola	Moxico	Leua	4331	2015	19.1	13.1	26.5
Angola	Moxico	Luacano	4332	2000	38.1	28.8	50.2
Angola	Moxico	Luacano	4332	2005	33.0	25.1	42.2
Angola	Moxico	Luacano	4332	2010	25.8	19.3	33.7

Admin 0	Admin 1	Admin 2	GAUL Code	Year	Neonatal mortality (per 1,000 livebirths)		
					Estimate	Lower bound, 95% UI	Upper bound, 95% UI
Angola	Moxico	Luacano	4332	2015	21.5	15.6	28.6
Angola	Moxico	Luau	4333	2000	45.8	33.9	60.1
Angola	Moxico	Luau	4333	2005	39.1	29.7	50.8
Angola	Moxico	Luau	4333	2010	29.5	22.0	38.9
Angola	Moxico	Luau	4333	2015	24.2	17.2	32.7
Angola	Moxico	Luchazes	4334	2000	33.5	24.9	44.3
Angola	Moxico	Luchazes	4334	2005	28.4	20.9	37.6
Angola	Moxico	Luchazes	4334	2010	22.8	16.4	30.1
Angola	Moxico	Luchazes	4334	2015	18.7	13.3	25.2
Angola	Moxico	Luena (moxico)	4335	2000	34.1	25.6	44.2
Angola	Moxico	Luena (moxico)	4335	2005	28.3	21.0	36.8
Angola	Moxico	Luena (moxico)	4335	2010	22.7	16.5	29.9
Angola	Moxico	Luena (moxico)	4335	2015	18.0	13.1	24.6
Angola	Moxico	Lumbala N'guimbo	4336	2000	38.3	29.2	48.4
Angola	Moxico	Lumbala N'guimbo	4336	2005	32.3	24.6	40.9
Angola	Moxico	Lumbala N'guimbo	4336	2010	26.1	19.7	33.3
Angola	Moxico	Lumbala N'guimbo	4336	2015	23.0	16.9	30.0
Angola	Namibe	Bibala	4337	2000	39.2	30.6	48.5
Angola	Namibe	Bibala	4337	2005	33.6	26.7	42.0
Angola	Namibe	Bibala	4337	2010	29.0	22.9	36.1
Angola	Namibe	Bibala	4337	2015	21.2	16.1	27.2
Angola	Namibe	Camacuio	4338	2000	41.0	31.0	52.7
Angola	Namibe	Camacuio	4338	2005	35.5	27.0	44.7
Angola	Namibe	Camacuio	4338	2010	30.8	23.3	39.6
Angola	Namibe	Camacuio	4338	2015	22.5	17.0	30.2
Angola	Namibe	Namibe	4339	2000	34.4	25.2	44.0
Angola	Namibe	Namibe	4339	2005	32.2	24.4	41.8
Angola	Namibe	Namibe	4339	2010	28.0	21.2	36.6
Angola	Namibe	Namibe	4339	2015	21.6	16.0	29.1
Angola	Namibe	Tombua	4340	2000	32.9	24.0	43.5
Angola	Namibe	Tombua	4340	2005	29.4	21.9	38.2
Angola	Namibe	Tombua	4340	2010	25.2	18.3	33.5
Angola	Namibe	Tombua	4340	2015	19.6	14.2	26.7
Angola	Namibe	Virei	4341	2000	35.2	26.7	44.4
Angola	Namibe	Virei	4341	2005	31.1	24.2	39.0
Angola	Namibe	Virei	4341	2010	26.3	20.1	33.7
Angola	Namibe	Virei	4341	2015	20.1	15.0	26.5
Angola	Uige	Alto Cauale	4342	2000	42.3	32.4	54.7
Angola	Uige	Alto Cauale	4342	2005	30.9	24.1	39.1
Angola	Uige	Alto Cauale	4342	2010	24.8	19.2	31.6
Angola	Uige	Alto Cauale	4342	2015	19.2	14.5	25.7
Angola	Uige	Ambuila	4343	2000	44.6	33.9	56.1
Angola	Uige	Ambuila	4343	2005	34.1	26.6	42.3
Angola	Uige	Ambuila	4343	2010	27.4	20.9	34.2
Angola	Uige	Ambuila	4343	2015	21.1	15.8	27.3
Angola	Uige	Bembe	4344	2000	46.6	35.3	59.3
Angola	Uige	Bembe	4344	2005	36.2	27.5	45.6
Angola	Uige	Bembe	4344	2010	28.5	21.3	36.3
Angola	Uige	Bembe	4344	2015	21.7	16.2	28.7
Angola	Uige	Buengas	4345	2000	47.3	37.1	59.4
Angola	Uige	Buengas	4345	2005	34.3	27.0	43.0
Angola	Uige	Buengas	4345	2010	27.2	21.1	34.4
Angola	Uige	Buengas	4345	2015	20.7	15.6	27.1
Angola	Uige	Bungo	4346	2000	45.3	35.8	58.0
Angola	Uige	Bungo	4346	2005	32.5	25.5	41.0
Angola	Uige	Bungo	4346	2010	25.9	20.0	33.0
Angola	Uige	Bungo	4346	2015	20.2	15.0	26.7
Angola	Uige	Damba	4347	2000	48.3	37.9	59.9
Angola	Uige	Damba	4347	2005	35.8	28.4	43.9
Angola	Uige	Damba	4347	2010	28.0	21.7	34.7
Angola	Uige	Damba	4347	2015	21.2	16.1	27.6
Angola	Uige	Maquela Do Zombo	4348	2000	47.6	38.6	57.1
Angola	Uige	Maquela Do Zombo	4348	2005	36.7	29.7	43.9
Angola	Uige	Maquela Do Zombo	4348	2010	28.0	22.5	34.1
Angola	Uige	Maquela Do Zombo	4348	2015	20.9	16.2	26.6
Angola	Uige	Mucaba	4349	2000	46.9	36.4	60.1
Angola	Uige	Mucaba	4349	2005	33.8	26.4	42.5
Angola	Uige	Mucaba	4349	2010	26.8	20.6	34.6
Angola	Uige	Mucaba	4349	2015	20.3	15.0	27.2
Angola	Uige	Negage	4350	2000	43.7	35.0	55.3
Angola	Uige	Negage	4350	2005	32.2	25.8	40.2
Angola	Uige	Negage	4350	2010	25.7	20.0	32.2
Angola	Uige	Negage	4350	2015	19.6	14.8	25.4
Angola	Uige	Puri	4351	2000	43.4	34.2	55.7
Angola	Uige	Puri	4351	2005	31.6	24.8	39.9
Angola	Uige	Puri	4351	2010	25.0	19.5	31.7
Angola	Uige	Puri	4351	2015	19.6	14.7	25.9
Angola	Uige	Quimbele	4352	2000	43.9	35.1	53.1
Angola	Uige	Quimbele	4352	2005	33.3	26.2	40.8
Angola	Uige	Quimbele	4352	2010	25.6	20.0	32.3
Angola	Uige	Quimbele	4352	2015	19.9	15.2	25.9
Angola	Uige	Quitexe	4353	2000	44.1	35.2	54.8
Angola	Uige	Quitexe	4353	2005	33.5	27.2	41.1
Angola	Uige	Quitexe	4353	2010	26.9	21.1	33.9
Angola	Uige	Quitexe	4353	2015	20.6	15.7	26.4

Admin 0	Admin 1	Admin 2	GAUL Code	Year	Neonatal mortality (per 1,000 livebirths)		
					Estimate	Lower bound, 95% UI	Upper bound, 95% UI
Angola	Uige	Santa Cruz	4354	2000	39.9	30.7	50.3
Angola	Uige	Santa Cruz	4354	2005	29.9	23.0	38.0
Angola	Uige	Santa Cruz	4354	2010	23.5	18.0	30.0
Angola	Uige	Santa Cruz	4354	2015	18.7	13.9	24.8
Angola	Uige	Sanza Pombo	4355	2000	43.2	33.5	55.0
Angola	Uige	Sanza Pombo	4355	2005	31.5	24.5	40.1
Angola	Uige	Sanza Pombo	4355	2010	24.9	19.2	31.8
Angola	Uige	Sanza Pombo	4355	2015	20.0	15.0	26.3
Angola	Uige	Songo	4356	2000	47.1	36.1	59.3
Angola	Uige	Songo	4356	2005	35.2	27.4	44.0
Angola	Uige	Songo	4356	2010	27.5	21.0	35.3
Angola	Uige	Songo	4356	2015	21.2	15.6	28.4
Angola	Uige	Songo/mucaba	4357	2000	48.5	37.3	61.8
Angola	Uige	Songo/mucaba	4357	2005	35.6	27.8	44.8
Angola	Uige	Songo/mucaba	4357	2010	27.7	20.8	35.9
Angola	Uige	Songo/mucaba	4357	2015	21.7	15.9	29.2
Angola	Uige	Uige	4358	2000	43.9	33.8	56.1
Angola	Uige	Uige	4358	2005	32.4	25.3	40.5
Angola	Uige	Uige	4358	2010	26.1	20.0	33.3
Angola	Uige	Uige	4358	2015	19.8	14.6	26.0
Angola	Zaire	Cuimba	4359	2000	47.2	36.6	59.6
Angola	Zaire	Cuimba	4359	2005	38.1	29.5	48.1
Angola	Zaire	Cuimba	4359	2010	29.0	22.1	37.0
Angola	Zaire	Cuimba	4359	2015	21.4	15.8	28.4
Angola	Zaire	M'banza Congo	4360	2000	45.1	34.5	56.5
Angola	Zaire	M'banza Congo	4360	2005	37.7	29.1	47.0
Angola	Zaire	M'banza Congo	4360	2010	28.7	22.1	36.4
Angola	Zaire	M'banza Congo	4360	2015	21.7	16.2	28.3
Angola	Zaire	N'zeto	4361	2000	41.0	30.8	52.6
Angola	Zaire	N'zeto	4361	2005	34.2	25.5	44.4
Angola	Zaire	N'zeto	4361	2010	28.3	21.3	36.9
Angola	Zaire	N'zeto	4361	2015	21.6	16.0	29.2
Angola	Zaire	Noqui	4362	2000	40.4	32.2	51.0
Angola	Zaire	Noqui	4362	2005	35.1	27.8	43.9
Angola	Zaire	Noqui	4362	2010	28.2	22.3	35.5
Angola	Zaire	Noqui	4362	2015	21.4	16.2	28.0
Angola	Zaire	Soyo	4363	2000	36.6	28.3	46.7
Angola	Zaire	Soyo	4363	2005	31.3	24.1	39.9
Angola	Zaire	Soyo	4363	2010	26.8	20.6	34.2
Angola	Zaire	Soyo	4363	2015	20.1	15.0	26.5
Angola	Zaire	Tomboco	4364	2000	41.6	31.7	53.2
Angola	Zaire	Tomboco	4364	2005	35.2	27.1	44.8
Angola	Zaire	Tomboco	4364	2010	28.4	21.4	36.5
Angola	Zaire	Tomboco	4364	2015	21.8	16.1	28.8
Benin	Alibori	Banikoara	5861	2000	38.4	32.9	44.1
Benin	Alibori	Banikoara	5861	2005	35.1	30.0	40.4
Benin	Alibori	Banikoara	5861	2010	28.6	23.8	33.7
Benin	Alibori	Banikoara	5861	2015	25.7	20.5	31.8
Benin	Alibori	Gogounou	5862	2000	35.1	30.6	40.0
Benin	Alibori	Gogounou	5862	2005	31.5	27.1	36.0
Benin	Alibori	Gogounou	5862	2010	26.4	22.2	31.1
Benin	Alibori	Gogounou	5862	2015	22.5	18.1	27.6
Benin	Alibori	Kandi	5863	2000	38.0	33.0	43.7
Benin	Alibori	Kandi	5863	2005	35.2	30.3	40.6
Benin	Alibori	Kandi	5863	2010	30.0	25.1	35.6
Benin	Alibori	Kandi	5863	2015	26.1	20.7	32.2
Benin	Alibori	Karimama	5864	2000	45.1	39.0	51.5
Benin	Alibori	Karimama	5864	2005	41.8	36.0	47.9
Benin	Alibori	Karimama	5864	2010	36.1	30.6	42.3
Benin	Alibori	Karimama	5864	2015	30.3	24.6	37.1
Benin	Alibori	Malanville	5865	2000	45.4	39.8	52.1
Benin	Alibori	Malanville	5865	2005	42.9	37.5	49.0
Benin	Alibori	Malanville	5865	2010	39.0	33.4	45.8
Benin	Alibori	Malanville	5865	2015	33.5	27.2	41.5
Benin	Alibori	Segbana	5866	2000	40.5	34.5	47.0
Benin	Alibori	Segbana	5866	2005	38.0	32.3	44.2
Benin	Alibori	Segbana	5866	2010	33.4	28.0	39.8
Benin	Alibori	Segbana	5866	2015	27.6	22.0	34.0
Benin	Atakora	Boukoumbe	5867	2000	33.1	29.1	37.3
Benin	Atakora	Boukoumbe	5867	2005	31.6	27.8	35.6
Benin	Atakora	Boukoumbe	5867	2010	25.6	22.3	29.5
Benin	Atakora	Boukoumbe	5867	2015	23.4	19.4	28.5
Benin	Atakora	Kerou	5868	2000	35.3	30.5	40.5
Benin	Atakora	Kerou	5868	2005	32.1	27.9	37.0
Benin	Atakora	Kerou	5868	2010	26.4	22.1	31.2
Benin	Atakora	Kerou	5868	2015	23.1	18.6	28.7
Benin	Atakora	Kobli	5869	2000	33.3	29.3	37.6
Benin	Atakora	Kobli	5869	2005	31.8	27.7	36.1
Benin	Atakora	Kobli	5869	2010	25.7	22.3	29.7
Benin	Atakora	Kobli	5869	2015	23.7	19.5	29.0
Benin	Atakora	Kouande	5870	2000	34.5	30.0	39.1
Benin	Atakora	Kouande	5870	2005	31.9	28.0	36.4
Benin	Atakora	Kouande	5870	2010	25.9	22.1	30.3
Benin	Atakora	Kouande	5870	2015	22.8	18.4	27.9
Benin	Atakora	Materi	5871	2000	35.1	30.4	39.7

Admin 0	Admin 1	Admin 2	GAUL Code	Year	Neonatal mortality (per 1,000 livebirths)		
					Estimate	Lower bound, 95% UI	Upper bound, 95% UI
Benin	Atakora	Materi	5871	2005	34.0	29.5	38.9
Benin	Atakora	Materi	5871	2010	27.3	23.6	31.6
Benin	Atakora	Materi	5871	2015	24.8	20.2	30.4
Benin	Atakora	Natitingou	5872	2000	32.1	28.1	36.5
Benin	Atakora	Natitingou	5872	2005	30.5	26.8	34.7
Benin	Atakora	Natitingou	5872	2010	24.9	21.5	28.9
Benin	Atakora	Natitingou	5872	2015	22.4	18.1	27.5
Benin	Atakora	Pehonko	5873	2000	34.5	29.4	39.9
Benin	Atakora	Pehonko	5873	2005	31.0	26.7	35.7
Benin	Atakora	Pehonko	5873	2010	25.4	21.3	30.1
Benin	Atakora	Pehonko	5873	2015	22.3	17.8	27.6
Benin	Atakora	Tanguieta	5874	2000	36.1	31.6	41.0
Benin	Atakora	Tanguieta	5874	2005	34.2	30.2	38.9
Benin	Atakora	Tanguieta	5874	2010	27.7	23.8	32.0
Benin	Atakora	Tanguieta	5874	2015	24.6	20.1	29.9
Benin	Atakora	Toukountouna	5875	2000	33.9	29.3	38.8
Benin	Atakora	Toukountouna	5875	2005	32.1	27.9	36.7
Benin	Atakora	Toukountouna	5875	2010	25.9	22.0	30.1
Benin	Atakora	Toukountouna	5875	2015	23.0	18.7	28.2
Benin	Atlantique	Abomey-calavi	5876	2000	33.9	30.6	37.5
Benin	Atlantique	Abomey-calavi	5876	2005	31.2	28.2	34.5
Benin	Atlantique	Abomey-calavi	5876	2010	30.1	26.4	34.1
Benin	Atlantique	Abomey-calavi	5876	2015	27.0	22.1	32.7
Benin	Atlantique	Allada	5877	2000	37.0	33.8	40.5
Benin	Atlantique	Allada	5877	2005	32.6	29.5	35.8
Benin	Atlantique	Allada	5877	2010	29.5	26.1	33.3
Benin	Atlantique	Allada	5877	2015	26.6	22.1	31.7
Benin	Atlantique	Kpomasse	5878	2000	35.6	31.9	39.6
Benin	Atlantique	Kpomasse	5878	2005	31.4	28.1	35.0
Benin	Atlantique	Kpomasse	5878	2010	29.1	25.6	32.9
Benin	Atlantique	Kpomasse	5878	2015	25.5	20.9	30.9
Benin	Atlantique	Ouidah	5879	2000	35.0	31.5	39.0
Benin	Atlantique	Ouidah	5879	2005	30.9	27.7	34.5
Benin	Atlantique	Ouidah	5879	2010	29.4	26.0	33.5
Benin	Atlantique	Ouidah	5879	2015	25.9	21.2	31.6
Benin	Atlantique	So-ava	5880	2000	37.1	33.8	40.5
Benin	Atlantique	So-ava	5880	2005	34.2	31.4	37.3
Benin	Atlantique	So-ava	5880	2010	31.9	28.3	35.8
Benin	Atlantique	So-ava	5880	2015	27.7	22.9	33.4
Benin	Atlantique	Toffo	5881	2000	38.5	35.2	42.1
Benin	Atlantique	Toffo	5881	2005	33.1	29.9	36.5
Benin	Atlantique	Toffo	5881	2010	30.4	26.7	34.2
Benin	Atlantique	Toffo	5881	2015	27.2	22.9	32.8
Benin	Atlantique	Tori-bossito	5882	2000	38.4	35.0	41.8
Benin	Atlantique	Tori-bossito	5882	2005	33.3	30.2	36.6
Benin	Atlantique	Tori-bossito	5882	2010	29.8	26.4	33.6
Benin	Atlantique	Tori-bossito	5882	2015	26.5	21.9	31.8
Benin	Atlantique	Ze	5883	2000	39.3	35.8	43.2
Benin	Atlantique	Ze	5883	2005	34.3	31.0	37.8
Benin	Atlantique	Ze	5883	2010	30.8	27.1	34.8
Benin	Atlantique	Ze	5883	2015	27.1	22.5	32.8
Benin	Borgou	Bembereke	5884	2000	34.3	29.5	39.8
Benin	Borgou	Bembereke	5884	2005	29.2	24.7	33.9
Benin	Borgou	Bembereke	5884	2010	25.1	21.0	29.7
Benin	Borgou	Bembereke	5884	2015	21.9	17.5	26.9
Benin	Borgou	Kalale	5885	2000	36.2	30.6	42.0
Benin	Borgou	Kalale	5885	2005	30.9	25.7	36.0
Benin	Borgou	Kalale	5885	2010	27.4	22.7	32.8
Benin	Borgou	Kalale	5885	2015	23.9	18.6	30.0
Benin	Borgou	Ndali	5886	2000	37.4	32.4	43.4
Benin	Borgou	Ndali	5886	2005	31.6	27.4	36.4
Benin	Borgou	Ndali	5886	2010	26.7	22.7	31.5
Benin	Borgou	Ndali	5886	2015	23.5	18.8	28.8
Benin	Borgou	Nikki	5887	2000	34.6	29.5	40.4
Benin	Borgou	Nikki	5887	2005	29.0	24.4	33.7
Benin	Borgou	Nikki	5887	2010	25.9	21.2	31.0
Benin	Borgou	Nikki	5887	2015	22.7	17.9	28.2
Benin	Borgou	Parakou	5888	2000	36.6	31.5	42.9
Benin	Borgou	Parakou	5888	2005	30.9	26.6	35.9
Benin	Borgou	Parakou	5888	2010	27.2	22.7	32.2
Benin	Borgou	Parakou	5888	2015	23.7	19.0	29.4
Benin	Borgou	Perere	5889	2000	36.1	31.0	42.4
Benin	Borgou	Perere	5889	2005	30.2	25.8	35.6
Benin	Borgou	Perere	5889	2010	26.3	22.0	31.3
Benin	Borgou	Perere	5889	2015	22.9	18.4	28.5
Benin	Borgou	Sinende	5890	2000	34.9	29.9	40.3
Benin	Borgou	Sinende	5890	2005	30.0	25.8	34.6
Benin	Borgou	Sinende	5890	2010	25.3	21.2	30.3
Benin	Borgou	Sinende	5890	2015	21.9	17.5	27.2
Benin	Borgou	Tchaourou	5891	2000	40.5	35.3	46.7
Benin	Borgou	Tchaourou	5891	2005	34.4	29.9	39.6
Benin	Borgou	Tchaourou	5891	2010	29.0	24.8	34.1
Benin	Borgou	Tchaourou	5891	2015	25.0	20.3	30.4
Benin	Collines	Bante	5892	2000	42.5	37.0	48.7
Benin	Collines	Bante	5892	2005	38.8	33.7	44.3

Admin 0	Admin 1	Admin 2	GAUL Code	Year	Neonatal mortality (per 1,000 livebirths)		
					Estimate	Lower bound, 95% UI	Upper bound, 95% UI
Benin	Collines	Bante	5892	2010	32.7	27.8	37.9
Benin	Collines	Bante	5892	2015	28.5	22.7	34.2
Benin	Collines	Dassa	5893	2000	42.7	37.9	48.8
Benin	Collines	Dassa	5893	2005	37.5	33.0	42.6
Benin	Collines	Dassa	5893	2010	32.9	28.4	37.8
Benin	Collines	Dassa	5893	2015	28.8	23.6	35.5
Benin	Collines	Glazoue	5894	2000	41.7	36.4	47.9
Benin	Collines	Glazoue	5894	2005	37.2	32.1	42.5
Benin	Collines	Glazoue	5894	2010	32.0	27.4	37.3
Benin	Collines	Glazoue	5894	2015	27.4	22.1	33.3
Benin	Collines	Ouesse	5895	2000	39.7	34.4	46.2
Benin	Collines	Ouesse	5895	2005	34.8	29.7	40.4
Benin	Collines	Ouesse	5895	2010	29.8	25.1	35.2
Benin	Collines	Ouesse	5895	2015	25.4	20.2	31.0
Benin	Collines	Savalou	5896	2000	43.2	37.8	49.4
Benin	Collines	Savalou	5896	2005	39.2	34.3	44.7
Benin	Collines	Savalou	5896	2010	34.1	29.2	39.1
Benin	Collines	Savalou	5896	2015	30.0	24.5	36.0
Benin	Collines	Save	5897	2000	39.3	33.8	45.9
Benin	Collines	Save	5897	2005	34.5	29.4	40.1
Benin	Collines	Save	5897	2010	30.6	25.8	35.9
Benin	Collines	Save	5897	2015	26.0	20.8	32.0
Benin	Couffo	Aplahoue	5898	2000	39.7	36.2	44.1
Benin	Couffo	Aplahoue	5898	2005	34.3	31.1	38.1
Benin	Couffo	Aplahoue	5898	2010	30.9	27.3	34.9
Benin	Couffo	Aplahoue	5898	2015	28.5	23.9	33.9
Benin	Couffo	Djakotome	5899	2000	37.4	34.0	41.1
Benin	Couffo	Djakotome	5899	2005	32.6	29.6	35.9
Benin	Couffo	Djakotome	5899	2010	29.1	25.7	32.5
Benin	Couffo	Djakotome	5899	2015	26.9	22.5	31.6
Benin	Couffo	Dogbo-tota	5900	2000	37.5	34.1	41.0
Benin	Couffo	Dogbo-tota	5900	2005	32.1	29.1	35.3
Benin	Couffo	Dogbo-tota	5900	2010	28.7	25.4	32.2
Benin	Couffo	Dogbo-tota	5900	2015	26.4	22.1	31.3
Benin	Couffo	Klouekanme	5901	2000	39.4	36.0	43.4
Benin	Couffo	Klouekanme	5901	2005	33.6	30.4	37.0
Benin	Couffo	Klouekanme	5901	2010	30.6	27.0	34.5
Benin	Couffo	Klouekanme	5901	2015	28.0	23.5	33.4
Benin	Couffo	Lalo	5902	2000	38.6	35.0	42.2
Benin	Couffo	Lalo	5902	2005	32.9	29.7	36.3
Benin	Couffo	Lalo	5902	2010	30.1	26.6	34.1
Benin	Couffo	Lalo	5902	2015	27.3	22.8	32.8
Benin	Couffo	Toviklin	5903	2000	38.3	34.8	42.0
Benin	Couffo	Toviklin	5903	2005	32.4	29.3	35.7
Benin	Couffo	Toviklin	5903	2010	29.5	26.1	33.2
Benin	Couffo	Toviklin	5903	2015	27.3	22.8	32.6
Benin	Donga	Bassila	5904	2000	43.0	37.2	49.5
Benin	Donga	Bassila	5904	2005	38.3	33.4	44.1
Benin	Donga	Bassila	5904	2010	31.6	27.0	36.8
Benin	Donga	Bassila	5904	2015	27.3	22.0	33.2
Benin	Donga	Djougou	5905	2000	40.5	35.5	46.0
Benin	Donga	Djougou	5905	2005	36.1	31.6	41.0
Benin	Donga	Djougou	5905	2010	29.8	25.5	34.7
Benin	Donga	Djougou	5905	2015	25.9	21.0	31.5
Benin	Donga	Kopargo	5906	2000	38.7	34.5	43.5
Benin	Donga	Kopargo	5906	2005	35.3	31.3	39.5
Benin	Donga	Kopargo	5906	2010	29.0	25.0	33.6
Benin	Donga	Kopargo	5906	2015	25.2	20.7	30.8
Benin	Donga	Ouake	5907	2000	39.6	34.9	44.4
Benin	Donga	Ouake	5907	2005	36.1	31.9	40.2
Benin	Donga	Ouake	5907	2010	29.7	25.9	34.1
Benin	Donga	Ouake	5907	2015	25.9	21.1	31.0
Benin	Littoral	Cotonou	5908	2000	31.3	28.0	34.9
Benin	Littoral	Cotonou	5908	2005	29.3	26.4	32.8
Benin	Littoral	Cotonou	5908	2010	28.9	25.3	32.9
Benin	Littoral	Cotonou	5908	2015	27.4	22.5	33.4
Benin	Mono	Athieme	5909	2000	36.1	32.3	40.1
Benin	Mono	Athieme	5909	2005	31.8	28.5	35.5
Benin	Mono	Athieme	5909	2010	28.1	24.7	31.7
Benin	Mono	Athieme	5909	2015	25.6	21.3	30.8
Benin	Mono	Bopa	5910	2000	37.2	33.9	40.6
Benin	Mono	Bopa	5910	2005	32.2	29.2	35.3
Benin	Mono	Bopa	5910	2010	29.2	25.9	32.7
Benin	Mono	Bopa	5910	2015	26.3	22.0	31.6
Benin	Mono	Come	5911	2000	33.9	29.7	38.8
Benin	Mono	Come	5911	2005	30.1	26.1	34.3
Benin	Mono	Come	5911	2010	28.1	24.0	32.4
Benin	Mono	Come	5911	2015	25.2	20.3	31.2
Benin	Mono	Grand-popo	5912	2000	35.5	31.2	40.4
Benin	Mono	Grand-popo	5912	2005	31.4	27.3	35.5
Benin	Mono	Grand-popo	5912	2010	28.4	24.3	32.8
Benin	Mono	Grand-popo	5912	2015	25.4	20.5	31.3
Benin	Mono	Houeyogbe	5913	2000	35.9	32.4	40.0
Benin	Mono	Houeyogbe	5913	2005	31.7	28.2	35.3
Benin	Mono	Houeyogbe	5913	2010	28.2	24.8	32.2

Admin 0	Admin 1	Admin 2	GAUL Code	Year	Neonatal mortality (per 1,000 livebirths)		
					Estimate	Lower bound, 95% UI	Upper bound, 95% UI
Benin	Mono	Houeyogbe	5913	2015	25.4	21.0	30.8
Benin	Mono	Lokossa	5914	2000	35.6	32.4	38.9
Benin	Mono	Lokossa	5914	2005	31.5	28.6	34.7
Benin	Mono	Lokossa	5914	2010	28.3	25.2	31.7
Benin	Mono	Lokossa	5914	2015	25.7	21.5	30.6
Benin	Oueme	Adjara	5915	2000	37.1	33.4	41.4
Benin	Oueme	Adjara	5915	2005	34.1	30.7	37.9
Benin	Oueme	Adjara	5915	2010	33.6	29.4	38.0
Benin	Oueme	Adjara	5915	2015	28.1	23.3	34.2
Benin	Oueme	Adjohoun	5916	2000	40.2	37.0	43.6
Benin	Oueme	Adjohoun	5916	2005	36.0	33.0	39.0
Benin	Oueme	Adjohoun	5916	2010	32.2	28.8	35.9
Benin	Oueme	Adjohoun	5916	2015	27.8	23.4	33.1
Benin	Oueme	Aguegue	5917	2000	38.8	35.4	42.8
Benin	Oueme	Aguegue	5917	2005	35.5	32.5	38.8
Benin	Oueme	Aguegue	5917	2010	32.9	29.1	37.1
Benin	Oueme	Aguegue	5917	2015	27.8	23.1	33.7
Benin	Oueme	Akpro-misserete	5918	2000	36.7	33.3	40.6
Benin	Oueme	Akpro-misserete	5918	2005	33.6	30.6	36.9
Benin	Oueme	Akpro-misserete	5918	2010	33.2	29.2	37.4
Benin	Oueme	Akpro-misserete	5918	2015	28.3	23.3	34.2
Benin	Oueme	Avrankou	5919	2000	40.1	36.3	44.3
Benin	Oueme	Avrankou	5919	2005	35.4	32.0	39.0
Benin	Oueme	Avrankou	5919	2010	33.7	29.6	37.9
Benin	Oueme	Avrankou	5919	2015	28.5	23.7	34.4
Benin	Oueme	Bonou	5920	2000	41.2	37.6	45.0
Benin	Oueme	Bonou	5920	2005	35.8	32.6	39.2
Benin	Oueme	Bonou	5920	2010	32.0	28.4	35.9
Benin	Oueme	Bonou	5920	2015	27.4	23.0	32.8
Benin	Oueme	Dangbo	5921	2000	39.1	35.8	42.6
Benin	Oueme	Dangbo	5921	2005	35.5	32.5	38.4
Benin	Oueme	Dangbo	5921	2010	32.2	28.6	36.0
Benin	Oueme	Dangbo	5921	2015	27.5	23.0	32.9
Benin	Oueme	Porto-novo	5922	2000	35.5	32.1	39.7
Benin	Oueme	Porto-novo	5922	2005	33.2	30.1	36.7
Benin	Oueme	Porto-novo	5922	2010	32.1	28.3	36.4
Benin	Oueme	Porto-novo	5922	2015	28.2	23.5	34.4
Benin	Oueme	Seme-kpodji	5923	2000	37.4	33.8	41.5
Benin	Oueme	Seme-kpodji	5923	2005	34.5	31.3	38.1
Benin	Oueme	Seme-kpodji	5923	2010	33.8	29.9	38.2
Benin	Oueme	Seme-kpodji	5923	2015	28.3	23.4	34.2
Benin	Plateau	Adja-ouere	5924	2000	42.9	38.6	47.6
Benin	Plateau	Adja-ouere	5924	2005	37.5	33.6	41.5
Benin	Plateau	Adja-ouere	5924	2010	33.0	29.0	37.3
Benin	Plateau	Adja-ouere	5924	2015	28.0	23.4	33.5
Benin	Plateau	Ifangni	5925	2000	40.5	36.9	44.6
Benin	Plateau	Ifangni	5925	2005	36.1	32.8	39.5
Benin	Plateau	Ifangni	5925	2010	34.3	30.5	38.3
Benin	Plateau	Ifangni	5925	2015	29.2	24.5	34.8
Benin	Plateau	Ketou	5926	2000	44.1	39.3	49.4
Benin	Plateau	Ketou	5926	2005	38.4	33.8	43.1
Benin	Plateau	Ketou	5926	2010	32.8	28.5	37.6
Benin	Plateau	Ketou	5926	2015	28.3	23.4	34.4
Benin	Plateau	Pobe	5927	2000	43.6	38.7	49.0
Benin	Plateau	Pobe	5927	2005	37.9	33.5	42.8
Benin	Plateau	Pobe	5927	2010	33.4	29.0	38.1
Benin	Plateau	Pobe	5927	2015	28.3	23.4	34.4
Benin	Plateau	Sakete	5928	2000	42.0	38.5	45.9
Benin	Plateau	Sakete	5928	2005	37.4	34.1	40.7
Benin	Plateau	Sakete	5928	2010	33.6	29.9	37.6
Benin	Plateau	Sakete	5928	2015	28.7	24.2	34.0
Benin	Zou	Abomey	5929	2000	37.8	34.1	41.8
Benin	Zou	Abomey	5929	2005	33.5	30.1	37.2
Benin	Zou	Abomey	5929	2010	31.3	27.5	35.3
Benin	Zou	Abomey	5929	2015	28.2	23.5	33.9
Benin	Zou	Agbangnizoun	5930	2000	38.9	35.4	42.8
Benin	Zou	Agbangnizoun	5930	2005	33.7	30.5	37.1
Benin	Zou	Agbangnizoun	5930	2010	31.3	27.7	35.6
Benin	Zou	Agbangnizoun	5930	2015	28.7	23.9	34.3
Benin	Zou	Bohicon	5931	2000	37.7	33.9	41.9
Benin	Zou	Bohicon	5931	2005	33.3	29.8	37.0
Benin	Zou	Bohicon	5931	2010	31.5	27.5	35.8
Benin	Zou	Bohicon	5931	2015	28.3	23.4	34.1
Benin	Zou	Cove	5932	2000	42.1	38.1	46.7
Benin	Zou	Cove	5932	2005	35.6	31.9	39.4
Benin	Zou	Cove	5932	2010	32.2	28.0	36.6
Benin	Zou	Cove	5932	2015	28.0	23.2	34.1
Benin	Zou	Djidja	5933	2000	43.2	39.2	47.6
Benin	Zou	Djidja	5933	2005	37.3	33.6	41.2
Benin	Zou	Djidja	5933	2010	33.5	29.4	37.9
Benin	Zou	Djidja	5933	2015	30.1	24.9	36.3
Benin	Zou	Ouinhi	5934	2000	42.7	38.7	47.1
Benin	Zou	Ouinhi	5934	2005	36.6	32.8	40.5
Benin	Zou	Ouinhi	5934	2010	32.4	28.6	36.7
Benin	Zou	Ouinhi	5934	2015	27.6	23.0	33.1

Admin 0	Admin 1	Admin 2	GAUL Code	Year	Neonatal mortality (per 1,000 livebirths)		
					Estimate	Lower bound, 95% UI	Upper bound, 95% UI
Benin	Zou	Za-kpota	5935	2000	41.9	38.0	46.2
Benin	Zou	Za-kpota	5935	2005	35.6	31.9	39.3
Benin	Zou	Za-kpota	5935	2010	32.4	28.3	36.7
Benin	Zou	Za-kpota	5935	2015	28.6	23.7	34.6
Benin	Zou	Zangnanado	5936	2000	42.7	38.4	47.4
Benin	Zou	Zangnanado	5936	2005	36.3	32.3	40.3
Benin	Zou	Zangnanado	5936	2010	32.4	28.1	37.1
Benin	Zou	Zangnanado	5936	2015	27.9	23.0	33.7
Benin	Zou	Zogbodome	5937	2000	40.7	37.5	44.2
Benin	Zou	Zogbodome	5937	2005	34.4	31.4	37.6
Benin	Zou	Zogbodome	5937	2010	31.4	27.9	35.3
Benin	Zou	Zogbodome	5937	2015	27.4	23.1	32.5
Botswana	Central	Central	6322	2000	18.0	15.4	21.1
Botswana	Central	Central	6322	2005	16.4	14.1	19.3
Botswana	Central	Central	6322	2010	12.0	10.1	14.1
Botswana	Central	Central	6322	2015	7.8	6.3	9.5
Botswana	Chobe	Chobe	6323	2000	22.1	17.7	26.8
Botswana	Chobe	Chobe	6323	2005	19.5	15.8	24.1
Botswana	Chobe	Chobe	6323	2010	13.2	10.5	16.4
Botswana	Chobe	Chobe	6323	2015	8.7	6.7	11.2
Botswana	Ghanzi	Ghanzi	6324	2000	22.7	17.3	28.8
Botswana	Ghanzi	Ghanzi	6324	2005	19.8	15.1	24.9
Botswana	Ghanzi	Ghanzi	6324	2010	13.1	10.1	16.6
Botswana	Ghanzi	Ghanzi	6324	2015	8.6	6.5	11.1
Botswana	Kgalagadi	Kgalagadi	6325	2000	19.5	15.2	24.6
Botswana	Kgalagadi	Kgalagadi	6325	2005	17.3	13.4	21.8
Botswana	Kgalagadi	Kgalagadi	6325	2010	11.9	9.1	15.2
Botswana	Kgalagadi	Kgalagadi	6325	2015	8.0	6.0	10.4
Botswana	Kgatleng	Kgatleng	6326	2000	18.5	15.3	22.3
Botswana	Kgatleng	Kgatleng	6326	2005	16.6	13.4	20.3
Botswana	Kgatleng	Kgatleng	6326	2010	12.0	9.5	15.1
Botswana	Kgatleng	Kgatleng	6326	2015	7.8	6.0	10.0
Botswana	Kweneng	Kweneng	6327	2000	19.5	16.0	23.3
Botswana	Kweneng	Kweneng	6327	2005	17.4	14.3	21.1
Botswana	Kweneng	Kweneng	6327	2010	12.3	9.8	15.2
Botswana	Kweneng	Kweneng	6327	2015	8.0	6.2	10.1
Botswana	Ngamiland	Ngamiland	6328	2000	25.5	20.3	31.6
Botswana	Ngamiland	Ngamiland	6328	2005	22.1	17.7	27.4
Botswana	Ngamiland	Ngamiland	6328	2010	14.9	11.8	18.6
Botswana	Ngamiland	Ngamiland	6328	2015	9.6	7.3	12.3
Botswana	North East	North East	6329	2000	15.1	12.2	18.4
Botswana	North East	North East	6329	2005	14.0	11.5	17.0
Botswana	North East	North East	6329	2010	10.6	8.5	12.9
Botswana	North East	North East	6329	2015	7.1	5.4	8.9
Botswana	South-East	South-East	6330	2000	17.7	14.7	21.0
Botswana	South-East	South-East	6330	2005	16.2	13.3	19.4
Botswana	South-East	South-East	6330	2010	11.6	9.3	14.3
Botswana	South-East	South-East	6330	2015	7.7	5.9	9.8
Botswana	Southern	Southern	6331	2000	18.9	15.9	22.4
Botswana	Southern	Southern	6331	2005	16.9	14.1	20.2
Botswana	Southern	Southern	6331	2010	12.0	9.7	14.6
Botswana	Southern	Southern	6331	2015	7.8	6.2	9.8
Burkina Faso	Boucle Du Mouhoun	Bale	154444	2000	39.3	35.0	43.7
Burkina Faso	Boucle Du Mouhoun	Bale	154444	2005	35.6	31.7	40.1
Burkina Faso	Boucle Du Mouhoun	Bale	154444	2010	31.8	27.8	36.4
Burkina Faso	Boucle Du Mouhoun	Bale	154444	2015	29.5	24.6	34.9
Burkina Faso	Boucle Du Mouhoun	Banwa	154442	2000	41.8	37.1	46.8
Burkina Faso	Boucle Du Mouhoun	Banwa	154442	2005	37.0	32.7	41.6
Burkina Faso	Boucle Du Mouhoun	Banwa	154442	2010	32.7	28.4	37.5
Burkina Faso	Boucle Du Mouhoun	Banwa	154442	2015	29.4	24.4	34.8
Burkina Faso	Boucle Du Mouhoun	Kossi	154443	2000	42.2	37.5	47.5
Burkina Faso	Boucle Du Mouhoun	Kossi	154443	2005	37.2	32.7	41.6
Burkina Faso	Boucle Du Mouhoun	Kossi	154443	2010	32.7	28.6	37.3
Burkina Faso	Boucle Du Mouhoun	Kossi	154443	2015	29.2	24.4	34.5
Burkina Faso	Boucle Du Mouhoun	Mouhoun	154445	2000	38.9	34.7	43.5
Burkina Faso	Boucle Du Mouhoun	Mouhoun	154445	2005	34.9	30.9	39.1
Burkina Faso	Boucle Du Mouhoun	Mouhoun	154445	2010	31.2	27.1	35.4
Burkina Faso	Boucle Du Mouhoun	Mouhoun	154445	2015	28.8	24.3	33.9
Burkina Faso	Boucle Du Mouhoun	Nayala	154446	2000	38.5	33.8	43.5
Burkina Faso	Boucle Du Mouhoun	Nayala	154446	2005	34.3	30.1	39.0
Burkina Faso	Boucle Du Mouhoun	Nayala	154446	2010	30.4	26.1	35.3
Burkina Faso	Boucle Du Mouhoun	Nayala	154446	2015	28.5	23.4	34.2
Burkina Faso	Boucle Du Mouhoun	Sourou	154447	2000	40.6	36.1	45.3
Burkina Faso	Boucle Du Mouhoun	Sourou	154447	2005	35.9	32.0	40.2
Burkina Faso	Boucle Du Mouhoun	Sourou	154447	2010	31.3	27.2	35.8
Burkina Faso	Boucle Du Mouhoun	Sourou	154447	2015	28.7	23.9	34.2
Burkina Faso	Cascades	Comoe	154448	2000	47.7	43.3	53.1
Burkina Faso	Cascades	Comoe	154448	2005	44.7	40.2	49.4
Burkina Faso	Cascades	Comoe	154448	2010	39.5	34.7	44.6
Burkina Faso	Cascades	Comoe	154448	2015	34.1	28.6	40.7
Burkina Faso	Cascades	Leraba	154449	2000	55.2	49.3	61.6
Burkina Faso	Cascades	Leraba	154449	2005	49.6	44.0	55.3
Burkina Faso	Cascades	Leraba	154449	2010	43.7	37.9	50.0
Burkina Faso	Cascades	Leraba	154449	2015	36.9	30.2	44.4
Burkina Faso	Centre	Kadiogo	154450	2000	32.0	28.5	35.7

Admin 0	Admin 1	Admin 2	GAUL Code	Year	Neonatal mortality (per 1,000 livebirths)		
					Estimate	Lower bound, 95% UI	Upper bound, 95% UI
Burkina Faso	Centre	Kadiogo	154450	2005	29.1	26.1	32.6
Burkina Faso	Centre	Kadiogo	154450	2010	29.5	26.0	33.7
Burkina Faso	Centre	Kadiogo	154450	2015	25.4	21.1	30.1
Burkina Faso	Centre-est	Boulgou	154451	2000	36.4	32.9	40.0
Burkina Faso	Centre-est	Boulgou	154451	2005	32.9	29.9	36.1
Burkina Faso	Centre-est	Boulgou	154451	2010	29.4	26.1	33.0
Burkina Faso	Centre-est	Boulgou	154451	2015	26.2	22.2	30.6
Burkina Faso	Centre-est	Koulpelogo	154452	2000	38.7	34.5	43.2
Burkina Faso	Centre-est	Koulpelogo	154452	2005	35.0	31.0	39.5
Burkina Faso	Centre-est	Koulpelogo	154452	2010	30.8	26.9	34.9
Burkina Faso	Centre-est	Koulpelogo	154452	2015	28.1	23.3	33.9
Burkina Faso	Centre-est	Kouritenga	154453	2000	37.7	34.1	41.8
Burkina Faso	Centre-est	Kouritenga	154453	2005	34.7	31.1	38.5
Burkina Faso	Centre-est	Kouritenga	154453	2010	31.2	27.5	35.3
Burkina Faso	Centre-est	Kouritenga	154453	2015	28.7	23.8	33.9
Burkina Faso	Centre-nord	Bam	154454	2000	39.6	35.2	43.7
Burkina Faso	Centre-nord	Bam	154454	2005	34.9	31.1	38.8
Burkina Faso	Centre-nord	Bam	154454	2010	31.0	27.1	35.3
Burkina Faso	Centre-nord	Bam	154454	2015	30.0	25.1	35.7
Burkina Faso	Centre-nord	Namentenga	154455	2000	41.4	37.5	45.9
Burkina Faso	Centre-nord	Namentenga	154455	2005	37.8	34.3	41.6
Burkina Faso	Centre-nord	Namentenga	154455	2010	33.1	29.2	37.5
Burkina Faso	Centre-nord	Namentenga	154455	2015	31.4	26.3	37.0
Burkina Faso	Centre-nord	Sanmatenga	154456	2000	39.2	35.6	43.2
Burkina Faso	Centre-nord	Sanmatenga	154456	2005	35.2	32.1	38.8
Burkina Faso	Centre-nord	Sanmatenga	154456	2010	31.7	28.2	35.4
Burkina Faso	Centre-nord	Sanmatenga	154456	2015	29.9	25.3	35.4
Burkina Faso	Centre-ouest	Boulkiemde	154457	2000	38.0	34.5	42.0
Burkina Faso	Centre-ouest	Boulkiemde	154457	2005	34.4	31.0	37.9
Burkina Faso	Centre-ouest	Boulkiemde	154457	2010	31.1	27.4	35.1
Burkina Faso	Centre-ouest	Boulkiemde	154457	2015	29.0	24.5	34.4
Burkina Faso	Centre-ouest	Sanguie	154458	2000	38.3	34.5	42.2
Burkina Faso	Centre-ouest	Sanguie	154458	2005	34.8	31.3	38.4
Burkina Faso	Centre-ouest	Sanguie	154458	2010	30.9	27.1	35.1
Burkina Faso	Centre-ouest	Sanguie	154458	2015	29.0	24.4	34.3
Burkina Faso	Centre-ouest	Sissili	154459	2000	40.7	36.5	45.4
Burkina Faso	Centre-ouest	Sissili	154459	2005	37.7	33.9	42.2
Burkina Faso	Centre-ouest	Sissili	154459	2010	32.8	28.7	37.3
Burkina Faso	Centre-ouest	Sissili	154459	2015	29.9	24.8	35.3
Burkina Faso	Centre-ouest	Ziro	154460	2000	41.8	37.4	46.6
Burkina Faso	Centre-ouest	Ziro	154460	2005	38.0	33.8	42.5
Burkina Faso	Centre-ouest	Ziro	154460	2010	33.3	29.0	38.1
Burkina Faso	Centre-ouest	Ziro	154460	2015	30.4	25.2	35.9
Burkina Faso	Centre-sud	Bazega	154461	2000	38.9	35.2	42.7
Burkina Faso	Centre-sud	Bazega	154461	2005	34.7	31.5	38.3
Burkina Faso	Centre-sud	Bazega	154461	2010	32.2	28.4	36.5
Burkina Faso	Centre-sud	Bazega	154461	2015	28.3	23.9	33.1
Burkina Faso	Centre-sud	Nahouri	154462	2000	36.3	32.6	40.5
Burkina Faso	Centre-sud	Nahouri	154462	2005	33.4	29.7	37.3
Burkina Faso	Centre-sud	Nahouri	154462	2010	29.6	26.2	33.7
Burkina Faso	Centre-sud	Nahouri	154462	2015	26.2	22.2	31.1
Burkina Faso	Centre-sud	Zoundweogo	154463	2000	37.3	33.7	41.3
Burkina Faso	Centre-sud	Zoundweogo	154463	2005	33.6	30.4	37.3
Burkina Faso	Centre-sud	Zoundweogo	154463	2010	30.3	26.9	34.4
Burkina Faso	Centre-sud	Zoundweogo	154463	2015	26.5	22.6	31.3
Burkina Faso	Est	Gnagna	154464	2000	44.0	39.1	48.7
Burkina Faso	Est	Gnagna	154464	2005	39.6	35.6	44.2
Burkina Faso	Est	Gnagna	154464	2010	35.8	31.3	40.7
Burkina Faso	Est	Gnagna	154464	2015	32.7	27.3	38.8
Burkina Faso	Est	Gourma	154465	2000	45.8	40.5	51.2
Burkina Faso	Est	Gourma	154465	2005	41.7	37.0	46.8
Burkina Faso	Est	Gourma	154465	2010	37.5	32.7	43.1
Burkina Faso	Est	Gourma	154465	2015	32.9	27.2	39.1
Burkina Faso	Est	Komonjdjari	154466	2000	48.6	42.2	55.5
Burkina Faso	Est	Komonjdjari	154466	2005	43.1	37.5	49.5
Burkina Faso	Est	Komonjdjari	154466	2010	40.0	33.9	47.1
Burkina Faso	Est	Komonjdjari	154466	2015	34.3	28.1	41.6
Burkina Faso	Est	Kompienga	154467	2000	41.4	36.2	46.9
Burkina Faso	Est	Kompienga	154467	2005	37.1	32.4	42.3
Burkina Faso	Est	Kompienga	154467	2010	33.5	28.6	38.8
Burkina Faso	Est	Kompienga	154467	2015	28.9	23.6	35.0
Burkina Faso	Est	Tapoa	154468	2000	47.6	41.5	54.1
Burkina Faso	Est	Tapoa	154468	2005	41.5	36.2	47.6
Burkina Faso	Est	Tapoa	154468	2010	36.3	31.0	41.8
Burkina Faso	Est	Tapoa	154468	2015	33.0	26.7	40.2
Burkina Faso	Hauts-bassins	Houet	154469	2000	39.0	35.1	43.5
Burkina Faso	Hauts-bassins	Houet	154469	2005	37.1	33.5	41.5
Burkina Faso	Hauts-bassins	Houet	154469	2010	33.4	29.2	38.3
Burkina Faso	Hauts-bassins	Houet	154469	2015	30.0	24.9	35.5
Burkina Faso	Hauts-bassins	Kenedougou	154470	2000	47.6	42.7	52.9
Burkina Faso	Hauts-bassins	Kenedougou	154470	2005	43.8	39.4	48.3
Burkina Faso	Hauts-bassins	Kenedougou	154470	2010	37.7	33.1	42.6
Burkina Faso	Hauts-bassins	Kenedougou	154470	2015	33.1	27.7	39.1
Burkina Faso	Hauts-bassins	Tuy	154471	2000	40.2	35.6	44.9
Burkina Faso	Hauts-bassins	Tuy	154471	2005	38.1	33.8	42.9

Admin 0	Admin 1	Admin 2	GAUL Code	Year	Neonatal mortality (per 1,000 livebirths)		
					Estimate	Lower bound, 95% UI	Upper bound, 95% UI
Burkina Faso	Hauts-bassins	Tuy	154471	2010	32.8	28.5	38.0
Burkina Faso	Hauts-bassins	Tuy	154471	2015	30.4	25.2	35.8
Burkina Faso	Nord	Loroum	154472	2000	43.7	38.8	48.8
Burkina Faso	Nord	Loroum	154472	2005	38.3	33.9	42.8
Burkina Faso	Nord	Loroum	154472	2010	34.0	29.8	39.1
Burkina Faso	Nord	Loroum	154472	2015	31.4	26.0	37.5
Burkina Faso	Nord	Passore	154473	2000	38.6	34.7	42.4
Burkina Faso	Nord	Passore	154473	2005	34.0	30.5	37.5
Burkina Faso	Nord	Passore	154473	2010	30.6	26.8	34.7
Burkina Faso	Nord	Passore	154473	2015	28.9	24.1	34.4
Burkina Faso	Nord	Yatenga	154474	2000	41.5	37.2	46.0
Burkina Faso	Nord	Yatenga	154474	2005	36.2	32.6	40.3
Burkina Faso	Nord	Yatenga	154474	2010	32.3	28.3	36.9
Burkina Faso	Nord	Yatenga	154474	2015	30.2	25.2	36.0
Burkina Faso	Nord	Zondoma	154475	2000	40.0	35.6	44.5
Burkina Faso	Nord	Zondoma	154475	2005	35.0	31.1	39.3
Burkina Faso	Nord	Zondoma	154475	2010	31.1	27.1	36.0
Burkina Faso	Nord	Zondoma	154475	2015	28.9	23.8	34.9
Burkina Faso	Plateau Central	Ganzourgou	154476	2000	37.7	34.0	41.7
Burkina Faso	Plateau Central	Ganzourgou	154476	2005	33.7	30.3	37.4
Burkina Faso	Plateau Central	Ganzourgou	154476	2010	30.7	27.0	34.8
Burkina Faso	Plateau Central	Ganzourgou	154476	2015	28.0	23.5	32.7
Burkina Faso	Plateau Central	Kourweogo	154477	2000	38.1	34.2	42.4
Burkina Faso	Plateau Central	Kourweogo	154477	2005	33.8	30.5	37.4
Burkina Faso	Plateau Central	Kourweogo	154477	2010	31.0	27.3	34.9
Burkina Faso	Plateau Central	Kourweogo	154477	2015	29.3	24.7	34.4
Burkina Faso	Plateau Central	Oubritenga	154478	2000	37.5	34.1	41.1
Burkina Faso	Plateau Central	Oubritenga	154478	2005	33.2	30.2	36.5
Burkina Faso	Plateau Central	Oubritenga	154478	2010	31.1	27.6	35.1
Burkina Faso	Plateau Central	Oubritenga	154478	2015	28.6	24.3	33.6
Burkina Faso	Sahel	Oudalan	154479	2000	49.2	42.6	56.0
Burkina Faso	Sahel	Oudalan	154479	2005	42.9	37.1	48.7
Burkina Faso	Sahel	Oudalan	154479	2010	40.8	34.5	47.4
Burkina Faso	Sahel	Oudalan	154479	2015	37.0	30.0	45.2
Burkina Faso	Sahel	Seno	154480	2000	47.1	42.1	52.7
Burkina Faso	Sahel	Seno	154480	2005	41.9	37.2	47.0
Burkina Faso	Sahel	Seno	154480	2010	38.4	32.9	44.4
Burkina Faso	Sahel	Seno	154480	2015	35.7	29.6	43.2
Burkina Faso	Sahel	Soum	154481	2000	45.4	40.2	50.9
Burkina Faso	Sahel	Soum	154481	2005	40.1	35.5	44.6
Burkina Faso	Sahel	Soum	154481	2010	35.2	30.5	39.9
Burkina Faso	Sahel	Soum	154481	2015	33.3	27.7	39.9
Burkina Faso	Sahel	Yagha	154482	2000	45.2	39.5	51.8
Burkina Faso	Sahel	Yagha	154482	2005	40.5	35.2	46.2
Burkina Faso	Sahel	Yagha	154482	2010	37.5	31.7	43.9
Burkina Faso	Sahel	Yagha	154482	2015	33.3	27.0	40.5
Burkina Faso	Sud-ouest	Bougouriba	154483	2000	43.6	38.4	49.3
Burkina Faso	Sud-ouest	Bougouriba	154483	2005	40.4	35.6	46.0
Burkina Faso	Sud-ouest	Bougouriba	154483	2010	35.3	30.1	40.7
Burkina Faso	Sud-ouest	Bougouriba	154483	2015	31.5	26.0	38.0
Burkina Faso	Sud-ouest	Ioba	154484	2000	40.9	36.5	45.5
Burkina Faso	Sud-ouest	Ioba	154484	2005	37.6	34.0	42.0
Burkina Faso	Sud-ouest	Ioba	154484	2010	33.2	29.1	37.6
Burkina Faso	Sud-ouest	Ioba	154484	2015	30.4	25.4	36.2
Burkina Faso	Sud-ouest	Noumbiel	154485	2000	43.9	38.9	49.8
Burkina Faso	Sud-ouest	Noumbiel	154485	2005	42.7	37.7	48.5
Burkina Faso	Sud-ouest	Noumbiel	154485	2010	36.6	31.5	42.0
Burkina Faso	Sud-ouest	Noumbiel	154485	2015	31.4	25.5	37.9
Burkina Faso	Sud-ouest	Poni	154486	2000	44.9	40.3	50.4
Burkina Faso	Sud-ouest	Poni	154486	2005	42.8	38.3	47.5
Burkina Faso	Sud-ouest	Poni	154486	2010	36.8	32.0	41.9
Burkina Faso	Sud-ouest	Poni	154486	2015	32.2	26.6	38.6
Burundi	Bubanza	Bubanza	40559	2000	43.7	39.9	47.7
Burundi	Bubanza	Bubanza	40559	2005	36.9	33.7	40.3
Burundi	Bubanza	Bubanza	40559	2010	31.7	28.8	35.1
Burundi	Bubanza	Bubanza	40559	2015	28.5	24.6	33.0
Burundi	Bubanza	Gihanga	48356	2000	41.9	38.0	46.1
Burundi	Bubanza	Gihanga	48356	2005	35.5	32.3	38.9
Burundi	Bubanza	Gihanga	48356	2010	30.6	27.7	33.9
Burundi	Bubanza	Gihanga	48356	2015	28.6	24.7	33.1
Burundi	Bubanza	Mpanda	40561	2000	42.8	39.1	46.8
Burundi	Bubanza	Mpanda	40561	2005	36.3	33.1	39.7
Burundi	Bubanza	Mpanda	40561	2010	31.0	28.1	34.3
Burundi	Bubanza	Mpanda	40561	2015	27.9	24.2	32.3
Burundi	Bubanza	Musigati	40562	2000	44.5	40.7	48.7
Burundi	Bubanza	Musigati	40562	2005	37.5	34.2	40.8
Burundi	Bubanza	Musigati	40562	2010	31.9	28.9	35.3
Burundi	Bubanza	Musigati	40562	2015	28.5	24.6	32.9
Burundi	Bubanza	Rugazi	40563	2000	43.0	39.5	46.9
Burundi	Bubanza	Rugazi	40563	2005	36.6	33.8	39.8
Burundi	Bubanza	Rugazi	40563	2010	31.2	28.5	34.3
Burundi	Bubanza	Rugazi	40563	2015	27.6	24.0	31.9
Burundi	Bujumbura Mairie	Buterere	40565	2000	38.3	34.2	42.6
Burundi	Bujumbura Mairie	Buterere	40565	2005	34.7	31.1	38.5
Burundi	Bujumbura Mairie	Buterere	40565	2010	29.9	26.8	33.4

Admin 0	Admin 1	Admin 2	GAUL Code	Year	Neonatal mortality (per 1,000 livebirths)		
					Estimate	Lower bound, 95% UI	Upper bound, 95% UI
Burundi	Bujumbura Mairie	Buterere	40565	2015	28.8	24.5	33.6
Burundi	Bujumbura Mairie	Buyenzi	40566	2000	34.4	30.5	38.7
Burundi	Bujumbura Mairie	Buyenzi	40566	2005	32.8	29.2	36.9
Burundi	Bujumbura Mairie	Buyenzi	40566	2010	29.3	26.0	33.0
Burundi	Bujumbura Mairie	Buyenzi	40566	2015	28.7	24.3	33.7
Burundi	Bujumbura Mairie	Bwiza	40567	2000	34.4	30.5	38.7
Burundi	Bujumbura Mairie	Bwiza	40567	2005	32.8	29.2	36.9
Burundi	Bujumbura Mairie	Bwiza	40567	2010	29.3	26.0	33.0
Burundi	Bujumbura Mairie	Bwiza	40567	2015	28.7	24.3	33.7
Burundi	Bujumbura Mairie	Cibitoke	40568	2000	34.4	30.5	38.7
Burundi	Bujumbura Mairie	Cibitoke	40568	2005	32.8	29.2	36.9
Burundi	Bujumbura Mairie	Cibitoke	40568	2010	29.3	26.0	33.0
Burundi	Bujumbura Mairie	Cibitoke	40568	2015	28.7	24.3	33.7
Burundi	Bujumbura Mairie	Gihosha	40569	2000	35.6	31.7	40.0
Burundi	Bujumbura Mairie	Gihosha	40569	2005	33.4	29.8	37.5
Burundi	Bujumbura Mairie	Gihosha	40569	2010	29.2	26.0	32.9
Burundi	Bujumbura Mairie	Gihosha	40569	2015	28.5	24.1	33.4
Burundi	Bujumbura Mairie	Kamenge	40570	2000	35.6	31.7	40.0
Burundi	Bujumbura Mairie	Kamenge	40570	2005	33.4	29.8	37.5
Burundi	Bujumbura Mairie	Kamenge	40570	2010	29.2	26.0	32.9
Burundi	Bujumbura Mairie	Kamenge	40570	2015	28.5	24.1	33.4
Burundi	Bujumbura Mairie	Kanyosha	40571	2000	36.3	32.4	40.8
Burundi	Bujumbura Mairie	Kanyosha	40571	2005	33.6	30.0	37.8
Burundi	Bujumbura Mairie	Kanyosha	40571	2010	29.2	25.9	32.9
Burundi	Bujumbura Mairie	Kanyosha	40571	2015	28.6	24.2	33.5
Burundi	Bujumbura Mairie	Kinama	40572	2000	38.6	34.6	42.9
Burundi	Bujumbura Mairie	Kinama	40572	2005	35.1	31.5	38.9
Burundi	Bujumbura Mairie	Kinama	40572	2010	29.9	26.8	33.4
Burundi	Bujumbura Mairie	Kinama	40572	2015	28.5	24.4	33.2
Burundi	Bujumbura Mairie	Kinindo	40573	2000	34.3	30.3	38.7
Burundi	Bujumbura Mairie	Kinindo	40573	2005	32.9	29.3	37.0
Burundi	Bujumbura Mairie	Kinindo	40573	2010	29.1	25.8	32.9
Burundi	Bujumbura Mairie	Kinindo	40573	2015	29.3	24.7	34.4
Burundi	Bujumbura Mairie	Musaga	40574	2000	34.3	30.3	38.7
Burundi	Bujumbura Mairie	Musaga	40574	2005	32.9	29.3	37.0
Burundi	Bujumbura Mairie	Musaga	40574	2010	29.1	25.8	32.9
Burundi	Bujumbura Mairie	Musaga	40574	2015	29.3	24.7	34.4
Burundi	Bujumbura Mairie	Ngagara	40575	2000	34.4	30.5	38.7
Burundi	Bujumbura Mairie	Ngagara	40575	2005	32.8	29.2	36.9
Burundi	Bujumbura Mairie	Ngagara	40575	2010	29.3	26.0	33.0
Burundi	Bujumbura Mairie	Ngagara	40575	2015	28.7	24.3	33.7
Burundi	Bujumbura Mairie	Nyakabiga	40576	2000	36.1	32.2	40.4
Burundi	Bujumbura Mairie	Nyakabiga	40576	2005	33.3	29.8	37.4
Burundi	Bujumbura Mairie	Nyakabiga	40576	2010	29.1	25.9	32.9
Burundi	Bujumbura Mairie	Nyakabiga	40576	2015	28.3	23.9	33.1
Burundi	Bujumbura Mairie	Rohero	40577	2000	34.9	31.0	39.4
Burundi	Bujumbura Mairie	Rohero	40577	2005	33.1	29.5	37.1
Burundi	Bujumbura Mairie	Rohero	40577	2010	29.1	25.8	32.9
Burundi	Bujumbura Mairie	Rohero	40577	2015	28.9	24.4	34.0
Burundi	Bujumbura Rural	Bugarama	40580	2000	39.3	34.1	44.8
Burundi	Bujumbura Rural	Bugarama	40580	2005	35.1	30.9	40.1
Burundi	Bujumbura Rural	Bugarama	40580	2010	29.6	26.0	33.9
Burundi	Bujumbura Rural	Bugarama	40580	2015	27.2	22.7	32.2
Burundi	Bujumbura Rural	Isale	40581	2000	40.2	36.5	44.5
Burundi	Bujumbura Rural	Isale	40581	2005	35.8	32.3	39.6
Burundi	Bujumbura Rural	Isale	40581	2010	29.8	26.7	33.4
Burundi	Bujumbura Rural	Isale	40581	2015	27.2	23.3	31.7
Burundi	Bujumbura Rural	Kabezi	40582	2000	38.6	34.2	43.4
Burundi	Bujumbura Rural	Kabezi	40582	2005	34.8	31.0	39.1
Burundi	Bujumbura Rural	Kabezi	40582	2010	29.5	26.3	33.3
Burundi	Bujumbura Rural	Kabezi	40582	2015	27.4	23.2	32.1
Burundi	Bujumbura Rural	Kanyosha1	40753	2000	38.1	34.2	42.5
Burundi	Bujumbura Rural	Kanyosha1	40753	2005	34.9	31.3	39.0
Burundi	Bujumbura Rural	Kanyosha1	40753	2010	29.6	26.4	33.2
Burundi	Bujumbura Rural	Kanyosha1	40753	2015	27.6	23.4	32.2
Burundi	Bujumbura Rural	Mubimbi	40584	2000	41.5	37.7	45.6
Burundi	Bujumbura Rural	Mubimbi	40584	2005	36.4	33.2	39.9
Burundi	Bujumbura Rural	Mubimbi	40584	2010	30.4	27.5	33.7
Burundi	Bujumbura Rural	Mubimbi	40584	2015	27.3	23.5	31.6
Burundi	Bujumbura Rural	Mugongomanga	40754	2000	39.9	36.2	43.8
Burundi	Bujumbura Rural	Mugongomanga	40754	2005	35.5	32.3	39.1
Burundi	Bujumbura Rural	Mugongomanga	40754	2010	29.8	27.1	33.0
Burundi	Bujumbura Rural	Mugongomanga	40754	2015	27.2	23.5	31.6
Burundi	Bujumbura Rural	Muhuta	40586	2000	39.1	34.3	44.2
Burundi	Bujumbura Rural	Muhuta	40586	2005	35.0	31.1	39.6
Burundi	Bujumbura Rural	Muhuta	40586	2010	29.6	26.2	33.5
Burundi	Bujumbura Rural	Muhuta	40586	2015	27.2	22.9	32.1
Burundi	Bujumbura Rural	Mukike	40755	2000	39.6	35.6	43.9
Burundi	Bujumbura Rural	Mukike	40755	2005	35.4	31.9	39.3
Burundi	Bujumbura Rural	Mukike	40755	2010	29.8	26.8	33.2
Burundi	Bujumbura Rural	Mukike	40755	2015	27.1	23.2	31.5
Burundi	Bujumbura Rural	Mutambu	40756	2000	39.0	34.8	43.6
Burundi	Bujumbura Rural	Mutambu	40756	2005	34.9	31.3	39.0
Burundi	Bujumbura Rural	Mutambu	40756	2010	29.6	26.5	33.2
Burundi	Bujumbura Rural	Mutambu	40756	2015	27.3	23.2	31.8

Admin 0	Admin 1	Admin 2	GAUL Code	Year	Neonatal mortality (per 1,000 livebirths)		
					Estimate	Lower bound, 95% UI	Upper bound, 95% UI
Burundi	Bujumbura Rural	Mutimbuzi	40589	2000	39.7	35.6	44.1
Burundi	Bujumbura Rural	Mutimbuzi	40589	2005	35.4	31.9	39.2
Burundi	Bujumbura Rural	Mutimbuzi	40589	2010	30.1	27.1	33.7
Burundi	Bujumbura Rural	Mutimbuzi	40589	2015	28.7	24.6	33.5
Burundi	Bujumbura Rural	Nyabiraba	40757	2000	39.5	35.6	43.7
Burundi	Bujumbura Rural	Nyabiraba	40757	2005	35.2	31.8	39.2
Burundi	Bujumbura Rural	Nyabiraba	40757	2010	29.8	26.8	33.2
Burundi	Bujumbura Rural	Nyabiraba	40757	2015	27.1	23.2	31.6
Burundi	Bururi	Burambi	40591	2000	39.3	34.4	44.6
Burundi	Bururi	Burambi	40591	2005	34.9	30.9	39.5
Burundi	Bururi	Burambi	40591	2010	29.6	26.0	33.8
Burundi	Bururi	Burambi	40591	2015	26.6	22.3	31.5
Burundi	Bururi	Bururi	40592	2000	37.9	33.6	42.4
Burundi	Bururi	Bururi	40592	2005	34.2	30.7	38.2
Burundi	Bururi	Bururi	40592	2010	29.0	25.6	32.7
Burundi	Bururi	Bururi	40592	2015	25.7	21.6	30.2
Burundi	Bururi	Buyengero	40593	2000	39.0	34.6	43.8
Burundi	Bururi	Buyengero	40593	2005	34.7	30.9	38.8
Burundi	Bururi	Buyengero	40593	2010	29.5	26.2	33.5
Burundi	Bururi	Buyengero	40593	2015	26.1	22.0	30.6
Burundi	Bururi	Matana	40594	2000	39.7	35.6	44.0
Burundi	Bururi	Matana	40594	2005	35.1	31.8	38.8
Burundi	Bururi	Matana	40594	2010	30.2	27.0	33.6
Burundi	Bururi	Matana	40594	2015	26.3	22.4	30.8
Burundi	Bururi	Mugamba	40595	2000	39.8	35.7	44.1
Burundi	Bururi	Mugamba	40595	2005	35.4	32.0	39.3
Burundi	Bururi	Mugamba	40595	2010	29.8	26.6	33.2
Burundi	Bururi	Mugamba	40595	2015	26.6	22.7	31.0
Burundi	Bururi	Rumonge	40596	2000	37.1	32.7	41.8
Burundi	Bururi	Rumonge	40596	2005	33.5	29.6	37.7
Burundi	Bururi	Rumonge	40596	2010	28.0	24.6	32.0
Burundi	Bururi	Rumonge	40596	2015	26.0	21.8	30.8
Burundi	Bururi	Rutovu	40597	2000	38.9	34.7	43.3
Burundi	Bururi	Rutovu	40597	2005	35.1	31.6	38.9
Burundi	Bururi	Rutovu	40597	2010	29.6	26.4	33.3
Burundi	Bururi	Rutovu	40597	2015	26.2	22.3	30.7
Burundi	Bururi	Songa	40598	2000	39.0	34.3	43.7
Burundi	Bururi	Songa	40598	2005	34.8	31.1	38.8
Burundi	Bururi	Songa	40598	2010	29.7	26.3	33.6
Burundi	Bururi	Songa	40598	2015	25.9	21.7	30.4
Burundi	Bururi	Vyanda	40599	2000	37.4	32.9	42.2
Burundi	Bururi	Vyanda	40599	2005	33.5	29.8	37.6
Burundi	Bururi	Vyanda	40599	2010	28.4	24.7	32.1
Burundi	Bururi	Vyanda	40599	2015	25.0	20.9	29.7
Burundi	Cankuzo	Cankuzo	40600	2000	42.5	37.8	47.8
Burundi	Cankuzo	Cankuzo	40600	2005	36.7	32.5	40.9
Burundi	Cankuzo	Cankuzo	40600	2010	30.6	27.0	34.5
Burundi	Cankuzo	Cankuzo	40600	2015	28.6	24.1	33.5
Burundi	Cankuzo	Cendajuru	40601	2000	40.9	36.2	46.2
Burundi	Cankuzo	Cendajuru	40601	2005	35.6	31.6	39.7
Burundi	Cankuzo	Cendajuru	40601	2010	29.9	26.2	33.7
Burundi	Cankuzo	Cendajuru	40601	2015	28.1	23.5	33.3
Burundi	Cankuzo	Gisagara	40602	2000	40.0	35.4	45.0
Burundi	Cankuzo	Gisagara	40602	2005	34.5	30.6	38.5
Burundi	Cankuzo	Gisagara	40602	2010	29.4	25.7	33.4
Burundi	Cankuzo	Gisagara	40602	2015	27.6	23.1	32.7
Burundi	Cankuzo	Kigamba	40603	2000	43.0	38.4	48.2
Burundi	Cankuzo	Kigamba	40603	2005	36.4	32.4	40.6
Burundi	Cankuzo	Kigamba	40603	2010	30.6	27.1	34.0
Burundi	Cankuzo	Kigamba	40603	2015	28.7	24.3	33.4
Burundi	Cankuzo	Mishiha	40604	2000	40.7	36.2	45.9
Burundi	Cankuzo	Mishiha	40604	2005	34.8	31.0	38.8
Burundi	Cankuzo	Mishiha	40604	2010	29.5	25.9	33.1
Burundi	Cankuzo	Mishiha	40604	2015	28.1	23.6	33.0
Burundi	Cibitoke	Buganda	40605	2000	44.2	40.0	48.9
Burundi	Cibitoke	Buganda	40605	2005	37.0	33.4	40.7
Burundi	Cibitoke	Buganda	40605	2010	31.5	28.2	35.1
Burundi	Cibitoke	Buganda	40605	2015	30.9	26.5	36.0
Burundi	Cibitoke	Bukinanyana	40606	2000	45.4	41.2	50.3
Burundi	Cibitoke	Bukinanyana	40606	2005	37.5	34.0	41.2
Burundi	Cibitoke	Bukinanyana	40606	2010	32.3	29.2	36.1
Burundi	Cibitoke	Bukinanyana	40606	2015	29.2	25.0	33.9
Burundi	Cibitoke	Mabayi	40607	2000	44.1	40.7	48.3
Burundi	Cibitoke	Mabayi	40607	2005	35.8	32.8	39.0
Burundi	Cibitoke	Mabayi	40607	2010	30.6	27.5	33.9
Burundi	Cibitoke	Mabayi	40607	2015	27.7	23.9	32.2
Burundi	Cibitoke	Mugina	40608	2000	43.0	39.2	47.4
Burundi	Cibitoke	Mugina	40608	2005	35.1	31.8	38.3
Burundi	Cibitoke	Mugina	40608	2010	30.0	26.8	33.3
Burundi	Cibitoke	Mugina	40608	2015	27.6	23.6	32.3
Burundi	Cibitoke	Murwi	40609	2000	45.0	40.9	49.6
Burundi	Cibitoke	Murwi	40609	2005	37.3	33.7	40.9
Burundi	Cibitoke	Murwi	40609	2010	32.1	28.9	35.7
Burundi	Cibitoke	Murwi	40609	2015	29.7	25.5	34.5
Burundi	Cibitoke	Rugombo	40610	2000	44.5	40.2	49.6

Admin 0	Admin 1	Admin 2	GAUL Code	Year	Neonatal mortality (per 1,000 livebirths)		
					Estimate	Lower bound, 95% UI	Upper bound, 95% UI
Burundi	Cibitoke	Rugombo	40610	2005	36.4	32.8	40.3
Burundi	Cibitoke	Rugombo	40610	2010	30.8	27.1	34.3
Burundi	Cibitoke	Rugombo	40610	2015	30.3	25.8	35.7
Burundi	Gitega	Bugendana	40611	2000	44.1	40.2	48.2
Burundi	Gitega	Bugendana	40611	2005	37.6	34.6	40.8
Burundi	Gitega	Bugendana	40611	2010	32.1	29.3	35.4
Burundi	Gitega	Bugendana	40611	2015	28.6	24.7	33.0
Burundi	Gitega	Bukirasazi	40612	2000	40.2	36.2	44.6
Burundi	Gitega	Bukirasazi	40612	2005	35.8	32.6	39.5
Burundi	Gitega	Bukirasazi	40612	2010	30.5	27.2	34.0
Burundi	Gitega	Bukirasazi	40612	2015	27.0	23.0	31.4
Burundi	Gitega	Buraza	40613	2000	39.8	35.8	44.1
Burundi	Gitega	Buraza	40613	2005	35.4	32.2	39.1
Burundi	Gitega	Buraza	40613	2010	30.2	27.1	33.7
Burundi	Gitega	Buraza	40613	2015	26.8	22.7	31.2
Burundi	Gitega	Giheta	40614	2000	43.2	39.3	47.5
Burundi	Gitega	Giheta	40614	2005	37.1	33.8	40.6
Burundi	Gitega	Giheta	40614	2010	31.8	28.8	35.2
Burundi	Gitega	Giheta	40614	2015	28.3	24.4	32.7
Burundi	Gitega	Gishubi	40615	2000	40.5	36.6	44.5
Burundi	Gitega	Gishubi	40615	2005	35.6	32.4	38.9
Burundi	Gitega	Gishubi	40615	2010	30.8	27.7	34.2
Burundi	Gitega	Gishubi	40615	2015	27.1	23.2	31.3
Burundi	Gitega	Gitega	40616	2000	41.8	37.7	46.2
Burundi	Gitega	Gitega	40616	2005	36.4	32.9	40.0
Burundi	Gitega	Gitega	40616	2010	31.1	27.9	34.7
Burundi	Gitega	Gitega	40616	2015	28.1	24.2	32.7
Burundi	Gitega	Itaba	40617	2000	41.1	36.9	45.4
Burundi	Gitega	Itaba	40617	2005	36.3	32.8	40.2
Burundi	Gitega	Itaba	40617	2010	31.1	27.7	34.6
Burundi	Gitega	Itaba	40617	2015	27.7	23.6	32.2
Burundi	Gitega	Makebuko	40618	2000	41.2	37.3	45.1
Burundi	Gitega	Makebuko	40618	2005	36.1	32.8	39.6
Burundi	Gitega	Makebuko	40618	2010	31.1	27.9	34.6
Burundi	Gitega	Makebuko	40618	2015	27.5	23.7	31.8
Burundi	Gitega	Mutaho	40619	2000	44.8	40.2	49.4
Burundi	Gitega	Mutaho	40619	2005	38.1	34.6	41.5
Burundi	Gitega	Mutaho	40619	2010	32.3	29.2	35.8
Burundi	Gitega	Mutaho	40619	2015	28.9	24.8	33.6
Burundi	Gitega	Nyanrusange	40620	2000	40.9	36.9	44.9
Burundi	Gitega	Nyanrusange	40620	2005	35.7	32.4	39.3
Burundi	Gitega	Nyanrusange	40620	2010	30.9	27.9	34.5
Burundi	Gitega	Nyanrusange	40620	2015	27.2	23.2	31.6
Burundi	Gitega	Ryansoro	40621	2000	40.1	36.1	44.2
Burundi	Gitega	Ryansoro	40621	2005	35.3	32.2	39.0
Burundi	Gitega	Ryansoro	40621	2010	30.4	27.3	33.8
Burundi	Gitega	Ryansoro	40621	2015	26.9	23.1	31.4
Burundi	Karuzi	Bugenyuzi	40622	2000	47.0	42.8	51.5
Burundi	Karuzi	Bugenyuzi	40622	2005	39.1	35.7	42.5
Burundi	Karuzi	Bugenyuzi	40622	2010	32.8	29.7	35.9
Burundi	Karuzi	Bugenyuzi	40622	2015	29.9	25.8	34.6
Burundi	Karuzi	Buhiga	40623	2000	47.3	42.9	52.0
Burundi	Karuzi	Buhiga	40623	2005	39.6	35.9	43.3
Burundi	Karuzi	Buhiga	40623	2010	32.9	29.8	36.3
Burundi	Karuzi	Buhiga	40623	2015	30.1	25.9	34.7
Burundi	Karuzi	Gihogazi	40624	2000	46.0	41.6	50.7
Burundi	Karuzi	Gihogazi	40624	2005	38.5	35.3	41.8
Burundi	Karuzi	Gihogazi	40624	2010	32.5	29.5	35.8
Burundi	Karuzi	Gihogazi	40624	2015	28.9	25.0	33.5
Burundi	Karuzi	Gitaramuka	40625	2000	48.2	43.6	53.5
Burundi	Karuzi	Gitaramuka	40625	2005	39.9	35.8	43.9
Burundi	Karuzi	Gitaramuka	40625	2010	33.2	29.8	36.8
Burundi	Karuzi	Gitaramuka	40625	2015	30.8	26.3	35.6
Burundi	Karuzi	Mutumba	40626	2000	46.0	41.9	50.5
Burundi	Karuzi	Mutumba	40626	2005	38.6	35.0	42.3
Burundi	Karuzi	Mutumba	40626	2010	32.4	29.1	35.5
Burundi	Karuzi	Mutumba	40626	2015	29.1	25.0	33.7
Burundi	Karuzi	Nyabikere	40627	2000	45.5	41.5	49.9
Burundi	Karuzi	Nyabikere	40627	2005	38.3	35.0	41.9
Burundi	Karuzi	Nyabikere	40627	2010	32.3	29.2	35.6
Burundi	Karuzi	Nyabikere	40627	2015	28.9	24.9	33.3
Burundi	Karuzi	Shombo	40628	2000	44.7	40.8	48.8
Burundi	Karuzi	Shombo	40628	2005	37.9	34.8	41.2
Burundi	Karuzi	Shombo	40628	2010	32.1	29.1	35.4
Burundi	Karuzi	Shombo	40628	2015	28.9	24.9	33.4
Burundi	Kayanza	Butaganzwa	40629	2000	44.7	40.9	48.8
Burundi	Kayanza	Butaganzwa	40629	2005	37.7	34.6	40.9
Burundi	Kayanza	Butaganzwa	40629	2010	32.0	29.1	35.0
Burundi	Kayanza	Butaganzwa	40629	2015	27.7	24.1	31.9
Burundi	Kayanza	Gahombo	40630	2000	46.4	42.4	51.0
Burundi	Kayanza	Gahombo	40630	2005	38.5	35.3	41.6
Burundi	Kayanza	Gahombo	40630	2010	32.6	29.5	35.9
Burundi	Kayanza	Gahombo	40630	2015	27.6	23.8	31.8
Burundi	Kayanza	Gatara	40631	2000	46.2	42.3	50.6
Burundi	Kayanza	Gatara	40631	2005	38.2	35.1	41.2

Admin 0	Admin 1	Admin 2	GAUL Code	Year	Neonatal mortality (per 1,000 livebirths)		
					Estimate	Lower bound, 95% UI	Upper bound, 95% UI
Burundi	Kayanza	Gatara	40631	2010	32.5	29.5	35.6
Burundi	Kayanza	Gatara	40631	2015	27.7	23.9	32.0
Burundi	Kayanza	Kabarore	40632	2000	46.7	42.6	51.6
Burundi	Kayanza	Kabarore	40632	2005	38.2	34.9	41.7
Burundi	Kayanza	Kabarore	40632	2010	32.5	29.3	36.0
Burundi	Kayanza	Kabarore	40632	2015	28.8	24.9	33.2
Burundi	Kayanza	Kayanza	40633	2000	46.5	42.7	50.8
Burundi	Kayanza	Kayanza	40633	2005	38.4	35.3	41.5
Burundi	Kayanza	Kayanza	40633	2010	32.7	29.6	35.9
Burundi	Kayanza	Kayanza	40633	2015	28.5	24.6	32.8
Burundi	Kayanza	Matongo	40634	2000	45.3	41.8	49.2
Burundi	Kayanza	Matongo	40634	2005	38.1	35.1	41.0
Burundi	Kayanza	Matongo	40634	2010	32.2	29.5	35.1
Burundi	Kayanza	Matongo	40634	2015	28.0	24.4	32.2
Burundi	Kayanza	Muhanga	40635	2000	46.6	42.1	51.5
Burundi	Kayanza	Muhanga	40635	2005	39.0	35.5	42.7
Burundi	Kayanza	Muhanga	40635	2010	32.6	29.4	36.0
Burundi	Kayanza	Muhanga	40635	2015	28.7	24.6	33.3
Burundi	Kayanza	Muruta	40636	2000	45.8	41.8	50.2
Burundi	Kayanza	Muruta	40636	2005	38.2	34.9	41.4
Burundi	Kayanza	Muruta	40636	2010	32.6	29.6	35.9
Burundi	Kayanza	Muruta	40636	2015	28.6	24.6	33.0
Burundi	Kayanza	Rango	40637	2000	45.5	41.3	49.9
Burundi	Kayanza	Rango	40637	2005	38.3	34.9	41.6
Burundi	Kayanza	Rango	40637	2010	32.1	29.2	35.4
Burundi	Kayanza	Rango	40637	2015	28.0	24.2	32.4
Burundi	Kirundo	Bugabira	40638	2000	45.4	41.9	49.2
Burundi	Kirundo	Bugabira	40638	2005	34.8	32.0	37.5
Burundi	Kirundo	Bugabira	40638	2010	29.4	26.7	32.2
Burundi	Kirundo	Bugabira	40638	2015	28.7	24.8	32.9
Burundi	Kirundo	Busoni	40639	2000	47.0	42.9	51.0
Burundi	Kirundo	Busoni	40639	2005	36.2	33.1	39.6
Burundi	Kirundo	Busoni	40639	2010	30.2	27.3	33.0
Burundi	Kirundo	Busoni	40639	2015	29.1	25.2	33.4
Burundi	Kirundo	Bwambarangwe	40640	2000	47.0	42.5	51.5
Burundi	Kirundo	Bwambarangwe	40640	2005	37.3	33.7	41.0
Burundi	Kirundo	Bwambarangwe	40640	2010	30.9	27.7	34.2
Burundi	Kirundo	Bwambarangwe	40640	2015	29.3	25.2	33.8
Burundi	Kirundo	Gitobe	40641	2000	48.8	44.8	53.0
Burundi	Kirundo	Gitobe	40641	2005	39.0	35.6	42.2
Burundi	Kirundo	Gitobe	40641	2010	31.6	28.7	34.7
Burundi	Kirundo	Gitobe	40641	2015	30.0	26.0	34.4
Burundi	Kirundo	Kirundo	40642	2000	48.1	44.3	51.9
Burundi	Kirundo	Kirundo	40642	2005	37.4	34.3	40.4
Burundi	Kirundo	Kirundo	40642	2010	31.0	28.2	33.9
Burundi	Kirundo	Kirundo	40642	2015	29.9	25.8	34.4
Burundi	Kirundo	Ntega	40643	2000	48.8	44.7	53.0
Burundi	Kirundo	Ntega	40643	2005	37.9	34.6	41.1
Burundi	Kirundo	Ntega	40643	2010	31.0	28.1	34.2
Burundi	Kirundo	Ntega	40643	2015	29.7	25.7	34.3
Burundi	Kirundo	Vumbi	40644	2000	49.1	45.2	53.1
Burundi	Kirundo	Vumbi	40644	2005	38.8	35.6	41.8
Burundi	Kirundo	Vumbi	40644	2010	32.1	29.2	34.9
Burundi	Kirundo	Vumbi	40644	2015	30.4	26.4	34.8
Burundi	Makamba	Kayogoro	40646	2000	35.8	31.3	40.7
Burundi	Makamba	Kayogoro	40646	2005	33.2	29.2	37.6
Burundi	Makamba	Kayogoro	40646	2010	28.0	24.2	32.0
Burundi	Makamba	Kayogoro	40646	2015	25.6	21.2	30.5
Burundi	Makamba	Kibago	40647	2000	35.3	30.7	39.9
Burundi	Makamba	Kibago	40647	2005	33.0	29.1	37.4
Burundi	Makamba	Kibago	40647	2010	27.4	23.9	31.3
Burundi	Makamba	Kibago	40647	2015	24.7	20.5	29.5
Burundi	Makamba	Mabanda	40648	2000	35.5	30.6	40.5
Burundi	Makamba	Mabanda	40648	2005	32.7	28.8	37.1
Burundi	Makamba	Mabanda	40648	2010	27.2	23.5	31.2
Burundi	Makamba	Mabanda	40648	2015	24.4	20.1	29.2
Burundi	Makamba	Makamba	40649	2000	36.3	31.7	40.8
Burundi	Makamba	Makamba	40649	2005	33.3	29.6	37.7
Burundi	Makamba	Makamba	40649	2010	27.9	24.3	31.6
Burundi	Makamba	Makamba	40649	2015	24.7	20.6	29.2
Burundi	Makamba	Nyanza-Lac	40650	2000	35.1	30.1	40.7
Burundi	Makamba	Nyanza-Lac	40650	2005	31.9	27.5	36.7
Burundi	Makamba	Nyanza-Lac	40650	2010	26.9	22.9	31.2
Burundi	Makamba	Nyanza-Lac	40650	2015	24.5	19.7	29.9
Burundi	Makamba	Vugizo	40651	2000	37.0	32.2	41.9
Burundi	Makamba	Vugizo	40651	2005	33.6	29.7	37.9
Burundi	Makamba	Vugizo	40651	2010	27.9	24.1	31.8
Burundi	Makamba	Vugizo	40651	2015	24.4	20.2	29.3
Burundi	Muramvya	Bukeye	40652	2000	43.4	39.9	47.1
Burundi	Muramvya	Bukeye	40652	2005	37.3	34.4	40.5
Burundi	Muramvya	Bukeye	40652	2010	31.4	28.7	34.4
Burundi	Muramvya	Bukeye	40652	2015	27.7	24.2	31.9
Burundi	Muramvya	Kiganda	40653	2000	42.4	39.0	46.1
Burundi	Muramvya	Kiganda	40653	2005	36.6	33.7	39.7
Burundi	Muramvya	Kiganda	40653	2010	30.7	28.1	33.7

Admin 0	Admin 1	Admin 2	GAUL Code	Year	Neonatal mortality (per 1,000 livebirths) Estimate	Lower bound, 95% UI	Upper bound, 95% UI
Burundi	Muramvya	Kiganda	40653	2015	27.5	23.8	31.6
Burundi	Muramvya	Mbuye	40654	2000	43.6	39.8	47.3
Burundi	Muramvya	Mbuye	40654	2005	37.4	34.4	40.5
Burundi	Muramvya	Mbuye	40654	2010	31.7	29.0	34.8
Burundi	Muramvya	Mbuye	40654	2015	27.8	24.1	31.9
Burundi	Muramvya	Muramvya	40655	2000	41.8	38.5	45.5
Burundi	Muramvya	Muramvya	40655	2005	36.4	33.5	39.6
[truncated: 1,270,848 more chars]
